# Supplementary material for: Magnetic Fields and Cancer: Epidemiology, Cellular Biology, and Theranostics
Source: Int J Mol Sci. 2022 Jan 25;23(3):1339. doi: 10.3390/ijms23031339 (PMC8835851; doi:10.3390/ijms23031339)
Supplement: Supplementary file 1 [file ijms-23-01339-s001.zip › Supplementary Data Set S1/MF and Cancer.Data/PDF/0062303658/72.full.pdf]

Future needs of occupational epidemiology of extremely low frequency electric and magnetic fields: review and recommendations

L Kheifets,[1] J D Bowman,[2] H Checkoway,[3] M Feychting,[4] J M Harrington,[5] R Kavet,[6] G Marsh,[7] G Mezei,[6] D C Renew,[8] E van Wijngaarden[9]

[1] Department of Epidemiology, UCLA School of Public Health, Los Angeles, California, USA; [2] National Institute for Occupational Safety and Health, Engineering and Physical Hazards Branch, Cincinnati, Ohio, USA; [3] Department of Environmental and Occupational Health Sciences, University of Washington, Seattle, Washington, USA; [4] Institute of Environmental Medicine, Karolinska Institutet, Stockholm, Sweden; [5] Institute of Occupational Health, University of Birmingham, Birmingham, UK; [6] Electric Power Research Institute, Palo Alto, California, USA; [7] Department of Biostatistics, Graduate School of Public Health, University of Pittsburgh, Pittsburgh, Pennsylvania, USA; [8] National Grid plc, London, UK; [9] Division of Epidemiology, Department of Community and Preventive Medicine, University of Rochester School of Medicine and Dentistry, Rochester, New York, USA

Correspondence to: Leeka Kheifets, UCLA School of Public Health, Department of Epidemiology, 73-284 CHS, 650 Charles E Young Drive South, Los Angeles, CA 90095-1772, USA; kheifets@ucla.edu

Accepted 30 July 2008
Published Online First 19 September 2008

ABSTRACT

The occupational epidemiological literature on extremely low frequency electric and magnetic fields (EMF) and health encompasses a large number of studies of varying design and quality that have addressed many health outcomes, including various cancers, cardiovascular disease, depression and suicide, and neurodegenerative diseases, such as Alzheimer disease and amyotrophic lateral sclerosis (ALS). At a 2006 workshop we reviewed studies of occupational EMF exposure with an emphasis on methodological weaknesses, and proposed analytical ways to address some of these. We also developed research priorities that we hope will address remaining uncertainties. Broadly speaking, extensive epidemiological research conducted during the past 20 years on occupational EMF exposure does not indicate strong or consistent associations with cancer or any other health outcomes. Inconsistent results for many of the outcomes may be attributable to numerous shortcomings in the studies, most notably in exposure assessment. There is, however, no obvious correlation between exposure assessment quality and observed associations. Nevertheless, for future research, the highest priorities emerge in both the areas of exposure assessment and investigation of ALS. To better assess exposure, we call for the development of a more complete job-exposure matrix that combines job title, work environment and task, and an index of exposure to electric fields, magnetic fields, spark discharge, contact current, and other chemical and physical agents. For ALS, we propose an international collaborative study capable of illuminating a reported association with electrical occupations by disentangling the potential roles of electric shocks, magnetic fields and bias. Such a study will potentially lead to evidence-based measures to protect public health.

A substantial segment of the epidemiology on electric and magnetic fields (EMF) has focused on occupational settings, where exposures that are generally greater than those in the general population provide the potential for easier detection of effects. More importantly, EMF exposure is increasing in some occupational settings and concerns a large number of workers in a variety of industries. Further research affords an opportunity to prevent adverse health effects through exposure reduction, if warranted.

Most of the prior reviews focus on environmental exposures or on particular diseases, leaving many questions unanswered. To identify the highest priority research needs in occupational extremely low frequency (ELF) EMF epidemiology and to develop practical proposals to address remaining uncertainties, the Energy Networks Association (ENA) in the UK sponsored a meeting to which an international group of experts was invited. The sponsors imposed no constraints on the group's review of the current state of knowledge or on their deliberations or recommendations. The meeting took place in Edinburgh, Scotland over 14–15 September 2006. In this summary report we review studies of occupational EMF exposure, discuss impediments in many of these studies, and present priority recommendations for research to address remaining uncertainties. Following extensive literature searches in PubMed and other databases, papers for detailed review were selected according to the judgment of workshop participants.

HISTORICAL OVERVIEW OF OCCUPATIONAL EMF EPIDEMIOLOGY

The occupational EMF literature encompasses a large number of studies with a variety of designs, such as proportional mortality studies, studies of workers in "electrical" occupations, studies of electric utility cohorts, and studies of the general population. The literature includes mortality and incidence studies.

Early studies were characterised by rather crude exposure assessment, sometimes limited to investigation of disease risk for individual job titles or to grouping of job titles as "electrical occupations"; often, an individual was classified as having an electrical occupation (variously defined) on the basis of a single job reported on a death certificate. Later studies improved exposure assessment through the use of job-exposure matrices (JEMs) based on workday measurements of magnetic field levels in various occupations. The most sophisticated and detailed JEMs relied on individual measurements for both individual work tasks and work environments. Initially, JEMs included occupations held predominantly by men and only recently have included those held mainly by women. Also recently, exposure assessment has shifted from jobs to relevant sources of exposure, permitting assessment of historical as well as current exposures.

Large cohorts of electric utility employees have contributed the most significant share of the occupational data.[1–7] In addition, disease registries linked to JEMs have included workers in other industries.[8–11] Following the first report by Wertheimer and Leeper in 1979 of an increased

Occup Environ Med: first published as 10.1136/oem.2007.037994 on 19 September 2008. Downloaded from http://oem.bmj.com/ on November 20, 2021 at Universita di Torino. Protected by copyright.

risk of adult cancer with work in electrical jobs,[12] initial studies by Milham[13] and others concentrated on leukaemia and brain cancer. Subsequent studies have investigated other cancers, notably breast cancer, and, more recently, cardiovascular and neurodegenerative diseases.

EPIDEMIOLOGICAL STUDIES OF OCCUPATIONAL EMF EXPOSURE AND HEALTH OUTCOMES

Cancer

A pooled analysis of major utility cohort studies published in 1999 found weak associations between occupational EMF exposure and leukaemia and brain cancer.[14] Recent analyses of large electric utility cohorts in Denmark[7] and the United Kingdom,[5 6] welders in Sweden[9] and railway workers in Switzerland[15] showed no consistent risk increases for leukaemia or brain cancer with elevated occupational magnetic field exposure. Reduced exposure misclassification through improvements in exposure assessment methods, such as the use of detailed individual job histories augmented with full-shift measurements or JEMs, did not result in higher risk. Although a 2008 update to meta-analyses conducted in 1995[16] and 1997[17] found small risk increases for all types of leukaemia and brain cancer, pooled risk estimates were lower than in past meta-analyses, and leukaemia subtypes showed no consistent pattern when past and present meta-analyses were compared.[18] Findings were not sensitive to assumptions, influential studies, weighting schemes, publication bias, study characteristics or funding source. Although a small risk increase for leukaemia and brain cancer cannot be entirely excluded, the lack of a clear exposure–outcome pattern suggests that magnetic fields as presently measured are by themselves not responsible for the observed excess risk. A recent World Health Organization review of occupational studies[19] did not alter the International Agency for Research on Cancer's 2002 conclusion that there is inadequate evidence to link magnetic field exposure to adult cancers.[20]

The hypothesis that magnetic field exposure increases breast cancer risk by suppressing nocturnal melatonin provided the motivation for studying breast cancer in relation to occupational magnetic field exposure. For male breast cancer, which is extremely rare, the literature consists mostly of studies with small numbers of cases. Both cohort and case–control studies show conflicting results, and overall do not provide strong support for an association.[21–25]

For female breast cancer, study results are mixed, with some studies reporting moderately elevated risks for premenopausal women and for oestrogen-receptor-positive breast cancers.[25–28] Risk did not increase with elevated magnetic field exposure in a recent case–control study of more than 20 000 female breast cancer cases in Sweden that used an elaborate, female-specific JEM and included adequate control for potential confounders.[10] Recent studies from Norway, the United States and China also indicated no association between occupational exposure to magnetic fields and female breast cancer.[29–31] The epidemiological evidence available to date thus does not provide support for an association.

Several studies examined various other types of cancer.[19 20 24 32–39] Overall, there is no consistent indication of any relationship between these cancers and occupational magnetic field exposure.

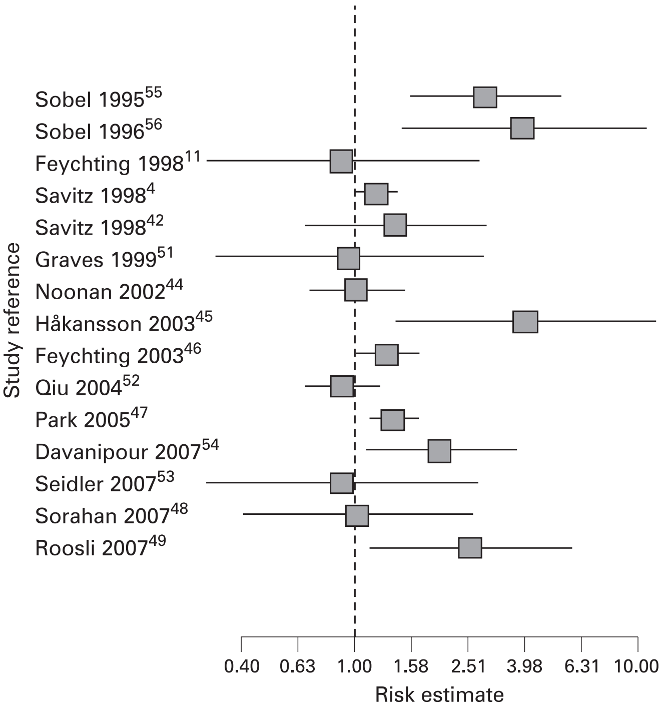

Figure 1 Results from individual studies of Alzheimer disease. For consistency between studies, the risk estimates presented are based on overall analyses of subjects' primary occupations. Included case–control and cohort studies were identified through a PubMed search using the keywords "neurodegenerative disease" or "Alzheimer's disease" and "electromagnetic fields" or "magnetic fields". The search was restricted to articles in English and to studies reporting results for Alzheimer's disease separately.

Neurodegenerative diseases

Research investigating risk of neurodegenerative diseases has focused mainly on Alzheimer disease (recently reviewed by Santibáñez *et al*[40]) and amyotrophic lateral sclerosis (ALS), and, to a lesser extent, on Parkinson disease.

A notable weakness in neurodegenerative disease studies is case identification. In some studies, cases were identified in hospitals and controls among patients with other diseases at the same hospitals or among friends or relatives of cases. These studies are likely to have greater potential for selection bias than population-based studies, which, on the other hand, have often identified cases from mortality registries and thus have greater potential for disease misclassification.[41]

No consistent evidence has linked Parkinson disease to high occupational EMF exposures. Most of the available studies found no evidence of increased risk.[4 42–49]

Early studies of Alzheimer disease provided weak evidence of an association with EMF.[50] The strongest evidence came from two clinic-based studies, whereas evidence from four population-based studies was considerably weaker and more inconsistent, for example, in terms of exposure timing.[4 11 42 51] Eight studies published in 2000 or later were population based, and again the results were mostly inconsistent,[44–49 52 53] whereas one clinic-based study[54] was consistent with previous studies by the same investigators that used their original design.[55 56]

Figure 1 provides risk estimates. Findings of increased risk do not seem to have been influenced by exposure assessment method or, with the exception of clinic-based studies, source of case identification. Subgroup analyses within studies strengthen the impression of inconsistency in the data. One recent study of mortality among electricity generation and transmission workers in the UK indicated no increase in Alzheimer disease,[48] whereas a Swiss study of railway employees found an increasing risk of Alzheimer disease with increasing exposure.[49]

Occup Environ Med: first published as 10.1136/oem.2007.037994 on 19 September 2008. Downloaded from http://oem.bmj.com/ on November 20, 2021 at Universita di Torino. Protected by copyright.

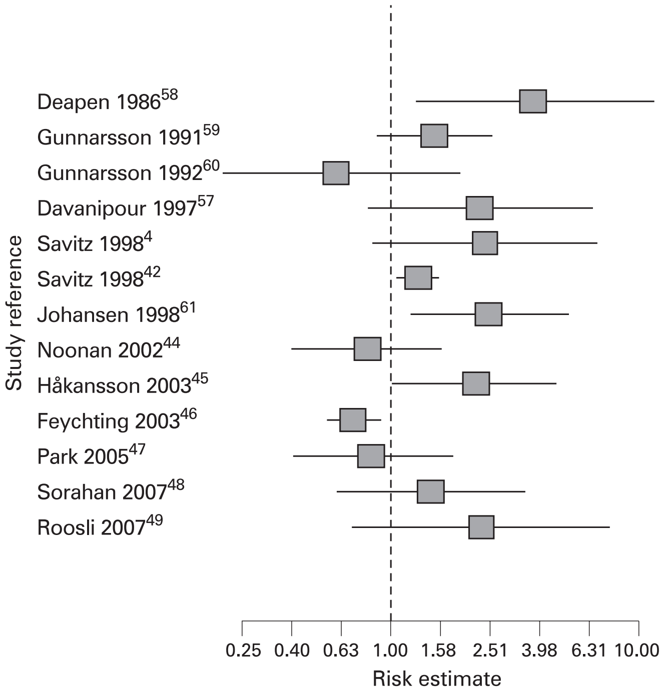

Figure 2 Results from individual studies of amyotrophic lateral sclerosis (ALS). Included case–control and cohort studies were identified through a PubMed search using the keywords "neurodegenerative disease" or "amyotrophic lateral sclerosis" and "electromagnetic fields" or "magnetic fields". The search was restricted to articles in English.

Table 1 summarises selected ALS studies; fig 2 shows risk estimates. Studies up to 2000 yielded more consistent evidence (table 1), with several clinic- and population-based studies suggesting an increased risk with employment in electrical occupations.[4 42 57–61] Two of six recent studies reported an increased risk with EMF exposure; one of the two may have been confounded by co-exposure to neurotoxic agents,[45] and the other was restricted to railway workers.[49] Three of the studies reporting no effect on ALS risk estimated magnetic field exposure through JEMs[44 46 47]; like the earlier studies, however, two of these studies[44 46] found an increased risk for electrical occupations. A recent UK study reported broadly similar results for magnetic field exposure but found no risk increases in any job categories for motor neuron disease mortality among electricity generation and transmission workers compared to the general population.[48] Thus, the evidence linking electrical occupations to an increased risk of ALS is remarkably consistent, but the evidence of an association with measured magnetic field levels is weaker. One explanation could be that another risk factor causes the increased risk in electrical occupations. Exposure to electric shock is a possibility,[50 58 61] although it is not likely to explain the entire risk increase.[46]

Other diseases

Cardiovascular disease

A 2007 review provides a detailed summary of studies of EMF and cardiovascular disease.[62] Early descriptive studies reporting arrhythmias and tachycardia among Russian high-voltage switchyard workers first raised concern about cardiovascular effects from EMF exposure. Later studies were designed to investigate the hypothesis that long-term exposure to EMF may increase cardiovascular disease mortality by reducing heart-rate variability.[63–67] In a 1999 cohort study of electric utility workers, Savitz *et al* found elevated risks of mortality from cardiovascular disease.[68] A number of epidemiological studies in several countries that used a variety of study designs did not confirm Savitz's findings.[62 69–73] Additionally, recent experimental studies of volunteers[74] found no decrease in heart-rate variability after exposure to EMF. We therefore conclude that occupational exposure to EMF does not appear to play a role in the aetiology of cardiovascular diseases.

Suicide

A pathway involving a possible effect of EMF on melatonin production, the role of melatonin in depression, and depression as an important risk factor for suicidal behaviour makes suicide a potential consequence of EMF exposure.[75 76] Selected occupational studies on this subject are summarised in table 2.

In a study of electric utility workers, van Wijngaarden *et al* observed an exposure–response relationship with recent but not distant magnetic field exposure, with an OR of 1.7 in the highest exposure group.[76] However, other electric utility worker studies provide only limited support.[61 77] Studies using job title as a surrogate for EMF exposure are equally inconsistent.[78–81] Lack of information on potential confounding factors in most studies further hampers the interpretation of these findings.

CHALLENGES

Occupational EMF exposure assessment

ELF magnetic field exposures

Exposure assessment, including the use of JEMs, remains a major challenge in occupational EMF epidemiology. For the past 15 years, the better studies have assessed the magnitude of time-weighted average (TWA) magnetic fields with JEMs based on personal measurements. JEMs for electric utility workers[82 83] and for the general population[84 85] have been constructed from extensive full-shift measurements with three-axis magnetic field meters that record an approximation to the "resultant" field every few seconds. These resultant data are combined with activity records to calculate either the TWA of the magnetic field or other metrics.

One problem with JEMs is that occupation is not the main determinant of magnetic field exposure. A person's occupational exposure depends on magnetic field sources encountered during work, the average strength of sources, and the proportion of time spent at different locations relative to sources. Consequently, measured exposure varies widely among individuals within occupational groups.[86] Furthermore, when JEMs are populated solely from measurements, they cannot properly represent historical exposures. Thus, JEMs can misclassify ELF magnetic field exposures.

Several improved JEMs have been developed. Miller *et al* constructed a JEM that classifies exposure measurements by location as well as by job.[87] Renew *et al* assessed exposures of power station workers with a magnetic field model that takes account of the engineering design, layout and operational history for each power station.[88] Results from this model were combined with the proportion of time spent in specific station areas for each job and with occupational history to give individual exposure values. This approach has the added advantage of reconstructing historical exposures. In another approach, Coble *et al* (submitted) modified the JEM's average magnetic field for an individual by using interview information on exposure times and locations relative to reported sources. A combination of these innovations might reduce exposure misclassification in future studies. However, considerable

Occup Environ Med: first published as 10.1136/oem.2007.037994 on 19 September 2008. Downloaded from http://oem.bmj.com/ on November 20, 2021 at Universita di Torino. Protected by copyright.

Occup Environ Med: first published as 10.1136/oem.2007.037994 on 19 September 2008. Downloaded from http://oem.bmj.com/ on November 20, 2021 at Universita di Torino. Protected by copyright.

Table 1 Main characteristics and findings of epidemiological studies of exposure to occupational electric and magnetic fields and risk of amyotrophic lateral sclerosis (ALS)

Author, year	Study population (location)	Exposure assessment	Covariates	Exposure group	RR (95% CI); observed*
Deapen and Henderson, 1986[58]	518 ALS cases identified from the ALS Society 1977–79, 518 friend controls (USA)	Exposure defined as "electrical occupations"		Electrical occupation	3.8 (1.4 to 13.0); 19
Gunnarsson *et al*, 1991[59]	1961 deaths from ALS in the Swedish population 1970–83, 2245 population-based controls (Sweden)	Occupational categories	Age, men only	Electricity workers	1.5 (0.9 to 2.6); 32
Gunnarsson *et al*, 1992[60]	92 prevalent cases of motor neuron disease at hospitals in central and southern Sweden, 372 population-based controls (Sweden)	Occupational categories	Age, men only	Electricity work	6.7 (1.0 to 32.1); 4
Davanipour *et al*, 1997[57]	28 clinic-based prevalent cases of ALS, 32 relatives controls (USA)	Occupations classified by industrial hygienist as electrical (high or medium) or non-electrical	Sex	Average lifetime occupational exposure, 75th percentile	2.3 (0.8 to 6.6)
Savitz *et al*, 1998[4]	Cohort of 139 905 men working at five US electric utility companies 1950–86. Mortality compared to the general population, and internal comparisons within cohort; 33 ALS cases identified (USA)	Used work-shift measurements of extremely low frequency fields in a sample of workers to categorise occupations	Age, calendar year, race, social class, work status, poly-chlorinated biphenyl and solvent exposure	Employment in exposed occupation, years	
				0–$<$5	1.0; 22
				5–$<$20	1.8 (0.7 to 4.7); 18
				$\geqslant$20	2.4 (0.8 to 6.7); 16
Savitz *et al*, 1998[42]	114 deaths from ALS 1985–91, matched controls selected from among persons who died from other causes not related to electric and magnetic fields (USA)	Occupations classified according to work in "electrical occupations"	Age, social class, men only	Electrical occupation	1.3 (1.1 to 1.6); 114
Johansen and Olsen, 1998†[61]	Cohort of 26 135 men working in an electric utility company 1900–93; 14 deaths from ALS identified. Mortality compared to national mortality rates (Denmark)	Job-exposure matrix; categorised workers into high, medium, low or background magnetic field exposure	Age, duration of employment, men only	Medium to high exposure ($\geqslant$0.3 μT)	2.5 (1.1 to 4.8); 9
Noonan *et al*, 2002[44]	312 deaths from ALS 1987–96 in Colorado, 1248 matched controls among other causes of death (USA)	Three methods: 1) primary occupation classified as electrical or non-electrical, 2) combination of occupation and industry codes classified into four exposure categories, 3) population-based job-exposure matrix constructed from 2400 magnetic field measurements	Age, race, occupational grouping, men only	Electrical occupation	2.3 (1.29 to 4.09); 19
				No exposure	1.0; 242
				Possible exposure	1.18 (0.83 to 1.67); 285
				Definite or probable exposure	1.75 (1.00 to 3.06); 19
				Average exposure	
				$<$0.1 μT	1.0; 59
				0.1–0.19 μT	0.79 (0.54 to 1.15); 134
				0.2–0.29 μT	1.21 (0.75 to 1.93); 51
				$\geqslant$0.3 μT	0.77 (0.37 to 1.59); 12
Håkansson *et al*, 2003[45]	Cohort of Swedish engineering industry workers with a large proportion of resistance welders, 537 692 men and 180 529 women followed in Causes of Death Registry 1985–96; 97 deaths from ALS identified (Sweden)	Information on occupation from censuses linked to a job-exposure matrix constructed from over 1000 measurements	Age, sex, socioeconomic status	Average exposure	
				$<$0.164 μT	1.0; 15
				0.164–0.250 μT	1.58 (0.88 to 2.81); 52
				0.250–0.530 μT	1.95 (0.97 to 3.92); 17
				$>$0.530 μT	2.16 (1.01 to 4.66); 13
Feychting *et al*, 2003[46]	Economically active Swedish population 1980, 4 812 646 persons, followed in the Causes of Death Registry 1981–95; 1965 deaths from ALS identified (Sweden)	Information on occupation from 1970 and 1980 censuses linked to a job-exposure matrix constructed from over 1000 measurements	Age, sex, socioeconomic status	Average exposure $\geqslant$3 μT	
				1970 and 1980	0.8 (0.6 to 1.1); 48
				1970 or 1980	0.8 (0.7 to 1.0); 197
				$\geqslant$5 μT	
				1970 and 1980	0.6 (0.4 to 1.0); 16
				1970 or 1980	0.7 (0.6 to 1.0); 83
				Electrical occupations	1.4 (1.0 to 1.8); 47
Park *et al*, 2005[47]	6347 deaths from motor neuron disease in 22 states during 1992–98; controls were deaths from other causes not related to electric and magnetic fields or solvents (USA)	Information about usual occupation from death certificate linked to job-exposure matrix constructed from 2400 magnetic field measurements	Age, sex, race, region, socio-economic status	Analysed as a continuous variable with 10 equal width exposure strata	0.94 (0.73 to 1.20); 5965

Continued

Table 1 Continued

Author, year	Study population (location)	Exposure assessment	Covariates	Exposure group	RR (95% CI); observed*
Sorahan and Kheifets, 2007[48]	Mortality 1973–2004 among 79 972 employees of the former Central Electricity Generating Board of England and Wales with computerised work histories 1971–93; 68 deaths from motor neuron disease identified. Compared to mortality rates for the general population of England and Wales and internal comparisons (Great Britain)	Information on job and facility (location) used to estimate exposures to magnetic fields. Power station workers considered to have highest exposure	Age, sex, calendar year, socioeconomic status (the latter only in internal comparisons)	Electricity generation and transmission workers†	0.87 (0.67 to 1.10); 68
				RR per μT year cumulative exposure	1.04 (0.84 to 1.30); 78
Roosli *et al*, 2007[49]	Mortality 1972–2002 among 20 141 Swiss railway employees. Internal comparisons within cohort, train drivers had highest exposure and station masters the lowest.	Exposure was assessed through on-site measurements and modelling of past exposure	Age, time period. Study was restricted to men	Railway train drivers	1.31 (0.31 to 5.59)
				Cumulative lifetime exposure >median	2.32 (0.70 to 7.73)

*Observed number of exposed ALS cases; †standardised mortality ratios (SMRs).

attention to detail is required when applying improved exposure metrics to ensure that they result in exposure assessments which are more accurate.

Electric fields, shocks and contact current

Magnetic fields are not the only aspect of the ELF electromagnetic environment, which also includes electric fields, electric shocks, microshocks (spark discharge) and contact current.

In electric utility environments, typical elevated electric fields induce greater current in the body than typical elevated magnetic fields, and far greater currents occur in the body during electric shocks, albeit very briefly. Miller reported an association for leukaemia in utility workers that was much stronger for electric fields than for magnetic fields,[87] and electric shocks have been suggested as a risk factor for ALS.

Elevated electric field exposures occur only where there are unshielded high-voltage conductors. Moderate-to-high levels (0.1 to >1 kV/m) are found only near high-voltage transmission lines or exposed busbars in substations. In these limited environments, adequate estimates of TWA electric field exposures can be obtained from spot measurements or computer modelling.[89]

A "taxonomy of electric shocks" that identifies the different types of shocks is needed for future investigations. Severe electric shocks among utility workers are rare and are reportable to government authorities in some countries; this may be useful for compiling data about the rate of severe but non-fatal shocks. Microshocks can be experienced by a person on touching a conducting object in an electric field greater than about 2 kV/m. Contact current flows imperceptibly into the person's body while the person remains in contact with the conductor.

Alternative magnetic field exposure metrics

The absence of an established mechanism by which magnetic fields could produce health effects[90] makes it difficult to identify the definitive metric for assessing exposure. Misspecification of the exposure metric is likely to bias risk estimates towards the null.

A possible source of misclassification in EMF exposure assessment is the sole use of the resultant magnetic field as the exposure metric, on the assumption that TWA adequately represents magnetic field exposure. Other magnetic field metrics that may be more biologically relevant include percentage of time above a threshold, induced current in the body, combinations of ac and dc fields, the ratio of the major and minor axes, and intermittency of exposures.[91] Although many alternative metrics such as these are highly correlated with the TWA, some are not. We can now measure alternatives to the resultant using instruments that capture the magnetic field vector waveform in great detail,[92] combined with software that calculates the alternative metrics from the waveforms (Bowman *et al*, in preparation). The use of multiple metrics for measuring EMF exposure is likely to lead to associations that are purely due to chance. To minimise this possibility, the number of metrics should be limited to either those used in previous studies or those arising from a particular biophysical interaction requiring epidemiological investigation.

Multivariable statistical techniques[93–97] may also be useful in analysing multiple exposure metrics obtained by using measurement data from vector waveform monitors.[97–99]

Health outcome assessment

Misclassification of health outcomes can be an important source of error in occupational epidemiology research. Occupational cohort studies frequently rely on mortality data for outcome determination. For diseases with high case fatality rates (for example, ALS and some cancers), mortality provides a reasonably complete and accurate indication of disease incidence. However, diagnostic information inferred from death certificates may be inaccurate for other outcomes, such as Alzheimer disease, cardiovascular disease and suicide.[41 100] In addition, cases may go undetected or, if identified, may be inaccurately reported. Where feasible, obtaining clinical and pathological information would highly benefit disease diagnosis.

Analyses

Control, evaluation and modelling of potential biases

Recent advances in statistical modelling provide powerful tools that may prove useful for resolving some of the thornier EMF-related analytical issues; for example, for disentangling individual and joint effects of jobs, shocks and magnetic fields in future ALS studies or for taking the exact timing of EMF and other exposures into account. For example, hierarchical regression methods provide a generalised framework for regression analysis that encompasses a number of different approaches, including non-parametric regression, empirical Bayes regression, random-coefficient regression, multilevel analysis, generalised estimating equations, G-estimation, penalised-likelihood analysis, ridge regression and Stein estimation.[101]

Traditional sensitivity analysis, which estimates what the true effect measure (for example, relative risk) would be in light of the observed data and a hypothetical level of bias, can also be

Occup Environ Med: first published as 10.1136/oem.2007.037994 on 19 September 2008. Downloaded from http://oem.bmj.com/ on November 20, 2021 at Universita di Torino. Protected by copyright.

Table 2 Main characteristics and findings of epidemiological studies of exposure to occupational electric and magnetic fields and risk of suicide

Author, year	Study population (location)	Exposure assessment	Covariates	Exposure group	RR (95% CI); observed*
Baris *et al*, 1996[77]	21 744 male electric utility workers followed from 1970 to 1988; case–cohort study of 49 suicide deaths and 217 randomly selected workers (Canada)	Job-exposure matrix based on positron measurements. Arithmetic mean (AM) and geometric mean (GM) of electric, magnetic and pulsed electromagnetic fields	Socioeconomic status, alcohol use, marital status and mental disorders	Cumulative electric fields (V/m) (AM)	
				<136.10	1.0 (–); 21
				136.10–<308.60	1.6 (0.5 to 5.1); 15
				308.60+	1.7 (0.4 to 8.0); 13
				Cumulative electric fields (V/m) (GM)	
				<23.10	1.0 (–); 16
				23.10–<40.30	2.8 (0.9 to 8.1); 20
				40.30+	1.8 (0.4 to 8.5); 13
Kelsh and Sahl, 1997[79]	40 335 electric utility workers followed from 1960 to 1991 (USA)	Collapsed similar job titles into homogenous occupational groups. Analysis based on usual occupation	Age, gender	Occupational category	
				Administrative /technical/clerical	1.0 (–); 18
				Management /professional	0.9 (0.3 to 2.5); 5
				Service/labour	2.2 (0.96 to 5.2); 8
				Linemen	2.0 (1.1 to 3.8); 22
				Meter reader/field service	2.0 (0.6 to 7.1); 3
				Plant operations	2.7 (1.3 to 5.5); 13
				Trade/craft	2.0 (1.0 to 3.8); 19
Johansen and Olsen, 1998†[61]	21 236 male electric utility workers followed from 1974 to 1993 (Denmark)	Job-exposure matrix for magnetic fields based on 24-h measurements and judgment	Age	Estimated average exposure (μT)	
				⩽0.09	1.0 (0.6 to 1.6); 19
				0.1–0.29	0.8 (0.6 to 1.1); 37
				0.3–0.99	0.9 (0.7 to 1.2); 41
				1.0+	1.4 (0.98 to 1.9); 36
Van Wijngaarden *et al*, 2000[76]	138 905 male electric utility workers followed from 1950 to 1988; nested case–control study of 536 suicide deaths and 5348 controls (USA)	Job-exposure matrix for magnetic fields based on time-weighted average measurements	Age, ethnicity, work status, social class, geographic location and exposure to solvents and sunlight	Recent cumulative exposure (past year) (μT-years)	
				0	1.0 (–); 294
				>0–0.029	1.2 (0.8 to 1.9); 58
				0.03–0.049	1.4 (0.9 to 2.3); 62
				0.05–0.11	1.6 (0.97 to 2.7); 62
				0.12+	1.7 (1.0 to 2.9); 60
Jarvholm and Stenberg, 2002‡[80]	33 719 male electricians and 72 653 male glass or woodworkers followed from 1971 to 1997 (Sweden)	Job title	Age, calendar year	Electricians	
				Compared with general population	0.6 (0.5 to 0.7); 95
				Compared with glass- and woodworkers	0.7 (0.6 to 0.9); 95
van Wijngaarden, 2003[81]	11 707 suicides and 132 771 deceased controls identified from death registry 1991–1992 (USA)	Job titles grouped according to presumed electromagnetic field exposure	Age, gender, race, marital status, geographic area, education	Occupational category	
				Not exposed	1.0 (–); 11,283
				Exposed§	1.3 (1.2 to 1.4); 424

*Observed number of suicide deaths; †standardised mortality ratios (SMRs), confidence intervals computed from information provided in original paper; ‡standardised mortality ratio; §electrical and electronics engineers; electrical, household and telephone repairers; electrical and electronics technicians; broadcast equipment operators; electricians and electrician apprentices; power plant operators; and motion picture projectionists.

improved through the use of Bayesian or Monte Carlo methods.[102–104]

Combining results across different studies

The proliferation of new EMF studies in recent years provides excellent opportunities for combining data across studies. Although attempts have been made to combine results using meta-analysis of summary results from published epidemiological studies of EMF-exposed workers,[18] the only effort to perform analyses of actual data sets pooled from multiple studies was attempted for the utility worker cohorts.[14] Pooled analyses can avoid many difficulties associated with meta-analytic techniques but require that participating investigators develop standardised protocols for reconciling study differences, for example, exposure assessment protocols and information on confounding factors and co-exposures.[101]

RESEARCH RECOMMENDATIONS

Much has been learned from occupational EMF epidemiology; however, several issues remain to be resolved. Research should employ improved case ascertainment and diagnosis, better exposure assessment, and better assessment of potential confounding factors. Future studies should also be large enough to investigate modifying factors such as age at disease onset, sex and the effect of exposure timing.

The highest occupational EMF research priorities emerge in the areas of exposure assessment and investigation of specific health outcomes.

Occup Environ Med: first published as 10.1136/oem.2007.037994 on 19 September 2008. Downloaded from http://oem.bmj.com/ on November 20, 2021 at Universita di Torino. Protected by copyright.

Health outcomes

The most promising research would focus on clarifying the reported association of electrical occupations with ALS. As ALS is almost invariably fatal within a few years of diagnosis, ALS deaths in the cohort mortality studies are likely to closely reflect incidence. However, extremely low incidence presents a major difficulty in studying ALS, even over a protracted period within a single cohort. Thus, the number of cases for analysis in individual studies has been small. A pooled analysis of previous cohorts, combined with a JEM refined to the extent possible for past job descriptions, could give a better indication of whether a true causal factor or the presence of biases produced the observations published to date.

Exposure assessment

Methods for exposure assessment remain among the most influential determinants of study quality; thus, broader and more precise exposure assessment can be an important advancement. First, the improved method developed to quantify magnetic field exposure for workers in power stations should be pursued in future studies of existing electric utility cohorts. New studies should include detailed information from interviews or company records for groups of jobs with regard to field sources encountered during work, the average strength of these sources (depending on the facility), and the proportion of time spent at different locations relative to sources. To estimate exposures for each subject, algorithms that combine this information with existing EMF exposure data need to be developed and validated against workplace measurements. Second, methods for assessing exposure to electric fields, spark discharge and contact current are worthy of further research, as is an investigation of alternative exposure metrics for magnetic fields. With new data, we can develop a more complete JEM that combines job title, work environment and task, and an index of exposure to electric fields, magnetic fields, spark discharge, contact current, and other chemical and physical agents.

CONCLUSIONS

Although the epidemiological evidence suggests excess risk with occupational EMF exposure for some health outcomes, there is no outcome for which the combined evidence is strong or consistent enough to support the conclusion that a health hazard exists. For some health outcomes, results from different studies are inconsistent; for others, the evidence is fragile because of small numbers and poor precision, or because of the inherent difficulties of studying particular conditions and exposures. In vitro, in vivo or mechanistic evidence has not provided clues for further investigation in occupational epidemiology. ALS is deemed the highest priority health outcome to pursue in future studies, along with improvements in exposure assessment. The development and application of methods to determine the presence and magnitude of biases using sensitivity analyses also deserves further consideration.

Acknowledgements: We thank the Energy Networks Association (ENA) and all participants at the ENA-sponsored workshop Future Needs of Occupational ELF Epidemiology for their contributions. Special thanks to Gail Lundell for expert scientific editing.

Competing interests: This paper is based in part on deliberations at a workshop sponsored by ENA, a UK trade association. L Kheifets and JM Harrington organised and chaired the workshop, with other co-authors as participants. R Kavet and G Mezei are employed by the Electric Power Research Institute; DC Renew is employed by National Grid plc and worked on this paper with the company's permission.

Disclaimers: The findings and conclusions in this paper have not been formally disseminated by the U.S. National Institute for Occupational Safety and Health and should not be construed to represent any agency determination or policy. The views expressed in this paper are those of the authors and not necessarily those of National Grid.

REFERENCES

1. **Sahl JD,** Kelsh MA, Greenland S. Cohort and nested case-control studies of hematopoietic cancers and brain cancer among electric utility workers. *Epidemiology* 1993;**4**:104–14.
2. **Theriault G,** Goldberg M, Miller AB, *et al*. Cancer risks associated with occupational exposure to magnetic fields among electric utility workers in Ontario and Quebec, Canada, and France: 1970–1989. *Am J Epidemiol* 1994;**139**:550–72.
3. **Savitz DA,** Loomis DP. Magnetic field exposure in relation to leukemia and brain cancer mortality among electric utility workers. *Am J Epidemiol* 1995;**141**:123–34.
4. **Savitz DA,** Checkoway H, Loomis DP. Magnetic field exposure and neurodegenerative disease mortality among electric utility workers. *Epidemiology* 1998;**9**:398–404.
5. **Sorahan T,** Nichols L, van Tongeren M, *et al*. Occupational exposure to magnetic fields relative to mortality from brain tumours: updated and revised findings from a study of United Kingdom electricity generation and transmission workers, 1973–97. *Occup Environ Med* 2001;**58**:626–30.
6. **Harrington JM,** Nichols L, Sorahan T, *et al*. Leukaemia mortality in relation to magnetic field exposure: findings from a study of United Kingdom electricity generation and transmission workers, 1973–97. *Occup Environ Med* 2001;**58**:307–14.
7. **Johansen C,** Raaschou Nielsen O, Olsen JH, *et al*. Risk for leukaemia and brain and breast cancer among Danish utility workers: a second follow-up. *Occup Environ Med* 2007;**64**:782–4.
8. **Floderus B,** Persson T, Stenlund C. Magnetic-field exposures in the workplace: reference distribution and exposures in occupational groups. *Int J Occup Environ Health* 1996;**2**:226–38.
9. **Hakansson N,** Floderus B, Gustavsson P, *et al*. Cancer incidence and magnetic field exposure in industries using resistance welding in Sweden. *Occup Environ Med* 2002;**59**:481–6.
10. **Forssen UM,** Rutqvist LE, Ahlbom A, *et al*. Occupational magnetic fields and female breast cancer: a case-control study using Swedish population registers and new exposure data. *Am J Epidemiol* 2005;**161**:250–9.
11. **Feychting M,** Pedersen NL, Svedberg P, *et al*. Dementia and occupational exposure to magnetic fields. *Scand J Work Environ Health* 1998;**24**:46–53.
12. **Wertheimer N,** Leeper E. Electrical wiring configurations and childhood cancer. *Am J Epidemiol* 1979;**109**:273–84.
13. **Milham S Jr.** Mortality from leukemia in workers exposed to electrical and magnetic fields. *N Engl J Med* 1982;**307**:249.
14. **Kheifets LI,** Gilbert ES, Sussman SS, *et al*. Comparative analyses of the studies of magnetic fields and cancer in electric utility workers: studies from France, Canada, and the United States. *Occup Environ Med* 1999;**56**:567–74.
15. **Roosli M,** Lortscher M, Egger M, *et al*. Leukaemia, brain tumours and exposure to extremely low frequency magnetic fields: cohort study of Swiss railway employees. *Occup Environ Med* 2007;**64**:553–9.
16. **Kheifets LI,** Afifi AA, Buffler PA, *et al*. Occupational electric and magnetic field exposure and brain cancer: a meta-analysis. *J Occup Environ Med* 1995;**37**:1327–41.
17. **Kheifets LI,** Afifi AA, Buffler PA, *et al*. Occupational electric and magnetic field exposure and leukemia. A meta-analysis. *J Occup Environ Med* 1997;**39**:1074–91.
18. **Kheifets L,** Monroe J, Vergara X, *et al*. Occupational electromagnetic fields and leukemia and brain cancer: an update to two meta-analyses. *J Occup Environ Med* 2008;**50**:677–88.
19. **WHO**. *Environmental health criteria 238. Extremely low frequency fields*. Geneva: World Health Organization, 2007.
20. **IARC**. *Non-ionizing radiation, part 1: static and extremely low frequency (ELF) electric and magnetic fields*. IARC monograph vol 80. Lyon, France: International Agency for Research on Cancer, 2002.
21. **Demers PA,** Thomas DB, Rosenblatt KA, *et al*. Occupational exposure to electromagnetic fields and breast cancer in men. *Am J Epidemiol* 1991;**134**:340–7.
22. **Rosenbaum PF,** Vena JE, Zielezny MA, *et al*. Occupational exposures associated with male breast cancer. *Am J Epidemiol* 1994;**139**:30–6.
23. **Pollan M,** Gustavsson P, Floderus B. Breast cancer, occupation, and exposure to electromagnetic fields among Swedish men. *Am J Ind Med* 2001;**39**:276–85.
24. **Stenlund C,** Floderus B. Occupational exposure to magnetic fields in relation to male breast cancer and testicular cancer: a Swedish case-control study. *Cancer Causes Control* 1997;**8**:184–91.
25. **Kheifets LI,** Matkin CC. Industrialization, electromagnetic fields, and breast cancer risk. *Environ Health Perspect* 1999;**107**(Suppl 1):145–54.
26. **Coogan PF,** Clapp RW, Newcomb PA, *et al*. Occupational exposure to 60-hertz magnetic fields and risk of breast cancer in women. *Epidemiology* 1996;**7**:459–64.
27. **Forssen UM,** Feychting M, Rutqvist LE, *et al*. Occupational and residential magnetic field exposure and breast cancer in females. *Epidemiology* 2000;**11**:24–9.
28. **Feychting M,** Forssen U. Electromagnetic fields and female breast cancer. *Cancer Causes Control* 2006;**17**:553–8.
29. **Kliukiene J,** Tynes T, Andersen A. Residential and occupational exposures to 50-Hz magnetic fields and breast cancer in women: a population-based study. *Am J Epidemiol* 2004;**159**:852–61.

Occup Environ Med: first published as 10.1136/oem.2007.037994 on 19 September 2008. Downloaded from http://oem.bmj.com/ on November 20, 2021 at Universita di Torino. Protected by copyright.

30. **van Wijngaarden E,** Savitz DA, Kleckner RC, *et al*. Mortality patterns by occupation in a cohort of electric utility workers. *Am J Ind Med* 2001;**40**:667–73.
31. **Gardner KM,** Ou Shu X, Jin F, *et al*. Occupations and breast cancer risk among Chinese women in urban Shanghai. *Am J Ind Med* 2002;**42**:296–308.
32. **Linet MS,** Malker HS, McLaughlin JK, *et al*. Non-Hodgkin's lymphoma and occupation in Sweden: a registry based analysis. *Br J Ind Med* 1993;**50**:79–84.
33. **Armstrong B,** Theriault G, Guenel P, *et al*. Association between exposure to pulsed electromagnetic fields and cancer in electric utility workers in Quebec, Canada, and France. *Am J Epidemiol* 1994;**140**:805–20.
34. **Schroeder JC,** Savitz DA. Lymphoma and multiple myeloma mortality in relation to magnetic field exposure among electric utility workers. *Am J Ind Med* 1997;**32**:392–402.
35. **Cano MI,** Pollan M. Non-Hodgkin's lymphomas and occupation in Sweden. *Int Arch Occup Environ Health* 2001;**74**:443–9.
36. **Charles LE,** Loomis D, Shy CM, *et al*. Electromagnetic fields, polychlorinated biphenyls, and prostate cancer mortality in electric utility workers. *Am J Epidemiol* 2003;**157**:683–91.
37. **Tynes T,** Reitan JB, Andersen A. Incidence of cancer among workers in Norwegian hydroelectric power companies. *Scand J Work Environ Health* 1994;**20**:339–44.
38. **Tynes T,** Klaeboe L, Haldorsen T. Residential and occupational exposure to 50 Hz magnetic fields and malignant melanoma: a population based study. *Occup Environ Med* 2003;**60**:343–7.
39. **Band PR,** Le ND, Fang R, *et al*. Identification of occupational cancer risks in British Columbia: a population-based case-control study of 769 cases of non-Hodgkin's lymphoma analyzed by histopathology subtypes. *J Occup Environ Med* 2004;**46**:479–89.
40. **Santibanez M,** Bolumar F, Garcia AM. Occupational risk factors in Alzheimer's disease: a review assessing the quality of published epidemiological studies. *Occup Environ Med* 2007;**64**:723–32.
41. **Jin YP,** Gatz M, Johansson B, *et al*. Sensitivity and specificity of dementia coding in two Swedish disease registries. *Neurology* 2004;**63**:739–41.
42. **Savitz DA,** Loomis DP, Tse CK. Electrical occupations and neurodegenerative disease: analysis of U.S. mortality data. *Arch Environ Health* 1998;**53**:71–4.
43. **Johansen C.** Exposure to electromagnetic fields and risk of central nervous system disease in utility workers. *Epidemiology* 2000;**11**:539–43.
44. **Noonan CW,** Reif JS, Yost M, *et al*. Occupational exposure to magnetic fields in case-referent studies of neurodegenerative diseases. *Scand J Work Environ Health* 2002;**28**:42–8.
45. **Hakansson N,** Gustavsson P, Johansen C, *et al*. Neurodegenerative diseases in welders and other workers exposed to high levels of magnetic fields. *Epidemiology* 2003;**14**:420–6; discussion 427–8.
46. **Feychting M,** Jonsson F, Pedersen NL, *et al*. Occupational magnetic field exposure and neurodegenerative disease. *Epidemiology* 2003;**14**:413–19; discussion 427–8.
47. **Park RM,** Schulte PA, Bowman JD, *et al*. Potential occupational risks for neurodegenerative diseases. *Am J Ind Med* 2005;**48**:63–77.
48. **Sorahan T,** Kheifets L. Mortality from Alzheimer's, motor neurone and Parkinson's disease in relation to magnetic field exposure: findings from the study of UK electricity generation and transmission workers, 1973–2004. *Occup Environ Med* 2007;**64**:820–6.
49. **Roosli M,** Lortscher M, Egger M, *et al*. Mortality from neurodegenerative disease and exposure to extremely low-frequency magnetic fields: 31 years of observations on Swiss railway employees. *Neuroepidemiology* 2007;**28**:197–206.
50. **Ahlbom A,** Cardis E, Green A, *et al*. Review of the epidemiologic literature on EMF and health. *Environ Health Perspect* 2001;**109**(Suppl 6):911–33.
51. **Graves AB,** Rosner D, Echeverria D, *et al*. Occupational exposure to electromagnetic fields and Alzheimer disease. *Alzheimer Dis Assoc Disord* 1999;**13**:165–70.
52. **Qiu C,** Fratiglioni L, Karp A, *et al*. Occupational exposure to electromagnetic fields and risk of Alzheimer's disease. *Epidemiology* 2004;**15**:687–94.
53. **Seidler A,** Geller P, Nienhaus A, *et al*. Occupational exposure to low frequency magnetic fields and dementia: a case-control study. *Occup Environ Med* 2007;**64**:108–14.
54. **Davanipour Z,** Tseng CC, Lee PJ, *et al*. A case-control study of occupational magnetic field exposure and Alzheimer's disease: results from the California Alzheimer's Disease Diagnosis and Treatment Centers. *BMC Neurol* 2007;**7**:13.
55. **Sobel E,** Davanipour Z, Sulkava R, *et al*. Occupations with exposure to electromagnetic fields: a possible risk factor for Alzheimer's disease. *Am J Epidemiol* 1995;**142**:515–24.
56. **Sobel E,** Dunn M, Davanipour Z, *et al*. Elevated risk of Alzheimer's disease among workers with likely electromagnetic field exposure. *Neurology* 1996;**47**:1477–81.
57. **Davanipour Z,** Sobel E, Bowman JD, *et al*. Amyotrophic lateral sclerosis and occupational exposure to electromagnetic fields. *Bioelectromagnetics* 1997;**18**:28–35.
58. **Deapen DM,** Henderson BE. A case-control study of amyotrophic lateral sclerosis. *Am J Epidemiol* 1986;**123**:790–9.
59. **Gunnarsson LG,** Lindberg G, Soderfeldt B, *et al*. Amyotrophic lateral sclerosis in Sweden in relation to occupation. *Acta Neurol Scand* 1991;**83**:394–8.
60. **Gunnarsson LG,** Bodin L, Soderfeldt B, *et al*. A case-control study of motor neurone disease: its relation to heritability, and occupational exposures, particularly to solvents. *Br J Ind Med* 1992;**49**:791–8.
61. **Johansen C,** Olsen JH. Mortality from amyotrophic lateral sclerosis, other chronic disorders, and electric shocks among utility workers. *Am J Epidemiol* 1998;**148**:362–8.
62. **Kheifets L,** Ahlbom A, Johansen C, *et al*. Extremely low-frequency magnetic fields and heart disease. *Scand J Work Environ Health* 2007;**33**:5–12.
63. **Graham C,** Sastre A, Cook MR, *et al*. Nocturnal magnetic field exposure: gender-specific effects on heart rate variability and sleep. *Clin Neurophysiol* 2000;**111**:1936–41.
64. **Graham C,** Sastre A, Cook MR, *et al*. Heart rate variability and physiological arousal in men exposed to 60 Hz magnetic fields. *Bioelectromagnetics* 2000;**21**:480–2.
65. **Graham C,** Sastre A, Cook MR, *et al*. Exposure to strong ELF magnetic fields does not alter cardiac autonomic control mechanisms. *Bioelectromagnetics* 2000;**21**:413–21.
66. **Graham C,** Cook MR, Sastre A, *et al*. Cardiac autonomic control mechanisms in power-frequency magnetic fields: a multistudy analysis. *Environ Health Perspect* 2000;**108**:737–42.
67. **Sastre A,** Cook MR, Graham C. Nocturnal exposure to intermittent 60 Hz magnetic fields alters human cardiac rhythm. *Bioelectromagnetics* 1998;**19**:98–106.
68. **Savitz DA,** Liao D, Sastre A, *et al*. Magnetic field exposure and cardiovascular disease mortality among electric utility workers. *Am J Epidemiol* 1999;**149**:135–42.
69. **Johansen C,** Feychting M, Moller M, *et al*. Risk of severe cardiac arrhythmia in male utility workers: a nationwide Danish cohort study. *Am J Epidemiol* 2002;**156**:857–61.
70. **Sahl J,** Mezei G, Kavet R, *et al*. Occupational magnetic field exposure and cardiovascular mortality in a cohort of electric utility workers. *Am J Epidemiol* 2002;**156**:913–18.
71. **Hakansson N,** Gustavsson P, Sastre A, *et al*. Occupational exposure to extremely low frequency magnetic fields and mortality from cardiovascular disease. *Am J Epidemiol* 2003;**158**:534–42.
72. **Ahlbom A,** Feychting M, Gustavsson A, *et al*. Occupational magnetic field exposure and myocardial infarction incidence. *Epidemiology* 2004;**15**:403–8.
73. **Sorahan T,** Nichols L. Mortality from cardiovascular disease in relation to magnetic field exposure: findings from a study of UK electricity generation and transmission workers, 1973–1997. *Am J Ind Med* 2004;**45**:93–102.
74. **Kurokawa Y,** Nitta H, Imai H, *et al*. Can extremely low frequency alternating magnetic fields modulate heart rate or its variability in humans? *Auton Neurosci* 2003;**105**:53–61.
75. **Wilson BW.** Chronic exposure to ELF fields may induce depression. *Bioelectromagnetics* 1988;**9**:195–205.
76. **van Wijngaarden E,** Savitz DA, Kleckner RC, *et al*. Exposure to electromagnetic fields and suicide among electric utility workers: a nested case-control study. *Occup Environ Med* 2000;**57**:258–63.
77. **Baris D,** Armstrong BG, Deadman J, *et al*. A case cohort study of suicide in relation to exposure to electric and magnetic fields among electrical utility workers. *Occup Environ Med* 1996;**53**:17–24.
78. **Baris D,** Armstrong B. Suicide among electric utility workers in England and Wales. *Br J Ind Med* 1990;**47**:788–9.
79. **Kelsh MA,** Sahl J D. Mortality among a cohort of electric utility workers, 1960–1991. *Am J Ind Med* 1997;**31**:534–44.
80. **Jarvholm B,** Stenberg A. Suicide mortality among electricians in the Swedish construction industry. *Occup Environ Med* 2002;**59**:199–200.
81. **van Wijngaarden E.** An exploratory investigation of suicide and occupational exposure. *J Occup Environ Med* 2003;**45**:96–101.
82. **Sahl JD,** Kelsh MA, Smith RW, *et al*. Exposure to 60 Hz magnetic fields in the electric utility work environment. *Bioelectromagnetics* 1994;**15**:21–32.
83. **Johansen C,** Raaschou-Nielsen O, Skotte J, *et al*. Validation of a job-exposure matrix for assessment of utility worker exposure to magnetic fields. *Appl Occup Environ Hyg* 2002;**17**:304–10.
84. **Forssen UM,** Mezei G, Nise G, *et al*. Occupational magnetic field exposure among women in Stockholm County, Sweden. *Occup Environ Med* 2004;**61**:594–602.
85. **Bowman JD,** Touchstone JA, Yost MG. A population-based job exposure matrix for power-frequency magnetic fields. *J Occup Environ Hyg* 2007;**4**:715–28.
86. **Kelsh MA,** Kheifets L, Smith R. The impact of work environment, utility, and sampling design on occupational magnetic field exposure summaries. *Am Ind Hyg Assoc J* 2000;**61**:174–82.
87. **Miller RD,** Neuberger JS, Gerald KB. Brain cancer and leukemia and exposure to power-frequency (50- to 60-Hz) electric and magnetic fields. *Epidemiol Rev* 1997;**19**:273–93.
88. **Renew DC,** Cook RF, Ball MC. A method for assessing occupational exposure to power-frequency magnetic fields for electricity generation and transmission workers. *J Radiol Prot* 2003;**23**:279–303.
89. **Bracken TD,** Senior R, Tuominen M. Evaluating occupational 60-hertz electric-field exposures for guideline compliance. *J Occup Environ Hyg* 2004;**1**:672–9.
90. **Swanson J,** Kheifets L. Biophysical mechanisms: a component in the weight of evidence for health effects of power-frequency electric and magnetic fields. *Radiat Res* 2006;**165**:470–8.
91. **Burch JB,** Reif JS, Yost MG, *et al*. Reduced excretion of a melatonin metabolite in workers exposed to 60 Hz magnetic fields. *Am J Epidemiol* 1999;**150**:27–36.
92. **Bowman JD,** Methner MM. Hazard surveillance for industrial magnetic fields: II. Field characteristics from waveform measurements. *Ann Occup Hyg* 2000;**44**:615–33.
93. **Armstrong BG,** Deadman JE, Theriault G. Comparison of indices of ambient exposure to 60-hertz electric and magnetic fields. *Bioelectromagnetics* 1990;**11**:337–47.
94. **Breysse PN,** Matanoski GM, Elliott EA, *et al*. 60 Hertz magnetic field exposure assessment for an investigation of leukemia in telephone lineworkers. *Am J Ind Med* 1994;**26**:681–91.
95. **Savitz DA,** Ohya T, Loomis DP, *et al*. Correlations among indices of electric and magnetic field exposure in electric utility workers. *Bioelectromagnetics* 1994;**15**:193–204.

Occup Environ Med: first published as 10.1136/oem.2007.037994 on 19 September 2008. Downloaded from http://oem.bmj.com/ on November 20, 2021 at Universita di Torino. Protected by copyright.

96. **Wenzl TB,** Kriebel D, Eisen EA, *et al*. Comparisons between magnetic field exposure indices in an automobile transmission plant. *Am Ind Hyg Assoc J* 1995;**56**:341–8.
97. **Zhang J,** Nair I, Sahl J. Effects function analysis of ELF magnetic field exposure in the electric utility work environment. *Bioelectromagnetics* 1997;**18**:365–75.
98. **Burstyn I.** Principal component analysis is a powerful instrument in occupational hygiene inquiries. *Ann Occup Hyg* 2004;**48**:655–61.
99. **Villeneuve PJ,** Agnew DA, Corey PN, *et al*. Alternate indices of electric and magnetic field exposures among Ontario electrical utility workers. *Bioelectromagnetics* 1998;**19**:140–51.
100. **Mant J,** Wilson S, Parry J, *et al*. Clinicians didn't reliably distinguish between different causes of cardiac death using case histories. *J Clin Epidemiol* 2006;**59**:862–7.
101. **Checkoway H,** Pearce N, Kriebe l D. *Research methods in occupational epidemiology*. 2nd edn. New York: Oxford University Press, 2004.
102. **Steenland K,** Greenland S. Monte Carlo sensitivity analysis and Bayesian analysis of smoking as an unmeasured confounder in a study of silica and lung cancer. *Am J Epidemiol* 2004;**160**:384–92.
103. **Kriebel D,** Zeka A, Eisen EA, *et al*. Quantitative evaluation of the effects of uncontrolled confounding by alcohol and tobacco in occupational cancer studies. *Int J Epidemiol* 2004;**33**:1040–5.
104. **Stromberg U,** Bjork J, Feychting M, *et al*. [True or false findings? Application of Bayes principles can improve the assessment]. *Lakartidningen* 2007;**104**:583–7.

Stay a step ahead with *Online First*

We publish all our original articles online before they appear in a print issue. This means that the latest clinical research papers go straight from acceptance to your browser, keeping you at the cutting edge of medicine. We update the site weekly so that it remains as topical as possible. Follow the Online First link on the home page and read the latest research.

Occup Environ Med: first published as 10.1136/oem.2007.037994 on 19 September 2008. Downloaded from http://oem.bmj.com/ on November 20, 2021 at Universita di Torino. Protected by copyright.
