# Supplementary material for: Magnetic Fields and Cancer: Epidemiology, Cellular Biology, and Theranostics
Source: Int J Mol Sci. 2022 Jan 25;23(3):1339. doi: 10.3390/ijms23031339 (PMC8835851; doi:10.3390/ijms23031339)
Supplement: Supplementary file 1 [file ijms-23-01339-s001.zip › Supplementary Data Set S1/MF and Cancer.Data/PDF/0072669420/nihms743249.pdf]

Author Manuscript
Author Manuscript
Author Manuscript
Author Manuscript

HHS Public Access
Author manuscript
Am J Ind Med. Author manuscript; available in PMC 2015 December 22.

Published in final edited form as:
Am J Ind Med. 2013 July ; 56(7): 791–805. doi:10.1002/ajim.22129.

Possible Health Benefits From Reducing Occupational Magnetic Fields

Joseph D. Bowman, PhD[1,*], **Tapas K. Ray, PhD**[2], and **Robert M. Park, MS**[3]

[1]Engineering and Physical Hazards Branch, National Institute for Occupational Safety and Health (NIOSH), Cincinnati, Ohio

[2]Organizational Sciences and Human Factors Branch, NIOSH, Cincinnati, Ohio

[3]Risk Evaluation Branch, NIOSH, Cincinnati, Ohio

Abstract

Background—Magnetic fields (MF) from AC electricity are a Possible Human Carcinogen, based on limited epidemiologic evidence from exposures far below occupational health limits.

Methods—To help formulate government guidance on occupational MF, the cancer cases prevented and the monetary benefits accruing to society by reducing workplace exposures were determined. Life-table methods produced Disability Adjusted Life Years, which were converted to monetary values.

Results—Adjusted for probabilities of causality, the expected increase in a worker's disability-free life are 0.04 year (2 weeks) from a 1 microtesla (μT) MF reduction in average worklife exposure, which is equivalent to $5,100/worker/μT in year 2010 U.S. dollars (95% confidence interval $1,000–$9,000/worker/μT). Where nine electrosteel workers had 13.8 μT exposures, for example, moving them to ambient MFs would provide $600,000 in benefits to society (uncertainty interval $0–$1,000,000).

Conclusions—When combined with the costs of controls, this analysis provides guidance for precautionary recommendations for managing occupational MF exposures.

Keywords

risk assessment; EMF; brain cancer; leukemia; DALY

INTRODUCTION

After electric and magnetic fields (EMF) at extremely low frequencies (ELF = 3–3,000 Hz) were declared a Possible Human Carcinogen [Kriteriegruppen for Fysickliska Riskfactorer, 1995; Portier and Wolfe, 1998; NRPB, 2001; IARC, 2002; Neutra et al., 2002; WHO, 2007], public health agencies faced difficult decisions on managing high-voltage transmission lines

*Correspondence to: Joseph Bowman, PhD, Mailstop R-5, 4676 Columbia Parkway, Cincinnati, OH 45226. jbowman@cdc.gov.

Disclosure statement: The findings and conclusions in this paper have not been formally disseminated by the National Institute for Occupational Safety and Health and should not be construed to represent any agency determination or policy.

Additional supporting information may be found in the online version of this article.

Author Manuscript Author Manuscript Author Manuscript Author Manuscript

and other sources of high EMF in homes, schools, and workplaces. There has been "limited epidemiologic evidence" of associations of ELF magnetic fields (MF) with childhood leukemia [Kriteriegruppen for Fysickliska Riskfactorer, 1995; Portier and Wolfe, 1998; NRPB, 2001; Neutra et al., 2002; IARC, 2002; WHO, 2007] and adult cancers [Portier and Wolfe, 1998; Neutra et al., 2002], but few toxicological and mechanistic studies provided support for a causal association [WHO, 2007]. Reviews of ELF-MF health risks have also noted epidemiologic associations with neurodegenerative diseases [Neutra et al., 2002; Kheifets et al., 2008a] and miscarriages [Neutra et al., 2002].

Most standard-setting organizations base their EMF exposure limits on acute neurological effects, while the cancer evidence is not generally considered sufficient to reduce exposures [IEEE, 2002; ACGIH, 2006; ICNIRP, 2010]. Consequently, MF exposure limits are more than a thousand times higher than the magnitudes associated with the cancer risks observed in epidemiologic studies, leaving millions of workers exposed to MF in this large gray area where the public health consequences are unclear.

For example, the National Institute for Occupational Safety and Health (NIOSH) has conducted three Health Hazard Evaluations (HHE) in which MF exposures fell in this ambiguous realm (Table I). In one study of an electro-steel furnace [Moss and Booher, 1994], the maximum magnetic field exposures of the six operators ranged from 80 to 148 μT, an order of magnitude below the threshold limit value (TLV) of 1,000 μT for 60 Hz magnetic fields [ACGIH, 2006]. However, the time-weighted average (TWA) magnetic fields were 11–17 μT, an order of magnitude above 0.2–1.0 μT, where occupational epidemiologic studies have reported elevated cancer risks [Floderus et al., 1993; Savitz and Loomis, 1995; Kheifets et al., 1999]. The three HHE reports suggested reductions in MF exposures at these workplaces, but the rationales for these interventions varied (Table I) [Moss and Booher, 1994; Malkin and Moss, 1995; Moss and Ragab, 1995].

Evidence-based approaches to managing workplace magnetic fields are clearly needed. The World Health Organization recommends: "Providing the health, social, and economic benefits of electric power are not compromised, implementing very low-cost precautionary procedures to reduce exposures is reasonable and warranted" [WHO, 2007]. For homes and schools, cost-effective precautionary measures have been identified by cost-benefit analyses of the childhood leukemia risks [Swedish National Board for Occupational Safety Health et al., 1995; California EMF Program, 2002a; von Winterfeldt et al., 2004; WHO, 2007].

In order to extend this precautionary approach to workplaces, we conducted a risk assessment on MF carcinogenesis and calculated the disease burden from magnetic field exposures, expressed as Disability-adjusted Life Years (DALY) lost. Because DALYs measure pain and suffering from the disease plus potential losses in productivity resulting from premature death, the monetary equivalent of intervention benefits equals the change in DALYs multiplied by a suitable value for a disability-free year of life [Kassouf et al., 2005; Forbes et al., 2006]. Under the utilitarian approach to public health policy [Swedish National Board for Occupational Safety Health et al., 1995; California EMF Program, 2002a; von Winterfeldt et al., 2004], an intervention is in the public interest when the potential reduction

in DALYs or gain in their monetary equivalent is greater than the associated intervention costs.

Another important component in practical precautionary recommendations is setting an exposure level below which health risks are minimal so mitigation efforts would not be justified. In setting this "precautionary level" (PL) for occupational MF, the most useful risk metrics for an exposure are (1) DALYs, a measure of the expected personal and economic costs, and (2) the cancer incident rate, a public health measure which NIOSH uses to establish recommended exposure limits [Reed et al., 1994]. After policy makers set *de minimis* levels for one of these risk metrics, a MF precautionary limit can be derived from the plots in this article. Our intention with this risk assessment is not to recommend mandatory exposure limits, but to help determine which MF exposures might reasonably be reduced under the precautionary principle.

With a determination of the interventions that are cost-effective, well-established strategies for managing workplace hazards can then be recommended for occupational MF [Bolte and Pruppers, 2006; Patterson and Hitchcock, 2008].

METHODS

Our methods for calculating DALYs and the monetary costs of a workplace MF exposure are outlined in Figure 1. The risk assessment concludes with uncertainty and sensitivity analyses of the calculated metrics. Our rationale for these procedures is given below, and the mathematical details accompany this article as Supplemental Online Material [See Section A,B,C,D,E,F in the Supplemental Information].

Disease Selection

To include a disease in this risk assessment, we required 1) a finding by a governmental or international authority that occupational ELF magnetic fields are a possible risk factor (equivalent to IARC's Group IIB carcinogens); and 2) a significant dose–response with magnetic field measurements found by pooling data from multiple workplace studies. Brain cancer and leukemia fulfill these two criteria. The California EMF Project conducted by California's health department found MF to be a Possible Human Carcinogen (Group IIB), based on epidemiologic associations with adult brain cancer and leukemia [Neutra et al., 2002]. Furthermore, limited epidemiologic evidence for leukemia in workplace studies was a factor in the Group IIB evaluations given by the Swedish Criteria Group [Kriteriegruppen for Fysickliska Riskfactorer, 1995] and a working group convened by the National Institute of Environmental Health Sciences [Portier and Wolfe, 1998]. Dose–response relationships were found for these two cancers in a comparative analysis [Kheifets et al., 1999] of four electric utility studies with full-shift personal MF monitoring [Sahl et al., 1993; Theriault et al., 1994; Savitz and Loomis, 1995; Miller et al., 1996].

Dose–Response Relationships

Kheifets et al. [1999] calculated dose–response (DR) relationships for brain cancer and leukemia with an exponential model:

Author Manuscript Author Manuscript Author Manuscript Author Manuscript

Author Manuscript Author Manuscript Author Manuscript Author Manuscript

$$RR = \exp(\beta C) = RR'^{C/10} \quad (1)$$

where C is the worker's cumulative MF exposure in μT-yr, and $RR' \equiv \exp(10\beta)$ is the DR slope expressed as a multiplier of the relative risk for each 10 μT-year increase in exposure [See Section A in the Supplemental Information]. Their results (Table II and Fig. 2) were significant for brain cancer ($P = 0.031$) and marginally significant for leukemia ($P = 0.050$).

NIOSH Exposure Scenario for Risk Assessments

NIOSH risk assessments calculate excess lifetime risk by specifying a constant exposure from starting work at age 20 until retiring up to 45 years later [Reed et al., 1994]. While meaningful over the range of cumulative exposures observed in the electric utility studies (Fig. 2), the exponential function (Eq. 1) under the NIOSH exposure scenario gives unrealistic RRs of over 100 for C > 45 μT-years, which would result from 45 years of work at the TWA MFs greater than 1 μT observed by NIOSH Health Hazard Evaluations (Table I).

This breakdown in the exponential model at higher cumulative exposures partly reflects the low duration of employment in the utility cohorts (means = 18.5–24.9 years) [Kheifets et al., 1999]. This employment in the study cohorts is clearly less than the workers' total work careers since the median age of cancer diagnosis or death ranged from 58 to 64 years [Kheifets et al., 1999], suggesting median work careers among cases of nearly 40 years. Therefore, the cumulative MF calculated from the utility data possibly underestimates the workers' true exposures including those from previous employment, resulting in over-estimates of the cancer risks from the exponential model.

Kheifets et al. [1999] used the exponential model for mathematical convenience with logistic regression, but a linear model is usually preferable for carcinogenesis. We therefore selected a linear model RR(C) = a + b C to represent the higher cumulative exposures. The slope and intercept of the linear model were chosen to match the exponential model and its slope at the relative risk in the highest exposure category from the electric utility studies (Table II and Fig. 2) [See Section A in the Supplemental Information].

Lifetime Cancer Incidence and Mortality

The life-table method was then used to calculate cause-specific lifetime mortality and incidence from a given MF exposure [BEIR, 1988]. Data for this calculation included mortality rates for the U.S. population [Arias, 2006], and cancer incidence and mortality rates from National Center for Health Statistics (NCHS) [SEER, 2006a,b].

We made two modifications to the life-table formulas for calculating excess mortality and cancer incidence among workers [BEIR, 1988]. First, all-cause mortality rates were adjusted for excess deaths with the MF exposure resulting from *two* diseases (brain cancer and leukemia) [See Section B in the Supplemental Information]. Second, rates for cancer mortality and incidence were adjusted for the cancers predicted to result from occupational MF exposures in the U.S. population [See Section B in the Supplemental Information]. For our population-based calculations, the best available occupational MF data are

Author Manuscript Author Manuscript Author Manuscript Author Manuscript

measurements from a 1,000-person randomized survey in the U.S. [Zaffanella, 1998]. From published descriptive statistics on the 525 working subjects, we derived a log-normal distribution of U.S. occupational MF exposures, which were used to adjust the reported cancer mortality and incidence rates to "non-exposed" levels [See Section B in the Supplemental Information].

Life-table methods were used to calculate lifetime cancer incidence and mortality with and without MF exposure. To obtain DALYs, we also calculated the excess incidence for leukemia subtypes [See Section B in the Supplemental Information].

Disability-Adjusted Life Years (DALY)

Developed by Murray and Lopez [1996a], the DALY combines measurements of premature death and disability to quantify the disease burden to human life. DALY extends the concept of potential years of life lost (YLL) resulting from premature death to include equivalent "years lived with disability" (YLD) by virtue of being in states of ill health. For a typical disease or health condition, DALYs are the sum of YLL and YLD. Therefore, a disease which causes great disability during and after treatment has a greater DALY than a disease with the same effect on life expectancy but little disability.

In order to calculate the DALY in this case, published age-specific life-expectancies [Arias, 2006] were multiplied by the excess cancer mortality for a MF exposure, B, from both cancers and summed across all ages from 20 up to 90 years of age to obtain the YLL(B) [See Section C in the Supplemental Information]. Next, YLD was calculated according to a "burden of disease" study from Victoria, Australia Victoria [Victoria Public Health Group, 1999ab]. The Victoria study is largely based on the methods of the Global Burden of Disease Study at the World Health Organization (WHO) [Murray and Lopez, 1996a]. To predict the lifetime YLD from a given MF exposure, age-dependent excess incidences were calculated by the life-table method for brain cancer and the five leukemia sub-types and then inserted into the YLD formulas from the Victorian study [Victoria Public Health Group, 1999a]. The resulting YLD(B) is simply added to YLL to get the DALY per person exposed to a TWA magnetic field B: DALY(B) = YLL(B) + YLD(B).

Discounted DALYs

Health economists often place a positive discount rate on future health effects, based on the opportunity costs of health investment, the diminishing marginal utility of life, and people's preference to have good health earlier rather than later in life [Weinstein, 1990; Olsen, 1993; Murray and Lopez, 1996a,b]. A 3% discount rate was used for cost effectiveness analyses as recommended by a multi-disciplinary "consensus panel" convened by the US Public Health Service [Lipscomb et al., 1996]. The discounted summed DALYs for brain cancer and leukemia are the average disease burden attributable to a MF exposure level. The difference in the discounted DALYs before and after the intervention is the predicted effectiveness of the MF reduction.

DALYs are often age weighted so as to provide more prominence to the young and mid adult life in terms of social responsibilities and productivity [Murray and Lopez, 1996b]. Since subjects in this study are predominantly of working age, age weighting according to

this Human Capital approach would imply valuing their productivity differently. As the study is not controlling for other related factors like education, occupation, and consumption pattern of individuals, we refrain from weighting DALYs according to age.

Monetary Benefits From the Reduction of Magnetic Field Exposures

Most economists and policy analysts agree on the general principle that the life-saving benefits from a preventive activity should be compared to its costs. DALYs measure health burden, while preventive interventions are compared on the basis of their cost-effectiveness ($/DALY) ratios, the one with the lowest being the most economically effective. Although the costs of MF reduction measures are not known, the DALYs from the cancer burden are converted to monetary values so that the benefits accruing from interventions can be easily compared to costs when available.

Conversion of DALYs to immediate monetary terms by multiplying with the value of a year of life is not uncommon. This study uses a conceptually similar approach of equating the value of one DALY to the value of a statistical life year (VSLY). Therefore the monetary cancer burden, b$(B), from a magnetic field exposure, B, is:

$$\mathrm{b\$(B)=VSLY \times DALY(B)} \quad (2)$$

VSLY in turn can be derived from the value of a statistical life (VSL) divided by the life expectancy conditional on surviving the current mortality risks.

For the VSLY and consequently the value of the DALY, the value of $100,000 is used which the U.S. Food and Drug Administration placed on each year of life saved by nutrition labeling in 1999 [FDA, 1999]. Similar values for a life-year have been suggested by other investigators [Zarkin et al., 1993; Tolley et al., 1994; Cutler and Richardson, 1997]. As the DALYs are already discounted to their present value, monetary benefits are not further discounted. However, the monetary benefits obtained in 1999 dollars were then converted to 2010 dollars adjusting for the rate of change in inflation as reflected in the consumer price index [Bureau of Labor Statistics, 2011].

Finally, the discounted DALYs (as a fraction of a 45 year work life) and the incidence rates for brain cancer plus leukemia were plotted against TWA magnetic fields in order to derive candidate precautionary limits (PL) on exposure reduction efforts. As a demonstration of the PL concept, several *de minimis* values of risk were selected arbitrarily, and the corresponding PL calculated. In these demonstrations, the only *de minimis* risk value with an occupational health justification is the incidence rate of 1 cancer per 1,000 exposed, which NIOSH and OSHA use for setting exposure limits [Reed et al., 1994].

Decision Analysis

After the conventional risk assessment is complete, uncertainty in the underlying causal association is incorporated through decision analysis [DeKay et al., 2002]. In conventional cost-benefit analyses, an intervention is justified if the discounted dollar value of the future lives saved is greater than the cost of the controls (c), or in other words, the net benefits to

Author Manuscript Author Manuscript Author Manuscript Author Manuscript

society are greater than zero. In order to incorporate uncertainty about the MF-cancer association into this cost-benefit analysis, decision analysis assumes a probability P_c that the reported DR is a causal relationship. Then a decision tree (Fig. 3) can be constructed on the principle that costs of an intervention should be less than or equal to the predicted costs of averted cancer deaths. The resulting decision rule (Fig. 3) can then be expressed in terms of the expected value of the net benefits from a reduced cancer burden:

$$P_c \times [b_{\$}(B_{before}) - b_{\$}(B_{after})] \geq c \quad (3)$$

where the TWA magnetic fields B are estimated before and after the proposed intervention.

In setting a precautionary limit, the probability of causation is treated differently because a *policy decision* justifies interventions, rather than a cost-benefit analysis. For a chosen risk metric z (i.e., the cancer incidence or the economic burden from a TWA exposure B), the decision rule for intervening can be written in terms of its expected value under the scientific uncertainty:

$$E[z(B_{before})] = P_c\, z_{causal}(B_{before}) + (1 - P_c) z_{no\ causation}(B_{before}) = P_c\, z_{causal}(B_{before}) = z_{policy} \quad (4)$$

where $z_{no\ causation} = 0$ for any MF exposure, and the value of z_{policy} (e.g., 1 in 1,000 cancer incidence) is chosen by policy makers. A precautionary limit on B_{before} can then be derived from $P_c\ z_{causal}(B_{precaution}) = z_{policy}$.

Probability of Causality

To make this decision analysis a practical tool for decision makers, an objective method is needed for evaluating the probability P_c that reported epidemiologic associations are causal relationships. The California EMF Project performed a "qualitative Bayesian analysis" which provides estimates of P_c, based on the same scientific literature reviewed by IARC and the other governmental risk assessments [Neutra et al., 2002].

In this approach, the attributes of the epidemiologic and toxicology evidence used in conventional risk evaluations (strength of association, bias, confounding, consistency, extrapolation from animals to humans, etc.) were evaluated by three scientists from the California health department as to their support for the causal and "no effect" (null) hypotheses. Motivated by Bayes Theorem, the evaluation starts with a subjective evaluation of the probability "prior" to any research that EMF "is capable of altering the risk of one or more cancers or other diseases" [Neutra et al., 2002, p. 63]. If each piece of evidence was better explained by the causal hypothesis than the null hypothesis, then the probability increased; if not, it decreased [Neutra et al., 2002]. After the reviewers evaluated all evidence, they arrived at their "posterior probability" that the EMF exposure caused the disease. To be more understandable to the general public, the results were reported as the "degree of certainty" on a scale of 0–100 that "EMFs increase disease risks to some degree" [Neutra et al., 2002], which is clearly equivalent to 100 times the Bayesian posterior probability P_c used in decision theory [DeKay et al., 2002].

The three reviewers from the California health department assigned degrees of certainty of 80, 50, and 60 to adult brain cancers with "margins of uncertainty"—with a low of 30 and a

Author Manuscript Author Manuscript Author Manuscript Author Manuscript

Author Manuscript Author Manuscript Author Manuscript Author Manuscript

high of 100, [Neutra et al., 2002, p. 165]—giving an average score of 63. For adult leukemia, the results were 80, 55, and 40 with uncertainty margins from 25 to 95 [Neutra et al., 2002, p. 120], which averages 58. Rounding off to one decimal place, the mean posterior probabilities for adult leukemia and brain cancer are both $P_c \approx 0.6$ with an uncertainty from 0.2 to 1.0.

Uncertainty Analysis

For our uncertainty analysis, we first identified all sources of variability and error in our risk metrics and when possible, quantified the uncertainty of the input variables for the risk calculations (Table III). The quantified uncertainties fall into three groups: the DR parameters and the MF exposure distribution whose statistical properties can be rigorously characterized, sources of bias for which the data is suggestive, and the parameters for which we have only a range of possible values (the monetary value of the DALY, the discount rate, and the posterior probability) [See Section D in the Supplemental Information]. For the DR and exposure distribution parameters, our uncertainty analysis consisted of a rigorous propagation of errors, which resulted in 95% confidence limits (95% CL) and *P*-values from one-tailed hypothesis tests for the metrics [See Section E in the Supplemental Information].

For the more poorly characterized parameters, our uncertainty analysis also used the propagation of error formulas but with approximate biases and standard errors derived from the range of possible values [See Section D in the Supplemental Information]. The 95% probability limits around a bias-adjusted mean derived from these more approximate error estimates are called *uncertainty limits* (UL) [Phillips et al., 1997; See Section E in the Supplemental Information]. When a calculated uncertainty limit exceeds the null-hypothesis value, the convention is to report the null-hypothesis value as the uncertainty limit [Phillips et al., 1997].

For the precautionary levels, a similar uncertainty analysis was conducted [See Section F in the Supplemental Information].

RESULTS

Table IV presents the years of life lost (YLL), years lost to disability (YLD), the expected values for the total DALY = P_c DALY(B), and the lifetime economic burden (discounted and undiscounted) for a logarithmic series of magnetic field levels. The regression coefficient for the expected economic burden with discounting versus the magnetic field is $5,100 per μT per worker exposed (Table V), which also approximates the benefit from a 1 μT reduction in a worker's MF exposure. Considering random errors in the dose–response estimate, these effects are significant (95% CL = $1,000–$9,000/worker/μT; $P < 0.03$). When the posterior probability and all other quantified sources of uncertainty (Table III) are included in the sensitivity analysis [See Section E in the Supplemental Information], the uncertainty limits range from $0 (no effect) to $12,000, in the absence of chemical exposures (Table V). When the reported effects of mercury, lead, arsenic, solvents, pesticides, or herbicides are present, the upper uncertainty limit increases by $3,000 per chemical (Table V).

Author Manuscript

Examining the YLL and YLD results more closely (Table V), the years lost to disability from cancers attributable to workplace MF are a small fraction of the years of life lost (5% for brain cancer and 12% for leukemia for the discounted values). This relationship is due to the increasing excess in cancer incidence with age due to a constant workplace MF exposure [See Section B in the Supplemental Information, Table S-I]. With these cancers, the excess incidence peaks with workers in their 70s when cancer is more often fatal than disabling. The same result was observed with leukemia due to workplace benzene [Driscoll et al., 2005]. Between the two cancers, the brain cancer YLL is +7% greater than the leukemia YLL, which is not significant (Fig. 4). Discounting reduces the YLL substantially (73% for brain cancer and 75% for leukemia), which is reflected in a similar diminution of the DALYs. All metrics are very close to linear functions of the TWA magnetic fields ($R^2 >$ 0.99 in all cases). Since deviations from linearity in YLL are well within the 95% confidence limits (Fig. 4), economic burdens and their uncertainties can be calculated for specific TWA exposures from the slopes in Table V without sacrificing precision.

Similarly, precautionary limits (PL) are useful for managing workplace MF because lower TWAs are expected to have minimal risks and/or economic burdens (as defined by stakeholders). A *de minimis* cancer risk of 1 per 1,000 gives a PL of 0.28 μT (95% CL = 0.14–9.3 μT; $P = 0.02$; UL = 0.17–∞ μT) with the uncertainty limits going to 0.14–∞ μT in the presence of mercury [See Section E in the Supplemental Information]. Figure 5 shows other possible PLs ranging from 0.16 to 0.53 μT that can be derived from alternative minimal values for the cancer risk or the discounted cancer burden, but they all have wide over-lapping confidence intervals.

These findings can be applied to the examples in Table I. In all three workplaces, the TWA exposures are above the 0.28 μT PL, so quantitative evaluations of interventions are justified by the possible cancer risks. As shown in Figure 6 and Table VI, the benefits of interventions with the electrosteel furnace can be calculated by taking the difference in the expected burden before and after an intervention. If the furnace's control room were moved away from its transformer to a location with the ambient TWA ≈ 0.1 μT, exposures would be reduced by 13.6 μT, so the expectation for the discounted lifetime benefits = 13.6 μT × $5,100 per μT = $69,000 approximately per worker exposed. For all nine control room operators affected by this exposure reduction, the expected lifetime benefits of the intervention would be 9 × $69,000 = $600,000 (rounded off to one significant figure). Since the HHE report mentions no exposures to the five chemicals with MF interactions (Table IV), the uncertainty limits on the benefits are $0–$1,000,000. Under a utilitarian approach to public health, moving the control room would be in the public interest if the moving costs were less than the total benefits for all exposed workers. The expected workforce benefits for the interventions proposed by the other two Health Hazard Evaluations are $30,000 ($0–$60,000 uncertainty) for the tax office and $50,000 ($0–$100,000 uncertainty) for the TV station (Table VI).

DISCUSSION

This work attempts to quantify the health benefits from reducing exposures to occupational ELF magnetic fields, which some have been classified as a Possible Human Carcinogen

Author Manuscript Author Manuscript Author Manuscript Author Manuscript

[Kriteriegruppen for Fysickliska Riskfactorer, 1995; Portier and Wolfe, 1998; Neutra et al., 2002].

These findings are based on the current state of etiologic inference: statistically significant but controversial risks for leukemia and brain cancer at exposures well below the present exposure limits derived from proven biological effects. The uncertainty, arising in part from exposure assessment errors, encompasses a wide range of possible risk whose upper end would have very substantial public health impact. A similar challenge exists today with the radio-frequency EMF from cell-phones, which were recently declared a Possible Human Carcinogen [Baan et al., 2011]. Since the possible carcinogen rating does not meet most standards for legally binding regulations, we undertook this risk assessment as guidance for public health agencies to identify cost-effective recommendations for avoiding ELF-MF exposures under the Precautionary Principle [Resnik, 2003; Tickner et al., 2003].

Many steps in our approach (Fig. 1) are established methods in occupational health and risk assessment: evaluating a worker's lifetime cancer risks from TWA measurements on a single shift [Reed et al., 1994], using life-table methods, quantifying disease burden with DALYs, discounting the burden, and incorporating uncertainty with decision theory. Nonetheless, these steps involve assumptions and inherent sources of uncertainty, which are summarized in Table III. This discussion focuses on the steps in our risk assessment that involved more discretion: disease selection, the dose–response relationships, the monetary costs of a DALY, the posterior probability for causation, and our underlying premise that a cost-benefit analysis can provide useful guidance for pre-cautionary measures.

In choosing data for our quantitative risk assessment, the Precautionary Principle is that scientific confirmation may be too strict a requirement for precautionary public health interventions. Public health agencies must be concerned about false negatives (type II errors) delaying interventions that might prevent diseases and deaths. In our approach, science-based public health policies may recommend interventions without full confirmation of the risks when their expected benefits credibly outweigh their costs.

To develop public health recommendations in the absence of scientific certainty, cost-benefit analyses combined with decision analysis are a rational approach as long as the underlying evidence meets some standard of credibility [Resnik, 2003]. Our approach to managing EMF's possible occupational hazards is consistent with some formulations of the Precautionary Principle, although that philosophy is not explicitly incorporated into the U.S. occupational health law [Resnik, 2003; Tickner et al., 2003; Graham, 2004].

To assure credible scientific evidence of possible harm, we decided on two criteria for disease selection in the Methods Section, but other choices are possible. For example, the finding of Possible Human Carcinogen (or its equivalent for non-cancerous outcomes) could be required from the most recent risk evaluation. The most recent evaluation is WHO's criteria monograph, which decided occupational EMF is not a possible carcinogen [WHO, 2007]. However, a public health policy based on the most recent report would be liable to change from year to year. Furthermore, a single report, even from a prestigious agency like WHO, is liable to errors. For example, WHO's monograph cites only the null associations

Author Manuscript

Author Manuscript

Author Manuscript

Author Manuscript

from a Swedish brain cancer study [WHO, 2007, p. 294], ignoring relative risks as large as 3.91 (95% CI 1.26–12.15) for exposures to both lead and TWA MF above 0.2 μT [Navas-Acien et al., 2002]. Since the overall literature on occupational EMF is clearly split between seeing no cancer association or a small risk of brain cancer and leukemia [Kheifets et al., 1999], our requirement of a single credible risk evaluation is a prudent approach to disease selection.

In the other direction, a disease could be included in the risk assessment if a significant dose–response is found by one well-done study, rather than by a meta-analysis of multiple studies. However, occupational EMF epidemiology has produced a bewildering mixture of significant and non-significant DRs [Kheifets et al., 2008a], which is expected from the Berkson errors created by job-exposure matrices [Thomas et al., 1993]. Therefore, appropriate meta-analytic modeling of the primary data from several high-quality studies appears to be the most reliable means of determining whether an EMF dose–response (or its absence) is due to chance.

Our risk and burden estimates would be greater if we had included higher prevalence diseases such as neurodegenerative diseases, and miscarriages that have been occasionally associated with magnetic fields [Lee et al., 2002; Park et al., 2005]. We rejected that approach because the absence of comparative epidemiologic studies for these diseases would give their dose–response slopes with very large confidence intervals, making decisions from the resulting DALYs even more uncertain [NRC, 2009]. On the other hand, MF risks would appear to be zero if the selection criteria were "sufficient, reliable evidence to conclude that long-term exposures … are adverse to human health or cause a disease" plus a "confirmed mechanism that would provide a firm basis to predict adverse effects," as the Institute of Electrical and Electronic Engineers requires for its ELF-EMF exposure limits [IEEE, 2002]. We rejected this option for our precautionary risk assessment because it rejects *a priori* any evaluation of the possible cancer risks reported by the epidemiologic studies.

In selecting data on the dose–response, we chose the comparative analysis of four electric utility studies [Kheifets et al., 1999] because it provided the significant DR slopes required for a quantitative risk assessment. However, this study omits many high-quality studies of brain cancer and leukemia, both positive [Floderus et al., 1993; Hakansson et al., 2002] and negative [Johansen et al., 2007; Sorahan et al., 2001]. These newer studies were included in a recent meta-analysis [Kheifets et al., 2008b], which again reported elevated risks but now with no significant dose–response for the two cancers. However, this degraded significance could be due to the meta-analysis using only the published risks from diverse exposure categories for its dose–response calculations, which can lead to exposure misclassification and bias towards the null—a defect avoided by a comparative study of the primary data. Furthermore, Kheifets et al. [2008b] omitted the elevated brain cancer risks from combined chemical and MF exposures [Navas-Acien et al., 2002] as well as elevated leukemia risks from a combination of occupational and residential MFs in a Swedish cohort living along high-voltage transmission lines [Feychting et al., 1997]. Therefore, the comparative analysis [Kheifets et al., 1999] appears to be the best data available for our quantitative risk assessment.

Author Manuscript

Another issue is the choice of an effectiveness measure that combines information on both morbidity and mortality. The U.S. Office of Management and Budget (OMB) prefers QALYs, although it does mention DALYs [Brown et al., 2001]. We chose DALYs primarily because they were used by WHO's global burden of occupational disease study [Nelson et al., 2005], allowing us to later compare the impact of MFs with other occupational carcinogens.

An alternative method for measuring the economic burden of disease is the "cost of illness method," which computes morbidity and mortality costs along with direct costs (largely medical expenditure). While DALYS incorporate the former, the latter remains unattended. A 2001 burden of illness study estimated the direct costs of all cancer-related cases to be $96.1 billion for 1990 [Brown et al., 2001]. From 1963 to 1995, cancer-related direct costs in the U.S. have held stable at less than 5% of overall health care expenditures and are estimated to average $35,418 per person [Goldman et al., 2003]. This measure of cancer costs per DALY underestimates the true economic burden as it does not account for the medical costs associated with treatment of the diseases and the productivity losses associated with replacement of the workforce.

The risk evaluation by the California EMF Program [Neutra et al., 2002] that we used for the posterior probabilities has been heavily criticized [California EMF Program, 2002b; von Winterfeldt et al., 2004]. Since California's pioneering effort at Bayesian risk evaluation is the only existing effort to systematically quantify EMF's probability of causality, its findings are essential if possible interventions are to be evaluated with decision theory. With the sharp bifurcation of opinion within the scientific community on whether EMF causes cancer [Neutra et al., 2002; Blackman et al., 2007; WHO, 2007], a naïve application of decision theory would average just the two branches of the decision tree (Fig. 4), which is equivalent to a prior probability of 0.5. In this light, the California review's posterior probability of 0.6 represents a small, cautious upward adjustment, which is a reasonable position for a public health agency. Nonetheless, our risk estimates would be more reliable if the Bayesian risk evaluation method used by the California EMF Project were repeated by another organization with an up-to-date review of ELF-MF research.

The precautionary limits are based on *de minimis* values for the cancer risk and economic burden (Fig. 5) that are somewhat arbitrary with the exception of the 1:1,000 disease risk used by NIOSH to establish Recommend Exposure Limits (RELs) for occupational hazards [Reed et al., 1994]. However, RELs are recommended to OSHA as not-to-be-exceeded limits to assure safety from recognize hazards, and are clearly different from a precautionary limit that justifies cost-effective controls of a possible hazard. Therefore, policy makers and stakeholders should discuss what risks and/or monetary expenditures they consider to be *de minimis* for magnetic fields. The proposed precautionary limits are approximately three orders of magnitude less than the present exposure guidelines [IEEE, 2002; ACGIH, 2006; ICNIRP, 2010], but these relatively low levels are consistent with the TWA magnetic fields where cancer associations were reported by some occupational epidemiological studies [WHO, 2007].

Our examples of using the benefit estimates (Tables I and VI) are based on NIOSH's exposure reduction recommendations for existing workplaces, but these results can contribute in other ways to the development of precautionary recommendations for workplace MF. By obtaining estimates of an intervention's costs, formal cost-benefit analyses can be performed on both changes with existing equipment and purchases of new equipment in order to justify investments in lower MF exposures.

Furthermore, our benefit estimates could be used to create precautionary MF control bands. The economic burden of MF sources can be calculated from available exposure data, and then grouped by the commensurate intervention (behavioral controls, engineering controls, shielding, etc.). The qualitative control band approach has proven useful for managing many occupational hazards, especially with large uncertainties on the risks [NIOSH, 2009], and has been applied to assuring compliance with the European Union's standards for ELF and RF EMF [Bolte and Pruppers, 2006].

Such broad "rules-of-thumb" [Gigerenzer et al., 1999] are more easily understood and implemented than detailed cost-benefit analyses that are not always persuasive to workers and employers due to their mathematical complexity and large uncertainty limits. Moreover, cost-benefit analyses have controversial assumptions that stem from the lack of understanding and accounting for true long-term impacts both on the cost and benefits side [Ackerman, 2008]. Therefore, risk assessment experts and economists often prefer precautionary approach in making decisions under uncertainty [Tickner et al., 2003; Ackerman, 2008]. In light of the ±130% uncertainty around our ELF-MF benefit estimate, the decision rule in Figure 3 should not be applied rigidly to choosing an intervention, but instead provide an indication of the scale of justifiable expenditures.

An example of the interplay between a cost-benefit analysis and precautionary regulations was the 2004 ruling on new transmission lines in California. The California EMF Program commissioned a cost-benefit analysis of residential MF from powerlines [von Winterfeldt et al., 2004]. However, its recommendations were never used by the California Public Utility Commission. Instead they simply decreed that 4% of the costs of new transmission lines be used for the mitigation of EMF exposures without any supporting data [California Public Utility Commission, 2006]. Nonetheless, this precautionary measure on new transmission lines has endured as an important policy resulting from the era of the California EMF Program.

Whether our benefit analysis will make precautionary recommendations for workplaces persuasive to employers and workers will require further investigation.

CONCLUSIONS

This risk assessment was undertaken to estimate the economic benefits from reducing occupational exposures to power-frequency magnetic fields as guidance on precautionary recommendations. A 1 μT reduction in a worker's TWA magnetic field exposure is expected to increase the worker's disability-free life by an average of 0.04 years (2 weeks), which is a $5,100 benefit to the U.S. in year 2010 dollars. These benefits from a MF reduction might

Author Manuscript Author Manuscript Author Manuscript Author Manuscript

increase by as much as 80% when exposures to mercury, arsenic, lead, pesticides, herbicides, or solvents also occur. Further, these reductions should be focused on TWA exposures exceeding a precautionary limit in a range of 0.2–0.5 μT, depending on the cancer risks and/or the costs of an intervention which are considered minimal.

These calculations are “evidence-based” in the sense that they are derived from publicly available data by widely used risk assessment methods. Where our precautionary risk assessment required innovations, our assumptions are stated clearly for users to evaluate, and the resulting mathematical formulas are derived in the Supplemental Online Material. Nonetheless, our findings would be more reliable if improved data on the dose–response parameters and the posterior probability could be produced.

These monetary benefit estimates can guide policy makers, employers and workers in developing precautionary approaches to managing workplace MF exposures in several ways. Cost-benefit analyses of possible interventions can be conducted by obtaining cost estimates for the exposure reduction alternatives. Alternatively, calculations of the cancer burdens can be used to group MF sources into control bands. In addition, a precautionary level from Figure 5 can be adopted to identify MF exposures that are too low to justify interventions. Although further work will be needed to successfully implement such strategies for managing possible workplace hazards, this quantitative risk assessment provides an objective basis for precautionary approaches.

Supplementary Material

Refer to Web version on PubMed Central for supplementary material.

References

Ackerman, F. Poisoned for pennies: The economics of toxics and precaution. Washington, DC: Island Press; 2008.

American Conference of Governmental Industrial Hygienists (ACGIH). Documentation of the threshold limit values and biological exposure indices. 7. Cincinnati, OH: American Conference of Governmental Industrial Hygienists; 2006. Threshold limit values for sub-radiofrequency (1 Hz to 30 kHz) magnetic fields.

Arias, E. Report # 54. Hyattsville, MD: National Center for Health Statistics; 2006. United States Life Table, 2003; p. 1-40.

Baan R, Grosse Y, Lauby-Secretan B, El Ghissassi F, Bouvard V, Benbrahim-Tallaa L, Guha N, Islami F, Galichet L, Straif K. on behalf of the WHO International Agency for Research on Cancer Monograph Working Group. Carcinogenicity of radiofrequency electromagnetic fields. Lancet Oncol. 2011; 12:624–626. [PubMed: 21845765]

Biological Effects of Ionizing Radiation (BEIR) Committee IV. Committee on the Biological Effects of Ionizing Radiation. Health risks of radon and other internally deposited alpha emitters. Washington, DC: National Academy Press; 1988. Measures of lifetime risks; p. 131-135.BEIR IV

Blackman, CF.; Blank, M.; Kundi, M.; Sage, C.; Carpenter, DO.; Davanipour, Z.; Gee, D.; Hardell, L.; Johansson, O.; Lai, H.; Mild, KH.; Sobel, E.; Xu, ZP.; Chen, GG.; Sage, SA. BioInitiative Report: A Rationale for a Biologically-based Public Exposure Standard for Electromagnetic Fields (ELF and RF). 2007. Web address: http://www.bioinitiative.org/report/docs/report.pdf

Bolte, JFB.; Pruppers, MJM. Report # 610015001. The Hague, The Netherlands: National Institute for Public Health and the Environment (RIVM); 2006. Electromagnetic fields in the working environment.

Author Manuscript Author Manuscript Author Manuscript Author Manuscript

Author Manuscript Author Manuscript Author Manuscript Author Manuscript

Brown ML, Lipscomb J, Snyder C. The burden of illness of cancer: Economic cost and quality of life. Annu Rev Public Health. 2001; 2001:91–113. [PubMed: 11274513]

Bureau of Labor Statistics. Consumer price index, Table CPI-U. U.S. Department of Labor; 2011. Web address: ftp://ftp.bls.gov/pub/special.requests/cpi/cpiai.txt

California EMF Program. Policy options in the face of possible risk from power frequency electric and magnetic fields (EMF). California: Department of Health Services; 2002a. Web address: http://www.ehib.org/emf/RiskEvaluation/PolicyOptionsF.pdf

California EMF Program. Summary of public comments received and EMF staff responses. 2002b. Web address: http://www.ehib.org/emf/RiskEvaluation/integratedresponse.pdf

California Public Utility Commission. PUC Actions Concerning EMF. State of California; 2006. Web address: http://www.cpuc.ca.gov/PUC/energy/Environment/ElectroMagnetic+Fields/action.htm

Cutler, DM.; Richardson, E. Measuring the health of the U.S. population. In: Cutler, DM.; Richardson, E.; Keeler, TE.; Staiger, D., editors. Brookings Papers on Economic Activity: Microeconomics, BEIR IV edition. Washington, DC: Brookings Institution Press; 1997. p. 217-271.

DeKay ML, Small MJ, Fishbeck PS, Farrow RS, Cullen A, Kadan JB, Lave LL, Morgan MG, Takemura K. Risk-based decision analysis in support of precautionary policies. J Risk Res. 2002; 5:391–417.

Driscoll T, Nelson DI, Steenland K, Leigh J, Concha-Barrientos M, Fingerhut M, Prüss-Ustün A. The global burden of disease due to occupational carcinogens. Am J Ind Med. 2005; 48:419–431. [PubMed: 16299703]

Feychting M, Forssen U, Floderus B. Occupational and residential magnetic field exposure and leukemia and central nervous system tumors. Epidemiology. 1997; 8:384–389. [PubMed: 9209851]

Floderus B, Persson T, Stenlund C, Wennberg A, Ost A, Knave B. Occupational exposure to electromagnetic fields in relation to leukemia and brain tumors: A case-control study in Sweden. Cancer Causes Control. 1993; 4:465–476. [PubMed: 8218879]

Food and Drug Administration (FDA). Food labeling: Trans fatty acids in nutrition labeling, nutrition content claims, and health claims: Proposed rule. Silver Spring, MD: Federal Register; 1999.

Forbes, MP.; Abelson, P.; Driscoll, T. Valuing the mortality and morbidity of work related injury and illness: A life-cycle costing of incidents reported in NSW in 2000–2001. Kensington, Australia: NSW Injury Risk Management Research Centre, The University of New South Wales; 2006.

Gigerenzer, G.; Todd, PM. ABC Research Group. Simple heuristics that make us smart. Oxford: Oxford University Press; 1999.

Goldman DP, Berry SH, McCabe MS, Kilgore ML, Potosky AL, Schoenbaum ML, Schonlau M, Weeks JC, Kaplan R, Escarce JJ. Incremental treatment costs in National Cancer Institute-sponsored clinical trials. JAMA. 2003; 289:2977.

Graham, JD. Speech to the Brookings-AEI Conference on "Risk, Science, and Public Policy: Setting Social and Environmental Priorities". U.S. Office of Management and Budget; 2004. Risk and precaution. Web address: http://georgewbush-whitehouse.archives.gov/omb/inforeg/speeches/101204_risk.html

Hakansson N, Floderus B, Gustavsson P, Johansen C, Olsen JH. Cancer incidence and magnetic field exposure in industries using resistance welding in sweden. Occup Environ Med. 2002; 59:481–486. [PubMed: 12107298]

International Agency for Research on Cancer (IARC). IARC monographs on the evaluation of carcinogenic risks to humans. Lyon, France: IARC Press; 2002. Non-ionizing radiation, Part I: Static and extremely low frequency electric and magnetic fields.

International Commission for Non-ionzing Radiation Protection (ICNIRP). Guidelines for limiting exposure to time-varying electric and magnetic fields (1 Hz to 100 kHz). Health Phys. 2010; 99:818–836. [PubMed: 21068601]

Institute of Electrical and Electronic Engineers (IEEE). Report # C95.6. New York, NY: Institute of Electrical and Electronic Engineers; 2002. IEEE standard for safety levels with respect to human exposure to electromagnetic fields, 0–3 kHz.

Author Manuscript Author Manuscript Author Manuscript Author Manuscript

Johansen C, Raaschou-Nielsen O, Olsen JH, Schuez J. Risk for leukaemia and brain and breast cancer among Danish utility workers—A second follow-up. Occup Environ Med. 2007; 62:782–784. [PubMed: 17472977]

Kassouf AL, Dorman P, Almedia AN. Costs and benefits of eliminating child labour in Brazil. Econ Apl. 2005; 9:343–368.

Kheifets L, Bowman JD, Checkoway H, Feychting M, Harrington JM, Kavet R, Marsh G, Mezei G, Renew DC, van Winjgaarden E. Future needs of occupational epidemiology of extremely low frequency (ELF) electric and magnetic fields (EMF): Review and recommendations. Occup Environ Med. 2008a; 66:72–80. [PubMed: 18805878]

Kheifets L, Monroe J, Vergara X, Mezei G, Afifi AA. Occupational electromagnetic fields and leukemia and brain cancer: An update to two meta-analyses. J Occup Environ Med. 2008b; 50:677–688. [PubMed: 18545095]

Kheifets LI, Gilbert ES, Sussman SS, Guenel P, Sahl JD, Savitz DA, Theriault G. Comparative analyses of the studies of magnetic fields and cancer in electric utility workers: Studies from France, Canada, and the United States. Occup Environ Med. 1999; 56:567–574. [PubMed: 10492657]

Kriteriegruppen for Fysickliska Riskfactorer. Magnetfalt och cancer—ett kriterieddokument [Abstract in English]. Arbete Och Halsa. 1995; 1995:1–10.

Lee GM, Neutra RR, Hristova L, Yost M, Hiatt RA. A nested case-control study of residential and personal magnetic field measures and miscarriages. Epidemiology. 2002; 13:21–31. [PubMed: 11805582]

Lipscomb, J.; Weinstein, M.; Torrance, GW.; Gold, MR.; Siegal, JE.; Russell, LB. Cost effectiveness in health and medicine. New York: Oxford University Press; 1996. Time preference; p. 214-233.

Malkin, R.; Moss, CE. Report # HETA 93-0424-2486. Cincinnati, OH: National Institute for Occupational Safety and Health; 1995. NIOSH Health Hazard Evaluation Report No. 93-0424-2486, Chicago Television Stations, Chicago, Illinois.

Miller AB, To T, Agnew DA, Wall C, Green LM. Leukemia following occupational exposure to 60-Hz electric and magnetic fields among Ontario electric utility workers. Am J Epidemiol. 1996; 144:150–160. [PubMed: 8678046]

Moss CE, Booher D. Assessment of nonionizing radiation exposure at a steel foundry. Appl Occup Environ Hyg. 1994; 9:465–471.

Moss, CE.; Ragab, M. Report # HETA 94-0300-2528. Cincinnati, OH: National Institute of Occupational Safety and Health; 1995. NIOSH Health Hazard Evaluation Report: County Board of Taxation, Cape May Court House, NJ.

Murray, CJL.; Lopez, AD. The global burden of disease: A comprehensive assessment of mortality and disability from diseases, injuries, and risk factors in 1990 and projected to 2020. Cambridge: Harvard University Press; 1996a.

Murray, CJL.; Lopez, AD. The global burden of disease: A comprehensive assessment of mortality and disability from diseases, injuries, and risk factors in 1990 and projected to 2020. Cambridge: Harvard University Press; 1996b. Rethinking DALYs; p. 1-79.

National Institute for Occupational Safety and Health (NIOSH). Qualitative Risk Characterization and Management of Occupational Hazards: Control Banding (CB). 2009. Report # DHHS (NIOSH) Publication No. 2009-152

National Radiation Protection Board (NRPB). ELF electromagnetic fields and the risk of cancer: Report of an advisory group on non-ionising radiation. Chilton, UK: National Radiological Protection Board; 2001.

National Research Council (NRC). Science and decisions: Advancing risk assessment. Washington, DC: National Academies Press; 2009. Uncertainty and variability: The recurring and recalcitrant elements of risk assessment; p. 93-126.

Navas-Acien A, Pollan M, Gustavsson P, Floderus B, Plato N, Dosemeci M. Interactive effect of chemical substances and occupational electromagnetic field exposure on the risk of gliomas and meningiomas in Swedish men. Cancer Epidemiol Biomarkers Prev. 2002; 11:1678–1683. [PubMed: 12496061]

Author Manuscript Author Manuscript Author Manuscript Author Manuscript

Nelson DI, Concha-Barrientos M, Driscoll T, Steenland K, Fingerhut M, Punnett L, Pruss-Ustun A, Leigh J, Corvalan C. The global burden of selected occupational diseases and injury risks: Methodology and summary. Am J Ind Med. 2005; 48:400–418. [PubMed: 16299700]

Neutra, RR.; DelPizzo, V.; Lee, GM. An evaluation of the possible risks from electric and magnetic fields (EMFs) from power lines, internal wiring, electrical occupations and appliances. California: Department of Health Services (now California Department of Public Health); 2002. Web address: http://www.ehib.org/emf/RiskEvaluation/riskeval.html

Olsen JA. On what basis should health be discounted? J Health Econ. 1993; 12:39–53. [PubMed: 10126489]

Park RM, Schulte PA, Bowman JD, Walker JT, Bondy SC, Yost MG, Touchstone JA, Dosemeci M. Potential occupational risks for neurodegenerative diseases. Am J Ind Med. 2005; 48:63–77. [PubMed: 15940722]

Patterson, RM.; Hitchcock, RT. Extremely low frequency (ELF) fields. In: DiNardi, SR., editor. The occupational environment: Its evaluation, control and management. Fairfax, VA: American Industrial Hygiene Association; 2008. p. 538-548.

Phillips SD, Eberhardt KR, Perry B. Guidelines for expressing the uncertainty of measurement results containing uncorrected bias. J Res Natl Inst Stand Technol. 1997; 102:577–585.

Portier, CJ.; Wolfe, MS. Assessment of Health Effects from Exposure to Power-line Frequency Electric and Magnetic Fields: NIEHS Working Group Report. Research Triangle Park, NC: National Institute of Environmental Health Sciences; 1998. Report # NIH Publication No. 98-3981

Reed, L.; Stayner, LT.; Ahlers, HW.; Zumwalde, RD.; Smith, CM.; Christiani, DC.; Kelsey, KT. The role of qualitative and quantitative risk assessment in the development of occupational health standards. In: Smith, C., editor. Chemical risk assessment and occupational health: Current applications, limitations, and future prospects. West-port, CT: Auburn House; 1994. p. 17-24.

Resnik DB. Is the precautional principle unscientific? Stud Hist Phil Biol Biomed Sci. 2003; 34:329–344.

Sahl JD, Kelsh MA, Greenland S. Cohort and nested case-control studies of hematopoietic cancers and brain cancer among electric utility workers. Epidemiology. 1993; 4:104–114. [PubMed: 8452898]

Savitz DA, Loomis DP. Magnetic field exposure in relation to leukemia and brain cancer mortality among electric utility workers. Am J Epidemiol. 1995; 141:123–134. [PubMed: 7817968]

Sorahan T, Nichols L, Vantongeren M, Harrington JM. Occupational exposure to magnetic fields relative to mortality from brain tumours: Updated and revised findings from a study of United Kingdom electricity generation and transmission workers, 1973–97. Occup Environ Med. 2001; 58:626–630. [PubMed: 11555682]

Swedish National Board for Occupational Safety and Health, National Board of Housing, Building and Planning, National Electrical Board, National Board of Health and Welfare, Radiation Protection Institute. Report # ADI 478. Solna, Sweden: Arbetarskyddsstryeisen; 1995. Low-frequency Electrical and Magnetic Fields: The Precautionary Principle for National Authorities. Guidance for Decision Makers; p. 1-11.

Supplemental Online Material. (Section A). Dose-response functions for cancer mortality and incidence. Bowman JD, Ray T, Park RM, Supplemental Online Material for “Possible Health Benefits from Reducing Occupational Magnetic Fields”, Section A. American Journal of Industrial Medicine. Web address: http://onlinelibrary.wiley.com/doi/10.1002/ajim.22129/suppinfo.

Supplemental Online Material. (Section B). Lifetable calculation of excess cancer mortality and incidence. Bowman JD, Ray T, Park RM, Supplemental Online Material for “Possible Health Benefits from Reducing Occupational Magnetic Fields”, Section B. American Journal of Industrial Medicine. Web address: http://onlinelibrary.wi-ley.com/doi/10.1002/ajim.22129/suppinfo.

Supplemental Online Material. (Section C). Calculating Disability-Adjusted Life Years (DALY). Bowman JD, Ray T, Park RM, Supplemental Online Material for “Possible Health Benefits from Reducing Occupational Magnetic Fields”, Section C. American Journal of Industrial Medicine. Web address: http://onlinelibrary.wiley.com/doi/10.1002/ajim.22129/suppinfo.

Supplemental Online Material. (Section D). Quantification of uncertainties. Bowman JD, Ray T, Park RM, Supplemental Online Material for “Possible Health Benefits from Reducing Occupational

Author Manuscript Author Manuscript Author Manuscript Author Manuscript

Magnetic Fields”, Section D. American Journal of Industrial Medicine. Web address: http://onlinelibrary.wiley.com/doi/10.1002/ajim.22129/suppinfo.

Supplemental Online Material. (Section E). Propagation of errors for the economic benefits. Bowman JD, Ray T, Park RM, Supplemental Online Material for “Possible Health Benefits from Reducing Occupational Magnetic Fields”, Section E. American Journal of Industrial Medicine. Web address: http://onlinelibrary.wiley.com/doi/10.1002/ajim.22129/suppinfo.

Supplemental Online Material. (Section F). The precautionary level and its uncertainty analysis. Bowman JD, Ray T, Park RM, Supplemental Online Material for “Possible Health Benefits from Reducing Occupational Magnetic Fields”, Section F. American Journal of Industrial Medicine. Web address: http://onlinelibrary.wiley.com/doi/10.1002/ajim.22129/suppinfo.

Surveillance, Epidemiology and End Results (SEER). Surveillance EaERP, SEER*Stat Database. National Cancer Institute; 2006a. Incidence—SEER 17 Registries (2000–2003). Web address: http://www.seer.cancer.gov

Surveillance, Epidemiology and End Results (SEER). Surveillance EaERP, SEER*Stat Database. National Cancer Institute; 2006b. Mortality—Total U.S. (1969–2003). Web address: http://www.seer.cancer.gov

Theriault G, Goldberg M, Miller AB, Armstrong B, Guenel P, Deadman J, Imbermon E, To T, Chevalier A, Cyr D, Wall C. Cancer risks associated with occupational exposure to magnetic fields among electric utility workers in Ontario and Quebec, Canada, and France: 1970–1989. Am J Epidemiol. 1994; 139:550–572. [PubMed: 8172168]

Thomas D, Stram D, Dwyer J. Exposure measurement error: Influence on exposure-disease. Relationships and methods of correction. Ann Rev Public Health. 1993; 14:69–93. [PubMed: 8323607]

Tickner JA, Kriebel D, Wright S. A compass for health: Rethinking precaution and its role in science and public health. Int J Epidemiol. 2003; 32:489–492. [PubMed: 12913012]

Tolley, G.; Kenkel, D.; Fabian, R. Valuing health for policy: An economic approach. Chicago: University of Chicago Press; 1994. State-of-the-art values.

Victoria Public Health Group. The Victorian Burden of Disease study: Morbidity. Australia: Victorian State Government; 1999a. Web address: http://www.health.vic.gov.au/healthstatus/downloads/morbidity.pdf

Victoria Public Health Group. The Victorian Burden of Disease study: Mortality. Australia: Victorian State Government; 1999b. Web address: http://www.health.vic.gov.au/healthstatus/downloads/mortality.pdf

von Winterfeldt D, Eppel T, Adams J, Neutra R, DelPizzo V. Managing potential health risks from electric powerlines: A decision analysis caught in controversy. Risk Anal. 2004; 24:1487–1502. [PubMed: 15660606]

Weinstein MC. Principles of cost-effective resource allocation in health care organizations. Int J Technol Assess Health Care. 1990; 6:93–103. [PubMed: 2113893]

World Health Organization (WHO). Extremely low frequency fields: Environmental health criteria 238. Geneva, Switzerland: World Health Organization; 2007.

Zaffanella, LE. Survey of Personal Magnetic Field Exposure, Phase II: 1000-Person Survey. EMF RAPID (Research and Public Information Dissemination). 1998. Web address: http://www.emf-data.org/rapid6-report.html

Zarkin GA, Dean N, Mauskopf JS, Williams R. Potential health benefits of nutrition label changes. Am J Public Health. 1993; 83:717–724. [PubMed: 8484455]

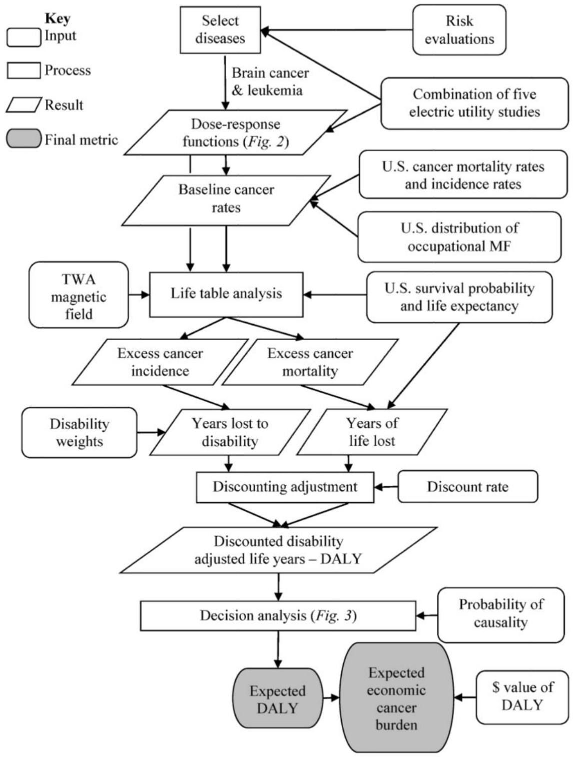

FIGURE 1.

Flow chart for the risk assessment.

Author Manuscript Author Manuscript Author Manuscript Author Manuscript

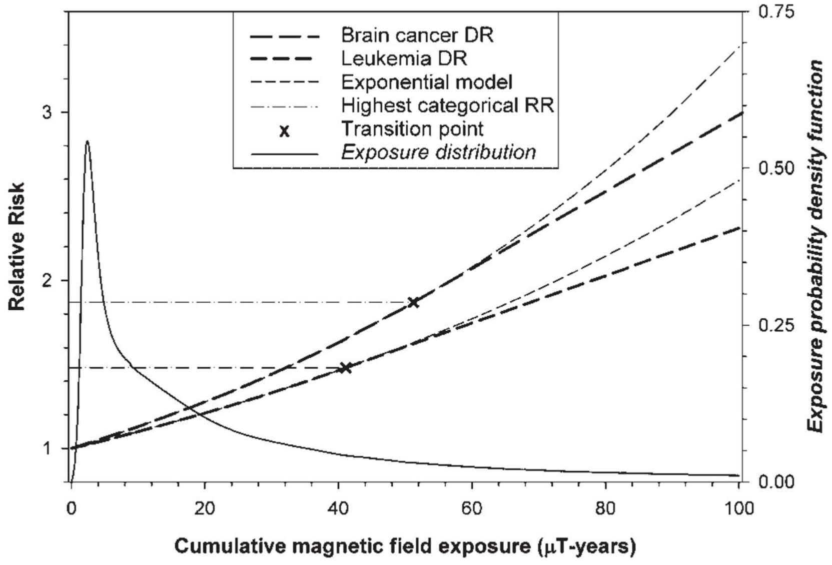

FIGURE 2.

The exponential (Eq. 1) and linear dose–response (DR) functions for brain cancer and leukemia compared with the cumulative magnetic field exposures (right axis) which were calculated from the combined electric utility studies [Kheifets et al., 1999].

Author Manuscript Author Manuscript Author Manuscript Author Manuscript

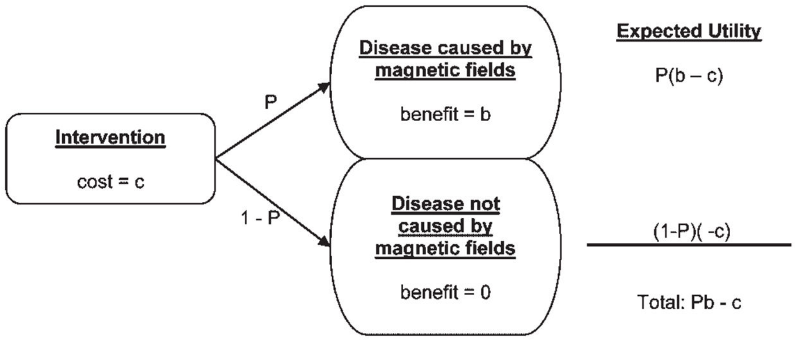

FIGURE 3.
Decision tree based on the costs of an intervention that lowers a magnetic field exposure from B_{before} to B_{after}, taking into account the posterior probability P_c that the reported cancer risks are causal.

Author Manuscript Author Manuscript Author Manuscript Author Manuscript

Author Manuscript Author Manuscript Author Manuscript Author Manuscript

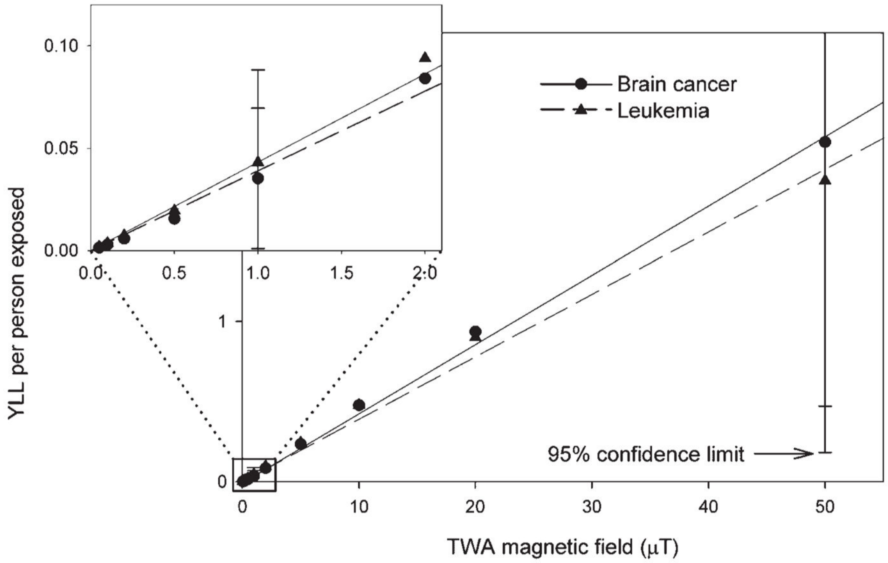

FIGURE 4.

Years of life lost (YLL) from brain cancer and leukemia as a function of the time-weighted average (TWA) magnetic fields, showing linear regression lines through the origin.

Author Manuscript Author Manuscript Author Manuscript Author Manuscript

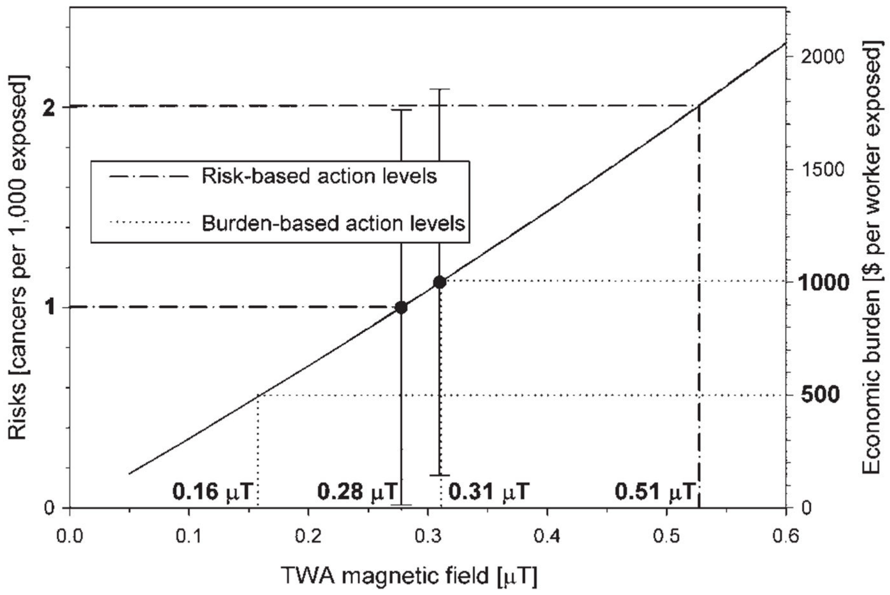

FIGURE 5.

Proposed precautionary limits (PL) for the TWA magnetic field below which the expected value of the cancer risks (excess incidence of brain cancer plus leukemia) or the discounted economic burden may be considered minimal (in bold). Ninety-five percent confidence limits shown for selected de minimis values.

Author Manuscript Author Manuscript Author Manuscript Author Manuscript

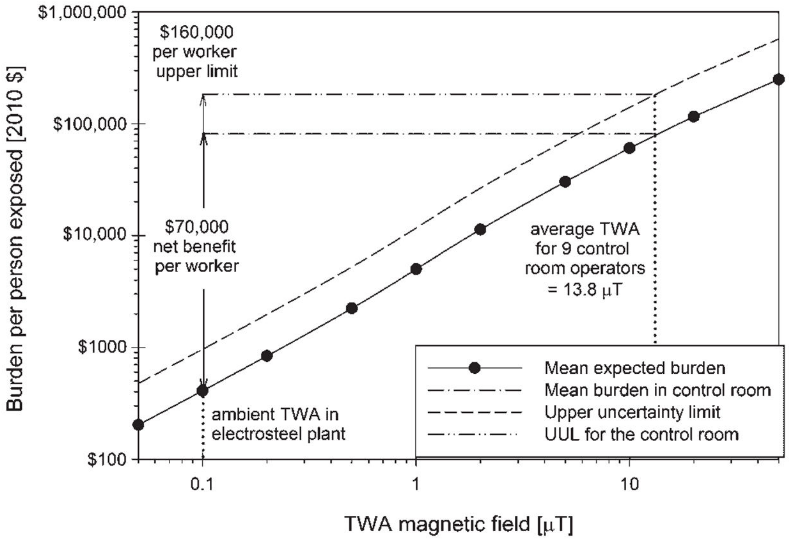

FIGURE 6.

The expected benefits and upper uncertainty limit from a proposed intervention [Moss and Booher, 1994] to move the control room for an electrosteel furnace away from the transformer's magnetic fields. (The lower uncertainty limit is zero.)

Author Manuscript Author Manuscript Author Manuscript Author Manuscript

TABLE I

Health Hazard Evaluations (HHE) of Magnetic Field (MF) Exposures Between the Threshold Limit Value[a] and Cancer Associations[b]

		MF exposures (μT)		
Site	MF source	Maximum	TWA	HHE report's recommendation
Steel plant	Transformer for induction furnace	79.4–148.2	10.8–16.9	"… the existing level of ELF[c] exposure to control room personnel is unnecessary and could be improved by relocating either the transformer or control room. While it is true that present levels are below existing occupational standards, it is also true that very little is known about biological effects of ELF. While some exposure may have to be encountered from control room activities, it does not have to be due to unnecessary exposure." [Moss and Booher, 1994]
Office	Power switchboard	6.1–39.8	0.3–3.4	"… the magnetic field levels in the back area of the Tax Office are at the higher end of the exposure level range documented in previous NIOSH evaluations, and all levels were below the current occupational exposure ceiling limit … As with many occupational exposures, however, employees or employers may wish to reduce them even if they do not exceed current limits." [Moss and Ragab, 1995]
TV stations	Video tape eraser (degausser)	133.7–330.6	2.2–3.9	"The degaussing machine should be relocated to an area where fewer people would be exposed to its emissions. If possible, a method to mechanically load tape into the machine without having an operator stand next to it should be developed." [Malkin and Moss, 1995]

[a]Threshold limit value = 1,000 μT maximum for 60 Hz magnetic fields.

[b]Associations with leukemia and brain cancer for time-weighted average (TWA) MF in categories whose lower bounds are 0.2–1 μT.

[c]ELF = extremely low frequencies = 3–3,000 Hz; NIOSH, National Institute for Occupational Safety and Health.

Author Manuscript
Author Manuscript
Author Manuscript
Author Manuscript

TABLE II

Relative Risks (RR) in the Highest Exposure Category and Dose–Response Slopes From the Comparative Study of Electric Utility Cohorts [Kheifets et al., 1999], Plus the Point of Transition From the Exponential Models to Their Linear Extensions (Fig. 2)

Cancer and magnetic field exposure	Relative risk (95% CI and one-tailed *P*-value)	Logistic-linear transition point[a]
Leukemia		
Highest category ≥16 μT-yr	1.48 (0.96–2.30, $P = 0.04$[*])	41.1 μT-yr
Dose–response in RR/10 μT-yr	1.10 (0.98–1.23, $P = 0.05$)	
Brain cancer		
Highest category ≥16 μT-yr	1.87 (1.17–2.98, $P = 0.004$[*])	51.2 μT-yr
Dose–response in RR/10 μT-yr	1.13 (0.99–1.29, $P = 0.03$)	

[*] Calculated from $P = 1 - N(\ln RR/SE)$ where the standard error (SE) in ln RR is estimated in Supplemental Online Material [See Section A in the Supplemental Information].

[a] Cumulative exposure at risk estimated for highest exposure category.

Author Manuscript

Author Manuscript

Author Manuscript

Author Manuscript

TABLE III

Sources of Random Error, Bias, and Other Uncertainties in the Risk Assessment

Source of uncertainty	Uncertainty estimate	Comments
Chance and inter-study variability in DR[a] slope β	%RSE = 52% for brain cancer and 60% for leukemia	Calculated from Table II
Chance and inter-study variability in RR for highest exposure category	%RSE = 38% for brain cancer and 57% for leukemia	Same as above
Bias in risks from working cumulative exposures calculated from a single company	% δ in DR slope and highest categorical risk = +69%	Bias estimated from proportion of U.S. average employment duration to the durations reported by the electric utility studies
Linear extrapolation of DR	Unquantifiable with exposures outside the epidemiologic data	NIOSH scenario creates cumulative exposures greater than found in single companies
Selection bias in DR	Unquantified and unlikely	Should be minimal in nested case–control studies
Information bias in DR	Unquantified bias towards null (assuming information errors are non-differential)	Diagnosis errors in mortality studies. A few subjects lost to follow-up in U.S. studies
Confounding in DR	Unlikely	None found among dozens of agents examined
Synergism of brain cancer risks with chemical exposures	% δ for brain cancer = +76% for lead, +83% for mercury, +77% for arsenic, +64% for pesticides and herbicides; +62% with solvents	Navas-Acien et al. [2002] reported significant increase in RRs for these chemicals when combined with MF exposures
Classical exposure assessment error in DR from MF metric	Unquantified bias towards null	All mechanisms proposed for MF biological effects involve unmeasured MF characteristics beyond the TWA magnitude
Berkson exposure assessment error in DR from job-exposure-matrices	Unquantified uncertainty in either direction	Berkson error certainly exists in occupational MF studies because occupation only accounts for 5% of measurement variability
Disability weights (DW)	Unquantified uncertainty in either direction	DW derived by “PersonTrade Off” method with small groups of health experts who determine weights for a set of indicator health conditions
Discount rate = 3% per year	Discount rates of 0 and 7% have been used in health economics and other social policy analyses	3% discount rate for the DALYs is consistent with CDC policy
Value of DALY = $100,000 per life year	Studies suggest values from $25 K (−75%) to $428 K (+330%)	Although based on data, the value of a life year is a policy decision
Posterior probability = 0.6	California EMF Project[4] estimates from 0.2 (−67%) to1.0 (+67%)	Some critics of this study would arrive at far lower values for *P*

See [See Section D in the Supplemental Information] for more details and references.

[a]DR, dose response; %RSE, percent relative standard error = 100% standard error/mean; %δ = percent bias = 100% (true-mean)/mean; CDC, Centers for Disease Control and Prevention; DALY, disability-adjusted life-years.

Author Manuscript Author Manuscript Author Manuscript Author Manuscript

TABLE IV

Metrics for the Cancer and Economic Burdens From Exposure to a Constant TWA Magnetic Field Over a Working Lifetime

	TWA magnetic field (μT)									
Metric	0.05	0.1	0.2	0.5	1	2	5	10	20	50
Results without discounting (average yr or $ per worker exposed)										
Brain cancer										
YLL[a]	0.00140	0.00284	0.00580	0.01558	0.0353	0.0841	0.234	0.478	0.936	2.11
YLD	0.00005	0.00010	0.00019	0.00052	0.0012	0.0028	0.008	0.016	0.032	0.07
Leukemia										
YLL	0.00178	0.00360	0.00734	0.01948	0.0430	0.0939	0.243	0.477	0.900	1.88
YLD	0.00016	0.00033	0.00068	0.00180	0.0040	0.0087	0.022	0.044	0.084	0.18
Expected[b] total DALY	0.002	0.004	0.008	0.02	0.05	0.1	0.3	0.6	1.0	3.0
Expected economic burden[c]	$267	$539	$1,102	$2,939	$6,565	$14,894	$39,908	$79,841	$153,369	$333,457
Discounted results										
Brain cancer										
YLL	0.00103	0.00208	0.00427	0.01150	0.0261	0.0623	0.173	0.351	0.684	1.530
YLD	0.00005	0.00009	0.00019	0.00051	0.0012	0.0027	0.008	0.016	0.031	0.071
Leukemia										
YLL	0.00134	0.00271	0.00553	0.0147	0.0325	0.0709	0.183	0.360	0.678	1.41
YLD	0.00016	0.00032	0.00065	0.0017	0.0038	0.008	0.022	0.042	0.080	0.17
Expected[b] total DALY	0.002	0.003	0.006	0.02	0.04	0.09	0.2	0.5	0.9	2.0
Expected economic burden[b]	$203	$410	$837	$2,236	$5,001	$11,343	$30,293	$60,448	$115,764	$249,394

[a] YLL, years of life lost; YLD, years lived with disability.

[b] Expectation values for the total DALYs and economic burdens calculated according to Fig. 3 and eq. 3.

[c] Economic burden in year 2010 U.S. dollars.

Author Manuscript Author Manuscript Author Manuscript Author Manuscript

TABLE V

Slopes for the Cancer and Economic Burden Metrics, Plus Their 95% Confidence Limits and Uncertainty Limits From Linear Regressions (Constant Term = 0)

	Regression coefficient[a] (yr or $/worker/μT)	
**Metric**	**Without discounting**	**With discounting**
Brain cancer		
YLL	0.0431	0.0312
YLD	0.0015	0.0014
Leukemia		
YLL	0.0390	0.0293
YLD	0.0036	0.0035
Expected total DALY	0.052	0.039
Expected economic burden[b]	$6,900	$5,100
Sensitivity analysis of the expected economic burden[b] with discounting:		
95% confidence limits from random errors		$1,000–9,000
Uncertainty limits with single company bias		$0–7,000
With *P* and DALY's value added		$0–12,000
With mercury exposure added		$0–15,000

[a] $R^2 > 0.99$ for all regressions, implying very linear overall relationship.

[b] Economic burden in year 2010 U.S. dollars after factoring in inflation rate as obtained from Bureau of Labor Statistics [2011].

Author Manuscript

Author Manuscript

Author Manuscript

Author Manuscript

TABLE VI

Expected Values and Upper Uncertainty Levels of the Benefits From the Interventions Proposed by NIOSH’s Health Hazard Evaluations [Moss and Ragab, 1995; Moss and Booher, 1994; Malkin and Moss, 1995]

			Mean TWA MF (μT)		Expected benefits (upper uncertainty limit)[a]	
Site	Intervention	# Workers exposed	Exposed	Elsewhere	Per person	Total
Steel plant	Move control room away from transformer	9	13.77	0.1	$70,000 ($200,000)	$600,000 ($1,000,000)
Office	Replace desks next to power switchboard with filing cabinets	5	1.22	0.13	$6,000 ($10,000)	$30,000 ($60,000)
TV stations	Mechanical loader for putting videotapes into the degausser	4	2.80	0.42[b]	$10,000 ($30,000)	$50,000 ($100,000)

Lower uncertainty limits are always zero benefits.

[a]Benefits in year 2010 U.S. dollars rounded off to one significant figure.

[b]Includes walk-by exposures to the degausser.
