# Supplementary material for: Magnetic Fields and Cancer: Epidemiology, Cellular Biology, and Theranostics
Source: Int J Mol Sci. 2022 Jan 25;23(3):1339. doi: 10.3390/ijms23031339 (PMC8835851; doi:10.3390/ijms23031339)
Supplement: Supplementary file 1 [file ijms-23-01339-s001.zip › Supplementary Data Set S1/MF and Cancer.Data/PDF/0114763731/Ptitsyna-Railway.pdf]

ResearchGate

See discussions, stats, and author profiles for this publication at: https://www.researchgate.net/publication/327386784

Railway-generated magnetic field: Environmental aspects

Chapter · January 2009

CITATIONS
5

READS
201

3 authors, including:

Natalia G. Ptitsyna
St. petersburg Filial of Pushkov Institute of Terrestrial Magnetism, Ionosphere and Radio Wave Propagation of Russian Academy of Sciences. St. Petersburg, Russia

147 PUBLICATIONS **862** CITATIONS

SEE PROFILE

Some of the authors of this publication are also working on these related projects:

Project Reconstruction of space weather in the 19th - mid 20th century from data of geomagnetic and other observations View project

All content following this page was uploaded by Natalia G. Ptitsyna on 04 September 2018.

The user has requested enhancement of the downloaded file.

Chapter

RAILWAY- GENERATED MAGNETIC FIELD: ENVIRONMENTAL ASPECTS

N.G. Ptitsyna (1), G. Villoresi (2) and Y.A. Kopytenko (1)
(1) St. Petersburg filial of Institute of Terrestrial Magnetism, Ionosphere, and Radiowave Propagation, , Russian Academy of Sciences, St. Petersburg, Russia
(2) University "Roma Tre", Physical Department, Rome, Italy

ABSTRACT

Recent epidemiological studies suggest a link between magnetic fields generated by electrified transportation systems and certain adverse health effects even though these magnetic fields are generally lower than international limits. Since many people ride on railways daily, such magnetic field exposures should be examined thoroughly. Here we will present a short review of epidemiological research among electrical transportation workers and research results of our waveform measurements of complex "real-world" magnetic fields generated by DC- and AC-powered railways. The obtained measurement information allowed quantitative characterization of specific magnetic field features that could be biologically important: frequency and intensity "windows", interaction with DC-field, geometry (e.g. polarization) and other aspects. Special attention will be drawn to explore the extent to which railway magnetic fields contribute to the total magnetic field environment, and to compare these fields with natural geomagnetic field background. We have conducted an extensive study of magnetic fields on different electrified rail systems. The studied transport technologies were: (i) Russian DC-powered locomotives; (ii) Swiss AC-powered (16.67 Hz) locomotives; (iii) Russian DC-powered trains formed by a number of self-powered electric motor units and units without motors. Measurements have been performed in the ULF-ELF frequency range (0-50 Hz) by means of a novel sophisticated portable computer-based waveform capture system. This measurement system (sampling rate up to 200 Hz) allows continuous recording and characterization of a field profile over time and frequency along three axes. We demonstrated the practicality of the data acquisition and analysis protocols used in this study to estimate exposure characteristics beyond TWA (time-weighted average) that might be of interest to health-related magnetic field researchers. We developed a set of improved methods, algorithms and software to quantify characteristics of complex-spectra railway magnetic fields, as variability in different frequency ranges, amplitude-frequency dependence, polarization

and intermittency. These exposure parameters have been determined in engineers' workplaces and passenger's coaches of both DC- and AC-powered railway systems. Magnetic fields encountered on DC- and AC-powered transport systems are different from power-line fields which are predominantly sinusoidal with a main frequency at 50 or 60 Hz. Railway magnetic fields present complex patterns resulting from the superposition of variations with different amplitudes, frequencies and geometries; the main energy is concentrated in the lowest frequencies. Possible recognition of the main peculiarities of the railway magnetic field could be useful in the identification of sources of these specific features. It will allow developing design-related preventive measures to diminish the health-hazardous potential of magnetic field.

Magnetic field survey and data analysis have been conducted in the frame of the European INCO-Copernicus project "Improvement of methods of exposure assessment for magnetic fields from electric traction with regard to coronary heart diseases".

1. Biological Effects of Railway- Generated Magnetic Field Environment

1.1. Introduction

During the last few decades the intensity level of electromagnetic environment has dramatically increased: devices generating ele ctromagnetic fields have proliferated in industrial plants, public transportation systems, office buildings, homes, etc. While the electromagnetic spectrum is in the range from 0 to above 10^{20} Hz, the main components of electromagnetic pollution are in the extra-low (ELF: 10-300 Hz) and in ultra-low (ULF: 0-10 Hz) frequency bands. In these frequency bands electric and magnetic fields can be treated independently. Experimental and epidemiological data, as well as theoretical arguments, suggest that the magnetic rather than the electric component of the electromagnetic field can be relevant to the human organism because magnetic field can penetrate freely within tissues. In the past there was considerable controversy as to whether weak electric and magnetic fields, at frequencies below 300 Hz, typical of our environment, could cause significant biological effects. These magnetic field intensity levels are usually lower or of the same order of magnitude of the Earth's static magnetic field (~50 microTesla) and they are considered as “weak“. However, research and clinical experience have shown that biological effects from such fields are not precluded simply because they are not perceived. At present time there is a broad consensus in the international scientific community that exposure to low-frequency, low-intensity environmental electric and magnetic fields can produce biological effects, in spite of the fact that the energy involved is quite small (see e.g. [1-3]).

Correspondingly, there has also been an increasing concern that these biological effects may result in health problems(see e.g. [4-6]). The main focus in studies of potential health hazards of electric and magnetic fields was on man-made fields at 50 and 60 Hz, particularly on power line fields, because of their possible association with increases in malignant diseases

More recently, magnetic fields encountered on electrified public transport have also came into consideration. Magnetic fields on electrified transport systems are generally lower than international limits. However, epidemiological studies showed that magnetic fields from AC- and DC-powered transport may be associated with possible health hazard [7-11]. Since many

people ride on electrified transport systems daily, such magnetic field exposures should be examined as thoroughly as power frequency exposures.

Here we present a short review of epidemiological research among transport workers and results of waveform measurements of complex "real-world" magnetic fields encountered onboard DC- and AC-powered railway systems. More extensive measurements have been done in DC-powered trains since there was lack of such material. The obtained measurement information allowed quantitative characterization of specific magnetic field features that could be biologically plausible: frequency and intensity "windows", interaction with DC-field, geometry (e.g. polarization) and other aspects.

Special attention will be drawn to explore the extent to which magnetic fields from electrified transport contribute to the total magnetic field environment, and to compare these fields with natural geomagnetic field and its variations.

Magnetic field survey and data analysis have been conducted by an international collaboration of the project "Improvement of methods of exposure assessment for magnetic fields from electric traction with regard to coronary heart diseases", supported by the European Commission [10-15].

1.2. Epidemiological Studies on Transport Employees

1.2.1. Malignant Diseases

In several Scandinavian studies it was found that railway employees had increased risk in specific types of cancer, though the total cancer incidence (all tumors included) was lower than in general Scandinavian population. In [16] a 12-fold excess in male breast cancer among Norwegian Municipal Tram workers was reported. In [7] it was found that engine drivers (railway engineers) and have a 3-fold increased risk for chronic lymphocytic leukemia. A study on engine drivers and conductors [9] showed that the incidence of chronic and acute lymphocytic leukemia was more than double than for the average Swedish male

In cohort studies based on workers recruited among the Swiss Federal Railways [8, 17, 18] about a 2-fold increased risk for leukemia mortality was observed in line and shunting yard engine drivers referred to train attendants and station managers, the latter being regarded as controls.

1.2.2. Cardiovascular Diseases

Laboratory studies suggest that electric and magnetic field exposure may affect heart rate and heart rate variability. Epidemiologic evidence indicates that depressed heart rate variability is associated with reduced survival from coronary heart disease as well as increased risk of developing coronary heart disease. In [19] it was examined mortality from cardiovascular disease in relation to occupational magnetic field exposure among a cohort of 138, 903 male electric utility workers. Cardiovascular disease deaths were categorized as arrhythmia related (n = 212), acute myocardial infarction (n = 4,238), atherosclerosis (n = 142), or chronic coronary heart disease (n = 2,210). Exposure was classified by duration of work in jobs with elevated magnetic field exposure and indices of cumulative magnetic field exposure. Adjusting for age, year, race, social class, and active work status, longer duration in jobs with elevated magnetic field exposure was associated with increased risk of death from

arrhythmia-related conditions and acute myocardial infarction. Indices of magnetic field exposure were consistently related to mortality from arrhythmia and acute myocardial infarction, with mortality rate ratios of 1.5–3.3 in the uppermost categories. No gradients in risk were found for atherosclerosis or for chronic coronary heart disease. These data suggest a possible association between occupational magnetic fields and arrhythmia-related heart disease. Elevated risk for cardiovascular diseases among railway employees have been found in [10, 11, 20]. These results will be discussed here in more detail.

In [11, 20] the results of studies of cardiovascular morbidity (CVD) among Russian railway workers, in particular among engine drivers, have been presented. Since magnetic field intensity decays with distance from the source, it was assumed that the associated risk would be greatest among engine drivers who work full-time close to the source of train magnetic field. To study the possible role of magnetic field exposure on the CVD incidence, it was necessary to identify populations with different occupational exposure to magnetic fields and with approximately the same "classic" cardiovascular risk factors, related to nutrition and smoking habits, labor stress, work conditions, etc. Thus the authors performed a comparative analysis of morbidity among subpopulations of engine drivers operating different kind of Russian DC-powered electrical trains EL (electric locomotives) and EMU (self-powered electric motor units). Since engines are distributed differently in EL and EMU, magnetic field exposure was expected to be also different. Morbidity data were collected in railway clinics located in different geographic zones of the former Soviet Union from sick-leave certificates that are related to monetary compensation (sick-pay to employees). The data were for ~12,000 age-specified and train-specified engineers (engine drivers) for three years (1975-1977). In Table 1 we show the data on total and CVD morbidity rates among EL and EMU engine drivers. Each group was formed by ~4,000 drivers each year. It is seen that for each age group the morbidity rate for all diseases is the highest among EMU engineers; the average morbidity is 1.35±0.01 times greater than in EL engineers. These differences are mainly attributable to different morbidity rates in respiratory- and gastric-tract diseases, in skin diseases, in trauma and accidents (International Classification of Diseases 9th, ICD: 460-577, 680-709, 800-999). The authors considered major cardiovascular diseases: coronary heart diseases (CHD) and hypertension incidence rates for different engine-driver subpopulations.

For hypertension, it was not found regular pattern of morbidity rate in the different age groups and there were no significant differences, within the statistical errors, in the average morbidity rates. On the contrary, for CHD, there was always the same regular pattern of morbidity rate: the highest incidence being observed in EL engine drivers and the lowest in EMU engine drivers in every age group. The analysis of CVD data among subpopulations of train-specified engine drivers showed that the CHD risk for EL engineers is 12.4±1.0 per thousand per year and for EMU engineers 6.2±0.7 per thousand per year. The occupational CHD risk between these two subpopulations of engine drivers differs by a factor 2.00±0.27. Moreover, the CHD incidence among EMU drivers is observed only after 40 years of age, while among EL drivers there were cases at ages <30. The authors examined the possible roles of different risk factors for these diseases and found that the two groups of engine drivers were most likely exposed equally to labor-stress risks, as they were to the other classic risk factors. The most remarkable difference in work conditions among the subgroups of engineers was the different exposure to quasi-static magnetic fields, with the greatest level of exposure observed in the EL workplace., as it was shown by our direct measurements.

Consequently, it was reasonably concluded that the elevated CHD risk in EL drivers indeed could be associated with an elevated occupational exposure to magnetic fields.

Table 1. Morbidity (in per thousand per year) among drivers of different types of trains: DC electric locomotive (EL), DC electric motor units (EMU). Collected from sick-leave certificates, former Soviet Union 1975-1977

		Age				
**Disease (9th ICD)**	**Workplace**	**20-29**	**30-39**	**40-49**	**50-59**	**Average**
All Cardiovascular Diseases (CVD) (390-458)	EL<br>EMU	15.8<br>—	29.5<br>48.4	67.0<br>36.3	149.7<br>161.3	58.9±2.2<br>57.6±2.2
Coronary heart diseases (CHD) (410-414)	EL<br>EMU	1.6<br>—	2.7<br>—	13.9<br>5.2	40.8<br>21.5	12.4±1.0<br>6.2±0.7
Hypertension (400-404)	EL<br>EMU	9.5<br>—	12.0<br>—	36.5<br>25.9	86.2<br>129.0	32.0±1.6<br>35.0±1.7
All Diseases (000-999)	EL<br>EMU	987<br>1000	1519<br>2484	1298<br>1554	1513<br>1915	1315±10<br>1774±12

The lack of significant difference between engine drivers for hypertension can indicate that the hypertension morbidity rate does not depend on magnetic field exposure. This is in agreement with results of biological studies in which no changes in blood pressure in humans exposed to magnetic fields have been found [21].

It should be emphasized that the contrast in CHD morbidity among drivers of different types of trains is likely to be unbiased, since the medical data collection in the above studies was performed blindly, with no regards to magnetic field exposure. These medical data were collected 25 years ago. At that time the findings were not understandable, because, generally, the EL drivers were supposed to have healthier work conditions than the EMU drivers. Consequently, it was easy to explain the lower morbidity rate for all diseases observed among EL drivers by the better job conditions. However, this viewpoint was inconsistent with the observation of opposite results in CHD morbidity, i.e. the 2-fold increased risk in EL drivers in comparison with EMU drivers. The explanation of this intriguing fact, suggested that railway magnetic fields can play an important role in initiating/promoting CHD.

In [10, 22] mortality from acute myocardial infarct among Swiss railway employees was examined. Apart from the main diagnosis, coronary heart diseases and hypertension were analyzed. A retrospective cohort database was established consisting of 18,070 men employed at, or retired from the Swiss Federal Railways in four occupations: line engineer, shunting yard engineer, train attendant and station master. The cohort was established from personnel and pension records and comprised all persons in active work or retired from the Swiss Federal Railways in the four job categories some time between 1972 and 1993. The main outcome was acute myocardial infarction (ICD-8: 410). Furthermore, deaths due to coronary heart diseases (410-414) and hypertension (400-404) were analyzed. As the four railway occupations are known to have increased exposure to ELF magnetic field, the mortality for acute myocardial infarction among these railway occupations was compared with both the general working population and specific occupations from machine and construction industry, as mechanics or engineer-technicians. There was a significant but modest increase in myocardial infarction mortality among railway occupations. The risk ratio

(including 95% confidence interval) with respect to the specific controls was: 1.264 (1.016-1.574) and 1.241 (1.047-1.472) referring to all Swiss males.

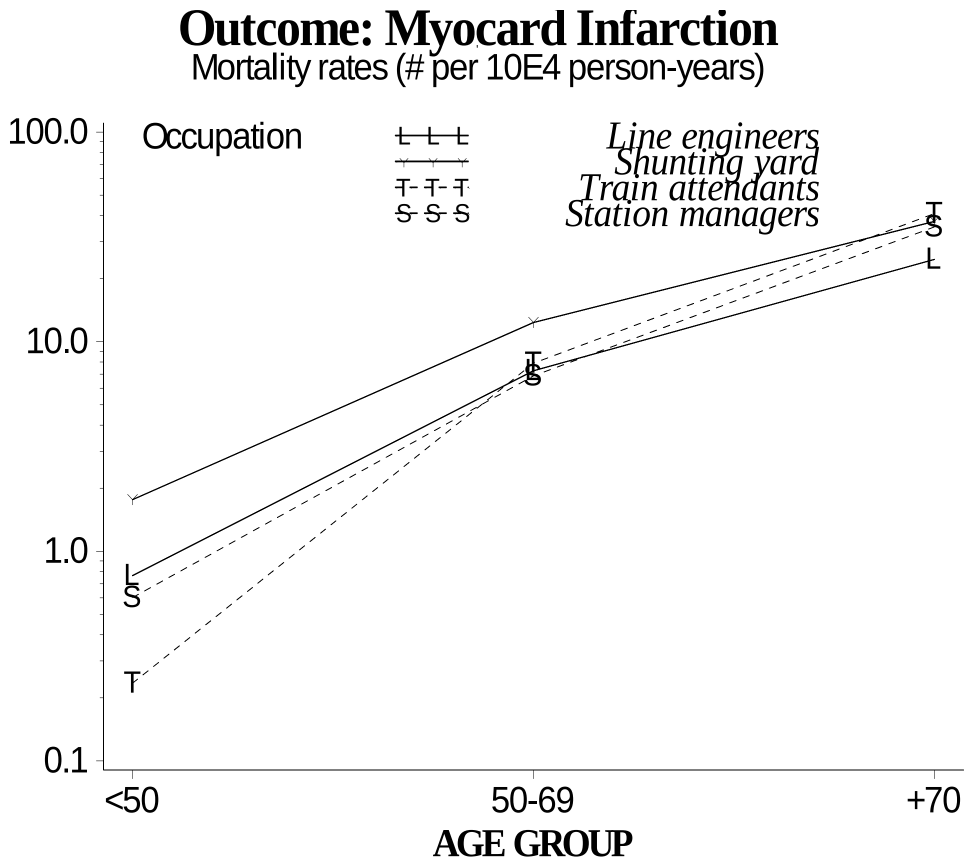

Figure 1. Mortality rates for myocardial infarction among Swiss railway workers.

The risk of coronary heart diseases was less pronounced: 1.125 (0.99-1.277) with regard to the specific controls and 1.202 (1.101-1.312) referred to all Swiss males. There was no increase in hypertension mortality, however. A tendency for an increase in myocardial infarction mortality fractions at younger ages was found. Comparison of mortality from infarctions among four groups of railway occupations was performed. Results showed that at age <50 years the two groups of Swiss engine drivers display higher rates of myocardial infarction mortality than train attendants and station managers. While the rates for line engineers and shunting yard engine drivers were 0.8 deaths/10000 person years and 1.8 respectively, the figures were 0.2 and 0.6 for train attendants and station managers (see Figure 1).

These results give evidence for an increase of myocardial infarctions in railway workers. Interestingly, the increase is not restricted to older ages. An excess confined to older ages would most probably reflect the role of confounding factors such as smoking or life style traits characterized by accumulating effects over life-time. Excess mortality at younger age is compatible with a work related mechanism.

1.3. ULF–ELF Magnetic Field Environment

1.3.1. General characteristics

Different systems of electromagnetic field measurement have produced different definitions of basic quantities, different units, and equations with additional or missing constants.

The magnetic field is characterized by the magnetic field strength, H. Magnetic field strength is a vector quantity (denoted by boldface) composed of three orthogonal vector components, H_X, H_Y, and H_Z. The quantity to characterize the magnetic field in this paper, however, is the magnetic flux density, B. The relation between the two quantities is B=ρH, where ρ is the magnetic permeability. Magnetic permeability is a characteristic of a particular medium. The MKS unit for B is the Tesla (T), however, the CGS (a system of units based on the centimeter, gram, and second) unit of Gauss (G) is used in this paper (1 T=10^4 G). Typical values of B will be in the microTesla (muT = μT) range (1 muT=10 mG). Natural geomagnetic field usually is measured in the nanoTesla (nT) range (1 muT=1000 nT).

Magnetic fields in our environment are produced by electric currents and the strength of these fields depends on the geometry of the circuit, the amount of current in the conductors of the circuit and the distance of the observer from the circuit. In the simple cases of long single wire, two parallel wires and loop of wire, the magnetic field B is proportional to $1/r^n$, where n is equal to 1, 2 and 3 for the three cases respectively. In reality, where more than one current is flowing and the circuit is of complex configuration, there are different orders of decay as a result of cancellation or additional effects ($B \sim \sum 1/r^n$).

The magnetic field environment comprises natural and man-made magnetic fields.

1.3.2. Natural Geomagnetic Background

The natural magnetic fields are the Earth's static field and the geomagnetic variations of interplanetary origin. The Earth's static magnetic field is generated mainly by circulating currents of unknown origin well below the crust. The magnitude of the field varies over the Earth's surface from about 350 mG at the equator to 650 mG near the pole.

Natural phenomena, such as solar and interplanetary activity related to large-scale phenomena, produce time-varying magnetic fields of large planetary extent mainly in the ULF range. Pulsations in the frequency range 0.001-10 Hz are frequently observed in the geomagnetic field records all over the world, with amplitudes between 0.01-1 mG (0.1-100 nT).

The arrival of high-speed solar plasma (generated by solar non-stationary processes) and the associated shockwave near the Earth cause geomagnetic storms. This allows the entry of new particles into the magnetosphere or the acceleration of the ambient plasma to keV energy, thus forming a complicated system of currents in the magnetosphere and ionosphere. During big geomagnetic storms sudden fluctuations due to superposition of equatorial ring current effects and auroral current effects can be identified during 1-2 days: they produce field variations as large as 200-300 nT at low latitudes and can exceed 1000 nT at high latitudes. Geomagnetic storms are associated with a dramatic power increase in all frequency bands (see e.g. [23]).

Several geomagnetic indexes have been devised to describe the intensity level of geomagnetic disturbances on 3-hourly basis. The most widely-used index is the planetary index K_p which is derived from the local K index of a number of selected observatories for 3-hour intervals. K index describes in logarithmic scale the magnetic field variations in such a way that the smallest and largest variations occurring at that station are represented by K=0 and K=9 respectively. The a_p index is a linear index derived from the 3-hourly planetary index K_p and it ranges from 0 to 400. A similar index, often used in solar-terrestrial physics, is the *aa* index in which the daily and yearly variations are removed.

1.3.3. Technological Magnetic Fields at Power Frequencies

Power frequency electric and magnetic fields are produced by the generation, transmission and use of electrical energy, at a frequency in USA and Canada of 60 Hz and 50 Hz in Europe. People are exposed to power frequency fields almost everywhere (e.g. near power lines, electrical wiring and appliances, etc.).

Large man-made magnetic fields can be encountered in close proximity of some home appliances: for instance, 1 muT from refrigerator, 10 muT from coffeemaker, 100 muT from microwave oven [24]. These fields mainly involve current loops of small diameter and fall off rapidly away from the device to almost zero levels at the distance of less than 0.5 m and, in any case, most people do not spend much time close to these bigger fields.

Magnetic fields at 50-60 Hz of larger spatial extent can be found in some "electrical worker" environment and from power lines. Magnetic fields levels associated with transmission and distribution power lines vary greatly depending on the voltage of the line, the amount of current in the line, the way the lines are spaced and distance from the line (since EMF is a byproduct of the use of electric power it is very localized and falls off rapidly with distance). Typically, magnetic field levels will decrease to background levels within a few to 100 m of the line. For instance, magnetic field levels were in the range of 3-5 muT and up to 10 muT in work areas in steel production with electric furnaces [25]. Field intensities under 735 kV power lines were found to be of the order of 5 muT and about 1 muT at a distance of ∼50 m [26].

Generally, EMF around the outside of a substation results from the electric lines entering and exiting the facility. The strength of the EMF from the equipment within the substation, such as transformers, capacitors and circuit breakers, decreases rapidly with increasing distance. Beyond the substation fence, the EMF from the substation equipment is typically slightly higher to indistinguishable from background levels.

However, the contribution of power frequency magnetic fields in general environment is predominantly small, since the phases of alternating current (AC) fields are typically in close proximity to one another, largely canceling each other.

1.3.4. Railway Magnetic Fields in our Environment

Magnetic Fields Far from Sources

The highest fields of large spatial extent in densely populated urban areas and in occupational environment are produced by rail public electrified transport. The magnetic fields for typical railway currents (500 A) schematically are shown in Figure 2 (adopted from

[24]). In this drawing a current of 500 A flows into the paper through the trolley wire and is returned in equal amounts by the rail.

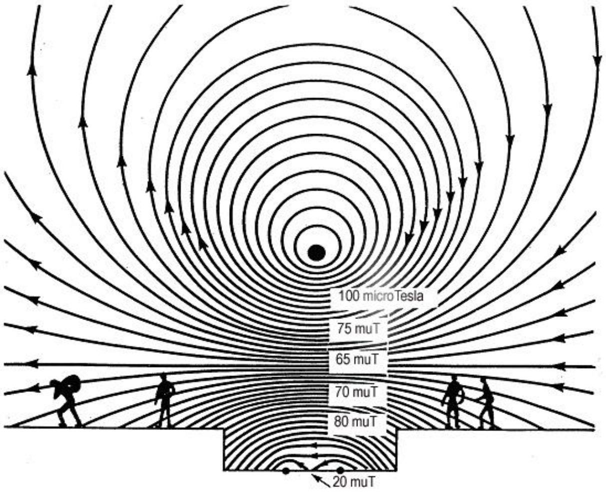

Figure 2. Distribution of magnetic fields for typical railway currents (500 A).

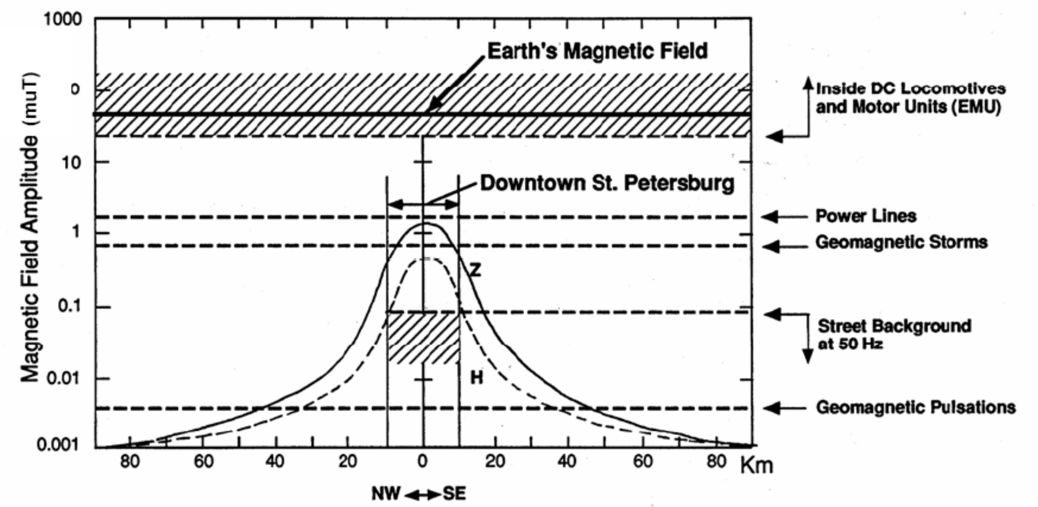

Figure 4. The spatial distribution of magnetic field in ULF range (0.001-10 Hz) in the region of a big industrial city (St. Petersburg). Magnetic field values due to typical natural and man-made sources are given for comparison.

Rails of DC electrified rail transport, being a part of the electric circuit, also produce ground leakage currents of large extent which generate significant magnetic fields: measurements show that ULF magnetic fields were about 1 muT at the distance of 100 m far from train rails [27] and about 0.1-0.2 muT at a distance of 1 km [28]. These currents are concentrated on materials with higher electrical conductivity in comparison with ground, as

metal surfaces of underground pipelines, plumbing lines, communication cables, etc., and may contribute substantially to magnetic field environment.

We performed magnetic field surveys in St. Petersburg and in two magnetic observatories located respectively 30 and 90 km far from the city center [29]. Results of these measurements are shown in Figure 3, which illustrates the distribution of ULF magnetic field inside and near a big industrial city. In the city the level of man-made magnetic fields is bigger than the level far from the city by a factor 10^3.

Figure 3 shows also the mean levels of ULF magnetic fields from different natural and man-made sources. Typical levels of 50 Hz street background and power line fields measured under the power line are shown.

Measurements performed in [27] revealed that the DC-operated San Francisco Bay Area Rapid Transit System (BART) was a powerful generator of ULF magnetic fields detectable throughout the Bay Area (~100 km^2). The primary source was identified to be the large current loops formed by the third rail, the BART trains, the running coils, and the substations providing the DC power. The magnetic field measured at a location 100 m from the tracks was predominantly vertical and has irregular variations, but it is possible to distinguish periodic bursts of heavy activity at 0.002-0.003 Hz which correspond to the schedule of trains. The authors found that BART increased the natural geomagnetic ULF background by 2-3 orders of magnitude.

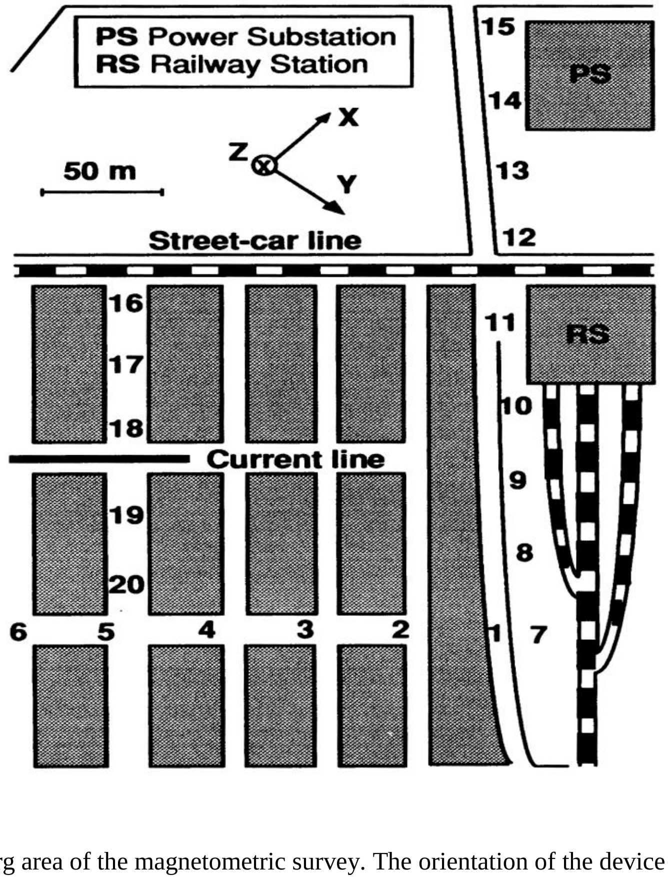

Figure 4. St. Petersburg area of the magnetometric survey. The orientation of the device is indicated Numbers indicate sites of measurements. RS-Railway station, PS – power station.

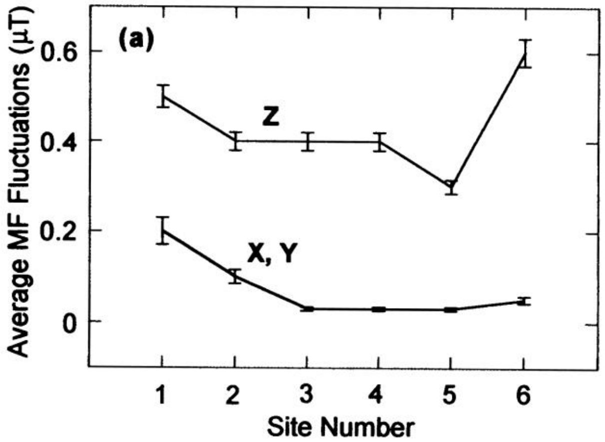

Figure 5. Distribution of average amplitudes of ULF magnetic field fluctuations along a profile perpendicular to railway tracks.

To recognize the characteristic peculiarities, the dynamics and distribution of transport magnetic fields in connection with transport lines in a big industrial city, we recorded ULF magnetic field background (0.001-10 Hz) in St. Petersburg (Russia) in 1998 in a number of sites near railway and street car tracks. In each site we took continuous measurements for 25 min. [30].

Our measurements revealed that magnetic fields in the frequency range 0.001-10 Hz were mainly produced by DC-powered railway.

In Figure 5 we show the average amplitude of magnetic field fluctuations measured along a profile perpendicular to railway tracks (sites № 1-6; the railway is situated in the site № 0). The sites were located at regular intervals of 35 m each. This plot shows that ULF magnetic field fluctuations tend to decrease with increasing the distance from the railway; local magnetic field sources may introduce distortions in this tendency, as for instance in site № 6. Maximum amplitudes were registered in Z-component

In Figure 6 results of magnetic field monitoring of Z-component on Sunday (upper plot) and Monday (lower plot) in one site are presented. From this figure we notice that magnetic field variations are bigger on Monday since railway traffic in working days is more intense. The most prominent signals look like peaks in Z-component in the frequency range 0.05-0.2 Hz with amplitudes up to 1 muT. These peaks appear often in sequences, with duration of 3-30 min**,** and in general they look like natural Pc2-Pc3 pulsations, but with much bigger amplitude. For instance they are bigger, by a factor 10^3-10^4, than Pc2-Pc3 pulsations observed during moderate geomagnetic activity (local *K*-index equal 3).

The second type of prominent fluctuations, observed in Z component, consists in long periods (200-800 s) of increased field amplitude**;** these long-period "waves" are similar to natural Pc5, Pc6, Pi3 geomagnetic pulsations (below 0.005 Hz). These technological long-period magnetic field "waves" have positive polarity and they can be as big as ~0.5 muT.

In general, transport-related magnetic fields in ULF range recorded at distances of tens meters from transport lines look similar to records of natural magnetic fields during geomagnetic disturbances.

Magnitudes of magnetic field fluctuations during big interplanetary-driven geomagnetic storms can be 0.4-0.5 muT at the latitude of St.

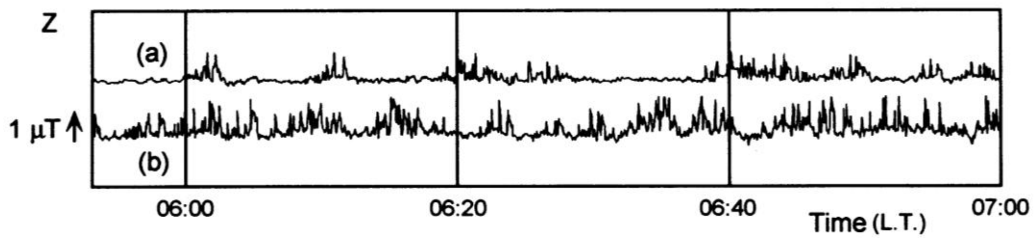

Figure 6. ULF magnetic fields generated by electrified transport in St. Petersburg on Sunday (a) and Monday (b).

Petersburg. Intensity levels of transport-related magnetic fields decrease with distance from sources; they become of the same order of magnitude of natural magnetic field variations at a distance of 30-100 m. However, the actual relation between magnitudes of natural and man-made fields depends on many factors (latitude, geomagnetic activity, distribution and character of man-made sources, underground conductive lines etc.).

Magnetic Fields Onboard and Near Electric Trains

In this paragraph we present a short review of previous measurements of magnetic fields produced by different electrified railway systems by rms (root-mean square) magnetometers. The rms monitors lack the ability to resolve the frequency characteristics of the magnetic field; they record the total magnetic field level within a particular frequency range.

The amplitudes of transport fields depend on the particular railway system, frequency range and point of measurement. Measurements at 16.67 Hz [31], done on a platform directly above the contact wire for electric traction, revealed magnetic field amplitudes in the range of 10-20 muT when a train passed. Magnetic fields measured in the last car of an Amtrak train in USA showed amplitudes of up to 30 muT at 60 Hz and up to 65 muT at 25 Hz [24]. Static magnetic fields, in the range 16-64 muT and up to 15 mT, were observed in the British electric transport system; time-varying magnetic fields were recorded in the range 5-50 muT at 50 Hz and up to 15 mT at 100 Hz near facilities [32]. Measurements performed at 16.67 Hz inside two types of locomotives of the AC Swiss railway system revealed that in the modern types the maximum magnetic fields are less than 200 muT, while in the older types the maximum field strength at the height of the calves is in the range 1640-6170 muT [18]. Measurements in Japanese trains revealed that magnetic fields were as follows: substation 0.3-3 muT; railway station 0.2-10 muT; DC train 0.5-5 muT (static magnetic field, 50-200 muT); AC train 0.2-150 muT (static, 100-4000 muT); AC/DC train 0.5-75 muT (static, 200-1000 muT); and AC/DC locomotive less than 4 muT (static, 50 muT). Maximum values around facilities and power-running devices found in Japanese trains were several times greater than these values [33].

Recently, a waveform monitoring system “Multiwave” was used for recording magnetic fields in AC-powered USA trains (25 Hz and 60 Hz) [34] and in Maglev Vehicle Transrapid TR07 in Germany [35, 36]. It was found in locomotive cabs magnetic field at principal components 25 and 60 Hz in the range of 3-5 muT for average values and maximum values of 8-21 muT; higher odd harmonics at smaller amplitudes were also present [35]. Maglev magnetic field data were significantly more complex than those found in the vicinity of power

lines; they have complex frequency spectra highly variable with time [36]. In Maglev, typically, about 80% of the time-varying magnetic fields were at frequencies below 47.5 Hz. In passenger compartments the average field levels ranged from approximately 10 muT near the floor of the vehicle to approximately 2 muT at standing head level. Except for the strong height dependence magnetic fields did not depend strongly on location within passenger compartment. The static magnetic field level near the floor was ~80 muT and reduced to 50 muT at standing level. Static fields were more spatially variable, but slightly more stable over time than alternating or time-varying fields. Within the waiting area of the passenger station time-varying magnetic field levels produced by the passing train was approximately 2 muT.

2. ULF-ELF Magnetic Field Testing of Railways

No commonly agreed theoretical explanation for the biological action of weak ambient electromagnetic fields, to which humans are routinely exposed in homes or workplaces, has emerged till now. This area of research is characterized by a significant gap between experimental results and biophysical theory. Without an established mechanism of biosystem-field interaction there is no guidance as what attributes of the field might be biologically important. Thus the question of “what to measure?” is open.

In most studies, magnetic field exposures are generally characterized only in terms of root-mean square (rms) strength of the field, a time-weighted average field-strength parameter. Meanwhile biological and epidemiological studies of possible effects of magnetic fields indicate that there is not a well established dose-response relationship when “dose” is the time-weighted average field strength of magnetic field (TWA). Frequency windows, intermittent components, DC/AC relationship, polarization can be important. However, till now there was little or no information on the above characteristics.

In our measurement project we considered gathering all aspects of magnetic field permitting maximum flexibility in metric extraction. Such an approach would allow the extraction of new metrics that may be proposed in future. For measurements in DC and AC (16.67 Hz) trains we focused on monitoring magnetic fields in the frequency range 0-50 Hz. This approach requires instruments with bandwidth of 0-50 Hz and able to collect data for long periods of time.

2.1. Instrumentation

2.1.1. Portable Waveform Capture System MVC-3

A novel sophisticated waveform capture system MVC-3 for recording magnetic fields in 0-50 Hz range is the magnetometric multivariation complex MVC-3. MVC-3 is an improved version of a 3-axis microcomputer-based portable waveform capture system, developed by SPBFIZMIRAN [37]. MVC-3 utilizes different principles than Faraday-effect gaussmeters which were commonly used in biologically-related magnetic surveys. MVC-3 belongs to the class of magnetometric devices of torsion type which were traditionally practiced for measurements of natural geomagnetic fields in magnetic observatories. MVC-3 is a new version of torsion magnetometer, based on modern technologies and designed for magnetic field monitoring onboard moving carriers, including electrified transport systems. The

magneto-sensitive element (MSE) of the torsion sensor (Figure 7) is a permanent magnet (1) suspended on quartz or metallic fibers (2) which serves as rotation axis of the MSE.

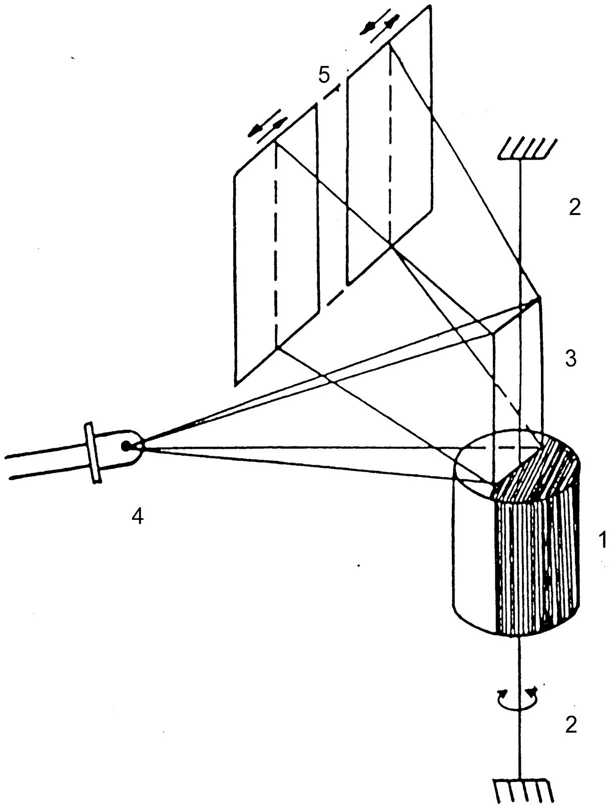

Figure 7. The magneto-sensitive element (MSE) of the torsion sensor utilized in the magnetometric complex MVC-3. For symbols see the text.

The angular displacement of MSE correspondent to magnetic field changes is transformed into an electric signal by means of a reflecting surface (3) and photoelectric converter (4 - photoemitter, 5 - photoreceiver). The output voltage is proportional to the B value and thus MSE reacts to both static and time-varying magnetic fields. The upper frequency limit for measurements was defined by the mechanical ability of the sensor to rotate. Due to mechanical inertia this limit was about 15 Hz, however using negative feedback we increased the upper frequency limit of MVC-3 till 50 Hz (sampling rate 200 Hz). MVC-3 has a flat response for analog output with accuracy of 0.05% in 0-20 Hz frequency range and 0.7% at 50-60 Hz. Dynamic range is ~120 dB. The tolerance to dynamic loads and mechanical vibrations for frequencies over 0.03 Hz was improved by filling the capsule containing MVC-3 sensors with a special damping liquid.

This computer-based system allows to capture a magnetic field profile and to characterize field magnitudes over time and frequency along three orthogonal axes. In so doing all information characterizing intensity, frequency domain and geometry can be captured.

2.1.2. Calibration and Quality Assurance Procedures

The reliability and stability of the device operation and the quality of data have been controlled through several calibrations and comparison procedures before, during, and after measurements:

(i) Placing each sensor in a known magnetic field at the center of calibration Helmholtz coils.
(ii) Comparing magnetic field waveforms obtained by MVC-3 and other waveform devices. This testing was done during an International Workshop on comparison of magnetometers used for the European Project EUROPROBE/SVEARKO (Nurmiyarvi Observatory, Finland, 1997).
(iii) Comparing our device with a commercial Bramur gaussmeter. For this comparison a number of subroutines have been done for calculating MVC-3 rms in the same way as it was done in Bramur.

We found that MVC-3 remained stable during the whole period of measurements. No differences in rms measured by Bramur and calculated from magnetic field measured by MVC-3 was found. Both instruments gave the same rms values at 16.67 Hz. The comparison of actual profiles showed that there is no distortion of waveforms measured by MVC-3.

2.2.Onboard Measurements

2.2.1. Railway Facilities

Measurements have been done in Russian and Swiss rail systems. Russian railways have an overhead DC 3 kV supply. Swiss railway has an overhead AC (16.67 Hz) supply of 15 kV. The current return is *via* the running rails. Measurements were performed onboard of:

(i) Russian electric locomotives (EL). The motors (six or eight) of the most commonly encountered mainline EL are in the range of 450-770 kW, 340-550 A each, depending on work regime.
(ii) Swiss electric locomotives (EL). The most popular locomotive Re 4/4-II has a maximum traction power of 4700 kW.
(iii) Russian suburban electric trains (EMU) formed by a number of self-powered electric motor units and units without motors. Four traction motors (140-178 kW, 100-175 A) are built into every second coach of EMU (the engineer's coaches are not motor units).

2.2.2. Measurement Protocol

The coordinate system of magnetometer was: X - horizontal component, directed along rails to the direction of motion; Y- horizontal component, perpendicular to the train axis; Z - vertical component, directed downward.

The three sensors were arrayed on a staff at 12.5 cm intervals. Most part of measurements were taken in driver’s cabs; in this case the staff was placed in a position near the head of the sitting engine driver. A number of measurements were done in other train coaches and in platforms.

2.2.3.Data Organization

Magnetic field monitoring was performed in Russian DC railways during several tens of working days. In Switzerland the time of measurement was much shorter. Waveforms of the three orthogonal components have been recorded by sampling amplitudes at rates up to 200 Hz and storing the digital values on computer disc. The three individual components have been converted to the total field by vector summation when desired during the data analysis. Thus the obtained records present the "moving picture" of the magnetic field and show the evolution of field characteristics over time, e.g. when the train accelerates, decelerates, makes use of its dynamic braking systems, passes distribution substations and so on.

At the beginning continuous measurements during some hours have been conducted. After the initial data treatment we considered to collect data over 20-min or 10-min intervals to facilitate data storage and further analysis. The interval between two subsequent data files was usually shorter than 1 min, just the time necessary for closing and opening files.

As a result of measurements we formed a database containing about 60 hours of continuous magnetic field recording in electrified trains. The archive contains 129 files of magnetic field in Russian trains (sampling rate from 20 Hz up to 200 Hz) and 46 files in Swiss trains (sampling rate 200 Hz). Files are followed by legend with information on route conditions (changes of current with time, acceleration, idling and braking phases, stops, power substations, oncoming trains, railway switches, bridges, etc.).

2.2.4. Measurements on Russian DC-Powered Railways

a. Source identification. Figure 8 shows "moving pictures" of measured magnetic fields.

The bottom frames of Figure 8 show the time variations of the three components of magnetic field measured in a Russian DC-powered electrified locomotive during 10 min, sampling rate is 100/s. A more detailed view of actual waveforms in a 20-s sample is plotted in the top frames of Figure 8.

The accompanying notes on route conditions allowed the identification of sources of different magnetic field variations [12].

Figure 9 shows the magnetic field (X, Y and Z components) pattern in a passengers' coach with motors. During these measurements the sensor was moved in a location above a motor. It is seen that the direction of magnetic field vector differs remarkably in the two positions and its magnitude can be as big as 150 muT. The magnetic field variations along rails (X component) are almost negligible in comparison to those perpendicular to rails. The braking phases introduced small disturbances in the magnetic field patterns; on the contrary oncoming or stationary trains encountered along the route introduced remarkable variations due to their ferromagnetic mass.

Figures 10 and 11 show magnetic field measurements taken in the engine driver's compartment (coach without motors). It is seen a much lower amplitude of magnetic field variations, usually of the order of 50 muT. Unusual variations are observed in the last part of the trip, entering St. Petersburg in close proximity of industrial zones (see Figure 11). In this case the magnetic field variations can reach up to 100 muT.

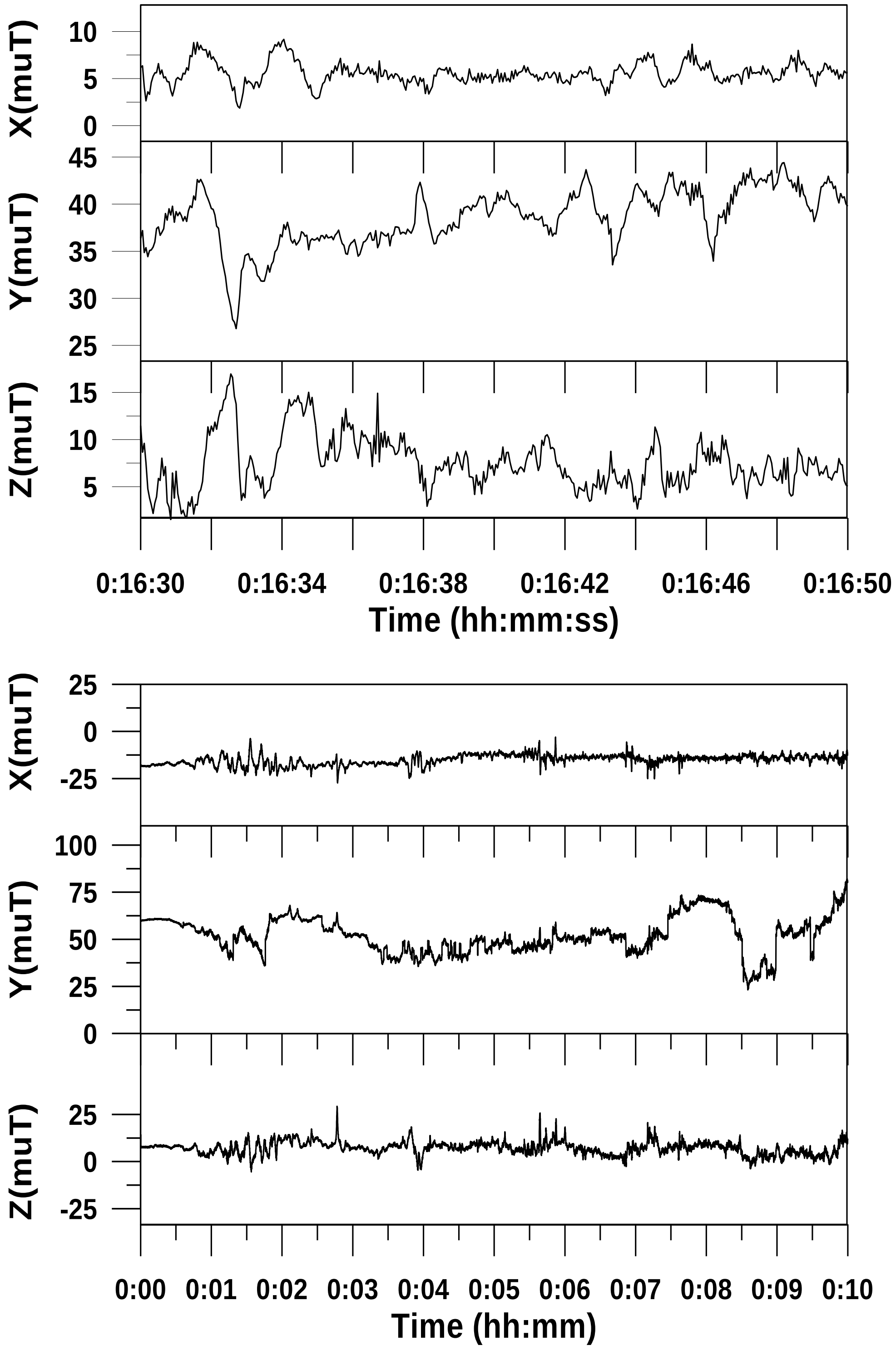

Figure 8. Magnetic field measured onboard Russian DC electric locomotives (EL) in a 10-min interval (lower plot) and in a 20-sec interval (lower plot).

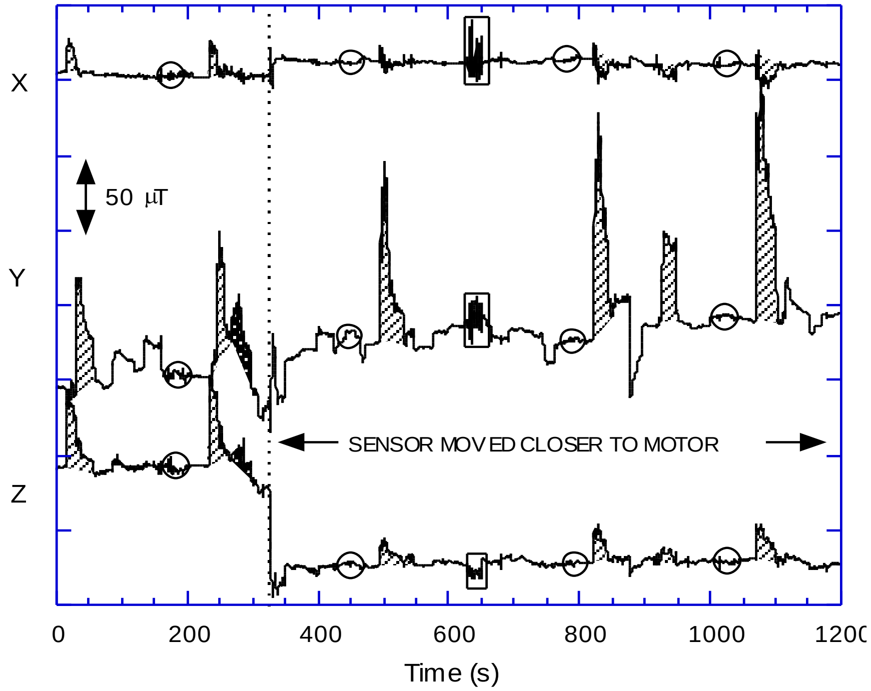

Figure 9. ULF magnetic field (X, Y, Z components) in a coach (with motors) of a DC electric motor unit (EMU) train. Dashed area: acceleration phase; black area: power substation; square: oncoming train; circle and ellipse: braking phase.

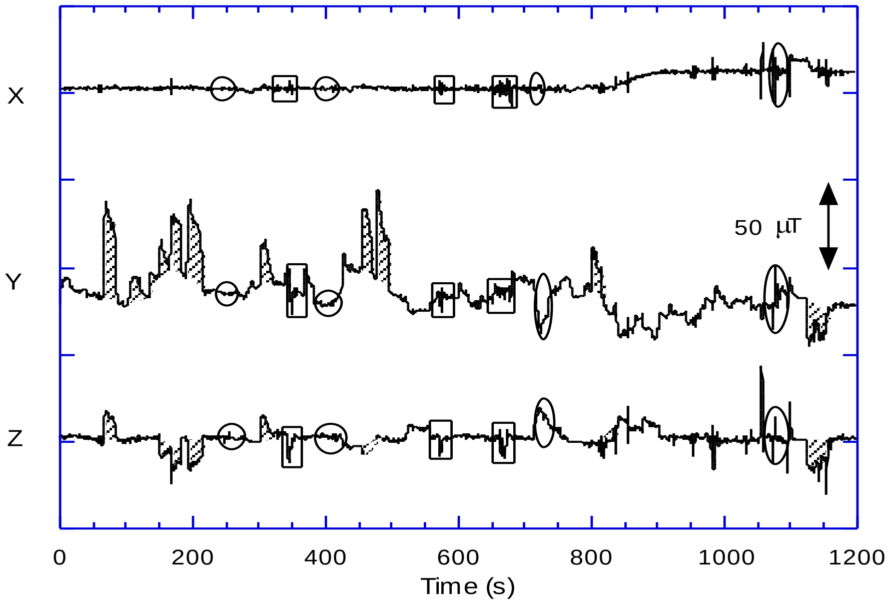

Figure 10. ULF magnetic field (X, Y, Z components) near the driver's workplace (car without motors) of a DC electric motor unit (EMU) train. For symbols' definition, see Figure 9.

The variations observed in the three components showed the same pattern, but with much smaller amplitude in X and Z. Here the zero levels of magnetic sensors were adjusted to the field values on the platform (general magnetic environment) before train departure; in such a way the background level of static field can be estimated. It is seen that the field value is usually much higher than the reference one, but sometimes (for instance when passing the first power substation) it becomes lower. Peak-to-peak variations can be as big as 100 muT.

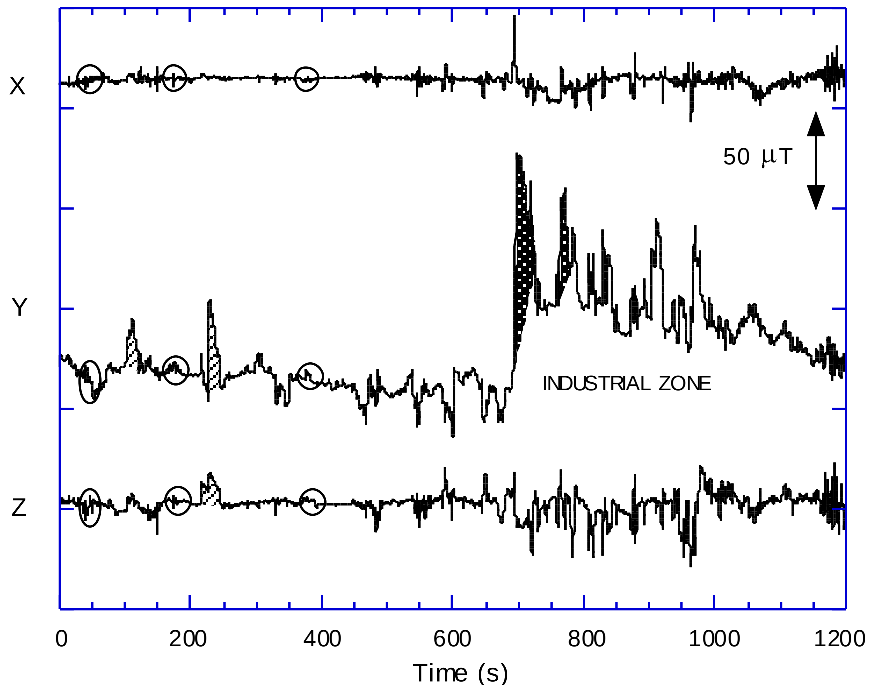

Figure 11. The same as for Figure 10. For symbols' definition, see Figure 9.

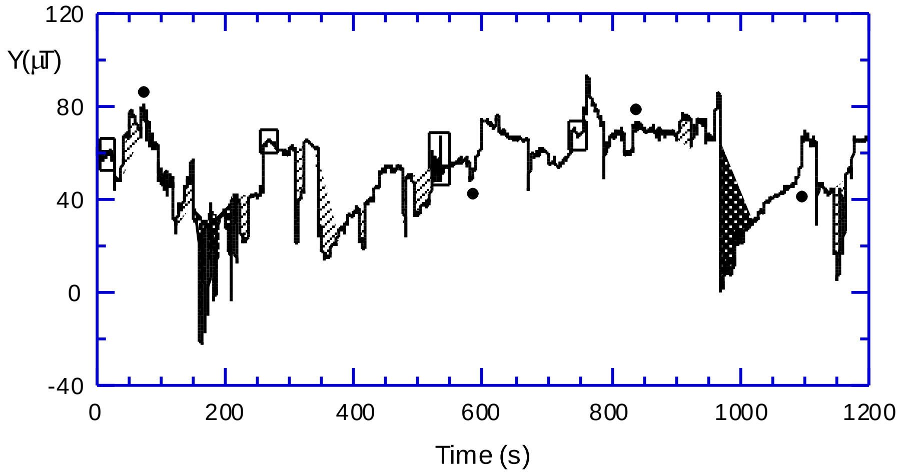

Figure 12. Y component of ULF magnetic field near the driver's workplace of a DC mainline electric locomotive (EL) [12]. *Black circle*: zero current phase. For the other symbols, see Figure 9.

Figure 12 shows the Y magnetic field component measured in a mainline electric locomotive EL, near engine driver's workplace.

Figure 13 shows variations of magnetic fields measured along the same route under different train speed regimes from 45 till 120 km/hour. It is visible from the Figure 13, that frequency and amplitudes of magnetic variations are proportional to train speed: higher frequencies and amplitudes were observed under higher speeds [13].

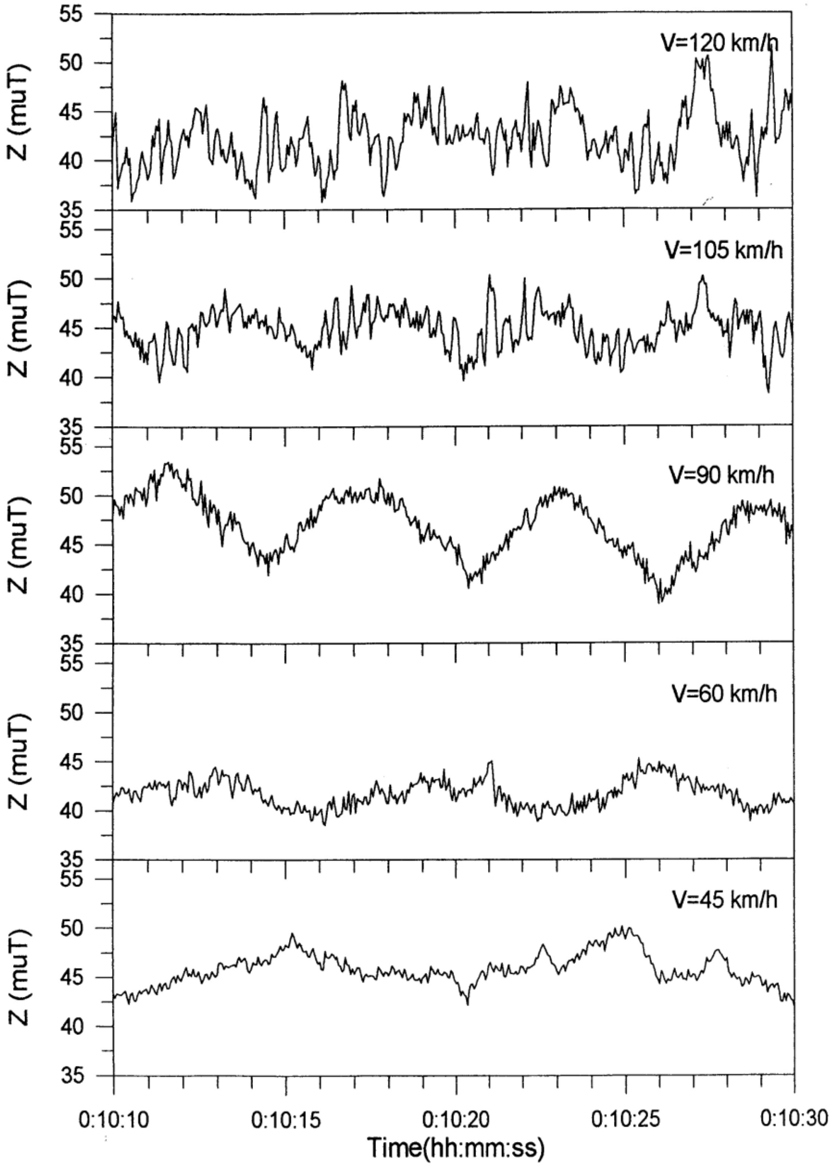

Figure 13. Magnetic field measured onboard Russian DC-powered locomotives under different train's speed.

Analisis of magnetic field variations measured onboard of different DC and AC railway systems allowed to define sources of these variations.

We can conclude that all sources of magnetic fields onboard can be classified as follows:

- static geomagnetic field;
- distortion of static magnetic field due to the iron/steel mass of electric train and ferromagneticobjects within the locomotive;
- varying magnetic field from catenary and rails current needed by the train itself and by all other trains in motion between two closest substations;
- varying magnetic field arising when passing different nearby stationary and moving ferromagnetic objects and along the wayside (oncoming trains, railway switches, bridges, etceteras). In this case geometry of fields (relative position) is important.
- varying magnetic fields from different kind of ground-based man-made current sources;
- varying magnetic field from different current systems onboard electrical train;
- varying geomagnetic field;
- varying magnetic field caused by variable induced current generated by train movement in the static geomagnetic field (change in direction, accelerations, braking, etc).

Our research enlighten the fact that onboard rail systems we measure a superposition of all magnetic fields produced by multiple sources. The superposition of variations with different amplitudes and frequencies determines complex magnetic field patterns. These patterns are highly variable with time due to changes in route conditions.

b. Frequency spectra. As we can see in Figures 8-13, magnetic field encountered on DC-operated rail systems look quite different from power line fields, which are predominantly sinusoidal with main frequency at 50 or 60 Hz. Magnetic field on DC trains exhibit complex frequency patterns, including quasi-static variations and pulses. These complex patterns resulted from superposition of variations with different frequencies, which covered the whole studied frequency range from 0 to 50 Hz. Magnetic field patterns were characterized by extremely complex combination of static and time-varying components.

The frequency of observed magnetic field variations depends on the train's speed: the higher frequencies were measured under higher speed. The amplitude of the variations were also higher under higher speed (see Figure 13).

To visualize and to analyze the highly variable behavior of magnetic field patterns we computed dynamic spectra of magnetic field records.

Method. To analyze magnetic field time variations for any chosen frequency band, we calculated dynamic power spectral density (DPSD) of the magnetic field data. The construction of DPSD is based on the computation of sliding spectra of a time-dependent process. The spectral-temporal representation is obtained by Fourier transforms of successively cut-out parts of the analyzed data. Power spectra were calculated for 20-s window sliding along the data set at 5-s steps.

For normalization the amplitude spectrum values were converted to decibels values according to the formula $A[dB]=20\log(A/A_{max})$, where A_{max} value was defined from all calculated dynamic amplitude spectrum values. The value 0 dB (maximal amplitude) corresponds to black color in the dynamic amplitude spectrum. Minimal (negative) amplitude corresponds to white color. A “grayscale” is shown at a right side of all dynamic spectra. We

remind that -20 dB in comparison with 0 dB means a decrease in amplitude by 10 times, -40 dB - by 100 times, etc. The DPSD were calculated in the frequency range 0.1-50 Hz.

Results. In Figure 14 we show an example of the analysis. The top panels show the 3-component magnetic field variations measured on a locomotive during 10-min interval. The dynamic amplitude spectrum for frequencies 0.1-50 Hz in the same time interval is plotted at the bottom frames of Figure 14. The spectrum shows the presence of magnetic field bursts in wide frequency range (0.1-50 Hz) in all three components, as manifested by dark vertical strips. The same kind of bursts in wide frequency range have been also observed in dynamic spectra of EMU magnetic field. These bursts of increased amplitudes appear 1-2 per min, defining intermittency characteristics in this time scale. Figure 14 evidences that a number of constant frequencies are present in EL field spectrum throughout the whole 10-min interval: 5, 12, 25, 32 Hz in X component, 5, 12, 32 Hz in Y component and 5, 12, 32 Hz in Z component. They are clearly seen in the dynamic amplitude spectra as distinct narrow horizontal strips in this frequency bands. This is probably indicative of the existence of onboard sources producing these magnetic field variations.

*c. Levels of fields***.** The greatest quasi-static variations (DC fields) in DC-powered Russian rail systems were observed in Y component perpendicular to rails, where peak-to-peak values reached 120 muT in EL during acceleration phases or when passing substations.

In Table 2 we show general statistical characteristics (mean values *m* and computed root mean square σ) for DC levels calculated for all measurement files:

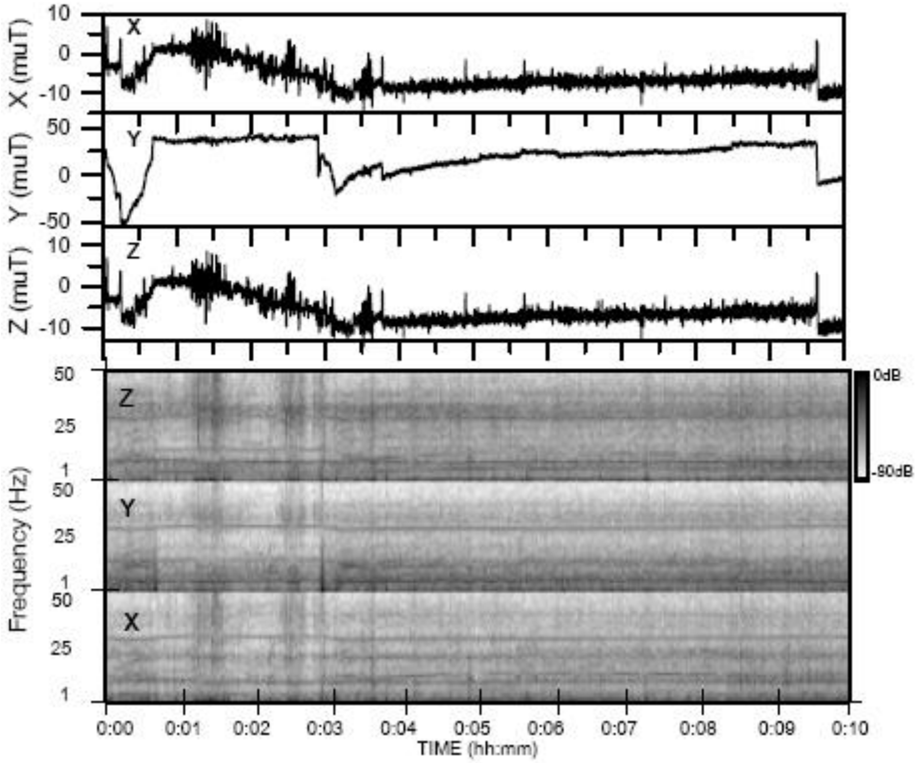

Figure 14. Magnetic field on DC-powered locomotives (engineer's workplace) and dynamic spectra in 1-50 Hz frequency range.

Table 2. Mean values m and root mean squares σ of DC levels in Russian trains

Train	X comp m(muT) σ (muT)		Y comp m(muT) σ(muT)		Z comp m(muT) σ(muT)	
EL	4.3	12.8	17.5	35.1	16.9	27.0
EMU	-8.0	24.5	21.3	31.6	4.6	12.5

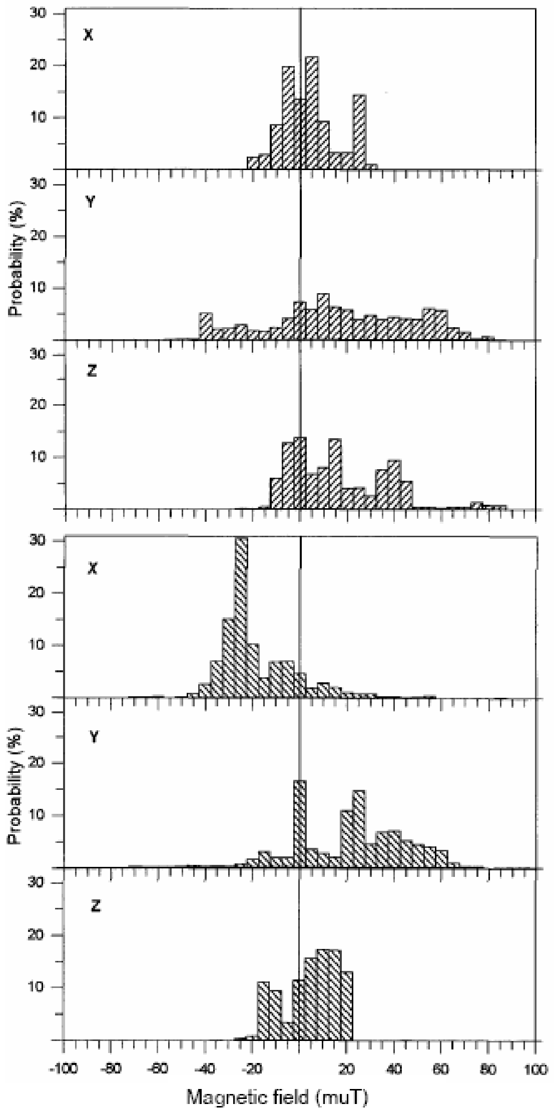

Figure 15. Distribution of quasi-static (<0.03 Hz) magnetic fields, X, Y, and Z components, on DC-powered EL (top) and EMU (bottom).

Distributions of probabilities of DC magnetic field values in EL and EMU trains are presented in Figure 15 as histograms for the three magnetic field components.

The probability for every DC magnetic field amplitude range was constructed as the ratio between the number of DC values getting into a definite interval and the number of all cases. The DC values were calculated as mean values in 30 s intervals. Thus fields at frequencies <0.03 Hz were considered as quasi-static fields (DC fields).

As we can see from Figure 15, DC levels are different for EL and EMU. The most pronounced differences are in X and Z components: X-component DC levels in EL are concentrated around zero, while in EMU they are shifted to negative values; Z-component DC levels in EL shows a much wider distribution, with values up to 90 muT, while in EMU they vary in the range ±20 muT.

The most probable DC levels in Russian DC trains have been found to be in the range 0-35 muT; higher levels of 100-120 muT are more probable for EL than for EMU.

Static magnetic field of the Earth in the site of measurements (St. Petersburg region) is *H*=15 muT, *Z*=49 muT, total *T*=51.5 muT. Thus quasi-static fields in DC trains were bigger than natural geomagnetic field, up to 8-10 times in horizontal components and 2 times in vertical component.

Magnetic field patterns showed irregular temporal variability in the whole studied frequency range (up to 50 Hz); the observed amplitudes of variations ranged from tenths up to tens of muT.

In Figure 16 is presented an example of magnetic field at 12 Hz (filtered data), where this particular component reaches maximum values of 14 muT in X and Z components and 8 muT in Y.

In Table 3 we present the computed rms for data sets filtered at different frequencies; rms were averaged for each magnetic field component over all measurement files.

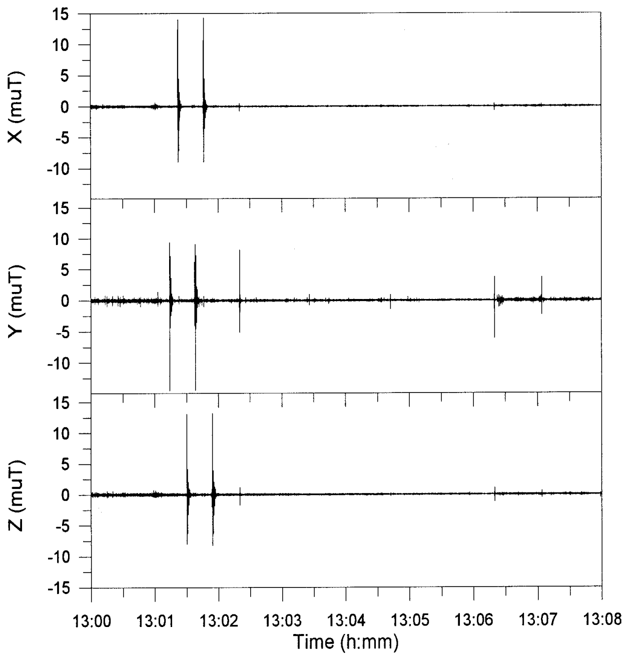

Figure 16. An example of intermittent behavior of magnetic field at 12 Hz. Original data measured in DC-powered EL were filtered by pass-band filter (11-13) Hz.

Table 3. Average rms values computed in different frequency ranges for DC-powered trains

Frequency bands	rms(X) (muT)	rms(Y) (muT)	rms(Z) (muT)
0.02-1 Hz	2.66	5.80	3.98
1-10 Hz	0.55	0.93	0.85
4-5 Hz	1.2	2.0	1.5
7.5-8.5 Hz	0.08	0.16	0.04

2.2.5.Measurements on Swiss AC-powered railways Frequency spectra.

A typical example of magnetic field onboard AC powered (16.67 Hz) locomotive is shown in Figure 17; measurements were conducted along the route Zurich-Bern. The bottom frames of Figure 17 show the 3-component magnetic field variations measured in a 10-min interval, with sampling rate 200 Hz. A more detailed view of actual waveforms in a 20-s sample is plotted at the top frames of Figure 17. This Figure shows "moving pictures" of the measured magnetic fields. It was possible to relate different magnetic field peculiarities to route conditions, as reported in Figure. In the engineer's workplace the main source of variations was concentrated in the catenary/rails circuit.

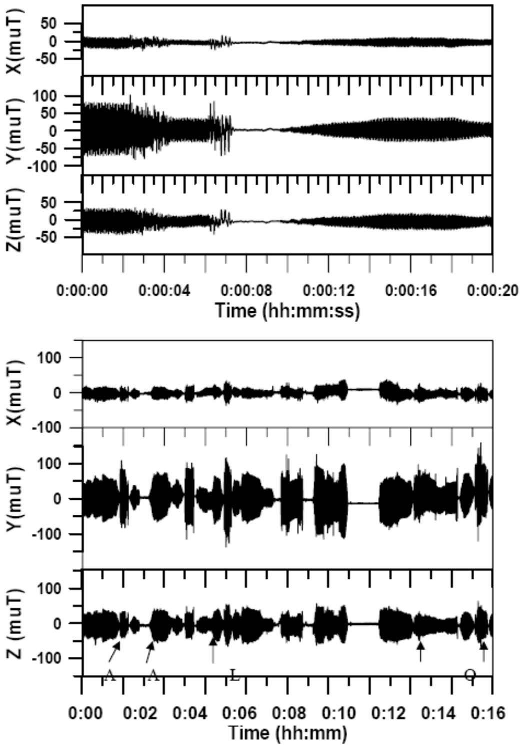

Figure 17. Magnetic field measured onboard Swiss AC (16.67 Hz) electric locomotive in a 20-min interval (lower plot) and in a 20-sec interval (upper plot). A: acceleration, L: Low speed, O: zero current.

The 16.67 Hz component of fields measured onboard Swiss trains (AC supply at 16.67 Hz) is obviously the dominant frequency (see Figure 22). Out of the dominant 16.67 Hz frequency, there are small peaks at higher frequency ranges that can be harmonics of the dominant frequency. In the frequency range below 16.67 Hz there is a clear peak at 5 Hz; this frequency was usually observed during the acceleration phase. A smaller peak at 12 Hz is also present in magnetic field spectra. Figure 18 shows the lowest part of frequency spectrum, 0.1-10 Hz. This Figure demonstrates that multifrequency pattern is a characteristic feature not only for DC- but also for AC-powered rail systems.

We studied the temporal evolution of the multifrequency patterns by means of calculation of dynamic spectra. Figure 19 shows an example of dynamic spectrum (top frames) together with original magnetic field data (bottom). We computed dynamic spectra of Swiss train magnetic field in 0.1-10 Hz frequency range to avoid drastic contribution of the dominant frequency. Examination of Figure 19 shows the presence of magnetic fields bursts in the whole studied frequency range (0.1-10 Hz) in all three magnetic field components, manifested as dark vertical strips. The same kind of bursts in wide frequency range have been also observed in dynamic spectra of magnetic field amplitude in DC EL and EMU. Intervals of intensification of 5 Hz-frequency are also visible in all magnetic field components; they appear during abrupt accelerations.

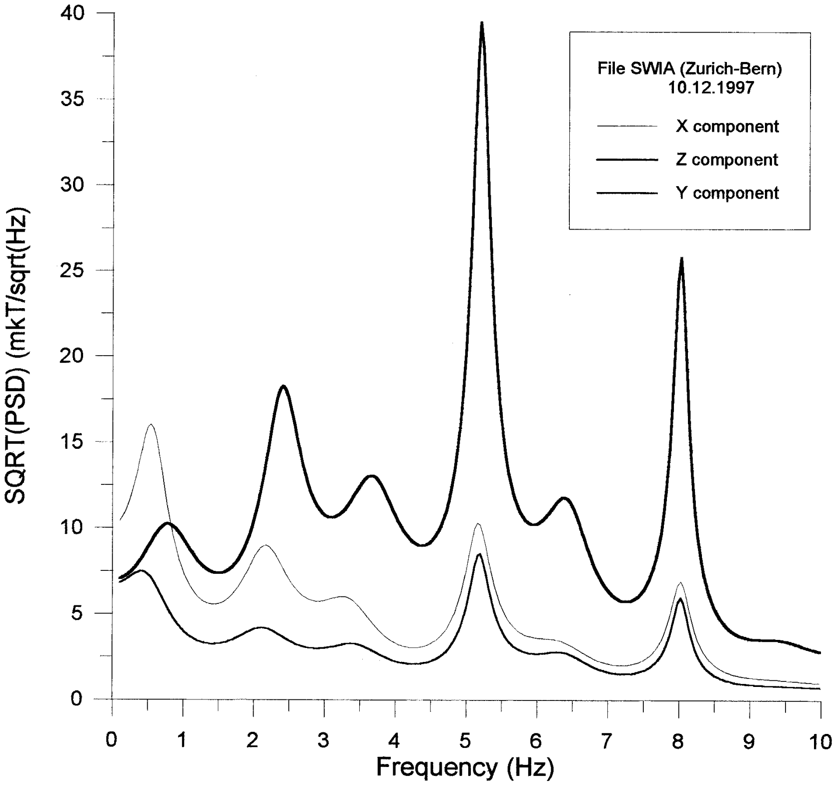

Figure 18. Power spectrum density of magnetic field in 0.2-10 Hz frequency band (horizontal X, Y components, and vertical Z) measured in engineer's workplace of AC (16.67 Hz)-powered locomotive.

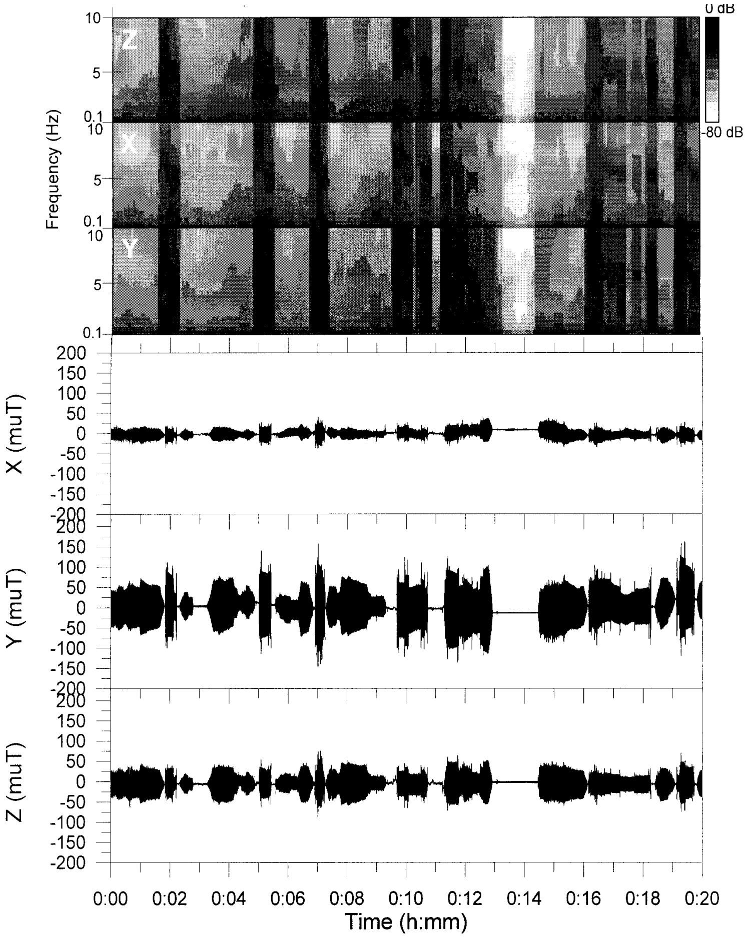

Figure 19. Magnetic field data measured in the engineer's cab of AC locomotive Re 4/4 II and their dynamic power spectra.

To investigate possible contribution of 16.67 Hz in the lower frequency range we computed the dynamic spectrum of the same data filtered by pass-band filter (0.1-10 Hz) (see Figure 20) [13]. It is seen that the dynamic amplitude spectra shown in Figure 19 and Figure 20 are in agreement, in spite of the difference in normalization between the two plots. Results reported in Figure 20 demonstrate the intermittent behavior of the lowest frequency band (0.1-10 Hz).

b. Levels of field. Magnetic fields encountered in AC-powered Swiss trains, as in DC Russian trains, show complex-spectra structure with significant contribution of components below 16.67 Hz. The amplitudes of these magnetic field components in the lowest frequency band in Swiss and Russian trains are comparable, being somewhat higher in Swiss AC trains. In Table 4 we present rms magnetic fields encountered in Swiss electric locomotive (EL Re4/4 II). The rms were calculated for the original data and for data sets filtered in different frequency ranges. We can see that contribution of frequency bands below the principal frequency 16.67 Hz is significant.

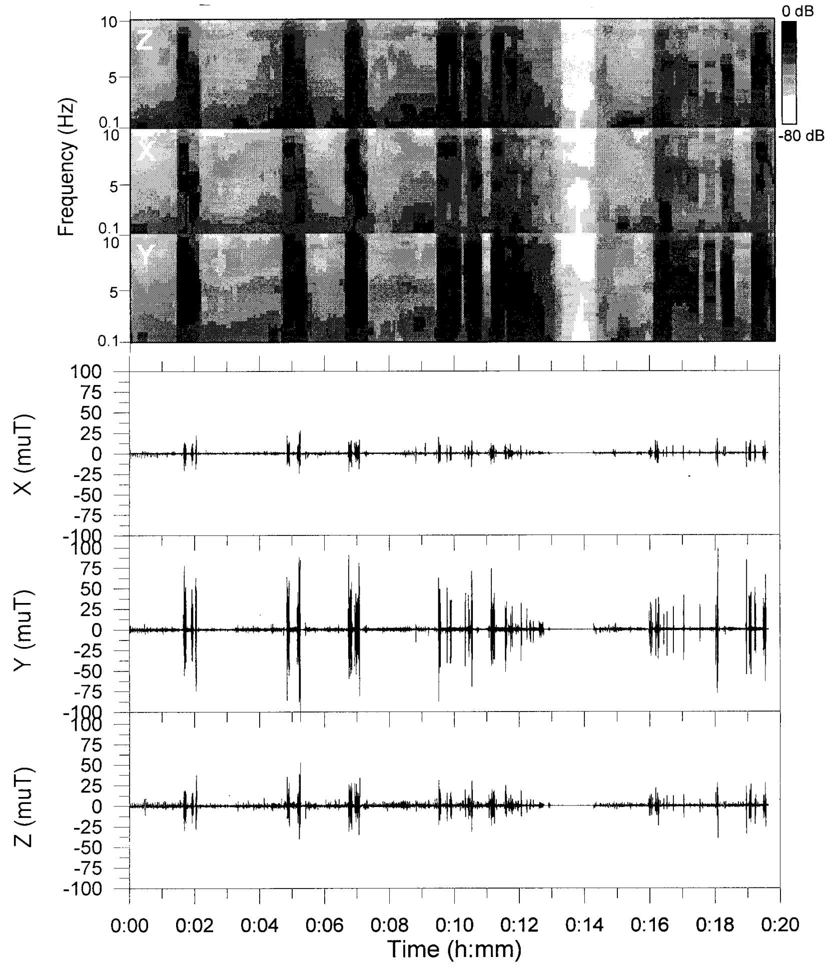

Figure 20. Magnetic field data measured in the engineer's cab of AC locomotive Re 4/4 II filtered by pass-band filter (0.2-10 Hz) and their dynamic power spectra.

Table 4. Average rms values computed in different frequency ranges for AC-powered trains

Frequency bands	rms(X) (muT)	rms(Y) (muT)	rms(Z) (muT)
Original data (0-50 Hz)	10.3	36.9	21.5
0.1-10 Hz	1.7	6.1	2.7
4-5 Hz	0.9	3.6	1.1
7.5-8.5 Hz	0.05	2.0	0.64

Changes in quasi-static fields at frequencies below 0.03 Hz (DC levels) are caused mostly by surrounding ferromagnetic masses or slow changes in direction. These changes in DC level have been estimated to be about 5-10 muT, which are much smaller than changes in Russian DC rail systems. The Earth's magnetic field in the area were magnetic survey in

trains was conducted (Bern region) is *H*=21.5 muT, *Z*= 42 muT, total *T* =47 muT. Thus, changes in DC level in AC trains are lower than the static geomagnetic field value.

3. BIOLOGICALLY IMPORTANT CHARACTERISTICS OF RAILWAY-GENERATED MAGNETIC FIELD

3.1. Plausible Mechanisms of Magnetic Field Interaction with Biological Systems

Magnetic field can penetrate right through bodies since the body tissues introduce a negligible reduction to the magnetic field strength. In fact, the amplitude of magnetic field harmonics at frequency ω in a closed loop at a depth h inside the body will be reduced by a factor

$$f_S(\omega, h, \sigma, \mu) = \exp(-h/\delta), \quad (3.1.1)$$

where the attenuation depth δ depends, according to [170], on permeability μ (≈1) and conductivity $\sigma : \delta = c(2\pi\mu\omega\sigma)^{-1/2}$. For $\omega < 10^6 s^{-1}$ we have $\delta > 10^3 cm$, then, for h≤10 cm, $f_S \approx 1$.

Two general types of effects from exposure to magnetic field have been postulated by theoretical calculations: magnetomechanical and electromagnetic [38].

Magnetomechanical effects can lead to changes in orientation and/or displacement of particles having magnetic properties. In recent years clusters or chains of magnetite crystals ("magnetosomes") were found in several living systems [39], including the human brain [40, 41]. A body of scientific evidence has been obtained indicating that these magnetic elements in migratory birds, fishes and insects are capable of sensing the geomagnetic field and using this information for orientation and navigation. The torque exerted by magnetic field on single-domain crystals, or the elastic effects of interactive forces between smaller, supermagnetic crystals, are somehow transduced into neural impulses that convey relevant information to specific processing centers in the brain [41-43].

The fact that an electrically conducting fluid moving in presence of a magnetic field produces electric currents, gave rise to several studies on magnetohydrodynamic mechanisms resulting in changes in cardiovascular circulatory system [44, 45]. The interaction between a magnetic field and an electric current produces forces which opposes or accelerates the movement of the conducting fluid, depending upon whether the speed of the moving magnetic field is higher or lower than that of the liquid. For instance, in case of static magnetic field and blood flow, the movement will be retarded**.** To produce measurable effects magnetic field values should be in the range of 1-10 T.

Another possibility is provided by Faraday's law of induction that states that time-varying magnetic field generate electric fields in a closed conducting loop.

The magnetic field $\vec{B}$ includes the natural geomagnetic field $\vec{B}_n$ and man-made magnetic field $\vec{B}_m$ and it can be divided in two parts, the constant field $\vec{B}_c = \vec{B}_{nc} + \vec{B}_{mc}$ and the variable one $\vec{B}_v = \vec{B}_{nv} + \vec{B}_{mv}$:

$$\vec{B} = \vec{B}_c + \vec{B}_v = \vec{B}_n + \vec{B}_m = \vec{B}_{nc} + \vec{B}_{nv} + \vec{B}_{mc} + \vec{B}_{mv} \ . \quad (3.1.2)$$

The magnetic flux Φ through a closed loop with length L and surface $\vec{S} = S\vec{n}$, where $\vec{n}$ is the unit vector normal to the surface, is

$$\Phi = \vec{B} \bullet \vec{S} = BS\cos\alpha \ , \quad (3.1.3)$$

α being the angle between $\vec{B}$ and $\vec{S}$. The induced electromotive force Ψ in the closed loop will be, according to Faraday's law, in MSKA system:

$$\Psi = \oint_L \vec{E} \bullet d\vec{l} = -\frac{d\Phi}{dt} = -S\cos\alpha\frac{dB}{dt} - B\cos\alpha\frac{dS}{dt} - BS\frac{d\cos\alpha}{dt} \quad (3.1.4)$$

Eq. (3.1.4) shows that there are three mechanisms of generation of Ψ in closed loops within the body: (i) time change of magnetic field intensity inside body, (ii) time change of cross-section of the closed loop in presence of magnetic field, (iii) time change of the angle α between the magnetic field direction and the normal to the closed loop.

In the Appendix, in accordance with [29], we will consider in detail these different possibilities to generate electromotive forces, by taking into account also rotations and movements of loops related to the body movements [30].

3.2.Magnetic Field Exposure Problem

Since mechanisms through which magnetic field might produce adverse health effects are still obscure, the possible relevant characteristics of magnetic fields are unclear. In most studies magnetic field exposures have been characterized only in terms of time-weighted average of the field strength (TWA). However, there is now evidence that the TWA procedure might not be the best magnetic field exposure metric. Biological and epidemiological studies evidenced that, differently from ionizing radiation and most chemical factors, the effect of low-frequency magnetic field appears to be highly non-linear, as indicated by the possible concern of intensity and frequency "windows" and resonance-like phenomena [46].

Many results lead to the conclusion that biological effects of magnetic fields take place only at particular combinations of frequencies and magnitudes, so-called "window effect" and, in many cases, depend on the field direction relative to the Earth's magnetic field (see e.g. [29] and refs. therein, [47, 48]).

Research on human subjects [49, 50], as well as several other studies (see e.g. [51]) have suggested that intermittent fields (i.e. changes in the steady state of the field in the time scale of seconds and minutes) and irregular waveforms of fields, can be more biologically effective than steady-state fields with regular sinusoidal waveforms. Also polarization, defined by the field 3-dimensional geometry, may play a role [52, 53].

Overall these results emphasize the possible importance of magnetic field exposure parameters other than the customary considered TWA. Thus for an adequate characterization of magnetic field exposure, especially for “real-world” variable multi-frequency transport fields, it is necessary to consider their specific features: complex-frequency characteristics, including intermittent components, and polarization.

We developed methods, algorithms and software to quantify the following aspects of magnetic fields: variability, amplitude-frequency dependencies, intermittency, polarization. However, since we do not know how biological systems interact with magnetic fields, there is no preference to different attributes of magnetic fields to characterize exposure. Thus, for the selection of the more biologically plausible exposure characteristics, we needed additional information. We used, as supplementary criterion for this selection, results reported in Section 1.2.: (i) Epidemiological data on employees of Russian DC-powered railways showed a 2-fold increase in the risk of coronary heart diseases in the job category of EL drivers, as compared to drivers of EMU. Moreover, EL engine drivers showed a higher morbidity at early ages in comparison to EMU engine drivers; (ii) Engine drivers of Swiss AC-powered trains showed a tendency for increased risks for myocardial infarction in comparison with other occupational groups and control, in particular at younger ages.

Thus the comparison of specific characteristics of magnetic fields encountered in Russian and Swiss trains, and the definition of common and different features may be indicative of potentially health-related magnetic field exposure parameters.

To be able to perform this comparative analysis we focused on developing methods of exposure assessment that can be used to evaluate the exposure potential of one type of electrified rail systems against another. Our interest was in defining comparative metrics that can be extracted from practical measurements of "real-word" complex-spectra magnetic field, as transport fields.

3.3. Exposure Assessment Methods for Railway Magnetic Field

We have quantified the following characteristics of "real-world" complex-spectra magnetic field that can be biologically important: amplitude-frequency dependence, polarization and intermittency. For quantifying the above aspects of magnetic field we used usual statistical methods (data filtration by pass-band filter to separate frequency ranges of interest, probability distributions, FFT and power spectrum computation), as well as specially developed methods, algorithms and software. Calculations of values for the considered magnetic field exposure metrics have been done directly from digital data files using algorithms written in FORTRAN and C^{++} languages.

Amplitude-frequency dependence. To estimate and compare amplitude-frequency characteristics for different types of trains we proceeded as follows:

(i) The power spectral densities (PSD) have been calculated by using the maximum entropy method for 250 equally spaced frequencies in the range 0.1-50 Hz for all three magnetic field components.
(ii) The amplitudes of magnetic field variations, for each of the 250 frequencies in every 10 min data file, have been computed as the square root of the PSD value multiplied by the square root of the frequency value.
(iii) The module of the total vector was calculated as the square root of the sum of squares of the three component amplitudes for each frequency.
(iv) Mean values of the amplitudes and 95% confidence intervals have been computed for every frequency by using previous results.

Calculations have been done in the frequency range 0.1-50 Hz for all magnetic field components and for the module of total vector in Russian EL and EMU respectively. As a result we obtained average "typical" curves of amplitude-frequency dependence for Russian trains. For Swiss locomotives, since the statistics was much lower, we calculated only individual amplitude-frequency dependencies in particular for the most prevalent Swiss engine Re 4/4 II.

Polarization. Since the magnetic field is a vector, its changes with time can be described as changes in vector’s length and in angular direction. An oscillation in length only (with a reversal in direction) is named linear polarization; a fixed vector changing only its direction (rotating vector) is named circular polarization. Mixture of linear and circular polarization gives elliptic polarization. For instance, magnetic fields in the vicinity of power lines are typically elliptically polarized.

Generally speaking, elliptical polarization can be obtained by two coils with axes at right angles. If the phase (i.e. the time pattern) of the currents in the coils is the same (phase shift 0° or 180°), the resulting magnetic field is linearly polarized. The phase of the current must be 90° out of step and the intensity of currents must be the same to produce circular polarization.

We elaborated special methods and software to analyze and quantify the polarization structure.

(i) *Qualitative analysis.* This method consists in the visualization of rotation of magnetic field vector in three orthogonal planes XY, YZ, and XZ. Magnetic field vectors were constructed in selected frequency bands. The vectors show the direction and the value of the projection of the full vector of magnetic field variation on each plane. The length and declination angle of vectors have been calculated by using magnetic field components values at steps of 0.1 s.
(ii) *Quantitative analysis.* We elaborated a special method and algorithm based on the computation of the probability distribution of phase shifts between magnetic field components in each of the three planes XY, YZ, and XZ. We proceeded as follows:

- -Magnetic field (all three components) filtration by pass-band filter to separate variations in specific frequency ranges.
- -Each component of 10-min data files was divided in 5-s intervals (120 intervals, each of 500 points).

- -We calculated the phase shift □ (in seconds) between two components in every plane (XY, XZ and YZ) for every interval. To do that a special computer program defines the minimum difference J between two data sets (two magnetic field components) according to the formula

$$J = \sum_{i=1}^{N} \left[B_1(t_i) - B_2(t_i + \tau) \right],$$

where N=500, t_i - current time, B_1 and B_2 - the corresponding magnetic field components.

- -The τ values in seconds have been converted to deg. One period of oscillation is equal to 360°.
- -Probabilities of phase shifts were calculated and plotted as histograms for planes XY, XZ, YZ.

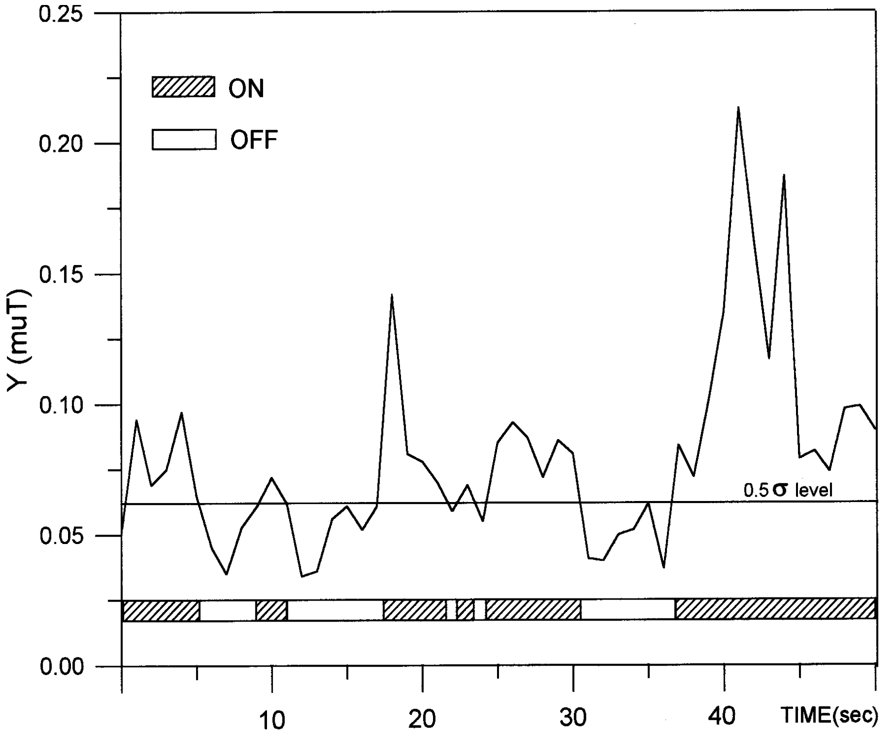

Figure 21. An example of calculation of intermittency characteristics (ON, OFF periods). EL data filtered by pass-band filter (11-13 Hz), rms values computed in 1-sec intervals.

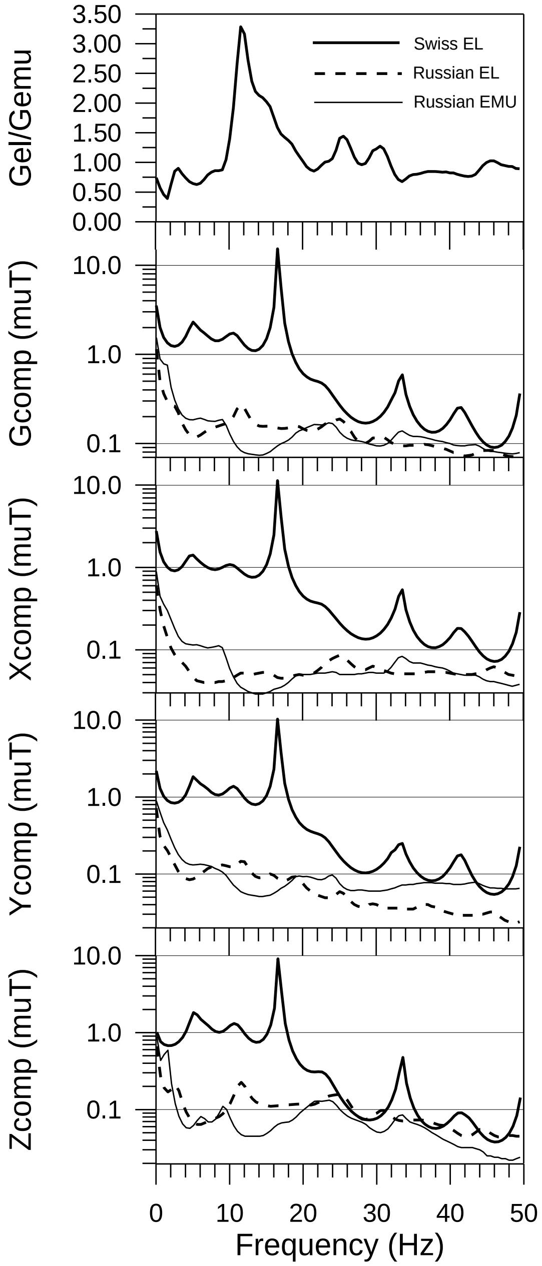

Figure 22. a): the ratio between the average amplitudes of spectral densities of module of magnetic field vector in the engineer's cab of Russian DC-powered locomotives (EL) and of distributed engines trains (EMU). b), c), d), e): the amplitudes of spectral densities of module (B) of magnetic field vector and of magnetic field components (X, Y, Z) for EL (dashed line), for EMU (thin line), and for the AC Swiss EL Re 4/4 II (thick line). respectively [14].

Intermittency. To quantify intermittency it is necessary to introduce a threshold level. Since train-magnetic field exhibits complex-spectra structures, in which different frequencies have different average amplitudes, it is not possible to use a single threshold value appropriate for all frequency ranges, that is, for the observed magnetic field pattern as a

whole. In our analysis we used a fraction of the computed root mean square σ as a threshold. ON intervals were defined as the intervals in which the amplitudes (rms) of magnetic field >0.5σ. We quantified intermittency in different frequency ranges. The method comprises:

(i) Filtration of the data for some selected frequency ranges; computation of σ for the 10-min files; calculation of the mean value of magnetic field (rms) in 1-s intervals and comparison of this value with the corresponding 0.5σ computed in the whole file interval (10 min).Figure 21 illustrates our method of computing ON and OFF intervals for 12 Hz component. This is an example of the analysis done on a 50 s EL magnetic field record; data were filtered by pass-band filter in the frequency range 11-13 Hz.

(ii) Computation of the following features which characterize intermittency:

- -summarized time in which magnetic field is ON in % of all time (10 min);
- -number and duration (in seconds) of ON intervals;
- -histograms of distribution of ON and OFF intervals.

3.4. Magnetic Field Characteristics Beyond TWA

3.4.1. Amplitude-Frequency Dependence

In Figure 22 we present the comparison between average "typical" spectral densities of magnetic field amplitude in DC Russian EL and EMU and in the most prevalent Swiss engine, Re 4/4 II. For AC Swiss locomotive the dominant frequency at 16.7 Hz can be clearly identified, as well as the harmonics at 33.4 Hz and 50 Hz. With regard to the frequency range below 16 Hz, the results show distinct peaks around 5 Hz and 12 Hz. The biggest spectral density components for Russian DC trains are concentrated in quasi-DC range (not shown here). A more detailed spectral analysis is given in Section 4.2.2. In the present Section we will consider the following question: which spectral part is the most relevant for the hypothesized link to myocardial heart attacks? To answer this question we have to look for differences between Russian DC EL and EMU on one side**,** and for congruent patterns between Russian DC EL and Swiss AC EL on the other side. For a better comparison between amplitude spectral densities of Russian EL and EMU, we show in the upper panel of Figure 22 the ratio of amplitudes of magnetic field variations on EL and EMU in the frequency range 0.01-50 Hz. It is seen that the main difference between EL and EMU magnetic field is in the frequency range 7-15 Hz in which the magnetic field variations in EL exceed variations in EMU by 400% (in Z-component this difference is as big as 700%). The maximum of this difference is observed at 12 Hz.

The relevance of the range 7-15 Hz is confirmed by the peak at 12 Hz observed also in Swiss AC EL engines. This peak value is mainly observed in the Z and Y components.

3.4.2. Polarization Characteristics

The qualitative analysis of magnetic field records showed that multi-frequency train fields are characterized by very complex polarization structure. This polarization structure is variable with time and might be different for different frequency ranges. Polarization is defined by route conditions and surrounding ferromagnetic environment [12].

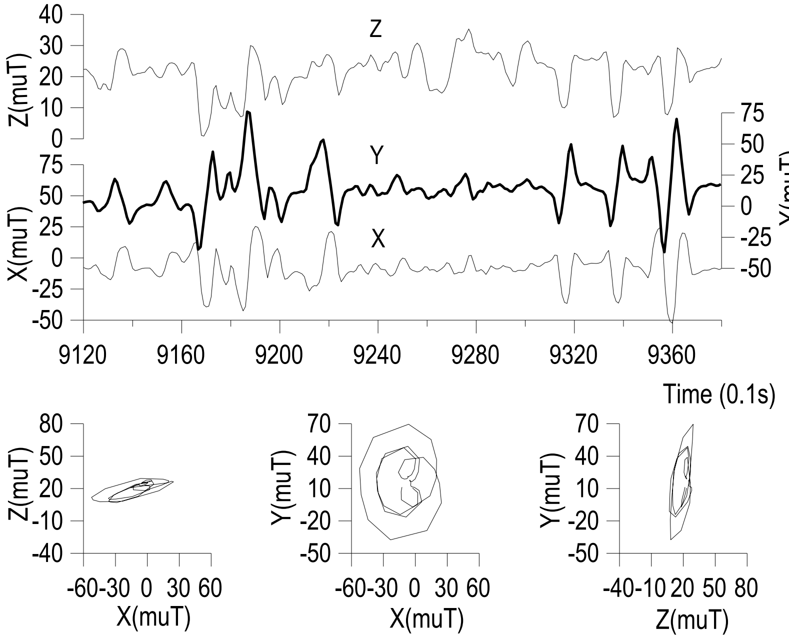

Figure 23. Three-component magnetic field variations (0-5 Hz) measured inside the engineer's cab of EMU train when crossing an oncoming train. Magnetic field hodograms are plotted in three orthogonal planes for the interval 931-937 s.

In Figure 23 we show an example of qualitative analysis of polarization. We can observe quasi circular polarization in horizontal plane XY and elliptical polarization in vertical ZX and ZY planes. For a better illustration we present hodograms for the last 3 pulses in which 3 circles in the XY plane and 3 ellipses in ZX and ZY plane are clearly observed. This particular pattern of polarization was related to a local magnetic anomaly caused by an oncoming train. In this case the train crosses the magnetic field lines distorted by the ferromagnetic masses of oncoming coaches; phase shifts, defining the character of polarization between different components, are observed.

Figure 24 visualizes the behavior of magnetic field vectors in 3 planes (XY, XZ, YZ) for 0.5-1 Hz frequency range. A rotation of the vectors is clearly seen near the center of the analyzed interval.

The further analysis, the quantification of polarization, has been focused on the frequency band 7-14 Hz. This frequency band was defined by results of the above analysis indicating the possible health relation of this frequency "window".

In Figure 25 we show the histograms for the frequency range 10-12 Hz. We consider magnetic field to be circularly polarized in the case of phase shift □ =90°±30°. In this case the ratio of two components, for instance, Y and Z, will be in the range: cos30° < Y/Z< 1/cos30°. Since the two components differ by about 15%, we consider them as equal. In this approximation, magnetic fields encountered in DC locomotives are predominantly circularly polarized.

In Swiss trains we found bipolar distribution of phase shift with main maximum around 90 and secondary maximum around 0 or 180 (linear polarization).

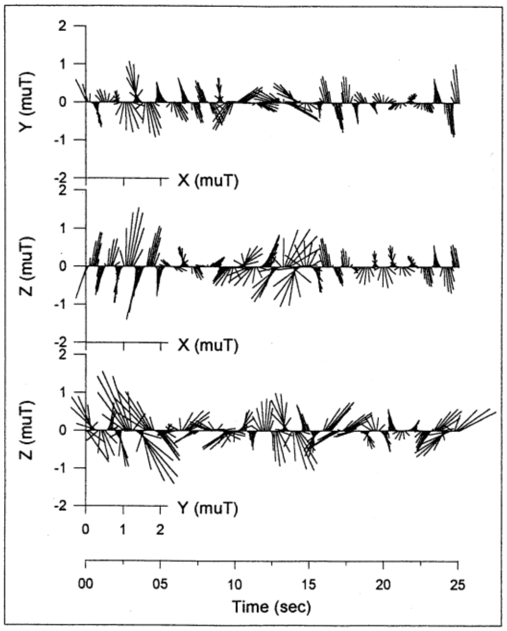

Figure 24. Example of time variations of magnetic field vectors in three orthogonal planes, as measured in an engineer's cab of EMU train.

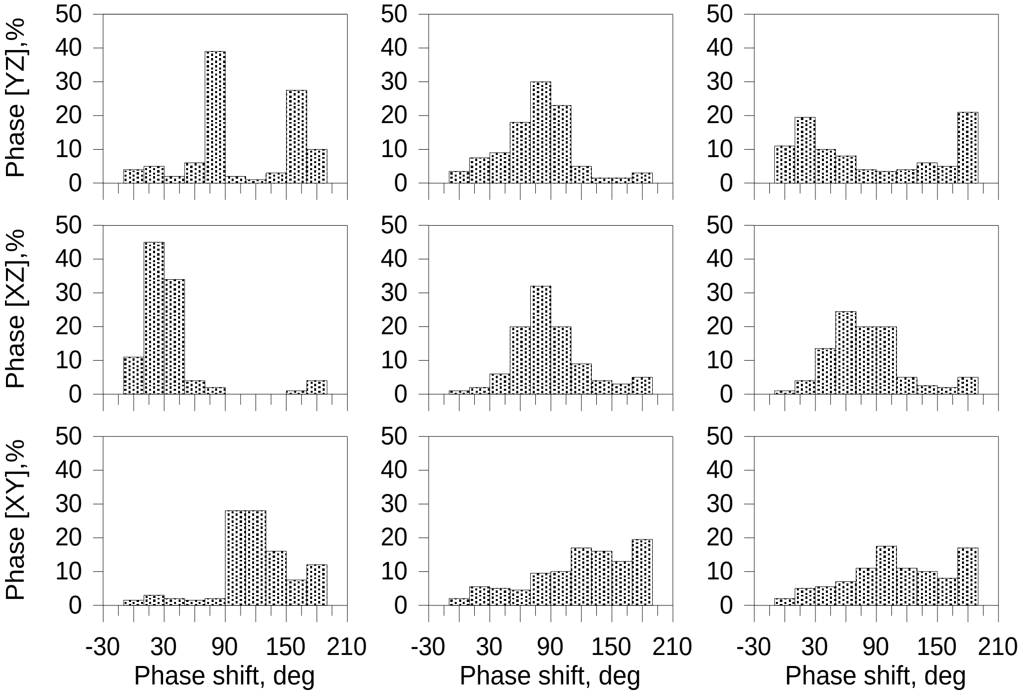

Figure 25. Phase characteristics of magnetic field vectors in three orthogonal planes, as measured in Swiss AC EL (left), Russian DC EL (center), and Russian DC EMU (right), in the range (10-12) Hz.

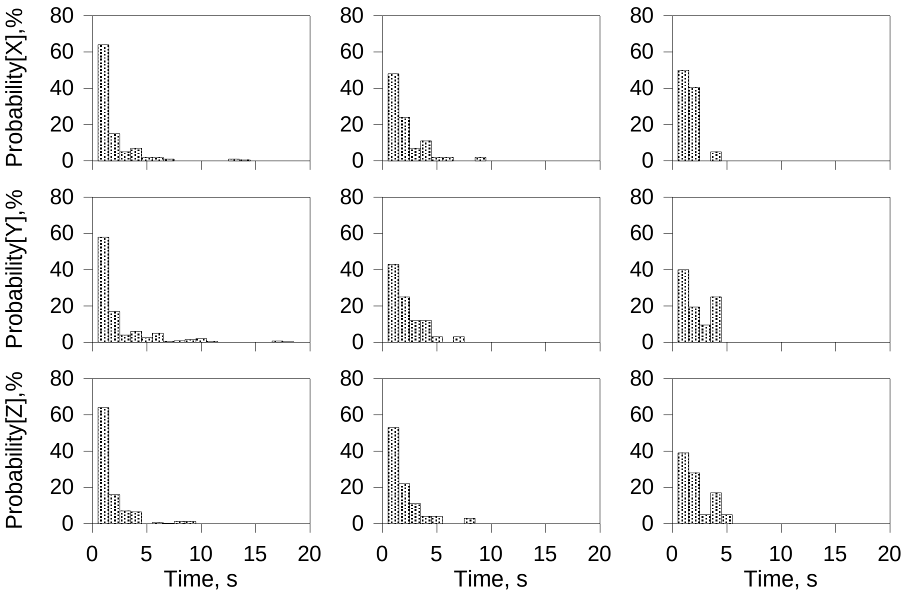

Figure 26. Intermittency characteristics, defined by the ON periods (see the text), of magnetic field components (X, Y, Z) as measured in Swiss AC EL (left), Russian DC EL (center), and Russian DC EMU (right), in the frequency range 10-12 Hz.

These results evidence that circular polarization is the most probable one in magnetic field records onboard Swiss and Russian El along different routes. For instance, the onboard-measured magnetic field in YZ plane in the frequency band 10-14 Hz is circularly polarized during 75% of time for Russian, and 50% for Swiss electric locomotives. This circular polarization is observed in a vertical or near-vertical plane relative to the Earth's surface. The declination of this plane, in which the magnetic field vector is rotating, changes with time.

For EMU the histograms of probabilities of phase shifts between magnetic field components are almost flat without any distinct peaks. Flat distribution of phase shifts evidence that for EMU a much more irregular polarization structure of magnetic field is observed, as compared with EL: usually EMU magnetic field are elliptically polarized in all three planes.

3.4.3. Intermittency Characteristics

Bottom frames of Figure 20 show the time behavior of the lowest frequency band 0-10 Hz observed in AC locomotive. These time domain plots demonstrate the intermittent behavior of magnetic field in this frequency range: bursts of increased magnetic field amplitudes (up to 90 muT in Y-component) on the much lower background level of about 1 muT. Figure 16 shows a typical example of time series of a “single” 12 Hz component in DC-powered locomotive EL, which also demonstrates an intermittent character of fields. One can observe bursts of increased amplitudes up to 10-15 muT on a background level lower than 1 muT. For EMU maximum amplitudes of bursts were as big as 2-3 muT. Characteristics of intermittency, for instance the time length of a burst (ON-regime period) and the time between bursts (OFF-regime periods), depend on the chosen threshold level [14].

Table 5. Characteristic features of magnetic field intermittency in different trains

Train type	Frequency (Hz)	Parameter	Magnetic field Component		
			X	Y	Z
AC EL (Swiss)	10-12	σ (muT)<br>ON (%)	0.58<br>38.6	2.32<br>51.2	0.92<br>38.1
DC EL (Russian)	10-12	σ (muT)<br>ON (%)	0.05<br>47.1	0.15<br>49.5	0.15<br>45.4
DC EMU (Russian)	10-12	σ (muT)<br>ON (%)	0.11<br>35.4	0.10<br>38.1	0.17<br>30.1
AC EL (Swiss)	8-14	σ (muT)<br>ON (%)	0.95<br>45.8	3.74<br>55.8	1.63<br>44.8
DC EL (Russian)	8-14	σ (muT)<br>ON (%)	0.09<br>46.4	0.27<br>49.5	0.25<br>48.8
DC EMU (Russian)	8-14	σ (muT)<br>ON (%)	0.18<br>36.2	0.20<br>39.4	0.25<br>38.9

The distributions of intermittency characteristics computed on a σ-based threshold (see Section 3.2) are plotted in Figure 26 for X, Y, and Z components of the magnetic field data in the 10-12 Hz frequency range. Occurrences of ON-regime periods are reported in this Figure. The ON-regime is the time interval in which the amplitude of magnetic field variations exceeds the level 0.5σ. As it can be seen, ON intervals last from 1 to 15 s. The shortest intervals of 1 s are the most probable ones for all types of trains and for all three components. The probability of existence of longer ON-regime intervals decreases exponentially. It means that the magnetic variations defining the intermittency are generated by occasional processes. In Table 5 we show values of σ for 10-min files and ON- regime (in % of the total period).

It can be seen that the amplitudes of magnetic variations are bigger for AC EL data, both in 10-12 Hz and 8-14 Hz frequency ranges. The occurrence of ON-regime is in the range 35-50 % for all types of trains.

Our results do not reveal any special differences in intermittency characteristics between EL (both AC and DC) and EMU.

4. Conclusion

We have conducted an extensive study of magnetic fields on different electrified transport systems. The studied transport technologies were: (i) Russian DC locomotives EL; (ii) Swiss AC (16.67 Hz) locomotives (EL); (iii) Russian trains EMU formed by a number of self-powered electric motor units and units without motors. Measurements have been performed by means of the portable computer-based waveform capture system MVC-3 with sampling rate up to 200 Hz. As a result of measurements we formed a database containing about 60 hours of continuous magnetic field recording in 0-50 Hz frequency range. Files are followed by legends with information on route conditions (changes of engines' current with

time and speed, acceleration, idling and braking phases, stops, power substations, oncoming trains, railway switches, bridges, etc.).

Magnetic fields encountered both on DC powered and AC powered transport systems are different from power-line fields which are predominantly sinusoidal with main frequency at 50 or 60 Hz. Transport magnetic fields present complex patterns resulting from the superposition of variations with different amplitudes, frequencies and different geometry. The frequency of observed magnetic field variations depends on the train's speed: the higher frequencies being measured under higher speed. The amplitude of the variations was also bigger under higher speed.

We showed how exposure (beyond TWA) to these complex magnetic fields might be estimated to evaluate differences and similarities in magnetic fields encountered in workplaces of engineers of various trains. To do that we elaborated for the first time a set of methods, algorithms and software to quantify the following specific features of complex-spectra train magnetic field: amplitude-frequency dependence, polarization and intermittency.

The comparative analysis of specific features of magnetic field encountered onboard DC- and AC-powered trains may be summarized as follows:

a) *Frequency spectra.* The most pronounced difference between spectral structures of magnetic field in Russian and Swiss trains was the dominant frequency 16.67 Hz in Swiss AC trains. However, Swiss magnetic fields, as Russian magnetic fields, show complex spectral structure with significant contribution of components below 16.67 Hz. Higher harmonics were also present. The main difference between magnetic fields encountered in DC EL and DC EMU was in the frequency range 7-15 Hz. In this range magnetic field variations in EL exceed variations in EMU by 350-700%, reaching the maximum difference at ~12 Hz. In this frequency range a peak value at 12 Hz in Swiss AC EL was also observed. The congruency appears to be more pronounced in the Z-component. The possible significance of the frequency "window" 7-15 Hz is in agreement with results of biological studies [54-56] in which it was reported that neurophysiological and cardiovascular effects of magnetic field appeared to be more pronounced for frequencies within this window.

b) *Amplitude-frequency characteristics.* The greatest quasi-static variations in DC Russian transport systems were observed in Y component perpendicular to rails, where peak-to-peak values reached 120 muT in EL during acceleration phases or when passing substations. The most probable DC levels in Russian DC trains were in the range 0-35 muT; higher levels of 100-120 muT are more probable for EL than for EMU. In AC Swiss trains these changes might be about 5-10 muT, being caused mostly by surrounding ferromagnetic masses or slow changes in train’s direction. Magnetic field encountered both in DC and AC powered rail systems showed irregular temporal variability in the whole studied frequency range (0-50 Hz). Average amplitudes of observed variations ranged from tenths of muT to several muT, maximum values were tens of muT, being even much higher for Swiss AC-powered locomotives. In AC trains the highest magnetic field values have been observed at the dominant frequency 16.67 Hz. In the most popular AC engine Re 4/4 II average values of about 10 muT in X, 40 muT in Y, 20 muT in Z component have been found.Changes in quasi-static fields in DC Russian trains in horizontal

components were 8-10 times bigger and in vertical component 2 times bigger than the geomagnetic field in the site of measurements (St. Petersburg region): *H*=15 muT, *Z*=49 muT, *T*=51.5 muT); in AC trains (Bern region: *H*=21.5 muT, *Z*= 42 muT, *T* =47 muT) these fields have been estimated somehow lower than the geomagnetic background.

c) *Polarization.* Magnetic fields show complex polarization structure, highly variable with time and different for different frequency ranges. Typically they were elliptically polarized as power-line fields. However, magnetic fields onboard both DC and AC-powered locomotives, in contrast to EMU trains, show a tendency for more regular polarization pattern in planes perpendicular to the Earth's surface: in this planes they were predominantly circularly polarized during considerable time. The declination of this plane, in which the magnetic field vector was rotating, changed with time.There is laboratory evidence pointing to a mechanism involving heart rate variability: in a recent human experimental study the nocturnal exposure to an intermittent, circularly polarized 60 Hz magnetic field at 20 muT significantly reduced heart rate variability [53]. This finding deserves particular attention since such reductions appear to be clinically relevant prognostic factors for cardiovascular risks. In this context the presence of circularly polarized magnetic fields in EL supports our results that magnetic fields encountered in EL have more pronounced health-hazardous potential than EMU magnetic field (see Section 1.2.2).

*Intermittency***.** Intermittency is a characteristic feature of both Russian and Swiss railway magnetic fields. Periods in which magnetic field >0.5σ (ON intervals) varied from 1 to 10-12 s. The shortest ON intervals were the most probable ones. Our results did not reveal any special differences in intermittency characteristics between EL (both AC and DC) and EMU.

Then, we can conclude that there are common features in magnetic field encountered both in DC and AC-powered railway systems. These magnetic field features might be responsible for similar adverse health effects in particular, in cardiovascular system (increase of myocardial infarctions etc) found for locomotive engineers powered both by DC and AC currents,. Our special animal experiment [57] clarified plausible biological pathways for such health effects. The common and peculiar features of magnetic field spectra encountered in Swiss AC and Russian DC railways helped to develop "typical railway" patterns. Such "railway" magnetic field patterns have been simulated in laboratory studies on biological responses in mice. Found results indicate that magnetic field with highly variable complex spectra, typical for railway magnetic environments, can be one of the factors increasing the loading for regulatory mechanisms of cardiovascular system. This increased loading could lead to development of classical pathogenesis states such as myocardial ischemia, arrhythmia and others, which lead to increased risk of cardio-vascular catastrophes [57].

Recognition of the main spectral features and other peculiarities of magnetic field recorded in Russian DC and Swiss AC trains connected to different route conditions can be useful in the identification of sources of these specific features. It will allow developing design-related preventive measures to diminish the health-hazardous potential of magnetic field. In particular, the similar geometrical regularity of polarization structure (predominantly circular polarization in vertical planes) of magnetic fields, found for Swiss and Russian electric locomotives EL, could be indicative of common peculiar constructing features

responsible for this kind of polarization. In case of further supporting evidence for potential cardiovascular risks of circularly polarized magnetic fields it could be possible to elaborate cost-effective preventive measures to diminish this component “at the source”.

APPENDIX

A.1. Electromotive Forces Ψ Produced by the Time Change of Magnetic Field Intensity within the Human Body

Let us consider in more detail the first term in the right-hand side of (3.1.4):

$$\Psi_1 = -S\cos\alpha \frac{dB}{dt} = -S\cos\alpha\left(\frac{\partial B}{\partial t} + \frac{\partial B}{\partial \vec{r}}\vec{V}\right) = \\ -S\cos\alpha_v\left(\frac{\partial B_v}{\partial t}\right) - S\cos\alpha_c \frac{\partial B_c}{\partial \vec{r}}\vec{V} - S\cos\alpha_v \frac{\partial B_v}{\partial \vec{r}}\vec{V}, \qquad (A.1.1)$$

where $\vec{V}$ is the velocity of the closed loop (which in most cases coincides with the velocity of the body) relative to the system of coordinates in which magnetic field (MF) is considered.

The first term in the right-hand side of (A.1.1) reflects the *influence of variable part of MF*:

$$\Psi_{11} = -S\cos\alpha\left(\frac{\partial B_v}{\partial t}\right) = -S\cos\alpha_n\left(\frac{\partial B_{nv}}{\partial t}\right) - S\cos\alpha_m\left(\frac{\partial B_{mv}}{\partial t}\right) = \\ S\sum_{i=1}^{l}\omega_{ni}B_{ni}\sin(\omega_{ni}t - \varphi_{ni})\cos\alpha_{ni} + S\sum_{k=1}^{p}\omega_{mk}B_{mk}\sin(\omega_{mk}t - \varphi_{mk})\cos\alpha_{mk} \qquad (A.1.2)$$

where we assumed that

$$B_{nv} = \sum_{i=1}^{l} B_{ni}\cos(\omega_{ni}t - \varphi_{ni}), \quad B_{mv} = \sum_{k=1}^{p} B_{mi}\cos(\omega_{mi}t - \varphi_{mi}), \qquad (A.1.3)$$

and considered that the angle α can be different for different harmonics: α_{ni} for natural geomagnetic field and α_{mk} for man-made MF.

Let us consider the second term of (A.1.1), i.e. the case in which ψ *is produced by the movement of the loop in presence of a space gradient in constant part of MF*:

$$\Psi_{12} = -S\cos\alpha \frac{\partial B_c}{\partial \vec{r}}\vec{V} = -S\cos\alpha_n \frac{\partial B_{nc}}{\partial \vec{r}}\vec{V} - S\cos\alpha_m \frac{\partial B_{mc}}{\partial \vec{r}}\vec{V} \qquad (A.1.4)$$

In this case the contribution of geomagnetic field B_{nc}, even in the case of a body moving with great velocity (for instance $8 \cdot 10^3$ ms^{-1} of spacecrafts) is very small due to the very small value of $\partial B_{nc}/\partial \vec{r}$, while the contribution of man-made MF in (A.1.4) can be much more important. The characteristic frequency of this Ψ will be $\nu_{mc} \approx V / r_{mc}$, where r_{mc} is the characteristic distance for man-made MF to change by a factor *e*. For example, if a person runs with $V \approx 4\ m/s$ and $r_{mc} \approx 2\ m$, then $\nu_{mc} \approx 2\ Hz$ which can be near to self frequencies in the human body. This is the case in which the resonance interaction can be realized. Let us assume that

$$\frac{\partial B_c}{\partial \vec{r}} \vec{V} \approx \frac{\partial B_{mc}}{\partial \vec{r}} \vec{V} = \sum_{r=1}^{x} b_{cr} \cos(\omega_{cr} t - \varphi_{cr}) \qquad \text{(A.1.5)}$$

and introduce this value in (A.1.4), thus obtaining for the influence of about constant part of MF:

$$\Psi_{12} = -S \sum_{r=1}^{x} b_{cr} \cos\alpha_{cr} \cos(\omega_{cr} t - \varphi_{cr}) \qquad \text{(A.1.6)}$$

in which it is taken into account that the angles α_{cr} can be different for different harmonics ω_{cr}.

Now we consider the last term in (A.1.1):

$$\Psi_{13} = -S\cos\alpha \frac{\partial B_v}{\partial \vec{r}} \vec{V} = -S\cos\alpha_n \frac{\partial B_{nv}}{\partial \vec{r}} \vec{V} - S\cos\alpha_m \frac{\partial B_{mv}}{\partial \vec{r}} \vec{V} \qquad \text{(A.1.7)}$$

which is determined by the *movement of the closed loop in presence of a gradient in the variable part of MF*. Also in this case the contribution of natural geomagnetic field can be neglected, then the only relevant effect is due to the gradient of variable part of man-made MF on moving body. Assuming that, as in (A.1.3), $B_v \approx B_{mv} = \sum_{k=1}^{p} B_{mk} \cos(\omega_{mk} t - \varphi_{mk})$ and that, as in A.1.5),

$$\frac{\partial B_{mk}}{\partial \vec{r}} \vec{V} = \sum_{j=1}^{q} b_{mkj} \cos(\omega_{mkj} t - \varphi_{mkj}), \qquad \text{(A.1.8)}$$

we obtain:

$$\Psi_{13v} = -S\sum_{k=1}^{p}\sum_{j=1}^{q} b_{mkj}\cos\alpha_{kj}\cos(\omega_{mk}t-\varphi_{mk})\cos(\omega_{mkj}t-\varphi_{mkj}) =$$

$$-S\sum_{k=1}^{p}\sum_{j=1}^{q} b_{mkj}\cos\alpha_{kj}\left\{\cos\left[(\omega_{mk}+\omega_{mkj})t-(\varphi_{mk}+\varphi_{mkj})\right]+\right. \tag{A.1.9}$$

$$\left.\cos\left[(\omega_{mk}-\omega_{mkj})t-(\varphi_{mk}-\varphi_{mkj})\right]\right\}$$

Eq. (A.1.9) shows that in this case there is interference of harmonics contained in B_{mv}, according to (A.1.3), and harmonics arising from the body's movement, according to (A.1.8). As a result of this interference the induced Ψ in the closed loop is formed by harmonics at frequencies $|\omega_{mk} \pm \omega_{mkj}|$ and amplitudes $Sb_{mkj}\cos\alpha_{kj}$.

A.2. Induced Ψ caused by changes in cross-section of closed loops in presence of MF of natural and man-made origin

We consider here the second term in (3.1.4):

$$\Psi_2 = -B\cos\alpha\frac{dS}{dt} = -B\cos\alpha\frac{\partial S}{\partial t} - B\cos\alpha\frac{\partial S}{\partial \vec{r}}\vec{V} . \tag{A.2.1}$$

The first part reflects the ψ produced by the time variations of the effective cross section of closed loops (for example the rhythmical changes in breathing, in heart beating, etc.) in natural and man-made MF. The second part reflects the situation when the change in S is caused by the change in position of closed loop with velocity V (e.g. the case of a moving body).

In the first part of (A.2.1), due to the *change of S with time,*

$$\Psi_{21} = -\left|\vec{B}_{nc}+\vec{B}_{mc}\right|\cos\alpha\frac{\partial S}{\partial t} - \left|\vec{B}_{nv}+\vec{B}_{mv}\right|\cos\alpha\frac{\partial S}{\partial t} . \tag{A.2.2}$$

the contribution of both constant MF, of natural and man-made origin, are present and the Ψ .strength is proportional to the total intensity of natural and man-made MF.

Supposing that the change of S with time can be described by

$$S(t) = \sum_{j=1}^{q} S_j\cos(\omega_{sj}t-\varphi_{sj}), \tag{A.2.3}$$

the contribution of the constant MF to Ψ_{21} will be

$$\Psi_{21c} = \left|\vec{B}_{nc} + \vec{B}_{mc}\right| \cos\alpha \sum_{j=1}^{q} \omega_{sj} S_j \sin(\omega_{sj} t - \varphi_{sj}). \quad \text{(A.2.4)}$$

The about constant part of man-made MF in specific places, for instance inside public transport, can be much bigger than natural field and it can generate much bigger Ψ in the rhythmically changing closed loops according to (A.2.4).

Let us assume that, for the effect of changes of S with time in presence of variable MF, the variable MF can be represented as in (A.1.3) and the change of S by (A.2.3). In this case we obtain:

$$\Psi_{21v} = \sum_{i=1}^{l}\sum_{j=1}^{q} B_{ni}\omega_{sj}S_j \cos\alpha_{ij} \cos(\omega_{ni} t - \varphi_{ni}) \sin(\omega_{sj} t - \varphi_{sj}) +$$

$$\sum_{k=1}^{p}\sum_{j=1}^{q} B_{mk}\omega_{sj}S_j \cos\alpha_{kj} \cos(\omega_{mk} t - \varphi_{mk}) \sin(\omega_{sj} t - \varphi_{sj}) =$$

$$\sum_{i=1}^{l}\sum_{j=1}^{q} B_{ni}\omega_{sj}S_j \cos\alpha_{ij} \left\{\sin\left[(\omega_{ni} + \omega_{sj})t - (\varphi_{ni} + \varphi_{sj})\right] + \sin\left[(\omega_{sj} - \omega_{ni})t - (\varphi_{sj} - \varphi_{ni})\right]\right\}$$

$$+\sum_{k=1}^{p}\sum_{j=1}^{q} B_{mk}\omega_{sj}S_j \cos\alpha_{kj} \left\{\sin\left[(\omega_{mk} + \omega_{sj})t - (\varphi_{mk} + \varphi_{sj})\right] + \sin\left[(\omega_{sj} - \omega_{mk})t - (\varphi_{sj} - \varphi_{mk})\right]\right\}. \quad \text{(A.2.5)}$$

Eq. (A.2.5) reflects the nonlinear interference of different harmonics of both variable natural and man-made MF, with harmonics describing the change in time of closed loops. This interference will produce new harmonics at frequencies $\omega_{ni} \pm \omega_{sj}$ and $\omega_{mk} \pm \omega_{sj}$ with amplitudes $B_{ni}\omega_{sj}S_j \cos\alpha_{ij}$ and $B_{mk}\omega_{sj}S_j \cos\alpha_{kj}$.

We consider now the second term of the right-hand side of (A.2.1), which reflects the situation when the *change of S is caused by the change in position of closed loop in space* with velocity $\vec{V} = d\vec{r}/dt$. This change of S can be rhythmical as, for example, during walking, running and exercising:

$$\Psi_{22} = -\left|\vec{B}_{nc} + \vec{B}_{mc}\right| \cos\alpha \frac{\partial S}{\partial \vec{r}} \vec{V} - \left|B_{nv} + B_{mv}\right| \cos\alpha \frac{\partial S}{\partial \vec{r}} \vec{V} \ . \qquad (A.2.6)$$

Assuming that $\frac{\partial S}{\partial \vec{r}} \vec{V}$ can be represented as

$$\frac{\partial S}{\partial \vec{r}} \vec{V} = \sum_{j=1}^{x} s_j \cos\left(\omega_{vj} t - \varphi_{vj}\right) \qquad (A.2.7)$$

in the presence of about constant parts of natural (B_{nc}) and man-made (B_{mc}) MF, the contribution of B_c to Ψ_{22} can be written as

$$\Psi_{22c} = -B_{nc} \cos\alpha_{nc} \sum_{j=1}^{x} s_j \cos\left(\omega_{vj} t - \varphi_{vj}\right) - B_{mc} \cos\alpha_{mc} \sum_{j=1}^{x} s_j \cos\left(\omega_{vj} t - \varphi_{vj}\right). \qquad (A.2.8)$$

Here we considered that the angles α_{nc}, α_{mc} between the normal to the closed loop and the direction of MF can be different for natural and man-made fields. In (A.2.8) the first term in the right-hand side reflects mostly a natural situation, to which people are adjusted by long-term evolution. The second term, which reflects the influence of about constant part of man-made MF, can be more important, especially in urban and technological areas, including public transport.

We consider now the second term in the right-hand side of (A.2.6), i.e. the influence of the variable part of MF on a changing S caused by the movement of the loop, by taking into account (A.2.7) and (A.1.3) for the variable part of MF. We obtain:

$$\Psi_{22v} = -\sum_{i=1}^{l} \sum_{j=1}^{q} B_{ni} s_j \cos\alpha_{ni} \cos\left(\omega_{ni} t - \varphi_{ni}\right) \cos\left(\omega_{vj} t - \varphi_{vj}\right) -$$

$$\sum_{k=1}^{p} \sum_{j=1}^{q} B_{mk} s_j \cos\alpha_{mk} \cos\left(\omega_{mk} t - \varphi_{mk}\right) \cos\left(\omega_{vj} t - \varphi vj\right) =$$

$$\left(1/2\right) \sum_{i=1}^{l} \sum_{j=1}^{q} B_{ni} s_j \cos\alpha_{ni} \left\{\cos\left[\left(\omega_{ni} + \omega_{vj}\right) t - \left(\varphi_{ni} + \varphi_{vj}\right)\right] + \cos\left[\left(\omega_{vj} - \omega_{ni}\right) t - \left(\varphi_{vj} - \varphi_{ni}\right)\right]\right\} \qquad (A.2.9)$$

$$+\left(1/2\right)\sum_{k=1}^{l}\sum_{j=1}^{q} B_{mk} s_j \cos\alpha_{mk} \left\{\cos\left[\left(\omega_{mk}+\omega_{vj}\right)t-\left(\varphi_{mk}+\varphi_{vj}\right)\right]+\right.$$
$$\left.\cos\left[\left(\omega_{vj}-\omega_{mk}\right)t-\left(\varphi_{vj}-\varphi_{mk}\right)\right]\right\}$$

where the two parts reflect the influence of variable natural (n) and man-made (m) MF, respectively. Eq. (A.2.9) shows that instead of harmonics with frequencies ω_{nk}, ω_{vj} and ω_{mk}, ω_{vj} we obtain, after the interaction of the variable MF with the rhythmical movement of body, a Ψ in moving closed loops characterized by harmonics with frequencies $\left|\omega_{ni} \pm \omega_{vj}\right|$ and amplitudes $\left(1/2\right) B_{ni} s_j \cos\alpha_{ni}$ for natural MF and $\left|\omega_{mk} \pm \omega_{vj}\right|$ and $\left(1/2\right) B_{mk} s_j \cos\alpha_{mk}$ for man-made MF.

A.3. Induced Ψ FEs in closed loops caused by changes of the angle between the normal to the loop and MF direction

We consider here the third term in (3.1.4), which reflects the generation of induced Ψ in closed loops caused by changes of the angle α between the normal to the loop and MF direction:

$$\Psi_3 = -BS\frac{d\cos\alpha}{dt} = -BS\frac{\partial\cos\alpha}{\partial t} - BS\frac{\partial\cos\alpha}{\partial\vec{r}}\vec{V}\ . \qquad \text{(A.3.1)}$$

The first part in the right-hand side reflects the generation of induced Ψ in closed loops caused by changes with time of the angle α, and the second part the induced Ψ caused by the body movement accompanied by changes in α.

We consider the first term in the right-hand side of (A.3.1):

$$\Psi_{31} = -BS\frac{\partial\cos\alpha}{\partial t} = S\left|\vec{B}_{nc}+\vec{B}_{mc}\right|\sin\alpha\frac{\partial\alpha}{\partial t} + S\left|\vec{B}_{nv}+\vec{B}_{mv}\right|\sin\alpha\frac{\partial\alpha}{\partial t}\ . \qquad \text{(A.3.2)}$$

Let suppose that the body rotates in some relatively short time period and this rotation can be characterized by frequency ω_r and phase φ_r: $\alpha = \omega_r t - \varphi_r$. In this case the first part in the right-hand side of (A.3.2) will be

$$\Psi_{31c} = S\left|\vec{B}_{nc}+\vec{B}_{mc}\right|\omega_r \sin\left(\omega_r t - \varphi_r\right) \qquad \text{(A.3.3)}$$

For the part in (A.3.2), which reflects the role of variable part of MF of natural and man-made origin, if we suppose again that the body in some relatively short time period rotates

with frequency ω_r characterized by phase φ_r, we obtain for variable MF described by (A.1.3):

$$\Psi_{31v} = S\sum_{i=1}^{l} \omega_r B_{ni} \cos(\omega_{ni} t - \varphi_{ni}) \sin(\omega_r t - \varphi_{nr}) + S\sum_{k=1}^{p} \omega_r B_{mk} \cos(\omega_{mk} t - \varphi_{mk}) \sin(\omega_r t - \varphi_{mr}), \qquad \text{(A.3.4)}$$

which can be written as

$$\Psi_{31v} = S\omega_r/2 \left\{ \sum_{i=1}^{l} B_{ni} \left[\sin\left((\omega_r + \omega_{ni})t - (\varphi_{nr} + \varphi_{ni})\right) + \sin\left((\omega_r - \omega_{ni})t - (\varphi_{nr} - \varphi_{ni})\right)\right] + \sum_{k=1}^{p} B_{mk} \left[\sin\left((\omega_r + \omega_{mk})t - (\varphi_{mr} + \varphi_{mk})\right) + \sin\left((\omega_r - \omega_{mk})t - (\varphi_{mr} - \varphi_{mk})\right)\right] \right\}. \qquad \text{(A.3.5)}$$

Also in this case harmonics of induced Ψ will be generated with amplitudes $(1/2)S\omega_r B_{ni}$ and frequencies $|\omega_r \pm \omega_{ni}|$ for the variable natural MF and with amplitudes $(1/2)S\omega_r B_{mk}$ and frequencies $|\omega_r \pm \omega_{mk}|$ for the variable man-made MF.

We consider now the second term in the right-hand side of (A.3.1) which described the induced Ψ in closed loops caused by the moving of the human body with velocity $\vec{V} = d\vec{r}/dt$ together with changing in α:

$$\Psi_{32} = -S\left|\vec{B}_{nc} + \vec{B}_{nv}\right| \frac{\partial \cos\alpha}{\partial \vec{r}} \vec{V} - S\left|\vec{B}_{mc} + \vec{B}_{mv}\right| \frac{\partial \cos\alpha}{\partial \vec{r}} \vec{V} . \qquad \text{(A.3.6)}$$

The first term describes the role of about constant and variable MF of natural origin, whose gradient on the Earth's surface is very small. This term is expected to be negligible ($\Psi_{32n} \approx 0$) even for great velocities of body movements (for instance by plane or by spacecraft). The second term shows the role of about constant and variable MF of man-made origin, that can be often characterized by rather big gradients. In such a case, if we can write:

$$\frac{\partial \cos\alpha}{\partial \vec{r}} \vec{V} = \sum_{j=1}^{x} A_{mcj} \cos(\omega_{mcj} t - \varphi_{mcj}) + \sum_{j=1}^{y} A_{mvj} \cos(\omega_{mvj} t - \varphi_{mvj}) \qquad \text{(A.3.7)}$$

then we obtain

$$\Psi_{32mc} = -SB_{mc}\sum_{j=1}^{x} A_{mcj}\cos\left(\omega_{mcj}t - \varphi_{mcj}\right), \tag{A.3.8}$$

$$\Psi_{32mv} = -S\sum_{k=1}^{p} B_{mk}\cos\left(\omega_{mk}t - \varphi_{mk}\right)\sum_{j=1}^{y} A_{mvj}\cos\left(\omega_{mvj}t - \varphi_{mvj}\right) =$$

$$-\left(1/2\right)S\sum_{k=1}^{p}\sum_{j=1}^{y} B_{mk}A_{mvj}\left\{\cos\left[\left(\omega_{mk} - \omega_{mvj}\right)t - \left(\varphi_{mk} + \varphi_{mvj}\right)\right] + \tag{A.3.9}$$

$$\cos\left[\left(\omega_{mk} - \omega_{mvj}\right)t - \left(\varphi_{mk} - \varphi_{mvj}\right)\right]\right\}$$

where for the variable part of man-made MF we took into account (A.1.3). Eq. (A.3.8) shows that, for a body moving in large MF gradient, the expected harmonics of Ψ for almost constant man-made MF, will have amplitudes $SB_{mc}A_{mcj}$ with frequencies ω_{mcj} and from variable man-made MF, according to (A.3.9), will have amplitudes $SB_{mk}A_{mvj}$ with frequencies $\left|\omega_{mvj} \pm \omega_{mk}\right|$.

A.4. On The Resonance Interaction of Self Electromotive Forces Ψ In Closed Loops With Ψ Induced By Mf Of Natural And Man-Made Origin

Let us suppose that in the closed loop S there are self electromotive forces Ψ as

$$\Psi_s = \Psi_{so}\sin\left(\omega_s t - \varphi_s\right), \tag{A.4.1}$$

Expression (A.4.1) is the solution of the equation:

$$\ddot{\Psi} = -\omega_s^2\Psi_s \tag{A.4.2}$$

with initial conditions $\Psi_s\left(=0\right) = -\Psi_{so}\sin\left(\varphi_s\right)$ $\dot{\Psi}_s = \Psi_{so}\cos\left(\varphi_s\right)$. Eq.(A.4.2) is analogous to the equation of self mechanical oscillations $F = -aX$, where $F = m\ddot{X}$, so that

$$\ddot{X} = -\omega_s^2 X, \tag{A.4.3}$$

where $\omega_s^2 = a/m$. Let us remember that if, in the presence of mechanical oscillations, there are external forces $F_e = -A_e\omega_e^2\sin\left(\omega_e t - \varphi_e\right)$, then Eq. (A.4.3) will be transformed in

$$\ddot{X} = -\omega_s^2 X - A_e \omega_e^2 \sin(\omega_e t - \varphi_e) \quad \text{(A.4.4)}$$

with solution for forced oscillations

$$X = A_e \omega_e^2 \sin(\omega_e t - \varphi_e) / \left|\omega_e^2 - \omega_s^2\right| . \quad \text{(A.4.5)}$$

If we take into account that each self frequency will have some half width Γ_s, the solution (A.4.5) will be transformed in

$$X = A_e \omega_e^2 \sin(\omega_e t - \varphi_e) / \left(\left|\omega_e^2 - \omega_s^2\right| + \Gamma_s^2\right) . \quad \text{(A.4.6)}$$

The resonance interaction will be important for all cases considered in previous sections. For example, let us consider the resonance interaction of self Ψ in closed loops with Ψ induced by the variable part of natural and man-made MF (see Section A.1). For this it is necessary to add to the right-hand side of Eq. (A.4.2) additional terms analogous to (A.4.4) on the basis of (A.1.2):

$$d^2\Psi_{11}/dt^2 = -\omega_s^2 \Psi_{11} - S\sum_{i=1}^{l} \omega_{ni}^3 B_{ni} \sin(\omega_{ni} t - \varphi_{ni}) \cos\alpha_{ni} +$$
$$-S\sum_{k=1}^{p} \omega_{mk}^3 B_{mk} \sin(\omega_{mk} t - \varphi_{mk}) \cos\alpha_{mk} . \quad \text{(A.4.7)}$$

The solution of (A.4.7), by taking into account (A.4.6), will be

$$\Psi_{11} = S\sum_{i=1}^{l} \omega_{ni}^3 \frac{B_{ni} \sin(\omega_{ni} t - \varphi_{ni}) \cos\alpha_{ni}}{\left|\omega_{ni}^2 - \omega_s^2\right| + \Gamma_s^2} +$$
$$S\sum_{k=1}^{p} \omega_{mk}^3 \frac{B_{mk} \sin(\omega_{mk} t - \varphi_{mk}) \cos\alpha_{mk}}{\left|\omega_{mk}^2 - \omega_s^2\right| + \Gamma_s^2} . \quad \text{(A.4.8)}$$

The solution (A.4.8) differs from (A.1.2) only in the frequency region near the self frequency ω_s, for which the background spectrum of induced Ψ will increase by a factor $\approx \omega_s^2 / \Gamma_s^2$, due to the resonance effect. For example, if $\Gamma_s \approx 0.1\omega_s$, then the increase will be by about 100 times; it means that the induced Ψ at this frequency will be amplified by about 100 times by the resonance effect. If some closed loops have several self frequencies ω_{s1}, ω_{s2},... ω_{sn} with half widths Γ_{s1}, Γ_{s2},... Γ_{sn}, then there will be several resonance increases in electromotive forces Ψ, by $\omega_{s1}^2/\Gamma_{s1}^2$, $\omega_{s2}^2/\Gamma_{s2}^2$,... $\omega_{sn}^2/\Gamma_{sn}^2$ times,

induced by the variable parts of natural and man-made MF at self frequencies ω_{s1}, ω_{s2},... ω_{sn} respectively.

ACKNOWLEDGMENTS

This research was partly supported by the European Commission (contract IC15-CT96-0303).

REFERENCES

[1] Foster K.R.1992. Health effects of low-level electromagnetic fields: phantom or not so phantom risk? *Health Phys*. *62*, 429-435.

[2] Baris D., Armstrong B., Deadman J. 1996. A mortality study of electrical utility workers in Quebec. *Occup Environ Med. 53*, 25-31.

[3] *NIEHS Working Group Report, Assessment of Health Effects from Exposure to Power-Line Frequency Electric and Magnetic Fields*; Editors, C.J. Portier, M.S. Wolfe; Research Triangle Park, NC 27709, *NIH Publ.* No. 98-3981, 1998.

[4] Van Wijngaarden E., Savitz D.A., Cai J., Loomis D., Kleckner R.C. 2000. Evidence of association between EMFs and exposed worker suicide. University of North Carolina News Release No. 147. http://www.unc.edu/news /newsserv /archives /mar00/savitz2031500.htm.

[5] Savitz D. A., Loomis D. P. 1995. Magnetic field exposure in relation to leukemia and brain mortality among electric utility workers. *Am J Epidemiol. 141*, 123-134.

[6] Kavet R.I. 1996. EMF and current cancer concepts. *Bioelectromagnetics. 17*, 339-357.

[7] Alfredsson L., Hammar N., Karlehagen S. 1996. Cancer incidence among male railway engine-drivers and conductors in Sweden. *Cancer Causes Control. 7*, 377-381.

[8] Minder C.E., Pfluger D.H. 2001. Leukemia, brain tumors and exposure to ELF electromagnetic fields in Swiss railway employees. *Am J Epidemiol. 153*, 825-835.

[9] Floderus B., Tornqvist S., Stenlund C. 1994. Incidence of selected cancers in Swedish railway workers. *Cancer Causes Control*. *5*, 189-194.

[10] Pfluger D., Villoresi G., Ptitsyna N., Temuriantz N., Martinyuk V., Kopytenko Y., Rasson J., Iucci N. 2000. Increased mortality for myocardial infarction in Swiss railway workers, *Proc Millenium Workshop on Biological Effects of Electromagnetic Fields*. Crete, Greece. 558-566.

[11] Ptitsyna N.G., Villoresi G., Kopytenko Y.A., Kudrin V.A., Tyasto M.I., Kopytenko E.A., Iucci N., Voronov P.M., Zaitsev D.B. 1996. Coronary heart diseases: an assesment of risk associated with work exposure to ultra-low frequency magnetic fields. *Bioelectromagnetic.17*, 436-444.

[12] Ptitsyna N.G., Y.A. Kopytenko, G. Villoresi, D.H. Pfluger, V. Ismaguilov, N. Iucci, E.A. Kopytenko, D.B. Zaitzev, P.M. Voronov, M.I Tyasto. 2003. Waveform Magnetic Field Survey in Russian DC- and Swiss AC-powered Trains: a Basis for Biologically Relevant Exposure Assessment. *Bioelectromagnetics*. *24*, 546-556.

[13]Ptitsyna N.G., Villoresi G., Kopytenko Y.A., Tyasto M.I., Kopytenko E.A., Iucci N., Voronov P.M., Zaizev D.M. Magnetic field monitoring on DC electrified transport in Russia. In *Electricity and Magnetism in Biology and Medicine.* Ed. F. Bersani, Plenum Publ. Corporation, NY, 1999, 773-776.

[14]Villoresi G., Kopytenko Y., Pfluger D., Ismagyilov V., Kopytenko E., Zaitsev D., Voronov P., Ptitsyna N., Tyasto M., Iucci N., Temuriantz N.,.Martinyuk V., Rasson J. 2000. Magnetic fields generated by electric trains. *Proc Millennium International Workshop on Biological Effects of Electromagnetic Fields*. Crete, Greece. 95-105.

[15]Ptitsyna N., Villoresi G., Kopytenko Y., Pfluger D., Ismagyilov V., Iucci N., Tyasto M., Kopytenko E., Temuriantz N., Martinyuk V. 2000. Assessment of biologically plausible exposure parameters for complex-spectra transport magnetic fields. *Proc Millennium International Workshop on Biological Effects of Electromagnetic Fields.* Crete, Greece. 109-114.

[16]Tynes T., Anderson L. 1990. Electromagnetic fields and male breast cancer. *Lancet. 336*, 8730-8737.

[17]Balli-Antunes M., Pfluger D.H., Minder C.E. 1990. The mortality from malignancies of haematopoietic and lymphatic systems among railway engine drivers *Environmetrics. 1*, 121-130.

[18]Minder Ch.E., Pfluger D.H. 1993. Extremely low frequency electromagnetic field measurements (ELF-EMF) in Swiss railway engines. *Radiation Protection Dosimetry. 48*, 351-354.

[19]Savitz D.A., Liao D., Sastre A., Kleckner R.C., Kavet R. 1999. Magnetic Field Exposure and Cardiovascular Disease Mortality among Electric Utility Workers. *Am J Epidemiol. 149*, 135-142.

[20]Villoresi G., Ptitsyna N.G., Kudrin V.A., Iucci N., Health effect among engine drivers: association with occupational exposure to magnetic fields from DC electrified transport. In *Electricity and Magnetism in Biology and Medicine*. Ed. F. Bersani. Plenum Publ. Corporation, NY, 1999, 777-780.

[21]Whittington C.J., Podd J.V., Rapley B.R. 1996. Acute effects of 50 Hz magnetic field exposure on human visual task and cardiovascular performance. *Bioelectromagnetics. 17*, 131-137.

[22]Graham C., Cohen H., Cook M., Phelps J., Cercovich M., Fotopoulos S. A double blind evaluation of 60-Hz effects on human performance, physiology and subjective state. In *Interaction of Biological Systems with Static and ELF Electric and Magnetic Field.* Eds. L. Anderson, R. Weigel, B. Kelman., NTIS, 23rd Annual Hanford Life Sciences Symp, 1987, Springfield, VA, 471-486.

[23]Pfluger D.H., Kopytenko Y.A., Villoresi G., Ptitsyna N.G. 1999. Mortality from myocardial attacks in Swiss Federal Railways employees. In *Proc 2nd Intern Conf Electromagnetic Fields and Human Health*. Moscow, Russia, 312-313.

[24]Nishida A. *Geomagnetic Diagnosis of the Magnetospher;* Springer Verlag: NY, Heidelberg, Berlin, 1978.

[25]Bennett W.R. Jr. 1994. Cancer and power lines. *Phys Today*. *47*, 23-29.

[26]Bracken T.D. 1993. Exposure assessment for power frequency electric and magnetic fields. *Am Ind Hyg Assoc J. 54*, 165-177.

[27]Kavet R.I., Banks R.S. 1986. Emerging issues in extremely low frequency electric and magnetic field health research. *Environ Res. 39*, 386-404.

[28]Ho A.M., Fraser-Smith A.C., Villard Jr. O. 1979. Large-amplitude ULF magnetic fields produced by a rapid transit system: close-range measurements. *Radio Sci. 14*, 1011-1015.

[29]Kazuo U. 1977. Magnetic field disturbances produced by electric railway, in *Memories of the Kakioka Magnetic Field Observatory (Suppl) 7*, 17-34.

[30]Ptitsyna N.G., Villoresi G., Dorman L.I., Iucci N., Tyasto M.I. 1998. Natural and man-made low-frequency magnetic fields as a health hazard, *Physics Uspekhi* (Advances in Physics Sciences). *41*, 687-709.

[31]Ptitsyna N.G., Villoresi G., Kopytenko Y.A., Tyasto M.I., Kopytenko E.A., Iucci N., Voronov P.M., Zaizev D.M. Magnetic field environment in ULF range (0-10 Hz) in urban areas: man-made and natural fields. In *Electricity and Magnetism in Biology and Medicine*. Ed. F. Bersani. Plenum Publ. Corporation, NY, 1999. 279-282.

[32]Paul H.U., Hirsch F., Studinger H., Hadrian W. In *Interference of Monitors by Magnetic Fields of Power Frequency Installations and Traction Lines.* 1990. CIGRE. Paris, France, 36-42.

[33]Allen S.G., Blackwell R.P., Chadwick P.J., Driscoll C.M.H., Pearson A.J., Unsworth C.C., Whillock M.J. *Review of Occupational Exposure to Optical Radiation and Electric and Magnetic Fields with Regard to the Proposed CEC Physical Agents Directive*. National Radiological Protection Board. Chilton, Didcot, Oxon, 1988, OX11 ORQ.

[34]Nakagava M., Koana T., EMF issues with Maglev in Japan. In *Electricity and Magnetism in Biology and Medicine*. Ed. M. Blank. Press Inc. San Francisco, Ca.1993, 264-266.

[35]Dietrich F.M., Feero W.E., Papas P.N., Steiner G.A. *Magnetic and Electric Field Testing of the Amtrak Northeast Corridor and New Jersey Transit/North Jersey Coast Line Rail Systes*. US Department of Transportation, DOT-VNTSC-FRA-93-4.1, 1993.

[36] Wilson B.W., Reiter R.J., Pilla A.A. Review of EMF bioeffects Literature Relative to Maglew-generated magnetic Fields. In *Electricity and Magnetism in Biology and Medicine*. Ed. M. Blank. Press Inc. San Francisco, CA.1993, 251-254.

[37] Dietrich F.M., Steiner G.A., Robertson D.C., Feero W.E. Magnetic field testing of TR07 Maglev system. In *Electricity and Magnetism in Biology and Medicine*. Ed. M. Blank. Press Inc. San Francisco, CA.1993, 267-270**.**

[38] Kopytenko Y.A., Kopytenko E.A., Amosov L.G., Zaitsev D.B., Voronov P.M., Timoshenkov Y.P. 1994. Magnetovariation complex MVC-2. *Proc VI Workshop on Geomagnetic Observatory Instruments, Data Acquisition and Processin.* Dourbes, Belgium, 24-31.

[39] Hilemann B. 1993. Health effects of electromagnetic fields remain unresolved. *Chem Eng News. 71,* 15-29.

[40] Neurath P.W. Simple theoretical models for magnetic intraction waith biological units. *In Biological effects of magnetic fields.* Ed M.F. Barnothy. Plenum Press, NY, 1, 1964, 25-32.

[41] Frankel R.B. Biological effects of static magnetic fields. In *CRC Handbook of biological effects of electromagnetaic fields.* Ed. Polk C., Postow E. Press. Inc. Boca Raton, FL. 1986, 180-181.

[42] Kirshvink J. L., Kobayashi-Kirshvink A., Woodford B. 1992. Magnetite biomineralization in the human brain. *Proc Nat Acad Sci USA. 89,* 7683-7698.

[43] Kirschvink J. L. Rock magnetism linked to human brain magnetite. 1994. *EOS Trans. Amer. Geophys Union. 75*(15), 178-182.

[44] Beason R. C., Nichols J. E. 1984. Magnetic orientation and magnetically sensitive material in a transequatorial migratory bird. *Nature 309* 151-153.

[45] Towne W. F., Gould J. L. Magnetic field sensitivity in honebees. In *Magnetic Biomineralization and Magnetoreception in Organisms.* Eds J. L. Kirschvink, D. S. Jones, B. J. MacFadden. Plenum Press. NY, 1985, 385-406.

[46] Korchevsky E M., Marochnik L. S. 1965. Magnetohydrodynamic version of movement of blood. *Biofizika 10* 371-374 (Engl. transl: Biophysics 10, 411-414).

[47] Morgan M.G., Nair I. 1992. Alternative functional relationships between ELF field exposure and possible health effects: report on an expert workshop. *Bioelectromagnetics 13*, 335-350.

[48] Cleary S.F.A. 1993. Review of in Vitro Studies: Low-frequency Electromagnetic fields. *Am Ind Hyg Assoc J. 54,* 178-185.

[49] Leal J., Shamsaifar K., Trillo M.A., Ubeda A. 1989. Embryonic development and weak changes of the geomagnetic field. *Boielectricity*. *7*, 141-152.

[50] Kavaliers M., Eckel L.A., Ossenkopp K.P. 1993. Brief exposure to 60 Hz magnetic fields improves sexually dimorphic spatial learning in the meadow vole, Microtus-pennsylvanicus. *J Comp Physiol A. 173,* 241-248.

[51] Lyskov E.B., Juutilainen J., Jousmaki V., Partanen J., Medvedev S., Hanninen O. 1993. Effects of EMF on the human brain activity. *Bioelectromagnetics. 14,* 87-95.

[52] Kavet R.I. A brief perspective on biological effects from intermittent EMF exposure. In *Future Epidemiologic Studies of Health Effects of Electric and Magnetic Fields.* Electric Power Research Institute EPRI report, TR-101175. Palo Alto, CA, 1992, A47-A53.

[53] Sait M.L., Wood A.W., Sadafi H.A. 1999. A study of heart rate and heart rate variability in human subjects exposed to occupational levels of 50 Hz circularly polarized magnetic fields. *Med Eng Phys. 21* (5), 361-369.

[54] SastreA., Cook M.R., Graham C., 1998. Nocturnal exposure to intermittent 60 Hz magnetic fields alters human cardiac rhythm. *Bioelectromagnetics. 19 (2)*, 98-106.

[55] Gavalas-Medici R., Day-Magdaleno S. 1976. ELF weak electric field effects scheduled-controlled behavior of monkeys. *Nature. 261*, 256-259.

[56] Makeev V.B., Temuryantz N.A. 1982. Research of frequency dependence on biological activity of magnetic field in geomagnetic pulsations range 0.01-100 Hz In *Problemi kosmicheskoy biologii [Problems of Cosmobiology]* Nauka, Moscow, Russia, *43*, 116-129.

[57] Maresh C.M., Cook M.R., Cohen H.D., Graham C. 1988. Exercise in the evaluation of human responses to powerline frequency fields. *Aviat Space Envirot Med. 59,* 1139-1145.

[58] Temuriantz N.A., Martinyuk V.S., Ptitsyna N.G., Villoresi G., Iucci N., Tyasto M.I., Dorman L.I. 2007. Complex-spectrum magnetic environment enhances and/or modifies bioeffects of hypokinetic stress condition. An animal study. *Adv Space Res. 40*(11), 1758-1763.

Reviewed by Dr. Kassinsky V.V., Institute of Railway Transportation Engineering, Irkutsk, Russia.

View publication stats
