# Supplementary material for: Magnetic Fields and Cancer: Epidemiology, Cellular Biology, and Theranostics
Source: Int J Mol Sci. 2022 Jan 25;23(3):1339. doi: 10.3390/ijms23031339 (PMC8835851; doi:10.3390/ijms23031339)
Supplement: Supplementary file 1 [file ijms-23-01339-s001.zip › Supplementary Data Set S1/MF and Cancer.Data/PDF/0140906127/1-s2.0-S0160412015301148-main.pdf]

Environment International 88 (2016) 36–43

Contents lists available at ScienceDirect

Environment International

journal homepage: www.elsevier.com/locate/envint

Meta-analysis of extremely low frequency electromagnetic fields and cancer risk: a pooled analysis of epidemiologic studies☆

Yemao Zhang [a], Jinsheng Lai [b], Guoran Ruan [b], Chen Chen [b,*], Dao Wen Wang [b]

[a] High Voltage Research Institute, China Electric Power Research Institute, Wuhan, People's Republic of China
[b] Department of Internal Medicine and the Institute of Hypertension, Tongji Hospital, Tongji Medical College of Huazhong University of Science and Technology, Wuhan, People's Republic of China

ARTICLE INFO

Article history:
Received 24 September 2015
Received in revised form 23 November 2015
Accepted 10 December 2015
Available online 15 December 2015

Keywords:
ELF-EMF
Cancer risk
Meta-analysis

ABSTRACT

Studies have suggested that extremely low frequency electromagnetic fields (ELF-EMF) may affect physiological functions in animal models. However, epidemiologic studies investigating the association of ELF-EMF with the susceptibility to cancer yield contradictory results. In this comprehensive analysis, we conducted a search for case–control surveys regarding the associations of ELF-EMF and cancer susceptibility in electronic databases. A total of 42 studies involving 13,259 cases and 100,882 controls were retrieved. Overall, increased susceptibility to cancer was identified in the ELF-EMF exposed population (OR = 1.08, 95% CI: 1.01, 1.15, $P = 0.02$). In the stratified analyses, increased risk was found in North America (OR = 1.10; 95% CI: 1.02, 1.20, $P = 0.02$), especially the United States (OR = 1.10; 95% CI: 1.01, 1.20, $P = 0.03$). However, studies from Europe contradict these results. Moreover, a higher risk was found to be statistically significantly associated with the residential exposed population (OR = 1.18; 95% CI: 1.02, 1.37, $P = 0.03$). Furthermore, an increased cancer risk was found in interview-based surveys (OR = 1.16; 95% CI: 1.00, 1.35, $P = 0.04$). In device measurement-based studies, a slight increased risk was found only in premenopausal breast cancer (OR = 1.23; 95% CI: 1.01, 1.49, $P = 0.04$). Our meta-analysis suggests that ELF-EMFs are associated with cancer risk, mainly in the United States and in residential exposed populations. Methodological challenges might explain the differences among studies.
© 2015 Elsevier Ltd. All rights reserved.

1. Introduction

The acceleration of high-voltage power line construction has increased the probability of human exposure to electric fields (Humans, 2002). Objects such as trees and other electrically grounded objects have a screening effect and generally reduce the strength of the electric fields in their vicinity. Buildings attenuate electric fields considerably, and the electric field strength may be one to three orders of magnitude less inside a building than outside of it. Electric fields to which people are exposed inside buildings are generally produced by internal wiring and appliances and not by external sources ((NRPB), 2001). The average magnetic flux density measured directly beneath overhead power lines can reach 30 μT for 765-kV lines and 10 μT for the more common 380-kV lines (Repacholi and Greenebaum, 1999). The overall evaluation conducted by the World Health Organization (WHO) indicated that extremely low-frequency magnetic fields (ELF-EMFs) are possibly carcinogenic to humans (Group 2B), while static electric and magnetic fields and extremely low-frequency electric fields are not classifiable as to their carcinogenicity to humans (Group 3)(Humans, 2002).

Human-made electromagnetic fields (EMFs), such as nonionizing radiation, are classified into three categories: extremely low frequency fields (<300 Hz), high frequency fields in the band of radio frequency fields (300 Hz to 3×10^8 Hz), and microwaves (3×10^8 Hz to 3×10^{11} Hz) (Humans, 2002; Lee et al., 2014). The direct biological effects of an EMF include thermal effects by EMF energy absorption, stimulation function by induced electric current, and athermic action by long-term exposure (Foster and Glaser, 2007). Different frequencies of EMFs may result in various biological effects. For example, thermal biological effects are mainly induced by radio frequencies. It is generally believed that the energy generated by extremely low frequency electromagnetic fields (ELF-EMFs) is too weak to directly damage DNA.

ELF-EMFs are mainly generated by power transmission lines, power equipment or appliances (Chen et al., 2013). Because of the rapid development of industry and society, humans are surrounded by various electric devices, and exposure to ELF-EMFs is increasing. Currently, the

Abbreviations: CI, confidence interval; OR, odds ratio; ELF-EMF, extremely low frequency electromagnetic field; EMF, electromagnetic field; RR, relative risk.

☆ Novelty and impact: Increased susceptibility to cancer was identified in total ELF-EMF exposed population. However, in stratified analyses, increased risk was found in North America, especially in the United States, while studies from Europe contradict these results. Furthermore, increased cancer risk was only found in interview-based surveys but not in device measurement-based studies. Our meta-analysis suggests that ELF-EMFs are associated with the risk of cancer, mainly in the United States and in residential exposed populations.

* Corresponding author at: Department of Internal Medicine, Tongji Hospital, Tongji Medical College, Huazhong University of Science and Technology, 1095# Jiefang Ave., Wuhan 430030, People's Republic of China.
E-mail address: chenchen@tjh.tjmu.edu.cn (C. Chen).

http://dx.doi.org/10.1016/j.envint.2015.12.012
0160-4120/© 2015 Elsevier Ltd. All rights reserved.

biological effects induced by ELF-EMFs on human health have become a cause for concern (Grellier et al., 2014; Zhang et al., 2015).

Laboratory studies on the biological effects of ELF-EMFs mainly focus on stem cells, tumor cells and different animal models. In mouse embryonic stem cells, no effects of short-time ELF-EMF exposure on mitochondrial function, nuclear apoptosis, cell proliferation, and chromosomal alterations were observed (Nikolova et al., 2005). On the contrary, 1 mT ELF-EMF exposure may induce impairment of recognition memory, resulting in changes in hippocampal dendritic spine density (Zhao et al., 2015). Moreover, 1 mT ELF-EMF exposure sensitized SH-SY5Y cells to the pro-Parkinson's disease toxin (Benassi et al., 2015). However, an even higher intensity of ELF-EMF (2 mT) did not affect tumor promotion in the skin of SENCAR mice (DiGiovanni et al., 1999).

Some scientists are interested in the therapeutic possibilities of ELF-EMF, such as wound healing (Funk et al., 2009). Studies have found three main effects of ELF-EMF on wound healing pathways: 1) an anti-inflammatory effect by the modulation of the cytokine profile that induces the transition of the healing process from a chronic pro-inflammatory to an anti-inflammatory state; 2) a neo-angiogenic effect by increasing endothelial cell proliferation and tubulization as well as the production of fibroblast growth factor (FGF)-2; and 3) a re-epithelialization effect by the stimulation of collagen formation (Costin et al., 2012).

However, more direct evidence about the biological effects of ELF-EMFs come from human observations, such as epidemiological investigations (Heath, 1991). As early as 1979, the first article about the association between increased risk of leukemia and ELF-EMF exposure was reported by Wertheimer and Leeper(Wertheimer and Leeper, 1979). Later, in 1987, the absence of increased risk for all leukemia or acute leukemia among ELF-EMF exposed welders was observed in Denmark [17]. Since then, reports on the associations between ELF-EMF exposure and human diseases, including behavioral alterations, neurodegenerative diseases and cancer, have provided abundant insight into the biological effects of ELF-EMF exposure in humans. Although the results regarding the adverse effects of ELF-EMF on the physiological/pathophysiological functions in humans or animals are inconclusive, the International Agency for Research on Cancer (IARC) has classified ELF-EMFs as “possibly carcinogenic” in 2002 (Humans, 2002).

To date, many studies explored the association between ELF-EMFs and the susceptibility to different cancers, mainly leukemia, breast cancer and brain cancer. No higher risk of leukemia for children living 0–199 m or for children living 200–599 m from a 132–400 kV overhead power line was observed in Denmark (Pedersen et al., 2014). Furthermore, Toledano et al. revealed that no evidence supports the epidemiologic association of adult cancers with residential magnetic fields in proximity to high-voltage overhead power lines (Elliott et al., 2013). However, three pooled analyses of case–control studies showed a 1.4- to 1.7-fold increase in childhood leukemia risk for ELF-EMF exposure levels above 0.3 μT (Teepen and van Dijck, 2012). Recently, a systematic analysis suggested that ELF-EMFs might be related to an increased risk of female breast cancer, especially for premenopausal and estrogen-positive females (Chen et al., 2013), while another meta-analysis suggested that ELF-EMF exposure had no association with the susceptibility to female breast cancer (Chen et al., 2010).

The results of these observations remain controversial and inconclusive. In general, animal experiments have produced positive results for all known human carcinogens, for which adequate testing has been performed (Humans, 2002). However, it is notable that childhood leukemia is the only cancer outcome for which this association has been consistently found using epidemiological methods (Grellier et al., 2014). It has been hypothesized that experiments designed following the classical two-step initiator–promoter concept of carcinogenesis may not be appropriate for understanding the biological effects of ELF-EMFs, as disease progression may result from complex interactions of genotoxic and non-genotoxic carcinogens (Juutilainen, 2008).

In the present study, we performed a meta-analysis to obtain more precise and comprehensive estimations of the associations between ELF-EMF exposure and susceptibility to cancers to estimate potential cross-study heterogeneity.

2. Material and methods

2.1. Study selection

We performed publication searches in PubMed, EMBASE, ISI Web of Science, The Cochrane Library, ScienceDirect, EBSCO, Ovid, Wiley Online Library, and HighWire databases with the following search terms: (extremely low frequency electromagnetic fields OR ELF-EMF) AND (cancer), by two independent investigators (Yemao Zhang and Jinsheng Lai, last search update: July 20, 2015). Hand searches were also conducted to identify additional articles in the reference lists of included articles not retrieved by the initial electronic search. The publication language was confined to English. Publication data were not restricted in our search. All studies matching the inclusion criteria were retrieved for further examination and data extraction. All of the investigators have received training in literature search, statistics and evidence-based medicine.

2.2. Inclusion and exclusion criteria

Studies included in the current meta-analysis had to meet all the following criteria: (1) evaluated the associations between ELF-EMF and cancer risk; (2) studied humans; (3) confirmed the disease by histology, imaging, pathology or a tumor registry; (4) had a case–control design; (5) provided detailed data for the calculation of odds ratio (OR) and 95% confidence interval (CIs); (6) included the latest study if serial studies of the same population from the same group were reported; (7) had no time-period limitation on the publications. Studies were excluded when they represented duplicates of previous publications or were meta-analyses, meeting abstracts, letters, reviews, or editorial articles. Studies on parental occupational exposure to extremely low frequency magnetic fields and cancer in the offspring were also excluded. Considering that some studies are more accurate and reliable, a quality assessment method for case and control studies has been developed based on the Newcastle-Ottawa Scale (Stang, 2010). All studies were assessed on three aspects, that including ten indicators for choosing the methods for cases and controls, the comparability of cases and controls and the exposure assessment.

2.3. Data extraction

All data from the included studies were independently extracted by two investigators (Yemao Zhang and Jinsheng Lai) using a standard protocol and a data-collection form according to the inclusion criteria listed above. A consensus was reached on all items. Recorded characteristics extracted from the eligible studies included the first author's name, year of publication, country of origin, study period, cancer type, characteristics of the study subjects (sample size, sampling methods, exposure methods, exposure level and type of measurement), and confounding factors that were controlled for by matching or adjustment. Studies involving multiple diseases were separated by disease. The study locations were categorized as Asia, Europe or North America. Disagreements were resolved by discussion. Senior investigators (Chen Chen and Dao Wen Wang) were invited to the discussion if disagreements still existed.

2.4. Statistical analysis

Relative risk (RR) was used as a measure of the relationship between ELF-EMF and the risk of cancer. For case–control studies, the relative odds were used as a surrogate measure of the corresponding relative

risk. Because the absolute risk of cancer is low, the relative odds approximate the relative risk.

OR corresponding to a 95% CI was used to assess the strength of association between ELF-EMF and the susceptibility to cancer. Subgroup analyses were performed by study location, cancer type and exposure mode. Statistical heterogeneity among the studies was estimated using chi-square-based Q-test, a P value greater than 0.1 indicates no statistically significant heterogeneity, and the pooled OR was estimated by the fixed-effects model (the Mantel–Haenszel method); otherwise, the random-effects model (the DerSimonian and Laird method) was employed (Wang et al., 2012). The potential for publication bias in the literature was evaluated by funnel plots and the linear regression asymmetry test by Egger et al. (Egger et al., 1997). An asymmetric plot suggests a possible publication bias, and a P value for Egger's test of less than 0.05 was considered to be representative of statistically significant publication bias. To estimate the robustness of our findings with respect to different assumptions, we conducted a sensitivity analysis by deleting one study at a time to examine the influence of individual data sets on the pooled ORs. Analyses were performed using Stata by means of the metainf program.

All statistical tests were performed with Review Manage (v.4.2; Oxford, England) and Stata software, version 11.0 (Stata Corporation, College Station, TX, US). All P values are two-sided.

3. Results

3.1. Study characteristics

After the first search in PubMed, EMBASE, ISI Web of Science, The Cochrane Library, ScienceDirect, EBSCO, Ovid, Wiley Online Library, and HighWire databases, 264, 1077, 348, 4, 3811, 154, 206, 2639, and 55 articles, respectively, were retrieved. Of these, 62 articles were relevant to the search words (Fig. 1). After further manual searches of reference lists, 28 more studies were included. Then, 48 studies were excluded (16 review/meta-analysis, 16 without detailed data and 16 non-case–control studies), and finally, 42 studies met the inclusion criteria and were subjected to further examination. The qualities of the studies were measured by the Newcastle-Ottawa Scale, and all selected studies were of high quality (score > 6). The characteristics of the included studies are summarized in Supplemental Table 1, including country, study period, exposure methods/strength/detection method and cancer type. All studies were case–control studies, including 23 breast cancer (Davis et al., 2002; Forssen et al., 2005; Gammon et al., 1998; Kabat et al., 2003; Kliukiene et al., 2003,2004; Labreche et al., 2003; London et al., 2003; Loomis et al., 1994; McElroy et al., 2001, 2007; Schoenfeld et al., 2003; Stenlund and Floderus, 1997; Tynes et al., 1996; Van Wijngaarden et al., 2001; Vena et al., 1994; Vena et al., 1991; Zheng et al., 2000; Zhu et al., 2003), 12 brain tumors (Baldi et al., 2011; Coble et al., 2009; Feychting and Ahlbom, 1993; Forssen et al., 2000; Grayson, 1996; Kroll et al., 2010; Li et al., 1997; Olsen et al., 1993; Parodi et al., 2014; Preston-Martin et al., 1996; Saito et al., 2010; Tynes and Haldorsen, 1997), 12 leukemia (Bethwaite et al., 2001; Feizi and Arabi, 2007; Feychting and Ahlbom, 1993; Investigators, 1999; Kroll et al., 2010; Li et al., 1997; Michaelis et al., 1997; Olsen et al., 1993; Salvan et al., 2015; Sermage-Faure et al., 2013; Wunsch-Filho et al., 2011), 1 testicular cancer (Stenlund and Floderus, 1997), 1 acoustic neuroma (Forssen et al., 2006), 1 endometrial cancer (Zhu et al., 2003) and 3 other cancer cases. Cancers were histologically or pathologically diagnosed in all studies. There were 3 studies from Asia (1 Iran, 1 Japan and 1 China), 18 from Europe (2 England, 2 France, 2 Italy, 1 German, 4 Norway, 6 Sweden and 1 Denmark), 20 from North America (18 United States, 1 Canada and 1 Brazil), and 1 from Oceania (New Zealand). Several exposure methods were employed in the studies, including 15 occupational exposures, 14 residential exposures, 6 home exposures, and 9 electric blanket exposures. Specifically, 3 types of measuring types were used: 16 by device measurements, 17 by program calculation, and 11 by interview about patterns of usage or exposure probability.

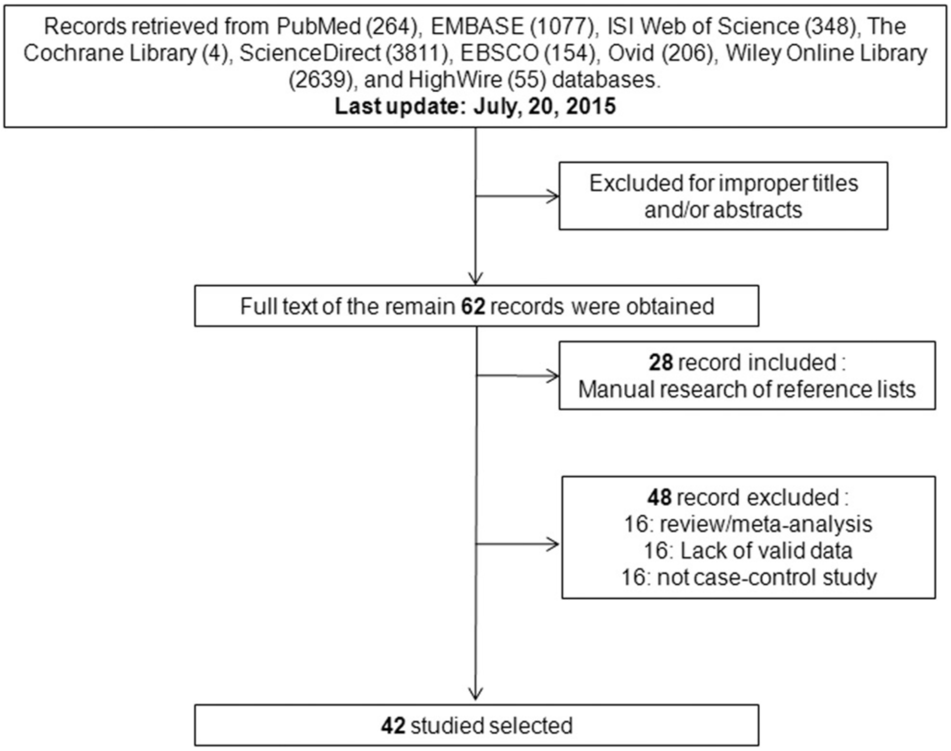

Fig. 1. Flow diagram of the study identification.

Table 1
Meta-analysis for ELF-EMF exposure and cancer risk.

Exposure model	Number of study	Pooled OR	95% CI	P h[a]	P [b]
Total	42	1.08	1.01, 1.15	<0.00001	0.02
Country					
North America	20	1.10	1.02, 1.20	<0.00001	0.02
United States	18	1.10	1.01, 1.20	<0.00001	0.03
Others	2	1.21	0.94, 1.57	0.85	0.14
Europe	18	1.01	0.90, 1.12	0.001	0.91
Sweden	6	0.90	0.84, 0.96	0.54	0.001
Norway	4	1.11	1.02, 1.21	0.80	0.02
England	2	0.82	0.69, 0.96	0.60	0.02
France	2	1.38	1.03, 1.84	0.31	0.03
Italy	2	0.82	0.51, 1.32	0.56	0.42
others	2	1.76	1.14, 2.72	0.36	0.01
Asia	3	1.43	0.81, 2.55	0.13	0.22
Others	1	1.97	1.08, 3.59	N/A	0.03
Cancer type[c]					
Breast cancer	23[d]	1.07	1.00, 1.15	<0.00001	0.06
Postmenopausal	4	1.00	0.88, 1.14	0.43	0.97
Premenopausal	3	1.57	0.95, 2.59	0.0002	0.08
Mixed	18	1.03	0.97, 1.10	0.04	0.30
Brain cancer	12	1.10	0.96, 1.26	0.20	0.16
Childhood	4	0.96	0.64, 1.44	0.37	0.84
Mixed	8	1.12	0.97, 1.29	0.14	0.12
Leukemia	12	1.18	0.89, 1.56	0.001	0.24
Childhood	6	1.09	0.71, 1.68	0.01	0.69
Mixed	6	1.32	0.97, 1.79	0.17	0.08
Others	6	1.01	0.84, 1.22	0.02	0.89
Exposure methods[e]					
Occupational exposures	15	1.06	0.97, 1.16	0.0004	0.22
Residential exposures	14	1.18	1.02, 1.37	0.06	0.03
Electric blanket exposures	9	1.13	0.98, 1.30	<0.00001	0.10
House exposures	6	0.98	0.77, 1.26	0.10	0.91
Measuring type					
Program calculation	16	1.07	0.97, 1.18	<0.00001	0.16
Device measurements	16	1.03[f]	0.92, 1.15[f]	0.17	0.62[f]
Interview	10	1.16	1.00, 1.35	<0.00001	0.04

[a] *P* h value of Q-test for heterogeneity test. Random-effects model was used when *P* value for heterogeneity test <0.1; otherwise, fixed-effects model was used.
[b] *P* value for significance.
[c] Some of the studies included several cancer types.
[d] Two of the 23 studies about breast cancer included both postmenopausal and premenopausal patients. The results were calculated by different degree of freedom.
[e] Some of the studies included several exposure methods.
[f] This subgroup was analyzed by random-effects model.

3.2. Meta-analysis results

The association between EMF-ELFs and the susceptibility to cancer was analyzed in 42 independent studies. Because different detecting methods may result in different data, we first used the exposure strength threshold from each study to generate the total exposure/non-exposure effects. The results in Fig. 2 show that weak association between EMF-ELF exposure and susceptibility to cancer was identified when all the eligible studies were pooled (OR = 1.08, 95% CI: 1.01, 1.15, *P* = 0.02) regardless of the exposure models or cancer types (Fig. 2).

Next, subgroup analyses were performed (Table 1). Twenty, eighteen and three out of the forty-two included studies were conducted in North American, European and Asian populations, respectively. In the country subgroup analysis, a statistically significant increase in risk was found in North America (15 breast cancer, 3 brain cancer, 1 leukemia and 1 other cancer; OR = 1.10, 95% CI: 1.02, 1.20, *P* = 0.02), mainly in the United States (14 breast cancer, 3 brain cancer and 1 other cancer; OR = 1.10, 95% CI: 1.01, 1.20, *P* = 0.03). On the contrary, no statistically significant association between EMF-ELFs and cancer risk was found in a global analysis of European studies (7 breast cancer, 7 brain cancer, 8 leukemia and 5 other cancers). However, when classified according to their countries, we obtained conflicting data. An increased risk of cancer was found in Norway (3 breast cancer, 1 brain cancer, 1 leukemia and 1 other cancer; OR = 1.11, 95% CI: 1.02, 1.21, *P* = 0.02) and France (1 brain cancer and 1 leukemia; OR = 1.38, 95% CI: 1.03, 1.84, *P* = 0.03), while a decreased risk was found in Sweden (4 breast cancer, 1 brain cancer, 1 leukemia and 1 other cancer; OR = 0.90, 95% CI: 0.84, 0.96, *P* = 0.001) and England (2 brain cancer, 2 leukemia and 2 other cancers; OR = 0.82, 95% CI: 0.69, 0.96, *P* = 0.02). In addition, a study from New Zealand also showed an increased cancer risk (1 leukemia; OR = 1.97, 95% CI: 1.08, 3.59, *P* = 0.03). Further subgroup analyses based on cancer type did not reveal any statistically significant associations in all of the analyzed types. When compared by exposure methods, an increased risk was only observed in residential exposure populations (OR = 1.18; 95% CI: 1.02, 1.37, *P* = 0.03). Considering the measurement type, an increased risk was only discovered in interview-based surveys.

Moreover, in view of the various exposure intensity thresholds in individual studies, the association between different EMF-ELF exposure models and the susceptibility to cancer was estimated in studies conducted by device measurements (Table 2). Only premenopausal breast cancer, but not all breast cancer types, was associated with EMF-ELF exposure.

3.3. Publication bias

Begg's funnel plot and Egger's test were performed to determine the publication bias of the included surveys. Symmetrical funnel plots were obtained and are shown in Fig. 3. Furthermore, Egger's test confirmed the absence of publication bias in all studies (*P* > 0.05).

3.4. Sensitivity analysis

The exclusion of one study at a time was performed to detect the influence of the individual data from each survey on the pooled ORs. The results showed that the pooled OR estimates were consistent with those of the excluded studies (Fig. 4).

4. Discussion

In the present study, ELF-EMF exposure and the susceptibility to cancer was evaluated in 13,259 cases and 100,882 controls. Overall, a statistically significant association between ELF-EMF exposure and cancer risk was identified. Subgroup analysis based on country revealed a statistically significant increase in cancer risk in North America, especially in the United States, while there was no statistically significant association in Europe. However, the data from individual European countries contradicted each other. Sub-analysis classified by cancer type did not reveal any association in all analyzed types. Moreover, increased risk was only observed in residential exposure populations or interview-based surveys when compared to exposure methods or measuring type, respectively. Further analyses in device-measured studies revealed no statistically significant association in total effects, but a mildly statistically significant increased risk was found in premenopausal breast cancer.

Electric power is an essential world commodity and is the key to the development of our technology-based society. In the modern world, the benefits obtained from the continuously progressing electric power industry are overwhelming, while public concerns are growing regarding exposure to ELF-EMFs associated with power distribution. Until now, numerous researchers around the world have examined the correlation between ELF-EMFs and different types of cancer. However, most epidemiological studies were conducted in developed countries, especially in the United States and Scandinavia. For a long time, developed countries were highly aware of environmental and labor protection, and this has motivated various organizations to monitor environmental exposure to ELF-EMFs. A European approach to the effects of extremely low-frequency electromagnetic fields on cancer by ELF-EMF was conducted

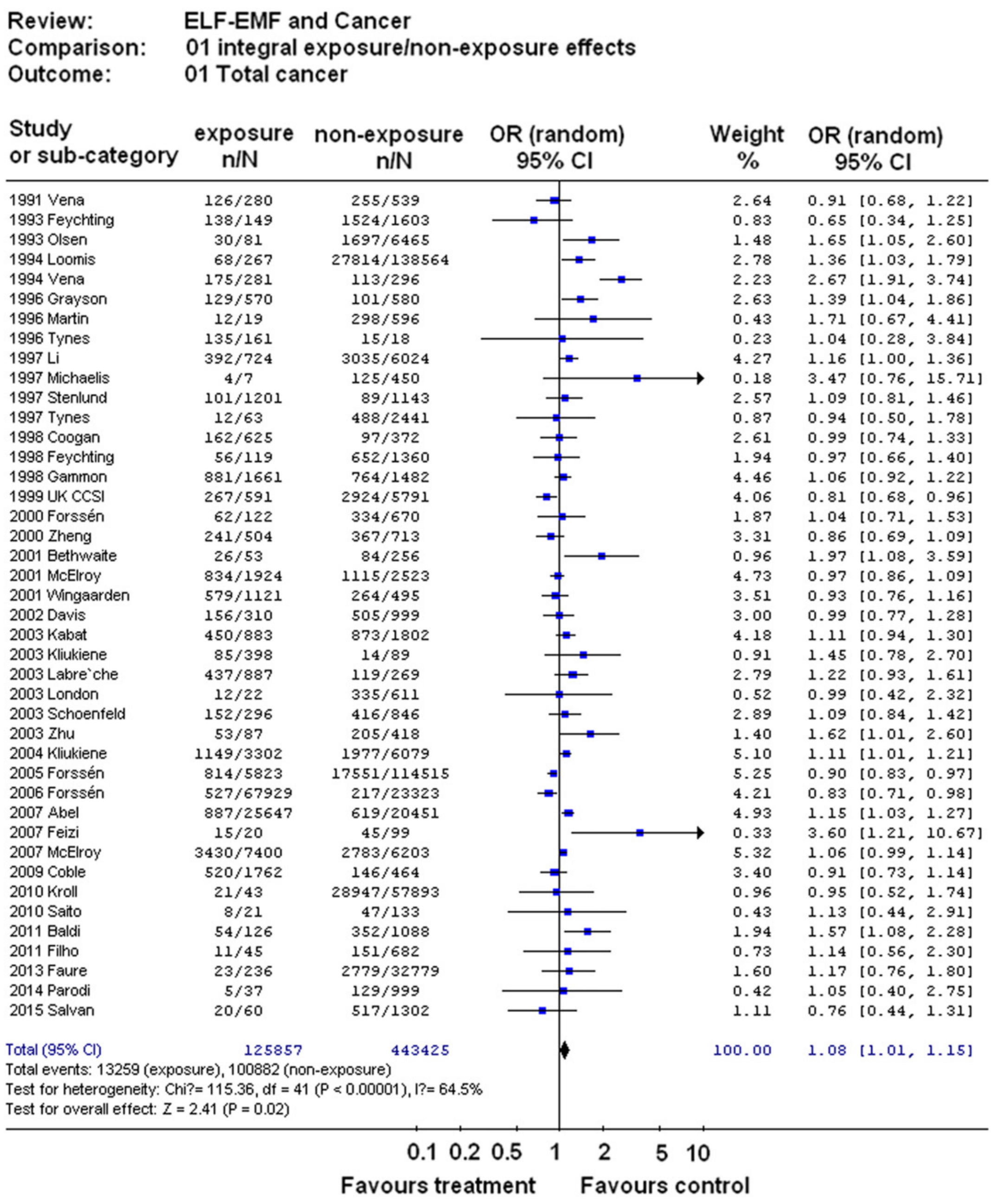

Fig. 2. Forest stereogram of the meta-analysis on the association between the exposure to ELF-EMFs and cancer.

by the European Feasibility Study Group in 1997 (Group, 1997). The environmental compositions and genetic backgrounds, which are also strongly associated with tumor susceptibility, vary among different ethnic groups. The initial observation of a higher response to gefitinib and erlotinib in patients of Asian origin was followed by the discovery that these patients harbor more frequent EGFR mutations in NSCLC; this raises the issue of ethnic diversity in the pathogenesis of given tumors. In a similar fashion, amplification of the closely related HER2 gene, which could also have implications for the treatment of NSCLC, is also more frequent in East Asian patients. On the other hand, EGFR gene amplification may be more prevalent in Western populations (Calvo and Baselga, 2006). In this meta-analysis, only a few studies were conducted in developing countries, including 1 from Iran, 1 from Brazil and 1 from China. Lack of diversity in the observed countries may lead to bias not only in the total effect but also in the sub-analysis of cancer types.

Interestingly, though no statistically significant association between EMF-ELFs and cancer risk was found in the global analysis of European studies, conflict conclusions were obtained when classified according to their countries. Increased risks were observed in Norway and France where the data were collected by interview or different calculation programs. However, decreased risks were observed in Sweden and England where most of the surveys were conducted by the same group with the same calculation program. Additionally, we hypothesized that different cancer types investigated in different countries may contribute to this heterogeneity.

Due to various exposure methods, the exposure intensity and time may also differ. The occupational exposure limit is often much higher than the public exposure limit. Although some studies have taken exposure time and different types of work places into consideration, most of the studies only focused on limited life habits, residential environmental factors or work environmental factors. The adjustment of limited factors may lead to the loss of the complexity of the situation.

Considering the measurement type, because of the different exposure standards in different countries, the results from qualitative studies

Table 2
Meta-analysis for ELF-EMF exposure and cancer risk in device measured studies.

Exposure model	Number of study	Pooled OR	95% CI	P h[a]	P [b]
Total	16	1.00[c]	0.93, 1.09[c]	0.17	0.92[c]
Country					
North America	7	1.05	0.95, 1.16	0.82	0.38
United States	6	1.05	0.94, 1.16	0.72	0.40
others	1	1.14	0.56, 2.30	N/A	0.72
Europe	7	0.90	0.79, 1.03	0.17	0.13
Norway	2	1.37	0.79, 2.39	0.65	0.26
Italy	2	0.82	0.51, 1.32	0.56	0.42
others	3	0.88	0.76, 1.02	0.05	0.09
others	2	1.68	1.01, 2.77	0.33	0.04
Cancer type[d]					
breast cancer	8[e]	1.05	0.95, 1.16	0.88	0.39
postmenopausal	2	0.96	0.81, 1.14	0.79	0.63
premenopausal	2	1.23	1.01, 1.49	0.18	0.04
mixed	6	1.05	0.90, 1.24	0.92	0.52
brain cancer	4	0.94	0.64, 1.39	0.40	0.77
childhood	3	0.92	0.60, 1.41	0.24	0.72
mixed	1	1.05	0.40, 2.75	N/A	0.91
leukemia	5	0.89	0.75, 1.07	0.02	0.21
childhood	3	0.84	0.69, 1.02	0.11	0.08
mixed	2	1.15	0.78, 1.71	0.02	0.48
others	2	1.01	0.79, 1.29	0.34	0.94
Exposure methods					
occupational exposures	5	1.06	0.91, 1.24	0.16	0.46
residential exposures	4	1.00	0.80, 1.26	0.99	0.99
electric blanket exposures	1	1.11	0.94, 1.30	N/A	0.22
house exposures	6	0.90	0.79, 1.03	0.10	0.14

[a] *P* h value of Q-test for heterogeneity test. Random-effects model was used when *P* value for heterogeneity test <0.1; otherwise, fixed-effects model was used.
[b] *P* value for significance.
[c] This subgroup was analyzed by fixed-effects model.
[d] Some of the studies included several cancer types.
[e] Two of the 8 studies about breast cancer included both postmenopausal and premenopausal patients. The results were calculated by different degree of freedom.

may lead to statistically significant heterogeneity. Therefore, we performed a sub-analysis in device-measured studies. As expected, the included studies showed no heterogeneity. Contrary to the total results in Table 1, no statistically significant increased risk was found in total cancers. As shown in Supplemental Table 1, most of the recent studies were not conducted only by interview. We believe that more convincing results will be achieved as more quantitative studies are conducted in the future.

Additionally, variations in study design, methodological challenges and the quality of the inclusion of study participants may also contribute to differences in the previous studies.

The first meta-analysis studying cancer risk and ELF-EMF exposure was published in 1994(Washburn et al., 1994), while the latest two were in 2013(Chen et al., 2013). Recently, there have been new studies, so the total effect should be re-analyzed. In view of the heterogeneity of cancer, we pooled all studies together at the initial exploration to analyze the total effects as performed previously (Hardell et al., 1995). Then, we conducted subgroup analysis to distinguish the specific diversity, including countries, cancer types, exposure methods and measuring type.

Different from past studies, we analyzed the specific association not only among different subgroups but also among various types of cancer. Our meta-analysis pooled the largest number of cases and controls from included studies, which statistically significantly increases the statistical power, but some limitations should be considered. First, due to different methods of investigation, cohort studies were not included. Second, the pathophysiological processes of cancer are heterogeneous (Hanahan and Weinberg, 2011). Cancer-susceptibility genes combined with environmental/occupational risk factors promote the progression of cancer (Foulkes et al., 2015). Genetic factors were not combined with environmental exposure in the current investigation. Third, although sensitivity analysis showed that each study does not affect the total result, some heterogeneity was evident in some of the comparisons. Finally, the variations in study design, methodological challenges and quality of the inclusion of study participations should also be taken into consideration.

In conclusion, in our study, relevant literature selected from broad databases with stringent standards revealed an increased risk of cancer upon ELF-EMF exposure. However, more quantitative studies will contribute to more comprehensive results in the future.

Supplementary data to this article can be found online at http://dx.doi.org/10.1016/j.envint.2015.12.012.

Acknowledgments

This work was supported by the Science and Technology Project of State Grid Corporation of China (Grant GY71-13-057 to C.C). The

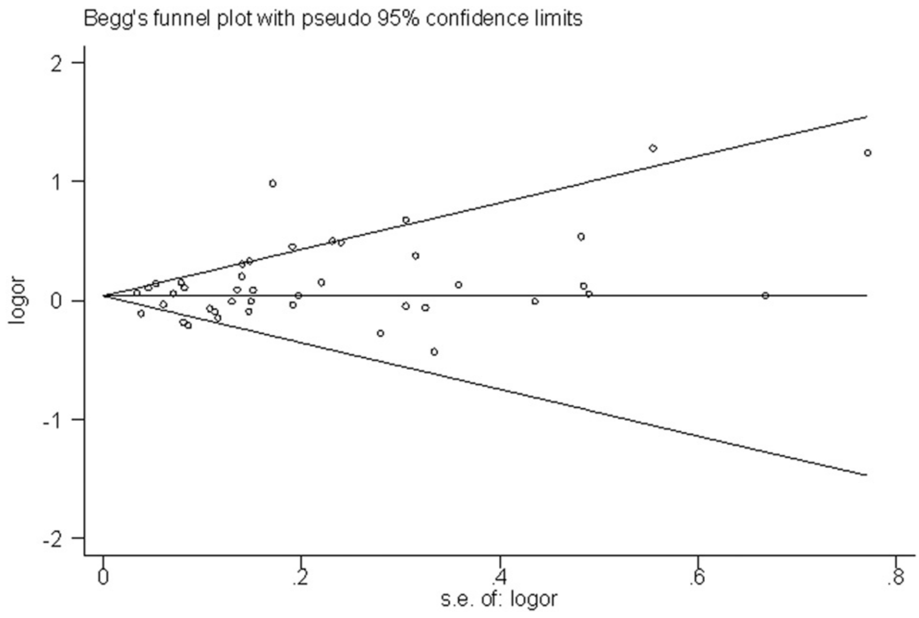

Fig. 3. Begg's funnel plot for the publication bias test. Each point represents a separate study for the indicated association. Log OR, natural logarithm of OR.

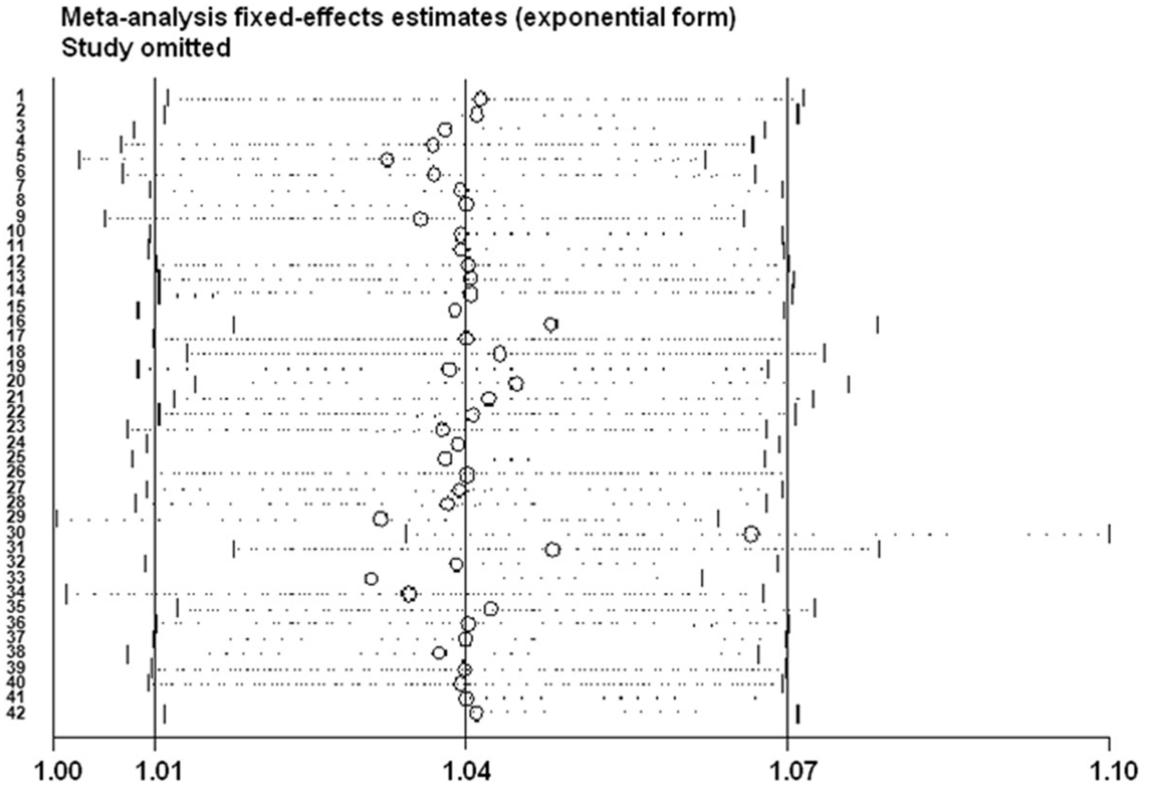

Fig. 4. Sensitivity analysis of the influence of a single study on the total meta-analysis estimate. The solid lines correspond to the pooled OR and 95% CI. Circles and dashed lines correspond to the specific OR and 95% CI without the omitted study.

funders had no role in the study design, data collection and analysis, decision to publish, or preparation of the manuscript.

The authors have declared that no competing interests exist.

The manuscript was edited by Elsevier Language Editing Services.

References

(NRPB), 2001. N.R.P.B. ELF Electromagnetic Fields and the Risk of Cancer, Report of an Advisory Group on Non-ionizing Radiation (Doc NRPB 12) Chilton, UK .

Baldi, I., Coureau, G., Jaffre, A., Gruber, A., Ducamp, S., Provost, D., Lebailly, P., Vital, A., Loiseau, H., Salamon, R., 2011. Occupational and residential exposure to electromagnetic fields and risk of brain tumors in adults: a case–control study in Gironde, France. Int. J. Cancer 129, 1477–1484.

Benassi, B., Filomeni, G., Montagna, C., Merla, C., Lopresto, V., Pinto, R., Marino, C., Consales, C., 2015. Extremely low frequency magnetic field (ELF-MF) exposure sensitizes SH-SY5Y cells to the pro-Parkinson's Disease toxin MPP. Mol. Neurobiol.

Bethwaite, P., Cook, A., Kennedy, J., Pearce, N., 2001. Acute leukemia in electrical workers: a New Zealand case–control study. Cancer Causes Control 12, 683–689.

Calvo, E., Baselga, J., 2006. Ethnic differences in response to epidermal growth factor receptor tyrosine kinase inhibitors. J. Clin. Oncol. Off. J. Am. Soc. Clin. Oncol. 24, 2158–2163.

Chen, C., Ma, X., Zhong, M., Yu, Z., 2010. Extremely low-frequency electromagnetic fields exposure and female breast cancer risk: a meta-analysis based on 24,338 cases and 60,628 controls. Breast Cancer Res. Treat. 123, 569–576.

Chen, Q., Lang, L., Wu, W., Xu, G., Zhang, X., Li, T., Huang, H., 2013. A meta-analysis on the relationship between exposure to ELF-EMFs and the risk of female breast cancer. PLoS ONE 8, e69272.

Coble, J.B., Dosemeci, M., Stewart, P.A., Blair, A., Bowman, J., Fine, H.A., Shapiro, W.R., Selker, R.G., Loeffler, J.S., Black, P.M., Linet, M.S., Inskip, P.D., 2009. Occupational exposure to magnetic fields and the risk of brain tumors. Neuro-Oncology 11, 242–249.

Costin, G.E., Birlea, S.A., Norris, D.A., 2012. Trends in wound repair: cellular and molecular basis of regenerative therapy using electromagnetic fields. Curr. Mol. Med. 12, 14–26.

Davis, S., Mirick, D.K., Stevens, R.G., 2002. Residential magnetic fields and the risk of breast cancer. Am. J. Epidemiol. 155, 446–454.

DiGiovanni, J., Johnston, D.A., Rupp, T., Sasser, L.B., Anderson, L.E., Morris, J.E., Miller, D.L., Kavet, R., Walborg Jr., E.F., 1999. Lack of effect of a 60 Hz magnetic field on biomarkers of tumor promotion in the skin of SENCAR mice. Carcinogenesis 20, 685–689.

Egger, M., Davey Smith, G., Schneider, M., Minder, C., 1997. Bias in meta-analysis detected by a simple, graphical test. BMJ 315, 629–634.

Elliott, P., Shaddick, G., Douglass, M., de Hoogh, K., Briggs, D.J., Toledano, M.B., 2013. Adult cancers near high-voltage overhead power lines. Epidemiology 24, 184–190.

Feizi, A.A., Arabi, M.A., 2007. Acute childhood leukemias and exposure to magnetic fields generated by high voltage overhead power lines — a risk factor in Iran. Asian Pac. J. Cancer Prev. 8, 69–72.

Feychting, M., Ahlbom, A., 1993. Magnetic fields and cancer in children residing near Swedish high-voltage power lines. Am. J. Epidemiol. 138, 467–481.

Forssen, U.M., Feychting, M., Rutqvist, L.E., Floderus, B., Ahlbom, A., 2000. Occupational and residential magnetic field exposure and breast cancer in females. Epidemiology 11, 24–29.

Forssen, U.M., Rutqvist, L.E., Ahlbom, A., Feychting, M., 2005. Occupational magnetic fields and female breast cancer: a case–control study using Swedish population registers and new exposure data. Am. J. Epidemiol. 161, 250–259.

Forssen, U.M., Lonn, S., Ahlbom, A., Savitz, D.A., Feychting, M., 2006. Occupational magnetic field exposure and the risk of acoustic neuroma. Am. J. Ind. Med. 49, 112–118.

Foster, K.R., Glaser, R., 2007. Thermal mechanisms of interaction of radiofrequency energy with biological systems with relevance to exposure guidelines. Health Phys. 92, 609–620.

Foulkes, W.D., Knoppers, B.M., Turnbull, C., 2015. Population genetic testing for cancer susceptibility: founder mutations to genomes. Nat. Rev. Clin. Oncol.

Funk, R.H., Monsees, T., Ozkucur, N., 2009. Electromagnetic effects — from cell biology to medicine. Prog. Histochem. Cytochem. 43, 177–264.

Gammon, M.D., Schoenberg, J.B., Britton, J.A., Kelsey, J.L., Stanford, J.L., Malone, K.E., Coates, R.J., Brogan, D.J., Potischman, N., Swanson, C.A., Brinton, L.A., 1998. Electric blanket use and breast cancer risk among younger women. Am. J. Epidemiol. 148, 556–563.

Grayson, J.K., 1996. Radiation exposure, socioeconomic status, and brain tumor risk in the US Air Force: a nested case–control study. Am. J. Epidemiol. 143, 480–486.

Grellier, J., Ravazzani, P., Cardis, E., 2014. Potential health impacts of residential exposures to extremely low frequency magnetic fields in Europe. Environ. Int. 62, 55–63.

Group, E.-E.E.F.S., 1997. Need for a European approach to the effects of extremely low-frequency electromagnetic fields on cancer. Scand. J. Work Environ. Health 23, 5–14.

Hanahan, D., Weinberg, R.A., 2011. Hallmarks of cancer: the next generation. Cell 144, 646–674.

Hardell, L., Holmberg, B., Malker, H., Paulsson, L.E., 1995. Exposure to extremely low frequency electromagnetic fields and the risk of malignant diseases—an evaluation of epidemiological and experimental findings. Eur. J. Cancer Prev. 4 (Suppl. 1), 3–107.

Heath Jr., C.W., 1991. Cancer risk and extremely low frequency electromagnetic radiation. CA Cancer J. Clin. 41, A21–A22.

Humans, I.W.G.o.t.E.o.C.R.t, 2002. Non-ionizing radiation, Part 1: static and extremely low-frequency (ELF) electric and magnetic fields. IARC monographs on the evaluation of carcinogenic risks to humans/World Health Organization 80. International Agency for Research on Cancer, pp. 1–395.

Investigators, U.C.C.S., 1999. Exposure to power-frequency magnetic fields and the risk of childhood cancer. Lancet 354, 1925–1931.

Juutilainen, J., 2008. Do electromagnetic fields enhance the effects of environmental carcinogens? Radiat. Prot. Dosim. 132, 228–231.

Kabat, G.C., O'Leary, E.S., Schoenfeld, E.R., Greene, J.M., Grimson, R., Henderson, K., Kaune, W.T., Gammon, M.D., Britton, J.A., Teitelbaum, S.L., Neugut, A.I., Leske, M.C., Group, E., 2003. Electric blanket use and breast cancer on Long Island. Epidemiology 14, 514–520.

Kliukiene, J., Tynes, T., Andersen, A., 2003. Follow-up of radio and telegraph operators with exposure to electromagnetic fields and risk of breast cancer. Eur. J. Cancer Prev. 12, 301–307.

Kliukiene, J., Tynes, T., Andersen, A., 2004. Residential and occupational exposures to 50-Hz magnetic fields and breast cancer in women: a population-based study. Am. J. Epidemiol. 159, 852–861.

Kroll, M.E., Swanson, J., Vincent, T.J., Draper, G.J., 2010. Childhood cancer and magnetic fields from high-voltage power lines in England and Wales: a case–control study. Br. J. Cancer 103, 1122–1127.

Labreche, F., Goldberg, M.S., Valois, M.F., Nadon, L., Richardson, L., Lakhani, R., Latreille, B., 2003. Occupational exposures to extremely low frequency magnetic fields and post-menopausal breast cancer. Am. J. Ind. Med. 44, 643–652.

Lee, S.K., Park, S., Gimm, Y.M., Kim, Y.W., 2014. Extremely low frequency magnetic fields induce spermatogenic germ cell apoptosis: possible mechanism. BioMed Res. Int. 567183, 2014.

Li, C.Y., Theriault, G., Lin, R.S., 1997. Residential exposure to 60-Hertz magnetic fields and adult cancers in Taiwan. Epidemiology 8, 25–30.

London, S.J., Pogoda, J.M., Hwang, K.L., Langholz, B., Monroe, K.R., Kolonel, L.N., Kaune, W.T., Peters, J.M., Henderson, B.E., 2003. Residential magnetic field exposure and breast cancer risk: a nested case–control study from a multiethnic cohort in Los Angeles County, California. Am. J. Epidemiol. 158, 969–980.

Loomis, D.P., Savitz, D.A., Ananth, C.V., 1994. Breast cancer mortality among female electrical workers in the United States. J. Natl. Cancer Inst. 86, 921–925.

McElroy, J.A., Newcomb, P.A., Remington, P.L., Egan, K.M., Titus-Ernstoff, L., Trentham-Dietz, A., Hampton, J.M., Baron, J.A., Stampfer, M.J., Willett, W.C., 2001. Electric blanket or mattress cover use and breast cancer incidence in women 50–79 years of age. Epidemiology 12, 613–617.

McElroy, J.A., Egan, K.M., Titus-Ernstoff, L., Anderson, H.A., Trentham-Dietz, A., Hampton, J.M., Newcomb, P.A., 2007. Occupational exposure to electromagnetic field and breast cancer risk in a large, population-based, case–control study in the United States. J. Occup. Environ. Med. 49, 266–274.

Michaelis, J., Schuz, J., Meinert, R., Menger, M., Grigat, J.P., Kaatsch, P., Kaletsch, U., Miesner, A., Stamm, A., Brinkmann, K., Karner, H., 1997. Childhood leukemia and electromagnetic fields: results of a population-based case–control study in Germany. Cancer Causes Control 8, 167–174.

Nikolova, T., Czyz, J., Rolletschek, A., Blyszczuk, P., Fuchs, J., Jovtchev, G., Schuderer, J., Kuster, N., Wobus, A.M., 2005. Electromagnetic fields affect transcript levels of apoptosis-related genes in embryonic stem cell-derived neural progenitor cells. FASEB J. 19, 1686–1688.

Olsen, J.H., Nielsen, A., Schulgen, G., 1993. Residence near high voltage facilities and risk of cancer in children. BMJ 307, 891–895.

Parodi, S., Merlo, D.F., Ranucci, A., Miligi, L., Benvenuti, A., Rondelli, R., Magnani, C., Haupt, R., Group, S.W., 2014. Risk of neuroblastoma, maternal characteristics and perinatal exposures: the SETIL study. Cancer Epidemiol. 38, 686–694.

Pedersen, C., Raaschou-Nielsen, O., Rod, N.H., Frei, P., Poulsen, A.H., Johansen, C., Schuz, J., 2014. Distance from residence to power line and risk of childhood leukemia: a population-based case–control study in Denmark. Cancer Causes Control 25, 171–177.

Preston-Martin, S., Navidi, W., Thomas, D., Lee, P.J., Bowman, J., Pogoda, J., 1996. Los Angeles study of residential magnetic fields and childhood brain tumors. Am. J. Epidemiol. 143, 105–119.

Repacholi, M.H., Greenebaum, B., 1999. Interaction of static and extremely low frequency electric and magnetic fields with living systems: health effects and research needs. Bioelectromagnetics 20, 133–160.

Saito, T., Nitta, H., Kubo, O., Yamamoto, S., Yamaguchi, N., Akiba, S., Honda, Y., Hagihara, J., Isaka, K., Ojima, T., Nakamura, Y., Mizoue, T., Ito, S., Eboshida, A., Yamazaki, S., Sokejima, S., Kurokawa, Y., Kabuto, M., 2010. Power-frequency magnetic fields and childhood brain tumors: a case–control study in Japan. J. Epidemiol. 20, 54–61.

Salvan, A., Ranucci, A., Lagorio, S., Magnani, C., Group, S.R., 2015. Childhood leukemia and 50 Hz magnetic fields: findings from the Italian SETIL case–control study. Int. J. Environ. Res. Public Health 12, 2184–2204.

Schoenfeld, E.R., O'Leary, E.S., Henderson, K., Grimson, R., Kabat, G.C., Ahnn, S., Kaune, W.T., Gammon, M.D., Leske, M.C., Group, E., 2003. Electromagnetic fields and breast cancer on Long Island: a case–control study. Am. J. Epidemiol. 158, 47–58.

Sermage-Faure, C., Demoury, C., Rudant, J., Goujon-Bellec, S., Guyot-Goubin, A., Deschamps, F., Hemon, D., Clavel, J., 2013. Childhood leukaemia close to high-voltage power lines—the Geocap study, 2002–2007. Br. J. Cancer 108, 1899–1906.

Stang, A., 2010. Critical evaluation of the Newcastle–Ottawa scale for the assessment of the quality of nonrandomized studies in meta-analyses. Eur. J. Epidemiol. 25, 603–605.

Stenlund, C., Floderus, B., 1997. Occupational exposure to magnetic fields in relation to male breast cancer and testicular cancer: a Swedish case–control study. Cancer Causes Control 8, 184–191.

Teepen, J.C., van Dijck, J.A., 2012. Impact of high electromagnetic field levels on childhood leukemia incidence. Int. J. Cancer 131, 769–778.

Tynes, T., Haldorsen, T., 1997. Electromagnetic fields and cancer in children residing near Norwegian high-voltage power lines. Am. J. Epidemiol. 145, 219–226.

Tynes, T., Hannevik, M., Andersen, A., Vistnes, A.I., Haldorsen, T., 1996. Incidence of breast cancer in Norwegian female radio and telegraph operators. Cancer Causes Control 7, 197–204.

Van Wijngaarden, E., Nylander-French, L.A., Millikan, R.C., Savitz, D.A., Loomis, D., 2001. Population-based case–control study of occupational exposure to electromagnetic fields and breast cancer. Ann. Epidemiol. 11, 297–303.

Vena, J.E., Graham, S., Hellmann, R., Swanson, M., Brasure, J., 1991. Use of electric blankets and risk of postmenopausal breast cancer. Am. J. Epidemiol. 134, 180–185.

Vena, J.E., Freudenheim, J.L., Marshall, J.R., Laughlin, R., Swanson, M., Graham, S., 1994. Risk of premenopausal breast cancer and use of electric blankets. Am. J. Epidemiol. 140, 974–979.

Wang, Z., Cao, Y., Jiang, C., Yang, G., Wu, J., Ding, Y., 2012. Lack of association of two common polymorphisms rs2910164 and rs11614913 with susceptibility to hepatocellular carcinoma: a meta-analysis. PLoS ONE 7, e40039.

Washburn, E.P., Orza, M.J., Berlin, J.A., Nicholson, W.J., Todd, A.C., Frumkin, H., Chalmers, T.C., 1994. Residential proximity to electricity transmission and distribution equipment and risk of childhood leukemia, childhood lymphoma, and childhood nervous system tumors: systematic review, evaluation, and meta-analysis. Cancer Causes Control 5, 299–309.

Wertheimer, N., Leeper, E., 1979. Electrical wiring configurations and childhood cancer. Am. J. Epidemiol. 109, 273–284.

Wunsch-Filho, V., Pelissari, D.M., Barbieri, F.E., Sant'Anna, L., de Oliveira, C.T., de Mata, J.F., Tone, L.G., Lee, M.L., de Andrea, M.L., Bruniera, P., Epelman, S., Filho, V.O., Kheifets, L., 2011. Exposure to magnetic fields and childhood acute lymphocytic leukemia in Sao Paulo, Brazil. Cancer Epidemiol. 35, 534–539.

Zhang, Y., Liu, X., Zhang, J., Li, N., 2015. Short-term effects of extremely low frequency electromagnetic fields exposure on Alzheimer's disease in rats. Int. J. Radiat. Biol. 91, 28–34.

Zhao, Q.R., Lu, J.M., Yao, J.J., Zhang, Z.Y., Ling, C., Mei, Y.A., 2015. Neuritin reverses deficits in murine novel object associative recognition memory caused by exposure to extremely low-frequency (50 Hz) electromagnetic fields. Sci. Rep. 5, 11768.

Zheng, T., Holford, T.R., Mayne, S.T., Owens, P.H., Zhang, B., Boyle, P., Carter, D., Ward, B., Zhang, Y., Zahm, S.H., 2000. Exposure to electromagnetic fields from use of electric blankets and other in-home electrical appliances and breast cancer risk. Am. J. Epidemiol. 151, 1103–1111.

Zhu, K., Hunter, S., Payne-Wilks, K., Roland, C.L., Forbes, D.S., 2003. Use of electric bedding devices and risk of breast cancer in African-American women. Am. J. Epidemiol. 158, 798–806.
