# Supplementary material for: Magnetic Fields and Cancer: Epidemiology, Cellular Biology, and Theranostics
Source: Int J Mol Sci. 2022 Jan 25;23(3):1339. doi: 10.3390/ijms23031339 (PMC8835851; doi:10.3390/ijms23031339)
Supplement: Supplementary file 1 [file ijms-23-01339-s001.zip › Supplementary Data Set S1/MF and Cancer.Data/PDF/0262626477/1-s2.0-S0079610704001075-main.pdf]

Available online at www.sciencedirect.com
SCIENCE DIRECT®

ELSEVIER

Progress in Biophysics and Molecular Biology 87 (2005) 225–239

Progress in Biophysics & Molecular Biology

www.elsevier.com/locate/pbiomolbio

Static magnetic fields: animal studies

Richard Saunders*

Radiation and Environmental Health Unit, Department of Protection of the Human Environment, Sustainable Development and Healthy Environment Cluster, World Health Organisation, 20, Avenue Appia, CH-1211 Geneva 27, Switzerland

Available online 28 October 2004

Abstract

Various experimental studies carried out over the last 30–40 years have examined the effects of the chronic or acute exposure of laboratory animals to static magnetic fields. Many of the earlier studies have been adequately reviewed elsewhere; few adverse effects were identified. This review focuses on studies carried out more recently, mostly those using vertebrates, particularly mammals. Four main areas of investigation have been covered, viz., nervous system and behavioural studies, cardiovascular system responses, reproduction and development, and genotoxicity and cancer. Work on the role of the natural geomagnetic field in animal orientation and migration has been omitted.

Generally, the acute responses found during exposure to static fields above about 4 T are consistent with those found in volunteer studies, namely the induction of flow potentials around the heart and the development of aversive/avoidance behaviour resulting from body movement in such fields. No consistently demonstrable effects of exposure to fields of ~1 T and above have been seen on other behavioural or cardiovascular endpoints. In addition, no adverse effects of such fields on reproduction and development or on the growth and development of tumours have been firmly established. Overall, however, far too few animal studies have been carried out to reach any firm conclusions.
© 2004 Elsevier Ltd. All rights reserved.

Keywords: Review; Animal studies; Static magnetic field; Behaviour; Physiology; Development

*Fax: +41 22 791 41 23.
E-mail address: saundersr@who.int (R. Saunders).

0079-6107/$ - see front matter © 2004 Elsevier Ltd. All rights reserved.
doi:10.1016/j.pbiomolbio.2004.09.001

1. Introduction

Living organisms are continuously exposed to the natural geomagnetic field of around 20–70 μT that exists over the surface of the Earth, and which is implicated in the orientation and migration of certain animal species. Naturally occurring magnetic minerals such as magnetite or 'loadstone' have been sometimes used for therapeutic purposes since the middle ages (Schenck, 2000). However, the development of electromagnets in the 19th century and of superconducting magnets in the mid-20th century made possible the exposure of humans to intense magnetic fields. With the further development of magnetic resonance imaging (MRI) for clinical use, such exposure, often to fields some tens of thousands of times the magnitude of the geomagnetic field, has become routine. Somewhat surprisingly, however, the possible adverse health effects of such exposure have been investigated in a somewhat desultory manner, mostly over the past 30–40 years. The earlier literature has been summarised by WHO (1987), Kowalczuk et al. (1991) and ICNIRP (1994, 1997), whilst more recent studies have been reviewed by Repacholi and Greenebaum (1999), IARC (2002), ICNIRP (2003) and McKinlay et al. (2004).

The focus of this review is on recent mammalian studies, where possible. These studies are covered under four main sections: nervous system and behavioural studies, cardiovascular system responses, reproduction and development, and genotoxicity and cancer. A study describing effects on the blood–brain barrier is also briefly described. However, the role of geomagnetic fields in the orientation and migration of various animal species has not been addressed.

2. Neurobehavioural studies

Electrophysiological responses have been reported by a few laboratories following exposure to relatively moderate static fields but the results of the few behavioural studies undertaken are consistent only with reasonably understood high field interaction mechanisms. The few neuroendocrine studies that have been undertaken have mostly focused on melatonin, a hormone which is secreted from the pineal gland in the brain and is implicated in the control of daily activities such as the sleep/wake cycle and in seasonal behaviour in some animal species.

2.1. Neurophysiological responses

The Lorentz force that acts on moving charge carriers such as ionic currents might be expected to affect ion channel conduction properties, thereby affecting nervous system function. However, calculation by Wikswo and Barach (1980) suggests that a field of ~24 T would be required to produce a 10% change in Na^{+} or K^{+} ion channel conductivity, with larger fields required for heavier ions.

Support for this view comes from the work of three groups. Schwartz (1978, 1979) found no effect of a 1.2 T field on action potential conduction velocity and ion channel currents in lobster giant axons. Similarly, Tenforde and colleagues found no effect of a 2 T field on conduction velocity, refractory period and excitation threshold in excised frog sciatic nerve preparations (Gaffey and Tenforde, 1983). Tenforde et al. (1985) also reported that a similar exposure (to 1.5 T) had no effect on the electro-retinogram response of cats and squirrel monkeys. Hong et al. (1986)

and Hong (1987) found no effect of exposure to a static field of up to 1.2 or to 1 T on nerve conduction velocity in anaesthetised rats nor in (awake) human subjects, respectively. In addition, no effect of exposure to a 1.5 T field was reported on the amplitude or latency of somatosensory evoked potentials in volunteers (Hong and Shellock, 1990).

An apparently contrary view comes from half a dozen or so studies carried out by Rosen and colleagues (e.g. Rosen, 1992, 1993, 1994, 1996, 2003a; Rosen and Lubowsky, 1987, 1990), summarised by Rosen (2003b). These authors found that exposure of cultured GH3 pituitary cells to 120 mT for 150 s resulted in a slow but pronounced (up to 200%) increase in the activation time-constants of the voltage-gated sodium and calcium ion channels (Rosen 1996, 2003a) which lasted during and at least 100 s after exposure, but with no change in the inactivation time-constant. These results appeared consistent with earlier observations of a reduced visual evoked potential and lower spontaneous discharge seen in the lateral geniculate body in the cat brain following similar exposures (Rosen and Lubowsky, 1987, 1990). Rosen (2003b) suggested that these effects result from the slow re-orientation of aligned groups of diamagnetic phospholipid molecules within the cell membrane distorting and thereby affecting ion channel function.[1]

Wieraszko and colleagues (Trabulsi et al., 1996; Wieraszko, 2000) reported that even weaker fields could modulate synaptic excitability as measured in mouse hippocampal slice preparations. The evoked population spike amplitude was initially depressed and then enhanced following a 20 min exposure to a 2–3 mT static field. The recovery, but not the initial depression, could be inhibited by dantrolene, an intracellular Ca^{2+} channel blocker. Further study suggested that the initial depression might result from inhibition of the voltage-gated Ca^{2+} channels on the presynaptic membrane, whereas the recovery phase was attributed to the release of calcium from internal stores.

The authors speculated that these results were consistent with the observations and suggestions of Rosen (1992). In contrast, Sonnier et al. (2000, 2003) found no changes in the resting membrane potential or action potential in neuroblastoma cells following brief exposures to fields of up to 7.5 mT. These included measurements, also based on patch-clamp methods, of the activation and inactivation time-constants of sodium ion channels and the inactivation rate of the potassium ion channel. Interestingly, these authors also found that static fields combined with 60 Hz time-varying fields of up to 0.5 mT also had no effect on these parameters. However, the studies described by Sonnier and colleagues were all carried out at 25 °C, whereas the effects described by Rosen (2003b) occurred only above temperatures of 35 °C.

2.2. Behavioural effects

If there are significant effects on nervous system function, then there may be behavioural consequences. One extensive study by Tenforde and colleagues found that the continuous exposure of mice for 72 h to a 1.5 T magnetic field had no effect on circadian rhythms of activity, nor on the retention of a learned avoidance task (Davis et al., 1984). In addition, no effect was seen on the threshold for a chemically (pentylenetetrazole) induced epileptic seizure.

[1]The degree to which the observations of a magnetic field induced reduction in Na^{+} channel activation time-constant are consistent with lack of effect seen on action potential conduction velocity is explored in a following paper (Hinch et al., 2004, this issue).

A series of more recent studies have looked at the effects of higher levels of exposure. Weiss et al. (1992) examined the behaviour of rats placed in a T-maze in which one of the arms of the maze extended into the bore of magnet that could be ramped from 0 to 4 T. The authors found that not only would rats not enter a 4 T field, but also that in a reversed maze, previously exposed rats turned away from the arm in which the magnetic field exposure would previously have occurred, indicating that the experience had induced an aversive response. A similar experiment by Nolte et al. (1998), in which rats, given a conditioned stimulus (a taste solution) followed by exposure for 30 min to 9.4 T magnetic field, showed a conditioned taste aversion that lasted for up to 8 days after the cessation of field exposure.

These behavioural effects of exposure to intense static fields were further explored in a series of recent studies by Houpt and colleagues (Snyder et al., 2000; Houpt et al., 2003; Lockwood et al., 2003). Exposure to a 9.4 T magnetic field for the same duration used by Nolte et al. (1998) resulted in increased c-Fos expression, taken as an index of neural activity, in the vestibular and visceral nuclei of the brain suggesting activation of the vestibular or visceral neural pathways (Snyder et al., 2000). These authors noted that such effects were consistent with the suggestion by Schenck (2000, 2004, this issue) that small head movements in a large static field could induce vestibular stimulation through the action of magnetohydrodynamic forces on the fluid within the semicircular canals. Further study by these authors (Houpt et al., 2003; Lockwood et al., 2003), using fields of 7 and 14 T, found that conditioned taste aversion and tight circling movements could be induced in rats and mice in an exposure duration/intensity dependent manner. All these responses were thought to be consistent with vestibular stimulation and the reports of vertigo and nausea in people working in high strength magnetic fields.

Previous behavioural studies, carried out at lower flux densities, have been more variable. Levine et al. (1995) and Levine and Bluni (1994) reported that exposure of rats to a static field of up to 2.0 T suppressed spatial discrimination learning using a T-maze task. However, Levine et al. (1995) reported that the 2.0 T field was applied ‘using a spin-echo technique’ duplicating a clinical exposure, raising the possibility that switched gradient fields may also have been present. Trzeciak et al. (1993) reported reduced ‘irritability’, i.e. responsiveness to being touched, in rats exposed to 0.49 T for 2 h day^{-1} for 20 days, but no effect on open field behaviour or locomotor activity. Nakagawa and Matsuda (1988) found that the night-time exposure of rats to a field of 0.6 T reduced the subsequent performance of two operant behaviour tasks (Sidman avoidance and discriminative avoidance tasks). Finally, Hong et al. (1988) reported that exposure of rats for 15 min day^{-1} from birth up to postnatal day 14 had no effect on the subsequent performance of a T-maze task.

2.3. Neuroendocrine studies

A number of studies, carried out mostly in the 1980s and early 1990s, suggest that manipulation of the ambient static magnetic field may disturb the normal circadian melatonin rhythm in rodents (see Kowalczuk et al., 1991; Reiter, 1993). In these studies, night-time inversion or changes in the orientation of the applied static field at flux densities approximately equivalent to that of the natural geomagnetic field produced changes in the melatonin content of the pineal gland or in enzymes involved in the metabolism of melatonin: weaker or stronger fields, and day-time exposure had lesser or no effects. (Such phenomena may be linked to the neurobiology of

magnetic field detection utilised in homing and migratory behaviour, e.g. Schneider et al., 1994.) However, Kroeker et al. (1996) reported that neither acute exposure at 7 T nor medium-term exposure at 80 mT had any effect on nocturnal pineal or serum melatonin levels in rats.

3. Blood–brain barrier

As part of an investigation into the effects of MRI, Prato et al. (1994) examined the effects of exposure to a 1.5 or 1.89 T static field on the permeability of the blood–brain barrier in rats. (Other groups were exposed to the full MRI complement of static, switched gradient and RF magnetic fields.) The authors examined the partition coefficient between brain tissue (the whole brain) and a blood sample of a radioactive tracer, ^{153}Gd-DTPA. They found that exposure to the static field increased the uptake of tracer by the brain, indicating that the blood–brain barrier permeability had increased. Interpretation is, however, complicated by the fact, as the authors noted, that the increased uptake could also have resulted from an increase in the cerebral blood volume, without any change to the permeability of the blood–brain barrier. Thus, it is difficult to draw any firm conclusion from this one study.

4. Cardiovascular system studies

Electrical potentials (flow potentials), generated across a blood vessel by the flow of blood in static magnetic field, have been recorded in a number of animal species exposed to magnetic fields greater than about 100 mT; their physiological significance, however, remains unclear. This phenomenon is described in detail by Tenforde (2004, this issue) and is summarised here. More widespread effects on cardiovascular function, including possible effects on arterial blood pressure and peripheral blood flow, are less clearly established.

4.1. Flow potential

Flow potentials result from Lorentz forces acting on moving charges and are generally associated with ventricular contraction and the ejection of blood into the aorta. They appear superimposed on the T-wave of the ECG, which indicates the repolarisation of the ventricular heart muscle when electrical excitability gradually recovers following contraction.

Briefly, flow potentials have been recorded in rats (Gaffey and Tenforde, 1981), rabbits (Togawa et al., 1967), dogs (Gaffey and Tenforde, 1979), monkeys (Beischer, 1969; Beischer and Knepton, 1964; Tenforde et al., 1983) and baboons (Gaffey et al., 1980) and are reviewed by ICNIRP (2003). In large animal species, the flow potential can be detected in the ECG at magnetic field levels above approximately 0.1 T, and is a linear function of field strength up to 1.0 T. At higher field levels, the total electrical potential at the T-wave locus in the ECG increases more rapidly as a function of magnetic field strength, possibly as a result of the superposition of additional, weaker flow potentials. It also increases with body size; in a 1.0 T field for example, the average increase in the T-wave signal amplitude in rats is ~75 μV whereas it is ~175 μV in juvenile baboons.

4.2. Cardiovascular system responses

The interaction between the applied magnetic field and a flowing electrolyte solution such as blood also generates a net volume force within the fluid which acts to reduce flow velocity and increase arterial blood pressure. However, calculation suggests that this will be very small, even in the human aorta, in fields of less than 1 T (Tenforde et al., 1985; ICNIRP, 2003). Tenforde et al. (1983) reported that exposure to static fields of up to 1.5 T had no effect on arterial blood pressure, a finding recently confirmed in humans exposed to static fields of up to 8 T (Kangarlu et al., 1999; Chakeres et al., 2003).

In contrast, two laboratories in Japan have found that exposure to static fields can have significant effects on the cardiovascular system, most notably on arterial blood pressure and skin blood flow. Ichioka et al. (1998, 2000, 2003) reported that the exposure of anaesthetised rats in an 8 T field significantly reduced the skin blood flow for the duration of exposure. This was associated with a decrease in the humidity of the air surrounding the exposed animals, which the authors suggest resulted from a magnetically induced movement of diamagnetic water molecules. There is, however, always some difficulty in interpreting thermoregulatory responses in anaesthetised animals.

The other group, Gmitrov (1996), Xu and Ohkubo (1997), Xu et al. (1998, 2000), Okano et al. (1999), Okano and Ohkubo (2001, 2003a, b), Gmitrov and Ohkubo (2002a, b) and Gmitrov et al. (2002) have reported significant effects on various aspects of cardiovascular system physiology in an extensive series of studies examining the effects of exposure, mostly to static magnetic fields of between 1 and 350 mT for anywhere between 10 min and 12 weeks. Parameters studied included arterial blood pressure and skin blood flow, mostly in rabbits, under conditions often involving significant pharmacological manipulation, including varying degrees of anaesthesia, and immobilisation. Some studies examined the impact of fluctuations in the natural geomagnetic field (~20–70 μT) on these effects.

Generally, the thrust of these studies has been the examination of the modulatory effects of static field exposure on spontaneous and induced hypertension and other measures of circulatory performance through their interaction with number of cardiovascular regulatory mechanisms, including baroreceptors and various hormonal and humoral agents such as angiotensin. The context in which these studies were undertaken was of the potential therapeutic effects of such fields on various disorders. However, the data often appear rather variable, although the statistical analysis seemed appropriate. Other potential difficulties include the fact that the endpoints examined are rather labile, a situation which may well have been complicated by the rather extensive pharmacological manipulation and immobilisation procedures. Overall, it is difficult to reach any firm conclusion about these studies without some independent replication of the effects.

5. Reproduction and development

Few studies have examined the effect of static magnetic fields on fertility; most concern possible effects on the developing embryo and fetus (teratogenic effects). Key factors in the investigation of the potential teratogenic effects of any agent include an awareness of the potential sensitivity of

the different developmental stages and the underlying developmental processes (see Edwards et al., 2003), and appropriate forms of statistical analysis. Periods of cell proliferation and migration are particularly vulnerable to many teratogens. With regard to statistical analyses, those based on the numbers of affected fetuses (used by some authors) will tend to overestimate the significance of any effect seen, because the assumption that individual fetuses within a litter are independent will lead to an underestimate the true variance (see AGNIR, 1994).

The studies reviewed below include those looking at intense static magnetic fields effects on male fertility, on cleavage and embryonic development in amphibia, which in these animal species is easy to visualise, and on embryo and fetal development in rats and mice, which are more relevant to humans. Since potential teratogenic effects are of considerable importance, studies of effects of MRI fields, which include switched gradient and RF magnetic fields, have also been reviewed.

5.1. Male fertility

Narra et al. (1996) reported slight changes in spermatogenesis and embryogenesis in mice exposed at 1.5 T for 30 min, although the data were rather variable. Tablado et al. (1996, 1998, 2000) reported that maturation of sperm movement in mice as well as postnatal testicular and epididymus development was largely unaffected by either single, short-term exposure or continuous, long-term exposure at 500–700 mT.

5.2. Cleavage and embryonic development in the amphibian embryo

The possibility that strong magnetic field gradients may affect embryonic development in amphibia has been raised by IARC (2002); early studies (Neurath, 1968; Ueno et al., 1984) had described abnormal growth and increased malformations in such embryos exposed to a static field of 1 T with field gradients of 10–1000 T m^{-1}. However, a later study by Ueno et al. (1994) briefly reported a lack of developmental effects, following exposure during the early stages of development, to 8 T fields, but it was not clear whether such exposure included strong field gradients.

More recently, Denegre et al. (1999) investigated the effect of exposure to static fields of up to ~17 T on the first three cleavages of the embryo of the African clawed toad *Xenopus laevis* used previously by Ueno et al. (1984, 1994). The authors found that the second and third cleavage would orient parallel to the plane of the magnetic field; the proportion of cleavages parallel to the field increasing with field strength above around 2 T to a maximum effect at around 17 T. The largest effects occurred in the homogeneous field rather than the gradient field and the authors suggested that the effect resulted from the interaction with diamagnetically anisotropic molecules in the mitotic apparatus, possibly the microtubules of the spindle formation since such alignment has been demonstrated in vitro elsewhere (Bras et al., 1998). However, it is not entirely clear whether such effects altered the proportion of embryos that developed into normal tadpoles.

5.3. Mammalian development—static field exposure

Studies of possible teratogenic effects on mammalian species are more relevant to humans. An early, rather comprehensive study by Sikov et al. (1979) did not find any effect of exposure to a

static field of 1 T, either before implantation, during organogenesis or during fetal development, on the pre- and post-natal development of mice. A later study by Konermann and Monig (1986), which focused particularly on cortical development in mice, also found no developmental effect of exposure to fields of 1 T. These findings, of a lack of effect of exposure during organogenesis, have been confirmed in more recent studies of mice exposed to fields of 4.7 T (Okazaki et al., 2001) and 6.3 T (Murakami et al., 1992).

More variable results have been seen in two studies looking at possible developmental effects in rats. Mevissen et al. (1994) reported a significant decrease in the number of live fetuses per litter in rats exposed for the entire period of gestation to a 30 mT static field. The authors suggest that such exposure might be embryotoxic. A significant increase on the total number of resorptions and number of fetuses with common skeletal variants was also reported, although, as indicated above, the significance of findings based on individual fetuses may well be overestimated.

A more recent study by High et al. (2000) reported a lack of postnatal developmental effects following exposure to 9.5 T for 3 h, twice a week, for 5 weeks before and 5 weeks after mating, which took place over 7 days during an intervening 2 week period. The authors examined a number of haematological, biochemical and behavioural endpoints in the adults and in their offspring. Unusually for an investigation of potential teratogenic effects, which here formed a small part of the main experiment on adults, it was not stated whether the date of mating was assessed, raising the possibility that gestational age during exposure may well have been only be roughly approximated. The authors themselves note only that exposure in utero took place during the embryo and fetal stages.

The results of the study on adults were marred by early problems with the anaesthesia regime used to sedate the animals during exposure, and with handling stress affecting the haematology and blood biochemistry results. With regard to the evaluation of effects in the offspring, examined 4 weeks postnatally, the authors stated that no treatment-related effects were seen, although no data were given describing the behavioural and neurophysiological assessments, nor was any evidence presented regarding the incidence of gross abnormalities and macro-pathological findings. Overall, regarding developmental outcome, the study was considered uninterpretable.

5.4. Mammalian development—magnetic resonance imaging exposures

The exposure of pregnant dams to all three magnetic field used in MRI, viz., static, gradient and RF, have been examined by three research groups. Whilst such exposures are more realistic (regarding MRI), any effects seen cannot reliably attributed to any single field component. Significant heating, which can result from excessive RF magnetic field absorption, is a known teratogen (see Edwards et al., 2003). In addition, high levels of acoustic noise, resulting from rapid gradient field switching, may induce stress-related effects. A lack of effect, however, would indicate that, if the experimental model used was appropriate and the experimental design of sufficient power, none of the above conditions would have significantly affected the outcome.

Tyndall (1993) examined the effects of exposure for 36 min to a 1.5 T field (plus unspecified gradient and RF fields) on day 7 of gestation, during development of the anterior neural plate. An increased percentage of fetuses per litter with reduced craniofacial perimeter and crown-rump length was reported in the exposed groups compared to sham exposed animals. The author discussed RF-induced heating as a possible mechanism, although no rise in body temperature was

recorded. Earlier studies (Tyndall, 1990; Tyndall and Sulik, 1991) reported that a similar exposure increased the incidence of eye abnormalities in the same strain of mouse (which is prone to this condition) but did not enhance the effect of X-ray induced increases in this endpoint.

Later studies by Magin and colleagues (Carnes and Magin, 1996; Magin et al., 2000) examined the effects of exposure of mice on days 9 and/or 12 gestation, i.e. during organogenesis, to static magnetic fields of around 4–5 T, combined with switched gradient and RF fields. In the study by Magin et al. (2000), mice were exposed to a static field of 4 T, a switched gradient field and a 170 MHz RF field, for which the average whole-body power absorption (specific energy absorption rate or SAR) was estimated to be 0.2 W kg^{-1}. Litter size was unaffected but significantly increased numbers of resorptions and fetal deaths occurred in the group exposed on day 12 of gestation, but not on day 9, nor on days 9 and 12 combined. In addition, a significant increase in the rate of acquisition of motor skills was seen in the mice exposed on day 9 of gestation, whereas this was decreased in the group exposed on day 12. However, the numbers of pregnant dams per treatment group were rather small, the analyses often based on total numbers of fetuses rather than numbers affected per litter, and the data were rather variable. Further, only the exposed groups experienced the loud (90–100 dB) acoustic noise generated by the switched gradient fields.

An earlier study (Carnes and Magin, 1996) had reported significantly reduced fetal weight, which is strongly influenced by litter size, in mice exposed for 8 h on day 9 gestation to a 4.7 T static magnetic field, a switched gradient field and a 200 MHz RF field where the whole-body SAR was estimated as 0.015 mW kg^{-1}. (Sound levels were not given). No effect was seen after exposure on day 12 of gestation, nor after exposure on days 9 and 12 combined. In addition, no effect was seen on the number of fetal deaths in any MRI exposure group. Sperm production, which was not significantly affected in the study described above (Magin et al., 2000), was significantly reduced in mice exposed on day 12 gestation, but not on day 9, nor on days 9 and 12 combined. Overall, despite the differences in experimental protocol, it is difficult to conclude that the effects described in the two studies are reproducible, either within or between the studies. A more likely explanation is of spurious differences introduced by small numbers, incomplete analysis and variable data.

Previously, Heinrichs et al. (1988) carried out a comprehensive study of the effects of exposure of mice for 16 h around the same period (~day 9 gestation) to MRI fields where the static magnetic field was 0.35 T; pulsed gradient and RF magnetic fields were also present. There were no effects on the incidence of prenatal deaths, nor that of skeletal defects but crown-rump length was significantly reduced in the MRI exposed group. This effect may have been overestimated, however, since the analysis was based on the number of affected fetuses and appeared to neglect litter effects (see above). The authors note that the noise generated within the magnet (by the switched gradient fields) may have been stressful and further comment on the 10% reduction in body weight due to dehydration over the 16 h treatment period seen in both exposed and sham exposed groups.

6. Genotoxicity and cancer

The genotoxicity of an agent is an indication of its potential to damage DNA, and is therefore implicated in the process of mutagenesis, whereby stable and heritable genetic mutations are generated, often through mis-repair. Mutations leading to the activation of oncogenes, or the inactivation of tumour suppressor genes, are early events in carcinogenesis, the formation of

cancers. Animal studies are often used in the evaluation of suspected human carcinogens (e.g. see AGNIR, 2001); either screening for an increased incidence of spontaneous tumours, or of the incidence of tumours induced by known carcinogens.

Genotoxic effects of exposure to static magnetic fields in have been mostly examine in cell cultures (see Myakoshi, 2004, this issue). Few in vivo studies of genotoxicity or possible effects on other carcinogenic processes have been carried out.

6.1. Genotoxicity

It is generally accepted that static fields below 1 T are not genotoxic (e.g. McCann et al., 1993; ICNIRP, 2003; McKinlay et al., 2004). However, a recent study by Suzuki et al. (2001) reported a significant, time and dose-dependent increase in micronucleus frequency in mice exposed to static magnetic fields of 2, 3 or 4.7 T for 24, 48 or 72 h, using a standard micronucleus assay. Bone marrow smears were taken immediately after exposure and the frequency of micronucleated polychromatic (immature) erythrocytes was scored. Micronucleus frequency was significantly increased following exposure to 4.7 T for all three time periods, and to 3 T after exposure for 48 or 72 h, whereas exposure to 2 T had no significant effect. The authors suggest that exposure to higher fields may have induced a stress reaction, or directly affected chromosome structure or separation during cell division.

6.2. Cancer studies

Few studies investigating the potential carcinogenicity of static magnetic fields have been carried out. With regard to possible effects on induced tumours, a lack of effect on survival time in mice with chemically induced epidermal tumours that were exposed to up to 800 mT for up to 1 h day^{-1} for 5 days week^{-1} until death was reported by Bellossi (1984). In a later study, Mevissen et al. (1993) reported that exposure of rats to a magnetic field of 15 mT for 13 weeks did not significantly affect the incidence of chemically induced mammary tumours, nor did it affect the number of tumours per animal compared with controls, although the weight per tumour was significantly increased.

The growth of transplanted tumours has been reported to be unaffected by exposure of mice to static fields of at least 1 T (Bellossi and Toujas, 1982; Bellossi, 1986). Bellossi and colleagues studied the effect of static field exposure on the growth of tumours in mice injected with Lewis Lung tumour cells. Exposure to uniform static fields (Bellossi and Toujas, 1982) of up to ~1 T for up to 8 h day^{-1} for 5 days week^{-1} until death or to non-uniform static fields (Bellossi, 1986) of up to ~1 T, with gradients of up to 3 T m^{-1} had no effect on the survival time. However, the experimental procedures and analysis of the data were described rather briefly, reducing the confidence that can be placed in these studies.

7. Summary and conclusions

It is clear from this and preceding reviews that there has been no systematic in vivo investigation of the biological effects of acute and/or chronic exposure to static magnetic fields, particularly the intense fields of more than 1 T now routinely employed in MRI.

Of those studies that have been carried out, the evidence suggests that the movement of laboratory animals in fields equal to or larger than 4 T may be unpleasant, probably resulting from forces acting on the vestibular apparatus of the inner ear. In addition, electrical potentials generated across the aorta and other major arteries by the flow of blood in a static field can be routinely seen in the ECG of animals, including primates, exposed to fields in excess of 100 mT. Both these effects have been reported in volunteer studies (see Chakeres et al., 2004, this issue). Otherwise, there are a number of reports from individual laboratories of effects on nervous system function, and of effects on blood flow, arterial blood pressure and other parameters of the cardiovascular system, often at fields much less than 1 T, which have not been corroborated elsewhere.

With regard to possible effects on reproduction and development, no adverse effects have been consistently demonstrated, but there have been few good studies, especially to fields in excess of 1 T. The existing evidence is, at present, inconclusive with regard to the possible effects that might ensue from exposure above this value. The MRI studies, taken as a whole, were inconclusive; the animal numbers were small, the data variable and the effect, if any, was impossible to disentangle from the other potential confounders present during exposure.

The evidence concerning carcinogenesis is inconclusive, too few studies have been carried out. A similar conclusion, that static magnetic fields are *not classifiable as to their carcinogenicity to humans*, has been published by IARC (2002).

Acknowledgements

My grateful thanks are given to Zenon Sienkiewicz, Christine Kowalczuk, Marisa Priestner and Anna Bottomley, NRPB, Chilton, Didcot, Oxon, UK, for their help in the preparation of this review.

References

AGNIR, 1994. Health effects related to the use of Visual Display Units. Report of an Advisory Group on Non-Ionising Radiation, vol. 5, no. 2. Documents of the NRPB, Chilton, UK.

AGNIR, 2001. ELF electromagnetic fields and the risk of cancer. Report of an Advisory Group on Non-Ionising Radiation, vol. 12, no. 1. Documents of the NRPB, Chilton, UK.

Bellossi, A., 1984. The effect of a static uniform magnetic field on mice a study of methylcholanthren carcinogenesis. Radiat. Environ. Biophys. 23, 107–109.

Bellossi, A., 1986. The effect of a static non-uniform magnetic field on mice a study of Lewis tumour graft. Radiat. Environ. Biophys. 25, 231–234.

Bellossi, A., Toujas, L., 1982. The effect of a static uniform magnetic field on mice. A study of a Lewis tumour graft. Radiat. Environ. Biophys. 20, 153–157.

Beischer, DE., 1969. Vectorcardiogram and aortic blood flow of squirrel monkeys (*Saimiri sciureus*) in a strong superconductive electromagnet. In: Barnothy, M.F. (Ed.), Biological Effects of Magnetic Fields, vol. 2. Plenum Press, New York, pp. 241–259.

Beischer, D.E., Knepton Jr., J.C., 1964. Influence of strong magnetic fields on the electrocardiogram of squirrel monkeys (*Saimiri sciureus*). Aerospa. Med. 35, 939–944.

Bras, W., Diakun, G.P., Diaz, J.F., Maret, G., Kramer, H., Bordas, J., Medrano, F.J., 1998. The susceptibility of pure tubulin to high magnetic fields: a magnetic birefringence and X-ray fibre diffraction study. Biophys. J. 74, 1509–1521.

Carnes, K.I., Magin, R.L., 1996. Effects of *in utero* exposure to 4.7 T MR imaging conditions on fetal growth and testicular development in the mouse. Magn. Reson. Imaging 14 (3), 263–274.

Chakeres, D.W., Kangarlu, A., Boudoulas, H., Young, D.C., 2003. Effect of static magnetic field exposure of up to 8 T on sequential human vital sign measurements. J. Magn. Reson. Imaging 18, 346–352.

Chakeres, D.W., et al., 2004. Static magnetic field effects on human subjects at 8 T. Prog. Biophys. Mol. Biol.

Davis, H.P., Mizumori, S.Y.J., Allen, H., Rosenzweig, M.R., Bennett, E.L., Tenforde, T.S., 1984. Behavioral studies with mice exposed to DC and 60-Hz magnetic fields. Bioelectromagnetics 5, 147–164.

Denegre, J.M., Valles, J.M., Lin, K., Jordan, W.B., Mowry, K.L., 1999. Cleavage planes in frog eggs are altered by strong magnetic fields. Proc. Natl. Acad. Sci. 95, 14729–14732.

Edwards, M.J., Saunders, R.D., Shiota, K., 2003. Effects of heat on embryos and fetuses. Proceedings of a WHO Workshop on Adverse Temperature Levels in the Human Body, Geneva, March 21–22, 2002. Int. J. Hyperther. 19 (3), 295–324.

Gaffey, C.T., Tenforde, T.S., 1979. Changes in the Electrocardiograms of Rats and Dogs Exposed to DC Magnetic Fields. Lawrence Berkeley Laboratory, LBL-9085. University of California, Berkeley, CA.

Gaffey, C.T., Tenforde, TS., 1981. Alterations in the rat electrocardiogram induced by stationary magnetic fields. Bioelectromagnetics 1, 357–370.

Gaffey, C.T., Tenforde, T.S., 1983. Bioelectric properties of frog sciatic nerves during exposure to stationary magnetic fields. Radiat. Environ. Biophys. 22, 61–73.

Gaffey, C.T., Tenforde, T.S., Dean, E.E., 1980. Alterations in the electrocardiograms of baboons exposed to DC magnetic fields. Bioelectromagnetics 1, 209.

Gmitrov, J., 1996. Static magnetic field effects on sinocarotid baroreceptors in humans. Electro. Magnetobiol. 15 (3), 183–189.

Gmitrov, J., Ohkubo, C., 2002a. Artificial static and geomagnetic field interrelated impact on cardiovascular regulation. Bioelectromagnetics 23, 329–338.

Gmitrov, J., Ohkubo, C., 2002b. Verapamil protective effect on natural and artificial magnetic field cardiovascular impact. Bioelectromagnetics 23, 531–541.

Gmitrov, J., Ohkubo, C., Okano, H., 2002. Effect of 0.25 T static field on microcirculation in rabbits. Bioelectromagnetics 23, 224–229.

Heinrichs, W.L., Fong, P., Flannery, M., Heinrichs, S.C., Crooks, L.E., Spindle, A., Pedersen, R.A., 1988. Mid-gestational exposure of BALB/c mice to magnetic resonance imaging. Magn. Reson. Imaging 6, 305–313.

High, W.B., Sikora, J., Ugurbil, K., Garwood, M., 2000. Subchronic in vivo effects of a high static magnetic field (9.4 T) in rats. J. Magn. Reson. Imaging 12, 122–139.

Hinch, R., Lindsay, K., Noble, D., Rosenburg, J.R., 2004. The effects of static magnetic field on action potential propagation and excitation in nerve. Prog. Biophys. Mol. Biol. doi:10.1016/j.pbiomolbio.2004.08.013.

Hong, C.-Z., 1987. Static magnetic field influence on human nerve function. Arch. Phys. Med. Rehabil. 68, 162–164.

Hong, C.-Z., Shellock, F.G., 1990. Short-term exposure to a 1.5 Tesla static magnetic field does not affect somato-sensory-evoked potentials in man. Magn. Reson. Imaging 8, 65–69.

Hong, C.-Z., Harmon, D., Yu, B., 1986. Static magnetic field influence on rat tail nerve function. Arch. Phys. Med. Rehabil. 67, 746–749.

Hong, C.-Z., Huestis, P., Thompson, R., Yu, J., 1988. Learning ability of young rats is unaffected by repeated exposure to a static electromagnetic field in early life. Bioelectromagnetics 9, 269–273.

Houpt, T.A., Pittman, D.W., Barranco, J.M., Brooks, E.H., Smith, J.C., 2003. Behavioural effects of high-strength static magnetic fields on rats. J. Neurosci. 23 (4), 1489–1505.

Ichioka, S., Iwasaka, M., Shibata, M., Nakatsuka, T., Harii, K., Kamiya, A., Ueno, S., 1998. Biological effects of static magnetic fields on the midrocirculatory blood flow in vivo: a preliminary report. Med. Biol. Eng. 36, 91–95.

Ichioka, S., Minegishi, M., Iwasaka, M., Shibata, M., Nakatsuka, T., Harii, K., Kamiya, A., Ueno, S., 2000. High-intensity static magnetic fields modulate skin microcirculation and skin temperature in vivo. Bioelectromagnetics 21, 183–188.

Ichioka, S., Minegishi, M., Iwasaka, M., Shibata, M., Nakatsuka, T., Ando, J., Ueno, S., 2003. Skin temperature changes induced by strong static magnetic field exposure. Bioelectromagnetics 24, 380–386.

ICNIRP, 1994. Guidelines on limits of exposure to static magnetic fields. Health Phys. 66 (1), 100–106.

ICNIRP, 1997. Biological effects of static and ELF electric and magnetic fields. In: Matthes, R., Bernhardt, J.H., Repacholi, M.H. (Eds.), Proceedings of the International Seminar on Biological Effects of Static and ELF Electric and magnetic Fields and Related Health Risks, Bologna, Italy, June 4 and 5, 1997. ICNIRP 4/97. Märkl-Druck, München.

ICNIRP, 2003. Exposure to static and low frequency electromagnetic fields. In: Matthes, R., McKinlay, A.F., Bernhardt, J.H., Vecchia, P., Veyret, B. (Eds.), Biological Effects and Health Consequences (0–100 kHz). ICNIRP 13/2003. Märkl-Druck, München.

International Agency for Research on Cancer (IARC), 2002. IARC Monographs on the Evaluation of Carcinogenic Risks to Humans. Non-Ionising Radiation. Part 1: Static and Extremely Low Frequency (ELF) Electric and Magnetic Fields, vol. 80. IARC, Lyon.

Kangarlu, A., Burgess, R.E., Zhu, H., Nakayama, T., Hamlin, R.L., Abduljalil, A.M., Robataille, P.M.L., 1999. Cognitive, cardiac, and physiological safety studies in ultra high field magnetic resonance imaging. Magn. Reson. Imaging 17, 1407–1416.

Konermann, G., Monig, H., 1986. Studies on the influence of static magnetic fields on prenatal development of mice. Radiologie 26, 490–497.

Kowalczuk, C.I., Sienkiewicz, Z.J., Saunders, R.D., 1991. Biological Effects of Exposure to Non-Ionising Electromagnetic Fields and Radiation. I. Static Electric and Magnetic Fields. NRPB-R238. NRPB, Chilton.

Kroeker, G., Parkinson, D., Vriend, J., Peeling, J., 1996. Neurochemical effects of static magnetic field exposure. Surg. Neurol. 45 (1), 62–66.

Levine, R.L., Bluni, T.D., 1994. Magnetic field effects on spatial discrimination in mice. Physiol. Behav. 55 (3), 465–467.

Levine, R.L., Dooley, J.K., Bluni, T.D., 1995. Magnetic field effects on spatial discrimination and melatonin levels in mice. Physiol. Behav. 58 (3), 535–537.

Lockwood, D.R., Kwon, B., Smith, J.C., Houpt, T.A., 2003. Behavioural effects of static high magnetic fields on unrestrained and restrained mice. Physiol. Behav. 78, 635–640.

McCann, J., Dietrich, F., Rafferty, C., Martin, A., 1993. A critical review of the genotoxic potential of electric and magnetic fields. Mutat. Res. 297, 61–95.

McKinlay, A.F., Allen, S.G., Cox, R., Dimbylow, P.J., Mann, S.M., Muirhead, C.R., Saunders, R.D., Sienkiewicz, Z.J., Stather, J.W., Wainwright, P.R., 2004. Review of the Scientific Evidence for Limiting Exposure to Electromagnetic Fields (0–300 GHz), vol. 15, no. 3. Documents of the NRPB, Chilton, UK.

Magin, R.L., Lee, J.K., Kintsova, A., Carnes, K.I., Dunn, F., 2000. Biological effects of long-duration, high field (4 T) MRI on growth and development in the mouse. J. Magn. Reson. Imaging 12, 140–149.

Mevissen, M., Butenkötter, S., Löscher, W., 1994. Effects of static and time-varying (50-Hz) magnetic fields on reproduction and fetal development in rats. Teratology 50, 229–237.

Mevissen, M., Stamm, A., Buntenkötter, S., Zwingleberg, R., Wahnschaffe, U., Löscher, W., 1993. Effects of magnetic fields on mammary tumour development induced by 7,12-dimethylbenz(a)anthracene in rats. Bioelectromagnetics 14, 131–143.

Murakami, J., Torii, Y., Masuda, K., 1992. Fetal development of mice following intrauterine exposure to a static magnetic field of 6.3 T. Magn. Reson. Imaging 10, 433–437.

Myakoshi, J., 2004. Effects of a static magnetic field at the cellular level. Prog. Biophys. Mol. Biol. doi:10.1016/j.pbiomolbio.2004.08.008.

Nakagawa, M., Matsuda, Y., 1988. A strong static-magnetic field alters operant responding by rats. Bioelectromagnetics 9, 25–37.

Narra, V.R., Howell, R.W., Goddu, S.M., Rao, D.V., 1996. Effects of a 1.5-Tesla static magnetic field on spermatogenesis and embryogenesis in mice. Invest. Radiol. 31 (9), 586–590.

Neurath, P.W., 1968. High gradient magnetic fields inhibits embryonic development of frogs. Nature 219, 1358–1359.

Nolte, C.M., Pittman, D.W., Kalevitch, B., Henderson, R., Smith, J.C., 1998. Magnetic field conditioned taste aversion in rats. Physiol. Behav. 63 (4), 683–688.

Okano, H., Ohkubo, C., 2001. Modulatory effects of static magnetic field on blood pressure in rabbits. Bioelectromagnetics 22, 408–418.

Okano, H., Ohkubo, C., 2003a. Effects of static magnetic fields on plasma levels of angiotensin II and aldosterone associated with arterial blood pressure in genetically hypertensive rats. Bioelectromagnetics 24, 403–412.

Okano, H., Ohkubo, C., 2003b. Anti-pressor effects of whole body exposure to static magnetic field on pharmacologically induced hypertension in conscious rabbits. Bioelectromagnetics 24, 139–147.

Okano, H., Gmitrov, J., Ohkubo, C., 1999. Biphasic effects of static magnetic fields on cutaneous microcirculation in rabbits. Bioelectromagnetics 20, 161–171.

Okazaki, R., Ootsuyama, A., Uchida, S., Norimura, T., 2001. Effects of a 4.7 T static magnetic field on fetal development in ICR Mice. J. Radiat. Res. (Tokyo) 42, 273–283.

Prato, F.S., Wills, J.M., Frappier, R.H., Drost, D.J., Lee, T.-Y., Shivers, R.R., Zabel, P., 1994. Blood–brain barrier permeability in rats is altered by exposure to magnetic fields associated with magnetic resonance imaging. Microsc. Res. Tech. 27, 528–534.

Reiter, R.J., 1993. Static and extremely low frequency electromagnetic field exposure: reported effects on the circadian production of melatonin. J. Cell Biochem. 51 (4), 394–403.

Repacholi, M.H., Greenebaum, B., 1999. Interaction of static and extremely low frequency electric and magnetic fields with living systems: health effects and research needs. Bioelectromagnetics 20 (3), 133–160.

Rosen, A.D., 1992. Magnetic field influences on acetylcholine release at the neuromuscular junction. Am. J. Physiol. 262, C1418–C1422.

Rosen, A.D., 1993. A proposed mechanism for the action of strong static magnetic fields on biomembrances. Int. J. Neurosci. 73, 115–119.

Rosen, A.D., 1994. Thresholds and limits of magnetic field action at the presynaptic membrane. Biochim. Biophys. Acta 1148, 317–320.

Rosen, A.D., 1996. Inhibition of calcium channel activation in GH3 cells by static magnetic fields. Biochim. Biophys. Acta 1282, 149–155.

Rosen, A.D., 2003a. Effect of a 125 mT static magnetic field on the kinetics of voltage activated Na^+ channels in GH3 cells. Bioelectromagnetics 24, 517–523.

Rosen, A.D., 2003b. Mechanism of action of moderate-intensity static magnetic fields on biological systems. Cell Biochem. Biophys. 39, 163–173.

Rosen, A.D., Lubowsky, J., 1987. Magnetic field influence on central nervous system function. Exp. Neurol. 95, 679–687.

Rosen, A.D., Lubowsky, J., 1990. Modification of spontaneous unit discharge in the lateral geniculate body by a magnetic field. Exp. Neurol. 108, 261–265.

Schenck, J.F., 2000. Safety of strong, static magnetic fields. J. Magn. Reson. Imaging 12, 2–19.

Schenck, J.F., 2004. Physical interactions of magnetic fields with living tissues. Prog. Biophys. Mol. Biol. doi:10.1016/j.pbiomolbio.2004.08.009.

Schneider, T., Thalau, H.P., Semm, P., 1994. Effects of light or different earth-strength magnetic fields on the nocturnal melatonin concentration in a migratory bird. Neurosci. Lett. 168 (1–2), 73–75.

Schwartz, J.-L., 1978. Influence of a constant magnetic field on nervous tissues: I. Nerve conduction velocity studies. IEEE Trans. Biomed. Eng. BME 25, 467–473.

Schwartz, J.-L., 1979. Influence of a constant magnetic field on nervous tissues: II. Voltage-clamp studies. IEEE Trans. Biomed. Eng. BME 26, 238–243.

Sikov, M.R., Mahlum, D.D., Montgomery, L.D., Decker, J.R., 1979. Development of mice after intrauterine exposure to direct-current magnetic fields. In: Phillips, R.D., Gillis, M.F., Kaune, W.T., Mahlum, D.D. (Eds.), Biological Effects of Extremely Low Frequency Electromagnetic Fields. 18th Hanford Life Sciences Symposium, Richland, WA, October, 1978. US Department of Energy, National Technical Information Service, Springfield, VA, pp. 462–473.

Snyder, D.J., Jahng, J.W., Smith, J.C., Houpt, T.A., 2000. c-Fos induction in visceral and vestibular nuclei of the rat brain stem by a 9.4 T magnetic field. NeuroReport 11, 2681–2685.

Sonnier, H., Kolomytkin, O.V., Marino, A.A., 2000. Resting potential of excitable neuroblastoma cells in weak magnetic fields. Cell. Mol. Life Sci. 57 (3), 514–520.

Sonnier, H., Kolomytkin, O., Marino, A.A., 2003. Action potentials from human neuroblastoma cells in magnetic fields. Neurosci. Lett. 337 (3), 163–166.

Suzuki, Y., Ikehata, M., Nakamura, K., Nishioka, M., Asanuma, K., Koana, T., Shimizu, H., 2001. Induction of micronuclei in mice exposed to static magnetic fields. Mutagenesis 16 (6), 499–501.

Tablado, L., Perez-Sanchez, F., Soler, C., 1996. Is sperm motility maturation affected by static magnetic fields? Environ. Health Perspect. 104 (11), 1212–1216.

Tablado, L., Perez-Sanchez, F., Nunez, J., Nunez, M., Soler, C., 1998. Effects of exposure to static magnetic fields on the morphology and morphometry of mouse epididymal sperm. Bioelectromagnetics 19 (6), 377–383.

Tablado, L., Soler, C., Nunez, M., Nunez, J., Perez-Sanchez, F., 2000. Development of mouse testis and epididymis following intrauterine exposure to a static magnetic field. Bioelectromagnetics 21 (1), 19–24.

Tenforde, T.S. Magnetically induced electric fields and currents in the circulatory system. Prog. Biophys. Mol. Biol.

Tenforde, T.S., Gaffey, C.T., Moyer, B.R., Budinger, T.F., 1983. Cardiovascular alterations in Macaca monkeys exposed to stationary magnetic fields: experimental observation and theoretical analysis. Bioelectromagnetics 4, 1–9.

Tenforde, T.S., Gaffey, C.T., Liburdy, R.P., Levy, L., 1985. Biological effects of magnetic fields. In: Biology and Medicine Division Annual Report 1985, Lawrence Berkeley Laboratories, LBL 20345. University of California, Berkeley, CA, p. 60.

Togawa, T., Okaim, O., Oshima, M., 1967. Observation of blood flow EMF in externally applied strong magnetic fields by surface electrodes. Med. Biol. Eng. 5, 169–170.

Trabulsi, R., Pawlowski, B., Wieraszko, A., 1996. The influence of steady magnetic fields on the mouse hippocampal evoked potentials in vitro. Brain Res. 728, 135–139.

Trzeciak, H.I., Grzesik, J., Bortel, M., Kuśka, R., Duda, D., Michnik, J., Malecki, A., 1993. Behavioral effects of long-term exposure to magnetic fields in rats. Bioelectromagnetics 14, 287–297.

Tyndall, D.A., 1990. MRI effects on the teratogenicity of x-irradiation in the C57BL/6J mouse. Magn. Reson. Imaging 8, 423–443.

Tyndall, D.A., 1993. MRI effects on cranio-facial size and crown-rump length in C57BL/6J mice in 1.5 T fields. Oral Surg. Oral Med. Oral Pathol. 76, 655–660.

Tyndall, D.A., Sulik, K.K., 1991. Effects of magnetic resonance imaging on eye development in the C57BL/6J mouse. Teratology 43, 263–275.

Ueno, S., Harada, K., Shiokawa, K., 1984. The embryonic development of frogs under strong DC magnetic fields. IEEE Trans. Magn. Fields MAG-20 (5), 1663–1665.

Ueno, S., Iwasaka, M., Shiokawa, K., 1994. Early embryonic development of frogs under intense magnetic fields up to 8 T. J. Appl. Phys. 75 (10), 7165–7167.

Weiss, J., Herrick, R.C., Taber, K.H., Contant, C., Plishker, G.A., 1992. Bio-effects of high magnetic fields: a study using a simple animal model. Magn. Reson. Imaging 10, 689–694.

WHO, 1987. Magnetic Fields, Environmental Health Criteria 69. World Health Organisation, Geneva.

Wieraszko, A., 2000. Dantrolene modulates the activity of steady magnetic fields on hippocampal evoked potentials in vitro. Bioelectromagnetics 21, 175–182.

Wikswo, J.P., Barach, J.P., 1980. An estimate of the steady magnetic field strength required to influence nerve conduction. IEEE Trans. Biomed. Eng. BME-27, 722–723.

Xu, S., Ohkubo, C., 1997. Acute effects of static magnetic fields on cutaneous microcirculation in rabbits. In Vivo 11, 221–226.

Xu, S., Okano, H., Ohkubo, C., 1998. Subchronic effects of static magnetic fields on cutaneous microcirculation in rabbits. In Vivo 12, 383–390.

Xu, S., Okano, H., Ohkubo, C., 2000. Acute effects of whole-body exposure to static magnetic fields and 50 Hz electromagnetic fields on muscle and microcirculation in anaesthetised mice. Bioelectrochemistry 53, 127–135.
