# Supplementary material for: Magnetic Fields and Cancer: Epidemiology, Cellular Biology, and Theranostics
Source: Int J Mol Sci. 2022 Jan 25;23(3):1339. doi: 10.3390/ijms23031339 (PMC8835851; doi:10.3390/ijms23031339)
Supplement: Supplementary file 1 [file ijms-23-01339-s001.zip › Supplementary Data Set S1/MF and Cancer.Data/PDF/0332452715/Zhou2018_Article_ReductionOfElectricFieldStren.pdf]

J. For. Res. (2018) 29(5):1415–1422
https://doi.org/10.1007/s11676-017-0541-1

ORIGINAL PAPER

Reduction of electric field strength by two species of trees under power transmission lines

Hongwei Zhou[1] · Liping Sun[1] · Yang Yang[1] · Che Liu[1] · Tianshi Liu[1] · Penghao Xie[2] · Ling Ma[1]

Received: 28 August 2016 / Accepted: 8 November 2016 / Published online: 15 November 2017
© Northeast Forestry University and Springer-Verlag GmbH Germany, part of Springer Nature 2017

Abstract Electrical pollution is a worldwide concern, because it is potentially harmful to human health. Trees not only play a significant role in moderating the climate, but also can be used as shields against electrical pollution. Shielding effects on the electric field strength under transmission lines by two tree species, *Populus alba* and *Larix gmelinii*, were examined in this study. The electrical resistivity at different heights of trees was measured using a PiCUS sonic tomograph, which can image the electrical impedance for trees. The electric field strength around the trees was measured with an elf field strength measurement system, HI-3604, and combined with tree resistivity to develop a model for calculating the electric field intensity around trees using the finite element method. In addition, the feasibility of the finite element method was confirmed by comparing the calculated results and experimental data. The results showed that the trees did reduce the electric field strength. The electric field intensity was reduced by 95.6%, and *P. alba* was better than *L. gmelinii* at shielding.

Keywords Trees · Power transmission lines · Electric environment · Tree electrical resistivity · Shielding · Finite element method

Project funding: This research was financially supported by the National Key Research and Development Program (2017YFD0600101), the Central University Basic Research and Operating Expenses of Special Funding (2572016CB04) and the Harbin Application Technology Research and Development Projects (2016RQQXJ134).

The online version is available at http://www.springerlink.com

Corresponding editor: Yu Lei.

✉ Hongwei Zhou
easyid@163.com

✉ Liping Sun
zdhslp@163.com

[1] Northeast Forestry University, Harbin 150040, People's Republic of China

[2] Department of Electrical Engineering, Tsinghua University, Beijing 100084, People's Republic of China

Introduction

As the Chinese economy and industrialization rapidly develops, urban consumption of electric it also increases rapidly. Unfortunately, the existing Chinese power grid structure cannot satisfy the power supply needed to sustain this growth, and more transmission systems are needed (Hu 2006). Recent research showed that the frequency of the electric field under transmission lines negatively influences people's nervous system and health (Farmer and Oman 1991; Greenland et al. 2000; Hakansson et al. 2005; Schuz and Ahlbom 2008; McNamee et al. 2009; Barth et al. 2010; Jong et al. 2012; Hasan et al. 2014; Costea et al. 2014; Elmas 2016).

The power grid usually crosses areas that are rich in vegetation or residential areas. Guo et al. (2014) investigated the impact of plants around 110 kV transmission lines on the strength of the power–frequency electric field and distribution of the magnetic field, finding that the plants distinctly contribute to reducing the power–frequency electric field strength; large plants in particular can weaken the electric field strength by more than 90%, enough to effectively reduce the harm caused by an electric field on human bodies. Hence, a deeper understanding of tree electrical properties and the influence of different tree species on electric fields should help prevent

electromagnetic radiation from reaching human bodies and serve as a reference for designing high voltage transmission lines through woods. Although many scholars in China and other countries have studied electromagnetic radiation under high voltage transmission lines, most of the studies have focused on buildings and human bodies, rarely on plants under the transmission lines.

Hagrey (2006) obtained a two-dimensional image of the distribution of resistivity in a trunk cross section, and Bao and Wang (2013), and Wang and Wang (2015) nondestructively measured tree resistivity. Sun et al. (2016) studied the influence of trees' dielectric constant on the power–frequency electric field strength.

Wu et al. (2011) indicated that trees under transmission lines played a part in reducing the electric field strength. Guo et al. (2014) analyzed the power–frequency electric field and the magnetic field of four sites with the same size area under 110 kV transmission lines, including an area without vegetation, a lawn, an area with shrubs, and an area with tall trees, and concluded that the trees weakened the electric field strength below the lines. Ismail and Alkandari (2009) used a charge simulation method to explore the effect of tree height and equivalent radius on electric field shielding of the transmission line, but the conclusions of these studies were based on the actual measurements or calculation methods, that lacked a simulation model based on actual measurements of tree resistivity and dielectric constant. Therefore, the electric field strength of a certain point around trees could not be obtained, nor could the maximum electric field strength be located or measured. We used field measurements and a finite element method to analyze electric field intensity in a no-tree-area and tree-area under the transmission lines for a coniferous and a broad-leaf trees. The relationship between electric field strength and distance between trees and transmission lines is also discussed. The trees were found to weaken the effect of trees on the electric field and demonstrates the rationality of using the finite element method to study the effect of trees on electric field strength under high-voltage transmission lines.

Materials and methods

Tree resistivity

Larix gmelinii is one of the most widespread species in northeastern China. *Populus alba* is commonly used in horticulture, wood crafts and shelter forests in China. *L. gmelinii* and *P. alba* growing under high voltage transmission lines in the forest farm of Northeast Forestry University were selected as the experimental material for outdoor measurement.

A PiCUS (argus electronic gmbh, Rostock, Germany) electrical resistance tomography imager was used to measure resistivity. The imager is an electrical impedance testing instrument based on electrical resistance tomography imaging (ERT) technology.

ERT technology uses current as the stimulus and voltage as the measured variable. The measured stumpage was connected to an alternating current through a pair of electrodes, resulted in the changes of resistivity distribution within the stumpage, and the voltage on the stumpage periphery reflected the resistance changes would change as well. According to the measured voltage of stumpage periphery, using imaging algorithm, a 2-D resistivity distribution image of stumpage section can be obtained. ERT technology as a kind of electrical resistance tomography imaging (EIT) is widely used (Liu et al. 2014) to monitor plant tuber and root growth (Amato et al. 2009; Dong 2015) and test trees nondestructively (Bao and Wang 2013). In the experiment, ERT technology was applied to measure resistivity of trees (Fig. 1a).

The test tree trunk was divided into 12 equal parts along the transverse direction, and electrodes were nailed into 12 equipartition points. Along the vertical direction, 2 m of trunk was chosen and divided into 5 equal parts. So the distance between each measuring point was 0.4 m. The electrode was a stainless steel nail about 0.05 m long. To ensure that the stainless steel nail penetrated the bark and reached the wood, 2/3 of the nail was hammered into the trunk. Sensors were installed on every electrode. For avoiding interference from experiment instrument and ensuring accuracy of the results, sensors were installed sequentially.

An HI-3604 power–frequency electromagnetic field measuring instrument (HOLADAYUSA) was used to test the surrounding electromagnetic field strength of the 50/60 Hz power lines, electric equipment and facility, and video display terminal. Using HI-3604 is convenient and intuitive for determining the position of electromagnetic field source and the strongest point of radiation. The single probe at full measuring range can directly obtain the high voltage transmission line field strength around the trees, which is suitable for field measurements (Fig. 1b).

In the experiment, ground wire in the forest farm was chosen as the starting point, then measurements of the electric field strength were taken 10 m from the trees, 1.5 m above the ground in an area without trees and in the area with *P. alba*. This process was then repeated in the opposite direction, and the mean of the two measurements were calculated.

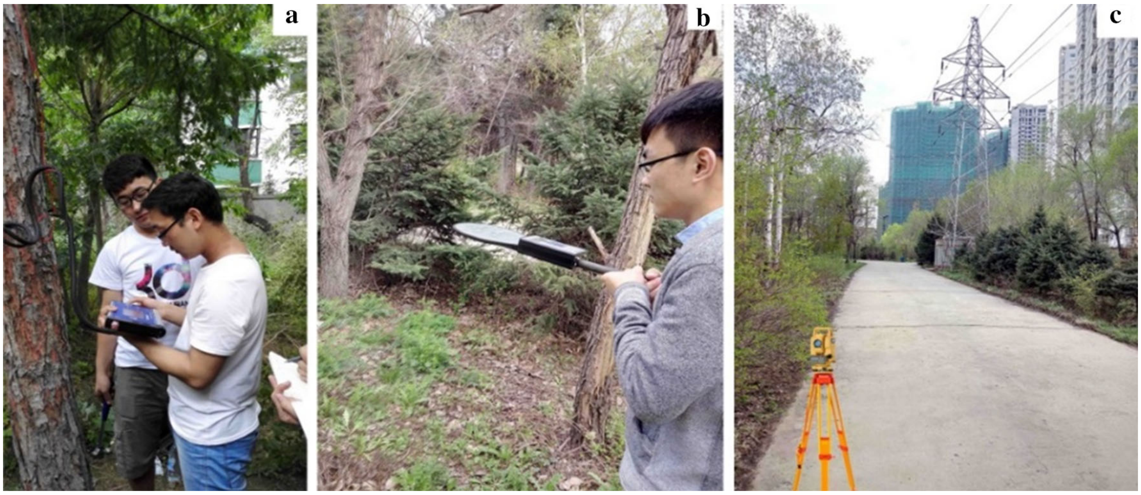

Fig. 1 Measuring resistivity of trees (**a**) and the space electric field around the trees (**b**) during the experiment and total station and transmission tower (**c**)

Transmission tower and tree variables

A total station (BTS-802E, boifgeo, China) and a measuring tape were used to measure the height of the high voltage transmission line, and the relative coordinates between the trees and the subpoints of the power lines on the ground and the two transmission towers. The total station is a surveying and mapping system that uses an electronic transit the odolite with a distance meter and software to determine horizontal and vertical angles, distance (slope distance, horizontal distance), and elevation differences with an accuracy to meet the demands of the experiment.

In the actual measurement process, the total station (Fig. 1c) and tape were utilized to measure the distance between the transmission towers and trees and the elevation angle, dip angle and horizontal angle. The relative position coordinates for the towers and trees can then be calculated using trigonometric functions.

The relative coordinates of trees and power lines: In (Fig. 2), O is the center point of tower 1 base, which is the origin point. P is the center point of tower 2 base. T1 is the coordinate of tree1 (*L. gmelinii*). T2 is the coordinate of tree 2 (*P. alba*). Figure 3 shows the coordinates of each hanging line.

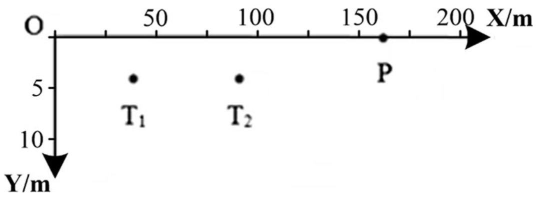

Fig. 2 Relative position of trees and transmission lines. The point T1 and T2 are trees, the point P is the observer

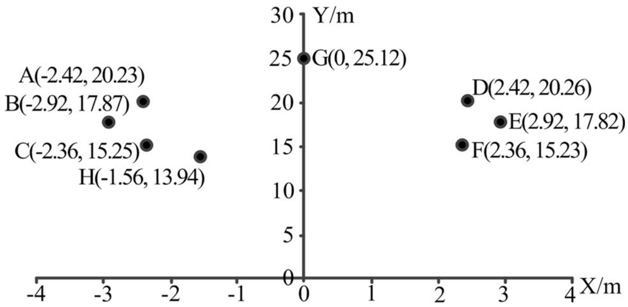

Fig. 3 Cross section of transmission lines. According to Fig. 1, the actual transmission line is measured to obtain the coordinates of each transmission line A–H

Electric field strength simulation

Finite element method

The finite element method is a highly efficient and commonly used numeral technique to solve a functional extremum problem instead of solving the given Poisson equation. This method was initially introduced with the development of variation principle, so it is used extensively in different physical fields described by Laplace's equation and Poisson equation. In 1965, A.M. Winslow was the first to apply the finite element method to solve electrical engineering problems. In 1969, P.P. Silvester used it to calculate the steady state solution of time-varying field.

The finite element, which is highly optimized for general use, has a high calculation accuracy and the ability to analyze and solve complex problems. The method is also widely accepted for 3-dimensional electromagnetic field analysis (Liao et al. 2004; Yamada et al. 2004; Titus 2002). Because the electric field around trees under power–frequency transmission lines is an electrostatic field, the

problem can be converted into a boundary integral equation of boundary value problem, then simulated using finite element method simulation (Lin et al. 2011; Simpson et al. 2012; Ren et al. 2014).

Setting parameters and modeling

Because the conductivity and the dielectric property of tree is between conductor and insulator, the relative analysis of a poor conductor such as construction, in the identical experimental condition can be taken as a reference to study the impact of trees on electric field strength.

The transmission lines are double circuit high voltage; the voltage grade is 63 kV. Coordinates of the cross section of each transmission line is calculated using data measured by the total station. Figure 3 shows a schematic diagram, where A–F are the coordinates of the six double circuit line cross sections, G is the coordinate of the ground wire cross section, and H is the coordinate of shielded wire cross section.

The distribution of transmission lines and the size of the tree model is determined by the measured data of full station. Resistivity of the trunk is the actual measured value, and the resistivity of the canopy is the average of the measured values at 2 m above the ground. Data from the literature (Sun et al. 2016) was applied in the analysis, where the dielectric constant of tree, air and earth is 9, 1 and 10, respectively, and the resistivity of earth is 10^4 Ω·m (Tian 2013). The models built by ANSYS MAXWELL 2015 are shown in Figs. 4 and 5.

Results

Trees resistivity

The two-dimensional resistivity distribution from different heights (0 to 2 m) of the trees of *L. gmelinii* and *P. alba* was obtained using the PiCUS tree electrical resistance tomography imager (Fig. 6).

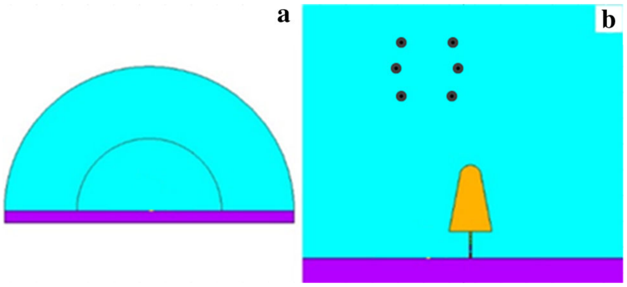

Fig. 4 Whole model (**a**) and the relative position of transmission line and tree (**b**). Purple represents the earth, and blue is the air, the black dot represents the power line, and the yellow represents the tree

The weighted average is computed by proportion of different colors in the two-dimensional diagram. Three different colors, red, yellow and blue, are distinguished from the diagram with image processing software. Then, the different color areas were separate, and the acreage of each area was calculated. Finally, the weighted average as model parameters used in finite element analysis based on the acreage acquired above was calculated. The average resistivity of the two species at different height is shown in Table 1.

Electric field strength under transmission lines

Using the methods mentioned above, we measured the electric field strength at 1.5 m above the ground in the no-tree area and the tree-area with the HI-3604 power–frequency electromagnetic measuring instrument; the line chart is shown in Fig. 7. The electric field strength was reduced 95.6% by the trees. The line chart can also be compared with the simulation curve to demonstrate the usefulness of the finite element method.

Parameters of transmission towers and trees

The computational results calculated using the data obtained above provided the values for the model simulation using the finite element method. Heights of transmission towers and trees areas are shown in Table 2, and the coordinates of the hanging lines are shown in Fig. 3. The relative coordinates between transmission lines and trees are shown in Table 3.

Simulation results

Finite element analysis software, ANSYS MAXWELL 2015, was used to build the computational model. Parameters in the simulation were set using the results calculated above. After the analysis and calculation, a path in the general post-process was defined as the same path used in the actual measuring process, i.e., the projection of the lightening protection wire was used as the starting point, with the path 0–10 m toward the tree direction; the curve graph of the electric field strength on the path was then simulated. The curve graph well-matched the graph plot by the actual data verifying the validity of the finite element method. The electric field distribution around trees is shown in Fig. 8. The curve of the electric field strength at 1.5 m from the ground is shown in Fig. 9.

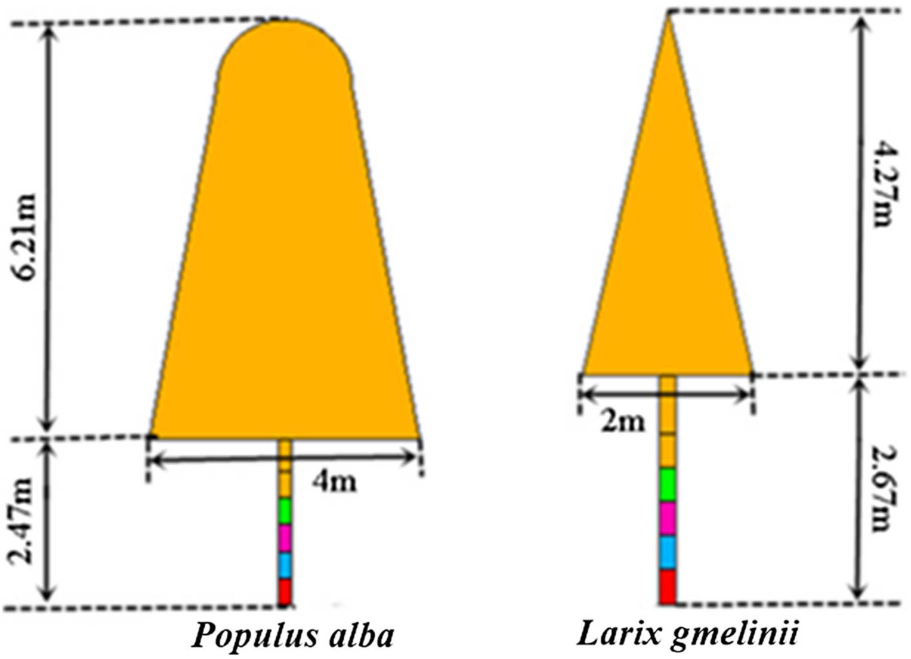

Fig. 5 Models of *Larix gmelinii* and *Populus alba* under high voltage transmission lines

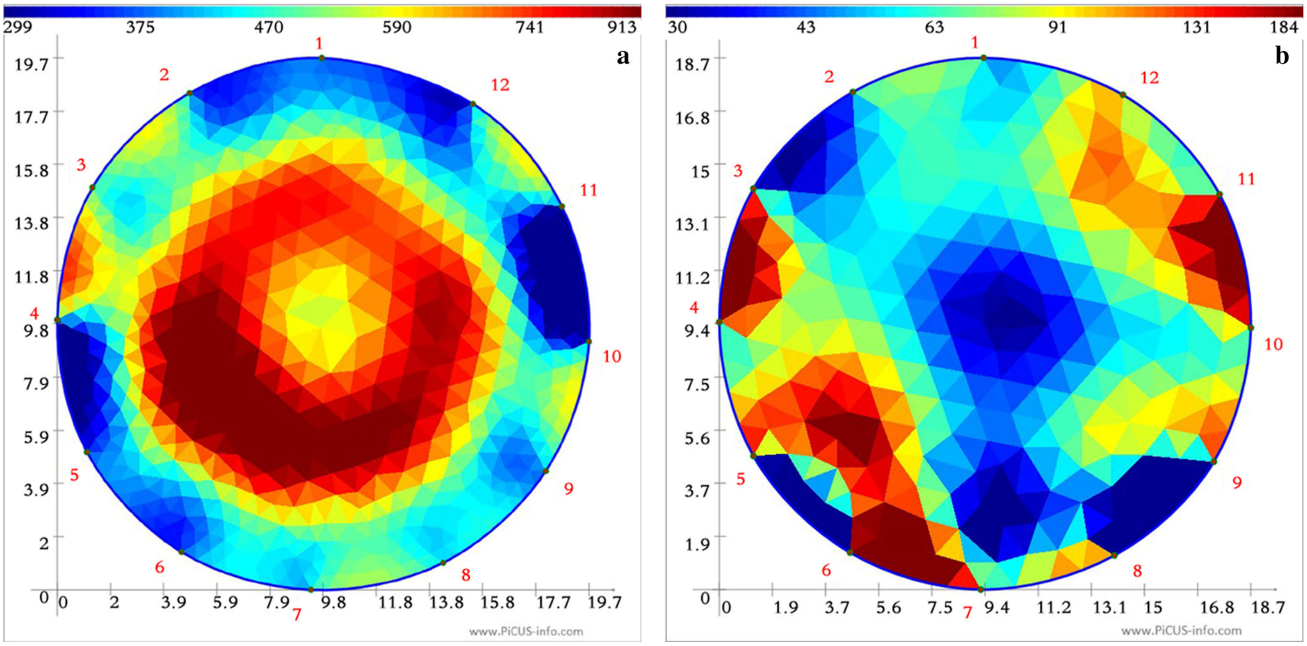

Fig. 6 Resistivity distribution for *Larix gmelinii* (**a**) and *Populus alba* (**b**) at 80 cm from the ground

Discussion and analysis

The resistivity values for *L. gmelinii* and *P. alba* showed that they differed in their resistivity and that the resistivity of the respective species differed depending on the height above the ground. Generally, the resistivity of the trunk decreased with an increase in height, consistent with the findings of Wang (2005) and Chen et al. (2010). Such differences can be due to many reasons, but the moisture content is one of the most significant factors (Hagrey 2006). The apparent rise in resistivity for *P. alba* at 0.8 m above the ground is probably due to an internal defect of the trunk (Wang and Wang 2015).

Field strength changes indicate that trees have a positive impact on the reduction of electric field strength, and the electric field strength was weakened by 95.6% at 1.5 m above the ground. Regarding the projection of the ground wire as 0 m, at 1.5 m above the ground in the area of

Table 1 Average resistivity of *Larix gmelinii* and *Populus alba* trunk at different heights

Height (m)	*Larix gmelinii* Resistivity (Ω·m)	*Populus alba* Resistivity (Ω·m)
0.4	701.3	467.13
0.8	597.64	1576.7
1.2	484.29	433.68
1.6	293.06	134.07
2	246.17	84.94

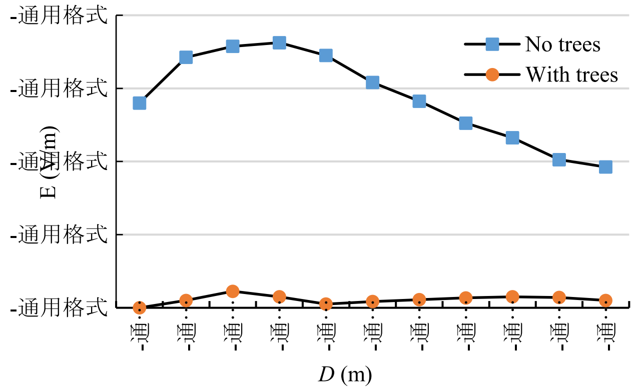

Fig. 7 Electric field strength below the transmission lines in the area with no trees and with trees

0–10 m, the field strength of the 63 kV double circuit transmission lines first increased, then declined.

The contrast between the actual measured curve and the simulation curve demonstrated that the two tree species differed in their ability to reduce the electric field strength under the high voltage transmission lines, and *P. alba* was better. In the no-tree area, the electric field strength was higher closer to the lines then decreased farther away, matching the simulated trend. From 0 to 4 m from the lines, on account of the tree shield effect, the field strength is low. From 4 to 4.2 m, the measurement path crossed through the tree, so the value equals to the electric field strength inside the trunk, which is 0. From 4.2 to 10 m, as the distance increases, the reduction lessens, but is still better than in areas without trees as predicted by the simulation. The change in the electric field strength of *L. gmelinii* and *P. alba* was similar. Due to the influence of environment factors, such as the surrounding trees, temperature, and moisture, the simulation model differed from the actual values, but the error is tolerant. Resistivity and permittivity are important variables that reflect electrical characteristics of trees, and the simulation model proved the feasibility of using the finite element method to study the effect of trees on electric field strength under high voltage transmission lines.

Table 2 Heights of trees and towers

Project	Tower 1	Tower 2	Total height of tree 1	Height of tree trunk 1	Total height of tree 2	Height of tree trunk 2
H (m)	25.00	25.00	6.94	2.67	8.68	2.47

Table 3 Relative coordinates of trees and towers

Point	O (Tower1)	P (Tower 2)	T1 (tree 1)	T2 (tree 2)
Coordinates (m)	(0, 0)	(156.154, 0)	(44.030, 4.082)	(94.547, 4.046)

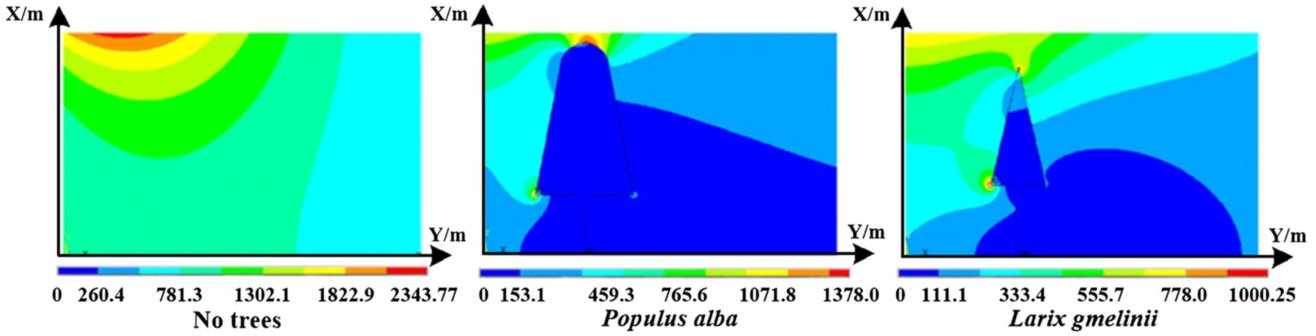

Fig. 8 Electric field distribution in the area without trees and around trees of each species. The color scale below the map shows the field strength ($V \cdot m^{-1}$)

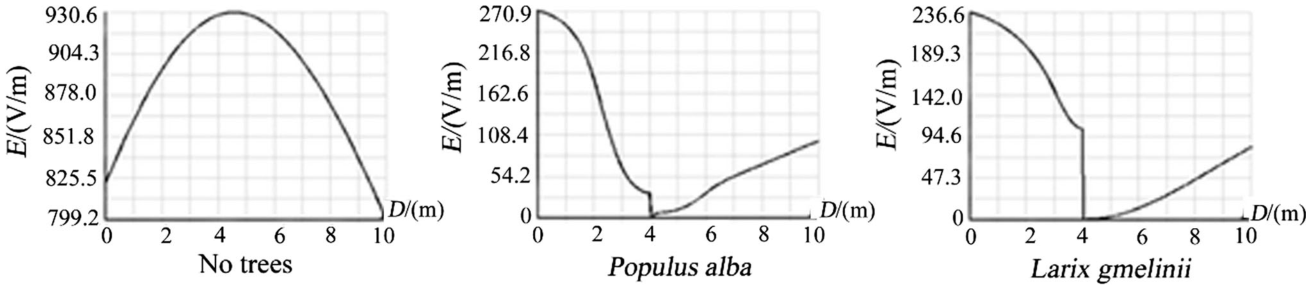

Fig. 9 Electric field strength at 1.5 m above the ground in the area without trees and the tree of each species under the transmission lines

Conclusion

Finite element method is suitable for analyzing and predicting the reductive impact of trees on the electric field strength under high voltage transmission lines.

The resistivity differed between the two species differs and decreased with increasing height of the trunk for each species.

With the ground wire set as 0 m, on 1.5 m above the ground at 0–10 m from the 63 kV double circuit transmission lines, the field strength first is increased, then declined.

The simulation results using finite element analysis and the actual values were similar enough to support the feasibility of using this method to study the effect of trees on electric field strength under high voltage transmission lines. According to the simulation, the electric field intensity was reduced by 95.6% at 1.5 m above the ground by trees, and *P. alba* was better than *L. gmelinii* at reducing the intensity.

Hence, planting a large arbor, such as *P. alba*, in transmission line corridors and residential areas can effectively reduce the electric field strength under transmission lines and prevent any harm caused by a power–frequency electric field. Planting large trees will also beautify and improve the environment, and promote harmony between socioeconomic development and environmental protection.

References

Amato M, Bitella G, Rossi R et al (2009) Multi-electrode 3D resistivity imaging of alfalfa root zone. Eur J Agron 31(4):213–222

Bao ZY, Wang LH (2013) Application of electrical resistance testing method in detection of decay in standing trees. For Eng 29(6):47–51 **(in Chinese)**

Barth A, Ponocny I, Ponocny-Seliger E et al (2010) Effects of extremely low-frequency magnetic field exposure on cognitive functions: results of a meta-analysis. Bioelec-tromagnetics 31:173–179

Chen CX, Yu KY, Cao ZN, Zhu YH (2010) Moisture content of tree stem of *Cyclobalanopsis myrsinaefolia* (Bl.). Oerst Nat For 30(12):39, 41–46 **(in Chinese)**

Costea M, Golovanov N, Grintescu IM (2014) Human exposure to electromagnetic fields produced by distribution electric power installations. Adv Electr Comput Eng 14(1):29–36

Dong Z (2015) Research on excitation/measurement strategy of tuber electrical resistance tomography monitoring system. Beijing Jiaotong University, Beijing, pp 9–10 **(in Chinese)**

Elmas O (2016) Effects of electromagnetic field exposure on the heart: a systematic review. Toxicol Ind Health 32(1):76–82

Farmer LE, Oman H (1991) Effects of electric and magnetic fields on people and animals. IEEE Aerosp Electron Syst Mag 6(9):26–29

Greenland S, Sheppard AR, Kaune WT et al (2000) A pooled analysis of magnetic fields, wire codes, and child-hood leukemia. Epidemiology 11:624–634

Guo JF, Wang D, Huang H, Shi JS, Zhang JF, Yang YQ (2014) Measurement and analysis of plants impact on 50 Hz EMFs of 110 kV overhead power transmission lines. Shenzhen Environ Monit Cent 05:397–399 **(in Chinese)**

Hagrey A (2006) Electrical resistivity imaging of tree trunks. Near Surf Geophys 4(12):179–187

Hakansson N, Stenlundc C, Gustavsson P et al (2005) Arc and resistance welding and tumours of the endocrine glands: a Swedish case–control study with focus on extremely low frequency magnetic fields. Occupy Environ Med 62(5):304–308

Hasan GM, Sheikh IA, Karim S et al (2014) Effect of electromagnetic radiations on neurodegenerative diseases-technological revolution as a curse in disguise. CNS Neurol Disord Drug Targets 13(8):1406–1412

Hu BX (2006) Research on electromagnetic environment of power frequency EHV and UHV transmission lines. Zhejiang University, Hangzhou, pp 3–4 **(in Chinese)**

Ismail HM, Alkandari AM (2009) Impact of using natural trees on the electric field reduction of Kuwait high voltage transmission systems. Kuwait J Sci Eng 36(1):131–145

Jong AD, Wardekker JA, Sluijs JPVD (2012) Assumptions in quantitative analyses of health risks of overhead power lines. Environ Sci Policy 16(7):114–121

Liao MF, Duan XY, Sun H (2004) 3D electric field calculation of vacuum circuit breakers with multiple breaks using the ANSYS finite element program. IEEE Conf Publ 2:601–605

Lin YJ, Mukherjee S, Nishimura N et al (2011) Recent advances and emerging applications of the boundary element method. Appl Mech Rev 64(3):1–38

Liu QF, Han Y, Zhang XX (2014) An image reconstruction algorithm based on Bayesian theorem for electrical resistance tomography. Opt Int J Light Electron Opt 125(20):6090–6097

McNamee DA, LEgros AG, Krewski DR et al (2009) A literature review: the cardiovascular effects of exposure to extremely low frequency electromagnetic fields. Int Arch Occup Environ Health 82:919–933

Ren Z, Kalscheuer T, Greenhalgh S et al (2014) A hybrid boundary element-finite element approach to modeling plane wave 3D electromagnetic induction responses in the Earth. J Comput Phys 258:705–717

Schuz J, Ahlbom A (2008) Exposure to electromagnetic fields and the risk childhood leukemia. Radiat Prot Dosim 132(2):202–211

Simpson RN, Bordas SPA, Trevelyan J et al (2012) A two-dimensional isodiametric boundary element method for electrostatic analysis. Comput Methods Appl Mech Eng 209:87–100

Sun LP, He J, Zhou HW, Du LJ (2016) Dielectric constant measurements of trees and the impact on the electric field. J Northwest For Univ 31(1):76–80 **(in Chinese)**

Tian ZS (2013) Calculation of power frequency electric field from AC overhead transmission lines and induced current in human body. Chongqing University, Chongqing, pp 27–49 **(in Chinese)**

Titus PH (2002) Disruption load calculations using ANSYS transient electromagnetic simulations for the allocator C-Mod antennas. In: Symposium on fusion engineering, pp 27–30

Wang LJ (2005) Study on evaluating wood properties of poplar plantation using non-destructive testing methods. Beijing Forestry University, Beijing, pp 26–33 **(in Chinese)**

Wang XL, Wang LH (2015) Effects of electrode quantity on log defect test based on electrical resistance tomography. China For Sci Technol 6:99–103 **(in Chinese)**

Wu GQ, Cai WB, Liu ZH, Wang ZZ (2011) Research of electric field for high voltage transmission lines over buildings 27(06):46–48 **(in Chinese)**

Yamada R, Marcsin E, Lee A et al (2004) 3D ANSYS quench simulation of cosine theta Nb3Sn high field dipole magnets. IEEE Trans Appl Supercond 14(2):291–294
