# Supplementary material for: Magnetic Fields and Cancer: Epidemiology, Cellular Biology, and Theranostics
Source: Int J Mol Sci. 2022 Jan 25;23(3):1339. doi: 10.3390/ijms23031339 (PMC8835851; doi:10.3390/ijms23031339)
Supplement: Supplementary file 1 [file ijms-23-01339-s001.zip › Supplementary Data Set S1/MF and Cancer.Data/PDF/0375241306/risa.12551.pdf]

Risk Analysis, Vol. 36, No. 6, 2016 DOI: 10.1111/risa.12551

Health-Economics Analyses Applied to ELF Electric and Magnetic Fields

Shaiela Kandel,[1,*] John Swanson,[2] and Leeka Kheifets[3]

Extremely low frequency electric and magnetic fields (ELF EMFs) are a common exposure for modern populations. The prevailing public-health protection paradigm is that quantitative exposure limits are based on the established acute effects, whereas the possible chronic effects are considered too uncertain for quantitative limits, but might justify precautionary measures. The choice of precautionary measures can be informed by a health-economics analysis (HEA). We consider four such analyses of precautionary measures that have been conducted at a national or state level in California, the Netherlands, the United Kingdom, and Israel. We describe the context of each analysis, examine how they deal with some of the more significant issues that arise, and present a comparison of the input parameters and assumptions used. The four HEAs are methodologically similar. The most significant qualitative choices that have to be made are what dose-response relationship to assume, what allowance if any to make for uncertainty, and, for a CBA only, what diseases to consider, and all four analyses made similar choices. These analyses suggest that, on the assumptions made, there are some low-cost measures, such as rephasing, that can be applied to transmission in some circumstances and that can be justifiable in cost-benefit terms, but that higher cost measures, such as undergrounding, become unjustifiable. Of the four HEAs, those in the United Kingdom and Israel were influential in determining the country's EMF policy. In California and Netherlands, the HEA may well have informed the debate, but the policy chosen did not stem directly from the HEA.

KEY WORDS: Cost-benefit analysis; ELF EMF policy; health-economics analysis; magnetic fields

1. INTRODUCTION

Extremely low frequency electric and magnetic fields (ELF EMFs) are a common exposure for modern populations, who have been exposed to these fields since the late 19th century with the advent of public electricity supplies. EMFs have well-established acute effects on the human body (principally the induction of electric fields in tissues) at levels at the upper end of those encountered in the environment. There are also suggestions of chronic effects (principally carcinogenesis) at much lower levels of magnetic fields, encountered more commonly in the environment. The prevailing public-health protection paradigm is that quantitative exposure limits are based on the established acute effects, whereas the possible chronic effects are considered too uncertain for quantitative limits, but might be sufficient for precautionary measures. To our knowledge as of 2006, 19 countries had adopted limits aimed mainly at preventing acute effects, and 14 had adopted some form of precautionary measures.[(1)]

It is important to acknowledge that there is uncertainty whether exposure to low levels of magnetic

[1]Independent, Jerusalem, Israel.
[2]National Grid plc, London, UK.
[3]Department of Epidemiology, UCLA School of Public Health, Los Angeles, CA, USA.
*Address correspondence to Shaiela Kandel, Independent, Jerusalem, Israel; fax: +972-77-5498873; shaielak@gmail.com.

 0272-4332/16/0100-1277$22.00/1 © 2016 Society for Risk Analysis

fields actually causes chronic effects. Even if it does, the relevant metric of exposure that is pertinent to public health is unknown; therefore, there is uncertainty as to what aspect of exposure to reduce. Long-term (24 hour or more) time-weighted average magnetic field exposure in the home has been extensively used in epidemiological studies, but it is likely to be just a proxy for a biologically relevant exposure (if any). It is usually assumed that reducing one aspect of exposure will also reduce any biologically relevant aspect, but this is just an assumption.

In deciding what level of protection to adopt, for EMFs or for any other agent, a common tool used is a health-economics analysis (HEA). In this article, we examine four instances where a formal HEA for ELF EMFs has been conducted at a national or state level. We first describe the circumstances where each of the analyses was performed. We then examine how the different analyses deal with some of the more significant issues that arise. Leading on from this, we present a comparison of the input parameters and assumptions used in each analysis and hence the conclusions reached. Finally, we consider the implications of the conclusions reached for ELF EMF policy.

2. TYPES OF HEALTH-ECONOMICS ANALYSIS

Since 1974, the use of Regulator Impact Assessment (RIA) has become widespread among OECD member countries, and in 2008 the number of OECD countries that require RIA of new regulatory proposals had grown to 26 out of 30 member states.(2) In RIA, the potential economic impacts of regulatory proposals are assessed, therefore encompassing some form of HEA.

All HEAs have a common goal of comparing the merits of alternative policies or interventions in a quantitative way. This inherently involves attempting to express the various relevant parameters on a common scale. The parameters related to costs are usually compared on a scale of monetary cost, and although there are numerous detailed difficulties in achieving this, the concept is straightforward. But there are different degrees of attempting to express benefits on a common scale, and this results in different types of analysis.

In cost-utility analysis, the benefits are measured in terms of reduced exposure, with no attempt to link this to any resulting health benefit. In EMF terms, therefore, a cost-utility analysis might compare the monetary cost per microtesla reduction of field. In cost-effectiveness analysis, the costs of achieving a certain health benefit are compared, for example, the cost per potential life saved. In cost-benefit analysis, the health benefit is expressed in the same units, usually monetary, as the costs. Cost-effectiveness analysis allows alternative approaches to achieving a given health benefit to be compared; cost-benefit analysis additionally allows a quantitative judgment to be made about the advisability of each measure in its own right. Community impact analysis,(3) multiattribute analysis, (4) and multicriteria decision analysis go beyond a single health-economics calculation, looking in broader terms as the entire distribution of incomes and outcomes of the intervention.

Of the formal HEAs, one, from the Netherlands, is a cost-utility analysis, the others are cost-benefit analyses.

3. IDENTIFIED COST-BENEFIT ANALYSES

We identified HEAs principally by a questionnaire distributed in 2006 to 47 countries, involved in the International EMF Project of WHO plus two additional countries (Romania and Luxembourg) that asked to be included. We assume that those countries concerned enough about EMFs to have invested the effort required to perform a HEA are those also interested enough in EMFs to have participated in the WHO project. Forty-three responses were received. Sixteen indicated they used some form of HEA in order to decide on an appropriate ELF policy. Of these, only three countries used a formal HEA (Australia, Germany, and the United Kingdom), with specified numerical values for the various parameters, 13 an informal HEA (Brazil, Bulgaria, China, Greece, Israel, the Netherlands, New Zealand, Norway, Russia, Singapore, Sweden, Turkey, and the United States), 18 stated that this framework was not used, and the remainder did not respond. We decided not to include the analysis from Australia,(5) which evaluated different approaches to implementing exposure guidelines for protecting against acute effects, rather than precautionary measures directed at possible chronic effects. In addition, the German HEA was not publicly available.

We also conducted a literature search that identified only a few old cost-benefit analysis that were based on the old scientific evidence and lacked sufficient detail; thus they were not considered further.(6,7) We identified one additional analysis from California.

Thus in the remainder of this article we focus on four HEAs that evaluated options for reducing EMF exposures (California, the Netherlands, the United Kingdom, and Israel). We convert all financial values to US$, using exchange rates of 1$ = 0.5£ = 0.8E (the average values, to one decimal place, for 2007 and 2004, the years of publication of the HEAs from the United Kingdom and the Netherlands, respectively).

3.1. California

3.1.1. Policy Context

The Public Utilities Commission for the State of California created a no- or low-cost policy for the construction of new power lines in 1993, which requires up to 4% of the cost of a project to be spent, provided field reduction of more than 15% can be achieved. Additionally, the Department of Education has a policy requiring schools to be set back specified distances from the edge of the right of way.(8)

3.1.2. Summary of Analysis

In 1993 the California Public Utilities Commission (CPUC) directed utilities to provide funds for research, policy analysis, and public education concerning EMFs. In addition to several research projects and risk assessment, a computer implementation of a cost-benefit analysis was developed, which we refer to here as the "framework."(9) The results were principally presented as a decision framework to allow users to see the effect on the outcomes of their own preferences for the various input parameters.(10) This analysis was never formally adopted, but informed a decision to retain the existing policy. As part of this effort, a "model" set of values to insert into cost-benefit analysis was presented by the California EMF Program,(11) which we use here and refer to as the "model," but we emphasize that the California analysis is not prescriptive of this single set of values and analysis.

3.2. The Netherlands

3.2.1. Policy Context

In the Netherlands there are no legally binding standards for ELF EMFs. The ICNIRP 1998 guidelines were recommended by the Dutch Health Council and used in practice, until in 2005, the State Secretary of Public Housing, Spatial Planning and the Environment recommended to local authorities increasing the distance to new power facilities from residential neighborhoods so that exposure to children will not exceed 0.4 μT. No change to existing facilities was recommended. (12)

3.2.2. Summary of Analysis

In the lead up to the recommendation, the Dutch government commissioned research into the costs and benefits of different measures to reduce the population's exposure to magnetic fields from existing power lines.(13)

3.3. Israel

3.3.1. Policy Context

In Israel as of 2014 there are no legally binding guidelines to ELF EMFs. The ICNIRP 1998 guidelines (100 μT) were implemented de facto until 2002. A precautionary policy of the Ministry of the Environment (MoE) was effective from 2002 until 2005, which recommended that newly constructed facilities should not exceed exposure levels of 1 μT (time-weighted average). The MoE updated its policy following the publication of the Final Report of Israeli ELF Expert Committee in March 2005.(14) The conclusion of the report was that the Israeli guidelines should be those recommended by ICNIRP 1998 (100 μT) but that precautionary measures, such as reduction of exposure levels to as low as possible, should be considered when residential exposure levels to magnetic fields were in the range from 0.4 to 100 μT. The report emphasized that precautionary measures should be implemented only at low or minimal cost, that an annual national budget be set aside for EMF field reduction, and that a national committee (established in 2007) would decide how this budget would be distributed in order to be cost effective. This Committee issued an intermediate report in 2011 (not publicly available) reaffirming that for new installations, power cancellation design should be used, and implemented for existing installations whenever possible and practicable. The Committee also recommended that for existing situations the annual-average magnetic field exposure should be gradually reduced to "a few milligauss" (namely, up to 0.1 μT) by using public funds, prioritized according to a multiattribute goal function developed by the Committee.

3.3.2. Summary of Analysis

A CBA was performed by the MoE in the early stages of the potential mitigation option assessments

mainly based on data from certain countries around the world, on environmental impact assessments submitted to the MoE, and on some assessments performed by the MoE. These drew mainly on the California analysis. Costs used in this analysis differed vastly from estimates provided by the government-run utility company. This difference was never resolved.

3.4. The United Kingdom

3.4.1. Policy Context

Until 2004 the United Kingdom followed guidelines created by its own National Radiological Protection Board (NRPB). In 2004 it adopted ICNIRP 1998 in the terms of the E.U. Recommendation but also decided to look at possible additional precautionary measures. This was done through a stakeholder group "SAGE" managed by the Department of Health. Its aim was to provide practical advice to government in making decisions about policy related to EMFs and health. It brought together around 40 key stakeholders from national government departments, regulators and advisory bodies, academics, individuals, local and national campaign groups, professional bodies, and industry. SAGE published its first assessment, covering high-voltage power lines and also house wiring and domestic equipment, in April 2007,[15] and government responded in October 2009, adopting optimal phasing for high-voltage power lines but rejecting more expensive measures.[16]

3.4.2. Summary of Analysis

SAGE's recommendations were informed by a detailed cost-benefit analysis, conducted as far as possible following general procedures for such analyses laid down by the U.K. Treasury. The government response in 2009, which largely adopted SAGE's recommendations into U.K. policy, implicitly endorsed this analysis and its conclusions.

4. SPECIFIC ISSUES IN THESE ANALYSES

4.1. Uncertainty

ELF magnetic fields are classified by IARC and WHO as "possibly carcinogenic," and authoritative review bodies agree that a causal link, even with childhood leukemia, the disease for which the evidence is strongest, is far from certain. However, three of the four HEAs chose not to include any numerical factor to account for this uncertainty and performed an analysis based on the assumption that the link is causal. CADHS presented individual degrees of certainty for its three authors, ranging from 10 to 100 for childhood leukemia (mid points of 55–95).[17]

Similarly, no analyses explicitly included other uncertainties, such as whether the exposure reduction would in practice be as effective as theoretically calculated (if, for example, the carcinogenic effect were caused by a specific aspect of exposure, which a given intervention may reduce more or less than average exposure). California, however, does allow for such uncertainties in its framework.[9] The framework presents some examples with an uncertainty of causation of 10%; the model implies use of the probabilities for "degree of confidence" of causation published by its own scientists.[17]

4.2. Treatment of Scientific Evidence

In the IARC classification scheme, ELF magnetic fields are classified as "possibly carcinogenic," and the monograph makes clear that this is only on the basis of the evidence for childhood leukemia.[18] WHO also concluded that the evidence for all other health effects (other cancers and noncancer) is "much weaker."[19] There has consequently been a tendency to base policy decisions about precautionary measures on consideration of childhood leukemia. The United Kingdom is explicit in performing quantitative analysis based on childhood leukemia, then giving indications of the equivalent result if other health effects were considered. (The U.K. government subsequently stated clearly that its policy decisions were based on childhood leukemia only.) The California analysis is generalized, but in places the discussion considers the application to childhood leukemia first and other diseases secondarily.[10] The Israeli analysis was mainly based on childhood leukemia; background information on other diseases, although not dismissed, was not quantified.

Because there is no established effect or mechanism, there is uncertainty as to the appropriate exposure-response relationship to consider. The epidemiological evidence on childhood leukemia is often described as showing a threshold, though other, progressive, relationships are also consistent with the data.[20] Three of the four HEAs chose to model the putative effect as a threshold, where moving a person

from above the threshold to below removes him or her from the increased risk. Israel used a threshold of 0.2 μT (and, in discussions, California also focuses on this threshold), the United Kingdom 0.4 μT, and the Netherlands a range of alternatives from 0.2 to 0.5 μT. The California framework allows for a linear risk between chosen thresholds, with no excess risk below and a flat risk above.[9] In all cases, the relative risk was taken as 2 (in California, RR = 2 at 0.2 μT with a linear dose response, rising further to a cap at RR = 5), a value readily derived from pooled analyses,[21,22] but making no allowance for factors that could have led the epidemiological studies either to have found elevated (e.g., bias) or reduced (e.g., exposure misclassification) risks compared to the true risk.

4.3. Benefit from Preventing Disease

For cost-benefit analysis, it is necessary to choose a numerical value for the value to society of preventing a case of disease or a fatality. There is an extensive literature on the choice of this value in non-EMF contexts. It is generally recognized that there are two main approaches, "human capital" and "willingness to pay," and that values by either method vary greatly from country to country.

The United Kingdom used a standard value from non-EMF risk management for the United Kingdom of $2M per adult fatality, then, still compatible with U.K. practice, increased it by two factors of two, one because the disease is a dreaded one and one because it affects children rather than adults. The United Kingdom also used a separate value for nonfatal cases, derived from an estimate of the quality adjusted life years (QALYs) lost from a case of leukemia, using a value of $60k per QALY, a value used in assessing medical interventions and drugs in the United Kingdom. These were combined to give a weighted value of $3.2M per case. The California framework suggests $100k per year of life expectancy lost and $300k per nonfatal cancer; the California model used $5M per fatality. This number was based on a review of numbers used by various California programs, which varied from $1M to $10M per death avoided. Stakeholders engaged in the process presented arguments about these and other factual matters; some were against assigning any number, which they considered to be too utilitarian. Israel implicitly discussed values from $1–5M based on a review of numbers used by various Israeli programs. The Netherlands did not need to select a value because it conducted a cost-utility analysis,

4.4. Time Period Considered and Discounting

For capital projects, costs are usually expended at the start of the project. But most benefits accrue over the lifetime of the project. Further, there may be ongoing financial costs, for instance, in the case of power lines, changes to the electrical losses, or maintenance costs from one engineering solution to another. Both costs and benefits falling in future years are typically valued by society as less than those falling in the present, and this is captured in a discount rate. The United Kingdom discounted future benefits (with a discount rate of 3.5%, but also assuming that the value of preventing a fatality would increase by 2% per year, giving a net discount rate of 1.5%). The California framework allows for discounting as well (and uses a discount rate of 3% in some example calculations); however, the model presents undiscounted numbers. California alone included ongoing costs. The California framework also included options for financing costs, though the model does not include these.

With a positive discount rate r, it is possible, in principle, to sum benefits from all future years, without limit, to a finite sum. However, it is more common to compare costs and benefits over a fixed period approximating to the life of the project. The United Kingdom used 50 years, California and Israel 35 years, and the Netherlands did not need a value. The overall discount factor over a period of T years is given by:

$$\sum_{t=1}^{T}\left(1-\frac{r}{100}\right)^{t-1}.$$

Thus the United Kingdom's 50 years with a discount rate of 1.5% per year gives a discount factor of 0.7, equivalent to 35 years with no discount rate; applying a discount rate of 1.5% indefinitely is equivalent to 67 years with no discount rate.

4.5. External Costs

As well as the immediate costs of an intervention (e.g., capital costs), there may be further costs to the electricity system (e.g., changes to electrical losses) or external to the electricity system being considered. The most obvious of these external costs is any effect on property values. Both the United Kingdom and California discuss these. The

United Kingdom considers first-round external costs (e.g., the devaluation of property immediately affected) relevant for some interventions, such as planning "corridors" around power lines, but not second-round external costs (e.g., knock-on increases in property values elsewhere, or beneficial effects to society of cheaper property), justified as being in line with U.K. Treasury advice.[23,24] The California framework discusses options of including various first-round external costs but similarly does not include second-round costs, and these costs are not included in the model. Both the United Kingdom and California show that for some options, effects on property values dominate the costs, and therefore their treatment is critical. California also considered several other external costs or consequences (e.g., electrocution, automobile accidents, pollution) but the framework suggested these would have small impacts and the model did not include them.

5. COMPARISON OF ANALYSES

Many possible EMF reduction policies exist, applying to many different sources of exposure. California considered interventions applicable to distribution wiring and ground currents as well as transmission; the United Kingdom considered house wiring as well as transmission (a subsequent assessment from SAGE considered distribution but did not use formal HEA); Israel and the Netherlands just considered transmission. For our analysis we consider just transmission as this is common to all four HEAs (though the definitions vary slightly). Reducing fields at the design stage is most cost effective and usually cheaper than retrofitting existing lines. The options considered by one or more HEAs include rephasing or split-phasing, compact lines, rerouting, separating lines and homes, and burying.

To formulate a framework that allows us to compare the four HEAs, we define the following parameters:

H: the number of houses with fields exceeding a threshold, in which the exposure is reduced to below the threshold, due to the intervention, per unit of intervention (e.g., per one km of line buried).

Ch: the average number of children per household.

P: the probability that exposure is causal and that the intervention reduces the exposure, and hence the disease, in practice.

I: the "base rate" or incidence of childhood leukemia in the population per year.

RR: the relative risk for exposure above the threshold considered compared to below it

T: the number of years for which the intervention is effective in reducing the exposure.

V: is the value associated with preventing a case of disease. (Where available, we separately define V_F: the value of a fatality, V_N: the value of a nonfatal case, and V_W: the weighted value of preventing any case).

D: the discount factor derived from the annual discount rate.

C: the initial cost per unit of intervention for the period of T years.

Then the expected number of children with reduced exposure per unit of intervention is:

$$H.Ch.P,$$

the number of cases avoided per child with reduced exposure over the entire period is:

$$I.(RR - 1).T,$$

and the total benefit per unit of intervention is:

$$B = H.Ch.P.I.(RR - 1).T.V_W.D.$$

The result of a CBA can be expressed in various ways. Conceptually the simplest is that if B>C, the CBA concludes that the intervention is justified, while if B<C then it is not. However, this requires the assessment to be performed for each separate specific value of H. A more useful approach, which we use for our comparison, is to express the same result in terms of a new "break-even" parameter H*, the number of houses that need to be affected by a unit of intervention such that the costs of intervention are equal to its benefits. If the estimate for a specific locality suggests a higher number of affected houses than H*, the intervention is justified in cost-benefit terms, otherwise it is not.

$$H^* = C / Ch.P.I.(RR - 1).T.V_w.D$$

We summarize the values for key parameters used in the four HEAs considered in Table I. (For California, we have constructed the HEA using the values from the model, applied to childhood leukemia only, and taking a threshold dose response.)

Table I. Values of Parameters from Different Countries

Parameter	UK[a]	Israel[b]	California[c]	Netherlands[d]
Year of Publication Analysis	2007	2005	2002–2004	2004
Parameters Relevant to Calculation of Benefit				
Threshold (μT)	0.4	0.2–1	0.2	0.2–0.5
B (incidence of childhood leukemia per year)	4.17×10^{-5}	5.0×10^{-5}	5.0×10^{-5}	3.6×10^{-5} [e]
T (number of years for which the intervention is effective)	50	35	35	–
Discount rate	1.5%	0	0	–
D (overall discount factor)	0.7	1	1	
RR (relative risk)	2	2	2	2
P (probability that intervention reduces disease)	1	1	0.7	1
Ch (average number of children per household)	0.45[f]	0.62[g]	1[h]	
Parameters Relevant to Assigning a Value to the Benefit				
Value of one life-year lost	$60k	–	$100k	–
V_F (value of a fatality)	$8M (children)	–	$5M (adults)	–
V_N (value of a nonfatal case)	$1M	–	$300k	–
V_W (weighted value of preventing any case)	$3.2M	$1–5M	$5M	–
Parameters Relevant to Calculating Costs				
Voltage lines considered	≥132 kV	≥22kV	≥69 kV	≥50 kV
Rephasing	$40k–800k per line (assume line is 20 km)	$22–50 k per km	$50k per km	$440k–1.6M per line
Compacting	not possible	$22–50k per km	$50k per km	
Splitting	–	–	$50k per km	$90–400k per km
Relocation	$500k per km	–	–	$400k–1500k per km
Undergrounding	$12–34M per km	$900k per km	$900k per km (range $310k–$3.75M)	$1.4–10M per km
Raise clearance	$200k per span	–	–	
Calculations of H* (Break-Even Number of Homes) Based on Parameters Above				
Rephasing	1–19	4–46	6	
Compacting		4–46	6	
Splitting			6	
Relocation	238			
Undergrounding	5700–16200	165	103 (range 35–429)	
Raise clearance	95			

Notes: Italic values not present in original analysis but added by us to allow comparisons between analyses. If the analysis concerns fatalities only, V_W is replaced by V_F. U.K. costs for rephasing are presented as "per line." To allow comparison with other "per km" figures we have assumed arbitrarily that the average line is 20 km. Separating homes and lines was considered in the United Kingdom as an option applied to the whole country and does not easily break down into unit costs, so is not included here, despite being the focus of greatest attention in the U.K. policy debate.

a (15)

b (25)

c (9,11)

d (13)

[e] Calculated based on data from [d] :110 new cases per year in population of 3 million children.

[f] Ref. 26 calculated based on data from supporting paper S15 : 10 million children in 22 million homes.

[g] Ref. 27, http://www.cbs.gov.il/reader/newhodaot/hodaa_template.html/hodaa=201411025. Scaled from average number of children per family to average number per household using U.K. ratio.

[h] Ref. 8.

5.1. Comparison with Actual Numbers of Homes Present

With the results of the CBA expressed in this way, as the break-even number of homes that must be present, we need to compare this number to the actual number of homes present. We present available information on this in the relevant countries in the Appendix. In summary, averaged over the whole country or state, typical numbers of homes per km may be less than 10 (United Kingdom, California, and Netherlands figures); averaged over lines where homes are present at all, they may be 10–60 per km (Netherlands and California); and the maximum may be 300 per km (United Kingdom). Some interventions, such as undergrounding, can remove most such homes from exposure, some only a fraction, and some, such as raising clearances, only a small fraction.

6. DISCUSSION

6.1. Methodology of HEAs Applied to EMFs

The four HEAs we have considered are methodologically quite similar. The most significant qualitative choices that have to be made when performing HEA on EMFs are what dose-response relationship to assume, what allowance if any to make for uncertainty, and, for a CBA only, what diseases to consider. Three HEAs assumed a threshold dose response, made no allowance for uncertainty, and gave prominence to childhood leukemia. The California framework allowed for both threshold dose response and a linear model (although with risk arbitrarily capped); it also allows for other health effects (and the model implicitly includes diseases such as adult brain tumors and ALS in addition to childhood leukemia). As well as these qualitative choices, several quantitative choices of parameter values are needed, but most values chosen were similar. Specifically, the value to society of preventing a fatality, which is known to vary greatly globally, varied by only 50% between the three countries (though with a larger range in Israel). There was a greater range used in the costs for various interventions. The greatest differences were in the costs of undergrounding. This is well-known as an area of dispute, with some of the difference accounted for by different voltages, different ratings, and inclusion of different components of total project cost. We consider that a genuinely like-for-like comparison between comparably industrialized countries would produce similar costs. Choices of whether to include discounting (United Kingdom) and ongoing costs (investigated in the California framework) did not make a great difference, as expected given that the discount rates are relatively low compared to the number of years considered, T, and that most of the costs occur at the initial point of creating the intervention. The issue of whether and how to include external costs, which is capable of making a big difference, was in fact treated similarly by all these HEAs.

Most of these interventions produce a fixed reduction in magnetic fields. As the magnetic-field threshold chosen in the HEA is reduced, the absolute number of homes exposed at that level increases, as does the absolute number of homes removed from that exposure by the intervention. The fraction of homes removed from the exposure, however, depends on the details of how the fields fall with distance before and after the intervention. For example, using the Netherlands data, as the threshold considered is reduced from 0.5 to 0.2 μT, the number of homes exposed prior to any intervention increases by a factor of 2.1, but the fraction removed from that exposure by a given intervention changes only from 0.32 to 0.37.

6.2. Results of HEAs

The interventions considered and the conclusions drawn about them can helpfully be analyzed by reference to two exemplar groups: relatively low-cost measures, such as rephasing and, in California, compacting; and relatively high-cost measures, such as undergrounding.

The low-cost interventions are, under the assumptions of these HEAs, justified if H, the number of homes per km removed from elevated exposure by the intervention, is of order 1–50. This number of homes appears to be found in the countries concerned, certainly in areas where power lines pass through housing at all. Whether the HEA indicates that these interventions are justified depends on how many of the homes are removed from exposure, but it certainly seems likely there will be circumstances where it does. By contrast, the high-cost interventions are similarly justified if H is of order 100 (California) up to thousands (United Kingdom). This appears unlikely to occur in the countries concerned. If lower thresholds are considered, whilst retaining the same relative risk and value of preventing a fatality, then of course interventions become

progressively more justified; but, when a given set of epidemiological findings are interpreted as a threshold dose response, a choice of lower threshold should be accompanied by a commensurately lower relative risk, countering this effect.

We stress that our analysis should not be taken as forming any policy recommendations; before considering an intervention in a given country, it would be necessary to revisit the figures and assumptions in the HEA.

6.3. Use of HEA in Policy Formation

The WHO EHC recommends "low cost" precautionary measures, without giving quantitative guidance as to what "low cost" means. Whilst only three countries have used a formal CBA to address this question, our analysis here shows that such HEAs are perfectly possible to perform. Although many assumptions and choices have to be made, the HEAs so far performed have, effectively independently, made sufficiently similar choices so that the conclusions are reasonably concordant.

7. CONCLUSIONS

These analyses suggest that, on the assumptions made, there are some low-cost measures, such as rephasing, that can be applied to transmission in some circumstances and that can be justifiable in cost-benefit terms, but that higher cost measures, such as undergrounding, become unjustifiable. However, the key choice made by three of the analyses is not to include any uncertainty factor (and the one that does, California, suggests greater certainty than would probably be the current consensus). Put another way, these analyses ask what measures would be justifiable if magnetic fields were known to cause childhood leukemia (but childhood leukemia alone), and, although a lesser factor, if the interventions are known to be reducing the most relevant aspect of exposure (recognizing that California does include options to allow for alternative exposure metrics). Allowing for significant uncertainty in whether the relationship with childhood leukemia is causal could render even low-cost interventions for transmission unjustifiable; including other diseases as well as childhood leukemia could render higher cost interventions justifiable.

Of the four HEAs, those in the United Kingdom and Israel were influential in determining the country's EMF policy. In California and the Netherlands, the HEA may well have informed the debate, but the policy chosen did not stem directly from the HEA.

ACKNOWLEDGMENTS

JS is employed by National Grid and worked on this article with its permission. The views expressed are the authors' alone and not necessarily those of National Grid, and no one in National Grid saw this article in advance of publication or exercised any control over it save JS in his capacity as author.

APPENDIX A: AVAILABLE INFORMATION ON NUMBERS OF HOMES PRESENT ALONG POWER LINES

Netherlands has 10 homes per km exposed to 0.2 μT (equivalent to a distance of 90 m from the centerline of the power line) and 6 per km exposed to 0.4 μT (65 m) averaged over all power lines of the voltages included. The Netherlands also provides data on the reduction in the number of homes exposed at each of the thresholds considered if each technical option were applied. The result for a threshold of 0.4 μT for vector sequence rearrangement is 20–74 homes; and for phase splitting 1.2–5.3, relocation 2.6–10, and undergrounding 1.6–12, all homes per km, and all applied only to lines that have homes present at all, not to all lines. There must be places where the housing density is higher than average but no data are provided on the extremes.

The United Kingdom also provides data on homes near lines, analyzed by spans. The data are for the number of homes within 50 m; this can be taken as roughly equivalent to an exposure of 0.4 μT. The average span length is 330 m so we convert homes-per-span to homes-per-km using this figure. The England and Wales average over all transmission lines is 7.3 homes per km. There are 12 spans (0.06%) with $>=$ 100 homes within 50 m (the highest category presented). We therefore take this figure, equivalent to 300 homes per km, as the highest likely to be found in the United Kingdom (the single span with the most homes equates to 600 homes per km). The U.K. analysis assumed that all these homes are removed from the exposure by undergrounding or rerouting, but only a fraction, not explicitly calculated but deduced from data provided as about 75%, by rephasing.

California estimates 63 homes per km "exposed to high fields" (above 0.2 μT) from transmission lines for the 5–8% of the length that runs through residential areas (7.4 homes per km for the total length of these lines).

For Israel, information on this key parameter is uncertain with estimates as to the percentage of the population exposed above 0.2 μT ranging from 7.6% to 66% (depending on the stakeholder evaluating this parameter).

REFERENCES

1. Kandel S. The source of variation in policies around the world: The case of protection of human health from extremely low frequency electromagnetic fields. Dissertation, Hebrew University of Jerusalem, 2010.
2. The Organisation for Economic Co-operation and Development (OECD), Regulatory Policy Division Directorate for Public Governance and Territorial Development. Building an Institutional Framework for Regulatory Impact Analysis (RIA): Guidance for Policy Makers, Version 1.1, Paris, 2008.
3. Lichfield N. Community Impact Evaluation. University College London Press, London, 1996.
4. Eduards W, Newman JR. Multi Attribute Evaluation. London: Sage Publications, 1982
5. Australian Radiation Protection and Nuclear Safety Agency (ARPANSA) Radiation Protection Series. Regulatory Impact Statement, Radiation Protection Standard, Maximum Exposure Levels to Electric and Magnetic Fields 0 Hz – 3 kHz, Consultation Draft, 2007.
6. Knudson PC, Sponsor. House Bill 127: Utility Siting Amendments. An Act Relating to Public Utilities; Placing Conditions on the Siting of Electrical Facilities by Local Governments; Requiring Local Governments to Pay for Costs Beyond Standard Costs, Including Costs of Buried Transmission Lines, Unless Those Costs are Collected from Consumers Pursuant to an Order or Rule of the Public Service Commission or are Apportioned to the Utility Pursuant to a Decision of the Electrical Facility Review Board Creating the Electrical Facility Review Board to Resolve Certain Disputes Regarding the Siting of Electrical Facilities; Granting Rulemaking Power to the Board; and Providing an Effective Date. State of Utah, House of Representatives, General Session, 1997 Jan 1.
7. Rodick S, Musser P. Evaluation of measures and costs to mitigate magnetic fields from transmission and distribution lines. P. 10 in Missouri Valley Electric Association Engineering Conference April 7, 1993; Kansas City, MO: Missouri Valley Electric Association, 1993.
8. California Department of Education (CDE). Power Line Setback Exemption Guidance. Available at: http://www.cde.ca.gov/ls/fa/sf/powerlinesetback.asp, Accessed September 10, 2013
9. Von Winterfeld D, Eppel T, Adams J, Neutra R, DelPizzo V. Managing potential health risks from electric power lines: A decision analysis caught in controversy. Risk Analysis, 2004; 24(6): 1487–1502.
10. Von Winterfeld D. Bridging the gap between science and decision making. PNAS, 2013; 10(suppl 3):14055–14061.
11. California EMF Program. Policy Options in the Face of Possible Risk from Power Frequency Electric and Magnetic Fields (EMF). California Department of Health Services, 2002. Available at: www.ehib.org/emf.
12. State Secretary of Housing Spatial Planning and the Environment of the Netherlands. Recommendations with regard to overhead high-voltage power lines, October 3, 2005; SAS/2005183118.
13. Kelfkens G. Costs and Benefits of Reduction of Magnetic Fields Due to Overhead Power Lines. National Institute for Public Health and the Environment (RIVM), KEMA T&D POWER, ARNHEM, Dutch Ministry of Housing, Spatial Planning and The Environment (VROM), The Netherlands, 2003.
14. Israel Expert Committee Report on Exposure to Magnetic Fields Generated by the Electricity Network, 2005. [Hebrew]. Available at http://www.sviva.gov.il/subjectsEnv/Radiation/Electrical_Facilities/Documents/vadat_mumchim_1.pdf, Accessed July 20, 2013.
15. Stakeholder Advisory Group on ELF EMF (SAGE). Precautionary approaches to ELF EMF: First Interim Assessment: Power Lines and Property, Wiring in Homes, and Electrical Equipment in Homes. R K Partnership LTD, UK, 2007.
16. Government response to the Stakeholder Advisory Group on extremely low frequency electric and magnetic fields (ELF EMFs) (SAGE) recommendations. Written Ministerial Statement 16 October 2009. Available at: www.dh.gov.uk/en/Publichealth/Healthprotection/DH_4089500, Accessed August 10, 2013.
17. Neutra R.R., DelPizzo V., Lee G.M. California EMF Program. An Evaluation of the Possible Risks from Electric and Magnetic Fields (EMF) from Power Lines, Internal Wiring, Electrical Occupations, and Appliances. California Department of Health Services, 2002. Available at www.ehib.org/emf, Accessed September 10, 2013.
18. International Agency for Research on Cancer (IARC). Non-Ionizing Radiation, Part 1: Static and Extremely Low-Frequency (ELF) Electric and Magnetic Fields. Monographs of the Evaluation of Carcinogenic Risks to Humans. International Agency for Research on Cancer, Vol. 80, 2002.
19. World Health Organization Environmental Health Criteria (WHO EHC). Extremely Low Frequency Electromagnetic Fields, No.238, 2007. Available at: www.who.int/emf.
20. Kheifets L, Afifi A, Monroe J, Swanson J. Exploring exposure-response for magnetic fields and childhood leukemia. Journal of Exposure Science and Environmental Epidemiology, 2011; 21:625–633.
21. Ahlbom A, Day N, Feychting M, Roman E, Skinner J, Dockerty J, Linet M, McBride M, Michaelis J, Olsen J. A pooled analysis of magnetic fields and childhood leukemia. British Journal of Cancer, 2000; 83:692–698.
22. Greenland S, Sheppard A, Kaune W, Poole C, Kelsh M. A pooled analysis of magnetic fields, wire codes, and childhood leukemia. Epidemiology, 2000; 11:624–34.
23. Doukas H, Karakosta C, Flamos A, Psarras J. Electric power transmission: An overview of associated burdens. International Journal of Energy Research, 2011; 35:979–988.
24. Health and Safety Executive (HSE). Decision making process "Reducing Risk, Protecting People," 2001. Available at: http://www.hse.gov.uk/risk/theory/r2p2htm.
25. Israeli Cost Benefit Analysis — In house document of the Radiation Abatement Division of the Ministry of the Environment, 2005. [Hebrew].
26. Supporting paper S15 from Stakeholder Advisory Group on ELF EMFs (SAGE). Precautionary Approaches to ELF EMFs: First Interim Assessment: Power Lines and Property, Wiring in Homes, and Electrical Equipment in Homes. R K Partnership LTD UK, 2007.
27. Israeli Central Bureau of Statistics. Household and Families Demographic Characteristics. Available at: http://www.cbs.gov.il/reader/newhodaot/hodaa_template.html?hodaa=2014 11025, Accessed January 2014.
