# Supplementary material for: Magnetic Fields and Cancer: Epidemiology, Cellular Biology, and Theranostics
Source: Int J Mol Sci. 2022 Jan 25;23(3):1339. doi: 10.3390/ijms23031339 (PMC8835851; doi:10.3390/ijms23031339)
Supplement: Supplementary file 1 [file ijms-23-01339-s001.zip › Supplementary Data Set S1/MF and Cancer.Data/PDF/0489929154/1-s2.0-S0079610718301007-main.pdf]

Progress in Biophysics and Molecular Biology 141 (2019) 25–36

Contents lists available at ScienceDirect

Progress in Biophysics and Molecular Biology

journal homepage: www.elsevier.com/locate/pbiomolbio

Biological effects of non-ionizing electromagnetic fields: Two sides of a coin

Timur Saliev [a, b, *], Dinara Begimbetova [b], Abdul-Razak Masoud [b], Bakhyt Matkarimov [b]

[a] Kazakh National Medical University Named After S.D. Asfendiyarov, Tole Bi Street 94, Almaty, 050000, Kazakhstan
[b] National Laboratory Astana, Nazarbayev University, 53 Kabanbay batyr Ave., Astana, 010000, Kazakhstan

A R T I C L E I N F O

Article history:
Received 11 April 2018
Received in revised form
12 July 2018
Accepted 16 July 2018
Available online 17 July 2018

Keywords:
Electromagnetic field
Bio-effect
DNA damage
Blood cancer
Reproduction
Therapy

A B S T R A C T

Controversial, sensational and often contradictory scientific reports have triggered active debates over the biological effects of electromagnetic fields (EMFs) in literature and mass media the last few decades. This could lead to confusion and distraction, subsequently hampering the development of a univocal conclusion on the real hazards caused by EMFs on humans. For example, there are lots of publications indicating that EMF can induce apoptosis and DNA strand-breaks in cells. On the other hand, these effects could rather be beneficial, in that they could be effectively harnessed for treatment of various disorders, including cancer. This review discusses and analyzes the results of various in vitro, in vivo and epidemiological studies on the effects of non-ionizing EMFs on cells and organs, including the consequences of exposure to the low and high frequencies EM spectrum. Emphasis is laid on the analysis of recent data on the role of EMF in the induction of oxidative stress and DNA damage. Additionally, the impact of EMF on the reproductive system has been discussed, as well as the relationship between EM radiation and blood cancer. Apart from adverse effects, the therapeutic potential of EMFs for clinical use in different pathologies is also highlighted.

© 2018 The Authors. Published by Elsevier Ltd. This is an open access article under the CC BY license (http://creativecommons.org/licenses/by/4.0/).

Contents

1. Introduction 25
2. Literature analysis and methodology 26
3. Induction of oxidative stress and DNA breaks by electromagnetic fields 26
4. Non-ionizing electromagnetic fields and blood cancer 29
5. Effect of non-ionizing electromagnetic fields on reproduction and fertility 30
6. Summary and perspectives 31
Declaration of interest 33
Acknowledgements 33
References 33

1. Introduction

The intensive and rapid development of wireless communication devices has inevitably led to public concern about the possible negative consequences of exposure to electromagnetic fields (EMF) on health. Global organizations and national health institutions have reacted to this public demand by organizing a number of studies and setting up special commissions. For instance, the International Agency for Research on Cancer (IARC), a WHO agency, was assigned to define the link between use of mobile phones and head/neck cancers. As a result, IARC has classified radio-frequency EMF as possibly carcinogenic to humans (Group 2B).

Scientific knowledge concerning the bio-effects of EMF has been

* Corresponding author. Kazakh National Medical University named after S.D. Asfendiyarov, Tole Bi Street 94, Almaty, 050000, Kazakhstan.
E-mail address: tim.saliev@gmail.com (T. Saliev).

https://doi.org/10.1016/j.pbiomolbio.2018.07.009
0079-6107/© 2018 The Authors. Published by Elsevier Ltd. This is an open access article under the CC BY license (http://creativecommons.org/licenses/by/4.0/).

accumulating over the previous decades, including the impact of low and high frequency EMFs on the human body and their associated health hazards (Kocaman et al., 2018). The ambient 'EMF' in the human environment covers a variety of frequencies ranging from low frequencies (0–50 Hz) up to high level at 5 GHz (wireless LAN). In addition to natural EMF of the planet (magnetic field 0.02–0.07 mT), new sources of EMF started emerging in the 20th century as a result of building new electricity supply grids, which have been considered as source of extremely low frequency EMF (REFLEX, 2004). The middle of 20th century was characterized by the growth of radio-broadcasting and the sprouting of thousands of AM/FM radio and TV stations (Fig. 1). The situation became even more complicated with the rapid development of new electronic wireless devices designed for communication purposes, and specifically for mobile phones along with associated infrastructure. In fact, the market for mobile telephony has demonstrated incredible growth worldwide in the last couple of decades. The estimated number of mobile phone users in 2019 is 5.07 billion (data of Statista, Inc., USA).

At present, there is a range of GSM (Global System for Mobile communications) band frequencies used for telecommunication applications. For example, GSM-900 and GSM-1800 modes are intensively being utilized in most countries, except both Americas, where 850 MHz and 1900 MHz bands are much more popular. The GSM-900 system exploits the frequency of 890–915 MHz range for sending and 960 MHz for receiving information packs. GSM 1800 utilizes 1785 MHz and 1880 MHz frequencies for receiving and downlink, respectively. The fact is that a mobile phone user is constantly exposed to radiations from the mobile phone even if the person does not actually use the device for communication. Additionally, the human body is subjected to perpetual EM exposure from mobile telephony infrastructure, particularly from the tower base stations (Levitt and Lai, 2011). The growth of wireless networking technology such as wireless local area network (WLAN) hotspots, including Wi-Fi networks, can also result in the exposure to excessive radiation at radiofrequency EM diapason (Fagua et al., 2016; Jurcevic and Malaric, 2016; Woelders et al., 2017). In fact, the level of multi-source EM radiation, including radiofrequency EM spectrum, was found to be high in urban areas across different countries (Sagar et al., 2018).

This review discusses the results of in vitro, in vivo and epidemiological studies on the effects of non-ionizing electromagnetic fields (EMFs) on cells and organs. Emphasis is laid on the analysis of recent reports on the role of EMF in the induction of oxidative stress and DNA damage. The impact of EMF on the reproductive system and the relationship between the exposure to EM radiation and blood cancer has been also highlighted. In addition to this, the therapeutic potential of EMFs for bio-medical applications and future perspectives are discussed too.

2. Literature analysis and methodology

The literature search was carried out using Scopus, Google Scholar, PubMed, Web of Sciences (ISI Web of Knowledge), Medline, and Wiley Online Library databases. Available publications (in English) in peer-reviewed journals on biological effects of non-ionizing EMF were selected for the analysis. The paper selection was restricted to the period between 1990 – 2018 (date of publication). The main focus was on EMF-induced effects on DNA, reproduction and blood cancer. The studies on the impact of EMF on other organs and systems were excluded from the literature search. The keywords used for literature search were: 'electromagnetic field', 'bio-effect', 'DNA damage', 'blood cancer', 'leukemia', 'reproduction' and 'therapy' (as a combination with 'electromagnetic field').

An analysis of 421 published works for the period 1990–2018, using 'electromagnetic field' and 'DNA damage' as keywords, revealed a stable growth tendency with a peak of publications in 2016 (32 documents found) (database of Scopus, Elsevier). The top 10 countries by number of publications in this field (in descending order) are: USA, Italy, China, Germany, Japan, France, UK, Canada, India and Russia (Scopus data, Elsevier).

The trend in publications addressing biological effects of mobile phones showed a similar growth pattern (based on 'mobile phone' and 'DNA damage' keywords search). The highest number of publications was in 2016: 20 documents found (Scopus data, Elsevier). Most of the papers were original/research articles (63.7%), thus reflecting the public and scientific demand for such information.

3. Induction of oxidative stress and DNA breaks by electromagnetic fields

Oxidative stress occurs if the equilibrium between formation of reactive oxygen species (ROS) and the capacity of antioxidant system to neutralize them is disturbed. The main types of ROS in living organism are hydroxyl radical and superoxide. They play a crucial

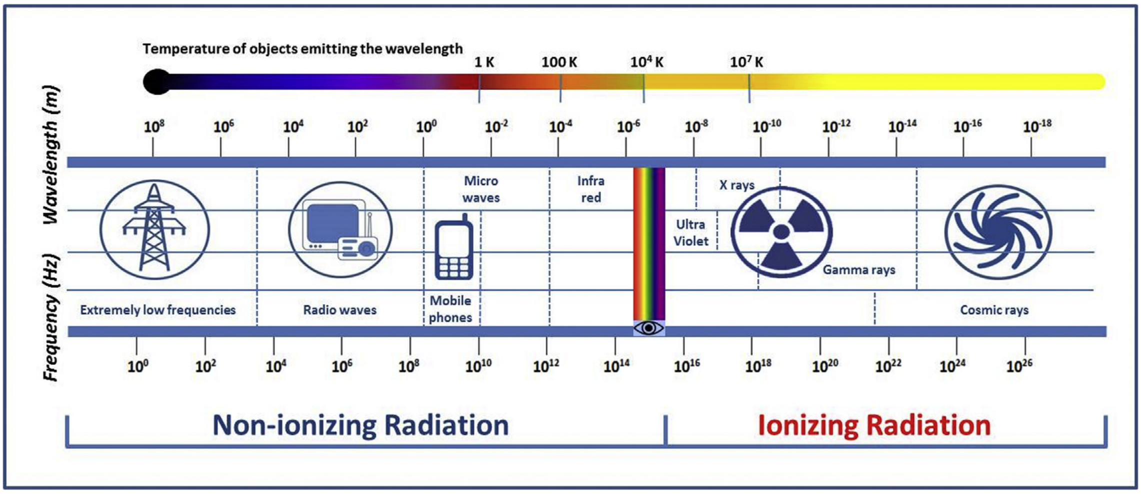

Fig. 1. Spectrum of electromagnetic radiation.

role in various biological reactions, but their uncontrolled overproduction can lead to DNA damage such as single/double-strand breaks and crosslinks (Menon et al., 2013; Uzunboy et al., 2016; Wells et al., 2015; Xu et al., 2016). In general, oxidative stress might be triggered by different internal or external factors, including gamma or UV radiation, which are thought to be responsible for the induction of free radicals formation and molecular oxidation (de Oliveira et al., 2013; Kovacs and Keresztes, 2002; Mendling and Haller, 1977).

Over the last few decades, it has been revealed that EMFs at extremely low frequencies are capable of increasing the production of free radicals, including hydroxyl free radicals, which can cause DNA double-strand breaks (Anderson, 1993; Buldak et al., 2012; Consales et al., 2012; Du et al., 2008; Esmaeili et al., 2017; Giorgi et al., 2011; Jouni et al., 2012; Koyu et al., 2009; Tkalec et al., 2007; Yokus et al., 2005). The results of in vivo and in vitro studies on the role of EMFs in induction of DNA damage and oxidative stress are summarized in Table 1.

Lai et al. reported that exposure of rats' brain cells to a 60-Hz magnetic field induced DNA single and double bond breaks (Lai and Singh, 1997). In this study the use of free radical scavengers prevented DNA damage, thus supporting the hypothesis of an active role of free radicals in EMF-induced processes. In a similar study conducted by Gao et al., natural oxidants, catechin and epicatechin, showed an ability to protect animals brains from oxidative stress induced by extremely low frequency EMF (50 Hz) (Gao et al., 2017).

In another work, Mihai et al. demonstrated that extremely low-frequency EMF are capable of inducing DNA strand breaks in normal human cells (Mihai et al., 2014). Human normal Vero cells were irradiated by extremely low-frequency EMF (100 Hz, 5.6 mT). Using comet assay and cell cycle analysis, a high number of cells with damaged DNA (increased tail length) were identified compared to unexposed cells from the control group. Cell cycle

Table 1
Summary of in vivo and in vitro studies on the role of EMFs in induction of DNA damage and oxidative stress.

Type of study	EMF parameters	Duration of exposure	Type of cells/animals	Results	References
in vivo	50 Hz (0.97 mT)	50/100 days	rats	long-term exposure induced oxidative DNA damage	Yokus, B. et al. (2005)
in vitro	935 MHz	1 min	human lymphocytes	no effect on DNA strand breakage	Stronati, L. et al. (2006)
in vitro	50 Hz (0.23, 0.47, 0.7 mT)	1,2,3 h	human lymphocytes	no effect on chromatid damage	Hone, P. et al. (2006)
in vivo	60 Hz (14.6 mT)	5 min on and 10 min off during 4 h	Salmonella enterica subspecies	no effect on DNA strand breakage	Williams, P. et al. (2006)
in vivo	834 MHz	7.5 h × 6 days	rats	no effect on induction of oxidative stress	Ferreira, A. et al. (2006)
in vivo	400 and 900 MHz	2 and 4 h	duckweed Lemna minor L.	EMF induced oxidative stress	Tkalec, M. et al. (2007)
in vitro	50 Hz (1 mT)	4 h (1 and 45 days)	Wistar rat tibial bone marrow cells	EMF demonstrated genotoxic potential	Erdal, N. et al. (2007)
in vitro	50 Hz (0.4 mT)	2,6,12,24,48 h	human lens epithelial cells	EMF is capable of inducing DNA double-strand breaks	Du, X. et al. (2008)
in vitro	900 MHz	1 h × 30 days	Sprague-Dawley rats	EMF induced oxidative stress	Ursache, M. et al. (2009)
in vitro	1.8 GHz	4,6,24 h	human trophoblast HTR-8/SVneo cells	high frequency EMF affects DNA integrity	Franzellitti, S. et al. (2010)
in vitro	900 MHz	5,10,20 min × 14 days	primary rat neocortical astroglial cell culture	low-intensity EMF induces ROS production and DNA damage	Campisi, A. et al. (2010)
in vitro	20,50,75 Hz	15 and 90 min	Escherichia coli bacteria	EMF affects bacterial transposition	Giorgi, G. et al. (2011)
in vivo	900 MHz	6 min	insect Drosophila melanogaster	EM radiation retards ovarian development in insects	Panagopoulos, D. J. (2012)
in vivo	1800 MHz	15 min/day x 7/14 days	rabbits	RF-EMF exposure led to an increase of lipid peroxidation	Guler, G. et al. (2012)
in vitro	50 Hz (1 mT)	16 min	AT478 murine squamous cell carcinoma cells	EMF induced oxidative stress and DNA damage	Buldak, R. et al. (2012)
in vitro	1800 MHz	1 h and 24 h	6 types of different cell lines	RF-EMF induces DNA damage in a cell type-dependent manner	Xu, S. et al. (2013)
in vitro	100 Hz (5.6 mT)	45 min	Vero cells	EMF had a genotoxic effect on Vero cells	Mihai, C. et al. (2014)
in vitro	50 Hz (1, 2,3 mT) and 1800 MHz	24 h	mouse spermatocyte-derived GC-2 cells	ELF-EMF (50 Hz) did not induce DNA damage. RF-EMF (1800 MHz) caused DNA strand breaks	Duan, W. et al. (2015)
in vitro	1800 MHz	1 h	mouse embryonic fibroblasts	RF-EMF induced significant DNA single-strand and double-strand breaks and activated repair mechanism	Sun et al. (2016)
in vitro	50 Hz (18.5 μT)	30, 60,120 min	human amniotic (FL) cell line	EMF increased mitochondrial ROS	Feng, B. et al. (2016)
in vitro	50 Hz (10 and 30 μT)	24 h	neuroblastoma and glioma cell lines	genotoxic effect and induction of oxidative stress	Kesari, K. et al. (2016)
in vitro	50 Hz (100 μT)	24 h	human SH-SY5Y neuroblastoma cells	EMF exposure can alter the G1 checkpoint response (cell cycle)	Luukkonen, J. et al. (2017)
in vivo	900 MHz	5–12 days	chick embryo	EMF caused structural changes in liver and DNA damage	D'Silva, M. et al. (2017)
in vitro	1950 MHz	50,100,150,200 h	human glioblastoma cell lines	EMF does not cause chromosomal damage	Al-Serori, H. et al. (2017)
in vitro	1951 MHz	20 h	Chinese hamster lung fibroblast cells	EMF exhibited dose-dependent genotoxicity	Sannino, A. et al. (2017)

analysis data demonstrated an increased frequency of cells in S phase indicating DNA single strand breaks. Mihai et al. hypothesized that the underlying mechanism of detected DNA damage induction is the production of ROS induced by EMF.

Edal et al. investigated the genotoxic and cytotoxic potential of extremely low frequency EMFs (50 Hz) (Erdal et al., 2007). The results of chromosomal aberration and micronucleus tests of Wistar rat tibial bone marrow cells showed that EMF did not cause chromosome aberration. However, mitotic index (percentage of cells undergoing mitosis) was significantly higher in the group exposed to long-term EM exposure (45 days) compared with short-term one (4 h). Duan and colleagues studied a potential genotoxicity of 50 Hz extremely low-frequency EMF and 1800 MHz radiofrequency EMF on mouse spermatocyte-derived GC-2 cell line (Duan et al., 2015). The results showed a significant increase in DNA strand breaks in the cells exposed to extremely low-frequency EMF. At the same time, the researchers reported that the exposure to radiofrequency EMF led to significant increase of oxidative DNA base damage (SAR 4 W/kg). On the contrary, the extremely low-frequency EMF radiation was not able to produce similar results. It indicates that both types of EM frequencies are potentially genotoxic at relative high intensities.

It must be noted that the 1800 MHz radiofrequency is associated with mobile telecommunication (GSM standard) and is widely used worldwide. In the United States, the Federal Communications Commission (FCC) set the specific absorption rate (SAR) level at a maximum of 1.6 W of energy absorbed per kilogram of body weight (W/kg). At the same time, European Committee for Electrotechnical Standardization (CENELEC, EU) followed standards established by the International Electro-technical Commission (IEC) for cell phones, where SAR is limited to 2 W/kg (IEC 62209–1).

In contrast to the reports on damaging effects of low frequency EMFs, Feng et al. demonstrated a beneficial ability of extremely low frequency EMF (frequency 50 Hz) to protect cells from apoptosis ("programmed cell death") induced by staurosporine, natural antibiotic (Feng et al., 2016). EMF was seen to trigger a release of mitochondrial ROS, and consequent activation of Akt signaling pathway, which is essential for anti-apoptotic action.

In a range of studies, it was also demonstrated that extremely low frequency EMFs can alter vital cellular functions such as regulation of proteins and cell cycle. Luukonen et al. showed that exposure of human SH-SY5Y neuroblastoma cells to extremely low frequency EMF led to a decrease in p21 protein level (after menadione treatment) (Luukkonen et al., 2017). It is known that p21 expression is associated with poor survival rate and chemoresistance. Additionally, an increase in cell number in the G1 phase and lowering of cell number in S phase after menadione treatment and EMF exposure was detected too (Luukkonen et al., 2017). In another study, menadione was also employed as a DNA damage co-factor in order to investigate the genotoxic effect of extremely low frequency EMFs (Kesari et al., 2016). In the study, human SH-SY5Y neuroblastoma cells and rat C6 glioma cells were subjected to EMF with a frequency of 50 Hz and magnetic fields of 10 or 30 μT. The genotoxic effect was highest in the group consisting SH-SY5Y cells treated by a combination of EMF and menadione. However, statistically significant effects were neither observed in the rat C6 cells in the EMF only group nor in the group consisting of its combination with menadione. At the same time, no effects of EMF on cytosolic superoxide production induced by menadione in human SH-SY5Y neuroblastoma cells were observed. The level of cytosolic superoxide was significantly higher in rat C6 cells exposed to the combination of menadione and EMF.

Conversely, there are also reports indicating genotoxic effects caused by exposure to high frequency EMF, including radiofrequency EM spectrum (D'Silva et al., 2017; Ferreira et al., 2006; Garaj-Vrhovac et al., 2011; Luukkonen et al., 2009; Ursache et al., 2009). Ruediger H. analyzed 101 studies of genotoxic effect induced by radiofrequency EMF (Ruediger, 2009). In 49 of the reports genotoxic effects were established, none was established in 42 and 8 reports had evidence of synergistic effects of chemical or physical agents along with radiofrequency EMF.

It must be noted that most of the studies on EMF induced DNA effects were conducted on peripheral blood lymphocytes, which may be explained by relative convenience, DNA content and availability of these types of human cells for experiments. For example, human peripheral blood lymphocytes were used to evaluate the impact of radiofrequency EMF (900 and 1800 MHz; GSM standard) emitted from mobile phone base stations on DNA and antioxidant status of volunteers (Zothansiama et al., 2017). The data analysis of samples from the group exposed to EMF revealed a higher frequency of micronuclei, suggesting EMF is able to induce DNA damage. In another work, Lantow et al. studied the effect of 1800 MHz radiofrequency EMF on the induction of reactive oxygen species (ROS) and changes in expression of heat shock protein 70 (Hsp70) in primary human monocytes and lymphocytes (Lantow et al., 2006). The researchers did not find a significant difference between groups of positive control and cells exposed to radiofrequency EMF.

Franzelletti and co-workers scrutinized the ability of high frequency EMF to cause DNA breaks (Franzellitti et al., 2010). The authors irradiated trophoblast cells (HTR-8/SVneo) with a 1.8 GHz continuous wave EMF as well as various GSM signals (mobile phone standard). It was revealed that high frequency EMF caused transient increases of DNA fragmentation (separation of DNA strands into pieces). At the same time, it was demonstrated that the cells were able to recover after EM exposure. A similar frequency (1.8 GHz) was utilized by Lasalvia et al. to demonstrate the feasibility of biochemical modifications in human peripheral blood lympho-monocytes exposed to radio frequency EMF, including the reduction of the DNA backbone-linked vibrational modes (Lasalvia et al., 2018).

Panagopulus et al. detected DNA fragmentation induced by EMF emitted from mobile phone (Panagopoulos et al., 2007). The researchers utilized Drosophila melanogaster as a biological model for studying the effect of EMF from common mobile phone (GSM 900-MHz and DCS, 1800-MHz standards) signals on Drosophila oogenesis. In this study, it was demonstrated that the radiation from both types of mobile phones signals induced DNA fragmentation and cell death.

An increase of 8-Oxo-2′-deoxyguanosine was observed in the liver of rabbits exposed to 1800 MHZ GSM-like RF signals (Guler et al., 2012). 8-Oxo-2′-deoxyguanosine is the main product of DNA oxidation, and it can be considered as an important contributor to carcinogenesis and aging. This indicates that intensive DNA oxidation and formation of free radicals occurred upon EM exposure.

Regarding mechanisms underlying the genetic effects of EMF exposure, it was revealed that EMF can initiate the displacement of electrons in DNA, which is accompanied by electron transfer (Blank and Goodman, 2008). The displacement directly affects the hydrogen bonds, which are responsible for DNA integrity and spatial DNA geometry. As a result, DNA chain undergoes separation and transcription.

All these works indicate the role of EMF in the induction and enhancement of activity of free radicals and triggering oxidative processes. However, the observed bio-effects are dependent on various conditions such as cells oxidative status, level of antioxidative enzymes, cell type and parameters of applied EMF (Lai and Singh, 2004; Nylund and Leszczynski, 2006; Phillips et al., 2009; Xu et al., 2013; Zhang et al., 2008).

It must be noted that the type of cell line is a crucial factor affecting the results of research on EMF induced biological effects. A range of studies have demonstrated that various cell lines do respond differently to EM exposure (Remondini et al., 2006; Schwarz et al., 2008; Xu et al., 2013). It is also worth noting that EMF parameters are important factors in EMF-elicited biological effects, particularly, the EMF wave-shape and type. For instance, Campisi et al. compared the genotoxic effect of two types of EMF (900 MHz): un-modulated continuous wave (CW) and amplitude modulated (AM) characterized by a modulation frequency (F_m) of 50 Hz, and 100% modulation. The authors observed an increase of production of reactive oxygen species (ROS) and DNA fragmentation, where the modulated wave was more effective in induction of bio-effects (Campisi et al., 2010). A similar effect was reported by Franzellitti et al. (2010). The authors demonstrated that exposure of trophoblasts to modulated GSM signal caused DNA damage, whilst the irradiation of cells by continuous-wave signal of the carrier frequency did not affect DNA. Contrary to these results, studies conducted by Luukkonen et al. (2009) and Zhang et al. (2008) indicated the efficacy of continuous EM wave in the triggering of DNA damage and gene expression. The discrepancy of above-mentioned results might be linked to the difference in cell lines and exposure protocols.

Apart from the biological aspect, the impact of shape of electromagnetic wave on the outcome of experiments must be also taken into consideration. The bio-effects of square-waves and sine-waves at extremely low frequency EMF (50 Hz; field strength from 1 microT up to 1mT) were experimentally compared by Wahab et al., where human lymphocytes were utilized as a cell model (Wahab et al., 2007). It was revealed that the square waves were more effective than their sinusoidal counterparts in the induction of sister chromatid exchange (signature of genotoxicity).

Chromatid damage mediated by EMF was a topic of a range of studies over the last decades. Khalil and Qassem reported on the cytogenetic effect of pulsing EMF (50 Hz, 1.05 mT) on human lymphocyte cultures. The results showed that long EM exposure of lymphocytes (72 h) led to significant suppression of mitotic activity, higher incidence of chromosomal aberrations, including an increase in the frequency of sister-chromatid exchanges (Khalil and Qassem, 1991). In contrast to this report, Heredia-Rojas et al. did not detect any significant impact of 60 Hz sinusoidal EMF (densities of 1.0, 1.5, and 2.0 mT) on the frequency of sister-chromatid exchanges in human lymphocytes (Heredia-Rojas et al., 2001). The similar findings about the absence of effect of 50 Hz EMF on chromatids integrity were reported by Hone at el. (Hone et al., 2006).

However, there is a plethora of publications indicating a potential genotoxic effect of EMF operating at radiofrequency band, in particular, an impact of radiation of mobile phones on human DNA. Mazor et al. scrutinized the effects of exposure of human lymphocyte to radiofrequency EMFs (800 MHz, continuous wave) on genomic instability (Mazor et al., 2008). The specific absorption rates (SARs) were 2.9 and 4.1 W/kg that is close to the current levels established by ICNIRP guidelines for mobile phones. The exposure resulted in an increase of the levels of aneuploidy. Aneuploidy is a signature of chromosal abnormality, which has been strongly associated with miscarriage and birth defects in humans. At cellular level, aneuploidy has been linked to cell malfunction and carcinogenesis.

In another work, also dedicated to studies of impact of mobile phones on the DNA, human peripheral blood lymphocytes were exposed to continuous 830 MHz EMF, which is in the range of GSM standard telephony (Mashevich et al., 2003). A linear increase in chromosome 17 aneuploidy as a function of the SAR value indicating a genotoxic effect of radiofrequency EMF was detected.

On the other hand, there is also a big cohort of studies indicating no harm effect of radiation emitted from mobile phones on human DNA. For example, Danese et al. reported an absence of bio-effects on DNA double strand breaks in human lymphocytes exposed to EMF with a frequency 900 MHZ (Danese et al., 2017). Such a frequency has been commonly utilized as a carrier frequency for telecommunication systems using Global System for Mobile Communications standard protocol (GSM). In another study, Stronati et al. did not observe any significant genotoxic effect of EMF with frequency 935 MHZ (GSM standard) and SAR 1–2 W/kg on human lymphocytes (Stronati et al., 2006).

Williams and co-workers exposed bacterial cultures of *Salmonella enterica* to low-frequency EMF (Williams et al., 2006). The results of the study demonstrated no signs of increase of DNA damage. However, the morphological difference between bacterial cells and normal mammalian must be taken into account. It concerns the difference in DNA geometrical structure, transcription and translation processes, organization of organelles, and cell shape as well. In this context, results provided by Williams et al. cannot be directly extrapolated to the mammalian/human cell lines.

Radiation from mobile phones operating in another standard protocol so-called 'Universal Mobile Telecommunication System' (UMTS) was also investigated in a range of studies. UMTS is a widely used communication protocol covering about 37% UMTS of total voice calls. It is the most common system for mobile telephony in North America, France, Greece, Italy, and Holland (Langer et al., 2017). Schwarz et al. studied the effect of radiation at UMTS frequency standard in the context of potential genotoxic risks (Schwarz et al., 2008). Human fibroblasts and human lymphocyte cultures were exposed to EMFs with 1.950 MHz frequency (UMTS standard) below the specific absorption rate (SAR) safety limit of 2 W/kg. The results demonstrated that UMTS radiation caused the DNA damage in human fibroblasts unlike in lymphocytes. So, this type of EMF was not genotoxic for lymphocytes.

In a recent study aimed at evaluating the potential genotoxic effect of EM of radiofrequency UMTS standard, human glioblastoma cells were subjected to EMF at different SAR doses (0.25, 0.50 and 1.00 W/kg), in the presence of mitomycin C (chemotherapeutic agent) (Al-Serori et al., 2017). No evidence of genotoxic effect was found. Recently, Sannino et al. also reported on both adverse and beneficial effects of exposure of Chinese hamster lung fibroblast cells to radiofrequency EMF with frequency 1950 MHz (UMTS standard) (Sannino et al., 2017).

4. Non-ionizing electromagnetic fields and blood cancer

Approximately 350 000 people were diagnosed with leukemia, a type of cancer of the blood/bone marrow, across the globe in 2010. In the United States, there were 43 050 new cases of leukemia and 21 840 leukemia-related deaths in 2010 (Jemal et al., 2010). Blood cancer (leukemia, lymphoma and myeloma) were a cause of the deaths of approximately 53 010 people in the US in 2011. 6590 new cases were diagnosed in 2016 in USA (Terwilliger and Abdul-Hay, 2017). The leukemia by itself causes almost one-third of all cancer deaths amongst the population younger than 15 years (USA).

The association between EMF and leukemia has long been discussed over the last three decades. There are 51 reports on the link between leukemia and EMF found on Medline database and 58 published papers in Web of Science system (data on the beginning of 2018). These publications, first and foremost, are related to epidemiological attempts to analyze the potential correlation of rate between blood cancers and exposure to different types of EMF (Greenland et al., 2000; Kheifets and Shimkhada, 2005; Reid et al., 2011b; Tabrizi and Hosseini, 2015; Valera et al., 2014; Wartenberg, 2001). Pooled analysis demonstrated two-fold risk increase of

childhood leukemia as a result of exposures to extremely low frequency EMF, along with the consistency of studies across different countries, types of study design, methods of exposure assessment, and systems of power transmission and distribution (Schuz, 2011; Schuz and Ahlbom, 2008).

Kirschenlohr and co-workers studied the effect of extremely low-frequency EMF (ELF −EMF) on gene expression in the context of possible association between EMF and childhood leukemia in volunteers (males, aged 20–30 years) (Kirschenlohr et al., 2012). No gene response correlated to repetitive exposure to ELF-EMF was found. Although the results revealed the non-harmful character of ELF-EMF, they could be considered as doubtful, because the study was conducted in the adult population. Thus, it cannot correctly reflect the correlation between childhood leukemia and EMF radiation.

Another report on the risk of developing of childhood acute lymphoblastic leukemia as a result of parental occupational exposure to extremely low frequency EMFs was carried out on base of case-control study among children aged <15 years in Australia (Reid et al., 2011a,b). The authors did not find an association between maternal or paternal exposure to low frequency ELF any time before the birth and risk of childhood acute lymphoblastic leukemia. Teepen and van Dijck evaluated the evidence of relation between EMF and childhood leukemia based on the analysis of available the epidemiological and biological data (Teepen and van Dijck, 2012). The authors pointed out that the cause of childhood leukemia is multi-factorial, and EMFs can be considered as one of environmental exposures factors.

Yang et al. analyzed the cases of acute leukemia in relation to electric transformers and power electric lines (Yang et al., 2008). The acquired data indicate that there is an association between electric lines and XRCC1 Ex9 + 16A allele in patients with childhood acute leukemia. In another study no direct relationship was established between the use of mobile phones and risk of leukemia (Cooke et al., 2010). Rodriguez-Garcia and Ramos reported a correlation between acute myeloblastic leukemia and residing closeness to thermoelectric power plant (TPP) and high-power lines (HPL) (Rodriguez-Garcia and Ramos, 2012). The authors assumed that living near TPP's and HPL's increased the risk of developing of acute myeloblastic leukemia. Recently, a population-based case-control study of the link between residential magnetic fields exposure (from power lines) and childhood leukemia was conducted by Kheifets et al. (2017). The authors reported on a slight risk deficit in two intermediate exposure groups and a small excess risk in the highest exposure group.

The association between prenatal and postnatal exposure to high voltage power lines and childhood acute lymphoblastic leukemia was also investigated by Tabrizi and Hosseini (2015). The results of this cross-sectional case control study indicated that prenatal and childhood exposure to high voltage power lines could be considered as the most important environmental risk factor.

Despite the above-mentioned findings and available information, carcinogenic potential of EMF, particularly its radiofrequency spectrum, remains uncertain (Kocaman et al., 2018). The same situation can be extrapolated to the role of EMF in the induction of blood cancers, including leukemia. The growing evidence of in vitro and in vivo studies must be verified by comprehensive, independent, multi-central and rigorous epidemiological research.

Among the reports on the possible harmful impact of EMF on blood cells, there is also an interest in harnessing EMF as a therapeutic modality (Saliev et al., 2014b; Vadala et al., 2016). For example, Kaszuba-Zwoińska et al. demonstrated a potential of exploiting of low-frequency pulsed EMF (PEMF) for inducing apoptosis in human cancer blood cells (Kaszuba-Zwoinska et al., 2015).

5. Effect of non-ionizing electromagnetic fields on reproduction and fertility

The last decades were marked by growing interest in understanding the effect of EMF on human reproduction (Bernabo et al., 2010; Celik et al., 2012; Fejes et al., 2005; Ozguner et al., 2005; Wdowiak et al., 2007). One of the reasons for concern was a possibility of risk of constant EMF exposure on reproductive organs, particular the testes, from mobile phones, which are usually carried at waist level. Many studies have demonstrated the effect of EMF at high and low frequencies on human sperm (Agarwal et al., 2009; Avendano et al., 2012; Chavdoula et al., 2010; De Iuliis et al., 2009; Desai et al., 2009; Hong et al., 2005; Iorio et al., 2011; Panagopoulos, 2012; Tas et al., 2013). In most of these studies the focus has been made on the examination of sperm motility after exposure to EMF.

Agarwal et al. conducted a study on bio-effects induced by mobile phone radiation on ejaculated human semen (Agarwal et al., 2009). The semen was subjected to the radiation from mobile phone in ‘talking mode’. This study revealed that exposure of semen to EMF led to decreased sperm motility and viability accompanied by augmentation of level of ROS and decrease in total antioxidant capacity. In the work of De Iuliis et al. human spermatozoa (male haploid gamete cell) were subjected to radiofrequency EMF at a frequency of 1.8 GHz and specific absorption rates (SAR) 0.4–27.5 W/kg, which reflects characteristics of standard cellular phones (De Iuliis et al., 2009). Spermatozoa are highly complex specialized cells designed to survive a long and perilous journey from the site of insemination to the upper reaches of the female reproductive tract where fertilization occurs. The results indicated that such exposure led to formation of ROS that decreased motility along with vitality but increased DNA adducts formation.

Nahla Al-Bayyari studied the effect of cell phone usage on semen quality and men's fertility (Al-Bayyari, 2017). No statistically significant difference between groups (active users vs. moderate users of mobile phones) regarding sperm quality parameters related to cell phone usage was found. However, statistical differences in the frequencies of sperm concentration, volume, viscosity, liquefaction time and means of immotile sperms and abnormal morphology were detected. The study conducted by Hagras et al. (2016) showed a decrease in motility ratio and the progressive motility percentage in patients with prolonged cell phone daily usage.

As an alternative to human studies on the effects of EMF on reproduction, various animal models have been also intensively utilized last decades. Celik et al. used Wistar-Kyoto male rats for the exposure to radiation from mobile phone followed by morphological and microscopic analysis (Celik et al., 2012). The researchers did not find a significant difference between parameters in the testes from exposed group vs. non-exposed one. But ultrastructural analysis (SEM) showed a growth of the thickness of membrane propria and amount of collagen fiber. An increased number of electron-dense mitochondria and cellular structures were observed as well. It indicates that the testes do react on the EM exposure through morphological re-organization.

Nisbet and co-workers also utilized a male rat model for the study of effects of whole-body exposure to EMF on reproduction (Ozlem Nisbet et al., 2012). They reported on an increased testosterone level in rats exposed to 1800-MHz and 900-MHz radiation compared to control animals. Spermatozoa motility and concentration were significantly higher in the exposed groups. Such findings can be considered as a positive bio-effect of EMF on reproduction function.

Contrary to the findings of Nisbet et al., Al-Damegh showed that EMF had a negative effect on testicular architecture and blood enzymatic activity (Al-Damegh, 2012). Similarly, Odaci et al. investigated the impact of prenatal exposure of pregnant rats to a

900 MHz frequency EMF (Odaci et al., 2016). The exposure led to an increase in apoptotic index, DNA oxidation and lowering of sperm motility and vitality. Immature germ cells in the seminiferous tubule lumen, and altered seminiferous tubule epithelium and seminiferous tubule structure also were detected in testes of newborn rats as a result of prenatal exposure to 900 MHz EMF.

The effect of long-term radiation of mobile phones (1800 MHz) on female reproduction and oxidative stress in mice was investigated by Shain et al. (Shahin et al., 2017). The data of the study indicated that mobile phone radiation is able to increase the levels of reactive oxygen species (ROS), nitric oxide, lipid peroxidation, total carbonyl content and serum corticosterone along with the decrease in antioxidant enzymes, ovary and uterus. Reduced number of developing and mature follicles, corpus lutea, low level of pituitary gonadotrophins, sex steroids and decreased expression of SF-1, StAR, P-450scc, 3β-HSD, 17β-HSD, cytochrome P-450 aromatase, ER-α and ER-β were detected in the groups exposed to EMF (1800 MHz). These data attest to the harmful impact of mobile phone's radiation on female fertility.

Turedi and co-workers reported on the disruption of the ovarian follicle function, including follicle degeneration, vasocongestion, a low level of increased stromal fibrotic tissue and cytoplasmic vacuolization, as a result of prenatal exposure of pregnant rats to a continuous 900 MHz electromagnetic field (Turedi et al., 2016). Safian and co-workers scrutinized the impact of radiation from mobile phone (900 MHz, GSM mode) on the survival of pre-implanted embryos in mice (Safian et al., 2016). The results of the study demonstrated that the rate of embryo survival was similar in the groups exposed and non-exposed to EMF. But the percentage of dead embryos was higher in EMF-exposed group compared to the non-exposed. In another study, Suzuki et al. studied and influence of radiofrequency EMF (1.95 GHz wideband) on fertilization and embryo development in mice (Suzuki et al., 2017). The researchers found out that the rates of fertilization, embryogenesis, and blastocyst formation did not change significantly in exposed and non-exposed to EMF groups indicating the potential safety of radiofrequency EM exposure. However, these findings cannot truly testify the safety of EMF for humans, because the study was conducted on animal models only.

The difference of effects on reproductive capacity of insects from modulated and non-modulated EMF was examined by Panagopoulos. Experimental data showed that exposure to non-modulated GSM 900 MHz signal led to a decrease in the insect's reproduction ability, while the modulated GSM 900-MHz signal caused a decrease in reproduction. It was clearly demonstrated that the modulated GSM signal (‘speaking’ mode) had a more significant impact on oogenesis of insects. In addition, the bio-effects from GSM-900 MHz and GSM-1800 MHz signals were studied and compared using the same biological model. A fall in reproductive capacity was detected for both types of GSM radiation. The work of Panagopulus concurs with other reports on the influence of radiation from mobile phone on reproductive functions and embryogenesis (Forgacs et al., 2006; Ozguner et al., 2005; Tas et al., 2013; Wdowiak et al., 2007). A recent study conducted by Manta et al., (2014) demonstrated an increase in reactive oxygen species in the ovaries of Drosophila after exposure to radiofrequency fields. This may account for the effects described above (Manta et al., 2014).

Tsybulin and co-workers investigated the effect of EMF (GSM 900 MHz signal) on embryo development of Japanese quails (*Coturnix japonica*) (Tsybulin et al., 2012). The computer-controlled “connecting” mode was used for exposure of fertilized eggs to the radiation (maximum intensity 0.2 $\mu W/cm^2$). The exposed eggs demonstrated a facilitating effect of EMF on embryogenesis, which could be explained by activity of ROS. Despite the fact that these findings are intriguing in the context of stimulation of embryo development, there is no firm evidence provided about mutagenic and teratogenic potential of EMF for humans yet.

6. Summary and perspectives

Currently, the applications and usage of wireless technology are expanding rapidly due to constant public demand for an increase in speed, availability and quality of transferred information. The bandwidth of utilized EM frequencies for wireless devices (IEEE standard 802.11) covers a wide diapason. For mobile phones, the frequency range is 900–1800 MHz for 3G standard, for 4G frequency is 2–8 GHz, and up to 60 Hz carrier frequency for 5G. Regarding frequencies corridors for Wi-Fi systems, each country has developed and approved its own standards for Wi-Fi channels bands. In general, it covers frequencies ranging from 2.4 GHz up to 60 GHz (ISM band).

Apart from the direct radiation from individual devices such as mobile phones, humans are also exposed to the EMF from basic radio, electric and telecommunication stations. It encompasses radiation from Wi-Fi home and office routers, including situations when Wi-Fi zones (from few sources) are being overlapped at one particular spot. Thus, each person is potentially exposed to various sources of EMFs at the same time.

Aside reports from International Agency for Research on Cancer (IARC), dated by 2011, classifying the radiofrequency EMFs as Group 2B (possible human carcinogen), there is a solid number of reports indicating more profound harmful non-thermal effects on the health (Abramson et al., 2009; Akdag et al., 2016; Fragopoulou et al., 2012; Hardell et al., 2010; Nittby et al., 2009; Sudan et al., 2016). The rigid position of WHO and IARC triggered the formation of alternative international research groups and associations such as Bio-Initiative Committee (Hardell, 2017). Both sides criticize each other by pointing on bias, lack of balance and high selectivity of analyzing data.

Though significant research effort and published reports demonstrated the ability of EMF to trigger DNA damage, there is still insufficient evidence to provide an irrevocable proof of its carcinogenic and mutagenic potential for humans. At this current stage, there is a range of in vitro and in vivo studies about induction of oxidative stress and DNA fragmentation by various types of EMFs. But it must be taken into account the fact that most of the studies claiming a potential threat of EMF for the health were conducted either using cell culture (in vitro conditions) or animal models (in vivo). Therefore, it makes it problematic to make a direct extrapolation of obtained data to humans.

Regarding human studies, due to evident technical and ethical problems, most of the research work was based on epidemiological research and observation. Such discrepancies hamper an unbiased evaluation of consequences of exposure to EMF for health. This issue requires a further unprejudiced and comprehensive approach, which should include multi-disciplinary and multi-center experimental research coupled with long-term population studies carried by independent laboratories.

It should be also noted that in the real situation humans are often being simultaneously subjected to EMF from different sources. So the biological effects of multi-source and multi-frequency EMFs have yet to be explored and fully understood. Up to date, there is no any available information on accumulative effect of such combined exposure.

In contrast to the reported harmful effects, EMF can be also harnessed for the treatment of different pathologies. In particular, non-ionizing EMF could be employed for the targeted induction of apoptosis (“programmed cell death”) and membrane permeability (make it passable for drugs) in cancer management due to the non-invasiveness and high penetration capability.

The majority of published data on beneficial effects of EMF is related to the applications of pulsed (PEMF) and low intensity EMFs. These types of EMF have a long history of bio-medical use, and they have been a topic of extensive research since the invention of electricity. Recently, in a range of studies it was demonstrated that PEMFs have a high therapeutic potential for joint and bone problems treatment (Ceccarelli et al., 2013; Chen et al., 2010; De Mattei et al., 2009; Fini et al., 2005; Johnson et al., 2001; Kapi et al., 2015; Luo et al., 2012; Manjhi et al., 2013; Miyagi et al., 2000; Ongaro et al., 2012; Park et al., 2013; Wang et al., 2014; Xie et al., 2016; Zhong et al., 2012), tissue repair (Zou et al., 2017), wound healing (Callaghan et al., 2008), cancer management (Tatarov et al., 2011; Zimmerman et al., 2012) and modulation of cytokine secretion (Gomez-Ochoa et al., 2011). Moreover, EMF showed a huge potential for treatment various neurological pathologies such as Alzheimer's, Parkinson, Multiple Sclerosis and others (Arendash et al., 2012; Capelli et al., 2017; Jeong et al., 2015; Morberg et al., 2017; Sandyk, 1996).

Bio-medical applications of EMF might be expanded to its combined use with stimuli responsive nano-platforms of different origin. Moreover, the nature of EM radiation allows its conjoint application with various pharmacological, genetic, chemical, and other physical modalities in order to achieve synergy and optimization of curative effect (Bajic et al., 2009; Rosado et al., 2018; Saliev et al., 2014a, 2014b; Zimmerman et al., 2013). The list of the studies indicating a potential therapeutic effect of EMF is provided in Table 2.

There was a paradigm shift, from the situation when EMF was unilaterally perceived as a negative ‘dark’ force, to a new understanding of EMF as a versatile platform for clinical applications in the last few decades (Fig. 2). It is easy to predict the rise of such portable EMF-based technologies in the near future. One can particularly, expect the rapid growth of new systems for speed diagnostics based on the latest achievements in programming and machine learning that harness EMFs for non-invasive screening and collecting the data on processes going-on in the organism. This

Table 2
The list of the studies indicating a potential therapeutic effect of EMF.

Type of study	EMF parameters	Duration of exposure	Type of cells/animals	Results	References
in vitro	pulsed EMF; 10 Hz (0.4–0.8 mT)	72 h	Dunn osteosarcoma cells	PEMF controlled cell growth depending on the degree of cell differentiation	Miyagi, N. et al. (2000)
in vitro	pulsed EMF; 60 Hz (0.1 mT) and 100 Hz (0.2 mT)	–	human lymphocytes	PEMF modulated T-cell proliferation	Johnson, M. (2001)
in vitro	pulsed EMF; 75 Hz (0.2–3.5 mT)	30, 45, 60, 90 and 120 min	human neutrophils	PEMF increased of adenylyl cyclase activity and a reduction of superoxide anion production	Varani et al. (2002)
in vivo	pulsed EMF; 75 Hz (1.6 mT)	4 and 12 h/day for 3 months	Dunkin Hartley guinea pigs	PEMF demonstrated chondroprotective effect	Fini, M. et al. (2005)
in vivo	pulsed EMF; 15 Hz	8 h × 24 days	mice; murine endothelial cells	PEMF accelerated wound healing	Callaghan, MJ et al. (2008)
in vitro	75 Hz (1.5 mT)	24 h	bovine synovial fibroblasts	ELF-EMF exhibited anti-inflammatory activity	De Mattei, M. et al. (2009)
in vitro	60 Hz (1.2 ± 0.1 mT)	72 h	HeLa (human cervical cancer) and PC-12 (rat pheochromocytoma) cells	ELF-EMF exposure led to cell proliferation rate reduction	Chen, YC et al. (2010)
in vivo	1 Hz (100 mT)	60, 180, or 360 min × per day (4 weeks)	Swiss outbred female nude mice	ELF-EMF reduced tumor growth and progression	Tatarov, I. et al. (2011)
in vivo	50 Hz (17.96 μT)	2 h × day for 8 weeks	male Wistar rats	ELF-EMF attenuated spinal cord injury-induced osteoporosis	Manjhi, J. et al. (2011)
in vitro	pulsed EMF; 50 Hz (2.25 mT)	15 min × 7,8,9 days	Fibroblast-like cells growth was obtained from human peripheral mononuclear cells	PEMF decreased proinflammatory cytokine secretion	Gómez-Ochoa, I. et al. (2011)
in vitro	13.75 Hz (2.5–18 μT)	5 days	c2c12 myoblast cells	ELF-EMF increased cell proliferation	De Carlo et al. (2012)
in vitro	5, 25, 50, 75, 100, and 150 Hz	30 min (2) × 7 days	human bone marrow-derived mesenchymal stem cells	ELF/PEMF induced expression of alkaline phosphatase and osteocalcin	Luo, F. et al. (2012)
in vitro	75 Hz (1.5 mT)	24 h	human osteoarthritic synovial fibroblasts	EMFs display anti-inflammatory effects in human osteoarthritic synovial fibroblasts	Ongaro, A. et al. (2012)
in vitro/in vivo	50 Hz (0.5 mT)	8 h × 12 days	mouse bone marrow stromal cells/mice	EMFs promoted bone formation, incl. early osseointegration and bone mineralization and maturation.	Zhong, C. et al. (2012)
in vitro	27.12 MHz (modulation freq. 100 Hz-21 kHz)	1 h, 3 h, 6 h × 7 days; or 3 h × 3 days	hepatocellular carcinoma (HCC) cells and breast cancer cells	RF-EMF selectively inhibited the growth of cancer cells, while normal cells were not affected	Zimmerman, J.W. et al. (2012)
in vitro	75 Hz (2 mT)	5.10, 30 min; 1,4,8 h × 21 days	human mesenchymal stem cells (MSCs): bone marrow and adipose-tissue MSCs	PEMF stimulatied bone extracellular matrix deposition	Ceccarelli, G. et al. (2013)
in vitro	50/100 Hz (1 mT)	90 min	human bone marrow mesenchymal stem cells	EMF induced neural differentiation	Park, J. et al. (2013)
in vitro	pulsed EMF; 15 Hz (9.6 gauses)	30,60,90 min	primary rat calvarial osteoblasts	PEMF enhanced the osteoblast compatibility on titanium surfaces	Wang, J. et al. (2014)
in vivo	pulsed EMF; 50 Hz (1.5 mT)	8 h	rats	PEMF exhibited osteogenic effect	Kapi, E. et al. (2015)
in vitro	pulsed EMF; 50 Hz (0.6 mT)	5–120 min	rat calvarial osteoblasts	PEMFs stimulated osteogenic differentiation and maturation of osteoblasts	Xie, Y.F. et al. (2016)
in vitro	pulsed EMF; 2 Hz (0.5–3.0 A/m)	30 min (2) × 7 days	primary rat nucleus pulposus cells	PEMF inhibits secretion of IL-1β and TNF-α and promotes tissue repair	Zou, J. et al. (2017)

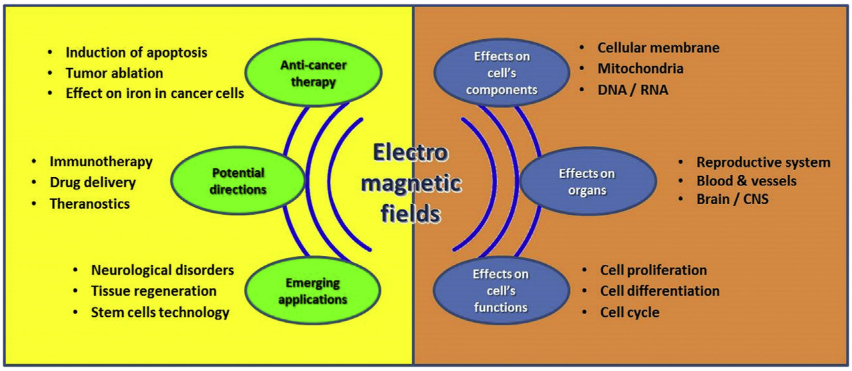

Fig. 2. Biological effects caused by electromagnetic fields, and their potential therapeutic applications.

would contribute to the development of a new model of telemedicine that encompasses a remote 'patient-doctor' interaction and the exchange of medical information between professionals. It would as well facilitate collection and analysis of data on public health by the authorized institutions. Up to date, this task has been alleviated by a rapid spread of health-related apps installable on portable mobile communication devices such as phones and tabs (Zhao et al., 2016). However, the potential hazard from EM radiation emitted by telecommunication appliances remains an issue for many customers and public institutions around the world. Thus, the development of EMF-based therapeutic systems should be accompanied by intensive parallel studies on the impact of EMF on the vital functions of biological systems and carcinogenicity.

Declaration of interest

The authors do report no conflicts of interest. The authors alone are responsible for the content and writing of the paper.

Acknowledgements

This work was funded by the grant of the Ministry of Education and Science of the Republic of Kazakhstan "Bio-effects of electromagnetic radiation emitted from mobile phones and Wi-Fi on the DNA of brain cells" (Grant number AP05133910).

References

Abramson, M.J., Benke, G.P., Dimitriadis, C., Inyang, I.O., Sim, M.R., Wolfe, R.S., Croft, R.J., 2009. Mobile telephone use is associated with changes in cognitive function in young adolescents. Bioelectromagnetics 30, 678–686.
Agarwal, A., Desai, N.R., Makker, K., Varghese, A., Mouradi, R., Sabanegh, E., Sharma, R., 2009. Effects of radiofrequency electromagnetic waves (RF-EMW) from cellular phones on human ejaculated semen: an in vitro pilot study. Fertil. Steril. 92, 1318–1325.
Akdag, M.Z., Dasdag, S., Canturk, F., Karabulut, D., Caner, Y., Adalier, N., 2016. Does prolonged radiofrequency radiation emitted from Wi-Fi devices induce DNA damage in various tissues of rats? J. Chem. Neuroanat. 75, 116–122.
Al-Bayyari, N., 2017. The effect of cell phone usage on semen quality and fertility among Jordanian males. Middle East Fertil. Soc. J. 22, 178–182.
Al-Damegh, M.A., 2012. Rat testicular impairment induced by electromagnetic radiation from a conventional cellular telephone and the protective effects of the antioxidants vitamins C and E. Clinics 67, 785–792.
Al-Serori, H., Kundi, M., Ferk, F., Misik, M., Nersesyan, A., Murbach, M., Lah, T.T., Knasmuller, S., 2017. Evaluation of the potential of mobile phone specific electromagnetic fields (UMTS) to produce micronuclei in human glioblastoma cell lines. Toxicol. Vitro : An Int. J. Publ. Assoc. BIBRA 40, 264–271.
Anderson, L.E., 1993. Biological effects of extremely low-frequency electromagnetic fields: in vivo studies. Am. Ind. Hyg. Assoc. J. 54, 186–196.
Arendash, G.W., Mori, T., Dorsey, M., Gonzalez, R., Tajiri, N., Borlongan, C., 2012. Electromagnetic treatment to old Alzheimer's mice reverses beta-amyloid deposition, modifies cerebral blood flow, and provides selected cognitive benefit. PLoS One 7 e35751.
Avendano, C., Mata, A., Sarmiento, C.A.S., Doncel, G.F., 2012. Use of laptop computers connected to internet through Wi-Fi decreases human sperm motility and increases sperm DNA fragmentation. Fertil. Steril. 97, 39–U93.
Bajic, V., Bajic, B., Milicevic, Z., Ristic, S., Nikolau, A., 2009. Oxidative stress is reduced in wistar rats exposed to smoke from tobacco and treated with specific broad-band pulse electromagnetic fields. Arch. Biol. Sci. 61, 353–366.
Bernabo, N., Tettamanti, E., Russo, V., Martelli, A., Turriani, M., Mattoli, M., Barboni, B., 2010. Extremely low frequency electromagnetic field exposure affects fertilization outcome in swine animal model. Theriogenology 73, 1293–1305.
Blank, M., Goodman, R., 2008. A mechanism for stimulation of biosynthesis by electromagnetic fields: charge transfer in DNA and base pair separation. J. Cell. Physiol. 214, 20–26.
Buldak, R.J., Polaniak, R., Buldak, L., Zwirska-Korczala, K., Skonieczna, M., Monsiol, A., Kukla, M., Dulawa-Buldak, A., Birkner, E., 2012. Short-term exposure to 50 Hz ELF-EMF alters the cisplatin-induced oxidative response in AT478 murine squamous cell carcinoma cells. Bioelectromagnetics 33, 641–651.
Callaghan, M.J., Chang, E.I., Seiser, N., Aarabi, S., Ghali, S., Kinnucan, E.R., Simon, B.J., Gurtner, G.C., 2008. Pulsed electromagnetic fields accelerate normal and diabetic wound healing by increasing endogenous FGF-2 release. Plast. Reconstr. Surg. 121, 130–141.
Campisi, A., Gulino, M., Acquaviva, R., Bellia, P., Raciti, G., Grasso, R., Musumeci, F., Vanella, A., Triglia, A., 2010. Reactive oxygen species levels and DNA fragmentation on astrocytes in primary culture after acute exposure to low intensity microwave electromagnetic field. Neurosci. Lett. 473, 52–55.
Capelli, E., Torrisi, F., Venturini, L., Granato, M., Fassina, L., Lupo, G.F.D., Ricevuti, G., 2017. Low-frequency pulsed electromagnetic field is able to modulate miRNAs in an experimental cell model of Alzheimer's disease. J. Healthc. Eng. 10, 2530270.
Ceccarelli, G., Bloise, N., Mantelli, M., Gastaldi, G., Fassina, L., De Angelis, M.G., Ferrari, D., Imbriani, M., Visai, L., 2013. A comparative analysis of the in vitro effects of pulsed electromagnetic field treatment on osteogenic differentiation of two different mesenchymal cell lineages. BioResearch Open Access 2, 283–294.
Celik, S., Aridogan, I.A., Izol, V., Erdogan, S., Polat, S., Doran, S., 2012. An evaluation of the effects of long-term cell phone use on the testes via light and electron microscope analysis. Urology 79, 346–350.
Chavdoula, E.D., Panagopoulos, D.J., Margaritis, L.H., 2010. Comparison of biological effects between continuous and intermittent exposure to GSM-900-MHz mobile phone radiation: detection of apoptotic cell-death features. Mutat. Res. 700, 51–61.
Chen, Y.C., Chen, C.C., Tu, W., Cheng, Y.T., Tseng, F.G., 2010. Design and fabrication of a microplatform for the proximity effect study of localized ELF-EMF on the growth of in vitro HeLa and PC-12 cells. J. Micromech. Microeng. 20.
Consales, C., Merla, C., Marino, C., Benassi, B., 2012. Electromagnetic fields, oxidative stress, and neurodegeneration. International journal of cell biology 2012, 683897.
Cooke, R., Laing, S., Swerdlow, A.J., 2010. A case-control study of risk of leukaemia in relation to mobile phone use. Br. J. Canc. 103, 1729–1735.
D'Silva, M.H., Swer, R.T., Anbalagan, J., Rajesh, B., 2017. Effect of radiofrequency radiation emitted from 2G and 3G cell phone on developing liver of chick embryo - a comparative study. J. Clin. Diagn. Res. : J. Clin. Diagn. Res. 11, AC05–AC09.
Danese, E., Lippi, G., Buonocore, R., Benati, M., Bovo, C., Bonaguri, C., Salvagno, G.L., Brocco, G., Roggenbuck, D., Montagnana, M., 2017. Mobile phone radiofrequency exposure has no effect on DNA double strand breaks (DSB) in human lymphocytes. Ann. Transl. Med. 5, 272.
De Carlo, F., Ledda, M., Pozzi, D., Pierimarchi, P., Zonfrillo, M., Giuliani, L., D'Emilia, E., Foletti, A., Scorretti, R., Grimaldi, S., Lisi, A., 2012. Nonionizing radiation as a noninvasive strategy in regenerative medicine: the effect of Ca(2+)-ICR on mouse skeletal muscle cell growth and differentiation. Tissue Eng. Part A 18 (21-22), 2248–2258.
De Iuliis, G.N., Newey, R.J., King, B.V., Aitken, R.J., 2009. Mobile phone radiation induces reactive oxygen species production and DNA damage in human

spermatozoa in vitro. PLoS One 4.
De Mattei, M., Varani, K., Masieri, F.F., Pellati, A., Ongaro, A., Fini, M., Cadossi, R., Vincenzi, F., Borea, P.A., Caruso, A., 2009. Adenosine analogs and electromagnetic fields inhibit prostaglandin E-2 release in bovine synovial fibroblasts. Osteoarthritis Cartilage 17, 252–262.
de Oliveira, G.C., Maia, G.A.S., Cortes, V.F., Santos, H.D., Moreira, L.M., Barbosa, L.A., 2013. The effect of gamma-radiation on the hemoglobin of stored red blood cells: the involvement of oxidative stress in hemoglobin conformation. Ann. Hematol. 92, 899–906.
Desai, N.R., Kesari, K.K., Agarwal, A., 2009. Pathophysiology of cell phone radiation: oxidative stress and carcinogenesis with focus on male reproductive system. Reprod. Biol. Endocrinol. : RBE (Rev. Bras. Entomol.) 7, 114.
Du, X.G., Xu, S.S., Chen, Q., Lu, D.Q., Xu, Z.P., Zeng, Q.L., 2008. [Effects of 50 Hz magnetic fields on DNA double-strand breaks in human lens epithelial cells], Zhejiang da xue xue bao. Yi xue ban = J. Zhejiang Unive. Med. Sci. 37, 9–14.
Duan, W., Liu, C., Zhang, L., He, M., Xu, S., Chen, C., Pi, H., Gao, P., Zhang, Y., Zhong, M., Yu, Z., Zhou, Z., 2015. Comparison of the genotoxic effects induced by 50 Hz extremely low-frequency electromagnetic fields and 1800 MHz radiofrequency electromagnetic fields in GC-2 cells. Radiat. Res. 183, 305–314.
Erdal, N., Gurgul, S., Celik, A., 2007. Cytogenetic effects of extremely low frequency magnetic field on Wistar rat bone marrow. Mutat. Res. Genet. Toxicol. Environ. Mutagen 630, 69–77.
Esmaeili, S.T., Majd, A., Irian, S., Nabiuni, M., Ghahremaninejad, F., 2017. Assessment of DNA damage using random amplified polymorphic DNA in vegetative-stage bean (Phaseolus Vulgaris L.) grown under a low frequency electromagnetic field. Appl. Ecol. Environ. Res. 15, 729–739.
Fagua, A.L.F., Abril, R.N.P., Casas, J.D.R., 2016. The adverse effects over human health caused by wireless wi-fi communication networks. Cultura Cientifica 34–45.
Fejes, I., Zavaczki, Z., Szollosi, J., Koloszar, S., Daru, J., Kovacs, L., Pal, A., 2005. Is there a relationship between cell phone use and semen quality? Arch. Androl. 51, 385–393.
Feng, B., Ye, C., Qiu, L., Chen, L., Fu, Y., Sun, W., 2016. Mitochondrial ROS release and subsequent Akt activation potentially mediated the anti-apoptotic effect of a 50-Hz magnetic field on FL cells. Cell. Physiol. Biochem. : Int. J. Exp. Cell. Physiol. Biochem. Pharmacol. 38, 2489–2499.
Ferreira, A.R., Bonatto, F., de Bittencourt Pasquali, M.A., Polydoro, M., Dal-Pizzol, F., Fernandez, C., de Salles, A.A., Moreira, J.C., 2006. Oxidative stress effects on the central nervous system of rats after acute exposure to ultra high frequency electromagnetic fields. Bioelectromagnetics 27, 487–493.
Fini, M., Giavaresi, G., Torricelli, P., Cavani, F., Setti, S., Cane, V., Giardino, R., 2005. Pulsed electromagnetic fields reduce knee osteoarthritic lesion progression in the aged Dunkin Hartley Guinea pig. J. Orthop. Res. 23, 899–908.
Forgacs, Z., Somosy, Z., Kubinyi, G., Bakos, J., Hudak, A., Surjan, A., Thuroczy, G., 2006. Effect of whole-body 1800 MHz GSM-like microwave exposure on testicular steroidogenesis and histology in mice. Reprod. Toxicol. 22, 111–117.
Fragopoulou, A.F., Samara, A., Antonelou, M.H., Xanthopoulou, A., Papadopoulou, A., Vougas, K., Koutsogiannopoulou, E., Anastasiadou, E., Stravopodis, D.J., Tsangaris, G.T., Margaritis, L.H., 2012. Brain proteome response following whole body exposure of mice to mobile phone or wireless DECT base radiation. Electromagn. Biol. Med. 31, 250–274.
Franzellitti, S., Valbonesi, P., Ciancaglini, N., Biondi, C., Contin, A., Bersani, F., Fabbri, E., 2010. Transient DNA damage induced by high-frequency electromagnetic fields (GSM 1.8 GHz) in the human trophoblast HTR-8/SVneo cell line evaluated with the alkaline comet assay. Mutat. Res. 683, 35–42.
Gao, Q.H., Cai, Q., Fan, Y.N., 2017. Beneficial effect of catechin and epicatechin on cognitive impairment and oxidative stress induced by extremely low frequency electromagnetic field. J. Food Biochem. 41.
Garaj-Vrhovac, V., Gajski, G., Pazanin, S., Sarolic, A., Domijan, A.M., Flajs, D., Peraica, M., 2011. Assessment of cytogenetic damage and oxidative stress in personnel occupationally exposed to the pulsed microwave radiation of marine radar equipment. Int. J. Hyg Environ. Health 214, 59–65.
Giorgi, G., Marcantonio, P., Bersani, F., Gavoci, E., Del Re, B., 2011. Effect of extremely low frequency magnetic field exposure on DNA transposition in relation to frequency, wave shape and exposure time. Int. J. Radiat. Biol. 87, 601–608.
Gomez-Ochoa, I., Gomez-Ochoa, P., Gomez-Casal, F., Cativiela, E., Larrad-Mur, L., 2011. Pulsed electromagnetic fields decrease proinflammatory cytokine secretion (IL-1beta and TNF-alpha) on human fibroblast-like cell culture. Rheumatol. Int. 31, 1283–1289.
Greenland, S., Sheppard, A.R., Kaune, W.T., Poole, C., Kelsh, M.A., 2000. A pooled analysis of magnetic fields, wire codes, and childhood leukemia. Childhood Leukemia-EMF Study Group. Epidemiology 11, 624–634.
Guler, G., Tomruk, A., Ozgur, E., Sahin, D., Sepici, A., Altan, N., Seyhan, N., 2012. The effect of radiofrequency radiation on DNA and lipid damage in female and male infant rabbits. Int. J. Radiat. Biol. 88, 367–373.
Hagras, A.M., Toraih, E.A., Fawzy, M.S., 2016. Mobile phones electromagnetic radiation and NAD+-dependent isocitrate dehydrogenase as a mitochondrial marker in asthenozoospermia. Biochimie open 3, 19–25.
Hardell, L., 2017. World Health Organization, radiofrequency radiation and health - a hard nut to crack (Review). Int. J. Oncol. 51, 405–413.
Hardell, L., Soderqvist, F., Carlberg, M., Zetterberg, H., Mild, K.H., 2010. Exposure to wireless phone emissions and serum beta-trace protein. Int. J. Mol. Med. 26, 301–306.
Heredia-Rojas, J.A., Rodriguez-De La Fuente, A.O., del Roble Velazco-Campos, M., Leal-Garza, C.H., Rodriguez-Flores, L.E., de La Fuente-Cortez, B., 2001. Cytological effects of 60 Hz magnetic fields on human lymphocytes in vitro: sister-chromatid exchanges, cell kinetics and mitotic rate. Bioelectromagnetics 22, 145–149.
Hone, P., Lloyd, D., Szluinska, M., Edwards, A., 2006. Chromatid damage in human lymphocytes is not affected by 50 Hz electromagnetic fields. Radiat. Protect. Dosim. 121, 321–324.
Hong, R., Zhang, Y., Liu, Y., Weng, E.Q., 2005. Effects of extremely low frequency electromagnetic fields on DNA of testicular cells and sperm chromatin structure in mice. Zhonghua lao dong wei sheng zhi ye bing za zhi = Zhonghua laodong weisheng zhiyebing zazhi = Chin. J. Ind. Hyg. Occup. Dis. 23, 414–417.
Iorio, R., Delle Monache, S., Bennato, F., Di Bartolomeo, C., Scrimaglio, R., Cinque, B., Colonna, R.C., 2011. Involvement of mitochondrial activity in mediating ELF-EMF stimulatory effect on human sperm motility. Bioelectromagnetics 32, 15–27.
Jemal, A., Siegel, R., Xu, J., Ward, E., 2010. Cancer statistics, 2010. CA: A Cancer J. Clin. 60 (5), 277–300.
Jeong, Y.J., Kang, G.Y., Kwon, J.H., Choi, H.D., Pack, J.K., Kim, N., Lee, Y.S., Lee, H.J., 2015. 1950 MHz electromagnetic fields ameliorate abeta pathology in Alzheimer's disease mice. Curr. Alzheimer Res. 12, 481–492.
Johnson, M.T., Vanscoy-Cornett, A., Vesper, D.N., Swez, J.A., Chamberlain, J.K., Seaward, M.B., Nindl, G., 2001. Electromagnetic fields used clinically to improve bone healing also impact lymphocyte proliferation in vitro. Biomed. Sci. Instrum. 37, 215–220.
Jouni, F.J., Abdolmaleki, P., Ghanati, F., 2012. Oxidative stress in broad bean (Vicia faba L.) induced by static magnetic field under natural radioactivity. Mutat. Res. 741, 116–121.
Jurcevic, M., Malaric, K., 2016. Assessment of wi-fi radiation on human health. In: 2016 24th International Conference on Software, Telecommunications and Computer Networks (Softcom), pp. 122–125.
Kapi, E., Bozkurt, M., Selcuk, C.T., Celik, M.S., Akpolat, V., Isik, F.B., Bozarslan, B.H., Celik, Y., 2015. Comparison of effects of pulsed electromagnetic field stimulation on platelet-rich plasma and bone marrow stromal stem cell using rat zygomatic bone defect model. Ann. Plast. Surg. 75, 565–571.
Kaszuba-Zwoinska, J., Cwiklinska, M., Balwierz, W., Chorobik, P., Nowak, B., Wojcik-Piotrowicz, K., Ziomber, A., Malina-Novak, K., Zaraska, W., Thor, P.J., 2015. Changes in cell death of peripheral blood lymphocytes isolated from children with acute lymphoblastic leukemia upon stimulation with 7 Hz, 30 Mt pulsed electromagnetic field. Cell. Mol. Biol. Lett. 20, 130–142.
Kesari, K.K., Juutilainen, J., Luukkonen, J., Naarala, J., 2016. Induction of micronuclei and superoxide production in neuroblastoma and glioma cell lines exposed to weak 50 Hz magnetic fields. J. R. Soc. Interface 13, 20150995.
Khalil, A.M., Qassem, W., 1991. Cytogenetic effects of pulsing electromagnetic field on human lymphocytes in vitro: chromosome aberrations, sister-chromatid exchanges and cell kinetics. Mutat. Res. 247, 141–146.
Kheifets, L., Crespi, C.M., Hooper, C., Cockburn, M., Amoon, A.T., Vergara, X.P., 2017. Residential magnetic fields exposure and childhood leukemia: a population-based case-control study in California. Canc. Causes Contr. : CCC (Cancer Causes Control) 28, 1117–1123.
Kheifets, L., Shimkhada, R., 2005. Childhood leukemia and EMF: review of the epidemiologic evidence. Bioelectromagnetics. Suppl 7, S51–S59.
Kirschenlohr, H., Ellis, P., Hesketh, R., Metcalfe, J., 2012. Gene expression profiles in white blood cells of volunteers exposed to a 50 Hz electromagnetic field. Radiat. Res. 178, 138–149.
Kocaman, A., Altun, G., Kaplan, A.A., Deniz, O.G., Yurt, K.K., Kaplan, S., 2018. Genotoxic and carcinogenic effects of non-ionizing electromagnetic fields. Environ. Res. 163, 71–79.
Kovacs, E., Keresztes, A., 2002. Effect of gamma and UV-B/C radiation on plant cells. Micron 33, 199–210.
Koyu, A., Ozguner, F., Yilmaz, H.R., Uz, E., Cesur, G., Ozcelik, N., 2009. The protective effect of caffeic acid phenethyl ester (CAPE) on oxidative stress in rat liver exposed to the 900 MHz electromagnetic field. Toxicol. Ind. Health 25, 429–434.
Lai, H., Singh, N.P., 1997. Melatonin and N-tert-butyl-alpha-phenylnitrone block 60-Hz magnetic field-induced DNA single and double strand breaks in rat brain cells. J. Pineal Res. 22, 152–162.
Lai, H., Singh, N.P., 2004. Magnetic-field-induced DNA strand breaks in brain cells of the rat. Environ. Health Perspect. 112, 687–694.
Langer, C.E., de Llobet, P., Dalmau, A., Wiart, J., Goedhart, G., Hours, M., Benke, G.P., Bouka, E., Bruchim, R., Choi, K.-H., Eng, A., Ha, M., Karalexi, M., Kiyohara, K., Kojimahara, N., Krewski, D., Kromhout, H., Lacour, B.t, Mannetje, A., Maule, M., Migliore, E., Mohipp, C., Momoli, F., Petridou, E., Radon, K., Remen, T., Sadetzki, S., Sim, M.R., Weinmann, T., Vermeulen, R., Cardis, E., Vrijheid, M., 2017. Patterns of cellular phone use among young people in 12 countries: implications for RF exposure. Environ. Int. 107, 65–74.
Lantow, M., Lupke, M., Frahm, J., Mattsson, M.O., Kuster, N., Simko, M., 2006. ROS release and Hsp70 expression after exposure to 1,800 MHz radiofrequency electromagnetic fields in primary human monocytes and lymphocytes. Radiat. Environ. Biophys. 45, 55–62.
Lasalvia, M., Scrima, R., Perna, G., Piccoli, C., Capitanio, N., Biagi, P.F., Schiavulli, L., Ligonzo, T., Centra, M., Casamassima, G., Ermini, A., Capozzi, V., 2018. Exposure to 1.8 GHz electromagnetic fields affects morphology, DNA-related Raman spectra and mitochondrial functions in human lympho-monocytes. PLoS One 13.
Levitt, B.B., Lai, H., 2011. Biological effects from exposure to electromagnetic radiation emitted by cell tower base stations and other antenna arrays (vol 18, pg 369, 2010). Environ. Rev. 19, 495–495.

Luo, F., Hou, T.Y., Zhang, Z.H., Xie, Z., Wu, X.H., Xu, J.Z., 2012. Effects of pulsed electromagnetic field frequencies on the osteogenic differentiation of human mesenchymal stem cells. Orthopedics 35, E526–E531.

Luukkonen, J., Hakulinen, P., Maki-Paakkanen, J., Juutilainen, J., Naarala, J., 2009. Enhancement of chemically induced reactive oxygen species production and DNA damage in human SH-SY5Y neuroblastoma cells by 872 MHz radiofrequency radiation. Mutat. Res. Fund Mol. Mech. Mutagen 662, 54–58.

Luukkonen, J., Hoyto, A., Sokka, M., Liimatainen, A., Syvaoja, J., Juutilainen, J., Naarala, J., 2017. Modification of p21 level and cell cycle distribution by 50Hz magnetic fields in human SH-SY5Y neuroblastoma cells. Int. J. Radiat. Biol. 93, 240–248.

Manjhi, J., Kumar, S., Behari, J., Mathur, R., 2013. Effect of extremely low frequency magnetic field in prevention of spinal cord injury-induced osteoporosis. J. Rehabil. Res. Dev. 50, 17–29.

Manta, A.K., Stravopodis, D.J., Papassideri, I.S., Margaritis, L.H., 2014. Reactive oxygen species elevation and recovery in Drosophila bodies and ovaries following short-term and long-term exposure to DECT base EMF. Electromagn. Biol. Med. 33 (2), 118–131.

Mashevich, M., Folkman, D., Kesar, A., Barbul, A., Korenstein, R., Jerby, E., Avivi, L., 2003. Exposure of human peripheral blood lymphocytes to electromagnetic fields associated with cellular phones leads to chromosomal instability. Bioelectromagnetics 24, 82–90.

Mazor, R., Korenstein-Ilan, A., Barbul, A., Eshet, Y., Shahadi, A., Jerby, E., Korenstein, R., 2008. Increased levels of numerical chromosome aberrations after in vitro exposure of human peripheral blood lymphocytes to radiofrequency electromagnetic fields for 72 hours. Radiat. Res. 169, 28–37.

Mendling, W., Haller, I., 1977. Effect of therapeutic doses of gamma-radiation on Candida-Albicans cells invitro. Geburtshilfe Frauenheilkd 37, 947–951.

Menon, R., Taylor, R.N., Urrabaz-Garza, R., Kechichian, T., Syed, T.A., Papaconstantinou, J., Saade, G., Boldogh, I., 2013. Reactive oxygen species (ROS) induce DNA damage and senescence in human amniochorionic membranes and amnion cells. Reprod. Sci. 20, 239a-239a.

Mihai, C.T., Rotinberg, P., Brinza, F., Vochita, G., 2014. Extremely low-frequency electromagnetic fields cause DNA strand breaks in normal cells. J. Environ. Health Sci. Eng. 12, 15.

Miyagi, N., Sato, K., Rong, Y., Yamamura, S., Katagiri, H., Kobayashi, K., Iwata, H., 2000. Effects of PEMF on a murine osteosarcoma cell line: drug-resistant (P-glycoprotein-positive) and non-resistant cells. Bioelectromagnetics 21, 112–121.

Morberg, B.M., Malling, A.S., Jensen, B.R., Gredal, O., Bech, P., Wermuth, L., 2017. Parkinson's disease and transcranial pulsed electromagnetic fields: a randomized clinical trial. Mov. Disord. 32, 625–626.

Nittby, H., Brun, A., Eberhardt, J., Malmgren, L., Persson, B.R., Salford, L.G., 2009. Increased blood-brain barrier permeability in mammalian brain 7 days after exposure to the radiation from a GSM-900 mobile phone. Pathophysiol : Offic. J. Int. Soc. Pathophysiol. 16, 103–112.

Nylund, R., Leszczynski, D., 2006. Mobile phone radiation causes changes in gene and protein expression in human endothelial cell lines and the response seems to be genome- and proteome-dependent. Proteomics 6, 4769–4780.

Odaci, E., Hanci, H., Yulug, E., Turedi, S., Aliyazicioglu, Y., Kay, H., Colakoglu, S., 2016. Effects of prenatal exposure to a 900 MHz electromagnetic field on 60-day-old rat testis and epididymal sperm quality. Biotech. Histochem. 91, 9–19.

Ongaro, A., Varani, K., Masieri, F.F., Pellati, A., Massari, L., Cadossi, R., Vincenzi, F., Borea, P.A., Fini, M., Caruso, A., De Mattei, M., 2012. Electromagnetic fields (EMFs) and adenosine receptors modulate prostaglandin E2 and cytokine release in human osteoarthritic synovial fibroblasts. J. Cell. Physiol. 227, 2461–2469.

Ozguner, M., Koyu, A., Cesur, G., Ural, M., Ozguner, F., Gokcimen, A., Delibas, N., 2005. Biological and morphological effects on the reproductive organ of rats after exposure to electromagnetic field. Saudi Med. J. 26, 405–410.

Ozlem Nisbet, H., Nisbet, C., Akar, A., Cevik, M., Karayigit, M.O., 2012. Effects of exposure to electromagnetic field (1.8/0.9 GHz) on testicular function and structure in growing rats. Res. Vet. Sci. 93, 1001–1005.

Panagopoulos, D.J., 2012. Effect of microwave exposure on the ovarian development of Drosophila melanogaster. Cell Biochem. Biophys. 63, 121–132.

Panagopoulos, D.J., Chavdoula, E.D., Nezis, I.P., Margaritis, L.H., 2007. Cell death induced by GSM 900-MHz and DCS 1800-MHz mobile telephony radiation. Mutat. Res. 626, 69–78.

Park, J.E., Seo, Y.K., Yoon, H.H., Kim, C.W., Park, J.K., Jeon, S., 2013. Electromagnetic fields induce neural differentiation of human bone marrow derived mesenchymal stem cells via ROS mediated EGFR activation. Neurochem. Int. 62, 418–424.

Phillips, J.L., Singh, N.P., Lai, H., 2009. Electromagnetic fields and DNA damage. Pathophysiol : Offic. J. Int. Soc. Pathophysiol/ISP 16, 79–88.

REFLEX, 2004. Project: Risk Evaluation of Potential Environmental Hazards from Low Energy Electromagnetic Fields (Emf) Exposure Using Sensitive in Vitro Methods QLK4-CT-1999–19901574; FP5 Thematic Programme Quality of Life and Management of Living Resources.

Reid, A., Glass, D.C., Bailey, H.D., Milne, E., de Klerk, N.H., Downie, P., Fritschi, L., Aus, A.L.L.C., Armstrong, B.K., Milne, E., van Bockxmeer, F.M., Haber, M., Scott, R.J., Attia, J., Norris, M.D., Bower, C., de Klerk, N.H., Fritschi, L., Kees, U.R., Miller, M., Thompson, J.R., Bailey, H.D., Alvaro, F., Cole, C., Dalla Pozza, L., Daubenton, J., Downie, P., Lockwood, L., Kirby, M., Marshall, G., Smibert, E., Suppiah, R., 2011a. Risk of childhood acute lymphoblastic leukaemia following parental occupational exposure to extremely low frequency electromagnetic fields. Br. J. Canc. 105, 1409–1413.

Reid, A., Glass, D.C., Bailey, H.D., Milne, E., de Klerk, N.H., Downie, P., Fritschi, L., Consortium, A.-A, 2011b. Risk of childhood acute lymphoblastic leukaemia following parental occupational exposure to extremely low frequency electromagnetic fields. Br. J. Canc. 105, 1409–1413.

Remondini, D., Nylund, R., Reivinen, J., Poulletier de Gannes, F., Veyret, B., Lagroye, I., Haro, E., Trillo, M.A., Capri, M., Franceschi, C., Schlatterer, K., Gminski, R., Fitzner, R., Tauber, R., Schuderer, J., Kuster, N., Leszczynski, D., Bersani, F., Maercker, C., 2006. Gene expression changes in human cells after exposure to mobile phone microwaves. Proteomics 6, 4745–4754.

Rodriguez-Garcia, J.A., Ramos, F., 2012. High incidence of acute leukemia in the proximity of some industrial facilities in el Bierzo, Northwestern Spain. Int. J. Occup. Med. Environ. Health 25, 22–30.

Rosado, M.M., Simkó, M., Mattsson, M.-O., Pioli, C., 2018. Immune-modulating perspectives for low frequency electromagnetic fields in innate immunity. Frontiers in Public Health 6.

Ruediger, H.W., 2009. Genotoxic effects of radiofrequency electromagnetic fields. Pathophysiol : Offic. J. Int. Soc. Pathophysiol/ISP 16, 89–102.

Safian, F., Khalili, M.A., Khoradmehr, A., Anbari, F., Soltani, S., Halvaei, I., 2016. Survival assessment of mouse preimplantation embryos after exposure to cell phone radiation. J. Reproduction Infertil. 17, 138–143.

Sagar, S., Adem, S.M., Struchen, B., Loughran, S.P., Brunjes, M.E., Arangua, L., Dalvie, M.A., Croft, R.J., Jerrett, M., Moskowitz, J.M., Kuo, T., Roosli, M., 2018. Comparison of radiofrequency electromagnetic field exposure levels in different everyday microenvironments in an international context. Environ. Int. 114, 297–306.

Saliev, T., Mustapova, Z., Kulsharova, G., Bulanin, D., Mikhalovsky, S., 2014a. Therapeutic potential of electromagnetic fields for tissue engineering and wound healing. Cell Prolif 47, 485–493.

Saliev, T., Tachibana, K., Bulanin, D., Mikhalovsky, S., Whitby, R.D., 2014b. Bio-effects of non-ionizing electromagnetic fields in context of cancer therapy. Front. Biosci. 6, 175–184.

Sandyk, R., 1996. Electromagnetic fields for treatment of multiple sclerosis. Int. J. Neurosci. 87, 1–4.

Sannino, A., Zeni, O., Romeo, S., Massa, R., Scarfi, M.R., 2017. Adverse and beneficial effects in Chinese hamster lung fibroblast cells following radiofrequency exposure. Bioelectromagnetics 38, 245–254.

Schuz, J., 2011. Exposure to extremely low-frequency magnetic fields and the risk of childhood cancer: update of the epidemiological evidence. Prog. Biophys. Mol. Biol. 107, 339–342.

Schuz, J., Ahlbom, A., 2008. Exposure to electromagnetic fields and the risk of childhood leukaemia: a review. Radiat. Protect. Dosim. 132, 202–211.

Schwarz, C., Kratochvil, E., Pilger, A., Kuster, N., Adlkofer, F., Rudiger, H.W., 2008. Radiofrequency electromagnetic fields (UMTS, 1,950 MHz) induce genotoxic effects in vitro in human fibroblasts but not in lymphocytes. Int. Arch. Occup. Environ. Health 81, 755–767.

Shahin, S., Singh, S.P., Chaturvedi, C.M., 2017. Mobile phone (1800MHz) radiation impairs female reproduction in mice, Mus musculus, through stress induced inhibition of ovarian and uterine activity. Reprod. Toxicol. 73, 41–60.

Stronati, L., Testa, A., Moquet, J., Edwards, A., Cordelli, E., Villani, P., Marino, C., Fresegna, A.M., Appolloni, M., Lloyd, D., 2006. 935 MHz cellular phone radiation. An in vitro study of genotoxicity in human lymphocytes. Int. J. Radiat. Biol. 82, 339–346.

Sudan, M., Olsen, J., Arah, O.A., Obel, C., Kheifets, L., 2016. Prospective cohort analysis of cellphone use and emotional and behavioural difficulties in children. J. Epidemiol. Community 70, 1207–1213.

Sun, C., Wei, X., Fei, Y., Su, L., Zhao, X., Chen, G., Xu, Z., 2016. Mobile phone signal exposure triggers a hormesis-like effect in Atm+/+ and Atm-/- mouse embryonic fibroblasts. Sci. Rep. 18, 6–37423.

Suzuki, S., Okutsu, M., Suganuma, R., Komiya, H., Nakatani-Enomoto, S., Kobayashi, S., Ugawa, Y., Tateno, H., Fujimori, K., 2017. Influence of radiofrequency-electromagnetic waves from 3rd-generation cellular phones on fertilization and embryo development in mice. Bioelectromagnetics 38, 466–473.

Tabrizi, M.M., Hosseini, S.A., 2015. Role of electromagnetic field exposure in childhood acute lymphoblastic leukemia and No impact of urinary alpha- amylase–a case control study in tehran, Iran. Asian Pac. J. Cancer Prev : Asian Pac. J. Cancer Prev. APJCP 16, 7613–7618.

Tas, M., Dasdag, S., Akdag, M.Z., Cirit, U., Yegin, K., Seker, U., Ozmen, M.F., Eren, L.B., 2013. Long-term effects of 900 MHz radiofrequency radiation emitted from mobile phone on testicular tissue and epididymal semen quality. Electromagn. Biol. Med. 33 (3), 216–222.

Tatarov, I., Panda, A., Petkov, D., Kolappaswamy, K., Thompson, K., Kavirayani, A., Lipsky, M.M., Elson, E., Davis, C.C., Martin, S.S., DeTolla, L.J., 2011. Effect of magnetic fields on tumor growth and viability. Comp. Med. 61, 339–345.

Teepen, J.C., van Dijck, J.A.A.M., 2012. Impact of high electromagnetic field levels on childhood leukemia incidence. Int. J. Canc. 131, 769–778.

Terwilliger, T., Abdul-Hay, M., 2017. Acute lymphoblastic leukemia: a comprehensive review and 2017 update. Blood Canc. J. 7, e577.

Tkalec, M., Malaric, K., Pevalek-Kozlina, B., 2007. Exposure to radiofrequency radiation induces oxidative stress in duckweed Lemna minor L. Sci. Total Environ. 388, 78–89.

Tsybulin, O., Sidorik, E., Kyrylenko, S., Henshel, D., Yakymenko, I., 2012. GSM 900 MHz microwave radiation affects embryo development of Japanese quails. Electromagn. Biol. Med. 31, 75–86.

Turedi, S., Hanci, H., Colakoglu, S., Kaya, H., Odaci, E., 2016. Disruption of the ovarian

follicle reservoir of prepubertal rats following prenatal exposure to a continuous 900-MHz electromagnetic field. Int. J. Radiat. Biol. 92, 329–337.
Ursache, M., Mindru, G., Creanga, D.E., Tufescu, F.M., Goiceanu, C., 2009. The effects of high frequency electromagnetic waves on the vegetal organisms. Rom. J. Phys. 54, 133–145.
Uzunboy, S., Cekic, S.D., Apak, R., 2016. Determination of reactive oxygen species induced dna damage using modified cupric reducing antioxidant capacity (CUPRAC) colorimetric method. FEBS J. 283, 397–398.
Vadala, M., Morales-Medina, J.C., Vallelunga, A., Palmieri, B., Laurino, C., Iannitti, T., 2016. Mechanisms and therapeutic effectiveness of pulsed electromagnetic field therapy in oncology. Cancer medicine 5, 3128–3139.
Valera, E.T., Brassesco, M.S., Tone, L.G., 2014. Electromagnetic fields at extremely low frequencies and the risk for childhood leukemia: do we have enough information to warrant this association? Leuk. Res. 38, 289.
Varani, K., Gessi, S., Merighi, S., Iannotta, V., Cattabriga, E., Spisani, S., Cadossi, R., Andrea, B.P., 2002. British J. Pharmacol. 136 (1), 57–66.
Wahab, M.A., Podd, J.V., Rapley, B.I., Rowland, R.E., 2007. Elevated sister chromatid exchange frequencies in dividing human peripheral blood lymphocytes exposed to 50 Hz magnetic fields. Bioelectromagnetics 28, 281–288.
Wang, J., An, Y.X., Li, F.J., Li, D.M., Jing, D., Guo, T.W., Luo, E.P., Ma, C.F., 2014. The effects of pulsed electromagnetic field on the functions of osteoblasts on implant surfaces with different topographies. Acta Biomater. 10, 975–985.
Wartenberg, D., 2001. Residential EMF exposure and childhood leukemia: meta-analysis and population attributable risk. Bioelectromagnetics. Suppl 5, S86–S104.
Wdowiak, A., Wdowiak, L., Wiktor, H., 2007. Evaluation of the effect of using mobile phones on male fertility. Ann. Agric. Environ. Med. : AAEM 14, 169–172.
Wells, P.G., Miller-Pinsler, L., Bhatia, S., Drake, D., Shapiro, A.M., 2015. Reactive oxygen species (ROS) formation, oxidative DNA damage and repair in teratogenesis. Birth Defects Res. Part A Clin. Mol. Teratol. 103, 359–359.
Williams, P.A., Ingebretsen, R.J., Dawson, R.J., 2006. 14.6 mT ELF magnetic field exposure yields no DNA breaks in model system Salmonella, but provides evidence of heat stress protection. Bioelectromagnetics 27, 445–450.
Woelders, H., de Wit, A., Lourens, A., Stockhofe, N., Engel, B., Hulsegge, I., Schokker, D., van Heijningen, P., Vossen, S., Bekers, D., Zwamborn, P., 2017. Study of potential health effects of electromagnetic fields of telephony and wi-fi, using chicken embryo development as animal model. Bioelectromagnetics 38, 186–203.
Xie, Y.F., Shi, W.G., Zhou, J., Gao, Y.H., Li, S.F., Fang, Q.Q., Wang, M.G., Ma, H.P., Wang, J.F., Xian, C.J., Chen, K.M., 2016. Pulsed electromagnetic fields stimulate osteogenic differentiation and maturation of osteoblasts by upregulating the expression of BMPRII localized at the base of primary cilium. Bone 93, 22–32.
Xu, S., Chen, G., Chen, C., Sun, C., Zhang, D., Murbach, M., Kuster, N., Zeng, Q., Xu, Z., 2013. Cell type-dependent induction of DNA damage by 1800 MHz radiofrequency electromagnetic fields does not result in significant cellular dysfunctions. PLoS One 8 e54906.
Xu, Z.Z., Fu, W.B., Jin, Z., Guo, P., Wang, W.F., Li, J.M., 2016. Reactive oxygen species mediate oridonin-induced apoptosis through DNA damage response and activation of JNK pathway in diffuse large B cell lymphoma. Leuk. Lymphoma 57, 888–898.
Yang, Y., Jin, X., Yan, C., Tian, Y., Tang, J., Shen, X., 2008. Case-only study of interactions between DNA repair genes (hMLH1, APEX1, MGMT, XRCC1 and XPD) and low-frequency electromagnetic fields in childhood acute leukemia. Leuk. Lymphoma 49, 2344–2350.
Yokus, B., Cakir, D.U., Akdag, M.Z., Sert, C., Mete, N., 2005. Oxidative DNA damage in rats exposed to extremely low frequency electromagnetic fields. Free Radic. Res. 39, 317–323.
Zhang, S.Z., Yao, G.D., Lu, D.Q., Chiang, H., Xu, Z.P., 2008. [Effect of 1.8 GHz radiofrequency electromagnetic fields on gene expression of rat neurons]. Zhonghua lao dong wei sheng zhi ye bing za zhi = Zhonghua laodong weisheng zhiyebing zazhi = Chin. J. Ind. Hyg. Occup. Dis. 26, 449–452.
Zhao, J., Freeman, B., Li, M., 2016. Can mobile phone apps influence People's health behavior Change? An evidence review. J. Med. Internet Res. 18.
Zhong, C., Zhang, X., Xu, Z.J., He, R.X., 2012. Effects of low-intensity electromagnetic fields on the proliferation and differentiation of cultured mouse bone marrow stromal cells. Phys. Ther. 92, 1208–1219.
Zimmerman, J.W., Jimenez, H., Pennison, M.J., Brezovich, I., Morgan, D., Mudry, A., Costa, F.P., Barbault, A., Pasche, B., 2013. Targeted treatment of cancer with radiofrequency electromagnetic fields amplitude-modulated at tumor-specific frequencies. Chin. J. Canc. 32, 573–581.
Zimmerman, J.W., Pennison, M.J., Brezovich, I., Yi, N., Yang, C.T., Ramaker, R., Absher, D., Myers, R.M., Kuster, N., Costa, F.P., Barbault, A., Pasche, B., 2012. Cancer cell proliferation is inhibited by specific modulation frequencies. Br. J. Canc. 106, 307–313.
Zothansiama, Zosangzuali, M., Lalramdinpuii, M., Jagetia, G.C., 2017. Impact of radiofrequency radiation on DNA damage and antioxidants in peripheral blood lymphocytes of humans residing in the vicinity of mobile phone base stations. Electromagn. Biol. Med. 36, 295–305.
Zou, J., Chen, Y., Qian, J., Yang, H., 2017. Effect of a low-frequency pulsed electromagnetic field on expression and secretion of IL-1β and TNF-α in nucleus pulposus cells. J. Int. Med. Res. 45, 462–470.
