# Supplementary material for: Magnetic Fields and Cancer: Epidemiology, Cellular Biology, and Theranostics
Source: Int J Mol Sci. 2022 Jan 25;23(3):1339. doi: 10.3390/ijms23031339 (PMC8835851; doi:10.3390/ijms23031339)
Supplement: Supplementary file 1 [file ijms-23-01339-s001.zip › Supplementary Data Set S1/MF and Cancer.Data/PDF/0537169225/ajpregu.00280.2002.pdf]

Am J Physiol Regul Integr Comp Physiol 284: R1529–R1535, 2003;
10.1152/ajpregu.00280.2002.

Magnetic fields and the melatonin hypothesis: a study of workers chronically exposed to 50-Hz magnetic fields

Yvan Touitou,[1] Jacques Lambrozo,[2] Françoise Camus,[1] and Henriette Charbuy[1]
[1]*Department of Biochemistry and Molecular Biology, Faculty of Medicine Pitié-Salpêtrière, 75013 Paris; and* [2]*Service des Etudes Médicales, Electricité de France/Gaz de France, 75009 Paris, France*

Submitted 17 May 2002; accepted in final form 13 February 2003

Touitou, Yvan, Jacques Lambrozo, Françoise Camus, and Henriette Charbuy. Magnetic fields and the melatonin hypothesis: a study of workers chronically exposed to 50-Hz magnetic fields. *Am J Physiol Regul Integr Comp Physiol* 284: R1529–R1535, 2003; 10.1152/ajpregu.00280.2002.—Because epidemiological studies report clinical disorders (mainly neurobehavioral alterations and/or cancer) that may be related to diminished melatonin secretion or to changes in its circadian rhythm in subjects living or working in environments exposed to magnetic fields, research on the effects of these fields in humans is particularly important. In this study, we examine the circadian rhythm of melatonin in 15 men exposed chronically and daily for a period of 1–20 yr, in the workplace and at home, to a 50-Hz magnetic field in search of any cumulative effect from those chronic conditions of exposure. The weekly geometric mean of individual exposures ranged from 0.1 to 2.6 μT. The results are compared with those for 15 unexposed men who served as controls (individual exposures ranged from 0.004 to 0.092 μT). Blood samples were taken hourly from 2000 to 0800. Nighttime urine was also collected and analyzed. This work shows that subjects exposed over a long period (up to 20 yr) and on a daily basis to magnetic fields experienced no changes in their plasma melatonin level, their urinary 6-sulfatoxymelatonin level, or the circadian rhythm of melatonin. Our data strongly suggest that magnetic fields do not have cumulative effects on melatonin secretion in humans and thus clearly rebut the "melatonin hypothesis" that a decrease in plasma melatonin concentration (or a disruption in its secretion) explains the occurrence of clinical disorders or cancers possibly related to magnetic fields.

melatonin; magnetic fields; 6-sulfatoxymelatonin; circadian rhythms.

BECAUSE ELECTRICITY APPLICATIONS are omnipresent, especially in the industrialized countries, exposure to electric and magnetic fields generated by the production, transport, and distribution of electricity (50 Hz in Europe, 60 Hz in North America) is ubiquitous. These fields are also encountered in most activities of daily life (lighting, heating, and other household applications of electricity). Light, that part of the electromagnetic spectrum with a wavelength between 730 and 400 nm, has been proven to inhibit the normal nocturnal secretion of melatonin by the light-sensitive pineal gland (12, 29). Because light is only the visible portion of the electromagnetic spectrum, other wavelengths may also inhibit melatonin secretion.

Thus most experimental studies of rats exposed to electric or magnetic fields have found a diminution in melatonin secretion (27, 32, 37, 38, 45, 46, 48, 49, 57). Similarly, Yellon and Truong (61) found that a brief exposure to magnetic fields 2 h before the onset of darkness blunted or delayed the nocturnal increase of melatonin in Djungarian hamsters but this effect was dependent on the exposure parameters (54, 62). Moreover, the importance of the length of magnetic field exposure in this inhibitory effect suggests that the effect of these fields on pineal function may be cumulative, at least in rats (46). In fact, much of the evidence for the melatonin hypothesis is based on data for rodents (with 25–40% reduction of the hormone, see review in Ref. 35). However, humans and rodents differ in regard to melatonin secretion as follows: rodents are nocturnally active, and they show differences in the anatomical location of the pineal gland and the geometry of the skull that may cause stronger eddy currents in field-exposed animals.

Results about the effects of magnetic fields in higher mammals (lambs and monkeys; see Refs. 26, 40, 41) and humans have been either negative or provide controversial results. Most work published so far has involved the acute exposure of healthy volunteers to magnetic fields and has not found it to affect melatonin secretion (2, 5, 6, 8, 14–18, 20, 24, 25, 34, 44, 60). It is nonetheless possible that chronic human exposure to magnetic fields might affect melatonin secretion, its circadian rhythm, or both. There are obvious technical difficulties in exposing healthy volunteers to magnetic fields for a long period, at a high intensity, or both. Thus the only feasible experimental approach toward the study of such chronic exposure involves the study of subjects exposed continually either on the job or at home (occupationally or residentially). We performed such a study among workers exposed to magnetic fields daily for 1 to 20 years, both in the workplace and at home.

Address for reprint requests and other correspondence: Y. Touitou, Dept. of Biochemistry and Molecular Biology, Faculty of Medicine Pitié-Salpétrière, 91, boulevard de l'Hôpital, 75013 Paris, France (E-mail: touitou@ccr.jussieu.fr).

The costs of publication of this article were defrayed in part by the payment of page charges. The article must therefore be hereby marked "*advertisement*" in accordance with 18 U.S.C. Section 1734 solely to indicate this fact.

 0363-6119/03 $5.00 Copyright © 2003 the American Physiological Society

Downloaded from journals.physiology.org/journal/ajpregu (002.235.186.236) on November 19, 2021.

MATERIALS AND METHODS

Subjects

Subject characteristics. Thirty male volunteers (15 exposed and 15 controls) participated in the study, which took place in the autumn, when the light-dark cycle was 10 h light-14 h dark. All subjects had similar schedules, with daytime activity from 0700 to 2300 and nocturnal rest; they were similar in age (mean age of exposed subjects = 38.0 ± 0.9 yr, range 31.5–46; mean age of control subjects = 39.4 ± 1.2 yr, range 34.5–47) and in physical activity. Subjects meeting the following criteria were eligible for the study: they were required to have no acute or chronic diseases, to have regular sleep habits, to do no night work, to have taken no transmeridian airplane flights during the preceding 2 mo, and to take no drugs. They were nonsmokers who used alcohol and coffee in moderate quantities. The exposed workers had not been on call in the 48 h preceding the experiment. They were asked not to use electric razors or hair dryers during the study or in the 24 h before the blood samples were taken.

All subjects (exposed and control) underwent a routine clinical and laboratory examination to verify that their general health, endocrine profile, and current blood counts were within the normal range. Women were not included in this study because the interaction of their ovarian hormone cycle with melatonin secretion might have made the study results difficult to interpret.

Conditions of the volunteers' magnetic field exposure. These men (n = 15) all worked in extra high voltage (EHV) substations in the Paris metropolitan region, operating and maintaining the EHV electricity transmission network (225 and 400 KV). Their work essentially involved installing couplings between EHV lines as well as voltage transformers. The many electric lines arriving in substations further increase the ambient magnetic field levels there.

The exposed subjects were also housed near the substations, which means that they were exposed to magnetic fields both while working and during their daily life in their lodgings. Ten of the 15 subjects were exposed from 7 to 20 yr, and 5 subjects were exposed from 1 to 4 yr.

Control subjects. The control subjects (n = 15) were recruited by the Centre d'Investigation Clinique (CIC) of Pitié-Salpêtrière Hospital and among the white-collar workers at EDF. None worked in a technical job that could have resulted in occupational exposure to magnetic fields. They were subjected only to the normal electromagnetic fields of our daily environment.

Measurement of Levels of Magnetic Field Exposure

Dosimeters. We used EMDEX II dosimeters (Enertech Consultants) to record the magnetic field exposure for 1 wk for each subject; its range is 10 nT to 300 μT (precision ± 10%) for frequencies between 40 and 800 Hz. The device is autonomous; it measures B magnetic induction in space in all three directions and then calculates the resulting value of B, which is the quadratic sum of each of the three directions

$$B = \sqrt{Bx^2 + By^2 + Bz^2}$$

The value of B thus calculated is then recorded in the device memory.

Dosimetry for exposed and control subjects. Exposure was continuously measured for 7 days, during the daytime and at night. Measurements were taken and recorded every 30 s all day long. Both exposed and control subjects wore the magnetic field recording device throughout the workday. At home, they placed it in a "public" room. Dosimeters were also worn at the CIC. Exposure at the CIC was low, similar to normal residential levels, on the order of 0.05 μT.

Statistical calculations for exposure measurements. Data for both groups were transferred by a serial link to a personal computer after 7 days of dosimeter operation, and the statistical calculations were performed with the EMCALC software furnished with the dosimeters (Enertech). We calculated the following parameters: lowest value, highest value, and weekly arithmetic and geometric means with their SDs and median. These parameters have been calculated for the total exposure (7 days) and for total daytime and nighttime exposure.

Experimental Protocol

The entire protocol was performed in autumn (10:14-h light-dark cycle) at the CIC of Pitié-Salpêtrière Hospital in Paris and was approved by the hospital ethics committee. This paper follows the principles for research involving human beings as published by the American Physiological Society (3a). The subjects arrived at the CIC between 1800 and 1830; the catheter (15 cm long, to avoid awakening subjects for the nocturnal blood samples) was inserted at 1900. The study extended over a 12-h period (from 2000 to 0800 the next morning). The subjects were free until 2200, but, to avoid even a minimal exposure to magnetic fields that might bias the study, they were not allowed to watch television or to play video games. Lights were turned off from 2200 to 0800. Because the CIC could house a maximum of two volunteers a night, the study was staggered over a 5-wk period for the exposed subjects and over 7 wk for the control subjects.

Blood samples were taken hourly from 2000 to 0800 the next morning, that is, 13 samples/subject. To standardize the posture-related sampling conditions, all subjects remained seated for 15 min preceding the samples taken at 2000 and 2100 and were lying down for all the samples thereafter (from 2200 to 0800 the next morning). The nighttime samples were taken under red light to avoid light-induced suppression of melatonin secretion. The blood samples were centrifuged, divided into aliquots, and frozen at −20°C until the assays. Urine was collected in a single 12-h fraction (2000 to 0800) to study the urinary melatonin metabolite, 6-sulfatoxymelatonin; its peak elimination period is the night. These were stored at −20°C until the assay.

Methods

Melatonin and 6-sulfatoxymelatonin assays. The plasma melatonin assays were performed in duplicate with a modified version of the RIA method described by Fraser et al. (13) using a ^{125}I-labeled melatonin tracer with some modifications previously described (55). All assays were performed blinded to the subject's case/control status and in a single series to avoid interassay variability. The intra-assay coefficient of variation was 6.1% (n = 10), for a value of 65 pg/ml. The sensitivity was 5–10 pg/ml.

The urinary 6-sulfatoxymelatonin assay was performed with a kit from Stockgrand. The RIA method used was modified from that described by Arendt et al. (4) with the use of iodinated 6-sulfatoxymelatonin (3). All assays were performed in a single series. The intra-assay coefficient of variation was 6% (n = 10), for a value of 30 ng/ml.

Statistical analysis. All the results obtained were expressed as the means ± SE. The statistical significance of the differences between the control subjects and the exposed subjects was determined with repeated-measures ANOVA. We also used the SAS mixed procedure with repeated mea-

Downloaded from journals.physiology.org/journal/ajpregu (002.235.186.236) on November 19, 2021.

sures and slope change to take into account the nocturnal profile of melatonin secretion (19, 22, 42). The differences were considered to be statistically significant when P was <0.05. The statistical analysis examined the following three factors: the “hour” factor, the “field effect” factor, and the “hour times field” interaction. Because our working hypothesis was that magnetic fields can affect the circadian profile of melatonin, 6-sulfatoxymelatonin, or both, we paid special attention to the “field” effect rather than to the hour factor, which corresponds to the hormone’s circadian variation, as established in several previous studies.

RESULTS

Results of the Magnetic Field Measurements

Exposed subjects. The weekly geometric mean of individual exposures ranged from 0.1 to 2.6 μT. The arithmetic mean of the 15 exposure values was 0.72 μT. The arithmetic mean of daytime (workday) exposure of the 15 exposed subjects was 0.64 μT and nighttime (residential) exposure 0.82 μT.

A subset of three subjects had a substantially higher exposure with a arithmetic mean of 2.10 μT. Their arithmetic mean workday exposure was 1.50 μT and nighttime residential exposure 2.71 μT.

Control subjects. The weekly geometric mean of individual exposures ranged from 0.004 to 0.092 μT. The arithmetic mean of the 15 exposure values was 0.04 μT. The arithmetic mean of both the workday and nighttime exposure for the 15 controls was 0.04 μT.

Comparison of Exposed and Control Subjects

This comparison between groups showed that melatonin secretion did not differ for the exposed and control groups ($n = 15$ in each group).

Figure 1 shows the circadian profile of the plasma melatonin and its urinary metabolite, 6-sulfatoxymelatonin. In both control and exposed subjects, the plasma values were low from 2000 to 2200; they began to increase from 2200 and reached their highest values between 0200 and 0500. Both ANOVA and the SAS mixed procedure showed an effect of time (circadian variation) whatever the condition but no effect of field or field times hour interaction (Table 1).

The 6-sulfatoxymelatonin assay tested all the urine collected by each subject in a single 12-h fraction, corresponding to the period from 2000 to 0800. The concentration of urinary 6-sulfatoxymelatonin did not differ significantly between the chronically exposed (mean = 11.98 ± 2.57 ng/mg creatinine) and the control (mean = 9.94 ± 1.78 ng/mg creatinine) subjects (Fig. 1).

Comparison of Control Subjects With Subjects Exposed to Fields Between 0.1 and 0.3 μT and With Subjects Exposed to Fields >0.3 μT

The comparison of subjects exposed to fields from 0.1 to 0.3 μT ($n = 6$) with controls ($n = 15$) did not show any significant difference between these two groups (Fig. 2). Similarly, the subjects exposed to >0.3 μT ($n = 9$) did not differ significantly from the control subjects

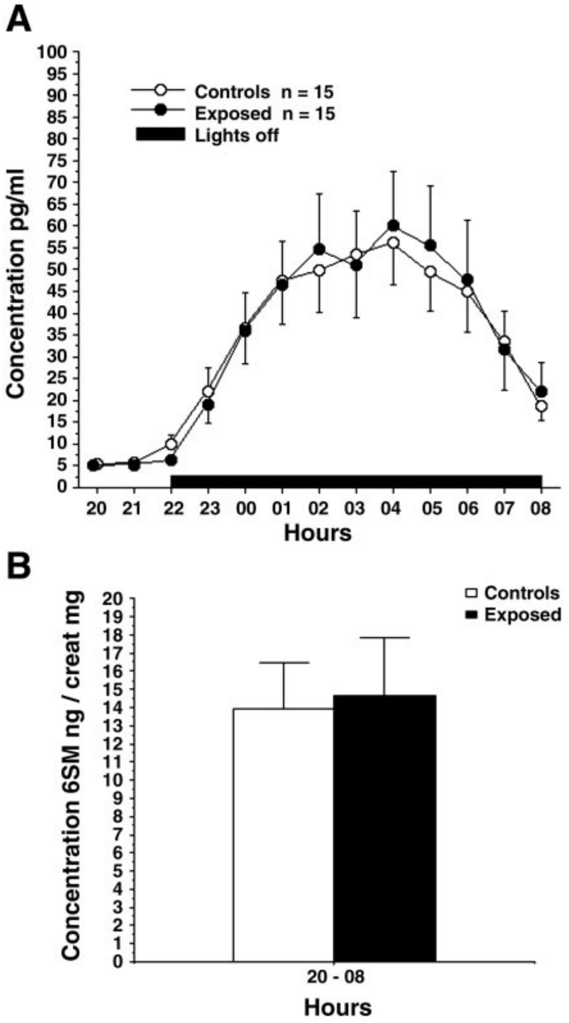

Fig. 1. Comparative nocturnal plasma melatonin profiles (*A*) and 6-sulfatoxymelatonin concentration (6SM; *B*) in the first-void morning urine (2000 to 0800) in 15 chronically exposed men and 15 control subjects. The weekly geometric mean of individual exposures ranged from 0.1 to 2.6 μT.

(Fig. 2) for their nocturnal profiles of melatonin secretion or their plasma concentrations at any of the times studied. ANOVA showed an effect of time but no effect of field or field times hour interaction. Finally, the nocturnal melatonin profiles in the subset of the three very highly exposed subjects (mean exposure: 2.10 μT) did not differ from those of the controls (field: $F = 0.27$, $P = 0.61$; hour: $F = 11.11$, $P < 0.0001$; hour-field interaction: $F = 0.20$, $P = 0.99$).

DISCUSSION

Research into the possible effects of magnetic fields on human melatonin secretion is important from a public health perspective, because alterations in the secretion of this hormone (for example, phase shifting or reduced amplitude) are likely to lead to clinical disorders involving fatigue, sleep and mood disturbances, altered performance, and depression, all signs that can be related to desynchronization of circadian rhythms (7, 56). Some epidemiological studies have

Downloaded from journals.physiology.org/journal/ajpregu (002.235.186.236) on November 19, 2021.

Table 1. *Repeated-measures ANOVA and mixed-procedure analysis on plasma melatonin*

	F	*P*
ANOVA		
Field	0.01	0.91
Hour	26.14	<0.0001
Field × h interaction	0.16	1.00
Mixed Procedure		
Field	0.15	0.6972
Hour (AS)	29.23	<0.0001
Hour (DS)	71.32	<0.0001
Field and hour (AS)	0.06	0.8076
Field and hour (DS)	0.07	0.7891

Comparison between chronically exposed men and control. Statistical analysis examined the following three factors : the hour factor, the field effect factor, and the hour-field interaction. Mixed-procedure takes into account the nocturnal profile of melatonin secretion ascendent slope (AS) and descendent slope (DS).

reported most of these clinical signs in subjects living or working in an environment exposed to magnetic fields (33, 36). Other epidemiological studies have examined the potential link between exposure to 50–60 Hz magnetic fields and the incidence of some types of cancer, leukemia in particular (1, 43, 58, 59), although these results are controversial (11, 30, 31). Because experimental studies have shown that melatonin has oncostatic properties (9, 39, 53), a reduction in its secretion caused by magnetic field exposure may be one possible explanation of epidemiological reports of a relation between magnetic fields and cancer, as hypothesized by Stevens (51) and Stevens and Davis (52).

We have previously shown that 50-Hz magnetic fields reduce melatonin secretion in rats at an intensity of 100 μT when the rats are subjected to acute exposure (12 h) and 10 μT when the exposure is chronic (1 mo). The appearance of an effect with a chronic 10-μT exposure is related to the duration of exposure and may therefore correspond to a cumulative effect of the magnetic fields (46). This effect has been related to diminished pineal *N*-acetyltransferase (NAT) activity (46, 47).

We found different results in humans; in a study of 32 healthy male volunteers aged 20–30 yr and subjected to nocturnal exposure to a 50-Hz (10 μT) magnetic field for 9 h, we observed no effect on the circadian profiles of either serum melatonin or urinary 6-sulfatoxymelatonin (44). Several other authors have also reported that magnetic fields do not affect humans, at least during acute exposure. Most studies of melatonin in humans exposed to magnetic fields have involved acute exposure, including high-intensity exposure (10, 21, 60). They have thus left unanswered the question of effects associated with chronic exposure. Until now, no study looked at serum melatonin values during occupational exposure to a 50-Hz magnetic field or to the harmonics and transient components that may play a biological role. We therefore conducted a study of workers regularly exposed to magnetic fields both at work and at home (EDF electrical workers). The exposed volunteers followed roughly the same schedule of daytime activity (from 0700 to 2300) and nocturnal rest. The weekly mean value of their chronic exposure was 0.72 μT (weekly geometric mean of individual exposures from 0.1 to 2.6 μT), with peak exposures during the workday reaching >100 μT for some of them. The results we obtained here indicate that nocturnal plasma melatonin secretion (sampled between 2000 and 0800) is not different in these workers than in a group of unexposed volunteer control subjects. No differences were noted for either the nocturnal plasma melatonin concentrations or the profile of melatonin secretion. Nor was there any effect on urinary 6-sulfatoxymelatonin, the melatonin metabolite. Nonetheless, it remains possible that responses may differ according to the circadian time of exposure. Our subjects were exposed 24 h daily, during the workday, and at home. This rules out the possibility that there

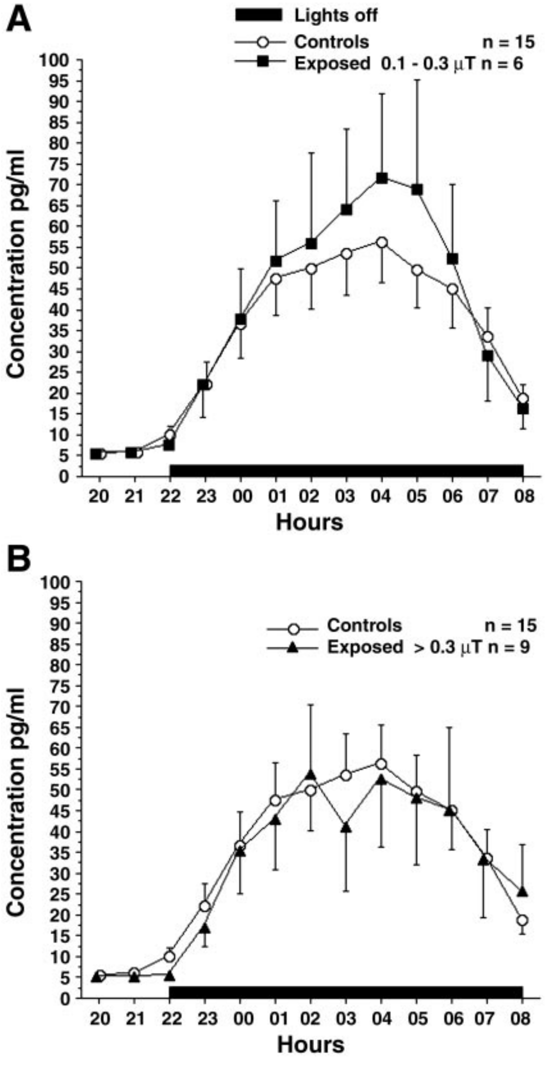

Fig. 2. Comparative nocturnal plasma melatonin profiles in healthy male volunteers chronically exposed to 50-Hz magnetic fields and at an intensity of between 0.1 and 0.3 μT (*A*) or >0.3 μT (*B*) and control subjects. The weekly geometric mean of individual exposures ranged from 0.1 to 2.6 μT.

Downloaded from journals.physiology.org/journal/ajpregu (002.235.186.236) on November 19, 2021.

was a period during the 24-h day during which the subjects were not exposed.

We also note that exposure was more intense at night, because the workers were housed very close to the substation and high voltage lines. The absence of effect on melatonin secretion therefore cannot be related to maximal exposure occurring during the daytime, when the disruptive effect is lessened or lost (as with light). Last, melatonin secretion in the three most highly exposed workers, with more than double the exposure of the others, did not differ from that in the others.

Our study can be compared with that by Wilson et al. (60), in which volunteers were exposed by sleeping under an electric blanket, and to the recent study by Hong et al. (21), which exposed nine male volunteers to an electric sheet for 11 wk with a median exposure at the level of the head of 0.70 μT. No changes in the level of urinary melatonin were observed. In another study in 39 women working in the garment industry (mean exposure from 0.3 to 1 μT), the authors observed a variation of 6-sulfatoxymelatonin during the workday but no relation between exposure and effect (23). They did not adjust for age or smoking, each of which could have explained the modifications. Finally, for residential exposure, Levallois et al. (28) did not find any modification in urinary excretion of 6-sulfatoxymelatonin in women living near electricity transmission lines, except in overweight women and in the oldest subjects. Some different results on residential exposure have been reported by Davis et al. (10), mostly in women using medications and only during the summer months.

In conclusion, this study of workers exposed daily to magnetic fields for a period of 1–20 yr in their workplace and at home shows that this exposure does not lead to alterations in their melatonin secretion. Ten of the 15 subjects were exposed from 7 to 20 yr, and 5 subjects were exposed from 1 to 4 yr. The clinical signs (depression, mood and sleep disorders, malignant diseases, etc.) reported in some studies of people living or working near electric lines or substations thus do not appear to be associated with a disturbance in their melatonin levels. It is possible that the difference in the effects observed in animals and humans is the result of both the anatomical configuration of the pineal gland and the principally nocturnal rhythm of rodent activity. A different sensitivity to magnetic fields between species could also be part of the explanation, as it is known that some species detect and perceive magnetic fields differently (50). It is also possible that some subjects are more sensitive to magnetic fields than others; this is very difficult to demonstrate in a case-control study because of the enormous interindividual variability of melatonin secretion and plasma melatonin concentrations in humans. To our knowledge, this study is the first to examine both plasma melatonin circadian rhythm and urinary 6-sulfatoxymelatonin concentration in subjects who have been exposed chronically and for a long period (1–20 yr) to magnetic fields at home and at work; it is thus the first to show that chronic magnetic field exposure appears to have no cumulative effect in humans on serum melatonin secretion and circadian rhythm or urinary excretion of 6-sulfatoxymelatonin, at least in men in their forties.

This protocol was performed at the Centre d'Investigation Clinique (CIC) of Pitié-Salpêtrière Hospital. We thank the nurses of the CIC, the occupational physicians at EDF (Drs. B. Boudin, P. Blaise, and A. Milliani) and their nurses (C. Renault and D. Pottier), who participated in this study, and Drs. M. Souques and F. Wallet for help.

REFERENCES

1. **Ahlbom A, Day N, Feychting M, Roman E, Skinner J, Dockerty J, Linet M, McBride M, Michaelis J, Olsen JH, Tynes T, and Verkasalo PK.** A pooled analysis of magnetic fields and childhood leukemia. *Br J Cancer* 83: 692–698, 2000.
2. **Akerstedt T, Arnetz B, Ficca G, and Paulsson LE.** Low frequency electromagnetic fields suppress SWS (Abstract). *J Sleep Res* 26: 260, 1997.
3. **Aldhous ME and Arendt J.** Radioimmunoassay for 6-sulphatoxymelatonin in urine and iodinated tracer. *Ann Clin Biochem* 25: 298–303, 1988.

3a. **American Physiological Society.** Guiding principles for research involving animals, and human beings. *Am J Physiol Regul Integr Comp Physiol* 283: R281–R283, 2002.

4. **Arendt J, Bojkowski C, Franey C, Wright J, and Marks V.** Immunoassay of 6-hydroxymelatonin sulfate in human plasma and urine: abolition of the urinary 24-hour rhythm with atenolol. *J Clin Endocrinol Metab* 60: 1166–1173, 1985.
5. **Arnetz BB and Berg M.** Melatonin and adrenocorticotropic hormone levels in video display unit workers during work and leisure. *J Occup Environ Med* 38: 1108–1110, 1996.
6. **Burch JB, Reif JS, Yost MG, Keefe TJ, and Pitrat CA.** Nocturnal excretion of a urinary melatonin metabolite in electric utility workers. *Scand J Work Environ Health* 24: 183–189, 1998.
7. **Claustrat B, Chazot G, and Brun J.** A chronobiological study of melatonin and cortisol secretion in depressed subjects: plasma melatonin, a biochemical marker in major depression. *Biol Psychiatry* 19: 1215–1228, 1984.
8. **Crasson M, Beckers V, Pequeux Ch, Claustrat B, and Legros JJ.** Daytime 50 Hz magnetic field exposure and plasma melatonin and urinary 6-sulfatoxymelatonin concentration profiles in humans. *J Pineal Res* 31: 234–241, 2001.
9. **Das Gupta TK and Terz J.** Influence of the pineal gland on growth and spread of melatonin in the hamster. *Cancer Res* 27: 1306–1311, 1967.
10. **Davis S, Kaune WT, Mirick DK, Chen C, and Stevens RG.** Residential magnetic fields, light-at-night, and nocturnal urinary 6-sulfatoxymelatonin concentration in women. *Am J Epidemiol* 154: 591–600, 2001.
11. **Day N and UK Childhood Cancer Study Investigators.** Exposure to power-frequency magnetic fields and the risk of childhood cancer. *Lancet* 354: 1925–1931, 1999.
12. **Depres-Brummer P, Levi F, Metzger G, and Touitou Y.** Light-induced suppression of the rat circadian system. *Am J Physiol Regul Integr Comp Physiol* 268: R1111–R1116, 1995.
13. **Fraser S, Cowen P, Franklin M, Franey C, and Arendt J.** Direct radioimmunoasssay for melatonin in plasma. *Clin Chem 29*: 396–397, 1983.
14. **Graham C, Cook MR, Gerkovich MM, and Sastre A.** Melatonin and 6-OHMS in high-intensity magnetic fields. *J Pineal Res* 31: 85–88, 2001.
15. **Graham C, Cook MR, Riffle DW, Gerkovich MM, and Cohen HD.** Nocturnal melatonin levels in human volunteers exposed to intermittent 60 Hz magnetic fields. *Bioelectromagnetics* 17: 263–273, 1996.
16. **Graham C, Cook MR, Riffle DW, Gerkovich MM, and Cohen HD.** Human melatonin during continuous magnetic field exposure. *Bioelectromagnetics* 18: 166–171, 1996.

Downloaded from journals.physiology.org/journal/ajpregu (002.235.186.236) on November 19, 2021.

17. **Graham C, Cook MR, Sastre A, Riffle DW, and Gerkovich MM.** Multi-night exposure to 60 Hz magnetic fields: effects on melatonin and its enzymatic metabolite. *J Pineal Res* 28: 1–8, 2000.
18. **Graham C, Sastre A, Cook MR, and Gerkovich MM.** All-night exposure to EMF does not alter urinary melatonin, 6-OHMS or immune measures in older men and women. *J Pineal Res* 31: 109–113, 2001.
19. **Harville DA.** *Mixed Models Methodology: Theoretical Justifications and Future Directions. Proceedings of the Statistical Computing Section.* New Orleans, LA: Am Stat Assoc, 1988, p. 41–49.
20. **Haugsdal B, Tynes T, Rotnes JS, and Griffiths D.** A single nocturnal exposure to 2–7 millitesla static magnetic fields does not inhibit the excretion of 6-sulfatoxymelatonin in healthy young men. *Bioelectromagnetics* 22: 1–6, 2001.
21. **Hong SC, Kurukowa Y, Kabuto M, and Ohtsuka R.** Chronic exposure to ELF magnetic fields during night sleep with electric sheet: effects on diurnal melatonin rhythms in men. *Bioelectromagnetics* 22: 138–143, 2001.
22. **Jennrich RI and Schluchter MD.** Unbalanced repeated-measures models with structured covariance matrice. *Biometrics* 42: 805–820, 1986.
23. **Juutilainen J, Stevens RG, Anderson LE, Hansen NH, Kilpelainen M, Kumlin T, Laitinen JT, Sobel E, and Wilson BW.** Nocturnal 6-hydroxymelatonin sulfate excretion in female workers exposed to magnetic fields. *J Pineal Res* 28: 97–104, 2000.
24. **Kaune W, Davis S, and Stevens R.** Relation between residential magnetic fields, light-at-night, and nocturnal urine melatonin levels in women. *EPRI Rep* TR-107242-VE1, 1997, p. 188.
25. **Kumlin T, Hansen NH, Kilpelainen M, Kukkonen S, Laitinen J, Stevens RW, Wilson BW, and Juutilainen J.** *Biological Effects of LF EMF*. Oslo, Norway: Norwegian Radiation Protection Authority, 1997, p. 67–68.
26. **Lee JM, Stormshak F, Thompson JM, Hess DL, and Foster DL.** Melatonin and puberty in female lambs exposed to EMF: a replicate study. *Bioelectromagnetics* 16: 119–123, 1995.
27. **Lerchl A, Nonaka KO, Stokkan KA, and Reiter RJ.** Marked rapid alteration in nocturnal pineal serotonin metabolism in mice and rats exposed to weak intermittent magnetic field. *Biochem Biophys Res Commun* 169: 102–108, 1990.
28. **Levallois P, Dumont M, Touitou Y, Gingras S, Masse B, Gauvin D, Kröger E, Bourdages M, and Douville P.** Effects of electric and magnetic fields from high-power lines on female urinary excretion of 6-sulfatoxymelatonin. *Am J Epidemiol* 154: 601–609, 2001.
29. **Lewy AJ, Wehr TA, Goodwin FK, Newsome DA, and Markey SP.** Light suppresses melatonin secretion in humans. *Science* 210: 1267–1269, 1980.
30. **Linet MS, Hatch EE, Kleinerman RA, Robison LL, Kaune WT, Friedman DR, Severson RK, Haines CM, Hartsock CT, Niwa S, Wacholder S, and Tarone RE.** Residential exposure to magnetic fields and acute lymphoblastic leukemia in children. *N Engl J Med* 337: 1–7, 1997.
31. **McBride ML, Gallagher RP, Theriault G, Armstrong BG, Tamaro S, Spinelli JJ, Deadman JE, Fincham S, Robson D, and Choi W.** Power-frequency electric and magnetic fields and risk of childhood leukemia in Canada. *Am J Epidemiol* 149: 831–842, 1999.
32. **Olcese J and Reuss S.** Magnetic fields on pineal gland melatonin synthesis: comparative studies on albino and pigmented rodents. *Brain Res* 369: 365–368, 1986.
33. **Perry FS and Pearl L.** Power frequency magnetic field and illness in multistory blocks. *Public Health* 102: 11–18, 1988.
34. **Pfluger DH and Minder CE.** Effects of exposure to 16.7 Hz magnetic fields on urinary 6-hydroxymelatonin sulfate excretion of Swiss railway workers. *J Pineal Res* 21: 91–100, 1996.
35. **Portier CJ and Wolfe MS.** *Assessment of Health Effects From Exposure to Power-line Frequency Electric, and Magnetic Fields*. Bethesda, MD: NIH, 1988, p. 244–255. (Working Group Rep. 98–3981)
36. **Reichmanis M, Perry FS, Marino AA, and Becker RO.** Relation between suicide and the electromagnetic field of overhead power lines. *Physiol Chem Phys Med NMR* 11: 395–403, 1979.
37. **Reiter RJ, Anderson LE, Buschbom RL, and Wilson BW.** Reduction of the nocturnal rise in pineal melatonin levels in rats exposed to 60-Hz electric fields in utero and for 23 days after birth. *Life Sci* 42: 2203–2206, 1988.
38. **Reuss S and Olcese J.** Magnetic field effects on the rat pineal gland: role of retinal activation by light. *Neurosci Lett* 64: 97–101, 1986.
39. **Rodin AE.** The growth and spread of walker 256 carcinoma in pinealectomized rats (Abstract). *Cancer Res* 23: 1545, 1963.
40. **Rogers WR, Reiter RJ, Barlow-Walden L, Smith HD, and Orr JL.** Regularly scheduled, day-time, slow-onset 60 Hz electric and magnetic field exposure does not depress serum melatonin concentration in nonhuman primates. *Bioelectromagnetics Suppl* 3: 111–118, 1995.
41. **Rogers WR, Reiter RJ, Smith HD, and Barlow-Walden L.** Rapid-onset/offset, variably scheduled 60 Hz electric and magnetic field exposure reduces nocturnal serum melatonin concentration in nonhuman primates. *Bioelectromagnetics Suppl* 3: 119–122, 1995.
42. **SAS Institute.** *SAS/STAT Software: Changes and Enhancements Through Release.* Cary, NC: SAS Institute, 1996.
43. **Savitz DA, Wachtel H, Barnes FA, John EM, and Tvrdik JG.** Case-control study of childhood cancer and exposure to 60 Hz magnetic fields. *Am J Epidemiol* 128: 21–38, 1988.
44. **Selmaoui B, Lambrozo J, and Touitou Y.** Magnetic fields and pineal function in humans: evaluation of nocturnal acute exposure to extremely low frequency magnetic fields on serum melatonin and urinary 6-sulfatoxy-melatonin circadian rhythm. *Life Sci* 58: 1539–1549, 1996.
45. **Selmaoui B, Lambrozo J, and Touitou Y.** Assessment of nocturnal exposure to 50-Hz magnetic fields on the human circadian system: a comprehensive study of biochemical variables. *Chronobiol Int* 16: 789–810, 1999.
46. **Selmaoui B and Touitou Y.** Sinusoidal 50-Hz magnetic fields depress rat pineal function: role of duration and intensity of exposure. *Life Sci* 57: 1351–1358, 1995.
47. **Selmaoui B and Touitou Y.** Age-related differences in serum melatonin and pineal NAT activity and in the response of rat pineal to a 50-Hz magnetic field. *Life Sci* 64: 2291–2297, 1999.
48. **Selmaoui B and Touitou Y.** *The Pineal Gland and Cancer: Neuroimmunoendocrine Mechanisms in Malignancy*. New York, NY: Springer, 2000, p. 534–540.
49. **Semm P.** Neurobiological investigation of the magnetic sensitivity of the pineal gland in rodents and pigeons. *Comp Biochem Physiol A* 76: 683–689, 1983.
50. **Semm P.** The magnetic detection system of the pigeon: involvement of pineal and retical photoreceptors and the vestibular system. In: *Electromagnetic Fields and Neurobehavioral Function*, edited by O'Conner ME and Lovely RH. New York: Liss, 1988.
51. **Stevens RG.** Breast cancer and electric power. *Biomed Pharmacother* 47: 435–438, 1993.
52. **Stevens RG and Davis S.** The melatonin hypothesis: electric power and breast cancer. *Environ Health Perspect* 104, *Suppl* 1: 135–140, 1996.
53. **Tamarkin L, Cohen M, Roselle D, Reichert C, Lippman M, and Chabner B.** Melatonin inhibition and pinealectomy enhancement of 7,12-dimethyl-benz(a)anthracene-induced mammary tumors in the rat. *Cancer Res* 41: 4432–4436, 1981.
54. **Truong H and Yellon SM.** Effect of various acute 60 Hz magnetic field exposures on the nocturnal melatonin rise in the adult Djungarian hamster. *J Pineal Res* 22: 177–183, 1997.
55. **Webley GE, Mehl H, and Willey KP.** Validation of a sensitive direct assay for melatonin for investigation of circadian rhythms in different species. *J Endocrinol* 106: 387–394, 1985.
56. **Wehr TA and Godwin FK.** *American Handbook of Psychiatry* (2nd ed.). New York: Basic Books, 1981, vol. 7, p. 46–74.
57. **Welker HA, Semm P, Willig RP, Commentz JC, Wiltschko W, and Vollrath L.** Effects of an artificial magnetic field on serotonin *N*-acetyltransferase activity and melatonin content in the rat pineal gland. *Exp Brain Res* 50: 426–432, 1983.

Downloaded from journals.physiology.org/journal/ajpregu (002.235.186.236) on November 19, 2021.

58. **Wertheimer N and Leeper E.** Electrical wiring configurations and childhood cancer. *Am J Epidemiol* 109: 273–284, 1979.
59. **Wertheimer N and Leeper E.** Adult cancer related to electrical wires near the home. *Int J Epidemiol* 11: 345–355, 1982.
60. **Wilson BW, Wright CW, Morris JE, Buschdom RL, Brown DP, Miller DL, Sommers-Flannigan R, and Anderson LE.** Evidence for an effect of ELF electromagnetic fields on human pineal gland function. *J Pineal Res* 9: 259–269, 1990.
61. **Yellon SM.** 60-Hz magnetic field exposure effects on the melatonin rhythm in the Djungarian Hamster. In: *The Melatonin Hypothesis In Breast Cancer and Use of Electric Power*, edited by Stevens RG, Wilson BW, and Anderson LE. Columbus, OH: Battelle, 1997, p. 503–526.
62. **Yellon SM and Truong HN.** Melatonin rhythm onset in the adult siberian hamster: influence of photoperiod but not 60-Hz magnetic field exposure on melatonin content in the pineal gland and in circulation. *J Biol Rhythms* 13: 52–59, 1998.

Downloaded from journals.physiology.org/journal/ajpregu (002.235.186.236) on November 19, 2021.
