# Supplementary material for: Magnetic Fields and Cancer: Epidemiology, Cellular Biology, and Theranostics
Source: Int J Mol Sci. 2022 Jan 25;23(3):1339. doi: 10.3390/ijms23031339 (PMC8835851; doi:10.3390/ijms23031339)
Supplement: Supplementary file 1 [file ijms-23-01339-s001.zip › Supplementary Data Set S1/MF and Cancer.Data/PDF/0616825461/bem.20326.pdf]

Bioelectromagnetics 28:546–552 (2007)

Investigation of Protein Expression in Magnetic Field-Treated Human Glioma Cells

M.H. Kanitz,[1]* F.A. Witzmann,[2] W.G. Lotz,[1] D. Conover,[1] and R.E. Savage Jr.[1]

[1]National Institute for Occupational Safety and Health, Cincinnati, Ohio
[2]Indiana University School of Medicine, Department of Cellular and Integrative Physiology, Indianapolis, Indiana

We previously reported phenotypic changes in human breast cancer cells following low-level magnetic field (MF) exposure. Here proteomic methods were used to investigate the biochemical effect of MF exposure in SF767 human glioma cells. Protein alterations were studied after exposure to 1.2 microTesla (μT) MF [12 milliGauss (mG), 60 Hertz (Hz)] ± epidermal growth factor (EGF). SF767 cells were exposed for 3 h to sham conditions (<0.2 μT ambient field strength) or 1.2 μT MF (±EGF; 10 ng/ml). Solubilized protein fractions (sham; 1.2 μT; sham + EGF; 1.2 μT + EGF) were loaded for electrophoresis by 2D-PAGE and stained using a colloidal Coomassie blue technique to resolve and characterize the proteins. Protein patterns were compared across groups via Student's *t*-test using PDQUEST software. Cell profiles revealed significant alterations in the spot density of a subset of treated cells. Automated spot excision and processing was performed prior to peptide mass fingerprinting proteins of interest. Fifty-seven proteins from the detectable pool were identified and/or found to differ significantly across treatment groups. The mean abundance of 10 identified proteins was altered following 1.2 μT exposure. In the presence of EGF six proteins were altered after low magnetic field treatment by increasing (4) or decreasing (2) in abundance. The results suggest that the analysis of differentially expressed proteins in SF767 cells may be useful as biomarkers for biological changes caused by exposure to magnetic fields. Bioelectromagnetics 28:546–552, 2007. © 2007 Wiley-Liss, Inc.

Key words: ELF magnetic field; glioma; proteomics; two-dimensional gel electrophoresis; protein expression

INTRODUCTION

Magnetic fields (MF) at occupational levels have been implicated in human cancer risk. Epidemiology studies have suggested that magnetic fields may play a causal role in cancer incidence [Feychting et al., 1998; Pollan and Gustavsson, 1999]. Feychting [1998] reported an increase in incidence among women with estrogen receptor positive breast cancer after exposure to magnetic fields. Environmental magnetic fields have been reported to inhibit the in vitro antiproliferative activity of tamoxifen in human breast cancer cells [Harland and Liburdy, 1997]. Accordingly, the National Institute for Occupational Safety and Health (NIOSH) has been engaged in studies of the biochemical effects of extremely low frequency magnetic fields. We have previously reported the effect of low-level fields on protein expression in MCF-7 human breast cancer cells [Kanitz et al., 1998].

We continue these investigations by studying protein profiles in magnetic field-treated human glioma cells. It has been demonstrated that a weak magnetic field reversed the cytostatic action of tamoxifen in SF-767, a human brain tumor line of glial cell origin [Liburdy et al., 1993]. An alteration in cell growth is a fundamental biological change usually accompanied by significant changes in protein expression. We hypothesize that treatment of the glioma cells with occupational-level magnetic fields may cause protein expression changes corresponding to the observed alteration in cell proliferation. Any observed changes

Disclaimers: Mention of company names and/or products does not constitute endorsement by the Centers for Disease Control and Prevention (CDC). The findings and conclusions in this report are those of the authors and do not necessarily represent the views of the National Institute for Occupational Safety and Health.

*Correspondence to: M.H. Kanitz, MGMT, BHAB, DART, NIOSH, 4676 Columbia Parkway, MS/C-23, Cincinnati, OH 45226-1998. E-mail: mhk2@cdc.gov

Received for review 5 May 2006; Final revision received 13 February 2007

DOI 10.1002/bem.20326
Published online 14 June 2007 in Wiley InterScience (www.interscience.wiley.com).

© 2007 Wiley-Liss, Inc.

[Savage et al., 2005] may then be fully analyzed to determine the possible functional role(s) of putative biomarkers for exposure.

Because quantitative differences in cellular gene expression and protein levels probably are responsible for most of the differences between malignant and normal phenotypes [Nicolson, 1991], high-resolution two-dimensional polyacrylamide gel electrophoresis, 2D-PAGE, represents an excellent tool to investigate protein biomarkers for effects of exposure. This method is widely used for the study of complex protein mixtures, including protein expression analysis during cellular proliferation and cancer development, and it has demonstrated promise in identifying protein biomarkers for the effects of exposure to occupational bladder carcinogens [Kanitz et al., 2004].

Two-dimensional PAGE was used to resolve hundreds of proteins in MF-treated human SF767 and to study putative phenotypic alterations in exposed cells. Gels were analyzed using PDQUEST, a software program which automatically detects specific spots, matches the protein patterns of multiple gels and compares them for significant differences. Peptide mass fingerprinting was then used to identify potential protein biomarkers for effects of exposure of SF767 human glioma cells to low-level magnetic fields. Observed protein changes were analyzed with regard to the possible subcellular functional roles within the magnetic field-exposed glioma cell.

MATERIALS AND METHODS

Glioma Cell Maintenance

Human glioma, SF767, cells (Brain Tumor Research Center Tissue Bank, University of California, San Francisco Department of Neurological Surgery) were maintained in 10% fetal calf serum (FCS)-MEM/HBSS and were transferred weekly at 1:10 (cell suspension to medium) ratio by exposure to trypsin-EDTA.

Magnetic Field Exposure

Cells were exposed for 3 h to magnetic fields (sham or 1.2 μT) concurrently with EGF (10 ng/ml) or cell culture media as a control. These exposures were replicated for a total of three experiments. The Department of Energy regional exposure MF facility (Columbia Magnetics, Kennewick, WA) at NIOSH consists of two concentric Merritt/Helmholtz coil systems, an inner coil for exposure and outer coil to limit stray fields. Computer control determines field configuration and is used to monitor temperature and MF flux density (.1 μT to 1 mT range). Within the coil systems are chambers isolated from exposure and maintained by a modified commercial incubator. The chambers are shielded from electric fields and equipped with magnetic field sensors and CO_2 ports. For sham exposure, double wound coils were energized with opposing current flow so that the net applied magnetic field was zero. Experimental blinding of exposures was used throughout the study.

Sample Preparation/2D-PAGE

SF767 cells were harvested following the three separate exposures, and proteins were resolved by 2D-PAGE using a modification of the method of Anderson [1988]. Briefly, cell pellets were placed in a lysis buffer (pH 9.5) containing 9 M urea (BDH Chemical Ltd., Poole, UK), 4% Igepal CA-630 ([octylphenoxy]polyethoxyethanol) (Sigma Aldrich, St. Louis, MO), 1% DTT (dithiothreitol) (Bio-Rad, Richmond, CA) and 2% ampholytes (pH 8–10.5) (Sigma Aldrich). The cells were solubilized by intermittent sonication and incubated at room temperature for 2 h. Samples in pre-labeled coded tubes were stored at −70 °C until 2D-PAGE. Protein from 2 million cells/sample (~175 μg) was loaded on each of 20 isoelectric focusing (IEF) tube gels (23.5 cm), and these were run simultaneously for 28,500 V h using progressively increasing voltage (500 V for 1 h, 750 V for 1 h, 1000 V for 1 h, and 1350 V for 19.5 h). A gradient casting system was used to prepare large format (25 cm × 22 cm × 1.5 mm) second dimension SDS gradient slab gels (9–19% T). The IEF gels were loaded directly onto the slab gels, electrophoresed at 8 °C for 18 h at 160 V, and stained using a colloidal Coomassie blue technique.

Gel Image Analysis

Stained gels were optically scanned at 200 μm/pixel resolution on a Fluor-S scanner (Bio-Rad), and images were processed using PDQuest software. 2D-PAGE patterns were assembled in matched sets (similar patterns matched across gels) and matched to a reference pattern. A computer composite of protein patterns from all the treatment groups or "master" pattern was used as a landmark dataset. It served as a baseline to which group patterns were compared to each other based on assigned spot numbers. During initial analysis a subgroup of specific protein spots was landmarked on each gel. Subsequently, the software used an automatic program to match additional spots to the master pattern using the manual landmark data as a basis. After the automatic matching, the results were analyzed for quantitative and qualitative differences. 2D-PAGE spot patterns corresponding to each treatment were compared. Spots were judged to be quantitatively different if the intensity distributions

indicated that the difference was statistically significant (as determined using two-tailed Student's t-test). The coefficient of variation (standard deviation/mean) was 15%. Proteins satisfying this criterion were represented as highlighted spots on computer-plotted protein maps and stored as spot populations. To eliminate quantitative differences due to gel loading or staining, groups of gels were normalized or scaled together by a linear procedure based on specific spots. A coordinated decrease or increase in the magnitude of several hundred proteins compared to the master would likely be caused by a difference in total protein applied to the gel. The computer multiplied the spot abundances for each protein pattern by a scaling factor to account for loading differences. This allowed for valid comparisons of protein expression among groups.

Peptide Mass Fingerprinting

Protein spots were excised from the gels robotically using the Protean® 2D Spot Cutter (Bio-Rad, Hercules, CA) and then processed automatically using the multifunctional MassPREP™ Station robot (MicroMass, Manchester, UK). In this automated system, the excised protein spots were de-stained robotically with 25 mM ammonium bicarbonate/50% acetonitrile (Sigma Aldrich) followed by 100% acetonitrile (Sigma Aldrich), reduced with 5 mM DTT in 25 mM ammonium bicarbonate (Sigma Aldrich), and alkylated with 10 mM iodoacetamide in 25 mM ammonium bicarbonate (Sigma Aldrich). The spots were tryptically digested using Promega sequence grade (Bio-Rad), modified trypsin at a final concentration of 26 ng/μl in 30 mM ammonium bicarbonate/0.005% SDS (Sigma Aldrich) in preparation for matrix-assisted laser desorption ionization mass spectrometry (MALDI-MS) of the resulting peptides. The peptides were then eluted, cleaned-up/desalted and robotically spotted on the MALDI-MS sample target along with matrix. The resulting tryptic peptides were analyzed directly by MALDI-MS using the MALDI™ (MicroMass) system by automated acquisition of optimized peptide mass spectra, monoisotopic peptide mass fingerprint determination by online interrogation of multiple FASTA databases. Protein identification was made using this methodology. Protein identity was deemed acceptably robust, though not necessarily conclusive, when the Profound™ Z-score exceeded 1.30, corresponding to the 90th percentile. We further assessed each protein's identification based on the observed correspondence of pI and MW on the calibrated 2D-PAGE gel patterns to those values predicted by Swiss-Prot for each protein so analyzed. If the protein ID failed to meet those three criteria (Z-score, pI and MW) it was not included in the table.

RESULTS

Using 2D-PAGE the treated SF767 cells were studied to automatically match multiple protein gel patterns. This technique resolves hundreds of cellular proteins based on their isoelectric point (pI) and relative molecular weight (M_r). Figure 1 demonstrates the two-dimensional master pattern of solubilized proteins and gives the coordinate position, M_r versus pI, of each protein. The master illustrates the range of spots and serves as a composite of proteins resolved under all treatment conditions. It also serves as a template to match single gel patterns between treatment groups and provide spot numbers for a subset of glioma cell proteins. Spots annotated by SSP, master spot number, were identifiable and/or significantly altered by varying exposures ($P < 0.05, n = 6$, Student's t-test).

After individual gels were matched to the master we used PDQUEST to compare the protein patterns of SF767 cells exposed for 3 h to magnetic fields (sham or 1.2 μT) concurrently with epidermal growth factor, EGF (10 ng/ml) or media control. Statistically significant quantitative differences in matched protein spot expression were determined. Using peptide fingerprinting we found 57 proteins, which were identifiable and/or differed significantly across treatment groups ($P < 0.05$, $n = 6$, Student's t-test). Of this number, 14 proteins were both identifiable and differed significantly across treatment groups. The functional role of these proteins covered a wide range of intracellular activities as discussed below.

The differential expression of SF767 glioma proteins was investigated following low magnetic field or sham exposure for 3 h. Ten identifiable proteins showed altered phenotypic expression after treatment with magnetic field ($P < 0.05$, $n = 6$, Student's t-test; Table 1). Figure 1 demonstrates the relative coordinate positions of these proteins in the master pattern. Following magnetic field treatment three proteins increased and seven decreased in mean abundance ($P < 0.05$, $n = 6$, Student's t-test; Table 1).

We compared changes in SF767 protein expression exposed for 3 h to low magnetic field or sham in the presence of EGF (10 ng/ml). Six proteins were significantly altered after low magnetic field treatment by increasing (4) or decreasing (2) in abundance ($P < 0.05$, $n = 6$, Student's t-test; Table 2).

A shift in pI reflects protein charge modifications or changes in normally expressed micro-heterogeneities and suggests that post-translational protein alterations may have occurred. However, no shifts in pI were observed.

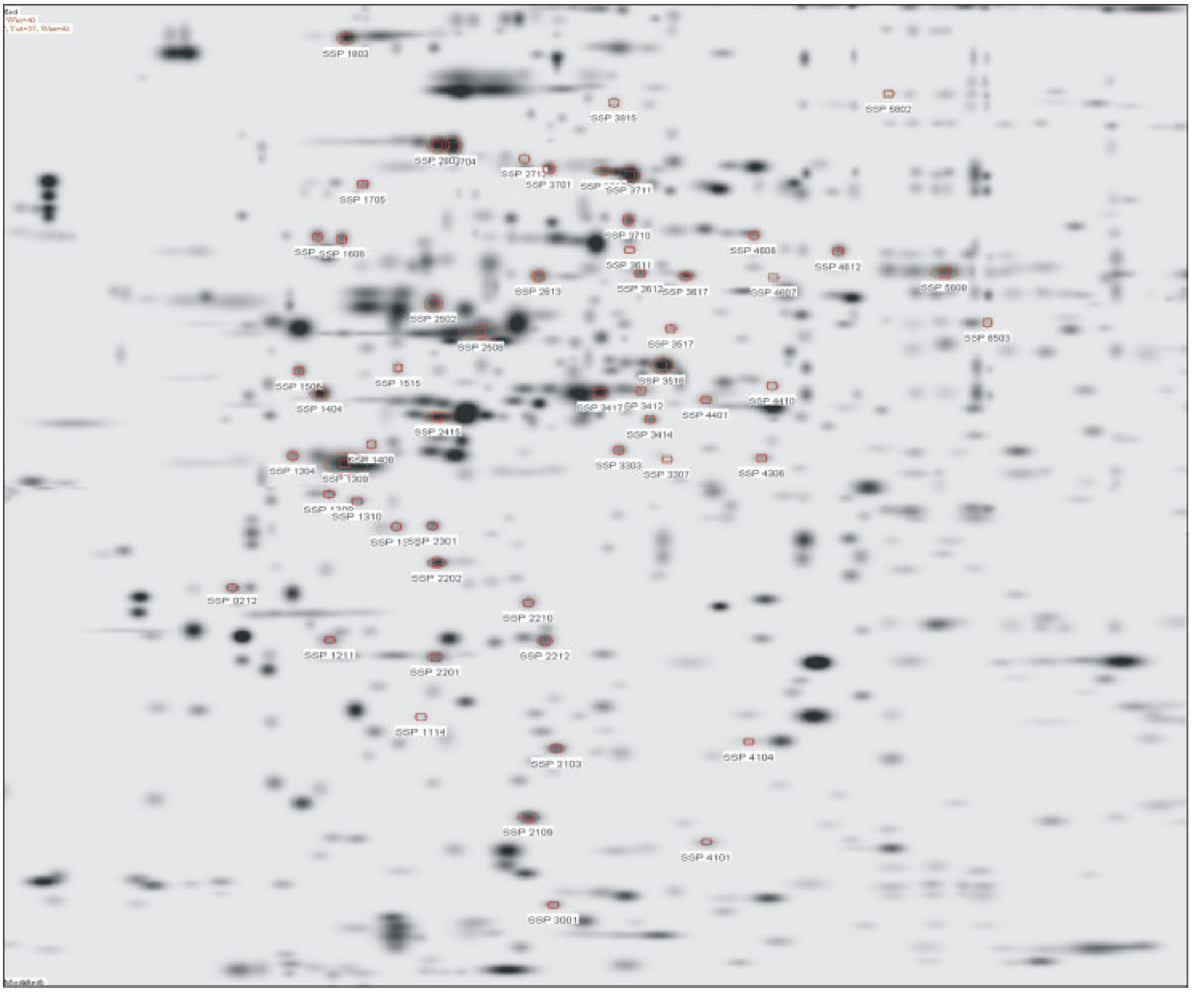

Fig. 1. 2D-PAGE protein master pattern of SF767 human glioma cells. The proteins are annotated by SSP (spot number). Acidic and basic proteins are oriented to the left and the right, respectively. Low M_r and high M_r proteins are oriented to the top and bottom, respectively. The gel pattern represents a vertical acrylamide gradient of approximately 9–19% and a horizontal pH gradient of approximately 4–7.5. Molecular weight (M_r; kDa) and isoelectric point (p*I*) calibrations are estimates from this master pattern based on the calculated M_r and p*I* of protein spots from samples separated on replicate gels. Using mass fingerprinting we identified 57 proteins here, a subset of which is significantly altered. ($P < 0.05$, $n = 6$ gels, Student's *t*-test; three experiments). [The color figure for this article is available online at www.interscience.wiley.com.]

DISCUSSION

We hypothesize that protein biomarkers exist which may be associated with phenotypic changes in SF767 human glioma cells induced by low-level magnetic fields. After automatically matching multiple gel patterns, individual proteins were analyzed for alterations in abundance and charge. The PDQUEST computerized system showed significant differences obscured by the minor changes in spot intensity that arise between separate cell cultures. Analysis is complicated by intraclonal variability in the synthesis of different proteins. The intensity of a fraction of the spots may be measurably different due to the accumulation of minor, but uncontrollable variations between different cultures. Computer analysis of the gel patterns allows one to search for changes that are consistent and statistically significant. The gels from three experiments were pooled and searched for spots that are different to the 95% significance level making them unlikely to be the result of intraclonal variation.

The ability to simultaneously determine changes made possible the selection of specific proteins that deserve further study and identification. A subset of soluble cellular proteins in SF767 cell lysates (57) were detected which were identifiable and/or differentially

TABLE 1. Biochemical Characteristics and Differential Expression of SF767 Human Glioma Proteins Following Magnetic Field Exposure

			Mean abundance[b]			
SSP no.[a]	M_r (kDa)	p*I*	Sham	1.2 μT	Protein[c]	Function
1114	22.3	5	40	157	Calcium binding protein (P22)	Constitutive cell membrane traffic
1515	50.6	5	121	287	Keratin type 1 cytoskeletal 18	Cytoskeleton; intermediate filament family
4410	42.7	5.7	35	209	Creatine kinase B chain	Catalyzes energy transduction
1211	26.4	5	1218	656	20S proteasome component C	Catalytic proteinase complex
1404	32.9	4.8	2042	1642	40S ribosomal protein SA (P40)	Believed to be a laminin receptor
1803	92.4	4.8	2312	1501	Endoplasmin precursor	Heat shock protein 90 member; molecular chaperone
2502	49.6	4.9	2743	1491	Keratin type 1 cytoskeletal 13	Cytoskeleton; intermediate filament family
3417	41.8	5.5	2380	1229	Actin-5C	Cell motility; muscle contraction; cytoskeleton structure
3612	51.8	5.4	869	667	P59 protein	Steroid hormone receptor trafficking
5608	56.8	6.1	2917	1755	ER60 protein	SS bond rearrangement

[a]Protein spot number in reference 2D pattern.
[b]$P < 0.05$, $n = 6$ gels, Student's *t*-test; coefficient of variation (SD/mean) = 15%; three experiments.
[c]Protein identification via peptide mass fingerprinting/database.

expressed between groups treated in the presence or absence of EGF under varying magnetic fields. These differences in mean spot density suggest altered regulation of the genome, change in protein turnover rate, or a change in protein stability. However, since no p*I* shift was observed, post-translational modifications following treatment were not likely.

Low-level magnetic field exposure (1.2 μT) significantly altered the abundance of 10 identified proteins in SF767 human glioma cells (Table 1). The range of functional roles of the affected proteins was diverse. It included cell membrane trafficking proteins (calcium binding protein P22 and P59 protein), cytoskeletal structural proteins (Keratin type 1 cytoskeletal protein 13 and Keratin type 1 cytoskeletal protein 18), protein catalysts (creatine kinase B chain and 20S proteasome component C), and cell motility regulators (Actin-5C).

Three of these 10 altered proteins showed a significant increase in spot density following MF

TABLE 2. Biochemical Characteristics and Differential Expression of EGF-Treated SF767 Human Glioma Proteins Following Magnetic Field Exposure

			Mean abundance[b]			
SSP no.[a]	M_r (kDa)	p*I*	Sham (+EGF)	1.2 μT (+EGF)[d]	Protein[c]	Function
2415	44.1	5.1	1472	2260	Keratin type 1 cytoskeletal 19	Cytoskeleton; intermediate filament family
2502	49.6	4.9	1619	2929	Keratin type 1 cytoskeletal 13	Cytoskeleton; intermediate filament family
4607	58.5	5.9	171	265	Glucose 6-P 1-dehydrogenase	Nucleic acid synthesis
4612	59.6	5.5	777	883	T-complex 1 theta subunit	Molecular chaperone
1505	49.1	4.7	696	570	Keratin type 1 cytoskeletal 15	Cytoskeleton; intermediate filament family
1515	50.5	5	144	114	Keratin type 1 cytoskeletal 18	Cytoskeleton; intermediate filament family

[a]Protein spot number in reference 2D pattern.
[b]$P < 0.05$, $n = 6$ gels, Student's *t*-test; coefficient of variation (SD/mean) = 15%; three experiments.
[c]Protein identification via peptide mass fingerprinting/database.
[d]EGF (10 ng/ml) for 3 h in vitro.

exposure. We observed a sixfold increase in Creatine kinase B chain, which is known to catalyze intracellular energy transduction. Similarly Nylund and Leszczynski [2004] used 2D-PAGE and observed alterations in human endothelial proteins associated with cellular energy production after mobile phone radiation exposure. In the same study they observed a significant increase in two cytoskeletal protein isoforms in human endothelial cells after radiation exposure. Nylund and Leszczynski postulated that alterations in the expression of these proteins indicate a cytoskeletal-related response might take place in these cells exposed to radiation. We also observed Keratin type 1 cytoskeletal 18, a cytoskeletal intermediate filament protein, increased over twofold after exposure to a low-level magnetic field.

Seven proteins showed a significant decrease in spot density following MF exposure. Interestingly, we noted a twofold decrease in P59 protein, a protein believed to be involved in steroid hormone receptor trafficking. As previously noted, SF767 human glioma cells appear to be tamoxifen-sensitive, since these cells have been shown to exhibit a cell growth response, i.e., reversal of inhibitory action of tamoxifen in a 1.2 μT magnetic field [Liburdy et al., 1999]. Whether P59 protein plays an associative role in the inhibitory action of the anti-estrogen in MF-exposed human glioma cells remains to be further investigated.

Several identified proteins were unchanged by low-level magnetic field exposure (Figure 1). Two heat shock proteins, 60 KDA Heat shock protein and Hsp70, as well as an activator of heat shock proteins, Protein C14ORF3, were not altered by MF treatment. Similarly Kang et al. [1998] reported that Hsp70 synthesis was not increased by exposure to 50 Hz electromagnetic field in transfected 34i cells. However, we also observed that Endoplasmin precursor, another member of the heat shock protein family, showed a decrease in abundance following MF exposure. Further, DiCarlo et al. [2002] observed that electromagnetic field exposure has led to observed decreases in Hsp70 levels in chick embryos. Therefore, the effect on heat shock protein expression is not presently clear.

With EGF pre-treatment additional proteins were altered by low-level magnetic field. These included glucose 6-P 1-dehydrogenase, which plays a role in nucleic acid synthesis, and T-complex 1 theta subunit, a molecular chaperone (Table 2). Four keratin type cytoskeletal proteins (types 13, 15, 18, and 19) were altered which further suggests an effect of magnetic field radiation in cytoskeletal-dependent processes (e.g., cell size, cell shape, and intercellular interactions). With regard to Keratin Type 1 cytoskeletal 13 and 18, pretreatment with EGF, which has an important role in cell–cell signaling during nervous system formation, appeared to reverse the observed changes in abundance following MF exposure alone. Further study of this apparent EGF induced reversal effect is indicated.

It had been previously shown that a weak magnetic field reversed the cytostatic action of a growth regulator in SF767 glioma cells in vitro. Protein changes have been observed with in vitro magnetic field exposure in the same cell line [Savage et al., 2005]. In this study new insights on the possible underlying biochemical mechanism for the observed effect are made. We noted here a significant increase in an intracellular energy transduction catalyst. Similarly Nylund and Leszczynski [2004] observed alterations in proteins associated with cellular energy production after mobile phone radiation exposure. They also noted a significant increase in two cytoskeletal protein isoforms after the same exposure and postulated that a cytoskeletal-related response might take place in endothelial cells exposed to radiation. Likewise we observed here a cytoskeletal intermediate filament protein increased following a low-level magnetic field. This work represents an early step towards understanding the complex series of intracellular changes which occur in response to environmental level magnetic fields.

REFERENCES

Anderson NL. 1988. Two-dimensional electrophoresis: Operation of the ISO-DALT system. Rockville, MD: Large Scale Biology Press.

DiCarlo A, White N, Guo F, Garrett P, Litovitz T. 2002. Chronic electromagnetic field exposure decreases Hsp70 levels and lowers cytoprotection. J Cell Biochem 84:447–454.

Feychting M, Forssen U, Rutgyist LE, Ahlbom A. 1998. Magnetic fields and breast cancer in Swedish adults residing near high-voltage power lines. Epidemiology 9:392–397.

Harland JD, Liburdy RP. 1997. Environmental magnetic fields inhibit the antiproliferative action of tamoxifen and melatonin in a human breast cancer cell line. Bioelectromagnetics 18:555–562.

Kang KI, Bouhouche I, Fortin D, Baulieu EE, Catelli MG. 1998. Luciferase activity and synthesis of Hsp70 and Hsp90 are insensitive to 50 Hz electromagnetic fields. Life Sci 63:489–497.

Kanitz MH, Afzal SMJ, Harland J, Liburdy RP, Savage RE. 1998. Two dimensional electrophoretic profile associated with EMF inhibition of cytostatic effect of tamoxifen on MCF-7 cell growth. Proc Am Assoc Cancer Res 39:2279.

Kanitz MH, Swaminathan S, Savage RE. 2004. Two dimensional electrophoretic protein profile analysis following exposure of human uroepithelial cells to occupational bladder carcinogens. Cancer Lett 205:121–131.

Liburdy RP, Sloma TR, Sokolic R, Yaswen P. 1993. ELF magnetic fields, breast cancer, and melatonin: 60 Hz fields: melatonin's

oncostatic action on ER+ breast cancer cell proliferation. J Pineal Res 14:89–97.

Liburdy RP, Levine GA, Harland JD. 1999. A 12 mGauss (1.2 μTesla) Magnetic Field Inhibits Tamoxifen's Oncostatic Action in a Second Human Breast Cancer Cell Line: T47D. In: Bersani F, editor. Electricity and magnetism in biology and medicine. New York: Plenum Press; pp. 201–212.

Nicolson GL. 1991. Quantitative variations in gene expression: Possible role in cellular diversification and tumor progression. J Cell Biochem 46:277–283.

Nylund R, Leszczynski D. 2004. Proteomic analysis of human endothelial cell line EA.hy926 after exposure to GSM 900 radiation. Proteomics 4:1359–1365.

Pollan M, Gustavsson P. 1999. High-risk occupations for breast cancer in the Swedish female working population. Am J Public Health 89:875–881.

Savage RE, Kanitz MH, Lotz WG, Conover D, Hennessey EM, Hanneman WH, Witzmann FA. 2005. Changes in gene and protein expression in magnetic field-treated human glioma cells. Tox Mech Meth 15:115–120.
