# Supplementary material for: Magnetic Fields and Cancer: Epidemiology, Cellular Biology, and Theranostics
Source: Int J Mol Sci. 2022 Jan 25;23(3):1339. doi: 10.3390/ijms23031339 (PMC8835851; doi:10.3390/ijms23031339)
Supplement: Supplementary file 1 [file ijms-23-01339-s001.zip › Supplementary Data Set S1/MF and Cancer.Data/PDF/0662023288/2016BelyaevEUROPAEMEMFGuideline2016forthepreve.pdf]

ResearchGate

See discussions, stats, and author profiles for this publication at: https://www.researchgate.net/publication/305689940

EUROPAEM EMF Guideline 2016 for the prevention, diagnosis and treatment of EMF-related health problems and illnesses

Article *in* Reviews on Environmental Health · July 2016

DOI: 10.1515/reveh-2016-0011

CITATIONS
112

READS
9,233

15 authors, including:

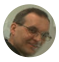

Igor Belyaev
Biomedical Research Center Slovak Academy of Sciences
134 PUBLICATIONS **3,317** CITATIONS

SEE PROFILE

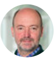

Hanns Moshammer
Medical University of Vienna
269 PUBLICATIONS **3,641** CITATIONS

SEE PROFILE

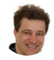

Piero Lercher
Medical University of Vienna
136 PUBLICATIONS **219** CITATIONS

SEE PROFILE

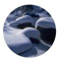

Gerd Oberfeld
Amt der Salzburger Landesregierung
29 PUBLICATIONS **976** CITATIONS

SEE PROFILE

Some of the authors of this publication are also working on these related projects:

Project Manual of Head anf Neck Reconstruction Using Regional and Free Flaps View project

Project Biodiversity and Health View project

All content following this page was uploaded by Igor Belyaev on 27 October 2017.

The user has requested enhancement of the downloaded file.

Igor Belyaev, Amy Dean, Horst Eger, Gerhard Hubmann, Reinhold Jandrisovits, Markus Kern, Michael Kundi, Hanns Moshammer, Piero Lercher, Kurt Müller, Gerd Oberfeld*, Peter Ohnsorge, Peter Pelzmann, Claus Scheingraber and Roby Thill

EUROPAEM EMF Guideline 2016 for the prevention, diagnosis and treatment of EMF-related health problems and illnesses

DOI 10.1515/reveh-2016-0011
Received March 16, 2016; accepted May 29, 2016

Abstract: Chronic diseases and illnesses associated with non-specific symptoms are on the rise. In addition to chronic stress in social and work environments, physical and chemical exposures at home, at work, and during leisure activities are causal or contributing environmental stressors that deserve attention by the general practitioner as well as by all other members of the health care community. It seems necessary now to take "new exposures" like electromagnetic fields (EMF) into account. Physicians are increasingly confronted with health problems from unidentified causes. Studies, empirical observations, and patient reports clearly indicate interactions between EMF exposure and health problems. Individual susceptibility and environmental factors are frequently neglected. New wireless technologies and applications have been introduced without any certainty about their health effects, raising new challenges for medicine and society. For instance, the issue of so-called non-thermal effects and potential long-term effects of low-dose exposure were scarcely investigated prior to the introduction of these technologies. Common electromagnetic field or EMF sources: Radio-frequency radiation (RF) (3 MHz to 300 GHz) is emitted from radio and TV broadcast antennas, Wi-Fi access points, routers, and clients (e.g. smartphones, tablets), cordless and mobile phones including their base stations, and Bluetooth devices. Extremely low frequency electric (ELF EF) and magnetic fields (ELF MF) (3 Hz to 3 kHz) are emitted from electrical wiring, lamps, and appliances. Very low frequency electric (VLF EF) and magnetic fields (VLF MF) (3 kHz to 3 MHz) are emitted, due to harmonic voltage and current distortions, from electrical wiring, lamps (e.g. compact fluorescent lamps), and electronic devices. On the one hand, there is strong evidence that long-term exposure to certain EMFs is a risk factor for diseases such as certain cancers, Alzheimer's disease, and male infertility. On the other hand, the emerging electromagnetic hypersensitivity (EHS) is more and more recognized by health authorities, disability administrators and case workers, politicians, as well

European Academy for Environmental Medicine (EUROPAEM) – EMF working group:
***Corresponding author: Gerd Oberfeld,** Department of Public Health, Government of Land Salzburg, Austria,
E-mail: gerd.oberfeld@salzburg.gv.at
Igor Belyaev: Cancer Research Institute BMC, Slovak Academy of Science, Bratislava, Slovak Republic; and Prokhorov General Physics Institute, Russian Academy of Science, Moscow, Russia
Amy Dean: American Academy of Environmental Medicine, Wichita, KS, USA
Horst Eger: Association of Statutory Health Insurance Physicians of Bavaria, Medical Quality Circle "Electromagnetic Fields in Medicine – Diagnostic, Therapy, Environment", no. 65143, Naila, Germany
Gerhard Hubmann: Center for Holistic Medicine "MEDICUS", Vienna, Austria; and Wiener Internationale Akademie für Ganzheitsmedizin (GAMED), Vienna, Austria
Reinhold Jandrisovits: Medical Association Burgenland, Environmental Medicine Department, Eisenstadt, Austria
Markus Kern: Medical Quality Circle "Electromagnetic Fields in Medicine – Diagnosis, Treatment and Environment", Kempten, Germany; and Kompetenzinitiative zum Schutz von Mensch, Umwelt u. Demokratie e.V., Kempten, Germany
Michael Kundi and Hanns Moshammer: Institute of Environmental Health, Medical University Vienna, Vienna, Austria
Piero Lercher: Medical Association Vienna, Environmental Medicine Department, Vienna, Austria
Kurt Müller: European Academy for Environmental Medicine, Kempten, Germany
Peter Ohnsorge: European Academy for Environmental Medicine, Wurzburg, Germany
Peter Pelzmann: Department of electronics and computer science engineering, HTL Danube City, Vienna, Austria
Claus Scheingraber: Working Group Electro-Biology (AEB), Munich, Germany and Association for Environmental- and Human-Toxicology (DGUHT), Wurzburg, Germany
Roby Thill: Association for Environmental Medicine (ALMEN), Beaufort, Luxembourg

- 10.1515/reveh-2016-0011
Downloaded from PubFactory at 07/26/2016 08:26:15AM
via Slovak Academy of Science

as courts of law. We recommend treating EHS clinically as part of the group of chronic multisystem illnesses (CMI), but still recognizing that the underlying cause remains the environment. In the beginning, EHS symptoms occur only occasionally, but over time they may increase in frequency and severity. Common EHS symptoms include headaches, concentration difficulties, sleep problems, depression, a lack of energy, fatigue, and flu-like symptoms. A comprehensive medical history, which should include all symptoms and their occurrences in spatial and temporal terms and in the context of EMF exposures, is the key to making the diagnosis. The EMF exposure is usually assessed by EMF measurements at home and at work. Certain types of EMF exposure can be assessed by asking about common EMF sources. It is very important to take the individual susceptibility into account. The primary method of treatment should mainly focus on the prevention or reduction of EMF exposure, that is, reducing or eliminating all sources of high EMF exposure at home and at the workplace. The reduction of EMF exposure should also be extended to public spaces such as schools, hospitals, public transport, and libraries to enable persons with EHS an unhindered use (accessibility measure). If a detrimental EMF exposure is reduced sufficiently, the body has a chance to recover and EHS symptoms will be reduced or even disappear. Many examples have shown that such measures can prove effective. To increase the effectiveness of the treatment, the broad range of other environmental factors that contribute to the total body burden should also be addressed. Anything that supports homeostasis will increase a person's resilience against disease and thus against the adverse effects of EMF exposure. There is increasing evidence that EMF exposure has a major impact on the oxidative and nitrosative regulation capacity in affected individuals. This concept also may explain why the level of susceptibility to EMF can change and why the range of symptoms reported in the context of EMF exposures is so large. Based on our current understanding, a treatment approach that minimizes the adverse effects of peroxynitrite – as has been increasingly used in the treatment of multisystem illnesses – works best. This EMF Guideline gives an overview of the current knowledge regarding EMF-related health risks and provides recommendations for the diagnosis, treatment and accessibility measures of EHS to improve and restore individual health outcomes as well as for the development of strategies for prevention.

Keywords: accessibility measures; Alzheimer's disease; cancer; chronic multisystem illnesses (CMI); diagnosis; electric; electromagnetic field (EMF); electromagnetic hypersensitivity (EHS); infertility; leukemia; magnetic; medical guideline; nitrosative stress; non-ionizing; oxidative stress; peroxynitrite; prevention; radiation; static; therapy; treatment.

Current state of the scientific and political debate about EMF-related health problems from a medical perspective

Introduction

The Environmental Burden of Disease Project assessed the influence of nine environmental stressors (benzene, dioxins including furans and dioxin-like PCBs, second-hand smoke, formaldehyde, lead, noise, ozone, particulate matter and radon) on the health of the population of six countries (Belgium, Finland, France, Germany, Italy, and the Netherlands). Those nine environmental stressors caused 3%–7% of the annual burden of disease in the six European countries (1).

The Bundespsychotherapeutenkammer (BPtK) study in Germany showed that mental disorders had increased further and especially burnout as a reason of inability to work increased seven-fold from 2004 to 2011 (2). In Germany, 42% of early retirements in 2012 were caused by mental disorders, depression being the leading diagnosis (3). In Germany, psychotropic drugs are in third place for the prescriptions of all drugs (4).

The consumption of methylphenidate (Ritalin, Medikinet, Concerta), a psychotropic drug prescribed as a treatment for attention deficit hyperactivity disorder (ADHD) especially for young children and adolescents, has increased alarmingly since the early 1990s. According to statistics of the German Federal Institute for Drugs and Medical Devices (Bundesinstitut für Arzneimittel und Medizinprodukte), prescriptions have increased even more dramatically since 2000 and reached a climax in 2012. In 2013, only a slight decline in the number of prescriptions was observed (5). Interestingly, the rapid increase in the use of methylphenidate coincides with the enormous expansion of mobile telecommunication and other related technologies, posing an open research question.

In Germany, work disability cases and absence days due to mental health disorders more than doubled from 1994 to 2011 (6). In the Organization for Economic Co-operation and Development (OECD) countries, a huge

- 10.1515/reveh-2016-0011
Downloaded from PubFactory at 07/26/2016 08:26:15AM
via Slovak Academy of Science

variability in the prescription of antidepressants has occurred and generally an increasing trend has been observed. Socioeconomic status and therapeutic standards cannot fully explain these observations (7). Functional disturbances like chronic inflammation and changes of neurotransmitter functions caused by environmental influences have hardly been investigated.

A steady increase in the prevalence of allergic/asthmatic diseases globally has occurred, with about 30%–40% of the world population now being affected by one or more allergic/asthmatic conditions (8).

It is suspected that environmental conditions such as the increasing exposure of the population to electromagnetic fields (EMFs) play a causal role for EMF-related health effects (9–12), including exposure to radio-frequency radiation (RF), which emanates from, e.g. cordless phones (DECT), mobile phone base stations, and mobile phones (GSM, GPRS, UMTS, LTE), especially smartphones, data cards for laptop and notebook computers, wireless LAN (Wi-Fi), wireless and powerline communication-based smart meters, but also exposure to extremely low frequency (ELF) electric fields (EF) and magnetic fields (MF) including "dirty electricity", which emanate from disturbances on electric wiring, power lines, electric devices, and other equipment. For the society and the medical community, all of this raises new challenges.

While biophysical and biochemical mechanisms of biological effects of EMF at low-intensity levels are not exactly known, significant progress has been achieved in the last decades, and there are numerous data indicating that these mechanisms may overlap for ELF and RF effects (13–18). In the following sections, we provide some background information on important aspects of EMF biological effects. However, this must not be misunderstood as a full review of the evidence. We do not always strictly differentiate between RF and ELF fields because of the above mentioned overlap in biological mechanisms. It should also be mentioned here that very specific exposure conditions may trigger biological responses in one individual, but not in others. Anecdotal reports, however, indicate that such individual responsiveness or susceptibility does expand over time and the intolerance then extends over a broad range of exposure conditions.

Chronic diseases and illnesses associated with unspecific symptoms are on the rise. In addition to chronic stress in social and work environments, physical and chemical exposures at home, at work, and during leisure activities are causal or contributing environmental stressors that deserve attention by the general practitioner as well as by all other members of the health care community. It seems certainly necessary now to take "new exposures" like EMF into account, or as stated by Hedendahl et al. (19): *"It is time to consider ELF EMF and RF EMF as environmental pollutants that need to be controlled"*.

Worldwide statements of organizations regarding EMF

The recommendations of the World Health Organization (WHO) regarding ELF electric and magnetic fields and RF radiation, compiled by the International Commission on Non-Ionizing Radiation Protection (ICNIRP) (20, 21), are based on currents induced in the body (ELF) and thermal effects (RF).

Thermal effects are defined as effects that originate in elevated temperatures from the absorption of electromagnetic energy. The specific absorption rate (SAR) is defined as the rate of absorption of electromagnetic energy in a unit mass of biological tissue. It is proportional to the incremental temperature increase in that tissue. Indeed while a significant temperature increase must be avoided as it can be of immediate adverse health consequences (tissue necrosis, cardiac stress, etc.) exposures can be without (measureable) temperature increase either because of heat dissipation or because the exposure is too low to be associated with relevant heating. The latter type of exposure is termed non-thermal. Biological and health-relevant effects at non-thermal levels have been shown and discussed by many research groups all over the world (9, 10, 22–24).

The ICNIRP recommendations were adopted by the EU in its Council Recommendation of 1999, without considering long-term non-thermal effects. However, it should be stressed that at an international EMF conference in London (2008), Professor Paolo Vecchia, ICNIRP Chairman from 2004 to 2012, said about the exposure guidelines "What they are not": *"They are not mandatory prescriptions for safety"*, *"They are not the' 'last word' on the issue"*, and *"They are not defensive walls for industry or others"* (25).

For all RF-based non-thermal EMF effects, SAR estimates are not an appropriate exposure metric, but instead either the field intensity or power density (PD) in combination with exposure duration should be used in safety standards (26, 14, 27). In contrast to the ICNIRP guidelines, the Russian safety standards, are based on non-thermal RF effects, which were obtained by several research institutes in the former Soviet Union during decades of studies on chronic exposures to RF (28, 29).

In contrast to the WHO headquarter in Geneva, the International Agency for Research on Cancer (IARC), a WHO-affiliated specialized agency in Lyon, classified

- 10.1515/reveh-2016-0011
Downloaded from PubFactory at 07/26/2016 08:26:15AM
via Slovak Academy of Science

extremely low frequency magnetic fields (ELF MF) as possibly carcinogenic to humans (Group 2B) in 2002 (30) and radio-frequency radiation in 2011 (24).

It should be noted that, during the last 20 years, more than 20 position papers and resolutions regarding EMF and health have been adopted by EMF researchers and physicians. These include the Vienna EMF Resolution, Austria, 1998; Stewart Report, UK, 2000; Salzburg Resolution, Austria, 2000; Freiburg Appeal, Germany, 2002; Catania Resolution, Italy, 2002; Irish Doctors' Environmental Association Statement, Ireland, 2005; Helsinki Appeal, Finland, 2005; Benevento Resolution, Italy, 2006; Venice Resolution, Italy, 2008; Porto Alegre Resolution, Brazil, 2009; Russian National Committee on Non-Ionizing Radiation Protection Resolution, Russia, 2001; International Doctors' Appeal, Europe, 2012; and the Report of the Standing Committee on Health, Canada, 2015 (31–34).

In August 2007 and December 2012, the BioInitiative Working Group, an international group of 29 experts with different competences, published two groundbreaking reports "BioInitiative 2007/resp. 2012 – A Rationale for a Biologically-based Public Exposure Standard for Electromagnetic Fields (ELF and RF)" edited by Cindy Sage and David O. Carpenter, calling for preventive measures against EMF exposure based on the available scientific evidence (9, 10). The BioInitiative reports are global milestones with respect to a comprehensive review of biological effects and health effects of low-intensity electromagnetic radiation as well as the conclusions and recommendations given for the public. The BioInitiative report 2012 includes sections on the evidence for effects on: gene and protein expression, DNA, immune function, neurology and behavior, blood-brain barrier, brain tumors and acoustic neuromas, childhood leukemia, melatonin, Alzheimer's disease, breast cancer, fertility and reproduction, fetal and neonatal disorders, autism, disruption by the modulating signal, EMF medical therapeutics, as well as sections on: statement of the problem, the existing public exposure standards, evidence for inadequacy of the standards, the precautionary principle, global public health examples, key scientific evidence and public health recommendations, and summary for the public and conclusions.

As it is mostly neglected as a health hazard, the European Environment Agency compared the risks of non-ionizing radiation (EMF) to other environmental hazards such as asbestos, benzene, and tobacco, urgently recommending to implement a precautionary approach regarding EMF (35). This position was confirmed and elaborated more comprehensibly in further publications in 2011 and 2013 (36, 37).

In September 2008, a statement of the European Parliament called for a review of the EMF limits set out in the EU Council Recommendation of 1999, which was based on the ICNIRP guidelines, with reference to the BioInitiative Report (38). This was further strengthened in the European Parliament resolution of April 2009 (39).

At the meeting in November 2009 in Seletun, Norway, a scientific panel adopted a Consensus Agreement that recommends preventative and precautionary actions that are warranted now, given the existing evidence for potential global health risks from EMF exposure (40). Besides general and specific recommendations, e.g. for mobile and cordless phone use, the panel recommended exposure limits for ELF magnetic fields and radio-frequency radiation. It was stated by the panel: "Numeric limits recommended here do not yet take into account sensitive populations (EHS, immune-compromised, the fetus, developing children, the elderly, people on medications, etc.). Another safety margin is, thus, likely justified further below the numeric limits for EMF exposure recommended here".

Since 2007 the Highest Health Council of the Ministry of Health in Austria has recommended to take preventive action by reducing exposure levels from RF devices which may lead to long-term human exposure of at least a factor of 100 below the guideline levels of the European Commission and by issuing rules on how to reduce one's individual exposure to RF radiation from mobile phones (41).

In May 2011, the Parliamentary Assembly of the Council of Europe adopted the report "The Potential Dangers of Electromagnetic Fields and their Effects on the Environment" (42). The Assembly recommended many preventive measures for the member states of the Council of Europe with the aim to protect humans and the environment, especially from high-frequency electromagnetic fields such as: *"Take all reasonable measures to reduce exposure to electromagnetic fields, especially to radiofrequencies from mobile phones, and particularly the exposure of children and young people who seem to be most at risk from head tumors"*, or *"Pay particular attention to 'electrosensitive' people who suffer from a syndrome of intolerance to electromagnetic fields and introduce special measures to protect them, including the creation of wave-free areas not covered by the wireless network"*.

Recognizing that patients are being adversely affected by EMF exposure, the American Academy of Environmental Medicine (AAEM) published recommendations regarding EMF exposure in July 2012. The AAEM called for physicians to consider electromagnetic exposure in diagnosis and treatment and to recognize that EMF exposure *"may be an underlying cause of the patient's disease process"* (43).

Since 2014, the Belgian government has prohibited the advertising of mobile phones for children under the age of

- 10.1515/reveh-2016-0011
Downloaded from PubFactory at 07/26/2016 08:26:15AM
via Slovak Academy of Science

7 and has required the specific absorption rate (SAR) of mobile phones be listed. Furthermore, at the point of sale, well-marked warnings must be posted that instruct users to use headsets and to minimize their exposure (44).

In January 2015, the French parliament adopted a comprehensive law that protects the general public from excessive exposure to electromagnetic waves. Among other things, it was passed to ban Wi-Fi in nurseries for children under the age of 3 and to enable Wi-Fi at primary schools with children under the age of 11 only when used specifically for lessons. Public places offering Wi-Fi must clearly advertise this fact on a sign. At the point of sale of mobile phones, the SAR value must be clearly shown. In the future, any mobile phone advertisement must include recommendations on how users can reduce RF radiation exposure to the head such as the use of headsets. Data on local EMF exposure levels shall be made more easily accessible to the general public, among others, through countrywide transmitter maps. Also, the French government will have to submit a report on electromagnetic hypersensitivity to the parliament within a year (45).

As of February 2016, 220 scientists from 42 countries have signed an international Appeal, directed to the United Nations (UN) and WHO, calling for protection from non-ionizing electromagnetic field exposure. The appeal addresses the scientifically proven effects on health and the inadequate international guidelines (ICNIRP) to date and their use by the WHO. In addition, nine requests were made, including that: "*the public be fully informed about the potential health risks from electromagnetic energy and taught harm reduction strategies*" and that "*medical professionals be educated about the biological effects of electromagnetic energy and be provided training on treatment of patients with electromagnetic sensitivity*" (46).

In September 2015 an International Scientific Declaration on Electromagnetic Hypersensitivity and Multiple Chemical Sensitivity was published by the Scientific Committee following the 5th Paris Appeal Congress, which took place on 18 May 2015 at the Royal Academy of Medicine, Brussels, Belgium. It calls upon national and international agencies and organizations to recognize EHS and multiple chemical sensitivity as a disease and urges particularly the WHO to include EHS and MCS in the International Classification of Diseases. It also asks national and international agencies and organizations to adopt simple precautionary measures of prevention, to inform the public, and to appoint truly independent expert groups to evaluate these health risks based on scientific objectivity, which is not the case today (47).

EMF and cancer

Except for a few investigations in occupational settings, epidemiological research of EMF started in 1979 when Wertheimer and Leeper published their study about the relationship between the proximity to so-called power line poles (ELF MF) with "service drop" wires and the occurrence of childhood cancer (specifically leukemia and brain tumors) (48). At the same time Robinette et al. studied mortality in a cohort of Korean War veterans having been trained on military radars (RF) in the early 1950s (49). Both studies found indications of increased risks and initiated a new era of studying health-relevant effects from exposure to EMFs.

ELF MF

In the following years, a large number of investigations about the relationship between childhood leukemia and extremely low frequency magnetic fields (ELF MF) have been published. However, the results seemed inconsistent until in 2000 two pooled analyses (50, 51) were conducted, providing little indication of inconsistency and demonstrating an increase of leukemia risk with increasing average exposure levels that was significant for levels above 0.3 or 0.4 μT relative to averages below 0.1 μT but without indication of a threshold. Based on these findings, the International Agency for Research on Cancer (IARC) classified ELF MF in 2002 as a Group 2B (possible) carcinogen (30). To this category belong, e.g. lead, DDT, welding fumes, and carbon tetrachloride.

Since then additional epidemiological studies have been conducted that gave essentially the same results (52, 53). The only study to date on the gene-environment interaction in relation to power-frequency MF reported a significant effect enhancement in children with a polymorphism in a DNA-repair gene (54). In a review on childhood leukemia and ELF MF, Kundi concluded that there is sufficient evidence from epidemiological studies of an increased risk for childhood leukemia from exposure to power-frequency MF that cannot be attributed to chance, bias, or confounding. Therefore, according to the rules of IARC, such exposures ought to be classified as a Group 1 (definitive) carcinogen (55).

The BioInitiative Report 2012 (56) stated: "*Children who have leukemia and are in recovery have poorer survival rates if their ELF exposure at home* (*or where they are recovering*) *is between 1mG* [*0.1 μT*] *and 2 mG* [*0.2 μT*] *in one study; over 3 mG* [*0.3 μT*] *in another study*" (56).

- 10.1515/reveh-2016-0011
Downloaded from PubFactory at 07/26/2016 08:26:15AM
via Slovak Academy of Science

RF

There were several mechanisms identified which might be responsible for carcinogenic effects of RF (23). Epidemiological studies of RF before the general rise in exposure to mobile telecommunication devices was very restricted and only a few studies had been conducted in the vicinity of radio transmitters, radar stations, for occupational exposures, and in radio amateurs. After the introduction of digital mobile telephony, the number of users of mobile phones increased dramatically and it was recommended in the 1990s to perform epidemiological studies with a focus on intracranial tumors. Since the first publication in 1999 by the Swedish group of Prof. Lennart Hardell (57), about 40 studies have been published. The majority of these studies investigated brain tumors, but salivary gland tumors, uveal melanoma, malignant melanoma of the skin, nerve sheath tumors, testicular cancer, and lymphoma were also studied. Many of these studies are inconclusive because exposure durations are too short; however, two series of investigations, the international Interphone Study conducted in 13 countries and the Swedish studies of the Hardell group, had a significant proportion of long-term mobile phone users and could in principle be used for risk assessment. In 2011, IARC classified radio-frequency electromagnetic fields (RF) as a Group 2B carcinogen based on evidence from epidemiological studies and animal experiments (24). Since then, additional studies have corroborated the assumption of a causal relationship between mobile phone use and cancer (58–60). Hardell and Carlberg (61) concluded that RF EMF ought to be classified as a definitive human carcinogen (IARC Group 1). The evidence for a causal relationship between long-term mobile and cordless phone use and the risk of glioma has increased further: in 2014, a study by Carlberg and Hardell (62) showed significantly decreased survival rates in patients with glioblastoma multiforme (astrocytoma grade IV) and the use of wireless phones and, in 2015, another pooled case-control study by Hardell and Carlberg (63) including latency periods of >25 years.

That also other tumors might be related to EMF exposure is exemplified by the observation in women who have worn their mobile phone in their bra for prolonged periods of time and later developed breast cancer at that site (64).

The Italian Supreme Court confirmed a previous decision by the Civil Court of Appeals of Brescia (no. 614 of 10 December 2009) that ruled that the National Institute for Workmen's Compensation (INAIL) must compensate a worker who had developed a tumor in the head due to long-term, heavy use of mobile phones while on the job. The case was an ipsilateral neuroma of the trigeminal nerve in a subject who had occupational exposure for >10 years, with >15,000 h on mobile and cordless phones. The court recognized that *"it is likely (qualified probability) that RF have a role which is at least contributory in the development of the origin of the tumor suffered by the subject"* (65).

Many modern devices emit EMF of different frequency ranges simultaneously. For example, mobile phones create EMF in RF, VLF, and ELF frequency ranges and also a static magnetic field; for a review see (23). Therefore, it is important to consider combined exposures for the assessment of health effects.

Genotoxic effects

Genotoxic effects of EMF dealing with DNA damage, mutations, chromatin structure, and DNA repair have recently been reviewed by Henry Lai in the Bioinititive Report (66) and by the IARC Working Group in the assessment of RF carcinogenicity (24). In general, about half of the available studies found genotoxicity (positive reports), although other studies did not (negative reports) (23). Of note, a similar ratio of positive and negative RF studies was reported for other biological endpoints (67–69). The evident reason for this eventual inconsistency is strong dependence of the EMF effects on a number of physical and biological parameters, which significantly varied between studies. These dependencies were established for both ELF (70–72) and RF effects (24, 27).

Among other parameters, in human lymphocytes, an individual variability in chromatin response to ELF has been reported, which might suggest a stronger response in cells from EHS individuals (72). The same research group performed comparative studies on genotoxicity with cells from EHS and carefully matched control subjects (73–75). The response of lymphocytes to RF from GSM mobile phones (915 MHz) and power-frequency magnetic fields (50 Hz) was investigated (73). The 53BP1 protein, which participates in the formation of DNA repair foci at the location of DNA double-strand breaks (DSB), was analyzed by immunostaining in situ. Exposure to either 915 MHz or 50 Hz significantly condensed chromatin and inhibited the formation of DNA repair foci. The EMF-induced responses in lymphocytes from healthy and hypersensitive donors were similar but not identical to the stress response induced by heat shock. The effects of GSM on chromatin and DNA repair foci in lymphocytes from EHS were further confirmed (74, 75). Although individual variability was observed, effects of RF from mobile phones strongly

- 10.1515/reveh-2016-0011
Downloaded from PubFactory at 07/26/2016 08:26:15AM
via Slovak Academy of Science

depended on the carrier frequency/frequency channel (74–77). Regardless of the cell type (human lymphocytes, fibroblasts, or stem cells), the effects at the 905 MHz/GSM channel 74 on DNA repair foci and chromatin were consistently lower as compared to the effects at the 915 MHz/GSM channel 124. The data also indicated stronger effects of exposure to RF from UMTS mobile phone radiation at the frequency of 1947.4 MHz. These data provided evidence that different frequency channels of different types of mobile communications technologies should be tested separately in provocation studies with EHS. While some minor differences were detected, very similar ELF/RF effects were observed in cells from EHS and matched control subjects. It is likely that compensatory reactions at a more complex level of biological organization such as reactions of tissues, organs, and organ systems are less efficient in persons with EHS, thereby providing a stronger connection of the EMF cellular response with symptoms of hypersensitivity.

Neurological effects of EMF

Neurological and behavioral effects were among the earliest topics of research on potential adverse effects of ELF as well as RF EMFs (78, 79). Concerning epidemiological evidence, more than a decade before the seminal publication of Wertheimer and Leeper (48), Haynal and Regli reported in 1965 an approximately four-fold higher prevalence of a history of electrical engineering jobs in patients with amyotrophic lateral sclerosis (ALS) than in control subjects (80).

Functional, morphological, and biochemical changes at the cellular, tissue, and organism level, as well as behavioral changes have been studied under experimental conditions, and epidemiology has assessed the association between occupational and residential exposure to EMFs and neurodegenerative diseases as well as neurological symptoms.

Research has shown that EMFs (RF and ELF) have deleterious effects on brain neurons and brain functioning (81). Epidemiological research has also shown an increased risk for Alzheimer's and dementia from occupational and residential exposure to ELF.

Neurological effects of radio-frequency radiation

Early studies of RF are difficult to assess because the descriptions of exposure conditions are often insufficient to derive the relevant dosimetric quantities. As early as 1932, Schliephake (82) reported effects that he considered to be non-thermal: *„Es treten Erscheinungen auf, wie wir sie bei Neurasthenikern zu sehen gewohnt sind: starke Mattigkeit am Tag, dafür in der Nacht unruhiger Schlaf, zunächst ein eigenartig ziehendes Gefühl in der Stirn und Kopfhaut, dann Kopfschmerzen, die sich immer mehr steigern, bis zur Unerträglichkeit. Dazu Neigung zu depressiver Stimmung und Aufgeregtheit.“* [*"Phenomena occur that we are accustomed to seeing in neurasthenics: pronounced fatigue during the day, however, restless sleep at night, in the beginning, a peculiar pulling sensation on the forehead and scalp, and then headaches that increase beyond the limit of tolerance. In addition, a tendency to depressive moods and agitation"*.] Such symptoms, not unlike those later summarized as microwave or radio wave sickness syndrome, have been found in a substantial percentage of exposed workers in the Soviet Union (83) and also in individuals presenting as electrohypersensitive (see below).

Experimental research in humans was scarce before the advent of digital mobile telephony. Since the earliest studies (84, 85) on brain electrical activity, a large evidence base has been compiled that indicates subtle changes in CNS function after and during short-term exposure to different types of RF. Experimental investigations were predominantly about effects on EEG power spectra (e.g. 86–96), event related potentials (e.g. 97–104), sleep (e.g. 105–119) and cognitive function (e.g. 120–131). A few investigations were about effects on glucose metabolism (132, 133) and regional cerebral blood flow (134, 135), applying PET scan imaging. Animal studies covered a wide variety of behavioral aspects, ranging from learning and memory (e.g. 136–141) to anxiety-related behavior (142).

The reaction of the CNS to RF is not restricted to the presence of the exposure but persists for some time after the exposure, making short-term cross-over studies uninformative. The location of exposure could be of relevance under certain circumstances, but often effects are bilateral after unilateral exposure, suggesting involvement of subcortical structures. Effects on sleep may depend on individual characteristics, which led to the conclusion that conflicting results are not strong evidence against an effect (113). Pulsed RF is more effective than continuous waves, but there is some evidence of the importance of exposure characteristics including the site of coupling of the RF field and its modulation.

In the 2012 update of the BioInitiative Report, Henry Lai summarized the experimental evidence as follows (143): *"Almost all the animal studies reported effects, whereas more human studies reported no effects than effects. This may be caused by several possible factors:* (a) *Humans are less susceptible to the effects of RFR than*

- 10.1515/reveh-2016-0011
Downloaded from PubFactory at 07/26/2016 08:26:15AM
via Slovak Academy of Science

are rodents. (b) *It may be more difficult to do human than animal experiments, since it is, in general, easier to control the variables and confounding factors in an animal experiment.* (c) *In the animal studies, the cumulative exposure duration was generally longer and studies were carried out after exposure, whereas in the human studies, the exposure was generally one time and testing was done during exposure. This raises the question of whether the effects of RFR are cumulative"*.

Neurological effects of extremely low frequency electromagnetic fields (ELF EMF)

Neurophysiological investigations of ELF EMFs were already conducted in the 1970s. Studies of chick and cat brain tissue (e.g. 144–146) revealed effects of weak ELF EMFs and ELF modulated RF fields that depended on intensity and frequency (so-called window effects). Adey proposed in 1981 (147) that effects are due to a primary interaction of EMFs at the cell membrane surface inducing a cascade of intracellular processes. This early insight has been corroborated by recent studies on various transmitter receptors in the brain such as N-methyl-D-aspartate receptors, dopamine and serotonin receptors (e.g. 148–151). Some of these more recent studies also reported frequency window effects as well as intensity window effects on the neurodevelopment in the rat (152).

Behavioral effects of ELF EMF have been studied at rather high levels in the 1970s and 1980s (e.g. 153, 154), while recent studies include low-level exposures and support effects on behavior at different levels of complexity. These include: changes in locomotor activity (e.g. 148, 149, 155, 156), anxiety (e.g. 157–159) and depression-like behavior (160, 161). *"Since different behavioral effects have been observed in different exposure conditions, species of animals, and testing paradigms, they provide the strongest evidence that exposure to ELF EMF can affect the nervous system"*. (Lai, 2012, BioInitiative Report, section 9, Evidence for effects on neurology and behavior effects, 143). Also in humans, effects were reported at low levels (e.g. 162–164).

Neurodegenerative diseases

The most prevalent of neurodegenerative diseases is Alzheimer's disease with an estimated 45 million patients worldwide for 2015, followed by Parkinson's disease, Huntington's disease, amyothrophic lateral sclerosis (ALS), and other motoneuron diseases (MND). To date, the pathophysiology of these diseases is incompletely understood. In many of these diseases, atypical protein assemblies, mitochondrial dysfunction, and programmed cell death play a role and some genetic changes have been detected. As some such changes could be a consequence of oxidative stress (see below), disruption of calcium homoeostasis, and disturbance of intracellular signaling pathways, there is a theoretical possibility that EMFs could contribute to the risk of these diseases. Since the 1980s, more than 30 epidemiological studies assessing the potential relationship between exposure to ELF EMFs and neurodegenerative diseases have been conducted. In the last years, several meta-analyses have been published. Concerning Parkinson's disease, there is little evidence of an association (165). Concerning ALS, Zhou et al. (166) summarize their results as follows: *"Although there are potential limitations from study selection bias, exposure misclassification, and the confounding effect of individual studies in this meta-analysis, our data suggest a slight but significant ALS risk increase among those with job titles related to relatively high levels of ELF EMF exposure"*. A review by Vergara et al. came to another conclusion (167): *"Our results do not support MF [magnetic fields] as the explanation for observed associations between occupational titles and MND"*. This discrepancy can be resolved by discriminating between different methods of endpoint assessment (incidence, prevalence or mortality data) and the potential for misclassification due to various sources of exposure data used. If these factors are considered, there is a consistent relationship between ELF EMF from occupational exposure and ALS/MND, and also the few studies about residential exposure are in line with an increased risk from exposure to MF (168).

Blood-brain barrier

All exchanges between blood and brain are strictly regulated by the blood-brain barrier (BBB). The BBB prevents the passage of various molecules from the blood into the brain and vice versa. An increase in a normally low BBB permeability for hydrophilic and charged molecules could potentially be detrimental. While the data on ELF effects are very sparse, several research groups investigated whether RF affects the BBB. These data have recently been reviewed (169–171). Although some BBB studies reported negative data, other studies, including replicated studies with rats from the Swedish group of Leif Salford and Bertil Persson, suggested that RF from mobile phones may affect the BBB under specific exposure conditions (171). More recent studies showing EMF effects at specific conditions of

- 10.1515/reveh-2016-0011
Downloaded from PubFactory at 07/26/2016 08:26:15AM
via Slovak Academy of Science

exposure (150, 172, 173) and not showing effects on the BBB under other conditions (174) are in line with this suggestion.

EMF and infertility and reproduction

Infertility and reproduction disorders are on the rise. Based on the BioInitiative Report (175), it should be concluded that men who use – and particularly those who wear a mobile phone, personal digital assistant (PDA) or pager on their belt or in a pocket – show adverse effects on sperm quality, motility, and pathology. The usage of mobile phones, the exposure to mobile phone radiation, or the storage of a mobile phone close to the testes of human males affects sperm count, motility, viability, and structure (176–184). Animal studies have demonstrated oxidative and DNA damage, pathological changes in the testes of animals, decreased sperm mobility and viability, and other measures of deleterious damage to the male germ line (182, 185–188).

There are also some studies of adverse birth outcomes in EMF-exposed women. A case-control study (189) and a population-based prospective cohort study (190) from California showed an association between miscarriage and the maximum value measured by a 24-h body-worn magnetic field dosimeter.

Electromagnetic hypersensitivity (EHS)

An increasing number of humans are continuously exposed in their daily life to increasing levels of a combination of static, ELF and VLF (very low frequencies, in general terms from 3 kHz to 3 MHz, in detailed terms from 3 kHz to 30 kHz) electric and magnetic fields and RF electromagnetic fields. These exposures are of different signal patterns, intensities, and technical applications for varying periods of time. All these fields are summarized as EMF, colloquially referred to as “electrosmog”.

Some historical examples of EHS from as early as 1932 (82, 83) are given in the chapter “Neurological effects of radio-frequency radiation”.

In a questionnaire survey in Switzerland in 2001, which was addressed to persons attributing specific health problems to EMF exposure, of the 394 respondents 58% suffered from sleep problems or disorders, 41% from headaches, 19% from nervousness, 18% from fatigue, and 16% from difficulties with concentration. The respondents attributed their symptoms to, e.g. mobile phone base stations (74%), mobile phones (36%), cordless phones (29%), and high-voltage power lines (27%). Two thirds of the respondents had taken measures to reduce their symptoms, the most frequent one being to avoid exposure (191).

In 2001, 63 persons who attributed health problems to environmental exposure were counseled in an interdisciplinary environmental medicine pilot project in Basel. An interdisciplinary expert team assessed the individual symptoms by a medical psychological-psychiatric and environmental examination, including visits and environmental measurements at home. With respect to the 25 persons with EHS, the expert team attested to the fact that in one third of them at least one symptom was plausibly related to electrosmog, although the EMF exposure was within the Swiss limits. They concluded that patients with EHS should be advised medically, psychologically, and environmentally (192, 193).

A questionnaire study of Finns (n=206), who describe themselves as suffering from electromagnetic hypersensitivity (EHS), revealed that the most common symptoms were related to the nervous system: stress (60%), sleeping disorders (59%) and fatigue (57%). The sources that were most often reported to have triggered EHS were: personal computers (51%) and mobile phones (47%). For 76% of the participants the reduction or avoidance of electromagnetic fields (EMF) helped in their full or partial recovery (194).

A representative telephone survey (n=2048; age>14 years) carried out in Switzerland in 2004 yielded a frequency of 5% (95% CI 4% to 6%) for having symptoms attributed to electrosmog, so-called EHS. In n=107 EHS persons, the most common symptoms being sleep problems (43%), headache (34%), and concentration difficulties (10%). Remarkably, only 13% consulted their family doctor. Individuals with a past history of symptoms attributable to EMF gave “turned off the source” as the answer to measures taken three times as often as the ones who still had symptoms (195).

In a Swiss questionnaire study of GPs in 2005, two-thirds of the doctors were consulted at least once a year because of symptoms attributed to EMF. Fifty-four percent of the doctors assessed a relation as possible. The doctors in this questionnaire asked for more general information about EMF and health and instructions on how to deal with patients with EHS (196).

In another questionnaire study, also mandated by the Swiss Federal Government and performed by the University of Bern in 2004, Swiss doctors working with complementary diagnostic and therapeutic tools reported that 71% of their consultations related to EMF. Remarkably, not only the patients but even more so the doctors suspected a possible relation between illness and EMF. The reduction or elimination of environmental sources was the main

- 10.1515/reveh-2016-0011
Downloaded from PubFactory at 07/26/2016 08:26:15AM
via Slovak Academy of Science

therapeutic instrument in treating symptoms related to EMF (197).

A questionnaire study of Austrian doctors yielded similar results. In this study, the discrepancy between the physicians' opinions and established national and international health risk assessments was remarkable, considering that 96% of the physicians believed to some degree in or were totally convinced of a health-relevant role of environmental electromagnetic fields (198).

In a survey conducted 2009 in a Japanese EHS and multiple chemical sensitivity (MCS) self-help group (n = 75), 45% of the respondents had EHS as a medical diagnosis and 49% considered themselves EHS. Every second respondent had medically diagnosed MCS (49%) and 27% had self-diagnosed MCS. The main EHS-related symptoms were fatigue, headache, concentration problems, sleep disorders, and dizziness. The most frequent causes included base stations, other persons' mobile phones, PC, power lines, television, own mobile phone, public transportation, cordless phones, air conditioner, and car. Suspected EMF source of EHS onset were: mobile phone base stations, PC, electric home appliances, medical equipment, mobile phones, power lines, and induction cookers (199).

In 2010, Khurana et al. reported that eight out of ten epidemiological studies that assessed health effects of mobile phone base stations reported an increased prevalence of adverse neurobehavioral symptoms or cancer in populations living at distances within 500 m from base stations. None of the studies reported exposure levels above accepted international guidelines, suggesting that current guidelines may be inadequate in protecting the health of human populations (200).

Carpenter reported in 2015 (201) a series of healthy people that developed EHS after a brief, high-intensity microwave radiation exposure. Typical symptoms included, for example, chronic headaches, irritability, and emotional lability, decreased libido, and memory problems, which in some patients, lasted for years.

Hedendahl et al. (19) reported two 15-year-old male students and one 47-year-old female teacher who experienced health effects like headaches, difficulties concentrating, tachycardia, poor memory, or dizziness when exposed to Wi-Fi in school. This example is mentioned to point specifically to the potential health impacts from increasing RF exposure of students and teachers by Wi-Fi.

The question, whether EHS is causally associated with EMF exposure is controversially discussed. On the one hand, physicians judge a causal association between EMF exposures as plausible based on case reports, on the other hand, national and international health risk assessments mostly claim that there is no such causal association, because provocation studies under controlled blinded conditions mostly failed to show effects. However, these studies have severe shortcomings that must be addressed: sequences of exposure conditions were often contiguous neglecting aftereffects of exposure; the exposure duration and the examined effects were short-term; the sham exposure was frequently under conditions that could provoke arousal in sensitive individuals; the time frame neglected the temporal conditions of symptom occurrence and disappearance, and/or the recruitment of persons with EHS was not medically assessed.

The WHO does not consider EHS as a diagnosis and recommends to medical doctors that the treatment of affected individuals should focus on the health symptoms and the clinical picture, and not on a person's perceived need for reducing or eliminating EMF in the workplace or at home (202). Based on the existing evidence and practical knowledge this view ignores a causal approach; see also (203).

The paper "Electromagnetic hypersensitivity: fact or fiction" by Genuis and Lipp (204) offers an instructive review of studies of the last decades concerning EHS, including historical milestones, reviews, pathogenesis, biochemical markers, therapeutic management, as well as the debate about the legitimacy of EHS.

In facial skin samples of electrohypersensitive persons, a profound increase of mast cells has been found (205). From this and other earlier studies when EHS manifested itself often during exposure to EMFs from cathode ray tubes (CRT), it became clear that the number of mast cells in the upper dermis is increased in the EHS group. A different pattern of mast cell distribution also occurred in the EHS group. Finally, in the EHS group, the cytoplasmic granules were more densely distributed and more strongly stained than in the control group, and the size of the infiltrating mast cells was generally found to be larger in the EHS group as well. It should be noted that increases of a similar nature were later demonstrated in an experimental situation, employing normal healthy volunteers in front of CRT monitors, including ordinary household television sets (206).

A French research group headed by Belpomme (207) investigated prospectively, since 2009, self-reported cases of EHS and/or MCS clinically and biologically in an attempt to establish objective diagnostic criteria and to elucidate the pathophysiological aspects of these two disorders. Based on 727 evaluable cases, the investigation showed a number of new and important insights such as:

(a) None of the biomarkers so far identified in the study are specific for EHS and/or MCS.
(b) Several biomarkers like histamine, nitrotyrosine, and circulating antibodies against O-myelin were

- 10.1515/reveh-2016-0011
Downloaded from PubFactory at 07/26/2016 08:26:15AM
via Slovak Academy of Science

increased. The 24-h urine melatonin/creatinine ratio was decreased.
(c) EHS and MCS are genuine somatic pathological entities.
(d) Under the influence of EMFs and/or chemicals a cerebral hypoperfusion/hypoxia-related neuroinflammation may occur.
(e) EHS and/or MCS patients might be potentially at risk of chronic neurodegenerative diseases and cancer.

While a 2006 study by Regel et al. (208) described no exposure effects, two provocation studies on exposure of "electrosensitive" individuals and control subjects to mobile phone base station signals (GSM, UMTS, or both) found a significant decline in well-being after UMTS exposure in the individuals reporting sensitivity (209, 210). Most so-called provocation studies with EHS show no effects. However, all these studies used a very limited number of exposure conditions and most have methodological weaknesses. Taking in account the strong dependence of EMF effects on a variety of physical and biological variables (27), available provocation studies are scientifically difficult to interpret and, in fact, are not suitable to disprove causality.

There is increasing evidence in the scientific literature of various subjective and objective physiological alterations, e.g. heart-rate variability (HRV) as apparent in some persons with EHS claiming to suffer after exposure to certain frequencies of RF like DECT or Wi-Fi (211–215). Analysis of the data available on the exposure of people living near mobile phone base stations has yielded clear indications of adverse health effects like fatigue, depression, difficulty in concentrating, headaches, dizziness, etc. (216–220). A synopsis of 30 studies on mobile phone base stations is given in the document "Leitfaden Senderbau" (221).

Residential EMF exposures in the VLF frequency range are often due to "dirty power"/"dirty electricity" originating from voltage and/or current perturbations from diverse sources like electronic power supplies for TVs, monitors, PCs, motor drives, inverters, dimmers, compact fluorescent lamps (CFLs), phase-angle control devices, as well as sparking and arcing from switching operations and from electric motors with brushes. The kHz waves/transients travel along the electric wiring and grounding systems (conducted emissions) and radiate electric and/or magnetic fields into free space (radiated emissions), leading to human exposures in the vicinity.

First epidemiological evidence links dirty electricity to most of the diseases of civilization including cancer, cardiovascular disease, diabetes, suicide, and attention deficit hyperactivity disorder in humans (222).

While the dependence of ELF effects on the local magnetic field has been reported by many research groups (13, 223), there are also a few studies which suggest that the RF effects are also dependent on slight changes in the local static magnetic field. In the review by Belyaev (224), a physical mechanism has been suggested to account for such effects (225). Slight changes in the local static magnetic field within 10 μT, which are usually observed within offices and homes due to ferromagnetic objects, were reported to induce biological effects that corresponded well to the predictions following from the mechanism of ion interference developed by Binhi (226).

On July 8, 2015, a court in Toulouse, France, ruled in favor of a woman with the diagnosis "syndrome of hypersensitivity to electromagnetic radiation" and determined her disability to be 85% with substantial and lasting restrictions on access to employment (227).

In France, the first low-EMF zone has been established at Drôme in July 2009 (228). In Austria, the construction of a multi-family house has been planned for 2015, which was designed by a team of architects, building biology professionals, and environmental medicine health care professionals to provide a sustainable healthy living environment. Both the outdoor and indoor environments were explicitly chosen and designed to meet low-EMF requirements (229). The implementation of low-EMF zones for electrosensitive individuals is pursued in numerous countries. The realization of such projects greatly depends on the understanding, knowledge, and tolerance of the members of the chosen community.

Possible mechanism of EHS

Based on the scientific literature on interactions of EMF with biological systems, several mechanisms of interaction are possible (14, 13, 22, 26). A plausible mechanism at the intracellular and intercellular level, for instance, is an interaction via the formation of free radicals or oxidative and nitrosative stress (230–238). It has been shown in many reports reviewed by Georgiu (15) that reactive oxygen species (ROS) may be involved in radical pair reactions; thus, radical pairs may be considered as one of the mechanisms of transduction able to initiate EMF-induced oxidative stress. Furthermore, many of the changes observed in RF-exposed cells were prevented by (pre)treatment with antioxidants and radical scavengers (24). While the data from different studies should be interpreted with care in view of variations in physical and biological parameters, a majority of the studies have shown effects of ELF and RF on the oxidative stress (239).

- 10.1515/reveh-2016-0011
Downloaded from PubFactory at 07/26/2016 08:26:15AM
via Slovak Academy of Science

The IARC monograph states: *"even small effects on radical concentration could potentially affect multiple biological functions"*, page 103 (24).

Yakymenko et al. (238) have summarized the current evidence: *"Analysis of the currently available peer-reviewed scientific literature reveals molecular effects induced by low-intensity RFR in living cells; this includes significant activation of key pathways generating reactive oxygen species (ROS), activation of peroxidation, oxidative damage of DNA and changes in the activity of antioxidant enzymes. It indicates that among 100 currently available peer-reviewed studies dealing with oxidative effects of low-intensity RFR, in general, 93 confirmed that RFR induces oxidative effects in biological systems. A wide pathogenic potential of the induced ROS and their involvement in cell signaling pathways explains a range of biological/health effects of low-intensity RFR, which include both cancer and non-cancer pathologies"*.

Reviews by Pall (12, 16, 240) provide evidence for a direct interaction between static and time-varying electric fields, static and time-varying magnetic fields and electromagnetic radiation with voltage-gated calcium channels (VGCCs). The increased intracellular Ca^{2+} produced by such VGCC activation may lead to multiple regulatory responses, including increased nitric oxide levels produced through the action of the two Ca^{2+}/calmodulin-dependent nitric oxide synthases, nNOS and eNOS. In most pathophysiological contexts, nitric oxide reacts with superoxide to form peroxynitrite, a potent non-radical oxidant, which can produce radical products, including hydroxyl and NO_2 radicals.

Peroxynitrite is by far the most damaging molecule that occurs during metabolism in our body. Although not a free radical, peroxynitrite is much more reactive than its parent molecules NO and O_2^-. The half-life of peroxynitrite is comparatively long (10–20 ms), sufficient to cross biological membranes, diffuse one to two cell diameters, and allow significant interactions with most critical biomolecules and structures (cell membranes, nucleus DNA, mitochondrial DNA, cell organelles), and a large number of essential metabolic processes (225). Elevated nitrogen monoxide, formation of peroxynitrite, and induction of oxidative stress can be associated with chronic inflammation, damage of mitochondrial function and structure, as well as loss of energy, e.g. via the reduction of adenosine triphosphate (ATP).

A significant increase of 3-nitrotyrosine was observed in the liver of Wistar rats exposed to ELF, suggesting a deteriorative effect on cellular proteins due to possible formation of peroxynitrite (241). Nitrotyrosin was found to be increased (>0.9 µg/mL) in 30% of the 259 tested EHS individuals (207).

A study by De Luca et al., in 2014 on 153 EHS and 132 controls showed metabolic pro-oxidant/pro-inflammatory alterations in EHS like decreased erythrocyte glutathione S-transferase (GST) activity, decreased reduced glutathione (GSH) levels, increased erythrocyte glutathione peroxidase (GPX) activity, an increased ratio of oxidized-CoQ10/total-CoQ10 in plasma, and a 10-fold increased risk associated with EHS for the detoxifying enzymes glutathione S transferase haplotype (null) GSTT1+(null) GSTM1 variants (242).

The importance of ATP has been shown for chronic fatigue syndrome (CFS) (243) and for stress control (244). Those patients describe the same symptoms as those suffering from CMI. This could indicate similarities in their pathomechanisms. Similar disturbances in neurotransmitter expression has been described both with chronic exposure to EMF (245) and in CMI patients (232, 246).

A study (247) proposed to investigate a possible association between RF exposure and myelin integrity via classical immunohistochemical markers for healthy and degenerated myelin, respectively, and for Schwann cells in general.

Complaints in chronic fatigue syndrome (CFS), fibromyalgia (FM), multiple chemical sensitivity (MCS), post-traumatic stress disorder (PTSD), and Gulf War syndrome (GWS) are almost the same. Meanwhile, they are summarized as chronic multisystem illnesses (CMI) (246). In all of them, various disturbances of functional cycles have been shown: activation of nitrogen oxide and peroxynitrite, chronic inflammation by activation of NF-kB, IFN-y, IL-1, IL-6, and interaction with neurotransmitter expression (232, 246, 248). We recommend classifying EHS as part of CMI (232, 249), but still recognizing that the underlying cause remains the environment (see Figure 1).

Other diseases that require attention with respect to EMF

Based on interactions between EMF exposure and biological responses that, e.g. lead to a disturbance of the oxidative/nitrosative homeostasis, a variety of diseases are possible and even expected to occur. Some examples are given here.

Havas reported in 2008 (250): *"Transient electromagnetic fields (dirty electricity), in the kilohertz range on electrical wiring, may be contributing to elevated blood sugar levels among diabetics and prediabetics. By closely following plasma glucose levels in four Type 1 and Type 2 diabetics, we find that they responded directly to the amount of dirty electricity in their environment. In an electromagnetically*

- 10.1515/reveh-2016-0011
Downloaded from PubFactory at 07/26/2016 08:26:15AM
via Slovak Academy of Science

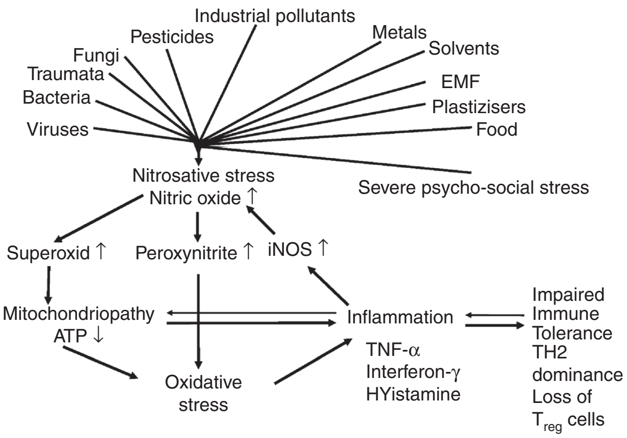

Figure 1: Pathogenesis of inflammation, mitochondriopathy, and nitrosative stress as a result of the exposure to trigger factors (248).

clean environment, Type 1 diabetics require less insulin and Type 2 diabetics have lower levels of plasma glucose. Dirty electricity, generated by electronic equipment and wireless devices, is ubiquitous in the environment. Exercise on a treadmill, which produces dirty electricity, increases plasma glucose. These findings may explain why brittle diabetics have difficulty regulating blood sugar. Based on estimates of people who suffer from symptoms of electrical hypersensitivity (3%–35%), as many as 5–60 million diabetics worldwide may be affected".

With respect to fetal and early childhood exposures to EMF, Sage in the BioInitiative Report 2012 (56) pointed out: *"Fetal (in-utero) and early childhood exposures to cell phone radiation and wireless technologies in general may be a risk factor for hyperactivity, learning disorders and behavioral problems in school." [&] "Common sense measures to limit both ELF EMF and RF EMF in these populations is needed, especially with respect to avoidable exposures like incubators that can be modified; and where education of the pregnant mother with respect to laptop computers, mobile phones and other sources of ELF EMF and RF EMF are easily instituted".*

In a 2013 review, Herbert and Sage (251, 252) reported remarkable similarities between pathophysiological phenomena found in autism spectrum conditions (ASCs) and the physiological impacts of ELF MF/RF, such as oxidative stress, free radical damage, malfunctioning membranes, mitochondrial dysfunction, inflammatory issues, neuropathological disruption and electrophysiological dysregulation, cellular stress proteins and deficiencies of antioxidants such as glutathione.

In a 6-year study, certain blood hormone levels were monitored in volunteers. Mobile phone use as well as close distances to mobile phone base stations were associated with decreased testosterone levels in males, as well as decreased ACTH, cortisol, T3 and T4 levels in males and females (253).

Recommendations for action

EUROPAEM has developed guidelines for differential diagnosis and potential treatment of EMF-related health problems with the aim to improve/restore individual health outcomes and to propose strategies for prevention. These recommendations are further outlined below.

These recommendations are preliminary and in large parts, although related to the whole body of evidence rooted in the experience of the team, cannot in every detail be strictly considered evidence-based.

Evidence of treatment strategies for EMF-related illness including EHS

There are only a few studies assessing therapeutic approaches to EHS. The interdisciplinary based assessing and counseling of EHS in the Swiss Environmental Pilot Project performed in 2001 showed, in an evaluation interview half a year after counseling, that 45% of the persons with EHS had benefitted from realizing certain advice, e.g. changing the bedroom (192, 193).

In the 2005 Swiss questionnaire study of physicians working with complementary therapeutic tools, two-thirds chose exposure reduction as a principal tool, whereas complementary therapeutics were only chosen as a supplement (197).

Since 2008, the Swiss Society of Doctors for the Environment has run a small interdisciplinary environmental medicine counseling structure for patients with EHS, which is embedded in everyday practice with a central coordination and consultation office as well as a network of general practitioners interested in environmental medicine who perform environmental medical assessments and consultations based on a standard protocol. If necessary, environmental experts are consulted and home inspections are conducted. The aim of the assessments is to detect or rule out common diseases and to analyze the impact of suspected environmental burdens on the complaints in order to find individual therapeutic approaches. The main instrument of the assessment is an extensive medical and psycho-social history with an additional environmental history, including a systematic questionnaire and environmental key questions.

- 10.1515/reveh-2016-0011
Downloaded from PubFactory at 07/26/2016 08:26:15AM
via Slovak Academy of Science

In the first years, the project was scientifically assessed. In a questionnaire 1 year after counseling, 70% of the persons recommended the interdisciplinary based counseling structure and 32% of them considered the counseling as being helpful. Therefore, a model based on such an interdisciplinary concept, embedded in the family doctor's holistic and lasting concept of treatment, seems to be promising for a better therapeutic approach to EHS, also including accessibility measures targeted at the actual environment (254).

In Finland, psychotherapy is the officially recommended therapy for EHS. In a questionnaire study of EHS people in Finland, symptoms, perceived sources and treatments, the perceived efficacy of medical and complementary alternative treatments (CAM) in regards to EHS were evaluated by multiple choice questions. According to 76% of the 157 respondents, the reduction or avoidance of EMF helped in their full or partial recovery. The best treatments for EHS were given as weighted effects: dietary change (69.4%), nutritional supplements (67.8%), and increased physical exercise (61.6%). The official treatment recommendations of psychotherapy (2.6%) were not significantly helpful, or for medication (–4.2%) even detrimental. The avoidance of electromagnetic radiation and fields effectively removed or lessened the symptoms in persons with EHS (194, 255).

Response of physicians to this development

In cases of unspecific health problems (see Questionnaire) for which no clearly identifiable cause can be found – besides other factors like chemicals, non-physiological metals, molds – EMF exposure should, in principle, be taken into consideration as a potential cause or cofactor, especially if the person presumes it.

A central approach for a causal attribution of symptoms is the assessment of variation in health problems depending on time and location and individual susceptibility, which is particularly relevant for environmental causes such as EMF exposure.

Regarding such disorders as male infertility, miscarriage, Alzheimer's, ALS, blood sugar fluctuations, diabetes, cancer, hyperactivity, learning disorders and behavioral problems in school, it would be important to consider a possible link with EMF exposure. Some people with EHS might be misdiagnosed with multiple sclerosis (MS) since many of the symptoms are similar. This offers an opportunity to causally influence the course of the disease.

How to proceed if EMF-related health problems are suspected

The recommended approach to diagnosis and treatment is intended as an aid and should, of course, be modified to meet the needs of each individual case (see Figure 2).

1. History of health problems and EMF exposure
2. Medical examinations and findings
3. Measurement of EMF exposure
4. Reduction and prevention of EMF exposure
5. Diagnosis
6. Treatment of the patient including the environment

History of health problems and EMF exposure

In order to put later findings into a larger context, a general medical history is necessary. Part of this history should include:

- Electrical trauma: multiple shocks, electrocution, struck by lightning.
- Chemical trauma: exposure to pesticides, metals, chlorinated hydrocarbons (PCBs, DDT, etc.)

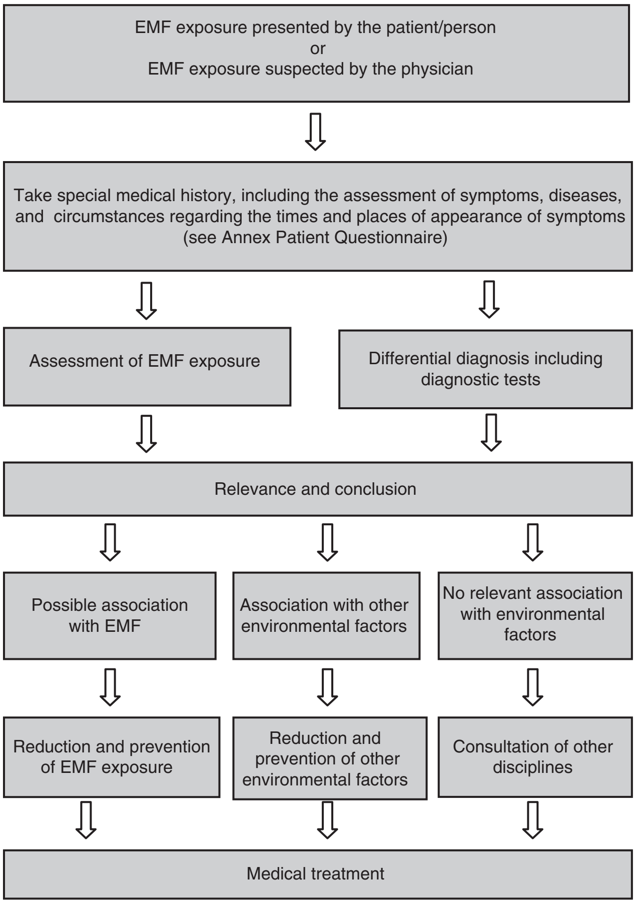

Figure 2: Flowchart for the handling of EMF-related health problems.

- 10.1515/reveh-2016-0011
Downloaded from PubFactory at 07/26/2016 08:26:15AM
via Slovak Academy of Science

- Biological trauma in the form of a large load of parasites, fungal infections, viral infections, etc.
- Physical trauma to the central nervous system in the form of whiplash, other accidents, spinal problems
- Autoimmune disorders

In the next steps, we focus only on EMF-related health effects.

A questionnaire to take a systematic history of health problems and EMF exposure, compiled by the EUROPAEM EMF Working Group, is available in the Annex of this EMF Guideline.

The questionnaire consists of three sections:

(a) List of symptoms
(b) Variation of health problems depending on time, location, and circumstances
(c) Assessment of certain EMF exposures that can be evaluated by questionnaire

The list of symptoms in the questionnaire serves to systematically quantify health problems regardless of their causes. It also includes questions as to when the health problems first occurred. Most EMF-related symptoms are nonspecific and fall within the scope of health problems due to inadequate regulation (decompensation), e.g. sleep problems, fatigue, exhaustion, lack of energy, restlessness, heart palpitations, blood pressure problems, muscle and joint pain, headaches, increased risk for infections, depression, difficulty concentrating, disturbances of coordination, forgetfulness, anxiety, urinary urgency, anomia (difficulty finding words), dizziness, tinnitus, and sensations of pressure in the head and ears.

The health problems may range in severity from benign, temporary symptoms, such as slight headaches or paresthesia around the ear, e.g. when using a mobile phone, or flu-like symptoms after maybe some hours of whole-body EMF exposure, to severe, debilitating symptoms that drastically impair physical and mental health. It has to be stressed that, depending on the individual state of susceptibility, EHS symptoms often occur only occasionally, but over time they may increase in frequency and severity. On the other hand, if a detrimental EMF exposure is sufficiently reduced, the body has a chance to recover and EHS symptoms will be reduced or will vanish.

Variation of health problems depending on time, location, and circumstances

The answers to questions of when and where the health problems occur or recede, and when and where the symptoms increase or are particularly evident, provide only indications. They must be interpreted by the investigator (e.g. regarding the correct attribution between location/EMF sources and health problems). Special attention should be drawn to sleeping areas, because of the duration of influence and the vital role of sleep for regeneration.

Assessment of certain EMF exposures that can be evaluated by questionnaire

The assessment of EMF exposure usually starts with certain questions of usual EMF sources. Regardless of whether or not the patient suspects EMF exposure as a cause, these questions should be used to assess the existing exposure level, at least as a rough estimate. It is important to note that only certain types of EMF exposure can be assessed by means of questions, such as the use of compact fluorescent lamps, mobile phones, and cordless phones. Detection of other types of EMF exposure, e.g. due to RF transmitter sites or the electric or magnetic fields from electric wiring, generally requires measurements. In principle, questions should be asked to assess EMF exposure at home and at work and when on holidays and so on, keeping in mind that the degree of EMF exposure may vary at different times.

Medical examinations and findings

We do not have any clinical findings yet that are specific to EMF, which makes diagnosis and differential diagnosis a considerable challenge.

A method that has proven useful is to use stress-associated findings for diagnosis and follow-up and to evaluate them synoptically. Basic diagnostic tests should be carried out as a first step, followed by measurements of EMF exposure as a second step. The core diagnosis should focus on investigations of nitric oxide production (nitrotyrosine), mitochondriopathy (intracellular ATP), oxidative stress-lipid peroxidation (MDA-LDL), inflammation [TNF-alpha, IFN-gamma-inducible protein 10 (IP-10), IL-1b, histamine], and the melatonin status (24 h urine melatonin/creatinine ratio).

Then additional diagnostic tests can be considered. Due to the differences in normal ranges between labs and different practices as to the units of measurement in different countries, we do not provide levels to be considered relevant in EHS. It is recommended to interpret them in context, focusing not only on out-of-range values. For example, when several parameters are simultaneously close to the border of the normal ranges, this could be instructive for forming a therapeutic or diagnostic opinion.

- 10.1515/reveh-2016-0011
Downloaded from PubFactory at 07/26/2016 08:26:15AM
via Slovak Academy of Science

Functional tests

Basic diagnostic tests

- Blood pressure and heart rate (in all cases resting heart rate in the morning while still in bed), including self-monitoring, possibly several times a day, e.g. at different locations and with journaling of subjective well-being for a week.

Additional diagnostic tests

- 24-h blood pressure monitoring (absence of nighttime decline)
- 24-h ECG (heart rhythm diagnosis)
- 24-h heart rate variability (HRV) (autonomous nervous system diagnosis)
- Ergometry under physical stress
- Sleep EEG at home

Laboratory tests

Basic diagnostic tests

- Blood
  - ACTH
  - Bilirubin
  - Blood count and differential blood count
  - BUN
  - Cholesterol, LDL, HDL, triglycerides
  - Coenzyme-Q10 ratio for oxidized-CoQ10/total-CoQ10
  - Creatinine kinases (CK-MB, CK-MM)
  - High-sensitivity C-reactive protein (hs-CRP)
  - Cystatin C (glomerular filtration rate)
  - Electrolytes
  - Fasting blood glucose
  - Ferritin
  - Glutathione S-transferase (GST)
  - Reduced glutathione (GSH)
  - Glutathione peroxidase (GPX)
  - HBA_{1c}
  - Histamine and diaminoxidase (DAO)
  - IFN-gamma-inducible protein 10 (IP-10)
  - Interleukin-1 (e.g. IL-1a, IL-1b)
  - Intracellular ATP
  - Liver enzymes (e.g. ALT, AST, GGT, LDH, AP)
  - Magnesium (whole blood)
  - Malondialdehyde (MDA)-LDL
  - Nitrotyrosine (NTT)
  - Potassium (whole blood)
  - Prolactin
  - Selenium (whole blood)
  - Testosterone
  - TSH
  - T3, T4
  - Tumor necrosis factor alpha (TNFα)
  - Vitamin D3
  - Zinc (whole blood)
- Standard urine
  - Leucocytes, erythrocytes, albumin, urobilinogen, pH, bacteria, glucose, microalbumin
- Second morning urine
  - Adrenaline
  - Dopamine
  - Noradrenaline
  - Noradrenaline/adrenaline ratio
  - Serotonin
  - Beta-phenylethyleamine (PEA)
- 24-h urine
  - 6-OH melatonin sulfate
  - Creatinine
  - 6-OH melatonin sulfate/creatinine ratio
- Saliva
  - Cortisol (8 a.m., 12 a.m., and 8 p.m.)

Additional diagnostic tests

- Urine
  - Metals (depending on case history, e.g. mercury, cadmium, lead, arsenic, aluminum)
- Second morning urine
  - Gamma-aminobutyric acid (GABA)
  - Glutamate
  - Cryptopyrrole
- Saliva
  - Dehydroepiandrosterone DHEA (8 a.m. and 8 p.m.)
  - Alpha-amylase
- Blood
  - 8-Hydroxydeoxyguanosine (DNA oxidation)
  - Biotin
  - Differential lipid profile
  - Folate
  - Holotranscobolamin
  - Homocysteine
  - Interferon-gamma (IFN-γ)
  - Interleukin-10 (IL-10)
  - Interleukin-17 (IL-17)
  - Interleukin-6 (IL-6)
  - Interleukin-8 (IL-8)
  - Intracellular glutathione (redox balance)
  - Lactate, pyruvate incl. ratio
  - Lipase
  - NF-kappa B
  - Vitamin B6 (whole blood)

Provocation tests

Special facilities with the use of a variety of signals, e.g. DECT or Wi-Fi exposure (e.g. 20–60 min, depending on

- 10.1515/reveh-2016-0011
Downloaded from PubFactory at 07/26/2016 08:26:15AM
via Slovak Academy of Science

the individual regulation capacity, susceptibility, and observed response)
- Heart rate variability (HRV) (autonomous nervous system diagnosis)
- Microcirculation
- Oxidative stress (lipid peroxidation, malondialdehyde, oxo-LDL)
- For diabetics, plasma glucose
- Live blood analysis (red blood cell aggregation in the form of rouleaux, blood viscosity, macrophage activity, lysis of red blood cell membrane)
- For people with neurological problems and problems with fine or gross motor coordination, a video of them walking before and after provocation and a photograph taken of a sample of handwriting before and after provocation.

Individual susceptibility

- Blood (genetic parameters and actual function)
  - Glutathione S transferase M1 (GSTM1) – detoxification
  - Glutathione S transferase T1 (GSTT1) – detoxification
  - Superoxide dismutase 2 (SOD2) – protection of mitochondria
  - Catechol-O-methyltransferase (COMT) – stress control

Measurement of EMF exposure

The evolutionary development of the human species took place under the presence of the natural electromagnetic spectrum (Earth's magnetic field, Earth's electric field, spherics, Schumann resonance). Those influences have been part of our biosphere like the oxygen content in the air or the visible light spectrum, and they have been integrated into the biological functions (14).

By now, nearly all non-ionizing parts of the electromagnetic spectrum are filled with artificial, technical EMF sources due to electrification and (wireless) communication technologies, but are very rarely found in nature (see Figure 3). EMF measurements and/or exposure damages are usually not covered by statutory health care insurance.

In general, a wide variety of EMF exposure types (static fields, ELF, VLF, and RF) should be considered.
- ELF magnetic fields may originate from, e.g. 12 V transformers, transformer stations, net currents on the electric wiring, water pipes, and other conductive materials, infrared heaters, heating blankets and different types of power lines.
- ELF electric fields may originate from, e.g. electrical wiring, lamps, and appliances.
- VLF magnetic fields ("dirty power") and/or VLF electric fields ("dirty electricity") may be emitted from electronic

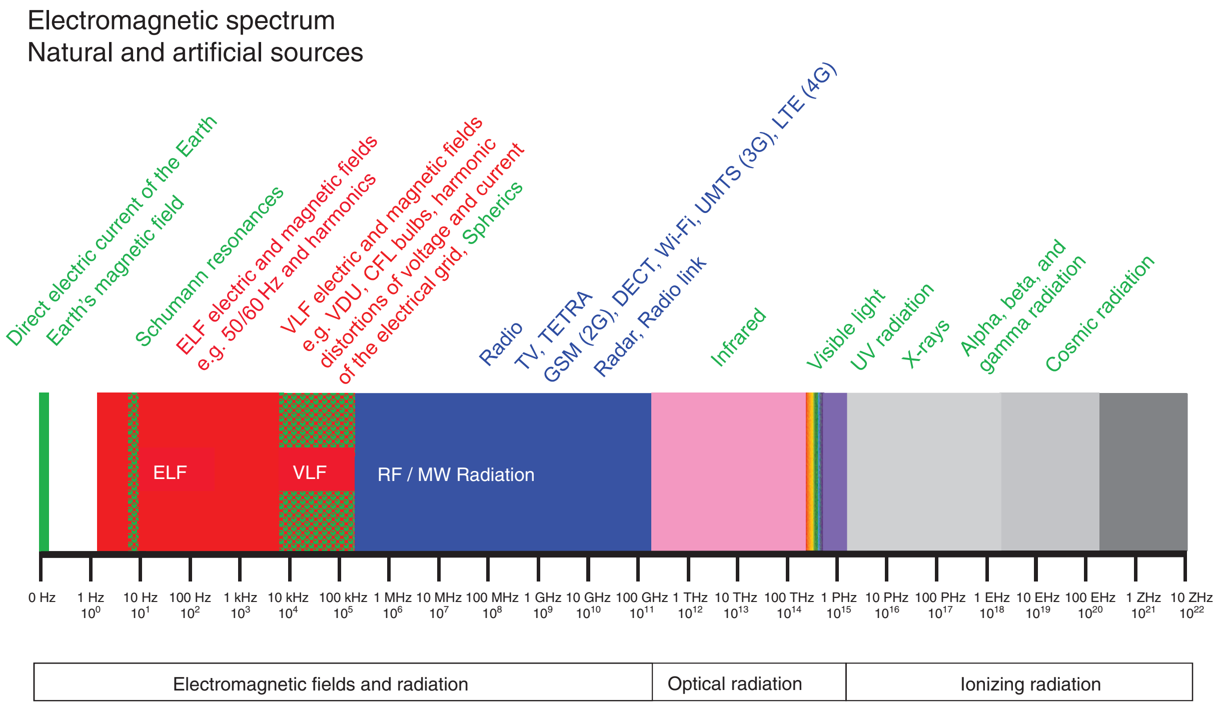

Figure 3: Examples of natural (green) and artificial (red and blue) EMF sources along the electromagnetic spectrum (256).

- 10.1515/reveh-2016-0011
Downloaded from PubFactory at 07/26/2016 08:26:15AM
via Slovak Academy of Science

devices like energy-efficient lighting, electronic transformers, induction cooker, variable speed frequency drives, light dimmer switches, power line communication (PLC) connected to the electrical grid. These devices use current and/or voltage in short pulses that might produce harmonics and VLF transients on the electrical circuits, earthed materials and the ground.
– Typical RF radiation sources include, e.g. cordless phones (DECT), wireless Internet access (Wi-Fi), mobile phones and their base stations, radio and TV broadcast antennas, radar (military, airport, marine, and weather), Bluetooth, and the microwave ovens.

In the sleeping area, the most important exposure point is the head and trunk region followed by all other points with chronic or high exposure.

EMF measurements should be planned and carried out by specially trained and experienced testing specialists and always in accordance with relevant standards, e.g. the VDB Guidelines of the German Association of Building Biology Professionals (257). In addition to the measurement results, the measurement report should also include suggestions on how to possibly reduce the EMF exposure.

To clarify certain issues, personal dosimeters with a data logging function are available to measure ELF magnetic fields and radio-frequency radiation.

After the measurements have been commissioned by the person and carried out, the results should be discussed with a physician familiar with the EMF issue.

EMF guidance values

In each case, the following aspects should be individually taken into account when evaluating EMF measurement results (27, 26):

- A person's individual susceptibility, which, e.g. may be based on previous history of trauma (electrical, chemical, biological and physical).
- A person's individual total body burden (e.g. exposure to noise, chemicals like neurotoxins)
- Duration of EMF exposure
- EMF exposure during the night and day
- Multiple exposure to different EMF sources
- Signal intensity: watt/m^2 (W/m^2), volt/m (V/m), ampere/m (A/m)
- Signal characteristics were taken into account in the EMF guidance values – see Supplement 3 (258)
  - Frequency
  - Risetime (ΔT) of bursts, transients, etc.
  - Frequency and periodicity of bursts, e.g. certain GSM base stations (8.3 Hz), Wi-Fi networks (10 Hz), DECT cordless phones (100 Hz)
  - Type of modulation (frequency modulation, amplitude modulation, phase modulation)

Regardless of the ICNIRP recommendations for specific acute effects, the following guidance values (Tables 1–3, 5 and 6) apply to sensitive locations with long-term exposure of more than 20 h per week (259). They are based on epidemiological studies (9, 10, 27, 221, 260–262), empirical observations, and measurements relevant in practice (258, 263), as well as recommendations by the Seletun Statement (40) and the Parliamentary Assembly of the Council of Europe (42). The proposed guidance values are based on scientific data including a preventive component and aim to help restore health and well-being in already compromised patients. All levels provided are for incident intensities and whole-body exposure.

ELF magnetic fields (extremely low frequency) (ELF MF) Measurement specifications

Frequency range: 50/60 Hz mains electricity, up to 2 kHz. 16.7 Hz railroad systems in Austria, Germany, Switzerland, Sweden, and Norway, 400 Hz on airplanes
Type of measurement: Magnetic induction or flux density [T; mT; μT; nT]
Field probe: Isotropic magnetic field probe (three orthogonal axes)
Detector mode: RMS (root mean square)
Measurement volume: Bed: Short-term measurements across entire sleeping area. Workplace: Short-term measurements across entire work area (e.g. sitting position). Long-term measurements: e.g. point close to the head/trunk in bed or at workplace
Measurement period: Short-term measurements to identify field sources. Long-term measurements during sleep and work shift
Basis for evaluation: Long-term measurements: maximum (MAX) and arithmetic mean (AVG)

Precautionary guidance values

In areas where people spend extended periods of time (>4 h per day), minimize exposure to ELF magnetic fields to levels as low as possible or below the precautionary guidance values specified below.

Table 1: Precautionary guidance values for ELF magnetic fields.

ELF magnetic field	Daytime exposure	Nighttime exposure	Sensitive populations
Arithmetic mean (AVG)	100 nT (1 mG)[1),2),3)]	100 nT (1 mG)[1),2),3)]	30 nT (0.3 mG)[5)]
Maximum (MAX)	1000 nT (10 mG)[2),4)]	1000 nT (10 mG)[2),4)]	300 nT (3 mG)[5)]

Based on: [1)]BioInitiative (9, 10); [2)]Oberfeld (262); [3)]Seletun Statement (40), [4)]NISV (264); [5)]Precautionary approach by a factor of 3 (field strength). See also IARC 2002 (30), Blank and Goodman (17), and TCO Development (265).

- 10.1515/reveh-2016-0011
Downloaded from PubFactory at 07/26/2016 08:26:15AM
via Slovak Academy of Science

Evaluation guidelines specifically for sleeping areas

Higher frequencies than the mains electricity at 50/60 Hz and distinct harmonics should be evaluated more critically. See also the precautionary guidance values for the VLF frequency range further below. If applicable, mains current (50/60 Hz) and traction current (16.7 Hz) should be assessed separately but added (squared average). Long-term measurements should be carried out especially at nighttime, but at least for 24 h.

ELF electric fields (extremely low frequency) (ELF EF) Measurement specifications

Frequency range: 50/60 Hz mains electricity, up to 2 kHz.16.7 Hz railroad systems in Austria, Germany, Switzerland, Sweden, and Norway
Type of measurement: Electric field [V/m] without ground reference (potential-free)
Field probe: Isotropic electric field probe (three orthogonal axes)
Detector mode: RMS (root mean square)
Measurement volume: Bed: Nine points across sleeping area. Workplace: Across entire work area (e.g. sitting position three or six points)
Measurement period: Spot measurements to assess the exposure as well as to identify field sources. Since electric field exposure levels in the ELF frequency range usually do not change, long-term measurements are not needed.
Basis for evaluation: Spot measurements (maximum) at relevant points of exposure

Precautionary guidance values

In areas where people spend extended periods of time (> 4 h per day), minimize exposure to ELF electric fields to levels as low as possible or below the precautionary guidance values specified below.

Table 2: Precautionary guidance values for ELF electric fields.

ELF electric field	Daytime exposure	Nighttime exposure	Sensitive populations
Maximum (MAX)	10 V/m[1), 2)]	1 V/m[2)]	0.3 V/m[3)]

Based on: [1)]NCRP Draft Recommendations on EMF Exposure Guidelines: Option 2, 1995 (261); [2)]Oberfeld (262); [3)]Precautionary approach by a factor of 3 (field strength). See also TCO Development (265).

Evaluation guidelines specifically for sleeping areas

Higher frequencies than the mains electricity at 50/60 Hz and distinct harmonics should be evaluated more critically. See also the precautionary guidance values for the VLF frequency range further below.

Radio-frequency radiation (RF) Measurement specifications

Frequency range: Radio and TV broadcast antennas, mobile phone base stations, e.g. TETRA (400 MHz),GSM (900 and 1800 MHz), UMTS (2100 MHz),LTE (800, 900, 1800, 2500–2700 MHz), cordless phone base stations, e.g. DECT (1900), Wi-Fi access points and clients (2450 and 5600 MHz), WiMAX (3400–3600 MHz). Above frequencies in MHz refer to European networks.
Type of measurement: Usually electric field [V/m] -> calculated power density [W/m²; mW/m²; µW/m²]; for conversion units see Table 4.
Field probe: Isotropic, biconical or logarithmic-periodic antennas
Detector mode: Peak detector with max hold
Measurement volume: Point of exposure across bed and workplace
Measurement period: Usually short-term measurements to identify RF field sources (e.g. acoustic analysis) and peak readings
Basis for evaluation: Band-specific or frequency-specific spot measurements (peak detector with max hold) of common signals at relevant points of exposure (e.g. with spectrum analyzer or at least band-specific RF meter)

Precautionary guidance values for selected RF sources

In areas where people spend extended periods of time (>4 h per day), minimize exposure to radio-frequency radiation to levels as low as possible or below the precautionary guidance values specified below. Frequencies to be measured should be adapted to each individual case. The specific guidance values take the signal characteristics of risetime (ΔT) *and periodic ELF "pulsing" into account* (258). *Note: Rectangular signals show short risetimes and consist of a broad spectrum of frequencies. The current density induced in the human body increases with increasing frequency in an approximately linear relationship* (266).

Table 3: Precautionary guidance values for radio-frequency radiation.

RF source Max Peak/ Peak Hold	Daytime exposure	Nighttime exposure	Sensitive populations[1)]
Radio broadcast (FM)	10,000 µW/m²	1000 µW/m²	100 µW/m²
TETRA	1000 µW/m²	100 µW/m²	10 µW/m²
DVBT	1000 µW/m²	100 µW/m²	10 µW/m²
GSM (2G) 900/1800 MHz	100 µW/m²	10 µW/m²	1 µW/m²
DECT (cordless phone)	100 µW/m²	10 µW/m²	1 µW/m²
UMTS (3G)	100 µW/m²	10 µW/m²	1 µW/m²
LTE (4G)	100 µW/m²	10 µW/m²	1 µW/m²
GPRS (2.5G) with PTCCH* (8.33 Hz pulsing)	10 µW/m²	1 µW/m²	0.1 µW/m²
DAB+ (10.4 Hz pulsing)	10 µW/m²	1 µW/m²	0.1 µW/m²
Wi-Fi 2.4/5.6 GHz (10 Hz pulsing)	10 µW/m²	1 µW/m²	0.1 µW/m²

*PTCCH, packet timing advance control channel.

Based on: BioInitiative (9, 10); Kundi and Hutter (260); Leitfaden Senderbau (221); PACE (42); Seletun Statement (40). [1)]Precautionary approach by a factor of 3 (field strength)= a factor of 10 (power density). See also IARC 2013 (24) and Margaritis et al. (267).

- 10.1515/reveh-2016-0011
Downloaded from PubFactory at 07/26/2016 08:26:15AM
via Slovak Academy of Science

Table 4: Conversion of radio-frequency radiation measurement units.

**Conversion**	mW/m²	10	1	0.1	0.01	0.001	0.0001
**of RF**	µW/m²	10,000	1000	100	10	1	0.1
**Measurement**	µW/cm²	1	0.1	0.01	0.001	0.0001	0.00001
**units**	V/m	1.9	0.6	0.19	0.06	0.019	0.006

Magnetic fields in the VLF range (VLF MF) Measurement specifications

Frequency range: 3 kHz–3 MHz. Frequency-specific measurements (spectrum analyzer/EMF meter), e.g. "dirty power", powerline communication (PLC), radio-frequency identification transmitters (RFID), compact fluorescent lamps (CFL)
Type of measurement: Magnetic field [A/m] – > calculated magnetic induction [T; mT; µT; nT]
Field probe: Isotropic or anisotropic magnetic field probe
Detector mode: RMS (root mean square)
Measurement volume: Point of exposure across bed and workplace
Measurement period: Short-term measurements to identify field sources. Long-term measurements during sleep and work shift
Basis for evaluation: Long-term measurements: RMS detector, arithmetic mean and maximum at relevant points of exposure
Note: If an elevated exposure is detected, power quality analyzers and oscilloscopes can be used on the actual wiring to trace the source of the dirty power.

Precautionary guidance values

In areas where people spend extended periods of time (>4 h per day), minimize exposure to VLF magnetic fields to levels as low as possible or below the precautionary guidance values specified below.

Table 5: Precautionary guidance values for VLF magnetic fields.

VLF magnetic field	Daytime exposure	Nighttime exposure	Sensitive populations
Arithmetic mean (AVG)	1 nT (0.01 mG)[1)]	1 nT (0.01 mG)[1)]	0.3 nT (0.003 mG)[2)]
Maximum (MAX)	10 nT (0.1 mG)[1)]	10 nT (0.1 mG)[1)]	3 nT (0.03 mG)[2)]

Based on: [1)]The current density induced in the human body increases with increasing frequency in an approximately linear relationship (266). Therefore, the guidance value of the magnetic field in the VLF frequency range should be lower than the one of the 50/60 Hz magnetic field, e.g. for 100 nT RMS/100=1 nT. For the rationale of 100 nT (avg) and 1 µT (max), see section ELF magnetic fields. [2)]Precautionary approach by a factor of 3 (field strength). See also TCO Development (265).

Electric fields in the VLF range (VLF EF) Measurement specifications

Frequency range: 3 kHz–3 MHz. Frequency-specific measurements (spectrum analyzer/EMF meter), e.g. "dirty electricity", powerline communication (PLC), radio-frequency identification transmitters (RFID), compact fluorescent lamps (CFL)
Type of measurement: Electric field [V/m]
Field probe: Isotropic, biconical, logarithmic-periodic electric field probe
Detector mode: RMS arithmetic mean
Measurement volume: Point of exposure across bed and workplace
Measurement period: Short-term measurements to identify field sources. Long-term measurements during sleep and work shift
Basis for evaluation: Long-term measurements: arithmetic mean at relevant points of exposure
Note: If an elevated exposure is detected, power quality analyzers and oscilloscopes can be used on the actual wiring to trace the source of the dirty power.

Precautionary guidance values

In areas where people spend extended periods of time (>4 h per day), minimize exposure to VLF electric fields to levels as low as possible or below the precautionary guidance values specified below.

Table 6: Precautionary guidance values for VLF electric fields.

VLF electric field	Daytime exposure	Nighttime exposure	Sensitive populations
Arithmetic mean (AVG)	0.1 V/m[1)]	0.01 V/m[1)]	0.003 V/m[2)]

Based on: [1)]The current density induced in the human body increases with increasing frequency in an approximately linear relationship (266). Therefore, the guidance value of the electric field in the VLF frequency range should be lower than the one of the 50/60 Hz electric field, e.g. for 10 V/m/100 = 0.1 V/m. For the rationale of 10 V/m and 1 V/m, see section ELF electric fields. [2)]Precautionary approach by a factor of 3 (field strength). See also TCO Development (265).

Reduction and prevention of EMF exposure

Preventing or reducing EMF exposure after consulting a testing specialist is advantageous for several reasons:

(a) To prevent and reduce risks to individual and public health,
(b) To identify any links to health problems,
(c) To causally treat the EMF-related health problems.

There are numerous potential causes of relevant EMF exposures, and this EMF guideline can only give a few examples. Further information can be found, for instance, in the document "Options to Minimize EMF/ RF/Static Field Exposures in Office Environments" (268) and "Elektrosmog im Alltag"

- 10.1515/reveh-2016-0011
Downloaded from PubFactory at 07/26/2016 08:26:15AM
via Slovak Academy of Science

(269). For detailed information on physics, properties, and measurement of EMF, see Virnich (270); regarding reduction of radio-frequency radiation (RF) in homes and offices, see Pauli and Moldan (271).

In most cases, it will be necessary to consult an expert (e.g. qualified EMF/RF engineer/ consultant) and/or electrician who will advise the person on what measures could be taken to reduce EMF exposure.

EMF exposure reduction – first steps

As a first step, recommendations are given (also as preventive measures) to eliminate or reduce typical EMF exposures, which may help alleviate health problems within days or weeks. The following actions may be suggested:

Preventing exposure to radio-frequency radiation (RF)

- Keep mobile phone/smartphone and cordless phone calls short; use the speakerphone function or a hands-free kit.
- Avoid wearing the mobile phone/smartphone close to the body.
- Deactivate all non-essential wireless mobile phone apps, which cause periodic radiation exposure.
- Keep mobile phones/smartphones in "airplane mode" whenever possible or deactivate mobile data, Wi-Fi, Bluetooth and near field communication (NFC) in the smartphone settings.
- Disconnect (unplug) the power supply of all DECT cordless phone base stations. So called "ECO Mode" or "zero-emission" DECT phones are only conditionally recommended because the exposure by the handset is still present. A "traditional" corded phone is recommended instead.
- Disconnect (unplug) the power supply to all Wi-Fi access points or Wi-Fi routers. Many LAN routers now come equipped with additional Wi-Fi. Call the provider of the LAN router and ask to have the Wi-Fi deactivated. It is usually also possible to do so online by following the provider's instructions.
- In case of external RF radiation sources, rooms – especially bedrooms – facing away from the source should be chosen.
- Avoid powerline communication for Internet access (dLAN) and instead use a hardwired Ethernet cable (LAN).
- Avoid exposure to RF radiation (e.g. wireless devices like, home entertainment, headsets, baby monitors, computer games, printers, keyboards, mouse, home surveillance systems) at home, in offices, and in cars.
- Avoid exposure to energy-efficient lighting (compact fluorescent lamps as well as some LEDs generate high frequency transients). These types of lamps can be replaced with incandescent or line-voltage halogen incandescent lamps until good-quality lighting energy-efficient lamps become commercially available.

Preventing exposure to ELF electric and magnetic fields

- Move the bed or desk away from the wiring in the walls and power cords. A minimum distance of 30 cm (1 ft) from the wall is recommended.
- As magnetic fields can pass through walls, make certain that there are no magnetic sources immediately beneath or above a bed or in an adjacent room.
- Another simple complementary action is to disconnect the power supply to the bedroom (turn off circuit breaker or fuse) for the nighttime while sleeping; try it for a test phase of, e.g. 2 weeks. In general, this measure is not always successful because circuits of adjacent rooms contribute to the electric field levels. ELF electric field measurements are required to know exactly which circuit breakers need to be disconnected. The benefits should be weighed against the potential risk of accidents; therefore, the use of a flashlight for the test phase should be recommended.
- Disconnect the power supply to all non-essential electric circuits, possibly in the entire apartment or house. (N.B. See note above.)
- Avoid using an electric blanket during sleep; not only turn it off, but also disconnect it.
- Avoid extended exposures close to running electric motors. As a first step, keep a minimum distance of 1.5 m (5 ft). As a second step, establish a safe distance based on magnetic field measurements.

Preventing exposure to static magnetic/static electric fields

- Sleep in a bed and mattress without metal.
- Avoid sleeping close to iron materials (radiator, steel, etc.)
- Wearing synthetic clothing and, e.g. rubber-soled shoes and not regularly being in contact with the earth can result in build up of static electricity. Cotton clothing and leather-soled shoes will help avoid static electricity.

EMF exposure reduction – second steps

As a second step, EMF measurements and mitigation measures should be carried out. Typical examples are:

- 10.1515/reveh-2016-0011
Downloaded from PubFactory at 07/26/2016 08:26:15AM
via Slovak Academy of Science

- Measure the ELF electric field in the bed. Based on the measurement results, install automatic demand switches in those circuits that increase the exposure.
- Measure the ELF electric field at all other places that are used for extended periods at home and at work. If necessary, choose lamps used close to the body with a shielded electric cable and a grounded lamp fixture (metal). Especially in lightweight construction (wood, gypsum board), electrical wiring without grounding (two-slot outlets) might have to be replaced with grounded electrical wiring or shielded electrical wiring. In special cases, shielded wiring and shielded outlets may have to be installed in the whole building.
- Measure the ELF magnetic field close to the bed, e.g. for 24 h. If net currents are detected, the electrical wiring and grounding system of the building must be corrected to reduce the magnetic fields.
- Install a residual current device (RCD) or ground-fault circuit interrupter (GFCI) to prevent electric shocks (safety measure).
- Measure radio-frequency radiation and mitigate high exposure levels by installing certain RF shielding materials for the affected walls, windows, doors, ceilings, and floors. For example, in a multiunit setting (condominiums or highrise apartments, townhomes), proximity to neighbors can contribute to inhome exposure.
- Measure dirty electricity/dirty power (electric and magnetic fields in the VLF frequency range) and identify the sources in order to remove them. If this is not possible, appropriate power filters in line with the source may be used.

Diagnosis

We will have to distinguish between EHS and other EMF-related health problems like certain cancers, Alzheimer's, ALS, male infertility, etc. that might have been induced, promoted, or aggravated by EMF exposure. An investigation of EHS and other EMF-related health problems will largely be based on a comprehensive case history, focusing, in particular, on correlations between health problems and times, places, and circumstances of EMF exposure, as well as the progression of symptoms over time and the individual susceptibility. In addition, measurements of EMF exposure and the results of additional diagnostic tests (laboratory tests, cardiovascular system) serve to support the diagnosis. Moreover, all other potential causes should be excluded as far as possible.

In 2000 the Nordic Council of Ministers (Finland, Sweden, and Norway) adopted the following unspecific ICD-10 code for EHS: Chapter XVIII, Symptoms, signs and abnormal clinical and laboratory findings, not elsewhere classified, code R68.8 "Other specified general symptoms and signs" (Nordic ICD-10 Adaptation, 2000) (272).

Regarding the current International Classification of Diseases (ICD), ICD-10-WHO 2015, we recommend at the moment:

(a) Electromagnetic hypersensitivity (EHS): to use the existing diagnostic codes for the different symptoms **plus** code R68.8 "Other specified general symptoms and signs" **plus** code Z58.4 "Exposure to radiation" and/or Z57.1 "Occupational exposure to radiation."
(b) EMF-related health problems (except EHS): to use the existing diagnostic codes for the different diseases/symptoms **plus code** Z58.4 "Exposure to radiation" and/or Z57.1 "Occupational exposure to radiation."

Regarding the next ICD update to be published in 2018 (ICD-11 WHO), we recommend:

(a) To create ICD codes for all environmentally induced chronic multisystem illnesses (CMI) like multiple chemical sensitivity (MCS), chronic fatigue syndrome (CFS), fibromyalgia (FM), and electromagnetic hypersensitivity (EHS) on the basis of their clinical and pathological description (187, 192).
(b) To expand chapter XIX, Injury, Poisoning and Certain Other Consequences of External Causes (T66-T78), to include/distinguish effects of EMF (static magnetic field, static electric field, ELF magnetic field, ELF electric field, VLF magnetic field, VLF electric field, radio-frequency radiation), infrared radiation, visible light, UV radiation and ionizing radiation.
(c) To expand chapter XXI, Factors Influencing Health Status and Contact with Health Services (Z00-Z99), to include/distinguish factors as EMF (static magnetic field, static electric field, ELF magnetic field, ELF electric field, VLF magnetic field, VLF electric field, radio-frequency radiation), infrared radiation, visible light, UV radiation, and ionizing radiation.

Treatment of the patient including the environment

The primary method of treatment should mainly focus on the prevention or reduction of EMF exposure that is reducing or eliminating all sources of EMF at home and in the workplace. The reduction of EMF exposure should also be extended to schools, hospitals, public transport, public places like libraries, etc. in order to enable EHS persons an unhindered use (accessibility measure). Many examples have shown that such measures can prove effective.

- 10.1515/reveh-2016-0011
Downloaded from PubFactory at 07/26/2016 08:26:15AM
via Slovak Academy of Science

With respect to total body load of other environmental influences, they must also be regarded.

Beside EMF reduction, other measures can and must be considered. These include a balanced homeostasis in order to increase the "resistance" to EMF. There is increasing evidence that a main effect of EMF on humans is the reduction of their oxidative and nitrosative regulation capacity. This hypothesis also explains observations of changing EMF sensitivity and the large number of symptoms reported in the context of EMF exposure. Based on currently available knowledge it appears useful to recommend a treatment approach, as those gaining ground for multisystem illnesses, that aims at minimizing adverse peroxynitrite effects. Measures that enhance the immune system and reduce stress in combination with detoxification will promote EHS recovery.

It should be stressed, that psychotherapy has the same significance as in other diseases. Products that are offered in the form of plaques and the like to "neutralize" or "harmonize" electrosmog should be evaluated with great restraint. Psychological stress generated by a lack of understanding or support by family, friends and physicians can exacerbate the symptoms of EHS as can stressing about exposure. For rapid recovery, the treatments need to apply to the body, mind and spirit of the individual.

In summary, the following treatment and accessibility measures appear advantageous, depending on the individual case:

Reduction of EMF exposure

This should include all types of EMF exposures relevant to the person, especially during sleep and at work – see Chapter "Reduction of EMF Exposure". For more information, see e.g. "Options to Minimize EMF/RF/Static Field Exposures in Office Environment" (268) and "Elektrosmog im Alltag" (269).

Environmental medicine treatments

Until now, no specific treatment of EHS has been established. The following paragraphs are recommendations based on the combined experience of the team. They can be considered either as an attempt to restore the full regulative capacity of the patients, as general advice for healthy living (that could and should be adapted to the cultural and individual situation of the patient), or as a more targeted approach to address the specific problems of EHS individuals according to the experience of the team.

Controlled clinical trials would be necessary to assess optimal treatment and accessibility measures. Actual data indicate that the functional deficits, which can be found in patients with EHS, correspond to those we can find in CMI such as MCS, CFS, and FM. The target of the therapy is the regulation of the physiological dysfunction detected by diagnostic steps (see chapter 2 "Examination and Findings"). The main therapeutic target includes both general and adjuvant procedures and specific treatments. The latter are challenging and need special knowledge and experience in clinical environmental medicine treatments. Main therapeutic targets include:

– **Control of total body burden**
  Besides the reduction of EMF exposure, the reduction of the total body burden by various environmental pollutants (home, workplace, school, hobby), food additives, and dental materials is indicated.
– **Reduction of oxidative and/or nitrosative stress**
  Reactive oxygen species (ROS) and reactive nitrogen species (RNS) are free radicals naturally produced in cells. Scavengers guarantee the balance between the production of free radicals and the rate of their removal. Many biologically important compounds with antioxidant (AO) function have been identified as endogenous and exogenous scavengers. Among the endogenous AO, we distinguish between enzymatic AO (catalase, glutathione peroxidase, glutathione reductase, superoxide dismutase) and non-enzymatic AO [bilirubin, ferritin, melatonin, glutathione, metallothionin, N-acetyl cysteine (NAC), NADH, NADPH, thioredoxin, 1,4,-bezoquinine, ubiquinone, uric acid]. They interact with exogenous dietary and/or synthetic AO (carotenoids, retinoids, flavonoids, polyphenols, glutathione, ascorbic acid, tocopherols). The complex regulation and use of these substances is the therapeutic challenge (232, 273).
– **Regulation of intestinal dysfunction**
  Endogenous and exogenous scavengers act synergistically to maintain the redox homeostasis. Therefore, dietary or natural antioxidants play an important role to stabilize this interaction.
  Treatment of a leaky gut, food intolerance, and food allergy is a prerequisite for maintaining redox homeostasis (274) and also requires special knowledge and experience.
– **Optimizing nutrition**
  Bioactive food is the main source of antioxidant components such as vitamin C, vitamin E, NAC, carotenoids, CoQ10, alpha-lipoic acid, lycopene, selenium, and flavonoids (275, 276). For instance, the regeneration of vitamin E by glutathione or vitamin C is needed to prevent lipid peroxidation. The dietary antioxidants only can have beneficial effects on the redox system if they are present in sufficient concentration

- 10.1515/reveh-2016-0011
Downloaded from PubFactory at 07/26/2016 08:26:15AM
via Slovak Academy of Science

levels (273). Alpha-lipoic acid acts directly and indirectly as a scavenger of free radicals including, singlet oxygen, superoxide, peroxyl radicals, and the breakdown radicals of peroxynitrite (232). It has been shown that the number of free electrons in micronutrients determines how effective they are. In organic food, the number of free electrons is higher than in conventionally produced food (277). Especially in the case of food intolerances, the tailored substitution of micronutrients in the form of supplements is necessary.

– **Control of (silent) inflammation**
  Elevated nitric oxide levels and the reaction with superoxide always leads to elevated peroxynitrate levels, which induce ROS levels as no other substance does (NO/$ONOO^-$ cycle). As a result, the nuclear factor κB (NF-κB) is activated, inducing inflammatory cytokines such as tumor necrosis factor α (TNF-α), interleukin-1β (IL-1β), interleukin-6 (IL-6), interleukin-8 (IL-8), and interferon gamma (IFN-γ) and activating various NO synthases (232). Tocopherols (278, 279), carotenoids at low concentration levels (280), vitamin C (281, 282), NAC (283), curcumin (284), resveratrol (285, 286), flavonoids (287) have shown to interrupt this inflammatory cascade at various points.
– **Normalization of mitochondrial function**
  Mitochondrial function may be disturbed in two ways. First: the high amount of free radicals may block production of adenosine triphosphate (ATP), leading to muscle pain and fatigue. Second: in the case of silent (smoldering) inflammation, the demand for more energy is elevated by 25% (236), causing a high consumption of ATP. In this case, NADH, L-carnitine, and CoQ10 are essential for ATP synthesis.
  Due to the lack of ATP, the stress regulation of catecholamines especially norepinephrine (NE) is reduced because catabolism of NE by S-adenosylmethionine is ATP dependent (288–290). Furthermore, stress regulation has a high demand for folate, vitamin B6, and methylcobalamine. Genetic polymorphisms of COMT and MTHFR influence the individual need for those substances (244, 291).
– **Detoxification**
  In humans, the accumulation of environmental toxins has an individual profile of many different inorganic and organic chemicals, which make up the total body load (292).
  Among the inorganic substances, metals and their salts play the dominant role and might be of importance to patients with EHS. Elemental mercury (Hg°) and other heavy metals such as lead (Pb) accumulate in the brain (293), especially at chronic low dose exposure. They may have toxic effects and can induce various immune reactions (294, 295). Whereas no specific active substance generally exists for the detoxification of chemicals, there are two groups of substances with more specific effects that can be used for the detoxification of metals.
  1. Substances with nonspecific physiological effects: glutathione, NAC, alpha-lipoic acid, vitamin C, and selenium.
  2. Chelating agents for detoxification of metals (296–298): the most important chelating agents are sodium thiosulfate 10%, DMPS (2,3-dimercapto-1-propanesulfonic acid), DMSA (meso-dimercaptosuccinic acid), and EDTA (2,22,23,232-ethane-1,2-diyldinitrotetraacetic acid).

  It should be noted that these substances should be used only by those designated as experts in this particular field.
– **Adjuvant therapies**
  1. Drinking water
  For detoxification reasons, a higher intake of high-quality drinking water with low mineral content and no CO_2 is needed. The intake quantity should range from 2.5 to 3.0 L (10–12 8-oz glasses) daily.

  2. Light
  Most of the people in central and northern Europe are depleted of vitamin D. Sufficient natural daylight exposure during the vitamin D-producing months (spring to fall) is one important factor. At the same time, prevention of actinic damage to the skin is necessary. In addition to natural sunlight, light therapy and low level lasers can promote healing, reduce inflammation, promote circulation, and enhance cellular ATP production.

  3. Sauna
  Sauna and therapeutic hyperthermia is an adjuvant therapy for the detoxification of almost all xenobiotics. These therapies have to be carefully used. An interaction with detoxifying drugs takes place. Sauna helps to regenerate tetrahydrobiopterin from dihydrobiopterin, which is essential for the metabolism of catecholamines and serotonin (299). However, not all saunas are alike. Traditional saunas or infrared saunas with low electric and low magnetic fields that do not use toxic glues and chemically treated wood are recommended.

- 10.1515/reveh-2016-0011
Downloaded from PubFactory at 07/26/2016 08:26:15AM
via Slovak Academy of Science

4. Oxygen
A part of patients with EHS suffer from mitochondrial dysfunction. Sufficient natural oxygen is helpful. As both hypoxia and hyperbaric oxygen can produce oxidative stress, hyperbaric oxygen therapy should only be performed if the patients are treated with sufficient antioxidants at the same time.

5. Exercise
The optimal amount of exercise is still being debated. A person's physical capacity should be assessed by ergometry in order to prescribe an individual exercise regime. Environmental medicine experience indicates that for sick people only low-impact aerobic exercise should be used. In general, start with a workload of 20–30 watts that often can be finished at 60–70 watts. Exercise on an ergometer allows better control of the consumption of energy compared to walking or running. No fatigue should result from exercising, at least after half an hour.

6. Sleep
Sleep problems are very common in patients with EHS. Sleep disturbance is associated with a reduced melatonin level. In the case of chronic inflammation, the activation of IDO (indolamine-2,3-dioxygenase) reduces the production of serotonin and, in turn, it also reduces melatonin levels. EMF exposure might block the parasympathetic activity while sympathetic activity persists. Concerning sleep disturbances, any therapy has to follow the pathogenic causes. Optimal sleep is necessary to save energy and to regulate the functions of the immune and neuroendocrine systems.

7. Protection from blue light
Wavelengths of visible light below 500 nm are called "blue light". Low doses of blue light can increase feelings of well-being, but larger amounts can be harmful to the eyes. In natural daylight, the harmful effects of "blue light" are balanced out by the regenerative effect of the red and infrared content. The escalating use of electronic light sources – such as fluorescent tubes and compact fluorescent lamps (CFL), computer screens, laptops, tablets, smartphones, and certain LED bulbs – has increased our exposure to "blue light", which at this level is suspected of playing a role in the development of age-related macular degeneration and circadian misalignment via melatonin suppression, which is associated with an increased risk of sleep disturbance, obesity, diabetes mellitus, depression, ischemic heart disease, stroke, and cancer. Extended exposure to artificial "blue light" in the evening should therefore be limited. Antioxidants, especially melatonin (300, 301), and blue light screen filters (302–304) could be helpful.

8. Exposure to the natural electromagnetic fields of the Earth.
Most people in urban centers are disconnected from the Earth's natural grounding/magnetic fields by walking with rubber-soled shoes, wearing synthetic clothing, driving in metal boxes with rubber wheels, and living and working in concrete buildings that are permeated with artificial electromagnetic fields and radiation. Spending time in the woods, walking barefoot along a beach, lying on the grass, sitting on rocks, or strolling outside after a rain shower help ground a person and help balance the often enhanced positively charged ions that are associated with ill health.

Dental medicine
Dental medicine still works with toxic or immunoreactive materials, e.g. mercury, lead oxide, gold, and titanium. Environmental dental medicine demands that these materials not be used (305–308). The removal of toxic dental materials must take place under maximum safety conditions (avoid inhalation!). The elimination of particularly heavy metals from the body might be indicated. In general terms, endoprosthetic materials should be inert with respect to immunoreactivity. Based on our current knowledge, zirconium dioxide seems to be a neutral material. However, mechanical abrasion of the coated surface by the dentist should be avoided.

Immunotoxic metals show a similar pathophysiology with respect to oxidative stress, mitochondriopathy, and inflammation.

Lifestyle coaching
Lifestyle coaching may include balanced exercise, nutrition, reduction of addictive substances, change of sleep habits, etc. and stress reduction measures (reduction of general stress and work stress), as well as methods to increase stress resistance via, e.g. autogenic training, yoga, progressive muscle relaxation, breathing techniques, meditation, tai chi, and qigong.

Treatment of symptoms
A well-balanced treatment of symptoms is justified until the causes have been identified and eliminated. However,

- 10.1515/reveh-2016-0011
Downloaded from PubFactory at 07/26/2016 08:26:15AM
via Slovak Academy of Science

it is of paramount importance to realize that the reduction of symptoms may put the person at risk for an increased environmental EMF load, thus generating possible future, long-term health effects, including neurological damage and cancer. The treating physician faces a very difficult ethical task when doing so, and the associated risks must be pointed out – in an equally well-balanced way – to the patient in question. From an ethical perspective, treating the symptoms is, of course, a very good start to provide immediate relief, but – without a concurrent environmental exposure reduction and lifestyle coaching – it may prove counter-productive in the long run. For a conventionally trained physician, this might seem a very new way of reasoning, but it is the only way to successfully and effectively alleviate symptoms and to achieve complete clinical recovery when dealing with chronic multisystem illnesses (CMI) and EHS. Though even if the causes are not known at the outset, it is already important at this stage to provide advice on how to reduce a person's exposure to electromagnetic fields and other environmental stressors to prevent further damage and promote healing.

References

1. Hanninen O, Knol AB, Jantunen M, Lim TA, Conrad A, et al. Environmental burden of disease in Europe: assessing nine risk factors in six countries. Environ Health Perspect 2014;122(5):439–46.
2. Bundespsychotherapeutenkammer. BPtK-Studie zur Arbeitsunfähigkeit – Psychische Erkrankungen und Burnout [Internet]. Berlin (DE): Bundespsychotherapeutenkammer, 2012:29. Report 2012. Available at: http://www.bptk.de/uploads/media/20120606_AU-Studie-2012.pdf.
3. Bundespsychotherapeutenkammer. BPtK-Studie zur Arbeits- und Erwerbsunfähigkeit – Psychische Erkrankungen und gesundheitsbedingte Frühverrentung [Internet]. Berlin (DE): Bundespsychotherapeutenkammer, 2013:66. Report 2013. Available at: http://www.bptk.de/uploads/media/20140128_BPtK-Studie_zur_Arbeits-und_Erwerbsunfaehigkeit_2013_1.pdf.
4. Fritze J. Psychopharmaka-Verordnungen: Ergebnisse und Kommentare zum Arzneiverordnungsreport 2011. Psychopharmakotherapie 2011;18:245–56.
5. Bundesinstitut für Arzneimittel und Medizinprodukte. Erstmals seit 20 Jahren kein Anstieg beim Methylphenidat-Verbrauch [Internet]. Bonn (DE): Bundesinstitut für Arzneimittel und Medizinprodukte, 2014 Apr 1. Pressemitteilung Nummer 05/14; Available at: https://www.bfarm.de/SharedDocs/Pressemitteilungen/DE/mitteil2014/pm05-2014.html.
6. Badura B, Ducki A, Schroder H, Klose J, Meyer M, editors. Fehlzeiten-Report 2012. Berlin, Heidelberg (DE): Springer Verlag, 2012:528pp.
7. OECD. Health at a Glance 2013: OECD Indicators [Internet]. Paris (FR): OECD Publishing, 2013:212 p. DOI: 10.1787/health_glance-2013-en. Available at: http://dx.DOI.org/10.1787/health_glance-2013-en.
8. Pawankar R, Canonica GW, Holgate ST, Lockey RF, editors. WAO White book on Allergy 2011–2012 [Internet]. Milwaukee, WI (US): World Allergy Organization, 2013:228. Available at: http://www.worldallergy.org/UserFiles/file/WAO-White-Book-on-Allergy.pdf.
9. BioInitiative Working Group, Sage C, Carpenter DO, editors. BioInitiative Report: A Rationale for a Biologically-based Public Exposure Standard for Electromagnetic Fields (ELF and RF) at www.bioinitiative.org, August 31, 2007.
10. BioInitiative Working Group, Sage C, Carpenter DO, editors. BioInitiative Report: A Rationale for a Biologically-based Public Exposure Standard for Electromagnetic Radiation at www.bioinitiative.org, December 31, 2012.
11. Levitt B, Lai H. Biological effects from exposure to electromagnetic radiation emitted by cell tower base stations and other antenna arrays. Environ Rev 2010;18:369–95.
12. Pall ML. Scientific evidence contradicts findings and assumptions of Canadian safety panel 6: microwaves act through voltage-gated calcium channel activation to induce biological impacts at non-thermal levels, supporting a paradigm shift for microwave/lower frequency electromagnetic field action. Rev Environ Health 2015;30(2):99–116.
13. Binhi VN. Magnetobiology: Underlying Physical Problems. San Diego: Academic Press, 2002:1–473.
14. Binhi VN. Principles of electromagnetic biophysics (in Russian). Moscow (RU): Fizmatlit, 2011:1–571.
15. Georgiou CD. Oxidative stress-induced biological damage by low-level EMFs: mechanism of free radical pair electron spin-polarization and biochemical amplification. In: Giuliani L, Soffritti M, editors. Non-thermal effects and mechanisms of interaction between electromagnetic fields and living matter. Bologna (IT): Ramazzini institute, 2010. European Journal of Oncology – Library Vol. 5. pp 63–113. Available at: http://www.icems.eu/papers.htm?f=/c/a/2009/12/15/MNHJ1B49KH.DTL.
16. Pall ML. Electromagnetic fields act via activation of voltage-gated calcium channels to produce beneficial or adverse effects. J Cell Mol Med 2013;17(8):958–65.
17. Blank M, Goodman R. Electromagnetic fields stress living cells. Pathophysiology 2009;16(2–3):71–8.
18. Blackman C. Cell phone radiation: evidence from ELF and RF studies supporting more inclusive risk identification and assessment. Pathophysiology 2009;16(2–3):205–16.
19. Hedendahl L, Carlberg M, Hardell L. Electromagnetic hypersensitivity – an increasing challenge to the medical profession. Rev Environ Health 2015;30(4):209–15.
20. International Commission on Non-Ionizing Radiation Protection. Guidelines for limiting exposure to time-varying electric, magnetic, and electromagnetic fields (up to 300 GHz). Health Physics 1998;74(4):494–522.
21. International Commission on Non-Ionizing Radiation Protection. Guidelines for limiting exposure to time-varying electric and magnetic fields (1 Hz to 100 kHz). Health Phys 2010;99(6):818–36.
22. Belyaev I. Biophysical mechanisms for nonthermal microwave effects. In: Markov M, editor. Electromagnetic fields in biology and medicine. Boca Raton, London, New York: CRC Press 2015:49–68.
23. Belyaev I. Electromagnetic field effects on cells and cancer risks from mobile communication. In: Rosch PJ, editor. Bioelectromagnetic and subtle energy medicine, 2nd ed. Boca Raton, London, New York: CRC Press, 2015:517–39.

- 10.1515/reveh-2016-0011
Downloaded from PubFactory at 07/26/2016 08:26:15AM
via Slovak Academy of Science

24. IARC Working Group on the Evaluation of Carcinogenic Risks to Humans. Non-Ionizing Radiation, Part 2: Radiofrequency Electromagnetic Fields. Lyon (FR): International Agency for Research on Cancer (IARC), 2013:480. IARC Monographs on the Evaluation of Carcinogenic Risks to Humans, Vol 102. Available at: http://monographs.iarc.fr/ENG/Monographs/vol102/.
25. Vecchia P. ICNIRP and international standards. London (GB): Conference EMF and Health, 2008:28. Available at: http://archive.radiationresearch.org/conference/downloads/021145_vecchia.pdf.
26. Panagopoulos DJ, Johansson O, Carlo GL. Evaluation of specific absorption rate as a dosimetric quantity for electromagnetic fields bioeffects. PLoS One 2013;8(6):e62663.
27. Belyaev I. Dependence of non-thermal biological effects of microwaves on physical and biological variables: implications for reproducibility and safety standards [Internet]. In: Giuliani L, Soffritti M, editors. Non-thermal effects and mechanisms of interaction between electromagnetic fields and living matter. Bologna (IT): Ramazzini institute, 2010. European Journal of Oncology – Library Vol. 5. pp 187–218. Available at: http://www.icems.eu/papers.htm?f=/c/a/2009/12/15/MNHJ1B49KH.DTL.
28. Grigoriev YG, Stepanov VS, Nikitina VN, Rubtcova NB, Shafirkin AV, et al. ISTC Report. Biological effects of radiofrequency electromagnetic fields and the radiation guidelines. Results of experiments performed in Russia/Soviet Union. Moscow: Institute of Biophysics, Ministry of Health, Russian Federation, 2003.
29. SanPiN 2.2.4/2.1.8. Radiofrequency electromagnetic radiation (RF EMR) under occupational and living conditions. Moscow: Minzdrav. [2.2.4/2.1.8.055-96] 1996.
30. IARC Working Group on the Evaluation of Carcinogenic Risks to Humans. Non-Ionizing Radiation, Part 1: Static and Extremely Low-Frequency (ELF) Electric and Magnetic Fields. Lyon (FR): International Agency for Research on Cancer (IARC), 2002:445. IARC Monographs on the Evaluation of Carcinogenic Risks to Humans, VOL 80. Available at: http://monographs.iarc.fr/ENG/Monographs/vol80/.
31. Oberfeld G. Precaution in Action – Global Public Health Advice Following BioInitiative 2007. In Sage C, Carpenter DO, editors. BioInitiative Report 2012: A Rationale for a Biologically based Public Exposure Standard for Electromagnetic Fields (ELF and RF), 2012. Available at: http://www.bioinitiative.org.
32. International Commission for electromagnetic safety (ICEMS), Resolutions. Available at: http://www.icems.eu/resolution.htm.
33. Radiofrequency electromagnetic radiation and the health of Canadians. Report of the Standing Committee on Health, JUNE 2015, Parliament of Canada, Ottawa, Ontario. Available at: http://www.parl.gc.ca/content/hoc/Committee/412/HESA/Reports/RP8041315/hesarp13/hesarp13-e.pdf.
34. Havas M. International expert's Perspective on the Health Effects of Electromagnetic Fields (EMF) and Electromagnetic Radiation (EMR) [Internet]. Peterborough, ON, (CD): 2011 June 11 (updated 2014 July). Available at: http://www.magdahavas.com/international-experts-perspective-on-the-health-effects-of-electromagnetic-fields-emf-and-electromagnetic-radiation-emr/.
35. European Environmental Agency. Radiation risk from everyday devices assessed [Internet]. Copenhagen (DK): 2007 Sept 17. Available at: http://www.eea.europa.eu/highlights/radiation-risk-from-everyday-devices-assessed.
36. European Environmental Agency. Health risks from mobile phone radiation – why the experts disagree [Internet]. Copenhagen (DK): 2011 Oct 12. Available at: http://www.eea.europa.eu/highlights/health-risks-from-mobile-phone.
37. European Environmental Agency. Late lessons from early warnings: science, precaution, innovation [Internet]. Copenhagen (DK): 2013 Jan 23. EEA Report No 1/2013. Available at: http://www.eea.europa.eu/publications/late-lessons-2.
38. EU Parliament. Report on health concerns associated with electromagnetic fields. Brussels (BE): Committee on the Environment, Public Health and Food Safety of the European Parliament. Rapporteur: Frederique Ries (2008/2211(INI) [Internet]. Available at: http://www.europarl.europa.eu/sides/getDoc.do?pubRef=-//EP//NONSGML+REPORT+A6-2009-0089+0+DOC+PDF+V0//EN.
39. EU Parliament. European Parliament resolution of 2 April 2009 on health concerns associated with electromagnetic fields [Internet]). Brussels (BE): European Parliament, 2009 Apr 2. Available at: http://www.europarl.europa.eu/sides/getDoc.do?pubRef=-//EP//TEXT+TA+P6-TA-2009-0216+0+DOC+XML+V0//EN.
40. Fragopoulou A, Grigoriev Y, Johansson O, Margaritis LH, Morgan L, et al. Scientific panel on electromagnetic field health risks: consensus points, recommendations, and rationales. Environ Health 2010;25(4):307–17.
41. Gesichtspunkte zur aktuellen gesundheitlichen Bewertung des Mobilfunks. Empfehlung des Obersten Sanitätsrates. Ausgabe 05/14; Bundesministerium für Gesundheit. Vienna (AT). Available at: http://www.bmg.gv.at/cms/home/attachments/1/9/2/CH1238/CMS1202111739767/mobilfunk_osr_empfehlungen.pdf.
42. Council of Europe – Parliamentary Assembly. The potential dangers of electromagnetic fields and their effect on the environment. Resolution, Doc. 1815, Text adopted by the Standing Committee, acting on behalf of the Assembly, on 27 May 2011 [Internet]. Available at: http://assembly.coe.int/nw/xml/XRef/Xref-XML2HTML-en.asp?fileid=17994&lang=en.
43. Dean AL, Rea WJ. American Academy of Environmental Medicine Recommendations Regarding Electromagnetic and Radiofrequency Exposure [Internet]. Wichita, KS (US): Executive Committee of the American Academy of Environmental Medicine, 2012 July 12. Available at: https://www.aaemonline.org/pdf/AAEMEMFmedicalconditions.pdf.
44. Federal Public Service (FPS) Health, Food Chain Safety and Environment. Mobile phones and children-New regulation for the sale of mobile phones as of 2014 [Internet]. Brussels (BE): Federal Public Service (FPS) Health, Food Chain Safety and Environment, 2016 Jan 12. Available at: http://www.health.belgium.be/en/mobile-phones-and-children.
45. Assemblée Nationale. PROPOSITION DE LOI relative a la sobriete, a la transparence, a l'information et a la concertation en matiere d'exposition aux ondes electromagnetiques. Paris (FR): Assemblée Nationale, France, 2015 Jan 29. Available at: http://www.assemblee-nationale.fr/14/pdf/ta/ta0468.pdf.
46. Blank M, Havas M, Kelley E, Lai H, Moskowitz JM. International EMF Scientist Appeal [Internet]. 2015 May 11. Available at: https://www.emfscientist.org/index.php/emf-scientist-appeal.
47. International Scientific Declaration on Electromagnetic Hypersensitivity and Multiple Chemical Sensitivity. Following the 5th Paris Appeal Congress that took place on the 18th of May, 2015 at the Royal Academy of Medicine, Brussels, Belgium. Available at: http://appel-de-paris.com/wp-content/uploads/2015/09/Statement-EN.pdf.

- 10.1515/reveh-2016-0011
Downloaded from PubFactory at 07/26/2016 08:26:15AM
via Slovak Academy of Science

48. Wertheimer N, Leeper E. Electrical wiring configurations and childhood cancer. Am J Epidemiol 1979;109(3):273–84.
49. Robinette CD, Silverman C, Jablon S. Effects upon health of occupational exposure to microwave radiation (radar). Am J Epidemiol 1980;112:39–53.
50. Ahlbom A, Day N, Feychting M, Roman E, Skinner J, et al. A pooled analysis of magnetic fields and childhood leukaemia. Br J Cancer 2000;83(5):692–8.
51. Greenland S, Sheppard AR, Kaune WT, Poole C, Kelsh MA. A pooled analysis of magnetic fields, wire codes, and childhood leukemia. Childhood Leukemia-EMF Study Group. Epidemiology 2000;11(6):624–34.
52. Kheifets L, Ahlbom A, Crespi CM, Draper G, Hagihara J, et al. Pooled analysis of recent studies on magnetic fields and childhood leukaemia. Br J Cancer 2010;103(7):1128–35.
53. Zhao L, Liu X, Wang C, Yan K, Lin X, et al. Magnetic fields exposure and childhood leukemia risk: a meta-analysis based on 11,699 cases and 13,194 controls. Leuk Res 2014;38(3):269–74.
54. Yang Y, Jin X, Yan C, Tian Y, Tang J, et al. Case-only study of interactions between DNA repair genes and low-frequency electromagnetic fields in childhood acute leukemia. Leuk Lymphoma 2008;29(12):2344.
55. Kundi M. Evidence for childhood cancers (Leukemia). In: Sage C, Carpenter DO, editors. The BioInitiative Report 2012. A Rationale for a Biologically-based Public Exposure Standard for Electromagnetic Fields (ELF and RF), 2012, http://www.bioinitiative.org/.
56. Sage C. Summary for the public. In: Sage C, Carpenter DO, editors. The BioInitiative Report 2012. A Rationale for a Biologically-based Public Exposure Standard for Electromagnetic Fields (ELF and RF), 2012. Available at: http://www.bioinitiative.org.
57. Hardell L, Nasman A, Pahlson A, Hallquist A, Hansson Mild K. Use of cellular telephones and the risk for brain tumours: a case-control study. Int J Oncol 1999;15(1):113–6.
58. Coureau G, Bouvier G, Lebailly P, Fabbro-Peray P, Gruber A, et al. Mobile phone use and brain tumours in the CERENAT case-control study. Occup Environ Med 2014;71(7):514–22.
59. Hardell L, Carlberg M, Soderqvist F, Mild KH. Case-control study of the association between malignant brain tumours diagnosed between 2007 and 2009 and mobile and cordless phone use. Int J Oncol 2013;43(6):1833–45.
60. Hardell L, Carlberg M, Soderqvist F, Mild KH. Pooled analysis of case-control studies on acoustic neuroma diagnosed 1997–2003 and 2007–2009 and use of mobile and cordless phones. Int J Oncol 2013;43(4):1036–44.
61. Hardell L, Carlberg M. Using the Hill viewpoints from 1965 for evaluating strengths of evidence of the risk for brain tumors associated with use of mobile and cordless phones. Rev Environ Health 2013;28:97–106.
62. Carlberg M, Hardell L. Decreased survival of glioma patients with astrocytoma grade IV (glioblastoma multiforme) associated with long-term use of mobile and cordless phones. Int J Environ Res Public Health 2014;11(10):10790–805.
63. Hardell L, Carlberg M. Mobile phone and cordless phone use and the risk for glioma – Analysis of pooled case-control studies in Sweden, 1997–2003 and 2007–2009. Pathophysiology 2015;22(1):1–13.
64. West JG, Kapoor NS, Liao SY, Chen JW, Bailey L, et al. Multifocal breast cancer in young women with prolonged contact between their breasts and their cellular phones. Case Rep Med 2013;2013:354682.
65. Levis AG, Gennaro V, Garbisa S. Business bias as usual: the case of electromagnetic pollution. In: Elsner W, Frigato P, Ramazzotti P, editors. Social Costs Today. Institutional Economics and Contemporary Crises. London and New York: Routledge (Taylor & Francis Group), 2012:225–68.
66. Lai H. Genetic Effects of Non-Ionizing Electromagnetic Fields Bioinitiative 2012: A Rationale for a Biologically based Public Exposure Standard for Electromagnetic Fields (ELF and RF). Sage C and Carpenter DO. http://www.bioinitiative.org/: 1-59.
67. Huss A, Egger M, Hug K, Huwiler-Müntener K, Röösli M. Source of funding and results of studies of health effects of mobile phone use: systematic review of experimental studies. Cien Saude Colet 2008;13(3):1005–12.
68. Apollonio F, Liberti M, Paffi A, Merla C, Marracino P, et al. Feasibility for microwaves energy to affect biological systems via nonthermal mechanisms: a systematic approach. IEEE Trans Microw Theory Tech 2013;61(5):2031–45.
69. Cucurachi S, Tamis WL, Vijver MG, Peijnenburg WJ, Bolte JF, et al. A review of the ecological effects of radiofrequency electromagnetic fields (RF-EMF). Environ Int 2013;51:116–40.
70. Belyaev IY, Alipov YD, Harms-Ringdahl M. Effects of weak ELF on E-coli cells and human lymphocytes: role of genetic, physiological, and physical parameters. In: Bersani F, editor. Electricity and magnetism in biology and medicine. New York: Kluwer Academic/Plenum Publ, 1999:481–4.
71. Belyaev IY, Alipov ED. Frequency-dependent effects of ELF magnetic field on chromatin conformation in Escherichia coli cells and human lymphocytes. Biochim Biophys Acta 2001;1526(3):269–76.
72. Sarimov R, Alipov ED, Belyaev IY. Fifty hertz magnetic fields individually affect chromatin conformation in human lymphocytes: dependence on amplitude, temperature, and initial chromatin state. Bioelectromagnetics 2011;32(7):570–9.
73. Belyaev IY, Hillert L, Protopopova M, Tamm C, Malmgren LO, et al. 915 MHz microwaves and 50 Hz magnetic field affect chromatin conformation and 53BP1 foci in human lymphocytes from hypersensitive and healthy persons. Bioelectromagnetics 2005;26(3):173–84.
74. Markovà E, Hillert L, Malmgren L, Persson BR, Belyaev IY. Microwaves from GSM Mobile Telephones Affect 53BP1 and gamma-H2AX Foci in Human Lymphocytes from Hypersensitive and Healthy Persons. Environ Health Perspect 2005;113(9):1172–7.
75. Belyaev IY, Markovà E, Hillert L, Malmgren LO, Persson BR. Microwaves from UMTS/GSM mobile phones induce long-lasting inhibition of 53BP1/g-H2AX DNA repair foci in human lymphocytes. Bioelectromagnetics 2009;30(2):129–41.
76. Sarimov R, Malmgren LO, Markova E, Persson BR, Belyaev IY. Nonthermal GSM microwaves affect chromatin conformation in human lymphocytes similar to heat shock. IEEE Trans Plasma Sci 2004;32(4):1600–8.
77. Markovà E, Malmgren LOG, Belyae IY. Microwaves from mobile phones inhibit 53BP1 focus formation in human stem cells more strongly than in differentiated cells: possible mechanistic link to cancer risk. Environ Health Perspect 2010;118(3):394–9.
78. World Health Organization (WHO). Radiofrequency and microwaves. Environmental Health Criteria 16, Geneva (CH): WHO, 1981. Available at: http://www.inchem.org/documents/ehc/ehc/ehc016.htm.
79. World Health Organization (WHO). Extremely low frequency (ELF) fields. Environmental Health Criteria 35, Geneva (CH): WHO,

- 10.1515/reveh-2016-0011
Downloaded from PubFactory at 07/26/2016 08:26:15AM
via Slovak Academy of Science

1984. Available at: http://www.inchem.org/documents/ehc/ehc/ehc35.htm.
80. Haynal A, Regli F. Zusammenhang der amyotrophischen Lateralsklerose mit gehäuften Elektrotraumata [Amyotrophic lateral sclerosis associated with accumulated electric injury]. Confin Neurol 1964;24:189–98.
81. Şahin A, Aslan A, Baş O, İkinci A, Özyılmaz C, et al. Deleterious impacts of a 900-MHz electromagnetic field on hippocampal pyramidal neurons of 8-week-old Sprague Dawley male rats. Brain Res 2015;1624:232–8.
82. Schliephake E. Arbeitsergebnisse auf dem Kurzwellengebiet [Work results in the area of short waves]. Dtsch Med Wochenschr 1932;58(32):1235–41.
83. Sadchikova MN. State of the nervous system under the influence of UHF. In: Letavet AA, Gordon ZV, editors. The biological action of ultrahigh frequencies. Moscow: Academy of Medical Sciences, 1960:25–9.
84. Von Klitzing L. Low-frequency pulsed electromagnetic fields influence EEG of man. Phys Medica 1995;11:77–80.
85. Reiser H, Dimpfel W, Schober F. The influence of electromagnetic fields on human brain activity. Eur J Med Res 1995;1(1):27–32.
86. Röschke J, Mann K. No short-term effects of digital mobile radio telephone on the awake human electroencephalogram. Bioelectromagnetics 1997;18(2):172–6.
87. Hietanen M, Kovala T, Hamalainen AM. Human brain activity during exposure to radiofrequency fields emitted by cellular phones. Scand J Work Environ Health 2000;26(2):87–92.
88. Croft R, Chandler J, Burgess A, Barry R, Williams J, et al. Acute mobile phone operation affects neural function in humans. Clin Neurophysiol 2002;113(10):1623–32.
89. Kramarenko AV, Tan U. Effects of high-frequency electromagnetic fields on human EEG: a brain mapping study. Int J Neurosci 2003;113(7):1007–19.
90. Vecchio F, Babiloni C, Ferreri F, Curcio G, Fini R, et al. Mobile phone emission modulates interhemispheric functional coupling of EEG alpha rhythms. Eur J Neurosci 2007;25(6):1908–13.
91. Vecchio F, Babiloni C, Ferreri F, Buffo P, Cibelli G, et al. Mobile phone emission modulates inter-hemispheric functional coupling of EEG alpha rhythms in elderly compared to young subjects. Clin Neurophysiol 2010;121(2):163–71.
92. Vecchio F, Buffo P, Sergio S, Iacoviello D, Rossini PM, et al. Mobile phone emission modulates event-related desynchronization of α rhythms and cognitive-motor performance in healthy humans. Clin Neurophysiol 2012;123(1):121–8.
93. Perentos N, Croft RJ, McKenzie RJ, Cvetkovic D, Cosic I. The effect of GSM-like ELF radiation on the alpha band of the human resting EEG. Conf Proc IEEE Eng Med Biol Soc 2008;1:5680–3.
94. Trunk A, Stefanics G, Zentai N, Kovács-Bálint Z, Thuróczy G, et al. No effects of a single 3G UMTS mobile phone exposure on spontaneous EEG activity, ERP correlates, and automatic deviance detection. Bioelectromagnetics 2013;34(1):31–42.
95. Ghosn R, Yahia-Cherif L, Hugueville L, Ducorps A, Lemarechal JD, et al. Radiofrequency signal affects alpha band in resting electroencephalogram. J Neurophysiol 2015;113(7):2753–9.
96. Roggeveen S, van Os J, Viechtbauer W, Lousberg R. EEG changes due to experimentally induced 3G mobile phone radiation. PLoS One 2015;10(6):e0129496.
97. Freude G, Ullsperger P, Eggert S, Ruppe I. Effects of microwaves emitted by cellular phones on human slow brain potentials. Bioelectromagnetics 1998;19(6):384–7.
98. Freude G, Ullsperger P, Eggert S, Ruppe I. Microwaves emitted by cellular telephones affect human slow brain potentials. Eur J Appl Physiol 2000;81(1–2):18–27.
99. Hladky A, Musil J, Roth Z, Urban P, Blazkova V. Acute effects of using a mobile phone on CNS functions. Cent Eur J Public Health 1999;7(4):165–7.
100. Hamblin DL, Wood AW, Croft RJ, Stough C. Examining the effects of electromagnetic fields emitted by GSM mobile phones on human event-related potentials and performance during an auditory task. Clin Neurophysiol 2004;115(1):171–8.
101. Yuasa K, Arai N, Okabe S, Tarusawa Y, Nojima T, et al. Effects of thirty minutes mobile phone use on the human sensory cortex. Clin Neurophysiol 2006;117:900–5.
102. Bak M, Dudarewicz A, Zmyślony M, Sliwinska-Kowalska M. Effects of GSM signals during exposure to event related potentials (ERPs). Int J Occup Med Environ Health 2010;23(2):191–9.
103. Maganioti AE, Hountala CD, Papageorgiou CC, Kyprianou MA, Rabavilas AD, et al. Principal component analysis of the P600 waveform: RF and gender effects. Neurosci Lett 2010;478(1):19–23.
104. Trunk A, Stefanics G, Zentai N, Bacskay I, Felinger A, et al. Lack of interaction between concurrent caffeine and mobile phone exposure on visual target detection: an ERP study. Pharmacol Biochem Behav 2014;124:412–20.
105. Mann K, Röschke J. 1996. Effects of pulsed high-frequency electromagnetic fields on human sleep. Neuropsychobiology 1996;33(1):41–7.
106. Borbely AA, Huber R, Graf T, Fuchs B, Gallmann E, et al. Pulsed high-frequency electromagnetic field affects human sleep and sleep electroencephalogram. Neurosci Lett 1999;275(3):207–10.
107. Huber R, Graf T, Cote KA, Wittmann L, Gallmann E, et al. Exposure to pulsed high-frequency electromagnetic field during waking affects human sleep EEG. Neuroreport 2000;11(15):3321–5.
108. Huber R, Treyer V, Borbély AA, Schuderer J, Gottselig JM, et al. Electromagnetic fields, such as those from mobile phones, alter regional cerebral blood flow and sleep and waking EEG. J Sleep Res 2002;11:289–95.
109. Huber R, Schuderer J, Graf T, Jutz K, Borbely AA, et al. Radio frequency electromagnetic field exposure in humans: Estimation of SAR distribution in the brain, effects on sleep and heart rate. Bioelectromagnetics 2003;24(4):262–76.
110. Regel SJ, Tinguely G, Schuderer J, Adam M, Kuster N, et al. Pulsed radio-frequency electromagnetic fields: dose-dependent effects on sleep, the sleep EEG and cognitive performance. J Sleep Res 2007;16(3):253–8.
111. Fritzer G, Göder R, Friege L, Wachter J, Hansen V, et al. Effects of short- and long-term pulsed radiofrequency electromagnetic fields on night sleep and cognitive functions in healthy subjects. Bioelectromagnetics 2007;28(4):316–25.
112. Lowden A, Akerstedt T, Ingre M, Wiholm C, Hillert L, et al. Sleep after mobile phone exposure in subjects with mobile phone-related symptoms. Bioelectromagnetics 2011;32(1):4–14.
113. Loughran SP, McKenzie RJ, Jackson ML, Howard ME, Croft RJ. Individual differences in the effects of mobile phone exposure on human sleep: rethinking the problem. Bioelectromagnetics 2012;33(1):86–93.

- 10.1515/reveh-2016-0011
Downloaded from PubFactory at 07/26/2016 08:26:15AM
via Slovak Academy of Science

114. Schmid MR, Loughran SP, Regel SJ, Murbach M, Bratic Grunauer A, et al. Sleep EEG alterations: effects of different pulse-modulated radio frequency electromagnetic fields. J Sleep Res 2012;21(1):50–58.
115. Schmid MR, Murbach M, Lustenberger C, Maire M, Kuster N, et al. Sleep EEG alterations: effects of pulsed magnetic fields versus pulse-modulated radio frequency electromagnetic fields. J Sleep Res 2012;21(6):620–9.
116. Nakatani-Enomoto S, Furubayashi T, Ushiyama A, Groiss SJ, Ueshima K, et al. Effects of electromagnetic fields emitted from W-CDMA-like mobile phones on sleep in humans. Bioelectromagnetics 2013;34(8):589–8.
117. Lustenberger C, Murbach M, Durr R, Schmid MR, Kuster N, et al. Stimulation of the brain with radiofrequency electromagnetic field pulses affects sleep-dependent performance improvement. Brain Stimul 2013;6(5):805–11.
118. Lustenberger C, Murbach M, Tüshaus L, Wehrle F, Kuster N, et al. Inter-individual and intra-individual variation of the effects of pulsed RF EMF exposure on the human sleep EEG. Bioelectromagnetics 2015;36(3):169–77.
119. Danker-Hopfe H, Dorn H, Bolz T, Peter A, Hansen ML, et al. Effects of mobile phone exposure (GSM 900 and WCDMA/UMTS) on polysomnography based sleep quality: An intra- and inter-individual perspective. Environ Res 2015;145:50–60.
120. Preece AW, Iwi G, Davies-Smith A, Wesnes K, Butler S, et al. Effect of a 915-MHz simulated mobile phone signal on cognitive function in man. Int J Radiat Biol 1999;75(4):447–56.
121. Koivisto M, Revonsuo A, Krause C, Haarala C, Sillanmaki L, et al. Effects of 902 MHz electromagnetic field emitted by cellular telephones on response times in humans. Neuroreport 2000;11(2):413–5.
122. Edelstyn N, Oldershaw A. The acute effects of exposure to the electromagnetic field emitted by mobile phones on human attention. Neuroreport 2002;13(1):119–21.
123. Lee TM, Lam PK, Yee LT, Chan CC. The effect of the duration of exposure to the electromagnetic field emitted by mobile phones on human attention. Neuroreport 2003;14(10):1361–4.
124. Curcio G, Ferrara M, De Gennaro L, Cristiani R, D'Inzeo G, et al. Time-course of electromagnetic field effects on human performance and tympanic temperature. Neuroreport 2004; 15(1):161–4.
125. Schmid G, Sauter C, Stepansky R, Lobentanz IS, Zeitlhofer J. No influence on selected parameters of human visual perception of 1970 MHz UMTS-like exposure. Bioelectromagnetics 2005;26(4):243–50.
126. Cinel C, Boldini A, Russo R, Fox E. Effects of mobile phone electromagnetic fields on an auditory order threshold task. Bioelectromagnetics 2007;28(6):493–6.
127. Luria R, Eliyahu I, Hareuveny R, Margaliot M, Meiran N. Cognitive effects of radiation emitted by cellular phones: the influence of exposure side and time. Bioelectromagnetics 2009;30(3):198–204.
128. Leung S, Croft RJ, McKenzie RJ, Iskra S, Silber B, et al. Effects of 2G and 3G mobile phones on performance and electrophysiology in adolescents, young adults and older adults. Clin Neurophysiol 2011;122(11):2203–16.
129. Mortazavi SM, Rouintan MS, Taeb S, Dehghan N, Ghaffarpanah AA, et al. Human short-term exposure to electromagnetic fields emitted by mobile phones decreases computer-assisted visual reaction time. Acta Neurol Belg 2012;112(2):171–5.
130. Wallace D, Eltiti S, Ridgewell A, Garner K, Russo R, et al. Cognitive and physiological responses in humans exposed to a TETRA base station signal in relation to perceived electromagnetic hypersensitivity. Bioelectromagnetics 2012;33(1):23–39.
131. Sauter C, Eggert T, Dorn H, Schmid G, Bolz T, et al. Do signals of a hand-held TETRA transmitter affect cognitive performance, well-being, mood or somatic complaints in healthy young men? Results of a randomized double-blind cross-over provocation study. Environ Res 2015;140:85–94.
132. Volkow ND, Tomasi D, Wang GJ, Vaska P, Fowler JS, et al. Effects of cell phone radiofrequency signal exposure on brain glucose metabolism. JAMA 2011;305(8):808–13.
133. Kwon MS, Vorobyev V, Kännälä S, Laine M, Rinne JO, et al. GSM mobile phone radiation suppresses brain glucose metabolism. J Cereb Blood Flow Metab 2011;31(12):2293–301.
134. Huber R, Treyer V, Schuderer J, Berthold T, Buck A, et al. Exposure to pulse-modulated radio frequency electromagnetic fields affects regional cerebral blood flow. Eur J Neurosci 2005;21(4):1000–6.
135. Aalto S, Haarala C, Brück A, Sipilä H, Hämäläinen H, et al. Mobile phone affects cerebral blood flow in humans. J Cereb Blood Flow Metab 2006;26(7):885–90.
136. Sienkiewicz ZJ, Blackwell RP, Haylock RG, Saunders RD, Cobb BL. Low-level exposure to pulsed 900 MHz microwave radiation does not cause deficits in the performance of a spatial learning task in mice. Bioelectromagnetics 2000;21(3):151–8.
137. Fragopoulou AF, Miltiadous P, Stamatakis A, Stylianopoulou F, Koussoulakos SL, et al. Whole body exposure with GSM 900 MHz affects spatial memory in mice. Pathophysiology 2010;17(3):179–87.
138. Aldad TS, Gan G, Gao XB, Taylor HS. Fetal radiofrequency radiation exposure from 800–1900 MHz-rated cellular telephones affects neurodevelopment and behavior in mice. Sci Re 2012;2:312.
139. Sharma A, Sisodia R, Bhatnagar D, Saxena VK. Spatial memory and learning performance and its relationship to protein synthesis of Swiss albino mice exposed to 10 GHz microwaves. Int J Radiat Biol 2013;90(1):29–35.
140. Shirai T, Imai N, Wang J, Takahashi S, Kawabe M, et al. Multigenerational effects of whole body exposure to 2.14-GHz W-CDMA cellular phone signals on brain function in rats. Bioelectromagnetics 2014;35(7):497–511.
141. Hu S, Peng R, Wang C, Wang S, Gao Y, et al. Neuroprotective effects of dietary supplement Kang-fu-ling against high-power microwave through antioxidant action. Food Funct 2014;5(9):2243–51.
142. Sokolovic D, Djordjevic B, Kocic G, Babovic P, Ristic G, et al. The effect of melatonin on body mass and behaviour of rats during an exposure to microwave radiation from mobile phone. Bratisl Lek Listy 2012;113(5):265–9.
143. Lai H. Neurological effects of non-ionizing electromagnetic fields. In: Sage C, Carpenter DO, editors. The bioinitiative report 2012, a rationale for a biologically-based public exposure standard for electromagnetic fields (ELF and RF), 2012. Available at: http://www.bioinitiative.org.
144. Adey WR. Evidence for cooperative mechanisms in the susceptibility of cerebral tissue to environmental and intrinsic electric fields. In: Schmitt FO, Schneider DN, Crothers DM, editors. Functional linkage in biomolecular systems. New York: Raven Press, 1975:325–42.

- 10.1515/reveh-2016-0011
Downloaded from PubFactory at 07/26/2016 08:26:15AM
via Slovak Academy of Science

145. Bawin SM, Sheppard AR, Adey WR. Possible mechanisms of weak electromagnetic field coupling in brain tissue. Bioelectrochem Bioenerg 1978;5:67–76.
146. Blackman CF, Benane SG, Kinney LS, Joines WT, House DE. Effects of ELF fields on calcium ion efflux from brain tissue in vitro. Radiat Res 1982;92:510–20.
147. Adey WR. Tissue interactions with nonionizing electromagnetic fields. Physiol Rev 1981;61(2):435–514.
148. Shin EJ, Jeong JH, Kim HJ, Jang CG, Yamada K, et al. Exposure to extremely low frequency magnetic fields enhances locomotor activity via activation of dopamine D1-like receptors in mice. J Pharmacol Sci 2007;105(4):367–71.
149. Shin EJ, Nguyen XK, Nguyen TT, Pham DT, Kim HC. Exposure to extremely low frequency magnetic fields induces fos-related antigen-immunoreactivity via activation of dopaminergic D1 receptor. Exp Neurobiol 2011;20(3):130–6.
150. Wang LF, Li X, Gao YB, Wang SM, Zhao L, et al. Activation of VEGF/Flk-1-ERK pathway induced blood-brain barrier injury after microwave exposure. Mol Neurobiol 2015;52(1): 478–91.
151. Ravera S, Bianco B, Cugnoli C, Panfoli I, Calzia D, et al. Sinusoidal ELF magnetic fields affect acetylcholinesterase activity in cerebellum synaptosomal membranes. Bioelectromagnetics 2010;31(4):270–6.
152. Fournier NM, Mach QH, Whissell PD, Persinger MA. Neurodevelopmental anomalies of the hippocampus in rats exposed to weak intensity complex magnetic fields throughout gestation. Int J Dev Neurosci 2012;30(6):427–33.
153. Gavalas RJ, Walter DO, Hamner J, Adey WR. Effect of low-level, low-frequency electric fields on EEG and behavior in Macaca nemestrina. Brain Res 1970;18:491–501.
154. Anderson LE, Phillips ED. Biological effects of electric fields: an overview. In: Gandolfo M, Michaelson S, Rindi A, editors. Biological effects and dosimetry of static and ELF electromagnetic fields. New York: Plenum Press, 1984.
155. Balassa T, Szemerszky R, Bárdos G. Effect of short-term 50 Hz electromagnetic field exposure on the behavior of rats. Acta Physiol Hung 2009;96(4):437–48.
156. Dimitrijević D, Savić T, Anđelković M, Prolić Z, Janać B. Extremely low frequency magnetic field (50 Hz, 0.5 mT) modifies fitness components and locomotor activity of Drosophila subobscura. Int J Radiat Biol 2014;90(5):337–43.
157. He LH, Shi HM, Liu TT, Xu YC, Ye KP, et al. Effects of extremely low frequency magnetic field on anxiety level and spatial memory of adult rats. Chin Med J (Engl) 2011;124(20):3362–6.
158. Korpinar MA, Kalkan MT, Tuncel H. The 50 Hz (10 mT) sinusoidal magnetic field: effects on stress-related behavior of rats. Bratisl Lek Listy 2012;113(9):521–4.
159. Salunke BP, Umathe SN, Chavan JG. Involvement of NMDA receptor in low-frequency magnetic field-induced anxiety in mice. Electromagn Biol Med 2014;33(4):312–26.
160. Szemerszky R, Zelena D, Barna I, Bárdos G. Stress-related endocrinological and psychopathological effects of short- and long-term 50Hz electromagnetic field exposure in rats. Brain Res Bull 2010;81(1):92–9.
161. Kitaoka K, Kitamura M, Aoi S, Shimizu N, Yoshizaki K. Chronic exposure to an extremely low-frequency magnetic field induces depression-like behavior and corticosterone secretion without enhancement of the hypothalamic-pituitary-adrenal axis in mice. Bioelectromagnetics 2013;34(1):43–51.
162. Stevens P. Affective response to 5 microT ELF magnetic field-induced physiological changes. Bioelectromagnetics 2007;28(2):109–14.
163. Ross ML, Koren SA, Persinger MA. Physiologically patterned weak magnetic fields applied over left frontal lobe increase acceptance of false statements as true. Electromagn Biol Med 2008;27(4):365–71.
164. Nishimura T, Tada H, Guo X, Murayama T, Teramukai S, et al. A 1-μT extremely low-frequency electromagnetic field vs. sham control for mild-to-moderate hypertension: a double-blind, randomized study. Hypertens Res 2011;34(3):372–7.
165. Huss A, Koeman T, Kromhout H, Vermeulen R. Extremely low frequency magnetic field exposure and parkinson's disease–a systematic review and meta-analysis of the data. Int J Environ Res Public Health 2015;12(7):7348–56.
166. Zhou H, Chen G, Chen C, Yu Y, Xu Z. Association between extremely low-frequency electromagnetic fields occupations and amyotrophic lateral sclerosis: a meta-analysis. PLoS One 2012;7(11):e48354.
167. Vergara X, Kheifets L, Greenland S, Oksuzyan S, Cho YS, et al. Occupational exposure to extremely low-frequency magnetic fields and neurodegenerative disease: a meta-analysis. J Occup Environ Med 2013;55(2):135–46.
168. Kundi M, Hutter HP. Umwelthygienische Bewertung des Berichtes zur Bestimmung der Feldstarken niederfrequenter magnetischer Wechselfelder im Bereich der 110 kV Hochspannungsleitung im Siedlungsbereich der Gemeinde Kottingbrunn von Dr.-Ing. Dietrich Moldan vom 20.8.2014 [Internet]. Kottingbrunn(AT): Gemeinde Kottinbrunn, 2014:69–104. Available at: www.kottingbrunn.or.at/system/web/GetDocument.ashx?fileid=972861.
169. Stam R. Electromagnetic fields and the blood-brain barrier. Brain Res Rev 2010;65(1):80–97.
170. Nittby H, Brun A, Strömblad S, Moghadam MK, Sun W, et al. Nonthermal GSM RF and ELF EMF effects upon rat BBB permeability. Environmentalist 2011; 31(2):140–8.
171. Salford LG, Nittby H, Persson BRR. Effects of electromagnetic fields from wireless communication upon the blood-brain barrier. In: Sage C, Carpenter DO. The BioInitiative Report 2012: A Rationale for a Biologically based Public Exposure Standard for Electromagnetic Fields (ELF and RF). Available at: http://www.bioinitiative.org/: 1–52.
172. Zhou JX, Ding GR, Zhang J, Zhou YC, Zhang YJ, et al. Detrimental effect of electromagnetic pulse exposure on permeability of in vitro blood-brain-barrier model. Biomed Environ Sci 2013;26(2):128–37.
173. Tang J, Zhang Y, Yang L, Chen Q, Tan L, et al. Exposure to 900 MHz electromagnetic fields activates the mkp-1/ERK pathway and causes blood-brain barrier damage and cognitive impairment in rats. Brain Res 2015;1601:92–101.
174. Masuda H, Hirota S, Ushiyama A, Hirata A, Arima T, et al. No dynamic changes in blood-brain barrier permeability occur in developing rats during local cortex exposure to microwaves. In Vivo 2015;29(3):351–7.
175. Sage C. Summary table 1-1. In: Sage C, DO Carpenter (editors.), The BioInitiative Report 2012: a rationale for a biologically-based public exposure standard for electromagnetic fields (ELF and RF), 2012. Available at: http://www.bioinitiative.org/.
176. Agarwal A, Deepinder F, Sharma RK, Ranga G, Li J. Effect of cell phone usage on semen analysis in men attending infertility clinic: an observational study. Fertil Steril 2008;89(1):124–8.

- 10.1515/reveh-2016-0011
Downloaded from PubFactory at 07/26/2016 08:26:15AM
via Slovak Academy of Science

177. Agarwal A, Desai NR, Makker K, Varghese A, Mouradi R, et al. Effect of radiofrequency electromagnetic waves (RF-EMF) from cellular phones on human ejaculated semen: an in vitro study. Fertil Steril 2009;92(4):1318–25.
178. Wdowiak A, Wdowiak L, Wiktor H. Evaluation of the effect of using mobile phones on male fertility. Ann Agric Environ Med 2007;14(1):169–72.
179. De Iuliis GN, Newey RJ, King BV, Aitken RJ. Mobile phone radiation induces reactive oxygen species production and DNA damage in human spermatozoa in vitro. PLoS One 2009;4(7):e6446.
180. Fejes I, Zavacki Z, Szollosi J, Koloszar Daru J, Kovacs L, et al. Is there a relationship between cell phone use and semen quality? Arch Androl 2005;51(5):385–93.
181. Aitken RJ, Bennetts LE, Sawyer D, Wiklendt AM, King BV. Impact of radio frequency electromagnetic radiation on DNA integrity in the male germline. Int J Androl 2005;28(3):171–9.
182. Kumar S, Behari J, Sisodia R. Impact of Microwave at X-Band in the aetiology of male infertility. Electromagnetic Electromagn Biol Med 2012;31(3):223–32.
183. Aitken RJ, Koopman P, Lewis SEM. Seeds of concern. Nature 2004;432(7013):48–52.
184. Erogul O, Oztas E, Yildirim I, Kir T, Aydur E, et al. Effects of electromagnetic radiation from a cellular phone on human sperm motility:an in vitro study. Arch Med Res 2006;37(7):840–3.
185. Dasdag S. Whole-body microwave exposure emitted by cellular phones and testicular function of rats. Urol Res 1999;27(3):219–23.
186. Yan JG, Agresti M, Bruce T, Yan YH, Granlund A, et al. Effects of cellular phone emissions on sperm motility in rats. Fertil Steril 2007;88(4):957–64.
187. Otitoloju AA, Obe IA, Adewale OA, Otubanjo OA, Osunkalu VO. Preliminary study on the reduction of sperm head abnormalities in mice, Mus musculus, exposed to radiofrequency radiations from global system for mobile communication base stations. Bull Environ Contam Toxicol 2010;84(1):51–4.
188. Behari J, Kesari KK. Effects of microwave radiations on reproductive system of male rats. Embryo Talk 2006;1(Suppl 1):81–5.
189. Neutra RR, Hristova L, Yost M, Hiatt RA. A nested case-control study of residential and personal magnetic field measures and miscarriages. Epidemiology 2002;13(1):21–31.
190. Li DK, Odouli R, Wi S, Janevic T, Golditch I, et al. A population-based prospective cohort study of personal exposure to magnetic fields during pregnancy and the risk of miscarriage. Epidemiology 2002;13(1):9–20.
191. Roosli M, Moser M, Baldinini Y, Meier M, Braun-Fahrlander C. Symptoms of ill health ascribed to electromagnetic field exposure – a questionnaire survey. Int J Hyg Environ Health 2004;207(2):141–50.
192. Huss A, Kuchenhoff J, Bircher A, Heller P, Kuster H, et al. Symptoms attributed to the environment-a systematic interdisciplinary assessment. Int J Hyg Environ Health 2004;207(3):245–54.
193. Huss A, Kuchenhoff J, Bircher A, Niederer M, Tremp J, et al. Elektromagnetische Felder und Gesundheitsbelastungen – Interdisziplinare Fallabklärungen im Rahmen eines umweltmediznischen Beratungsprojektes. Umweltmed Forsch Prax 2005;10(1):21–8.
194. Hagstrom M, Auranen J, Ekman R. Electromagnetic hypersensitive Finns: symptoms, perceived sources and treatments, a questionnaire study. Pathophysiology 2013;20(2):117–22.
195. Schreier N, Huss A, Roosli M. The prevalence of symptoms attributed to electromagnetic field exposure: a cross-sectional representative survey in Switzerland. Soz Praventivmed 2006;51(4):202–9.
196. Huss A, Roosli M. Consultations in primary care for symptoms attributed to electromagnetic fields–a survey among general practitioners. BMC Public Health 2006;6:267.
197. Ausfeld-Hafter B, Manser R, Kempf D, Brandli I. Komplementärmedizin. Eine Fragebogenerhebung in schweizerischen Arztpraxen mit komplementärmedizinischem Diagnostik- und Therapieangebot. Studie im Auftrag vom Bundesamt für Umwelt. Universitat Bern. Kollegiale Instanz für Komplementärmedizin (KIKOM) [Internet]. Bern (CH): Bundesamt für Umwelt. 2006 Oct 5. Available at: https://www.diagnose-funk.org/publikationen/artikel/detail&newsid=720.
198. Leitgeb N, Schrottner J, Bohm M. Does "electromagnetic pollution" cause illness? An inquiry among Austrian general practitioners. Wien Med Wochenschr 2005;155(9–10):237–41.
199. Kato Y, Johansson O. Reported functional impairments of electrohypersensitive Japanese: a questionnaire survey. Pathophysiology 2012;19(2):95–100.
200. Khurana VG, Hardell L, Everaert J, Bortkiewicz A, Carlberg M, et al. Epidemiological evidence for a health risk from mobile phone base stations. Int J Occup Environ Health 2010;16(3):263–7.
201. Carpenter DO. The microwave syndrome or electro-hypersensitivity: historical background. Rev Environ Health 2015;30(4):217–22.
202. World Health Organization. Factsheet Nr. 296, Elektromagnetische Felder und Öffentliche Gesundheit – Elektromagnetische Hypersensitivität (Elektrosensibilität) [Internet]. Genf (CH): WHO, 2005 Dec. Available at: http://www.who.int/peh-emf/publications/facts/ehs_fs_296_german.pdf.
203. Tresidder A, Bevington M. Electrosensitivity: sources, symptoms, and solutions. In: Rosch PJ, editor. Bioelectromagnetic and subtle energy medicine, 2nd ed. Boca Raton, FL, (USA): CRC Press, Taylor & Francis Group Version Date: 20141107, ISBN-13: 978-1-4822-3320-9 (eBook – PDF).
204. Genuis SJ, Lipp CT. Electromagnetic hypersensitivity: fact or fiction? Sci Total Environ 2012;414:103–12.
205. Johansson O, Liu P-Y. "Electrosensitivity", "electrosupersensitivity" and "screen dermatitis": preliminary observations from on-going studies in the human skin. In: Simunic D, editor. Proceedings of the COST 244: Biomedical Effects of Electromagnetic Fields – Workshop on Electromagnetic Hypersensitivity. Brussels/Graz: EU/EC (DG XIII) 1995:52–57.
206. Johansson O, Gangi S, Liang Y, Yoshimura K, Jing C, et al. Cutaneous mast cells are altered in normal healthy volunteers sitting in front of ordinary TVs/PCs – results from open-field provocation experiments. J Cutan Pathol 2001;28(10):513–9.
207. Belpomme D, Campagnac C, Irigaray P. Reliable disease biomarkers characterizing and identifying electrohypersensitivity and multiple chemical sensitivity as two etiopathogenic aspects of a unique pathological disorder. Rev Environ Health 2015;30(4):251–71.
208. Regel SJ, Negovetic S, Roosli M, Berdinas V, Schuderer J, et al. UMTS base station-like exposure, well-being, and cognitive performance. Environ Health Perspect 2006;114(8):1270–5.

- 10.1515/reveh-2016-0011
Downloaded from PubFactory at 07/26/2016 08:26:15AM
via Slovak Academy of Science

209. Zwamborn APM, Vossen SHJA, van Leersum BJAM, Ouwens MA, Makel WN. Effects of global communication system radio-frequency fields on well being and cognitive functions of human subjects with and without subjective complaints. The Hague (NL): TNO Physics and Electronics Laboratory, 2003 Sept, 86p. TNO-report FEL-03-C148. Available at: https://www.salzburg.gv.at/gesundheit_/Documents/tno-fel_report_03148_definitief.pdf.
210. Eltiti S, Wallace D, Ridgewell A, Zougkou K, Russo R, et al. Does short-term exposure to mobile phone base station signals increase symptoms in individuals who report sensitivity to electromagnetic fields? A double-blind randomized provocation study. Environ Health Perspect 2007;115(11):1603–8.
211. McCarty DE, Carrubba S, Chesson AL, Frilot C, Gonzalez-Toledo E, et al. Electromagnetic hypersensitivity: evidence for a novel neurological syndrome. Int J Neurosci 2011;121(12):670–6.
212. Havas M, Marrongelle J, Pollner B, Kelley E, Rees CR, et al. Provocation study using heart rate variability shows microwave radiation from 2.4 GHz cordless phone affects autonomic nervous system [Internet]. In: Giuliani L, Soffritti M, editors. Non-thermal effects and mechanisms of interaction between electromagnetic fields and living matter. Bologna (IT): Ramazzini institute, 2010. European Journal of Oncology – Library Vol. 5. pp 187–218. Available at: http://www.icems.eu/papers.htm?f=/c/a/2009/12/15/MNHJ1B49KH.DTL.
213. Havas M. Radiation from wireless technology affects the blood, the heart, and the autonomic nervous system. Rev Environ Health 2013;28(2–3):75–84.
214. Tuengler A, von Klitzing L. Hypothesis on how to measure electromagnetic hypersensitivity. Electromagn Biol Med 2013;32(3):281–90.
215. Klitzing L. Einfluss elektromagnetischer Felder auf kardiovaskulare Erkrankungen. umwelt medizin gesellschaft 2014;27(1):17–21.
216. Santini R, Santini P, Danze JM, Le Ruz P, Seigne M. Investigation on the health of people living near mobile telephone relay stations: I/Incidence according to distance and sex. Pathol Biol (Paris) 2002;50(6):369–73.
217. Navarro EA, Segura J, Portoles M, Gomez-Perretta de Mateo C. The microwave syndrome: a preliminary study in Spain. Electromagn Biol Med 2003;22(2–3):161–9.
218. Hutter HP, Moshammer H, Wallner P, Kundi M. Subjective symptoms, sleeping problems, and cognitive performance in subjects living near mobile phone base stations. Occup Environ Med 2006;63(5):307–13.
219. Abdel-Rassoul G, El-Fateh OA, Salem MA, Michael A, Farahat F, et al. Neurobehavioral effects among inhabitants around mobile phone base stations. Neurotoxicology 2007;28(2):434–40.
220. Blettner M, Schlehofer B, Breckenkamp J, Kowall B, Schmiedel S, et al. Mobile phone base stations and adverse health effects: phase 1 of a population-based, cross-sectional study in Germany. Occup Environ Med 2009;66(2):118–23.
221. Molla-Djafari H, Witke J, Poinstingl G, Brezansky A, Hutter HP, et al. Leitfaden Senderbau -Vorsorgeprinzip bei Errichtung, Betrieb, Um- und Ausbau von ortsfesten Sendeanlagen. Wien (AT): Ärztinnen und Ärzte für eine gesunde Umwelt e.V. (Hrsg.), 2014 Oct. 2. Auflage, 42 p, Available at: www.aegu.net/pdf/Leitfaden.pdf.
222. Milham S, Stetzer D. Dirty electricity, chronic stress, neurotransmitters and disease. Electromagn Biol Med 2013;32(4):500–7.
223. Blackman C. Evidence for disruption by the modulating signal. In: Sage C, Carpenter DO, editors. The bioInitiative report 2007: a rationale for a biologically-based public exposure standard for electromagnetic fields (ELF and RF), 2007. Available at: http://www.bioinitiative.org/.
224. Belyaev I. Evidence for disruption by modulation: role of physical and biological variables in bioeffects of non-thermal microwaves for reproducibility, cancer risk and safety standards. In: Sage C, Carpenter DO, editors. BioInitiative report 2012: a rationale for a biologically based public exposure standard for electromagnetic fields (ELF and RF), 2012, Available at: http://www.bioinitiative.org/.
225. Matronchik AI, Belyaev IY. Mechanism for combined action of microwaves and static magnetic field: slow non uniform rotation of charged nucleoid. Electromagn Biol Med 2008;27:340–54.
226. Binhi VN, Alipov YD, Belyaev IY. Effect of static magnetic field on E. coli cells and individual rotations of ion-protein complexes. Bioelectromagnetics 2001;22(2):79–86.
227. Première reconnaissance d'un handicap dû à l'électrosensibilité en France. Le Monde fr avec AFP | 25.08.2015. Available at: http://www.lemonde.fr/planete/article/2015/08/25/premiere-reconnaissance-en-justice-d-un-handicap-du-a-l-electrosensibilite_4736299_3244.html.
228. Abelous D. France has its first radiation-free refuge in the Drome [Internet]. EURRE/Drome (FR): Agence France Presse (AFP), 2009 Oct 9. Available at: http://www.next-up.org/pdf/AFP_France_has_its_first_radiation_free_refuge_in_the_Drome_09_10_2009.pdf.
229. Ecoforma. Mit einem schadstofffreiem Haus gegen Schlafprobleme [Internet]. Sarleinsbach (AT): Ecoforma, 2014 Sept 9. Available at: http://www.ecoforma.co.at/holzbau-ecobau-lehrbaustelle/.
230. Friedmann J, Kraus S, Hauptmann Y, Schiff Y, Seger R. Mechanism of short-term ERK activation by electromagnetic fields at mobile phone frequencies. Biochem J 2007;405(3):559–68.
231. Simko M. Cell type specific redox status is responsible for diverse electromagnetic field effects. Curr Med Chem 2007;14(10):1141–52.
232. Pall ML. Explaining "Unexplained Illnesses": disease paradigm for chronic fatigue syndrome, multiple chemical sensitivity, fibromyalgia, post-traumatic stress disorder, Gulf War Syndrome, and others. New York, NY (US), London (GB): Harrington Park Press/Haworth Press, 2007, ISBN 978-0-7890-2388-9.
233. Bedard K, Krause KH. The NOX Family of ROS-Generating NADPH oxidases: physiology and pathophysiology. Physiol Rev 2007;87(1):245–313.
234. Pacher P, Beckman JS, Liaudet L. Nitric oxide and peroxynitrite in health and disease. Physiol Rev 2007;87(1):315–424.
235. Desai NR, Kesari KK, Agarwal A. Pathophysiology of cell phone radiation: oxidative stress and carcinogenesis with focus on male reproductive system. Reprod Biol Endocrinol 2009;7:114.
236. Straub RH, Cutolo M, Buttgereit F, Pongratz G. Energy regulation and neuroendocrine-immune control in chronic inflammatory diseases. J Intern Med 2010;267(6):543–60.
237. Gye MC, Park CJ. Effect of electromagnetic field exposure on the reproductive system. Clin Exp Reprod Med 2012;39(1):1–9.
238. Yakymenko I, Tsybulin O, Sidorik E, Henshel D, Kyrylenko O, et al. Oxidative mechanisms of biological activity of lowintensity radiofrequency radiation. Electromagn Biol Med 2015;19:1–16.

- 10.1515/reveh-2016-0011
Downloaded from PubFactory at 07/26/2016 08:26:15AM
via Slovak Academy of Science

239. Consales C, Merla C, Marino C, Benassi B. Electromagnetic fields, oxidative stress, and neurodegeneration. Int J Cell Biol 2012;2012:683897.
240. Pall ML. Microwave frequency electromagnetic fields (EMFs) produce widespread neuropsychiatric effects including depression. J Chem Neuroanat 2015. pii: S0891-0618(15)00059-9. DOI: 10.1016/j.jchemneu.2015.08.001. [Epub ahead of print].
241. Erdal N, Gurgul S, Tamer L, Ayaz L. Effects of long-term exposure of extremely low frequency magnetic field on oxidative/nitrosative stress in rat liver. J Radiat Res 2008;49(2):181–7.
242. De Luca C, Thai JC, Raskovic D, Cesareo E, Caccamo D, et al. Metabolic and genetic screening of electromagnetic hypersensitive subjects as a feasible tool for diagnostics and intervention. Mediat Inflamm 2014;2014:924184.
243. Myhill S, Booth NE, McLaren-Howard J. Chronic fatigue syndrome and mitochondrial dysfunction. Int J Clin Exp Med 2009;2(1):1–16.
244. Müller KE. Stressregulation und Mitochondrienfunktion. Zs f Orthomol Med 2012;1:1–13.
245. Buchner K, Eger H. Veränderung klinisch bedeutsamer Neurotransmitter unter dem Einfluss modulierter hochfrequenter Felder – Eine Langzeiterhebung unter lebensnahen Bedingungen. umwelt medizin gesellschaft 2011;24(1):44–57.
246. Hill HU, Huber W, Müller KE. Multiple-Chemikalien-Sensitivität (MCS) – Ein Krankheitsbild der chronischen Multisystemerkrankungen, umweltmedizinische, toxikologische und sozialpolitische Aspekte. Aachen (DE): Shaker-Verlag, 2010 Apr, 3rd edition, 500p. ISBN: 978-3-8322-9046-7.
247. Redmayne M, Johansson O. Could myelin damage from radiofrequency electromagnetic field exposure help explain the functional impairment electrohypersensitivity? A review of the evidence. J Toxicol Environ Health B Crit Rev 2014;17(5):247–58.
248. Von Baehr V. Rationelle Labordiagnostik bei chronisch entzündlichen Systemerkrankungen. umwelt medizin gesellschaft 2012;25(4):244–7.
249. Warnke U, Hensinger P. Steigende. "Burn-out"-Inzidenz durch technisch erzeugte magnetische und elektromagnetische Felder des Mobil- und Kommunikationsfunks. umwelt-medizin-gesellschaft 2013;26(1):31–8.
250. Havas M. Dirty electricity elevates blood sugar among electrically sensitive diabetics and may explain brittle diabetes. Electromagn Biol Med 2008;27(2):135–46.
251. Herbert MR, Sage C. Autism and EMF? Plausibility of a pathophysiological link – Part I. Pathophysiology 2013;20(3):191–209.
252. Herbert MR, Sage C. Autism and EMF? Plausibility of a pathophysiological link part II. Pathophysiology 2013;20(3):211–34.
253. Eskander EF, Estefan SF, Abd-Rabou AA. How does long term exposure to base stations and mobile phones affect human hormone profiles? Clin Biochem 2012;45(1–2):157–61.
254. Steiner E, Aufdereggen B, Bhend H, Gilli Y, Kalin P, et al. Erfahrungen des Pilotprojektes „Umweltmedizinisches Beratungsnetz" des Vereins Aerztinnen und Aerzte für Umweltschutz (AefU). Therapeutische Umschau 2013;70(12):739–45.
255. Hagstrom M, Auranen J, Johansson O, Ekman R. Reducing electromagnetic irradiation and fields alleviates experienced health hazards of VDU work. Pathophysiology 2012;19(2):81–7.
256. Oberfeld G. Die Veränderung des EMF Spektrums und ihre Folgen. In: Baubiologische EMF-Messtechnik. München, Heidelberg (DE): Hüthig and Pflaum Verlag, 2012. ISBN 1438-8707.
257. Berufsverband Deutscher Baubiologen. VDB-Richtlinien, Physikalische Untersuchungen, Band 1: Fürth (DE): Verlag AnBUS eV, 2006. 2nd edition. ISBN 3-9808428-6-X.
258. Virnich M. Gutachten über die messtechnische Untersuchung der Charakteristik von Funksignalen [Internet]. Salzburg (AT): Land Salzburg, 2015 Jun 26, 141p. Available at: https://www.salzburg.gv.at/gesundheit_/Seiten/technik.aspx.
259. Bundesamt für Umwelt. Orte mit empfindlicher Nutzung (OMEN) [Internet]. Bern (CH): Bundesamt für Umwelt, 2010 Mar 4. Available at: http://www.bafu.admin.ch/elektrosmog/13893/15175/15257/index.html?lang=de.
260. Kundi M, Hutter HP. Mobile phone base stations – Effects on wellbeing and health. Pathophysiology 2009;16(2–3):123–35.
261. National Council on Radiation Protection and Measurements (NCRP). Draft Report of NCRP Scientific Committee 89-3 on Extremely Low Frequency Electric and Magnetic Fields [Internet]. 1995 Jun 13. Available at: https://www.salzburg.gv.at/gesundheit_/Documents/ncrp_draft_recommendations_on_emf_exposure_guidelines_1995.pdf.
262. Oberfeld G. Prüfkatalog des Fachbereiches Umweltmedizin für das Vorhaben 380kV Freileitung von St. Peter a. H. zum Umspannwerk Salzach Neu (Salzburgleitung) der Verbund-Austrian Power Grid AG. [Internet] Salzburg (AT): Land Salzburg, 2006 Feb 27. Available at: https://www.salzburg.gv.at/gesundheit_/Documents/Umweltmedizin-Sbg.pdf.
263. Baubiologie Maes/Institut für Baubiologie + Nachhaltigkeit (IBN). Building Biology Evaluation Guidelines for Sleeping Areas (SBM-2015). Neuss, Rosenheim (DE): Baubiologie Maes, IBN., 2015 May, 3p. Available at: http://www.baubiologie.de/site/wp-content/uploads/richtwerte-2015-englisch.pdf.
264. Der Schweizerische Bundesrat. Verordnung über den Schutz vor nichtionisierender Strahlung (NISV) vom 23. Dezember 1999 [Internet]. Bern (CH): Der Schweizerische Bundesrat, 2012 Jul 1. Available at: https://www.admin.ch/opc/de/classified-compilation/19996141/index.html.
265. TCO Certified Displays 7.0-11 November 2015 [Internet]. TCO Development. Available at: http://tcodevelopment.com/files/2015/11/TCO-Certified-Displays-7.0.pdf.
266. Vignati M, Giuliani L. Radiofrequency exposure near high-voltage lines. Environ Health Perspect 1997;105(Suppl 6):1569–73.
267. Margaritis LH, Manta AK, Kokkaliaris KD, Schiza D, Alimisis K et al. Drosophila oogenesis as a bio-marker responding to EMF sources. Electromagn Biol Med 2014;33(3):165–89.
268. Gustavs K. Options to minimize non-ionizing electromagnetic radiation exposures (EMF/RF/Static fields) in office environments [Internet]. Victoria, BC (CA): Katharina Consulting, 2008 Nov 14. Available at: http://www.katharinaconsulting.com/s/2008_Gustavs_Low_EMF_Office_Environment.pdf.
269. Oberfeld G, Gutbier J. Elektrosmog im Alltag [Internet]. Stuttgart (DE): Diagnose Funk, 2013 Nov 10, 44p. Available at: https://www.salzburg.gv.at/gesundheit_/Seiten/infoblaetter.aspx.
270. Virnich M. Baubiologische EMF-Messtechnik – Grundlagen der Feldtheorie, Praxis der Feldmesstechnik. München, Heidelberg (DE): Hüthig & Pflaum Verlag, 2012. ISBN 1438-8707.
271. Pauli P, Moldan D. Reduzierung hochfrequenter Strahlung im Bauwesen: Baustoffe und Abschirmmaterialien. Fürth (DE): Hrsg. Berufsverband Deutscher Baubiologen VDB e.V., Verlag AnBUS e.V. 2015. ISBN 978-3-9814025-9-9.

- 10.1515/reveh-2016-0011
Downloaded from PubFactory at 07/26/2016 08:26:15AM
via Slovak Academy of Science

272. Levy F, Wannag A, editors. Nordic adaptation of classification of occupationally related disorders (diseases and symptoms) to ICD-10 [Internet]. Oslo (NO): Nordic council of ministers, 2000, 53p. DIVS: 2000:839, ISBN: 92-893-0559-2. Available at: http://www.nordclass.se/ICD-10_Nordic%20Occupational_2000.pdf.
273. Bansal M, Kaushal N. Oxidative stress mechanisms and their modulation. New Delhi (IN): Springer, 2014:167.
274. Brostoff J, Challacombe S. Food allergy and intolerance. London (GB): Balliere Tindall, 1987.
275. Andre CM, Larondelle Y, Eners D. Dietary antioxidants and oxidative stress from a human and plant perspective, a review. Curr Nutr Food Sci 2010;6(1):2–12.
276. Bouayed J, Bohn T. Exogenous antioxidants-double edged swords in cellular redox state; health beneficial effects at physiological doses versus deleterious effects at high doses. Oxid Med Cell Longev 2010;3(4):228–37.
277. Hoffmann W, Staller B. Prävention durch richtige Ernährung. umwelt medizin gesellschaft 2012;25(2):115–7.
278. Suzuki YJ, Packer L. Inhibition of NFkB activation by vitamin E derivates. Biochem Biophys Res Commun 1993;193(1):277–83.
279. Zingg JM. Modulation of signal transduction by vitamin E. Mol Aspects Med 2007;28(5–6):481–506.
280. Yeh SL, Wang HM, Chen PY, Wu TC. Interaction of ß-Carotin and flavonoids on the secretion of inflammatory mediators in an in vitro system. Chem Biol Interact 2009;179(2–3): 386–93.
281. Carcamo JM, Pedraza A, Borquez-Ojeda O, Golde DW. Vitamin C suppresses TNF alpha-induced NF kappa B activation by inhibiting I kappa B alpha phosphorylation. Biochemistry 2002;41(43):12995–3002.
282. Carcamo JM, Pedraza A, Borquez-Ojeda O, Zhang B, Sanchez R, et al. Vitamin C is a kinase inhibitor: dehydroascorbic acid inhibits IkappaBalphakinase beta. Mol Cell Biol 2004; 24(15):6645–52.
283. Kyaw M, Yoshizumi M, Tsuchya K, Suzaki Y, Abe S, et al. Antioxidants inhibit endothelin-1 (1-31)-induced proliferation of vascular smooth muscle cells via the inhibition of mitogenactivated protein (MAP) kinase and activator protein-1 (AP-1). Biochem Pharmacol 2002;64(10):1521–31.
284. Lubbad A, Oriowo MA, Khan I. Curcumin attenuates inflammation through inhibition of TLR-4 receptor in experimental Colitis. Mol Cell Biochem 2009;322(1–2): 127–35.
285. Woo JH, Lim JH, Kim YH, Soh SI, Min DS, et al. Resveratrol inhibits phorbol myristate acetate-induced matrix metalloproteinase-9 expression by inhibiting JNK and PKC delta signal transduction. Oncogene 2004;23(10):1845–53.
286. Nonn L, Duong D, Pechl DM. Chemopreventive anti-inflammatory activities of curcumin and other phytochemicals mediated by MAP kinase phosphatase-5 in prostata cells. Carcinogenesis 2007;28(6):1188–96.
287. Khan N, Mukhtar H. Multitargeted therapy of cancer by green tee polyphenols. Cancer Lett 2008;269(2):269–80.
288. Roskoski R. Biochemistry. Philadelphia, PA, USA: W.B. Saunders Company, 1996:530pp.
289. Devlin TM, editor. Textbook of Biochemistry with Clinical Correlations, 5th ed. New York, NY (US): Wiley-Liss, 2002.
290. Rassow J, Hauser K, Netzker, Deutzmann R. Biochemie. 2nd ed. Stuttgart (DE): Thieme, 2008:872pp.
291. Müller KE. Genetische Polymorphismen der Catechol-O-Methyltransferase (COMT). umwelt medizin gesellschaft 2007;20(4):282–8.
292. Rea WJ. Chemical Sensitivity, Vol. 2: Sources of Total Body Load, 1st ed. Boca Raton, FL (US): CRC Press/Lewis Publishers, 1994:569pp.
293. Schäfer SG, Elsenhans B, Forth W, Schumann K. Metalle. In: Marquardt H, Schäfer SG, editors. Lehrbuch der Toxikologie. Heidelberg (DE): Spektrum Akademischer Verlag, 1997:504–49pp.
294. Goyer RA, Cherian GM, editors. Toxicology of Metals. Berlin, Heidelberg (DE): Springer-Verlag, 1995:467pp.
295. Müller KE. Immuntoxikologie der Metalle. umwelt medizin gesellschaft 2004;17(4):299–301.
296. Aposian HV, Malorino RM, Gonzales-Ramirez D, Zuniga-Charles M, Xu Z, et al. Mobilization of heavy metals by newer, therapeutically useful chelating agents. Toxicology 1995;97(1–3):23–38.
297. Flora SJ, Pachauri V. Chelation in Metal Intoxication. Int J Environ Res Public Health 2010;7(7):2745–88.
298. Jennrich P. Detoxifikation von Schwermetallen. umwelt medizin gesellschaft 2012;25(4):24–7.
299. Pall ML. Do sauna therapy and exercise act by raising the availability of tetrahydrobiopterin? Med Hypotheses 2009;73(4):610–3.
300. Rozanowska M, Jarvis-Evans J, Korytowski W, Boulton ME, Burke JM, et al. Blue-light induced reactivity of retinal age pigment. In vitro generation of oxygen-reactive species. J Biol Chem 1995;270(32):18825–30.
301. Tolentino M, Morgan G. Popularity of electronic devices, "greener" light bulbes increases blue light exposure. Pri Care Optometry News 2012;18–9.
302. van der Lely S, Frey S, Garbazza C, Wirz-Justice A, Jenni OG, et al. Blue blocker glasses as a countermeasure for alerting effects of evening light-emitting diode screen exposure in male teenagers. J Adolesc Health 2015;56(1):113–9.
303. Narimatsu T, Negishi K, Miyake S, Hirasawa M, Osada H, et al. Blue light-induced inflammatory marker expression in the retinal pigment epithelium-choroid of mice and the protective effect of a yellow intraocular lens material in vivo. Exp Eye Res 2015;132:48–51.
304. Nishi T, Saeki K, Obayashi K, Miyata K, Tone N, et al. The effect of blue-blocking intraocular lenses on circadian biological rhythm: protocol for a randomised controlled trial (CLOCK-IOL colour study). BMJ Open 2015;5(5):e007930.
305. Mutter J, Naumann J, Schneider R, Walach H, Haley B. Mercury and autism: accelerating evidence? Neuro Endocrinol Lett 2005;26(5):439–46.
306. Mutter J, Naumann J, Guethlin C. Comments on the article "the toxicology of mercury and its chemical compounds" by Clarkson and Magos (2006). Crit Rev Toxicol 2007;37(6):537–49; discussion 551–2.
307. Mutter J, Curth A, Naumann J, Deth R, Walach H. Does inorganic mercury play a role in Alzheimer's disease? A systematic review and an integrated molecular mechanism. J Alzheimers Dis 2010;22(2):357–74.
308. Geier DA, King PG, Sykes LK, Geier MR. A comprehensive review of mercury provoked autism. Indian J Med Res 2008;128(4):383–411.

Supplemental Material: The online version of this article (DOI: 10.1515/reveh-2016-0011) offers supplementary material, available to authorized users.

- 10.1515/reveh-2016-0011
Downloaded from PubFactory at 07/26/2016 08:26:15AM
via Slovak Academy of Science

View publication stats
