# Supplementary material for: Magnetic Fields and Cancer: Epidemiology, Cellular Biology, and Theranostics
Source: Int J Mol Sci. 2022 Jan 25;23(3):1339. doi: 10.3390/ijms23031339 (PMC8835851; doi:10.3390/ijms23031339)
Supplement: Supplementary file 1 [file ijms-23-01339-s001.zip › Supplementary Data Set S1/MF and Cancer.Data/PDF/0947243666/A27593D678F5E4835AE750371276B67F.pdf]

RESEARCH COMMUNICATION

Pituitary Toxicity but Lack of Rat Colon Carcinogenicity of a DC-Magnetic Field in a Medium-Term Bioassay

Elsayed I Salim[1]*, Khalid M Omar[2], Hassan A Abou-Hattab[1], Fouad A Abou-Zaid[1]

Abstract

The present study was designated to evaluate the effect of direct current induced permanent magnetic field (DC-MF) on chemically induced rat colon carcinogenesis. Five experimental groups of male S.D. rats were injected with 1,2-dimethylhydrazine (DMH) subcutaneously, 20 mg/kg b.wt., once a week for four weeks, with exposure to 1 mT DC-MF (12 hours/day) as follows: Before (pre) the carcinogen administration (group 1), simultaneously (group 2), after (post) the carcinogen administration (group 3) and daily from the beginning to the end of the experiment after 12 weeks (group 4). Rats of group 5 served as carcinogen-only treated controls while those of group 6 were non-treated controls. There were no differences in the incidences and multiplicities of colonic aberrant crypt foci (ACF), putative preneoplastic lesions, among all groups except that large foci in group 1 were significantly fewer in numbers than those found in group 5. Proliferating cell nuclear antigen labeling indexes (PCNA-LI) in the colon epithelium were essentially the same in MF-treated and control rats. Histopathological examination showed evident hemorrhage in the pituitary glands of some rats of groups 1-3, and in most rats of group 4. Transmission electron microscopy also revealed ultrastructural changes, but DNA ploidy analysis revealed no carcinogenicity to MF-exposed pituitary glands. Serum levels of AST, ALT, total protein, creatinine, albumin, albumin/globulin ratio and growth hormone levels did not change among the groups. The present study revealed that the action of an artificial MF on rats is not carcinogenic/or cancer-promoting, at least in the present protocol for colon carcinogenesis.

Key Words: DC magnetic field - rat colon carcinogenesis - ACF - pituitary gland - TEM - DNA ploidy

Asian Pacific J Cancer Prev, **9**, 131-140

Introduction

Electromagnetic fields (EMFs) generated from all kinds of electrical equipment have caused concern as potential human health hazards for several decades. These weak fields induce different effects in the human body, but the resulting impact on human health is still unclear. There is very little theoretical reason to suspect that EMF might cause or contribute to cancer or any other human health problems, and there are very limited laboratory and epidemiological evidence for any connection. During recent decades, the most public concern about EMF and cancer has concentrated on power-frequency, microwave (MW) and radiofrequency (RF) fields. Possible effects of MF have been the subject of many reports from epidemiological, animal or cellular studies in different countries (Szuba, 2008).

EMF is composed of electric and magnetic fields that are related to each others. The fields induce currents and penetrate different cells in the body, e.g. in the nervous system. Nevertheless, despite experiments having shown effects on biological systems after external exposure to EMF, our understanding of the primary mechanism behind these findings is still fragmentary and obscures. The MFs from man-made sources generally have higher intensities than the naturally occurring fields. In the home and public places, magnetic flux densities ranging from 0.03 mT to 30 mT are produced around household appliances, and up to 35 mT near transmission lines (50 and 60 Hz), depending on the current carried and the distance from the line (Svedenstål, 1997). For magnetically levitated transportation systems, MFs of 6-60 mT are expected in the region of a passenger’s head. Security systems in libraries and storehouses produce fields of up to about 1 mT and operate at frequencies between 0.1 and 10 kHz (Lin-Liu and Adey, 1982).

Epidemiological studies from many countries showed possible carcinogenic effects of MF on populations at risk to high exposure values (Feychting et al., 2005). Floderus et al., (1993) showed a connection between exposure to MF and an increased risk of brain tumors in humans. Bethwaite et al., (2001) found a risk of acute leukemia for electrical workers, and risk was also found for the specific occupational categories of welders/flame cutters and

[1]*Zoology Department,* [2]*Physics Department, Faculty of Science, Tanta University, Tanta 31527, Egypt *For Correspondence: Telefax: +20-40-3302803, E-mail: elsalem_777@yahoo.com*

telephone line workers (Man and Shahidan, 2007). Hakansson et al., (2002) found earlier that men exposed to high levels of extremely low frequency magnetic fields (ELF-MF) showed an increased incidence of tumors of the kidney, pituitary gland, biliary passages and liver. Moreover, Li et al., (2003) noted an association between MF exposure and a greater mean age at diagnosis for brain tumors. Generally, all these studies mainly approached reasonable latencies found an increased cancer risk associated with the exposure to MF (Kundi et al., 2004).

On experimental levels, exposure to MF has been suggested to be a cancer promoter factor in animal experiments (Löscher and Mevissen, 1994; Saunders, 2005). A flux density of 1 mT significantly reduced the levels of nocturnal melatonin in blood-plasma and pineal gland in rats (Kato et al., 1993) and a decreased level of melatonin was also found changed after MF exposure to breast cancer cells (Touitou et al., 2003). Moreover, evidence was shown for leukemia initiation in hamsters (Holmberg, 1995). On the other hand, MF has been found to produce a great variety of effects such as increase in ornithine decarboxylase (ODC) activity in mammary epithelium of SD rats and in beta-galactosidase gene expression and oncogene transcription (Fedrowitz et al., 2004). In a case-control animal study, Reif et al., (1995) found a higher risk for lymphoma in pet dogs exposed to very high residential current codes. In contrast, in a review of different animal models used in tumor development studies, no effects of MF exposure on cancer development was concluded (Holmberg and Rannug, 1995).

Previous two-year studies of the USA National Toxicology Program (NTP) in EMF promotion/DMBA initiation in rats and mice indicated a thyroid C-cell focal hyperplasia increase in rats. Under the conditions of this study, NTP concluded that there was “equivocal evidence of carcinogenic activity” of 60-Hz MFs in male F344/N rats. On the other hand, they concluded that there was “no evidence of carcinogenic activity” in female F344/N rats or male or female B6C3F1 mice exposed to 0.02, 2, or 10 G, or 10 G intermittent 60-Hz MFs. Some support in female rat data for a trichoepithelioma association was obtained from NTP 26-week initiation/promotion studies, even if the experimental designs were different (NTP technical reports on EMF, 1998).

Although MFs may enhance tumorigenesis, they are not considered mutagenic. Some studies showed that induced MF did not cause chromosomal aberrations (Cooke and Morris, 1981; Peteiro-Cartelle and Cabezas-Cerrato, 1989), DNA strand breaks (Raylman et al., 1996), sister chromatid exchange, cell transformation, mutations or micronucleus formation (Suzuki et al., 2001). In contrast to these findings, one report using the comet assay, showed previously an increased number of DNA single-strand breaks in brain cells of rats after exposure to 0.1 mT MF (Lai and Singh, 1995). The mechanism of possible tumorigenic effects of MF has not been clearly elucidated. However, one report suggested previously that modulation of the kinetics of the transition from apoptosis to secondary necrosis by MF *in vivo* may play a role in inflammation and tumorigenesis in experimental animals (Teodori et al., 2002).

Several in vitro, epidemiological and cohort studies for humans have indicated variable effects of EMFs on the colon. A variable low-intensity low-frequency EMF caused increase in growth of human colon adenocarcinoma cells at 1 Hz and 25 Hz when the tissues were treated for 15 minutes, but a contrasting decrease was evident after treatment with 1 Hz for 360 minutes (Ruiz Gomez et al., 1999). Colon cancer was positively associated with EMF exposure in one epidemiological study (Floderus et al., 1993). However, a cohort study in Sweden investigating the cancer incidence in workers exposed to high levels of extremely low frequency EMFs, showed a significant reduction in the incidence of colon and connective tissue/muscle cancers, although incidences of kidney, pituitary gland, and biliary liver tumors were increased in men (Hakansson et al., 2002). There are also some studies suggesting that EMF might affect the pituitary gland. Acute exposure to linearly polarized 10 mT MFs at 50-Hz was shown to affect the hormones of the hypothalamic-pituitary-thyroid and hypothalamic-pituitary-adrenal axes of man (Selmaoui et al., 1997).

Aberrant crypt foci (ACF) of the colon are considered putative preneoplastic lesions because they share many morphological and molecular characteristics with tumors, including a comparable increase in cell proliferation, elevated expression of tumor-associated antigens and dysplasia (Kukitsu, et al., 2008). DNA ploidy was proved to be an important factor in the assessment of malignant tumors because it provides valuable information regarding tumor behavior (Xing et al., 2005). Determination of DNA content has been performed also by many methods such as flow cytometry and image cytometrical analysis. Variations in DNA content are common in many carcinogenic preneoplastic lesions and tumor cells (Zetteberg and Forsslund, 1991). This is because the cells which constitute the tumor may either gain additional DNA, or lose DNA, and this imbalance in DNA content contributes to the behavior of the tumor. A secondary result of ploidy analysis is identification of the percentage of cells that are proliferating (proliferating cells double their DNA, then divide), so, DNA ploidy analysis could be used as an indicator for the level of cellular proliferation in certain cells (Sasaki et al., 1999).

To study the effect of DC-MF on chemically induced colon carcinogenesis in rats, the numbers of ACF were taken as surrogate end point biomarkers for colon carcinogenesis in the present study. The study considered also any possible related toxic effects of DC-MF on blood biochemistry and pathology of most organs. Due to the previous epidemiological data revealing some effects of MF on brain tumors induction, special concern was given to organs of specialization such as brain and pituitary glands.

Materials and Methods

Test chemical for carcinogenesis study:

1,2-Dimethylhydrazine dihydrochloried (DMH. 2HCl) was purchased from Fluka co. (Sigma - Aldrich, St. Louis, MO, USA). Animals in test groups were given

subcutaneous (s.c.) injections of DMH dissolved in 0.9% saline solution, 20 mg/kg body wt, once a week for 4 weeks.

Animal husbandry and diet

Healthy, 7-week-old male Sprague- Dawley (SD) rats were obtained from the experimental animal breeding facility, Helwan University, Helwan- Giza, Egypt, and allocated to dielectric polymer cages with wood chips for bedding and allowed to acclimate for 1 week in animal house conditions before experimentation. The institutional animal care and use facility of the Faculty of Science, Tanta University, Tanta, Egypt, approved the experimental design. Target values for temperature and relative humidity measured in the animal house were about 22 ± 1˚C and 55 ± 5% respectively. The lighting was 12h /12h light – dark cycle. The rats were given drinking water and normal experimental animal food adjusted to be less-fat - less-fiber *ad libitium*. Animals body weights, food consumption, and water intakes were measured precisely every week.

Magnetic flux pattern due to current in a solenoid

The MF producing system was originally designed in the laboratories of the Physics and Zoology Departments of the Faculty of Science, Tanta University, Tanta, Egypt. It was depicted to suit a typical laboratory arrangement for application of a MF to test biological subjects. The MF exposure system was manufactured to provide a uniform field distribution. During construction, silicon was layered between coils to glue them together. This minimizes vibration noise when the coils are activated. The coils were wound on tube of conducting matterial (iron), therefore it is completely shielded against emission of electric fields. The coil was connected in series with a battery, ammeter, rheostat, fan and switch. The current was adjusted to at least 2A (Fig. 1). The current through the coils gives rise to MFs that are reasonably uniform near the common axis of the coils.

The field strength measured in Tesla (T) and the MFs distribution inside the cages and special uniformity of the field were evaluated and standardized along the bottom line of the cage using a probe field meter (a variable FH 51 Gauss-/Teslameter (MAGNET-PHYSIK, model No. 2000510, Köln, Germany) with a small probe 761. Since the field meter disturbed the MF inside the cage, field intensity measurements were performed only along the axis of the solenoid coil. Animals were exposed to a D.C.-MF in the centre of coil at flux intensity of (1 mT) for 12h/day as workday period. The present used strength was chosen because the level of human occupational exposure is about 1 mT (Kowalczuk et al., 1995).

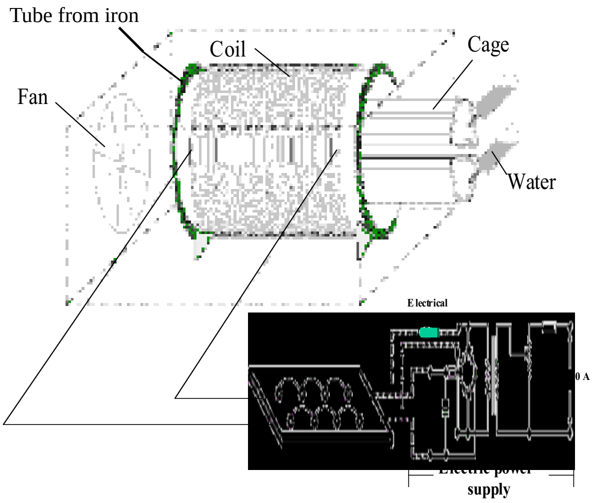

Figure 1. Exposure System for the Magnetic Field

Experimental procedures

A total of 48 rats were divided by body weight into six groups with equal number of animals. Rats in all groups, except those of group 6, were given DMH as described above from the end of the fourth week till the end of the seventh week of the experiment. Rats were exposed to DC-MF by being placed in special cages inside the instrument tube (12 hours/day from 8 am- to- 8 pm) according to the following protocols: Rats of group 1 were exposed to DC-MF for four weeks before (pre) the carcinogen administration. Rats of group 2 were exposed simultaneously during the DMH administration from week 4 till the end of week 7. Rats of group 3 were exposed for four weeks after (post) the carcinogen administration. Those of group 4 were exposed every day from the beginning to the end of the experiment. Rats of group 5 served as carcinogen-treated controls and rats of group 6 were injected with 0.9% saline in the same period as DMH administration, and served as non-treated controls.

Termination and histopathological preparations

After 12 weeks of treatment, all rats were sacrificed under excess of ether anesthesia. Gross examinations were performed on all rats during sacrifice. Absolute and relative kidneys and liver weights were taken for all rats. Specimens from the liver, kidney, spleen, colon, brain, pineal gland, and pituitary gland were preserved in 10% phosphate-buffered formalin until routinely prepared for histopathological examination. After embedding in paraffin wax, 4-5 mm sections of the collected tissues were stained with haematoxylin and eosin.

Electron microscopy

The pituitary glands from groups 4 and 6 were excised during necropsy and divided sagittally into two halves. One half was immediately fixed in cold 4% phosphate-buffered glutaraldehyede (PH 7.4) for 24 hours, and the other half was fixed in 10% phosphate buffered formalin. The material in glutaraldehyede was washed in Millonig's phosphate buffer (PH 7.3) and then post-fixed in 2% osmic acid in Millonig's phosphate buffer at 4 °C for 4 hours. The specimens were then dehydrated and then air-dried. These specimens were blocked in epoxy resin and semi thin sections were stained by methylene blue. Thin sections were mounted on copper grids and viewed under electron microscope (Philips 400 T) at 80 Kv, and micrographs of selected areas of different regions were taken by an attached camera and were enlarged as required. The classification of cellular criteria of rat pituitary glands was distinguished according to Nogami and Yoshimura, (1982).

Preparation of the colon and ACF counting

At necropsy, the entire colon was removed, opened longitudinally and washed in saline solution. The colon was divided into three equal segments, referred to as proximal colon (adjacent to the cecum), middle colon (mid colon) and distal colon (adjacent to the rectum). The segments were fixed in 10 % phosphate buffered formalin at 4 C° for at least 48 hours, and then stained with 0.2% methylene blue for ACF counting under light microscope. The number of ACF was determined, and their distribution patterns along the colon, and crypt multiplicities were scored. After ACF counting, 2 pieces of each of the colonic segments were embedded in paraffin, stained with H&E and examined for histopathological changes.

Blue Feulgen DNA ploidy analysis

A Blue Feulgen DNA Ploidy Analysis Staining Kit (ScyTek Labs. Inc., West Logan UT, UK) was used on formalin fixed paraffin embedded tissue sections of the from groups 4 and 5 pituitary glands. It was optimized for DNA ploidy specimens for staining then scanning and viewing on an image analysis system (Leica Qwin 500 Image Analyzer (LEICA Imaging Systems Ltd, Cambridge, England). Feulgen–thionin-stained slides were used to quantify nuclear DNA content. After DNA staining, the nuclear-integrated optical density is the cytometric equivalent of its DNA content. The technology was adapted to the recommendations of the European Society for Analytical Cellular Pathology (ESACP) Task Force on standardization of diagnostic DNA image cytometry (Böcking et al., 1995). Many fields were selected until the desired number of nuclei 100-150 could be measured. Before measurement, the cell galleries were reviewed to eliminate nuclei that were blurred or overlapping.

DNA cytometry and interpretation of DNA histograms

The results were displayed as a frequency histogram on the monitor generated by plotting the DNA content versus the number of nuclei counted. The DNA histograms were classified according to Danque et al., (1993) as diploid (euploid), and aneuploid DNA based on the amount of DNA relative to the normal control reference (the diploid position (2c) determined after calibration of the system). Briefly, the normal 2c reference value was established by measuring the normal well-preserved lymphocytes in each slide as internal reference cells, whose co-efficient of variation was <12.5% (S.D./mean). The measurement of the DNA content of diploid nuclei of experimental groups by the system was 1.5c-.2.5c with a DNA index (DI) ranging from 0.9 to 1.1, and fewer than 20% of the cells are present at the tertaploid position. A cell was defined as aneuploid if its DNA content deviated from 1.5c.-2.5c or when at least 10% of the total events show a distinct abnormal peak outside the 2C or 4C position. Cells with DNA index below <0.9 with ploidy <1.5c were classified separately. Tetraploid type was determined with a DNA index ranging from 1.8 to 2.2c. The proliferation index (PI) was automatically expressed as the percentage of cells engaged in the S-phase of the cell cycle with a DNA index between 2.5c-3.5c. It was classified according to Lee et al., (1994). 5c exceeding rate (5cER) was defined as the percentage of aneuploid cells having DNA content >5c.

Proliferating cell nuclear antigen (PCNA) immunohistochemistry

Immunohistochemical staining for PCNA was performed by the avidin-biotin complex method (Vecstain Elite ABC Kit, Vector Labs. Inc., Burlingame, CA). Colon tissue sections were deparaffinized, hydrated and immersed in 0.3% hydrogen peroxide for 30 min to block endogenous peroxidase activity. The sections were then incubated with 10% normal horse serum at room temperature for 45 min to block background staining and then incubated with PCNA antibody diluted 1:1000 in tris(hydroxymethyl)aminomethane-buffered saline (pH: 7.1) over night. The sections were then exposed to biotinylated horse anti-mouse IgG (Vector, Burlinghame, CA) for 30 min. Finally peroxidase activity in the colonic cell nuclei was visualized by treatment with 0.02% diaminobenzidine. Nuclei were counterstained with hematoxilin. At least 5 sections from each part of each colon was obtained from all animals and stained for PCNA. To evaluate PCNA-labeling index (PCNA-LI %), numbers of positively stained nuclei in all complete crypts were counted and divided by the total number of nuclei of each crypt to generate PCNA-LI (%).

Blood biochemistry

At the time of final autopsy, blood samples were collected from all rats via the abdominal aorta. Haematological analysis was performed on blood serum using colorimetric determination. Serum biochemistry data for aspartate aminotransferase (AST) activity, alanine aminotransferase (ALT) activity, total protein, albumin,

Table 1. Data for Final Average Body, Liver and Kidney Weights, and Food Consumption

Group	Treatment	No. of Rats	Body Weight Initial	Body Weight Final	Liver Weight Absolute	Liver Weight Relative (%)	Kidney Weight Absolute	Kidney Weight Relative (%)	Average food Consumption (g/rat/day)
1	MF (pre) +DMH	8	111.3 ± 5.1	211.1 ± 5.8	6.5 ± 0.7	3.1 ± 0.3	1.7 ± 0.3	0.8 ± 0.1	11.2
2	MF (simult.) +DMH	8	115.1 ± 6.1	214.5 ± 5.2	7.1 ± 0.7	3.3 ± 0.4	1.7 ± 0.3	0.8 ± 0.1	10.4
3	MF (post) +DMH	8	113.3 ± 5.2	210.4 ± 6.3	6.2 ± 0.5*	2.9 ± 0.3	1.4 ± 0.1	0.7 ± 0.1	9.5
4	MF (continous) +DMH	8	113.3 ± 6.5	170.6 ± 5.6*	5.9 ± 0.8*	3.5 ± 0.7	1.3 ± 0.1*	0.7 ± 0.1	8.3
5	DMH (only)	8	114.6 ± 5.9	218.6 ± 5.5	7.1 ± 0.7	3.3 ± 0.4	1.6 ± 0.3	0.7 ± 0.2	11.1
6	Saline (only)	8	113.9 ± 6.7	225.9 ± 0.2	7.2 ± 1.0	3.2 ± 0.4	3.2 ± 0.4	0.7 ± 0.1	11.2

[a]: Values are Means ± S.D. ; *: Significantly different vs. group 5 at P<0.05

Table 2. Effects of MF on ACF Formation in Colon

Group	Treatment	No. of Rats	Total No. of ACF	Number of ACF containing			
				1AC	2AC	3AC	≥4AC
1	MF (pre) +DMH	8	112.7 ± 16.5*	61.3 ± 2.9	27.3 ± 5.5	13.3 ± 4.8*	10.9 ± 4.7*
2	MF (simult.) +DMH	8	168.0 ± 18.6	81.0 ± 4.1	44.3 ± 4.5	25.3 ± 8.7	17.6 ± 6.3
3	MF (post) +DMH	8	132.4 ± 17.4	68.4 ± 5.8	35.1 ± 2.4	16.9 ± 6.6	12.0 ± 5.9
4	MF (contin.) +DMH	8	124.6 ± 27.6	51.6 ± 8.4	34.7 ± 6.9	21.6 ± 5.6	16.7 ± 6.8
5	DMH (only)	8	168.4 ± 18.6	65.6 ± 3.8	48.0 ± 5.9	31.0 ± 3.5	23.9 ± 5.0
6	Saline (only)	8	0	0	0	0	0

[a]: Values are Means ± S.D. ; *: Significantly different vs. group 5 at $P<0.05$

Table 3. ACF Density (No/cm^2)

Group	Treatment	No. of Rats	Colonic Region			
			Entire colon	Proximal	Middle	Distal
1	MF (pre) + DMH	8	2.7 ± 0.4 [a]	0.7 ± 0.6	3.1 ± 0.4*	4.2 ± 0.5
2	MF (simult.) + DMH	8	3.8 ± 0.6	1.9 ± 0.5	6.4 ± 0.5	3.0 ± 0.6
3	MF (post) + DMH	8	2.9 ± 0.5	0.4 ± 0.5	4.4 ± 0.4	3.8 ± 0.4
4	MF (contin.) + DMH	8	3.4 ± 0.7	1.3 ± 0.4	5.4 ± 0.6	3.6 ± 0.5
5	DMH (only)	8	3.5 ± 0.5	0.8 ± 0.6	6.0 ± 0.4	3.5 ± 0.5
6	Saline (only)	8	0	0	0	0

[a]: Values are Means ± S.D. ; *: Significantly different vs. corresponding colonic region in group 5 at $P<0.021$.

albumin/globulin ratio (A/G ratio), creatinine (Doumas and Bigges, 1972), were measured in all rats and growth hormone enzyme immunoassay was measured in rats of groups 4 and 5 using the kit: Quantitative Determination Of Growth Hormone In Serum Catalogue #KH4005, CA, USA (Van Wyk and Underwood, 1978).

Statistical Analysis

The data obtained in the present work were represented in tables as mean ± standard deviation (S.D.). The significance of the difference between the data means was calculated according to Student's *t* - test.

Results

Body and organs weights and average food consumption

Table 1 shows data for the initial and final body weights, relative and absolute liver and kidney weights and calculations for the average food consumption. Figure 2 shows growth curves of animals in all experimental groups. Only rats of group 4 had significant body weight loss as compared with group 5 ($p<0.05$). Food consumption was almost similar among the groups except that of group 4 which showed a slight decrease in consumption as compared to group 5. Absolute liver weights of groups 3 and 4 were significantly decreased as compared to those of group 5 but data for relative liver weights and absolute and relative kidney weights did not significantly differ between the groups.

Number, multiplicity and region density of DMH-induced ACF

Table 2 summarizes the data for the effects of MF on induction and development of ACF in all DMH administered groups. Rats in groups 1-5 had 100% incidence of colonic ACF in contrast to the complete lack of such lesions without DMH treatment in group 6. In general, there was no promoting effect of MF on ACF induction in all MF-treated groups. In contrast, the total numbers of ACF and the numbers of large foci containing 3 and ≥4 ACs, were significantly decreased by pre exposure to MF in group 1 as compared to those found in group 5. Table 3 summarizes data for ACF density (No. of ACF/cm2) in different parts of the colon (proximal, mid and distal) and in the entire colon. There were no clear differences between ACF density in MF treated

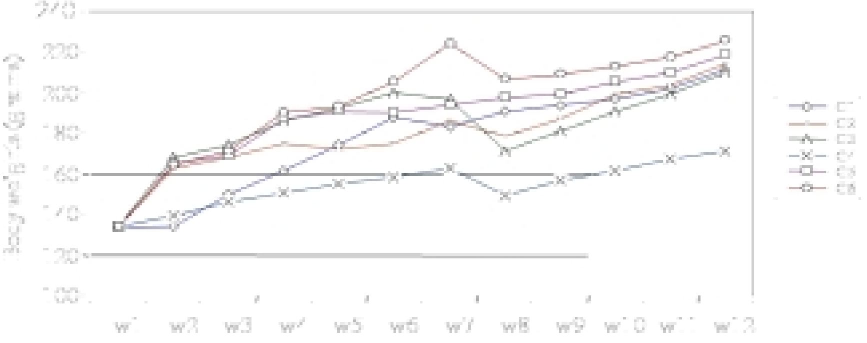

Figure 2. Growth Curves of Rats in the Experimental Groups

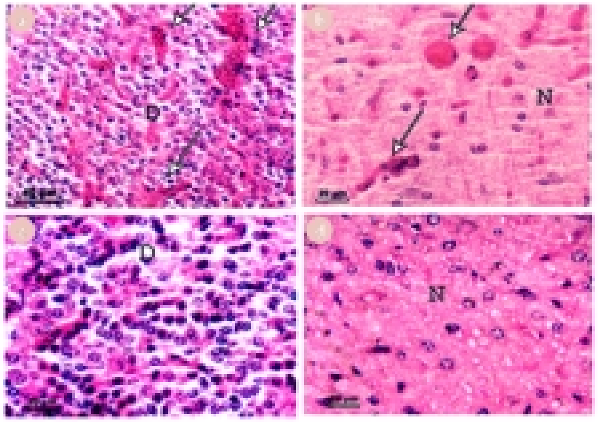

Figure 3. a-d: Photomicrographs of Sagittal Sections of Pituitary Glands of MF-exposed (a,b), and Normal Rats (c,d). D: pars distalis areas of the adenohypophysis. N: Neuropophysis (pars nervosa). Arrows indicate areas of haemorrhage, seen only in MF-exposed pituitary glands

groups as compared with control group 5 except in group 1 that showed ACF density in the mid colonic areas to be significantly less than those found in the corresponding area of group 5 (p<0.021).

PCNA-LI (%) in the colonic epithelium

DMH treatment increased generally the length of the proliferating zone in the colonic crypts to the middle and the upper thirds as compared with the lower one third of the colonic crypts in non-treated rats of group 6. Generally, the LI were not significantly MF-treated colons and the DMH-treated controls of group 5. Corresponding figures of the mean PCNA-LI (%) were 10.8%, 11.3%, 11.4%, 11.5%, 11.7% and 9.5% for groups 1-6, respectively.

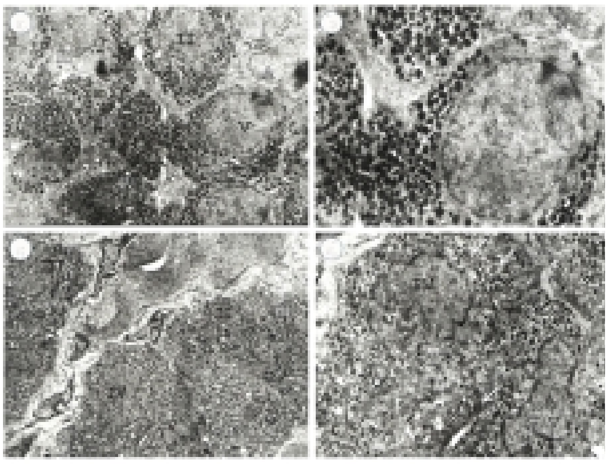

Figure 4. Electron micrographs of pituitary glands. a): normal rat pituitary showing type I (corticotroph), Type II (thyrotroph), type III (gonadotroph), type IV (prolactotroph), type V (somatotroph) and agranular type (A) cells. x4,480; (b): a magnified portion of (a). x12,800. Note rounded nuclei, homogenously distributed chromatin, smooth nuclear envelopes and cellular membranes and cytoplasmic electron dark granules; (c): the pituitary gland of an MF-exposed rat showing Type II (thyrotroph), type III (gonadotroph), type IV (prolactotroph) and type V (somatotroph) cells. x4,000; (d): a magnified portion of (c). Note that all cells are relatively small in size, with elongated nuclei containing rough and wrinkled nuclear envelopes and cellular membranes. Note also the large electron opaque granules (arrows) which are absent in normal pituitary glands. x12,800

Histopathology of the pituitary gland of the MF-exposed rats

Histopathological examination of the pituitary glands of the MF-exposed rats in group (4) revealed some abnormal features as compared to control groups 5 and 6. Twenty five percent (25%) of rats in group 1, 50% of rats in groups 2 and 3, and most rats (about 90%) of group 4 showed hemorrhage in pituitary glands (Figure 3). No hemorrhage was observed in the pituitary glands of rats in groups 5 or 6. The MF-treated pituitary glands of all groups (1-4) had smaller cells, with more elongated darkly stained basophilic nuclei as compared to control cells in groups 5 and 6.

Ultrastructure of the pituitary glands of MF-exposed rats

In MF-exposed pituitary glands of group 4, evident changes were seen particularly in the granular Type II; thyrotroph (TCH) cells, Type III; gonadotroph (GTH) cells, type IV; prolactotroph (LTH) cells, and type V; somatotroph (STH) cells as compared to corresponding control cells (Figure 4). The treated cells appeared smaller in size with more or less pyknoic nuclei and dense marginal chromatin. The cellular membranes and the nuclear envelops were apparently rough and wrinkled in contrast to the smooth and rounded membranes in the control cases. The cytoplasmic granules and vesicles were generally smaller in size than those found in control corresponding granular cells with many larger-electron opaque vesicles which were not noticed in the control pituitary glands. Also a few changes were noticed in the agranular Type (A) cells, which showed more elongated and oval nuclei with rough and wrinkled nuclear envelopes and condensed marginal chromatin. The control case pituitary gland cells had obviously rounded nuclei with smooth nuclear envelopes and homogenously distributed nuclear chromatin.

DNA ploidy analysis in pituitary gland cells

All MF-treated and control cases revealed predominantly diploid histograms (Figure 5,6). The mean percentage of diploid cells was 66.13% ± 0.24 in MF-treated -and 61.48% ± 0.28 in control pituitary glands, while the mean percentage of cells at S-phase (proliferation index) was 28.23% ± 0.28 and 22.13% ± 0.3 respectively. The mean DNA indexes in both groups were 1.12 % ± 0.6 and 0.99 % ± 0.3 respectively. The percentage of cells with DNA ploidy < 1.5c was much fewer in group 4 (3.23 %) than in the control group (16.8 %) but without significant difference. The mean percentage of cells in tetraploid status (3.5-4.5c) in group 4 tended to increase (3.23 %) as compared to control rats (0.82 %) but still in normal average. Cells beyond tetraploidy status (>4.5c) and those exceeding >5c (5cER) were absent. In general, there were no significant differences in DNA content between groups 4 and 6.

Blood biochemistry

Serum levels of AST and ALT, total proteins, albumin and the albumin/globulin ratio, creatinine in rats exposed to MF in groups 1, 2, 3, and 4 did not differ significantly

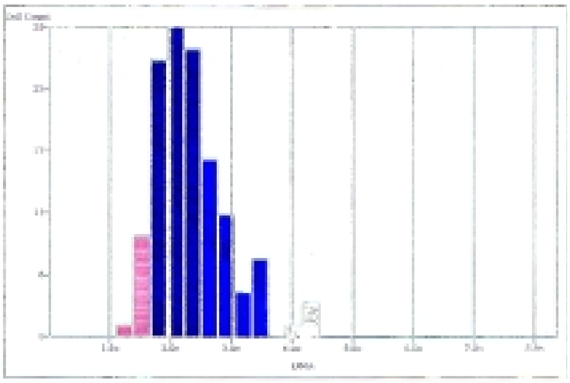

Figure 5. DNA Ploidy Status in MF-exposed Pituitary Glands (Group 4)

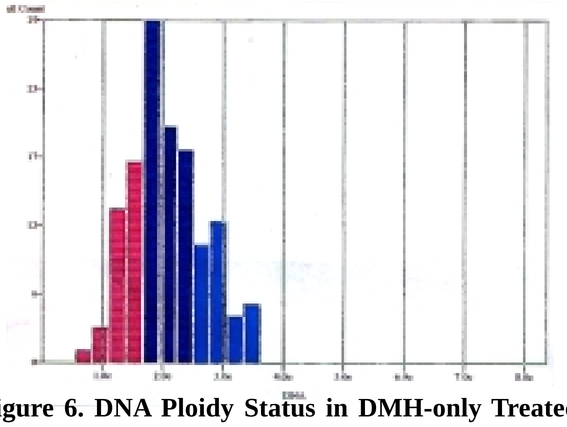

Figure 6. DNA Ploidy Status in DMH-only Treated Control Pituitary Glands (Group 5)

from levels of group 5 (DMH control). Serum growth hormone levels measured in groups 4 and 5 also did not differ between the two groups (Table 4).

Discussion

The increasing use of MF-producing equipment in industrial processes, research facilities, energy production and distribution, new transportation technologies, and medical practice, increases the possibility of human exposure to MF. Although up to now, both occupational and general-population exposures to MF have generally been at low levels, some new technologies, e.g., magnetically-levitated trains, and cellular phones and their magnification towers might result in exposure of the general population to levels comparable with the highest ones in some working environments (Stavroulakis, 2003). The present debate, dividing science in two opposite fields: one of them denying any appreciable carcinogenic effect of MFs, and the other one hypothesizing their dramatic effects, are presently devoid of reliable and exhaustive scientific support, which could only be provided by further research (Zapponi and Marcello, 2004). Fedrowitz et al., (2004) showed that MF exposure significantly increased mammary tumor development and growth in SD1 but not SD2 rats. This study indicated that the genetic background plays a pivotal role in effects of MF exposure. Exposure of female SD rats in 50 Hz MFs in the micro tesla range significantly facilitated the development and growth of mammary tumors. One possible explanation for this finding would be enhanced proliferation of breast epithelial stem cells by MF exposure, thereby increasing the sensitivity of these cells to chemical carcinogens (Mevissen et al., 1999). The action of EMF on different pathways related to cell physiology, proliferation, toxicity of chemicals, gene expression, etc., are currently being investigated in many laboratories worldwide, although the results are still not conclusive and even conflicting.

The present study aimed to evaluate the potential properties of MF against rat colonic ACF, that have been widely used as surrogate biomarkers to detect the effects of environmental risk factors (Salim et al., 2002) or modulators for colon cancer (Mori et al., 2004). In the present study, the MF exposure showed no obvious carcinogenic or cancer-promoting effects on colon carcinogenesis in rats exposed to MF in pre, simultaneous or post to the carcinogen (DMH) administration (initiation, promotion or progression stages of carcinogenesis). However, the results indicated that the MF exhibited some inhibitory effects on ACF formation when the rats were exposed prior (pre) to the DMH administration in group 1. In this respect, the total numbers of ACF as well as the average numbers of large ACF containing 3 or ≥4 crypts/focus were significantly reduced as compared to the ACF values in the DMH-treated control rats in group 5. In literature, it is documented that the colon tumor incidence in rats correlates directly with the numbers of large ACF (≥4 crypts/focus), which are more likely to persist, increase in size through multiplication and develop into tumors (Pretlow et al., 1992). Large ACF are also linked with colon cancer development from the viewpoint of dysplastic change, altered proliferative pattern, DNA adduct formation and genetic mutations (Shivapurkar et al., 1994; Salim, 2006). Moreover, there were no significant differences in PCNA-LI between groups 1-5 indicating no proliferative activity in colonic epithelial cells due to exposure to MF. Measurement of blood biochemistry levels in treated groups showed that only

Table 4. Data for Blood Concentration Levels of Total Proteins, Albumin, A/G Ratio, Creatinine and Growth Hormone (GH)

Group	Treatment	No. of samples measured	Total protein (g/dl)	Albumin (g/dl)	A/G Ratio	Creatinine (mg%)	GH (ng/m)
1	MF (pre) + DMH	6	8.0 ± 1.0[a]	5.9 ± 0.6	2.3 ± 0.6	1.2 ± 0.3	n.i.
2	MF (simult.) + DMH	6	7.2 ± 1.1	6.0 ± 1.0	2.1 ± 0.7	1.6 ± 0.4	n.i.
3	MF (post) + DMH	6	7.8 ± 1.3	5.7 ± 0.5	2.4 ± 0.7	1.6 ± 0.4	n.i.
4	MF (contin.) + DMH	6	6.3 ± 0.8	5.4 ± 0.4	2.1 ± 0.3	1.8 ± 0.2	< 0.05
5	DMH (only)	6	7.6 ± 1.4	5.8 ± 0.3	2.5 ± 0.5	1.7 ± 0.4	< 0.05
6	Saline (only)	6	8.4 ± 0.5	6.2 ± 0.7	2.7 ± 0.7	1.9 ± 0.5	n.i.

[a]: Values are Means ± S.D. ; n.i. not investigated. No significant values were obtained.

AST and ALT levels in group 4 were higher than normal control levels of group 6, probably due to unhealthy conditions of rats in this group after longer exposure periods to MF (12 weeks) coordinated with the carcinogen administration. Further studies are needed to verify the underlying reasons of the changes found herein in AST and ALT serum levels.

In therapeutic levels, electric or MFs were found recently to enhance chemotherapy, where the primary tumors could be exposed to a low EMF in combination with chemotherapeutic agents in the extracellular compartment of the tumor. This could cure mice with CT-26 colon carcinoma by inducing anti-tumor immunity (Plotnikov et al., 2004). When applied to murine B-16 melanoma with different chemotherapeutic drugs was found to achieve a cure level up to 30% (Plotnikov et al., 2003). The underlying mechanism of this uptake was explained previously to involve an electric field-induced endocytotic-like process (Rosemberg and Korenstein, 1990). The idea of using MF in colon cancer therapy was observed earlier when low frequency MFs (50 Hz) could produce apoptosis and small variations in cell cycle distribution on colon cancer cells, while lower frequencies did not have a similar pronounced effect (Ruiz Gomez et al., 2001). In a rat model of colon tumors induced by N-methyl-N-nitosurea (MNU), and exposed to 50 Hz frequency MF, it was suggested that the MF resulted in significant alterations in cellular adhesion (Tuncel et al., 2003).

To the authors' knowledge, there is a few previous epidemiological observations of an increased risk of colon cancer among people exposed to MFs (Tynes et al., 1992), whereas three other studies published have found no excess risks of colon cancer (see Theriault et al., 1994). As to the possible carcinogenic mechanism in colon cancer it has been suggested that the earlier observation of high stores of iron in the body increasing the risk might be explained by iron having a role in catalyzing oxygen radicals (Stevens, 1993). On the other hand, 60 Hz MFs have been reported to produce expression of transferrin receptors on human colon cancer cells in vitro (Phillips et al., 1986) that is, body iron could act as a co-carcinogen with extremely low frequency MFs.

In the present study, there was evident hemorrhage in the pituitary glands of some rats of groups 1-3 and in most rats of group 4 that were continuously exposed to MF. Electron microscopy investigation showed that the cells of the MF-exposed pituitary gland were obviously smaller in size with irregular and compact elongated and dense nuclei as compared to control cells. The DNA ploidy investigation revealed that there were no clear carcinogenic effects or differences in DNA content between the MF-exposed and control pituitary glands. Alterations in DNA ploidy status and cell proliferation are known to be prognostic factors for colorectal cancer (Lammering et al., 2000). Abbas et al., (2001) showed previously that DNA ploidy correlated with the tumor histological grading of urinary bladder transitional cell carcinomas, as there was an increase in the percentage of aneuploid tumors with increase in tumor grading. DNA aneuploidy was related to high grade tumors (GIII) while all cases of grade I revealed predominantly diploid histograms. Moreover, the increase in tetraploidy was shown to be related to moderately differentiated urinary bladder tumors (Lipponen and Eskelinen, 1992). The no carcinogenic effect on the pituitary glands was indicated here by the findings that the ratio of diploid (euploid) cells, DNA indexes (DI) and cellular proliferation index (PI) were almost similar in both MF-treated and non-treated groups. Moreover, the percentages of 5cER were 0% in the two cases. 5cER in addition to another DNA ploidy parameter; the 2c deviation index (2cDI) are two parameters applied recently to calculate the malignancy grade (MG) in malignant tumors (Xing et al., 2005).

The present data which shows no obvious carcinogenic effects of the MF on the rat colon and pituitary gland, confirm most of the recent developing evidence of safety of MF for cancer initiation, at least at the studied exposure levels in experimental animals. The possibility that thermal effects could cause the present profound pathological findings in the pituitary glands of the MF-exposed rats apparently needs further confirmation. Published laboratory studies have reported that static MFs do not cause any of the effects that indicate genotoxicity. Imaida et al., (1998) reported that no effects of a EMF-near field of 929.2 MHz pulse modulated microwaves that are used for portable cellular phones were observed on rat liver in a medium-term liver bioassay, which is well established for detecting carcinogens and promoters on hepatocarcinogenesis.

Rannug et al., (1993) used 50 Hz MFs that generated with equipment consisting of copper coils surrounding racks of animal cages, and the rats, which had undergone partial hepatectomy and diethylnitrosamine treatment, were exposed for 12 weeks. The numbers of both g-glutamyl trans-peptidase and GST-P positive foci in treated groups were within the control range. Thus neither a MF of 50 Hz nor a high frequency electromagnetic near-field influences the development of pre-neoplastic hepatocellular lesions in the rat. In line with the present results, Takahashi et al., (2002) reported the lack of mutation induction or any direct effect with exposure to 1.5 GHz electromagnetic near fields used for cellular phones in brains of Big Blue mice, not only in terms of brain DNA damage but also in proliferation of glial cells. Their findings of normal in DNA mutations in brain cells were also in line with other previous reports (Nishino et al., 1996; Malyapa et al., 1998).

Although the MF used in the present study might have exerted some pathological effects in the pituitary gland tissues, these effects are not carcinogenic. Furthermore, the MF did not exert any promoting effect on colon carcinogenesis and did not show any carcinogenic effect on any other organs. These results are in line with the most recent studies worldwide on the non-carcinogenic effects of MFs particularly on the experimental levels.

References

Abbas N, El-Sharkawy S, Shatta W, et al(2001). PCNA expression and DNA ploidy in transitional cell carcinoma of urinary bladder: Correlation with tumour grading. *Med J Cairo Univ*, **69**, 83-90.

Bethwaite P, Cook A, Kennedy J, et al (2001). Acute leukemia in electrical workers: a New Zealand case-control study. *Cancer Causes Control*, **12(8)**, 683-9.

Böcking A, Giroud F, Reith A (1995). Consensus report of the ESACP task force on standardization of diagnostic DNA image cytometry. *Anal Cell Pathol*, **8**, 67–74.

Cooke P, Morris PG (1981). The effects of NMR exposure on living organisms. II. A genetic study of human lymphocytes. *Br J Radiol*, **54**, 622-5.

Danque PO, Chen HB, Patil J, et al (1993). Image analysis versus flow cytometry for DNA ploidy quantitation of solid tumors: a comparison of six methods of sample preparation. *Mod Pathol*, **6**, 270-5.

Doumas BT, Bigges HG (1972). Standard methods of clinical chemistry (G. A. Cooper, ed.), New York, Academic Press, vol. 7.

Fedrowitz M, Kamino K, Loscher W (2004). Significant differences in the effects of magnetic field exposure on 7,12-dimethylbenz(a)anthracene-induced mammary carcinogenesis in two substrains of Sprague-Dawley rats. *Cancer Res*, **64**, 243-51.

Feychting M (2005). Health effects of static magnetic fields-a review of the epidemiological evidence. *Prog Biophys Mol Biol*, **87**, 241-6.

Floderus B, Persson T, Stenhmd C, et al (1993). Occupational exposure to electromagnetic fields in relation to leukemia and brain tumors: a case-control study in Sweden. *Cancer Causes and Control*, **4**, 465-76.

Hakansson N, Floderus B, Gustavsson P, et al (2002). Cancer incidence and magnetic field exposure in industries using resistance welding in Sweden. *Occup Environ Med*, **59**, 481-6.

Holmberg B (1995). Magnetic fields and cancer: animal and cellular evidence: an overview. *Environ Health Perspect*, **103**, 63-7.

Holmberg B, Rannug A (1995). Magnetic fields and cancer development in animal model. *Radio Science*, **30**, 223-31.

Imaida K, Hagiwara A, Yoshino H, et al (2000). Inhibitory effects of low doses of melatonin on induction of preneoplastic liver lesions in a medium-term liver bioassay in F344 rats: relation to the influence of electromagnetic near field exposure. *Cancer Lett*, **155**, 105-14.

Imaida K, Taki M, Yamaguchi T, et al (1998). Lack of promoting effects of the electromagnetic near-field used for cellular phones (929.2 MHz) on rat liver carcinogenesis in a medium-term liver bioassay. *Carcinogenesis*, **19**, 311-4.

Kato M, Honma KL, Shigemitsu T, et al (1993). Effects of exposure to a circularly polarized 50 Hz magnetic field on plasma and pineal melatonin levels in rats. *Bioelectromagnetics*, **14**, 97-106.

Kowalczuk CI, Robbins L, Thomas JM, et al (1995). Dominant lethal studies in male mice after exposure to a 50 Hz magnetic field. *Mutat Res*, **328**, 229-37.

Kukitsu T, Takayama T, Miyanishi K, et al (2008). Aberrant crypt foci as precursors of the dysplasia-carcinoma sequence in patients with ulcerative colitis. *Clin Cancer Res*, **14**, 48-54.

Kundi M, Mild K, Hardell L, et al (2004). Mobile telephones and cancer-a review of epidemiological evidence. *J Toxicol Env Health*, **7**, 351-84.

Lai H, Singh NP (1995). Acute exposure to a 60 Hz magnetic field increases DNA single-strand breaks in brain cells of Die rat. Abstract of the Annual Review of Research on Biological Effects of Electric and Magnetic Fields from the Generadon, Delivery and Use of Electricity, Albuquerque, New Mexico.

Lammering G, Taher MM, Gruenagel H-H, Bet al (2000). Alteration of DNA Ploidy Status and Cell Proliferation Induced by Preoperative Radiotherapy Is a Prognostic Factor in Rectal Cancer. *Clin Cancer Res*, **6**, 3215–21.

Lee YT, Kuo SH, Lai, et al (1994). Nuclear area and DNA content in tumor and nontumor portions of hepatocellular carcinoma. *Anal Quant Cytol Histol*, **16**, 153-8.

Li CY, Lin RS, Sung FC (2003). Elevated residential exposure to power frequency magnetic field associated with greater average age at diagnosis for patients with brain tumors. *Bioelectromagnetics*, **24**, 218-21.

Lin-Liu S, Adey WR (1982). Low frequency amplitude modulated microwave fields change calcium efflux rates from synaptosomes. *Bioelectromagnetics*, **3**, 309-22.

Lipponen PK, Eskelinen MJ (1992). Cell proliferation of transitional cell bladder tumors determined by PCNA/cyclin immunostaining and its prognostic value. *Br J Cancer*, **66**, 171-6.

Löscher W, Mevissen M (1994). Animal studies on the role of 50/60-Hertz magnetic fields in carcinogenesis. *Life Sciences*, **54**, 1531-43.

Malyapa RS, Ahern EW, Bi C, et al (1998). DNA damage in rat brain cells after in vivo exposure to 2450 MHz electromagnetic radiation and various methods of euthanasia. *Radiat Res*, **149**, 637- 45.

Man AK, Shahidan R (2007). Variations in occupational exposure to magnetic fields among welders in Malaysia. Radiat Prot Dosimetry. Nov 28; [Epub ahead of print].

Mevissen M, Haussler M, Löscher W (1999). Alterations in ornithine decarboxylase activity in the rat mammary gland after different periods of 50 Hz magnetic field exposure. *Bioelectromagnetics*, **20**, 338-46.

Mori H, Yamada Y, Kuno T, et al (2004). Aberrant crypt foci and beta-catenin accumulated crypts: significance and roles for colorectal carcinogenesis. *Mutat Res*, **566**, 191-208.

Nishino H, Schaid DJ, Buettner VL, et al (1996). Mutation frequencies but not mutant frequencies in Big Blue mice fit a Poisson distribution. *Environ Mol Mutagen*, **28**, 414-7.

Nogami H, Yoshimura F (1982). Fine structural criteria of prolactin cells identified immunohistchemically in the male rat. *Anat Rec*, **202**, 261-74.

NTP Peer Review of Draft Technical Reports of Toxicology and Carcinogenesis Studies of Electric and Magnetic Fields by the BSC Technical Reports Review Subcommittee: Meeting Minutes March 11, 1998. http://ntp.server.niehs.nih.gov/index.cfm.

Peteiro-Cartelle FJ, Cabezas-Cerrato J (1989). Absence of kinetic and cytogenetic effects on human lymphocytes exposed to static magnetic fields. *Bioelectromagnetics*, **8**, 11-9.

Phillips JL, Rutledge L, Winters WD (1986). Transferrin binding to two human colon carcinoma cell lines: characterization and effect of 60-Hz electromagnetic fields. *Cancer Res*, **46**, 239-44.

Plotnikov A, Fishman D, Tichler T, et al (2004). Low electric field enhanced chemotherapy can cure mice with CT-26 colon carcinoma and induce anti-tumour immunity. *Clin Exp Immunol*, **138**, 410-6.

Pretlow TP, Cheyer C, O'Riordan M (1994). Aberrant crypt foci and colon tumors in F344 rats have similar increases in proliferative activity. *Int J Cancer*, **56**, 599-02.

Rannug A, Holmberg B, Mild KH (1993). A rat liver foci promotion study with 50-Hz magnetic fields. *Environ Res*, **62**, 223–9.

Raylman RR, Clavo AC, Wahl RL (1996). Exposure to strong static magnetic field slows the growth of human cancer cells in vitro. *Bioelectromagnetics*, **17**, 358-63.

Reif JS, Lower KS, Ogilvie GK (1995). Residential exposure to magnetic fields and risk of canine lymphoma. *Am J*

Epidemiol, **141**, 352-9.
Rosemberg Y, Korenstein R (1990). A novel method for measuring membrane conductance changes by a voltage-sensitive optical probe. *FEBS Lett*, **263**, 155-8.
Ruiz Gomez MJ, Pastor Vega JM, de la Pena L, et al (1999). Growth modification of human colon adenocarcinoma cells exposed to a low-frequency electromagnetic field. *J Physiol Biochem*, **55**, 79-83.
Ruiz Gomez MJ, De la Pena L, Pastor JM, et al (2001). 25 Hz electromagnetic field exposure has no effect on cell cycle distribution and apoptosis in U-937 and HCA-2/1cch cells. *Bioelectrochemagnetics*, **53**, 137-40.
Salim EI (2006). Molecular alterations in mucin genes expression and histochemical properties of mucosa during rat colon carcinogenesis. *J Egy German Soci Zool*, **31**, 66-78.
Salim EI, Wanibuchi H, Morimura K, et al (2002). Induction of tumors in the colon and liver of the immunodeficient (SCID) mouse by 2-amino-3-methylimidazo[4,5-f]quinoline. Modulation by long-chain fatty acids. *Carcinogenesis*, **23**, 1519-29.
Sasaki O, Kido K, Nagahama S (1999). DNA ploidy, Ki-67 and p53 as indicators of lymph node metastasis in early gastric carcinoma. *Analyt Quant Cytol Histol*, **21**, 85-8.
Saunders R (2005). Static magnetic fields: animal studies. *Prog Biophys Mol Biol*, **87**, 225-39.
Selmaoui B, Lambrozo J, Touitou Y (1997). Endocrine functions in young men exposed for one night to a 50-Hz magnetic field. A circadian study of pituitary, thyroid and adrenocortical hormones. *Life Sci*, **61**, 473-86.
Shivapurkar N, Tang Z, Ferreira A, et al (1994). Sequential analysis of K-ras mutations in aberrant crypt foci and colonic tumors induced by azoxymethane in Fischer-344 rats on high-risk diet. *Carcinogenesis*, **15**, 775-8.
Stavroulakis P (2003): Biological effects of electomagnetic fields. Springer-Verlag Berlin Heidelberg. http://www.springer.de/engine/
Stevens RG (1993). Biologically based epidemiological studies of electric power and cancer. *Environ Health Persp Suppl*, **101**, 93- 100.
Suzuki Y, Ikehata M, Nakamura K, et al (2001). Induction of micronuclei in mice exposed to static magnetic fields. *Mutagenesis*, **16**, 499-501.
Svedenstål BM (1997). Embryonic development and cellular proliferation in mice exposed to Electromagnetic fields. Swedish University of Agricultural Science (publisher). Acta Universitatis Agriculturae Sueciae, 47.
Szuba M (2007). Evaluation of reports on environmental measurements of electromagnetic fields generated by high voltage transmission lines and substations. *Med Pr*, **58**, 169-75.
Takahashi S, Inaguma S, Cho YM, et al (2002). Lack of mutation induction with exposure to 1.5 GHz electromagnetic near fields used for cellular phones in brains of Big Blue Mice. *Cancer Res*, **62**, 1956-60.
Teodori L, Grabarek J, Smolewski P, et al (2002). Exposure of cells to static magnetic field accelerates loss of integrity of plasma membrane during apoptosis. *Cytometry*, **49**, 113-8.
Theriault G, Goldberg M, Miller AB, et al (1994). Cancer risks associated with occupational exposure to magnetic fields among electric utility workers in Ontario and Quebec, Canada, and France: 1970-1989. *Am J Epidemiol*, **139**, 550-72.
Touitou Y, Lambrozo J, Camus F, et al (2003). Magnetic fields and the melatonin hypothesis: a study of workers chronically exposed to 50-Hz magnetic fields. *Am J Physiol*, **284**, R1529-35.
Tuncel H, Shimamoto F, Cagatay P, et al (2003). E-cadherin expression in a MNU-induced colon tumor model in rats which exposed with 50 Hz frequency sinusoidal magnetic field. *Tohoku J Exp Med*, **198**, 245-9.
Tynes T, Andersen A, Langmark F (1992). Incidence of cancer in Norwegian workers potentially exposed to electromagnetic fields. *Am J Epidemiol*, **136**, 81- 8.
Van Wyk JJ, Underwood LE (1978). Growth Hormone, Somatomedins and Growth Failure. *Hospital Practice*, **13**, 57-8.
Xing S, Khanavkar B, Nakhosteen, JA, et al (2005). Predictive value of image cytometry for diagnosis of lung cancer in heavy smokers. *Eur Respir J*, **25**, 956–63.
Zapponi GA, Marcello I (2004). Recent experimental data on Extremely Low Frequency (ELF) magnetic field carcinogenic risk: open questions. *J Exp Clin Cancer Res*, **23**, 353-64.
Zetteberg A, Forsslund G (1991). Ploidy level and tumour progression in prostatic carcinoma. *Acta Oncol*, **30**, 193-9.
