# Supplementary material for: Magnetic Fields and Cancer: Epidemiology, Cellular Biology, and Theranostics
Source: Int J Mol Sci. 2022 Jan 25;23(3):1339. doi: 10.3390/ijms23031339 (PMC8835851; doi:10.3390/ijms23031339)
Supplement: Supplementary file 1 [file ijms-23-01339-s001.zip › Supplementary Data Set S1/MF and Cancer.Data/PDF/0967242665/art00006.pdf]

Comparative Medicine
Copyright 2011
by the American Association for Laboratory Animal Science

Vol 61, No 4
August 2011
Pages 339–345

Effect of Magnetic Fields on Tumor Growth and Viability

Ivan Tatarov,[1] Aruna Panda,[1,*] Daniel Petkov,[7] Krishnan Kolappaswamy,[1,2] Keyata Thompson,[3,5] Anoop Kavirayani,[8] Michael M Lipsky,[1] Edward Elson,[6] Christopher C Davis,[6] Stuart S Martin,[3,5] and Louis J DeTolla[1,2,4]

Breast cancer is the most common nonskin cancer and is the second leading cause of cancer-related deaths in women. Most methods of intervention involve combinations of surgery, chemotherapy, and ionizing radiation. Both chemotherapy and ionizing radiation can be effective against many types of cancer, but they also harm normal tissues. The use of nonionizing, magnetic fields has shown early promise in a number of in vitro and animal studies. Our study tested the effect of varying durations of magnetic exposure on tumor growth and viability in mice injected with breast cancer cells. Cancer cells were labeled through stable expression of firefly luciferase for monitoring of tumor growth and progression by using an in vivo imaging system. We hypothesized that magnetic field exposure would influence tumor growth and progression. Our results showed that exposure of the mice to magnetic fields for 360 min daily for as long as 4 wk suppressed tumor growth. Our study is unique in that it uses an in vivo imaging system to monitor the growth and progression of tumors in real time in individual mice. Our findings support further exploration of the potential of magnetic fields in cancer therapeutics, either as adjunct or primary therapy.

Abbreviation: TUNEL, terminal deoxynucleotidyl transferase (TdT)-mediated dUTP nick end labeling.

In the United States, breast cancer is the most common nonskin cancer and is the second leading cause of cancer-related deaths in women. Significant funds are allocated toward cancer research each year.[1] Most methods of intervention involve combinations of surgery, chemotherapy and/or ionizing radiation. Even when surgery is selected as the primary approach the latter therapies are often employed because the extent of the disease is uncertain or metastatic disease is apparent. Both chemotherapy and ionizing radiation can be effective against many types of cancer but they also harm or kill normal tissues. Because of this limitation, use of doses high enough to kill all cancer cells may be impossible without also producing serious and possibly life-threatening morbidity.

The use of nonionizing, magnetic fields has shown early promise in a number of in vitro and animal studies, and warrants a thoughtful trial, whether it ultimately has a role as adjunctive therapy or even a primary role in certain forms of human cancer.[2,5,10,17-19,21] An added potential advantage is that magnetic fields have the potential to cause less normal tissue damage. Our study tested the effect of magnetic fields on cancer cell growth. We investigated multiple exposure levels using mice that had been injected with mouse breast cancer cells. The cancer cells were labeled bioluminescently via expression of the luciferase enzyme, which could be identified through an in vivo imaging system. The growth and spread of the resulting tumors in inoculated mice were viewed with the in vivo optical imaging system that monitored tumor progression in a single animal in a real-time fashion. Our results may lead to the development of newer and less toxic methods of primary or adjunctive cancer therapies.

Materials and Methods

Mice. The study was carried out at the AAALAC-accredited animal facility in the Program of Comparative Medicine at the University of Maryland School of Medicine (Baltimore, MD). All procedures were carried out according to the guidelines of the *Guide for the Care and Use of Laboratory Animals*[6] and the IACUC policies of the facility. All procedures complied with the CDC/NIH *Biosafety in Microbiologic and Biomedical Laboratories*.[4] Swiss outbred female nude mice (Cr:NIH(S)-*nu/nu*; age, 3 to 4 wk; National Cancer Institute, Frederick, MD) were used in this study. Mice were housed in intraventilated microisolation cages with bedding. Feed (autoclaved) and water (hyperchlorinated) were available ad libitum to all mice throughout the study period. A surveillance program was in place that maintains the mouse facility free of adventitious pathogens.

Cells. The metastatic mouse breast tumor cell line EpH4-MEK-Bcl2[13] was used. Mice were injected via the mammary fat pad route with 1×10^6 cells. Cells were transfected with a luciferase expression vector (pβP2-PolII-luciferase) prior to injection. The cells were grown aseptically under biosafety level 2 conditions and were confirmed to be free of HIV, hepatitis B and C viruses and mouse adventitious pathogens such as mouse rotavirus (epizootic diarrhea of infant mice), mouse hepatitis virus, mouse parvovirus, minute virus of mice, parvovirus nonstructural protein-1, ectromelia virus, Theiler murine encephalomyelitis virus,

Received: 05 Nov 2010. Revision requested: 14 Dec 2010. Accepted: 18 Mar 2011.
[1]Comparative Medicine Program, Department of Pathology, [2]Department of Epidemiology and Public Health, [3]Department of Physiology, and [4]Department of Medicine (Infectious Diseases), School of Medicine, University of Maryland, Baltimore, Maryland; [5]University of Maryland Marlene and Stewart Greenebaum NCI Cancer Center, Baltimore, Maryland; [6]Department of Electrical and Computer Engineering, University of Maryland, College Park, Maryland; [7]Animal Health Unit, University of Calgary, Calgary, Canada; and [8]Department of Histopathology and Microscopic Sciences, The Jackson Laboratory, Bar Harbor, Maine.
[*]Corresponding author. Email: apanda@vetmed.umaryland.edu

lymphocytic choriomeningitis virus, mouse adenovirus, *Mycoplasma*, polyoma virus, reovirus type 3, and Sendai virus.

Experimental design. A total of 21 mice were used in this study. To assess the effect of magnetic field on the general health of the animals, we performed a preliminary study with 9 mice. Mice were divided into 3 groups (3 mice per group) and exposed to 100 mT, 1-Hz, half-sine-wave unipolar magnetic fields. The 3 groups were exposed to the magnetic field directly daily for 60, 180, or 360 min. Mice were observed for any signs of clinical diseases or weight loss. All mice were euthanized at the end of the study and tissues collected for histopathologic analysis. In the next part of the study, mice were divided into 4 groups (3 mice per group). Mice were injected with the mouse breast cancer cell line EpH4-MEK-Bc12 labeled with luciferase (1×10^6 cells suspended in 100 µL sterile PBS). Mice in the negative control group (NCG) were inoculated with the cancer cell line but not exposed to magnetic fields. Mice in the remaining 3 groups were exposed daily to 100-mT, 1-Hz half-sine-wave unipolar magnetic fields for as long as 4 wk. Time of exposure was 60 min (group G60), 180 min (G180), or 360 min (G360). Similar exposure levels had been tolerated well in prior experiments.[5,19] All mice were euthanized at week 4 of the study. All mice were housed in a room that did not contain the magnet source. The treated groups were brought daily to the room containing the magnet for varying periods of time for magnetic exposure and then returned to the animal housing room. The untreated group of mice was not brought into the room containing the magnet. The effect of magnetic fields in inoculated mice was assessed by comparing the exposed groups with the unexposed group.

The exposure system (magnetic device) used a water-cooled Helmholtz coil driven by a controllable high-power alternating-current supply (SDR TH 40–250, Sodilec, Bordeaux, France) whose output waveform could be adjusted by using an external signal generator. The Helmholtz coils had an inner diameter of 152 mm, an outer diameter of 406 mm, and spacing of 83 mm. Each coil had a resistance of 0.42 Ω. With the coils connected in parallel at 20 V, the nominal drive current was close to 100 A and produced a direct-current field of 0.094 T. This instrument was generously lent to us by Professor Bernard Veyret (University of Bordeaux, France). For the exposures reported herein, the power supply was modulated with a square-wave generator and produced a magnetic field waveform that was a unipolar half-cycle sine wave. The half-cycles were 0.5 s long with a repetition frequency of 1 Hz and peak field of 0.1 ± 0.006 T. The magnetic field was measured by using 2 different probes (MAG-03 MC, Bartington, Witney, United Kingdom, and model 5180, FW Bell, Milwaukie, OR). The Bell probe was used to calibrate the Helmholtz coil during direct-current tests, because it could record the large fields produced. The Bartington probe had a much faster response and was used near the magnet, but not in the peak field region, to measure the precise time variation of the field. Progression and spread of tumors in inoculated mice were monitored and measured by using an in vivo imaging system (Xenogen IVIS 200, Alameda, CA). Tumor sizes were measured by using calipers.

Tumor growth progression. Mice were monitored for tumor growth once every 2 to 4 d over the course of 4 wk with whole-body bioluminescence imaging for 2 to 3 min by using the in vivo imaging system. This noninvasive and novel imaging system detected live luciferase-labeled tumor cells, enabling real-time monitoring of tumor growth and spread in the mice. For imaging, mice were sedated with 2% isofluorane in 100% oxygen at 3.5 L/min (for induction) in the anesthesia chamber of the imaging system. Images were taken every 3 min as a sequence of 10 images for every group of mice, 2 times each week for a total of 4 wk. Progression and spread of tumors were evaluated by the average maximal radiance values of tumors from inoculated mice. Tumor sizes were measured by using calipers on a weekly basis.

Statistical analysis. We compared the average maximal radiance values of tumors (measured in photons/s/cm^2/steradian, that is, p/s/cm^2/sr) of all groups of mice by using ANOVA. We used Duncan multiple-range tests to compare differences in the average maximal radiance among groups. All statistical analyses were performed by using SAS 9.1 (Cary, NC). Mean fold increase in luminescence values was compared by using *t* tests. Statistical significance was defined as a P value of less than 0.05.

Euthanasia and pathologic assessment. All mice were euthanized by asphyxiation with an overdose of carbon dioxide gas followed by cervical dislocation on day 28 (endpoint) of the study. Alternative endpoints in this study included animals displaying signs of discomfort such as lethargy, inability to move because of increased sizes or location of tumors, dehydration, inappetence, and severe weight loss or animals that developed ulceration of the tumors or had tumors larger than 2 cm in diameter. Mice that displayed these signs were euthanized before the study endpoint. Tissues such as skin, liver, lung, spleen, and tumors were collected from each animal at euthanasia. Skin, liver, lung, and spleen samples were collected from mice in the preliminary study, which was conducted to assess the effect of magnetic field on the general health of the animals. Paraffin sections of formalin-fixed tumors were stained with hematoxylin and eosin and evaluated by a board-certified veterinary pathologist.

Cell death assay. To check whether the mechanism of cell death in tumors was due to apoptosis, paraffin-embedded tumor sections were analyzed by terminal deoxynucleotidyl transferase (TdT)-mediated dUTP nick end labeling (TUNEL) assays to detect apoptotic cells (performed at Histoserve, Germantown, MD). Briefly, blocks were deparaffinized with xylene and graded ethanol and washed with distilled water. Slides then were treated with proteinase K followed with EDTA, washed with distilled water, and blocked with bovine serum albumin. Slides were incubated with equilibration buffer at 37 °C and washed with 1× SSC buffer. Slides were blocked with bovine serum albumin and underwent antidigoxin (1:1000; catalog no. 1093274, Roche Diagnostics, Indianapolis, IN) treatment. The color was developed with fuchsin, followed by counterstaining with hematoxylin. Slides were dehydrated with graded ethanol and cleared in ClearRite solution (Thermo Fisher Scientific, Kalamazoo, MI). Coverslips were mounted by using Permount (Fisher Scientific, Pittsburg, PA) mounting media.

Results

Gross tumor lesions. Tumor growth and progression in control and study groups of mice were assessed as mean fold increase in luminescence value compared with week 1 (calculated by normalizing average values of maximum radiance). Inoculated mice that were not exposed to the magnetic radiation (NCG group) had very large tumors. NCG mice had a 500-fold increase in tumor growth at week 4 compared with week 1. Tumor growth of mice in the G60 and G180 groups also showed robust increases (900 and 200 times, respectively) at week 4 when compared with week 1

of the study. In contrast, the G360 group showed only a 44-fold increase in their tumor growth at week 4 compared with week 1. In addition, tumors in G360 mice included extensive areas of necrosis (Table1). The progression of tumors in mice belonging to the G60 and G180 groups did not differ from that of untreated (NCG) mice (Figure 1). However, mice that were treated with 360 min of magnetic field showed suppression of tumor growth (Figure 1). Figure 1 also shows a clear trend toward tumor suppression with increasing magnetic field treatment. Figure 2 compares tumor growth and progression in mice in the different groups at week 4. One NCG mouse was euthanized at week 3 of the study as it met its alternative end point for euthanasia. Caliper measurements indicated that NCG mice displayed the largest tumors, followed by the mice in the G360, G60, and G180 groups (data not shown).

Histopathology. The tumors in all groups of mice were poorly differentiated carcinomas with limited mammary differentiation. Most tumors had prominent areas of necrosis with polymorphonuclear leukocyte infiltration. Tumors in the G360 group had more extensive areas of necrosis when compared with the mice in the NCG group (Figure 3). TUNEL assays showed that the mechanism of cell death in some cells in these tumors may have been apoptosis. These cells displayed fragmented nuclei characteristic of programmed cell death (Figure 4). Mice that were not inoculated with cancer cells but exposed to magnetic fields did not display any histopathologic abnormalities in their lung, liver, or skin (mammary gland) tissues (Figure 5). In addition, none of the organs from any of the groups of mice displayed any gross pathology at necropsy.

Discussion

We used T-cell–immunodeficient Swiss outbred nude mice to investigate the effect of magnetic fields in tumor growth and viability. Our data show that mouse breast tumor cells grew more rapidly into grossly visible tumors in the unexposed group of mice when compared with those exposed to magnetic fields. The in vivo imaging system we used detects live tumor cells in the various groups of mice (that is, measures spread and progression of the tumors in a real-time manner). Tumors were largest (caliper measurements) and most rapidly growing (imaging results) in unexposed (NCG group) mice. Although tumor sizes were comparatively larger in mice exposed to magnetic fields for 360 min daily (G360 group) than in those exposed for 60 or 180 min, the G360 mice showed decreases in the rates of tumor growth and progression. In addition, this group of mice had the most extensive areas of necrosis, as evidenced by the histopathology data. Because the imaging system detects only live tumor cells, the bioluminescence values of G360 mice were less than those of the other groups. Tumors in mice belonging to the NCG, G60, and G180 groups showed increases in tumor size and growth when measured over a period of 4 wk. These findings provide insights into the effects of magnetic field exposure on tumor growth and viability.

Although targeted therapies, especially the use of 'designer' drugs, are under intensive investigation, their ultimate efficacy is not yet established.[8,12,16] Previous studies on the tumor-reducing effects of magnetic fields represent a great variety in cell culture systems, animal models (mice), field sources, waveforms, field strengths, and exposure protocols. Many of the cell culture studies of tumor lines report significant cell death after magnetic exposure compared with that of control cultures. With a few exceptions, most in vivo studies have reported a relative reduction in tumor size in tumors exposed to magnetic fields compared with controls.[3,5,9,10,11,17-21] Reduction in tumor size occurs across a broad variety of field strengths, waveforms, exposure durations, animal models, tumor lines, and other procedural aspects. No general theory has accounted for such a ubiquitous response, although a number of biochemical and cellular structural changes[3] have been well documented. One report describes inhibition of tumor cell proliferation in vitro[14] in the presence of a static magnetic field.

Table 1. Normalized average fold measurements of maximal average radiance ($p/s/cm^2/sr$) in mice inoculated with mouse breast cancer cells

Group	Week 1	Week 2	Week 3	Week 4
G60	1.0	5.69	80.58	979.33
G180	1.0	8.16	51.95	236.48
G360	1.0	9.06	29.40	44.02
NCG	1.0	14.25	206.67	523.53

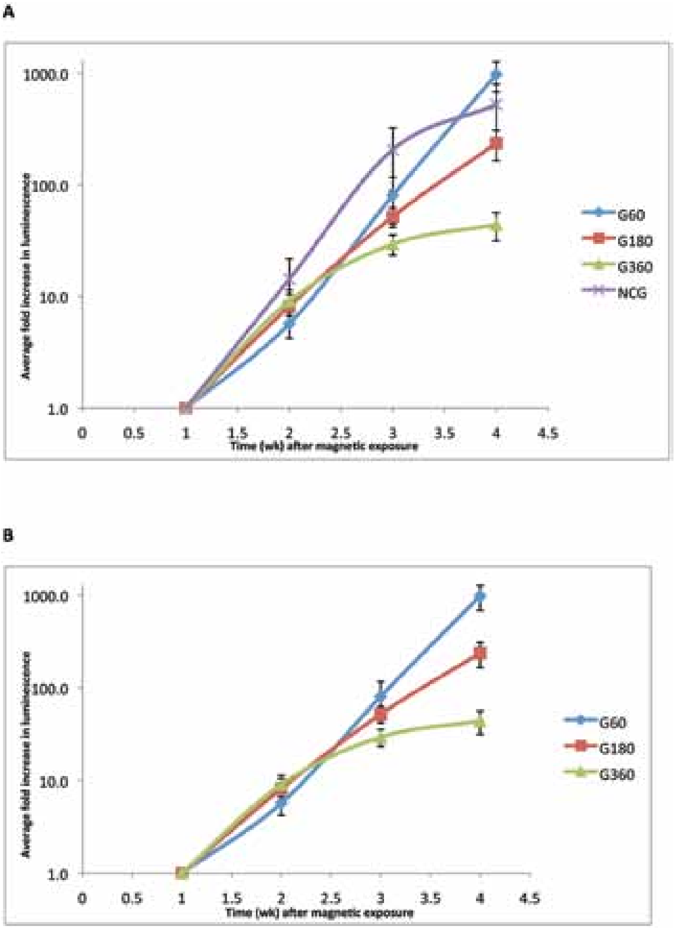

Figure 1. Fold luminescence increase (mean ± SEM compared with week 1 value) in tumors under different magnetic field treatments. Tumors in mice treated for either 60 min (G60) or 180 min (G180) did not differ significantly from those in untreated (NCG) mice. Mice treated for 360 min (G360) showed significant (t test, $P < 0.05$) suppression of tumor growth.

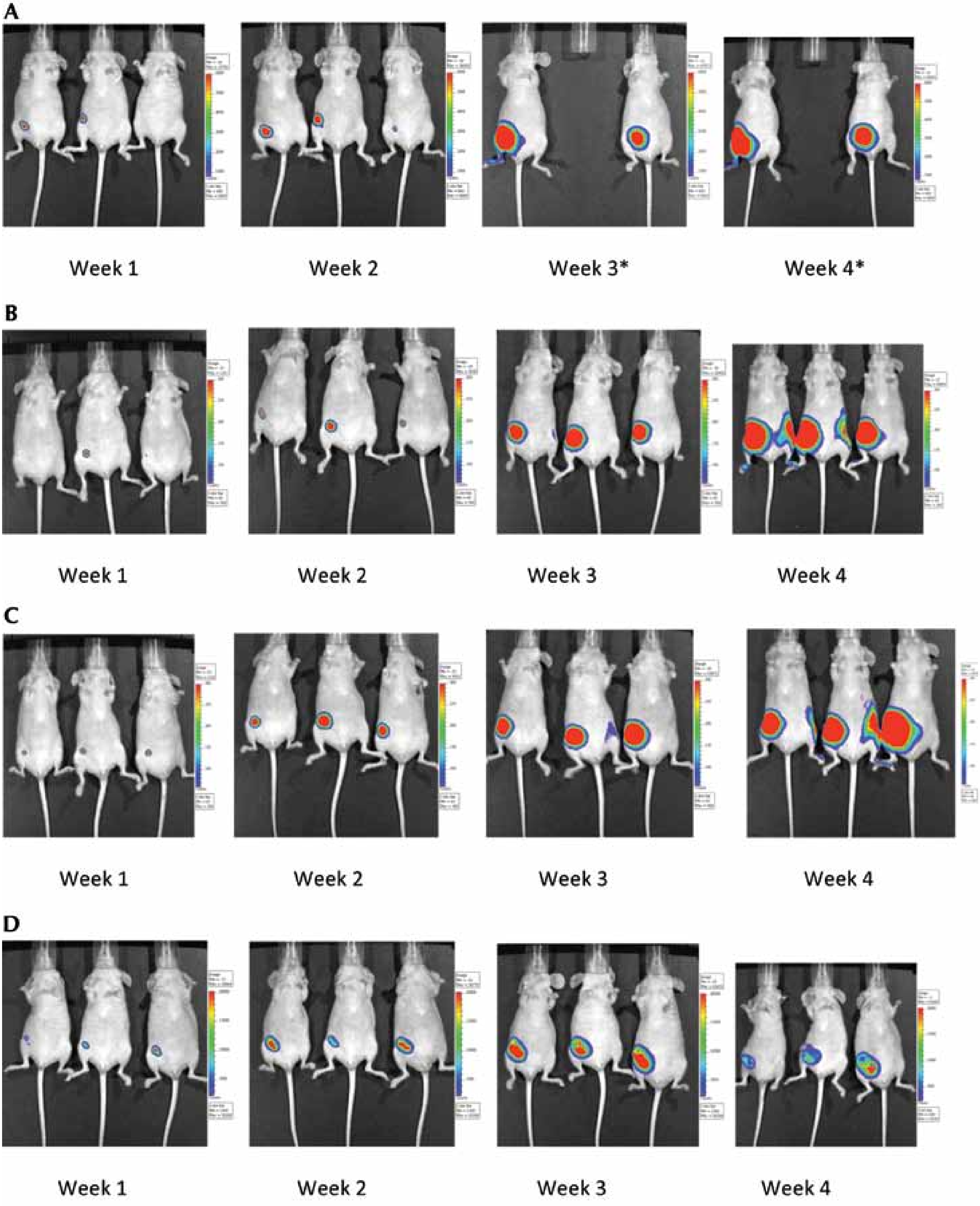
A
Week 1
Week 2
Week 3*
Week 4*
B
Week 1
Week 2
Week 3
Week 4
C
Week 1
Week 2
Week 3
Week 4
D
Week 1
Week 2
Week 3
Week 4

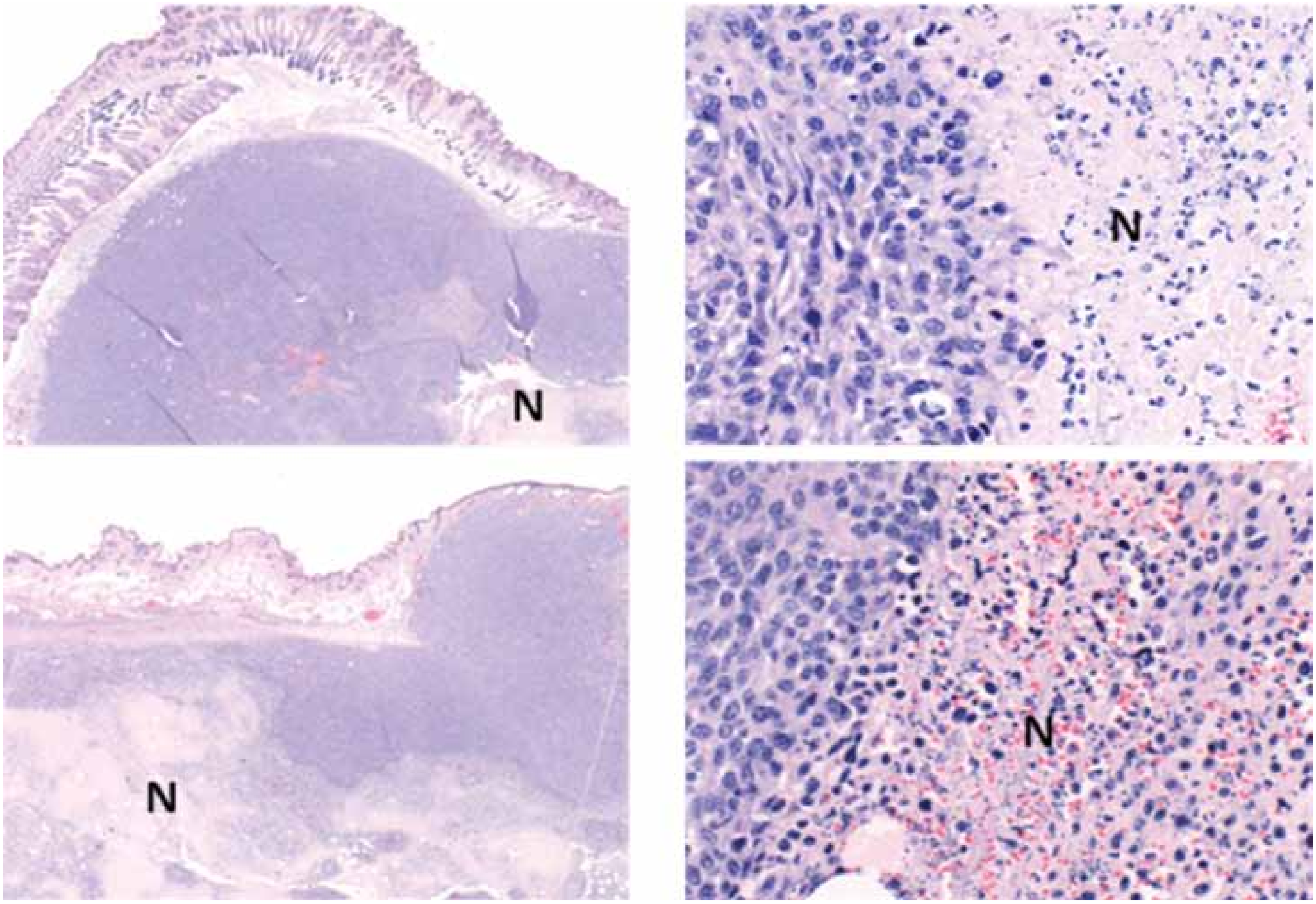

Figure 3. Sections of tumors [magnification, ×20 (left) and ×400 (right)] from mice in the NCG group (top panel) and G360 group (bottom panel) at week 4 of study. Note the extensive areas of necrosis (N) in the G360 group compared with necrotic areas in the tumors of NCG mice.

Because size-reductive effects have been seen with many different waveforms, an important goal should be to find the waveform with optimal activity. A previous study[3] investigated the addition of magnetic fields to ionizing radiation or chemotherapy. In the current study, our general goal was to find an optimal magnetic field exposure regime that delivers the most efficient cancer-cell killing, that is, to evaluate the ultimate potential of electromagnetic fields in the first instance and then to evaluate their role, when designing clinical studies, whether as adjunctive or primary therapy.

A previous study reported that exposure to therapeutic electromagnetic fields significantly reduced tumor growth and extent of tumor vascularization, with a concomitant increase in the extent of tumor necrosis.[19] The authors[19] concluded that this treatment safely reduced growth and vascularization of implanted breast cancers in mice and that therapeutic electromagnetic fields may prove a useful adjuvant to increase the therapeutic index of conventional cancer therapy. The results of our current study are in agreement with this previous study.[19] Compared with our study, the previous study[19] used lower strength field exposures (15 and 20 mT) and much shorter exposure periods (10 min per day). The optimal exposure regime remains unknown. We believe that weaker and briefer exposures to magnetic fields are less likely to have an effect, compared with longer and stronger exposures. Another study[21] investigated the effect of magnetic radiation on tumors in mice injected with sarcoma ascites cells. Sarcomas formed 4 to 5 d after inoculation. Mice were exposed to pulsed-gradient magnetic fields of 0.6 to 2.0 T with a gradient of 10 to 200 T/m, pulse width of 20 to 200 ms, and frequency of 0.16 to 1.34 Hz for 15 min daily for 28 d,[21] at which time the mice were euthanized. The mean tumor weight of treated animals was 1.40 ± 0.81 g compared with 2.45 ± 0.95 for control animals, a statistically significant difference. Microscopy showed extensive necrosis in tumor sections and associated evidence of apoptosis in samples from treated animals.

Figure 2. Tumor growth and progression in mice inoculated with mouse cancer cells and (A) not exposed to magnetic radiation (NCG group; one mouse in this group had to be euthanized at week 3 and therefore was not included in the rest of the study) or exposed to (B) 60 min (G60 group), (C) 180 min (G180 group), or (D) 360 min (G360 group) of magnetic radiation daily for a period of 4 wk. Pictures were taken at weekly intervals by using the in vivo imaging system.

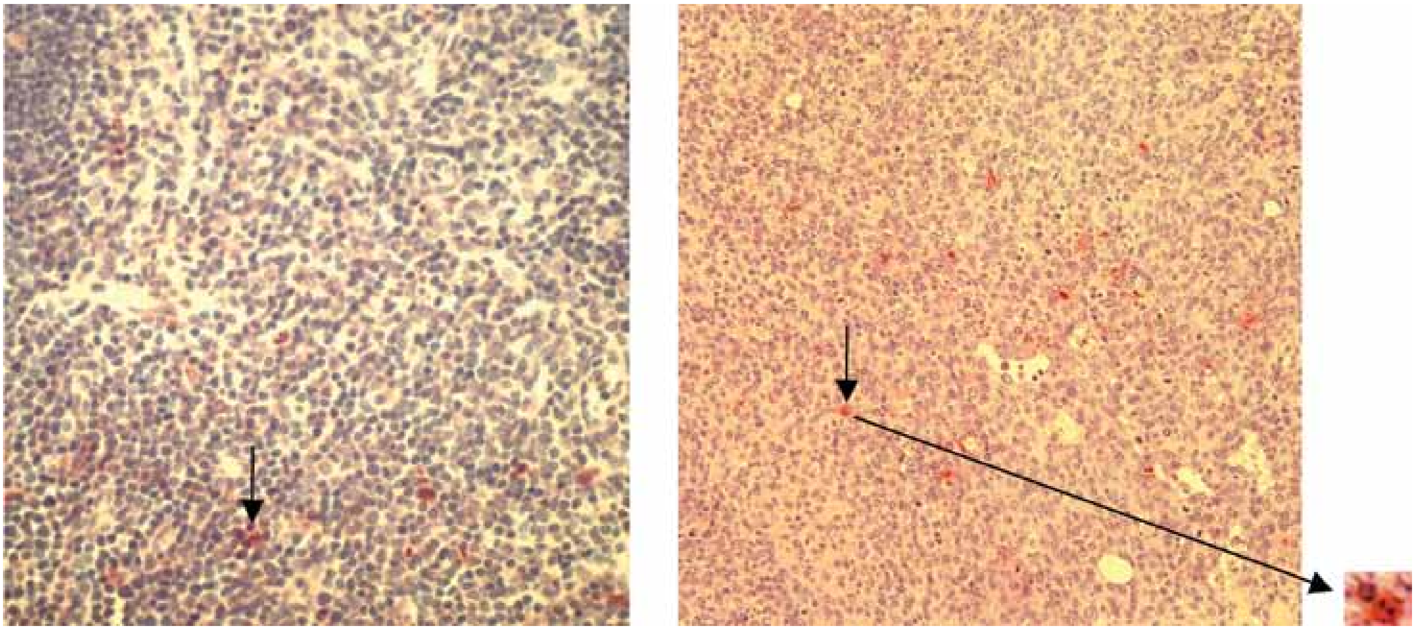

Figure 4. Section of tumor from a G360 mouse (right panel) at week 4. The TUNEL assay was used to reveal fragmented nuclei (stained red and marked with black arrows) in apoptotic cells. A positive control (left panel) showing apoptotic cells is displayed for comparison. Magnification, ×200.

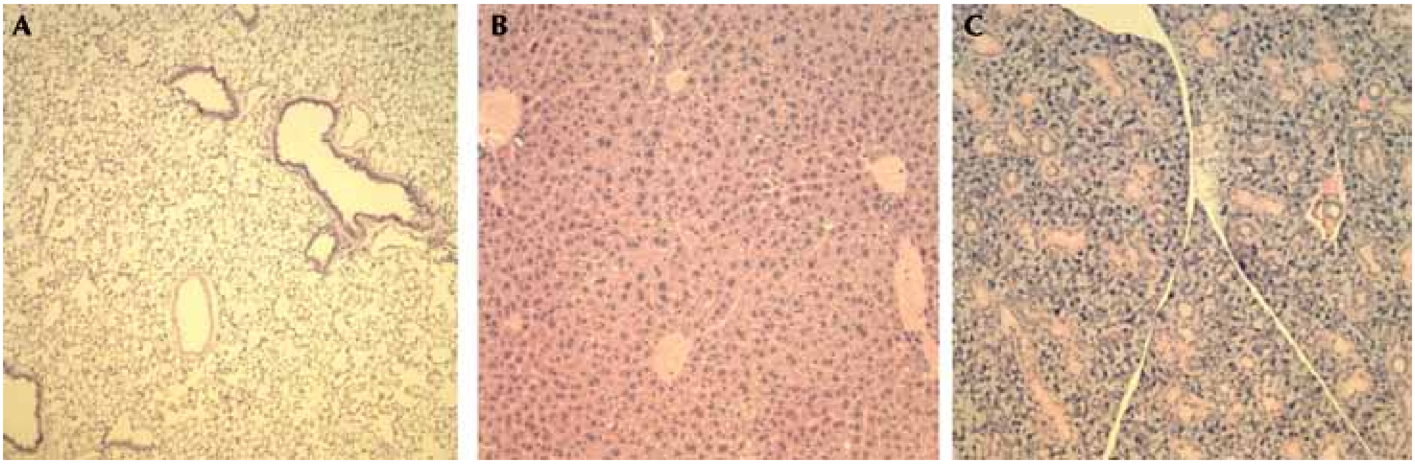

Figure 5. (A) Lung, (B) liver, and (C) mammary gland tissue from a mouse exposed to magnetic fields for 60 min daily. No abnormalities were detected. Hematoxylin and eosin stain; magnification, ×200.

The numerous differences in waveform, intensity, duration of exposure, and response evaluation prevents direct comparison among the results of the current and previous studies.[3,5,9,10,11,17-21] Regardless, in each case the outcome appeared to be relative reduction in tumor size, probably through cytoreduction, and the appearance of tumor necrosis. On the basis of these and other associated studies, apoptosis appears to play a major role, and an antiangiogenic property of the fields may strongly be suspected. To investigate the possible mechanisms of cell death in the tumors, including apoptosis, we performed TUNEL assays on tumor sections from our mice. Our results indicate that apoptosis may have played a role in cell death in the tumors (Figure 4). A previous study[3] indicated that magnetic fields may suppress tumor growth by suppressing angiogenesis and blocking blood supply to the tumor tissues. We speculate that the cell death and necrosis seen in the tumors of our mice may be due to the combined effect of suppression of angiogenesis, blood supply blockage to tumor tissues, and cell death due to apoptosis.

Previous studies[3,4] report no morbidity or mortality or abnormalities in normal tissues due to exposure to magnetic fields. In our study, mice exposed to magnetic fields for 60, 180, or 360 min daily did not display any signs of clinical disease and/or weight loss (data not shown). Tissues collected from these mice at necropsy did not display any gross or histopathologic abnormalities (Figure 5). These findings support the likelihood that magnetic field exposure is not harmful to the general health of mice.

In sum, we report that direct exposure of mice to magnetic fields reduced tumor growth and progression. Mice exposed to magnetic fields for 360 min daily for as long as 4 wk showed extensive areas of necrosis in their tumors. Mice in the unexposed control group developed large tumors. In addition, the time of exposure of these tumors to magnetic fields is critical. Mice exposed

for shorter durations (that is, 60 or 180 min daily for wk) did not show a reduction in tumor size or growth. The main weakness of our study was the small number of animals used in each group. Our goal was to perform a preliminary study to assess the effects of magnetic fields in tumor growth and viability. In addition, we wanted to optimize the time and duration of the magnetic field exposure. Previous reports[2,3,7,15,17] lead us to hypothesize that much longer exposure times for multiple months should be attempted.

Our findings, along with the reports of others, support further exploration of the potential of magnetic fields in cancer therapeutics, either as adjunctive therapy or, in some as yet to be determined specific cases, as primary therapy. In particular, prolonged exposure times and different field strengths and waveforms should be explored.

Acknowledgments

We thank Professor Bernard Veyret at the University of Bordeaux, France, who lent us the magnet used for this study. We also thank Rebecca Yerkey, Elisa Luna, Dawn McKenna, Theresa Alexander, and Leisha Alexander for providing excellent veterinary technical support.

References

1. **American Cancer Society.** Breast cancer facts and figures. 2007–2008. Atlanta (GA): American Cancer Society.
2. **Cameron IL, Short NJ, Markov MS.** 2007. Safe alternative cancer therapy using electromagnetic fields. Environmentalist **27:**453–456.
3. **Cameron IL, Sun LZ, Short N, Hardman WE, Williams CD.** 2005. Therapeutic electromagnetic field (TEMF) and γ irradiation on human breast cancer xenograft growth, angiogenesis, and metastasis. Cancer Cell Int **5:**23–36.
4. **Centers for Disease Control and Prevention and National Institutes of Health.** [Internet]. 2007. Biosafety in microbiological and biomedical laboratories (BMBL), 5th ed. [Cited March 2011]. Available at http://www.cdc.gov/OD/ohs/biosfty/bmbl5/bmbl5toc.htm.
5. **de Seze R, Tuffet S, Moreau JM, Veyret B.** 2000. Effects of 100-mT time-varying magnetic fields on the growth of tumors in mice. Bioelectromagnetics **21:**107–111.
6. **Institute of Laboratory Animal Resources.** 1996. Guide for the care and use of laboratory animals. Washington (DC): National Academies Press.
7. **Kim SH, Lee HJ, Choi SY, Gimm YM, Pack JK, Choi HD, Lee YS.** 2006. Toxicity bioassay in Sprague–Dawley rats exposed to 20-kHz triangular magnetic field for 90 d. Bioelectromagnetics **27:**105–111.
8. **Koh EK, Ryu BK, Jeong DY, Bang IS, Nam MH, Chae KS.** 2008. A 60-Hz sinusoidal magnetic field induces apoptosis of prostate cancer cells through reactive oxygen species. Int J Radiat Biol **84:**945–955.
9. **Liang Y, Hannan CJ Jr, Chang BK, Schoenlein PV.** 1997. Enhanced potency of daunorubicin against multidrug resistant subline KB-ChR-8-5-11 by a pulsed magnetic field. Anticancer Res **17:**2083–2088.
10. **Novikov VV, Novikov GV, Fesenko EE.** 2009. Effect of weak combined static and extremely low-frequency alternating magnetic fields on tumor growth in mice inoculated with the Ehrlich ascites carcinoma. Bioelectromagnetics **30:**343–351.
11. **Omote Y, Howokawa M, Komatsumoto M, Namieno T, Nakajima S, Kubo Y, Kobayashi H.** 1990. Treatment of experimental tumors with a combination of a pulsing magnetic field and an antitumor drug. Jpn J Cancer Res **81:**956–961.
12. **Ozpolat B, Sood AK, Lopez-Berestein G.** 2010. Nanomedicine based approaches for the delivery of siRNA in cancer. J Intern Med **267:**44–53.
13. **Pinkas J, Martin SS, Leder P.** 2004. Bcl2-mediated cell survival promotes metastasis of EpH4 βMEKDD mammary epithelial cells. Mol Cancer Res **2:**551–556.
14. **Raylman RR, Clavo AC, Wahl RL.** 1996. Exposure to strong static magnetic field slows the growth of human cancer cells in vitro. Bioelectromagnetics **17:**358–363.
15. **Ronchetto F, Barone D, Cintorino M, Berardelli M, Lissolo S, Orlassino R, Ossola P, Tofani S.** 2004. Extremely low-frequency-modulated static magnetic fields to treat cancer: a pilot study on patients with advanced neoplasm to assess safety and acute toxicity. Bioelectromagnetics **25:**563–571.
16. **Ruoslahti E, Bhatia SN, Sailor MJ.** 2010. Targeting of drugs and nanoparticles to tumors. J Cell Biol **188:**759–768.
17. **Tofani S, Barone D, Cintorino M, de Santi MM, Ferrara A, Orlassino R, Ossola P, Peroglio F, Rolfo K, Ronchetto F.** 2001. Static and ELF magnetic fields induce tumor growth inhibition and apoptosis. Bioelectromagnetics **22:**419–428.
18. **Tofani S, Cintorino M, Barone D, Berardelli M, De Santi MM, Ferrara A, Orlassino R, Ossola P, Rolfo K, Ronchetto F, Tripodi SA, Tosi P.** 2002. Increased mouse survival, tumor growth inhibition, and decreased immunoreactive p53 after exposure to magnetic fields. Bioelectromagnetics **23:**230–238
19. **Williams CD, Markov MS, Hardman WE, Cameron IL.** 2001. Therapeutic electromagnetic field effects on angiogenesis and tumor growth. Anticancer Res **21:**3887–3892.
20. **Yamaguchi S, Ogiue-Ileda M, Sedino M, Ueno S.** 2006. Effects of pulsed magnetic stimulation on tumor development and immune functions in mice. Bioelectromagnetics **27:**64–72.
21. **Zhang X, Zhang H, Zheng C, Li C, Zhang X, Xiong S.** 2002. Extremely low frequency (ELF) pulsed-gradient magnetic fields inhibit malignant tumor growth at different biological levels. Cell Biol Int **26:**599–603.
