# Supplementary material for: Magnetic Fields and Cancer: Epidemiology, Cellular Biology, and Theranostics
Source: Int J Mol Sci. 2022 Jan 25;23(3):1339. doi: 10.3390/ijms23031339 (PMC8835851; doi:10.3390/ijms23031339)
Supplement: Supplementary file 1 [file ijms-23-01339-s001.zip › Supplementary Data Set S1/MF and Cancer.Data/PDF/1103778740/bem.20252.pdf]

Bioelectromagnetics 27:560–577 (2006)

Biological Effects of 6 mT Static Magnetic Fields: A Comparative Study in Different Cell Types†

Bernadette Tenuzzo, Alfonsina Chionna, Elisa Panzarini, Remigio Lanubile, Patrizia Tarantino, Bruno Di Jeso, Majdi Dwikat, and Luciana Dini*

Department of Biological and Environmental Science and Technology, University of Lecce, Lecce, Italy

The present work was a comparative study of the bio-effects induced by exposure to 6 mT static magnetic field (MF) on several primary cultures and cell lines. Particular attention was dedicated to apoptosis. Cell viability, proliferation, intracellular Ca^{2+} concentration and morphology were also examined. Primary cultures of human lymphocytes, mice thymocytes and cultures of 3DO, U937, HeLa, HepG2 and FRTL-5 cells were grown in the presence of 6 mT static MF and different apoptosis-inducing agents (cycloheximide, H_2O_2, puromycin, heat shock, etoposide). Biological effects of static MF exposure were found in all the different cells examined. They were cell type-dependent but apoptotic inducer-independent. A common effect of the exposure to static MF was the promotion of apoptosis and mitosis, but not of necrosis or modifications of the cell shape. Increase of the intracellular levels of Ca^{2+} ions were also observed. When pro-apoptotic drugs were combined with static MF, the majority of cell types rescued from apoptosis. To the contrary, apoptosis of 3DO cells was significantly increased under simultaneous exposure to static MF and incubation with pro-apoptotic drugs. From these data we conclude that 6 mT static MF exposure interfered with apoptosis in a cell type- and exposure time-dependent manner, while the effects of static MF exposure on the apoptotic program were independent of the drugs used. Bioelectromagnetics 27:560–577, 2006. © 2006 Wiley-Liss, Inc.

Key words: thymocytes; lymphocytes; apoptosis; combined effects; necrosis; Ca^{2+} concentration

INTRODUCTION

One possible mechanism for induction of cancer is the disruption of the equilibrium between the processes of proliferation and apoptosis [Alenzi, 2004; Liu et al., 2004; Radeff-Huang et al., 2004]. Apoptosis is part of normal cell physiology, as are proliferation and differentiation, and is a major component of both normal cell development and disease [see for review Guimaraes and Linden, 2004; Moreira and Barcinski, 2004; Dini, 2005]. The morphological phenotype of apoptosis is characterised by rapid condensation of the cytoplasm and nuclear chromatin, resulting in DNA fragmentation and membrane blebbing followed by fragmentation of the cells into apoptotic bodies, made up of condensed cytoplasm, nuclear material and/or whole organelles surrounded by intact plasma membranes [Dini et al., 1996]. The increasing production of electric (E) and magnetic fields (MFs), due to the use of electronic devices, is prompting studies of the effects of (E) MFs on living organisms, aimed to protect human health against their possible unfavourable effects [Wartenberg, 2001; Karasek and Lerchl, 2002].

Apoptosis, spontaneous and induced, has been reported to be influenced by static MF [Fanelli et al., 1999; Teodori et al., 2002a; Chionna et al., 2003, 2005; Dini and Abbro, 2005]. The influence of static MFs on biological system has been studied for many years in

Abbreviations: static MF, static magnetic field; EMF, electromagnetic field; T, tesla; mT, millitesla; CHX, cycloheximide; PMC, puromycin; HS, heat shock; LM, light microscopy; TEM, electron microscopy; SEM, scanning electron microscopy.

†This work is dedicated to the memory of Luigi Abbro.

Grant sponsor: MIUR ex60%.

*Correspondence to: Prof. Luciana Dini, Department of Biological and Environmental Science and Technology, University of Lecce, Via per Monteroni 73100 Lecce, Italy.
E-mail: luciana.dini@unile.it

Received for review 24 March 2005; Final revision received 28 March 2006

DOI 10.1002/bem.20252
Published online 24 May 2006 in Wiley InterScience (www.interscience.wiley.com).

© 2006 Wiley-Liss, Inc.

several biosystems, often with controversial results [Rosen, 2003]. To date working environment or acute exposure to static MF at flux densities below 2 T were not unequivocally found to have adverse health consequences. However, at the current state of knowledge, the biological effects, both in vivo and in vitro, connected to MFs are not univocally interpreted. A deeper knowledge of the negative and/or positive effects of MFs on cellular processes, including apoptosis is, therefore, important.

The unbalance of the apoptotic process could be linked to Ca^{2+} fluxes that are, in turn, dependent on the effect on the plasma membrane exerted by static MF. In fact, by virtue of its bioelectrical properties, plasma membrane is the site where MFs exert their primary effects [Rosen, 1993, 2003]. Therefore, plasma membrane structural and biophysical changes would affect, in turn, receptor binding or activation and thereby affect cell function in general [Dini and Abbro, 2005]. In particular, it has been suggested that static MFs alter the function of the organism's transmembrane calcium flux in diverse experimental models [Rosen and Rosen, 1990; Fanelli et al., 1999]. Ca^{2+} ions as mediators of intracellular signalling are crucial for the development of apoptosis: more frequently, an increase of $[Ca^{2+}]i$ due to emptying of intracellular $[Ca^{2+}]i$ stores and to $[Ca^{2+}]i$ influx from the extracellular medium, committees to apoptosis, independent of the apoptotic stimulus [Bian et al., 1997]. However, a general rule cannot be drawn since $[Ca^{2+}]i$ increase exerts different effects in different cell systems [Magnelli et al., 1994; Teodori et al., 2002a,b]. Other possible effects of static MFs leading to perturbation of the apoptotic rate [Fanelli et al., 1999; Jajte, 2000; Chionna et al., 2003], such as an alteration of the gene pattern expression (personal communication) or increment of oxygen free radicals cannot be excluded.

The aim of the present work has been the comparative study of the effect of static MF of 6 mT of intensity on various cell types, differing in embryonic origin and type of culture, that is primary culture or cell line, in suspension or in adhesion growth. Apoptosis (spontaneous and drug-induced), cell viability, proliferation, Ca^{2+} concentration and morphology were examined during the exposure of cells to 6 mT static MF for up to 48 h in the absence or in the presence of apoptosis-inducing drugs.

MATERIALS AND METHODS

Cell Cultures

Different cell types in culture (primary cells and cell lines) were used: isolated human lymphocytes, thymocytes, FTRL-5, Hep G2, U937, HeLa and 3DO cells.

Human isolated lymphocytes. Peripheral blood mononuclear cells were obtained after Ficoll gradient separation of buffy coats from blood donations of non-smoking healthy males, aged 25–45 years. Donors gave their consent for the experimental use of their blood cells. Lymphocytes were separated from monocytes by double adherence to plastic; they were over 95% pure as judged by morphological criteria. During and after the treatments, they were maintained at a cell density of 1×10^6 cells/ml in complete culture medium (RPMI 1640) at 37 °C supplemented with 10% inactivated fetal calf serum (FCS), L-glutamine (2 mM), 100 IU/ml penicillin and streptomycin in a humidified atmosphere of 5% CO_2; cells were used on the first day of explant.

Thymocytes. Thymocyte suspensions were prepared by gently pressing the individual thymus, explanted from male Swiss mice (weighting 25–30 g, fed with standard laboratory diet and maintained with a normal light/dark cycle) through a number 26 gauge steel wire mesh into cold RPMI 1640 medium, which was supplemented with 10% FCS, L-glutamine (2 mM), 2×10^{-2} M HEPES and 50 µg gentamicin sulfate/ml. The cells were washed twice with RPMI 1640 medium and then resuspended at the concentration of 10^6 cells/ml in the same culture medium.

FRTL-5. FRTL-5 cells (ATCC CRL 8305) are a continuous, cloned line of thyroid differentiated cells [Ambesi-Impiombato and Villone, 1987, US patent no. 4608341]. FRTL-5 cells were grown in Coon's modified Ham's F-12 medium (Sigma, St. Louis, MO, USA) supplemented with 5% calf serum (Sigma) and a mixture of hormones and growth factors (insulin, 1 µg/ml; TSH, 1 mIU/ml; glycyl-histidyl-L-lysine, 10 ng/ml; human transferrin, 5 µg/ml; cortisone, 1×10^{-8} M; somatostatin, 10 ng/ml) (Sigma) [Ambesi-Impiombato and Villone, 1987]. These had a doubling time of 30–40 h and remained diploid and differentiated.

HepG2 cells. The HepG2 cells, hepatic transformed cell-line were cultured in DMEM medium supplemented with 10% fetal bovine serum, L-glutamine (2 mM), penicillin and streptomycin solution 100 IU/ml and nystatin (antimycotic solution) 10 000 U/ml. The cells were incubated at 37 °C in a humidified atmosphere of 5% CO_2. Stock cultures of HepG2 cells were maintained in 25 cm^2 flasks, and the culture medium was changed every 2 days, since cells double their number every 2 days. When the cells reached confluence, they were detached from the flask with 0.25% of trypsin plus

0.02% EDTA in NaCl normal saline for 5–7 min at 37 °C and were diluted with fresh medium and then centrifuged at 200*g* for 10 min.

The cells were seeded at a density of 3×10^5 cells per well in a 24-well plates (containing the prepared circular cover slip) for Scanning Electron Microscopy (SEM) preparation, and at a density of 15×10^5 cells in each 75 cm^2 flask for TEM examination. The concentration of cells was determined by using the Bürker's chamber.

HeLa cells. HeLa cells (ATCC, Rockville, MD, USA) were cultured at a density of 5×10^5 cells in 75 cm^2 flasks in Dulbecco's minimal essential medium supplemented with 10% fetal bovine serum, L-glutamine (2 mM), 100 IU/ml of penicillin and streptomycin in 5% CO_2 humidified atmosphere at 37 °C. Every 12–24 h the cell number doubled. Twenty-four hours before the experiments, cells were seeded on coverslips in 6-well plates for fluorescence imaging, or in 75 cm^2 flask for electron microscopy.

U937 cells and 3DO cells. U937 monoblastic cells and T hybridoma 3DO cells grow in suspension. They were kept in a log phase (these cells double their number every 24–48 h) in RPMI 1640 medium at 37 °C supplemented with 10% inactivated FCS, L-glutamine (2 mM), 10 000 IU/ml nystatin, 100 IU/ml penicillin and streptomycin in a humidified atmosphere of 5% CO_2 at 37 °C; cells were used at a density of 1×10^6 cells/ml.

Magnetic Field Application

Static MF was produced by Neodymium magnetic disks (10 mm in diameter and 5 mm in height) of known intensity supplied by Calamit Ltd. (Milano, Italy) placed under the culture Petri dishes. The intensity of the field generated by the magnet was checked by means of a gaussmeter with a range of operating temperature of 0–50 °C and an accuracy (at 20 °C) of ±1% (Hall-effect gaussmeter, GM04 Hirst Magnetic Instruments Ltd., UK). Field intensities were measured in three different zones of the dish bottom [Chionna et al., 2005]. In the zone corresponding to the area of the magnetic disk, the field intensity was 6.00 ± 0.1 mT. The zone corresponding to the area from 5 to 100 mm from the centre of the dish, had intensity of 5.90 ± 0.06 mT The peripheral part of the culture dish, corresponding to area 100–155 mm from the centre of the dish, showed a field intensity of 5.9 ± 0.1 mT. A detailed scheme is reported in Chionna et al. [2005].

For monolayer-growing cells, field intensity of 6 mT is obtained on the bottom of the culture dish at 2.5 cm from the magnet. This distance was obtained by interposing between the magnetic disk and the Petri dish two disks of the same diameter as the culture dish, one iron disk (in order to minimise the differences in the field intensity across the whole bottom of the dish) and one of inert material (Teflon). For cells growing in suspension, the height of the inert material was calculated considering that the volume of the cell suspensions is 3 ml and it occupies 6 mm of the height of the capsule.

Static MF was applied continuously for up to 24 or 48 h, unless otherwise specified. During the 24 or 48 h of experiment no increase in temperature was observed. Exposures were carried out in a blind manner. Simultaneous experiments omitting the magnets were performed as controls. Dishes of cells were always placed on the same two shelves in a tissue culture incubator where the ambient 50 Hz MF inside the incubator was 0.95/0.62 μT (heater on/off) and static magnetic flux densities were 5.5 μT (vertical component). In laboratory areas between incubators, worktops and tissue culture hood MFs measured 0.08–0.14 μT (50 Hz). In the room the background flux density was 10 μT (static) and the local geomagnetic field was approximately 43 μT.

Induction of Apoptosis

Apoptosis of cells was induced with different inducers: cycloheximide (CHX), 10^{-2} M, for up to 24 h; H_2O_2, 10^{-3} M, for 1 or 4 h, followed by 1 h recovery in fresh medium; puromycin (PMC), 10^{-6} M, for up to 24 h; heat shock (HS), 43 °C for 1 h followed by 1 h recovery in fresh medium.

Evaluation of Viability, Proliferation and Apoptosis

The evaluation of the percentage of viable, proliferating and apoptotic cell fractions was performed with different techniques. Cell viability was assessed by membrane impermeably to trypan blue and MT test. Apoptosis was investigated by using light microscopy of haematoxylin/eosin stained cells, Hoechst-33342 labelled cells, DNA laddering and flow cytometry.

MTT assay. 3-(4,5-dimethylthiazol-2-yl)-2,5-diphenyltetrasolium bromide (MTT) assay (MTT 98% Sigma–Aldrich, St. Louis, MO, USA) that is a method of elucidating cytotoxicity, was performed according to the modified MTT method described by Sladowski et al. [1993]. Briefly, at fixed recovery times following the RBAc incubation and irradiation, the cells were incubated with 1 mg/ml culture medium (EMEM) of MTT for 2 h; they were then washed three times with PBS. Surviving cell number was determined indirectly by MTT dye reduction. The amount of MTT-formazan

produced is directly associated with cell vitality and it can be determined spectrophotometrically (DU 640 B, Beckman Coulter, Fullerton, CA, USA) at 570 nm after solubilisation of crystal in 2 ml of DMSO.

Cell growth rate. Cells were cultured in 6-well plates and the growth rate was assayed using a Cell Counting kit-1 (Dojindo Laboratories, Tokyo, Japan), based on the conversion of 2-(4-iodophenyl)-3-(4-nitrophenyl)-5-(2,4-disulfophenyl)-2H-tetrazolium monosodium salt (WST-1) into WST-1 formazan by succinate-tetrazolium reductase. This enzyme is a part of the mitochondrial respiratory chain and is active in viable cells. All manipulations were performed according to the manufacturer's instructions. Reaction was stopped by the addition of 10 µl of 0.1 N HCl to each incubation well. Measurements were taken using a Jasco FP-750 spectrofluorometer at 450 nm.

Apoptosis. Apoptosis was estimated by morphological analysis and flow cytometry and further characterised by DNA fragmentation. Light microscopic analysis of apoptosis was done on Hoechst-33342 or haematoxylin/eosin stained cells on slides. In monolayer growing cells, the analysis of apoptotic fraction has been done on two subset population: substrate attached cells and detached cells. The fraction of cells with fragmented, crescent-shaped or shrunken nuclei, all features of apoptotic morphologies, were evaluated by counting at least 500 cells in at least five randomly selected fields. The number of apoptotic cells was expressed as number per microscopic field 40×. The percentage of apoptotic cell fractions was also performed by flow cytometry with EPICS XL flow cytometer (Coulter Electronic, Inc. Hialegh, FL, USA) equipped with a 5 W argon laser having a 488 nm excitation wavelength. The fixed cells were stained with propidium iodide (10 µg/ml) in phosphate buffered saline containing 40 U/ml RNase and 0.5% Tween-20. The 635 nm emission wavelength was monitored for propidium iodide emission. Flow cytometry of iodide propidium stained cells permits to analyse the percentage of cells according to the amount of DNA per event detected by the flow cytometer. Apoptotic cells are characterised by a low amount of DNA (sub G1 peak) and their percentage can be determined by the cytograms. For each of the flow cytometry analysis at least 10 000 events were calculated.

To assess DNA fragmentation, a total of 10^6 cells was lysed in a buffer containing 10^{-2} M EDTA, 10^{-1} M mM Tris, pH 8.0, 0.5% sodium lauroyl sarkosine, 200 µg/ml proteinase K. Nucleic acids were extracted by phenol–chloroform: isoamylic alocohol (24:1), ethanol precipitated, and incubated in 100 µg/ml RNase A for 60 min at 37 °C. The purified DNA was loaded on a 1.5% agarose gel in TAE buffer, stained with 10 µg/ml ethydium bromide, and visualised on a 254 nm UV transilluminator.

Haematoxylin-eosin slides were also examined for the scoring of cells in mitosis and for the scoring of the morphologies (normal or altered shape). The number of mitotic and the number of cells with normal or altered morphologies was randomly counted in 50–100 high-power microscopic fields (40×). Approximately 300 nuclei per slides were counted.

Transmission and Scanning Electron Microscopy

Ultrastructure of cells was obtained by transmission (TEM) and SEM. Cells were processed for conventional electron microscopy following standardised procedures. Cells were fixed with 2.5% glutaraldehyde in cacodylate buffer, pH 7.4, for 1 h at ice temperature and postfixed with 1% OsO_4 in the same buffer; afterwards samples were dehydrated, embedded in Spurr resin and examined under a Zeiss 910 TEM electron microscope operating at 80 kV. For SEM observations, cells, after fixation and aceton dehydratation, were further dehydrated by using the Critical Point Dryer 020 Balzer. For the final preparation steps samples were gold coated by using a Sputter Coated 040 Balzer. Cells were examined under a Philips XL50 scanning microscope operating at 20 kV.

Measurements of Ca^{2+} Levels

Cells (5×10^7 cells at a concentration of 1×10^6/ml) were washed twice with loading buffer (120 mM: NaCl, 5.4 mM: KCl, 4.2 mM: $NaHCO_3$, 1.2 mM: KH_2PO_4, 1.3 mM: $CaCl_2$, 1.3 mM: $MgSO_4$, 20 mM: Hepes, 15 mM: glucose, 2% BSA equilibrated with CO_2), resuspended at a final concentration of 2×10^7 cells/ml and then loaded with 4 µM fura-2 acetoxymethylesther (AM) for 30 min at room temperature. After the dye loading procedure, cells were washed twice with the same loading buffer and then resuspended in fresh loading buffer at the final concentration of 3×10^6 cells/ml. Cells were stored at room temperature until use and pre-warmed at 37 °C for 2 min before measurements. The fluorescence of fura-2 was measured using a Jasco FP-750 spectrofluorometer, equipped with an electronic stirring system and a thermostabilised (37 °C) cuvette holder, and monitored by a personal computer running the Jasco Spectra Manager software for Windows 95 (Jasco Europe s.r.l., Lecco, Italy). The excitation wavelengths are 340 and 380 nm and the emission wavelength is 510 nm; the slit widths were set to 10 nm. Two millilitres of cell suspension at the final concentration of 3×10^6 cells/ml, was placed

in a glass cuvette, and fluorescence values were converted to $[Ca^{2+}]i$ values according to Grynkiewicz et al. [1995].

Statistical Analysis

Statistical analyses were performed using one-way analysis of variance (ANOVA) with 95% confidence limits. Data are presented in text and figures as mean ± SE. Tukey HSD test for post hoc test was applied when necessary (i.e., Fig. 9a–d).

RESULTS

Each different cell type, normal primary cultures (thymocytes and lymphocytes), normal stabilised cell line (FRTL-5), and transformed cell lines (3DO, HepG2, HeLa and U937), was analysed for viability, apoptosis, proliferation and necrosis after exposure to 6 mT static MFs alone or to 6 mT static MFs and apoptotic inducers simultaneously. Cells were monitored by Hoechst 33342 labelling, MT test, DNA laddering, flow cytometry analysis, morphology and spectrofluorimetric measurements of $[Ca^{2+}]i$. The results are summarised in Tables 1–3 and Figures 1 and 2. The data obtained by the different methods were similar. In general, exposure to 6 mT static MF modified to different extent cell shape, rate of apoptosis and mitosis but not necrosis, and increased the intracellular levels of $[Ca^{2+}]i$. When apoptotic inducing drugs were administered simultaneously with static MF, the majority of cells rescued from apoptosis. However, simultaneous exposure to static MF and incubation with drugs increased significantly apoptosis in 3DO.

Cells Growing in Suspension

Human lymphocytes and 3DO cells. Twenty-four hours exposure to static MF determined opposite effects on human lymphocytes and 3DO cells, a primary cell culture and a hybridoma cell line, respectively (Fig. 3). Human lymphocytes exposed to 6 mT static MF showed no signs of apoptosis, while static MF was able to induce apoptosis in 3DO cells (>100% increment after 48 h exposure) (Table 1). In addition, an increasing percentage of human lymphocytes with elongate shape was observed with time of exposure and, after 24 h, many lamellar microvilli were seen on their surfaces (Fig. 3). On the contrary, cell shape of 3DO cells never underwent modifications even at longer time of exposure, unless very few blebbing bearing cells. When lymphocytes were treated with apoptotic drugs under static MF, a significant decrement of apoptotic cells was measured (about 25–30% less) not correlated with the apoptotic inducers (Table 2). Also, in these conditions 3DO cells behaved differently from lymphocytes: apoptosis, measured after incubation with CHX 10^{-2} M for 24 h in the presence of static MF exposure, slightly increased (about 5–9%). Mitosis of 3DO was also increased under static MF; conversely, lymphocytes were not induced to proliferate (Table 3). Static MF did not induce necrosis, neither in lymphocyte nor in 3DO cell cultures, at least for the period under investigation.

Thymocytes

Thymocytes maintained in culture for up to 3 days, showed no signs of necrosis and about 10% of spontaneous apoptosis (Fig. 4). Within 24 h from isolation, thymocytes rescued from the stress (Tables 1 and 3) and at 24 h from isolation started to enter mitosis. The exposure to static MF for up to 24 h led to a faster rescue from the isolation stress; in fact, apoptosis decreased and mitosis were detected as soon as 3 h of continuous exposure. The increment of mitosis was of about 200% (Table 3). Necrosis was negligible in the control

TABLE 1. Evaluation of the Percentage of Viable and Apoptotic Cells After 24 or 48 h of Exposure to 6 mT Static MF on Haematoxylin/Eosin Stained Cells

	24 h				48 h			
	% Viability		% Apoptosis		% Viability		% Apoptosis	
Cells	−MF	+MF	−MF	+MF	−MF	+MF	−MF	+MF
Lymphocytes	94	93	6	7	90	90	9	10
3 DO	95	90	5	10*	93	75*	7	25*
Thymocytes	90	96	5	2	93	92	7	8
U937	93	87	9	10	93	85	7	11
Hep G2	96	69*	5	30*	93	67*	8	32*
HeLa	94	90	4	3	90	87	6	5
FRTL-5	95	87	5	13*	93	87	6	14*

Values are the mean of three independent experiments.
*$P > 0.1$ versus unexposed to MF. SE does not exceed 5%.

TABLE 2. Flow Cytometry Evaluation of the Percentage of Induced Apoptotic Cells in the Presence or in the Absence of Exposure to 6 mT Static MF for 24 h

	% Apoptosis							
	CHX		H_2O_2		PMC		HS	
Cells	−MF	+MF	−MF	+MF	−MF	+MF	−MF	+MF
Lymphocytes	28	18	22	15*	40	25	20	10*
3 DO	85	89*	ND	ND	85	93	75	80
Thymocytes	80	35*	60	47*	75	26*	60	50*
U937	90	68*	72	45*	80	55*	78	58*
Hep G2	85[a]	45[a,*]	71[a]	32[a,*]	90[a]	65[a,*]	ND	ND
	95[b]	65[b,*]	83[b]	53[b,*]	98[b]	73[b,*]	ND	ND
HeLa	8[a]	3[a,*]	5[a]	2[a,*]	9[a]	4[a,*]	ND	ND
	63[b]	47[b,*]	59[b]	42[b,*]	71[b]	62[b,*]	ND	ND
FRTL-5	70[a]	54[a,*]	ND	ND	50[a]	33[a,*]	ND	ND
	93[b]	82[b]	ND	ND	78[b]	58[b,*]	ND	ND

Values are the mean of three independent experiments. SE does not exceed 5%.
ND, not determined.
[a]Values measured on the attached subset population.
[b]Values measured on the detached subset population.
$^*P < 0.1$ versus −MF.

cultures with values unchanged in the presence or in the absence of static MF. Significant modifications of cell morphology was not observed in thymocytes exposed to static MF.

Thymocytes showed different apoptotic rates when treated with HS, CHX or PMC, ranging between 60 and 80% (Table 2). When apoptosis was induced in the presence of static MF an overall significant reduction of the apoptotic rate, independent of the apoptotic inducer, was measured. The reduction of apoptosis was higher at the longer time examined (data not shown).

U937 Cells

Viability of U937 cells was not affected when cells were cultured under static MF for up to 48 h [Table 1. See also Chionna et al., 2003]. Mitosis increased of about 15% after 24 h of continuous exposure (Table 3). However, the most impressive effect of static MF on U937 cells was the progressive modification of the cell shape and surface with time exposure: cells became more and more elongate: (Fig. 5). Like human lymphocytes, U937 cells showed many lamellar microvilli on the cell surface during the exposure to static MF. Apoptosis of U937 cells, was induced with different drugs, that is CHX, PMC, H_2O_2, HS and ranged between 70 and 90% (Fig. 1). When cells were simultaneously exposed to static MF and apoptotic drugs, a significant decrement of apoptosis (between 20 and 30%) was measured. Interestingly, the modifications of apoptosis rate were time-dependent but inducer-independent (Fig. 1, panel C).

Monolayer-growing Cells

In monolayer-growing cells each analysis was performed on two cell subsets: adherent or detached

TABLE 3. Summaries of Main Differences Under 24 h Exposure to 6 mT Static MF

		Apoptosis			
Cells	Cell viability	S	D	Mitosis	Ca^{2+}
Lymphocytes	=	=	−25%	=	+200%
3 DO	=	+3%	+9%	+4%	+100%
3DO plus 1 h recovery		−3%	=		
Thymocytes	+10%	−10%	−60%	+200%	+100%
U937	=	=	−20%	+15%	+200%
Hep G2	−30%	+3%	−40%	+5%	+40%
HeLa	+10%	=	−50%	+20%	+100%
FRTL-5	−15%	+15%	−20%	−8%	ND

+, Indicates an increment increase, −, Indicates a decrease in percentage (%) in exposed compared to unexposed cells. =, Indicates unmodified values; ND, not determined; S, spontaneous apoptosis; D, drug-induced apoptosis; Cell viability, apoptosis and mitosis were evaluated by counting at least 500 cells in at least five randomly selected fields [Ca^{2+}] was measured with the spectrophotometer.

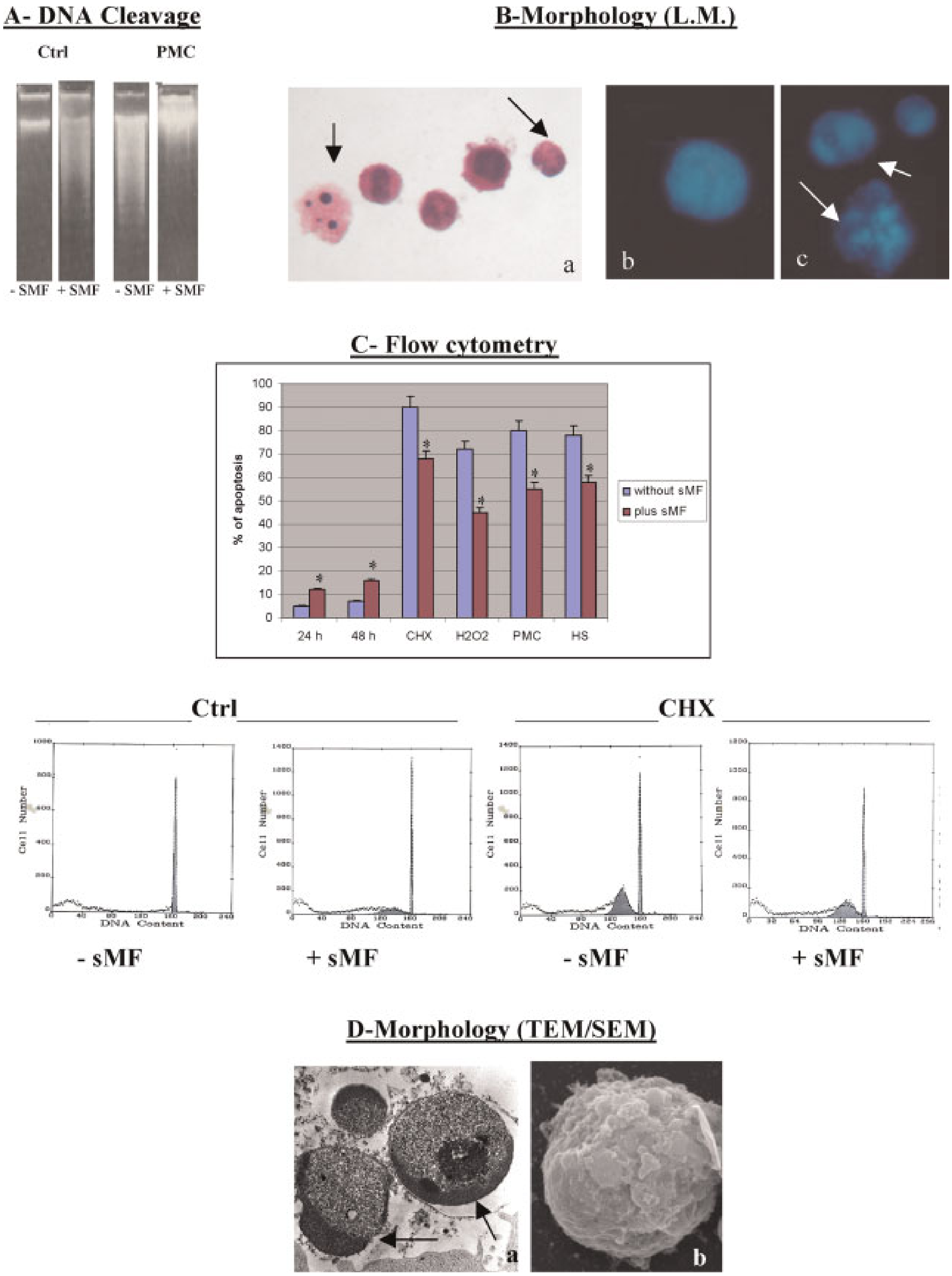

Fig. 1. Representative data from the diverse methods used to visualise and quantify apoptosis in the presence or absence of 6 mT static MF in U937 cells. The data obtained by the different methods are consistent each other. Representative results from three independent experiments are shown. **A**: DNA from U937 cells in the absence and in the presence of 6 mT static MF for 48 h on 1.5% agarose gel, as described in Material and Methods. The ladder-like pattern of apoptosis is well evident in apoptosis inducing drug-exposed cells in the absence of static MF. **B**: Light microscopy of haematoxylin/eosin (**a**) or Hoechst-33342 (**b** and **c**) stained U937 cells after 24 h of 6mT static MF (b) or apoptosis induction under 24 h field exposure (c). Arrows indicate the nuclear fragments. **C**: Flow cytometry of control and apoptotic U937 cells in the absence and in the presence of 6 mT static MF; for each measurement at least 10 000 cells were counted. Representative cytograms are reported below. Cycloeximide (CHX), puromycin (PMC), heat shock (HS). Significantly different (*) from unexposed, $P < 0.1$. Error bars represent the standard deviation $\pm$ SE of three independent experiments, each done in duplicate. **D**: transmission (TEM, **a**) and scanning electron microscopy (SEM, **b**) of apoptotic U937 cells induced to apoptosis under static MF for 24 h. Arrows indicate crescent shaped condensed chromatin. Note the smoothness of surface in b. [The color figure for this article is available online at www.interscience.wiley.com.]

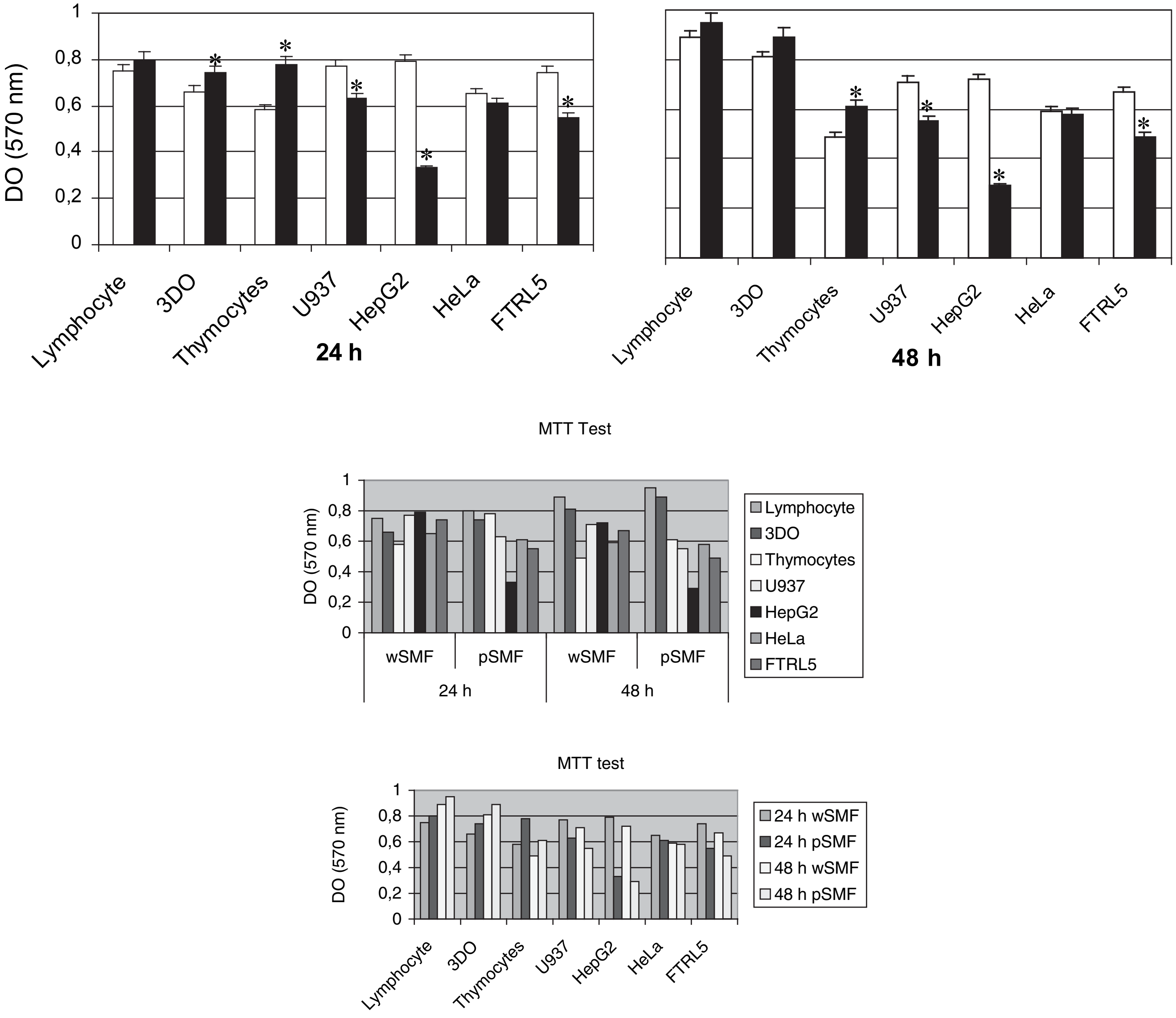

Fig. 2. Surviving cell number, after 24 or 48 exposure to 6 mT static MF, was determined indirectly by MTT dye reduction. The amount of MTT-formazan produced is determined spectrophotometrically at 570 nm and shown as absorbance values. White columns represent unexposed cells; black columns represent exposed cells. Significantly different (*) from unexposed, $P < 0.1$. Error bars represent the standard deviation $\pm$ SE of three independent experiments, each done in duplicate.

cells. In general, in cells triggered to apoptosis only a small number of typical apoptotic figures, whose range was related to the cell type and to the apoptotic inducer, were present in the attached subset cell population. On the contrary, floating cells were enriched in apoptotic morphologies, due to the progressive loss of cell adhesion during apoptosis.

FRTL-5

When FRTL-5 cells were exposed to static MF in the absence of apoptotic inducers an increment of apoptosis was observed (Tables 1 and 3). Indeed, 24 h exposure to static MF increased apoptosis of about twice (Table 1). Mitosis decreased of about 8% during exposure to static MF, while necrosis was never observed. Modifications of cell shape were also never observed for exposures longer than 24 h. However, static MF decreased the adhesion to the substrate; many floating cells were found in the culture medium.

FRTL-5 cells treated with CHX for 24 h or PMC for 4 h underwent apoptosis to different extents (70 and

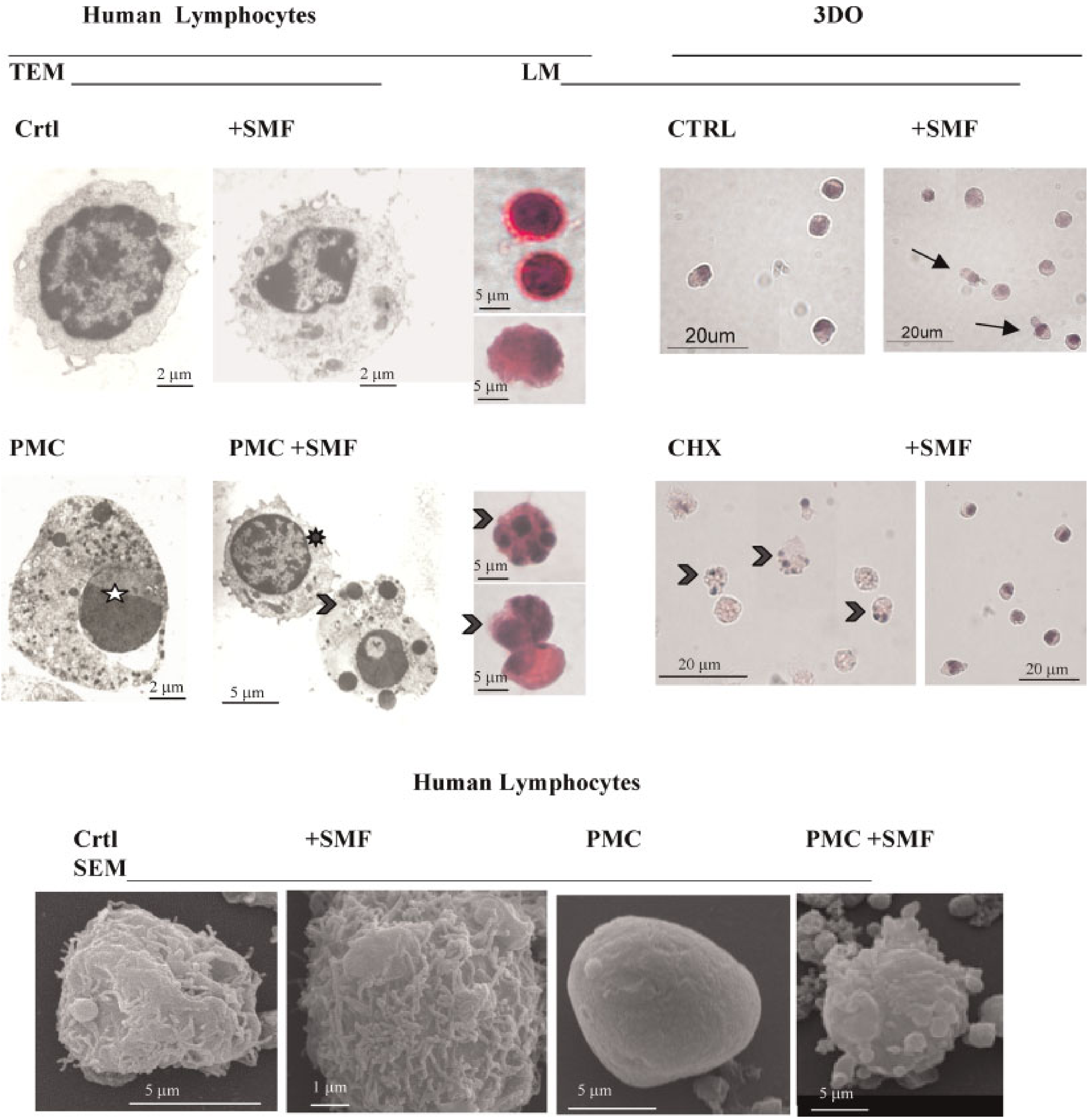

Fig. 3. Morphological modifications of human lymphocytes and 3DO cells exposed to 6 mT static MFs for up to 24 h in the presence and the absence of CHX (10^{-2} M) or PMC (10^{-6} M). Micrographs are TEM (left), LM (right) images. Arrows indicate morphological modifications (large blebs) in 3DO cells; arrowheads indicate apoptotic cells with fragmented nuclei in both cell types; asterisk indicates the presence of a normal cell among the apoptotic ones when the death induction is performed under static MF; white star indicates condensed chromatin. SEM micrographs show modification of cell surfaces of control and apoptotic human lymphocytes in the presence and the absence of static MF. [The color figure for this article is available online at www.interscience.wiley. com.]

50%, respectively) (Table 2). The number of apoptotic cells increased after 48 h of incubation with CHX. Apoptotic FRTL-5 showed an extensive cell blebbing and nuclear fragmentation (Fig. 6). Conversely, a reduction of apoptosis of about 20% and 15% at 24 h and at 48 h of exposure, respectively, was measured when cells were triggered to apoptosis to in the presence of static MF.

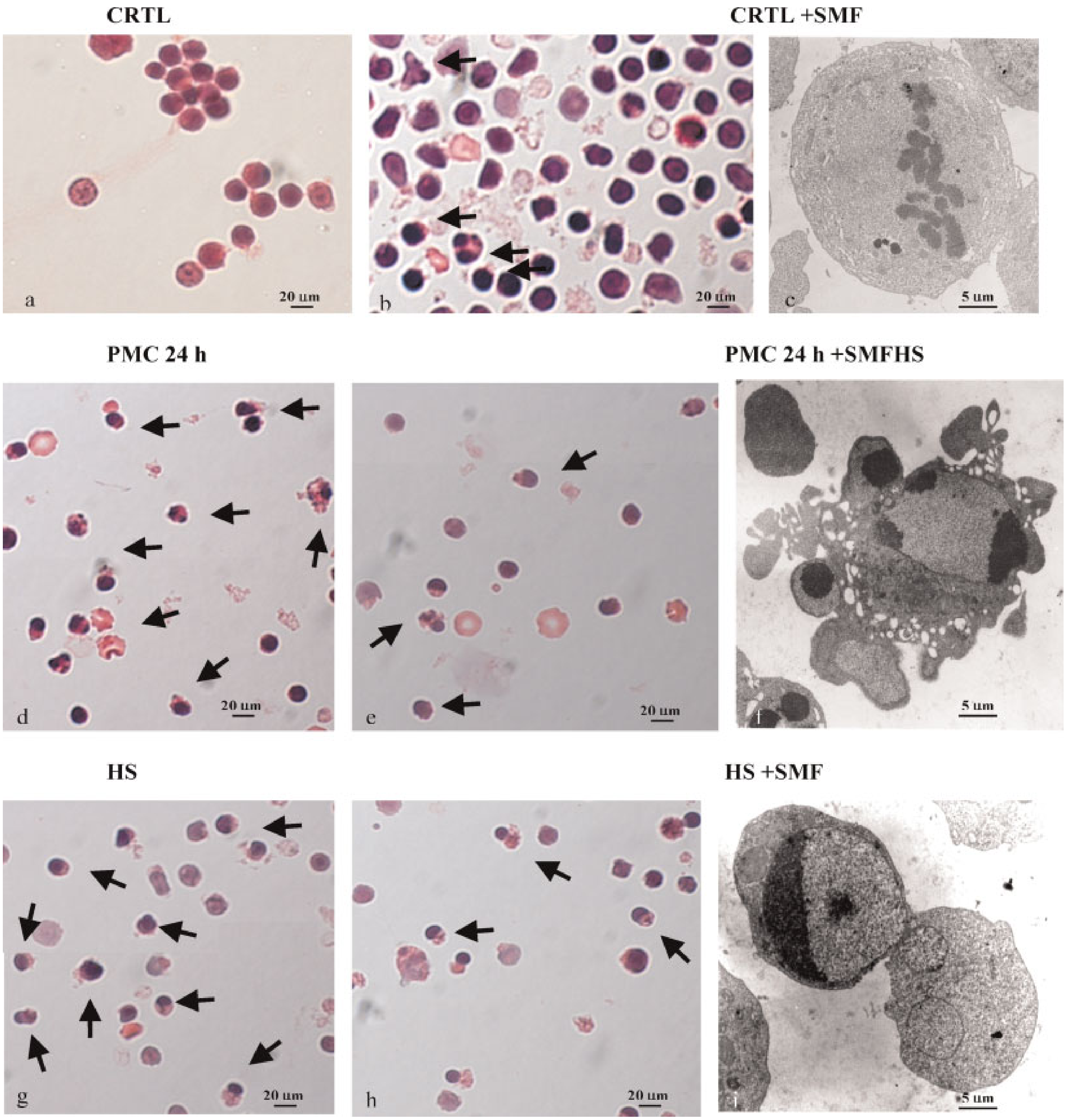

Fig. 4. LM (**a**, **b**, **d**, **e**, **g**, **h**) and TEM (**c**, **f**, **i**) images showing the morphology of isolated mice thymocytes exposed to static MFs of 6 mT for up to 24 h in the presence and the absence of PMC 10^{-6} M or HS (1 h at 43 °C). Apoptosis is shown by arrows. TEM images represent the morphology of exposed thymocytes in the presence of apoptotic-inducing drugs. c: mitotic thymocytes; (f, i) two different morphologies of apoptotic thymocytes. [The color figure for this article is available online at www.interscience.wiley.com.]

HepG2 Cells

Viability of HepG2 cells significantly decreased (30% less) within the first 4 h of exposure to static MF and remained constant during the entire experimental time, that is 48 h (Table 1). Loss in viability was likely due to apoptosis (Fig. 7). Abundant nuclei with the typical feature of apoptosis were visible in HepG2 cells

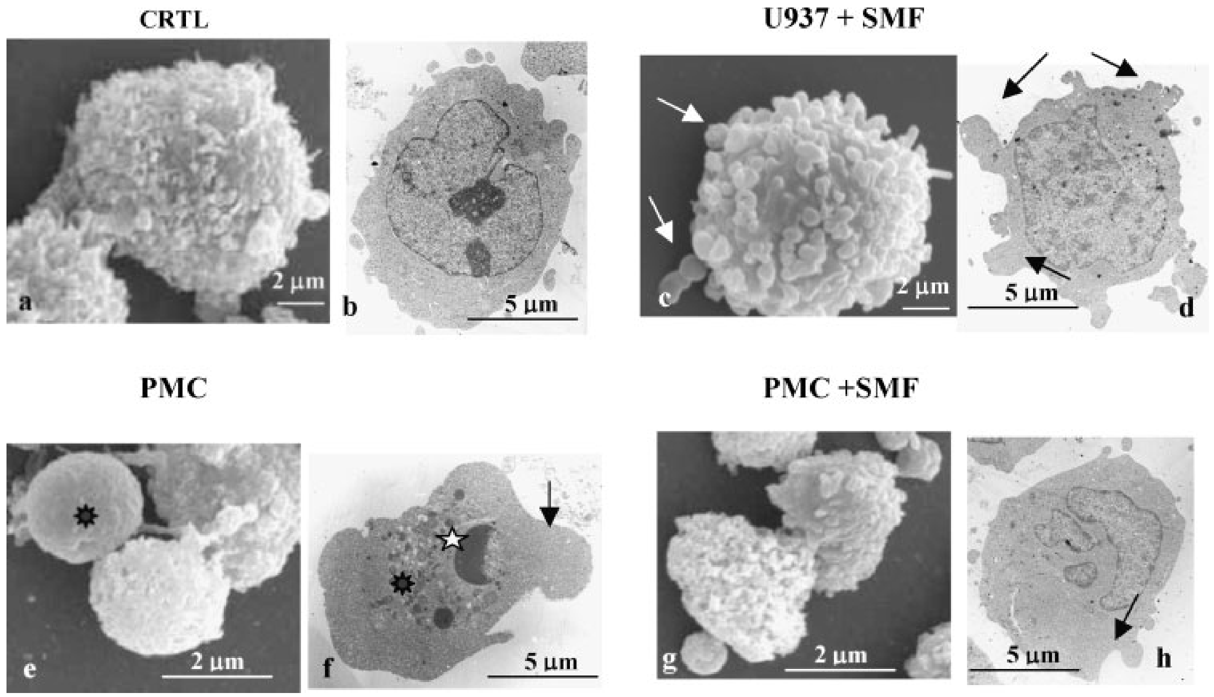

Fig. 5. SEM (**a**, **c**, **e**, **g**) and TEM (**b**, **d**, **f**, **h**) micrographs showing cell shape modification of U937 cells exposed to 6 mT static MFs for up to 24 h. a: and e: Controls of U937 cells are characterised by a round shape with microvilli randomly distributed all over the cell surface. The continuative exposure to static MFs up to 24 h leads to profound cell shape distortion and/or lamellar or bubble-like microvilli presence (c and d, arrows) when compared to controls. Organelles are well preserved even after 24 h of exposure to static MFs. Apoptotic cells are characterised by a smooth surface, an almost round shape (e and f, asterisk) and a condensed chromatin (f, white star). Typical apoptotic features of U937 cells after incubation with puromycin (PMC) 10^{-6} M for 8 h (e and f) are reduced (note in particular the loss in chromatin condensation) when cells are simultaneously incubated with PMC and exposed to static MFs (g and i). Arrows in c, d and h indicate blebs.

exposed to static MF for 24 h; indeed, apoptotic rate reached the value of 30% at 24 h of exposure. However an increment of mitosis was measured as well (Table 3).

HepG2 cells continuously grow in monolayer and show an epithelial morphology. Control unexposed HepG2 cells have a flat and polyhedric shape tightly attached to the culture plate; tiny short microvilli are randomly distributed on the cell surface (Fig. 7). Morphological modifications were progressively evident with time of exposure (up to 48 h), leading to dramatic changes of cell shape: cells slowly acquired a fibroblast-like shape (Fig. 7). The cytoplasm concentrated around the nucleus, making this part of the cells more thick and round. In parallel, cell surface was enriched with many microvilli, that with time of exposure become round and/or lamellar giving rise to rough, foamy-like surfaces.

The simultaneous exposure to static MF and PMC or H_2O_2 or CHX rescued cells to enter apoptosis of about 40% (Table 2). Accordingly, cell modifications typical of apoptosis were prevented by static MF exposure (Fig. 7).

HeLa Cells

The effect of CHX, H_2O_2 or PMC in the presence or absence of static MF on monolayer-growing HeLa cells was analysed (Tables 1 and 2 and Fig. 8). As an effect of the treatment, cells progressively detached from the solid support. Only a small number of typical apoptotic figures (ranging between 5 and 10%) was present in the attached cells. In addition, attached cells under static MF showed evident signs of morphological modifications of the adhesion plaques. Conversely, the floating cells were enriched (about 70%) of apoptotic morphologies, due to progressive loss of cell adhesion during apoptosis. Chromatin condensation was clearly visible. Electron microscopy analysis (Fig. 8) confirmed the morphological changes visualised by nuclear staining. Control HeLa cells express many tiny microvilli on their surface and a long nucleus with a big nucleolus. Cytoplasm is full of organelles and vacuoles. In the apoptotic HeLa cells

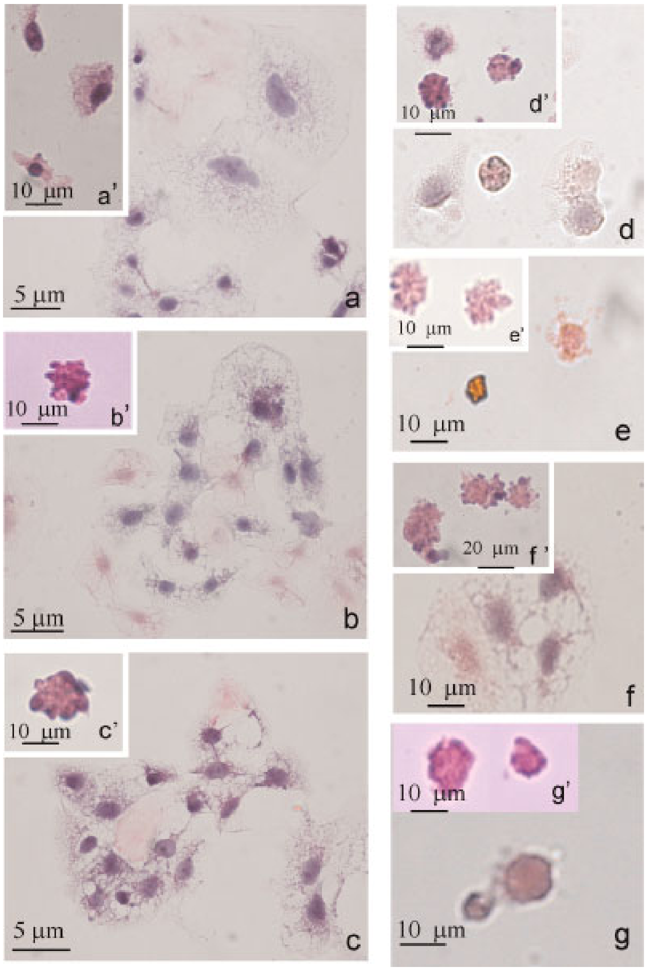

Fig. 6. LM micrographs showing cell shape modification of FRTL-5 cells exposed to 6 mT static MFs for up to 48 h. Control cells, adhering to the culture dishes, are characterised by a flat and polyedric shape (**a**). Irregularly shaped cells flow in the culture medium (**a′**). The continuative exposure to static MFs for 24 h (**b**) and 48 h (**c**) do not lead to profound cell shape distortion but to an increase of detached cells, many of them were apoptotic (**b′** and **c′**) when compared to controls. FTRL-5 cells underwent apoptosis upon incubation with CHX for 24 and 48 h (**d** and **e**). Many apoptotic cells are found in the culture medium (**d′** and **e′**). The simultaneous incubation of cells with CHX 10^{-2} M and 6 mT static MF reduced the number of apoptotic cells in the culture medium (**f′** and **g′**) and, in particular after 24 h (**f**) of exposure, increased the number of still substrate adhering cells. In (**g**) are shown 48 h 6 mT MF exposed cells. [The color figure for this article is available online at www.interscience.wiley.com.]

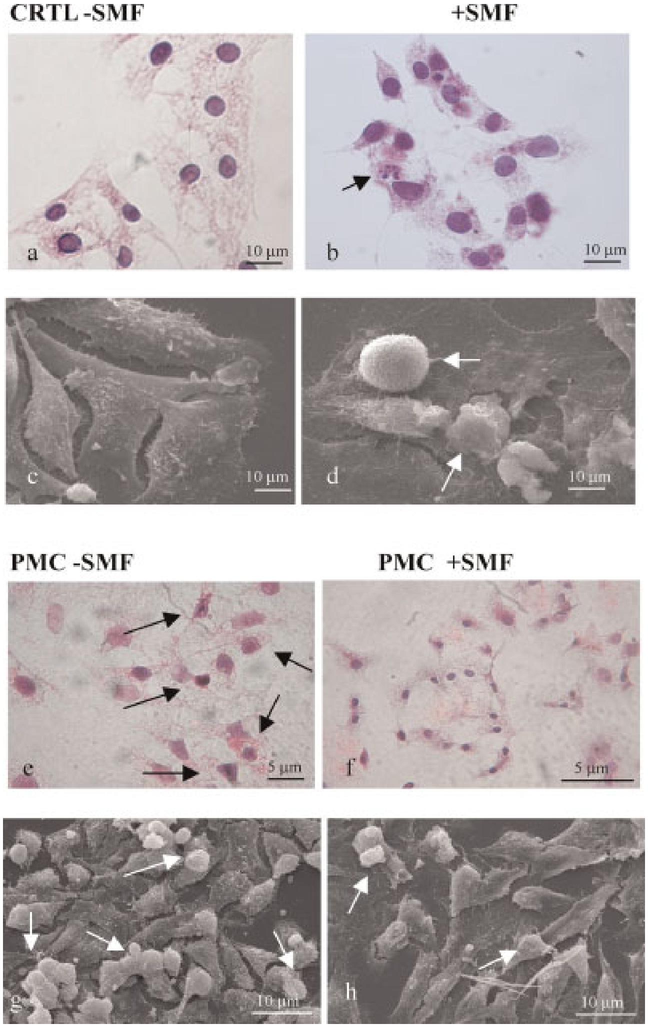

Fig. 7. LM (**a**, **b**, **e**, **f**) and SEM (**c**, **d**, **g**, **h**) micrographs showing cell shape modification of HepG2 cells exposed to 6 mT static MFs for up to 24 h. a and c: Controls of HepG2 cells are characterised by a flat and polyedric shape with microvilli randomly distributed all over the cell surface. Exposure to static MFs up to 24 h leads to profound cell shape distortion and/or lamellar and bubble-like microvilli presence (b and d). Apoptotic cells are observed as well (b and d, arrows). After induction of apoptosis with PMC (10^{-6} M) for 8 h in the absence of 6 mT static MF, almost all the cells died (e and g, apoptotic cells are indicated by arrows). Conversely, apoptotic induction in the presence of 6 mT static MF, significantly reduced the number of cell deaths (f and h, arrows). [The color figure for this article is available online at www.interscience.wiley.com.]

microvilli disappeared as well as the majority of organelles. Vacuoles, conversely, remained and increased their size. Nucleous size was reduced and chromatin was condensed and, in some cases, fragmented. Enlargement of the nuclear cisternae was observed facing the condensed chromatin.

The presence of static MF during the apoptotic treatment significantly reduced the rate of apoptosis in a drug-independent manner. As just found for other cellular types, static MF induced the formation of microvilli, that appeared particularly elongated with the simultaneous presence of static MF and apoptosis inducing drugs (Fig. 8).

Calcium Ion Concentration

Modulation of apoptosis could be due to modulation of $[Ca^{2+}]i$ exerted upon exposure to static MF. $[Ca^{2+}]i$ were measured with the spectrophotometer at

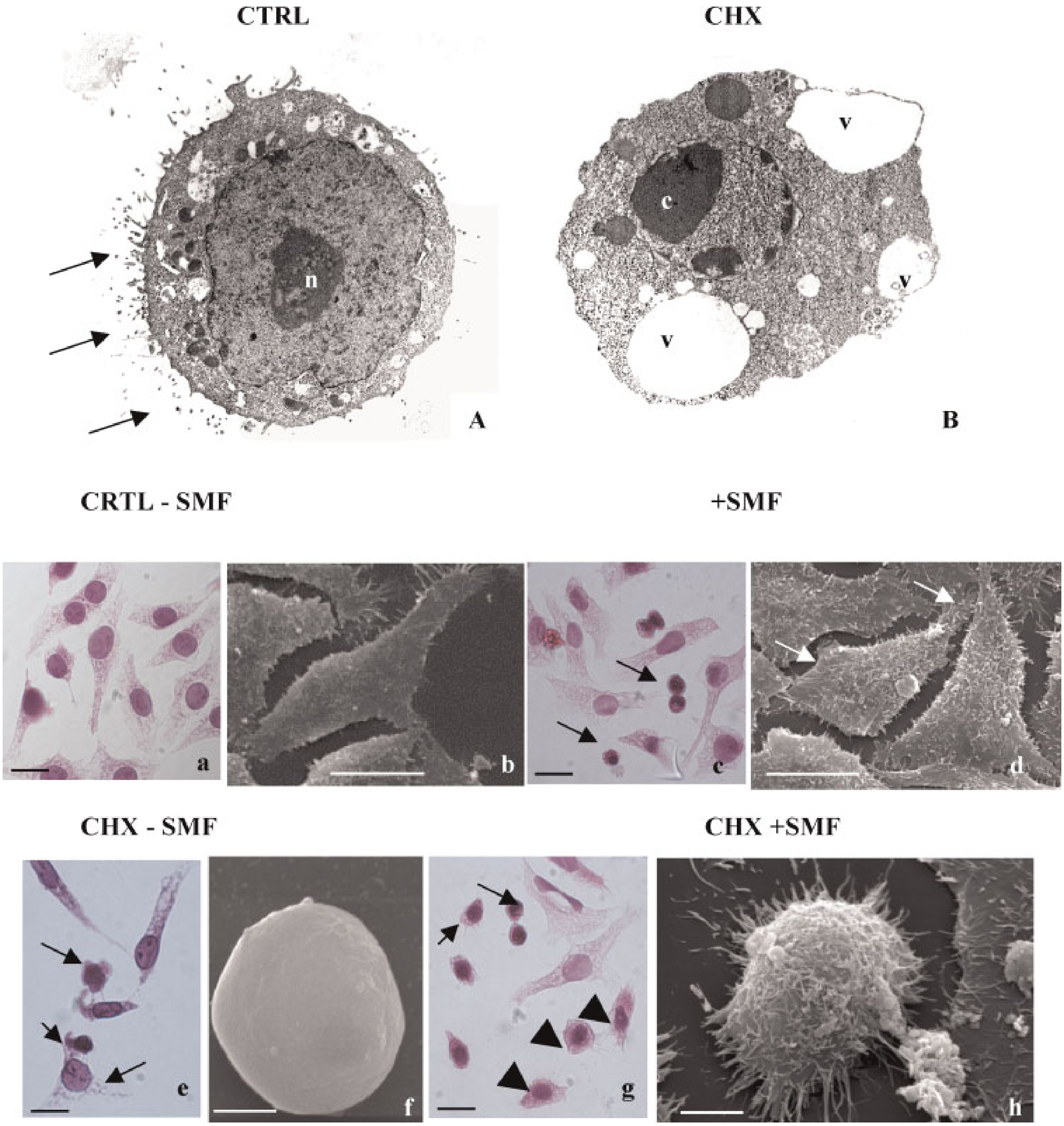

Fig. 8. TEM (**A** and **B**) micrographs show control and apoptotic HeLa cells. Many tiny microvilli on the surface of control HeLa cell (A), a big nucleous and nucleolus (n) and many organelles and vacuoles (v) in the cytoplasm are shown. In the apoptotic HeLa cell (B) chromatin condensation, big vacuoles and loss of microvilli were clearly visible. SEM (**b**, **d**, **f**, **h**) and LM (**a**, **c**, **e**, **g**) micrographs showing cell shape modification of HeLa cells exposed to 6 mT static MFs for up to 24 h. Control HeLa cells (a, b) are characterised by a flat and polyedric shape with microvilli randomly distributed all over the cell surface. Exposure to static MF up to 24 h leads to loss of the polyedric shape and to the appearance of many and filamentous microvilli presence (d arrows). Apoptosis is also increased (c, arrows). CHX 10^{-2} M for up to 24 h induced apoptosis in the cell cultures (e, f). Apoptotic cells (e) are shown by arrows. The surface of apoptotic cells could become dramatically smooth (f). The simultaneous exposure of static MF and CHX rescued cells to enter apoptosis and prevented the modifications typical of apoptosis. g: arrows indicate apoptotic cells; arrorheads indicate rounded up cells with many elongate microvilli, whose SEM image is shown in h. Bars: 5 µm. [The color figure for this article is available online at www.interscience.wiley.com.]

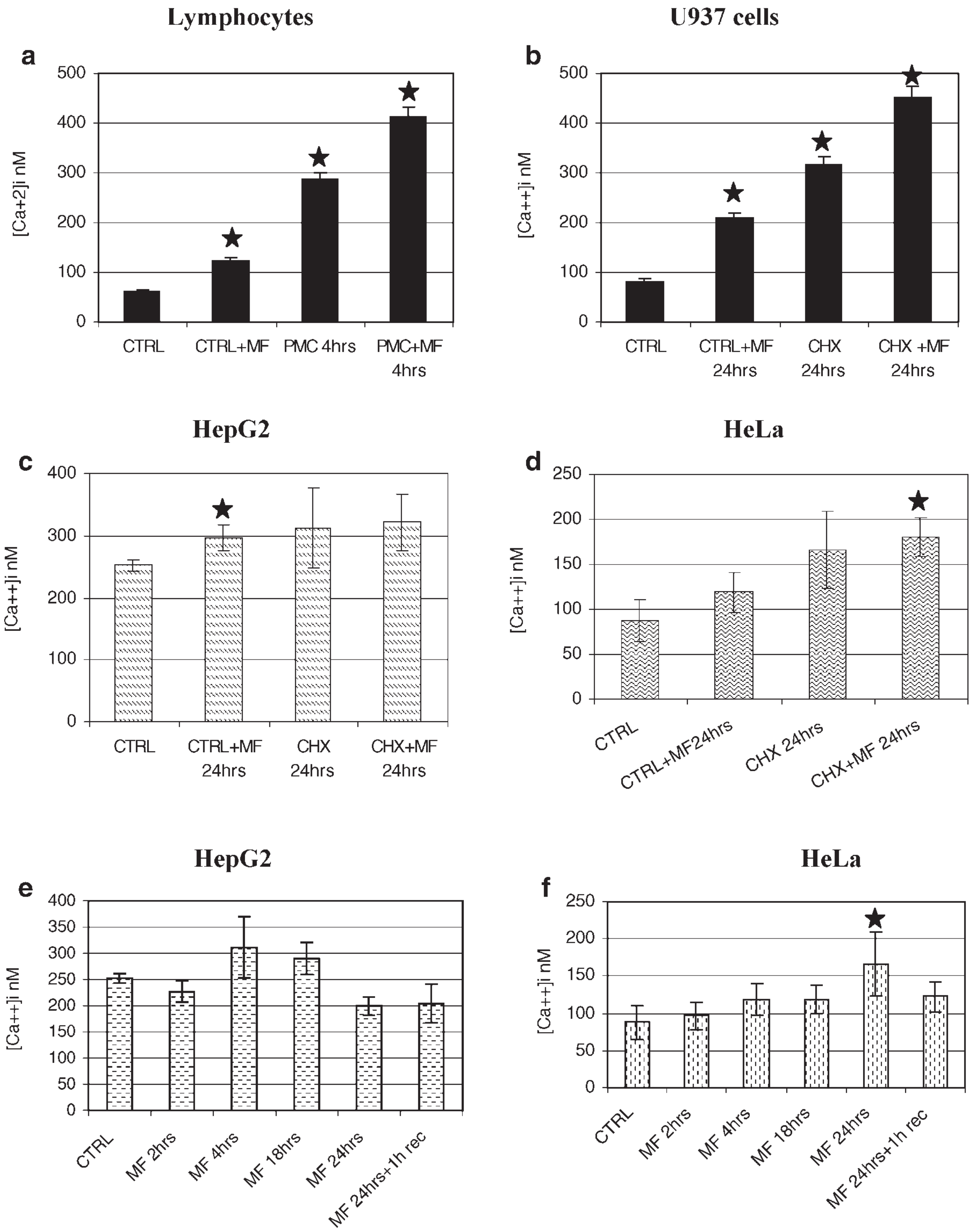

Fig. 9. Concentration of [Ca^{2+}]i in lymphocytes (**a**), U937 cells (**b**), HepG2 cells (**c**) and HeLa cells (**d**) after 24 h exposure to 6 mT static magnetic field and during induction of apoptosis in the presence or absence of static MF. Time-course of the modulation of [Ca^{2+}]i in HepG2 (**e**) and HeLa (**f**) cells during the 24 h exposure to 6 mT static MF. Significantly different from control and from unexposed corresponding (*), $P < 0.1$. Error bars represent the standard deviation $\pm$ SE of three independent experiments, each done in duplicate.

fixed times during the exposure to static MF and during the induction of apoptosis in the presence of static MF (Fig. 9). Increased values of $[Ca^{2+}]i$ were measured during the static MF exposure as well as during the induction of apoptosis. A further increment was measured when apoptosis was induced in the presence of static MF. $[Ca^{2+}]i$ increments differed in the various cell types. For example, in the lymphocytes and in U937 cells the $[Ca^{2+}]i$ increased by 200 and 300%, respectively, after 24 h of simultaneous incubation with apoptotic drugs and static MF, while in HepG2 and HeLa cells the increase was 40 and 100%, respectively. Time course of $[Ca^{2+}]i$ measurement revealed that $[Ca^{2+}]i$ were modulated during the entire period investigated in HepG2 and HeLa cells; in particular, the highest values of $[Ca^{2+}]i$ in HepG2 cells were measured after 4 h of exposure (about 35% of increment) while in HeLa cells the peak was after 24 h of exposure (about 100% of increment).

DISCUSSION

In this work, the comparative study of in vitro biological effects of 6 mT static MF on different cell types, that is primary culture, transformed or stabilised cell lines, with different embryonic tissue derivation, has been described. The research was mainly focused on the effect of exposure to static MF on the process of apoptosis (spontaneous and induced). Proliferation, necrosis, Ca^{2+} ions concentration and modifications of cell shape were also monitored. Our data showed that 6 mT static MF exerts strong and reproducible effects on all the cells under investigation. In particular, static MF influenced both apoptosis and mitosis. The type (increase and/or decrease) and the extent of modulation of apoptosis was dependent on cell type and on time of exposure. The apoptotic inducers did not modulate neither type or extent of apoptosis, suggesting that static MFs interact with the apoptotic process but not with the apoptotic inducer.

Our results also suggest that the conflicting results present in literature on static MF are not only due to different experimental conditions (type and intensity of field), but also to type of cells (normal, stabilised or transformed). Indeed, the different responses to the static MF of normal, stabilised or transformed cells is in agreement with the different electrical behaviour of tumour and normal cells [Cuzick et al., 1998; Tofani et al., 2003]. It is known that rapidly proliferating and transformed cells have differently polarised cell membranes compared with normal cells [Marino et al., 1994] and that epithelial cells lose their transepithelial potential during carcinogenesis [Capko et al., 1996]. Altered cell survival was assumed to come with electric disorders and different tissue and cell electrical behaviour. Consequently static MFs, by acting on charged matter motion, may selectively modulate different cell signalling pathways in different cells, depending of their membrane potential and exerting different effects on survival [Tofani et al., 2003].

Cell survival is regulated by apoptosis that, in turn, is modulated by static MF [Fanelli et al., 1999; Teodori et al., 2002a,b; Chionna et al., 2003; Dini and Abbro, 2005]. However, contrasting results were reported. Indeed, apoptosis seems to be very sensitive to MFs exposure via modulation of $[Ca^{2+}]i$ fluxes, which are modified by exposure [Fanelli et al., 1999; Chionna et al., 2003]. In our experiments a mobilisation of Ca^{2+} ions during exposure is a constant finding. This seems to be a crucial event, since Ca^{2+} can contribute to many cellular functions. In fact, Ca^{2+} ions are mediators of intracellular signalling crucial for the development of apoptosis. An increase of $[Ca^{2+}]i$ in cells committed to apoptosis, due to emptying of intracellular $[Ca^{2+}]i$ stores and to $[Ca^{2+}]i$ influx from the extracellular medium, is quite a general phenomenon, independent of the apoptotic stimulus [Bian et al., 1997]. Nevertheless, the role of $[Ca^{2+}]i$ increase during apoptosis is ambiguous because it exerts different effects in different cell systems [Magnelli et al., 1994; Teodori et al., 2002a,b].

A general mechanism for the action of moderate intensity static MFs on biological systems would be by virtue of their effect on the molecular structure of excitable membranes, an effect sufficient to modify the function of embedded ion-specific channels, [Fanelli et al., 1999; Teodori et al., 2002a,b; Rosen, 2003]. This hypothesis would explain virtually all of the bioeffects attributed to these fields, including the modulation of apoptosis, and is testable using several different neurophysiological techniques [Rosen, 2003]. Other possible effects of static MFs leading to perturbation of the apoptotic rate [Fanelli et al., 1999; Jajte, 2000; Chionna et al., 2003], such as alteration of the gene pattern expression [Marinelli et al., 2004; L. Dinti, personal communication, 2006] or increment of oxygen free radicals can not be excluded. It is well-known that free radicals are mediators of apoptosis [Brune, 2003]. Interestingly, the concentration of free radicals in transformed cells and tissues is higher than in their normal counterparts [Szatrowski and Nathan, 1991]. In addition, concentration of free radicals has been described as increasing in different conditions of exposure, from static MF to pulsed MF to EMF [Jaite et al., 2002; Stevens, 2004]. The role of oxygen free radicals in balancing the apoptosis (spontaneous and drug-induced) needs to be deeply investigated.

The abnormal formation of microvilli, which at very long times of exposure can show lamellar or foamy shape, is in line with what is described by other authors [Popov et al., 1991; Chionna et al., 2003] and with the recently formulated general concept of regulation of ion and substrate pathways by microvilli. The actin-based core of microfilaments in microvilli has been proposed to represent a cellular interaction site for MFs [Gartzke and Lange, 2002]. The duration of the exposure (within some limits) is important in determining the extent of the plasma membrane and cell shape modifications in response to static MFs. It is known that cell shape modifications are a function of duration of static MFs exposition up to a 'limit', above which longer durations are not associated with further cell shape and plasma membrane modifications [Rosen, 1993; Chionna et al., 2003, 2005].

However, in addition to the exposure time, cell type also is crucial for morphological modifications. Indeed, dramatic differences in the morphology of HepG2, U937, HeLa cells and lymphocytes after a short or a long time exposure to 6 mT static MF were described. Since high MFs in vitro promoted cytoskeleton reorganisation by assembling their component and by modulating their orientation [Bras et al., 1998], it is likely that these cytoskeleton modifications induced changes of cell shape [Popov et al., 1991; Santoro et al., 1997; Chionna et al., 2003]. Indeed, modulation of the Ca^{2+} influx could also promote modifications of the cell shape, since the mechanism of reorganisation and breakdown of different cytoskeleton elements (polimerisation of F-actin is Ca^{2+} dependent) is related to modified Ca^{2+} homeostasis or altered phosphorylation/dephosphorylation.

Differences in exposure systems and conditions complicate the evaluation of studies indicating biological effects of MFs. This is also true for the induction to proliferation exerted by MFs; some reports indicate that exposure to static or oscillating MFs enhance proliferation in prokaryotic cells [Roman et al., 2002; Potenza et al., 2004] while other reports show no modification [Gluck et al., 2001; Yoshizawa et al., 2002] or a decrement [Cohly et al., 2003]. Indeed, contradictory results were also reported in the present paper. The type of the cell seems to be crucial also for this type of cell response. However, no contradictions were described regarding induction of necrosis: this form of cell death was never observed in the cell cultures above basal levels (about 2–5%). Therefore, decrement of cell viability, when present, is exclusively due to apoptosis and it is counterbalanced in some cases by an increase in cell proliferation. It could be hypothesised that static MFs are lethal only for a small portion of cells—those in a peculiar metabolic state or in a specific phase of the cell cycle—that are therefore removed from the cultures through apoptosis. Conversely, the increased proliferation could be considered an adaptation response of the cells to the continuous and prolonged exposure to static MFs.

It has been recently suggested that cell processes can be influenced by the combination of MFs and drugs, thus leading to a new fascinating possible chemotherapy of cancers that is evolving currently [Gray et al., 2000; Sabo et al., 2002]. Extremely low frequency (ELF) MFs exposure may potentiate the effects of known carcinogens only when the animals are exposed to both ELF MF and carcinogen during an extended period of tumour development [Loscher, 2001]. Indeed, modulation of apoptosis, as likely suggested from the data here shown, could be one possible mechanism of this type of cancer therapy. However, further investigations are needed to define the type and the intensity of field and the type of transformed/tumour cells sensitive to the combined treatment (drugs and MF).

In conclusion, the data described in the present paper suggest a modulation by static MFs on apoptosis in different cells. In particular, there is specific interference of the exposure with the drug-induced apoptosis. Time of exposure and cell type have been found to be important factors for the quantity and quality of the effects produced; for example apoptosis increases in some cells and decreases in the other. The unbalance of the apoptotic process, which could be linked to Ca^{2+} fluxes, could be in turn a co-carcinogenic factor leading normal cells, most likely with other sublethal changes, to the development of diseases; on the other hand, modulation of Ca^{2+} fluxes could promote induction of apoptosis in transformed cells, acting in synergy with apoptotic inducing drugs. Thus the modulation of the apoptotic process by MFs could be used to develop new therapeutic strategies for cancer cells that have become chemoresistant.

REFERENCES

Alenzi FQ. 2004. Links between apoptosis, proliferation and the cell cycle. Brit J Biom Sci 61:99–102.

Ambesi-Impiombato FS, Villone G. 1987. The FRTL-5 thyroid cell strain as a model for studies on thyroid cell growth. Acta Endocrinol Suppl (Copenh) 281:242–245.

Bian X, Hughes FM Jr, Huang Y, Cidlowski JA, Putney JW Jr. 1997. Roles of cytoplasmic Ca^{2+} and intracellular Ca^{2+} stores in induction and suppression of apoptosis in S49 cells. Am J Phys 272:1241–1249.

Bras W, Diakun GP, Diaz JF, Maret G, Kramer H, Bordas J, Medrano FJ. 1998. The susceptibility of pure tubulin to high magnetic fields: A magnetic birefrengence and X-ray fiber diffration study. Biophys J 74:1509–1521.

Brune B. 2003. Nitric oxide: NO apoptosis or turning it ON? Cell Death Diff 10:864–869.

Capko D, Zhuravkov A, Davies RJ. 1996. Transepithelial depolarization in breast cancer. Breast Cancer Res Treat 41:230–239.

Chionna A, Dwikat M, Panzarini E, Tenuzzo B, Carlà EC, Verri T, Pagliara P, Abbro L, Dini L. 2003. Cell shape and plasma membrane alterations after static magnetic fields exposure. Europ J Histoch 47:299–308.

Chionna A, Tenuzzo B, Panzarini E, Dwikat M, Abbro L, Dini L. 2005. Time-dependent modifications of Hep G2 cells during exposure to static magnetic fields. Bioelectromagnetics 26: 275–286.

Cohly HH, Abraham GE III, Ndebele K, Jenkins JJ, Thompson J, Angel MF. 2003. Effects of static electromagnetic fields on characteristics of MG-63 osteoblasts grown in culture. Biomed Sci Instrument 39:454–459.

Cuzick J, Holland R, Barth V, Davies R, Faupel M, Fentiman I, Frischbier HJ, LaMarque JL, Merson M, Sacchini V, Vanel D, Veronesi U. 1998. Electropotential measurements as a new diagnostic modality for breast cancer. Lancet 352:359–363.

Dini L. 2005. Phagocyte and apoptotic cell interplay. In: Scovassi AI, editor. Apoptosis. Kerala, India: Research Signpost. pp 107–129.

Dini L, Abbro L. 2005. Bioeffects of moderate-intensity static magnetic fields on cell cultures. Micron 36:195–217.

Dini L, Coppola S, Ruzittu M, Ghibelli L. 1996. Multiple pathways for apoptotic nuclear fragmentation. Exp Cell Res 223:340–347.

Fanelli C, Coppola S, Barone R, Colussi C, Gualaldi G, Volpe P, Ghibelli L. 1999. Magnetic fields increase cell survival by inhibiting apoptosis via modulation of Ca^{++} influx. FASEB J 13:95–102.

Gartzke J, Lange K. 2002. Cellular target of weak magnetic fields: Ionic conduction along actin filaments of microvilli. Am J Phys Cell Physiol 283:1333–1346.

Gluck B, Guntzschel V, Berg H. 2001. Inhibition of proliferation of human lymphoma cells U937 by a 50 Hz electromagnetic field. Cell Mol Biol (Noisy-le-grand) 47: Online Pub: OL115–OL117.

Gray JR, Frith CH, Parker JD. 2000. In vivo enhancement of chemotherapy with static electric or magnetic fields. Bioelectromagnetics 21:575–583.

Grynkiewicz G, Poenie M, Tsien RJ. 1995. A new generation of $Ca2+$ indicators with greatly improved fluorescence properties. J Biol Chem 260:3440–3450.

Guimaraes CA, Linden R. 2004. Programmed cell death. Apoptosis and alternative death styles. Eur J Biochem 271:1638–1650.

Jaite J, Grzegorczyk J, Zmyslony M, Rajkoska E. 2002. Effect of 7 mT static magnetic field and iron ions on rat lymphocytes: Apoptosis, necrosis and free radical processes. Bioelectromagnetics 57:107–111.

Jajte JM. 2000. Programmed cell death as a biological function of electromagnetic fields at a frequency of (50/60 Hz). Med Pr 51:383–389.

Karasek M, Lerchl A. 2002. Melatonin and magnetic fields. Neur Let 23:84–87.

Liu MC, Marshall JL, Pestell RG. 2004. Novel strategies in cancer therapeutics: Targeting enzymes involved in cell cycle regulation and cellular proliferation. Curr Canc Drug Targets 4:403–424.

Loscher W. 2001. Do cocarcinogenic effects of ELF electromagnetic fields require repeated long-term interaction with carcinogens? Charcteristic of positive studies using the DMBA breast cancer model in rats. Bioelectromagnetics 22:603–614.

Magnelli L, Cinelli M, Turchetti A, Chiarugi VP. 1994. Bcl-2 overexpression abolishes early calcium waving preceding apoptosis in NIH-3T3 murine fibroblasts. Biochim Biophys Res Commun 204:84–90.

Marinelli F, La Sala D, Cicciotti G, Cattini L, Trimarchi C, Putti S, Zamparelli A, Giuliani L, Tommassetti G, Cinti C. 2004. Exposure to 900 Mhz electromagnetic field induces an unbalance between pro-apoptotic and pro-survival signals in T-lymphoblastoid leukaemia CCRF-CEM cells. J Cell Physiol 198:479–480.

Marino AA, Iliev IG, Schwalke MA, Gonzales E, Marler KC, Flanagan CA. 1994. Association between cell membrane potential and breast cancer. Tumor Biol 15:82–89.

Moreira ME, Barcinski MA. 2004. Apoptotic cell and phagocyte interplay: Recognition and consequences in different cell systems. Annual Acad Brasil Sci 76:93–115.

Popov SV, Svitkina TM, Margolis LB, Tsong TY. 1991. Mechanism of cell protrusion formation in electrical field: The role of actin. Biochem Biophys Acta 1066:151–158.

Potenza L, Ubaldi L, De Sanctis R, De Bellis R, Cucchiarini L, Dacha M. 2004. Effects of a static magnetic field on cell growth and gene expression in *Escherichia coli*. Mutat Res 561:53–62.

Radeff-Huang J, Seasholtz TM, Matteo RG, Brown JH. 2004. G protein mediated signaling pathways in lysophospholipid induced cell proliferation and survival. J Cell Biochem 92:949–966.

Roman A, Vetulani J, Nalepa I. 2002. Effect of combined treatment with paroxetine and transcranial magnetic stimulation (TMS) on the mitogen-induced proliferative response of rat lymphocytes. Pol J Pharmacol 54:633–639.

Rosen AD. 1993. Membrane response to static magnetic fields: Effect of exposure duration. Biochem Biophys Acta 1148: 317–320.

Rosen AD. 2003. Mechanism of action of moderate-intensity static magnetic fields on biological systems. Cell Biochem Biophys 39:163–173.

Rosen MS, Rosen AD. 1990. Magnetic field influence on Paramecium motility. Life Sci 46:1509–1515.

Sabo J, Mirossay L, Horovcak L, Sarissky M, Mirossay A, Mojzis J. 2002. Effects of static magnetic field on human leukemic cell line HL-60. Bioelectrochemistry 56:227–231.

Santoro N, Lisi A, Pozzi D, Pasquali E, Serafino A, Grimaldi S. 1997. Effect of extremely low frequency (ELF) magnetic field exposure on morphological and biophysical properties of human lymphoid cell line (Raji). Biochem Biophys Acta 1357:281–290.

Sladowski D, Steer SJ, Clothier RH, Balls M. 1993. An improved MTT assay. J Immunol Methods 157(1–2):203–207.

Stevens RG. 2004. Electromagnetic fields and free radicals. Environ Health Perspect 112:687–694.

Szatrowski TP, Nathan CF. 1991. Production of large amounts of hydrogen peroxide by human tumor cells. Cancer Res 51:794–798.

Teodori L, Gohde W, Valente MG, Tagliaferri F, Coletti D, Perniconi B, Bergamaschi A, Cerella C, Ghibelli L. 2002a. Static magnetic fields affect calcium fluxes and inhibit stress-induced apoptosis in human glioblastoma cells. Cytometry 49:143–149.

Teodori L, Grabarek J, Smolewski P, Ghibelli L, Bergamaschi A, De Nicola M, Darzynkiewicz Z. 2002b. Exposure of cells

to static magnetic fields accelerates loss of integrity of plasma membrane during apoptosis. Cytometry 49:113–118.

Tofani S, Barone D, Berardelli M, Berno E, Cintorino M, Foglia L, Ossola P, Ronchetto F, Toso E, Eandi M. 2003. Static and ELF magnetic fields enhance the in vivo anti tumor efficacy of cis-platin against lewis lung carcinoma, but not for cyclophosphamide against B16 melanotic melanoma. Pharmacol Res 48:83–90.

Wartenberg D. 2001. Residential EMF exposure and childhood leukemia: Metanalysis and population attributable risk. Biolectromagnetics 5:86–104.

Yoshizawa H, Tsuchiya T, Mizoe H, Ozeki H, Kanao S, Yomori H, Sakane C, Hasebe S, Motomura T, Yamakawa T, Mizuno F, Hirose H, Otaka Y. 2002. No effect of extremely low-frequency magnetic field observed on cell growth or initial response of cell proliferation in human cancer cell lines. Biolectromagnetics 23:355– 368.
