# Supplementary material for: Magnetic Fields and Cancer: Epidemiology, Cellular Biology, and Theranostics
Source: Int J Mol Sci. 2022 Jan 25;23(3):1339. doi: 10.3390/ijms23031339 (PMC8835851; doi:10.3390/ijms23031339)
Supplement: Supplementary file 1 [file ijms-23-01339-s001.zip › Supplementary Data Set S1/MF and Cancer.Data/PDF/1216369685/ehp.9195147.pdf]

Environmental Health Perspectives
Vol. 95, pp. 147-156, 1991

Extremely Low Frequency Electromagnetic Fields and Cancer: The Epidemiologic Evidence

by Michael N. Bates*

This paper reviews the epidemiologic evidence that low frequency electromagnetic fields generated by alternating current may be a cause of cancer. Studies examining residential exposures of children and adults and studies of electrical and electronics workers are reviewed. Using conventional epidemiologic criteria for inferring causal associations, including strength and consistency of the relationship, biological plausibility, and the possibility of bias as an explanation, it is concluded that the evidence is strongly suggestive that such radiation is carcinogenic. The evidence is strongest for brain and central nervous system cancers in electrical workers and children. Weaker evidence supports an association with leukemia in electrical workers. Some evidence also exists for an association with melanoma in electrical workers. Failure to find consistent evidence of a link between residential exposures and adult cancers may be attributable to exposure misclassification. Studies so far have used imperfect surrogates for any true biologically effective magnetic field exposure. The resulting exposure misclassification has produced relative risk estimates that understate any true risk.

Introduction

Since the discovery that electricity could be put to work in the service of mankind, people have been increasingly exposed to electromagnetic radiation as electrical transmission lines and electrically powered devices have proliferated. For a long time, such radiation was considered benign, although the evidence that it could have biological effects, at least in experimental systems, was increasing (*1*). It was not until 1979, with the publication of a study (*2*) associating 60-Hz electromagnetic fields with an increase in childhood cancer incidence, that concern began to mount. Since then, many other studies have investigated whether there is an association between exposure to such fields and cancer. This review considers the evidence from studies of direct or *in utero* exposure published in the peer-reviewed literature.

When alternating electric current (AC) flows, it generates an electromagnetic field of corresponding frequency (*3*). In North America, the AC frequency is 60 Hz, although in most other countries it is 50 Hz. These frequencies fall into the narrow range at one end of the electromagnetic spectrum, known as the extremely low frequency (ELF) region (up to 100 Hz). This spectrum, from the ELF region to gamma rays, covers a frequency range of at least 22 orders of magnitude (*4*). Between ELF and gamma rays, other frequency band designations include radio and television, radar and microwaves, infrared, visible light and ultraviolet rays, and X-rays. Different frequency ranges in the spectrum can have different biological effects. For example, exposure to the high-energy ionizing radiation at the high frequency end of the spectrum can cause cancer and other illnesses.

*Department of Biomedical and Environmental Health Sciences, School of Public Health, University of California, Berkeley, CA 94720.

ELF fields usually have both electric and magnetic field components. Electric fields are generated by any electrically charged body; magnetic fields are only generated when a current flows (*3*). Although both types of ELF field can induce very small, localized electric eddy currents in exposed individuals, the two field types have an important difference: Electric fields are easily shielded by virtually all electrically conducting materials, including buildings and human bodies; magnetic fields pass easily through almost all materials (*5*). Studies of the potential health effects of electromagnetic fields have concentrated on the magnetic field because it is generally assumed to be the component most likely to have biological effects.

Although the Earth's magnetic field has a 50 to 60 Hz component, it is several orders of magnitude weaker than the man-made fields to which people are commonly exposed. The main geomagnetic field component is static and, unlike time-varying ELF fields, does not induce flows of electric current in human bodies (*3*).

Measurements of Exposure and Their Limitations

Critical in the epidemiologic estimation of the magnitude of effect are measurements of exposure and, when the effect in question is cancer, historical measurements are particularly important. For ELF field effects, adequate historical data does not exist, and it is not known with any certainty what exactly is the biologically most important field component to consider even in current studies. For example, it is not known whether it is the peak field strength or cumulative exposure or if the relative orientation of the field is important. Added to that is the fact that

everybody is exposed to electromagnetic spectrum components from a multitude of sources, which are constantly changing over time and as one moves around. For this reason, all epidemiologic studies have found it necessary to use various estimators of exposure. In the studies discussed below, most such estimators fall into one of four categories: residential wire coding, direct field measurements, self-reports of appliance use, and occupational classifications. Some studies employ more than one method. However, all four estimators suffer from common limitations: They may estimate exposure either at home or at work, but take no account of exposure elsewhere, and their ability to provide reliable estimates of historical exposures is unknown.

Wire coding, used by Wertheimer and Leeper in their landmark study (*2*), involves a categorization of each dwelling according to the configuration of the electrical wires outside the residence and/or the nature of any electrical structures nearby (e.g., substations). Wire coding categories are ranked according to their estimated potential for generating magnetic field exposure. They have been shown to be correlated to some extent with ELF magnetic field measurements (*6,7*). A particular advantage of wire codings is that they may easily be assessed from the street, avoiding the necessity of entering the houses. In some studies, the wire codings are dichotomized by collapsing them into two categories, referred to as "high current configuration" (HCC) and "low current configuration" (LCC).

Most direct measurements of electric or magnetic fields have been made in or around residences. Usually these are one-time measurements, made at the time of interview. As such they do not necessarily reflect true individual exposure over time. Some studies have simply taken measurements at the front door of the residence; others have taken measurements in various rooms with most appliances either off or on.

Self-reports of exposures, particularly to chemicals, are a common method of assessing exposures in epidemiological studies. Some studies have used self-reports of household appliance use as a measure of ELF field exposure. The electric and magnetic fields emitted by most such appliances have been well characterized. Generally, however, most appliances are used intermittently and for short periods, and, when averaged over time, the overall exposure arising from their use is low. Electric blankets and water bed heaters give rise to relatively high ELF exposures because of more prolonged and intimate contact (*6*).

The use of occupational titles, commonly grouping occupations involved in electrical or electronic work, is the usual method of classifying workers as "exposed" or "unexposed." Usually these studies contain no measurements of electric or magnetic field exposures, although limited data show that electrical workers are more highly exposed to ELF fields than other workers (*8*). However, there is wide exposure variation among the various electrical and electronic job categories, and job titles are clearly imprecise estimators of exposure.

Studies Involving Residential Exposures

Studies examining childhood and adult cancers are considered below in the approximate chronological order of their publication. Selected risk estimates from these studies are summarized in Table 1. In Table 1, and in Tables 2–4, a variety of estimators of the relative risk are used. These are defined in the text. Some studies contain so many relative risk estimates for different situations and different subgroups that it is not possible to show them all, or even most, in the tables. In those cases an effort has been made to select the risk estimates that are the most representative of the studies in question.

Table 1. Studies involving residential exposures.

Reference	Study design	Exposure estimator	Cancer type[a]	Relative risk estimate		95% CI
Childhood cancers						
(*2*)	Case control	Wire code (birth addresses)	Leukemia	OR=2.28		1.97-2.65
			Nervous system	2.48		1.16-2.36
			Lymphoma	2.36		1.66-3.35
(*9*)	Case control	Wire code	Leukemia	OR=1.08		1.00-1.16
(*11*)	Case control	Field measurement	All	OR=2.12		1.73-2.59
			Nervous system	3.86		1.63-8.39
			Leukemia	0.34		0.17-0.68
(*12*)	Case control	Wire code	All	OR=1.53		1.04-2.26
			Nervous system	2.04		1.11-3.76
			Leukemia	1.54		0.90-2.63
(*13*)		Prenatal electric blanket use	Leukemia	1.7		0.8 -3.6
			Brain	2.5		1.1 -5.5
		Postnatal electric blanket use	Leukemia	1.5		0.5 -5.1
			AcLL	1.9		0.6 -6.5
			Brain	1.2		0.3 -5.7
Adult cancers						
(*6*)	Case control	Wire code	All	OR=1.39		1.21-1.58
(*18*)	Cohort	Distance	All	SMR=0.87	(men)	0.78-0.95
				SMR=0.92	(women)	0.83-1.01
			Leukemia	SMR=0.61	(men)	0.07-2.19
				SMR=1.54	(women)	0.42-3.94
(*19*)	Case control	Electric bed heaters	ANLL	OR=1.47		0.9 -2.4
(*20*)		Wire code		0.79		0.22-2.89
(*22*)	Case control	Electric blanket use	AML	OR=0.9		0.5 -1.6
			CML	0.8		0.4 -1.6
(*23*)	Case control	Distance to power lines	Leukemia	OR=1.45		0.54-3.88
		Distance to substation	Leukemia	0.99		—
(*24*)	Case control	Electric blanket use	Testicular	OR=1.0		0.7 -1.4

[a]AML, acute myelogenous leukemia; CML, chronic myelogenous leukemia; ANLL, acute nonlymphoid leukemia; AcLL, acute lymphoid leukemia.

Childhood Cancers

The first epidemiologic study to investigate a possible association between cancer and electromagnetic field exposure was a case–control study by Wertheimer and Leeper (*2*). Cases were 344 Denver, Colorado, persons who had died of cancer before the age of 19. Wire codes were assessed for both birth and "death" addresses.

From the data presented, it is possible to calculate odds ratios (ORs). These associate HCC exposure, at both birth and death addresses, with leukemia, lymphoma, and nervous system cancers (Table 1).

Fulton et al. (*9*) carried out a similar study of 119 Rhode Island leukemia cases with age of onset 0 to 20 years. An exposure index based on wire codes was assessed. No association between the exposure index and leukemia was found.

Wertheimer and Leeper (*10*) criticized the Fulton et al. study on several grounds. In particular, the addresses assessed for the controls were only the birth addresses, which were occupied in the 1950s. However, for the cases, the addresses assessed were all those occupied during their lifetimes. They argued that, because of migration to the suburbs in that period, the birth residences were more likely to have been urban addresses, which have a greater probability of being associated with HCC exposure. This would tend to bias the relative risk estimate downward, toward unity (i.e., no association). Wertheimer and Leeper recalculated the Rhode Island data, restricting the case and control addresses to those occupied by families from 1957 until diagnosis. This resulted in a moderate association between HCC exposure and leukemia (OR = 1.7, 95% confidence interval [CI]: 1.0–2.7).

Tomenius (*11*) carried out a case–control study of 716 Swedish cancer cases aged 0 to 18 years. Dwellings occupied at birth and at diagnosis were identified. For each of these, electrical structures occurring within 150 m were recorded (i.e., high voltage wires, substations, transformers, electric railroads, and subways). The 50-Hz magnetic field strength was measured outside the entrance to each dwelling.

The presence of electrical constructions within 150 m of a dwelling was associated with a slightly elevated cancer risk (OR = 1.3, $p < 0.05$). This association was stronger when only the presence of 200-kV wires was considered (OR = 2.1, $p < 0.05$). An elevated risk was also found when dwellings were dichotomized by the magnetic field strength measured outside the front door using a cut-point of 0.3 μT (microtesla) (OR = 2.12, 95% CI: 1.73–2.59). Paradoxically, this risk was even stronger for those dwellings where there were no visible electrical constructions (OR = 2.3, $p < 0.05$) and did not hold where 200-kV wires or other electrical constructions were present. However, the electric power distribution system in Sweden consists mostly of buried cables, rather than overhead lines. This may have accounted for these anomalous results if these cables generated most of the measured magnetic fields.

The risk of nervous system tumors was elevated in association with a front-door magnetic field strength of > 0.3 μT (OR = 3.86, 3.86, 95% CI: 1.63–8.39). For leukemia, the odds ratio was 0.3 (95% CI: 0.17–0.68), implying a protective effect of EMF exposure.

In a case–control study involving 356 childhood cancers from the Denver area, Savitz et al. (*12*) assessed residential wire codes and electric and magnetic field measurements inside homes. Magnetic field measurements obtained with appliances switched on were weakly associated with cancer incidence (test for trend: $p = 0.14$). No such trend was apparent for measurements with appliances off.

Wire codes were more strongly associated with cancer risk than field measurements, and there was a dose–response relationship.This association was strongest for homes occupied 2 years before diagnosis. When wire codes at time of diagnosis were dichotomized, significant associations were found for all cancers and for nervous system cancers (Table 1).

Parents were interviewed about exposures of their children to electric appliances. This produced some weak evidence of associations with leukemia and brain cancer for electric blanket use by the mother during pregnancy (prenatal exposure), and with acute lymphoid leukemia for electric blanket use by the child (postnatal) (*13*).

Finally, while not strictly the subject of this review, it should be mentioned that four studies have investigated the association of parental exposure to electromagnetic radiation with childhood neuroblastoma. This is a rare peripheral nervous system tumor with a median age of diagnosis of about 2 years. The first study (*14*) found an OR of 2.13 (95% CI: 1.05–4.35) for a combined group of parents with potential for electromagnetic exposure. The OR for electronic workers only was 11.75 (95% CI: 1.40–98.55). Three subsequent studies failed to confirm these associations (*15–17*). However, if these results were borne out by future studies it would raise the possibility that, for the other child cancer studies, the indices of residential exposure should actually be considered as measures of parental exposure. At this stage, however, the biologic plausibility of such a hypothesis seems low, as it would seem to require a mechanism by which ELF radiation produced mutations in specific genes of the gametes. As discussed later, ELF radiation has not been shown to cause damage to DNA. Therefore, the remainder of this review will proceed on the assumption that direct exposure of children to electromagnetic radiation is the event of interest.

Studies of Adult Cancers

Wertheimer and Leeper also conducted a case–control study in Colorado of 1179 adult cancers and ELF exposure (*6*). Wire codings were assessed for the address at which the case or control had spent most of the period from 3 to 10 years before diagnosis.

The odds ratio for cases (all cancers) having higher exposure wire codings than the controls was 1.39 (95% CI: 1.21–1.58). A dose–response relationship was also evident. Although no data were supplied, the authors stated that statistically significant associations were obtained with lymphomas and with cancers of the nervous system, uterus, and breast.

McDowall (*18*) identified a cohort of 7631 individuals living within 50 m of a substation or other electrical installation or within 30 m of an overhead power cable. Standardized mortality ratios (SMRs) showed no excess risks for all cancer or for specific cancers, except for female lung cancer (SMR = 1.75, 95% CI: 1.07–2.71) and leukemia mortality (SMR = 1.54, 95% CI: 0.42–3.94).

A case–control study of 114 nonlymphoid leukemia cases in Washington state used interviews, wire codings (for all addresses

Table 2. Studies involving leukemia and occupational exposures.

Reference	Occupation	Histological type	Relative risk estimate	95% CI
Studies that looked at leukemia only				
(*25*)	Telephone operators	All leukemia	SMR = 1.03	0.53–1.65
(*26*)	Electrical workers	All leukemia	PMR = 1.37	1.12–1.67
		Acute	1.63	1.14–2.25
(*28*)	Electrical workers	All leukemia	PIR = 1.29	0.85–1.88
		Acute	1.73	0.93–2.93
		Acute myeloid	2.07	1.02–3.75
(*29*)	Electrical workers	All leukemia	PMR = 0.98	0.78–1.21
		Lymphoid	1.00	0.66–1.45
		Myeloid	1.07	0.81–1.44
		Acute myeloid	OR = 2.1	1.3 –3.6
(*30*)	Electrical workers	All leukemia	PRR = 1.17	0.96–1.41
		Acute lymphoid	1.46	0.75–1.79
		Chronic lymphoid	1.29	0.89–1.81
		Acute myeloid	1.23	0.86–1.76
		Chronic myeloid	0.91	0.52–1.48
(*31*)	Electrical workers	All leukemia	OR = 1.70	0.97–2.97
		Acute myeloid	1.19	0.42–3.38
(*33*)	Amateur radio operators	All leukemia	PMR = 1.91	1.22–2.84
		Acute myeloid	2.89	1.61–4.55
		Chronic myeloid	2.67	0.72–6.82
(*34*)	Electrical engineers	All leukemia	PMR = 1.86	0.99–3.18
		Acute	2.57	1.11–5.06
(*35*)	Electrical workers	Acute myeloid	OR = 3.8	1.5 –9.5
(*36*)	Electricians	All leukemia	OR = 3.00	1.29–6.98
		Myeloid	2.33	0.77–7.06
		Lymphoid	6.00	1.47–24.45
	Welders	All leukemia	OR = 2.25	0.92–5.53
		Myeloid	3.83	1.28–11.46
(*32*)	Electrical workers	All leukemia	OR = 1.62	1.04–2.52
		Chronic	2.12	1.19–3.76
		Acute	1.25	0.62–2.54
		Lymphoid	1.73	0.89–3.37
		Myeloid	1.22	0.60–2.48
		Acute myeloid	1.16	0.48–2.84
(*37*)	Electrical workers	All leukemia	OR = 0.9	0.6 –1.3
		Acute myeloid	0.9	0.5 –1.8
Leukemia risks from studies involving all cancer types				
(*45*)	Electrical workers	All leukemia	PMR = 1.36	1.14–1.59
		Acute	1.62	1.26–2.08
(*46*)	Electrical engineers	All leukemia	SMR = 0.9	0.1 –3.2
(*49*)	Linesmen	All leukemia	SMR = 1.3	0.7 –2.1
(*50*)	Amateur radio operators	All leukemia	SMR = 1.24	0.87–1.72
		Acute myeloid	1.76	1.03–2.85

occupied within 15 years before the diagnosis), and indoor electric and magnetic field measurements to estimate exposures (*19,20*). Apart from a weak association with the use of electric bed heaters, none of the exposure surrogates provided evidence of an association with ELF field exposure.

After dichotomizing the wire codings, Wertheimer and Leeper (*21*) reanalyzed the data from this study taking into account the time of exposure. Their reanalysis suggested an association between acute nonlymphoid leukemia and both wiring configuration and the use of electrically heated beds. They found risks to be highest among the group that had had such exposure during the 3 years preceding diagnosis.

A case–control study of 116 acute myeloid leukemia (AML) and 108 chronic myeloid leukemia (CML) cases found no association with regular use of electric blankets (*22*). Another case–control study of 771 leukemia cases investigated the effect of living in proximity to electrical constructions (*23*). When distance to the residence was dichotomized at 100 m, the OR for power lines was 1.45 (95% CI: 0.54–3.88) and for substations 0.99. Finally, a case–control study of testicular cancer found no association with electric blanket use (OR = 1.0, 95% CI: 0.7–1.4) (*24*).

Studies Involving Occupational Exposure

A number of studies have examined cancer risks in occupational groups. Invariably these studies have used job classification as a surrogate for exposure. For convenience these studies are considered below in three categories: *a*) studies that have examined leukemia only; *b*) studies that have examined brain cancer; and *c*) studies that have looked at all cancers.

Studies That Have Examined Leukemia Only

A number of studies have specifically examined the association between leukemia and occupational exposures to electromagnetic radiation. Results of these studies are summarized in Table 2.

Wiklund et al. (*25*) investigated an alleged increased risk of leukemia in Swedish telephone operators, but found no associa-

tion (SMR = 1.03). The first publication suggesting that occupations involving exposure to electromagnetic fields may be associated with leukemia was that of Milham in 1982 (*26*). He found that 10 of 11 occupations in Washington state that were presumed to involve exposure to ELF field radiation had elevated proportionate mortality ratios (PMR) for all leukemia, with an overall PMR for the exposed occupations of 1.37 (95% CI: 1.12–1.67). For acute leukemia, 8 out of 11 occupations had elevated PMRs, with an overall PMR of 1.63 (95% CI: 1.14–2.25).

This study and a number of subsequent ones have used proportionate measures of effect (i.e., PMRs, proportional incidence ratios, and proportional registration ratios). These estimators of the relative risk have the potential to be misleading if there is incomplete ascertainment of deaths from all causes or if a particular cause of death is likely to be recorded preferentially. For example, if there was incomplete ascertainment of deaths other than from leukemia, then a PMR for leukemia might spuriously appear to be elevated (*27*). Odds ratios from case–control studies do not suffer from this potential problem.

Also in 1982, Wright et al. (*28*) published proportional incidence ratios (PIRs) for leukemia in white males in Los Angeles County. For occupations likely to be exposed to ELF field radiation, evidence of increased risks was found, particularly for AML.

A similar study by McDowall (*29*) also found elevated PMRs, particularly for myeloid leukemia in several electrical occupations. However, the number of cases in any individual occupation was very small, and overall PMRs (Table 2) were not raised. A case–control study of 537 AML deaths by the same author found an odds ratio of 2.1 (95% CI: 1.3–3.6) for electrical occupations (*29*).

An overall proportional registration ratio (PRR) estimate of 1.17 (95% CI: 0.96–1.41) was obtained for all leukemias in 10 electrical work occupations in England (*30*). Excesses were also found for acute lymphoid, chronic lymphoid, and acute myeloid leukemias.

In a New Zealand case-control study involving 546 male leukemia patients, Pearce et al. (*31,32*) found an excess for all leukemia in occupational groups with exposure to ELF fields (OR = 1.70, 95% CI: 0.97–2.97). This was entirely due to excesses for radio and television repairmen (OR = 8.17, 95% CI: 1.49–44.74) and electricians (OR = 4.75, 95% CI: 1.59–14.23). There was no convincing evidence of an elevated overall risk for AML (OR = 1.19, 95% CI: 0.42–3.28).

Milham (*33*) obtained death certificates for 1691 amateur radio operators from California and Washington state listed in the "Silent Keys" section of the American Radio Relay League's monthly magazine. The PMR for all leukemias was 1.91 (95% CI: 1.22–2.84). The excess risk was attributable to AML (SMR = 2.89, 95% CI: 1.61–4.55) and CML (SMR = 2.67, 95% CI: 0.72–6.82).

No excess leukemia mortality was found in men who had worked in electrical occupations in Wisconsin (*34*). However, electrical engineers had an increase in all leukemia (PMR = 1.86, 95 % CI: 0.99–3.18) and acute leukemia (PMR 2.57, 95% CI: 1.11–5.06). In a case–control study of 59 Swedish AML cases, electrical workers had an elevated risk (OR = 3.8, 95% CI: 1.5–9.5) (*35*).

A case–control study was carried out with 53 male leukemia cases who had worked in a shipyard between 1952 and 1977 (*36*). Associations with leukemia were found for having worked as an electrician or as an electric welder (Table 2).

Pearce et al. (*32*) found a leukemia OR for New Zealand electrical workers of 1.62 (95% CI: 1.04–2.52) in a case–control study. Contrary to most other studies, the ORs were higher for chronic and lymphatic leukemias than for acute and myeloid leukemias (Table 2). Also contrary to other findings, Loomis and Savitz (*37*), in a case-control study of U.S. men, found no elevated risk for total leukemia in electrical workers (OR = 0.9, 95% CI: 0.6–1.3) or for acute myeloid leukemia (OR = 0.9, 95% CI: 0.5–1.8). However, certain subgroups of electrical workers had elevated leukemia mortality, particularly electrical and electronic technicians (OR = 1.9) and electricians (OR = 1.8).

Studies of Brain Cancer Only

A number of studies (summarized in Table 3) have examined the relationship of brain cancer with electrical occupations. The first of these was a study of central nervous system neoplasms in Los Angeles County, California, which produced a PIR of 1.42 (95% CI: 0.71–2.54) for white male electricians (*38*).

Lin et al. (*39*) performed a case–control study with 951 white males who died of brain cancer in Maryland. Job types were categorized on the basis of likely exposure to ELF fields; i.e., definite, probable, possible, or no exposure. This was the first study to attempt to systematically categorize electrical jobs in terms of their degree of exposure. Cases and matched controls were analyzed in two separate groups according to whether there was a case diagnosis of glioma (519 pairs) or whether the type of brain tumor was unspecified (432 pairs). For both groups, a positive trend with increasing likelihood of exposure was found. This trend was strongest for the glioma group.

Thomas et al. (*40*) carried out a case–control study involving 435 brain tumor cases (including 300 gliomas). For occupations considered to have involved exposure to microwave and radio frequency radiation, the OR for all brain tumors was 1.6 (95% CI: 1.0–2.4). For those who had worked as electronics workers, the OR for gliomas was 4.6 (95% CI: 1.9–12.2), and for electrical tradesmen, the corresponding OR was 1.8 (95% CI: 0.8–3.9). For electronics workers, there was also an exposure-duration–response relationship for gliomas ($p < 0.05$). For electrical tradesmen, the exposure–duration–response relationship was much weaker. The latter group was regarded as having been exposed to ELF radiation, whereas the former group was exposed to very high frequency and ultra-high frequency electromagnetic radiation and may also have been exposed to chemicals, including soldering fumes and solvents.

A case–control study of east Texas residents who had died of glioma found men employed in occupations involving electromagnetic field exposure to have an elevated risk (OR = 3.94, 95% CI:1.52–10.20) (*41*). When degree of exposure was categorized according to the scheme of Lin et al. (*39*), a linear trend for risk associated with increasing exposure was found ($p < 0.01$), with ORs ranging from 1.15 to 2.86.

A New Zealand case-control study found no increased brain cancer risk for electrical workers overall (OR = 1.01, 95% CI: 0.56–1.82), although there were elevated risks for electrical engineers (OR = 4.74, 95% CI: 1.65–13.63) and electricians (OR = 1.91, 95% CI: 0.84–4.33) (*32*). Another case–control study of U.S. brain cancer cases found an OR of 1.5 (95% CI: 1.0–2.1),

Table 3. Studies of brain cancer and occupational exposures.

Reference	Occupation	Cancer type	Relative risk estimate	95% CI
Studies of brain cancer only				
(*38*)	Electricians	CNS	PIR=1.42	0.71- 2.54
(*39*)	Electrical workers	Glioma (no exposure)	OR=1.00	—
		(possible exposure)	1.44	1.06- 1.95
		(probable exposure)	1.95	0.94- 3.91
		(definite exposure)	2.15	1.10- 4.0
		Test for trend: $p < 0.05$		
(*40*)	Electronics workers	Glioma	OR=4.6	1.9 -12.2
	Electrical tradesmen		OR=1.8	0.8 - 3.9
(*41*)	Electrical workers	Glioma	OR=3.94	1.52-10.20
(*32*)	Electrical workers	Brain	OR=1.01	0.56- 1.82
	Electricians		1.91	0.84- 4.33
	Electrical engineers		4.74	1.65-13.63
(*37*)	Electrical workers	Brain	OR=1.5	1.0 - 2.1
(*42*)	Electrical workers	Astrocytoma	OR=10.3	1.3 -80.8
		Glioma	1.7	0.7 - 4.4
Brain cancer risks from studies of all cancer types				
(*44*)	Thermoelectric power workers	Brain	SMR=4.76	0.06-26.5
(*45*)	Electrical workers	Brain	PMR=1.23	1.00- 1.50
	Electricians		1.55	1.13- 2.05
(*46*)	Electrical engineers	Brain	SMR=1.0	0.1 - 3.7
(*47*)	Telecommunication workers	Nervous system	SMR=1.03	0.3 - 2.3
(*49*)	Linesmen	Nervous system	SMR=1.5	0.9 - 2.4
(*50*)	Amateur radio operators	Brain	SMR=1.39	0.93- 2.00

with the excess mainly found among electrical and electronic technicians (OR = 3.1) and electric power repairers and installers (OR = 2.4) (*37*). Finally, a Los Angeles case–control study found an OR of 10.3 (95% CI: 1.3–80.8) for astrocytoma associated with employment in electrical industries for more than 10 years. An increasing risk with length of employment was found (test for trend: $p = 0.01$) (*42*).

Studies of All Cancers

Some studies have examined the risks for any or all cancers in workers in electrical occupations. With the exception of the risks for leukemia and brain cancer (see Tables 2 and 3), the results of these studies are summarized in Table 4.

Vagero and Olin (*43*) studied cancer risks in the Swedish electronics industry. Elevated risks of cancer were found for a number of sites, including melanoma (Table 4), but not leukemia. As the authors point out, studying the electronics industry as a whole is likely to obscure any causal associations occurring in particular occupational subgroups. Also, work categories were classified on the basis of the job reported at the time of the 1960 census. Thus, misclassification of exposure status is likely to bias relative risk estimates toward unity.

Cammarano et al. (*44*) studied mortality in 270 men who had worked in an Italian thermoelectric power plant for at least 6 months. Fifteen cancer deaths were distributed over a number of sites (no leukemias), and an excess was found in those who had worked at the plant for over 10 years (SMR = 2.76, 95% CI: 1.43–4.82).

In a PMR study of men who had been employed in occupations with presumed electrical exposure in Washington State, Milham (*45*) found significantly elevated risks for a number of cancers including cancers of the pancreas, respiratory tract and brain, as well as lymphomas and leukemias (particularly acute leukemia) (Tables 2 and 4).

Olin et al. (*46*) studied mortality in 1254 Swedish electrical engineers. Overall cancer mortality was only half that expected and, with the exception of malignant melanoma (SMR = 3.2, 95% CI: 0.7–9.4), all relative risk estimates were below unity.

The same authors (*47*) carried out a cancer incidence study of Swedish telecommunications workers. The 2918 subjects had worked for the company for at least 6 months during the period 1956 to 1960. SMRs were generally unremarkable, except for an excess of malignant melanoma (SMR = 2.5, 95% CI: 1.1–4.9). This association increased when cases were limited to those who had worked in soldering departments and who had at least 3 years of exposure (SMR = 3.9, 95% CI: 1.4–8.5).

An increased mortality from melanoma was also found in a cohort of 1807 British semiconductor workers (SMR = 4.4, 95% CI: 1.58–15.05) (*48*). However, it is not clear whether this occupation involves substantial exposure to ELF radiation.

In a cohort study of 3358 Swedish power linesmen and 6703 power station operators, a total of 699 cancer cases were identified, including 26 leukemias (*49*). No significant excesses of any cancers were found in either group.

Following up his earlier study of mortality in amateur radio operators (*33*), Milham (*50*) identified 2485 deaths during 1979 to 1984 of individuals listed in the 1984 Federal Communications Commission Amateur Radio Station and/or Operator License File. Mortality from all malignancies was low (SMR = 0.89). However, mortality from all leukemias was elevated (SMR = 1.24, 95% CI: 0.87–1.72), as was mortality from AML (SMR = 1.76, 95% CI: 1.03–2.85) and brain cancer (SMR = 1.39, 95% CI: 0.93–2.0).

Discussion

The evidence for a causal association between ELF electromagnetic fields and cancer is appraised below in terms of several widely accepted epidemiologic criteria for inferring cause-and-effect relationships. None of these criteria are

Table 4. Studies of all cancer types and occupational exposures (results for leukemias and brain cancers are presented in the Tables 2 and 3).

Reference	Occupation	Cancer type	Relative risk estimate	95% CI
(*43*)	Electronics workers	All	RR=1.15	1.10-1.20
		Larynx	1.46	1.05-2.03
		Mesopharynx	2.30	1.11-4.79
		Lung	1.52	1.35-1.72
		Colon	1.20	1.02-1.43
		Bladder	1.22	1.02-1.47
		Cervix	1.14	1.04-1.26
		Melanoma	1.35	1.05-1.76
(*44*)	Thermoelectric power workers	All	SMR=2.76	1.43-4.82
(*45*)	Electrical workers	Pancreas	PMR=1.17	1.00-1.35
		Respiratory	1.14	1.06-1.22
		Lymphomas	1.64	1.22-2.16
(*46*)	Electrical engineers	All	SMR=0.5	0.3 -0.7
		Melanoma	3.2	0.7 -9.4
(*47*)	Telecommunication workers	All	SMR=1.03	0.8 -1.2
		Melanoma	2.5	1.1 -4.9
(*48*)	Semiconductor workers	All	SMR=1.03	0.76-1.36
		Melanoma	4.4	1.58-15.05
(*49*)	Linesmen	All	SMR=1.1	1.0 -1.2
(*50*)	Amateur radio operators	All	SMR=0.89	0.82-0.95

necessary preconditions for a judgment of causality. However, each may add considerable weight for or against the existence of a causal association.

Strength of the Association

This criterion is usually assessed in terms of the magnitude of the relative risk estimates. The closer the estimates to unity, the weaker the associations and the more likely they might be accounted for by chance or bias (see below). Most of the relative risk estimates in Tables 1–4 fall below 3, and many below 2. The most strikingly elevated risks are for cancers of the brain or nervous system, both for children and occupationally exposed adults. Risk estimates for leukemia in occupational groups are generally lower and many are proportional measures, which may be particularly subject to selection bias (see below).

In all studies, the exposure estimators were crude. Thus, it is certain there will be considerable misclassification of the degree of individual exposure, although in most cases there is no obvious reason to suggest that this would be differential between cases and controls. [Possible exceptions to this are studies (*2,6*) in which wire coding of residences was not done blind to case or control status). The usual effect of nondifferential exposure misclassification is to bias relative risk estimates toward unity; in other words, to cause these estimates to understate the true risk of exposure to ELF electromagnetic fields.

Consistency

Provided they cannot be explained in terms of a common bias, relative risk estimates consistently raised across a number of studies may provide powerful evidence in favor of a causal association (or, conversely, for a protective effect if estimates are less than unity). The most consistent finding in the four studies of childhood tumors (Table 1) is elevated risks of tumors of the nervous system (all statistically significant) found by the three studies which included cases of this tumor (*2,11–13*). All four studies estimated leukemia risk, which was elevated in two (*2,12,13*). Another study (*9*) showed no association, while one indicated a protective effect (*11*). All three studies that included all childhood tumors found increased overall risks.

As a group, the six studies that have examined the association of adult cancers with residential ELF radiation exposure show no pattern of increased cancer risks (Table 1). One study (*6*) found a small, but statistically significant, increase for the risks of all tumors combined, and a dose–response relationship was apparent. Risks of lymphoma and cancers of the breast, uterus, and nervous system were reported to be significantly elevated, but no data were presented. Another study found a weak, statistically nonsignificant association between nonlymphoid leukemia and electric bed-heater use (*19*). In the same study, both wire codes and ELF field measurements produced no evidence of such an association.

The possibility must be considered that the reason no consistent adult effect has been found is that residential measures of exposure represent only a small proportion of the biologically effective ELF dose that most adults receive. If the bulk of adult exposures are received at work and elsewhere, then it would be no surprise that the relatively crude measures used to assess residential exposure have generally been insufficient for an effect to be detected. On the other hand, children have spent a greater proportion of their lives around the home and, therefore, they are probably subject to less exposure misclassification than adults.

Twenty-seven studies of occupational exposure have been reviewed (Tables 2–4). Twelve of these looked specifically at leukemia risks (*25,26,28–37*). With the exception of one study (*25*), these all found elevated leukemia risks in at least certain electrical occupations. Such elevations were sometimes confined to histological subtypes, particularly AML. Some occupations showed evidence of higher risks than others, although this varied from study to study.

Seven studies (*32,37–42*) looked specifically at risks of brain or nervous system cancer. All seven produced evidence of an association with electromagnetic radiation exposure in at least some occupational categories.

Eight studies looked at risks associated with electrical occupations for all cancer types (Table 4). Three of these studies

showed some evidence of elevated risks for leukemia (*45,49,50*), and four produced increased risks of cancer of the brain or nervous system (*44,45,49,50*) (Tables 2 and 3). In addition, four of these studies showed an increase in the risks of malignant melanoma (*43,46–48*). In one study the risks of a wide range of cancers were raised (*43*).

In summary, the most consistent findings are increased risks of leukemia and brain cancer among electrical workers. There are also consistent increases in brain cancers among children with higher exposures to electromagnetic fields. Some consistency also exists in the evidence for an association between malignant melanoma and electrical work.

Dose–Response Relationships

It is generally accepted that in a true causal association, higher exposures to the causal agent will be associated with greater risks. Although most studies reviewed did not mention having examined this possibility, six did report dose–response relationships based on either estimated magnitude of exposure or likelihood of exposure: four investigations of cancers of the brain and nervous system associated with occupational exposures (*39–42*), one study of adult cancers (*6*), and one of childhood cancers (*12*).

Bias

Low relative risk estimates (less than, say, 2) are subject to the suspicion that they may be attributable to some systematic bias in the data or their analysis. The various types of epidemiologic bias can be grouped under three general headings: confounding bias, selection bias and information bias.

Confounding Bias. This bias occurs when a causal factor for the disease is also correlated with the exposure measure of interest. This can lead to finding spurious associations between the disease and the exposure measure. Provided the confounding factor has been measured, its presence can be taken into account in the analysis.

Looking first at the occupational studies, several possible confounders have not yet been ruled out. Perhaps the most likely is a chemical widely used in electrical or electronic work and causal for, say, leukemia. Benzene, a solvent widely used in the past, is a known leukemogen, particularly for acute myeloid leukemia (*51*). PCBs, which are established animal carcinogens, have been widely used as dielectrics in transformers and capacitors. Most occupational studies reviewed had insufficient data to permit adjustment for exposure to solvents or other chemicals. Nor was smoking generally adjusted for, although there is evidence that smoking may be a cause of leukemia, particularly AML (*52,53*).

Another possibility is electromagnetic radiation other than in the ELF range. Studies have focused on the ELF range largely because of the first study involving residential exposure (2). However, this does not preclude the possibility that other frequency ranges may be more carcinogenic. Thomas et al. (*40*) provided suggestive evidence for this when they showed that occupations more likely to be exposed to radio frequency and microwave radiation had higher brain cancer risks than those occupations primarily exposed to ELF radiation. This suggestion receives some support from a meta-analysis (*54*), which found consistently elevated leukemia risks across studies for telegraph, radio and radar operators. For this group, the pooled relative risk estimate for leukemia across studies was 1.8 (95% CI: 1.4–2.6) and for AML, 2.6 (95% CI: 1.4–4.4).

It is less easy to postulate confounding factors for the residential exposures because it is not obvious what would correlate with wire codes or ELF field measurements and, at the same time, be causal for the cancers in question. To explain the findings with wire codes, any confounding factor would have to be much more strongly associated with wire codes than are ELF field measurements (*4*). Two possibilities are traffic density and water quality. The former may occur if wires carrying higher current loads are more likely to run along main thoroughfares. Savitz et al. (*12*) adjusted for traffic load and found it had no effect on risk estimates.

Residential electric power supplies are often grounded through the domestic plumbing system. Such currents might have an electrolytic effect on pipe materials, releasing various metals into the water supply. This possibility has not been tested in any of the studies to date. However, no metals have yet been implicated as causes of brain cancer (or leukemia).

Selection Bias. When there is a systematic difference between the subjects selected for study and those not selected, selection bias occurs. This bias is of most concern in the studies, mostly of leukemia in electrical workers, which used proportionate measures of effect (PMRs, PIRs, and PRRs). These may be biased if all causes of death in the cohort are not equally liable to be ascertained, or if the so-called healthy worker effect is operating, such that incidence of or mortality from most other diseases in the cohort is low. In one study the controls were selected differently from the cases (*9*). This could have obscured an association with childhood leukemia.

One further possibility is publication bias. The overall findings of this review may be distorted if studies that find associations are more likely to be published than those that do not. This bias is very difficult to assess. However, in an area of such widespread interest the results of at least large studies are unlikely to go unpublished, whatever their outcome.

Information Bias. This bias occurs when the quality of information on exposure or disease outcome is unsatisfactory, leading to misclassification of subjects in the statistical analysis. Such a bias has certainly occurred in the studies reviewed here, because the estimates of ELF field exposures were imperfect surrogates for the true ELF exposures. It seems probable that in most studies such a bias would have been nondifferential between cases and controls. This would lead to underestimates of any true risks associated with ELF exposure.

Biological Plausibility

The plausibility of a putative cause and effect relationship is enhanced when results of experimental animal or *in vitro* studies support the epidemiologic findings. However, it has not so far been demonstrated that ELF field exposures are carcinogenic in experimental animals or genotoxic in *in vitro* test systems. Thus, the hypothesis associating human cancers and ELF fields may to some extent be regarded as lacking in experimental support. This is not altogether unprecedented: Inorganic arsenic is a well-established human carcinogen, but has not been shown convincingly to cause cancer in animals, or point mutations in *in vitro* systems. However, unlike ELF field exposure, arsenic has been

shown to increase the rate of chromosomal abnormalities and sister chromatid exchange when it is present during DNA replication (*55*).

Experimental studies have shown that exposure to electromagnetic fields can have biological effects other than carcinogenesis (although the replicability of some of these studies may be in question). These include changes in calcium efflux from cell walls, effects on trout fertility, alterations in circadian rhythms in several species, effects on pineal gland function and melatonin content in rats, and alterations in heart rate in humans (*1,3,4*). These have led to the development of new hypotheses about how cancer might be caused by ELF exposure. It seems likely that if ELF field exposure is carcinogenic, then it is probably acting through a promotional mechanism, rather than as an initiator.

Conclusions

The evidence for a carcinogenic effect of ELF radiation is strongly suggestive, although not yet conclusive. The evidence is strongest for brain cancer in both exposed workers and children. The relative risk estimates are sufficiently consistent, elevated, and precise that they are unlikely to be explained by chance or bias. The finding of dose–response relationships in four occupational brain cancer studies further increases the likelihood of a causal connection. It may be more than a coincidence that between 1968 and 1987 mortality rates from cancers of the brain have approximately doubled in persons aged 65 and older in seven industrialized countries (*56*).

The evidence for leukemia is less strong. There is an impressive consistency in the occupational studies in the direction of relative risk estimates for leukemia, particularly acute myeloid leukemia. However, these risk estimates are generally low, and the possibility that they might be explained by confounding or selection bias cannot be ruled out. So far the evidence for childhood leukemia is weak, although several studies did find elevated relative risk estimates. However, the studies are relatively few and such findings could easily be due to chance.

The evidence for a carcinogenic effect from adult residential exposures is inconsistent and unconvincing at present. However, this might be attributable to exposure misclassification. Risks for malignancies other than brain cancer and leukemia cannnot be ruled out on the basis of current evidence. The evidence for melanoma in several occupational studies is particularly suggestive. However, more narrowly focused studies will be required if other cancers are to be properly investigated.

As noted above, the exposure measures that have been used in the epidemiologic studies to date are imprecise surrogates for a true biologically effective exposure, which remains unidentified. The resulting exposure misclassification will certainly have depressed any truly elevated relative risk estimates toward the null value. It is perhaps remarkable, given the uncertainties in our understanding of the exposure of interest, that elevated risks have been detected at all. For that reason, if a causal relationship between ELF fields and cancer is eventually confirmed, the true relative risks will almost certainly turn out to be appreciably higher than those listed above. Further epidemiological studies will need to concentrate on developing more appropriate measures of exposure to define what these risks might be.

The author expresses appreciation for valuable comments and advice from Mark Mendell and Irva Hertz-Picciotto, Leeka Kheifets, Geri Lee, Raymond Neutra, Neil Pearce, David Savitz, Allan Smith, and Michael Yost. This work was supported by the Toxic Substances Research and Teaching Program of the University of California.

REFERENCES

1. Papatheofanis, F. J. Bioelectromagnetics: Biophysical Principles in Medicine and Biology. Karger, New York, 1987.
2. Wertheimer, N., and Leeper, E. Electrical wiring configuration and childhood cancer. Am. J. Epidemiol. 109: 273–284 (1979).
3. World Health Organization. Magnetic Fields. Environmental Health Criteria 69, WHO, Geneva, 1987.
4. Savitz, D. A., Pearce, N. E., and Poole, C. Methodological issues in the epidemiology of electromagnetic fields and cancer. Epidemiol. Rev. 11: 59–78 (1989).
5. World Health Organization. Extremely low frequency (ELF) fields. Environmental Health Criteria 35, WHO, Geneva, 1984.
6. Wertheimer, N., and Leeper, E. Adult cancer related to electrical wires near the home. Int. J. Epidemiol. 11: 345–355 (1982).
7. Kaune, W. T., Stevens, R. G., Callahan, N. J., Severson, R. K., and Thomas, D. B. Residential magnetic and electric fields. Bioelectromagnetics 8: 315–335 (1987).
8. Bowman, J. D., Garabant, D. H., Sobel, E., and Peters, J. M. Exposures to extremely low frequency (ELF) electromagnetic fields in occupations with elevated leukemia rates. Appl. Ind. Hyg. 3: 189–194 (1988).
9. Fulton, J. P., Cobb, S., Preble, L., Leone, L., and Forman, E. Electrical wiring configurations and childhood leukemia in Rhode Island. Am. J. Epidemiol. 111: 292–296 (1980).
10. Wertheimer, N., and Leeper, E. Electrical wiring configurations and childhood leukemia in Rhode Island. (Letter to the Editor). Am. J. Epidemiol. 111: 461–462 (1980).
11. Tomenius, L. 50-Hz electromagnetic environment and the incidence of childhood tumors in Stockholm County. Bioelectromagnetics 7: 191–207 (1986).
12. Savitz, D. A., Wachtel, H., Barnes, F. A., John, E. M., and Tvrdik, J. G. Case-control study of childhood cancer and exposure to 60-Hz magnetic fields. Am. J. Epidemiol. 128: 21–38 (1988).
13. Savitz, D. A., John, E. M., and Kleckner, R. C. Magnetic field exposure from electrical appliances and childhood cancer. Am. J. Epidemiol. 131: 763–773 (1990).
14. Spitz, M. R., and Johnson, C. C. Neuroblastoma and parental occupation: a case-control analysis. Am. J. Epidemiol. 121: 924–929 (1990).
15. Nasca, P. C., Baptiste, M. S., MacCubbin, P. A., Metzger, B. B., Carlton, K., Greenwald, P., Armbrustmacher, V. W., Earle, K. M., and Waldman, J. An epidemiologic case-control study of central nervous system tumors in children and parental occupational exposures. Am. J. Epidemiol. 128: 1256–1265 (1988).
16. Bunin, G. R., Ward, E., and Kramer, S. Neuroblastoma and parental occupation. Am. J. Epidemiol. 131: 776–780 (1990).
17. Wilkins, J. R., and Hundley, V. D. Paternal occupational exposure to electromagnetic fields and neuroblastoma in offspring. Am. J. Epidemiol. 131: 995–1008 (1990).
18. McDowall, M. E. Mortality of persons resident in the vicinity of electricity transmission facilities. Br. J. Cancer 53: 271–279 (1986).
19. Stevens, R. G. Epidemiological studies of cancer and residential exposure to electromagnetic fields. Contractor's final report, Part I. New York State Power Lines Project Contract #218218, Albany, NY, 1987.
20. Severson, R. K., Stevens, R. G., Kaune, W. T., Thomas, D. B., Heuser, L., Davis, S., and Sever, L. E. Acute nonlymphocytic leukemia and residential exposure to power frequency magnetic fields. Am. J. Epidemiol. 128: 10–20 (1988).
21. Wertheimer, N., and Leeper, E. Acute nonlymphoid leukemia and residential exposure to power frequency magnetic fields. (Letter to the Editor). Am. J. Epidemiol. 130: 423–425 (1989).
22. Preston-Martin, S., Peters, J. M., Yu, M. C., Garabrant, D. H., and Bowman, J. D. Myelogenous leukemia and electric blanket use. Bioelectromagnetics 9: 207–213 (1988).
23. Coleman, M. P., Bell, C. M. J., and Primic-Zakelj, M. Leukemia and residence near electricity transmission equipment: a case-control study. Br. J. Cancer 60: 793–798 (1989).
24. Verreault, R., Weiss, N. S., and Hollenbach, K. A. Use of electric blankets and testicular cancer. Am. J. Epidemiol. 131: 759–762 (1990).

25. Wiklund, K., Einhorn, J., and Eklund, G. An application of the Swedish Cancer-Environment Registry. Leukemia among telephone operators at the Telecommunications Administration in Sweden. Int. J. Epidemiol. 10: 373-376 (1981).
26. Milham, S. Mortality from leukemia in workers exposed to electrical and magnetic fields. (Letter to the Editor). N. Engl. J. Med. 307: 249 (1982).
27. Checkoway, H., Pearce, N. E., and Crawford-Brown, D. J. Research Methods in Occupational Epidemiology. Oxford University Press, New York, 1989.
28. Wright, W. E., Peters, J. M., and Mack, T. M. Leukemia in workers exposed to electrical and magnetic fields. (Letter to the Editor). Lancet ii: 1160-1161 (1982).
29. McDowall, M. E. Leukemia mortality in electrical workers in England and Wales. (Letter to the Editor). Lancet i: 246 (1983).
30. Coleman, M., Bell, J., and Skeet, R. Leukemia incidence in electrical workers. (Letter to the Editor). Lancet i: 982-983 (1983).
31. Pearce, N. E., Sheppard, R. A., and Howard, J. K. Leukemia in electrical workers in New Zealand. Lancet i: 811-812 (1985).
32. Pearce, N., Reif, J., and Fraser, J. Case-control studies of cancer in New Zealand electrical workers. Int. J. Epidemiol. 18: 55-59 (1989).
33. Milham, S. Silent keys: Leukemia mortality in amateur radio operators. (Letter to the Editor). Lancet i: 812 (1985).
34. Calle, E., and Savitz, D. A. Leukemia in occupational groups with presumed exposure to electrical and magnetic fields. (Letter to the Editor). N. Engl. J. Med. 313: 1476-1477 (1985).
35. Flodin, U., Fredriksson, M., Axelson, O., Persson, B., and Hardell, L. Background radiation, electrical work, and some other exposures associated with acute myeloid leukemia in a case-referent study. Arch. Environ. Health 41: 77-84 (1986).
36. Stern, F. B., Waxweiler, R. A., Beaumont, J. J., Lee, S. T., Rinsky, R. A., Zumwalde, R. D., Halperin, W. E., Bierbaum, P. J., Landrigan, P. J., and Murray, W. E. A case-control study of leukemia at a naval nuclear shipyard. Am. J. Epidemiol. 123: 980-992 (1986).
37. Loomis, D. P., and Savitz, D. A. Brain cancer and leukemia mortality among electrical workers (abstract). In: Abstracts of the Society for Epidemiologic Research, 22nd Annual Meeting, 1989, Birmingham, AL. Society for Epidemiological Research, Baltimore, MD.
38. Preston-Martin, S., Henderson, B. E., and Peters, J. M. Descriptive epidemiology of central nervous system neoplasms in Los Angeles County. Ann. N.Y. Acad. Sci. 381: 202-208 (1982).
39. Lin, R. S., Dischinger, P. C., Conde, J., and Farrell, K. P. Occupational exposure to electromagnetic fields and the occurrence of brain tumors. J. Occup. Med. 27: 413-419 (1985).
40. Thomas, T. L., Stolley, P. D., Stemhagen, A., Fontham, E. T. H., Bleecker, M. L., Stewart, P. A., and Hoover, R. N. Brain tumor mortality risk among men with electrical and electronics jobs: a case-control study. J. Natl. Cancer Inst. 79: 233-238 (1987).
41. Speers, M. A., Dobbins, J. G., and Miller, V. S. Occupational exposures and brain cancer mortality: a preliminary study of East Texas residents. Am. J. Ind. Med. 13: 629-638 (1988).
42. Preston-Martin, S., Mack, W., and Peters, J. Astrocytoma risk related to job exposure to electric and magnetic fields (abstract). In: Abstracts of the Society for Epidemiologic Research, 23rd Annual Meeting, 1990, Snowbird, UT. Society for Epidemiologic Research, Baltimore, MD.
43. Vagero, D., and Olin, R. Incidence of cancer in the electronics industry: using the new Swedish Cancer Environment Registry as a screening instrument. Br. J. Ind. Med. 40: 188-192 (1983).
44. Cammarano, G., Crosignani, P., Berrino, F., and Berra, G. Cancer mortality among workers in a thermoelectric power plant. Scand. J. Work Environ. Health 10: 259-261 (1984).
45. Milham, S. Mortality in workers exposed to electromagnetic fields. Environ. Health Perspect. 62: 297-300 (1985).
46. Olin, R., Vagero, D., and Ahlbom, A. Mortality experience of electrical engineers. Br. J. Ind. Med. 42: 211-212 (1985).
47. Vagero, D., Ahlbom, A., Olin, R., and Sahlsten, S. Cancer morbidity among workers in the telecommunications industry. Br. J. Ind. Med. 42: 191-195 (1985).
48. Sorahan, T., Waterhouse, J. A. H., McKiernan, M. J., and Aston, R. H. Cancer incidence and cancer mortality in a cohort of semiconductor workers. Br. J. Ind. Med. 42: 546-550 (1985).
49. Tornqvist, S., Norell, S., Ahlbom, A., and Knave, B. Cancer in the electric power industry. Br. J. Ind. Med. 43: 212-213 (1986).
50. Milham, S. Increased mortality in amateur radio operators due to lymphatic and hematopoietic malignancies. Am. J. Epidemiol. 127: 5054 (1988).
51. Savitz, D. A., and Pearce, N. E. Occupational leukemias and lymphomas. Sem. Occup. Med. 2: 283-289 (1987).
52. Severson, R. K., Davis, S., Heuser, L., Daling, J. R., and Thomas, D. B. Cigarette smoking and acute non-lymphocytic leukemia. Am. J. Epidemiol. 132: 418-422 (1990).
53. McLaughlin, J. K., Hrubec, Z., Lint, M. S., Heineman, E. F., Blot, W. J., and Fraumeni, J. F. Cigarette smoking and leukemia. J. Natl. Cancer Inst. 81: 1262-1263 (1989).
54. Savitz, D. A., and Calle, E. E. Leukemia and occupational exposure to electromagnetic fields: review of epidemiologic surveys. J. Occup. Med. 29: 47-51 (1987).
55. Jacobson-Kram, D., and Montalbano, D. The Reproductive Effects Assessment Group's Report on the mutagenicity of inorganic arsenic. Environ. Mutagen. 7: 787-804 (1985).
56. Davis, D. L., Hoel, D., Fox, J., and Lopez, A. International trends in cancer mortality in France, West Germany, Italy, Japan, England and Wales, and the USA. Lancet 336: 474-481 (1990).
