# Supplementary material for: Magnetic Fields and Cancer: Epidemiology, Cellular Biology, and Theranostics
Source: Int J Mol Sci. 2022 Jan 25;23(3):1339. doi: 10.3390/ijms23031339 (PMC8835851; doi:10.3390/ijms23031339)
Supplement: Supplementary file 1 [file ijms-23-01339-s001.zip › Supplementary Data Set S1/MF and Cancer.Data/PDF/1243332475/1-s2.0-S0269749116309526-main.pdf]

Environmental Pollution 221 (2017) 501–505

Contents lists available at ScienceDirect

Environmental Pollution

journal homepage: www.elsevier.com/locate/envpol

Letter to the Editor

When theory and observation collide: Can non-ionizing radiation cause cancer?☆

Keywords:
Non-ionizing radiation
Oxidative stress
Free radicals
Cancer

ABSTRACT

This paper attempts to resolve the debate about whether non-ionizing radiation (NIR) can cause cancer—a debate that has been ongoing for decades. The rationale, put forward mostly by physicists and accepted by many health agencies, is that, "*since NIR does not have enough energy to dislodge electrons, it is unable to cause cancer.*" This argument is based on a flawed assumption and uses the model of ionizing radiation (IR) to explain NIR, which is inappropriate. Evidence of free-radical damage has been repeatedly documented among humans, animals, plants and microorganisms for both extremely low frequency (ELF) electromagnetic fields (EMF) and for radio frequency (RF) radiation, neither of which is ionizing. While IR directly damages DNA, NIR interferes with the oxidative repair mechanisms resulting in oxidative stress, damage to cellular components including DNA, and damage to cellular processes leading to cancer. Furthermore, free-radical damage explains the increased cancer risks associated with mobile phone use, occupational exposure to NIR (ELF EMF and RFR), and residential exposure to power lines and RF transmitters including mobile phones, cell phone base stations, broadcast antennas, and radar installations.

© 2016 Elsevier Ltd. All rights reserved.

1. Introduction

Whether power frequency electromagnetic fields (EMF), radiofrequency (RF) and microwave (MW) radiation can cause cancer, or other health effects, has been debated since the 1960s.[1] Scientists, who study electromagnetic energy, find themselves aligned with one of two groups and these two groups are becoming increasingly polarized.

One group adheres to the concept that, the only harmful effects associated with RF and MW radiation are due to heating, and that below thermal guidelines, this energy is safe (see quotes below). They state that the scientific evidence documenting adverse health effects is inconsistent and inconclusive and, while certain types of cancers can't be ignored, for example, childhood leukemia with residential magnetic field exposure (Ahlbom et al., 2001), the risks are small and may be due to confounders. This group relies on the well-established theory that non-ionizing radiation (NIR) does not have enough energy to dislodge electrons and therefore is unable to cause cancer.

Key authorities have made the following statements regarding health effects of NIR:

ICNIRP 2016—*The overall evaluation of all the research on HF* [*high frequency*] *fields leads to the conclusion that HF exposure below the thermal threshold is unlikely to be associated with adverse health effects*. [Note: ICNIRP (1998) recommends NIR guidelines to the WHO.]

National Cancer Institute, U.S. 2016—*Radiofrequency energy, unlike ionizing radiation, does not cause DNA damage that can lead to cancer. Its only consistently observed biological effect in humans is tissue heating.*

WHO, 2014—*A large number of studies have been performed over the last two decades to assess whether mobile phones pose a potential health risk. To date, no adverse health effects have been established as being caused by mobile phone*[2] *use.*

Health Protection Agency, U.K. 2012—*In summary, although a substantial amount of research has been conducted in this area* [*i.e. radiofrequency radiation*], *there is no convincing evidence that RF field exposure below guideline levels causes health effects in adults or children.*

Health Canada, 2010—*Based on scientific evidence, Health Canada has determined that low-level exposure to radiofrequency (RF) energy from Wi-Fi equipment is not dangerous to the public. This conclusion is consistent with the findings of other international bodies and regulators.*

☆ This paper has been recommended for acceptance by David Carpenter.

[1] NOTE: In this document ELF refers to frequencies below 300 Hz and RF refers to frequencies up to 300 GHz. MW are radio frequencies between 300 MHz and 300 GHz.

[2] NOTE: WHO makes the same statement about cell phone base stations, DECT phones, and Wi-Fi.

http://dx.doi.org/10.1016/j.envpol.2016.10.018
0269-7491/© 2016 Elsevier Ltd. All rights reserved.

FCC, 2010—*There is no scientific evidence to date that proves that wireless phone usage can lead to cancer or a variety of other health effects, including headaches, dizziness or memory loss.*

New Zealand Ministry for the Environment, 2008—*The Ministry of Health considers there are no established adverse effects from exposures to radiofrequency fields which comply with the ICNIRP guidelines and the New Zealand Standard.*

Swedish Radiation Protection Authority, 2002— *there is no biologically plausible mechanism to support a carcinogenic effect of non-ionizing RF waves.*

Clearly leading authorities state that RFR is safe below thermal guidelines and the public has nothing to be concerned about.

The other group believes that NIR is harmful at levels well below thermal guidelines, levels that are now ubiquitous in urban centers. They believe that NIR causes cancer, reproductive problems, and a range of symptoms that have been classified as electrohypersensitivity (EHS) or idiopathic environmental intolerance according to the WHO; that children and pregnant women are particularly vulnerable; and that many of the chronic illnesses common in our society are due, in part, to electromagnetic pollution or electrosmog[3] exposure. They base this belief on the numerous studies documenting adverse biological and health effects of low-level NIR (Lee et al., 1996; Havas, 2000, 2013; Carpenter and Sage, 2007, 2012; Levitt and Lai, 2010; Blank et al., 2015).

Which group is right and how do we move beyond this impasse?

2. Discussion

Let's start with the statement that: *Non-ionizing radiation doesn't have enough energy to dislodge electrons and thus cannot cause cancer.*

This assertion consists of two parts. The first part (*non-ionizing radiation doesn't have enough energy to dislodge electrons*) is based on photon energy and electromagnetic forces. The second part (*and thus cannot cause cancer*) is a conclusion based on the assumption that radiation can cause cancer *only* by dislodging electrons and breaking chemical bonds. This assumption is flawed.

Models of chemicals and ionizing radiation (IR) are repeatedly and inappropriately used to interpret the effects of NIR. With IR, photon energy is the critical criterion. With chemical toxicants, speciation and the dose are critical. With NIR we have evidence of effects within narrow intensity and frequency windows and evidence that waveform and modulation are biologically important (Blackman et al., 1989; Litovitz et al., 1990; Wei et al., 1990; Adey, 1993; Liboff, 1997; Markov, 2005). Neither chemical nor ionization models can adequately explains these observations.

In an attempt to answer the question, *Can non-ionizing radiation cause cancer,* let's begin with what appears to be a scientific anomaly.

2.1. Free-radicals, oxidative stress and DNA damage

Studies show that exposure to RFR increases free radicals in the body—leading to oxidative stress—which can account for many of the biological responses and adverse health effects, including cancer, that are documented in the scientific literature.

Since NIR doesn't have enough energy to dislodge electrons and thus create free radicals how can it contribute to an increase in free radicals?

Free radicals can “build up” in the body in one of two ways. One way is to increase free radical formation, which is what happens with ionizing radiation and certain chemicals. The other way is to interfere with the production of anti-oxidants that neutralizes free radicals. The body produces free radicals during metabolic activity and it also produces anti-oxidants as part of its natural repair mechanism. If the anti-oxidant repair mechanism is impaired free radical damage can result. The Fenton reaction that depends on free iron may play a key role in this process (Phillips et al., 2009).

Yakymenko et al. (2015) reviewed the scientific literature in a paper entitled, *Oxidative mechanisms of biological activity of low-intensity radiofrequency radiation*. In this review they provide evidence for the following:

1. RF activation of key pathways generating reactive oxygen species (ROS),
2. Activation of peroxidation,
3. Oxidative damage of DNA, and
4. Changes in the activity of antioxidant enzymes.

Ninety-three of the 100 available peer-reviewed studies, dealing with oxidative effects with low-intensity RF exposure, confirmed that RF induces oxidative stress in biological systems. The research includes studies with humans, plants and animals. Yakymenko et al. conclude that low intensity RFR is an oxidative agent for living cells and is one of the primary mechanisms accounting for the biological activity of this kind of radiation. They also claim that EHS-like conditions can be attributed, at least partially, to ROS overproduction in cells due to RFR exposures (Yakymenko et al, 2015).

Lai (2014a) tabulated abstracts of articles related to RF and free radicals. He found that 93 of 106 papers, (i.e. 88% of the studies) documented significant effects. Clearly, many publications in this field report oxidative stress associated with low-intensity RF exposure.

ELF EMF (less than 300 Hz) has even less energy than RFR and yet these frequencies have also been associated with free radical production and oxidative stress. Lai (2014b) tabulated scientific abstracts dealing with the effects of ELF EMF on free radicals. Studies include both *in vivo* and *in vitro* experiments with either acute or chronic exposure of humans, animals, plants and microorganisms. Lai (2014b) found that 97 of 110 studies (i.e. 84% of the publications) reported effects. These effects include production of free radicals and reactive oxygen species (ROS); evidence of oxidative damage including DNA and neurological damage; apoptosis; altered antioxidant enzyme activity (both increase and decrease); and altered immune system response. Forty-five of the combined RFR and ELF EMF studies (Lai, 2014a, 2014b) reported changes within the brain. Supplementation with anti-oxidant (Zn, Se, Vitamin C, and melatonin) appeared to ameliorate the harmful effects of NIR exposure.

Critics of non-thermal mechanisms are likely to argue that the evidence cited by Yakymenko et al. (2015) and Lai (2014a,b) is biased; that the studies were flawed; that specific findings are not replicable; and that most studies did not control adequately for thermal effects. So I contacted the authors to enquire how they did their searches.

Lai (personal communication) obtained his references using PubMed that he monitored almost daily for the following search terms: radiofrequency, cell phone, mobile phone, ELF magnetic field, electric field; and occasionally for specific frequencies (800 MHz, 900 MHz, 2450 MHz, etc.). His research findings included all types of responses to NIR. The 2014a,b compilation of abstracts are limited to references dealing only with free radicals and either RF or ELF EMF exposure.

Yakymenko (personal communication) stated that they analyzed all peer-reviewed experimental publications that they could find concerned with possible oxidative effects of low

[3] Note: *Electrosmog* is applied to anthropogenic sources of ELF, RF, and MW radiation and can be considered a form of non-chemical air pollution.

intensity RFR/MW in living systems. They analyzed only the effects of *low-intensity* RFR, which—by definition—refers to intensities that do not induce significant thermal effects in biological tissues.

The publications cited by Yakymenko et al. (2015) and Lai (2014a, b) represent comprehensive and unbiased collections of peer-reviewed research at levels far below observable thermal effects in biological tissues and far below safety limits of the International Commissions on Non-Ionizing Radiation Protection (ICNIRP, 1998). When so many studies—using a variety of organisms—point in the same direction, they cannot be ignored.

A preponderance of scientific evidence clearly indicates that NIR, both ELF and RF, causes oxidative stress in living cells. This oxidative stress, while not the only mechanism, may be the key mechanism involved in carcinogenicity and it may also be involved with other effects including symptoms of electrohypersensitivity (EHS) and reproductive problems due to impaired sperm (Agarwal et al., 2009; Kesari and Behari, 2012). The damage is generated not by direct ionization of atoms and molecules but rather by interference with anti-oxidant repair mechanisms. A key player in this drama may very well be peroxynitrite (Pacher et al., 2007).

Production of free radicals by NIR is not the only mechanism with scientific support. Production of heat shock proteins indicating physiological stress; altered calcium flux and intracellular calcium signaling; induced ornithine decarboxylase activity; increased permeability of the blood brain barrier; reduced oncostatic effect of melatonin; single and double strand DNA breaks; as well as other mechanisms have been documented (Byrus et al., 1987; Liburdy et al., 1993; Mullins et al., 1999; Schirmacher et al., 2000; Blackman et al., 2001; Leszczynski, 2002; Lai and Singh, 2005; Blank and Goodman, 2009; Pall, 2015; Salford et al., 1994, 2003).

Even though both IR and NIR are part of the electromagnetic spectrum, application of the ionization model to non-ionizing energy is inappropriate. Both produce biological effects but through different pathways. IR breaks chemical bonds and thus directly damages DNA. NIR alters the anti-oxidant repair mechanism, resulting in a build up of ROS, which—in turn, disrupts cellular processes and damages DNA.

If the only studies documenting oxidative stress and its biological consequences were restricted to laboratory experiments, we may ask if this could happen under realistic exposure conditions outside of the lab. For this we need to turn our attention to epidemiological studies.

2.2. Epidemiological studies

Since the 1960s we have evidence of increased cancers associated with RF and MW exposure (Glaser et al., 1971). The more recent evidence includes:

1. Residential RF/MW exposure and cancers near cell phone base stations, broadcast antennas, and radar installations (Dolk et al., 1997; Goldsmith, 1997; Michelozzi et al., 2002; Park et al., 2004; Wolf and Wolf, 2004; Ha et al., 2007; Khurana et al., 2010; Dode et al., 2011; Inskip et al., 2010; Levitt and Lai, 2010; Yakymenko et al., 2011).
2. Occupational RF/MW exposure and cancers, such as testicular cancer, breast cancer, brain tumors, and leukemia (Davis and Mostofi, 1993; Szmigielski, 1996; Finklestein, 1998; Milham, 2004);
3. Long-term (10 plus years) mobile phone use and ipsilateral tumors including parotid gland tumors; acoustic neuromas, gliomas and possibly meningiomas (Hardell et al., 2005, 2008; Sadetzki et al., 2008; Cardis et al., 2011; INTERPHONE study Group, 2010, 2011; Coureau et al., 2014; Morgan et al., 2015); as well as breast cancer among women who keep their cell phone in their bra (West et al., 2013).
4. Rapid increase in thyroid cancers in various countries may also be implicated with cell phone use (Ahn et al., 2004), although there is some evidence that it may be due to improved detection of small tumors (Davies and Welch, 2004). Time will tell which is the case.

We also have evidence since the 1970s of cancers associated with low frequency EMFs.

1. Residential power frequency magnetic field exposure and childhood leukemia (Wartenberg, 2001; Alhbolm et al., 2001) that increased *de novo* with electrification (Milham and Ossiander, 2001); that may have an epigenetic component that alters DNA repair genes (Yang et al., 2008); and may be increased with metallic plumbing and ground current (Wertheimer et al., 1995);
2. Intermediate frequencies (kHz range) and various types of cancers (Milham and Morgan, 2008);
3. Occupational exposure to ELF EMFs and breast cancer among both men and women; leukemia; and brain tumors (Demers et al., 1991; Tynes et al., 1992; Floderus et al., 1993; Armstrong et al., 1994; Lee et al., 1996; Miller et al., 1996; Havas, 2000).

The International Agency for Research on Cancer (IARC), a branch of the World Health Organization (WHO), classified both ELF magnetic fields in 2002 and RFR in 2011 as possible human carcinogens (Class 2b) (IARC, 2002, 2012). One key reason for the IARC classification as “possible” (Class 2b) rather than “probable” (Class 2a) is expressed by the Swedish Radiation Protection Authority (2002) namely, *there is no biologically plausible mechanism to support a carcinogenic effect of non-ionizing RF waves.*

We now have a plausible mechanism to support a carcinogenic effect of NIR. This mechanism involves production of free radicals and is supported by many peer-reviewed scientific publications. Perhaps it is time for IARC to review the classification of both ELF EMF and RFR in light of this mechanism and it is time for guidelines recommended by ICNRIP to be reexamined in light of this evidence.

3. Conclusions

The key points of this commentary are as follows: (1) Application of the IR model to NIR is inappropriate as the mechanisms of biological interactions are different; (2) Sufficient scientific evidence exists of cellular damage caused by NIR at levels well below thermal guidelines; and (3) Various mechanisms have been documented that involve oxidative stress and can account for the increase in tumors documented in epidemiological studies at both low frequency and radio frequency electromagnetic exposure. Indeed, this type of oxidative stress may account for damage to sperm exposed to RFR and to some of the symptoms classified as electrohypersensitivity (EHS).

While science, to some degree, is based on consensus building, the majority has been known to be wrong in the past. It is my contention that the agencies and governing bodies mentioned in the Introduction are wrong about their assertions that NIR is “safe” and is not carcinogenic below existing thermal guidelines. Not only is NIR harmful, but we also know that it increases free radicals and causes oxidative damage in cells, which is likely to be one of the key mechanism involved in cancer and some of the other symptoms documented in the scientific literature.

The time is long overdue for the scientific community to move beyond this decades-old debate about whether NIR can cause

cancer at non-thermal levels. While there is much that remains unknown, currently the debate is based more on economic and political issues than on scientific evidence.

The rapid deployment of wireless technology needs to be reconsidered. The potentially harmful effects of Wi-Fi in schools; smart meters on homes; the 5G network, as well as previous generations of cellular telecommunication have not been adequately tested for biological compatibility. Evidence so far, indicates that we are already paying the price for this technology in terms of human and environmental health and the cost is likely to increase with increasing exposure. The longer we delay acting to reduce electrosmog exposure the more it will cost in terms of public health, absenteeism from work and school, quality of life, and increased morbidity (Carpenter, 2013).

Steps that need to be taken by individuals as well as by governing bodies and industry are provided in the EMF Scientist Appeal (Blank et al., 2015). We know enough to act and we need to take steps to reduce public exposure if we are to minimize an emerging health crisis.

Funding

This research did not receive any specific grant from funding agencies in the public, commercial, or not-for-profit sectors.

Declaration of interest

The author reports no conflicts of interest and is solely responsible for the content and writing of this article.

Acknowledgements

I would like to thank those scientists who conduct research despite inadequate funding and those who have retired yet continue to contribute to research in this field and the two reviewers who reviewed this paper.

References

Adey, W.R., 1993. Biological effects of electromagnetic fields. J. Cell. Biochem. 51, 410–416.

Agarwal, A., Desai, N.R., Makker, K., Varghese, A., Mouradi, R., Sabanegh, E., Sharma, R., 2009. Effects of radiofrequency electromagnetic waves (RF-EMW) from cellular phones on human ejaculated semen: an in vitro pilot study. Fertil. Steril. 92 (4), 1318–1325.

Ahlbom, A., Cardis, E., Green, A., Linet, M., Savitz, D., Swerdlow, A., 2001. Review of the epidemiologic literature on EMF and health. Environ. Health Perspect. 109 (Suppl. 6), 911–933.

Ahn, H.S., Kim, H.J., Welch, H.G., 2014. Korea's Thyroid-Cancer "epidemic" — screening and over diagnosis. N. Engl. J. Med. 371, 1765–1767.

Armstrong, B., Theriault, G., Guenel, P., Deadman, J., Goldberg, M., Heroux, P., 1994. Association between exposure to pulsed electromagnetic fields and cancer in electric utility workers in Quebec, Canada and France. Am. J. Epidemiol. 140 (9), 805–820.

Blackman, C.F., Kinney, L.S., House, D.E., Joines, W.T., 1989. Multiple power-density windows and their possible origin. Bioelectromagnetics 10, 115–128.

Blackman, C.F., Benane, S.G., House, D.E., 2001. The influence of 1.2 microT, 60 Hz magnetic fields on melatonin-and tamoxifen-induced inhibition of MCF-7 cell growth. Bioelectromagnetics 22, 122–128.

Blank, M., Goodman, R., 2009. Electromagnetic fields stress living cells. Pathophysiology 16, 71–78.

Blank, M., Havas, M., Kelley, E., Lai, H., Moskowitz, J., 2015. International appeal: scientists call for protection from non-ionizing electromagnetic field exposure. Eur. J. Oncol. 20 (3/4), 180–182. www.emfscientist.org.

Byus, C.V., Pieper, S., Adey, W.R., 1987. The effect of low-energy 60 Hz environmental electromagnetic fields upon the growth related enzyme ornithine decarboxylase. Carcinogenesis 8, 1385–1389.

Cardis, E., Armstrong, B.K., Bowman, J.D., Giles, G.G., Hours, M., Krewski, D., McBride, M., Parent, M.E., Sadetzki, S., Woodward, A., Brown, J., Chetrit, A., Figuerola, J., Hoffmann, C., Jarus-Hakak, A., Montestruq, L., Nadon, L., Richardson, L., Villegas, R., Vrijheid, M., 2011. Risk of brain tumours in relation to estimated RF dose from mobile phones: results from five Interphone countries. Occup. Environ. Med. 68, 631–640.

Carpenter, D.O., 2013. Human disease resulting from exposure to electromagnetic fields. Rev. Environ. Health 28 (4), 159–172, 2013.

Carpenter, D.O., Sage, C. (Eds.), 2007. BioInitiative Report: A Rationale for a Biologically-based Public Exposure Standard for Electromagnetic Fields (ELF and RF). www.bioInitiative.org.

Carpenter, D.O., Sage, C. (Eds.), 2012. BioInitiative Report: A Rationale for Biologically–based Public Exposure Standards for Electromagnetic Fields (ELF and RF). www.bioinitiative.org.

Coureau, G., Bouvier, G., Lebailly, P., Fabbro-Peray, P., Gruber, A., Leffondre, K., Guillamo, J.S., Loiseau, H., Mathoulin-Pélissier, S., Salamon, R., Baldi, I., 2014. Mobile phone use and brain tumours in the CERENAT case-control study. Occup. Environ. Med. 71 (7), 514–522.

Davies, L., Welch, H.G., 2006. Increasing incidence of thyroid Cancer in the United States, 1973-2002. JAMA 295 (18), 2164–2167.

Davis, R.L., Mostofl, F.K., 1993. Cluster of testicular cancer in police officers exposed to hand-held radar. Am. J. Ind. Med. 24, 231–233.

Demers, P.A., Thomas, D.B., Rosenblatt, K.A., et al., 1991. Occupational exposure to electromagnetic fields and breast cancer in men. Am. J. Epidemiol. 134, 340–347.

Dode, A.C., MMD Leão, c, FdeAF, Tejo, Gomes, A.C.R., Dode, D.C., Dode, M.C., Moreira, C.W., Condessa, V.A., Albinatti, C., Caiaffa, W.T., 2011. Mortality by neoplasia and cellular telephone base stations in the Belo Horizonte municipality, Minas Gerais state, Brazil. Sci. Total Environ. 409, 3649–3665.

Dolk, H., Shaddick, G., Walls, P., Grundy, C., Thakrar, B., Kleinschmidt, I., Elliott, P., 1997. Cancer incidence near radio and television transmitters in Great Britain, I Sutton Coldfield transmitter. Am. J. Epidemiol. 145 (1), 1–9.

FCC, 2010. Federal Communications Commission, How Safe Are Mobile and Portable Phones? http://www.fcc.gov/oet/rfsafety/rf-faqs.html.

Finkelstein, M.M., 1998. Cancer incidence among Ontario police officers. Am. J. Ind. Med. 34, 157–162.

Floderus, B., Persson, T., Stenlund, C., Wennberg, A., Ost, A., Knave, B., 1993. Occupational exposure to electromagnetic fields in relation to leukemia and brain tumors: a case-control study in Sweden. Cancer Causes Control 4 (5), 465–476.

Glaser, Z.R., 1971. Bibliography of Reported Biological Phenomena ('effects') and Clinical Manifestations Attributed to Microwave and Radiofrequency Radiation. Naval Medical Research Institute Research Report Project MF12.524.015–0004B, Report No. 24 October 1971.

Goldsmith, J.R., 1997. Epidemiologic evidence relevant to radar (microwave). Eff. Environ. Health Perspect. 105 (Suppl. 6), 1579–1587.

Ha, M., Im, H., Lee, M., Kim, H.J., Kim, B.C., et al., 2007. Radio-frequency radiation exposure from AM radio transmitters and childhood leukemia and brain cancer. Am. J. Epidemiol. 166, 270–279.

Hardell, L., Carlberg, M., Hansson Mild, K., 2005. Case-control study on cellular and cordless telephones and the risk for acoustic neuroma or meningioma in patients diagnosed 2000–2003. Neuroepidemiology 25, 120–128.

Hardell, L., Carlberg, M., Söderqvist, F., Hansson Mild, K., 2008. Meta-analysis of long-term mobile phone use and the association with brain tumours. Int. J. Oncol. 32 (5), 1097–1103.

Havas, M., 2000. Biological effects of non-ionizing electromagnetic energy: a critical review of the reports by the US National Research Council and the US National Institute of Environmental Health Sciences as they relate to the broad realm of EMF bioeffects. Environ. Rev. 8, 173–253.

Havas, M., 2013. Radiation from wireless technology affects the blood, the heart and the autonomic nervous system. Rev. Environ. Health 28 (2–3), 75–84.

Health Canada. 2010. Health Canada, Royal Society of Canada (RSC): Expert Panel Report on Radiofrequency Fields. Recent Advances in Research on Radiofrequency Fields and Health: 2001-2003. http://rsc-src.ca/sites/default/files/pdf/expert_panel_radiofrequency_update2.pdf Safety Code: http://www.hc-sc.gc.ca/ewh-semt/pubs/radiation/radio_guide-lignes_direct-eng.php Also: http://www.hc-sc.gc.ca/ewh-semt/radiation/cons/wifi/faq-eng.php.

Health Protection Agency, U.K, 2012. Previously Called the National Radiological Protection Board (NRPB) "Review of the Scientific Evidence for Limiting Exposure to Electromagnetic Fields (0–300 GHz)". http://www.hpa.org.uk/webc/HPAwebFile/HPAweb_C/1194947383619.

IARC (International Agency for Research on Cancer), 2002. IARC monographs on the evaluation of carcinogenic risks to humans. In: Non-ionizing Radiation. Part 1: Static and Extremely Low-frequency (ELF) Electric and Magnetic Fields, vol. 80. IARC, Press, Lyon.

IARC Monographs on the Evaluation of Carcinogenic Risks to Humans. Non-ioniz. radiation, Part 2 radiofrequency electromagnetic fields. Volume 102. Available at: http://monographs.iarc.fr/ENG/Monographs/vol102/mono102.pdf.

ICNIRP, 2016. International Commission for Non-ionizing Radiation Protection, a Group that Advises Governing Bodies Around the World Including the WHO, Has the Following Statement on Their Website for High Frequency (100 KHz–300 GHz) Electromagnetic Radiation. Accessed July 2016, date last reviewed is not available]. http://www.icnirp.org/en/frequencies/high-frequency/index.html.

ICNIRP, 1998. Guidelines for limiting exposure to time-varying electric, magnetic, and electromagnetic fields (up to 300 GHz). Health Phy. 74, 494–522, 1998.

Inskip, P.D., Hoover, R.N., Devesa, S.S., 2010. Brain cancer incidence trends in relation to cellular telephone use in the United States. Neuro-Oncol. 12 (11), 1147–1151.

INTERPHONE Study Group, 2010. Brain tumour risk in relation to mobile telephone use: results of the INTERPHONE international case-control study. Int. J.

Epidemiol. 39, 675–694.
INTERPHONE Study Group, 2011. Acoustic neuroma risk in relation to mobile telephone use: results of the INTERPHONE international case-control study. Cancer Epidemiol. 35, 453–464, 2011.
Kesari, K.K., Behari, J., 2012. Evidence for mobile phone radiation exposure effects on reproductive pattern of male rats: role of ROS. Electromagn. Biol. Med. 31 (3), 213–222.
Khurana, V.G., Hardell, L., Everaert, J., Bortkiewicz, A., Carlberg, M., et al., 2010. Epidemiological evidence for a health risk from mobile phone base stations. Int. J. Occup. Environ. Health 16, 263–267.
Lai H, 2014a, RFR Free radical Abstracts, List of 106 abstracts, Updated March 2014; abstracts cover the research published between 1990-2014 and are downloadable at www.bioInitiative.org/research-summaries
Lai H, 2014b ELF EMF Free Radical Abstracts, List of 123 abstracts, Updated March 2014; abstracts cover the research published between 1990-2014 and are downloadable at www.bioInitiative.org/research-summaries/.
Lai, H., Singh, N.P., 2005. Interaction of microwaves and a temporally incoherent magnetic field on single and double DNA strand breaks in rat brain cells. Electromagn. Biol. Med. 24, 23–29.
Lee, J.M., Pierce, K.S., Spiering, C.A., Steams, R.D., VanGinhoven, G., 1996. Electrical and Biological Effects of Transmission Lines: a Review. Bonneville Power Administration Portland, Oregon.
Leszczynski, D., Joenvaara, S., Reivinen, J., Kuokka, R., 2002. Non-thermal activation of the hsp27/p38MAPK stress pathway by mobile phone radiation in human endothelial cells: molecular mechanism for cancer-and blood-brain barrier-related effects. Differentiation 70, 120–129.
Levitt, B.B., Lai, H., 2010. Biological effects from exposure to electromagnetic radiation emitted by cell tower base stations and other antenna arrays. Environ. Rev. 18, 369–395.
Liboff, A.R., 1997. Electric-field ion cyclotron resonance. Bioelectromagnetics 18 (1), 85–87.
Liburdy, R.P., Sloma, T.R., Sokolic, R., Yaswen, P., 1993. ELF magnetic fields, breast cancer, and melatonin: 60 Hz fields block melatonin's oncostatic action on ER+ breast cancer cell proliferation. J. Pineal Res. 14, 89–97.
Litovitz, T.A., Montrose, C.J., Goodman, R., Elson, E.C., 1990. Amplitude windows and transiently augmented transcription from exposure to electromagnetic fields. Bioelectromagnetics 11 (4), 297–312.
Markov, M.S., 2005. "Biological windows": a tribute to W. Ross Adey. Environ. 25, 67–74.
Michelozzi, P., Capon, A., Kirchmayer, U., Forastiere, F., Biggeri, A., et al., 2002. Adult and childhood leukemia near a high-power radio station in Rome, Italy. Am. J. Epidemiol. 155, 1096–1103.
Milham, S., 2004. Brief report a cluster of male breast Cancer in office workers. Am. J. Ind. Med. 46, 86–87.
Milham, S., Morgan, L.L., 2008. A new electromagnetic exposure metric: high frequency voltage transients associated with increased cancer incidence in teachers in a California school. Am. J. Ind. Med. 51 (8), 579–586.
Milham, S., Ossiander, E.M., 2001. Historical evidence that residential electrification caused the emergence of the childhood leukemia peak. Med. Hypotheses 56 (3), 290–295.
Miller, A.B., To, T., Agnew, D.A., Wall, C., Lois, M., 1996. Leukemia following occupational exposure to 60-Hz electric and magnetic fields among Ontario electric utility workers. Am. J. Epidemiol. 144, 150–160.
Morgan, L.L., Miller, A.B., Sasco, A., Davis, D.L., 2015. Mobile phone radiation causes brain tumors and should be classified as a probable human carcinogen (2A) (Review). Int. J. Oncol. 44 (5), 1865–1878.
Mullins, J.M., Penafiel, L.M., Juutilainen, J., Litovitz, T.A., 1999. Dose response of electromagnetic field-induced ornithine decarboxylase activity. Bioelectrochem. Bioenerg. 48, 193–199.
National Cancer Institute, U.S, 2016. Cell Phones and Cancer Risk Fact Sheet updated May 27, 2016. www.cancer.gov/about-cancer/causes-prevention/risk/radiation/cell-phones-fact-sheet.
New Zealand Ministry for the Environment, 2008. National Environmental Standards for Telecommunication Facilities. http://www.mfe.govt.nz/laws/standards/telecommunication-standards.html. http://www.mfe.govt.nz/publications/rma/radio-freq-guidelines-dec00.html.
Pacher, P., Beckman, J.S., Liaudet, L., 2007. Nitric oxide and peroxynitrite in health and disease. Physiol. Rev. 87 (1), 315–424.
Pall, M.L., 2015. Scientific evidence contradicts findings and assumptions of Canadian safety panel 6: microwaves act through voltage-gated calcium channel activation to induce biological impacts at non-thermal levels, supporting a paradigm shift for microwave/lower frequency electromagnetic field action. Rev. Environ. Health 30 (2), 99–116.
Park, S.K., Ha, M., Im, H.J., 2004. Ecological study on residences in the vicinity of AM radio broadcasting towers and cancer death: preliminary observations in Korea. Int. Arch. Occup. Environ. Health 77, 387–394.
Phillips, J.L., Singh, N.P., Lai, H., 2009. Electromagnetic fields and DNA damage. Pathophysiology 16 (2–3), 79–88.
Sadetzki, S., Chetrit, A., Jarus-Hakak, A., Cardis, E., Deutch, Y., et al., 2008. Cellular phone use and risk of benign and malignant parotid gland tumors-a nationwide case-control study. Am. J. Epidemiol. 167, 457–467.
Salford, L.G., Brun, A., Sturesson, K., Eberhardt, J.L., Persson, B.R.R., 1994. Permeability of the blood-brain barrier induced by 915 MHz electromagnetic radiation, continuous wave and modulated at 8, 16, 50 and 200 Hz. Microsc. Res. Tech. 27 (6), 535–542.
Salford, L.G., Brun, A.E., Eberhardt, J.L., Malmgren, L., Persson, B.R.R., 2003. Nerve cell damage in mammalian brain after exposure to microwaves from GSM mobile phones. Environ. Health Perspect. 111 (7), 881–883.
Schirmacher, A., Winters, S., Fischer, S., Goeke, J., Galla, H.-J., Kullnick, U., Ringelstein, E.B., StÎgbauer, F., 2000. Electromagnetic fields (1.8 GHz) increase the permeability to sucrose of the blood-brain barrier in vitro. Bioelectromagnetics 21, 338–345.
Swedish Radiation Protection Authority, 2002. Epidemiological Studies of Cellular Telephones and cancer Risk – a Review. Swedish Radiation Protection Authority, Stockholm (2002). http://w3.iec.csic.es/textos/ssi_rapp_2002_16.pdf.
Szmigielski, S., 1996. Cancer morbidity in subjects occupationally exposed to high frequency (radiofrequency and microwave) electromagnetic radiation. Sci. Total Env. 180, 9–17.
Tynes, T., Anderson, A., Langmark, F., 1992. Incidence of cancer in Norwegian workers potentially exposed to electromagnetic fields. Am. J. Epidemiol. 136 (1), 81–88.
Wartenberg, D., 2001. Residential EMF exposure and childhood leukemia: meta-analysis and population attributable risk. Bioelectromagnetics (Suppl. 5), S86–S104.
Wei, L.X., Goodman, R., Henderson, A., 1990. Changes in levels of c-myc and histone H2B following exposure of cells to low-frequency sinusoidal electromagnetic fields: evidence for a window effect. Bioelectromagnetics 11 (4), 269–272.
Wertheimer, N., Savitz, D.A., Leeper, E., 1995. Childhood cancer in relation to indicators of magnetic fields from ground current sources. Bioelectromagnetics 16, 86–96.
West, J.G., Kapoor, N.S., Liao, S.-Y., Chen, J.W., Bailey, L., Nagourney, A.R., 2013. Multifocal breast cancer in young women with prolonged contact between their breasts and their cellular phones. Case Rep. Med. 2013, 5.
WHO, 2014. Media Centre, fact sheet on "Electromagnetic fields and public health: mobile phones" (Fact Sheet No. 193, reviewed October 2014), http://www.who.int/mediacentre/factsheets/fs193/en/ They make the same statement about cell phone antennas, DECT phones, and Wi-Fi.
Wolf, R., Wolf, D., 2004. Increased incidence of cancer near a cell-phone transmitter station. Int. J. Cancer Prev. 1, 123–128.
Yakymenko, I., Sidorik, E., Kyrylenko, S., Chekhun, V., 2011. Long-term exposure to microwave radiation provokes cancer growth: evidence from radars and mobile communication Systems. Exp. Oncol. 33 (2), 62–67.
Yakymenko, I., Tsybulin, O., Sidorik, E., Henshel, D., Kyrylenko, O., Kyrylenko, S., 2015. Oxidative mechanisms of biological activity of low-intensity radiofrequency radiation. Electromagn. Biol. Med. 35 (2), 186–202.
Yang, Y., Jin, X., Yan, C., Tian, Y., Tang, J., et al., 2008. Case-only study of interactions between DNA repair genes (hMLH1, APEX1, MGMT, XRCC1 and XPD) and low-frequency electromagnetic fields in childhood acute leukemia. Leuk. Lymph. 49, 2344–2350.

Magda Havas, B.Sc., Ph.D.
Trent School of the Environment, Trent University, 1600 West Bank Drive, Peterborough, ON, K9J 0G2, Canada
E-mail address: mhavas@trentu.ca.

23 August 2016
Available online 28 November 2016
