# Supplementary material for: Magnetic Fields and Cancer: Epidemiology, Cellular Biology, and Theranostics
Source: Int J Mol Sci. 2022 Jan 25;23(3):1339. doi: 10.3390/ijms23031339 (PMC8835851; doi:10.3390/ijms23031339)
Supplement: Supplementary file 1 [file ijms-23-01339-s001.zip › Supplementary Data Set S1/MF and Cancer.Data/PDF/2176577523/Morsing2019_Article_HybridPETMRIInMajorCancers.pdf]

European Journal of Nuclear Medicine and Molecular Imaging (2019) 46:2138–2151
https://doi.org/10.1007/s00259-019-04402-8

REVIEW ARTICLE

Hybrid PET/MRI in major cancers: a scoping review

Anni Morsing[1,2] · Malene Grubbe Hildebrandt[1,3,4] · Mie Holm Vilstrup[1] · Sara Elisabeth Wallenius[1] · Oke Gerke[1] · Henrik Petersen[1] · Allan Johansen[1] · Thomas Lund Andersen[1,2] · Poul Flemming Høilund-Carlsen[1,3]

Received: 27 March 2019 / Accepted: 13 June 2019 / Published online: 2 July 2019
© Springer-Verlag GmbH Germany, part of Springer Nature 2019

Abstract

Purpose PET/MRI was introduced for clinical use in 2011 and is now an established modality for the imaging of brain and certain pelvic cancers, whereas clinical use for the imaging of other forms of cancer is not yet widespread. We therefore systematically investigated what has been published on the use of PET/MRI compared to PET/CT in the imaging of cancers outside the brain, focusing on clinical areas of application related to diagnosis, staging and restaging.

Methods A systematic search of PubMed/MEDLINE, Embase and the Cochrane Library was performed. Studies evaluating the diagnostic performance of simultaneous PET/MRI in cancer patients were chosen.

Results A total of 3,138 publications were identified and 116 published during the period 2012–2018 were included and were grouped according to the major cancer forms: 13 head and neck (HNC), 9 breast (BC), 21 prostate (PC), 14 gynaecological, 13 gastrointestinal (GIC), and 46 various cancers. Data from studies comparing PET/MRI and PET/CT for staging/restaging suggested the superiority of ^{18}F-FDG PET/MRI for the detection of tumour extension and retropharyngeal lymph node metastases in nasopharyngeal cancer, and for the detection of liver metastases and possibly bone marrow metastases in high-risk BC. FDG PET/MRI tended to be inferior for the detection of lung metastases in HNC and BC. ^{68}Ga-PSMA-11 PET/MRI was superior to PET/CT for the detection of local PC recurrence. FDG PET/MRI was superior to FDG PET/CT for the detection of local tumour invasion in cervical cancer and had higher accuracy for the detection of liver metastases in colorectal cancer.

Conclusion The scoping review methodology resulted in the identification of a huge number of records, of which less than 5% were suitable for inclusion and only a limited number allowed conclusions on the advantages/disadvantages of PET/MRI compared to PET/CT in the oncological setting. There was evidence to support the use of FDG PET/MRI in staging of nasopharyngeal cancer and high-risk BC. Preliminary data indicate the superiority of PET/MRI for the detection of local recurrence in PC, local tumour invasion in cervical cancer, and liver metastases in colorectal cancer. These conclusions are based on small datasets and need to be further explored.

Keywords PET/MRI · PET/CT · Oncology · Staging · ^{18}F-FDG

Introduction

Since the introduction of clinical FDG PET/CT about two decades ago, its use in patient treatment has increased dramatically and is now well established. Being a whole-body molecular imaging modality, FDG PET/CT plays an important role in most cancers and is used for staging, treatment planning, recurrence detection, and lately also for response evaluation in certain cancers. In parallel, MRI has evolved into a clinical modality used for T and N staging of cancers, in which high spatial resolution and soft-tissue contrast and/or radiation exposure are issues.

Integrated PET/MRI was introduced for clinical use in 2011, and is now an established modality in the imaging of

This article is part of the Topical Collection on Oncology - General

Electronic supplementary material The online version of this article (https://doi.org/10.1007/s00259-019-04402-8) contains supplementary material, which is available to authorized users.

✉ Anni Morsing
Anni.morsing@dadlnet.dk

1 Department of Nuclear Medicine, Odense University Hospital, Odense, Denmark

2 MAgNetic Resonance Technology for Response Adapted Radiotherapy (MANTRA), Odense University Hospital, Odense, Denmark

3 Research Unit of Clinical Physiology and Nuclear Medicine, Department of Clinical Research, University of Southern Denmark, Odense, Denmark

4 Centre for Innovative Medical Technology (CIMT), Odense University Hospital, Odense, Denmark

brain and certain pelvic cancers, whereas clinical use for the imaging of other forms of cancer is not yet widespread. Whole-body PET/MRI undoubtedly holds potential for improved diagnostic evaluation by merging PET-based molecular imaging with the high spatial resolution, high tissue contrast and information from functional parameters more easily obtained by MRI. The question is, however, in which diseases and for what purposes is this hybrid technique preferable to PET/CT. In an attempt to elucidate this, we used the scoping review technique to investigate systematically what has been published on the use of PET/MRI in the imaging of cancers outside the brain. Disregarding cancer entities for which the number of publications is still too small to provide meaningful information, and studies with inhomogeneous and mixed cancer patient populations, we identified five cancer groups with a sufficient number of publications to allow conclusions with regard to the advantages and disadvantages of using PET/MRI: head and neck cancer (HNC), breast cancer (BC), prostate cancer (PC), gynaecological cancer (GC) and gastrointestinal cancer (GIC). Within this scope we focused on three clinical areas of application: (1) initial diagnosis and staging, (2) disease monitoring and response evaluation, and (3) recurrence detection and restaging. For each of the five major cancers, we briefly mention all the studies fulfilling the inclusion criteria, and discuss in depth only those studies assessing the diagnostic performance of PET/MRI compared to that of PET/CT, thereby highlighting areas in which the replacement of PET/CT by PET/MRI could have a clinical impact.

Materials and methods

Search strategy

This systematic scoping review was conducted in accordance with established scoping review methodology [1, 2]. Studies were identified by searching MEDLINE (via PubMed), Embase and the Cochrane Library databases (Supplementary data S1). The search included articles published before 1 September 2018. The search strategy was developed by two senior consultant reviewers (A.M., M.G.H.) and a senior health sciences research librarian from the University Library of Southern Denmark. Both index terms (e.g. MESH terms) and text words were included in the searches. To ensure high sensitivity in relation to identification of relevant studies, the final search strings were broad and thus expected a priori to have relatively low specificity. The PICOS (population, intervention, comparison, outcome, and study types) approach [3] was used and comprised:

- Patients with cancer (excluding central nervous system (CNS)) evaluated for staging (initial or restaging), surgical resectability, radiation therapy planning, response, or suspected recurrence
- Studies in which the primary aim was evaluation of hybrid PET/MRI
- No restrictions on comparator modalities
- Diagnostic performance, lesion detection, quantitative evaluation, or feasibility
- Original, peer-reviewed studies without time restriction to year of publication

Exclusion criteria were nonhuman studies, publications not written in English or German, case reports, editorials, commentaries, reviews, meta-analyses, guidelines, book chapters, technology assessment reports, conference proceedings, studies comprising post-hoc fusion of separately performed PET and MRI, non-hybrid PET/MRI systems or trimodality PET/CT/MRI systems, studies including ten or fewer patients, and studies on dedicated PET/MRI breast imaging and CNS cancers.

Study selection

The search records from the three databases were transferred to the Endnote reference tool, where duplicates and book sections were identified and removed. Records were then screened by title and abstract by one senior researcher (A.M.). The included studies were transferred to the Covidence reference tool and screened independently by title and abstract according to strict inclusion and exclusion criteria by two senior researchers (A.M., M.G.H.). Disagreements were resolved by consensus. One senior researcher (A.M.) went through the full text of the included papers in a second step checking for their eligibility for final inclusion.

Data extraction

Data were extracted from each of the selected articles to a data extraction form. Studies were grouped according to cancer type, and the following characteristics were extracted: study design and objective, population and size, year, and follow-up data. Cancer entities for which the number of publications was too small to provide meaningful information and studies including inhomogeneous and mixed patient populations with various cancer types were disregarded. Five cancer groups with a sufficient number of publications to allow conclusions with regard to the advantages and disadvantages of using PET/MRI in relation to PET/CT were identified: HNC, BC, PC, GC and GIC.

The reference list was split into two: a shorter one, which follows after the body of the text and contains only references necessary for the scoping review method plus references comparing PET/CT and PET/MRI, and the full list, arranged

according to cancer type, provided in the Supplementary material (Supplementary data S2).

Results

Search results

Figure 1 shows the literature retrieval workflow according to the PRISMA statement [3]. The database search revealed 3,138 papers of which 2,384 remained after checking for duplicates. After screening of abstracts and full texts for eligibility, 549 studies remained for full-text assessment according to the inclusion and exclusion criteria. Finally, 116 studies published during the period 2012–2018 were included and were grouped according to cancer type (Supplementary data S2): 13 were on HNC; 9 were on BC; 21 were on PC; 14 were on GC; 13 were on GIC; 19 included populations with various cancers (14 on lesion detection/staging and 5 on quantitative FDG uptake); 10 were on haematological cancers (1 multiple myeloma and 9 Hodgkin and non-Hodgkin disease with various indications not meeting the inclusion criteria); 11 were on lung cancer/nodules; and 6 were on other forms of cancer with information outside the scope of this review including two small paediatric studies. Of the last six paediatric studies, one compared different PET/MRI reading protocols applied to the same study in 12 patients with various types of lymphoma (Supplementary data S2, 114), and the other included 18 patients with various cancers (Supplementary data S2, 115).

Head and neck cancer

Of the 13 studies on HNC, 6 concerned staging/restaging (Table 1), and seven were explorative studies investigating associations between functional MRI and quantitative PET (Supplementary data S2, 29–35). Three studies, two prospective and one retrospective, concerned staging/restaging in head and neck squamous cell carcinoma. Chan et al. compared FDG PET/MRI and FDG PET/CT for initial staging in a prospective study including 113 patients with nasopharyngeal cancer using histopathology or follow-up imaging as the reference standard [4]. Both modalities detected all primary

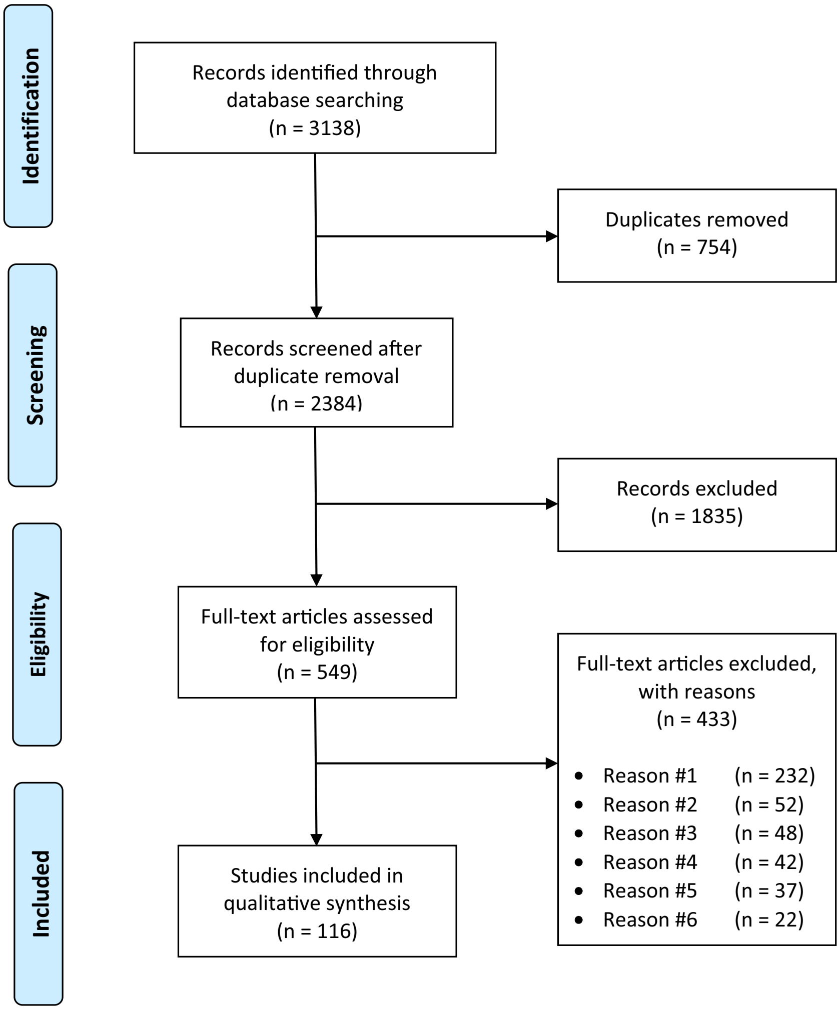

Fig. 1 PRISMA flow diagram modified to fit the present material. Exclusion reasons: *Reason #1* case reports, editorials, commentaries, reviews, meta-analyses, guidelines, book chapters, technology assessment reports, conference proceedings; *Reason #2* post-hoc fusion of separately performed PET and MRI, nonhybrid PET/MRI systems or trimodality PET/CT/MRI systems; *Reason #3* studies including ten or fewer patients; *Reason #4* studies in CNS cancer; *Reason #5* primary aim technical aspects, PET tracer development, radiomics/textural analysis, radionuclide therapy delivery; *Reason #6* other (not cancer, dedicated PET/MRI breast imaging, publications not written in English or German, duplicate)

Table 1 PET/MRI compared to PET/CT in head and neck cancer: study designs and outcomes

Reference	Number of patients	Population	Study design	PET tracer	Clinical area of application	Reference standard	Findings
[4]	113	Nasopharyngeal carcinoma	Prospective	^{18}F-FDG	Initial diagnosis and staging	Histopathology or follow-up imaging	No difference in detection of tumour and metastases (N and M); better detection of local tumour extension on PET/MRI
[5]	25	HNSCC	Retrospective	^{18}F-FDG	Initial diagnosis and staging (12), recurrence detection and restaging (13)	Histopathology or follow-up imaging	Correct T stage in 59% vs. 75% and N stage in 77% vs. 71% on PET/CT and PET/MRI, respectively (NS); no difference in diagnostic accuracy for recurrence detection (72% vs. 72%)
[6]	17	Suspected HNC	Prospective	^{18}F-FDG	Initial diagnosis and staging (7), recurrence detection and restaging (10)	Consensus diagnosis based on available information	Sensitivity 82.7% for PET/CT and 80.5% for PET/MRI for detection of 23 malignant tumours
[7]	31	Dedifferentiated DTC	Prospective	^{18}F-FDG	Recurrence detection and restaging	Histopathology or consensus diagnosis	No difference in detection rate at the patient level (both 25/26 patients) or of malignant lesions (PET/CT 113/116 vs. PET/MRI 99/113); lower sensitivity of PET/MRI for detecting lung metastases (77.9% vs. 100%, $p < 0.001$), 14 lesions identified on PET/CT
[8]	12	Iodine-negative DTC	Prospective	^{68}Ga-DOTATATE	Recurrence detection and restaging	Histopathology or follow-up imaging	No difference in lesion detection rate (79/85 vs. 69/85) in 11 patients; higher sensitivity of FDG PET/CT for detection of lung metastases in 8 patients (100% vs. 71%, $p < 0.0001$)
[9]	65	DTC	Retrospective	^{124}I	Initial diagnosis and staging (37), disease monitoring and response evaluation (7), recurrence detection and restaging (21)	None	PET/MRI detected significantly more lesions than PET/CT (remnants 72 vs. 60, metastases 100 vs. 80); no difference at the patient level (21/65 vs. 17/65 patients with malignant lesions)

DTC differentiated thyroid cancer, *HNC* head and neck cancer, *HNSCC* head and neck squamous cell carcinoma, *NS* not significant

tumours, but FDG PET/MRI detected local tumour extension (T4) more often than FDG PET/CT (pterygopalatine fossa 14 vs. 9 patients, intracranial extension 25 vs. 14 patients, and perineural infiltration 21 vs. 7 patients, respectively). The sensitivity for detection of regional lymph node and distant metastases did not differ significantly between FDG PET/MRI and FDG PET/CT (99.5% vs. 90.9%, and 90% vs. 83.3%) although FDG PET/MRI correctly identified 26 retropharyngeal lymph node metastases that were false-negative on FDG PET/CT. Three lung metastases of unknown size were reported and identified on both FDG PET/CT and FDG PET/MRI. The two smaller studies including more heterogeneous populations of patients with primary or recurrent HNC at various locations in the head and neck region showed no statistically significant differences in staging or restaging between FDG PET/MRI and FDG PET/CT [5, 6].

Three studies compared PET/MRI and PET/CT for staging of differentiated thyroid cancer. In 65 patients, Binse et al.

found that ^{124}I PET/MRI of the neck detected more iodine-positive thyroid remnants and metastases than ^{124}I PET/CT (72 vs. 60, $p = 0.002$, and 100 vs. 80, $p = 0.001$, respectively) because of higher sensitivity of the ^{124}I PET component due to the longer acquisition time and better scanner resolution of the former [9]. However, the number of patients identified with at least one tumour lesion did not differ significantly (^{124}I PET/MRI 21/65 vs. ^{124}I PET/CT 17/65). Vrachimis et al. compared FDG PET/MRI and FDG PET/CT in 31 patients with increased thyroglobulin levels and iodine-negative suspected recurrence [7]. They found that both modalities correctly identified 26 cervical tumours in 26 of 31 patients, but that FDG PET/MRI failed to detect 14 lung metastases identified on FDG PET/CT (sensitivity for detection of lung metastases of 77.9% vs. 100%, $p < 0.001$). Vrachimis et al. also compared ^{68}Ga-DOTA-Tyr3-octreotate (^{68}Ga-DOTATATE) PET/MRI and FDG PET/CT in 12 patients [8]. In this study, both PET/MRI and PET/CT correctly identified cervical tumour in 11 patients, but ^{68}Ga-DOTATATE PET/MRI missed a total of 12 lung metastases resulting in significantly lower sensitivity for detection of lung metastases (71% vs. 100%, $p < 0.0001$) [8].

Breast cancer

Five of the nine studies on BC concerned staging/restaging [10–14]. Two studies investigated dynamic MRI and quantitative PET as biomarkers for histological phenotype or response to neoadjuvant chemotherapy, and two studies compared quantitative FDG PET/CT and quantitative FDG PET/MRI (Supplementary data S2, 36–39). Four studies, two prospective and two retrospective, compared FDG PET/MRI and FDG PET/CT for whole-body staging/restaging, while one retrospective study evaluated bone metastases only (Table 2). One pilot study compared FDG PET/MRI and FDG PET alone from the same study [14].

Catalano et al. found discordant staging in 17 out of 51 patients, with 50 patients correctly staged by FDG PET/MRI compared to 38 by FDG PET/CT ($p < 0.01$), yielding a statistically significantly difference in staging accuracy of 98% versus 75% ($p = 0.005$). The reference standard consisted of pathology and follow-up imaging with FDG PET/MRI detecting non-avid FDG bone metastases and subcentimetre liver metastases, which were missed by FDG PET/CT in three patients [10]. The authors considered this result to be due to misclassification of bone marrow changes on PET/CT as benign, when MRI showed changes interpreted as malignant, but they failed to demonstrate convincingly the validity of this claim. In a retrospective study without histopathology as part of the reference standard, Catalano et al. found, that FDG PET/MRI detected more presumed bone metastases than FDG PET/CT (141 vs. 90 bone metastases, respectively, $p < 0.001$) in 25 out of 109 patients including three patients who were negative for bone metastases on FDG PET/CT [13]. Of 51 presumed metastases observed on PET/MRI and not on PET/CT, 44 were so-called permeative bone changes. Melsaether et al. compared FDG PET/MRI and FDG PET/CT for the detection of metastatic lesions in 51 heterogeneous patients referred for primary staging, restaging, response to chemotherapy or monitoring of metastatic disease, and found no differences in detection of metastatic lymph nodes (19 nodes in eight patients) or distant metastatic lesions (242 lesions in 30 patients) [12]. However, diffusion-weighted and contrast-enhanced FDG PET/MRI yielded higher sensitivity for the detection of liver metastases (40 patients, 100% and 80% by readers 1 and 2 vs. 75% and 70% by readers 3 and 4, $p < 0.001$) compared to unenhanced FDG PET/CT. In the 23 patients with lung metastases, the sensitivity of FDG PET/MRI tended to be lower (87% and 74% by readers 1 and 2) vs. 100% and 96% by readers 3 and 4, $p = 0.65$). Sawicki et al. compared FDG PET/MRI and FDG PET/CT for restaging, and both modalities identified the 17 of 21 patients with recurrence according to the reference standard and did not differ in detection of lesions, probably because 108 of the 116 malignant lesions were FDG-avid [11]. The remaining eight lesions were stated as non-FDG-avid bone metastases detected by PET/MRI only, but not further described. However, no lung metastases were reported.

Prostate cancer

Of the 21 studies on PC, five compared PET/MRI and PET/CT for primary staging and/or restaging in patients with biochemical recurrence, three investigated the correlation between PET/MRI findings and prostate-specific antigen (PSA) levels or clinical nomograms, two compared quantitative standardized uptake value (SUV) by PET and dynamic MRI parameters, ten investigated intraprostatic quantitative imaging features from combined PET/multiparametric MRI (mpMRI), and one was a feasibility study comparing quantitative PET values obtained by PET/MRI and PET/CT (Supplementary data S2, 40–55).

The five studies comparing PET/MRI and PET/CT for staging/restaging are presented in Table 3. One involved primary staging of metastatic PC, two involved restaging of patients with biochemical recurrence, and two included mixed populations of patients referred for staging or restaging. Two prospective studies used ^{11}C-choline and three retrospective studies used ^{68}Ga-PSMA-11 as the PET tracer.

With regard to primary staging, Freitag et al. found that in 26 patients with metastatic PC, ^{68}Ga-PSMA-11 PET/MR and ^{68}Ga-PSMA-11 PET/CT were concordant in detecting 98.5% of 64 lymph node metastases in 20 patients and 100% of 28 bone lesions in eight patients, all PSMA-positive, according to the reference standard of histopathology, response to therapy, follow-up imaging or decrease in PSA level [18]. In accordance with the findings of Freitag et al., Domachevsky et al.

Table 2 PET/MRI compared to PET/CT in breast cancer: study designs and outcomes

Reference	Number of patients	Population	Study design	PET tracer	Clinical area of application	Reference standard	Findings
[10]	51	IDC	Retrospective	^{18}F-FDG	Initial diagnosis and staging	Histopathology or follow-up imaging	PET/MRI correctly staged 50 patients and PET/CT 38 compared to the reference standard ($p < 0.01$)
[11]	21	IDC/ILC	Prospective	^{18}F-FDG	Recurrence detection and restaging	Histopathology or follow-up imaging	Both modalities identified the 17 patients with recurrence; PET/MRI detected all 116 malignant lesions compared to 108 on PET/CT
[12]	51	BC	Prospective	^{18}F-FDG	Initial diagnosis and staging (6), disease monitoring and response evaluation (39), recurrence detection and restaging (6)	Histopathology (4), clinical or imaging follow-up	No difference in detection of lymph node or distant metastases; PET/MRI had higher sensitivity for liver metastases (100%/80% vs. 75%/70%[a], $p < 0.001$)
[13]	109 (only 25 with bone metastases included)	BC	Retrospective	^{18}F-FDG	Detection of bone metastases	Prior imaging or follow-up imaging	PET/MRI detected 141 metastatic bone lesions compared to 90 on PET/CT in 25 patients ($p < 0.001$)

BC breast cancer, *IDC* invasive ductal carcinoma, *ILC* invasive lobular carcinoma

[a] Readers 1–4

found no difference in the number of PSMA-positive lesions detected by ^{68}Ga-PSMA-11 PET/MRI and ^{68}Ga-PSMA-11 PET/CT (61 vs. 63 lesions) in 21 patients referred for staging/restaging [15]. However, the identified lesions were not assessed by a reference standard in the latter study.

In restaging of PC with biochemical recurrence, Freitag et al. compared abdominal ^{68}Ga-PSMA-11 PET/mpMRI and ^{68}Ga-PSMA-11 PET/CT in 119 patients, and found a significantly higher detection rate with PET/MRI than PET/CT (18 and 9 patients, respectively, $p = 0.004$) which was ascribed to mpMRI [17]. They also found that detection of local recurrence by ^{68}Ga-PSMA-11 PET was significantly influenced by proximity to the bladder ($p = 0.028$) because of interfering accumulation of PSMA in the bladder. Eiber et al. compared ^{11}C-choline PET/MRI and ^{11}C-choline PET/CT in 75 patients with biochemical recurrence, and found that PET/CT identified more lymph node and bone metastases than PET/MRI (74/87 vs. 60/87, $p = 0.014$, and 57/64 vs. 52/64, $p < 0.001$) [16]. In this study two reader teams evaluated the scans independently, and a discrepancy was found with regard to the detection of local recurrence, as one of the reader teams found that ^{11}C-choline PET/MRI detected more lesions than ^{11}C-choline PET/CT (36/37 vs. 24/37, $p = 0.001$) and only in patients with a PSA level above 2 ng/mL. In a study without a reference standard, Souvatzoglou et al. not find any difference in the numbers of choline-positive lesions in a mixed population of patients with primary PC/biochemical recurrence (PSA 0.3–208 ng/mL) on ^{11}C-choline PET/MRI and ^{11}C-choline PET/CT (77/80 vs. 79/80) [19].

Gynaecological cancer

Of the 14 studies on GC, four compared FDG PET/MRI and FDG PET/CT for primary staging and/or restaging, four compared FDG PET/MRI and stand-alone MRI for staging/restaging, and six investigated quantitative PET and mpMRI in comparison with histopathology or follow-up imaging (Supplementary data S2, 56–65). No studies compared PET/MRI with PET/CT in combination with pelvic MRI. The four studies comparing FDG PET/MRI and FDG PET/CT for staging/restaging are presented in Table 4; two were prospective and two were retrospective.

In the primary staging of 18 patients with cervical (11 patients) or endometrial (7 patients) cancer, Schwartz et al. found that FDG PET/MRI of the abdomen/pelvis and FDG PET/CT did not differ in detection of 17 primary tumours, 26 regional lymph nodes (in 7 patients), and six hepatic and splenic nodules (in 1 patient) [20]. Two patients had thoracic or supraclavicular lymph node metastases on FDG PET/CT which were outside the FDG PET/MRI imaging field. However, in cervical cancer, FDG PET/MRI identified local tumour invasion into the parametrial area or the bladder that was not detected by FDG PET/CT, and led to upstaging in five patients.

Table 3 PET/MRI compared to PET/CT in prostate cancer: study designs and outcomes

Reference	Number of patients	Population	Study design	PET tracer	Clinical area of application	Reference standard	Findings
[15]	21	High risk PC or biochemical recurrence	Retrospective	^{68}Ga-PSMA-11	Initial diagnosis and staging (13), recurrence detection and restaging (8)	None	No difference in number of PSMA-positive lesions (61 PET/MRI vs. 63 PET/CT)
[16]	75	Biochemical recurrence	Prospective	^{11}C-Choline	Recurrence detection and restaging	Histopathology (19) or tumour board consensus from imaging and clinical data (66)	PET/MRI identified more LR (36/37 vs. 24/37, $p = 0.001$, for reader team 1); PET/CT identified more lymph node and bone metastases (74/87 vs. 60/87, $p = 0.014$, and 57/64 vs. 52/64, $p < 0.001$)
[17]	119	Biochemical recurrence	Retrospective	^{68}Ga-PSMA-11	LR detection[a]	Histopathology or follow-up imaging and PSA response after salvage therapy	LR in 18/119 patients on mpMRI of PET/MRI but only 9 were PET-positive on PET/CT and PET/MRI ($p = 0.004$); detection of LR on PET was influenced by proximity to the bladder ($p = 0.028$)
[18]	26	Metastatic PC	Retrospective	^{68}Ga-PSMA-11	Initial diagnosis and staging	Histopathology or response to treatment on follow-up imaging and PSA	No difference in detection of PSMA-positive lymph node and bone metastases on PET/MRI and PET/CT (98.5% of 64 lymph nodes and 100% of 28 bone lesions concordant)
[19]	32	Newly diagnosed PC or biochemical recurrence	Prospective	^{11}C-Choline	Initial diagnosis and staging (9), recurrence detection and restaging (23)	None	No significant difference in choline-positive lesions on PET/MRI and PET/CT (77/80 vs. 79/80)

MpMRI multiparametric MRI, *LR* local recurrence, *PC* prostate cancer

[a] Comparison restricted to the abdomen with focus on local recurrence

Three studies compared FDG PET/MRI and FDG PET/CT for restaging of suspected recurrence of malignancy. In a prospective study including 19 patients with clinically suspected recurrence of ovarian (11 patients) or cervical (8 patients) cancer, Beiderwellen et al. found that both FDG PET/MRI and FDG PET/CT correctly identified all 58 metastatic lesions in 16 patients according to the reference standard of histopathology, follow-up imaging or consensus reading based on available prior imaging [21]. Of the 58 malignant lesions, 57 were FDG-avid and the four lung metastases detected were 7–10 mm in size. Rated on a three-point scale, the mean diagnostic confidence for malignant and benign lesions was significantly higher for FDG PET/MRI than for FDG PET/CT (2.78 vs. 2.65, $p < 0.01$, and 2.18 vs. 1.74, respectively, $p < 0.05$), meaning that the readers felt more secure in the discrimination of benign and malignant findings on PET/MRI. In accordance with these findings, in the retrospective study by Kirchner et al. including 43 patients with suspected recurrence of pelvic malignancy, FDG PET/MRI and FDG PET/CT identified 36 and 37, respectively, of the 38 patients with tumour relapse and did not differ in the detection of the totals of 113 malignant and 41 benign lesions [22]. In the retrospective study by Grueneisen et al. including 24 patients with suspected recurrence of ovarian (13 patients), cervical (7 patients) or endometrial (4 patients) cancer, FDG PET/MRI and FDG PET/CT correctly identified 20 of the 21 patients with tumour relapse and did not differ in the detection of 66 of 81 malignant lesions [23].

Gastrointestinal cancer

Of the 13 studies on GIC, eight were outside the scope of this review, three were explorative investigating the correlation between mpMRI values and prognosis, response to treatment or survival in pancreatic and gastric

Table 4 PET/MRI compared to PET/CT in gynaecological cancer: study designs and outcomes

Reference	Number of patients	Population	Study design	PET tracer	Clinical area of application	Reference standard	Findings
[20]	18	Cervical (11) or endometrial (7) cancer	Prospective	^{18}F-FDG	Initial diagnosis and staging	Histopathology and clinical/-surgical staging	No difference in detection of primary tumour, regional lymph nodes or abdominal metastases; parametrial or bladder invasion in 6 patients identified on PET/MRI only
[21]	19	Suspected recurrence of ovarian (11) or cervical (8) cancer	Prospective	^{18}F-FDG	Recurrence detection and restaging	Histopathology or follow-up imaging	The 58 metastatic lesions found in 16 patients were correctly identified on both PET/MRI and PET/CT
[22]	43	Suspected recurrence of pelvic malignancy	Retrospective	^{18}F-FDG	Recurrence detection and restaging	Histopathology or follow-up imaging	PET/MRI and PET/CT correctly identified 36/38 and 37/38 patients with tumour relapse; no difference in detection of the 113 malignant lesions
[23]	24	Suspected recurrence of ovarian (13), cervical (7), or endometrial (4) cancer	Retrospective	^{18}F-FDG	Recurrence detection and restaging	Histopathology or follow-up imaging	PET/MRI and PET/CT both correctly identified 20 of the 21 patients with tumour relapse; no difference in detection of number of malignant lesions

cancer, three compared PET/MRI and MRI, CT or PET alone for staging/restaging in gastric and colorectal cancer, and one was a feasibility study evaluating the image quality of PET in neuroendocrine tumour (NET) (Supplementary data S2, 66–73). Five studies compared PET/MRI and PET/CT in staging/restaging: two prospective studies using ^{68}Ga-DOTA-labelled PET tracers in NET, one prospective study in pancreatic cancer, and two retrospective studies in colorectal cancer (Table 5).

In 30 patients with NET referred for staging/restaging, Sawicki et al. found no difference in staging or number of detected lesions with ^{68}Ga-DOTATOC PET/MRI and ^{68}Ga-DOTATOC PET/CT [24]. With both modalities, staging was in agreement with the reference standard of histopathology or a combination of previous and follow-up imaging in patients who were tumour-free ($n = 5$), had localized or locoregional metastases ($n = 3$), or distant metastases ($n = 22$), and ^{68}Ga-DOTATOC PET/MRI and ^{68}Ga-DOTATOC PET/CT detected 183 and 185 of a total of 142 NET and 55 non-NET lesions, respectively. However, ^{68}Ga-DOTATOC PET/MRI correctly identified a significantly higher proportion of NET-like lesions compared to the reference standard than ^{68}Ga-DOTATOC PET/CT (90.8% vs. 86.7%, $p = 0.031$). Of note, ^{68}Ga-DOTATOC PET/CT failed to detect ten PET-negative subcentimetre liver metastases, and ^{68}Ga-DOTATOC PET/MRI failed to detect three subcentimetre bone metastases and two pulmonary metastases. Berzaczy et al. compared ^{68}Ga-DOTANOC PET/MRI and ^{68}Ga-DOTANOC PET/CT for the detection of distant metastatic disease in 28 patients referred for staging (6 patients) or restaging after surgery (22 patients) [25]. This study had several methodological flaws, and the number of lesions identified was not stated, characterized or related to the reference standard, hampering meaningful evaluation and interpretation.

In 37 patients with pancreatic cancer, Joo et al. compared FDG PET/MRI and FDG PET/CT plus multidetector CT for preoperative evaluation of resectability (local tumour extent and presence/absence of distant metastases) and staging. FDG PET/MR and FDG PET/CT did not differ in accuracy in the patients with confirmed information on resectability (8 resectable, 12 unresectable), N stage (3 node-negative, 10 node-positive), and M stage (13 M0, 4 M1) [26].

In 26 patients with colorectal cancer referred for initial staging/restaging, Catalano et al. compared FDG PET/MRI and FDG PET/CT and found concordant staging in 19 patients which was correct in 18 patients and incorrect in one. In the seven patients with discordant staging by the two modalities, the FDG PET/MRI stages were correct and led to upstaging in four patients, as FDG PET/MRI identified peritoneal carcinomatosis and lymphadenopathy missed by FDG PET/CT [27]. In 15 patients with metastatic colorectal cancer, Brendle et al. retrospectively compared FDG PET/MRI and FDG PET/CT of the abdominal and pelvic regions and basal lungs for staging/restaging [28]. They evaluated 180 lesions of which 110 were malignant, and found no difference between FDG PET/MRI including diffusion-weighted

Table 5 PET/MRI compared to PET/CT in gastrointestinal cancer: study designs and outcomes

Reference	Number of patients	Population	Study design	PET tracer	Clinical area of application	Reference standard	Findings
[24]	30	NET	Prospective	^{68}Ga-DOTATOC	Initial diagnosis and staging (2), recurrence detection and restaging (17), follow-up after remission (11)	Histopathology or follow-up imaging	Identical staging in per-patient analysis; PET/MRI yielded a higher proportion of correctly rated NET lesions than PET/CT
[25]	28	NET	Prospective	^{68}Ga-DOTANOC	Staging (6), follow-up after surgery (22)	Composite from previous and/or follow-up examinations	Inconclusive
[26]	37	Pancreatic cancer	Prospective	^{18}F-FDG	Initial diagnosis and staging	Surgical records, histopathology and imaging-based decisions	No difference between PET/MRI and PET/CT in evaluation of resectability or N and M staging
[27]	26	CRC	Retrospective	^{18}F-FDG	Initial diagnosis and staging (14), recurrence detection and restaging (12)	Histopathology or prior/follow-up imaging	PET/MRI and PET/CT were discordant in the staging of 7 patients, with PET/MRI staging being correct and overall more accurate ($p = 0.02$)
[28]	15	Metastatic CRC	Retrospective	^{18}F-FDG	Initial diagnosis and staging (14), recurrence detection and restaging (12)	Histopathology and/or follow-up imaging	No difference in PET/MRI (including diffusion-weighted) and PET/CT for overall staging; PET/MRI had higher accuracy for detection of liver metastases

CRC colorectal cancer, *NET* neuroendocrine tumour

imaging and FDG PET/CT in overall staging or lesion-based accuracy (0.69 for FDG PET/MRI vs. 0.66 for FDG PET/CT). However, FDG PET/MRI had significantly higher accuracy for the detection of liver metastases (0.74 vs. 0.56, $p = 0.006$).

Discussion

The principal finding of this systematic scoping review was that only a limited number of studies comparing PET/MRI and PET/CT in clinical application areas outside the brain have been published so far. Although PET/MRI has an added clinical value in paediatric oncology, only two paediatric studies were identified and they did not meet with the inclusion criteria. Disregarding studies with very inhomogeneous and mixed cancer populations or on lung cancer, the evidence up to now relates to five major cancers: HNC, BC, PC, GC and GIC. There is evidence to suggest that FDG PET/MRI is superior for staging in nasopharyngeal cancer and in advanced BC, detection of cervical cancer invasion, and detection of local recurrence in PC. FDG PET/MRI tends to be inferior with regard to detection of lung metastases. In colorectal cancer, FDG PET/MRI may be equal or superior to FDG PET/CT for staging and may show higher accuracy for the detection of liver metastases.

Search strategy

Our ambition and goal with this review were to provide an overview of the pros and cons for using PET/MRI instead of PET/CT in the evaluation of major cancers. This is a fundamental question in clinical departments starting up a new PET/MRI facility: which oncological patients should undergo PET/MRI rather than PET/CT and for what reasons? To cover this field we chose the scoping review methodology that provides a new way of mapping and examining available evidence in an emerging field [29, 30]. A scoping review may be particularly useful when the topic has not yet been extensively reviewed or is of a complex or heterogeneous nature, as is the case for the clinical use of PET/MRI in oncology. However, although sharing a number of steps and processes with the systematic review methodology, the scoping method mainly provides a descriptive overview in terms of volume,

nature and characteristics of the primary research, and does not allow quality assessment or critical appraisal of individual studies. Another limitation is that with its broad focus and large yield of articles, the elaboration of a comprehensive synthesis of the literature retrieved by the scoping approach is a challenge. We, therefore, limited our search and focused on cancer entities outside the brain, since cerebral malignancies are a particularly comprehensive field which deserves its own review.

Findings in head and neck cancer

Prospective data from a large and homogeneous patient population have shown that the diagnostic performance of FDG PET/MRI is at least as good as that of FDG PET/CT for initial staging in nasopharyngeal carcinoma and that FDG PET/MRI may have higher accuracy with regard to determining local tumour extension into poorly accessible anatomical spaces and the detection of retropharyngeal lymph node metastases than FDG PET/CT and stand-alone MRI [4]. Nasopharyngeal carcinoma may metastasize to the lungs, and both PET and MRI have poor sensitivity for the detection of subcentimetre lung nodules. In the study by Chan et al. only three lung metastases of unreported size were identified and visible on both FDG PET/CT and FDG PET/MRI [4]. Other studies have shown that metastatic lung nodules less than 5–6 mm in size may be overlooked on PET/MRI [31–33], and further studies are therefore needed to clarify whether supplementary lung CT is or is not necessary for initial staging in nasopharyngeal carcinoma. We identified two additional studies comparing FDG PET/CT and FDG PET/MRI in patients with HNC [5, 6]. Neither of these showed a difference between FDG PET/CT and FDG PET/MRI in the detection of tumour or recurrence, but the study populations were small and heterogeneous. This and suboptimal study designs, insufficient reference standards, and risk of type II errors, meant that conclusions as to the superiority of one of these hybrid imaging techniques could not be drawn from either of these studies. On the whole, it appears that FDG PET/MRI may be a valuable tool for whole-body staging of nasopharyngeal and possibly other types of HNC because of superiority in the identification of local tumour extension and detection of retropharyngeal lymph nodes, but further prospective studies are clearly needed to confirm this and clarify whether additional lung CT is needed.

PET/MRI may theoretically be an attractive solution for the evaluation of differentiated thyroid cancer where the use of diagnostic CT with iodinated contrast agents may interfere with subsequent radioiodine treatment. We identified three studies comparing PET/MRI and PET/CT in differentiated and dedifferentiated thyroid cancer using ^{18}F-FDG, ^{124}I or ^{68}Ga-DOTATATE, and none of them showed any difference between modalities with regard to tumour detection at the patient level [7–9]. However, Vrachimis et al. found that ^{68}Ga-DOTATATE PET/MRI was inferior in the evaluation as it missed lung metastases detected by low-dose ^{68}Ga-DOTATATE PET/CT [7]. Thus, the limited data available are not yet in favour of PET/MRI replacing current clinical algorithms or playing a significant role in the evaluation of thyroid cancer.

Findings in breast cancer

The four studies comparing FDG PET/MRI and FDG PET/CT for staging/restaging of BC concurrently indicated superior diagnostic performance of FDG PET/MRI for the detection of small liver metastases and possibly bone marrow metastases, but inferior sensitivity for the detection of lung metastases.

The role of hybrid imaging in BC is not fully established, and clinical practice guidelines in oncology consider FDG PET/CT optional in patients with intermediate- to high-risk or recurrent disease. Probably therefore, studies directly comparing FDG PET/MRI and FDG PET/CT in BC are not currently available. However, there are studies showing superiority of FDG PET/CT for the detection of metastatic disease compared to conventional imaging in patients with American Joint Committee on Cancer stage IIB–IV leading to significant upstaging, which has implications for the choice of treatment [34, 35]. As MRI is a suitable imaging modality for evaluation of the brain and liver which are frequent metastatic sites in BC, PET/MRI may be a superior modality for staging/restaging of BC. Furthermore, PET/MRI may offer a one-stop comprehensive diagnostic modality for patients with high-risk BC who currently undergo numerous supplementary imaging studies such as bone scan, CT, ultrasonography, MRI and possibly PET/CT.

Catalano et al. compared FDG PET/MRI and FDG PET/CT for initial staging in patients with newly diagnosed BC and found significantly better staging performance with FDG PET/MRI [10]. The superiority of FDG PET/MRI was ascribed to higher sensitivity of MRI for detecting non-FDG-avid bone and liver lesions. Of note, the head was not included in the scan field and potential brain metastases could not be evaluated. Also, Catalano et al. concluded that FDG PET/MRI was superior to FDG PET/CT for detecting bone metastases based on findings in 25 patients with initial or recurrent BC including three patients who were bone metastasis-negative on FDG PET/CT [13]. Of the 141 bone lesions detected on FDG PET/MRI, 68 were classified as having a permeative appearance potentially sparing the cortex. If non-FDG-avid, these metastases would probably not be identified by the CT

part of PET/CT due to lack of cortical changes, suggesting that permeative skeletal MRI changes might to some degree represent malignant bone marrow changes different from late-appearing structural changes, that are frequently seen on both CT and MRI, but which do not necessarily represent active malignancy [36]. The improved sensitivity of FDG PET/MRI for the detection of liver metastases was confirmed by Melsaether et al. in a prospective study including BC patients referred for staging, disease monitoring or restaging [12]. None of the studies showed a difference in the detection of lymph node metastases. However, the two mentioned staging studies concurrently showed inferior sensitivity of FDG PET/MRI for the detection of lung metastases, suggesting that supplementary lung CT evaluation may be needed for comprehensive evaluation.

Findings in prostate cancer

The five studies comparing PET/MRI and PET/CT for staging/restaging of PC suggest that ^{68}Ga-PSMA-11 PET/MRI may be a one-stop modality with superior diagnostic value in patients with biochemical recurrence of PC. ^{68}Ga-PSMA-11 PET/MRI and ^{68}Ga-PSMA-11 PET/CT had equal diagnostic performance in primary staging, probably reflecting the fact that PSMA is a strong biomarker of PC lesions. The three retrospective studies using ^{68}Ga-PSMA-11 for staging/restaging concurrently showed equal performance of PET/MRI and PET/CT with regard to detection of lymph node and bone metastases, all of which were PET-positive [15, 17, 18]. Together the studies indicate equal diagnostic performance of ^{68}Ga-PSMA-11 PET/MRI and ^{68}Ga-PSMA-11 PET/CT for initial staging and the superiority of PET/MRI in restaging of biochemical recurrence, suggesting that ^{68}Ga-PSMA-11 PET/MRI should be reserved for the latter where it may be a superior and one-stop modality. Importantly, these indications are based on small datasets from retrospective single-centre studies, but are nevertheless promising and should be further explored in large prospective and, preferably, multicentre trials including homogeneous patient populations.

The results and conclusions of the two prospective studies using ^{11}C-choline for staging/restaging were different. Eiber et al. found that PET/MRI detected more local recurrences but fewer lymph node and bone metastases than PET/CT [16], whereas Souvatzoglou et al. did not find any difference between the modalities, but in the absence of a reference standard confirming the suspicion of malignancy [19]. Overall, the findings confirm that choline is a tracer with known limited sensitivity for the detection of PC lesions especially in patients with low PSA levels, which leaves a more prominent role for the CT and MRI parts. The clinical significance of this is probably low as the focus is moving towards the use of PSMA, because of its higher specificity.

Findings in gynaecological cancer

The four studies comparing FDG PET/MRI and FDG PET/CT for staging/restaging of GC did not show any difference in lesion detection suggesting that there is neither diagnostic benefit nor disadvantage in using FDG PET/MRI in the clinical evaluation of these patients. However, the data are limited and must be confirmed with elimination of the risk of type II error before FDG PET/MRI can be recommended as a substitute for FDG PET/CT in patients in whom radiation exposure is an issue.

Only one study compared FDG PET/MRI and FDG PET/CT for primary staging and showed no differences in detection of tumour, regional lymph nodes or abdominal metastases in a heterogeneous cohort of 18 patients with cervical or endometrial cancer [20]. However, parametrial and bladder invasion by cervical cancer seen on FDG PET/MRI and not detected on FDG PET/CT resulted in upstaging in five patients, suggesting improved diagnostic accuracy and added clinical value of PET/MRI. The study protocol did not include MRI outside the abdominal–pelvic region, and evaluation of thoracic and supraclavicular lymph node metastases or potential lung metastases was not possible and should be addressed in future studies before a position on FDG PET/MRI as a one-stop modality can be taken.

Three studies compared FDG PET/MRI and FDG PET/CT for restaging in patients with suspected recurrence of pelvic malignancy and showed no difference in lesion detection. The studies included small, heterogeneous populations of patients with cervical, ovarian, endometrial, vulva and vaginal cancers with different tumour biologies and characteristics, and the findings may have been influenced by type II errors. Furthermore, in the study by Beiderwellen et al., all detected malignant lesions including subcentimetre lung metastases showed increased FDG uptake and thus, on this basis, differences between the two modalities are unlikely [21].

Although FDG PET/CT in combination with pelvic MRI is part of standard clinical work-up in initial staging of ovarian and cervical cancers in many centres, we did not find any studies comparing FDG PET/MRI with this approach. Nevertheless, the available data from studies comparing FDG PET/CT and FDG PET/MRI may be used to identify clinical areas in which PET/CT combined with MRI or PET/MRI as one-stop modality may be of added value compared with PET/CT alone. Larger prospective studies may be able to elucidate whether such combinations could be of value in patients at risk of local tumour invasion or with suspected tumour recurrence in previously treated areas where malignant lesions may be difficult to distinguish from benign posttherapy changes.

Findings in gastrointestinal cancer

Two studies compared PET/MRI and PET/CT using DOTA PET tracers in NET. As one of the studies had several methodological flaws with data presentation insufficient for evaluation and interpretation in the context of this review [25], data from only one study in 30 patients referred for staging/restaging of NET are presented [24]. The study showed identical staging results with ^{68}Ga-DOTATOC PET/MRI and ^{68}Ga-DOTATOC PET/CT on a per-patient basis as both modalities correctly identified patients as being tumour-free, having local disease only or having metastatic disease according to the reference standard. However, in a lesion-based analysis, ^{68}Ga-DOTATOC PET/MRI correctly detected a significantly higher proportion of NET lesions than ^{68}Ga-DOTATOC PET/CT, and detected liver metastases not identified on PET/CT. The discrepancy between lesion-based and patient-based findings can probably be explained by the fact that the cohort consisted of patients with advanced NET in whom the detection of additional metastases in the liver did not change overall staging or management. The superiority of PET/MRI for lesion detection in NET is therefore not clear and should be further evaluated in studies including larger cohorts. The same goes for pancreatic cancer, since available data from only one small study did not show any difference in diagnostic performance in the preoperative evaluation of tumour resectability and staging [26].

Two studies with small and heterogeneous populations compared FDG PET/MRI and FDG PET/CT for staging/restaging in colorectal cancer. Catalano et al. found that FDG PET/MRI was superior for staging allowing accurate local and overall staging and restaging in a significant number of patients [27]. In contrast, in a smaller cohort, Brendle et al. did not find any difference in overall staging or lesion-based accuracy [28]. However, FDG PET/MRI had significantly higher accuracy for the detection of liver metastases. The clinical impact of this may be high as the liver is the most significant site of GIC metastases so that detection and characterization of liver metastases is all-important for treatment and prognosis. These limited data suggest a role for FDG PET/MRI in staging/restaging of colorectal cancer, possibly as one-stop modality providing the presence of lung metastases is unlikely, which however, was not addressed in the available studies.

Conclusion

Among the studies included in this scoping review, only a limited number allowed conclusions on the advantages and disadvantages of PET/MRI compared with PET/CT in the oncological setting. In general, study designs, populations and indications varied widely, emphasizing the need for prospective studies with more well-defined purposes and more homogeneous populations to allow a better definition of the clinical role of PET/MRI in oncology. There is current evidence to support the use in staging of primary nasopharyngeal cancer and high-risk BC. The sensitivity of FDG PET/MRI in the detection of lung metastases with is inferior to that of FDG PET/CT, and thus, based on the current evidence, FDG PET/MRI cannot be used as single one-stop modality for staging of cancers where lung metastases are likely. The preliminary data indicate the superiority of PET/MRI for the detection of local recurrence in PC, local tumour invasion in cervical cancer, and liver metastases and staging in colorectal cancer. PET/MRI may be a promising one-stop modality with improved accuracy and added clinical value in PC with biochemical recurrence, advanced cervical cancer and advanced colorectal cancer. These statements are based on small datasets with the risk of type II errors, and need to be confirmed by in well-powered, preferably multicentre, trials including homogeneous patient populations.

Optimal future approaches should include well-designed and well-powered studies focusing not only on diagnostic accuracy but to a high degree also on the impact of PET/MRI on diagnostic and clinical thinking, and on patient management and patient outcome. When possible, they should include cost–benefit or cost-effectiveness analyses of the use of PET/MRI, but without losing focus on what benefits patients most.

Acknowledgements Research librarian L. Østengaard, PhD, University Library of Southern Denmark and Head of Department, and Associate Professor J.S. Madsen, Biochemistry and Immunology, Institute of Regional Health Research, University of Southern Denmark are acknowledged for help with the literature search.

Compliance with ethical standards

Conflicts of interest None.

Ethical approval This article does not describe any studies with human participants or animals performed by any of the authors.

References

1. Peters MD, Godfrey CM, Khalil H, McInerney P, Parker D, Soares CB. Guidance for conducting systematic scoping reviews. Int J Evid Based Healthc. 2015;13:141–6. https://doi.org/10.1097/XEB.0000000000000050.
2. Tricco AC, Lillie E, Zarin W, O'Brien KK, Colquhoun H, Levac D, et al. PRISMA extension for scoping reviews (PRISMA-ScR): checklist and explanation. Ann Intern Med. 2018;169:467–73. https://doi.org/10.7326/M18-0850.
3. Moher D, Liberati A, Tetzlaff J, Altman DG, Group P. Preferred reporting items for systematic reviews and meta-analyses: the PRISMA statement. BMJ. 2009;339:b2535. https://doi.org/10.1136/bmj.b2535.
4. Chan SC, Yeh CH, Yen TC, Ng SH, Chang JT, Lin CY, et al. Clinical utility of simultaneous whole-body (18)F-FDG PET/MRI

as a single-step imaging modality in the staging of primary nasopharyngeal carcinoma. Eur J Nucl Med Mol Imaging. 2018;45:1297–308. https://doi.org/10.1007/s00259-018-3986-3.
5. Schaarschmidt BM, Heusch P, Buchbender C, Ruhlmann M, Bergmann C, Ruhlmann V, et al. Locoregional tumour evaluation of squamous cell carcinoma in the head and neck area: a comparison between MRI, PET/CT and integrated PET/MRI. Eur J Nucl Med Mol Imaging. 2016;43:92–102. https://doi.org/10.1007/s00259-015-3145-z.
6. Kubiessa K, Purz S, Gawlitza M, Kuhn A, Fuchs J, Steinhoff KG, et al. Initial clinical results of simultaneous 18F-FDG PET/MRI in comparison to 18F-FDG PET/CT in patients with head and neck cancer. Eur J Nucl Med Mol Imaging. 2014;41:639–48. https://doi.org/10.1007/s00259-013-2633-2.
7. Vrachimis A, Burg MC, Wenning C, Allkemper T, Weckesser M, Schafers M, et al. [(18)F]FDG PET/CT outperforms [(18)F]FDG PET/MRI in differentiated thyroid cancer. Eur J Nucl Med Mol Imaging. 2016;43:212–20. https://doi.org/10.1007/s00259-015-3195-2.
8. Vrachimis A, Stegger L, Wenning C, Noto B, Burg MC, Konnert JR, et al. [(68)Ga]DOTATATE PET/MRI and [(18)F]FDG PET/CT are complementary and superior to diffusion-weighted MR imaging for radioactive-iodine-refractory differentiated thyroid cancer. Eur J Nucl Med Mol Imaging. 2016;43:1765–72. https://doi.org/10.1007/s00259-016-3378-5.
9. Binse I, Poeppel TD, Ruhlmann M, Gomez B, Umutlu L, Bockisch A, et al. Imaging with (124)I in differentiated thyroid carcinoma: is PET/MRI superior to PET/CT? Eur J Nucl Med Mol Imaging. 2016;43:1011–7. https://doi.org/10.1007/s00259-015-3288-y.
10. Catalano OA, Daye D, Signore A, Iannace C, Vangel M, Luongo A, et al. Staging performance of whole-body DWI, PET/CT and PET/MRI in invasive ductal carcinoma of the breast. Int J Oncol. 2017;51:281–8. https://doi.org/10.3892/ijo.2017.4012.
11. Sawicki LM, Grueneisen J, Schaarschmidt BM, Buchbender C, Nagarajah J, Umutlu L, et al. Evaluation of (18)F-FDG PET/MRI, (18)F-FDG PET/CT, MRI, and CT in whole-body staging of recurrent breast cancer. Eur J Radiol. 2016;85:459–65. https://doi.org/10.1016/j.ejrad.2015.12.010.
12. Melsaether AN, Raad RA, Pujara AC, Ponzo FD, Pysarenko KM, Jhaveri K, et al. Comparison of whole-body (18)F FDG PET/MR imaging and whole-body (18)F FDG PET/CT in terms of lesion detection and radiation dose in patients with breast cancer. Radiology. 2016;281:193–202. https://doi.org/10.1148/radiol.2016151155.
13. Catalano OA, Nicolai E, Rosen BR, Luongo A, Catalano M, Iannace C, et al. Comparison of CE-FDG-PET/CT with CE-FDG-PET/MR in the evaluation of osseous metastases in breast cancer patients. Br J Cancer. 2015;112:1452–60. https://doi.org/10.1038/bjc.2015.112.
14. Taneja S, Jena A, Goel R, Sarin R, Kaul S. Simultaneous whole-body (18)F-FDG PET-MRI in primary staging of breast cancer: a pilot study. Eur J Radiol. 2014;83:2231–9. https://doi.org/10.1016/j.ejrad.2014.09.008.
15. Domachevsky L, Bernstine H, Goldberg N, Nidam M, Stern D, Sosna J, et al. Early (68)GA-PSMA PET/MRI acquisition: assessment of lesion detectability and PET metrics in patients with prostate cancer undergoing same-day late PET/CT. Clin Radiol. 2017;72:944–50. https://doi.org/10.1016/j.crad.2017.06.116.
16. Eiber M, Rauscher I, Souvatzoglou M, Maurer T, Schwaiger M, Holzapfel K, et al. Prospective head-to-head comparison of (11)C-choline-PET/MR and (11)C-choline-PET/CT for restaging of biochemical recurrent prostate cancer. Eur J Nucl Med Mol Imaging. 2017;44:2179–88. https://doi.org/10.1007/s00259-017-3797-y.
17. Freitag MT, Radtke JP, Afshar-Oromieh A, Roethke MC, Hadaschik BA, Gleave M, et al. Local recurrence of prostate cancer after radical prostatectomy is at risk to be missed in (68)Ga-PSMA-11-PET of PET/CT and PET/MRI: comparison with mpMRI integrated in simultaneous PET/MRI. Eur J Nucl Med Mol Imaging. 2017;44:776–87. https://doi.org/10.1007/s00259-016-3594-z.
18. Freitag MT, Radtke JP, Hadaschik BA, Kopp-Schneider A, Eder M, Kopka K, et al. Comparison of hybrid (68)Ga-PSMA PET/MRI and (68)Ga-PSMA PET/CT in the evaluation of lymph node and bone metastases of prostate cancer. Eur J Nucl Med Mol Imaging. 2016;43:70–83. https://doi.org/10.1007/s00259-015-3206-3.
19. Souvatzoglou M, Eiber M, Takei T, Furst S, Maurer T, Gaertner F, et al. Comparison of integrated whole-body [11C]choline PET/MR with PET/CT in patients with prostate cancer. Eur J Nucl Med Mol Imaging. 2013;40:1486–99. https://doi.org/10.1007/s00259-013-2467-y.
20. Schwartz M, Gavane SC, Bou-Ayache J, Kolev V, Zakashansky K, Prasad-Hayes M, et al. Feasibility and diagnostic performance of hybrid PET/MRI compared with PET/CT for gynecological malignancies: a prospective pilot study. Abdom Radiol (NY). 2018;43:3462–7. https://doi.org/10.1007/s00261-018-1665-2.
21. Beiderwellen K, Grueneisen J, Ruhlmann V, Buderath P, Aktas B, Heusch P, et al. [(18)F]FDG PET/MRI vs. PET/CT for whole-body staging in patients with recurrent malignancies of the female pelvis: initial results. Eur J Nucl Med Mol Imaging. 2015;42:56–65. https://doi.org/10.1007/s00259-014-2902-8.
22. Kirchner J, Sawicki LM, Suntharalingam S, Grueneisen J, Ruhlmann V, Aktas B, et al. Whole-body staging of female patients with recurrent pelvic malignancies: ultra-fast 18F-FDG PET/MRI compared to 18F-FDG PET/CT and CT. PLoS One. 2017;12:e0172553. https://doi.org/10.1371/journal.pone.0172553.
23. Grueneisen J, Schaarschmidt BM, Heubner M, Suntharalingam S, Milk I, Kinner S, et al. Implementation of FAST-PET/MRI for whole-body staging of female patients with recurrent pelvic malignancies: a comparison to PET/CT. Eur J Radiol. 2015;84:2097–102. https://doi.org/10.1016/j.ejrad.2015.08.010.
24. Sawicki LM, Deuschl C, Beiderwellen K, Ruhlmann V, Poeppel TD, Heusch P, et al. Evaluation of (68)Ga-DOTATOC PET/MRI for whole-body staging of neuroendocrine tumours in comparison with (68)Ga-DOTATOC PET/CT. Eur Radiol. 2017;27:4091–9. https://doi.org/10.1007/s00330-017-4803-2.
25. Berzaczy D, Giraudo C, Haug AR, Raderer M, Senn D, Karanikas G, et al. Whole-body 68Ga-DOTANOC PET/MRI versus 68Ga-DOTANOC PET/CT in patients with neuroendocrine tumors: a prospective study in 28 patients. Clin Nucl Med. 2017;42:669–74. https://doi.org/10.1097/rlu.0000000000001753.
26. Joo I, Lee JM, Lee DH, Lee ES, Paeng JC, Lee SJ, et al. Preoperative assessment of pancreatic cancer with FDG PET/MR imaging versus FDG PET/CT plus contrast-enhanced multidetector CT: a prospective preliminary study. Radiology. 2017;282:149–59. https://doi.org/10.1148/radiol.2016152798.
27. Catalano OA, Coutinho AM, Sahani DV, Vangel MG, Gee MS, Hahn PF, et al. Colorectal cancer staging: comparison of whole-body PET/CT and PET/MR. Abdom Radiol (NY). 2017;42:1141–51. https://doi.org/10.1007/s00261-016-0985-3.
28. Brendle C, Schwenzer NF, Rempp H, Schmidt H, Pfannenberg C, la Fougere C, et al. Assessment of metastatic colorectal cancer with hybrid imaging: comparison of reading performance using different combinations of anatomical and functional imaging techniques in PET/MRI and PET/CT in a short case series. Eur J Nucl Med Mol Imaging. 2016;43:123–32. https://doi.org/10.1007/s00259-015-3137-z.
29. Pham MT, Rajic A, Greig JD, Sargeant JM, Papadopoulos A, McEwen SA. A scoping review of scoping reviews: advancing the approach and enhancing the consistency. Res Synth Methods. 2014;5:371–85. https://doi.org/10.1002/jrsm.1123.
30. Munn Z, Peters MDJ, Stern C, Tufanaru C, McArthur A, Aromataris E. Systematic review or scoping review? Guidance for authors when choosing between a systematic or scoping review

approach. BMC Med Res Methodol. 2018;18:143. https://doi.org/10.1186/s12874-018-0611-x.

31. Chandarana H, Heacock L, Rakheja R, DeMello LR, Bonavita J, Block TK, et al. Pulmonary nodules in patients with primary malignancy: comparison of hybrid PET/MR and PET/CT imaging. Radiology. 2013;268:874–81. https://doi.org/10.1148/radiol.13130620.
32. Sawicki LM, Grueneisen J, Buchbender C, Schaarschmidt BM, Gomez B, Ruhlmann V, et al. Comparative performance of (18)F-FDG PET/MRI and (18)F-FDG PET/CT in detection and characterization of pulmonary lesions in 121 oncologic patients. J Nucl Med. 2016;57:582–6. https://doi.org/10.2967/jnumed.115.167486.
33. Rauscher I, Eiber M, Furst S, Souvatzoglou M, Nekolla SG, Ziegler SI, et al. PET/MR imaging in the detection and characterization of pulmonary lesions: technical and diagnostic evaluation in comparison to PET/CT. J Nucl Med. 2014;55:724–9. https://doi.org/10.2967/jnumed.113.129247.
34. Groheux D, Cochet A, Humbert O, Alberini JL, Hindie E, Mankoff D. (18)F-FDG PET/CT for staging and restaging of breast cancer. J Nucl Med. 2016;57(Suppl 1):17S–26S. https://doi.org/10.2967/jnumed.115.157859.
35. Riedl CC, Slobod E, Jochelson M, Morrow M, Goldman DA, Gonen M, et al. Retrospective analysis of 18F-FDG PET/CT for staging asymptomatic breast cancer patients younger than 40 years. J Nucl Med. 2014;55:1578–83. https://doi.org/10.2967/jnumed.114.143297.
36. Hoilund-Carlsen PF, Hess S, Werner TJ, Alavi A. Cancer metastasizes to the bone marrow and not to the bone: time for a paradigm shift! Eur J Nucl Med Mol Imaging. 2018;45:893–7. https://doi.org/10.1007/s00259-018-3959-6.

Publisher's note Springer Nature remains neutral with regard to jurisdictional claims in published maps and institutional affiliations.
