# Supplementary material for: Magnetic Fields and Cancer: Epidemiology, Cellular Biology, and Theranostics
Source: Int J Mol Sci. 2022 Jan 25;23(3):1339. doi: 10.3390/ijms23031339 (PMC8835851; doi:10.3390/ijms23031339)
Supplement: Supplementary file 1 [file ijms-23-01339-s001.zip › Supplementary Data Set S1/MF and Cancer.Data/PDF/2190692548/fasebj.12.6.395.pdf]

Biological responses to electromagnetic fields[1]

ADAM LACY-HULBERT,[2] JAMES C. METCALFE, AND ROBIN HESKETH
Department of Biochemistry, University of Cambridge, Cambridge CB2 1TP, England

ABSTRACT **Electrification in developed countries has progressively increased the mean level of extremely low-frequency electromagnetic fields (ELF-EMFs) to which populations are exposed; these humanmade fields are substantially above the naturally occurring ambient electric and magnetic fields of ~10^{-4} Vm^{-1} and ~10^{-13} T, respectively. Several epidemiological studies have concluded that ELF-EMFs may be linked to an increased risk of cancer, particularly childhood leukemia. These observations have been reinforced by cellular studies reporting EMF-induced effects on biological systems, most notably on the activity of components of the pathways that regulate cell proliferation. However, the limited number of attempts to directly replicate these experimental findings have been almost uniformly unsuccessful, and no EMF-induced biological response has yet been replicated in independent laboratories. Many of the most well-defined effects have come from gene expression studies; several attempts have been made recently to repeat these key findings. This review analyses these studies and summarizes other reports of major cellular responses to EMFs and the published attempts at replication. The opening sections discuss quantitative aspects of exposure to EMFs and the incidence of cancers that have been correlated with such fields. The concluding section considers the problems that confront research in this area and suggests feasible strategies.—Lacy-Hulbert, A., Metcalfe, J. C., Hesketh, R. Biological responses to electromagnetic fields. *FASEB J.* 12, 395–420 (1998)**

Key Words: ELF-EMFs · protein kinase C · tumor development · growth factors · cell proliferation · geomagnetic field

THERE HAVE BEEN considerable concern and controversy recently about the effects on health from the increasing exposure of populations to extremely low-frequency electromagnetic fields (ELF-EMFs).[3] These concerns have centered principally on childhood cancer, but other diseases have been similarly implicated. It is generally accepted that EMFs can exert biological effects; they have been widely used in clinical practice to promote processes such as neural regeneration and bone repair (1). However, these treatments generally use magnetic field strengths that exceed those encountered in residential and commercial environments, commonly being high-frequency fields of 1 to 100 mT with a pulsed waveform. Exposure to such fields will not be further considered here both for this reason and because the complex waveforms introduce several confounding exposure parameters not associated with exposure to low-frequency sinusoidal fields (such as the generation of harmonic fields, induction of large electric fields dependent on the rate of switching, and other effects of high-intensity fields).

In contrast to the fields used in clinical practice, those normally encountered by the population are of much lower magnitude and frequency, arising from power distribution systems in which the frequencies generated are 60 Hz [in the United States (U.S.)] or 50 Hz (in Europe and much of the world) and immediate harmonics. Electric and magnetic fields of 50 Hz measured beneath energized power transmission lines are generally in the ranges of 1—10 kVm^{-1} and 1–10 μT, respectively (but can reach 11 kVm^{-1} and 100 μT), and greatly exceed those present in most domestic and commercial environments. Background electric fields in homes are generally 1–10 Vm^{-1}, rising to 20–200 Vm^{-1} near small appliances such as electric razors or hairdryers (2, 3). In immediate proximity to electric blankets or heated water beds, these fields can be as high as several kVm^{-1} and have been implicated in pregnancy complications such as spontaneous abortion or intrauterine devel-

[1] This review is dedicated to Dr. Brian Maddock, who died suddenly in October 1997. Brian Maddock had been Administrator of the EMF Biological Research Trust in the U.K. since its inception and played a major role in coordinating and implementing the research programs funded by the Trust. Through his work in that position, he was well known to everyone involved in EMF research in the U.K., and also to many of our colleagues in the U.S. and in Europe. Throughout this community, he was regarded with much respect and affection.

[2] Correspondence: Department of Medicine, University College London, Rayne Institute, 5 University St., London WC1E 6JJ, U.K. E-mail: a.lacy-hulbert@ucl.ac.uk

[3] Abbreviations: ELF-EMFs, extremely low-frequency electromagnetic fields; PKC, protein kinase C; concanavalin A, Con A; ODC, ornithine decarboxylase; ALL, acute lymphoblastic leukemia; AML, acute myeloid leukemia; RER^+, replication error phenotype; HNPCC, hereditary nonpolyposis colorectal carcinoma; PDGF, platelet-derived growth factor; TPA, 12-*O*-tetradecanoylphorbol 13-acetate; $[Ca^{2+}]_i$, concentration of intracellular free calcium; PHA, phytohemoglutanin; rms, root mean square; IP_3, inositol 1,4,5-triphosphate; IL, interleukin.

opment disorders (4). Background 50 Hz magnetic fields in typical homes are between 0.01 and 1 μT, with appliances generating fields of 0.1–100 μT (**Table 1**; 2, 3). Electric fields arise wherever there is a voltage, regardless of any current in the conductor, and are strongly attenuated by buildings and other objects, including the human body. Magnetic fields, however, are directly proportional to the current flowing in the conductor and are very weakly attenuated by the objects they encounter, although they decrease rapidly in magnitude with distance from the source. Thus, the magnetic fields emitted by domestic appliances are generally undetectable at a distance of 1 m.

The magnetic fields generated by electrical equipment are many times higher than those occurring naturally, and their prevalence is a consequence of technological developments in the second half of the 20th century. Since 1940, U.S. per capita power generation has increased by a factor of 10 and per capita consumption by a factor of 20 (5). Therefore, there has also been a dramatic increase in the exposure of the general population to EMFs over that period of time. Although the increase in exposure is less than that for per capita generation and consumption because of progressive improvements in, for example, underground wiring technology, the collective exposure dose has nonetheless increased by perhaps four- to fivefold since 1940.

EMFs of the magnitude to which we are now regularly exposed have been implicated as a contributory factor in the incidence of childhood cancers, particularly leukemia and brain cancer. Cancers afflict 1 in 600 children below the age of 15 and account for 16% of all United Kingdom (U.K.) childhood fatalities. Brain cancers account for about one-quarter of the childhood cancers and leukemias for one-third, of which the most common is acute lymphoblastic leukemia (ALL). The prognosis for childhood cancers has improved over the last 20 years (the death rate has been halved and the 5-year survival rate for ALL has risen from <10% to >70%) largely as a result of the introduction of combination chemotherapy, but the reported *incidence* of childhood leukemia has also increased, by between 10 and 20%, since 1945. This increase does not necessarily imply an increase in exposure of the population to agents that cause or promote cancer, but could instead arise from improvements in diagnosis and/or widespread changes in childhood disease patterns due to increasing control of infectious diseases. Nevertheless, the incidence of cancer in children and the emotive nature of serious childhood disease mean that reports linking environmental agents to childhood cancer attract considerable public attention; this has been particularly true recently for EMFs, with a number of legal claims against electricity generators and distributors receiving widespread media attention. These claims cite a wide range of epidemiological and biological studies as evidence for a contributory role of EMFs in cancer. This review is intended to survey and review the most significant evidence and to summarize the main issues that EMF research must address.

TABLE 1. *Average magnetic field strengths near commonly encountered sources (U.K.)*

	Average magnetic field, μT[a]			
Distance from source, feet	0.5	1	2	4
Domestic appliances				
Hair dryer	30	4	—	—
Razor	10	2	—	—
Oven	1	0.4	—	—
Microwave oven	20	4	1	0.2
Refrigerator	0.2	—	—	—
Washing machine	2	0.7	0.1	—
Kettle	4	—	—	—
Iron	0.8	0.1	—	—
Toaster	1	0.3	—	—
Vacuum cleaner	30	6	1	0.1
Color television		2	0.2	—
Clock		20	0.2	—
Digital clock	0.4	0.1	—	—
Baby monitor	0.6	0.1	—	—
240V/12V transformer		10	—	—
Office appliances				
Personal computer	10	0.1	—	—
Laser printer	2	0.5	—	—
Electric railway train				
Multiple unit, 25 kV a.c. overhead (measured adjacent to cable drop from roof)	200 (accelerating) 10–30 (cruising)			

[a] 1 μT = 10 mG.

EPIDEMIOLOGY

Early studies of childhood leukemias

The first report of an association between EMFs and cancer emerged from a study of child cancer deaths in which it was concluded that children from homes exposed to high levels of EMFs had a two- to threefold greater risk of developing leukemias or lymphomas than children exposed to lower levels (6). Exposure was classified by using a 'wiring configuration' assessed from observations of electrical wiring and transformers in the vicinity of residences. Further surveys were conducted over the next 12 years (**Table 2**), some of which also concluded that residential exposure to EMFs carried an increased risk of cancers, particularly leukemias, although other studies concluded that there was no significant risk. However, many of these studies were based on small numbers

TABLE 2. *Summary of results of epidemiological studies investigating relationships between residential EMF exposure and cancer*[a]

Reference	Conclusion	Number of cases	EMF exposure assessment	Measure of risk	Cancer risk	Risk estimate (95% CI)
Wertheimer and Leeper, 1979 (6)	Increased risk for all cancer, leukemia, and brain tumors	344	Wiring configuration high/low current	OR	All cancers	2.25 (1.6–3.5)
					Leukemia	2.98
					Brain tumors	2.40
					Other cancer	1.48
Savitz et al., 1988 (15)	Increased risk for all cancer, leukemia, and brain tumors	356	Wiring configuration supplemented by measurements in 50% of homes	OR	All cancers	1.52 (1.04–2.25)
					Leukemia	1.54
					Brain tumors	2.04 (1.11–3.76)
					Other cancer	1.37 (0.84–2.23)
Tomenius, 1986 (16)	Increased risk for all cancer	716	Direct measurements	OR	All cancers	2.05 (1.1–4.2)
					Leukemia	0.34
					Brain tumors	3.86
					Other cancer	14.87
London, 1991 (17)	Increased risk for leukemia	232	Wiring configuration 24 h and single measurements	OR	Leukemia	1.68 (1.1–2.5)
						1.70*
Lin and Lu, 1989 (142)	No increased risk	216	Distance from EMF sources	OR	All cancer	1.30 (0.92–1.84)
					Leukemia	1.31 (0.72–2.21)
					Brain tumors	1.09 (0.50–2.37
Fulton et al., 1980 (143)	No increased risk	119	Wiring configuration	OR	Leukemia	1.09 (0.7–1.6)
Myers et al., 1989 (144)	No increased risk	374	Distance from EMF sources	OR	All cancer	0.98
					Leukemia	1.14
Olsen et al., 1993 (9)	Some evidence of increased risk of all cancers	1707	Distance from EMF source	RR	All cancers	5.6 (1.6–19)*
					Leukemia	6.0 (0.8–44)
					CNS tumors	6.0 (0.7–44)
					Lymphoma	5.0 (0.3–82)
Verksalo et al., 1993 (10)	No increased risk	140	Distance from EMF sources	SIR	All cancers	1.5 (0.74–2.7)
					CNS tumors	2.3 (0.75–5.4)
					Leukemia	1.6 (0.32–4.5)
					Other cancers	1.2 (0.26–3.6)
Feychting and Ahlbom, 1993 (8)	Some evidence of increased risk of childhood leukemia, but not other childhood cancers	142 (39 leuk 33 CNS)	Distance from wires; measurements and calculations from records	OR	Childhood leukemia	2.7 (1.0–6.3)† 3.8 (1.4–9.3)‡
					CNS tumors	No increased risk
					All cancers	No increased risk
Feychting and Ahlbom, 1994 (145)	No increased risk of adult cancers	325 leuk 223 CNS			CNS tumors	No increased risk
					Adult leukemia	No increased risk
Coleman et al., 1989 (147)		771	Distance from EMF sources	OR	Childhood leukemia	1.68
					Adult leukemia	1.00 (0.76–1.31)
Severson et al., 1988 (147)	No increased risk of leukemia	133	Wiring configuration	OR	Acute nonlymphocytic	1.5 (0.8–2.9)
			Measured fields			1.12 (0.57–2.18)§
Youngson et al., 1991 (148)	No increased risk of leukemia/lymphoma	3144	Distance from EMF source	OR	Leukemia or lymphoma	1.27 (0.97–1.66)
Wertheimer and Leeper, 1982 (149)			Wiring configuration	OR	Cancer	1.3 (1.1–1.5)
Linet et al., 1997 (14)	No significant excess of childhood ALL with residual magnetic fields	638	Measured fields	OR	Acute lymphoblastic leukemia (ALL)	1.24 (0.86–1.79)§
			Wiring configuration			0.88 (0.48–1.63)
Tynes, 1997 (150)	No association between childhood cancer and residence near power lines		Calculations from power line distance and records of load		All cancer	No increased risk
					Brain tumors	No increased risk
					Leukemia	No increased risk
					Lymphoma	No increased risk

[a] Risk estimates are shown for the occurrence of cancer based on comparisons with normal subjects: OR, odds ratio; RR, relative risk; SIR, standardized incidence ratio. 95% confidence intervals (CI) are given where available. Except where marked otherwise, the risk estimate is given for the highest exposure group where exposure was assessed by wiring configuration, for >0.30 μT where field exposure was measured or calculated, and for <50 m where exposure was assessed by residence close to sources of EMFs. (Adapted from refs 19, 150). The odds ratio or relative risk is determined by dividing the case exposure ratio (the ratio of those exposed to those not exposed in the group having the disease) by the control exposure ratio. The number of cases refers to the total number of cancer cases included in the study; the number of cases of cancer in the highest exposure groups was much lower. *For highest exposure group, calculated at >0.4 μT. † For exposure >0.2 μT. ‡ For exposure >0.3μT. § For exposure >0.2 μT.

of high field-exposed leukemia cases and many relied on the arbitrary wiring configuration method or distance from transmission lines and distribution centers to estimate exposure. These drawbacks, together with the marginal nature of the positive correlations found, mean that no definite conclusions regarding

direct exposure to high levels of EMFs can be drawn from these studies. Furthermore, the indirect measures of EMF exposure used may also correlate with other factors such as social status or chemical pollution. These unconsidered factors may have contributed to the cancer rates reported and also to the conflicting conclusions arrived at by various EMF studies. It is notable that Wertheimer and Leeper (6) commented that *indirect* effects of EMFs, for example, that change the distribution of particles emitting ionizing radiation could cause cancer, which suggests that factors besides EMF strength could also affect the capability for EMF exposure to cause cancer. This suggestion has recently been extended to include the possibility that aerosol particles containing radon daughter nuclei (^{218}Po and ^{214}Po) undergo enhanced deposition in the lungs under the influence of strong electric fields (7).

The Nordic studies

Three recent reports, often collectively referred to as the Nordic studies (8–10; Table 2), are carefully executed surveys that represent considerable advances over previous reports in terms of exposure measurement and data analysis.

The Swedish case control study (8) was confined to children living near power lines and concluded that there was an increased risk of childhood leukemia at exposure levels of >0.2 μT (odds ratio, O.R., 2.7) or >0.3 μT (O.R. 3.8). The study further concluded that for lymphoma, central nervous system tumors, and all childhood cancers combined, there was no significantly increased risk at any of the exposure levels. Note that the total number of leukemia cases in the high exposure (>0.2 μT) category was only seven. Furthermore, although these authors gave careful consideration to the problems associated with such a survey, there are inconsistencies in the results. For example, leukemia data were analyzed for one-family homes and apartments: for the latter there was no increase in risk, even though spot measurements indicated that fields were significantly higher than in one-family homes.

The Danish case control study (9) concluded there was no significant association of calculated exposures $\geqslant 0.2$ μT with childhood cancers (O.R. 1.5), but that fields $\geqslant 0.4$ μT showed an association with major childhood cancers. This deduction was based on a total of six patients. For leukemia, the case exposure ratio was 3/829, the control exposure ratio 1/1659 (O.R. 6.0); for central nervous system tumors, the corresponding ratios were 2/623 and 1/1864 (O.R. 6.0), for malignant lymphoma 1/247 and 1/1247 (O.R. 5.0).

In the Finnish cohort study (10), ''*no statistically significant increases in all cancers and in leukemia or lymphoma were found in children at any exposure level.*'' A statistically significant excess of nervous system tumors was detected in boys exposed to $\geqslant 0.2$ μT (five cases), but no such tumors occurred in girls similarly exposed. This result probably arose from an anomaly in the study's design, since the excess of brain tumors can be attributed to one boy with three tumors counted as three cases; the relative risk is no longer significant if he is counted as only one case. The conclusion was that ''residential magnetic fields of transmission power lines do not constitute a major public health problem regarding childhood cancer. The small numbers do not allow further conclusions about the risk of cancer in stronger magnetic fields''.

The data from these three studies have been pooled (although the studies do not allow a full meta-analysis) in a subsequent report concluding that, taken together, the studies ''support the hypothesis that exposure to magnetic fields of the type generated by transmission lines has some etiolgical role in the development of leukemia in children'' (11). Therefore, these recent studies together provide evidence for an association between childhood cancers and exposure to EMFs. However, the inconsistencies within individual studies and between the broadly similar studies, along with the small magnitude of the positive associations reported, indicate the difficulties of acquiring convincing epidemiological data in this field. The lack of evidence for an association between brain tumors and EMF exposure is confirmed by some recent studies (12, 13).

The National Cancer Institute Study

The most comprehensive study to date of residential exposure to EMFs and childhood ALL involved 629 children with leukemia, 619 controls, and the direct measurement of magnetic fields at multiple locations in the current and former homes of the subjects (14). Wire code classifications were also assigned to 408 matched pairs of children; all magnetic field measurements and wire code estimations were carried out blind with respect to the health status of the resident subject. This study revealed no association between a risk for ALL and electromagnetic field strength within the homes of the children, nor was there any increase in risk with increasing wire code. Moreover, there was no association between risk of ALL and magnetic field strength or wire code in the residences occupied by the mothers while pregnant in those cases analyzed. These data contrast with earlier studies (6), but are consistent with others (15–17) that have shown no significant increase in risk of ALL for children exposed to residential levels of magnetic fields that exceed 0.2 μT. The study by Linet et al. (14) carries considerable weight not only because of the large number of subjects and the care taken to minimize bias in data acquisition, but also because magnetic field measurements were generally made

within 2 years of diagnosis of leukemia, a much shorter interval than in most earlier surveys.

Adult studies

Numerous investigations of the incidence of adult cancers have used approaches similar to those of the childhood surveys summarized above (Table 2). None have provided convincing evidence that exposure to above-ambient levels of EMFs in the home influences the development of leukemias, lymphomas, or solid tumors (18). However, several studies have considered whether employment in occupations with high EMF exposure may be associated with an elevated incidence of brain cancers and leukemias. As with the many residential studies, the initial occupational surveys were based on crude classifications. Fields were not measured in individual cases, the evidence for increased exposure being based either on a classification of the occupation or, later, on a measurement to determine an average exposure for a given occupation. This feature of the study, coupled with the marginal effects in some studies, the lack of correlations in the remainder, and the possibility of a publication bias in favor of reports showing increases, limits the overall conclusions that can be drawn (19, 20).

Five large and more detailed recent surveys show broad agreement and indicate a marginally increased risk of brain cancer and/or leukemia. Of these, a joint France/Canada study of hydroelectric power workers (21, 22) revealed a significant increase in acute myeloid leukemia (AML) and chronic nonlymphocytic leukemia with greater than median EMF exposure (relative risk estimate for leukemia of 1.5 and for AML of 2.7), although there was no correlation between risk and length of exposure and the survey showed no consistency between the results obtained from different utility companies. The risk of brain cancer was increased in individuals who experienced the highest 10% of the exposure range. Similarly, a study of French electricity workers showed an increased risk of brain cancers for those exposed to the highest 10% of electric fields (OR 3.08 with 95% confidence interval of 1.08–8.74), but no corresponding increase for leukemia (23). Further studies of hydroelectric power workers in Ontario revealed a significant increase in leukemia with high exposure to electric and magnetic fields, with a dose response for exposure to electric, but not magnetic, fields (24). An American study of five major utilities (25) showed a slight increase in brain cancer mortality with cumulative exposure (relative risk factor of 1.94 per μT year of exposure in the 2–10 years before diagnosis), but no increase for leukemia. A Swedish survey (26) estimated exposures of individuals with leukemia or brain cancer (posthumously for many of the cases); this, too, showed an increased risk for chronic lymphocytic leukemia and a slight, but not statistically significant, increase for brain cancer. Although many increases in these studies were not statistically significant, nevertheless every set of data reveals an increase in the number of individuals contracting brain cancer relative to the general population, and three of the studies show statistically significant increases for leukemia. A similar small increase in brain cancers and leukemias alone of 20 different cancers is revealed in a British study of correlations between occupations and cancer and between mortality and occupation. This study is biased slightly toward recording occupations in electrical industries. Conversely, a cohort study of British electricity workers showed no significant increased risk of mortality with exposure to high magnetic fields (27).

Analysis of these studies

It is clear that the epidemiological studies discussed above do not provide a clear answer to the question of whether EMF exposure influences the development of cancer. This is due in part to two shortcomings shared by all of the studies: *1*) small sample number, and *2*) no specific known EMF exposure parameter with which correlations should be made. The problem of small sample numbers in such surveys can be countered in part by undertaking more comprehensive studies, such as the U.K. child cancer study currently in progress. The second problem is more difficult to address because it comprises two components. Regardless of whether exposure is assessed by using a wiring configuration, measurement of distance from source, direct measurement of fields (either as continuous exposure or in bursts), or by classification of occupation, a large subjective element can arise. Second, until the mechanism of putative EMF interactions with organisms is understood, these figures must all be considered as mere surrogates for exposure, and their relationship to any critical parameter of EMF will vary from case to case. This may provide an explanation for apparent anomalies in epidemiological data such as those in the studies by Feychting and Ahlbom (8) and Pool (28), in which estimated risk varied with the exposure classification used. Thus, Feychting and Ahlbom (8) obtained an odds ratio of 1.7 for estimated magnetic fields of $\geqslant$0.2 μT and AML, but the risk estimate was close to unity when spot measurements of magnetic field were used. Finally, the complexity of genetic and epigenetic factors that promote cancers (see below) mitigates against clear epidemiological correlations in all but the most statistically overwhelming cases (e.g., smoking, alcohol, hepatitis B virus infection).

TUMOR DEVELOPMENT

Tumors result from subversion of the processes that control the normal growth, location, and mortality of

cells. Their development is a multistep process involving the activation of proto-oncogenes and/or the loss or inactivation of tumor suppressor genes, which may be inherited or accumulated through somatic mutation. The normal products of proto-oncogenes are components of the signaling pathways that regulate proliferation, which in their mutated forms become dominant oncogenes. Tumor suppressor genes generally exhibit recessive behavior, and their loss of function leads to deregulated control of cell cycle progression and cellular adhesion, etc. (**Fig. 1**).

Changes detected in DNA in cancer range from single base changes to gross chromosomal abnormalities (e.g., translocations and deletions). Tumor initiators such as chemical carcinogens, radiation, UV light, or free radicals cause direct damage to DNA (base alterations, cleavage of DNA strands), which must avoid detection and correct repair by the cell to become a sustained mutation. Components of the cellular DNA repair machinery may also undergo mutation, providing an additional mechanism of genetic alteration leading to cancer. Homozygous mutation in a family of DNA mismatch repair genes leads to a mutator or replication error phenotype (RER^+) characterized by a high level of genetic instability. This is most readily detected in regions of repeated short DNA sequences (microsatellite repeats), which are susceptible to mutation due to their highly repetitive structure. RER^+ cells show shortening or lengthening of microsatellite repeats (microsatellite instability) when compared with normal tissue. Widespread microsatellite instability is a characteristic feature of hereditary nonpolyposis colorectal carcinoma (HNPCC), suggesting the trait is inherited in HNPCC families, but somatic loss of DNA mismatch repair occurs in a large variety of types of tumors, including chronic lymphocytic leukemia. Cells of the RER^+ phenotype will be particularly vulnerable to the effects of free radicals or other DNA damaging agents, since their capacity to repair DNA damage is diminished.

Although abnormally high rates of proliferation can give rise to cancers, tumor development is not merely a consequence of deregulated proliferation. An additional cause is the modulation of the normal processes that lead to cell loss (programmed cell death or apoptosis). Apoptosis occurs normally in the body as a mechanism for clearance of redundant cells in development (e.g., in selection of appropriate reactive cells of the immune response), for the removal of cells at the end of their 'active life' (e.g., granulocytic cells after wound healing), and also as a protective mechanism, removing damaged cells. The tumor suppressor gene product p53 is essential for many apoptotic responses (including response to some DNA damaging agents or to hypoxia). Loss of function of p53 may contribute to tumor development either by permitting damaged cells to continue to proliferate or by preventing differentiation or death of stem cells, both possible precursors of tumor development. These and other patterns of cell behavior have been described mathematically, the model accommodating observed features of cancer development that include the formation of early premalignant lesions that can regress spontaneously (e.g., in cervical intraepithelial neoplasia) and the occurrence of long-lag phases of growth that persist until mutations arise that eventually cause exponential growth (29).

Control of proliferation

The growth of cells can be controlled by the interaction of growth factors with their receptors on the plasma membrane. Such interactions may, for example, cause quiescent, somatic cells to leave G_0, traverse G_1, and enter S phase, whereupon they are normally committed to at least one round of the cell cycle (Fig. 1). Activated growth factors [e.g., platelet-derived growth factor (PDGF) and epidermal growth factor; **Fig. 2**] generate one or more primary signals that promote a sequence of metabolic events, including ionic changes (Ca^{2+} signals, Na^+ influx), activation of protein kinase C (PKC) isoforms, increases in protein phosphorylation, increased activity of ornithine decarboxylase (ODC), activation of glucose, and amino acid uptake and stimulation of glycolysis. In parallel with these events, the coordinated transcription of ~100 genes is activated (30, 31). The 'immediate early response genes' activated within approximately 1 h include ornithine decarboxylase (*ODC1*), *ETS1* and *ETS2*, the *JUN* family (*JUN*, *JUNB*, and *JUND*), and the *FOS* family (*FOS*, *FRA1*, *FRA2*, and *FOSB*). The proto-oncogenes *MYC* and *MYB* are also transcriptionally ac-

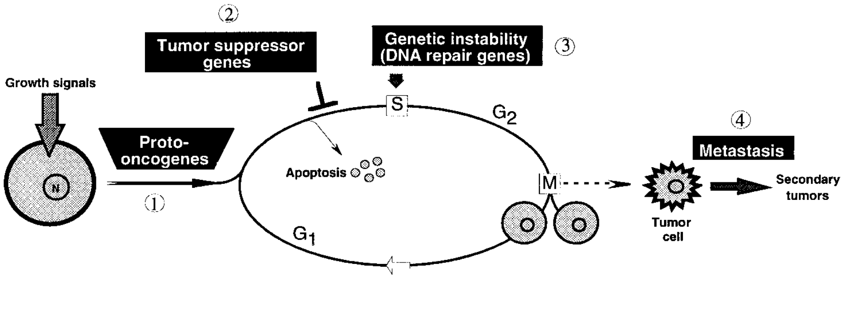

Figure 1. Genetic signals in cancer development. Three broad functional categories may be distinguished within which mutations may arise: *1, 2*) pathways driving cell proliferation and controlling cell cycle progression and apoptosis (proto-oncogenes and tumour suppressor genes), *3*) in the promotion of genetic instability through mutations in DNA repair genes, and *4*) effects associated with metastasis. Reprinted from ref 152 by permission of the publisher (Academic Press, Ltd., London).

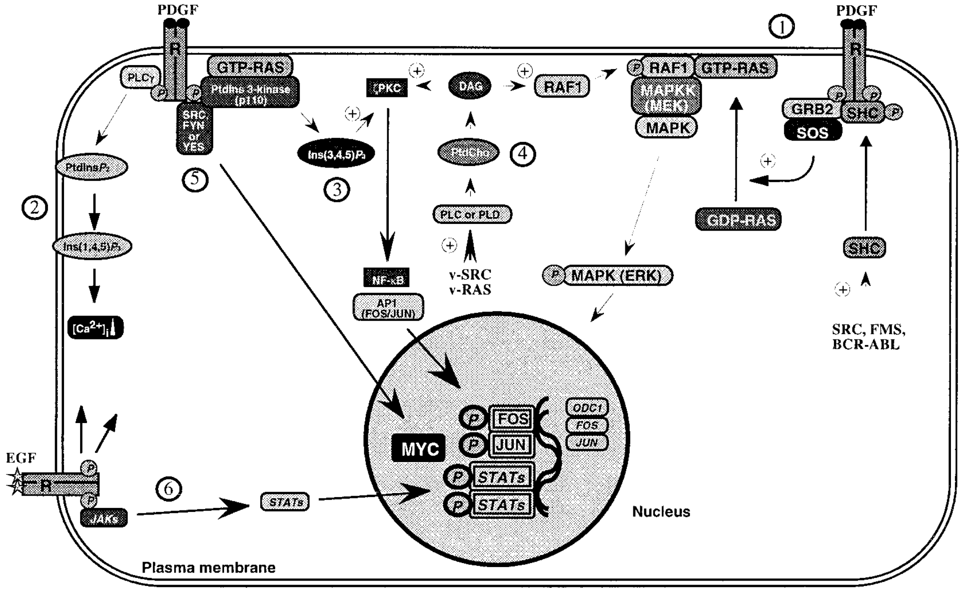

Figure 2. Major growth factor signaling pathways activated by platelet-derived growth factor (PDGF) and epidermal growth factor (EGF). *1*) A major pathway stimulated by PDGF (or by oncoproteins such as SRC, BCR/ABL, FMS) arises from interaction with SH2 domain adapter proteins (SHC, GRB2, SOS) to activate RAS. Activated RAS (GTP-RAS) then causes the initiation of a cascade of protein phosphorylation by serine/threonine kinases including RAF1, MAPKK [or MEK, (*M*AP kinase or *E*RK *k*inase)], and extracellular signal-regulated kinases (ERKs or MAPKs), which ultimately leads to the activation of nuclear transcription factors (152). Additional pathways (not shown) that control cytoskeletal organization may diverge from RAS *via* RHO and RAC. *2*) Activation of phospholipase C γ (PLCγ) leads to elevation of the concentration of intracellular free calcium ($[Ca^{2+}]_i$). *3*) Phosphatidylinositol 3-kinase (PtdIns 3-kinase) also associates with PDGFRs; a product of the activation of this enzyme, phosphatidylinositol 3,4,5-triphosphate (Ins(3,4,5)P_3), activates protein kinase C ζ (PKCζ) *in vitro* (153). PKCζ is a Ca^{2+}-independent isoform that is not activated by phorbol esters, but interacts with RAS-GTP which may promote its translocation to the membrane. Diacylglycerol (DAG) is generated both by the action of PLCγ and the hydrolysis of phosphatidylcholine (PC) by PC-PLC (*4*). The latter, which can be promoted by v-RAS or v-SRC, appears sufficient to transform fibroblasts, the DAG produced stimulating the activity of RAF1 (154). *5*) SRC family kinases (SRC, FYN, or YES) can also bind to the PDGFR to control *Myc* transcription (155). Additional cellular factors not shown here that can bind to the PDGFR include tyrosine phosphatase SYP, NCK, and RAS-GAP. A variety of cytokines (including interleukins, interferons, erythropoietin, growth hormone, prolactin, and granulocyte colony-stimulating factor) signal by activating members of the Janus family of protein kinases (JAK1, JAK2, JAK3, and TYK3) that phosphorylate on tyrosine residues various STAT proteins (signal transducers and activators of transcription), which are then translocated to the nucleus to direct gene transcription. In addition to cytokines, growth factors can also activate the JAK/STAT signaling pathway. *6*) Epidermal growth factor (EGF) causes the tyrosine phosphorylation of STAT1, which in turn mediates the activation of *FOS* transcription. Thus EGF, which can activate the RAS signaling pathway *via* SHC and GRB2/SOS, as well as PtdIns 3-kinase and PLCγ (not shown), can also activate the JAK/STAT pathway. Reprinted from ref 152 by permission of the publisher (Academic Press Ltd., London).

tivated approximately 2 and 6 h after cell stimulation, respectively. Appropriate combinations of these responses drive cells through the cell cycle and, in principle, any component of these pathways possesses oncogenic potential (Fig. 2).

Patterns of mutations in cancers

It is not known how many mutations are required for the genesis of a specific cancer, but it is generally supposed that more than five are involved; in all but a small proportion of cases (<1%), these mutations are acquired somatically. Malignant colorectal carcinomas have been particularly informative in this respect (32). The majority of these cancers develop from benign adenomas in a process in which DNA hypomethylation occurs together with the acquisition of mutations in *KRAS2*, *APC*, *MCC*, *DCC*, and *P53*. The mutation or deregulation of a single component of a signaling pathway is not sufficient for the immediate development of a tumor, and recognizable stages of tumor progression can often be defined. This is well illustrated by in vitro cellular studies that show cell transformation to be at least a two-stage process, one oncogene being needed for immortalization (proliferation of cell through endless rounds of cell division without senescence) and another for full transformation (loss of checks to growth such as contact inhibition, stimulation by growth factors). Thus, primary fibroblasts can often be immortalized by *RAS*. Full transformation, however, generally requires the expression of two complementing oncogenes, typically *RAS* and *MYC*.

Animal models of tumorigenesis

The notion of progressive accumulation of mutations is also suggested by experimental animal models of

tumorigenesis. The chemical induction of tumors generally requires the sequential application of two types of agents: an 'initiator' and a 'promoter'. The initiator is usually a mutagen that has irreversible effects and must be administered before the promoter. These model systems indicate that the initiating event in tumorigenesis is mutation and that subsequent tumor progression may be mediated by either genetic or epigenetic mechanisms. The classical system for studying the chemical initiation of tumors in animals is the mouse skin carcinoma model (33). The application of a low dose of a carcinogen to mouse skin can cause mutations in oncogenes, most frequently in *Hras-1* (34), but causes carcinoma only if followed by multiple treatments with a tumor promoter. Tumor promoters are not themselves mutagens; alone they have no carcinogenic properties, but can stimulate cell proliferation. It seems probable that tumor promoters act in carcinogenesis to stimulate growth of a population of cells carrying an initial mutation, thereby increasing both the number of cells that carry this mutation and the probability of subsequent mutations occurring as cells are driven through successive rounds of DNA replication. Any process that perturbs the normal cell growth control mechanisms, for example, by modulating the activity of a component of a signaling pathway, could promote tumor formation either by stimulating cell proliferation or by removing checks to growth. Tumor promoters such as TPA (12-*O*-tetradecanoylphorbol 13-acetate) act by direct activation of PKC or by inactivation of protein phosphatases, and are presumed to act by increasing phosphorylation of substrates involved in mitogenic signaling pathways. It is established that anomalous stimulation by, for example, heat shock or UV radiation can dramatically activate transcription of the early response genes *FOS* (**Fig. 3**) and *JUN*. Normal growth factors synthesized in an appropriate setting may also act as 'promoters' in the early developmental stages of cancer. For example, gastric-releasing peptide (GRP or mammalian bombesin) functions as an autocrine growth factor in small cell lung cancer: these tumor cells produce large amounts of the peptide, which causes phosphatidylinositol-(4,5)-bisphosphate hydrolysis and an increase in the concentration of intracellular free calcium ($[Ca^{2+}]_i$), characteristic responses of cells entering the cell cycle (35). In this situation, the growth factor may act selectively to promote the proliferation of a clone of tumor cells.

HOW MIGHT EMFS CAUSE CANCER?

The concepts of initiation, promotion, and sequential accumulation of mutations prompt the consideration of two major pathways whereby an environmental agent such as EMF might cause cancer.

EMFs as mutagenic agents

EMFs might act in the same manner as conventional carcinogens to cause mutations that either initiate the progression of a cell to acquisition of a fully tumorigenic complement of abnormalities or accelerate its progress along that pathway. Although the energy associated with environmental EMFs is too low to cause direct changes to the structure of DNA, EMFs might affect the production of agents such as free radicals, which themselves can react with DNA, or of other agents that cause chromosomal damage, instigating translocation by inducing DNA breaks or by formation of unnatural DNA structures. Interfering with the mechanisms of DNA repair or chromosomal replication and segregation can also cause

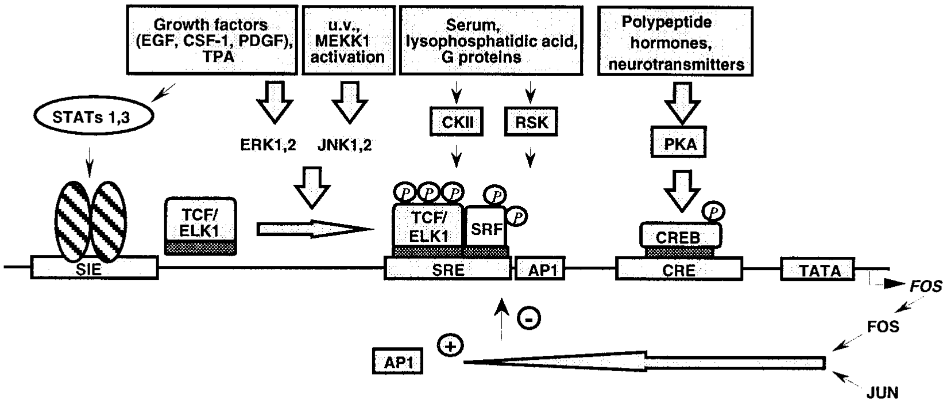

Figure 3. Transcriptional control of *FOS*. The activation of *FOS* transcription through a number of control elements (SIE, SRE, AP1, CRE) is represented as being activatable by a variety of stimuli. Activation may be promoted *via* STATs 1 or 3 (signal transducers and activators of transcription), ERKs 1 or 2, JUN amino-terminal kinases 1 or 2 [JNKs, also called stress-activated protein kinases (SAPKs)], casein kinase II (CKII), S6 kinase (RSK), or cAMP-dependent protein kinase (PKA). Adapted from M. Karin (1994) Signal transduction from the cell surface to the nucleus through the phosphorylation of transcription factors. *Curr. Opin. Cell Biol.*, vol. 6, pp. 415–424, by permission of the publisher (Current Biology Ltd).

DNA damage or increase the probability that a particular DNA sequence will be lost from the genome.

A wide variety of experimental approaches have been used to detect mutagenic effects of EMFs, including assays for point mutations or analysis of gross DNA alterations such as DNA breaks, translocations, the formation of micronuclei, or the presence of cells with an inappropriate chromosome complement (**Table 3**). In bacterial studies, strains of Salmonella exposed to 15 or 30 mT magnetic fields at 0.3 Hz, 1–10 μT sinusoidal magnetic fields at 50 Hz applied in 2 μs pulses or to a wide range of radio frequency and microwave fields showed no increase in mutational frequency (36). Fields (0.2 mT) at 60 Hz have been reported to be comutagenic in Salmonella, causing a 14% increase in azide-induced mutations after 48 h exposure (37). Although EMF exposure increased the number of revertant colonies in every experiment described (a total of 15 experiments), the number of revertant colonies per 'sector' of Petri dish (separate regions of the dishes in which colonies were counted) varied considerably in every experiment, and the resultant errors were significant at the 90%, but not the 95%, confidence level (P=0.078; paired Student's t test). Human lymphocytes exposed to sinusoidal 50 Hz, 60 Hz, or pulsed EMFs did not undergo detectable chromosomal alterations or inhibition of DNA repair mechanisms (36, 38–43). Primary human lymphocytes have been reported to show increased micronuclei formation when exposed to 75 or 150 μT fields at 32 Hz, but only when a parallel 42 μT d.c. field was applied concurrently to establish the cyclotron and paramagnetic resonance condition for calcium (44). The statistical analysis applied led to the conclusion that there was no increase in micronuclei formation in the absence of a d.c. field or at 50 Hz. However, as several of the individual experiments in the absence of a static field showed percentage increases comparable to or exceeding those detected when the 42 μT d.c. field was applied, these data appear to be equivocal. Nordenson et al. (45) reported increases in chromosome defects (gap and break frequencies) in human amniotic cells exposed to a continuous or intermittent 30 μT sinusoidal field at 50 Hz, but attempts to reproduce this work have been unsuccessful (46). The same field exposure conditions were used to investigate the fidelity of chromosome segregation in Chinese hamster cells and no effect of field exposure was seen (47). Hintenlang (48) reported that exposure to ionizing radiation, followed by 60 Hz magnetic fields of up to 1.4 mT, enhanced the frequency of near-tetraploid chromosome complements in human peripheral blood lymphocytes, an effect not seen with ionizing radiation alone. Negative effects have been reported in whole-animal studies: 50 Hz electric fields of 20 kVm^{-1} failed to increase the frequency of dominant lethal mutations in male mice, and 60 Hz fields of as much as 50 kVm^{-1} with magnetic fields of up to 1 mT did not affect the frequency of sister chromatid exchange in mouse bone marrow (19, 20). Thus, there are some reports of positive EMF effects. However, the inadequate internal controls and the failure of replication attempts suggest that EMFs of these strengths are not mutagenic.

EMF effects on chemical reactions involving free radicals

Free radicals are generated as intermediates in metabolism and may attack lipids, proteins, and DNA. Thus, any elevation in free radical production could increase the rate of chemical damage to DNA as occurs, for example, as a consequence of sustained activation of the immune system in response to chronic infection. Magnetic fields of more than 1 mT can have measurable effects on the kinetics and yield of chemical reactions that use geminate radical pairs through their effect on the spin precession rates of unpaired electrons and consequent effects on the lifetime of radicals (49–52). The magnetic field can increase or decrease precession rates between singlet and triplet spin-correlated states. Hence, a geminate radical pair born in the singlet spin state may rapidly recombine; after precession to the triplet spin state, recombination is prohibited by the Pauli exclusion principle, resulting in a longer radical lifetime. The consequence of this may be, for example, increased enzyme product or release of radicals from the enzyme (**Fig. 4**).

Harkins and Grissom (53) have demonstrated the effects of magnetic fields on the enzyme B12 ethanolamine ammonia lyase. Fields of greater than 50 mT caused a decrease in the ratio V_{max}/K_m of the enzyme that was maximal (20% reduction) at 100 mT. McLauchlan et al. (51) describe effects of magnetic fields on a variety of photochemical reactions: the reaction of pyrene with dicyanobenzene generates radical pairs that fluoresce when they recombine. If the reaction is performed in a fluctuating magnetic field of a few mT, the fluorescence generated by the reaction fluctuates with the applied magnetic field. In more complex photochemical reactions, radicals are generated in the triplet state (spins parallel) and must move to the singlet state (spins antiparallel) before reacting. This interchange can occur as a result of local magnetic fields from nearby magnetic nuclei through the hyperfine interaction. When the reaction is performed in a static magnetic field, these interconversions are affected. The magnetic field removes the degeneracies of the triplet state sublevels, and at higher magnetic fields can cause separation between triplet states greater than the hyperfine interaction, effectively preventing interchange of electrons and stopping up to two-thirds of radical pairs reacting (Fig. 4). An opposite,

TABLE 3. *Reported biological effects of ELF-EMFs*[a]

I EMFs as mutagenic agents

Target	Experimental system	Field exposure conditions	Result	Assay	Ref
DNA mutation	Salmonella	15–30 mT, 0.3 Hz<br>1–10 μT, 50 Hz in 2 μs pulses<br>Variety of RF and microwave fields	No increase	Mutation frequency	(36)
	Salmonella treated with azide	0.2 mT, 60 Hz for 48 h	14% increase	Mutation frequency	(37)
	Human lymphocytes	75 or 150 μT at 32 Hz with 42 μT static field	Increased micronuclei	Micronuclei formation	(44)
	Mice	20 kV/m, 50 Hz<br>1 mT, 50 kV/m, 60 Hz	No effect<br>No effect	Gene mutation<br>Chromatid exchange	(19)
Chromosome alterations	Human lymphocytes	Range of sinusoidal fields at 50 Hz, 60 Hz, and pulsed EMFs	No effect	Chormosomal alterations	(37)
	Human lymphocytes	50 Hz, 60 Hz and pulsed EMFs	No effect	Chromosomal alterations	(38–40)
	Human lymphocytes	1.4 mT, 60 Hz, after ionizing radiation exposure	Increased	Chromosome complement	(48)
	Chinese hamster cells	30 μT, 50 Hz	No effect	Chromosome segregation	(47)

II EMF effects on chemical reactions involving free radicals

Experimental system	Field exposure conditions	Result	Assay	Ref
B12 ethanolamine ammonia lyase	>50 mT, maximal effects at 100 mT, static fields	Decrease in activity	Spectroscopic detection of reaction products	(53)
Laser-induced free radicals	0–10 T	Altered recombination of radicals	Fluorescence detection of radicals	(49)
Neutrophil respiratory burst	0.1 mT, 60 Hz	Increase in superoxide production	Fluorescence detection of superoxide products	(54)
Macrophage NO production	1–100 mT static fields or 1.6 mT, 1 Hz fields	No effect	Measurement of nitrite	(55)

III EMFs as tumor promoters

Experimental system		Field exposure conditions	Result	Assay	Ref
Skin tumor promotion	DMBA-treated mice	50 or 500 μT, 50Hz, intermittent 20 h/day, 2 yrs	Small increase	Tumor incidence	(56)
	DMBA/TPA-treated mice	2 mT, 60 Hz, 6 h/day, 23 wk	Small increase	Rate of tumor development	(58, 59)
Mammary tumor promotion	DMBA-treated rats	30 mT, 50 Hz	30% increase	Tumor incidence	(60)

IV In vitro cellular studies

Experimental system		Field exposure conditions	Result	Assay	Ref
DNA synthesis	Stimulated lymphocytes	2.5 mT, 50 Hz sawtooth, 6 h	Increase	Thymidine incorporation	(61)
Cell proliferation	Stimulated lymphocytes	5 mT, 50 Hz sinusoidal		S-phase entry	(43)
	Stimulated lymphocytes	4.5 mT, 3 Hz sawtooth<br>6 mT, 3 Hz square wave	Decreased<br>Decreased	Cell number	(157)
	Fibroblast and mutant cell cultures	0.1 mT, 60 Hz, 1 h/day, 18 days	Twofold increase	Focus formation	(66)
	Tamoxifen/ melatonin-treated breast cancer cells	1.2 μT, 60 Hz	Increased	Cell number	(69, 158)

continued on next page

TABLE 3. (*continued*)

IV In vitro cellular studies

Experimental system		Field exposure conditions	Result	Assay	Ref
Ca^{2+} signaling	Jurkat cells	0.1 mT, 50 μV/m, 50 Hz, 15–20 s	Increased	Ca^{2+} oscillations	(81, 82)
			Increased	IP_3 concentration	
	Stimulated lymphocytes	4.5 mT, 3 Hz sawtooth	Decreased	$^{45}Ca^{2+}$ uptake	(62)
		6 mT, 3 Hz square wave			
	Lymphocytes	6.5 mT, 60 Hz, 1 h	Increased	Con-A induced $^{45}Ca^{2+}$ uptake	(84)
		6.5 mT, 3 Hz square wave, 1 h	Inhibited		
		2 mT, 60 Hz, 2 min	Increased	Mn^{2+} influx	(87)
	Brain tissue		Small changes	$^{45}Ca^{2+}$ uptake	(73)
	Cultured cells		No effect	Ca^{2+} transport	(75)
			No effect	Cytosolic Ca^{2+}	(76)
ODC activity	Human lymphoma cells	1 V/m, 60 Hz, 1 h	Increased	ODC activity	(89)
RNA synthesis		3.5 mT, 2 h	Increased	Uridine incorporation	(159)
Gene expression	Dipteran salivary glands	1 mT, 60, 72 Hz sinusoidal and pulsed signals	Increased	Uridine incorporation	(160)
	T-lymphoid cells	100 μT, 60 Hz, 15–20 min	Increased and decreased	Nuclear run-on assays	(95)
SRC family kinases	Lymphoid cells	100 μT, 60 Hz, 2 min	Stimulated	LYN and SYK kinase activity	(117)
Neurite outgrowth	NGF stimulated rat nerve cells		Increase	Length of cell processes	(70)

V Cell-free systems

Experimental system		Field exposure conditions	Result	Assay	Ref
Calcium–protein interactions	Fluorescent calmodulin binding	20.9 μT sinusoidal field around 16 Hz (cyclotron resonance conditions)	No effect	Fluorescence	(129)
Membrane channel conductance	Black lipid membranes	50–5000 μT	No effect	K^+ conductance	(132)
	GRI-GI cell line		No effect	ATP sensitive K^+ channels	
Membrane ATP-ases	Mitochondria		Stimulate	ATP synthesis	(135)
			Alter activity	ATP synthesis	
Transcription and translation	*E. coli* lysate	70 μT–1.1 mT, 72 Hz	Increased	Radioactive precursor incorporation	(136)

[a] Published effects subjected to rigorous attempts at replication are omitted here, but are summarized in Table 4.

smaller effect may occur at very low field strengths to increase reaction yields by a few percent (due to the Zeeman splitting that removes the constraints of spin angular momentum conservation of the system, allowing singlet radical pairs to interconvert to more triplet states).

Alternating magnetic fields superimposed on static magnetic fields can further affect reactions by providing quanta of energy equal to the gap between singlet and triplet states, allowing transition of radicals and hence increasing reaction probability. The effect requires both static magnetic fields and fields fluctuating at a resonance frequency (generally in the range between low audio and microwave frequencies), dependent on the magnetic fields applied. Magnetic fields of only a few mT can affect chemical reactions and some enzymes that use radical intermediates. The theoretical explanation of these effects is that the fields do not alter the nature of reaction products, merely the yield, although it is possible that radicals escaping reaction due to these effects may leave the vicinity of the reactions and react with other components of a biological system. These examples represent clear, reproducible effects of magnetic fields on biochemical systems with a firm theoretical basis and provide a plausible approach to investigating other reported biological effects of low-strength magnetic fields.

These principles of magnetic field interactions have been investigated in a few intact cell systems,

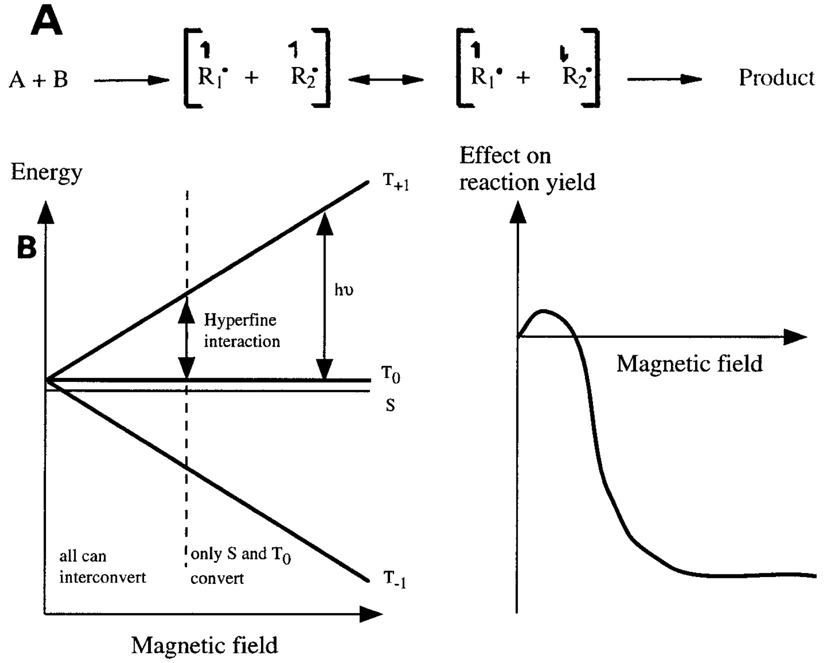

Figure 4. Electromagnetic field effects on free radical processes. *A*) A reaction between two species can generate a pair of radicals in the triplet state with parallel electron spin. If one of the electrons converts to a different state, changing its spin, the radical pair can react to form product. *B*) This change involves transfer of the electron between the three triplet states: T_0, T_{-1}, and T_{+1}. These states are normally degenerate, but in a magnetic field the energies separate. When this separation is less than the hyperfine reaction for the system, the radicals created in the triplet states can be transformed into singlets and react. When the separation is greater than the hyperfine reaction, radicals created in T_{-1} and T_{+1} triplet states cannot interconvert and hence reaction cannot take place. However, an alternating magnetic field of frequency υ can excite electron transitions between levels, allowing transition to the singlet states even in high magnetic fields. The figure also shows the expected effect of increased static magnetic field on a chemical reaction (adapted from ref 156).

notably in neutrophils that generate reactive oxygen species, including the superoxide radical, from plasma membrane NADPH oxidase as a defense against bacteria. Superoxide can be further converted by superoxide dismutase to peroxide or by myeloperoxidase to hypochlorite. The generation of these reactive oxygen species can be monitored in cells in real time, using fluorescent dyes for oxidants. In such experiments, Roy et al. (54) have reported effects of 0.1 mT fields at 60 Hz on the phorbol ester-induced respiratory burst in primed rat neutrophil preparations. In all sets of experiments (five total), cells in magnetic fields showed up to 10% greater fluorescence than unexposed cells, although the effect was consistently smaller than the standard deviation of the measurements (standard deviations were as much as ±20%); there was also considerable variation in fluorescence between experiments, probably arising from differences in cell preparations on different days. Other groups have examined the effect of magnetic fields on nitric oxide production by murine macrophages (55) and observed no effect of exposure for 14 h to static fields of 1, 10, 50, and 100 mT or 1 Hz sinusoidal fields of 1.6 mT on nitrite levels.

Currently available data therefore do not provide any consistent evidence that ELF-EMFs affect cellular systems via free radical mechanisms. However, the data reviewed above indicate that complex effects can occur in defined chemical systems exposed to EMFs.

A free radical basis for magnetic field effects would have some important implications for investigations and epidemiological studies. The processes affected occur very rapidly, and so at the level of simple effects are independent of frequency; in many cases, the geomagnetic field exposure would far outweigh the alternating field. However, as described above, more complex effects can occur in vitro with specific combinations of static and alternating magnetic fields, and these combinations vary with the free radical species involved. These factors, together with the probable magnitude of any ELF-EMF-induced biological effect that may occur, suggest that it will be difficult to define quantitatively the required magnetic field variables.

EMFs as tumor promoters: modulation of function in signaling pathways

The foregoing discussion indicates that despite a possible mechanism whereby EMFs affect free radical formation, there is no firm evidence that EMFs cause damage to DNA; many experiments show no effect of EMF on DNA damage. Hence, the more plausible route by which EMFs might contribute to carcinogenesis is by acting as a promoter, increasing the probability that previously initiated cells will proliferate and progress to neoplasticity. This hypothesis has been tested in well-established animal model systems for tumor promotion, but with equivocal results (Table 3). Rannug and co-workers (56) used two different chemically induced tumor systems in rats and mice to examine the long-term effects of field exposure on tumor development. Mice were treated with subcarcinogenic amounts of the tumor initiator DMBA and exposed to 50 Hz sinusoidal fields of 50 or 500 μT for 20 h/day for 2 years. EMFs had no effect on any aspect of carcinogenesis when the fields were applied continuously, but some effects were seen if the fields were applied as intermittent signals

(15 s on/15 s off, again for 20 h/day, 103 wk; ref 57). Mice exposed to intermittent signals showed an increased skin tumor incidence of around twofold over nonexposed animals, and this was further increased in mice exposed at the higher field strength. However, neither increase was statistically significant. No measurable effect on endothelial hyperplasia or tumor load was seen. As a comparison, TPA, a phorbol ester and strong promoting agent, increased tumor incidence by almost 20-fold (from 5% in DMBA treated mice to 98%) and also had extensive effects on endothelial hyperplasia, time of appearance of first tumor, and tumor load per mouse.

In later studies in which Stuchly and co-workers (58, 59) used the mouse skin model, mice were treated with a single dose of DMBA and subsequent weekly treatments of the tumor promoter TPA. Half of these mice were also exposed to 2 mT fields at 60 Hz (6 h/day, 5 day/wk for 23 wk). The only aspect of carcinogenesis that appeared to be field dependent was the initial rate of tumor development, although the differences observed were not statistically significant at all time points. No effect of EMF exposure was seen at higher doses of TPA. Other known promoters with TPA—for example, prostaglandin—affected the rate and incidence of tumor development and the tumor load in individual mice. Considering the variability of the data (statistically significant differences at some time points only) and the small, rather anomalous effects, it is reasonable to conclude that no robust copromoting effect of EMFs was demonstrated. Magnetic fields (30 mT) at 50 Hz increased the number of mammary tumors induced by DMBA in rats by approximately 30% in some experiments reported by Mevissen et al. (60), although identical experiments reported in the same paper showed a decrease of similar magnitude in EMF-exposed rats relative to controls; the authors acknowledge that the small sample size and large variability limited the conclusions that could be drawn. These factors, coupled with the size and nature of any effects observed, apply equally well to all of the studies discussed here, and we conclude from the whole-animal studies that no unequivocal effects of EMFs on tumor promotion have been demonstrated.

IN VITRO CELLULAR STUDIES

In principle, intervention in proliferation control pathways in a manner similar to that of tumor promoters could be mediated by exposure to ELF-EMFs. Alternatively, EMFs may directly or indirectly modulate the function of proto-oncogene products, even though no mechanism is experimentally established whereby such effects could be manifested. These possibilities have prompted many experimental attempts to demonstrate EMF-induced responses in vitro (Table 3); major examples of these are summarized below.

Cell proliferation

Mitogen-stimulated lymphocyte growth

Numerous studies have determined the effects of EMF exposure on cell proliferation by counting the number of cells, estimating cell cycle phase progression, or measuring incorporation of radioactively labeled thymidine into DNA. EMF exposure (50 Hz sawtooth signals of 2.5 mT peak field for at least 6 h) increased thymidine incorporation by 20% in primary human lymphocytes stimulated with phytohemoglutanin (PHA), a T cell mitogen; it caused cells to enter the cell cycle and proliferate, but had no effect on unstimulated cells (61). Larger effects were seen with lymphocytes prepared from older donors (DNA synthesis increased by at least 60%), and the most dramatic effects were seen in B cells from patients with chronic lymphocytic leukemia, for which the mitotic index increased by threefold and thymidine incorporation by 100%. Similar but smaller effects (8% increase in cell cycle progression, assessed by proportion of cells undergoing mitosis) were seen by Rosenthal and Obe (43) under similar exposure conditions (50 Hz sinusoidal signal at 5 mT root mean square, or rms) although other independent laboratories (62–65) have reported inhibition of lymphocyte proliferation by EMF exposure [3 Hz square wave signal of 6 mT peak field, Conti et al. (62, 63); 3 Hz sawtooth signal of 4.5 mT, Mooney et al. (64)]. Interchange of exposure apparatus between laboratories suggests that the effect (inhibition or stimulation of proliferation) is dependent on the exposure apparatus and signal used (38). These results are therefore suggestive of small comitogenic effects of EMF on stimulated primary cell cultures, strengthening the case for EMFs as tumor promoters, even though there are major discrepancies concerning the nature of effects (stimulatory or inhibitory). Cadossi (61) further stresses the importance of the cells' origin, pointing out that samples from aged or diseased donors or suboptimally stimulated cells are more responsive to EMFs than other cells. Clearly these results do not yet constitute a well-defined and reproducible magnetic field effect. Conti et al. (62) have also measured the effect of 3 Hz signals on calcium fluxes and report that EMF exposure abolishes increased $^{45}Ca^{2+}$ uptake by human lymphocytes stimulated with PHA or TPA. For PHA stimulation, the effects reported are small (PHA stimulation of $^{45}Ca^{2+}$ uptake is at most 50% above unstimulated cells), and in only one of three experiments did the effect appear significant. The effect of EMF on TPA stimulation of calcium uptake was more consistent. These

TABLE 4. *Attempts to directly reproduce ELF-EMF biological effects*[a]

Original work	Experimental system	Replication studies	Result of replication
Nordenson et al., 1994 (45)	Chromosome defects in human amniotic cells	Galt et al., 1995 (46)	Failure
Greene et al., 1991 (93)	$[^3H]$uridine incorporation into HL60 cells	Azadniv and Miller, 1992 (94)	Failure
Goodman and Shirley-Henderson, 1991 (98)	*MYC* and β-actin transcription in HL60 cells	Lacy-Hulbert et al., 1995 (106) Saffer and Thurston, 1995 (108)	Failure
Litovitz et al., 1991, 1994 (90, 91)	ODC activity in mouse L929 cells	Azadniv et al., 1995 (92)	Failure
Smith et al., 1987 (74)	Diatom motility	Parkinson and Sulik, 1992 (140)	Failure
		Prasad et al., 1994 (161)	Failure
		Saalman et al., 1991 (47)	Failure
		Davies et al., 1993, 1994 (162, 163)	Partial
		Reese et al., 1991 (164)	Partial
Shuvalova et al., 1991 (128)	Calmodulin dependent myosin	Hendee et al., 1996 (127)	Failure
Markov et al., 1993 (129)	phosphorylation	Coulton et al., 1997 (130)	Failure
Rozek et al., 1987 (77)	$^{45}Ca^{2+}$ uptake by human	Prasad et al., 1991 (79)	Failure
Liboff et al., 1987 (78)	lymphocytes	Coulton and Barker, 1993 (80)	
Lindstrom et al., 1993 (81)	Ca^{2+} oscillations in Jurkat T cells	Walleczek et al., 1994 (83)	Failure

[a] Replication studies are confined to those specifically designed to reproduce a previously reported EMF-induced response.

results may be compared with other calcium flux studies, reviewed below.

Cain et al. (66) report a copromotional effect of 60 Hz 0.1 mT fields with TPA on cocultures of fibroblasts and transformed daughter cells. A twofold increase in the number of foci, area of foci per dish, and number of cells per focus was seen.

The effects of a variety of 50 Hz magnetic fields (vertical fields of 6 μT to 2 mT, or Ca^{2+} ion cyclotron resonance conditions, for as many as 21 days) on growth characteristics of the multipotential hemopoietic cell line, FDCP mix (A4), have been studied by Reipert et al. (67, 68; B. M. Reipert, D. Allan, and T. M. Dexter, unpublished results), who report no effect on growth rate, cell cycle state, clonogenic efficiency, or apoptosis.

Effects of melatonin and tamoxifen on the growth of breast tumor cell lines

Harland and Liburdy (69) have reported that continuous exposure to 60 Hz, 1.2 μT EMFs can block the cytostatic action of melatonin and tamoxifen on the human breast cancer cell line MCF-7. The effects were not seen at lower field strengths (0.2, 0.6 μT) and no effect of field exposure without melatonin/tamoxifen treatment was seen. These experiments used a well-defined cell system with a reliable assay (cell number) and simple exposure conditions.

Neurite outgrowth

Blackman et al. (70) have reported that EMF exposure of rat pheochromoctytoma cells (PC12 cells) treated with nerve growth factor can either increase or decrease neurite outgrowth, depending on the subset of PC12 cells used. In view of this variability, it may be concluded that this system is unsuitable for studies of EMF effects.

Intracellular responses

Calcium flux and intracellular free calcium ($[Ca^{2+}]_i$) measurements

Early studies used $^{45}Ca^{2+}$ to measure fluxes, notably in brain tissue (71, 72). The effects detected were small (generally 10% over control samples; maximal effects of 30%) and varied significantly between laboratories. For example, Blackman et al. (73) observe that "although the results of exposure of isolated chicken brains to ELF fields as published by Bawin and colleagues and by us agreed both in the frequency dependence and generally in the intensity dependence of the results, there was one major difference. Blackman et al. (72) reported an enhancement in calcium ion efflux whereas Bawin and Adey (71) reported a reduction in efflux."

Transmembrane Ca^{2+} fluxes control the mobility of *Amphora coffeaeformis* (diatoms), and it has been reported that diatom mobility can be stimulated by specific combinations of d.c. and low-frequency EMF exposure in a manner consistent with the cyclotron resonance theory of EMF/Ca^{2+} interactions (74). Even though in principle these experiments are simple to perform, several attempts at replication have thus far failed outright or did not demonstrate consistent effects (**Table 4**). Furthermore, no effect of

magnetic fields satisfying the resonance condition for calcium on Ca^{2+} transport was detected in the membranes of cultured cells (RINm5F-cloned insulin-secreting β cells; ref 75) or in cytosolic calcium concentrations in a number of cultured cell lines (3T3 fibroblasts, L929, V-79, and rat osteosarcoma cell lines; ref 76). Effects of magnetic fields satisfying the resonance conditions for calcium on Ca^{2+} transport have been reported for primary human lymphocytes (77, 78), but these have also proved irreproducible (79, 80; Table 4). Other effects of EMF on calcium transport in lymphocytes appear more robust and are discussed below.

Increases in $[Ca^{2+}]_i$ are essential for the initiation of growth in newly fertilized eggs and, as indicated above, the frequent association of increases in $[Ca^{2+}]_i$ with stimulated proliferation of somatic mammalian cells implicates calcium-regulated proteins as crucial components of the growth signaling pathway. It has been postulated that EMFs may modulate cellular calcium regulatory mechanisms by, for example, affecting the function of transmembrane proteins or the affinity of calcium binding proteins such as calmodulin. Lindström et al. (81) have reported effects of 50 Hz, 100 μT magnetic field exposure on spontaneous $[Ca^{2+}]_i$ oscillations in Jurkat cells. Using the fluorescent indicator fura 2, $[Ca^{2+}]_i$ was measured every 2 s in individual cells. Calcium oscillations began 15–20 s after applying magnetic fields (0.1 mT, 50 Hz, generating electric fields of <50 μV/m), with $[Ca^{2+}]_i$ rising from a baseline of 20–100 nM to 200–400 nM, similar to the effect of CD3 receptor complex activation. The largest response was seen with fields of 150 μT (81). EMF-induced $[Ca^{2+}]_i$ oscillations were dependent on the presence of an intact CD45 intracellular phosphatase domain, which is also necessary for early events in T cell receptor-mediated signaling and coupling to inositol phosphate signaling pathways. Lindström et al. (82) have subsequently shown that inositol 1,4,5-triphosphate (IP_3) levels are also increased in Jurkat cells after short exposures to 0.1 mT, 50 Hz fields. IP_3 increased from 2 to 30–40 pmol/10^6 cells within a few minutes, similar to the response to anti-CD3 antibody, although with a different time course (EMF response maximal at 2 min: 6 min for anti-CD3); EMF response occurred in the presence of intracellular calcium chelators. In the unexposed control experiments, however, levels rose by eightfold to 17 pmol/10^6 cells. Thus, it is not clear that these experiments are rigorously controlled. The EMF response was not seen in all cells: although 85% of the Jurkat cells responded, only 10% of peripheral blood lymphocytes did so. An independent group has been unable to repeat these $[Ca^{2+}]_i$ observations, although the data have been published only as an abstract (83). Walleczek et al. (86, 87) report that only 50% of Jurkat cells showed stable $[Ca^{2+}]_i$ levels before exposure; of those 'stable' cells, 14% showed deviations from basal $[Ca^{2+}]_i$ during EMF exposure, and a similar proportion showed deviation during a sham exposure. Hence, the experimental system appears too unstable to quantify small $[Ca^{2+}]_i$ changes.

Walleczek (84) and others have reported effects of short exposures (30 min–1 h) to higher EMFs (2–22 mT) on $^{45}Ca^{2+}$ uptake by lymphocytes. Enhanced $^{45}Ca^{2+}$ influx was detected only in cells treated with concanavalin A (Con A), was dependent on the magnitude of both the applied magnetic field and the induced electric field, and resulted from increased calcium influx through plasma membrane channels rather than release from intracellular stores (85). The response also depended on the sensitivity of each primary cell culture to the mitogen. Hence, 60 min exposure to 60 Hz sinusoidal fields of 6.5 mT enhanced the response of cells to Con A, increasing Ca^{2+} uptake over control cells by 3.9-fold, compared to only 2.5-fold in cells treated with Con A alone. When cells were exposed to a 3 Hz rectangular wave signal of 6.5 mT peak field with Con A treatment, EMF exposure inhibited Con A-induced Ca influx in Con A-responsive primary cultures, but introduced a 'Con A'-like Ca influx in cultures that did not respond to Con A alone. These effects are similar to those of Conti et al. (62), described above. As the fields used are much higher and in many cases of a different waveform than those of central concern to this review, these results will not be discussed further; nevertheless, the results seen with 3 Hz fields are to some degree consistent between groups (86), are consistent with an effect at the cell plasma membrane, and many reports conclude that the magnitude and type of EMF effect depend on the degree of cell stimulation by other agents.

In more recent work, Walleczek et al. (87) reported a 5% increase in calcium influx (measured by single cell fluorescence spectroscopy and Mn^{2+} influx) into human Jurkat T cells after 2 min exposure to 60 Hz, 2 mT EMFs. Again, the effect was critically dependent on the biological state of the cells, characterized by the initial (preexposure) Ca^{2+} influx rate.

Barbier et al. (88) have reported sustained increases in $[Ca^{2+}]_i$ in rat pituitary cells at either 25°C or 37°C after exposure to 50 Hz sinusoidal fields of 50 μT (30 min or 3 h). $[Ca^{2+}]_i$ rose from ~180 nM (range 80 to 314 nM) to ~350 nM (range 109 nM to 4267 nM). No effects were seen at 2, 10, or 250 μT. $[Ca^{2+}]_i$ was assayed by indo-1 fluorescence; after exposure, individual cells were identified by double label immunochemistry. Only the lactotrophs (29% of pituitary cells) were responsive. In the absence of extracellular Ca^{2+}, the $[Ca^{2+}]_i$ increase was (on the basis of one experiment shown) greatly reduced but still significant (a two to threefold rise). The major component was thus ascribed to Ca^{2+} influx via voltage-dependent, dihydropyridine-sensitive channels

(inhibited by PN 200–110). Double-wound coils were used, and in sham-exposed controls there was no change in $[Ca^{2+}]_i$. $[Ca^{2+}]_i$ was measured before and after field exposure in these experiments, although continuous monitoring would have been preferable and feasible.

Ornithine decarboxylase

ODC activity is significantly stimulated (to high levels from almost undetectable levels in control cells) by treatment with phorbol ester and is elevated in all rapidly growing cells, including transformed cells and cells isolated from tumors. Stimulation of ODC activity has been detected in cultured human lymphoma cells (fivefold over controls) and mouse myeloma cells (1.7-fold over controls) after 1 h exposure to 1 Vm^{-1} electric fields at 60 Hz (89). Stimulation was transient, the maximum occurring 1–2 h after termination of exposure. However, no effect of 50 Hz, 30 μT fields on ODC activity was detected in human amniotic cells (46). More recently, Litovitz et al. (90, 91) reported a large increase (twofold and greater) in ODC activity in L929 cells exposed to 60 Hz magnetic fields of 10 μT for 4 h. Azadniv and co-workers (92) attempted to repeat these experiments and found no effect of field exposure on ODC activity, although the cells used could be shown to respond to the phorbol ester TPA, yielding a large increase in ODC activity. The researchers showed some variation in the measurement of ODC activity and, by using statistical methods, demonstrated that this was due to interexperimental variation and did not correlate with EMF exposure.

Uridine uptake and incorporation

Numerous experiments have indicated that EMFs stimulate both the uptake of uridine by cells and its incorporation into RNA. Greene et al. (93) reported a 50–60% increase in uridine incorporation in human leukemic (HL60) cells after exposure to 1 mT fields at 60 Hz for 1 h. However, Azadniv and Miller (94) repeated these experiments and found no effect of EMFs on uridine incorporation. Phillips et al. (95) also reported two- to threefold increases in uridine incorporation after treatment with pulsed fields of 3.5 mT peak strength, which were maximal after 2 h and remained elevated for 22 h. Goodman, Henderson and co-workers have reported the effects of a variety of fields (60 and 72 Hz sinusoidal fields and several pulsed waveforms) on the incorporation of tritiated uridine into nascent RNA on polytene chromosomes of *Dipteran* insect salivary glands. Fractionation of pulse-labeled total cellular RNA suggested that incorporation was into mRNA. These experiments could be taken to suggest that general RNA synthesis can be stimulated by EMF exposure, but the exquisite sensitivity of uridine transport to cell perturbation (96) indicates that the experiments require further verification.

Early response genes: FOS and JUN

Nuclear run-on assays used to measure gene transcription in T lymphoblastoid cells exposed to 100 μT 60 Hz fields for 15–120 min indicated that *FOS, JUN*, and *MYC* transcription may be modulated by EMF exposure (95). *FOS* transcription was induced maximally (2.5-fold) after 30 min of exposure and returned to control levels by 60 min. *MYC* was also increased, reaching a plateau of 1.5- to 2-fold control levels after 30 min. *JUN* transcription was affected in a cell density-dependent manner, being decreased by 70% after 30 min at 5×10^5 cells/ml, but increased by 2.2-fold after 60 min at 1×10^6 cells/ml. In both cases, expression returned to control levels after 24 h. PKC expression was also altered by EMF exposure, being maximally stimulated after 15 to 30 min. These data are in many ways consistent with the hypothesis that EMFs activate a mitogenic signaling pathway. *FOS* induction was rapid and transient, with approximately the same time course that follows mitogenic stimulation. *MYC* expression also followed a time course similar to that seen in mitogenically stimulated cells, though the magnitude of the effect (2- to 2.5-fold over control levels) was small compared with that seen in mitogenic activation. However, in contrast to conventional practice, in this study cells were exposed to the putative mitogen (EMFs) while in the logarithmic phase of growth in the presence of 10% serum. This will give relatively high levels of transcription against which any EMF-induced effects must be detected, although it is, of course, arguable that cells that are already partially activated may be ideally primed to respond to a further weak stimulus.

The effect of EMFs on early response gene expression (*Fos, Jun*, and *JunB*) in a murine hematopoietic progenitor cell line [FDCP mix(A4)] transfected with a *Bcl-2* expression vector has been studied. BCL-2 synthesis maintains the cells as a quiescent but viable line in the absence of interleukin 3 (IL-3). Cells were stimulated by addition of IL-3 at a suboptimal concentration for the transcription of early response genes expression, and cells were exposed to 6 μT 50 Hz sinusoidal fields for 20 min to determine whether there were any additive or synergistic responses. No significant effects on *Fos, Jun*, or *JunB* transcription were detected (67).

MYC

The most extensive series of investigations of EMF effects on gene expression in human cells has been the joint studies of Goodman and Henderson (97–105). They have reported increases in *MYC*, β-actin,

and histone H2B transcript levels of up to threefold when measured by dot blot analysis in HL60 cells after exposure for 20 min to any one of five different EMF signals, including 1.1 and 1.5 mT sinusoidal magnetic fields at 60 and 72 Hz, respectively, and three pulsed fields of the form used clinically to treat non-union bone fractures (97). Exposure for 20 min to a 45 Hz sinusoidal magnetic field with an rms value of 0.8 mT increased *MYC* and histone H2B mRNA levels by sevenfold; a 5.7 μT, 60 Hz field caused a fourfold increase in mRNA levels, with transcription of β-actin, hsp 70, *SRC*, and α-tubulin being stimulated to the same extent as *MYC* and histone H2B (98, 99). At other field strengths (0.57, 57, and 570 μT), mRNA levels were increased by two- to threefold. Other waveforms at frequencies of 15 to 150 Hz stimulated gene expression by approximately twofold, although it is not possible from these experiments to obtain a clear picture of the relationship between field strength or frequency and stimulation of gene expression, since both signal parameters were changed between experiments. Increased transcription was detected after a 4-min field exposure, being maximal at 20 min and returning to control levels after 4 h exposure (100).

Closely similar patterns of gene expression stimulated by EMFs in six different types of eukaryotic cells have also been reported by Goodman and Henderson [<1 h exposure, 60 Hz, 5.7 μT–57 μT (98, 101–104), 1.1 mT (97, 105)]. The responses included activated gene expression in yeast (104), mouse myeloma cells (103), and three types of human cells (98, 102, 103), as well as changes in protein synthesis in *Sciara* (101, 105), with no reports of cell types that were insensitive to ELF-EMFs.

Several conclusions were drawn by Goodman and Henderson on the basis of these results. *1*) A subset of genes including *MYC*, β-actin, histone H2B, hsp 70, *SRC*, and α-tubulin were EMF responsive, showing closely similar time courses and quantitative increases in transcription. Only one gene—β2-microglobulin—was nonresponsive to EMFs. *2*) Induction was rapid (detectable within 4 min) and transient; transcript levels were maximal after 20 min exposure and returned to control levels within 4 h of first exposure. *3*) Responses were frequency and field strength dependent, the largest response consistently reported as occurring after 20 min exposure to a 5.7 μT sinusoidal magnetic field at 60 Hz. *4*) Quantitatively similar patterns of EMF-stimulated gene expression occurred in at least six different types of eukaryotic cell.

In view of the well-established individuality of genes in terms of the temporal and quantitative nature of their responses to cell activating signals, it seems improbable that so many genes in such a diverse set of cell types would respond almost identically to stimulus by EMFs. Nevertheless, the central role played by MYC in controlling cell proliferation and apoptosis and the fact that *MYC* expression is anomalously regulated in the majority of human cancers imbued these findings with great significance; rigorous attempts have been made in two independent laboratories to reproduce the findings of Goodman and Henderson (106–109).

Each of these independent studies comprised two main components: *1*) very close reproduction of the experimental conditions used by Goodman and Henderson, and *2*) the design of systems and protocols to achieve maximum sensitivity in the identification of any EMF-induced effect on the transcription of *MYC* and other genes, together with maximum reproducibility of data.

One of the replicative studies (107) used exposure apparatus and signal generators built to the same specifications as those of Goodman and Henderson and closely followed their protocols for cell handling, field exposure, RNA preparation, and in some experiments, the exact methods for quantification of RNA transcripts. The other study (109) included experiments performed in Henderson's laboratory while using their exposure apparatus and cells.

Further experiments in both replicative studies refined several aspects of the original work of Goodman and Henderson by using more controlled experimental conditions and accurate methods to quantify gene transcripts, which were accurate to within 10%.

Neither replicative study (106–109) detected any effect of ELF-EMF exposure on gene expression for exposure periods of up to 1 h to 60 Hz fields of 0.1 to 100 μT. These findings directly contradict the multiplicity of reports generated by Goodman and Henderson (98–100). The following points have been raised to account for the irreproducibility of the findings by Goodman and Henderson, but none offer escape from the dichotomy. The issues have been analyzed previously (106–109) and are briefly summarized here.

1. HL60 cell phenotype

EMF responsiveness may be critically dependent on the phenotype of the subculture of cells used. The HL60 cells used in both replication studies were obtained from established cell culture collections. Both showed typical patterns of HL60 cell-surface antigen expression and both responded normally to the stimulation of differentiation by TPA. Furthermore, experiments by Saffer (108, 109) in New York, and subsequently in his own laboratories with cells obtained from Henderson (109), showed no EMF response. However, Goodman and Henderson (97–105) have reported responses to EMFs in six different types of eukaryotic cells as well as in HL60 cells, with no reports of cells that are insensitive to EMFs. It is therefore highly im-

probable that both replication studies selected unresponsive HL60 cell sublines, whereas Goodman and Henderson consistently selected responsive cells from a wide variety of sources for the diverse eukaryotic systems studied.

2. Field exposure

The electric fields and currents induced in cell suspensions by a given magnetic field vary considerably with the orientation and type of exposure system; hence replication studies may not reproduce critical parameters of the original EMF signal. This was not relevant in these two replication attempts. Saffer conducted experiments in Henderson's laboratory using their equipment, and experiments were performed by Lacy-Hulbert et al. (106, 107), who used exposure coils made to Goodman and Henderson's (98) specifications. Furthermore, Goodman and Henderson have reported that a wide variety of waveforms, field strengths, and exposure systems caused large changes in transcription.

3. Geomagnetic fields

Local magnetic fields may affect EMF responsiveness. In the first experiments reported by Goodman and Henderson, no magnetic shielding was used and background geomagnetic fields at the sample position were reported as 46 μT (Goodman laboratory; ref 97) and 8.2 μT (Henderson laboratory; ref 98). Later experiments used mu-metal shielding, and background geomagnetic fields were <0.1 μT (110, 111). The response of cells to ELF-EMFs therefore was not dependent on a large geomagnetic field component. In both replication studies, background magnetic fields were reduced by mu-metal shielding to <0.1 μT.

4. Magnetite

The presence of precipitates of the ferromagnetic mineral magnetite (Fe_3O_4) may contribute to in vitro EMF effects (112). It is possible that biological responses to EMFs are mediated by ferromagnetic minerals; magnetite crystals have been implicated in the mechanism by which migratory birds and bees detect the Earth's magnetic field (113). Hence, the absence of magnetite in any of the studies might prevent an EMF response. However, magnetite is omnipresent in laboratory culture reagents and plasticware, and no precautions against magnetite contamination were taken in any of the studies; therefore, whatever role it may play in EMF-induced effects, magnetite cannot explain the failure to reproduce the Goodman and Henderson data.

5. RNA extraction procedure

Selective extraction may arise from the use of different procedures. It has been suggested that detection of EMF-induced effects may depend on the extraction methods used (114). One replicative study tested both guanidine extraction (extracting total cellular RNA) and the SDS/phenol extraction method used by Goodman and Henderson (extracting cytoplasmic RNA), without significant difference. The other study used the cytoplasmic RNA preparation protocol described by Goodman and Henderson.

6. Pretreatment of cells

The immediate prehistory of the cells is critical. The Goodman and Henderson protocol assayed gene expression only 1 h after cell centrifugation and resuspension in fresh medium. However, as it is established that centrifugation and associated manipulations greatly perturb cell metabolism and transcription, the most probable effect of such a method would be to increase the variability of the results. The replicative studies repeated the 1 h preincubation protocol, but also used a 12 h preincubation in a controlled field environment, together with cell harvesting procedures that eliminated exposure to uncontrolled, ambient fields.

Consideration of these points indicates that it is improbable that the failure of two independent laboratories to reproduce the Goodman and Henderson findings arises from inadequacies in the replicative procedures. We conclude that the most convincing explanation for the conflicting data on activation of gene expression is that there were systematic errors in the protocols of Goodman and Henderson for cell handling and field exposure, methods of transcript quantification, and/or data analysis. This conclusion is entirely consistent with their data: Goodman and Henderson observed that all EMF-responsive genes in all organisms they studied were stimulated with similar time courses and to a similar extent (two- to fourfold increased levels, with maximal effects between 20 min and 1 h exposure; these include the early response gene *FOS*, *MYC*, the heat shock gene hsp70, β-actin, histone H2B, α-tubulin, and *SRC* in HL60 cells, SV-40 large T antigen, and the yeast genes URA3 and SSA1). This is not consistent with any known mitogenic stimulation or stress response, but is entirely consistent with effects arising from systematic errors.

The following possible sources of systematic error can be identified. *1)* In some experiments, field-exposed and control unexposed trials were performed either sequentially or by using separate incubators (97). *2)* Most transcriptional data were obtained when using dot blot assays, without internal controls being applied to normalize RNA loading, nor were appropriate internal controls used to quantify transcript levels in reverse transcription and PCR experiments. *3)* Data were presented as ratios of expression in exposed samples to that in control samples (E/C

ratios) in order to give a measure of stimulation by EMF exposure that allowed data from different experiments to be compared. Mean E/C ratios were calculated from these ratios, a process that introduces statistical bias to the final results, generating 'EMF effects' where there may be none. Bias arises because distribution of the ratios of numbers taken from a normal distribution is not symmetrical about the mean and ratios should be log transformed before calculation of the mean. The bias toward positive effects is greatest when the variation in the measured parameter is large, as in the Goodman and Henderson experiments.

Several aspects of the protocols used suggest there may be systematic errors in the experiments; this conclusion is further strengthened by consideration of two striking features of the Goodman and Henderson data. *1*) The gene expression changes reported are not consistent, and inconsistencies within a single paper pass unremarked. For example, in HL60 cells exposed to 57 μT fields at 60 Hz for 20 min, histone H2B expression increased by 8 (±2) -fold (Fig. 1 from ref 115) and by 2.3 (±0.3) -fold (Fig. 2 from ref 115). A similarly marked trend of decreasing response is evident within a single set of experiments: the effect of EMF exposure on β-actin levels decreasing from a 1.8-fold stimulation to 1.05-fold over 14 consecutive experiments (Fig. 2 from ref 116). *2*) A long-term trend is clearly observable when the absolute EMF-induced increases in *MYC* expression reported by Goodman and Henderson are plotted as a function of publication year (**Fig. 5**). The magnitude of the maximum *MYC* response declined from fourfold in 1991 to 30–50% in 1995. In the only experiments in which internal controls were used (normalization to the housekeeping gene β2-microglobulin), and hence in which discussion of small changes in transcriptional data may be meaningful, the reported increases in gene expression (*MYC* and GAPDH) were very small (approx. 30%) and probably did not exceed the limits of overall experimental error. This trend may reflect progressive improvement in experimental design, reducing the experimental noise and removing sources of systematic error.

These points, coupled with the lack of effect observed by Saffer and Thurston (108, 109) and Lacy-Hulbert et al. (106, 107) in experiments where internal controls were used for systematic error and experimental noise, strongly suggest that the EMF effects reported by Goodman and Henderson were due solely to systematic error introduced by experimental protocols and quantification methods. The accuracy of the replicative studies (±10%) therefore sets an upper limit to any EMF transcriptional effects that the field exposure conditions can induce in HL60 cells (106–109). These studies do not, of course, eliminate the possibility that small effects on gene expression in HL60 cells within this limit may be induced by ELF-EMFs. These would be undetectable by current quantification techniques.

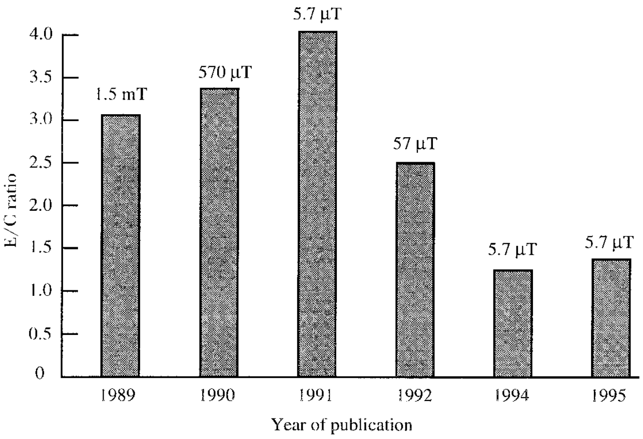

Figure 5. Stimulation of *MYC* expression in HL60 cells exposed to 60 Hz EMFs of the field strengths indicated for 20 min, reported by Goodman and Henderson. Gene expression is represented as exposed:control ratios taken from the original papers (97–100, 165, 166).

SRC family kinases (LYN)

In contrast to the substantial number of reports of EMF effects on ionic signals, transport mechanisms, and transcription, there has been only one report of the activation of a proto-oncogene-encoded kinase. In this study, LYN kinase activity in B-lineage lymphoid cells was stimulated by exposure to 100 μT, 60 Hz electromagnetic fields (117). The effect was rapid (<2 min) and large. Double-wound coils were used to show that no enhanced kinase activity was detectable when the magnetic field was neutralized. Furthermore, EMF-stimulated LYN activity activated SYK, previously shown to be a target of the SRC family kinase; in $LYN^{-/-}$ cells, SYK activation did not occur upon exposure to fields that activated LYN. In the established signaling pathway for LYN after B cell receptor activation, SYK phosphorylates SHC, promoting its association with GRB2 (see Fig. 2).

This is a potentially important result, not merely because the experiments appear to have been rigorous but because the lymphoid cell line used may provide an appropriate model for a prelymphoma. Furthermore, LYN is known to be activated in HL60 cells by ionizing radiation (118) and it is possible that this protein is particularly sensitive to mild exogenous stress. Some SRC family kinases (SRC, FYN, and YES) have recently been shown to associate with the activated PDGF receptor and to then stimulate *MYC* expression. It remains to be seen whether ELF-EMF-activated LYN can modulate transcription of specific genes.

Cell-free systems

A few studies have described effects of EMF on cell-free systems. These results are often not directly transferable to whole-cell assays and generally do not offer an obvious mechanism for other reported EMF effects, but such studies do have a role in furthering understanding of EMF effects and they offer the opportunity to define the conditions under which effects may occur.

Ion cyclotron resonance and calcium–calmodulin interactions

Some studies (74, 78, 119–123) have described biological responses to magnetic fields modulated at the cyclotron resonance frequency of the unhydrated calcium ion. Theories attempting to explain these effects (78, 124–126) predict a maximal effect of ELF-EMFs at a frequency dependent on the mass of the ion and its charge: $f_c = qB_o/2\pi pm$, where B_o = d.c. magnetic field strength, q = ionic charge, f_c = cyclotron frequency, m = mass of ion. The critical involvement of calcium in many cellular processes implies that an EMF cyclotron effect on this ion could significantly modulate cell behavior. However, a number of recent, careful studies have failed to detect any such effects on either calcium or potassium. One of the most sensitive measures of a cyclotron effect on calcium is to determine whether any change in affinity for calcium binding proteins occurs at the resonance frequency. A detailed study of calcium/calmodulin interaction in which binding of a fluorescent peptide to the complex was assayed detected no effect of a 20.9 μT sinusoidal field over a frequency range centered at 16 Hz, the cyclotron frequency of the unhydrated Ca^{2+} ion (127). The same study also detected no effect of combined d.c. and a.c. magnetic fields on myosin light chain kinase activity over the same frequency range. These findings clash with the results of both Lednev (128) and Markov and colleagues (129), who reported field-induced changes in phosphorylation of myosin light chain in two different calmodulin-dependent cell-free systems, assayed either by 2-dimensional PAGE or by incorporation of radioactive precursors. However, a further direct attempt to replicate the findings of Markov et al. (130) has also proved negative. We conclude therefore that there is no reliable evidence that supports the ion cyclotron resonance model for calcium-mediated magnetic field effects.

Black lipid membranes

The application of the cyclotron resonance theory to explain the effect of calcium on diatom mobility in the experiments discussed in the section on 'Calcium flux and $[Ca^{2+}]_i$ measurements' implied that magnetic fields act directly on membrane-permeating ions. Black lipid membranes provide a simple and well-defined system for the study of such effects; in particular, the pores formed by the linear pentapeptide gramicidin A that are selective for monovalent ions are the most thoroughly understood of membrane channels. A number of studies have indicated that ELF-EMFs have no effect on the potassium conductance of gramicidin channels (131), the most extensive being those of Wang and Hladky (132), who showed that fields from 50 to 5000 μT at frequencies between 10 and 200 Hz, together with combinations of static and low-frequency fields satisfying the predicted cyclotron resonance condition, were without effect. These authors also found there was no effect of K^+ resonance fields on ATP-sensitive potassium channels in membranes from the GRI-GI insulin-secreting cell line (133).

Membrane ATPase

Effects of electric and/or magnetic field exposure on membrane ATPases have been reported by several groups (134). Membrane ATPases (e.g., F_0F_1-ATPase) use electrochemical gradients derived from glucose metabolism and the citric acid cycle in mitochondria or photosynthesis in chloroplasts. It appears that the enzymes can also be stimulated by externally applied electric fields; isolated submitochondrial particles or chloroplasts can be induced to synthesize ATP by exposure to short (μs-ms) electric pulses of several $kVcm^{-1}$. The effect requires functional ATP synthesis machinery, membrane integrity, and impermeability, but occurs independently of components of the mitochondrial electron transport chain or chloroplast photosystems. In mitochondria, the effect is observed with fields of over 10–15 $kVcm^{-1}$, generating transmembrane electric fields of 200–300 $kVcm^{-1}$ that are of the same order of magnitude as the fields generated in vivo for ATP synthesis. In the presence of suitable reducing agents (dithiothreitol), five to eight molecules of ATP were generated per enzyme molecule per pulse (100 μs decay time). It is intriguing that much lower fields of up to 60 Vcm^{-1} a.c. (10 Hz optimal frequency), when applied for longer periods of time (10 min to 1 h), can also stimulate ATP synthesis, generating a very low yield of only 10^{-3} molecules of ATP/molecule of enzyme per cycle of a.c., which results in an overall yield of more than 10 molecules ATP/molecule of enzyme over the prolonged exposure time.

Effects of EMF exposure on the activity of Na^+, K^+-ATPase, the enzyme principally responsible for establishing ion gradients across the cell membrane, have also been reported (135). Weak 100 Hz electric fields (maximal effects observed at 5 $mVcm^{-1}$ with a threshold for effects estimated by extrapolation of 5 μVcm^{-1}) inhibited enzyme activity when the enzyme

was operating under optimal conditions, but increased enzyme activity when enzyme activity was inhibited by ouabain or low temperature. The maximum magnitude of any effect was 10–20%, but the paper gives no estimate of the experimental errors involved in the measurements of enzyme activity. The magnitude and nature of the effect (stimulatory or inhibitory) varied considerably as many of experimental parameters were altered, including the ionic strength of the medium, the concentration of specific ions, and the size and frequency of the applied field.

Tsong (134) and others have proposed various resonance theories to explain the effects observed at low electric fields, and these models may provide a mechanistic framework in which to explain effects of electric fields on biological systems and the appearance of signal frequency or amplitude 'windows' of maximal response. However, as with many of the mechanisms proposed, in the absence of effects that are quantitatively significant when the probable experimental error is considered, these mechanisms and proposed effects remain speculative.

Cell-free transcription

Goodman et al. (136) have reported effects of 72 Hz magnetic fields from 70 μT to 1.1 mT on transcription and translation in a cell-free system derived from *Escherichia coli* and in intact *E. coli* (137). However, no replication attempts of these experiments have been reported.

CONCLUSIONS

The cumulative evidence summarized above indicates that epidemiological studies are unable to provide a clear correlation between exposure to ELF-EMFs and the development of cancers. In part this is because childhood leukemias, considered particularly important in this context, occur with low frequency in the general population. Such surveys are further confounded by the fact that no mechanism is known by which EMFs might contribute to tumorigenesis, and hence it is impossible to define relevant exposure parameters. The latter fact has also contributed to the failure of animal experiments to produce convincing evidence for mutagenic or tumor-promoting effects of EMFs; these studies suffer further from the difficulties associated with using a sufficiently large number of animals to acquire statistically significant data.

Because of these problems and perhaps also because of the relative ease with which they can be performed, a great many in vitro studies of intact cells and cell-free systems have been published. In vitro studies are more tractable than epidemiological or whole-animal studies in overcoming the problems of accumulation of statistically significant data and of defining dosimetry by applying a wide range of EMF parameters. However, it is evident that a high proportion of these are flawed either in design, execution, or analysis of data and should therefore be disregarded. Although in vitro EMF experiments are basically simple, usually involving assays that are routine in other contexts, the effects that have been reported are generally very small. This necessitates rigorous design of experiments comparing EMF-exposed with unexposed samples and a thorough consideration of the variability inherent in the assays used. The required levels of rigor usually have not been met. Independent reproducibility is crucial for the acceptance of positive effects, but thus far very few such replication attempts have been published; many that have were unable to repeat the initial findings (Table 4).

The detailed summary of in vitro observations given above invites the conclusion that no convincing evidence currently exists that ELF-EMFs damage DNA either by point mutations, gross chromosomal alterations, or micronuclei formation. Furthermore, most studies of the effects on cellular signaling pathways (e.g., (co-)mitogenic effects, activation of transcription, calcium fluxes, protein kinase activities) demonstrate no convincing responses. Nevertheless, a small number of positive effects remain that are difficult to reject and so merit further investigation. These include activation of *FOS* transcription (95) and LYN kinase (117) and stimulation of Ca^{2+} influx (87). Some of these effects are currently being pursued under the sponsorship of either the U.S. Rapid program or the EMF Biological Trust programs in the U.K.

The establishment of a robust, independently reproducible ELF-EMF effect is not contingent on an understanding of its mechanism. However, it is desirable that there is at least a plausible explanation for any consistent effect that is discovered. The most persuasive model is that of free radical generation by EMFs, an established phenomenon in chemical systems. However, although an EMF effect has been demonstrated on an isolated enzyme system at relatively high field strengths (50 mT; ref 53), no EMF-induced free radical effect has yet been demonstrated in a cellular biological system at environmental field strengths, and cells have powerful mechanism with which to quench free radicals. Nonetheless, this mechanism has the advantage of overcoming a problem faced by most other models, which is that the energy supplied by ELF-EMFs is minute in comparison with the energy of continuous, random thermal events within the cell.

Much interest has focused on models by which EMF-generated electric fields may modulate ion transport processes across membranes, including the surface compartment model (138), and they offer a

theoretical explanation for how oscillating currents and fields could cause transient and ion-selective modulation of fluxes. However, it should be emphasized that the most rigorous analysis of ELF-EMF effects on model ion channel systems (132) have detected no modulation of their behavior.

Ion cyclotron resonance models explain how the motion of a charged particle may be modulated by energy derived from a varying electric field and predict that at the resonance frequency the particle will receive energy continuously. This theory has attracted considerable publicity mainly as an explanation for the observations of rat behavior in 60 Hz fields (139) and of diatom mobility (74). However, the diatom observations have proved only partly reproducible (140) and the behavioral mechanisms have not been repeated. Many other negative studies using the predicted cyclotron resonance frequencies, together with a number of theoretical papers arguing against this theory, suggest that the relevance of cyclotron resonance theory to ELF-EMF-induced biological effects is questionable. Other theories also advanced include those of Lawrence and Adey (141), but as with cyclotron resonance, no convincing experimental data have yet been provided in their support.

An alternative proposal, that ELF-EMF effects may be mediated indirectly—for example, by charged aerosol particles accumulating on surfaces in response to electric fields—has already been mentioned. This phenomenon undoubtedly occurs at the levels of the fields in question, and recent data indicate that significant deposition of radon-derived isotopes can occur on domestic conductors. However, there is no evidence indicating that such deposition can occur within, say, the respiratory tract and lung epithelia.

The ubiquitous presence of the ferri-magnetic material magnetite Fe_3O_4 has also been suggested as a potential mediator of ELF-EMF effects (112). Magnetite particles within or adsorbed on the surface of cells acquire a mechanical energy from weak fields that greatly exceeds that of the thermal background within cells, and they may therefore interact with, for instance, ion channels to modulate their function. However, as with the other hypotheses summarized above, there is no direct evidence for such a mechanism.

The absence of an identified mechanism whereby ELF-EMFs can exert biological effects clearly constitutes a problem in that experimental protocols are devised and measurements are made on the basis of complete ignorance about the susceptibility of biological systems. The recurring theme of this review has been the overriding need to demonstrate a single, unequivocal ELF-EMF-induced response that will be consistently reproducible in independent laboratories. Only then will the normal process of scientific development of the field become possible. Until this is achieved, the topic of biological responses to ELFl-EMFs will continue to be regarded with great skepticism by the scientific community at large. FJ

REFERENCES

1. Bassett, C. A. (1993) Beneficial effects of electromagnetic fields. *J. Cell. Biochem.* **51**, 387–93
2. Swanson, J., and Renew, D. C. (1994) Power frequency fields and people. *Engin. Sci. Educ. J.* 71–80
3. Merchant, C. J., Renew, D. C., and Swanson, J. (1994) Exposure to power-frequency magnetic fields in the home. *J. Radiol. Protect.* **14**, 77–87
4. Wertheimer, N., and Leeper, E. (1986) Possible effects of electrical blankets and heated water beds on fetal development. *Bioelectromagnetics* **7**, 13–22
5. Jackson, J. D. (1992) Are the stray 60-Hz electromagnetic fields associated with the distribution and use of electric power a significant cause of cancer? *Proc. Natl. Acad. Sci. USA* **89**, 3508–3510
6. Wertheimer, N., and Leeper, E. (1979) Electrical wiring configurations and childhood cancer. *Am. J. Epidemiol.* **109**, 273–384
7. Henshaw, D. L., Ross, A. N., Fews, A. P., and Preece, A. W. (1996) Enhanced deposition of radon daughter nuclei in the vicinity of power frequency electromagnetic fields. *Int. J. Rad. Biol.* **69**, 25–38
8. Feychting, M., and Ahlbom, A. (1993) Magnetic fields and cancer in children residing near Swedish high-voltage power lines. *Am. J. Epidemiol.* **138**, 467–481
9. Olsen, J. H., Nielsen, A., and Schulgen, G. (1993) Residence near high voltage facilities and risk of cancer in children. *Br. Med. J.* **307**, 891–894
10. Verksalo, P. K., Pukala, E., Hongisto, M. Y., Valjus, J. E., Järvinen, P. J., Heikkilä, K. V., and Koskenvuo, M. (1993) Risk of cancer in Finnish children living close to power lines. *Br. Med. J.* **307**, 895–899
11. Ahlbom, A., Feychting, M., Koskenvuo, M., Olsen, J. H., Pukkala, E., Schulgen, G., and Verkasalo, P. (1993) Electromagnetic-fields and childhood-cancer. *Lancet* **342**, 1295–1296
12. Gurney, J. G., Mueller, B. A., Davis, S., Schwartz, S. M., Stevens, R. G., and Kopechy, K. J. (1996) Childhood brain tumour occurrence in relation to residential power line configurations, electric heating sources and electric appliance use. *Am. J. Epidemiol.* **143**, 120–128
13. Preston Martin, S., Navidi, W., Thomas, D., Lee, P. J., Bowman, J., and Pogoda, J. (1996) Los Angeles study of residential magnetic fields and childhood brain tumors. *Am. J. Epidemiol.* **143**, 105–119
14. Linet, M. S., Hatch, E. E., Kleinerman, R. A., Robison, L. L., Kaune, W. T., Friedman, D. R., Severson, R. K., Haines, C. M., Hartsock, C. T., Niwa, S., Wacholder, S., and Tarone, R. E. (1997) Residential exposure to magnetic fields and acute lymphoblastic leukemia in children. *New Engl. J. Med.* **337**, 1–7
15. Savitz, D. A., Wachtel, H., Barnes, F. A., John, E. M., and Tvrdik, J. G. (1988) Case control study of childhood cancer and exposure to 60 Hz magnetic fields. *Am. J. Epidemiol.* **128**, 21–38
16. Tomenius, L. (1986) 50-Hz electromagentic environment and the incidence of childhood tumours in Stockholm county. *Bioelectromagnetics* **7**, 191
17. London, S. J. (1991) Exposure to residential electric and magnetic fields and risk of childhood leukaemia. *Am. J. Epidemiol.* **134**, 923–937
18. Li, C. Y., Theriault, G., and Lin, R. S. (1996) Epidemiological appraisal of studies of residential exposure to power frequency magnetic fields and adult cancers. *Occup. Environ. Med.* **53**, 505–510
19. National Radiological Protection Board (1992) Electromagnetic fields and the risk of cancer: Report of an advisory group on non-ionising radiation. *Doc. NRPB* **3**, 1–138

20. National Radiological Protection Board (1994) Electromagnetic fields and the risk of cancer: Supplementary report by the advisory group on non-ionising radiation. *Doc. NRPB* **5**, 77–81
21. Theriault, G., Goldberg, M., Miller, A. B., Armstrong, B., Guenel, P., Deadman, J., Imbernon, E., To, T., Chevalier, A., Cyr, D., and Wall, C. (1994) Cancer risks associated with cccupational exposure to magnetic-fields among electric utility workers in Ontario and Quebec, Canada, and France—1970–1989. *Am. J. Epidemiol.* **139**, 550–572
22. Theriault, G., Goldberg, M., and Miller, A. B. (1994) Cancer risks associated with occupational exposure to magnetic-fields among electric utility workers in Ontario and Quebec, Canada, and France—1970–1989. *Am. J. Epidemiol.* **139**, 1053–1053
23. Guenel, P., Nicolau, J., Imbernon, E., Chevalier, A., and Goldberg, M. (1996) Exposure to 50-Hz electric field and incidence of leukemia, brain tumors, and other cancers among French electric utility workers. *Am. J. Epidemiol.* **144**, 1107–1121
24. Miller, A. B., To, T., Agnew, D. A., Wall, C., and Green, L. M. (1996) Leukemia following occupational exposure to 60-Hz electric and magnetic fields among Ontario electric utility workers. *Am. J. Epidemiol.* **144**, 150–160
25. Savitz, D. A., and Loomis, D. P. (1995) Magnetic-field exposure in relation to leukemia and brain cancer mortality among electric utility workers. *Am. J. Epidemiol.* **141**, 123–134
26. Floderus, B., Persson, T., Stenlund, C., Wennberg, A., Ost, A., and Knave, B. (1993) Occupational exposure to electromagnetic-fields in relation to leukemia and brain-tumors—a case-control study in Sweden. *Cancer Causes & Control* **4**, 465–476
27. Harrington, J. M., McBride, D. I., Sorahan, T., Paddle, G. M., and van Tongeren, M. (1997) Occupational exposure to magnetic fields in relation to mortality from brain cancer among electricity generation and transmission workers. *Occup. Environ. Med.* **54**, 7–13
28. Pool, R. (1990) Is there an EMF-cancer connection? *Science* **249**, 1096–1098
29. Tomlinson, I. P. M., Novelli, M. R., and Bodmer, W. F. (1996) The mutation rate and cancer. *Proc. Natl. Acad. Sci. USA* **92**, 14800–14803
30. Zipfel, P. F., Irving, S. G., Kelly, K., and Siebenlist, U. (1989) Complexity of the primary response to mitogenic activation of human T cells. *Mol. Cell. Biol.* **9**, 1041–1048
31. Almendral, J. M., Sommer, D., MacDonald-Bravo, H., Burckhardt, J., Perera, J., and Bravo, R. (1988) Complexity of the early genetic response to growth factors in mouse fibroblasts. *Mol. Cell. Biol.* **8**, 2140–2148
32. Fearon, E. R., and Vogelstein, B. (1990) A genetic model for colorectal tumorigenesis. *Cell* **61**, 759–767
33. Brown, K., Buchmann, A., and Balmain, A. (1990) Carcinogen-induced mutations in the mouse c-Ha-*ras* gene provide evidence of multiple pathways for tumour progression. *Proc. Natl. Acad. Sci. USA* **87**, 538–542
34. Barbacid, M. (1987) *Ras* genes. *Annu. Rev. Biochem.* **56**, 779–827
35. Minna, J. D. (1988) Autocrine growth factor production, chromosomal deletions and oncogene activation in the pathogenesis of lung cancer. *Lung Cancer* **4**, 6–10
36. Nafziger, J., Desjobert, H., Benamar, B., Guillosson, J. J., and Adolphe, M. (1993) DNA mutations and 50 Hz electromagnetic fields. *Bioelectrochem. Bioenerg.* **30**, 133–141
37. Tabrah, F. L., Mower, H. F., Batkin, S., and Greenwood, P. B. (1994) Enhanced mutgenic effect of a 60 Hz time-varying magnetic field on numbers of azide-induced TA100 revertant colonies. *Bioelectromagnetics* **15**, 85–93
38. Cadossi, R., Bersani, F., Cossarizza, A., Zucchini, P., Emilia, G., Torelli, G., and Franceschi, C. (1992) Lymphocytes and low-frequency electromagnetic fields. *FASEB J.* **6**, 2667–2674
39. Cohen, M. M., Kunska, A., Astemborski, J. A., McCulloch, D., and Pasekewitz, D. A. (1986) Effect of low-level 60 Hz electromagnetic fields on human lymphoid cells: I. Mitotic rate and chromosome breakage in human peripheral lymphocytes. *Bioelectromagnetics* **7**, 415–423
40. Cohen, M. M., Kunska, A., Astemborski, J. A., McCulloch, D., and Pasekewitz, D. A. (1986) Effect of low-level 60 Hz electromagnetic fields on human lymphoid cells: II. Sister chromatid exchanges in peripheral lymphocytes and lymphoblastoid cell lines. *Mutat. Res.* **172**, 177–184
41. Frazier, M. E., Samuel, J. E., and Kaune, W. T. (1984) Viabilities and mutation frequencies of CHO-K1 cells following exposure to 60-Hz electric fields. *232nd Hanford Life Sciences Symposium*, pp. 255–267, Richland, Oregon
42. Livingstone, G. K., Gandi, O. P., Chatterjee, I., Witt, K., and Roti Roti, J. L. (1986) Reproductive integrity of mammalian cells exposed to 60 Hz electromagnetic fields. (Report submitted to New York State power lines project)
43. Rosenthal, M., and Obe, G. (1989) Effect of 50 Hz electromagnetic fields on proliferation and on chromosomal alterations in human peripheral lymphocytes untreated or pre-treated with chemical mutagens. *Mutat. Res.* **210**, 329–335
44. Tofani, S., Ferrara, A., Anglesio, L., and Gilli, G. (1995) Evidence for genotoxic effects of resonant ELF magnetic fields. *Bioelectrochem. Bioenerg.* **36**, 9–13
45. Nordenson, I., Mild, K. H., Andersson, G., and Sandström, M. (1994) Chromosomal aberrations in human amniotic cells after intermittent exposure to fifty hertz magnetic fields. *Bioelectromagnetics* **15**, 293–301
46. Galt, S., Wahlström, J., Hammenius, Y., Holmquist, D., and Johannesson, T. (1995) Study of effects of 50Hz magnetic field on chromosome aberrations and the growth related enzyme ODC in human amniotic cells. *Bioelectrochem. Bioenerg.* **36**, 1–8
47. Saalman, E., Önfelt, A., and Gillstedt-Hedman, B. (1991) Lack of c-mitotic effects in V79 Chinese hamster cells exposed to 50 Hz magnetic fields. *Bioelectrochem. Bioenerg.* **26**, 335–338
48. Hintenlang, D. E. (1993) Synergistic effects of ionizing radiation and 60 Hz magnetic fields. *Bioelectromagnetics* **14**, 545–551
49. Batchelor, S. N., McLauchlan, K. A., and Shkrob, I. A. (1992) Reaction yield detected magnetic resonance and magnetic field effect studies of radical pairs containing electronically excited organic radicals. *Mol. Physics* **77**, 75–110
50. Batchelor, S. A., Kay, C. W. M., McLauchlan, K. A., and Yeung, M. T. (1993) Electron spin polarisation (CIDEP) and magnetic field effects (MFE) in systems involving sulfur-centered radicals. *J. Phys. Chem.* **97**, 4570–4572
51. Hamilton, C. A., Hewitt, J. P., and McLauchlan, K. A. (1988) High resolution studies of the effects of magnetic fields on chemical reactions. *Mol. Physics* **65**, 423–438
52. Batchelor, S. N., Kay, C. W. M., McLauchlan, K. A., and Shkrob, I. A. (1993) Time-resolved and modulation methods in the study of the effects of magnetic-fields on the yields of free-radical reactions. *J. Phys. Chem.* **97**, 13250–13258
53. Harkins, T. T., and Grissom, C. B. (1994) Magnetic field effects on B12 ethanolamine ammonia lyase: Evidence for a radical mechanism. *Science* **263**, 958–960
54. Roy, S., Noda, Y., Eckert, V., Traber, M. G., Mori, A., Liburdy, R., and Packer, L. (1995) The phorbol 12-myristate 13-acetate (PMA)-induced oxidative burst in rat peritoneal neutrophils is increased by a 0.1 mT (60 Hz) magnetic-field. *FEBS Lett.* **376**, 164–166
55. Mnaimneh, S., Bizri, M., and Veyret, B. (1996) No effect of exposure to static and sinusoidal magnetic fields on nitric oxide production by macrophages. *Bioelectromagnetics* **17**, 519–521
56. Rannug, A., Ekstrom, T., Mild, K. H., Holmberg, B., Gimenez-conti, I., and Slaga, T. J. (1993) A study on skin tumour-formation in mice with 50 Hz magnetic field exposure. *Carcinogenesis* **14**, 573–578
57. Rannug, A., Holmberg, B., Ekstrom, T., Mild, K. H., Gimenez-conti, I., and Slaga, T. J. (1994) Intermittent 50 Hz magnetic field and skin tumour promotion in SENCAR mice. *Carcinogenesis* **15**, 153–157
58. McLean, J. R. N., Stuchly, M. A., Mitchell, R. E. J., Wilkinson, D., Goddard, M., Lecuyer, D. W., Schunk, M., and Callery, E. (1991) Cancer promotion in a mouse skin model by a 60 Hz magnetic field. *Bioelectromagnetics* **12**, 273–288
59. Stuchly, M. A., McLean, J. R., Burnett, R., Goddard, M., Lecuyer, D. W., and Mitchell, R. E. (1992) Modification of tumour promoters in the mouse skin by exposure to an alternating magnetic field. *Cancer Lett.* **65**, 1–7
60. Mevissen, M., Stamm, A., Buntenkotter, S., Zwingelberg, R., Wahnschaffe, U., and Loscher, W. (1993) Effects of magnetic fields on mammary tumour development induced by 7, 12-dimethylbenz(a)anthracene in rats. *Bioelectromagnetics* **14**, 131–144
61. Cossarizza, A., Monti, D., Bersani, F., Cantini, M., Cadossi, R., Saachi, A., and Franceschi, C. (1989) Extremely low frequency pulsed electromagnetic fields increase cell proiferation in lym-

phocytes from young and aged subjects. *Biochem. Biophys. Res. Commun.* **160**, 692–698
62. Conti, P., Gigante, G. E., Cifone, M. G., Alesse, E., Fieschi, C., and Angeletti, P. U. (1985) Effect of electromagnetic field on two calcium dependent biological systems. *J. Bioelect.* **4**, 227–236
63. Conti, P., Gigante, G. E., Alesse, E., Cifone, M. G., Fieschi, C., Reale, M., and Angeletti, P. U. (1985) A role for calcium in the effect of low frequency electromagnetic field on the blastogenesis of human lymphocytes. *FEBS Lett.* **181**, 28–32
64. Mooney, N. A., Smith, R., and Watson, B. W. (1986) Effect of extremely-low-frequency pulsed magnetic fields on the mitogenic response of peripheral blood mononuclear cells. *Bioelectromagnetics* **7**, 387–394
65. Petrini, M., Polidori, R., Ambrogi, F., Vaglini, F., Zaniol, P., Ronca, G., and Conte, A. (1990) Effects of different low-frequency electromagnetic fields on lymphocyte activation. *J. Bioelectromagnetics* **9**, 159–166
66. Cain, C. D., Thomas, D. L., and Adey, W. R. (1993) 60 Hz magnetic field acts as co-promoter in focus formation of C3H/10T1/2 cells. *Carcinogenesis* **14**, 955–960
67. Reipert, B. M., Allan, D., and Dexter, T. M. (1996) Exposure to extremely low frequency magnetic fields has no effect on growth rate or clonogenic potential of multipotential haemopoietic progenitor cells. *Growth Factors* **13**, 205–217
68. Reipert, B. M., Allan, D., Reipert, S., and Dexter, T. M. (1997) Apoptosis in haemopoietic progenitor cells exposed to low-frequency magnetic fields. *Life Sci.* **61**, 1571–1582
69. Harland, J. D., and Liburdy, R. P. (1994) ELF inhibition of melatonin's and tamoxifen's action on MCF-7 cell proliferation. *Annu. Rev. Res. Biol. Effects of Electric and Magnetic Fields from the Generation, Delivery and Use of Electricity*, Albuquerque, New Mexico
70. Blackman, C. F., Benane, S. G., and House, D. E. (1993) Evidence for direct effect of magnetic fields on neurite outgrowth. *FASEB J.* **7**, 801–806
71. Bawin, S. M., and Adey, W. R. (1976) Sensitivity of calcium binding in cerebral tissue to weak environmental fields oscillating at low frequency. *Proc. Natl. Acad. Sci. USA* **73**, 1999–2003
72. Blackman, C. F., Benane, S. G., Kinney, L. S., House, D. E., and Joines, W. T. (1982) Effects of ELF fields on calcium ion efflux from brain tissue in vitro. *Radiat. Res.* **92**, 510–520
73. Blackman, C. F., Benane, S. G., Rabinowitz, J. R., House, D. E., and Joines, W. T. (1985) A role for the magnetic field in the radiation induced efflux of calcium ions from brain tissue *in vitro. Bioelectromagnetics* **6**, 327–337
74. Smith, S. D., McLeod, B. R., Liboff, A. R., and Cooksey, K. E. (1987) Calcium cyclotron resonance and diatom mobility. *Bioelectromagnetics* **8**, 215–227
75. Hojevik, P., Sandblom, J., Galt, S., and Hamnerius, Y. (1995) Ca^{2+} ion transport through patch-clamped cells exposed to magnetic fields. *Bioelectromagnetics* **16**, 33–40
76. Parkinson, W. C., and Hanks, C. T. (1989) Search for cyclotron resonance in cells in vitro. *Bioelectromagnetics* **10**, 129–145
77. Rozek, R. J., Sherman, M. L., Liboff, A. R., McLeod, B. R., and Smith, S. D. (1987) Nifedipine is an antagonist to cyclotron resonance enhancement of ^{45}Ca incorporation in human lymphocytes. *Cell Calcium* **8**, 413–427
78. Liboff, A. R., Rozek, R. J., Sherman, M. L., McLeod, B. R., and Smith, S. D. (1987) Ca^{2+}-45 cyclotron resonance in human lymphocytes. *J. Bioelect.* **6**, 13–22
79. Prasad, A. V., Miller, M. W., Carstensen, E. L., Cox, C., Azadniv, M., and Brayman, A. A. (1991) Failure to reproduce increased calcium uptake in human lymphocytes at purported cyclotron resonance exposure conditions. *Radiat. Environ. Biophys.* **30**, 305–320
80. Coulton, L. A., and Barker, A. T. (1993) Magnetic fields and intracellular calcium: effects on lymphocytes exposed to conditions of 'cyclotron resonance'. *Phys. Med. Biol.* **38**, 347–360
81. Lindström, E., Lindström, P., Berglund, A., Mild, K. H., and Lundgren, E. (1993) Intracellular calcium oscillations induced in a T-cell line by a weak 50-Hz magnetic-field. *J. Cell. Physiol.* **156**, 395–398
82. Korzhsleptsova, I. L., Lindström, E., Mild, K. H., Berglund, A., and Lundgren, E. (1995) Low-frequency Mfs increased inositol 1,4,5-trisphosphate levels in the Jurkat cell-line. *FEBS Lett.* **359**, 151–154
83. Walleczek, J., Killoran, P. L., Stagg, R. B., and Adey, W. R. (1994) Single cell fluorescence imaging study of the intracellular calcium concentration in human jurkat T-cells under the influence of 50 Hz magentic fields, *16th Annual Meeting of Bioelectromagnetics Society*, Copenhagen (abstr. 89)
84. Walleczek, J., and Liburdy, R. P. (1990) Nonthermal 60 Hz sinusoidal magnetic-field exposure enhances 45-Ca^{2+} uptake in rat thymocytes: dependence on mitogen activation. *FEBS Lett.* **271**, 157–160
85. Liburdy, R. P., Callahan, D. E., Harland, J., Dunham, E., Sloma, T. R., and Yaswen, P. (1993) Experimental evidence for 60 Hz magnetic fields operating through the signal transduction cascade. Effects on calcium influx and c-MYC mRNA induction. *FEBS Lett.* **334**, 301–308
86. Walleczek, J. (1992) Electromagnetic field effects on cells of the immune system: the role of calcium signaling. *FASEB J.* **6**, 3177–3185
87. Walleczek, J., Killoran, P. L., and Adey, W. R. (1994) Acute 60 Hz magnetic field effects on Ca^{2+} (Mn^{2+}) influx in human Jurkat T-cells: strict dependence on cell state. *16th Annual Meeting of Bioelectromagnetics Society*, Copenhagen (abstr. 76)
88. Barbier, E., Dufy, B., and Veyret, B. (1996) Stimulation of Ca^{2+} influx in rat pituitary cells under exposure to a 50 Hz magnetic field. *Bioelectromagnetics* **17**, 303–311
89. Byus, C. V., Pieper, S. E., and Adey, W. R. (1987) The effects of low-energy 60-Hz environmental fields upon the growth-related enzyme ornithine decarboxylase. *Carcinogenesis* **8**, 1385–1389
90. Litovitz, T. A., Krause, D., and Mullins, J. M. (1991) Effect of coherence time of the applied magnetic field on ornithine decarboxylase activity. *Biochem. Biophys. Res. Commun.* **178**, 862–865
91. Litovitz, T. A., Krause, D., Montrose, C. J., and Mullins, J. M. (1994) Temporally incoherent magnetic fields mitigate the response of biological systems to temporally coherent magnetic fields. *Bioelectromagnetics* **15**, 399–409
92. Azadniv, M., Klinge, C. M., Gelein, R., Carstensen, E. L., Cox, C., Brayman, A. A., and Miller, M. W. (1995) A test of the hypothesis that a 60-Hz magnetic field affects ornithine decarboxylase activity in mouse L929 cells in vitro. *Biochem. Biophys. Res. Commun.* **214**, 627–631
93. Greene, J. J., Skowronski, W. J., Mullins, J. M., and and, N. R. M. (1991) Delineation of electric and magnetic field effects of extremely low frequency electromagnetic radiation on transcription. *Biochem. Biophys. Res. Commun.* **174**, 742–749
94. Azadniv, M., and Miller, M. W. (1992) ^{3}H-Uridine uptake in human leukemia HL-60 cells exposed to extremely low frequency electromagnetic fields. *Biochem. Biophys. Res. Commun.* **189**, 437–444
95. Phillips, J. L., Haggren, W., Thomas, W. J., Ishida, J. T., and Adey, W. R. (1992) Magnetic field induced changes in specific gene transcription. *Biochim. Biophys. Acta* **1132**, 140–144
96. Morris, J. D. H. (1984) Biochemical events in the mitogenic stimulation of 3T3 fibroblasts. Ph.D. thesis, University of Cambridge, Cambridge, England
97. Goodman, R., Wei, L. X., Xu, J. C., and Henderson, A. (1989) Exposure of human cells to low-frequency electromagnetic fields results in quantitative changes in transcripts. *Biochim. Biophys. Acta* **1009**, 219–220
98. Goodman, R., and Shirley-Henderson, A. (1991) Transcription and translation in cells exposed to extremely low frequency electromagnetic fields. *Bioelectrochem. Bioenerg.* **25**, 335–355
99. Wei, L. X., Goodman, R., and Henderson, A. (1990) Changes in level of c-myc and histone H2B following exposure of cells to low-frequency sinusoidal electromagnetic fields: evidence for a window effect. *Bioelectromagnetics* **11**, 269–272
100. Goodman, R., Wei, L. X., Bumann, J., and Shirley-Henderson, A. (1992) Exposure to electric and magnetic (EM) fields increases transcripts in HL-60 cells: does adaptation to EM fields occur? *Bioelectrochem. Bioenerg.* **29**, 185–192
101. Blank, M., Khorkova, O., and Goodman, R. (1993) Similarities in the proteins synthesised by Sciara salivary gland cells in response to electromagnetic fields and to heat shock. *Bioelectrochem. Bioenerg.* **31**, 27–38
102. Gold, S., Goodman, R., and Shirley-Henderson, A. (1994) Exposure of simian virus-40-transformed human-cells to magnetic-fields results in increased levels of human T-antigen mRNA and protein. *Bioelectromagnetics* **15**, 329–336

103. Lin, H., Goodman, R., and Shirley-Henderson, A. (1994) Specific region of the c-myc promoter is responsive to electric and magnetic fields. *J. Cell. Biochem.* **54**, 281–288
104. Weisbrot, D. R., Khorkova, O., Lin, H., Henderson, A. S., and Goodman, R. (1993) The effect of low-frequency electric and magnetic fields on gene expression in saccharomyces cerevisiae. *Bioelectrochem. Bioenerg.* **31**, 167–177
105. Goodman, R., and Henderson, A. S. (1988) Exposure of salivary gland cells to low-frequency electromagnetic fields alters polypeptide synthesis. *Proc. Natl. Acad. Sci. USA* **85**, 3928–3932
106. Lacy-Hulbert, A., Wilkins, R. C., Hesketh, T. R., and Metcalfe, J. C. (1995) Cancer risk and electromagnetic fields. *Nature (London)* **375**, 23
107. Lacy-Hulbert, A., Wilkins, R. C., Hesketh, T. R., and Metcalfe, J. C. (1995) No effect of 60 Hz electromagnetic fields on *MYC* or β-actin expression in human leukemic cells. *Radiat. Res.* **144**, 8–17
108. Saffer, J. D., and Thurston, S. J. (1995) Cancer risk and electromagnetic fields. *Nature (London)* **375**, 22–23
109. Saffer, J. D., and Thurston, S. J. (1995) Short exposures to 60 Hz magnetic fields do not alter *MYC* expression in HL60 or Daudi cells. *Radiat. Res.* **144**, 18–25
110. Goodman, R., Blank, M., Lin, H., Dai, R., Khorkova, O., Soo, L., Weisbrot, D., and Henderson, A. (1994) Increased levels of hsp70 transcripts induced when cells are exposed to low frequency electromagnetic fields. *Bioelectrochem. Bioenerg.* **33**, 115–120
111. Lin, H., and Goodman, R. (1995) Electric and magnetic noise blocks the 60 Hz magnetic field enhancement of steady state c-*myc* transcript levels in human leukemia cells. *Bioelectrochem. Bioenerg.* **36**, 33–37
112. Kobayashi, A. K., Kirschvink, J. L., and Nesson, M. H. (1995) Ferromagnetism and EMFs. *Nature (London)* **374**, 123
113. Hsu, C.-Y., and Li, C.-W. (1994) Magnetoreception in honeybees. *Science* **265**, 95–97
114. Blank, M. (1995) EMF studies. *Science* **270**, 1104–1105
115. Goodman, R., Bumann, J., Wei, L. X., and Shirley-Henderson, A. (1992) Exposure of human-cells to electromagnetic-fields—effect of time and field-strength on transcript levels. *Electro-. Magnetobiol.* **11**, 19–28
116. Broude, N., Karabakhtsian, R., Shalts, N., Goodman, R., and Henderson, A. S. (1994) Correlation between the amplitude of plasma membrane fluctuations and the response of cells to electric and magnetic fields. *Bioelectrochem. Bioenerg.* **33**, 19–23
117. Uckun, F. M., Kurosaki, T., Jin, J., Jun, X., Morgan, A., Takata, M., Bolen, J., and Luben, R. (1995) Exposure of B-lineage lymphoid cells to low energy electromagnetic fields stimulates Lyn kinase. *J. Biol. Chem.* **270**, 27666–27670
118. Kharbanda, S., Yuan, Z. M., Rubin, E., Weichselbaum, R., and Kufe, D. (1994) Activation of Src-like p56/p53lyn tyrosine kinase by ionizing radiation. *J. Biol. Chem.* **269**, 20739–20743
119. Lerchl, A., Reiter, R. J., Howes, K. A., Nonaka, K. O., and Stokkan, K. A. (1991) Evidence that extremely low frequency Ca^{2+}-cyclotron resonance depresses pineal melatonin synthesis in vitro. *Neurosci. Lett.* **124**, 213–215
120. Yost, M. G., and Liburdy, R. P. (1992) Time-varying and static magnetic fields act in combination to alter calcium signal transduction in the lymphocyte. *FEBS Lett.* **296**, 117–122
121. Deibert, M. C., Mcleod, B. R., Smith, S. D., and Liboff, A. R. (1994) Ion resonance electromagnetic field stimulation of fracture healing in rabbits with a fibular ostectomy. *J. Orthop. Res.* **12**, 875–885
122. Fitzsimmons, R. J., Ryaby, J. T., Magee, F. P., and Baylink, D. J. (1994) Combined magnetic fields increased net calcium flux in bone cells. *Calcified Tissue Int.* **55**, 376–380
123. Jenrow, K. A., Smith, C. H., and Liboff, A. R. (1995) Weak extremely-low-frequency magnetic fields and regeneration in the planarian Dugesia tigrina. *Bioelectromagnetics* **16**, 106–112
124. Lednev, V. V. (1991) Possible mechanism for the influence of weak magnetic fields on biological systems. *Bioelectromagnetics* **12**, 71–75
125. Chiabrera, A., Bianco, B., Tommasi, T., and Moggia, E. (1992) Langevin-Lorentz and Zeeman-Stark models of bioelectromagnetic effects. *Acta Pharm.* **42**, 315–322
126. Blanchard, J. P., and Blackman, C. F. (1994) Clarification and application of an ion parametric resonance model for magnetic field interactions with biological systems. *Bioelectromagnetics* **15**, 217–238
127. Hendee, S. P., Faour, F. A., Christensen, D. A., Patrick, B., Durney, C. H., and Blumenthal, D. K. (1996) The effects of weak extremely low frequency magnetic fields on calcium/calmodulin interactions. *Biophys. J.* **70**, 2915–2923
128. Shuvalova, L. A., Ostrovskaya, M. V., Sosunov, V. A., and Lednev, V. V. (1991) Influence of a weak magnetic field under conditions of paramagnetic resonance on the rate of calmodulin-dependent phophorylation of myosin in solution. *Proc. Natl. Acad. Sci. USSR* **317**, 227–230
129. Markov, M. S., Wang, S., and Pilla, A. A. (1993) Effects of weak low frequency sinusoidal and DC magnetic fields on myosin phosphorylation in a cell free system. *Bioelectrochem. Bioenerg.* **30**, 119–126
130. Coulton, L. A., Barker, A. T., Andrea, J. E., and Walsh, M. P. (1997) The effect of static magnetic fields on the rate of phosphorlyation of myosin light-chain. *Biochem. Biophys. Res. Commun.* In Press
131. Galt, S., Sandblom, J., Hamnerius, Y., Hojevik, P., Saalman, E., and Norden, B. (1993) Experimental search for combined AC and DC magnetic field effects on ion channels. *Bioelectromagnetics* **14**, 315–27
132. Wang, K. W., and Hladky, S. B. (1994) Absence of effects of low-frequency, low-amplitude magnetic fields on the properties of gramicidin A channels. *Biophys. J.* **67**, 1473–1483
133. Wang, K. W., and Hladky, S. B. (1994) An upper limit for the effect of low frequency magnetic fields on ATP-sensitive potassium channels. *Biochim. Biophys. Acta* **1195**, 218–222
134. Tsong, T. Y. (1992) Molecular recognition and processing of periodic signals in cells: study of activation of membrane ATPases by alternating electric fields. *Biochim. Biophys. Acta* **1113**, 53–70
135. Blank, M. (1992) NaK-ATPase function in alternating electric fields. *FASEB J.* **6**, 2434–2438
136. Goodman, E. M., Greenebaum, B., and Marron, M. T. (1993) Altered protein synthesis in a cell-free system exposed to a sinusoidal magnetic field. *Biochim. Biophys. Acta* **1202**, 107–112
137. Goodman, E. M., Greenebaum, B., and Marron, M. T. (1994) Magnetic fields alter translation in *Escherichia coli*. *Bioelectromagnetics* **15**, 77–83
138. Blank, M. (1987) The surface compartment model: a theory of ion transport focused on ionic processes in the electrical double layers at membrane protein surfaces. *Biochim. Biophys. Acta* **906**, 277–294
139. Thomas, J. R., Schrot, J., and Liboff, A. R. (1986) Low-intensity magnetic fields alter operant behavior in rats. *Bioelectromagnetics* **7**, 349–357
140. Parkinson, W. C., and Sulik, G. L. (1992) Diatom response to extremely low-frequency magnetic fields. *Radiat. Res.* **130**, 319–330
141. Lawrence, A. F., and Adey, W. R. (1982) Nonlinear wave mechanisms in interactions between excitable tissue and electromagnetic fields. *Neurol. Res.* **4**, 115–153
142. Lin, S. R., and Lu, P. Y. (1989) An epidemiologic study of childhood cancer in relation to residential exposure to electromagnetic fields. Abstract from DOE/EPRI Contractors Meeting, Portland, Oregon
143. Fulton, J. P., Cobb, S., Preble, L., Leone, L., and Forman, E. (1980) Electrical wiring configurations and childhood leukemia in Rhode island. *Am. J. Epidemiol.* **111**, 292–296
144. Myers, A., Clayden, A. D., Cartwright, R. A., and Cartwright, S. C. (1989) Childhood cancer and overhead powerlines: a case-control study. *Br. J. Cancer* **63**, 977–978
145. Feychting, M., and Ahlbom, A. (1994) Magnetic-fields, leukemia, and central-nervous-system tumors in Swedish adults residing near high-voltage power-lines. *Epidemiology* **5**, 501–509
146. Coleman, M. P., Bell, C. M. J., Taylor, H.-L., and Primic-Zakelj, M. (1989) Leukemia and residence near powerlines: A case-control study. *Br. J. Cancer* **60**, 793–798
147. Severson, R. K., Stevens, R. G., Kaune, W. T., Thomas, D. B., Heuser, L., Davis, S., and Sever, L. E. (1988) Acute non-lymphocytic leukemia and residential exposure to power frequency magnetic fields. *Am. J. Epidemiol.* **128**, 10–20
148. Youngson, J. H. A. M., Clayden, A. D., Myers, A., and Cartwright, R. A. A. (1991) A Case/control study of adult haematological

malignancies in relation to overhead powerlines. *Br. J. Cancer* **63**, 977–985
149. Wertheimer, N., and Leeper, E. (1982) Adult cancer related to electrical wiring near the home. *Int. J. Epidemiol.* **11**, 345–355
150. Tynes, T., and Haldorsen, T. (1997) Electromagnetic fields and cancer in children residing near Norwegian high-voltage power lines. *Am. J. Epidemiol.* **145**, 219–226
151. Marino, A. A. (1993) Electromagnetic fields, cancer, and the theory of neuroendocrine-related promotion. *Bioelectrochem. Bioenerg.* **29**, 255–276
152. Hesketh, R. (1994) *Oncogenes*, Academic Press, New York
153. Nakanishi, O., Shibasaki, F., Hidaka, M., Homma, Y., and Takenawa, T. (1993) Phospholipase C-g1 associates with viral and cellular src kinases. *J. Biol. Chem.* **268**, 10754–10759
154. Johansen, T., Bjorkoy, G., Overvatn, A., Diaz-Meco, M. T., Traavik, T., and Moscat, J. (1994) NIH 3T3 cells stably transfected with the gene encoding phosphatidylcholine-hydrolyzing phospholipase C from *Bacillus cereus* acquire a transformed phenotype. *Mol. Cell. Biol.* **14**, 646–654
155. Barone, M. V., and Courtneidge, S. A. (1995) Myc but not Fos rescue of PDGF signalling block caused by kinase-inactive Src. *Nature (London)* **378**, 509–512
156. McLauchlan, K. (1992) Are environmental magnetic fields dangerous? *Physics World* **92**, 41–45
157. Conti, P., Gigante, G. E., Cifone, M. G., Alesse, E., Ianni, G., Reale, M., and Angeletti, P. U. (1983) Reduced mitogenic stimulation of human lymphocytes by extremely low frequency electromagnetic fields. *FEBS Lett* **162**, 156–160
158. Liburdy, R. P., Sloma, T. R., Sokolic, R., and Yaswen, P. (1993) ELF magnetic fields, breast cancer, and melatonin: 60 Hz fields block melatonin's oncostatic action on ER^+ breast cancer cell proliferation. *J. Pineal Res.* **14**, 89–97
159. Phillips, J. L., and McChesney, L. (1991) Effect of 72 Hz pulsed magnetic field exposure on macromolecular synthesis in CCRF-CEM cells. *Cancer Biochem. Biophys.* **12**, 1–7
160. Goodman, R., and Henderson, A. S. (1986) Sine waves enhance cellular transcription. *Bioelectromagnetics* **7**, 23–29
161. Prasad, A. V., Miller, M. W., Cox, C., Carstensen, E. L., Hoops, H., and Brayman, A. A. (1994) A test of the influence of cyclotron resonance exposures on diatom motility. *Health Phys.* **66**, 305–312
162. Davies, M., Dixey, R., and Green, J. (1993) The uptake of calcium as indicated by changes in the motility of diatoms, *Report to the National Grid Biological Effects Workshop, Royal Society*, London
163. Davies, M., Dixey, R., and Green, J. (1994) The uptake of calcium as indicated by changes in the motility of diatoms, Report to the EMF Biological Trust, Royal Society, London
164. Reese, J. A., Frazier, M. E., Morris, J. E., Buschbom, R. L., and L, M. D. (1991) Evaluation of changes in diatom motility after exposure to 16-Hz electromagnetic fields. *Bioelectromagnetics* **12**, 21–25
165. Lin, H., and Goodman, R. (1995) Electric and magnetic noise blocks the 60 Hz magnetic field enhancement of steady state c-*myc* transcript levels in human leukemia cells. *Bioelectrochem. Bioenerg.* **36**, 33–37
166. Karabakhtsian, R., Broude, N., Shalts, N., Kochlatyi, S., Goodman, R., and Henderson, A. S. (1994) Calcium is necessary in the cell response to EM fields. *FEBS Lett.* **349**, 1–6
