# Supplementary material for: Magnetic Fields and Cancer: Epidemiology, Cellular Biology, and Theranostics
Source: Int J Mol Sci. 2022 Jan 25;23(3):1339. doi: 10.3390/ijms23031339 (PMC8835851; doi:10.3390/ijms23031339)
Supplement: Supplementary file 1 [file ijms-23-01339-s001.zip › Supplementary Data Set S1/MF and Cancer.Data/PDF/2259223092/radiol.2019182947.pdf]

Radiology

Breast MRI: State of the Art

Ritse M. Mann, MD, PhD • Nariya Cho, MD • Linda Moy, MD

From the Department of Radiology and Nuclear Medicine, Radboud University Medical Centre, Nijmegen, the Netherlands (R.M.M.); Department of Radiology, the Netherlands Cancer Institute-Antoni van Leeuwenhoek Hospital, Amsterdam, the Netherlands (R.M.M.); Department of Radiology, Seoul National University Hospital, Seoul, Republic of Korea (N.C.); Department of Radiology, Seoul National University College of Medicine, Seoul, Republic of Korea (N.C.); Department of Radiology, Laura and Isaac Perlmutter Cancer Center, New York University School of Medicine, 160 E 34th St, New York, NY 10016 (L.M.); and Bernard and Irene Schwartz Center for Biomedical Imaging, Center for Advanced Imaging Innovation and Research, New York University School of Medicine, New York, NY (L.M.). Received December 27, 2018; revision requested February 8, 2019; final revision received March 27; accepted April 8. **Address correspondence** to L.M. (e-mail: *linda.moy@nyulangone.org*).

Conflicts of interest are listed at the end of this article.

Radiology 2019; 292:520–536 • https://doi.org/10.1148/radiol.2019182947 • Content codes: BR MR OI

MRI of the breast has the highest sensitivity for breast cancer detection among current clinical imaging modalities and is indispensable for breast imaging practice. While the basis of breast MRI consists of T1-weighted contrast-enhanced imaging, T2-weighted, ultrafast, and diffusion-weighted imaging may be used to improve lesion characterization. Such multiparametric assessment of breast lesions allows for excellent discrimination between benign and malignant breast lesions. Indications for breast MRI are expanding. In preoperative staging, multiple studies confirm the superiority of MRI to other imaging modalities for tumor size estimation and detection of additional tumor foci in the ipsilateral and contralateral breast. Ongoing studies show that in experienced hands this can be used to improve breast cancer surgery, although there is no evidence of improved long-term outcomes. Screening indications are likewise growing as evidence is accumulating that OncologicRI depicts cancers at an earlier stage than mammography in all women. To manage the associated costs for screening, the use of abbreviated protocols may be beneficial. In patients treated with neoadjuvant chemotherapy, MRI is used to document response. It is essential to realize that oncologic and surgical response are different, and evaluation should be adapted to the underlying question.

©RSNA, 2019

Online supplemental material is available for this article.

Online SA-CME • *See www.rsna.org/learning-center-ry*

Learning Objectives:

After reading the article and taking the test, the reader will be able to:

- Describe how multiparametric breast MRI protocols can be modified to address a particular clinical indication
- Identify the benefits and limitations of a preoperative breast MRI to stage local breast cancer, modify surgical approaches, and improve surgical outcomes
- Assess some state-of-the-art breast MRI techniques such as diffusion-weighted imaging, ultrafast imaging, and abbreviated MRI protocols

Accreditation and Designation Statement

The RSNA is accredited by the Accreditation Council for Continuing Medical Education (ACCME) to provide continuing medical education for physicians. The RSNA designates this journal-based SA-CME activity for a maximum of 1.0 *AMA PRA Category 1 Credit*™. Physicians should claim only the credit commensurate with the extent of their participation in the activity.

Disclosure Statement

The ACCME requires that the RSNA, as an accredited provider of CME, obtain signed disclosure statements from the authors, editors, and reviewers for this activity. For this journal-based CME activity, author disclosures are listed at the end of this article.

Breast MRI is an indispensable modality, along with mammography and US. Its main indications are staging of known cancer, screening for breast cancer in women at increased risk, and evaluation of response to neoadjuvant chemotherapy (1–3). As opposed to mammography and US, MRI is a functional technique. Heywang et al (4) and Kaiser and Zeitler (5) independently introduced this technique in the 1980s. Contrast material–enhanced MRI evaluates the permeability of blood vessels by using an intravenous contrast agent (gadolinium chelate) that shortens the local T1 time, leading to a higher signal on T1-weighted images (6). The underlying principle is that neoangiogenesis leads to formation of leaky vessels that allow for faster extravasation of contrast agents (7), thus leading to rapid local enhancement. Despite improvements in the technique of breast MRI, this principle is still the basis of all clinical MRI protocols. However, most MRI protocols nowadays are multiparametric (8,9). This review describes the current state of the art in breast MRI, with a focus on the major indications and the potential indication-based adaptations to the imaging protocol to maximize its value.

Requirements for Breast MRI

Breast MRI studies should be interpreted by radiologists with expertise in breast imaging, including mammographic and US studies, as these examinations are often complementary. Although empirical data on the learning curve for breast MRI are lacking, some studies showed improved performance of radiologists over time (10), and reporting breast MRI studies requires sufficient exposure to the technique.

It is best practice to use a field strength of at least 1.5 T to acquire images at a sufficiently high spatial resolution (1–3). Utilization of a dedicated breast coil is mandatory to obtain images of diagnostic quality. Women lie in the prone position with the breasts hanging free in the recesses of the coil. This design allows the breast tissue to spread, which facilitates detection of abnormalities and prevents motion artifacts induced by respiration (11,12). A breast coil should have at least four channels, but modern designs

This copy is for personal use only. To order printed copies, contact *reprints@rsna.org*

Abbreviations

ADC = apparent diffusion coefficient, BCS = breast-conserving surgery, BI-RADS = Breast Imaging Reporting and Data System, DCIS = ductal carcinoma in situ, DWI = diffusion-weighted imaging, EPI = echo-planar imaging, HER2 = human epidermal growth factor receptor 2, NAC = neoadjuvant chemotherapy, NME = non-mass enhancement, pCR = pathologic complete response, SNR = signal-to-noise ratio

Summary

Indications for breast MRI are consolidating; MRI for screening leads to earlier cancer detection in virtually all evaluated populations; in the hands of experienced teams, MRI allows for improvement of surgical practice, reducing the number of re-excisions while preventing unnecessary mastectomies; and MRI allows for patient selection for neoadjuvant chemotherapy and is the technique of choice to support modification of therapeutic agents and for presurgical assessment of residual tumor size to determine breast conservation surgery candidacy.

Essentials

- Breast MRI is a key imaging technique for breast imaging.
- Multiparametric breast MRI protocols can be adapted to the clinical indication.
- Translating preoperative MRI for extent of disease evaluation to better surgical outcomes requires experience in incorporating MRI findings for MRI-guided surgery, with lesion localization where appropriate.
- Screening with breast MRI leads to earlier cancer detection in all women.
- In the neoadjuvant setting, enhancement characteristics of lesions change, and assessment should be adapted to the clinical question that is to be answered.

have 16 channels or more, and sometimes also dedicated channels for the axillary region (13,14). In general, coils with more channels obtain a higher signal-to-noise ratio (SNR). More channels also enable the use of higher parallel imaging factors, which can increase the speed of image acquisition (13–15).

Because breast MRI aims to depict lesions that are occult with other modalities, it is essential that imaging facilities have tools to biopsy and localize these lesions for surgery (16,17). This may require an additional biopsy coil, as the latest generations of breast coils have closed recesses because of the high number of channels that are brought in proximity to the breast, consequently blocking access to the breast for interventional procedures. Also, a device that immobilizes the breast during biopsy is indispensable because introduction of a needle will change the shape of the breast and the position of the lesion.

Components of the Multiparametric Breast MRI Protocol

Breast MRI has evolved from a primarily contrast-enhanced technique to a multiparametric technique, in which T2-weighted and diffusion-weighted imaging (DWI) are routinely performed. Still, the basis for any MRI protocol is a dynamic T1-weighted contrast-enhanced sequence.

T1-weighted Imaging

For the T1-weighted series, intravenous access is required. T1-weighted imaging may be performed with or without fat suppression. Images are usually acquired in the axial plane, which is faster than sagittal acquisition and provides a better overview of both breasts. A native T1-weighted acquisition should be obtained prior to contrast material administration. Contrast material should be administered at a maximum dose of 0.1 mmol per kilogram of body weight, as there is no evidence for better performance with higher doses (18,19). Preferentially, a power injector should be used at a flow rate of 2 mL/sec. The contrast material bolus should be flushed with saline (a bolus of approximately 20 mL).

After contrast material administration, the T1-weighted acquisition is repeated to depict enhancing abnormalities. It is essential to obtain an image approximately 60–90 seconds after contrast material administration, as most breast cancers will show peak enhancement at that time. Lesion detection is primarily performed by using these postcontrast images. For images obtained without fat suppression, creating subtraction images from the pre- and postcontrast acquisitions is required (20). Subtraction images are also helpful for acquisitions with fat suppression because they help differentiate truly enhancing structures from lesions with native high signal intensity at T1 (21). Generating maximum intensity projections from these subtracted images aids in rapid lesion detection (22,23). However, motion artifacts, chemical shift artifacts, and poor fat suppression may obscure small lesions on maximum intensity projection images.

By convention, breast MRI should depict all enhancing cancers 5 mm or larger in size. Therefore, the section thickness of T1-weighted acquisitions should be no more than 2.5 mm. Because morphologic evaluation requires much finer detail, the in-plane pixel size should be 1×1 mm or lower (1–3). By using modern MRI units and breast coils, much higher resolutions (1 mm isotropic and lower) are easily obtainable without lengthening the acquisition time per volume beyond 90 seconds. This allows for reconstruction in any plane, facilitating evaluation of lesions, especially the distribution of non-mass lesions.

For lesion detection, the acquisition of two T1-weighted acquisitions at the specified time points (one before and one approximately 90 seconds after contrast material administration) is usually sufficient, as can be inferred from the success of abbreviated protocols for breast MRI (24). All other sequences improve the differentiation of breast lesions, with the aim to prevent false-positive and false-negative classification.

Dynamic Evaluation with Time–Signal Intensity Curves

Dynamic analysis investigates the permeability of the vessels that supply a lesion (5). This is done by obtaining a series of T1-weighted acquisition between 5 and 7 minutes after contrast material administration (25,26). In the case of leaky vessels, the peak contrast material accumulation will have passed, and contrast material is being removed from the lesion. In lesions with less-permeable vessels, the contrast gradient over the vessel wall will still be positive, and therefore the enhancement of the lesion still increases. This is reflected in the shape of the time–signal intensity curves; a persistent increase is most commonly seen in benign lesions, whereas a decrease in the late phase is

common in malignant lesions (27). To improve lesion classification, the most suspicious curve observed (washout > plateau > persistent) within a small region of interest (ROI) (typically 3 × 3 × 1 voxels) in the lesion is used. Approximately 85% of cancers manifest with a washout curve (26–29). Persistent curves are rare in malignancies, although they may be present in ductal carcinoma in situ (DCIS) and more diffuse-growing invasive cancers, particularly lobular breast cancers. ROI-based measurement of time–signal intensity curves can be replaced by soft-copy reading with visual assessment of the enhancement behavior of the entire tumor (ie, scrolling through the dynamic series of a given section). Also, software programs are available that generate color map overlays of the enhancement curve distribution within a lesion, which also replaces drawing ROIs and generating time–signal intensity curves.

Ultrafast Breast MRI

Ultrafast breast imaging documents the early inflow of contrast material in a lesion (28–30). Malignancies enhance both earlier and faster than benign lesions. Consequently, the first lesion that enhances in the breast is the most suspicious. Most breast cancers start to enhance within 10 seconds after the arrival of contrast material in the major vessels, whereas benign lesions, on average, enhance later (>15 seconds) (31,32). Faster enhancement translates to a steeper upslope of the first part of the time–signal intensity curve. Lesions can therefore be classified by using the time to enhancement relative to that in the descending aorta and the maximum slope of the enhancement curve (Fig 1). Accurate determination of these parameters requires very fast imaging techniques, typically on the order of 5 seconds per whole breast volume. When used for visual inspection, these volumes should still meet the minimum spatial requirements for T1-weighted images described above. This has become possible by using view-sharing and compressed sensing techniques at 3.0 T (33,34) but is not feasible with all clinical MRI units. However, ultrafast breast imaging enables dynamic evaluation of lesions without the penalty of a longer acquisition time and provides a similar accuracy for lesion classification.

T2-weighted Imaging

T2-weighted imaging is included in the standard MRI protocol (35,36). T2-weighted imaging with fat suppression enables easy visualization of cysts. T2-weighted imaging without fat suppression allows better depiction of lesion morphology. Most masses with high signal intensity at T2-weighted imaging are benign (eg, apocrine metaplasia, cyst, myxoid fibroadenoma, fat necrosis, and lymph nodes) (36). Most cancers do not show high signal intensity relative to parenchyma at T2-weighted imaging because of their high cellularity and low water content. However, mucinous carcinoma, necrotic cancer, and metaplastic carcinoma can have high signal intensity on T2-weighted images (2). T2-weighted imaging also allows the depiction of perifocal or prepectoral edema within the breast, which improves lesion classification (lesions with edema are more often malignant) and is a poor prognostic sign in patients with known breast cancer (37,38). Several studies have reported that T2-weighted imaging increases the specificity for differentiation of benign and malignant lesions (39,40). However, other investigators have questioned the added value of T2-weighted imaging in routine breast MRI, especially for inversion-recovery pulse sequences (1).

DWI Sequences

DWI quantifies the random movement of water molecules in tissue, which is influenced by tissue microstructure and cell density. This is achieved by applying motion-sensitizing gradients (*b* factors) to an (in essence) T2-weighted echo-planar imaging (EPI) sequence (41,42). Cancers show decreased water diffusion because of increased cell density, which leads to higher signal intensity at DWI. DWI is performed in a short acquisition time and does not rely on the administration of a contrast agent. To obtain adequate DWI acquisitions, the selection of appropriate *b* values, adequate fat suppression, minimization of artifacts, and sufficient SNR are crucial (42).

The apparent diffusion coefficient (ADC) is a quantitative measure of diffusivity derived from DWI (Appendix E1 [online]).

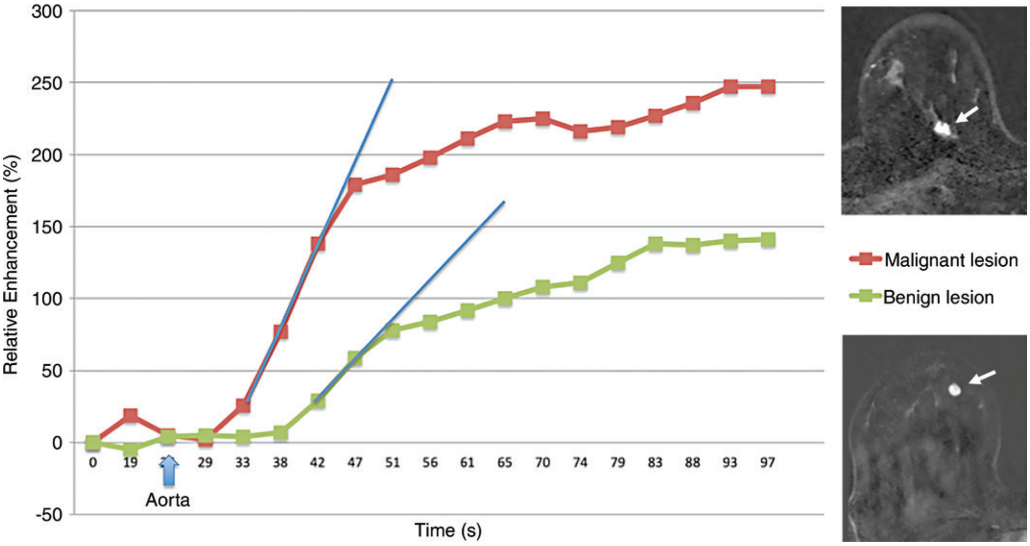

Figure 1: Graph shows inflow curves obtained from ultrafast MRI for two breast lesions. The red curve is obtained from an invasive ductal carcinoma manifesting as an irregular mass (arrow) located posteriorly in the right breast of a 53-year-old woman (upper inset, axial view). The curve shows early (only 8 seconds after the aorta) and rapid (steep slope) enhancement. Note that noise in the acquisition may lead to some fluctuation of the signal prior to contrast material administration. The green curve is obtained from a fibroadenoma manifesting as a well-circumscribed mass (arrow) located anterior in the right breast of a 46-year-old woman (lower inset, axial view). The curve shows late (17 seconds after the aorta) and intermediate enhancement.

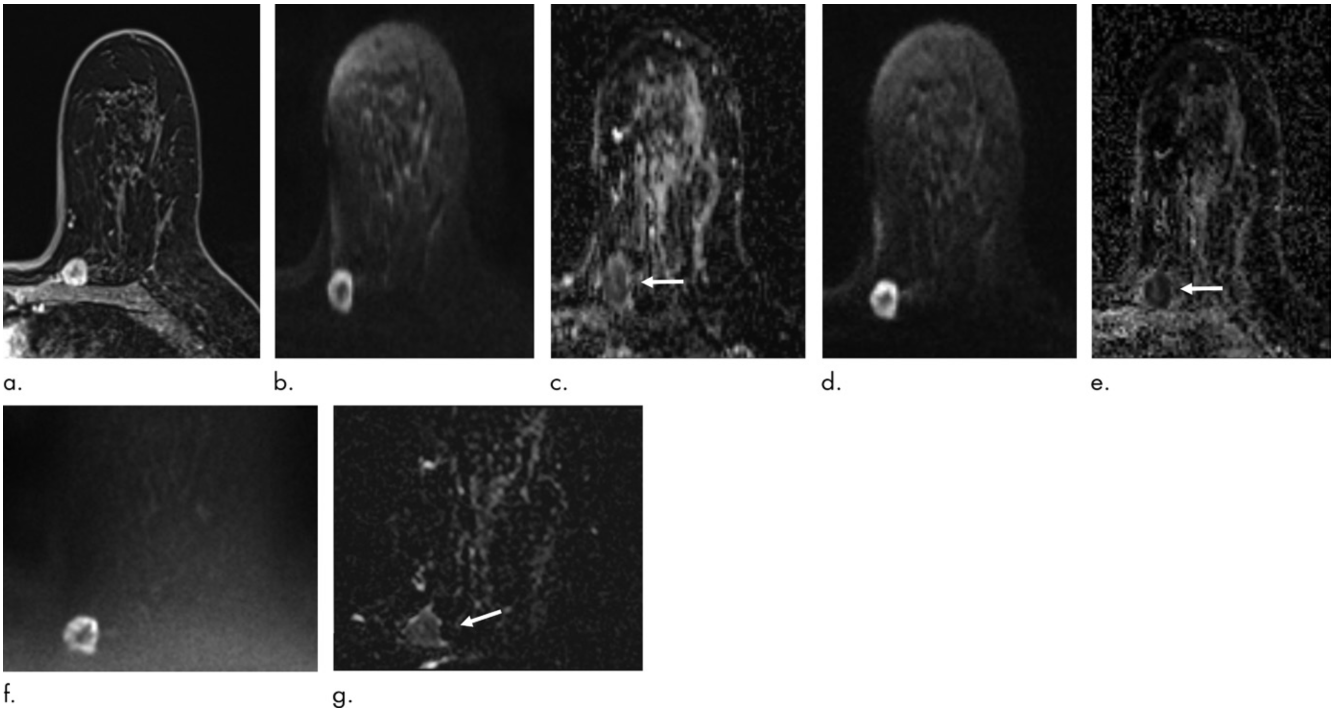

Figure 2: Axial images from breast MRI in a 52-year-old woman with an invasive ductal carcinoma. **(a)** Contrast-enhanced T1-weighted image shows a round rim-enhancing mass. **(b)** Image from single-shot echo-planar imaging (EPI) (conventional) diffusion-weighted imaging (DWI) shows more artifacts in the background. The shape of the mass is distorted. **(c)** Apparent diffusion coefficient (ADC) map from conventional DWI shows the mass with decreased signal intensity, suggesting diffusion restriction (arrow). **(d)** Image from read-out segmented EPI (rs-EPI) DWI and **(e)** ADC map of rs-EPI DWI show improved depiction of lesion morphology (conspicuity) (arrow) and fewer artifacts in the background. **(f)** Image from reduced field of view (rFOV) DWI and **(g)** ADC map of rFOV DWI show improved depiction of morphologic detail and intratumoral heterogeneity (arrow). (Images courtesy of Hee Jung Shin, MD, PhD, Department of Radiology, Asan Medical Center, University of Ulsan College of Medicine, Seoul, Korea.)

Values are usually expressed in 10^{-3} mm^2/sec. Because of the hindered diffusion in cancers, mean ADCs are generally low (range, 0.8–1.3 × 10^{-3} mm^2/sec) compared with those in benign lesions (range, 1.2–2.0 × 10^{-3} mm^2/sec) (43). Consequently, cancers have a low signal intensity on the derived ADC maps.

Studies have reported that diagnostic performance in distinguishing benign and malignant lesions is affected neither by the choice of field strength (1.5-T or 3.0-T MRI unit [43]) nor by the combination of multiple *b* values (44,45). Although sensitivity and specificity were not affected by the combination of *b* values in a meta-analysis of 26 studies, the ADC measurements did vary depending on *b* values (45) because the signal decay is not truly monoexponential but in fact decreases at higher *b* values. Consequently, using lower *b* values yields higher ADCs, and using higher *b* values yields lower ADCs (42). A study (46) has shown that the contrast of tumor to normal parenchyma increases with a *b* value from 0 to 1500 sec/mm^2 and decreases with a *b* value greater than 1500 sec/mm^2, but the higher *b* values lead to decreased SNR. In addition, DWI performed by using a *b* value of less than 1000 sec/mm^2 showed the greatest accuracy in distinguishing benign and malignant lesions (47,48). Considering these studies and to ensure reproducibility of ADCs between sites, *b* values of 0 and 800 sec/mm^2 are recommended for clinical practice, providing a balance between sufficient diffusion weighting and acceptable SNR. In addition, although contrast material injection before DWI does not significantly affect the diagnostic properties of the ADC, ADCs may change depending on the exact sequence type used (45). Consequently, it may be beneficial to perform DWI before contrast material administration.

Adequate fat suppression is critical in DWI to minimize EPI-related artifacts such as ghosting and chemical shift. Also, if fat suppression is inadequate, measurement of ADC is underestimated (49). Spectral selection-attenuated inversion recovery, or SPAIR, is commonly used (50). Short inversion time inversion recovery is more robust but results in a lower SNR and more variability in ADC measurements (51).

Single-shot EPI–based readout is the standard sequence for DWI acquisition, because it is fast and motion insensitive and yields a high SNR (52). However, it is susceptible to artifacts such as ghosting, chemical shift, and distortion, which are mainly due to the slow traversal through k-space along the phase-encoding direction. To reduce these artifacts, readout-segmented EPI has been used to restrict the readout acquisition in each shot, leading to a reduction of susceptibility artifacts at the expense of longer imaging time (52). Several studies have demonstrated the superior image quality of readout-segmented EPI DWI compared with single-shot EPI DWI (53,54), although the SNR is lower.

Alternatively, reduced field of view techniques obtain images for a target by reducing matrix size, also leading to decreased susceptibility artifacts and increased spatial resolution at the expense of longer imaging time compared with single-shot EPI DWI. A study (55) reported that reduced field of view EPI provided higher image quality, lesion conspicuity, and SNR than did readout-segmented EPI (Fig 2).

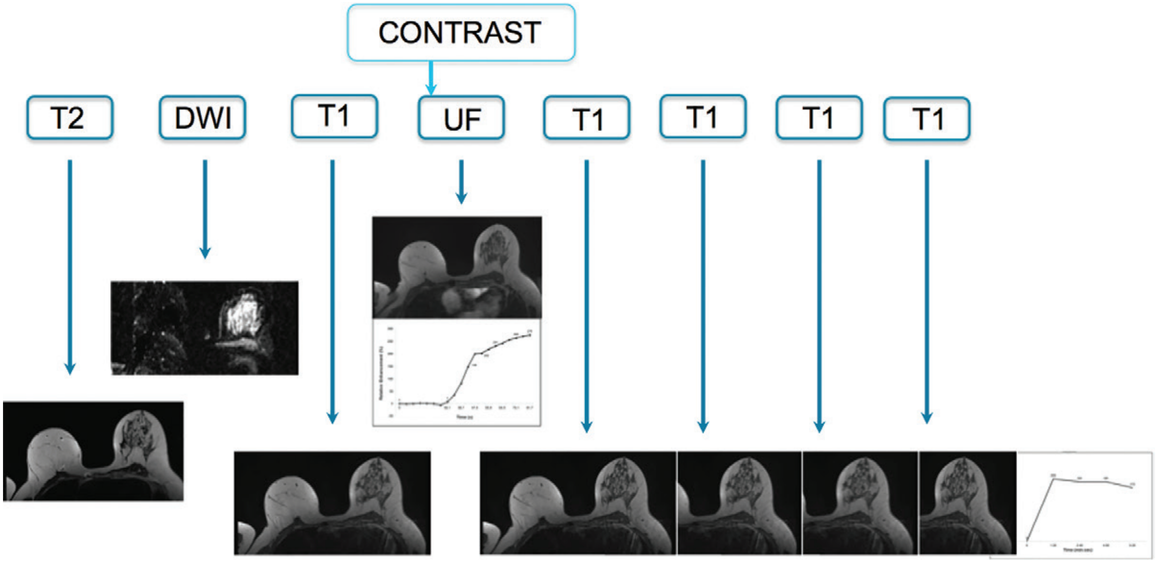

Figure 3: Components of the basic multiparametric breast MRI protocol. In general, the protocol is begun with the non–contrast-enhanced acquisitions (T2-weighted and diffusion-weighted imaging [DWI]). This is followed by a native T1-weighted acquisition and subsequently the contrast-enhanced series (ultrafast [UF] imaging and regular T1-weighted imaging). For screening purposes, this protocol may be abbreviated to contain only the T1-weighted acquisitions before and directly after contrast material administration, with or without the acquisition of ultrafast images (or only ultrafast images if they are of sufficiently high resolution). For lesion discrimination, adding T2-weighted imaging and DWI is beneficial. The information from ultrafast images is in essence similar to (although somewhat more discriminative than) the delayed phase dynamics, and these can therefore both be used. After neoadjuvant chemotherapy the delayed phase is essential to document the presence of residual ductal carcinoma in situ.

The Multiparametric Protocol

The various components of the basic multiparametric protocol are shown in Figure 3. For further improvement of lesion classification, other sequence types and postprocessing methods are being evaluated. These include the quantitative assessment of contrast material enhancement, advanced DWI techniques, and spectroscopic imaging. Several techniques are described in Appendix E1 (online).

Breast Lesion Evaluation at Breast MRI

Reporting of breast MRI is standardized in the American College of Radiology Breast Imaging Reporting and Data System (BI-RADS) (56). A standard report contains the clinical indication, the MRI sequences and postprocessing methods that were used, and the amount and type of contrast agent administered. Subsequently, the composition of the breast and the amount of background parenchymal enhancement (BPE) should be stated. For both measures, a higher fraction is associated with a higher likelihood of malignancy being present (57,58). Still, the correlation between the amount of fibroglandular tissue, amount of BPE, and breast cancer risk in the future is incompletely understood. A higher fraction of BPE leads to a higher risk of false-positive findings (59).

The morphologic and kinetic features of findings are described by using the BI-RADS lexicon. Lesions are categorized as foci (<5 mm of enhancement and by definition too small to characterize any further, but standing out from the surroundings), masses (space-occupying lesions), and non-mass enhancement (NME) (areas of enhancement without a clear space-occupying lesion present). Masses are further characterized on the basis of their shape, margins, and internal enhancement pattern. Areas of NME are further described according to distribution and internal enhancement pattern. For both lesion types, initial and delayed phase enhancement are described to improve the differential diagnosis.

Approximately two-thirds to three-quarters of cancers manifest as a mass, including most invasive ductal cancers (60,61); the remainder are visible as areas of NME, including the majority of cases of DCIS (60). Typical malignant masses have an irregular size and margin, heterogeneous or rim enhancement patterns, and show washout (Fig 2). Classic malignant areas of NME have a segmental distribution and a clumped or clustered ring pattern of internal enhancement (Fig 4). While most cancers are easily recognizable by their morphologic features alone, smaller lesions are more difficult to assess (62). In general, the features of NME are less specific than those of masses (60,63,64). Foci have a likelihood of malignancy of 2.9%–6% (65).

On the basis of the above analysis of morphologic and kinetic features, the radiologist assigns a final assessment, or BI-RADS score, from 0 to 6. However, unlike the Prostate Imaging Reporting and Data System, or PI-RADS, lexicon, the BI-RADS lexicon does not provide information on the associated "likelihood of malignancy" of the individual findings. It provides descriptor terms—not an interpretation guideline. To aid in the classification, a tree flowchart has been developed in which a decision rule assigns the levels of suspicion to specific combinations of imaging features. The decision tree may standardize reporting and improve the discrimination between benign and malignant lesions. It incorporates some of the BI-RADS descriptors as well as the presence or absence of a root sign and edema. A root sign

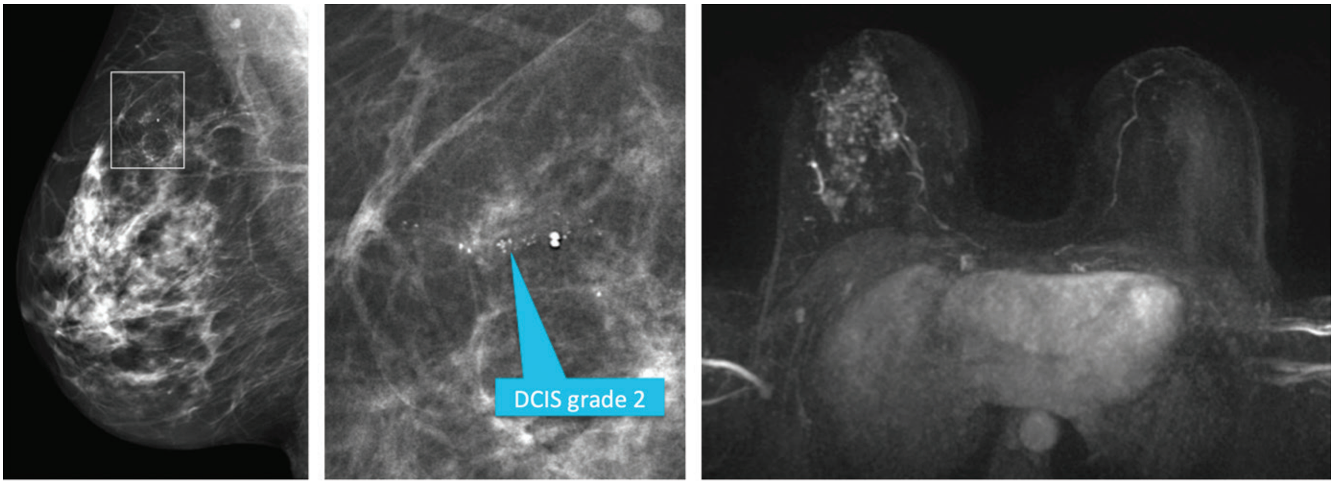

a. b. c.

Figure 4: **(a)** Mediolateral oblique view from a screening mammogram in a 54-year-old woman shows a small cluster of microcalcifications in the upper outer quadrant of the right breast. A detailed spot magnification view is given in (**b**). Stereotactic biopsy revealed grade 2 ductal carcinoma in situ (DCIS). **(c)** Maximum intensity projection from subsequent staging MRI shows a large area of segmental clumped non-mass enhancement in the right breast, representing the actual extent of disease.

is a spiculelike extension from the lesion margin, even when the rest of the margin is smooth; its presence strongly increases the likelihood of malignancy. Study results show the decision tree improved the diagnostic accuracy of inexperienced readers and reduced the number of benign findings at biopsy by more than 25% (66,67).

Although findings from ultrafast acquisitions and DWI have not been incorporated into the decision tree, many studies have shown that the combination of ultrafast acquisitions, T2-weighted imaging, and DWI improves the diagnostic accuracy of discriminating benign from malignant masses. Late and slow initial enhancement, high T2 signal, and high ADC substantially decrease the likelihood of malignancy and may be used to avoid biopsy. In particular, ADCs greater than 1.4×10^{-3} mm^2/sec are exceptionally rare in cancers. On the other hand, early and fast enhancement, a low T2 signal, the presence of edema, and a low ADC increase the level of suspicion. A specificity of 90% has been reported with use of a multiparametric approach (68). BI-RADS and supplemental descriptors are presented in Table 1.

Breast MRI in Clinical Practice

Staging in Women with Known Breast Cancer

Preoperative MRI for local staging of a known breast cancer is a common but controversial indication. The detection of more disease at MRI has not translated into improved outcomes. Consequently, guidelines differ widely in their recommendations for the performance of preoperative breast MRI in women with a new diagnosis of breast cancer (1–3,69–72) (Table 2).

Women with a diagnosis at a young age; those with an initial cancer manifesting as interval cancer; those with hormone receptor–negative cancers or dense breasts; and breast conservation without radiation therapy are all associated with an increased risk of invasive interval cancers in the postoperative period (73,74). Women with any of these factors are therefore good candidates for preoperative evaluation with MRI. In addition, most guidelines recommend MRI for the staging of invasive lobular cancers, as the performance of conventional modalities and clinical breast examination is limited.

Quality of preoperative staging.—By using breast MRI, approximately 75% of cancers are measured within 1 cm from the pathologic size, with similar percentages of over- and underestimation. However, reported accuracies of tumor size measurement vary widely, ranging from less than 50% of cancers measured within 1 cm to more than 80% of cancers measured within 5 mm. Regardless, most studies conclude that size estimations with MRI are more reliable than those with clinical examination, mammography, or US. Accuracy decreases in larger cancers and is worse in NME than in mass lesions (75–77). It is important to realize that the pathologic reference standard has substantial limitations. For example, the pathologic evaluation of breast specimens changes with gross anatomy, and a perfect correlation can never be achieved.

The benefit of using MRI to assess tumor size is particularly strong for invasive lobular carcinomas (78–80). Likewise, the depiction of DCIS components related to invasive cancers is much better than that with mammography, the latter missing more than half of all lesions, whereas the sensitivity for large DCIS components with MRI approaches 100% (81,82). Breast MRI is also more accurate in the depiction of pure DCIS lesions, particularly high-grade lesions (83,84) (Fig 4); however, small DCIS lesions detected because of calcifications at mammography may be occult at MRI.

Management of lesions detected at MRI.—With preoperative MRI, the detection of mammographic occult lesions in the affected breast is common. A meta-analysis of 50 studies (85) found that two-thirds of additional findings (67%) are malignant, and

Table 1: BI-RADS and Supplemental Descriptors for the Evaluation of Lesions at Breast MRI

Sequence and Description	Descriptor	Terms
T1 native		
Breast composition	Fibroglandular tissue	Almost entirely fatty; scattered; heterogeneous; extreme
T1 postcontrast (approximately 90 sec)/SUB		
Background signal	Background parenchymal enhancement	Minimal; mild; moderate; marked
Lesion	Lesion type	Focus; mass; non-mass
Mass		
	Shape	Round; oval; irregular
	Margin	Circumscribed; irregular; spiculated
	Internal enhancement pattern	Homogeneous; heterogeneous; rim enhancement; dark internal septations
Non-mass enhancement		
	Distribution	Focal; linear; segmental; regional; multiple regions; diffuse
	Internal enhancement pattern	Homogeneous; heterogeneous; clumped; clustered ring
T1 dynamic		
Signal intensity vs time curve	Initial enhancement (relative enhancement at 90 sec)	Slow, <50%; medium, 50%–100%; fast, >100%
	Delayed phase (relative enhancement compared with peak)	Persistent, >10% increase; plateau, –10% to +10%; washout, >10% decrease
Ultrafast		
Inflow curve	Time to enhancement (sec)	Early, <10; intermediate, 10–15; late, >15
	Maximum slope (%/sec)	Slow, <6.4; intermediate, 6.4–13.3; rapid, >13.3
T2		
Lesion T2 signal intensity	Signal intensity	High; intermediate, low
Edema	Presence	Absent; perifocal; prepectoral; unilateral diffuse; bilateral diffuse
DWI		
Lesion diffusion level	ADC (mm^2/sec)	Very low, <0.9; low, 0.9–1.3; intermediate, 1.3–1.7; high, 1.7–2.1; very high, > 2.1

Note.—ADC = apparent diffusion coefficient, BI-RADS = Breast Imaging Reporting and Data System, DWI = diffusion-weighted imaging, SUB = subtraction.

Table 2: Recommendations for Selecting Patients with Known Breast Cancer for Preoperative Evaluation with Breast MRI according to Various National and International Guidelines

Guideline	Recommendation
EUSOBI (2008)	Dense breasts; invasive lobular carcinoma; screening of the contralateral breast (all patients)
EUSOMA (2010)	Invasive lobular carcinoma; patients at high risk; patients < 60 years with size discrepancy between mammography and US > 1 cm; patients eligible for partial breast irradiation
ACR practice guideline (2018)	No subgroups defined; MRI useful for determining extent of tumor, evaluation of the tumor's relation to the deep fascia, and screening of the contralateral breast
NCCN breast cancer guideline (2018)	No subgroups defined; MRI is optional
NICE breast cancer guideline (2018)	Discrepancy in clinical examination, mammography, and US; dense breasts precluding size assessment at mammography; invasive lobular carcinoma if breast-conserving therapy is planned
Dutch breast cancer guideline (2018)	Discrepancy in clinical examination, mammography, and US; invasive lobular carcinoma if breast-conserving therapy is planned; high-grade DCIS and uncertain extent; DCIS with microinvasion
AGO (German Gynecologic Oncology group) (2018)	MRI optional in dense breasts, nipple involvement, invasive lobular carcinoma, suspicion of multifocal disease, and patients at high risk

Note.—ACR = American College of Radiology, AGO = Association of Gynecological Oncology, DCIS = ductal carcinoma in situ, EUSOBI = European Society of Breast Imaging, EUSOMA = European Society of Breast Cancer Specialists, NCCN = National Comprehensive Cancer Network, NICE = National Institute for Health and Care Excellence.

additional disease that impacts treatment is detected in 20% of patients. Therefore, findings that impact the planned surgical procedure should be confirmed at pathologic examination prior to treatment. In initial studies, conversion of breast-conserving surgery (BCS) to mastectomy for benign lesions was reported (86), which should be prevented. The high frequency of incidental lesions makes the use of a multiparametric protocol very valuable, because it may allow the classification of lesions as certainly benign and obviate biopsy. For indeterminate lesions, MRI-directed US allows for a US-guided biopsy in 57.5% of lesions, albeit more often for mass lesions than for areas of NME (87). Lesions that are occult at MRI-directed US remain suspicious and should be sampled with MRI-guided biopsy.

Using MRI findings in surgery.—Despite more accurate staging with MRI, this information has not translated into improved surgical outcomes. Study results show that women undergoing MRI have a higher likelihood of undergoing mastectomy; an odds ratio of 1.39 was reported in the latest meta-analysis (88). However, incorporating the MRI information to reduce the re-excision rates in women treated with BCS is challenging. In the Comparative Effectiveness of MRI in Breast Cancer, or COMICE, trial (89), a nationwide British prospective randomized controlled study, surgeons did not use the better staging information from preoperative MRI, leading to a virtually identical re-excision rate of 19% in both arms. Moreover, additional lesions led to mastectomy because they were presumed to be malignant because MRI-guided biopsy was not commonly available. However, a Swedish trial (90) showed an overall reduction in re-excision rates, from 15% to 5%. These two articles illustrate the importance of MRI guidance of surgery. MRI-guided localization, or MRI-guided bracketing of the extent of a larger tumor or DCIS, helps surgeons use the information MRI offers (91). For lobular cancers, evidence clearly points to a reduction in the rate of re-excisions without increasing the rate of mastectomies (91–93).

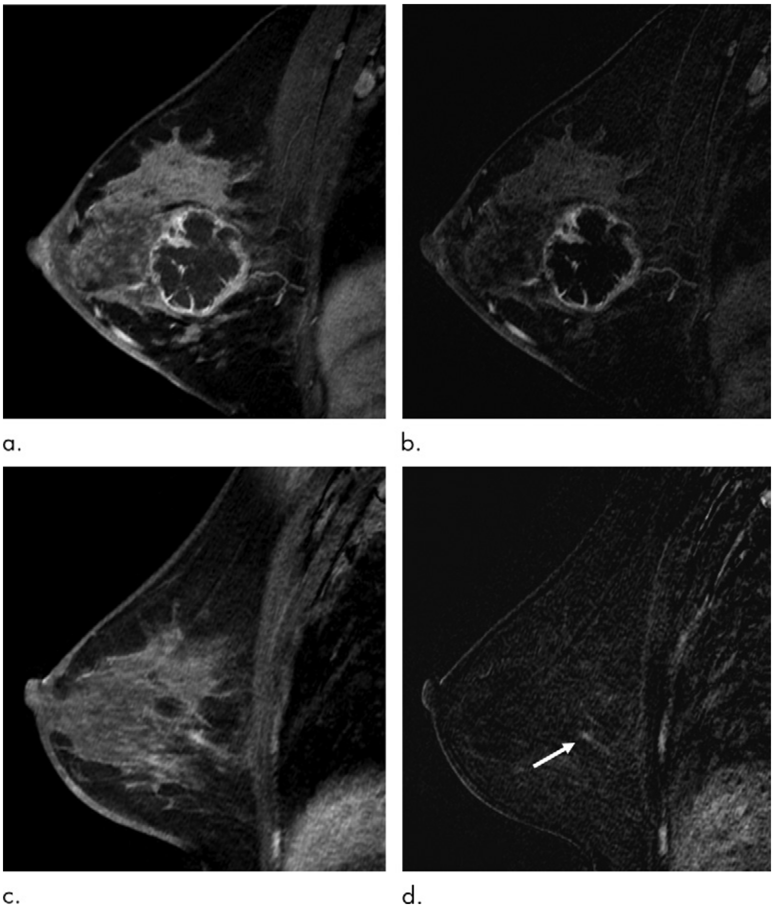

Figure 5: Sagittal images from breast MRI in a 36-year-old woman with an invasive ductal carcinoma. **(a)** Image from pre-chemotherapy contrast-enhanced T1-weighted MRI shows a round, rim-enhancing mass. **(b)** Subtraction image on which the maximal diameter of the mass was measured up to 5.4 cm. **(c)** On image obtained after completion of six cycles of chemotherapy, the mass is not seen, and there is no residual enhancement on **(d)** the subtraction image (arrow). Surgical histopathologic examination revealed no residual invasive or in situ cancer, indicating pathologic complete response.

These differences likely reflect the experience of breast surgeons with breast MRI and whether these findings are discussed in the multidisciplinary team. Also, the use of modern oncoplastic surgical techniques may affect the value of MRI. Unfortunately, these factors are not incorporated in any of the meta-analysis available, and because the largest trials failed to account for experience, available meta-analytic data are unreliable. The Preoperative Breast MRI in Clinical Practice: Multicenter International Prospective Meta-Analysis of Individual Data, or MIPA, trial is designed to overcome these issues by prospectively monitoring the effect of preoperative breast MRI in expert centers. Preliminary data in 2425 patients were reported at the European Congress of Radiology in 2018 (94). Results showed that women scheduled to undergo mastectomy are in fact more likely to undergo breast MRI, which explains the higher odds ratio for undergoing mastectomy mentioned above. Overall, breast MRI reduces the rate of re-excisions from 13.4% to 8%, while changes to more extensive surgery (12.5%) are balanced by changes to less extensive surgery in a virtually equal proportion of patients (12.7%). Whether these results are specific to certain groups of women with breast cancer remains to be evaluated.

Detection of contralateral breast cancer.—Breast MRI depicts occult contralateral disease in 5.5%–9.3% of women with known unilateral breast cancer; 37%–48% of these findings (2%–4%) are malignant (85,95). The detected cancers are small (<1 cm), and about one-third are DCIS. In essence, the detection of contralateral cancer is a form of high-risk screening, with a cancer yield that exceeds the yield in *BRCA* mutation carriers. To date, no factors, including breast density, are associated with the likelihood of detecting contralateral cancer (96).

Long-term outcome effects of preoperative MRI.—To our knowledge, only a few studies have examined the long-term outcome effects of preoperative MRI. Most studies do not show a reduction in metachronous second breast cancers (97,98), although one study showed a significant reduction in the occurrence of metachronous contralateral breast cancer (99). There is no evidence for improved disease-free or overall survival owing to preoperative MRI, although there is evidence that early detection of second cancers improves relative overall survival (100,101), particularly in younger women (102). However, these studies are mostly based on early reports that did not show a surgical benefit of MRI. These initial studies did not incorporate the MRI data (eg, MRI-guided biopsies were not performed for suspicious findings). More recent but underpowered studies have shown nonsignificant reductions of ipsilateral tumor recurrence in women undergoing preoperative MRI (103,104). In women who undergo breast-conserving treatment without radiation therapy, preoperative MRI improves breast cancer survival (odds ratio, 0.57) (98).

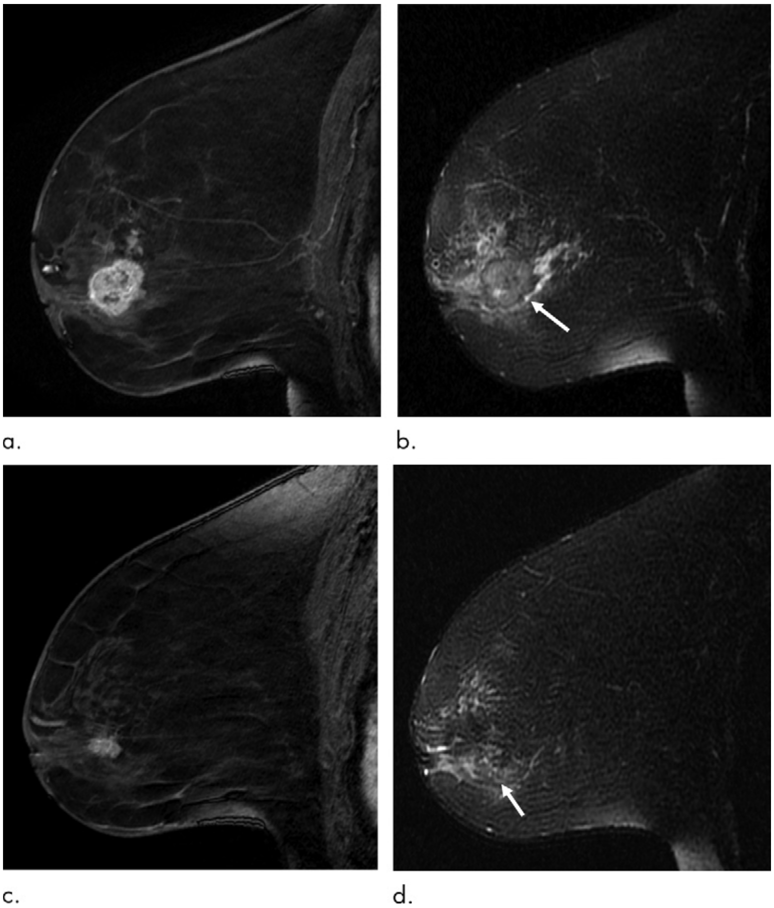

Figure 6: Sagittal images from breast MRI in a 69-year-old woman with an invasive ductal carcinoma of the triple-negative subtype. **(a)** Image from pre-chemotherapy contrast-enhanced T1-weighted MRI shows a round, enhancing mass. The maximal diameter of the mass was measured as 3.3 cm. **(b)** Image from pre-chemotherapy T2-weighted MRI shows a round mass with peritumoral edema (arrow). **(c)** On image obtained after the completion of six cycles of chemotherapy, the mass shows a concentric shrinkage pattern. The maximal diameter of the residual mass was measured as 1.4 cm in the early postcontrast phase, and $[(3.3 - 1.4)/3.3] \cdot 100\% = 58\%$ reduction in the initial tumor diameter. It is suggestive of partial response. **(d)** Image from post-chemotherapy T2-weighted MRI shows an indistinct mass with disappeared peritumoral edema (arrow). Surgical histopathologic examination revealed a 1.5-cm invasive ductal carcinoma.

Screening Breast MRI

In high-risk populations, MRI is recommended as a supplemental screening examination by multiple national and international guidelines (105–107). This consensus is based on multiple studies that showed that MRI identified earlier stage disease than mammography and that combined MRI and mammography is associated with improved survival rates (108,109). One should note that the literature on screening MRI is focused on high-risk women, in whom there is a higher prevalence of breast cancer and the sensitivity of mammography is lower. The widely adopted American Cancer Society guidelines stratified three categories of risk, on the basis of literature and/or expert consensus, to identify those women who may benefit from annual screening breast MRI (105). The risk factors include various germline mutations, family history, and personal risk factors. Women may undergo genetic testing to determine if they carry these deleterious mutations, and there are various risk prediction models to determine their lifetime risk. The Tyrer-Cuzick (International Breast Cancer Intervention Study, or IBIS) model is considered to be the most comprehensive model and is often used to determine whether breast MRI should be performed.

High-risk women.—The American Cancer Society and the American College of Radiology categorize women with a lifetime risk of more than 20% as high risk and recommend annual screening MRI and mammography in this subset of women (105,106). This high-risk group includes many genetic mutations (eg, *BRCA1, BRCA2, PALPB2, TP53, PTEN, CHECK2, CDH1, ATM,* and *STK11*). Most of the literature in the setting of high-risk screening focuses on *BRCA1/2* carriers, for whom sensitivities between 75.2% and 100% and specificities between 83% and 98.4% are reported (108,109). The cancer detection rate among known *BRCA1/2* carriers was 26.2 per 1000, compared with 5.4 per 1000 in high-risk non-mutation carriers (110). Annual screening MRI is also recommended in women who underwent chest irradiation before the age of 30 years, usually for treatment of lymphoma. Study results demonstrated higher sensitivity of both mammography and MRI—from 94% to 100%, with additional cancer detection rates of 4.1% using combined MRI and mammography (111,112). Of note, recent studies show virtually no incremental cancer detection rate of mammography in all high-risk individuals younger than age 40 years, if screening MRI is also performed (113,114). Also, the cancers detected with MRI are both invasive carcinomas and DCIS, while the cancers detected solely with mammography are mainly DCIS. This is clinically significant because women at

Table 3: Evaluation of Target and Non-Target Lesions according to RECIST, Version 1.1

Type of Lesion and Pathologic Response	Criteria
Target lesions	
Complete response (CR)	Disappearance of all target lesions. Any pathologic lymph nodes (when target or non-target) must have reduction in short axis to < 10 mm
Partial response (PR)	At least a 30% decrease in the sum of diameters of target lesions, taking as reference the baseline sum diameters
Progressive disease (PD)	At least a 20% increase in the sum of diameters of target lesions, taking as reference the smallest sum on study (this includes the baseline sum if that is the smallest on study). In addition to the relative increase of 20%, the sum must also demonstrate an absolute increase of at least 5 mm. (Note: The appearance of one or more new lesions is also considered progression)
Stable disease (SD)	Neither sufficient shrinkage to qualify for PR nor sufficient increase to qualify for PD, taking as reference the smallest sum diameters while on study
Non-target lesions	
Complete response (CR)	Disappearance of all non-target lesions. All lymph nodes < 10 mm in short axis
Non-CR/non-PD	Persistence of one or more nontarget lesion(s)
Progressive disease (PD)	Unequivocal progression of existing nontarget lesions. Appearance of one or more new lesions

Note.—RECIST = Response Evaluation Criteria in Solid Tumors.

increased risk, particularly *BRCA1* mutation carriers, are susceptible to the effects of radiation, and there is increasing evidence to reconsider the role of mammography if a concurrent breast MRI examination is also being performed (115,116).

Intermediate-risk women.—The American Cancer Society guidelines consider women to be at an intermediate risk of breast cancer if they have a lifetime risk of between 15% and 20%, a personal history of breast cancer, dense breasts at mammography, or a history of high-risk lesions at biopsy (specifically, atypical ductal hyperplasia, atypical lobular hyperplasia, and lobular carcinoma in situ) (105). These guidelines, published in 2007, concluded that there was insufficient evidence to recommend for or against screening. However, multiple studies have since been published that support the role of MRI, especially among women with a personal history of breast cancer. In this setting, the sensitivity of mammography for early detection of second breast cancers is lower because of overlying postoperative changes. Studies in patients with personal histories of breast cancer show a consistently high cancer detection rate with MRI, ranging from 10 to 29 cancers per 1000 (117,118). In fact, MRI performed better in this subset of women than in patients with genetic or family histories, with fewer false-positive findings, higher specificity, and equivalent sensitivity and cancer detection rates (118). As a result, the American College of Radiology now recommends annual breast MRI for women with dense breasts and a previous breast cancer diagnosis at an age younger than 50 years (106).

Although the American Cancer Society recommends against screening breast MRI in women at average risk (lifetime risk < 15%), Kuhl et al (119) reported detection of 22.6 additional cancers per 1000 screening examinations in women with negative mammography and US examinations in a prevalent round (105). In subsequent incident rounds, the cancer detection rate with MRI was 6.9 per 1000. Hence, the decision to recommend screening MRI only in high-risk women in whom there is a higher prevalence of breast cancer may need to be revisited.

The study demonstrates that there is a potentially larger number of women who may benefit from a screening breast MRI examination. It also highlights a strength of MRI: the low false-negative rates, as demonstrated by the low number of interval cancers (120,121). Also, because of improved spatial and temporal resolution and increased experience among radiologists, the number of false-positive findings has decreased. Lee et al (122) recently showed that community practices in the Breast Cancer Surveillance Consortium met and/or approached the American College of Radiology BI-RADS performance benchmarks for screening MRI.

Abbreviated MRI.—High costs and a limited availability of MRI units are the main factors that preclude the widespread use of screening MRI. Abbreviated MRI, with shorter image acquisition and interpretation times, may increase the availability of breast MRI and reduce the costs. Kuhl et al (23) introduced the concept of an abbreviated protocol that consisted of one pre- and one postcontrast T1-weighted acquisition and found equivalent diagnostic accuracy for the abbreviated and full protocols among 443 women and 606 MRI studies. A recent review of 21 studies (24) on abbreviated breast MRI performed in eight countries and in more than 4500 women confirmed the diagnostic accuracy was similar to the full MRI protocol. These studies evaluated a variety of abbreviated protocols. Also, ultrafast sequences may be used to obtain dynamic information without lengthening the protocol, maintaining a high diagnostic accuracy (123). Although breast MRI without intravenous contrast material administration would be useful for screening, the current techniques, particularly DWI, are not sensitive enough to replace contrast-enhanced breast MRI (124,125).

Overall, it is important to offer breast MRI to those women who benefit most. Risk prediction models may incorporate additional imaging biomarkers, such as mammographic density and background parenchymal enhancement at MRI (126,127). Single nucleotide polymorphisms (SNPs) in saliva specimens are

promising genetic markers that may also improve risk prediction models (128). Personalized screening, with the assessment of multiple risk factors and the use of "big data" analytics, is being proposed as new paradigm to allow for more nuanced stratification of breast cancer risk.

Evaluation of Women Treated with Neoadjuvant Chemotherapy

One of the major benefits of neoadjuvant chemotherapy (NAC) is achieving pathologic complete response (pCR) in women with human epidermal growth factor receptor 2 (HER2)-enriched cancer, triple-negative cancer, and many luminal-B cancers. Also, NAC may decrease the size of locally advanced breast tumors, increasing the chance for BCS, and result in downstaging of the axilla, possibly avoiding axillary lymph node dissection. Although achieving pCR is a favorable prognostic factor, survival outcomes of patients treated with preoperative chemotherapy are similar to those of patients treated with adjuvant chemotherapy (129,130). To evaluate residual tumor size, physical examination, mammography, US, and MRI have been used. Of these, MRI is the most accurate method, as it is difficult for other modalities to distinguish posttreatment fibrosis or postbiopsy change from residual tumor following NAC (131,132). However, because findings of breast cancers following NAC vary depending on tumor subtype, histologic type, and time points of MRI acquisitions, a refined strategy for accurate interpretation is crucial. The purpose of the MRI examination should also be understood. From an oncologist's perspective, assessing response to a specific regimen and measuring changes in invasive tumor size is important. Residual DCIS might not be the oncologist's concern. For a surgeon, to achieve a negative margin during BCS, tumor extent, including DCIS, should be measured. Thus, separate strategies might be needed to determine response to a specific chemotherapy regimen and to determine residual tumor size after NAC.

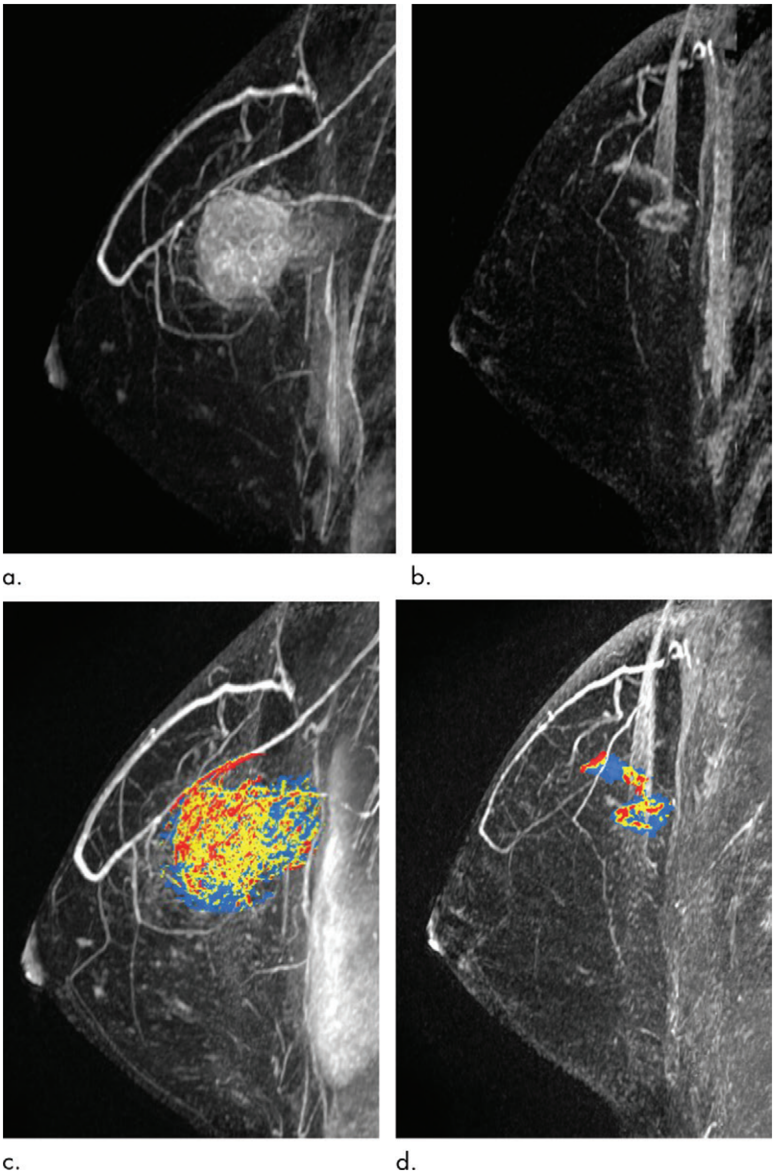

Figure 7: Sagittal images from breast MRI in a 31-year-old woman with an invasive ductal carcinoma. **(a)** Pre-chemotherapy maximal intensity projection (MIP) image from contrast-enhanced MRI shows a 4.9-cm round mass. **(b)** Post-chemotherapy MIP image from contrast-enhanced MRI shows a 1.7-cm irregular mass, and [(4.9 − 1.7)/4.9] · 100% = 65% reduction of the initial tumor diameter. It is suggestive of partial response. **(c, d)** Computer-aided volumetry images show 94% volume reduction, from **(c)** 26.5 cm^3 to **(d)** 1.6 cm^3.

Tumor response assessment during chemotherapy.—To assess response to chemotherapy, changes in maximum tumor size, tumor volume, and enhancement kinetics at imaging have been investigated (133), and functional techniques, including various DWI approaches and molecular imaging techniques, are being investigated. The Response Evaluation Criteria in Solid Tumors. or RECIST, are the most widely used standardized criteria for response assessment (134). Four categories of response—complete response (Fig 5), partial response (Fig 6), stable disease, and progressive disease—are recognized (Table 3).

Semiautomated volumetric measurements are available with three-dimensional software for MRI (Fig 7). Tumor volume changes have a stronger association with recurrence-free survival than other prognostic indicators, such as tumor diameter change (135). Furthermore, volume change showed the strongest predictive performance for pCR after the first cycle of chemotherapy. Modification of an ineffective chemotherapeutic regimen at an early time point when MRI indicates absence of response is important to avoid the unnecessary toxicity of chemotherapeutic agents but is currently mostly restricted to adaptive clinical trials. Changes in time–signal intensity curve analysis or pharmacokinetic modeling are also associated with response to NAC, with an early decrease in enhancement as an important predictor of eventual response (136,137). Likewise, an increase in ADC is predictive of response (138,139), with a reported sensitivity and specificity of 88% and 79% for the prediction of pCR (139). However,

to implement volumetric or functional assessment as alternatives to conventional size measurements, standardization of image acquisition, wide availability of postprocessing software, and further studies are needed.

Presurgical assessment after NAC to assess the potential of BCS.—BCS following NAC is considered safe because no significant difference has been found in local recurrence rates between a BCS group and a mastectomy group or between a downstaged BCS group and a preplanned BCS group (140,141). An age of younger than 40 years is associated with a higher rate of local recurrence (142). MRI can guide BCS following NAC, although it has not been proven that preoperative MRI is associated with improved surgical outcomes or recurrence rates (143).

For the detection of residual cancer after NAC, a meta-analysis including 44 studies (131) found that the median sensitivity was 92% and the median specificity was 90%. Although MRI has a better correlation with pathologic tumor size after NAC than physical examination, mammography, or US (131,132), underestimation or overestimation can occur because of scattered microscopic foci of residual tumor or fibrosis (144). Tumor size evaluation after NAC is therefore somewhat less reliable than that before NAC.

Size estimations in triple-negative tumors or HER2-positive tumors have been shown to be more accurate compared with those in hormone receptor–positive/HER2-negative tumors in the evaluation of residual tumor and the prediction of pCR (144,145) (Fig 8). The size of lobular or hormone receptor–positive/HER2-negative tumors tends to be underestimated (146) (Fig 9). These results can be explained by the fact that hormone receptor–positive/HER2-negative tumors more frequently manifest as diffuse NME and reduce into multiple small foci following chemotherapy (147). The presence of NME at preoperative MRI is also associated with worse local-regional recurrence-free survival in patients who underwent BCS after NAC (142), with recurrences usually manifesting in the same quadrant as the original tumor.

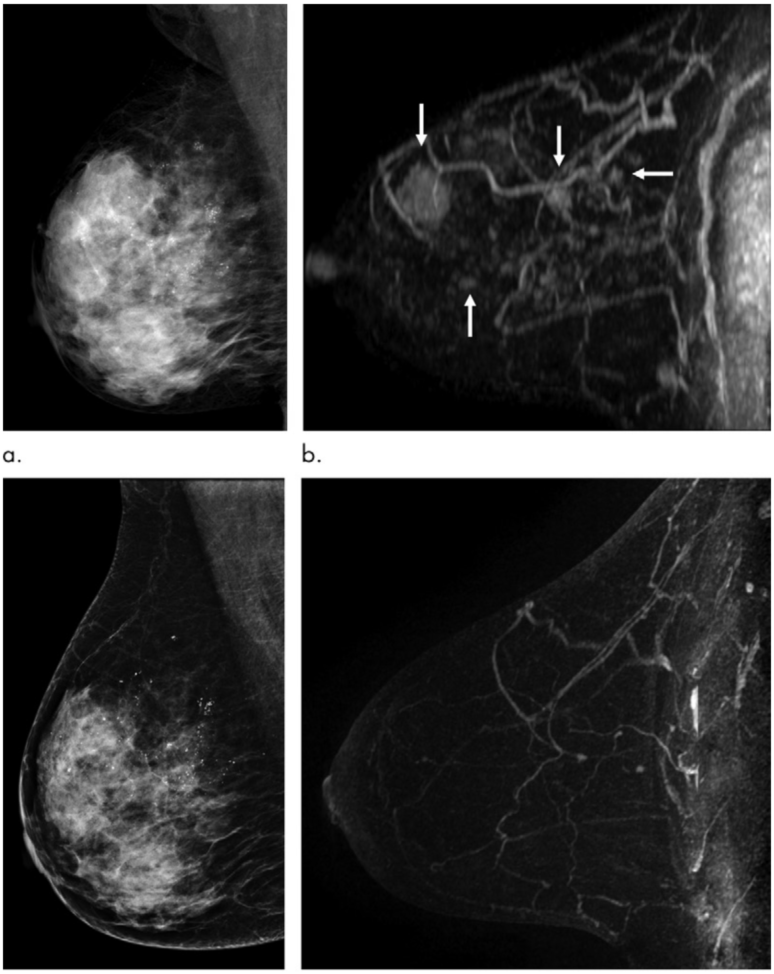

a. b. c. d.

Figure 8: Images in 35-year-old woman with an invasive ductal carcinoma of the human epidermal growth factor receptor 2–positive/hormone receptor–negative tumor subtype. **(a)** Pre-chemotherapy mammogram in the mediolateral oblique view shows diffuse, multifocal masses with calcifications in the upper breast. **(b)** Image from pre-chemotherapy MRI (maximum intensity projection of early postcontrast series in the sagittal plane) shows multiple masses with areas of non-mass enhancement (arrows) in the upper breast. Although **(c)** post-chemotherapy mammogram in the mediolateral oblique view shows residual regional calcifications, **(d)** image from post-chemotherapy MRI (maximum intensity projection of early postcontrast series in the sagittal plane) shows no enhancing lesion. Surgical histopathologic examination revealed a pathologic complete response. MRI was more accurate than mammography in the evaluation of residual tumor extent after chemotherapy.

Triple-negative tumors more often manifest as unifocal or multifocal masses than as areas of NME and often show a shrinking reduction pattern after chemotherapy (147) (Fig 6). Therefore, tumor size measurement after NAC on MRI is more challenging for hormone receptor–positive/HER2-negative tumors than for other tumor subtypes, and a tailored interpretation strategy is needed that takes tumor subtype and MRI phenotype into account.

To better determine the surgical tumor size after NAC, it may be beneficial to evaluate delayed phase images (obtained 6 minutes after contrast material administration), as these better show residual DCIS components (146). Evaluation of the invasive component, as well as determination of pCR, is more accurate with the standard postcontrast series (Fig 10, Table 4). These results can be explained by the finding that enhancement of residual DCIS after NAC tends to be delayed as a result of the antiangiogenic effect of chemotherapy (148). Consequently, for this indication, inflow dynamics cannot completely replace late phase information.

Presurgical assessment for prediction of pCR.—With the advent of targeted therapy, the rates of pCR have been markedly increased, up to 50%–60%, especially for HER2-positive and triple-negative breast cancers (149). Consequently, there is a growing interest to evaluate whether the omission of surgery might be feasible in patients in whom imaging findings indicate

that pCR is achieved (150,151). MRI has consistently shown high sensitivities (accurate identification of residual cancer) of 83%–92% and intermediate specificities (accurate identification of pCR) of 47%–63% in the prediction of pCR (131,132). Absence of enhancement in the tumor bed at visual assessment is the most commonly used imaging criterion for pCR. It has been reported that the absence of enhancement on images from delayed phase MRI increases the probability of pCR 28 times when compared with the presence of residual enhancement (148). Current trials selecting patients for percutaneous biopsy to omit surgery generally use absence of residual enhancement for patient selection. Preliminary results show that percutaneous biopsy increases the negative predictive value of MRI in the diagnosis of pCR from 76.7% to 94.4% (151), thus rendering this approach potentially feasible. However, chemotherapy-induced fibrosis, inflammation, or granulation tissue even without residual cancer might still lead to enhancement at the tumor site, mimicking residual cancer. Quantification of residual enhancement may yield a higher sensitivity for pCR at the cost of a reduction in specificity (152).

Other Indications for Breast MRI

Carcinoma of unknown primary origin.—In the scenario of an axillary carcinoma of unknown primary origin with suspicion of origin in the breast, treatment of the affected breast is important because local recurrence decreases and overall survival increases (153). MRI depicts the primary cancer in the breast in up to 60% of cases and allows planning of appropriate treatment. Importantly, when the MRI findings are

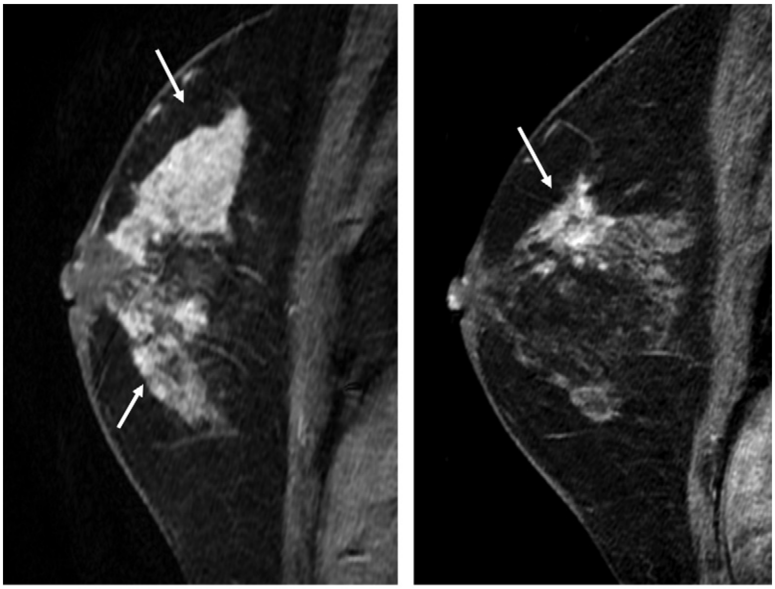

a. b.

Figure 9: Images from contrast-enhanced breast MRI in a 48-year-old woman with an invasive ductal carcinoma of the hormone receptor–positive/human epidermal growth factor receptor 2–negative subtype. **(a)** Image from pre-chemotherapy MRI shows multiple, irregular areas of non-mass enhancement (NME) (arrows) in the upper and lower breast. **(b)** On image obtained after completion of six cycles of chemotherapy, the areas of NME have decreased in size. The residual tumor in the upper breast was measured as 5.3 cm (arrow). The lower area of NME was considered to have resolved. However, surgical histopathologic examination revealed a 12.1-cm invasive ductal carcinoma in the upper and lower breast. Residual tumor size was therefore strongly underestimated at post-chemotherapy MRI.

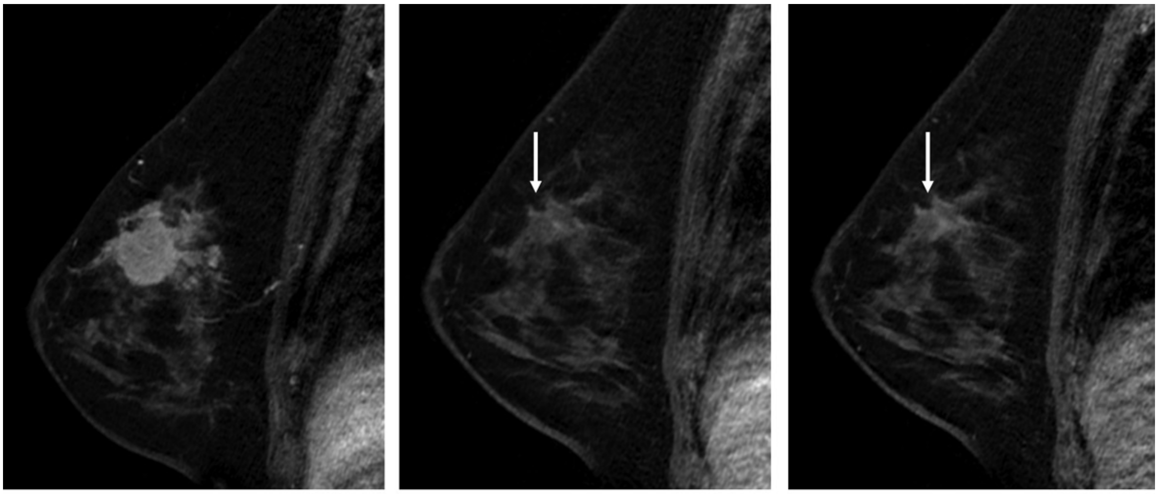

a. b. c.

Figure 10: Images from contrast-enhanced breast MRI in a 46-year-old woman with an invasive ductal carcinoma of the human epidermal growth factor receptor 2–positive subtype. **(a)** Image from pre-chemotherapy MRI shows a 5.5-cm irregular enhancing mass in upper breast. **(b)** Image obtained after completion of six cycles of taxane and herceptin in the early phase (90 seconds) of postcontrast MRI shows an architectural distortion without a definite enhancing lesion in the upper breast (arrow). **(c)** On image obtained in the delayed phase (360 seconds) of postcontrast MRI, the enhancing lesion was measured as 2.6 cm (arrow). Surgical histopathologic examination revealed a 0.5-cm invasive cancer and a 2.2-cm total tumor size (including both invasive tumor and ductal carcinoma in situ).

Table 4: Application of Post-Chemotherapy Breast MRI according to the Phases of Dynamic Contrast-enhanced MRI and Histopathologic Findings

Application	Early Phase of Dynamic Contrast-enhanced MRI	Delayed Phase of Dynamic Contrast-enhanced MRI
Treatment planning	Response monitoring following chemotherapy; prediction of pathologic complete response	Evaluation of candidacy for breast-conserving surgery (residual tumor extent)
Histopathology	Invasive tumor size alone	Size of invasive tumor with in situ tumor
Breast cancer subtype	Residual tumor extent for triple-negative tumor, HER2-positive/hormone receptor–negative tumor	Residual tumor extent for invasive lobular cancer, non-mass enhancement at MRI, hormone receptor–positive/HER2–negative tumor

Note.—HER2 = human epidermal growth factor receptor 2.

negative, radiation therapy to the ipsilateral breast is as safe as mastectomy, and therefore MRI in this setting may prevent unnecessary mastectomies (154,155).

Problem solving.—Problem solving relies on the high negative predictive value (NPV) of breast MRI. It is typically used for findings that are not certainly benign but that cannot be sampled for biopsy by using conventional imaging guidance. The most common example are mammographic asymmetries that are visible only in one view where a negative MRI effectively rules out the presence of cancer (156,157). Equivocal findings from breast tomosynthesis can also be evaluated in this fashion (158). In a meta-analysis, a sensitivity of 99% with an NPV of 100% was reported for the evaluation of noncalcified equivocal findings (159). In mammographically detected calcified lesions, the NPV of breast MRI is not high enough to exclude malignancy. In meta-analysis, the absence of enhancement at the site of calcifications is associated with an NPV of 93% (160), although the presence of invasive cancer is unlikely in this setting. For nipple discharge, MRI outperforms galactography, with a sensitivity for causative lesions of 92% versus 69%. In these patients, cancers are detected with an equally high sensitivity and a high specificity of 97% (161).

Discussion

Technical developments have improved the quality of breast MRI, allowing the acquisition of isotropic high-resolution images. In addition, multiparametric breast MRI has largely replaced the conventional approach, which was primarily based on conventional contrast-enhanced sequences alone for lesion classification. Multiparametric evaluation of lesions allows for a positive predictive value of biopsy that is comparable to that for mammography. Other techniques currently being investigated may enable a more quantitative approach to MRI evaluation, further improving reproducibility and consistency over clinical sites. Indications for breast MRI are consolidating. Although there is concern about the long-term deposition of gadolinium in patients undergoing an annual screening MRI examination, this examination leads to earlier cancer detection in virtually all evaluated populations at high sensitivity and with very low interval cancer rates. The supplemental value of mammography is, especially in younger *BRCA* mutation carriers, now questionable (113,114). In the hands of experienced teams, MRI allows for improvement of surgical practice, reducing re-excisions while preventing unnecessary mastectomies. Likewise, MRI enables patient selection to neoadjuvant chemotherapy and is the modality of choice for modification of therapeutic agents, for presurgical assessment of residual tumor size to determine breast-conserving surgery candidacy, and for prediction of pathologic complete response to triage patients to clinical trials omitting surgery. From an economic perspective, and to improve patient comfort, breast MRI can be optimized by adjusting the protocol with regard to the indication. For indications where the exclusion of disease is most important, abbreviated protocols may be used. On the other hand, when lesions need to be characterized in detail, or when the frequency of findings is high, multiparametric protocols are mandatory. Because therapy may reduce enhancement in breast lesions, during and after chemotherapy, evaluation of late phase enhancement may still be important.

Disclosures of Conflicts of Interest: **R.M.M.** Activities related to the present article: disclosed no relevant relationships. Activities not related to the present article: is a consultant for Transonic Imaging; institution has grants or grants pending from Siemens Healthineers, Bayer Healthcare, Medtronic, Screenpoint Medical, Seno Medical, Identification Solutions, and Micrima. Other relationships: disclosed no relevant relationships. **N.C.** disclosed no relevant relationships. **L.M.** Activities related to the present article: disclosed no relevant relationships. Activities not related to the present article: has received personal fees for consulting from Lunit; institution has a research grant or grant pending with Siemens. Other relationships: disclosed no relevant relationships.

References

1. Mann RM, Kuhl CK, Kinkel K, Boetes C. Breast MRI: guidelines from the European Society of Breast Imaging. Eur Radiol 2008;18(7):1307–1318.
2. Sardanelli F, Boetes C, Borisch B, et al. Magnetic resonance imaging of the breast: recommendations from the EUSOMA working group. Eur J Cancer 2010;46(8):1296–1316.
3. Newell MS, Giess CS, Argus AD, et al. ACR practice parameter for the performance of contrastenhanced magnetic resonance imaging (MRI) of the breast. Reston, Va: American College of Radiology, 2018.
4. Heywang SH, Hahn D, Schmidt H, et al. MR imaging of the breast using gadolinium-DTPA. J Comput Assist Tomogr 1986;10(2):199–204.
5. Kaiser WA, Zeitler E. MR imaging of the breast: fast imaging sequences with and without Gd-DTPA. Preliminary observations. Radiology 1989;170(3 Pt 1):681–686.
6. Knopp MV, Weiss E, Sinn HP, et al. Pathophysiologic basis of contrast enhancement in breast tumors. J Magn Reson Imaging 1999;10(3):260–266.
7. Carmeliet P, Jain RK. Angiogenesis in cancer and other diseases. Nature 2000;407(6801):249–257.
8. Marino MA, Helbich T, Baltzer P, Pinker-Domenig K. Multiparametric MRI of the breast: a review. J Magn Reson Imaging 2018;47(2):301–315.
9. Rahbar H, Partridge SC. Multiparametric MR imaging of breast cancer. Magn Reson Imaging Clin N Am 2016;24(1):223–238.
10. Warner E, Causer PA, Wong JW, et al. Improvement in DCIS detection rates by MRI over time in a high-risk breast screening study. Breast J 2011;17(1):9–17.
11. Konyer NB, Ramsay EA, Bronskill MJ, Plewes DB. Comparison of MR imaging breast coils. Radiology 2002;222(3):830–834.

12. Yeh ED, Georgian-Smith D, Raza S, Bussolari L, Pawlisz-Hoff J, Birdwell RL. Positioning in breast MR imaging to optimize image quality. RadioGraphics 2014;34(1):E1–E17.
13. Marshall H, Devine PM, Shanmugaratnam N, et al. Evaluation of multicoil breast arrays for parallel imaging. J Magn Reson Imaging 2010;31(2):328–338.
14. Hancu I, Fiveland E, Park K, Giaquinto RO, Rohling K, Wiesinger F. Flexible, 31-channel breast coil for enhanced parallel imaging performance at 3T. Magn Reson Med 2016;75(2):897–905.
15. Nnewihe AN, Grafendorfer T, Daniel BL, et al. Custom-fitted 16-channel bilateral breast coil for bidirectional parallel imaging. Magn Reson Med 2011;66(1):281–289.
16. Heywang-Köbrunner SH, Sinnatamby R, Lebeau A, et al. Interdisciplinary consensus on the uses and technique of MR-guided vacuum-assisted breast biopsy (VAB): results of a European consensus meeting. Eur J Radiol 2009;72(2):289–294.
17. Ferré R, Ianculescu V, Ciolovan L, et al. Diagnostic performance of MR-guided vacuum-assisted breast biopsy: 8 years of experience. Breast J 2016;22(1):83–89.
18. Pediconi F, Catalano C, Padula S, et al. Contrast-enhanced MR mammography: improved lesion detection and differentiation with gadobenate dimeglumine. AJR Am J Roentgenol 2008;191(5):1339–1346.
19. Carbonaro LA, Pediconi F, Verardi N, Trimboli RM, Calabrese M, Sardanelli F. Breast MRI using a high-relaxivity contrast agent: an overview. AJR Am J Roentgenol 2011;196(4):942–955.
20. Flanagan FL, Murray JG, Gilligan P, Stack JP, Ennis JT. Digital subtraction in Gd-DTPA enhanced imaging of the breast. Clin Radiol 1995;50(12):848–854.
21. Cheung HS, Tse GM, Lai SY, Yeung DK. Relationship between lesion size and signal enhancement on subtraction fat-suppressed MR imaging of the breast. Magn Reson Imaging 2004;22(9):1259–1264.
22. Mango VL, Morris EA, David Dershaw D, et al. Abbreviated protocol for breast MRI: are multiple sequences needed for cancer detection? Eur J Radiol 2015;84(1):65–70.
23. Kuhl CK, Schrading S, Strobel K, Schild HH, Hilgers RD, Bieling HB. Abbreviated breast magnetic resonance imaging (MRI): first postcontrast subtracted images and maximum-intensity projection-a novel approach to breast cancer screening with MRI. J Clin Oncol 2014;32(22):2304–2310.
24. Leithner D, Moy L, Morris EA, Marino MA, Helbich TH, Pinker K. Abbreviated MRI of the breast: does it provide value? J Magn Reson Imaging 2018 Sep 8 [Epub ahead of print].
25. Kuhl CK, Mielcareck P, Klaschik S, et al. Dynamic breast MR imaging: are signal intensity time course data useful for differential diagnosis of enhancing lesions? Radiology 1999;211(1):101–110.
26. Daniel BL, Yen YF, Glover GH, et al. Breast disease: dynamic spiral MR imaging. Radiology 1998;209(2):499–509.
27. Partridge SC, Stone KM, Strigel RM, DeMartini WB, Peacock S, Lehman CD. Breast DCE-MRI: influence of postcontrast timing on automated lesion kinetics assessments and discrimination of benign and malignant lesions. Acad Radiol 2014;21(9):1195–1203.
28. Abe H, Mori N, Tsuchiya K, et al. Kinetic analysis of benign and malignant breast lesions with ultrafast dynamic contrast-enhanced MRI: comparison with standard kinetic assessment. AJR Am J Roentgenol 2016;207(5):1159–1166.
29. Mann RM, Mus RD, van Zelst J, Geppert C, Karssemeijer N, Platel B. A novel approach to contrast-enhanced breast magnetic resonance imaging for screening: high-resolution ultrafast dynamic imaging. Invest Radiol 2014;49(9):579–585.
30. Boetes C, Barentsz JO, Mus RD, et al. MR characterization of suspicious breast lesions with a gadolinium-enhanced TurboFLASH subtraction technique. Radiology 1994;193(3):777–781.
31. Mus RD, Borelli C, Bult P, et al. Time to enhancement derived from ultrafast breast MRI as a novel parameter to discriminate benign from malignant breast lesions. Eur J Radiol 2017;89:90–96.
32. Onishi N, Kataoka M, Kanao S, et al. Ultrafast dynamic contrast-enhanced MRI of the breast using compressed sensing: breast cancer diagnosis based on separate visualization of breast arteries and veins. J Magn Reson Imaging 2018;47(1):97–104.
33. Heacock L, Gao Y, Heller SL, et al. Comparison of conventional DCE-MRI and a novel golden-angle radial multicoil compressed sensing method for the evaluation of breast lesion conspicuity. J Magn Reson Imaging 2017;45(6):1746–1752.
34. Vreemann S, Rodriguez-Ruiz A, Nickel D, et al. Compressed sensing for breast MRI: resolving the trade-off between spatial and temporal resolution. Invest Radiol 2017;52(10):574–582.
35. Kuhl CK, Klaschik S, Mielcarek P, Gieseke J, Wardelmann E, Schild HH. Do T2-weighted pulse sequences help with the differential diagnosis of enhancing lesions in dynamic breast MRI? J Magn Reson Imaging 1999;9(2):187–196.
36. Westra C, Dialani V, Mehta TS, Eisenberg RL. Using T2-weighted sequences to more accurately characterize breast masses seen on MRI. AJR Am J Roentgenol 2014;202(3):W183–W190.
37. Cheon H, Kim HJ, Kim TH, et al. Invasive breast cancer: prognostic value of peritumoral edema identified at preoperative MR imaging. Radiology 2018;287(1):68–75.
38. Uematsu T, Kasami M, Watanabe J. Can T2-weighted 3-T breast MRI predict clinically occult inflammatory breast cancer before pathological examination? a single-center experience. Breast Cancer 2014;21(1):115–121.
39. Ballesio L, Savelli S, Angeletti M, et al. Breast MRI: Are T2 IR sequences useful in the evaluation of breast lesions? Eur J Radiol 2009;71(1):96–101.
40. Arponen O, Masarwah A, Sutela A, et al. Incidentally detected enhancing lesions found in breast MRI: analysis of apparent diffusion coefficient and T2 signal intensity significantly improves specificity. Eur Radiol 2016;26(12):4361–4370.
41. Le Bihan D, Iima M. Diffusion magnetic resonance imaging: what water tells us about biological tissues. PLoS Biol 2015;13(7):e1002203 [Published correction appears in PLoS Biol 2015;13(9):e1002246.].
42. Partridge SC, McDonald ES. Diffusion weighted magnetic resonance imaging of the breast: protocol optimization, interpretation, and clinical applications. Magn Reson Imaging Clin N Am 2013;21(3):601–624.
43. Shi RY, Yao QY, Wu LM, Xu JR. Breast lesions: diagnosis using diffusion weighted imaging at 1.5T and 3.0T–systematic review and meta-analysis. Clin Breast Cancer 2018;18(3):e305–e320.
44. Peters NH, Vincken KL, van den Bosch MA, Luijten PR, Mali WP, Bartels LW. Quantitative diffusion weighted imaging for differentiation of benign and malignant breast lesions: the influence of the choice of b-values. J Magn Reson Imaging 2010;31(5):1100–1105.
45. Dorrius MD, Dijkstra H, Oudkerk M, Sijens PE. Effect of b value and pre-admission of contrast on diagnostic accuracy of 1.5-T breast DWI: a systematic review and meta-analysis. Eur Radiol 2014;24(11):2835–2847.
46. Tamura T, Murakami S, Naito K, Yamada T, Fujimoto T, Kikkawa T. Investigation of the optimal b-value to detect breast tumors with diffusion weighted imaging by 1.5-T MRI. Cancer Imaging 2014;14:11.
47. Pereira FP, Martins G, Figueiredo E, et al. Assessment of breast lesions with diffusion-weighted MRI: comparing the use of different b values. AJR Am J Roentgenol 2009;193(4):1030–1035.
48. Bogner W, Gruber S, Pinker K, et al. Diffusion-weighted MR for differentiation of breast lesions at 3.0 T: how does selection of diffusion protocols affect diagnosis? Radiology 2009;253(2):341–351.
49. Partridge SC, Singer L, Sun R, et al. Diffusion-weighted MRI: influence of intravoxel fat signal and breast density on breast tumor conspicuity and apparent diffusion coefficient measurements. Magn Reson Imaging 2011;29(9):1215–1221.
50. Baron P, Dorrius MD, Kappert P, Oudkerk M, Sijens PE. Diffusion-weighted imaging of normal fibroglandular breast tissue: influence of microperfusion and fat suppression technique on the apparent diffusion coefficient. NMR Biomed 2010;23(4):399–405.
51. Wenkel E, Geppert C, Schulz-Wendtland R, et al. Diffusion weighted imaging in breast MRI: comparison of two different pulse sequences. Acad Radiol 2007;14(9):1077–1083.
52. Partridge SC, Nissan N, Rahbar H, Kitsch AE, Sigmund EE. Diffusion-weighted breast MRI: clinical applications and emerging techniques. J Magn Reson Imaging 2017;45(2):337–355.
53. Kim YJ, Kim SH, Kang BJ, et al. Readout-segmented echo-planar imaging in diffusion-weighted mr imaging in breast cancer: comparison with single-shot echo-planar imaging in image quality. Korean J Radiol 2014;15(4):403–410.
54. Bogner W, Pinker-Domenig K, Bickel H, et al. Readout-segmented echo-planar imaging improves the diagnostic performance of diffusion-weighted MR breast examinations at 3.0 T. Radiology 2012;263(1):64–76.
55. Park JY, Shin HJ, Shin KC, et al. Comparison of readout segmented echo planar imaging (EPI) and EPI with reduced field-of-view diffusion-weighted imaging at 3T in patients with breast cancer. J Magn Reson Imaging 2015;42(6):1679–1688.
56. American College of Radiology. BI-RADS atlas. 5th ed. Reston, Va: American College of Radiology, 2013.
57. King V, Brooks JD, Bernstein JL, Reiner AS, Pike MC, Morris EA. Background parenchymal enhancement at breast MR imaging and breast cancer risk. Radiology 2011;260(1):50–60.
58. Dontchos BN, Rahbar H, Partridge SC, et al. Are qualitative assessments of background parenchymal enhancement, amount of fibroglandular tissue on MR images, and mammographic density associated with breast cancer risk? Radiology 2015;276(2):371–380.
59. Ray KM, Kerlikowske K, Lobach IV, et al. Effect of background parenchymal enhancement on breast MR imaging interpretive performance in community-based practices. Radiology 2018;286(3):822–829.
60. Jansen SA, Shimauchi A, Zak L, Fan X, Karczmar GS, Newstead GM. The diverse pathology and kinetics of mass, nonmass, and focus enhancement on MR imaging of the breast. J Magn Reson Imaging 2011;33(6):1382–1389.

61. Gutierrez RL, DeMartini WB, Eby PR, Kurland BF, Peacock S, Lehman CD. BI-RADS lesion characteristics predict likelihood of malignancy in breast MRI for masses but not for nonmasslike enhancement. AJR Am J Roentgenol 2009;193(4):994–1000.
62. Kawai M, Kataoka M, Kanao S, et al. The value of lesion size as an adjunct to the BI-RADS-MRI 2013 descriptors in the diagnosis of solitary breast masses. Magn Reson Med Sci 2018;17(3):203–210.
63. Baltzer PA, Benndorf M, Dietzel M, Gajda M, Runnebaum IB, Kaiser WA. False-positive findings at contrast-enhanced breast MRI: a BI-RADS descriptor study. AJR Am J Roentgenol 2010;194(6):1658–1663.
64. Chikarmane SA, Michaels AY, Giess CS. Revisiting nonmass enhancement in breast MRI: analysis of outcomes and follow-up using the updated BI-RADS atlas. AJR Am J Roentgenol 2017;209(5):1178–1184.
65. Machida Y, Shimauchi A, Kuroki Y, et al. Single focus on breast magnetic resonance imaging: diagnosis based on kinetic pattern and patient age. Acta Radiol 2017;58(6):652–659.
66. Baltzer PA, Dietzel M, Kaiser WA. A simple and robust classification tree for differentiation between benign and malignant lesions in MR-mammography. Eur Radiol 2013;23(8):2051–2060.
67. Woitek R, Spick C, Schernthaner M, et al. A simple classification system (the Tree flowchart) for breast MRI can reduce the number of unnecessary biopsies in MRI-only lesions. Eur Radiol 2017;27(9):3799–3809.
68. Pinker K, Helbich TH, Morris EA. The potential of multiparametric MRI of the breast. Br J Radiol 2017;90(1069):20160715.
69. National Comprehensive Cancer Network. Breast Cancer Screening and Diagnosis. https://www.nccn.org/professionals/physician_gls/pdf/breast-screening.pdf. Published 2018. Accessed DATE.
70. National Institute for Health and Care Excellence. Early and locally advanced breast cancer: diagnosis and management. NICE guideline [NG101]. https://www.nice.org.uk/guidance/ng101. Published 2018. Accessed DATE.
71. Federatie Medische Specialisten. Borstkanker - MRI bij PA-bevestigde borstkanker. https://richtlijnendatabase.nl/richtlijn/borstkanker/diagnostiek/preoperatieve_stadiering/mr.html. Published 2018. Accessed DATE.
72. Liedtke C, Jackisch C, Thill M, et al. AGO recommendations for the diagnosis and treatment of patients with early breast cancer: update 2018. Breast Care (Basel) 2018;13(3):196–208.
73. Houssami N, Abraham LA, Kerlikowske K, et al. Risk factors for second screen-detected or interval breast cancers in women with a personal history of breast cancer participating in mammography screening. Cancer Epidemiol Biomarkers Prev 2013;22(5):946–961.
74. Lee JM, Abraham L, Lam DL, et al. Cumulative risk distribution for interval invasive second breast cancers after negative surveillance mammography. J Clin Oncol 2018;36(20):2070–2077.
75. Yoo EY, Nam SY, Choi HY, Hong MJ. Agreement between MRI and pathologic analyses for determination of tumor size and correlation with immunohistochemical factors of invasive breast carcinoma. Acta Radiol 2018;59(1):50–57.
76. Haraldsdóttir KH, Jónsson Þ, Halldórsdóttir AB, Tranberg KG, Ásgeirsson KS. Tumor size of invasive breast cancer on magnetic resonance imaging and conventional imaging (mammogram/ultrasound): comparison with pathological size and clinical implications. Scand J Surg 2017;106(1):68–73.
77. Rominger M, Berg D, Frauenfelder T, Ramaswamy A, Timmesfeld N. Which factors influence MRI-pathology concordance of tumour size measurements in breast cancer? Eur Radiol 2016;26(5):1457–1465.
78. Parvaiz MA, Yang P, Razia E, et al. Breast MRI in invasive lobular carcinoma: a useful investigation in surgical planning? Breast J 2016;22(2):143–150.
79. Selvi V, Nori J, Meattini I, et al. Role of magnetic resonance imaging in the preoperative staging and work-up of patients affected by invasive lobular carcinoma or invasive ductolobular carcinoma. BioMed Res Int 2018;2018:1569060.
80. Mann RM, Veltman J, Barentsz JO, Wobbes T, Blickman JG, Boetes C. The value of MRI compared to mammography in the assessment of tumour extent in invasive lobular carcinoma of the breast. Eur J Surg Oncol 2008;34(2):135–142.
81. Schouten van der Velden AP, Boetes C, Bult P, Wobbes T. Magnetic resonance imaging in size assessment of invasive breast carcinoma with an extensive intraductal component. BMC Med Imaging 2009;9(1):5.
82. Kuhl CK, Strobel K, Bieling H, et al. Impact of preoperative breast MR imaging and MR-guided surgery on diagnosis and surgical outcome of women with invasive breast cancer with and without DCIS component. Radiology 2017;284(3):645–655.
83. Sung JS, Stamler S, Brooks J, et al. Breast cancers detected at screening MR imaging and mammography in patients at high risk: method of detection reflects tumor histopathologic results. Radiology 2016;280(3):716–722.
84. Kuhl CK, Schrading S, Bieling HB, et al. MRI for diagnosis of pure ductal carcinoma in situ: a prospective observational study. Lancet 2007;370(9586):485–492.
85. Plana MN, Carreira C, Muriel A, et al. Magnetic resonance imaging in the preoperative assessment of patients with primary breast cancer: systematic review of diagnostic accuracy and meta-analysis. Eur Radiol 2012;22(1):26–38.
86. Houssami N, Ciatto S, Macaskill P, et al. Accuracy and surgical impact of magnetic resonance imaging in breast cancer staging: systematic review and meta-analysis in detection of multifocal and multicentric cancer. J Clin Oncol 2008;26(19):3248–3258.
87. Spick C, Baltzer PA. Diagnostic utility of second-look US for breast lesions identified at MR imaging: systematic review and meta-analysis. Radiology 2014;273(2):401–409.
88. Houssami N, Turner RM, Morrow M. Meta-analysis of pre-operative magnetic resonance imaging (MRI) and surgical treatment for breast cancer. Breast Cancer Res Treat 2017;165(2):273–283.
89. Turnbull L, Brown S, Harvey I, et al. Comparative effectiveness of MRI in breast cancer (COMICE) trial: a randomised controlled trial. Lancet 2010;375(9714):563–571.
90. Gonzalez V, Sandelin K, Karlsson A, et al. Preoperative MRI of the breast (POMB) influences primary treatment in breast cancer: a prospective, randomized, multicenter study. World J Surg 2014;38(7):1685–1693.
91. Lobbes MB, Vriens IJ, van Bommel AC, et al. Breast MRI increases the number of mastectomies for ductal cancers, but decreases them for lobular cancers. Breast Cancer Res Treat 2017;162(2):353–364.
92. Sinclair K, Sakellariou S, Dawson N, Litherland J. Does preoperative breast MRI significantly impact on initial surgical procedure and re-operation rates in patients with screen-detected invasive lobular carcinoma? Clin Radiol 2016;71(6):543–550.
93. Mann RM, Loo CE, Wobbes T, et al. The impact of preoperative breast MRI on the re-excision rate in invasive lobular carcinoma of the breast. Breast Cancer Res Treat 2010;119(2):415–422.
94. Sardanelli F. Preoperative staging with MRI: did the MIPA trial solve all issues? Insights into Imaging - ECR 2018 - BOOK OF ABSTRACTS. 2018; 106.
95. Brennan ME, Houssami N, Lord S, et al. Magnetic resonance imaging screening of the contralateral breast in women with newly diagnosed breast cancer: systematic review and meta-analysis of incremental cancer detection and impact on surgical management. J Clin Oncol 2009;27(33):5640–5649.
96. Lehman CD, Gatsonis C, Kuhl CK, et al. MRI evaluation of the contralateral breast in women with recently diagnosed breast cancer. N Engl J Med 2007;356(13):1295–1303.
97. Houssami N, Turner R, Macaskill P, et al. An individual person data meta-analysis of preoperative magnetic resonance imaging and breast cancer recurrence. J Clin Oncol 2014;32(5):392–401.
98. Wang SY, Long JB, Killelea BK, et al. Associations of preoperative breast magnetic resonance imaging with subsequent mastectomy and breast cancer mortality. Breast Cancer Res Treat 2018;172(2):453–461.
99. Kim JY, Cho N, Koo HR, et al. Unilateral breast cancer: screening of contralateral breast by using preoperative MR imaging reduces incidence of metachronous cancer. Radiology 2013;267(1):57–66.
100. Robertson C, Arcot Ragupathy SK, Boachie C, et al. The clinical effectiveness and cost-effectiveness of different surveillance mammography regimens after the treatment for primary breast cancer: systematic reviews registry database analyses and economic evaluation. Health Technol Assess 2011;15(34):v–vi, 1–322.
101. Houssami N, Ciatto S, Martinelli F, Bonardi R, Duffy SW. Early detection of second breast cancers improves prognosis in breast cancer survivors. Ann Oncol 2009;20(9):1505–1510.
102. Hartman M, Czene K, Reilly M, et al. Incidence and prognosis of synchronous and metachronous bilateral breast cancer. J Clin Oncol 2007;25(27):4210–4216.
103. Hill MV, Beeman JL, Jhala K, Holubar SD, Rosenkranz KM, Barth RJ Jr. Relationship of breast MRI to recurrence rates in patients undergoing breast-conservation treatment. Breast Cancer Res Treat 2017;163(3):615–622.
104. Gervais MK, Maki E, Schiller DE, Crystal P, McCready DR. Preoperative MRI of the breast and ipsilateral breast tumor recurrence: long-term follow up. J Surg Oncol 2017;115(3):231–237.
105. Saslow D, Boetes C, Burke W, et al. American Cancer Society guidelines for breast screening with MRI as an adjunct to mammography. CA Cancer J Clin 2007;57(2):75–89.
106. Monticciolo DL, Newell MS, Moy L, Niell B, Monsees B, Sickles EA. Breast cancer screening in women at higher-than-average risk: recommendations from the ACR. J Am Coll Radiol 2018;15(3,3 Pt A):408–414.
107. Mann RM, Balleyguier C, Baltzer PA, et al. Breast MRI: EUSOBI recommendations for women's information. Eur Radiol 2015;25(12):3669–3678.
108. Warner E, Messersmith H, Causer P, Eisen A, Shumak R, Plewes D. Systematic review: using magnetic resonance imaging to screen women at high risk for breast cancer. Ann Intern Med 2008;148(9):671–679.
109. Evans DG, Harkness EF, Howell A, et al. Intensive breast screening in BRCA2 mutation carriers is associated with reduced breast cancer specific and all cause mortality. Hered Cancer Clin Pract 2016;14(1):8.
110. Kriege M, Brekelmans CT, Boetes C, et al. Efficacy of MRI and mammography for breast-cancer screening in women with a familial or genetic predisposition. N Engl J Med 2004;351(5):427–437.

111. Tieu MT, Cigsar C, Ahmed S, et al. Breast cancer detection among young survivors of pediatric Hodgkin lymphoma with screening magnetic resonance imaging. Cancer 2014;120(16):2507–2513.
112. Freitas V, Scaranelo A, Menezes R, Kulkarni S, Hodgson D, Crystal P. Added cancer yield of breast magnetic resonance imaging screening in women with a prior history of chest radiation therapy. Cancer 2013;119(3):495–503.
113. Phi XA, Saadatmand S, De Bock GH, et al. Contribution of mammography to MRI screening in BRCA mutation carriers by BRCA status and age: individual patient data meta-analysis. Br J Cancer 2016;114(6):631–637.
114. Vreemann S, van Zelst JCM, Schlooz-Vries M, et al. The added value of mammography in different age-groups of women with and without BRCA mutation screened with breast MRI. Breast Cancer Res 2018;20(1):84.
115. Obdeijn IM, Winter-Warnars GAO, Mann RM, Hooning MJ, Hunink MGM, Tilanus-Linthorst MMA. Should we screen BRCA1 mutation carriers only with MRI? a multicenter study. Breast Cancer Res Treat 2014;144(3):577–582.
116. Lo G, Scaranelo AM, Aboras H, et al. Evaluation of the utility of screening mammography for high-risk women undergoing screening breast MR imaging. Radiology 2017;285(1):36–43.
117. Gweon HM, Cho N, Han W, et al. Breast MR imaging screening in women with a history of breast conservation therapy. Radiology 2014;272(2):366–373.
118. Lehman CD, Lee JM, DeMartini WB, et al. Screening MRI in women with a personal history of breast cancer. J Natl Cancer Inst 2016;108(3):djv349.
119. Kuhl CK, Strobel K, Bieling H, Leutner C, Schild HH, Schrading S. Supplemental Breast MR imaging screening of women with average risk of breast cancer. radiology 2017;283(2):361–370.
120. Yamaguchi K, Schacht D, Newstead GM, et al. Breast cancer detected on an incident (second or subsequent) round of screening MRI: MRI features of false-negative cases. AJR Am J Roentgenol 2013;201(5):1155–1163.
121. Vreemann S, Gubern-Merida A, Lardenoije S, et al. The frequency of missed breast cancers in women participating in a high-risk MRI screening program. Breast Cancer Res Treat 2018;169(2):323–331.
122. Lee JM, Ichikawa L, Valencia E, et al. Performance benchmarks for screening breast MR imaging in community practice. Radiology 2017;285(1):44–52.
123. van Zelst JCM, Vreemann S, Witt HJ, et al. Multireader study on the diagnostic accuracy of ultrafast breast magnetic resonance imaging for breast cancer screening. Invest Radiol 2018;53(10):579–586.
124. Pinker K, Moy L, Sutton EJ, et al. Diffusion-weighted imaging with apparent diffusion coefficient mapping for breast cancer detection as a stand-alone parameter: comparison with dynamic contrast-enhanced and multiparametric magnetic resonance imaging. Invest Radiol 2018;53(10):587–595.
125. Baltzer PA, Benndorf M, Dietzel M, Gajda M, Camara O, Kaiser WA. Sensitivity and specificity of unenhanced MR mammography (DWI combined with T2-weighted TSE imaging, ueMRM) for the differentiation of mass lesions. Eur Radiol 2010;20(5):1101–1110.
126. Brentnall AR, Cuzick J, Buist DSM, Bowles EJA. Long-term accuracy of breast cancer risk assessment combining classic risk factors and breast density. JAMA Oncol 2018;4(9):e180174.
127. Kerlikowske K, Scott CG, Mahmoudzadeh AP, et al. Automated and clinical Breast Imaging Reporting and Data System density measures predict risk for screen-detected and interval cancers: a case-control study. Ann Intern Med 2018;168(11):757–765.
128. van Veen EM, Brentnall AR, Byers H, et al. Use of single-nucleotide polymorphisms and mammographic density plus classic risk factors for breast cancer risk prediction. JAMA Oncol 2018;4(4):476–482.
129. King TA, Morrow M. Surgical issues in patients with breast cancer receiving neoadjuvant chemotherapy. Nat Rev Clin Oncol 2015;12(6):335–343.
130. Cortazar P, Zhang L, Untch M, et al. Pathological complete response and long-term clinical benefit in breast cancer: the CTNeoBC pooled analysis. Lancet 2014;384(9938):164–172.
131. Marinovich ML, Houssami N, Macaskill P, et al. Meta-analysis of magnetic resonance imaging in detecting residual breast cancer after neoadjuvant therapy. J Natl Cancer Inst 2013;105(5):321–333.
132. Scheel JR, Kim E, Partridge SC, et al. MRI, clinical examination, and mammography for preoperative assessment of residual disease and pathologic complete response after neoadjuvant chemotherapy for breast cancer: ACRIN 6657 trial. AJR Am J Roentgenol 2018;210(6):1376–1385.
133. Le-Petross HT, Lim B. Role of MR imaging in neoadjuvant therapy monitoring. Magn Reson Imaging Clin N Am 2018;26(2):207–220.
134. Eisenhauer EA, Therasse P, Bogaerts J, et al. New response evaluation criteria in solid tumours: revised RECIST guideline (version 1.1). Eur J Cancer 2009;45(2):228–247.
135. Hylton NM, Gatsonis CA, Rosen MA, et al. Neoadjuvant chemotherapy for breast cancer: functional tumor volume by MR imaging predicts recurrence-free survival—results from the ACRIN 6657/CALGB 150007 I-SPY 1 TRIAL. Radiology 2016;279(1):44–55.
136. Ah-See ML, Makris A, Taylor NJ, et al. Early changes in functional dynamic magnetic resonance imaging predict for pathologic response to neoadjuvant chemotherapy in primary breast cancer. Clin Cancer Res 2008;14(20):6580–6589.
137. Dogan BE, Yuan Q, Bassett R, et al. Comparing the performances of magnetic resonance imaging size vs pharmacokinetic parameters to predict response to neoadjuvant chemotherapy and survival in patients with breast cancer. Curr Probl Diagn Radiol 2018 Mar 28 [Epub ahead of print].
138. Partridge SC, Zhang Z, Newitt DC, et al. Diffusion-weighted MRI findings predict pathologic response in neoadjuvant treatment of breast cancer: the ACRIN 6698 Multicenter Trial. Radiology 2018;289(3):618–627.
139. Chu W, Jin W, Liu D, et al. Diffusion-weighted imaging in identifying breast cancer pathological response to neoadjuvant chemotherapy: a meta-analysis. Oncotarget 2017;9(6):7088–7100.
140. Fisher B, Brown A, Mamounas E, et al. Effect of preoperative chemotherapy on local-regional disease in women with operable breast cancer: findings from National Surgical Adjuvant Breast and Bowel Project B-18. J Clin Oncol 1997;15(7):2483–2493.
141. van der Hage JA, van de Velde CJ, Julien JP, Tubiana-Hulin M, Vandervelden C, Duchateau L. Preoperative chemotherapy in primary operable breast cancer: results from the European Organization for Research and Treatment of Cancer trial 10902. J Clin Oncol 2001;19(22):4224–4237.
142. Shin SU, Cho N, Lee HB, et al. Neoadjuvant chemotherapy and surgery for breast cancer: preoperative MRI features associated with local recurrence. Radiology 2018;289(1):30–38.
143. Hylton NM. Residual disease after neoadjuvant therapy for breast cancer: can MRI help? Radiology 2018;289(2):335–336.
144. Straver ME, Loo CE, Rutgers EJ, et al. MRI-model to guide the surgical treatment in breast cancer patients after neoadjuvant chemotherapy. Ann Surg 2010;251(4):701–707.
145. McGuire KP, Toro-Burguete J, Dang H, et al. MRI staging after neoadjuvant chemotherapy for breast cancer: does tumor biology affect accuracy? Ann Surg Oncol 2011;18(11):3149–3154.
146. Kim SY, Cho N, Park IA, et al. Dynamic contrast-enhanced breast MRI for evaluating residual tumor size after neoadjuvant chemotherapy. Radiology 2018;289(2):327–334.
147. Loo CE, Straver ME, Rodenhuis S, et al. Magnetic resonance imaging response monitoring of breast cancer during neoadjuvant chemotherapy: relevance of breast cancer subtype. J Clin Oncol 2011;29(6):660–666.
148. Santamaría G, Bargalló X, Fernández PL, Farrús B, Caparrós X, Velasco M. Neoadjuvant systemic therapy in breast cancer: association of contrast-enhanced MR imaging findings, diffusion-weighted imaging findings, and tumor subtype with tumor response. Radiology 2017;283(3):663–672.
149. von Minckwitz G, Untch M, Blohmer JU, et al. Definition and impact of pathologic complete response on prognosis after neoadjuvant chemotherapy in various intrinsic breast cancer subtypes. J Clin Oncol 2012;30(15):1796–1804.
150. van la Parra RF, Kuerer HM. Selective elimination of breast cancer surgery in exceptional responders: historical perspective and current trials. Breast Cancer Res 2016;18(1):28.
151. Heil J, Schaefgen B, Sinn P, et al. Can a pathological complete response of breast cancer after neoadjuvant chemotherapy be diagnosed by minimal invasive biopsy? Eur J Cancer 2016;69:142–150.
152. Kim SY, Cho N, Shin SU, et al. Contrast-enhanced MRI after neoadjuvant chemotherapy of breast cancer: lesion-to-background parenchymal signal enhancement ratio for discriminating pathological complete response from minimal residual tumour. Eur Radiol 2018;28(7):2986–2995.
153. Pentheroudakis G, Lazaridis G, Pavlidis N. Axillary nodal metastases from carcinoma of unknown primary (CUPAx): a systematic review of published evidence. Breast Cancer Res Treat 2010;119(1):1–11.
154. McCartan DP, Zabor EC, Morrow M, Van Zee KJ, El-Tamer MB. Oncologic outcomes after treatment for MRI occult breast cancer (pT0N+). Ann Surg Oncol 2017;24(11):3141–3147.
155. Olson JA Jr, Morris EA, Van Zee KJ, Linehan DC, Borgen PI. Magnetic resonance imaging facilitates breast conservation for occult breast cancer. Ann Surg Oncol 2000;7(6):411–415.
156. Giess CS, Chikarmane SA, Sippo DA, Birdwell RL. Clinical utility of breast MRI in the diagnosis of malignancy after inconclusive or equivocal mammographic diagnostic evaluation. AJR Am J Roentgenol 2017;208(6):1378–1385.
157. Moy L, Elias K, Patel V, et al. Is breast MRI helpful in the evaluation of inconclusive mammographic findings? AJR Am J Roentgenol 2009;193(4):986–993.
158. Niell BL, Bhatt K, Dang P, Humphrey K. Utility of Breast MRI for further evaluation of equivocal findings on digital breast tomosynthesis. AJR Am J Roentgenol 2018;211(5):1171–1178.
159. Bennani-Baiti B, Bennani-Baiti N, Baltzer PA. Diagnostic performance of breast magnetic resonance imaging in non-calcified equivocal breast findings: results from a systematic review and meta-analysis. PLoS One 2016;11(8):e0160346.
160. Bennani-Baiti B, Baltzer PA. MR imaging for diagnosis of malignancy in mammographic microcalcifications: a systematic review and meta-analysis. Radiology 2017;283(3):692–701.
161. Berger N, Luparia A, Di Leo G, et al. Diagnostic performance of MRI versus galactography in women with pathologic nipple discharge: a systematic review and meta-analysis. AJR Am J Roentgenol 2017;209(2):465–471.
