# Supplementary material for: Magnetic Fields and Cancer: Epidemiology, Cellular Biology, and Theranostics
Source: Int J Mol Sci. 2022 Jan 25;23(3):1339. doi: 10.3390/ijms23031339 (PMC8835851; doi:10.3390/ijms23031339)
Supplement: Supplementary file 1 [file ijms-23-01339-s001.zip › Supplementary Data Set S1/MF and Cancer.Data/PDF/2300834828/Occupational Radiation Exposures and Air Traff.pdf]

EMBRY-RIDDLE Aeronautical University
SCHOLARLY COMMONS

International Journal of Aviation, Aeronautics, and Aerospace

Volume 7 | Issue 2 Article 6

2020

Occupational Radiation Exposures in Aviation: Air Traffic Safety Systems Considerations

Adeyinka Olumuyiwa Osunwusi
National Open University of Nigeria, aosunwusi@yahoo.com

Follow this and additional works at: https://commons.erau.edu/ijaaa

Part of the Aviation Commons

Scholarly Commons Citation
Osunwusi, A. O. (2020). Occupational Radiation Exposures in Aviation: Air Traffic Safety Systems Considerations. *International Journal of Aviation, Aeronautics, and Aerospace, 7*(2). https://doi.org/10.15394/ijaaa.2020.1476

This Literature Review is brought to you for free and open access by the Journals at Scholarly Commons. It has been accepted for inclusion in International Journal of Aviation, Aeronautics, and Aerospace by an authorized administrator of Scholarly Commons. For more information, please contact commons@erau.edu.

Occupational Radiation Exposures in Aviation: Air Traffic Safety Systems Considerations

Cover Page Footnote

____ *An earlier abridged version of this paper by the author was published as a full-length research paper titled “Occupational radiation exposures: The air traffic safety electronics personnel’s perspective” in Issue 5 (04/2014) of NAAEMAG (Official print journal of the National Association of Air Traffic Engineers – Nigeria), then edited by the author. The present work – though incorporating a sizeable amount of the literature cited in the earlier paper – has been developed following a thorough revision exercise that involved the adoption of a different methodology in an attempt to provide up-to-date insights into issues surrounding occupational exposures to non-ionizing radiation in aviation operations. **The author is also an ICAO TRAINAIR PLUS certified Aviation Training Instructor and a Subject Matter Expert (SME) on air traffic safety systems (Communications/ATSEP Basic Competency), Nigerian College of Aviation Technology, Zaria, Nigeria and was, until October 2018, the Training Manager, Directorate of Safety Electronics and Engineering Services, Nigerian Airspace Management Agency, Murtala Muhammed Airport, Lagos, Nigeria.

This literature review is available in International Journal of Aviation, Aeronautics, and Aerospace: https://commons.erau.edu/ijaaa/vol7/iss2/6

Radiation is an inevitable phenomenon of everyday human existence. Virtually everyone is exposed to a complex mix of electric and magnetic fields at many different frequencies both at home and at work (World Health Organization [WHO], 2002). Mankind is generally exposed to radiation from two main sources: man-made, and natural sources. The natural sources consist mainly of ionizing radiation (IR) exposure, which exceeds that from all man-made sources combined (UNSCEAR, 2008). IR results from background radiation including high-energy particles originating from the sun (known as solar cosmic radiation – SCR) and radiation from other stars in our galaxy, known as galactic cosmic radiation or GCR (Bagshaw, 1999; Barish, 1999; Friedberg et al., 1992) as well as bursts of energetic particles from the sun referred to as solar particle events or SPEs (Bagshaw, n.d.).

Mankind is also exposed to radiation originating from releases to the environment of radioactive material from man-made sources and from the use of fuels or materials containing naturally occurring radionuclides (UNSCEAR, 2008) as well as emissions from trace amounts of radioactive minerals in the ground (Barish, 1999; Friedberg et al., 1992). Additionally, "we are exposed internally to small amounts of radioactive substances that make their way into food and become incorporated into the body's cellular structure" (Barish, 1999, p. 195) and radiation emanating from some building materials (Bagshaw, n.d.; Friedberg & Copeland, 2003). The increasing multiplicity of technological advancements and the growing complexity of urbanization processes are also combining to elevate the probability of human exposure to non-ionizing radiation (NIR) from a wide range of artificial sources, particularly electromagnetic fields (EMFs).

The occupational radiation exposures of workers in the aviation industry have become very topical in recent times. The International Commission on Radiological Protection (ICRP) made the first reference to IR exposure resulting from flying at high altitude as early as the 1960s (ICRP, 1965). In 1977, the ICRP observed that flying at high altitude can increase exposure to IR (ICRP, 1977, para. 88). Although the ICRP ultimately identified airline flight crews as an occupationally exposed group in 1990 (Desmaris, 2015), it was not until 1994, according to Friedberg and Copeland (2003), that the Federal Aviation Administration (FAA) of the United States formally recognized that air carrier crewmembers are occupationally exposed to IR. In the European Union, aircraft crewmembers have been recognized as an occupationally exposed group at typical flight altitudes of 8-12km since 1996 (ICRP, 2016).

Although the 2006 report of the United Nations Scientific Committee on the Effects of Atomic Radiation, cited by Simon and Linet (2014), argued that epidemiological studies have not shown consistent radiation-related risks for aircrew, dose and risk assessments carried out so far by a wide variety of investigators have brought out the need for further efforts at quantifying the potential health risks associated with radiation exposure of flight crew and air

travelers. Recent investigations and expert opinions also appear to be converging regarding the fact that airline crew members and passengers, particularly those flying long-haul high-altitude routes, are subjected to exposure levels that pose great health risks, specifically the risks of cancers including prostate cancer (Gudmundsdottir, Hrafnkelsson, & Rafnsson, 2017; Pukkala et al., 2003), malignant melanoma and other skin cancers (Gudmundsdottir et al., 2017; Gundestrup & Storm, 1999; Liu et al., 2018; Pukkala et al., 2003), brain cancers including meningioma (Braganza et al., 2012), nuclear cataracts (Rafnsson et al., 2005), acute leukemias, particularly acute myeloid leukemia (Gundestrup & Storm, 1999; Lee, Kang & Yoon, 2019), basal cell carcinoma of skin and of trunk (Gudmundsdottir et al., 2017), and female breast cancer among flight attendants (McNeely et al., 2018). A handful of studies, though, have found no significantly elevated health risk in relation to thyroid cancer incidence or mortality (Liu et al., 2018; Pukkala et al., 2003), rectal cancers (Gundestrup & Storm, 1999), and brain tumors in the U.S. Air Force population (Grayson, 1996).

In recent times, works revolving around in-flight radiation exposures have dominated enquiries into aviation occupational radiation exposures. Whereas the vast majority of studies and expert opinion have centered predominantly on the radiation exposures of air carrier crew members (Aw, 2003; Desmaris, 2015; Friedberg & Copeland, 2003; Lee et al., 2019; Kojo, Aspholm, & Auvinen, 2004) and, to a lesser extent, air passengers (Alvarez, Eastham & Barrett, 2016; Enyinna, 2016; Mohler, 2003) and air traffic controllers (e.g., dos Santos Silva et al., 2013), little or no attention has been given to the occupational radiation exposures of aviation personnel in the CNS/ATM (communication, navigation, surveillance/air traffic management) technical realm. Although the World Health Organization established the International EMF Project in 1996 against the backdrop of growing concerns over possible health effects of EMFs, there is yet no consensus regarding the existence of relationships between possible hazards to human health or well-being and exposures to NIR. This is in spite of the seeming ubiquity of literature on the associations between the various types of NIR to which CNS/ATM technical personnel and other allied workers are occupationally exposed and a wide variety of health risk sets such as acute leukemia and other leukemias (Floderus et al., 1993; Guenel et al., 1993; Minder & Pfluger, 2001; Roosli et al., 2007; Theriault et al., 1994; Willet et al., 2003), cancers (Carpenter, 2010; Variani et al., 2019), neurodegenerative diseases including Alzheimer's disease and amyotrophic lateral sclerosis (Carpenter, 2010; Consales et al., 2012; Feychting et al., 2003), brain tumors (Carlberg, Koppel, Ahonen, & Hardell, 2018; Minder & Pfluger, 2001; Turner et al., 2014), Hodgkin's disease (Roosli et al., 2007), and breast cancer (Guenel et al., 1993; Stevens & Davis, 1996). Against the backdrop of methodological or other limitations in previous studies and the increasing evidence of possible health effects in populations occupationally exposed, several reviews

DOI: https://doi.org/10.15394/ijaaa.2020.1476

and meta-analyses have also stressed the need for further in-depth studies (Atzmon, Linn, Richter, & Portnov, 2016; Consales, Merla, Matino, & Benassi, 2012; Lewczuk et al., 2014; Zhi, Wang, & Hu, 2017).

This paper, therefore, explores - based on a systematic review of extant literature and research evidence on workplace radiation risks - issues surrounding occupational radiation exposures in aviation with an emphasis on the occupational radiation exposures of ATSEP (air traffic safety electronics personnel) involved in the installation, commissioning, maintenance, supervision, calibration, and operation of CNS/ATM systems.

Objective, Scope, and Definitions

This paper explored the subject-matter of occupational radiation exposures in aviation in the context of specific biological effects that are consistent with the exposures. It is primarily focused on the occupational exposures of CNS/ATM technical personnel otherwise referred to as ATSEPs. The paper is also specific to NIR from EMFs. The goal is to: facilitate an understanding of perspectives relating to the possible health effects from occupational exposures to NIR; and provoke large-scale regulatory and organizational responses to issues surrounding the potential risks of occupational exposures to non-ionizing EMFs in aviation.

Radiation is defined as the emission (sending out) of energy from any source (American Cancer Society, 2019). Although radiation exposure generally means being subjected to an IR hazard, either by irradiation or contamination (Simon & Linet, 2014), this paper conceptualizes radiation exposure principally in terms of occupational exposures to NIR hazards. In this context, therefore, the term "non-ionizing radiation" incorporates that portion of the electromagnetic spectrum that includes extremely low-frequency (ELF) and low frequency (LF) from 0 to 300 Hz, very low-frequency (VLF) or intermediate fields (300 Hz to 10 MHz), radiofrequencies (RFs) ranging from 10 MHz to 300 GHz, microwaves (with frequency ranging from 300 MHz to 300 GHz), infrared, visible light and ultraviolet (UV) light. The term *electromagnetic radiation* is defined within the confines of that portion of the spectrum that incorporates RF radiation (ranging in frequency from 300 kHz to 300 GHz) and microwave (MW) radiation (300 MHz to 300 GHz). The MW range, though, effectively begins from 1 GHz.

Generally speaking, EMFs are static electric and magnetic and time-varying electric and magnetic as well as electromagnetic fields with different frequencies. They exist wherever electricity exists, albeit they are essentially a combination of electric and magnetic fields. Electric fields are associated only with electric charge, while magnetic fields result from the physical movement of electric charge (ICNIRP, 1998). Lewczuk et al (2014, p. 2) describe electric and magnetic fields in the context of the special theory of relativity as "two aspects of the same phenomenon on a chosen reference frame of observation" where "an electrical field

in one reference frame may be perceived as a magnetic field in a different reference frame". This, however, depends on the coupling characteristic, which is frequency-dependent. At frequencies in excess of 3000 Hz, EMFs are propagated as tightly coupled electric and magnetic fields whereby the magnitude of the electric field can be determined from the magnetic field and vice versa while "in the ELF range, electric and magnetic fields are effectively uncoupled and can be evaluated separately as if they arose from independent sources" (IARC, 2002, p. 37).

In the context of this paper, the term *occupational exposure* relates strictly to radiation exposures incurred in the course of normal duties. The International Labour Organization (ILO) defines occupational exposure as "exposure of a worker received or committed during a period of work" (ILO, 1987, p. 41) while the International Commission on Non-Ionizing Radiation Protection (ICNIRP) sees occupational exposure as "All exposure to EMF experienced by individuals as a result of performing their regular or assigned job activities" (ICNIRP, 2010, p. 835).

The term "risk" is used both in terms of health situations and radiation exposures to define the probability of the occurrence of one or more adverse health risks or negative health outcomes as a result of radiation exposures.

Method

The paper deployed systematic procedures for an exploration and analysis of perspectives on the health outcomes of NIR exposures based on the qualitative synthesis technique. According to O'Flaherty and Liddy (2018), the qualitative synthesis approach serves the purpose of describing "the nature of the evidence in the literature, and interpret the possible effect of convergence and divergence among studies" (p. 1034). Within this context, literature searches involved the use of the keywords: "occupational radiation exposures", "occupational radiation exposures in aviation", "ionizing radiation", "non-ionizing radiation", "health effects: non-ionizing radiation", "electromagnetic frequency radiation", "occupational exposures to EMFs", and "radar hazards". Description and interpretation of results of studies on NIR occupational exposure were limited to the period from 1985 to 2019.

Radiation Typology

Broadly speaking, radiation can be either *ionizing* or *non-ionizing* with the distinction essentially determined by the quantum of the photon energies involved. Ionizing radiation simply results from atomic ionization. It is a process by which electrons are stripped from atoms and molecules (Cleveland & Ulcek, 1999). Examples of IR are neutrons, protons, photons (X-rays and gamma rays), electrons, and positrons (Friedberg & Copeland, 2003). IR can either be *particulate radiation* (alpha particles, beta particles, neutrons, and so on) or *electromagnetic radiation* (gamma rays, and X-rays).

DOI: https://doi.org/10.15394/ijaaa.2020.1476

In the case of NIR, the photon energies are not so great enough to strip atoms and molecules of electrons. They are, however, capable of moving or causing atoms to vibrate in a molecule. NIR types include power-frequency waves, RF waves, microwaves, laser, radar, infrared and UV light. UV light, though, can ionize an atom or a molecule.

Quantities of Measurement

Radiation quantification is usually done in terms of exposure or radiation dose. In the International System of Units (SI System), *absorbed dose* (that is, the energy absorbed per unit mass of tissue or organ when exposed to IR) is measured in Gray (Gy) while the unit of both *equivalent dose* and *effective dose* is the sievert (Sv). Cosmic radiation exposure measurements, though, are usually done in units of millisieverts (mSv). One Gray (1 Gy) is equivalent to 1 joule/kg (that is, an absorption of 1 joule of energy by one kilogram of material). 1 Gy is also equivalent to 100 rad, while one milliGray (1 mGy) is equivalent to 100 mrad. One sievert (1 Sv) is equivalent to 1000 millisieverts, while 1 millisieverts (1 mSv) is equivalent to 1000 microsieverts (µSv). While *absorbed dose* is physically measurable, both *equivalent dose* and *effective dose* are usually calculated as they cannot be measured directly. The quantities, *Roentgen Absorbed Dose* (Rad), *Roentgen Equivalent man* (Rem) and *Roentgen* (R), are the conventional units that are sometimes used, particularly in the United States of America, to measure absorbed dose, effective dose or equivalent dose, and radiation exposure respectively. Both *Rad* and *Rem*, though, are now obsolete.

The UNSCEAR uses the quantity "Collective Dose," which is expressed in units of man-Sieverts (man Sv) and defined as the sum of all the individual effective doses received in a given group under consideration, to compare the total radiation dose from various sources incurred by different groups (UNSCEAR, 2008).

When measuring RF emission in terms of power density, the unit used is the microwatts per square centimeter (μWcm^{-2}). The quantity used to determine the amount of RF energy absorbed is called the *Specific Absorption Rate* (SAR). It is usually expressed in units of watts per kilogram (W/kg) or milliwatts per gram (mW/g) (Cleveland & Ulcek, 1999). It is also used to measure absorbed dose of RF fields between about 1MHz and 10GHz, while power density in watts per square metre (W/m^2) or milliwatts per square metre (mW/m^2) is applicable to RF fields above 10GHz (WHO, 2014). The quantity, *electric field strength*, is measured in units of volts per metre (V/m or $V\ m^{-1}$) or kilovolts per metre (kV/m or $kV\ m^{-1}$), while *magnetic field* is measured in units of gauss (G) or Tesla (T), with 1G equal to 1,000 mG and 10 milligauss (mG) equal 1µT (1 microtesla). The magnitudes of electric and magnetic fields are customarily expressed as root-mean-square (rms) values (IARC, 2002). Magnetic field intensity is sometimes measured in Amperes per metre (A/m), while current density is expressed in unit of ampere per square meter ($A\ m^{-2}$).

For human exposure to ultraviolet radiation (UVR), two quantities are used. These are: *radiant exposure*, which refers to the radiant energy per unit area exposed to over a period of time and is quantified in joules per square meter or joules per square centimeter (**J/m^2 or J/cm^2**); and *irradiance*, which refers to surface exposure dose rate in terms of radiant flux density and is measured in watts per square meter or watts per square centimeter (**Wm^{-2} or Wcm^{-2}**).

Occupational Radiation Exposures of ATSEPs

Quite a staggering number of studies exist on the health consequences of occupational exposures to NIR. A number of spot measurements of EMFs have also shown high levels of radiation exposure particularly within the vicinity of air traffic control system areas. Cooper, Mann, Blackwell, and Allen (2007), for example, undertook spot measurements of electric and magnetic field strength and limb current in an attempt to investigate the range of exposures encountered by workers in the broadcast, telecommunications and air traffic control industries. Narrowband measurements carried out at one operating radar site yielded electric strengths of 91 Vm^{-1}, 240-560 Vm^{-1}, and 1.9-4.2 Vm^{-1} and mean of 1.1, 2.3-3.6, and 0.006-0.013 inside the transmission room, outside the radar site, and inside the radar control room respectively. Broadband measurements at another radar site yielded electric strengths of 23 Vm^{-1} and 19-51 Vm^{-1} at a location close to the travelling wave tube inside the transmitter room, and at the aerial platform respectively. An earlier study, Cooper et al. (2004) had reported, in relation to RF transmissions, the highest instantaneous exposures within close proximity to antennas associated with high-power VHF transmitters while the mean exposures at VHF/UHF sites were generally greatest at sites transmitting the highest powers. A seminal study conducted in Belgium (Joseph et al., 2012) assessed occupational and public exposure to EMFs emitted by 14 types of air traffic control systems. The study investigated 50 sites and a total of 1,073 locations (with frequency ranging from 255 kHz to 24 GHz) and found that NDBs (Non-directional beacons) and DVORs (Doppler VHF Omni-directional Range) both gave high levels of up to 881.6 Vm^{-1} and 92.3 Vm^{-1} respectively. The study also concluded, in respect of cumulative exposure of all RF sources, that emission from ATC sources actually dominated the total exposure in the neighborhood of the ATC systems.

The following six sections address the critical areas of the research and expert opinions relating to occupational NIR exposures vis-à-vis typical ATSEP working environments. Table 1 presents a synopsis of some of the studies relating to the health outcomes of occupational NIR exposure.

Electromagnetic Fields Radiation

Quite a staggering number of studies have investigated possible associations between occupational exposure to EMFs and a wide variety of health outcomes. A spectrum of expert opinion and empirical conclusions also exists regarding the possibility of carcinogenic risks (e.g., Carpenter, 2010; Floderus et

al, 1993; Miller et al., 2019), genotoxic or cytotoxic effects (Herbert & Sage, 2012), hematological effects (Bonhomme-Faivre et al., 1998) as well as certain neurological and reproductive impairments (Taki & Watanabe, 2001). Feychting, Johnson, Pedersen, and Ahlbom (2003), for example, investigated the potential risk factor for neurodegenerative diseases and found that EMF exposure increases the risk of early-onset Alzheimer's disease, albeit amyotrophic lateral sclerosis (ALS) was not found to be associated with EMF exposure. In relation to carcinogenic consequences, Turner et al. (2014) found that occupational exposure to ELF-EMFs may play a role in the later stages of specific histologic types of brain tumor (i.e., glioma and meningioma). An earlier study (Karipidis et al., 2007), however, found no evidence of an association between adult glioma and occupational exposure to ionizing, RF, and ELF radiation. A 2007 cohort study (Roosli et al., 2007) found no association between ELF-MFs exposure and brain tumor, non-Hodgkin's disease and lymphoid leukemia, albeit the study found some evidence of an exposure-response association for myeloid leukemia and Hodgkin's disease. Minder and Pfluger (2001) also found no dose-response relation between ELF-MFs exposure and risk of brain tumor mortality, albeit a significant increase in leukemia mortality was found. Baldi et al (2011) found a non-significant increase in risk of brain tumors for occupational exposure to EMFs [odds ratio (OR = 1.52, 0.92-2.51)] and a significant increase for meningioma for occupational exposure to ELF.

A number of studies have actually reported reproductive impairments. DeIullis et al., cited by Behari and Rajamani (2012), reported significant reduction in sperm motility and vitality after RF radiation exposure with significant elevation ($p<0.001$) of the mitochondrial generation of reactive oxygen species and DNA fragmentation. A Norwegian study (Møllerløkken & Moen, 2008) reported an increased risk of infertility among personnel engaged in radar/sonar operations with odds ratio (OR = 2.28, 95% confidence interval 1.27-4.09). Khillare and Behari (1998), cited by the National Radiological Protection Board (NRPB, 2004b), have also reported that male fertility could be decreased by prolonged exposure to 200 MHz fields modulated at 16 Hz at a level of about $2Wkg^{-1}$. Otitoloju et al., cited in Sage & Carpenter (2012), has, following a laboratory investigation in which mice were exposed for six months to base-station level RF/MW at 70 to 100 nanowatts/cm^2 (0.07 – 0.1 µW/cm^2), reported:

Table 1
A Synopsis of Studies on the Health Outcomes of Occupational NIR Exposures

Exposure	Study Design	Risk Set(s)	Findings	References
**RF/MW/radar radiation**	Meta-analysis	cancer	- No significant increase in overall mortality rate ratio and cancer risk ratio from occupational exposure to radar frequency.	Variani et al., 2019.
	Meta-analysis	Cancers	- An increased risk of lymphoma, leukemia, melanoma, breast and brain/CNS cancers.	Atzmon et al., 2016.
**MFs* (50-60 Hz)**	Nested case-control	31 cancer types	- Elevated risk for ANL* and AML* for mean exposure above 0.2 µT and levels higher than the media cumulative exposure of 3.1 µT-years;<br>- Men with cumulative exposure above the 90th percentile (15.7 µT-years) had an elevated risk for brain cancer, specifically astrocytoma, that was not statistically significant;<br>- No observed risk for other cancer types, including CLL*, skin melanoma, male breast cancer, and prostate cancer.	Theriault et al., 1994.
**EMFs***	Case-control	Acute leukemia	- No evidence of an association between acute leukemia and EMFs exposure in any time window relative to diagnosis;<br>- No association between AML* and EMFs exposure among either men or women;<br>- Increased risk of ALL* among women exposed at work.	Willet et al., 2003.
	Cohort study	Cancer	- Men with continuous exposure has an excess risk of leukemia;<br>- Intermittent exposure not associated with an increased risk of leukemia, brain tumors, or melanoma;<br>- Risk of breast cancer suggested in exposed men but not women.	Guenel et al., 1993.
		Brain tumors		

	Nested case-control	Brain tumors	- Little association between both ELF and RF/MW EMF exposure and brain tumor;<br>- Military rank was positively associated with brain tumor risk.	Grayson, 1996.
	Population-based case-control		- Non-significant increase in brain tumor risk for occupational exposure to EMFs;<br>- Significant increase in risk of meningioma for occupational exposure to ELF;<br>- No significant association with RF.	Baldi et al., 2011.
	Population-based case-control	Brain tumors	- No association between lifetime cumulative ELF exposure and glioma or meningioma risk;<br>- Positive associations between cumulative ELF 1 to 4 years before diagnosis date and glioma;<br>- No association with lifetime cumulative exposure for meningioma.	Turner et al., 2014.
	Case-control	Meningioma	- Occupational exposure to ELF-EMF not associated with an increased risk of meningioma.	Carlberg et al., 2018.
**ELF-EMFs***	Cohort study	Brain tumors & leukemia	- Positive association between heavy exposure to ELF-MFs and leukemia;<br>- No dose-response relation between brain cancer risk and ELF-MF exposure.	Minder & Pfluger, 2001.
	Case-control	Brain tumors & leukemia	- Positive association between the average, mean, daily level of EMF and CLL;<br>- No association for AML.	Floderus et al., 1993.
	Cohort study	Neurodegenerative diseases	- Occupational EMF exposure increases the risk of early-onset Alzheimer's disease;<br>- No increased risk for ALS.	Feychting et al., 2003.
**ELF/RF and IR*/UVR***	Case-control	Glioma	- No evidence of an association between glioma and occupational exposure to IR, UVR, RF, and ELF;<br>- UVR associated with increased glioma risk for men.	Karipidis et al., 2007.

*Abbreviations:** ALL – Acute lymphoblastic leukemia; AML – Acute myeloid leukemia; ANL – Acute non-lymphoid leukemia; ALS – Amyotrophic lateral sclerosis; CNS – Central nervous system; CLL – Chronic lymphoid leukemia; ELF-EMFs – Extremely low-frequency electromagnetic fields; EMFs – Electromagnetic fields; IR – Ionizing radiation; MFs – Magnetic fields; MW- microwave; RF – radiofrequency; UVR – ultraviolet radiation

> *The major abnormalities observed were knobbed hook, pin-head and banana-shaped sperm head. The occurrence of sperm head abnormalities was also found to be dose dependent. The implications of the observed increased occurrence of sperm head abnormalities on the reproductive health of humans living in close proximity to GSM base stations were discussed* (n.p.).

Some studies have, however, found no link between preconceptional parental occupational exposure to EMFs and the risk of childhood cancer (e.g., Kuijten et al., 1992). Kuijten et al. (1992), though, found a significantly elevated risk of childhood astrocytoma (a type of brain tumor) in respect of the children of electrical or electronic repairmen.

Largely, reports concerning the genotoxic or cytotoxic effects and the carcinogenic risks of EMFs exposure have been mixed with results exhibiting inconsistencies across studies. Whereas a number of studies have reported no significant genotoxic or cytotoxic effects, particularly in respect of LF/ELF EMFs exposure (e.g., Scarfi et al., 2005; Testa et al., 2004), others have reported significant effects (e.g., Buldak et al., 2012; Zmyslony et al., 2004;). Lai (2007) reported, following a review of 28 studies, a 50-50 situation with 50% of the studies reporting genotoxic effects and 50% reporting no significant effect. However, a 2014 supplementary report (Lai, 2014) revealed that 65% of studies have reported genotoxic effects while 35% reported no significant effect.

Evidence also suggests “that leukemia is the cancer most likely to show elevated risk with whole body exposure to a variety of EMFs frequencies” (Carpenter, 2010, p. 2). However, a 2003 case-control study (Willet et al., 2003) reported no evidence of an association between acute leukemia, specifically acute myeloid leukemia (AML), and occupational exposure to EMFs among either men or women, albeit the study reported that women exposed at work exhibited an increased risk of acute lymphoblastic leukemia (ALL). Most earlier and subsequent studies have, also, reported quite contradictory results with quite a sizeable number confirming elevated risks of not only leukemia (a cancer of the blood) but also brain and central nervous system (CNS) tumors, breast cancer, and melanoma (e.g. Atzmon et al., 2016; Floderus et., 1993; Minder & Pfluger, 2001). There is little evidence suggesting possible bio-effects of exposure to static magnetic fields (30-50 Hz). A handful of studies, though, have found dose-response relations between leukemia mortality and occupational exposure to MFs below 30 Hz (Minder & Pfluger, 2001). Concerns also remain concerning the association between power frequency (50/60 Hz) fields and cancer, particularly childhood leukemia (NRPB,

DOI: https://doi.org/10.15394/ijaaa.2020.1476

2004a). The International Agency for Research on Cancer (IARC) has observed that studies conducted in the 1980s and early 1990s have actually found a possible increased risk of leukemia, brain tumors, and male breast cancer in occupations with presumed exposure to ELF electric and magnetic fields above average levels (IARC, 2002).

The effects of external exposure to EMFs on the human body depend primarily on the frequency and magnitude of the EMF with LF-EMF passing through the body while RF-EMFs are partially absorbed and penetrate only a short depth into the tissue (WHO, 2002). In the case of time-varying EMF, the effects are internal body currents and energy absorption in tissues, which depend “on the coupling mechanisms and the frequency involved” (ICNIRP, 1998, p. 496). In terms of exposure to EMFs of frequencies below about 100kHz, the main physical effect of high levels of exposure is the induction of electric fields and currents in body tissues (IARC, 2002; NRPB, 2004a), with the magnitudes and spatial patterns of the fields depending on whether the external field is electric or magnetic, its characteristics and the size, shape and electric properties of the exposed body (IARC, 2002). With respect to these frequencies, the WHO observed in its 2007 Environmental Health Criteria 238:

> *Acute biological effects have been established for exposure to ELF electric and magnetic fields in the frequency range up to 100 kHz that may have adverse consequences on health. Therefore, exposure limits are needed. International guidelines exist that have addressed this issue. Compliance with these guidelines provides adequate protection.* (p. 355)

In relation to EMFs in the RF/MW and ELF ranges, there are suggestions that chronic exposure to EMFs may cause disruption of pineal physiology and decrease circulating levels of melatonin (hormone of the circadian timing system secreted by the pineal gland), thus leading to an increase in the risk of breast cancers and other tumors (Stevens & Davis, 1996). Lewczuk et al. (2014) have also observed that current stage of knowledge suggests that ELF magnetic fields and RF-EMFs have a potentially negative impact on the circadian system function consequent upon the considerable number of studies that have demonstrated alterations in sleep as well as in the secretion of melatonin and cortisol (hormone of the circadian timing system secreted by the adrenal gland) following exposure to these fields. Lyskov, Mild, and Sandström (2004, p. 91) have also found “distinctive signs of autonomous imbalance with a trend to sympathetic over activity and deviated circadian rhythmic” specifically in subjects reporting electromagnetic hypersensitivity symptoms, thus suggesting the possibility of interactions “with the ‘internal clock’ and circadian fluctuations of hormones and other basic physiological parameters.”

A popular argument has always been that because EMFs radiation lack sufficient energy to occasion damage to DNA, it should not be considered as having

the potential to cause hazardous health outcomes, particularly carcinogenic consequences. However, in 2001, the World Health Organization classified ELF-EMFs, as 2B Possible Human Carcinogen. This classification was essentially premised upon the consistent association of an increased risk of childhood leukemia with ELF-EMFs (Consales et al., 2012). In May 2011, the IARC expert group classified RF-EMFs in the frequency range 30 kHz – 300 GHz as possible human carcinogen (Group 2B) "based on an increased risk for glioma and acoustic neurinoma in human case-control studies" (Carlberg et al., 2018, p. 1). The 2B classification, though, simply refers to "Exposure circumstances for which there is limited evidence of carcinogenicity in humans and less than sufficient evidence of carcinogenicity in experimental animals" (IARC, 2002; WHO, 2002, p. 5).

Thermal Versus Non-Thermal Effects

The interaction of EMFs with biological systems is known to produce two distinct effects, namely a *thermal effect* (characterized by the heating of biological tissues as a result of the ability of EMFs to transfer their energy to the tissues), and a *non-thermal effect* (or *athermal effect*), which does not involve biological tissue heating but can, nevertheless, induce biological alterations. These alterations are both dependent not only on the intensity of the EMF but also on its frequency (Frigura-IIIasa et al, 2019).

Tissue cells may be damaged as a result of heat, cold, vibration and radiation albeit there is, throughout life, "a continuous ongoing cycle of cell damage and repair utilizing the body's self-repair mechanism" (Bagshaw, n.d., p. 9). A clearly measurable effect of EMFs exposure is the thermal effect. Consales et al. (2012) describe this thermal effect as being strictly dependent "on both the water content of the biological target, the frequency, and intensity of the electromagnetic (EM) radiation" (p. 2). Thus, in terms of the frequency of the EMF, the heating effects of exposures to EMFs with frequencies below 100 kHz are quite negligible (HSE, 2016). The distinguishing factor between radiofrequency introduced heating and other means of heating, according to Scherer (1994), is "the rapidity of heating, the depth of penetration, and the existence of internal hot-spots that can result in tissue damage long before the overall body temperature increases dramatically" (n.p.).

Two structures in the human body – the eyes and the testes – are particularly susceptible to the thermal effects of RF/MW radiation. Exposure to high levels of RF radiation can cause the formation of protein coagulation and opacities in the lens of the eyes, leading to cataracts with the damaging effect especially considerable at frequencies above 800 MHz (Scherer, 1994). Adverse health consequences such as cataracts and skin burns have especially been attributed to exposure to RF fields above 10 GHz at power densities over 1000 Wm^{-2} (WHO, 2014).

Quite a number of athermal effects, for example the occurrence of *microwave hearing effect* (characterized by buzzing, hissing or clicking sound)

particularly in relation to pulsed RF fields exposure at frequencies between 200 MHz and 6.5 GHz, have been reported (Cleveland & Ulcek, 1999; Taki & Watanabe, 2001; WHO, 2014). Effects involving the alteration of calcium ion mobility – which controls information transmission in tissue cells – have also been reported (WHO, 2014). The potential effects of EMFs on the permeability of the Blood-Brain Barrier (BBB) – a special barrier that insulates the brain from harmful compounds in the blood – have been widely investigated. Recent studies have reported increased BBB permeability for RF exposures at SARs as low as 0.016 Wkg^{-1} (Repacholi, 2003). Although some studies, mainly carried out on animals, have reported the potency of EMF exposure towards BBB disruption, there is still a need for in-depth research into this. Salford, Nittby and Persson (2012) have observed: *However, the fact that an abundance of studies do show effects is an important warning. This is true even if it can be summarized that the effects most often are weak and are seen in about 40% of the exposed animals* (p. 44).

Radar Hazards

Radar (Radio detection and ranging) systems are designed to detect the presence, range, direction of motion or speed of usually moving objects in the air, on land, or on the sea. Radars operate in the microwave region of the electromagnetic spectrum, typically at radiofrequencies of between 300MHz and 15GHz, depending on the application. They are generally characterized by pulsed operation and scanning antenna beams (Turner, 1962) and also radiate energy which could have an enormous value in kW/m^2 or even MW/m^2 in peak (Kubacki, Szmigielski, & Aniolczyk, n.d.). Radars are used for a wide variety of applications from air traffic surveillance (air traffic control radars) to weather forecasting (weather radars), marine navigation (marine radars), military applications (military radars) and road traffic monitoring (speed control radars).

Several recent studies have implicated microwave radiation exposure from radar and GSM systems for a variety of effects such as body tissue heating (Taki & Watanabe, 2001), burns (Bolen, 1994), and a range of reproductive (e.g., Goldsmith, 1997; Zaroushani, Khavanin & Mortazavi, 2014), cytotoxic and genotoxic (e.g., Garaj-Vrhovac et al., 2011), cardiovascular (Bolen, 1994), carcinogenic (e.g., Atzmon et al., 2016), hematological (e.g., Sarimov et al., 2004), and neurological effects (e.g. Zhi, Wang, & Hu, 2017). Studies have also shown that radar and GSM frequencies can induce cataracts (Goldsmith, 1997), tissue damage (Bolen, 1994), and reduced levels of leukocytes (white blood cells) and thrombocytes – blood cells responsible for blood clotting (Goldsmith, 1997). An Australian exposure survey study (Joyner & Bangay, 1986) reported the strong possibility of civilian airport radar workers getting exposed to hazardous MW radiation levels, especially when working on open waveguide or within transmitter cabinets. Richter et al. (2000), based on investigations on exposure-effect relationship in sentinel patients and their co-workers who were radar technicians,

reported findings that suggested that young persons exposed to high levels of RF/MW radiation for long periods in environments with inadequate preventive measures were at increased risk for cancer. An earlier study (Cohen et al., cited in ICNIRP, 1998, p. 504), however, found no association between MW exposure and the risk of Down's syndrome in children of male radar workers.

Interestingly, a number of factors are known to aid the reduction of exposure to radar electromagnetic waves. The first is the fact that the RF energy emitted by a radar antenna is in the form of narrow, directional beams. Secondly, radar electromagnetic waves are emitted as pulses, which drastically peg the average power emitted far below the peak pulse power. This, according to Kubacki et al. (n.d.), is because "the pulse duration (t_p) of the radar radiation is hundreds of times shorter than pulse repetition (T_p), thus an average value of power density is hundreds of times lower than a peak value of the radiation" (p. 1). The other factor has to do with the rotation of radar antennas with the implication that a point is exposed to the beam periodically within a very short time. This is, however, dependent upon the speed of rotation of an antenna as well as the width of the main lobe.

Computers and Visual Display Terminals

Computer screens or visual display terminals (VDTs) associated with computer systems and some measurement devices are known to emit a variety of EMF radiation. Mild (2004) divides the fields present in the vicinity of the cathode ray tube-based VDT into the following types: "an electrostatic field, ELF electric and magnetic fields with the refresh rate frequency, VLF electric and magnetic field with line frequency" (p. 68).

Interactions with VDTs and computer systems carry with them the potential for exposure to varying magnetic flux densities with the intensities depending on the distance from the system/equipment. For some VDTs, the equivalent electrostatic surface potential on the screen can reach up to 20 kV while both the ELF electric fields at a distance of 0.5m in front of the VDT and the VLF electric fields can reach up to tens of volts per meter (Mild, 2004). There is some suggestion that electrostatic fields associated with work with VDTs may aggravate existing skin conditions (NRPB, 2004b). The phenomenon of electromagnetic hypersensitivity (EHS) and the attendant dermatological symptoms such as erythema and eczema as well as a range of itching, burning and stinging sensations have also been attributed to VDT work (Stenberg, 2004).

Ultraviolet Radiation Hazards

Ultraviolet radiation (UVR) emanates from both natural and man-made sources. The main source of natural UVR is the sun albeit UV rays make up just a small portion of the sun's rays. Man-made UVR sources include a wide variety of optical radiation sources such as welding touches, specialized illumination or incandescent lamps and lasers, whose spectral band of emission is of particular

significance in terms of potential health risks. While some man-made sources such as lasers and special-application lamps emit in only one spectral band, other sources such as welding arcs and most illumination lamps emit broad-band radiation in all three spectral regions (IFATSEA, 1991).

ATSEPs are no doubt at risk of UVR. ATSEPs work outdoor at communications, surveillance and navigational aids sites particularly during those times of the day when UV radiation is most intense. It has been established that "Outdoor workers receive significant exposure to solar UVR and are thereby at increased risk of the adverse consequences associated with UVR exposure of the eyes and skin" (Vecchia, Hietanen, Stuck, van Deventer, & Niu, 2007, p. 11). The magnitude of the dermatological risk, though, is greatly dependent upon climatological factors and personal sensitivity to UVR while no racial or individual susceptibility is applicable to the risk for the eyes. Aside from this, ATSEPs also interact with a wide variety of man-made optical radiation sources.

In terms of the bio-effects of natural UVR exposure, scientists commonly divide UVR into three spectral components, namely: UV-A, which have the least energy with wavelengths ranging from 315 to 400 nanometres (nm); UV-B from 280 to 315 nm; and UV-C from 100 to 280nm. UV radiation is known to cause skin cancers including malignant melanoma, basal cell carcinomas and squamous cell carcinomas, as well as ocular damages such as pterygium and cataracts (Emslie, n.d.; Vecchia et al., 2007). It has also been reported to be associated with an increased risk of glioma, a malignant brain tumor (Karipidis et al., 2007). It has equally been established that UVR affects immunity and can, thus, aggravate some systemic diseases such as Lupus erythematoses as well as exacerbate infections leading to the development of skin cancer (Vecchia et al., 2007). While the bio-effects attributable to UV-C rays have been found to be negligible due to the fact that they are effectively absorbed by the earth's ozone layer, studies have linked UV-B radiation to a wide variety of bio-effects. The direct absorption of UV-B radiation has been linked with damage to DNA. UV-B has also been associated with skin cancer (Emslie, n.d.) around the eyes as well as other ocular impairments including degenerative changes of the cornea, damages to the crystalline lens of the eye and photokeratitis. Both UV-A and UV-B have also been implicated in melanomagenesis (Emslie, n.d.). Scientific evidence has also shown that the potential hazards from high intensity optical sources include: sunburn, particularly for lightly pigmented skin types; erythema and skin blistering reactions (Vecchia et al., 2007); skin or corneal (photochemical) injury from actinic UVR (IFATSEA, 1991); and the formation of acute cataract especially from UVR at wavelengths greater than 310nm (Vecchia et al., 2007).

Exposure Standards and Limits

A wide variety of EMFs standards and guidelines exist for the primary purpose of specifying permissible exposure limits (PEL) both from the standpoints

of the radiation-emitting devices (emission standards) and human exposure (exposure standards). In North America and most of Europe, exposure standards and guidelines have generally been based on exposure levels where effects considered harmful to human occur (Cleveland & Ulcek, 1999).

In terms of exposure to RF radiation, Richter et al. (2000) stressed the need to prevent exposures in the range of 10-100µW/cm^2 based on calculations derived from a linear model of dose-response. To limit thermal-related effects, the NRPB recommends 1˚C temperature increase limit for the testes, 38˚C and 39˚C for the head/spinal and the neck/trunk respectively with regard to partial-body heating, as well as limiting whole-body heat load in terms of occupational exposure to RF fields of less than 0.4Wkg^{-1} (NRPB, 2004a). This limit actually corresponds to absorbed energy of 28W in the body for a 70 kg weight, which is insignificant when compared with 60W metabolic heat produced in the body in the basal condition (Taki & Watanabe, 2001). For public exposure, an additional safety factor of 5 is specified to yield an average whole-body SAR limit of 0.08 Wkg^{-1}. The US Occupational Safety and Health Administration specifies in OSHA 29 CFR 1910.97 a radiation protection limit of 10mW/cm^2, averaged over any possible 0.1 hour period (6 minute period) for normal environmental conditions as well as for incident electromagnetic energy of frequencies from 10MHz to 100 GHz. The US Federal Communications Commission's (FCC) limit for maximum permissible exposure in the frequency range between 30MHz and 300MHz is 1.0mW/cm^2, averaged over 6 minutes. Occupational/controlled exposure limits are pegged at 0.4 W/kg averaged over the whole body and spatial peak SAR not exceeding 8 W/kg as averaged over any 1 gram of tissue with the exception of the limbs where the spatial peak SAR shall not exceed 20 W/kg averaged over any 10 grams of tissue (Sage, 2012). For occupational exposure to frequencies in the 100 kHz-10 MHz range and the 10 MHz-10GHz range, the ICNIRP recommends limits of 0.4 Wkg^{-1}, 10 Wkg^{-1}, and 20 Wkg^{-1} for whole-body average SAR, localized SAR (head and trunk), and localized SAR (limbs) respectively (ICNIRP, 1998).

In terms of exposure to static electric and magnetic fields, the NRPB has recommended the restriction of whole-body time-weighted average exposure to a magnetic flux density of 200mT for occupational exposure to static magnetic fields with an instantaneous ceiling of 2T while a ceiling of 5T is appropriate for exposure of the limits (NRPB, 2004a). The restriction of induced electric field in the central, autonomic and enteric nervous systems is put at less than 100mVm^{-1}, while the limit on induced current density in the central nervous system (CNS) for occupational exposure to frequencies in the 4 Hz to 1 kHz range is pegged at 10mAm^{-2} (ICNIRP, 1998; NRPB, 2004a) based on the observation that the thresholds for acute changes in CNS excitability and other acute effects are exceeded at levels of induced current density above 100 mAm^{-2} for frequencies in the range of a few Hz to 1 kHz (ICNIRP, 1998).

https://commons.erau.edu/ijaaa/vol7/iss2/6
DOI: https://doi.org/10.15394/ijaaa.2020.1476

For UVR exposure, the American Conference of Governmental Industrial Hygienists (ACGIH) exposure limit is 3mJ/cm^2 at 270nm for an eight-hour exposure period (IFATSEA, 1991). Similarly, the International Commission on Non-Ionizing Radiation Protection guidelines for human exposure of the eye and the skin to UVR is 3mJ/cm^2 - effective (Vecchia et al., 2007).

Discussions and Conclusion

The health risks of exposure to NIR is real in spite of the common excuse that some studies have failed to establish clear exposure-response relationships. As Sage and Carpenter (2012) have reiterated: *"The time for arguing whether EMF health effects exist is over. We know they exist and that they result in human disease"* (n.p.). Empirical evidence actually points towards two directions: the clear establishment of bio-effects linked to NIR exposure; and the fact that the levels of exposure at which specific bio-effects have been established are hundreds of times lower than the levels on which present exposure standards are built.

To be sure, ascertaining whether a specific person or a specific occupational group faces the risks associated with radiation exposures requires specific evidence of exposure, which can be obtained from measurements as well as from evidence derived specifically from the observations of specific biological effects that are consistent with radiation exposure. As it were, the observation of biological effects is a relative phenomenon, given the widespread conceptualization of biological effects of radiation in terms of impact on cells and tissues in human body. A biological effect only becomes a safety hazard when it "causes detectable impairment of the health of the individual or of his or her offspring" (Cleveland & Ulcek, 1999, p. 6). A decision in this regard would no doubt rest squarely on robust and highly reliable risk or exposure assessment drives. But as Carpenter (2010) has sadly observed, exposure assessment remains an extremely difficult thing especially when it comes to studying the effects of EMFs, thus resulting in poor exposure assessment with "a great likelihood that the total risk is underappreciated" (p. 4).

Given the findings and conclusions of the growing number of epidemiological and experimental studies on possible bio-effects of EMFs, it can be concluded that NIR exposure actually induces biological effects and that ATSEPs constitute an occupationally exposed aviation professional group. CNS/ATM technical personnel are also at a higher risk of exposure to NIR than the general public and any other working groups that are exposed to one form of NIR or the other.

Since the exposure of ATSEPs to NIR is essentially occupational, employers and relevant regulatory authorities incur responsibilities in ensuring not only the protection of ATSEPs through the institutionalization of diverse measures but also designing and implementing commensurate mitigation and compensation

regimes for ATSEPs. Aside from this, there is the need for the establishment of exposure standards that are clearly consistent with current evidence regarding the adverse health effects of occupational exposures to NIR. As Atzmon et al. (2016) have rightly observed, existing safety standards appear not to be sufficient to protect against both public and occupational exposures to EMFs, thus requiring revisions that take cognizance of not only the burgeoning scientific evidence but also the long-term effects of EMFs exposure.

Although it is acknowledged that a number of the research evidence and data buttressing the conclusions relating to the occupational exposures of ATSEPs in this paper are more than two decades old, quite a greater chunk of recently published studies and expert opinions sufficiently support the conclusion that exposure to EMFs can trigger serious biological effects. While issues surrounding the occupational exposure of ATSEPs remain a topical subject for debate, future research should be directed towards both laboratory-based and epidemiological investigations of possible health risks consequent upon consistent exposure to NIR. With the sheer paucity of personal exposure data, there is also the need for extensive job exposure matrices-based investigations aimed at quantifying occupational radiation exposures of ATSEPs. This is especially important in order to sufficiently respond to insinuations that ATSEP exposure has been inferred largely from job classification rather than on the basis of empirical determination of the exposure. It should be put on record, though, that it is not completely out of place to use active engagement in radiation-exposed occupations as a proxy for actual exposure.

Suggestions

To reduce the risks associated with exposure to EMF radiation, the following points are important from the perspectives of ATSEPs and relevant organizations:

- The time spent near or in the vicinity of EMF radiation sources should be limited.
- When working around satellite communications and radar antenna systems, the area around the reflection and the feed horn should be considered hazardous especially when the systems are energized.
- Waveguides should be properly shielded to protect personnel.
- Adequate administrative and engineering control measures should be put in place to enforce strict compliance with permissible exposure limits as well as ensure that the transmitting regions of radar and other systems are shielded. Joints should be checked for RF leakage and such leakages, when observed, should be promptly blocked. Restrictions should also be placed on access to system areas.
- A safety distance of at least 1m from the central DVOR antenna should be maintained with the antenna preferably switched off in the event of any adjustments (Joseph et al., 2012).

- Adequate grounding should be implemented to guide against a hazardous accumulation of static electric charges.
- Personnel should observe precautionary and safety measures in relevant standard operating procedure manuals and prioritize the use of protective items such as insulated gloves, safety shoes and protective clothing. The WHO (2014) has, however, recommended the exercise of great care in using protective items. The organization advises that a protective equipment should be used only when the attenuation properties of the equipment are known at a given frequency.
- Air navigation service organizations incur crucial responsibilities in ensuring that CNS/ATM personnel are adequately advised on the nature, magnitude and the health risks of their exposures.
- As protective measures against UVR in the event of outdoor maintenance activities, Vecchia et al. (2007, p. 56) recommends, *inter alia*: the use of clothing and eyewear that are designed to protect against UVR; use of hats with broad brims; and eye protection with wrap-around design or sunglasses with side panels.

References

Alvarez, L. E., Eastham, S. D., & Barrett, S. R. (2016). Radiation dose to the global flying population. *Journal of Radiological Protection*, *36*, 93-103. doi:10.1088/0952-4746/36/1/Keping Mike muted is probably a good decision!93

American Cancer Society. (2019). *Ultraviolet (UV) radiation, July 10, 2019*. Retrieved from www.cancer.org/cancer/cancer-causes/radiation-exposure/uv-radiation.html

Atzmon, I., Linn, S., Richter, E. D., & Portnov, B. (2016). Microwave/radiofrequency (MW/RF) radiation exposure and cancer risk: Meta-analysis of accumulated empirical evidence. *International Journal of Cancer and Clinical Research*, *3*(1), 1-9.

Aw, J. J. (2003). Cosmic radiation and commercial air travel. *Journal of Travel Medicine*, *10*(1), 19-28.

Bagshaw, M. (n.d.). *Cosmic radiation in commercial aviation*. Retrieved from www.iaasm.org/documents/Cosmic_Radiation.pdf/

Bagshaw, M. (1999). Cosmic radiation measurements in airline service. *Radiation Protection Dosimetry*, *86*(4), 333-334.

Baldi, I., Coureau, G., Jaffré, A., Gruber, A., Ducamp, S., Provost, D., . . . Salamon, R. (2011). Occupational and residential exposure to electromagnetic fields and risk of brain tumors in adults: A case-control study in Gironde, France. *International Journal of Cancer*, *129*, 1477-1484.

Barish, R. J. (1999). In-flight radiation: Counseling patients about risk. *Journal of the American Board of Family Practice*, *12*(3), 195-199.

Behari, J., & Rajamani, P. (2012). Electromagnetic field exposure effects (ELF-EMF and RFR) on fertility and reproduction. In: C. Sage & D. O. Carpenter (Eds.), *BioInitiative Report: A rationale for biologically-based exposure standards for low-intensity electromagnetic radiation* (Section 18), *BioInitiative Working Group 2012*. Retrieved from www.bioinitiative.org/

Bolen, S. M. (1994). *Radiofrequency/microwave radiation biological effects and safety standards: A review*. In-House Report (RL-TR-94-53). New York: Rome Laboratory, Griffiss Air Force Base.

Bonhomme-Faivre, L., Marion, S., Bezie, Y., Auclair, H., Fredj, G., & Hommeau, C. (1998). Study of human neurovegetative and hematologic effects of Environmental low-frequency (50-Hz) electromagnetic fields produced by transformers. *Archives of Environmental Health*, *53*(2), 87-92.

Braganza, M. Z., Kitahara, C. M., Berrington de Gonzalez, A., Inskip, P. D., Johnson, K. J., & Rajaraman, P. (2012). Ionizing radiation and the risk of

brain and central nervous system tumors: A systematic review. *Neuro-Oncology*, *14*(11), 1316-1324.

Buldak, R. J., Polaniak, R., Buldak, L., Zwirska-Korczala, K., Skonieczna, M., Monsiol, A., . . . Birkner, E. (2012). Short-term exposure to 50 Hz ELF-EMF alters the cisplatin-induced oxidative response in AT478 murine squamous cell carcinoma cells. *Bioelectromagnetics*, *33*(8), 641-651. doi:10.1002/bem.21732

Carlberg, M., Koppel, T., Ahonen, M., & Hardell, L. (2018). Case-control study on occupational exposure to extremely low-frequency electromagnetic fields and the association with meningioma. *BioMed Research International*, *2018*, 1-6. https://doi.org/10.1155/2018/5912394

Carpenter, D. O. (2010). Human health effects of EMFs: The cost of doing nothing. *IOP Conference Series: Earth and Environmental Science, 10*, 012004. doi:10.1088/1755-1315/10/1/012004

Cleveland, R. F., & Ulcek, J. K. (1999). *Questions and answers about biological effects and potential hazards of radiofrequency electromagnetic fields*. OET Bulletin 56, Fourth Edition. Washington, DC: Office of Engineering and Technology, FCC.

Consales, C., Merla, C., Marino, C., & Benassi, B. (2012). Electromagnetic fields, oxidative stress, and neurodegeneration. *International Journal of Cell Biology*, *2012*, 1-16. doi:10.1155/2012/683897

Cooper, T. G., Allen, S. G., Blackwell, R. P., Litchfield, I., Mann, S. M., Pope, J. M., & van Tongeren, M. J. A. (2004). Assessment of occupational exposure to Radiofrequency fields and radiation. *Radiation Protection Dosimetry*, *111*(2), 191-203. doi:10.1093/rpd/nch334

Cooper, T. G., Mann, S. M., Blackwell, R. P., & Allen, S. G. (2007). *Occupational exposure to electromagnetic fields at radio transmitter sites*. Oxfordshire: Radiation Protection Division, Health Protection Agency (HPA).

Desmaris, G. (2015). Cosmic radiation in aviation: Radiological protection of Air France aircraft crew. *International Commission on Radiological Protection (ICRP) 2015 Proceedings*. Retrieved from https://journals.sagepub.com/doi/pdf/10.1177/0146645316636009.pdf

dos Santos Silva, I., De Stavola, B., Pizzi, C., Evans, A. D., & Evans, S. A. (2013). Cancer incidence in professional flight crew and air traffic control officers: Disentangling the effect of occupational versus lifestyle exposures. *International Journal of Cancer*, *132*(2), 374-384.

Emslie, N. (n.d.). *The elevated risk of melanoma among pilots: Could UVA be implicated?* Retrieved from www.exeley.com/exeley/journals/journal_australiasian_society_aerospace_medicine/11/i_current/pdf/10.21307_asam-2019-004.pdf/

Enyinna, P. I. (2016). Radiological risk assessment of cosmic radiation at aviation altitudes (A trip from Houston Intercontinental Airport to Lagos International Airport). *Journal of Medical Physics*, *41*(3), 205-209.

Feychting, M., Jonsson, F., Pedersen, N. L., & Ahlbom, A. (2003). Occupational magnetic field exposure and neurodegenerative disease. *Epidemiology*, *14*(4), 413-419.

Floderus, B., Persson, T., Stenlund, C., Wennberg, A., Ost, A., & Knave, B. (1993). Occupational exposure to electromagnetic fields in relation to leukemia and brain tumors: A case-control study in Sweden. *Cancer Causes and Control*, *4*, 465-476.

Friedberg, W., & Copeland, K. (2003). *What aircrews should know about their occupational exposure to ionizing radiation*. Report #DOT/FAA/AM-03/16. Washington DC: Office of Aerospace Medicine, Federal Aviation Administration.

Friedberg, W., Snyder, L., Faulkner, D. N., Darden, E. B., & O'Brien, K. (1992). *Radiation exposure of air carrier crewmembers II*. Final Report No. DOT/FAA/AM-92/2. Washington DC: Civil Aerospace Medical Institute, Federal Aviation Administration.

Frigura-IIiasa, M., Baloi, F. I., Frigura-IIiasa, F. M., Simo, A., Musuroi, S., & Andea, P. (2019). Health-related electromagnetic field assessment in the proximity of high voltage power equipment. *Applied Sciences*, *10*(260), 1-16. doi: 10.3390/app10010260

Garaj-Vrhovac, V., Gajski, G., Pazanin, S., Sarolic, A., Domijan, A. M., Flajs, D., & Peraica, M. (2011). *International Journal of Hygiene and Environmental Health*, *214*(1), 59-65. doi:10.1016/j.ijheh.2010.08.003

Goldsmith, J. R. (1997). Epidemiologic evidence relevant to radar (microwave) effects. *Environmental Health Perspectives*, *105*(6), 1579-1587.

Grayson, J. K. (1996). Radiation exposure, socioeconomic status, and brain tumor risk in the US Air Force: A nested case-control study. *American Journal of Epidemiology, 143*(5), 480-486.

Guenel, P., Raskmark, P., Andersen, J. B., & Lynge, E. (1993). Incidence of cancer in persons with occupational exposure to electromagnetic fields in Denmark. *British Journal of Industrial Medicine*, *50*, 758-764.

Gudmundsdottir, E. M., Hrafnkelsson, J., & Rafnsson, V. (2017). Incidence of cancer among licensed commercial pilots flying North Atlantic routes. *Environmental Health*, *16*(86). doi:10.1186/s12940-017-0295-4

Gundestrup, M., & Storm, H. H. (1999). Radiation-induced acute myeloid leukemia and other cancers in commercial jet cockpit crew: A population-based cohort study. *The Lancet*, 354, 2029-2031.

Herbert, M., & Sage, C. (2012). Findings in Autism (ASD) consistent with electromagnetic fields (EMF) and radiofrequency radiation (RFR). In: C.

Sage & D. O. Carpenter (Eds.), *BioInitiative Report: A rationale for biologically-based exposure standards for low-intensity electromagnetic radiation* (Section 20), *BioInitiative Working Group 2012*. Retrieved from www.bioinitiative.org/

HSE. (2016). Electromagnetic fields at work: A guide to the control of the electromagnetic fields at work, HSG281. Retrieved from www.hse.gov.uk/ pubns/priced/hsg281.pdf/

IARC. (2002). *Static and extremely low-frequency (ELF) electric and magnetic fields*. IARC Monographs on the Evaluation of Carcinogenic Risks to Humans, Non-Ionizing Radiation, Part 1, Vol. 80. Lyon: IARC.

ICNIRP. (1998). ICNIRP guidelines for limiting exposure to time-varying electric, magnetic and electromagnetic fields (up to 300 GHz). *Health Physics*, *74*(4), 494-522.

ICNIRP. (2010). ICNIRP guidelines for limiting exposure to time-varying electric and magnetic fields (1 Hz-100 kHz). *Health Physics*, *99*(6), 818-836.

ICRP. (1965). *Recommendations of the international commission on radiological protection*, ICRP Publication 9. Oxford: Pergamon.

ICRP. (1977). Recommendations of the ICRP. ICRP Publication 26. *Annals of the ICRP*, *1*(3).

ICRP. (2016). Radiological protection from cosmic radiation in aviation. ICRP Publication 132. *Annals of the ICRP*, *45*(1), 1-48.

IFATSEA. (1991). Ultraviolet, visible and infrared radiation. IFATSEA Manual on Radiation Protection in Aviation, Chapter II. *Navaire*, *14,* 19-25.

ILO. (1987). *Radiation protection of workers – Ionizing Radiation*, ILO Code of Practice. Geneva: International Labour Office.

Joseph, W., Goeminne, F., Vermeeren, G., Verloock, L., & Martens, L. (2012). Occupational and public field exposure from communication, navigation, and radar systems used for air traffic control. *Health Physics*, *103*(6), 750-762. doi:10.1097/HP.0b013e31825f78d5.

Joyner, K. H., & Bangay, M. J. (1986). Exposure survey of civilian airport radar workers in Australia. *Journal of Microwave Power and Electromagnetic Energy*, *21*(4), 209-219. doi:10.1080/08327823.1986.11687996

Karipidis, K. K., Benke, G., Sim, M. R., Kauppinen, T., & Giles, G. (2007). Occupational exposure to ionizing and non-ionizing radiation and risk of glioma. *Occupational Medicine*, *57,* 518-524.

Kojo, K., Aspholm, R., & Auvinen, A. (2004). Occupational radiation dose estimation for Finnish aircraft cabin attendants. *Scandinavian Journal of Work, Environment and Health*, *30*(2), 157-163. doi:10.5271/sjweh.773

Kubacki, R., Szmigielski, S., & Aniolczyk, H. (n.d.). *Measurement of microwave radiation from rotating radar antennas and proposals for limiting exposure to pulse-modulated radiation in general public and*

occupational. Retrieved from www.who.int/peh-emf/meetings/en/ 7Kubacki-Measurement_of_Microwave_Rad.pdf/

Kuijten, R. R., Bunin, G. R., Nass, C. C., & Meadows, A. T. (1992). Parental occupation and childhood astrocytoma: Results of a case-control study. *Cancer Research*, 52, 782-786.

Lai, H. (2007). Evidence for genotoxic effects (RFR and ELF genotoxicity). A report prepared for the BioInitiative working group, July 2007. In: C. Sage & D.O. Carpenter (Eds.), *BioInitiative Report: A rationale for biologicallybased exposure standards for low-intensity electromagnetic radiation* (Section 6), *BioInitiative Working Group 2012*. Retrieved from www.bioinitiative.org/

Lai, H. (2014). *Genetic effects of non-ionizing electromagnetic fields*. 2014 Supplement prepared for the BioInitiative Working Group, March 2014. Retrieved from www.bioinitiative.org/

Lee, W., Kang, M.-Y., & Yoon, J.-H. (2019). Cancer incidence among air transportation industry workers using the national cohort study of Korea. *International Journal of Environmental Research and Public Health* 16, 2906, 1-12. doi:10.3390/ijerph16162906.

Lewszuk, B., Redlarski, G., Zak, A., Ziolkowska, N., Przybylska-Gornowicz, B., & Krawczuk, M. (2014). Influence of electric, magnetic, and electromagnetic fields on the circadian system: Current stage of knowledge. *BioMed Research International*, *2014*, 1-13.

Liu, G. S., Cook, A., Richardson, M., Vail, D., Holsinger, F. C., & Oakley-Girvan, I. (2018). Thyroid cancer risk in airline cockpit and cabin crew: A meta-analysis. *Cancers of the Head and Neck*, *3*(7), 1-8. https://doi.org/10.1186/s41199-018-0034-8

Lyskov, E., Mild, K. H., & Sandström, M. (2004). Sensor reactivity and autonomous regulation in persons with perceived electrical hypersensitivity. In: K.H. Mild, M. Repacholi, E. van Deventer, & P. Ravazzani (Eds.), *Electromagnetic Hypersensitivity* (pp. 87-92), Proceedings of the International Workshop on EMF Hypersensitivity, Prague, Czech Republic, 25-27 0ctober, 2004.

McNeely, E., Mordukhovich, I., Staffa, S., Tideman, S., Gale, S., & Coull, B. (2018). Cancer prevalence among flight attendants compared to the general population. *Environmental Health*, 17(49), 1-9. https://doi.org/10.1186/s12940-018-0396-8.

Mild, K. H. (2004). Electromagnetic environments in electrical hypersensitives homes and workplaces. In: K.H. Mild, M. Repacholi, E. van Deventer, & P.Ravazzani (Eds.), *Electromagnetic Hypersensitivity* (pp. 63-77), Proceedings of the International Workshop on EMF Hypersensitivity, Prague, Czech Republic, 25-27 0ctober, 2004.

Miller, A. B., Sears, M. E., Morgan, L. L., Davis, D. L., Hardell, L., Oremus, M., & Soskolne, C. L. (2019). Risks to health and well-being from radio-frequency radiation emitted by cell phones and other wireless devices. *Frontiers in Public Health*, 7, 1-10. doi:10.3389/fpubh.2019.00223

Minder, C. E., & Pfluger, D. H. (2001). Leukemia, brain tumors, and exposure to Extremely low frequency electromagnetic fields in Swiss railway Employees. *American Journal of Epidemiology*, 153(9), 825-835.

Mohler, S. R. (2003). Galactic radiation exposure during commercial flights: Is there a risk? *Canadian Medical Association Journal*, 168(9), 1157-1158.

Møllerløkken, O. J., & Moen, B. E. (2008). Is fertility reduced among men exposed to radiofrequency fields in the Norwegian Navy? *Bioelectromagnetics*, *29*(5), 345-352. doi:10.1002/bem.20400

NRPB. (2004a). *Advice on limiting exposure to electromagnetic fields (0-300 GHz)*. NRPB Document, Vol. 15, No. 2. Oxfordshire: National Radiological Protection Board.

NRPB. (2004b). *Review of the scientific evidence for limiting exposure to electromagnetic fields (0- 300 GHz)*. NRPB Document, Vol. 15, No.3. Oxfordshire: National Radiological Protection Board.

O'Flaherty, J., & Liddy, M. (2018). The impact of development education and education for sustainable development interventions: A synthesis of the research. *Environmental Education Research*, *24*(7), 1031-1049.

Pukkala, E., Aspholm, R., Auvinen, A., Eliasch, H., Gundestrup, M., Haldorsen, . . . Tveten, U. (2003). Cancer incidence among 10,211 airline pilots: A Nordic study. *Aviation Space and Environmental Medicine*, *74*(7), 699-706.

Rafnsson, V., Olafsdottir, E., Hrafnkelsson, J., Sasaki, H., Arnarsson, A., & Jonasson, F. (2005). Cosmic radiation increases the risk of nuclear cataract in airline pilots: A population-based case-control study. *Arch Ophthalmology*, *123*(8), 1102-1105. doi:10.1001/archopht.123.8.1102

Repacholi, M. H. (2003). An overview of WHO's project and the health effects of EMF exposure. *Proceedings of the International Conference on Non-Ionizing Radiation at UNITEN (ACNIR 2003) Electromagnetic Fields and our Health*, 20th October 2003.

Richter, E., Berman, T., Ben-Michael, E., Laster, R., & Westin, J. B. (2000). Cancer in radar technicians exposed to radiofrequency/microwave radiation: Sentinel episodes. *International Journal of Occupational and Environmental Health*, *6*(3), 187-193.

Roosli, M., Lortscher, M., Egger, M., Pfluger, D., Schreier, N., Lortscher, E., . . . Minder, C. (2007). Leukaemia, brain tumours and exposure to extremely low frequency magnetic fields: Cohort study of Swiss railway employees.

Occupational and Environmental Medicine, *64*, 553-559. doi:10.1136/oem.2006.030270.

Sage, C (2012). The existing public exposure standards. In: C. Sage & D.O. Carpenter (Eds.), *BioInitiative Report: A rationale for biologically-based exposure standards for low-intensity electromagnetic radiation* (Section 3). *BioInitiative Working Group 2012*. Retrieved from www.bioinitiative.org/

Sage, C., & Carpenter, D. O. (2012). Key scientific evidence and public health policy recommendations (2012 Supplement). In: C. Sage & D. O. Carpenter (Eds.), *BioInitiative Report: A rationale for biologically-based exposure standards for low-intensity electromagnetic radiation* (Section 24), *BioInitiative Working Group 2012*. Retrieved from www.bioinitiative.org/

Salford, L. G., Nittby, H., & Persson, B. R. R. (2012). Effects of electromagnetic fields from wireless communication upon the Blood-Brain Barrier. In: C. Sage & D. O. Carpenter (Eds.), *BioInitiative Report: A rationale for biologically-based exposure standards for low-intensity electromagnetic radiation* (Section 10), *BioInitiative Working Group 2012*. Retrieved from www.bioinitiative.org/

Sarimov, R., Malmgren, L. O. G., Markova, E., Persson, B. R. R., & Belyaev, I. Y. (2004). Non thermal GSM microwaves affect chromatin conformation in human lymphocytes similar to heat shock. *IEEE Transaction on Plasma Science*, *32*(4), 1600-1608. doi:10.1109/TPS.2004.832613

Scarfi, M. R., Sannino, A., Perrotta, A., Sarti, M., Mesirca, P., & Bersani, F. (2005). Evaluation of genotoxic effects in human fibroblasts after intermittent exposure of 50 Hz electromagnetic fields: A confirmatory study. *Radiation Research*, *164*(3), 270-276. doi:10.1667/rr3427.1

Scherer, W. W. (1994). *Biological effects of radiofrequency and microwave radiation: Application, hazards, and safeguards*. Retrieved from www.reach.net/~scherer/p/biofx.htm/

Simon, S. L., & Linet, M. S. (2014). Radiation-exposed populations: Who, why and how to study. *Health Physics*, *106*(2), 182-195.

Stenberg, B. (2004). Characterizing electrical hypersensitivity. In: K.H. Mild, M. Repacholi, E. van Deventer, & P. Ravazzani (Eds.), *Electromagnetic Hypersensitivity* (pp. 29-37), Proceedings of the International Workshop on EMF Hypersensitivity, Prague, Czech Republic, 25-27 0ctober, 2004.

Stevens, R. G., & Davis, S. (1996). The melatonin hypothesis: Electric power and breast cancer. *Environmental Health Perspectives*, *104*(1), 135-140.

Taki, M., & Watanabe, S. (2001). Biological and health effects of exposure to electromagnetic field from mobile communications systems. *IATSS Research*, *25*(2), 40-50.

Testa, A., Cordelli, E., Stronati, L., Marino, C., Lovisolo, G. A., Fresegna, A.M., . . . Villani, P. (2004). Evaluation of genotoxic effect of low level50 Hz magnetic fields on human blood cells using different cytogenetic assays. *Bioelectromagnetics*, 25(8), 613-619. doi:10.1002/bem.20048

Theriault, G., Goldberg, M., Miller, A. B., Armstrong, B., Guenel, P., Deadman, J., . . . Wall, C. (1994). Cancer risks associated with occupational exposure to magnetic fields among electric utility workers in Ontario and Quebec, Canada, and France" 1970-1989. *American Journal of Epidemiology*, 139(6), 550-572.

Turner, M. C., Benke, G., Bowman, J. D., Figuerola, J., Fleming, S., Hours, . . . Cardis, E. (2014). Occupational exposure to extremely low-frequency magnetic fields and brain tumor risks in the INTEROCC study. *Cancer, Epidemiology, Biomarkers and Prevention*, 23(9), 1863-1872. doi:10.1158/1055-9965.EPI-14-0102

Turner, J. J. (1962). *The effects of radar on the human body*. Report No. RM-TR-62-1. New Jersey: US Army Ordnance Missile Command. Retrieved from www.dtic.mil/dtic/tr/fulltext/u2/273787.pdf/

UNSCEAR. (2008). *Sources and effects of ionizing radiation*. United Nations Scientific Committee on the Effects of Atomic Radiation (UNSCEAR) 2008 Report, Vol. 1 (Sources). New York: United Nations.

Variani, A. S., Saboori, S., Shahsavari, S., Yari, S., & Zaroushani, V. (2019). Effect of occupational exposure to radar radiation on cancer risk: A systematic review and meta-analysis. *Asian Pacific Journal of Cancer Prevention*, *20*(11), 3211-3219. doi:10.31557/APJCP.2019.20.11.3211

Vecchia, P., Hietanen, M., Stuck, B. E., van Deventer, E., & Niu, S. (Eds.). (2007). ICNIRP. (2007). *Protecting workers from ultraviolet radiation*. Retrieved from https://www.who.int/uv/publications/Protecting_ Workers_UV_pub.pdf

World Health Organization. (2002). *Establishing a dialogue on risks from electromagnetic fields*. Geneva: Author.

World Health Organization. (2007). *Extremely low frequency fields (Environmental Health Criteria 238*). Geneva: Author.

World Health Organization. (2014). *Electromagnetic fields and public health: Radars and human health*. WHO Fact Sheet No. 226. Retrieved from www.who.int/peh-emf/publications/Facts/Fs226/en/

Willet, E. V., McKinney, P. A., Fear, N. T., Cartwright, R. A., & Roman, E. (2003). Occupational exposure to electromagnetic fields and acute leukemia: Analysis of a case-control study. *Occupational and Environmental Medicine*, *60*, 577-583.

Zaroushani, V., Khavanin, A., & Mortazavi, S. B. (2014). Nonthermal effects of radar exposure on human: A review article. *Iranian Journal of Health, Safety and Environment*, *1*(1), 43-52.

Zhi, W.-J., Wang, L.-F., & Hu, X.-J. (2017). Recent advances in the effects of microwave radiation on brains. *Military Medical Research*, *4*(29), 1-14.

Zmyslony, M., Palus, J., Dziubaltowska, E., Politanski, P., Mamrot, P., Rajkowska, E., & Kamedula, M. (2004). Effects of in vitro exposure to power frequency magnetic fields on UV-induced DNA damage of rat lymphocytes. *Bioelectromagnetics*, *25*(7), 560-562.
