# Supplementary material for: Magnetic Fields and Cancer: Epidemiology, Cellular Biology, and Theranostics
Source: Int J Mol Sci. 2022 Jan 25;23(3):1339. doi: 10.3390/ijms23031339 (PMC8835851; doi:10.3390/ijms23031339)
Supplement: Supplementary file 1 [file ijms-23-01339-s001.zip › Supplementary Data Set S1/MF and Cancer.Data/PDF/2674911271/Karimi2020_Article_InsightsInTheBiologyOfExtre.pdf]

Molecular Biology Reports (2020) 47:5621–5633
https://doi.org/10.1007/s11033-020-05563-8

REVIEW

Insights in the biology of extremely low-frequency magnetic fields exposure on human health

Abbas Karimi[1,2] · Farzaneh Ghadiri Moghaddam[1,3] · Masoumeh Valipour[3]

Received: 16 April 2020 / Accepted: 27 May 2020 / Published online: 8 June 2020
© Springer Nature B.V. 2020

Abstract
The extremely low-frequency magnetic fields (ELF-EMF) are generated by electrical devices and power systems (1 to 300 Hz). In recent decades, exposure to ELF-EMF has emerged potential concerns on public health. Here, we discuss recent progress in the understanding of ELF-EMF biology with a focus on mechanisms of ELF-EMF-mediated disease and summarize the results of more recent experimental and epidemiological studies of ELF-EMF exposure effects on cancer, neurological, cardiovascular, and reproductive disorders. Current views on genomic instability effects, as well as scientific evidence about ELF-EMF therapy, are put forth. According to our literature review, exposure to ELF-EMF has an adverse biological effect depending on the current intensity, strength of the magnetic field, and duration of exposure. Accumulated epidemiologic evidence indicates a correlation between exposure to ELF-EMF and childhood cancer incidence, Alzheimer's disease (AD), and miscarriage. However, adult cancer does not show augmented risk caused by the ELF-EMF. Also, no consistent evidence exists in cardiovascular disease mortality due to ELF-EMF exposure. There is a lack of comprehensive mechanisms for explaining the biological effect of ELF-EMF. Eventually, more studies are needed to clarify the mechanisms of these magnetic fields.

Keywords Extremely low-frequency electromagnetic fields (ELF-EMFs) · Long-term exposure · Leukemia · Fertility · Alzheimer disease

Introduction

Both natural and human-made sources produce magnetic fields (MF) and Electromagnetic fields (EMF), and electric magnetic current is flowing everywhere. Extremely-low-frequency magnetic fields originating from human-made sources generally have much higher intensities than the naturally occurring atmospheric fields [1]. Electric and magnetic fields, which we continuously expose in wherever electricity is generated, transmitted, or distributed, have three frequency ranges including the low-frequency (LF) fields (1 Hz–100 kHz), high-frequency fields in the band of radiofrequency (100 kHz–3 GHz) and microwaves (above 3 GHz) [2, 3]. MFs, happen when there is electric current flow, with varied frequencies are measured in Hertz (Hz), and size of waves. The lowest rate (0 Hz) is represented by direct current or static fields. The higher frequency than 10^{16} Hz, comprises ionizing radiations X-rays, Gama rays, and ultraviolet light (UV). The extremely low-frequency electromagnetic field (ELF-EMF) has a long wavelength and occupies the range between 3 and 300 Hz. The electric power network results in extremely low-frequency fields, ranging from 50 Hz in Europe, 60 Hz in North America [4]. The ELF-EMF is non-ionizing radiation (NIR) and does not carry enough energy per quantum to ionize atoms or molecules [5]. The common sources of ELF-EMF in the home appliances are refrigerators, vacuum cleaners, TV, computer monitors. Anyone at home and work are exposed to a combination of weak electrical and magnetic fields emitted by power lines and electronic devices. Indeed, enhanced demand for electricity-leading technologies and changes in social behavior have increased the resource of these fields,

✉ Abbas Karimi
karimia@tbzmed.ac.ir

1 Biotechnology Research Center, Tabriz University of Medical Sciences, Tabriz, Iran

2 Department of Molecular Medicine, Faculty of Advanced Medical Sciences, Tabriz University of Medical Sciences, Tabriz, Iran

3 Department of Biology, Faculty of Science, Azarbaijan Shahid Madani University, Tabriz, Iran

so the human-made origin of these types of radiations is prominent than the natural source. What extends the ELF-EMF may affect biological condition is dependent on the field strength, distance from the source, and the exposure time. The highest rate is when a person is very close to a high power source and long exposure time [6, 7].

Since the first evidence published in 1979 and determined the relation between the ELF-EMF and leukemia in children, [8] studies in this context increased until the International Agency for Research on Cancer (IARC) classified the ELF-EMF in group 2B, a "possible carcinogen" to humans in 2002. This classification remains up to now [9]. Two decades of the study confirmed the association between ELF-EMF and childhood cancers, especially leukemia [10]; likewise, there are reports of a twofold increase in the risk of childhood leukemia from pooled analyses of previous studies [11]. Recently published studies data did not show consistent results to support the association between ELF-EMF and some types of cancer, such as glioma risk [12]. However, several pieces of evidence report the harmful effects of ELF-EMF on the brain (Table 1). The ELF-EMF exposure influence a wide variety of diseases; a meta-analysis indicates that occupational exposure to ELF-EMF increases the risk of AD [13].

Moreover, there are hypotheses that ELF-EMF exposure can cause heartbeat disturbances and cardiovascular diseases [37]. The in vivo and in vitro studies have reported that exposure to residential and occupational EMF affect endocrine system function, reproductive function (such as sperm motility, male germ cell death, and reproductive endocrine hormones) and fetal development of animals [38] (Fig. 1). Albeit, there is a clear consensus on the EMFs adverse effect; however, some studies highlight the positive effects of magnetic field therapy, in particular, in the rehabilitation of post-stroke patients and cancer treatment, specially in combination with an anticancer drug [39, 40].

Table 1 Possible effects of occupational exposure to ELF and diseases in recent 10-year studies

Study	Condition	Positive evidence	References No
Turner et al. (2017)	Meningioma[a]	No	[14]
Carlberg et al. (2018)	Meningioma	No	[12]
Carlberg et al. (2017)	Astrocytoma grade IV	Yes	[15]
Turner et al. (2014)	Glioma	Yes	[16]
Oraby et al. (2018)	Brain tumors	No	[17]
Li et al. (2009)	Childhood brain tumors	Yes	[18]
Huss et al. (2018)	Acute myeloid leukemia	yes	[19]
Talibov et al. (2019)	Childhood leukemia*	No	[20]
Su et al. (2016)	Parental occupational ELF-MF exposure and childhood leukemia risk*	No	[21]
Koeman et al. (2014)	Follicular lymphoma (FL)	yes	[22]
Li et al. (2013)	Breast cancer	No	[23]
Zhou et al. (2012)	ALS*	No	[24]
Parlett et al. 2011)	ALS	No	[25]
Huss et al. (2015)	ALS	Yes	[26]
Koeman et al. (2017)	ALS	Yes	[27]
Peters et al. (2019)	ALS	Yes	[28]
Pedersen et al. (2017)	Dementia, motor neurone disease, multiple sclerosis and epilepsy,	Yes	[29]
Vergara et al. (2013)	Primarily Alzheimer disease (AD) and motor neuron diseases (MNDs)[b,*]	–	[30]
van der Mark et al. (2015)	Parkinson's disease (PD)	No	[31]
Brouwer et al. (2015)	PD mortality (occupational exposure to pesticides and ELF-MF)	Yes	[32]
Koeman et al. (2013)	Cardiovascular disease (CVD)	No	[33]
Migault et al. (2020)	Prematurity or small for gestational age (SGA)	No	[34]
Migault et al. (2018)	Moderate prematurity or small for gestational age	No	[35]

Asterisk indicate the studies included in the meta-analysis

Only findings from the studies in the last ten years evaluated occupational exposure of ELF on various diseases, and conditions based on JEM methods are listed here. The ELF-MF JEM reflects the intensity of timeweighted average exposure in micro-Tesla (μT) by job-based on available measurement data. Bowman et al. have evaluated how magnetic field JEMs can be used in population-based epidemiologic studies [36]

[a]Interactions between ELF and any of the chemical exposures

[b]Moderately increased risk estimates for MND and AD studies with considerable heterogeneity due to the methodologic differences among the studies. Conflicting results are due to the misclassification of disease and imprecise exposure assessment in these studies

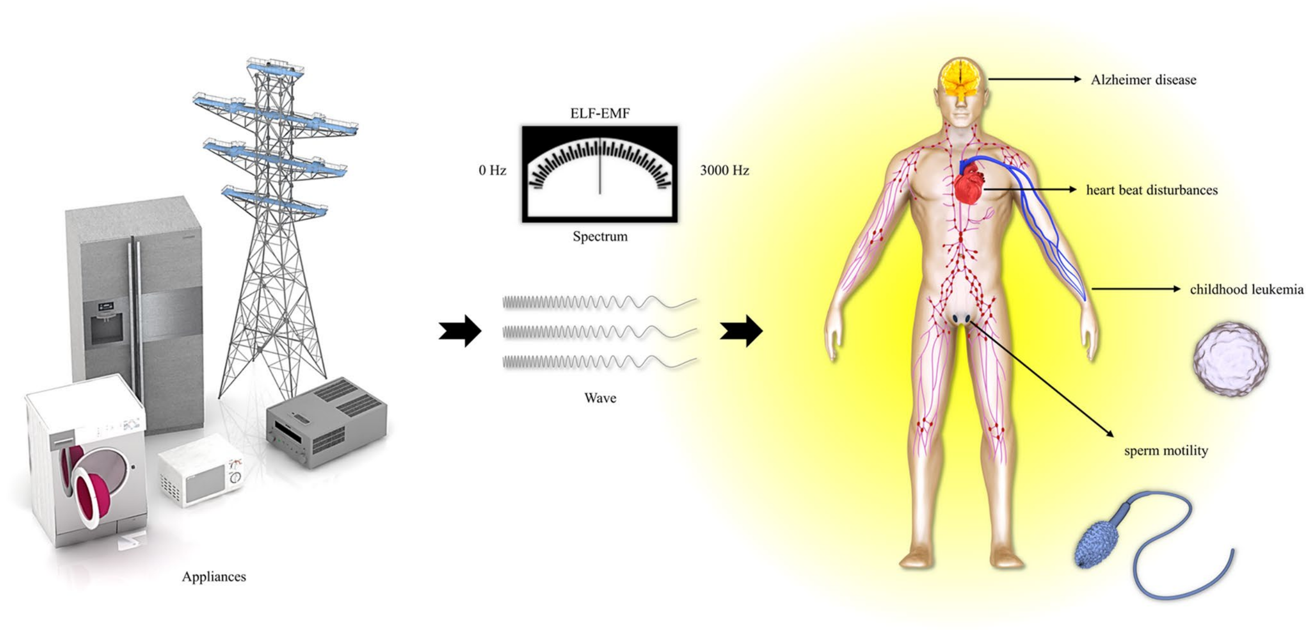

Fig. 1 Schematic illustration of ELF-EMF effects on human health. The excessive exposure to extremely low-frequency magnetic fields from power lines and electrical devices led to an increase in the risk of neurodegenerative disorders, cancer, cardiovascular diseases. It also has destructive effects on the reproductive system in which the most common are Alzheimer disease, childhood leukemia, heartbeat disturbances, and sperm motility, respectively

Although epidemiological studies provide destructive and beneficial effects of EMF, still there is no precise mechanism for explaining these processes. There are various underlying molecular mechanisms for ELF-EMF exposure; changes in free radical activities, including reactive oxygen and nitrogen species (ROS)/(RNS) species and endogenous antioxidant enzymes and compounds that maintain physiological free radical concentrations in cells. These changes can affect many physiological functions including DNA damage; immune response; inflammatory response; cell proliferation and differentiation; wound healing process; neural activities; and behavior [41]. Here, we review the role of ELF–EMF-mediated diseases, with a focus on the mechanism of action from published investigations. The weakness of these studies prevents any firm conclusion, and those effects are not that impressive.

ELF-EMF and cancer

Cancer is one of the significant problems of global health; it is believed that occupational and residential exposure to ELF-EMF can be carcinogenic. It was assumed that people living near power lines and who have the occupational and residential exposure to ELF-EMF have the chance of developing cancer, as described for the first time in 1979 for childhood leukemia [8]. Published meta-analyses between 1998 and 2000 concluded that there is positive evidence of elevated risk of childhood leukemia concerning residential proximity to high-current power lines [42]. A pooled analysis has concluded that exposure to ELF-EMF with ≥ 0.4 μT intensity increase twofold the risk of childhood leukemia; however, there is little evidence for linking childhood brain tumors and exposed to ELF-EMF with ≥ 0.4 μT intensity [11, 42]. Notwithstanding, some studies in various countries have not found a significant association between exposure to ELF-EMF and the risk of childhood leukemia based on job-exposure matrix (JEM) method [20, 21], which is a tool for assessing exposures to power–frequency (ELF-EMF) in retrospective epidemiologic studies (Table 1) [43, 44]. It may be due to the lack of appropriate animal models recapitulating the natural history of leukemia development. In childhood B-cell acute lymphoblastic leukemia (B-ALL), the common chromosomal alteration is the ETV6-RUNX1 fusion gene. The B-ALL mouse model for the human ETV6-RUNX1 + preleukemic state can provide an in vivo tool to probe the epidemiologically observed association of childhood leukemia with ELF-MF exposure [45]. Although some studies indicate associations between parental occupational ELF-MF exposure and childhood cancer [46]; however, there are inconsistent data regarding parental occupational exposure to ELF-MF and risk of ALL and acute myeloid leukemia (AML) in their offspring [47]. Findings from the Childhood Leukemia International Consortium (CLIC) did not find any associations between parental occupational ELF-MF exposure and childhood

leukemia [20]. Some studies have concluded occupational ELF-EMF was not associated with increased risk of childhood brain cancer, including meningioma [12, 48, 49]. The large-scale INTEROCC study using JEM to different levels of ELF-EMFs indicated positive associations between ELF and glioma [50]. A meta-analysis of breast cancer risk and ELF-EMF exposure indicates ELF-EMF can increase the risk of breast cancer in postmenopausal women [51]. At the same time, other studies revealed no significant increased risk of breast cancer [23, 52–54].

Mechanism of action

Despite many studies, the carcinogenic mechanisms related to ELF-EMF are still unclear. Ramazzini Institute in Italy has conducted the two large systematic and integrated projects of long-term bioassays on over 7000 Sprague Dawley rats to show the carcinogenic potential of non-ionizing radiation focusing on sinusoidal-50 Hz magnetic field (S-50 Hz MF) from electric power. According to this report, sinusoidal-50 Hz Magnetic Field (S-50 Hz MF) combined with acute exposure to gamma radiation for 104 weeks induces a significantly increased incidence of malignant tumors in male and female mice [55]. The exposure to ELF-EMF may result in various changes at the cellular level that may lead to cancer. To find out the mechanism of ELF-EMF related childhood leukemia in transgenic animals exposed to ELF-EMF, T-cells reduction, especially CD8 + cells has been observed [56]. Male C57BL/6 J mice exposed to 7.5 kHz MF at 12 or 120 µT for continuously 5 weeks, and rat primary astrocytes exposed to a 7.5 kHz MF at 30 or 300 µT for 24 h emerged the same results and proposed that magnetic field may increase cell proliferation or suppression of cell death [57]. In male Wistar rats, exposure to 5.5 mT ELF-EMF for 7 days induced an increased level of lipid peroxidation and superoxide anion production at the brain [58]. Exposure of human HaCaT cells for 144 h by 60 Hz ELF-EMF at 1.5 mT activates the ATM/ChK2 signaling pathway and increases the expression of p21 protein [59].

The mitogen-activated protein kinases (MAPKs), regulate essentially all stimulated cellular processes, include the extracellular signal-regulated kinases 1/2 (ERK1/2) that are responsive to extracellular cues. Single or repetitive exposure of HeLa and primary IMR-90 fibroblast for 168 h to a 60 Hz ELF-EMF at 6 mT neither induced DNA damage nor affected cell viability. However, continuous exposure increased the cell proliferation and phosphorylation of AKT and ErK1/2 and decreased the intracellular reactive oxygen species [60]. These results demonstrate that EMF uniformity at an extremely low frequency (ELF) is an important factor in the cellular effects of ELF-EMF. Kapri-Pardes et al. showed that the application of various field strengths ELF-MF and time periods to eight different cell types increase ERK1/2 phosphorylation. In this study, 0.15 µT ELF-MF had the lowest and ~10 µT had maximal effect on ERK1/2. However, the phosphorylation of ERK1/2 is likely too low to induce ELF-MF-dependent proliferation or oncogenic transformation [61].

In another study MCF10A, MCF7, Jurkat, and NIH3T3 cell line exposed for 4 or 16 h to a 60 Hz at 1mT; Jurkat and NIH3T3 cells showed no change, but MCF7 and MCF10A had a significant decrease in cell count and DNA synthesis followed by upregulation of PMA/P1 gene in MCF7 cells [62]. Exposure of five tumor-derived cell line (HL-606K562, MCF-7, A375, HH4) to 50 and 60 Hz of ELF-EMF at a 2, 20, 100, and 500 µT density for 3 days has demonstrated that this electromagnetic field does not affect cell growth or initial response of cell proliferation [63]. Exposure of human umbilical vein endothelial cells (HUVECs) to sinusoidal 50 Hz EMF at 1 mT for up to 12 h of EMF can cause an increase in cell proliferation and the phosphorylation and overall expression of VEGF receptor 2 (KDR/FIK-1) [64]. In myelogenous leukemia cell line K562 exposed to 50 Hz ELF-EMF at 1 mT, significant modulation of INOS, CAT, and Cytochrome P450 expression has been reported [65].

Despite the numerous studies, up to now, there is no specific and unique biological mechanism for the potential carcinogenesis of ELF-EMF. Anyway, as mentioned above, ELF-EMF can induce cancer through stimulatory and inhibitory effects on the immune system by affecting cell cycle regulators and signaling pathways, which potentially can affect cell proliferation and death. ELF-EMF can also interfere with angiogenesis activity by effecting the VEGF-related signaling pathway. Also, the ELF-EMF can modulate cell cycle, apoptosis, angiogenesis, invasion, and metastasis that lead to cancer by impacting the free radical production.

ELF-EMF and neurodegenerative diseases

Evidence from the studies in the last 10 years on ELF-EMF exposure on neurodegenerative diseases are inconsistent and conflicting [30, 31] (Table 1). The effect of ELF magnetic fields on neurodegenerative diseases was first described in 1996 by Eugene Sobel and colleagues. In this study, occupational exposure from moderate to high EMF was significantly associated with an increased risk of Alzheimer's disease (AD) [66]. The results of other studies were in line with this study and confirmed the impact of occupational exposure of ELF-EMF on AD development [13, 67]. A report from Switzerland indicates that residential magnetic field exposure from power lines has considerable effects on AD, senile dementia, amyotrophic lateral sclerosis (ALS), multiple sclerosis, and Parkinson's disease occurrence [68]. However, some studies report no association between occupational exposure to the ELF-EMF and Parkinson's condition

[31, 69]. In the Netherlands, a potential association between ALS-related mortality among men and occupational exposure to ELF-EMF has been reported [27].

Mechanism of action

Epidemiological and animal studies from research focusing on a possible contribution of ELF-EMF and the development of neurodegenerative disorders show conflicting data. Findings from primary mouse neuronal cultures indicate that prolonged exposure to the ELF-MFs changes the intracellular biochemical and epigenetic balance that might progressively boost neurons toward a degenerative phenotype. In neuronal-like SH-SY5Y neuroblastoma cells exposed to ELF-EMF (50 Hz/1 mT) the balance between generation and elimination of reactive oxygen species, and the balance between pro- and anti-inflammatory cytokines linked to oxidative stress, is maintained indicating that cells respond correctly to ELF-EMF exposure. Although in this study following 1 mT ELF exposure, 5-hydroxyindoleacetic acid/5-hydroxytryptamine ratio reflecting the rate of transmitter synthesis, catabolism and release are increased while matrix metalloproteinases that play critical roles in neuronal cell death were not significantly altered that did not provide a positive link between ELF-EMFs and neurodegeneration [70]. Also, ELF-MFs exposure (50-Hz (1 mT)) on SH-SY5Y cells and mouse primary cortical neurons reduce the expression of miR-34a that regulates neural stem cell differentiation [71].

One of the proposed mechanisms for AD is the decline of melatonin (MLT) function. As reported by Kolbabová et al. the secretion of salivary MLT is decreased following exposure of 1–2 months old cattle calves to 50 Hz-MF. According to this study, ELF exposure decrease and increase MLT secretion in winter and summer, respectively [72]. Overnight exposure of H4 neuroglioma cells to 50 Hz ELF-EMF at 3.1 mT intensity induces a significant increase of amyloid-beta peptide secretion that is in keeping with beta-amyloid effects on the risk of AD development [73]. Also, a recent study has shown that above 50 μT ELF-MF may induce chromosome instabilities as those found in AD patients [74]. In familial Amyotrophic Lateral Sclerosis (fALS) mouse model that carrying two mutant variants of the superoxide dismutase 1 (SOD1) gene, prolonged ELF stimulation (50 Hz, 1 mT) does not affect the viability and redox homeostasis, but significantly impairs the expression of iron-regulating genes (i.e., TfR1, MNFR1, and IRP1) [75]. There is growing evidence regarding ELF-EMF and neurodegenerative diseases; the sensible discrepancy is observed in the result of such studies. Most of them report a direct relationship between ELF-EMF and AD and ALS; however, there is little evidence that is negligible for connecting the ELF-EMF exposure and Parkinson’s disease needing further evaluation.

Cardiovascular diseases and ELF-EMF

Epidemiological studies report that exposure to ELF-EMF alters heart rate variability (HRV) as predictive of specific cardiovascular pathologies [76]. Heart rate variability (HRV) is the physiologic phenomenon of variation in the time interval between heartbeat and results from the action of neuronal and cardiovascular reflexes, including those involved in the control of temperature, blood pressure, and respiration. Laboratory research into the cardiovascular effects of ELF showed that HRV is reduced after nocturnal exposure to intermittent 60-Hz magnetic fields, and long-term exposure to ELF-MF may be associated with acute myocardial infarction and arrhythmia-related deaths [77]. However, a pooled analysis of laboratory studies did not show a consistent impact on cardiovascular effects in particular on microcirculatory indicators such as heart rate, HRV, and blood pressure [76].

In a community-based prospective cohort study, Koeman et al. reported no association between occupational ELF-MF exposure and CVD mortality, including ischaemic heart disease (IHD), acute myocardial infarction (AMI), subacute and chronic IHDs, arrhythmias, atherosclerosis and cerebrovascular diseases mortality [33]. In the study of Johansen et al. on the impact of occupational exposure to ELF-EMF on severe cardiac arrhythmia in employees, no increased risk of severe cardiac arrhythmia at 50 and 60 Hz of ELF-EMF was reported [78]. Another study report that occupational exposure to ELF-EMF could slightly increase the risk of acute myocardial infarction [79]. Similarly, the 60-Hz magnetic field at 1800-μT intensity did not affect desired cardiovascular parameters [80].

Mechanism of action

The cell membrane is the first-line and as a primary site of interaction with the low-frequency fields that interact with moving charges in cells and change their velocities. Therefore, the alterations in these charges and molecules affect the production of biological effects as the magnetic field interact with moving charges and change enzymatic activity and the distribution of ions and dipoles [81, 82]. Such change may pave molecular alterations in the cardiac function. EMF exposure can affect the structure and function of the cardiovascular system in rates and may facilitate myocardial infarction by the nuclear changing of cardiomyocytes. ELE exposure also induces increases in the activities of serum creatinine phosphokinase, lactate dehydrogenase and aspartate aminotransferase enzymes. Besides, it causes oxidative stress and impaired antioxidant system [81].

Exposure of cardiomyocytes isolated from neonatal Sprague–Dawley rats to 15, 50, 75, and 100 Hz ELF-EMF at 2 mT density indicated that ELF-EMF could regulate calcium-associated activities in cardiomyocytes [83]. Furthermore, exposure of the adult male Wistar rats to 60 Hz ELF-EMF at 2 mT for 2 h showed the possible decrease in the Glutathione (GSH) content in the heart [84] (Fig. 2). In guinea pigs exposed to 50 Hz MFs of 1, 2, and 3 mT for 4 h/day and 8 h/day for 5 day-duration, EMF affected the formation of free radicals and the activity of the antioxidant enzymes in the heart in proportion to intensity and duration of exposure [85]. Inducing apoptotis, dark brown stain muscle fiber nuclei, hyperemia muscle fiber degeneration, distortion of some cardiac myocytes,

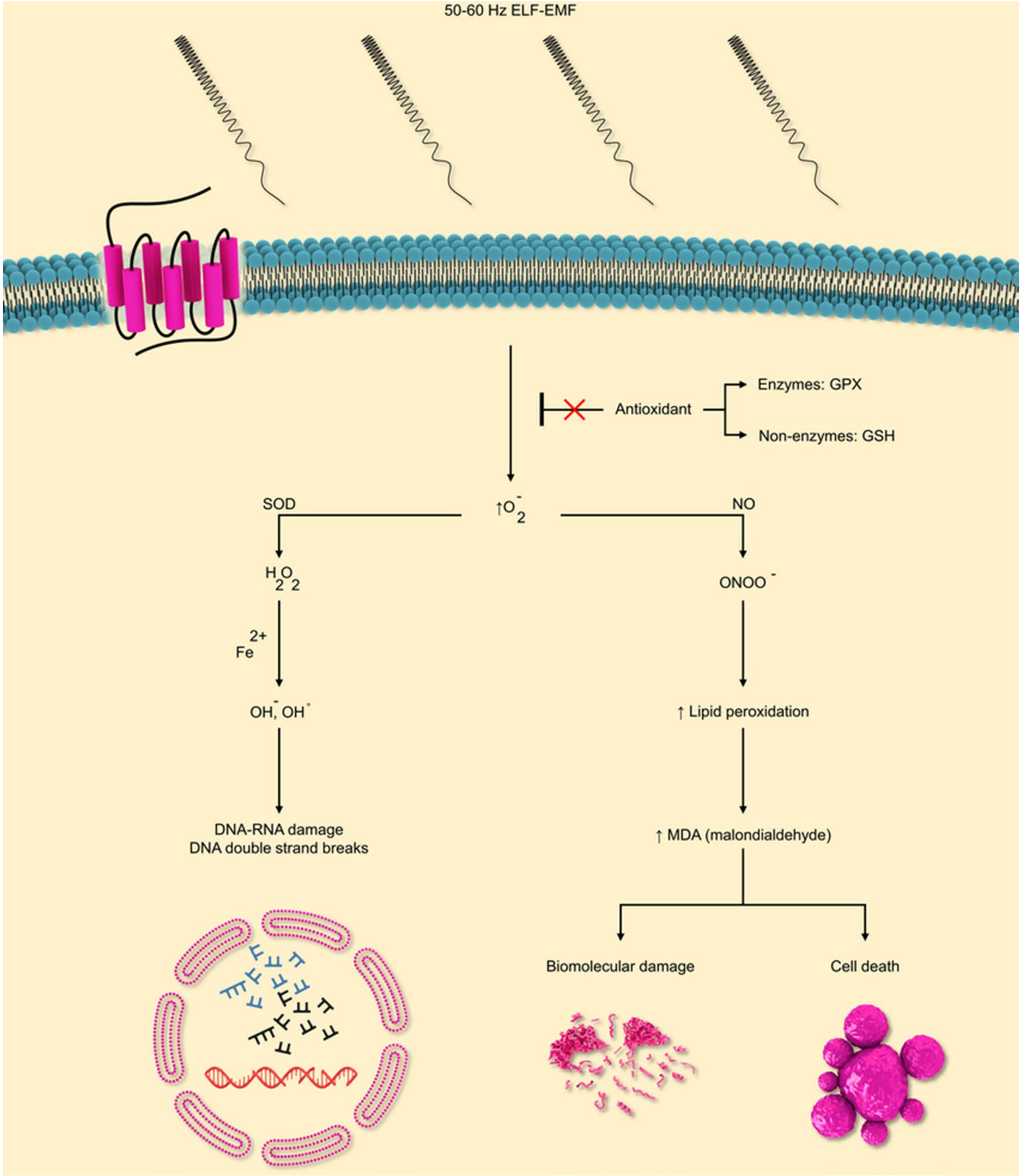

Fig. 2 The molecular mechanisms of ELF-EMF effects on cell function. The ELF-EMF decreases antioxidants concentration, which antioxidants have a defense mechanism against free radicals. The ELF-EMF could also induce the production of O_2^- in the cellular environment, that play a major role in oxidative damage by two pathway which includes excessive MDA production and Fenton pathway that is subsequently lead to biomolecular damage, DNA double-strand breaks, DNA/RNA damage, and cell death

mononuclear cellular infiltration and histological structure of the myocytes spaces are serious histopathological changes following ELE exposure in animal modesl [81, 82]. Despite extensive research efforts to date, there is no evidence to conclude that exposure to ELF-EMF can cause cardiovascular disease or cardiovascular-related mortality. It is believed that ELF-EMF may harm the heart function through free radical production and antioxidant enzymes reduction.

Reproductive system and ELF-EMF

The adverse effects of ELF-MF on reproductive health are controversial. Recent studies indicate that exposure to ELF-EMF is a negative factor that contributes to its role in miscarriage [86–88]. Women exposed to ELF-EMF during pregnancy may have a high risk of spontaneous abortion [89]. Fetal development may be affected by electric blankets and heated waterbeds usage among pregnant women as a consequence of the heat or electromagnetic field [90]. Nevertheless, ELF-EMF effects on fetal growth and development during human pregnancy have not yet been reported [34, 35, 91]. Also, exposure to ELF-EMF can increase sperm motility, depending on the field characteristics [92]. However, a recent study demonstrated that laptop computers potentially decrease sperm motility and increase DNA fragmentation in sperms [93]. In addition to that, no association has been reported for distance to residential exposure to ELF power transmission lines and stillbirth mortality; however, more efforts are needed for closer distances [94].

Mechanism of action

The isolated trophoblasts from first-trimester human chorionic villi exposed to a 50-Hz MF of 0.4 mT for 72 h suggest that MF inhibits the secretion of Human chorionic gonadotropin (hCG) and progesterone by trophoblast. However, to what extent apoptosis affected in trophoblasts is unknown [95]. In the study of Aydin et al. the adult Wistar female rats were kept with 7.5 m vertical distance to a power line and exposed for 1, 2 and 3 months continuously to ELF-EMF; significant changes in plasma catalase activities, without any effects on the morphological structure, weight of uterus and ovaries was reported [96]. In female Wistar albino rats exposed to 50-Hz 1 mT ELF-MF for 3 h/day for 50 and 100 days, the alterations in malondialdehyde (MDA) concentrations and ultrastructural changes or irregularity in nucleus and nucleolus in germinal epithelial cells of the rat ovaries and uterus has been reported [97] (Fig. 2). In the study of Liu et al. the mouse spermatocyte-derived GC-2 cells intermittent exposed to a 50-Hz ELF-EMF at 1, 2, and 3 mT intensities for 72 h. The ELF-EMF at 1 mT intensity reduced the expression of DNMT1 and DNMT3b and decreased genome-wide methylation, while 3 mT intensity induced DNMT1 expression and increased the genome-wide methylation [98]. In the study of Al-Akhras et al. the adult female Sprague–Dawley rats were exposed to a 50-Hz sinusoidal MF at 25 μT for 18 weeks, depending on the duration of exposure, alteration in LH, FSH, estrogen, and progesterone levels but no effect on ovary weight was reported [99]. In the study of Elbetieha et al. the adult male and female mice were exposed to a 50-Hz sinusoidal MF at 25 μT for 90 days, no effect on fertility and reproduction was found [100]. Also, in another study by Al-Akhras et al. exposure to 50-Hz ELF-EMF at 25 μT for 18 weeks on male rats showed no effect on the weight of the body and the testes. Nevertheless, an increase in the level of LH and a decrease in the sperm count, testosterone level, and the weights of seminal vesicles and preputial glands was observed [101]. In the zebrafish fertilized embryos exposed to 50-Hz sinusoidal MF at 30, 100, 200, 400, and 800 μT intensity for 96 h, adverse effect on the embryonic development by affecting the hatching, decreasing the heart rate, and inducing apoptosis were revealed [102]. In the study of Koziorowska et al. the porcine uterus tissues were exposed to 50 and 120 Hz EMF at 8 mT for 2, and 4 h in the presence or absence of progesterone, according to frequency and duration of exposure to ELF-EMF, alteration in the synthesis and release of oestradiol-17β (E2) in uterine tissues was observed [103]. In Mouse spermatocyte-derived GC-2 cells exposed to a 50-Hz ELF-EMF at 1, 2, and 3 mT intensities for 72 h, no effect on the growth, apoptosis or cell cycle was revelaed but in differenet intensities an altration on the miRNAs expression was reported [104]. El-Hussein et al. showed that exposure to 125-Hz PMF at 1.0 μT for 48 h induces developmental abnormalities [105]. According to the studies mentioned above, there are relevant evidence concerning-EMF-mediate embryonic developmental abnormalities. On the other hand, the in vivo and in vitro studies report that these fields can disrupt the balance of the synthesis and secretion of hormones in animals. Furthermore, ELF-EMF exposure can affect reproduction and fertility in animals. Further studies are needed to find out ELF-EMF effects on human reproduction and fertility.

ELF-EMF and genome instability

Numerous in vivo and in vitro studies have been carried out to investigate ELF-EMF influences on DNA damage [106–109] and DNA repair [110–112]. DNA damage can be one of the sources of genome instability. DNA damage can lead to cell death, aging, and cancer [113]. The most apparent ELF-EMF influence on DNA is strand breaks that include single-strand breaks (SSB), and double-strand

breaks (DSB) [114, 115]. Some studies show an association between ELF-EMF exposure and chromosomal damage [116], and oxidative DNA damage [117]. DNA strands breaks are produced as a result of endogenous agent effects; for instance, free radicals and exposure to exogenous agents such as ionizing and none-ionizing radiation and chemical. Exposure to ELF-EMF can cause DNA strand breaks and cell death through an increase in free radical formation [118–120] (Fig. 2). Findings from a cross-sectional study from Iran on power plant workers indicated occupational exposure to ELF-MF is associated with SSBs in DNA of the peripheral blood cells of power line workers [121].

A recent study by Wilson et al. reports that highly unstable expanded simple tandem repeat (ESTR) loci that have high rates of spontaneous mutation in mouse genome can provide new insights for the mutation induction—that is a hallmark of genomic instability—in the germline of mice exposed to ELF-EMF [122]. Albeit c-Myc activation is associated with transformation and genomic instability; however, in vitro exposure to 1 mT of 60 Hz magnetic field does not affect DNA double-strand breaks genomic instability mediated by c-Myc [123]. Monitoring of mutation rate in male mice exposed to 10, 100, or 300 µT of 50 Hz magnetic fields for 2 or 15 h did not show significant increases in the frequency ESTR mutation rate than germline, which indicates a controversy on ELF and genomic instability [122].

Another genome instability factor is mobile genetic elements, which can be affected by environmental factors [124, 125]. The human neuroblastoma BE(2) cells exposed to 50-Hz PMF at 1 mT for 48 h caused a decrease in retrotransposition events [126]. The mechanism of this effect is still unknown. Up to 45% of the human genome is made up of transposable elements (TEs), the extent to which TEs (in particular HERVs elements) may be affected upon exposure to ELF-EMF, and PMF are remained to be explored.

ELF-EMF therapy

Recent studies have provided some evidence in favor of ELF-EMF beneficial effects in treating some medical conditions such as tissue reconstruction [127–130]. A recent comprehensive study represents that in the wound healing process, ELF-EMF can drive the transition from a chronic pro-inflammatory state to an anti-inflammatory by modulation of cytokine profiles [131]. Another study showed similar results of the potential therapeutic role of ELF-EMF in wound healing processes by an increase in cell proliferation, volatility, and change in expression or activity in the mediators of inflammation, such as nitric oxide synthase (NOS) and other nitrogen intermediates and COX-9 (cyclooxygenase-2) [132]. A recent study found that ELF-EMF improves functional recovery in stroke patients by the effect on generation and metabolism of nitric oxide (NO) [39]. Exposed to ELF-PMF for six weeks and 8 h in a day in adult male diabetic rats caused the improvement of diabetic nephropathy (DN) symptoms [133]. IAD rat model exposed to 50-Hz ELF-EMF for 14 days showed an improvement in learning and memory impairments [134]. In a study of the diabetic wounded mice exposed to pulsed EMF, a positive effect resulted, including wound healing through increased expression of FGF2 and the prevention of tissue necrosis [135]. The Pulsed electromagnetic fields (PEMF) can cause healing tissues through increased angiogenesis by stimulating the endothelial release of FGF-2 [136]. A systematic review by Elmas represents evidence that exposure to EMF can treat myocardial ischemia [137].

Conclusion

This review suggest that exposure to ELF-EMF has an adverse biological effect, which depends on the current intensity, strength of the magnetic field, and duration of exposure. Accumulated epidemiologic evidence indicates a correlation between exposure to ELF-EMF and childhood cancer incidence, AD, and miscarriage. However, adult cancer does not show augmented risk caused by the ELF-EMF. Besides, no consistent evidence exists on the mortality of cardiovascular disease due to ELF-EMF exposure. Additional epidemiological studies in large study populations with improved exposure assessments are needed to clarify current inconclusive relationships. The in vivo and in vitro evidence confirms the association between DNA strands breaks and exposure to ELF-EMF. On the other hand, some studies show the therapeutic effect of these fields. There is a lack of a comprehensive mechanism for explaining the biological effect of ELF-EMF on human health. Eventually, more studies are needed to clarify the mechanisms of these magnetic fields.

Acknowledgements The authors thank the Faculty of Advanced Medical Sciences, Tabriz University of Medical Sciences, Tabriz, Iran. This project was financially supported by Biotechnology Research Center, Tabriz University of Medical Sciences, Tabriz, Iran (Grant/Award Number: 61741)

Compliance with ethical standards

Conflicts of interest The authors declare no conflict of interest.

Ethical approval This study was approved by the Tabriz University of Medical Sciences Human Research Ethics Committee in Iran (Ref no: IR.TBZMED.REC.1397.973).

Research involving human participants and/or animals In this review research paper, we had not any experiments on human and animal samples.

Informed consent In this study, we did not deal with the research participants, and we did not need for informed consent.

References

1. Tenforde T (1992) Biological interactions and potential health effects of extremely-low-frequency magnetic fields from power lines and other common sources. Annu Rev Public Health 13(1):173–196
2. Touitou Y, Selmaoui B (2012) The effects of extremely low-frequency magnetic fields on melatonin and cortisol, two marker rhythms of the circadian system. Dialogues Clin Neurosci 14(4):381
3. Lee S-K, Park S, Kim Y-W (2016) The Effects of extremely low-frequency magnetic fields on reproductive function in rodents, insights from animal reproduction. IntechOpen, London
4. Marcilio I, Habermann M, Gouveia N (2009) Campos magnéticos de frequência extremamente baixa e efeitos na saúde: revisão da literatura. Rev Bras Epidemiol 12:105–123
5. Ng K-H (2003) Non-ionizing radiations–sources, biological effects, emissions and exposures. In: Proceedings of the international conference on non-ionizing radiation at UNITEN
6. Feychting M, Ahlbom A, Kheifets L (2005) EMF and health. Annu Rev Public Health 26:165–189
7. D'Angelo C, Costantini E, Kamal M, Reale M (2015) Experimental model for ELF-EMF exposure: concern for human health. Saudi J Biol Sci 22(1):75–84
8. Wertheimer N, Leeper E (1979) Electrical wiring configurations and childhood cancer. Am J Epidemiol 109(3):273–284
9. Cancer IAFRO (2013) Non-ionizing radiation, part 2: radiofrequency electromagnetic fields. IARC Monogr Eval Carcinog Risks Hum 102:1–421
10. Schmiedel S, Blettner M (2010) The association between extremely low-frequency electromagnetic fields and childhood leukaemia in epidemiology: enough is enough? Br J Cancer 103:931
11. Schüz J (2011) Exposure to extremely low-frequency magnetic fields and the risk of childhood cancer: update of the epidemiological evidence. Prog Biophys Mol Biol 107(3):339–342
12. Carlberg M, Koppel T, Ahonen M, Hardell L (2018) Case-control study on occupational exposure to extremely low-frequency electromagnetic fields and the association with meningioma. Biomed Res Int. https://doi.org/10.1155/2018/5912394
13. Jalilian H, Teshnizi SH, Röösli M, Neghab M (2018) Occupational exposure to extremely low frequency magnetic fields and risk of Alzheimer disease: a systematic review and meta-analysis. Neurotoxicology 69:242–252
14. Turner MC, Benke G, Bowman JD, Figuerola J, Fleming S, Hours M, Kincl L, Krewski D, McLean D, Parent ME, Richardson L, Sadetzki S, Schlaefer K, Schlehofer B, Schuz J, Siemiatycki J, Tongeren MV, Cardis E (2017) Interactions between occupational exposure to extremely low frequency magnetic fields and chemicals for brain tumour risk in the INTEROCC study. Occup Environ Med 74(11):802–809. https://doi.org/10.1136/oemed-2016-104080
15. Carlberg M, Koppel T, Ahonen M, Hardell L (2017) Case-control study on occupational exposure to extremely low-frequency electromagnetic fields and glioma risk. Am J Ind Med 60(5):494–503. https://doi.org/10.1002/ajim.22707
16. Turner MC, Benke G, Bowman JD, Figuerola J, Fleming S, Hours M, Kincl L, Krewski D, McLean D, Parent ME, Richardson L, Sadetzki S, Schlaefer K, Schlehofer B, Schüz J, Siemiatycki J, van Tongeren M, Cardis E (2014) Occupational exposure to extremely low-frequency magnetic fields and brain tumor risks in the INTEROCC study. Cancer Epidemiol Biomark Prev 23(9):1863–1872. https://doi.org/10.1158/1055-9965.epi-14-0102
17. Oraby T, Sivaganesan S, Bowman JD, Kincl L, Richardson L, McBride M, Siemiatycki J, Cardis E, Krewski D (2018) Berkson error adjustment and other exposure surrogates in occupational case-control studies, with application to the Canadian INTEROCC study. J Eposure Sci Environ Epidemiol 28(3):251–258. https://doi.org/10.1038/jes.2017.2
18. Li P, McLaughlin J, Infante-Rivard C (2009) Maternal occupational exposure to extremely low frequency magnetic fields and the risk of brain cancer in the offspring. Cancer Causes Control CCC 20(6):945–955. https://doi.org/10.1007/s10552-009-9311-5
19. Huss A, Spoerri A, Egger M, Kromhout H, Vermeulen R (2018) Occupational extremely low frequency magnetic fields (ELF-MF) exposure and hematolymphopoietic cancers—Swiss National Cohort analysis and updated meta-analysis. Environ Res 164:467–474. https://doi.org/10.1016/j.envres.2018.03.022
20. Talibov M, Olsson A, Bailey H, Erdmann F, Metayer C, Magnani C, Petridou E, Auvinen A, Spector L, Clavel J, Roman E, Dockerty J, Nikkila A, Lohi O, Kang A, Psaltopoulou T, Miligi L, Vila J, Cardis E, Schuz J (2019) Parental occupational exposure to low-frequency magnetic fields and risk of leukaemia in the offspring: findings from the Childhood Leukaemia International Consortium (CLIC). Occup Environ Med 76(10):746–753. https://doi.org/10.1136/oemed-2019-105706
21. Su L, Fei Y, Wei X, Guo J, Jiang X, Lu L, Chen G (2016) Associations of parental occupational exposure to extremely low-frequency magnetic fields with childhood leukemia risk. Leuk Lymphoma 57(12):2855–2862. https://doi.org/10.3109/10428194.2016.1165812
22. Koeman T, van den Brandt PA, Slottje P, Schouten LJ, Goldbohm RA, Kromhout H, Vermeulen R (2014) Occupational extremely low-frequency magnetic field exposure and selected cancer outcomes in a prospective Dutch cohort. Cancer Causes Control CCC 25(2):203–214. https://doi.org/10.1007/s10552-013-0322-x
23. Li W, Ray RM, Thomas DB, Yost M, Davis S, Breslow N, Gao DL, Fitzgibbons ED, Camp JE, Wong E, Wernli KJ, Checkoway H (2013) Occupational exposure to magnetic fields and breast cancer among women textile workers in Shanghai. China Am J Epidemiol 178(7):1038–1045. https://doi.org/10.1093/aje/kwt161
24. Zhou H, Chen G, Chen C, Yu Y, Xu Z (2012) Association between extremely low-frequency electromagnetic fields occupations and amyotrophic lateral sclerosis: a meta-analysis. PLoS ONE 7(11):e48354. https://doi.org/10.1371/journal.pone.0048354
25. Parlett LE, Bowman JD, van Wijngaarden E (2011) Evaluation of occupational exposure to magnetic fields and motor neuron disease mortality in a population-based cohort. J Occup Environ Med 53(12):1447–1451. https://doi.org/10.1097/JOM.0b013e318237a1d0
26. Huss A, Spoerri A, Egger M, Kromhout H, Vermeulen R (2015) Occupational exposure to magnetic fields and electric shocks and risk of ALS: the Swiss National Cohort. Amyotroph Lateral Scler Frontotemporal Degener 16(1–2):80–85. https://doi.org/10.3109/21678421.2014.954588
27. Koeman T, Slottje P, Schouten LJ, Peters S, Huss A, Veldink JH, Kromhout H, van den Brandt PA, Vermeulen R (2017) Occupational exposure and amyotrophic lateral sclerosis in a prospective cohort. Occup Environ Med 74(8):578–585. https://doi.org/10.1136/oemed-2016-103780
28. Peters S, Visser AE, D'Ovidio F, Beghi E, Chiò A, Logroscino G, Hardiman O, Kromhout H, Huss A, Veldink J, Vermeulen R, van den Berg LH (2019) Associations of electric shock and

extremely low-frequency magnetic field exposure with the risk of amyotrophic lateral sclerosis. Am J Epidemiol 188(4):796–805. https://doi.org/10.1093/aje/kwy287
29. Pedersen C, Poulsen AH, Rod NH, Frei P, Hansen J, Grell K, Raaschou-Nielsen O, Schuz J, Johansen C (2017) Occupational exposure to extremely low-frequency magnetic fields and risk for central nervous system disease: an update of a Danish cohort study among utility workers. Int Arch Occup Environ Health 90(7):619–628. https://doi.org/10.1007/s00420-017-1224-0
30. Vergara X, Kheifets L, Greenland S, Oksuzyan S, Cho YS, Mezei G (2013) Occupational exposure to extremely low-frequency magnetic fields and neurodegenerative disease: a meta-analysis. J Occup Environ Med 55(2):135–146. https://doi.org/10.1097/JOM.0b013e31827f37f8
31. van der Mark M, Vermeulen R, Nijssen PC, Mulleners WM, Sas AM, van Laar T, Kromhout H, Huss A (2015) Extremely low-frequency magnetic field exposure, electrical shocks and risk of Parkinson's disease. Int Arch Occup Environ Health 88(2):227–234. https://doi.org/10.1007/s00420-014-0949-2
32. Brouwer M, Koeman T, van den Brandt PA, Kromhout H, Schouten LJ, Peters S, Huss A, Vermeulen R (2015) Occupational exposures and Parkinson's disease mortality in a prospective Dutch cohort. Occup Environ Med 72(6):448–455. https://doi.org/10.1136/oemed-2014-102209
33. Koeman T, Slottje P, Kromhout H, Schouten LJ, Goldbohm RA, van den Brandt PA, Vermeulen R (2013) Occupational exposure to extremely low-frequency magnetic fields and cardiovascular disease mortality in a prospective cohort study. Occup Environ Med 70(6):402–407
34. Migault L, Garlantezec R, Piel C, Marchand-Martin L, Orazio S, Cheminat M, Zaros C, Carles C, Cardis E, Ancel PY, Charles MA, de Seze R, Baldi I, Bouvier G (2020) Maternal cumulative exposure to extremely low frequency electromagnetic fields, prematurity and small for gestational age: a pooled analysis of two birth cohorts. Occup Environ Med 77(1):22–31. https://doi.org/10.1136/oemed-2019-105785
35. Migault L, Piel C, Carles C, Delva F, Lacourt A, Cardis E, Zaros C, de Seze R, Baldi I, Bouvier G (2018) Maternal cumulative exposure to extremely low frequency electromagnetic fields and pregnancy outcomes in the Elfe cohort. Environ Int 112:165–173. https://doi.org/10.1016/j.envint.2017.12.025
36. Bowman JD, Touchstone JA, Yost MG (2007) A population-based job exposure matrix for power-frequency magnetic fields. J Occup Environ Hyg 4(9):715–728. https://doi.org/10.1080/15459620701528001
37. Jauchem JR (1997) Exposure to extremely-low-frequency electromagnetic fields and radiofrequency radiation: cardiovascular effects in humans. Int Arch Occup Environ Health 70(1):9–21
38. Gye MC, Park CJ (2012) Effect of electromagnetic field exposure on the reproductive system. Clin Exp Reprod Med 39(1):1–9
39. Cichoń N, Czarny P, Bijak M, Miller E, Śliwiński T, Szemraj J, Saluk-Bijak J (2017) Benign effect of extremely low-frequency electromagnetic field on brain plasticity assessed by nitric oxide metabolism during poststroke rehabilitation. Oxidat Med Cell Longev. https://doi.org/10.1155/2017/2181942
40. Mansourian M, Firoozabadi M, Hassan ZM (2020) The role of 217-Hz ELF magnetic fields emitted from GSM mobile phones on electrochemotherapy mechanisms. Electromagn Biol Med. https://doi.org/10.1080/15368378.2020.1762635
41. Lai H (2019) Exposure to static and extremely-low frequency electromagnetic fields and cellular free radicals. Electromagn Biol Med 38(4):231–248. https://doi.org/10.1080/15368378.2019.1656645
42. Carpenter DO (2019) Extremely low frequency electromagnetic fields and cancer: how source of funding affects results. Environ Res 178:108688. https://doi.org/10.1016/j.envres.2019.108688
43. Pedersen C, Raaschou-Nielsen O, Rod NH, Frei P, Poulsen AH, Johansen C, Schüz J (2014) Distance from residence to power line and risk of childhood leukemia: a population-based case–control study in Denmark. Cancer Causes Control 25(2):171–177
44. Sermage-Faure C, Demoury C, Rudant J, Goujon-Bellec S, Guyot-Goubin A, Deschamps F, Hemon D, Clavel J (2013) Childhood leukaemia close to high-voltage power lines–the Geocap study, 2002–2007. Br J Cancer 108(9):1899
45. Campos-Sanchez E, Vicente-Duenas C, Rodriguez-Hernandez G, Capstick M, Kuster N, Dasenbrock C, Sanchez-Garcia I, Cobaleda C (2019) Novel ETV6-RUNX1 mouse model to study the role of ELF-MF in childhood B-acute lymphoblastic leukemia: a pilot study. Bioelectromagnetics 40(5):343–353. https://doi.org/10.1002/bem.22193
46. Pearce MS, Hammal DM, Dorak MT, McNally RJ, Parker L (2007) Paternal occupational exposure to electro-magnetic fields as a risk factor for cancer in children and young adults: a case-control study from the North of England. Pediatr Blood Cancer 49(3):280–286. https://doi.org/10.1002/pbc.21021
47. Reid A, Glass DC, Bailey HD, Milne E, de Klerk NH, Downie P, Fritschi L (2011) Risk of childhood acute lymphoblastic leukaemia following parental occupational exposure to extremely low frequency electromagnetic fields. Br J Cancer 105(9):1409–1413. https://doi.org/10.1038/bjc.2011.365
48. Mezei G, Gadallah M, Kheifets L (2008) Residential magnetic field exposure and childhood brain cancer: a meta-analysis. Epidemiology 19:424–430
49. Kheifets L, Ahlbom A, Crespi C, Feychting M, Johansen C, Monroe J, Murphy M, Oksuzyan S, Preston-Martin S, Roman E (2010) A pooled analysis of extremely low-frequency magnetic fields and childhood brain tumors. Am J Epidemiol 172(7):752–761
50. Turner MC, Benke G, Bowman JD, Figuerola J, Fleming S, Hours M, Kincl L, Krewski D, McLean D, Parent M-E (2014) Occupational exposure to extremely low-frequency magnetic fields and brain tumor risks in the INTEROCC study. Cancer Epidemiol Prev Biomark 23(9):1863–1872
51. Zhao G, Lin X, Zhou M, Zhao J (2014) Relationship between exposure to extremely low-frequency electromagnetic fields and breast cancer risk: a meta-analysis. Eur J Gynaecol Oncol 35(3):264–269
52. Sorahan T (2012) Cancer incidence in UK electricity generation and transmission workers, 1973–2008. Occup Med 62(7):496–505
53. Elliott P, Shaddick G, Douglass M, de Hoogh K, Briggs DJ, Toledano MB (2013) Adult cancers near high-voltage overhead power lines. Epidemiology 24:184–190
54. Koeman T, Van Den Brandt PA, Slottje P, Schouten LJ, Goldbohm RA, Kromhout H, Vermeulen R (2014) Occupational extremely low-frequency magnetic field exposure and selected cancer outcomes in a prospective Dutch cohort. Cancer Causes Control 25(2):203–214
55. Soffritti M, Giuliani L (2019) The carcinogenic potential of non-ionizing radiations: the cases of S-50 Hz MF and 1.8 GHz GSM radiofrequency radiation. Basic Clin Pharmacol Toxicol 125(Suppl 3):58–69. https://doi.org/10.1111/bcpt.13215
56. Schüz J, Dasenbrock C, Ravazzani P, Röösli M, Schär P, Bounds PL, Erdmann F, Borkhardt A, Cobaleda C, Fedrowitz M (2016) Extremely low-frequency magnetic fields and risk of childhood leukemia: a risk assessment by the ARIMMORA consortium. Bioelectromagnetics 37(3):183–189
57. Herrala M, Kumari K, Koivisto H, Luukkonen J, Tanila H, Naarala J, Juutilainen J (2018) Genotoxicity of intermediate frequency magnetic fields in vitro and in vivo. Environ Res 167:759–769

58. Jelenković A, Janać B, Pešić V, Jovanović D, Vasiljević I, Prolić Z (2006) Effects of extremely low-frequency magnetic field in the brain of rats. Brain Res Bull 68(5):355–360
59. Huang C-Y, Chang C-W, Chen C-R, Chuang C-Y, Chiang C-S, Shu W-Y, Fan T-C, Hsu IC (2014) Extremely low-frequency electromagnetic fields cause G1 phase arrest through the activation of the ATM-Chk2-p21 pathway. PLoS ONE 9(8):e104732
60. Song K, Im SH, Yoon YJ, Kim HM, Lee HJ, Park GS (2018) A 60 Hz uniform electromagnetic field promotes human cell proliferation by decreasing intracellular reactive oxygen species levels. PLoS ONE 13(7):e0199753
61. Kapri-Pardes E, Hanoch T, Maik-Rachline G, Murbach M, Bounds PL, Kuster N, Seger R (2017) Activation of signaling cascades by weak extremely low frequency electromagnetic fields. Cell Physiol Biochem 43(4):1533–1546
62. Lee HC, Hong MN, Jung SH, Kim BC, Suh YJ, Ko YG, Lee YS, Lee BY, Cho YG, Myung SH (2015) Effect of extremely low frequency magnetic fields on cell proliferation and gene expression. Bioelectromagnetics 36(7):506–516
63. Yoshizawa H, Tsuchiya T, Mizoe H, Ozeki H, Kanao S, Yomori H, Sakane C, Hasebe S, Motomura T, Yamakawa T (2002) No effect of extremely low-frequency magnetic field observed on cell growth or initial response of cell proliferation in human cancer cell lines. Bioelectromagnetics 23(5):355–368
64. Delle Monache S, Alessandro R, Iorio R, Gualtieri G, Colonna R (2008) Extremely low frequency electromagnetic fields (ELF-EMFs) induce in vitro angiogenesis process in human endothelial cells. Bioelectromagnetics 29(8):640–648
65. Patruno A, Tabrez S, Pesce M, Shakil S, Kamal MA, Reale M (2015) Effects of extremely low frequency electromagnetic field (ELF-EMF) on catalase, cytochrome P450 and nitric oxide synthase in erythro-leukemic cells. Life Sci 121:117–123
66. Sobel E, Davanipour Z (1996) Electromagnetic field exposure may cause increased production of amyloid beta and eventually lead to Alzheimer's disease. Neurology 47(6):1594–1600
67. Davanipour Z, Tseng C-C, Lee P-J, Sobel E (2007) A case-control study of occupational magnetic field exposure and Alzheimer's disease: results from the California Alzheimer's Disease Diagnosis and Treatment Centers. BMC Neurol 7(1):13
68. Huss A, Spoerri A, Egger M, Röösli M, Study SNC (2008) Residence near power lines and mortality from neurodegenerative diseases: longitudinal study of the Swiss population. Am J Epidemiol 169(2):167–175
69. Huss A, Koeman T, Kromhout H, Vermeulen R (2015) Extremely Low frequency magnetic field exposure and Parkinson's disease—a systematic review and meta-analysis of the data. Int J Environ Res Public Health 12(7):7348–7356
70. Reale M, D'Angelo C, Costantini E, Tata AM, Regen F, Hellmann-Regen J (2016) Effect of environmental extremely low-frequency electromagnetic fields exposure on inflammatory mediators and serotonin metabolism in a human neuroblastoma cell Line. CNS Neurol Disord 15(10):1203–1215. https://doi.org/10.2174/1871527315666160920113407
71. Consales C, Cirotti C, Filomeni G, Panatta M, Butera A, Merla C, Lopresto V, Pinto R, Marino C, Benassi B (2018) Fifty-hertz magnetic field affects the epigenetic modulation of the miR-34b/c in neuronal cells. Mol Neurobiol 55(7):5698–5714. https://doi.org/10.1007/s12035-017-0791-0
72. Kolbabová T, Malkemper EP, Bartoš L, Vanderstraeten J, Turčáni M, Burda H (2015) Effect of exposure to extremely low frequency magnetic fields on melatonin levels in calves is seasonally dependent. Sci Rep 5:14206
73. Del Giudice E, Facchinetti F, Nofrate V, Boccaccio P, Minelli T, Dam M, Leon A, Moschini G (2007) Fifty Hertz electromagnetic field exposure stimulates secretion of β-amyloid peptide in cultured human neuroglioma. Neurosci Lett 418(1):9–12
74. Maes A, Anthonissen R, Wambacq S, Simons K, Verschaeve L (2016) The cytome assay as a tool to investigate the possible association between exposure to extremely low frequency magnetic fields and an increased risk for Alzheimer's Disease. J Alzheimer's Dis JAD 50(3):741–749. https://doi.org/10.3233/jad-150669
75. Consales C, Panatta M, Butera A, Filomeni G, Merla C, Carri MT, Marino C, Benassi B (2019) 50-Hz magnetic field impairs the expression of iron-related genes in the in vitro SOD1(G93A) model of amyotrophic lateral sclerosis. Int J Radiat Biol 95(3):368–377. https://doi.org/10.1080/09553002.2019.1552378
76. McNamee DA, Legros AG, Krewski DR, Wisenberg G, Prato FS, Thomas AW (2009) A literature review: the cardiovascular effects of exposure to extremely low frequency electromagnetic fields. Int Arch Occup Environ Health 82(8):919–933. https://doi.org/10.1007/s00420-009-0404-y
77. Sastre A, Cook MR, Graham C (1998) Nocturnal exposure to intermittent 60 Hz magnetic fields alters human cardiac rhythm. Bioelectromagnetics 19(2):98–106
78. Johansen C, Feychting M, Møller M, Arnsbo P, Ahlbom A, Olsen JH (2002) Risk of severe cardiac arrhythmia in male utility workers: a nationwide Danish cohort study. Am J Epidemiol 156(9):857–861
79. Håkansson N, Gustavsson P, Sastre A, Floderus B (2003) Occupational exposure to extremely low frequency magnetic fields and mortality from cardiovascular disease. Am J Epidemiol 158(6):534–542
80. McNamee DA, Corbacio M, Weller JK, Brown S, Prato FS, Thomas AW, Legros AG (2010) The cardiovascular response to an acute 1800-μT, 60-Hz magnetic field exposure in humans. Int Arch Occup Environ Health 83(4):441–454
81. Azab AE, Ebrahim SA (2017) Exposure to electromagnetic fields induces oxidative stress and pathophysiological changes in the cardiovascular system. J Appl Biotechnol Bioeng 4(4):540
82. Goodman R, Blank M (2002) Insights into electromagnetic interaction mechanisms. J Cell Physiol 192(1):16–22. https://doi.org/10.1002/jcp.10098
83. Wei J, Sun J, Xu H, Shi L, Sun L, Zhang J (2015) Effects of extremely low frequency electromagnetic fields on intracellular calcium transients in cardiomyocytes. Electromagn Biol Med 34(1):77–84
84. Martínez-Sámano J, Torres-Durán PV, Juárez-Oropeza MA, Elías-Viñas D, Verdugo-Díaz L (2010) Effects of acute electromagnetic field exposure and movement restraint on antioxidant system in liver, heart, kidney and plasma of Wistar rats: a preliminary report. Int J Radiat Biol 86(12):1088–1094
85. Canseven AG, Coskun S, Seyhan N (2008) Effects of various extremely low frequency magnetic fields on the free radical processes, natural antioxidant system and respiratory burst system activities in the heart and liver tissues. Indian J Biochem Biophys 45:326
86. Lee GM, Neutra RR, Hristova L, Yost M, Hiatt RA (2002) A nested case-control study of residential and personal magnetic field measures and miscarriages. Epidemiology 13(1):21–31
87. Hardell L, Sage C (2008) Biological effects from electromagnetic field exposure and public exposure standards. Biomed Pharmacother 62(2):104–109
88. Wang Q, Cao Z, Qu Y, Peng X, Guo S, Chen L (2013) Residential exposure to 50 Hz magnetic fields and the association with miscarriage risk: a 2-year prospective cohort study. PLoS ONE 8(12):e82113
89. Shamsi Mahmoudabadi F, Ziaei S, Firoozabadi M, Kazemnejad A (2013) Exposure to extremely low frequency electromagnetic fields during pregnancy and the risk of spontaneous abortion: a case-control study. J Res Health Sci 13(2):131–134

90. Wertheimer N, Leeper E (1986) Possible effects of electric blankets and heated waterbeds on fetal development. Bioelectromagnetics 7(1):13–22
91. Mahram M, Ghazavi M (2013) The effect of extremely low frequency electromagnetic fields on pregnancy and fetal growth, and development. Arch Iran Med 16(4):221
92. Iorio R, Scrimaglio R, Rantucci E, Monache SD, Di Gaetano A, Finetti N, Francavilla F, Santucci R, Tettamanti E, Colonna R (2007) A preliminary study of oscillating electromagnetic field effects on human spermatozoon motility. Bioelectromagnetics 28(1):72–75
93. Avendano C, Mata A, Sarmiento CAS, Doncel GF (2012) Use of laptop computers connected to internet through Wi-Fi decreases human sperm motility and increases sperm DNA fragmentation. Fertil Steril 97(1):39–45
94. Auger N, Park AL, Yacouba S, Goneau M, Zayed J (2012) Stillbirth and residential proximity to extremely low frequency power transmission lines: a retrospective cohort study. Occup Environ Med 69(2):147–149
95. Sun W, Tan Q, Pan Y, Fu Y, Sun H, Chiang H (2010) Effects of 50-Hz magnetic field exposure on hormone secretion and apoptosis-related gene expression in human first trimester villous trophoblasts in vitro. Bioelectromagnetics 31(7):566–572
96. Aydin M, Cevik A, Kandemir F, Yuksel M, Apaydin A (2009) Evaluation of hormonal change, biochemical parameters, and histopathological status of uterus in rats exposed to 50-Hz electromagnetic field. Toxicol Ind Health 25(3):153–158
97. Aksen F, Akdag MZ, Ketani A, Yokus B, Kaya A, Dasdag S (2006) Effect of 50-Hz 1-mT magnetic field on the uterus and ovaries of rats (Electronmicroscopy evaluation). Med Sci Monitor 12(6):215–220
98. Liu Y, Liu W-B, Liu K-J, Ao L, Zhong JL, Cao J, Liu J-Y (2015) Effect of 50 Hz extremely low-frequency electromagnetic fields on the DNA methylation and DNA methyltransferases in mouse spermatocyte-derived cell line GC-2. Biomed Res Int. https://doi.org/10.1155/2015/237183
99. Al-Akhras M-A (2008) Influence of 50 Hz magnetic field on sex hormones and body, uterine, and ovarian weights of adult female rats. Electromagn Biol Med 27(2):155–163
100. Elbetieha A, AL-Akhras MDA, Darmani H (2002) Long-term exposure of male and female mice to 50 Hz magnetic field: Effects on fertility. Bioelectromagnetics 23(2):168–172
101. Al-Akhras MDA, Darmani H, Elbetieha A (2006) Influence of 50 Hz magnetic field on sex hormones and other fertility parameters of adult male rats. Bioelectromagnetics 27(2):127–131
102. Li Y, Liu X, Liu K, Miao W, Zhou C, Li Y, Wu H (2014) Extremely low-frequency magnetic fields induce developmental toxicity and apoptosis in zebrafish (Danio rerio) embryos. Biol Trace Elem Res 162(1–3):324–332
103. Koziorowska A, Waszkiewicz EM, Romerowicz-Misielak M, Zglejc-Waszak K, Franczak A (2018) Extremely low-frequency electromagnetic field (EMF) generates alterations in the synthesis and secretion of oestradiol-17β (E2) in uterine tissues: an in vitro study. Theriogenology 110:86–95
104. Liu Y, Liu W-b, Liu K-j, Ao L, Cao J, Zhong JL, Liu J-y (2015) Extremely low-frequency electromagnetic fields affect the miRNA-mediated regulation of signaling pathways in the GC-2 cell line. PLoS ONE 10(10):e0139949
105. El-Hussein A, Kasem M, Saad AEH, Hamblin MR, Harith M (2018) An extremely low frequency-weak magnetic field can induce alterations in a biological system: a case study in chick embryo development. Prog Biophys Mol Biol S0079-S6107(18):30202–30205
106. Svedenstål B, Johanson K, Mild KH (1999) DNA damage induced in brain cells of CBA mice exposed to magnetic fields. vivo 13(6):551–552
107. Delimaris J, Tsilimigaki S, Messini-Nicolaki N, Ziros E, Piperakis S (2006) Effects of pulsed electric fields on DNA of human lymphocytes. Cell Biol Toxicol 22(6):409–415
108. Ahuja Y, Vijayashree B, Saran R, Jayashri E, Manoranjani J, Bhargava S (1999) In vitro effects of low-level, low-frequency electromagnetic fields on DNA damage in human leucocytes by comet assay. Indian J Biochem Biophys 36:318
109. Lourencini da Silva R, Albano F, Lopes dos Santos L, Tavares A Jr, Felzenszwalb I (2000) The effect of electromagnetic field exposure on the formation of DNA lesions. Redox Rep 5(5):299–301
110. Schmitz C, Keller E, Freuding T, Silny J, Korr H (2004) 50-Hz magnetic field exposure influences DNA repair and mitochondrial DNA synthesis of distinct cell types in brain and kidney of adult mice. Acta Neuropathol 107(3):257–264
111. Robison JG, Pendleton AR, Monson KO, Murray BK, O'Neill KL (2002) Decreased DNA repair rates and protection from heat induced apoptosis mediated by electromagnetic field exposure. Bioelectromagnetics 23(2):106–112
112. Chow K-C, Tung WL (2000) Magnetic field exposure enhances DNA repair through the induction of DnaK/J synthesis. FEBS Lett 478(1–2):133–136
113. Phillips JL, Singh NP, Lai H (2009) Electromagnetic fields and DNA damage. Pathophysiology 16(2–3):79–88
114. Ivancsits S, Diem E, Pilger A, Rüdiger HW, Jahn O (2002) Induction of DNA strand breaks by intermittent exposure to extremely-low-frequency electromagnetic fields in human diploid fibroblasts. Mutat Res 519(1–2):1–13
115. Ivancsits S, Pilger A, Diem E, Jahn O, Rüdiger HW (2005) Cell type-specific genotoxic effects of intermittent extremely low-frequency electromagnetic fields. Mutat Res 583(2):184–188
116. Winker R, Ivancsits S, Pilger A, Adlkofer F, Rüdiger H (2005) Chromosomal damage in human diploid fibroblasts by intermittent exposure to extremely low-frequency electromagnetic fields. Mutat Res 585(1–2):43–49
117. Yokus B, Cakir DU, Akdag MZ, Sert C, Mete N (2005) Oxidative DNA damage in rats exposed to extremely low frequency electro magnetic fields. Free Radical Res 39(3):317–323
118. Lai H, Singh NP (2004) Magnetic-field-induced DNA strand breaks in brain cells of the rat. Environ Health Perspect 112(6):687–694
119. Lai H, Singh NP (1997) Melatonin and N-tert-butyl-α-phenylnitrone block 60-Hz magnetic field-induced DNA single and double strand breaks in rat brain cells. J Pineal Res 22(3):152–162
120. Jajte J, Zmyślony M, Palus J, Dziubałtowska E, Rajkowska E (2001) Protective effect of melatonin against in vitro iron ions and 7 mT 50 Hz magnetic field-induced DNA damage in rat lymphocytes. Mutat Res 483(1–2):57–64
121. Zendehdel R, Yu IJ, Hajipour-Verdom B, Panjali Z (2019) DNA effects of low level occupational exposure to extremely low frequency electromagnetic fields (50/60 Hz). Toxicol Ind Health 35(6):424–430. https://doi.org/10.1177/0748233719851697
122. Wilson JW, Haines J, Sienkiewicz Z, Dubrova YE (2015) The effects of extremely low frequency magnetic fields on mutation induction in mice. Mutat Res 773:22–26. https://doi.org/10.1016/j.mrfmmm.2015.01.014
123. Prochownik EV (2008) c-Myc: linking transformation and genomic instability. Curr Mol Med 8(6):446–458. https://doi.org/10.2174/156652408785747988
124. Cordaux R, Batzer MA (2009) The impact of retrotransposons on human genome evolution. Nat Rev Genet 10(10):691
125. Kidwell MG, Lisch DR (2001) Perspective: transposable elements, parasitic DNA, and genome evolution. Evolution 55(1):1–24

126. Del Re B, Marcantonio P, Gavoçi E, Bersani F, Giorgi G (2012) Assessing LINE-1 retrotransposition activity in neuroblastoma cells exposed to extremely low-frequency pulsed magnetic fields. Mutat Res 749(1–2):76–81
127. Strauch B, Patel MK, Navarro JA, Berdichevsky M, Yu H-L, Pilla AA (2007) Pulsed magnetic fields accelerate cutaneous wound healing in rats. Plast Reconstr Surg 120(2):425–430
128. Athanasiou A, Karkambounas S, Batistatou A, Lykoudis E, Katsaraki A, Kartsiouni T, Papalois A, Evangelou A (2007) The effect of pulsed electromagnetic fields on secondary skin wound healing: an experimental study. Bioelectromagnetics 28(5):362–368
129. Grant DN, Cozad MJ, Grant DA, White RA, Grant SA (2015) In vitro electromagnetic stimulation to enhance cell proliferation in extracellular matrix constructs with and without metallic nanoparticles. J Biomed Mater Res B Appl Biomater 103(8):1532–1540
130. Ottani V, De Pasquale V, Govoni P, Franchi M, Ruggeri A, Zaniol P (1988) Effects of pulsed extremely-low-frequency magnetic fields on skin wounds in the rat. Bioelectromagnetics 9(1):53–62
131. Pesce M, Patruno A, Speranza L, Reale M (2013) Extremely low frequency electromagnetic field and wound healing: implication of cytokines as biological mediators. Eur Cytokine Netw 24(1):1–10
132. Patruno A, Amerio P, Pesce M, Vianale G, Di Luzio S, Tulli A, Franceschelli S, Grilli A, Muraro R, Reale M (2010) Extremely low frequency electromagnetic fields modulate expression of inducible nitric oxide synthase, endothelial nitric oxide synthase and cyclooxygenase-2 in the human keratinocyte cell line HaCat: potential therapeutic effects in wound healing. Br J Dermatol 162(2):258–266
133. Li F, Lei T, Xie K, Wu X, Tang C, Jiang M, Liu J, Luo E, Shen G (2016) Effects of extremely low frequency pulsed magnetic fields on diabetic nephropathy in streptozotocin-treated rats. Biomed Eng Online 15(1):8
134. Akbarnejad Z, Esmaeilpour K, Shabani M, Asadi-Shekaari M, Saeedi Goraghani M, Ahmadi-Zeidabadi M (2018) Spatial memory recovery in Alzheimer's rat model by electromagnetic field exposure. Int J Neurosci 128(8):691–696
135. Callaghan MJ, Chang EI, Seiser N, Aarabi S, Ghali S, Kinnucan ER, Simon BJ, Gurtner GC (2008) Pulsed electromagnetic fields accelerate normal and diabetic wound healing by increasing endogenous FGF-2 release. Plast Reconstr Surg 121(1):130–141
136. Tepper OM, Callaghan MJ, Chang EI, Galiano RD, Bhatt KA, Baharestani S, Gan J, Simon B, Hopper RA, Levine JP (2004) Electromagnetic fields increase in vitro and in vivo angiogenesis through endothelial release of FGF-2. FASEB J 18(11):1231–1312
137. Elmas O (2016) Effects of electromagnetic field exposure on the heart: a systematic review. Toxicol Ind Health 32(1):76–82

Publisher's Note Springer Nature remains neutral with regard to jurisdictional claims in published maps and institutional affiliations.
