# Supplementary material for: Magnetic Fields and Cancer: Epidemiology, Cellular Biology, and Theranostics
Source: Int J Mol Sci. 2022 Jan 25;23(3):1339. doi: 10.3390/ijms23031339 (PMC8835851; doi:10.3390/ijms23031339)
Supplement: Supplementary file 1 [file ijms-23-01339-s001.zip › Supplementary Data Set S1/MF and Cancer.Data/PDF/2802571610/Evaluation_of_Continuous_Exposure_to_Magnetic_.pdf]

Evaluation of Continuous Exposure to Magnetic Field From AC Overhead Transmission Lines Via Historical Load Databases: Common Procedures and Innovative Heuristic Formulas

Giovanni Mazzanti, *Member, IEEE*

***Abstract*—Public concern about electromagnetic fields from power systems calls for a proper evaluation of continuous exposure of the population to power frequency magnetic fields, especially those from overhead transmission lines, felt as impacting human activities and the environment. Sophisticated measuring and computing tools now available for continuous exposure evaluation may be ineffective without a sound knowledge of power system theory and of typical line loads. In this perspective, the paper firstly recalls the fundamentals of power line magnetic field calculation and shows that a proper use of historical load databases is essential for associating residential magnetic fields with load diagrams of the lines. Then, innovative heuristic formulas for ac double-circuit overhead transmission lines with independent circuits are proposed, so that line databases can be used accounting for phase-shift effects between line currents in an easier and faster way. Such formulas provide a good approximation, via one single fixed-load calculation, of the median/mean magnetic field over a reference operational period of the line (e.g., one year) and are successfully applied to an existing line, resorting to a historical load database of the line recorded from 1994 to 2001.**

***Index Terms*—Magnetic field effects, magnetic fields, power transmission lines, power transmission planning.**

I. Introduction

ELECTROMAGNETIC fields from power lines are a subject of growing concern among the population, particularly power-frequency magnetic fields for their controversial long-term effects on human health (see, e.g., [1]–[3]) and overhead transmission lines for their impact on both human activities and the environment. In this framework, the evaluation of continuous exposure to magnetic field from ac overhead transmission lines, to be compared with a proper exposure limit, is of major importance according to standards and guidelines about this subject [4]–[6]. Such limit (referred to as maximum permissible exposure [4] or as reference level [5], [6]) should be never exceeded for avoiding harmful short-term effects on human health. In year 2003 Italian Law [7] has added to this limit a lower one, referred to as attention value [7], that should not be exceeded in sensitive areas (e.g., residential buildings) under normal line operation for preventing long-term effects due to continuous exposure to magnetic fields; other countries, e.g., Switzerland, are following such approach currently or in the next future [8].

Skipping the troubled discussion about the opportunity of such conservative approach, focus is made here on continuous exposure of the general public[1] to magnetic field from transmission lines, i.e., on the field that these lines generate day by day, acknowledging that its estimation gives a deeper insight into their impact on human life and a remarkable help in power transmission planning. Continuous exposure is often meant as the mean or median value of rms magnetic flux density over a reference period (e.g., one day, one year, etc.) that accounts for typical line load variations. Continuous exposure can be either measured *in situ*—through long, complex and costly procedures—or derived as "historical calculated field" by applying *ad hoc* computational techniques to databases of electrical quantities recorded over the reference period. There is no universal agreement about the relative merits of "contemporary measurements" and "historical calculated fields" [9], [10], though the latter approach is supported, e.g., in [7].

In this paper, the common evaluation procedures of historical magnetic fields from ac overhead transmission lines (ACOHL) are recalled and the possible relevant errors are discussed (Section II). Then, innovative heuristic formulas for ac double-circuit overhead transmission lines (ACDOHL) with independent circuits are described, that account for the non-trivial phase-shift effects between circuit currents through a simplified and faster use of line databases (Section III). These innovative heuristic formulas—applied for the 1st time in [11] to one single year of a historical database of an existing ACDOHL—are shown here to perform quite well over the whole database recording period of the line, i.e., 8 years (Section IV).

It must be pointed out that the novel contribution of this paper with respect to [11] and other previous works by the same author [12], [13] is twofold: 1) a comprehensive and organic framework that encompasses both the common evaluation procedures for continuous exposure to magnetic field from ACOHL via historical databases and the innovative heuristic formulas proposed by the author; 2) the application of these innovative heuristic formulas to a multi-year load database with good performances over the whole database period.

Manuscript received June 09, 2009. First published December 11, 2009; current version published December 23, 2009. Paper no. TPWRD-00036-2009.

The author is with the Department of Electrical Engineering, Faculty of Engineering, University of Bologna, 40136 Bologna, Italy (e-mail: giovanni.mazzanti@mail.ing.unibo.it).

Digital Object Identifier 10.1109/TPWRD.2009.2035390

[1]Overhead transmission lines avoid urban areas, but some of them cross densely-populated suburban zones, thus the evaluation of continuous exposure of the general public to fields from such lines is important.

0885-8977/$26.00 © 2009 IEEE

II. Evaluation of Continuous Exposure to Magnetic Fields From AC Overhead Transmission Lines (ACOHL)

A. Calculation of Magnetic Fields From ACOHL

Commonly the calculation of magnetic flux density strength generated by a power line is based on the Biot-Savart law [14], [15]. Conductors are regarded as cylindrical wires with small cross-section, having curved layout between adjacent towers and hanging over a large flat conducting ground. Strictly speaking this geometry requires 3-D codes, but for ACOHL the following hypotheses—that involve acceptable errors [12], [14], [15]—turn magnetic field calculation into a purely 2-D problem: 1) infinite ground resistivity; 2) balanced phase currents; 3) no eddy currents in conductive elements close to the line (towers, pipes, etc.); 4) negligible harmonic currents; 5) straight, horizontal, infinitely-long wires.

Then magnetic field is calculated by setting at a given line section a 2-D Cartesian coordinate system orthogonal to line axis (with the origin on line axis, the x-axis lying on the ground and the y-axis perpendicular to the ground) and N field points where magnetic field is to be calculated, each identified by a coordinate vector $\boldsymbol{x}_j = \{x_j, y_j\}(j = 1, \ldots, N)$. Now, the rms value of magnetic flux density strength at a given location $\boldsymbol{x}_j, B(\boldsymbol{x}_j)$—the reference quantity in the evaluation of human exposure to low frequency magnetic fields from power lines [4]–[7]—can be computed according to the 2-D equations reported in [12]. They can be summarized as follows:

$$B(\boldsymbol{x}_j) = m\ f(\boldsymbol{x}_j, \boldsymbol{x}_i, I_i, \Delta_i) \quad (1)$$

where $m = \mu_0/(\pi\sqrt{2})$—being $\mu_0 = 4\pi 10^{-7}$ H/m—and $f(\boldsymbol{x}_j, \boldsymbol{x}_i, I_i, \Delta_i)$ is in general a non-linear function of $\boldsymbol{x}_j$ and:

- $\boldsymbol{x}_i = \{x_i, y_i\}$, the coordinate vector of the ith line conductor, that carries the ith phase current $(i = 1, \ldots, M$, being $M = 3$ for single-circuit lines, $M = 6$ for double-circuit lines[2])l
- I_i, the rms value of ith phase current;
- Δ_i, a quantity equal to zero for 1st circuit phase currents and to $\Delta\varphi$ for 2nd circuit phase currents, $\Delta\varphi$ being the phase-shift angle between the three symmetric current phasors of the 1st and the 2nd circuit.

Denoting the 1st circuit as circuit I and the relevant phase current indexes as $i = 1, 2, 3$, the 2nd circuit (if any) as circuit II and the relevant current indexes as $i = 4, 5, 6$, then—from above hypothesis $2 - I_{\rm I} = I_1 = I_2 = I_3$ is the rms value of circuit I phase currents and $I_{\rm II} = I_4 = I_5 = I_6$ is the rms value of circuit II phase currents. $B(\boldsymbol{x}_j)$ is referred to as B-field from now on [12].

Of course, $\Delta\varphi$ is zero for ac single-circuit overhead transmission lines (ACSOHL). As to ac double-circuit overhead transmission lines (ACDOHL), if the ACDOHL is a so-called "split-phase line", i.e., if it comes from the duplication of a single three-phase line, the phase currents flowing in the two circuits (at a given time and at a certain location along the line) can be described by two phasor terns in phase with each other; then, $\Delta\varphi = 0$. On the contrary, independent-circuit ACDOHL—i.e., ACDOHL made of two independent single-circuits that link different nodes of the transmission grid, but are placed on the same towers for a part of their length—exhibit a phase-shift between the three symmetric current phasors of the two circuits for most service conditions, that affects significantly the B-field from these lines [11], [12].

[2]Lines with more than two circuits are not considered here for brevity.

B. Historical Calculated Fields for ACOHL

As pointed out at Section I, at a residential location $\boldsymbol{x}_j$ the maximum permissible exposure of population should not be exceeded by the maximum magnetic field generated by an ACOHL line. Such "peak" B-field, yielded by the highest rms current carried by the line, can be derived trivially through (1). On the contrary, the attention value of [7] should be compared with a value of B-field representative of typical operating conditions of the line. As mentioned in Section I, this B-field value can be calculated from a historical database of the line; the calculations should be performed over a proper reference period, within which line currents vary in time according to the typical load profile of the line. The computation of such B-field value, referred to as "continuous exposure" from now on, is neither trivial nor free from errors and uncertainties.

Indeed, load variation is inherently stochastic, thus B-field can be regarded as a random variable and continuous exposure can be estimated as a reference percentile of the sampling distribution of B-field generated by the line at a given location $\boldsymbol{x}_j$ over a selected reference period [12]. The mean and the median value of B-field are natural candidates as the most significant percentiles for continuous exposure estimate: the mean value—usually the arithmetic average of all B-field values over the reference period—has the advantage to encompass every possible field value, while the median value—usually the central value of the same (sorted) values—has the advantage to account for field levels observed more frequently, neglecting rather uncommon extreme conditions (as supported by [7]). Here, for the sake of completeness, both the mean and the median value of B-field are treated; unless they are explicitly mentioned as median (symbol B_{50}) or mean (symbol $\langle B \rangle$) they are referred to both as "continuous B-field" and indicated both with one same symbol, i.e., B_C. Note that "$(\boldsymbol{x}_j)$" is dropped in the following for the sake of simplicity.

Once the reference percentile (mean or median) and the reference period have been selected, continuous B-field calculation consists of the following steps (see left branch in Fig. 1):

a) a historical database that contains load quantities recorded over the reference period is to be found;

b) by processing database quantities—essentially real and reactive power, plus voltage—the reference percentiles (i.e., median/mean values) of rms currents (referred to as "continuous currents" and denoted as $I_{{\rm I},C}, I_{{\rm II},C}$) and phase-shift angle (referred to as "continuous phase-shift angle" and denoted as $\Delta\varphi_C$) over the reference period are obtained from basic power system equations [16];

c) the relevant B-field values over the reference period are calculated by means of (1);

d) continuous B-field is derived by processing statistically all calculated B-field values. The continuous B-field obtained in this way is referred to as "calculated continuous B-field" and indicated as $B_{C,C}$ from now on.

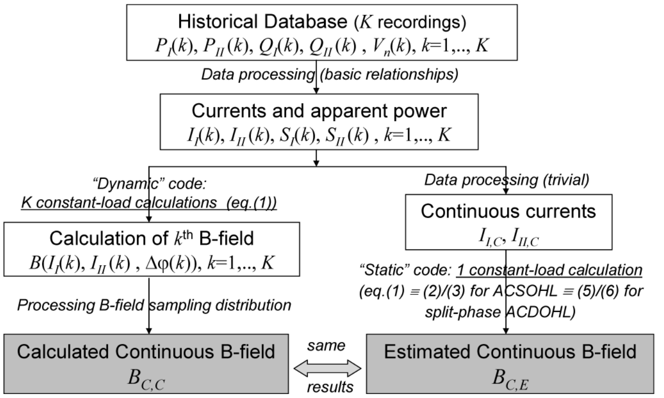

Fig. 1. Flowchart of the general procedure for deriving continuous B-field (i.e., continuous exposure) from line database for ACSOHL and split-phase ACDOHL; it can be obtained both as calculated and estimated continuous B-field, the results being the same.

The computation of $B_{C,C}$ is prone to errors, possibly due to:

i) an improper selection of the reference period;
ii) a wrong processing of the database quantities.

According to the strictest approach, the reference period should encompass all past service years of the line, but it was argued that the most recent load conditions may represent better the operation of the line as it affects human health at present [9]. Thus in practice, for easing the analysis a much closer and shorter reference period is often chosen (e.g., the last year, a recent month, in some cases even a recent day), also according to the availability of recorded data. Severe errors are avoided only by checking this choice carefully on the basis of the experience about the service of the line.

Whatever the selected reference period, achieving calculated continuous B-field is complex, because:

1) most codes available for the calculation of magnetic field from power lines work under fixed load (here they are referred to as "static codes" as in [12]) and cannot repeat automatically the calculation throughout many different load conditions, as needed for computing $B_{C,C}$;
2) the calculation of all field values over the reference period takes a more or less long computing time, depending on the length of the chosen reference period, and can be subjected to both input and computational errors.

Continuous exposure estimation over a given reference period is strongly eased and sped up if continuous B-field can be derived via a "static code" through one single calculation based on continuous currents and phase-shift angle. In principle, such currents and phase-shift angle can be achieved easily—e.g., by means of electronic spreadsheets—by processing database quantities relevant to the reference period and the calculation of all time-dependent B-field values can be skipped, thereby cutting computing time a lot (right branch in Fig. 1). The relevant B-field at a given location $\boldsymbol{x}_j$ is referred to as "estimated continuous B-field" and indicated as $B_{C,E}$—unless it is explicitly mentioned as estimated median B-field (symbol $B_{50,E}$) or estimated mean B-field (symbol $\langle B\rangle_E$).

In the most general case of an ACDOHL, the median phase currents, $I_{\mathrm{I},50}$ and $I_{\mathrm{II},50}$, and the mean phase currents, $\langle I_{\mathrm{I}}\rangle$ and $\langle I_{\mathrm{II}}\rangle$, of both circuits over the reference period can be computed, respectively, as the central values and the arithmetic averages of all rms phase currents of each circuit. On the other hand, the derivation of the median phase-shift angle, $\Delta\varphi_{50}$, and of the mean phase-shift angle, $\langle\Delta\varphi\rangle$, is more cumbersome. Indeed, strictly speaking the estimated median/mean $\Delta\varphi$ could be derived simply as the median/mean of all values of $\Delta\varphi$ over the reference period, as for median/mean values of I_{I} and I_{II}; nevertheless, this estimate would not be consistent, since—being $\Delta\varphi$ a periodical quantity of period 360°—it would lead to wrong compensations between values (e.g., 1° and 359°) very different from a numerical viewpoint, but coincident in practice as to the field from the line, thereby involving inconsistencies in the estimate of the sampling distribution, of the arithmetic average and of the central value of $\Delta\varphi$.

Moreover, further problems peculiar to the median and to the mean value of B-field arise. As to the median B-field, the "central values" of the sampling distribution of I_{I}, I_{II} and $\Delta\varphi$—that could be chosen in principle as the relevant median values for the derivation of the median B-field—are not synchronous to each other, since line databases show that $I_{\mathrm{I}}, I_{\mathrm{II}}$ and $\Delta\varphi$ behave as fully-uncorrelated random variables over the reference period. Hence, for a certain reference period the median B-field estimated through one single "static code calculation" based on central values of currents and phase-shift angle differs surely from the median value of all time-dependent B-fields over the same period.

As to the mean B-field, other problems arise and partially-different arguments hold. They are dealt with extensively in a comprehensive way together with the median B-field estimates in the next Subsection.

C. Continuous Exposure: ACSOHL vs. ACDOHL

The above-mentioned strong simplification of mean exposure evaluation takes place if B-field in (1) can be expressed as a linear function of rms currents and phase-shift angle. Such a linear expression exists and is trivial for ACSOHL, since in this case the only field source are the three symmetric current phasors of the single-circuit. Indeed, from (1) with $M = 3$ and $\Delta\varphi = 0$ one obtains that the mean value of B-field from an ACSOT line at a field point $\boldsymbol{x}_j$ over a chosen time period, $\langle B\rangle_E$, is a linear function of the mean value of single-circuit rms phase current over that period, $\langle I_{\mathrm{I}}\rangle$, namely [13]

$$\langle B\rangle_E = m\,F_{\mathrm{I}}\langle I_{\mathrm{I}}\rangle \tag{2}$$

where F_{I} is a proper function of $\boldsymbol{x}_i, \boldsymbol{x}_j$ reported in [13].

Therefore, for an ACSOHL mean B-field over a reference period can be determined through a single run of a "static code", having as input the mean value of rms phase currents calculated from the line database relevant to that period.

Of course (see Subsection II.B), an expression analogous to (2) is valid for median B-field from an ACSOHL, i.e.,

$$B_{50,E} = m\,F_{\mathrm{I}}\,I_{\mathrm{I},50}. \tag{3}$$

The same also holds, though approximately, for split-phase ACDOHL, because the time variation of currents depends on the 3-phase circuit whose splitting gives rise to the double-circuit line and the three symmetric current phasors of the two single-circuits are always in phase with each other; only the double-circuit geometry has to be accounted for. Thus, from (1) with $M = 6$ (double circuit) and $\Delta\varphi = 0$, after proper rearrangements and by defining other functions of $\boldsymbol{x}_i, \boldsymbol{x}_j - F_{\mathrm{II}}, M_{\mathrm{I,II}}$, reported in [13], one obtains

$$B = m\sqrt{F_{\mathrm{I}}^2 I_{\mathrm{I}}^2 + F_{\mathrm{II}}^2 I_{\mathrm{II}}^2 + 2M_{\mathrm{I,II}} I_{\mathrm{I}} I_{\mathrm{II}}}. \quad (4)$$

Since in general it holds $M_{\mathrm{I,II}} \neq F_{\mathrm{I}} F_{\mathrm{II}}$ [13], relationship (4) is not a perfect square and B-field in (4) is a non-linear function of I_{I} and I_{II}. Hence, strictly speaking, the mean value of B-field from split-phase ACDOHL at a field point $\boldsymbol{x}_j$ over a time period, $\langle B \rangle$, is not a linear function of the mean value of rms phase currents of circuit I and II over that period, $\langle I_{\mathrm{I}} \rangle$ and $\langle I_{\mathrm{II}} \rangle$, respectively. Nevertheless, it was shown in [13] that the errors involved when assuming $\langle B \rangle$ as a linear function of the mean value of rms phase currents of both circuits over that period, $\langle I_{\mathrm{I}} \rangle$ and $\langle I_{\mathrm{II}} \rangle$, are largely acceptable, especially outside the right of way of the line. Therefore, a very good estimate of the mean value of B-field for split-phase ACDOHL can be determined directly by means of a single run of a "static code" having as input the mean values of rms phase currents of both circuits calculated from the line database. Such estimate, $\langle B \rangle_E$, has the following expression:

$$\langle B \rangle_E = m\sqrt{F_{\mathrm{I}}^2 \langle I_{\mathrm{I}} \rangle^2 + F_{\mathrm{II}}^2 \langle I_{\mathrm{II}} \rangle^2 + 2M_{\mathrm{I,II}} \langle I_{\mathrm{I}} \rangle \langle I_{\mathrm{II}} \rangle}. \quad (5)$$

Of course (see Section II-B), an expression analogous to (5) is valid for median B-field for split-phase ACDOHL, i.e.,

$$B_{50,E} = m\sqrt{F_{\mathrm{I}}^2 I_{\mathrm{I},50}^2 + F_{\mathrm{II}}^2 I_{\mathrm{II},50}^2 + 2M_{\mathrm{I,II}} I_{\mathrm{I},50} I_{\mathrm{II},50}} \quad (6)$$

with the only limitation that the median value of rms phase currents of both circuits over the reference period, $I_{\mathrm{I},50}$ and $I_{\mathrm{II},50}$ are synchronous neither to each other, nor to the calculated median B-field.

As illustrated in Fig. 1, it can be concluded that continuous B-field (i.e., continuous exposure) for ACSOHL and split-phase ACDOHL can be obtained from line database both as calculated continuous B-field (left branch of the flowchart) and estimated continuous B-field (right branch of the flowchart), the results being practically the same.

For independent-circuit ACDOHL (i.e., ACDOHL with independent single-circuits), the three symmetric current phasors of the two single-circuits are mostly—if not always—phase-shifted from each other, with a phase-shift angle $\Delta\varphi$ dependent on time, as phase currents. Moreover, the dependence of time-varying B-field on $\Delta\varphi$ and on rms currents I_{I} and I_{II} is not linear. Indeed, for independent-circuit ACDOHL, rearranging (1) with $M = 6, \Delta\varphi \neq 0$—and by defining $M_{\mathrm{I,II}}(\Delta\varphi)$ and $G_{\mathrm{II}}(\Delta\varphi)$, functions of $\boldsymbol{x}_i, \boldsymbol{x}_j$ and $\Delta\varphi$ reported in [13], one gets

$$B = m\sqrt{F_{\mathrm{I}}^2 I_{\mathrm{I}}^2 + G_{\mathrm{II}}^2(\Delta\varphi) I_{\mathrm{II}}^2 + 2M_{\mathrm{I,II}}(\Delta\varphi) I_{\mathrm{I}} I_{\mathrm{II}}}. \quad (7)$$

In general $M_{\mathrm{I,II}}(\Delta\varphi) \neq F_{\mathrm{I}} G_{\mathrm{II}}(\Delta\varphi)$ and considering (7) as a perfect square implies severe errors [13]. Thus B-field in (7) is a non-linear function of I_{I}, I_{II} and $\Delta\varphi$. For independent-circuit ACDOHL, it is then clear that $\langle B \rangle$ is not a linear function of $\langle I_{\mathrm{I}} \rangle$, $\langle I_{\mathrm{II}} \rangle$ and $\langle \Delta\varphi \rangle$ (the latter being the mean value of $\Delta\varphi$ over the reference period). In fact, it holds

$$\langle B \rangle = m \left\langle \sqrt{F_{\mathrm{I}}^2 I_{\mathrm{I}}^2 + G_{\mathrm{II}}^2(\Delta\varphi) I_{\mathrm{II}}^2 + 2M_{\mathrm{I,II}}(\Delta\varphi) I_{\mathrm{I}} I_{\mathrm{II}}} \right\rangle. \quad (8)$$

As to the median B-field, the problem relevant to the non-isochronicity of currents adds up to that of the inconsistency in the estimate of the sampling distribution, the arithmetic average and the central value of $\Delta\varphi$ (see Subsection II.B). Therefore, an expression analogous to (8) holds, namely

$$B_{50} = m \left(\sqrt{F_{\mathrm{I}}^2 I_{\mathrm{I}}^2 + G_{\mathrm{II}}^2(\Delta\varphi) I_{\mathrm{II}}^2 + 2M_{\mathrm{I,II}}(\Delta\varphi) I_{\mathrm{I}} I_{\mathrm{II}}} \right)_{50}. \quad (9)$$

In conclusion, for independent-circuit ACDOHL continuous B-field can be achieved only as "calculated continuous B-field" (i.e., by calculating the mean/median of all time-varying B-field values over the reference period for the examined line). Approximate expressions for mean/median B-field vs. mean/median values of I_{I}, I_{II} and $\Delta\varphi$ can be derived from (8), (9), in the following form:

$$\begin{aligned} \langle B \rangle_E = m &\left[F_{\mathrm{I}}^2 \langle I_{\mathrm{I}} \rangle^2 + G_{\mathrm{II}}^2(\langle \Delta\varphi \rangle) \langle I_{\mathrm{II}} \rangle^2 \right. \\ &\left. + 2M_{\mathrm{I,II}}(\langle \Delta\varphi \rangle) \langle I_{\mathrm{I}} \rangle \langle I_{\mathrm{II}} \rangle \right]^{1/2} \quad (10) \\ B_{50,E} = m &\left[F_{\mathrm{I}}^2 I_{\mathrm{I},50}^2 + G_{\mathrm{II}}^2(\Delta\varphi_{50}) I_{\mathrm{II},50}^2 \right. \\ &\left. + 2M_{\mathrm{I,II}}(\Delta\varphi_{50}) I_{\mathrm{I},50} I_{\mathrm{II},50} \right]^{1/2} \quad (11) \end{aligned}$$

but in general they are more or less wrong, as shown in [13].

III. Innovative Heuristic Formulas for Continuous Exposure to Independent-Circuit ACDOHL

A. *Historic Databases for Independent-Circuit ACDOHL*

For the sake of simplicity, let us focus on independent-circuit ACDOHL whose single-circuit lines have a common close end at network node A and share the same towers for some kilometers, with a remarkable saving of space and money; at the far end, line *I* enters node B_{I}, while line *II* enters node B_{II}[3]. For lines of such kind, continuous exposure evaluation via load databases requires the historical values of real power P and reactive power Q for both circuits, as well as of common rms line-to-line voltage V_n, recorded over the chosen reference period at the common end of circuits, i.e., node A.

Then, for both circuit *I* and *II* the values of apparent power, $S_{\mathrm{I},k}$ and $S_{\mathrm{II},k}$, and of rms phase current, $I_{\mathrm{I},k}$ and $I_{\mathrm{II},k}$, can be calculated at every single kth recording time ($k = 1, \ldots, K, K$ being the total number of recording times) from the relevant measured values of active power, $P_{\mathrm{I},k}$ and $P_{\mathrm{II},k}$, reactive power, $Q_{\mathrm{I},k}$ and $Q_{\mathrm{II},k}$, and line-to-line voltage, $V_{n,k}$, through basic equations [11], [16]. Further, the values of power factor angle

[3]Independent-circuit ACDOHL that share neither ends also exist, but they are far less common than those that share one end.

at each kth acquisition time for lines I and II, $\varphi_{\mathrm{I},k}$ and $\varphi_{\mathrm{II},k}$ respectively, as well as of phase-shift angle $\Delta\varphi_k$, can been derived as[4] [11]

$$\varphi_{\mathrm{I},k} = \arccos(P_{\mathrm{I},k}/S_{\mathrm{I},k}) \quad (12)$$

$$\varphi_{\mathrm{II},k} = \arccos(P_{\mathrm{II},k}/S_{\mathrm{II},k}) \quad (13)$$

$$\Delta\varphi_k = \varphi_{\mathrm{II},k} - \varphi_{\mathrm{I},k} \quad (14)$$

thereby giving rise to an exhaustive post-processed database for B-field calculation at every single recording time.

B. New Heuristic Formulas for Continuous Exposure

The evaluation of continuous exposure to magnetic fields would be strongly simplified also for independent-circuit ACDOHL by finding an estimate of continuous $\Delta\varphi$ over the reference period that, together with continuous currents, provides—by means of one single "static code" calculation based on (10)/(11)—an estimated continuous B-field, $B_{C,E}$, close to the calculated continuous B-field, as can be done for ACSOHL through (2)/(3) or for split-phase ACDOHL through (5)/(6). From now on, such an estimate of continuous $\Delta\varphi$ is referred to as "estimated continuous $\Delta\varphi$" and indicated as $\Delta\varphi_{C,E}$—unless it is explicitly mentioned as estimated median $\Delta\varphi$ (symbol $\Delta\varphi_{50,E}$) or estimated mean $\Delta\varphi$ (symbol $\langle\Delta\varphi\rangle_E$, see (10), (11)). Although $\Delta\varphi_{C,E}$ cannot be attained analytically, as previously pointed out, heuristic expressions based on a careful analysis of line database can be derived *a priori* and can then be checked *a posteriori* by comparing the relevant $B_{C,E}$ with the calculated continuous B-field, i.e., $B_{C,C}$.

As argued above, $\Delta\varphi_{C,E}$ cannot be derived simply as the median/mean of all values of $\Delta\varphi$ calculated according to (12)–(14) over the reference period, because $\Delta\varphi$ is a periodical quantity and this affects its sampling distribution. The first step for solving this inconsistency was taken in [11] by expressing $\Delta\varphi_E$ as the difference of the estimated continuous values of φ_{II} and φ_{I}, $\varphi_{\mathrm{II},C,E}$ and $\varphi_{\mathrm{I},C,E}$ respectively, i.e.,

$$\Delta\varphi_{C,E} = \varphi_{\mathrm{II},C,E} - \varphi_{\mathrm{I},C,E} \quad (15)$$

thereby displacing the problem to the search of estimated continuous values for φ_{I} and φ_{II} that are satisfactory from the viewpoint of the evaluation of continuous exposure. Though φ_{I} and φ_{II} are periodical quantities of period 360° as $\Delta\varphi$, φ_{I} and φ_{II} have the advantage that the relevant median/mean values can be evaluated by processing suitably database quantities relevant to one single-circuit at a time, i.e., in practice on the basis of proper combinations of the estimates of median/mean values of P, Q, S, processed statistically in a separate way for circuit I and II [see (12), (13)].

Processing procedures of these quantities are not trivial as well. Indeed, $P_{\mathrm{I}}, P_{\mathrm{II}}$ and $Q_{\mathrm{I}}, Q_{\mathrm{II}}$ are sometimes positive, sometimes negative, depending on whether they enter or go out of node A; thus, the relevant averages are not consistent as to the magnetic field generated by the line, since they imply the cancellation of opposite values of P and Q that do contribute to the field. In addition, the averages of P and Q are not consistent with the average of S, being S a positive-definite quantity, while P, Q are not positive-definite.

In order to solve these inconsistencies for the estimation of $\varphi_{\mathrm{II},C,E}$ and $\varphi_{\mathrm{I},C,E}$, a heuristic procedure based on a lengthy "trial and error" processing of database quantities was carried out. Quantities that turned out to be useful were the continuous (i.e., median/mean) values of $P_{\mathrm{I}}^2, P_{\mathrm{II}}^2$ and $Q_{\mathrm{I}}^2, Q_{\mathrm{II}}^2$, denoted as $P_{\mathrm{I},C}^2, P_{\mathrm{II},C}^2$ and $Q_{\mathrm{I},C}^2, Q_{\mathrm{II},C}^2$, respectively, as well as the rms values of $P_{\mathrm{I}}, P_{\mathrm{II}}$ and $Q_{\mathrm{I}}, Q_{\mathrm{II}}$ over the reference period, denoted as $P_{\mathrm{I,rms}}, P_{\mathrm{II,rms}}$ and $Q_{\mathrm{I,rms}}, Q_{\mathrm{II,rms}}$, respectively. The best solution found takes two alternative forms depending on the result of a check condition about the continuous values of P^2 and Q^2, and is reported in Table I as two different sets of equations, numbered for the sake of clarity as (15)–(25). For brevity Table I refers to continuous φ-estimate relevant to circuit I only, i.e., to $\varphi_{\mathrm{I},C,E}$, but similar relationships hold also for $\varphi_{\mathrm{II},C,E}$. The flow chart of Fig. 2 shows the whole procedure.

Although (15)–(25) miss a thorough theoretical support, being derived through a heuristic procedure, they are consistent with basic power system equations [16] and make some sense, as briefly explained in the Table.

Heuristic formulas (15)–(25) were presented for the 1st time in [11], where they are formalized in terms of mean quantities and estimates, while here they are generalized in terms of "continuous quantities and estimates". In [11] they were applied successfully to an existing ACDOHL over the whole year of operation 2001, for which a historic line database was available. Since then, these formulas were not improved, but their performances proved to be successful over the whole database period, i.e., from 1994 to 2001, as shown in Section IV.

IV. The New Formulas Applied to a Real ACDOHL

A. The Existing ACDOHL Treated in the Application

The existing ACDOHL, located in Italy, is described in detail in [11], [12], but its main features are recalled here for the sake of clarity. The phase arrangement is "phase in order"; conductors consist of a triple Aluminum Conductor Steel Reinforced (ACSR) bundle, with 3×585 mm^2 total cross-section. The line is made of two independent 380 kV single-circuit lines, that have a common close end at sub-station A, near a high-demand suburban area, and share the same towers for 2 km, crossing a densely-populated zone. The line database, supplied by TERNA (the Italian TSO), contains the values of real power P and reactive power Q for circuits I and II, as well as of common rms line-to-line voltage V_n, recorded at sub-station A bus-bars every hour over the years from 1994 to 2001. The database also reports the values of apparent power S and rms phase current I for both circuits. From database quantities, the values of power factor angles at every kth recording time for lines I and II, $\varphi_{\mathrm{I},k}$ and $\varphi_{\mathrm{II},k}$, respectively, as well as of phase-shift angle $\Delta\varphi_k$, have been calculated via (12)–(14).

The database shows that the power flows of the two circuits are fairly independent of each other, resulting in a significant phase-shift angle $\Delta\varphi$ between the relevant current terns; $\Delta\varphi$ varies from 0° to 360° (or equivalently from $-180°$ to $+180°$), though some values are more frequent [11]. Hence, current

[4] A further check about the sign of reactive powers $Q_{\mathrm{I},k}, Q_{\mathrm{II},k}$ enables the proper location of $\varphi_{\mathrm{I},k}, \varphi_{\mathrm{II},k}$ in either the [0°;180°] or the [180°;360°] range.

TABLE I
INNOVATIVE HEURISTIC FORMULAS FOR INDEPENDENT-CIRCUIT ACDOHL. THE TWO ALTERNATIVE TEST CONDITIONS TO BE CHECKED, THE RELEVANT EQUATIONS TO BE USED AND A CORRESPONDING TENTATIVE EXPLANATION FROM THE VIEWPOINT OF POWER SYSTEM THEORY ARE ALSO REPORTED. FOR BREVITY REFERENCE IS MADE TO CONTINUOUS φ-ESTIMATE RELEVANT TO CIRCUIT I ONLY

test condition		explanation	test condition		explanation
if $P_{I,C}^2 >> Q_{I,C}^2$ (or if $P_{I,rms} > Q_{I,rms}$ when $P_{I,C}^2 \approx Q_{I,C}^2$) ⇓		"more real than reactive power" (in rms sense)	else if $P_{I,C}^2 << Q_{I,C}^2$ (or if $P_{I,rms} < Q_{I,rms}$ when $P_{I,C}^2 \approx Q_{I,C}^2$) ⇓		"more reactive than real power" (in rms sense)
equations to be used if the above test condition is matched	eq. No.	explanation	equations to be used if the above test condition is matched	eq. No.	explanation
$\cos\varphi_{I,C,E} = P_{I,C} / S_{I,C}$ $\lvert\sin\varphi_{I,C,E}\rvert = \sqrt{1-(\cos\varphi_{I,C,E})^2}$	(16) (17)	P plays a major role, providing $\cos\varphi_{I,C,E}$ and $\lvert\sin\varphi_{I,C,E}\rvert$	$\sin\varphi_{I,C,E} = Q_{I,C} / S_{I,C}$ $\lvert\cos\varphi_{I,C,E}\rvert = \sqrt{1-(\sin\varphi_{I,C,E})^2}$	(21) (22)	Q plays a major role, providing $\sin\varphi_{I,C,E}$ and $\lvert\cos\varphi_{I,C,E}\rvert$
$sign(\sin\varphi_{I,C,E}) = sign(Q_{I,C} / S_{I,C})$	(18)	Q plays a minor role, yielding only the sign of $\sin\varphi_{I,C,E}$	$sign(\cos\varphi_{I,C,E}) = sign(P_{I,C} / S_{I,C})$	(23)	P plays a minor role, yielding only the sign of $\cos(\varphi_{I,C,E})$
$\sin\varphi_{I,C,E} =$ $= sign(\sin\varphi_{I,C,E})\lvert\sin\varphi_{I,C,E}\rvert$ $\varphi_{I,C,E} =$ $= sign(\sin\varphi_{I,C,E})\arccos(\cos\varphi_{I,C,E})$	(19) (20)	$\varphi_{I,C,E}$ is a combination between $\cos\varphi_{I,C,E}$ and the sign of $\sin\varphi_{I,C,E}$	$\cos\varphi_{I,C,E} =$ $= sign(\cos\varphi_{I,C,E})\lvert\cos\varphi_{I,C,E}\rvert$ $\varphi_{I,C,E} =$ $= sign(\cos\varphi_{I,C,E})\arcsin(\sin\varphi_{I,C,E})$	(24) (25)	$\varphi_{I,C,E}$ is a combination between $\sin\varphi_{I,C,E}$ and the sign of $\cos\varphi_{I,C,E}$

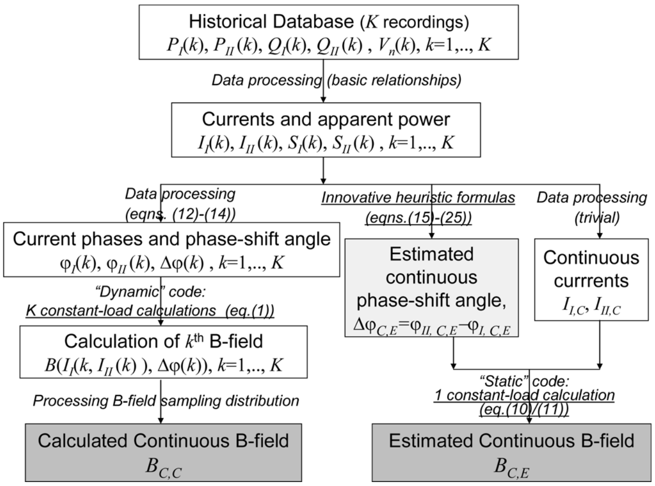

Fig. 2. Flowchart of the general procedure for deriving continuous B-field from line database for independent-circuit ACDOHL. The heuristic formulas provide an estimate of continuous B-field that cuts by much the calculations with good results (see Section IV).

phase-shift plays a major role on magnetic field established by this line, as the theoretical analysis carried out in [12] proves.

The B-field Double-circuit Phase-shift (BDP) code, developed and validated by the author [11]–[13], is used here for the 2-D calculation of B-field according to (1) at the various recording times of the database. Only the mid-span section between two adjacent towers of the considered ACDOHL is analyzed, being the most critical for human exposure to magnetic fields; a total number $N = 101$ of field points is set at mid-span, having constant height above ground $y_i = 1$ m $(i = 1, \ldots, N)$ and distance from line axis in the range $[x_1 = -100$ m; $x_N = 100$ m$]$. Database quantities recorded at sub-station A can be used for field calculations at all mid-span sections of the ACDOHL, since along its short length the phases and rms values of currents show negligible variations.

B. The Heuristic Formulas Applied to Years From 1994 to 2001

The preliminary case study reported in [11] showed the good performances of the new heuristic formulas presented at Section III for the treated line all over year 2001. Here, focus is on the period from 1994 to 2000 in order to check the performances of the proposed formulas also for these years.

Within every year, reference periods of different length have been selected, i.e., 1 day, 1 week, 1 month and the whole year. Among reference periods smaller than the whole year, four months have been chosen first, i.e., January, April, July, October, as being each central over the relevant season, thus well representative of the different seasonal load conditions. Within each month, weeks from the 8th to the 14th day have been selected as typical of average weekly load conditions. Within each week, Monday, being a mix-up of weekday and weekend load conditions, has been singled out as typical of average daily load. Then, magnetic field has been computed at all mid-span section field points for every single kth recording time of the line database, giving rise to the relevant kth "B-field profile."[5] Further, time-varying B-field profiles have been processed over

[5]B-field profiles consist of all B-field values over the mid-span section, that are rms quantities changing with load conditions (as I_{I} and I_{II}) and can be averaged over reference periods. Also $\Delta\varphi$ is implicitly assumed as a time-varying quantity of this type.

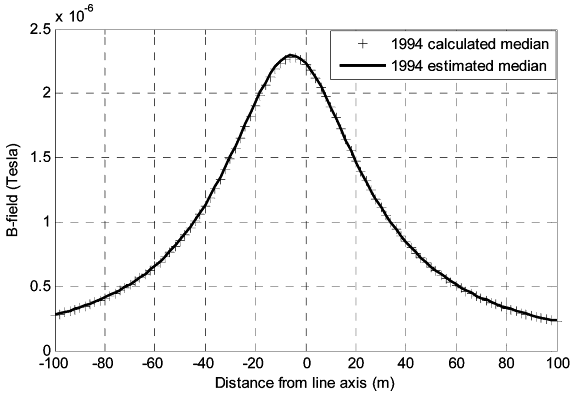

Fig. 3. Estimated (line) and calculated (crosses) median B-field profiles relevant to year 1994 for the considered ACDOHL at mid-span section (height of field points above ground 1 m).

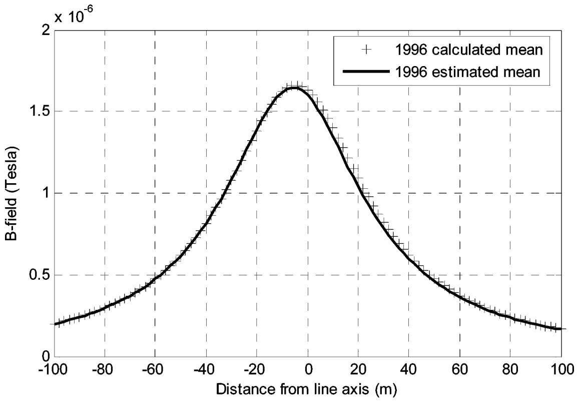

Fig. 4. Same as Fig. 2, but relevant to mean B-field for year 1996.

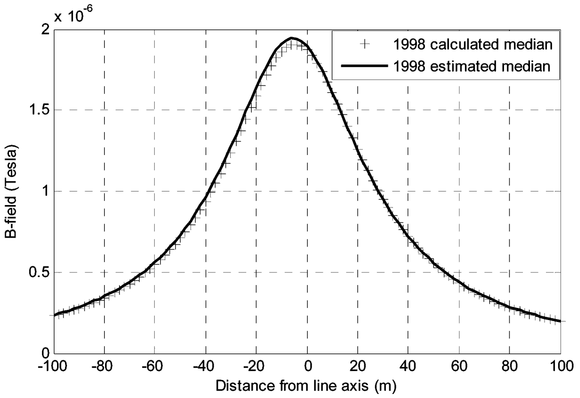

Fig. 5. Same as Fig. 2, but relevant to median B-field for year 1998.

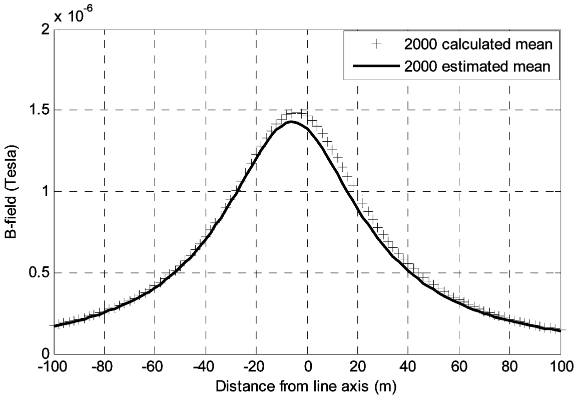

Fig. 6. Same as Fig. 2, but relevant to mean B-field for year 2000.

the various selected reference periods, thereby acquiring the relevant (daily, weekly, monthly, yearly) calculated continuous (i.e., median/mean) B-field profiles.

The performances of the proposed estimates of continuous $\Delta\varphi$ have been checked by processing line database quantities according to (15)–(25) over the various reference periods, thereby achieving the relevant values of $\Delta\varphi_{C,E}$. Then, the relevant values of $B_{C,E}$ at all N field points over mid-span section—referred from now on as estimated continuous B-field profiles—have been derived according to (10), (11) via one single "static" calculation carried out by the BDP-code, having as input $\Delta\varphi_{C,E}$ plus continuous currents. Finally, estimated continuous B-field profiles are compared with calculated continuous B-field profiles over the various reference periods and the deviation of estimated from calculated profiles gives an indication of how formulas (15)–(25) perform in estimating continuous exposure to magnetic field from the treated line.

An immediate and synthetic comparison is provided by the graphical superposition of estimated and calculated continuous B-field profiles over the various reference periods. Since extreme load situations are more easily compensated over longer periods and seasonal characteristics take place year by year in a similar way, particular significance is attributed to the whole years, being more representative of the typical "average" operation of the line; for this reason and for the sake of brevity, the illustration is restricted here to whole years only.

The performances of the proposed heuristic formulas is quite satisfactory for all the years from 1994 to 2000, as shown e.g., in Figs. 3–6 with reference to mean or median B-field profiles relevant to years 1994, 1996, 1998, 2000. The maximum difference between calculated and estimated continuous B-field always occurs close to line axis, whereas estimated B-field practically coincides with calculated B-field for distances from line axis over, say, 30 m, i.e., outside the right of way; this is where people lives, thus the proposed heuristic formulas are excellent in the most important area for human exposure to magnetic fields generated by the line. Note also that B-field profiles are asymmetric with respect to line axis, as expected for independent-circuit ACDOHL with phase-in-order conductor arrangement, even in the case of equal currents in the two circuits [12]. Similar results are obtained for reference periods shorter than one year, the agreement between calculated and estimated continuous B-field profiles being sometimes better, sometimes worse, but with a trend to improve for longer periods, especially as to median values.

For a less subjective evaluation, the maximum percent deviation between estimated and calculated median/mean B-field profiles at mid-span, $\varepsilon_{B,50}/\varepsilon_{B,m}$, has been derived as follows:

$$\varepsilon_{B,50} = 100\max\{|B_{50,E} - B_{50,C}|/B_{50,C}\} \tag{26}$$

$$\varepsilon_{B,m} = 100\max\{|\langle B\rangle_E - \langle B\rangle_C|/\langle B\rangle_C\}. \tag{27}$$

The values of $\varepsilon_{B,50}$ and $\varepsilon_{B,m}$ for all the years from 1994 to 2001 are reported in Table II. The Table shows that $\varepsilon_{B,50}$ is al-

TABLE II
ESTIMATED AND CALCULATED MEDIAN/MEAN VALUES OF PHASE-SHIFT ANGLE (AND RELEVANT ERROR PARAMETERS) FOR YEARS FROM 1994 TO 2001 FOR THE TREATED ACDOHL

year	$\varepsilon_{B,50}$ [%]	$\Delta\varphi_{50,E}$ [°]	$\Delta\varphi_{50C}$ [°]	$\Delta\varphi_{cen}$ [°]	$\varepsilon_{cen,B,50}$ [%]	$\varepsilon_{0,B,50}$ [%]	$\varepsilon_{90,B,50}$ [%]	$\varepsilon_{180,B,50}$ [%]	$\varepsilon_{270,B,50}$ [%]	$\varepsilon_{B,m}$ [%]	$<\Delta\varphi>_E$ [°]	$<\Delta\varphi>_C$ [°]	$\Delta\varphi_{ave}$ [°]	$\varepsilon_{ave,B,m}$ [%]	$\varepsilon_{0,B,m}$ [%]	$\varepsilon_{90,B,m}$ [%]	$\varepsilon_{180,B,m}$ [%]	$\varepsilon_{270,B,m}$ [%]
94	1.44	96.5	98	187	20.4	24.3	3.83	20.8	5.86	2.64	95,1	92	192	24.5	23.4	1.69	25.6	3.45
95	4.40	93.7	82	196	21.0	16.3	3.30	22.2	2.17	3.69	94.28	89	200	23.5	21.1	2.13	25.9	1.81
96	4.37	102.8	95	216	17.1	24.8	3.26	23.9	4.64	4.59	104.2	98	233	11.7	30.6	5.18	27.6	7.13
97	4.67	99.1	105	209	21.7	39.3	9.66	28.9	12.5	6.06	108.0	102	227	16.4	41.2	9.50	34.8	11.7
98	3.16	91.9	96	225	14.6	24.5	3.61	24.8	4.33	3.96	96.1	92	244	9.42	26.4	2.55	30.3	3.54
99	2.51	121.9	122	216	7.77	32.0	11.6	13.7	14.0	11.3	122.9	109	231	9.88	49.2	14.8	32.7	17.6
00	5.65	108.8	114	197	22.2	50.8	17.5	25.6	20.9	8.88	109.1	98	207	32.5	81.4	7.95	40.3	10.7
01	7.60	126.8	130	179	29.2	96.5	46.1	29.2	50.4	17.5	130.7	117	207	32.5	81.4	32.2	49.1	35.4

ways less than 8%, ranging from 1.44% to 7.60%; hence, as to the median B-field, the innovative heuristic formulas perform very well over the whole database time interval. Dealing with $\varepsilon_{B,m}$, it is mostly below 9%, ranging from 2.64% to 8.88%, with the exception of years 1999 (11.3%) and 2001 (17.5%). This is consistent with what already observed in [11] for periods within year 2001, as well as for the whole year, i.e., the agreement between estimated and calculated B-field values for year 2001 is satisfactory for mean values, even if not so good as for median values. The better agreement for yearly $B_{50,E}$ with respect to yearly $\langle B\rangle_E$ may be due to the fact that mean values are more sensitive to extreme values that occur rarely, as pointed out above; for this reason Italian law focuses on median values for evaluating the exposure to fields from power lines [7]. Therefore, the quite good agreement between calculated and estimated median B-field values is essential within this investigation and indicates a very satisfactory performance of the proposed median $\Delta\varphi$ estimate.

However, mean estimates are satisfactory on the whole, too, when considering also the much simpler and faster way in which they are attained with respect to calculated mean values. Indeed, computing time is strongly reduced: from ~25 minutes to fractions of a second when using a PC with 2 GHz processor and 1 Gbyte of RAM; and the reduction would be more significant if database quantities were recorded more frequently than in this case (i.e., if $K > 8760$). Moreover, outside the right of way mean estimates are as accurate as the median. In addition, apart year 2001 (for which (15)–(25) provide the worst estimates) $\langle\Delta\varphi\rangle_E$ is almost as satisfactory as $\Delta\varphi_{50,E}$.

Table II also reports the so-called "calculated continuous $\Delta\varphi$," denoted as $\Delta\varphi_{C,C}$, i.e., the values of $\Delta\varphi$ that inserted directly as input of the BDP-code in (10), (11)—together with continuous rms currents—provide the closest continuous B-field profiles to the calculated continuous B-field profiles; thus, $\Delta\varphi_{C,C}$ can be deemed as an *a posteriori* estimate of $\Delta\varphi_{C,E}$. An *ad-hoc* iterative procedure has been set up for deriving the values of $\Delta\varphi_{C,C}$, that have provided B-field profiles with maximum percent deviations below 3% from the calculated continuous B-field profiles for the considered years; thus $\Delta\varphi_{C,C}$ give an acceptable indication about what the "exact" values of $\Delta\varphi_{C,E}$ should be. As can be argued from Table II, $\Delta\varphi_{50,C}$ ranges from 82° to 130°, while $\langle\Delta\varphi\rangle_C$ from 89° to 117°: this proves that the treated ACDOHL has experienced a wide variety of power flows and operating conditions from 1994 to 2001. The deviations of $\Delta\varphi_{50,E}$ from $\Delta\varphi_{50,C}$ range from $-5.9°$ to $+11.7°$, while the deviations of $\langle\Delta\varphi\rangle_E$ from $\langle\Delta\varphi\rangle_C$ range from $+3.1°$ to $+13.9°$. However, these deviations should be regarded with care, since it must be remembered that (10), (11) are approximate relationships and that the dependence of B-field on $I_{\mathrm{I}}, I_{\mathrm{II}}$ and $\Delta\varphi$ is non-linear; as a consequence, small or large variations of B-field occur within different ranges of values of $\Delta\varphi$ having the same width, thus a large deviation between $\Delta\varphi_{C,E}$ and $\Delta\varphi_{C,C}$ could not be necessarily an indication of large deviations between estimated and calculated B-field profiles: e.g., the largest deviation between $\Delta\varphi_{50,E}$ and $\Delta\varphi_{50,C}$, 11.7° is observed for year 1995, but the relevant value of $\varepsilon_{B,50}$ is 4.40%, i.e., the maximum percent difference between estimated and calculated B-field profiles is quite small. As a consequence, the most decisive indication about the correctness of the estimate of continuous B-field is always the deviation between calculated and estimated B-field profiles.

According to this guideline, in order to assess fully the performance of the proposed heuristic formulas, continuous B-field profiles obtained via (15)–(25) have been compared with alternative continuous B-field profiles computed by inserting in (10), (11) noticeable values of phase-shift angle $\Delta\varphi$ that could be chosen as alternative aprioristic guesses of $\Delta\varphi_{C,E}$: first of all, the central value, $\Delta\varphi_{\mathrm{cen}}$ and the arithmetic average, $\Delta\varphi_{\mathrm{ave}}$, of the sampling distribution of $\Delta\varphi$; in addition, $\Delta\varphi_Q = 0°$, 90°, 180°, 270°, i.e., the values that fix the boundaries of the four quadrants of the periodic variation of $\Delta\varphi$. The relevant deviations between calculated B-field profiles and B-field profiles obtained via such aprioristic guesses of continuous $\Delta\varphi$ are also reported in Table II; they are defined as follows:

$$\varepsilon_{\mathrm{cen},B,50} = 100\max\{|B_{50}(\Delta\varphi_{\mathrm{cen}}) - B_{50,C}|/B_{50,C}\} \quad (28)$$

$$\varepsilon_{\mathrm{ave},B,m} = 100\max\{|\langle B(\Delta\varphi_{\mathrm{ave}})\rangle - \langle B\rangle_C|/\langle B\rangle_C\} \quad (29)$$

$$\varepsilon_{Q,B,50} = 100\max\{|B_{50}(\Delta\varphi_Q) - B_{50,C}|/B_{50,C}\} \quad (30)$$

$$\varepsilon_{Q,B,m} = 100\max\{|\langle B(\Delta\varphi_Q)\rangle - \langle B\rangle_C|/\langle B\rangle_C\}. \quad (31)$$

As Table II shows, $\varepsilon_{\text{cen},B,50}$ ranges from 7.77% to 29.2% and $\varepsilon_{\text{ave},B,m}$ from 9.42% to 32.5%, hence they are much higher than the relevant values of $\varepsilon_{B,50}$ and $\varepsilon_{B,m}$, thereby confirming that the central value and the arithmetic average of the sampling distribution of $\Delta\varphi$ are unsatisfactory guesses of $\Delta\varphi_{50}/\langle\Delta\varphi\rangle$, due to the aforementioned inconsistencies in the sampling distribution. This is also witnessed by the fact that $\Delta\varphi_{\text{cen}}$ ranges from 179° to 225° and $\Delta\varphi_{\text{ave}}$ from 192° to 244°, thus far outside the relevant ranges of $\Delta\varphi_{50,C}$ and $\langle\Delta\varphi\rangle_C$[6].

As far as $\varepsilon_{0,B,50}$ and $\varepsilon_{0,B,m}$ are concerned, it can be observed that their values are always very high: the former ranges from 16.3% to 96.5%, while the latter from 21.1% to 81.4%, hence far larger than the relevant values of $\varepsilon_{B,50}$ and $\varepsilon_{B,m}$. Quite high values, though smaller, are observed also for $\varepsilon_{180,B,50}$ and $\varepsilon_{180,B,m}$: the former ranges from 13.7% to 29.2%, while the latter from 25.6% to 49.1%, hence far larger than the relevant values of $\varepsilon_{B,50}$ and $\varepsilon_{B,m}$. This involves that 0° and 180° are both unacceptable guesses for the values of $\Delta\varphi_{50}/\langle\Delta\varphi\rangle$, at least for the considered line; nevertheless, such values are often claimed by practicians as the only possible—or most frequently occurring—values of phase-shift angle for ACDOHLs, since currents are often considered as being either in phase ("currents in the same direction," i.e., $\Delta\varphi = 0°$) or in opposition ("currents in the opposite direction," i.e., $\Delta\varphi = 180°$).

As far as $\varepsilon_{90,B,50}$ and $\varepsilon_{90,B,m}$ are concerned, it can be observed that their values are low for years 1994, 1995, 1996, 1998 (the former ranges from 3.26% to 3.83%, while the latter from 1.69% to 5.18%): for these years they are comparable to the values of $\varepsilon_{B,50}$ and $\varepsilon_{B,m}$: in particular, $\varepsilon_{90,B,50}$ is lower than $\varepsilon_{B,50}$ twice, $\varepsilon_{90,B,m}$ is lower than $\varepsilon_{B,m}$ three times out of four. Nevertheless, for years 1997, 1999, 2000 and 2001 $\varepsilon_{90,B,50}$ and $\varepsilon_{90,B,m}$ are much higher (the former ranges from 9.66% to 46.1%, while the latter from 7.95% to 32.2%): for these years they are significantly larger than $\varepsilon_{B,50}$ and $\varepsilon_{B,m}$, particularly as far as the median value is concerned. Thus it can be concluded that an aprioristic guess of $\Delta\varphi = 90°$ could perform the same as $\Delta\varphi_{50,E}$ and maybe better than $\langle\Delta\varphi\rangle_E$ over one half of the database recording period of the treated line, but over the other half it performs much worse than $\Delta\varphi_{50,E}$ and $\langle\Delta\varphi\rangle_E$, particularly as far as the median B-field estimate (i.e., the most important one) is concerned; in particular, for years 1999 and 2001 $\Delta\varphi = 90°$ is far from a correct description of the load regime of the line. Moreover, while an aprioristic guess of 90° could be hardly justified for lines different from that analyzed here, the innovative heuristic estimates proposed here find some support from basic power system relationships (see Section III).

Coming to $\varepsilon_{270,B,50}$ and $\varepsilon_{270,B,m}$, their values are similar to those of $\varepsilon_{90,B,50}$ and $\varepsilon_{90,B,m}$, though a bit higher. Indeed, for years 1994, 1995, 1996, 1998 the former ranges from 2.17% to 5.86%, the latter from 1.81% to 7.13%, whereas for years 1997, 1999, 2000 and 2001 the former ranges from 12.5% to 50.4%, the latter from 11.7% to 35.4%. Therefore, the above considerations about $\Delta\varphi = 90°$ hold a fortiori as to $\Delta\varphi = 270°$. In conclusion, also $\Delta\varphi = 270°$ should be regarded as a worse estimate of $\Delta\varphi_{50}/\langle\Delta\varphi\rangle$ than the proposed heuristic formulas for the examined line, as well as not aprioristically justifiable in general for other lines.

[6]Though some similarities between B-field profiles relevant to $\Delta\varphi$ and $360° - \Delta\varphi$ are observed, nevertheless these profiles differ more or less [12].

TABLE III
MEDIAN ($I_{\text{I},50}, I_{\text{II},50}$) AND MEAN ($\langle I_{\text{I}}\rangle, \langle I_{\text{II}}\rangle$) CURRENTS OF CIRCUITS I, II OF THE TREATED LINE, CORRESPONDING PERCENT DIFFERENCE AND B-FIELD ERROR PARAMETER FROM 1994 TO 2001

year	$I_{I,50}$ [A]	$I_{II,50}$ [A]	$\Delta I_{\%,50}$ [%]	$\varepsilon_{B,50}$ [%]	$\langle I_I\rangle$ [A]	$\langle I_{II}\rangle$ [A]	ΔI_{100} [%]	$\varepsilon_{B,m}$ [%]
'94	165	935	80.3	1.44	182	923	82.4	2.64
'95	120	764	80.9	4.40	141	737	84.3	3.69
'96	132	685	77.1	4.37	156	682	80.7	4.59
'97	152	589	70.6	4.67	180	613	74.2	6.06
'98	154	782	77.0	3.16	179	779	80.3	3.96
'99	150	563	69.7	2.51	175	578	73.4	11.3
'00	173	638	67.3	5.65	196	599	72.9	8.88
'01	197	519	54.1	7.60	231	503	62.0	17.5

V. DISCUSSION

As shown by the detailed analysis of the applicative results carried out at Section IV, the proposed heuristic formulas for the estimation of continuous phase-shift angle provide a quite good continuous B-field estimation for the considered line over the whole database recording period, and perform surely better than other commonly-used aprioristic guesses. Though being applied to a particular line, the methods and the results illustrated here are expected to be valid in general for independent-circuit ACDOHL, provided that the basic simplifying hypotheses listed at Section II hold, as is mostly the case for ACDOHL. However, even if these hypotheses are not fully matched, this study provides simple and useful tools for a preliminary analysis, that can help in interpreting the results provided by more refined and complex calculations.

A further extensive investigation of the whole line database has been carried out for gaining a deeper insight into the statistical features of the sampling distributions of powers and other quantities related to continuous values of both phase-shift angle and B-field. In particular, an uneven current distribution between the two circuits over the years is observed, with circuit I carrying always less current and power than circuit II; this can be seen in Table III, that quotes median and mean currents of circuits I, II, the corresponding percent difference between median/mean line currents, $\Delta I_{\%,50}/\langle\Delta I_{\%}\rangle$, and the relevant median/mean B-field error parameter, $\varepsilon_{B,50}/\varepsilon_{B,m}$, already listed in Table II. Table III shows that there is some correlation between $\varepsilon_{B,50}/\varepsilon_{B,m}$ and $\Delta I_{\%,50}$ and $\langle\Delta I_{\%}\rangle$, so that the higher is $\Delta I_{\%,50}/\langle\Delta I_{\%}\rangle$, the lower is $\varepsilon_{B,50}/\varepsilon_{B,m}$. Nonetheless, this and other interesting features of the database gave no decisive indication for an improvement of the proposed estimates for continuous phase-shift angle and B-field values.

VI. CONCLUSION

This paper reviews the main concepts about the computation of historical magnetic fields from ac overhead transmission

lines and possible errors relevant to the time-varying nature of line load, thereby showing that a proper use of historical load databases is essential for associating residential magnetic fields with load diagrams of the lines. Moreover, innovative heuristic formulas for ac double-circuit overhead transmission lines with independent circuits are proposed, that enable line databases to be used accounting for the non-trivial phase-shift effects between circuit currents of these lines. Such formulas provide, via one single fixed-load calculation, a good approximation of the median/mean magnetic field profiles over a reference operational period of the line, thereby easing and shortening considerably the estimation of median/mean magnetic field. The proposed formulas have been shown to have very satisfactory performances for an existing line over all the years of availability of line database, i.e., from 1994 to 2001, especially as far as the median estimate over the whole year is concerned, which is of primary importance from the viewpoint of evaluating the exposure levels to magnetic fields from power lines.

Though being applied to a particular line for the sake of illustration, the results and the methods discussed here are expected to be valid for ACDOHLs in general. However, even when not fully applicable, this analysis provides useful tools that can help in interpreting the results provided by more sophisticated calculation methods.

Acknowledgment

The author gratefully acknowledges the contributions of Dr. A. Giorgi (TERNA S.p.A.) for supplying line database and of Dr. P. Concas for help with the calculations.

References

[1] N. Wertheimer and E. Leeper, "Electrical wiring configurations and childhood cancer," *Am. J. Epidemiology*, vol. 109, pp. 273–284, 1979.

[2] T. Jones, "EMF wire code research," *IEEE Power Eng. Rev.*, vol. 13, no. 11, pp. 10–12, Nov. 1993.

[3] G. Mezei, M. Gadallah, and L. Kheifets, "Residential magnetic field exposure and childhood brain cancer: A meta-analysis," *Epidemiology*, vol. 19, no. 3, pp. 424–430, May 2008.

[4] *IEEE Standard for Safety Levels With Respect to Human Exposure to Electromagnetic Fields, 0 to 3 kHz*, IEEE Standard PC95.6-2002.

[5] "Guidelines for limiting exposure to time varying electric, magnetic and electromagnetic fields (up to 300 GHz)," *Health Phys.*, vol. 74, pp. 494–522, Apr. 1998, ICNIRP.

[6] "Council recommendation of July 12th 1999 on the limitation of exposure of the general public to electromagnetic fields (0–300 GHz)," *Official J. Eur. Comm.*, vol. L199, Jul. 1999, European Union.

[7] Decreto del Presidente del Consiglio dei Ministri July 8th 2003, G.U. n. 200 August 29, 2003 (in Italian).

[8] [Online]. Available: http://www.who.int/topics/electromagnetic_fields/en/

[9] K. C. Jaffa, H. Kim, and T. E. Aldrich, "The relative merits of contemporary measurements and historical calculated fields in the Swedish childhood cancer study," *Epidemiology*, vol. 11, no. 3, pp. 353–356, May 2000.

[10] M. Feychting and A. Ahlbom, "With regard to the relative merits of contemporary measurements and historical calculated fields in the Swedish childhood cancer study," *Epidemiology*, vol. 11, no. 3, pp. 357–358, May 2000.

[11] G. Mazzanti, "The role played by current phase-shift on magnetic field established by double-circuit overhead transmission lines. Part II: Dynamic analysis," *IEEE Trans. Power Del.*, vol. 21, no. 2, pp. 949–958, Apr. 2006.

[12] G. Mazzanti, "The role played by current phase-shift on magnetic field established by double-circuit overhead transmission lines. Part I: Static analysis," *IEEE Trans. Power Del.*, vol. 21, no. 2, pp. 939–948, Apr. 2006.

[13] G. Mazzanti, "The calculation of exposure levels of general public to magnetic field from ac overhead transmission lines," in *Proc. IEEE PES General Meeting*, San Francisco, CA, Jun. 12–16 2005, ISBN 0-7803-9156-X/05.

[14] "Magnetic fields from electric power lines. Theory and comparison to measurements," *IEEE Trans. Power Del.*, vol. 3, pp. 2127–2136, Oct. 1988, IEEE Magnetic Fields Task Force.

[15] J. Swanson, "Magnetic fields from transmission lines: Comparison of calculations and measurements," *Proc. Inst. Elect. Eng.-Gener. Transm. Distrib.*, vol. 142, pp. 481–486, Sept. 1995.

[16] B. M. Weedy and B. J. Cory, *Electrical Power Systems*, 4th ed. New York: Wiley, 1998.

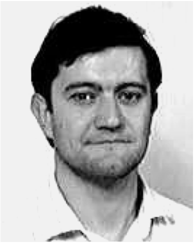

Giovanni Mazzanti (M'04) was born in Bologna, Italy, on July 12, 1962. He received the master degree in nuclear engineering and the Ph.D. in electrical engineering from the University of Bologna, in 1986 and 1992, respectively.

Currently, he is an Associate Professor of HV Engineering and Power Quality at the University of Bologna. His scientific interests are reliability and diagnostics of HV insulation, and human exposure to electromagnetic fields. He is the author or coauthor of more than 160 published papers (about 40 in IEEE TRANSACTIONS).
