# Supplementary material for: Magnetic Fields and Cancer: Epidemiology, Cellular Biology, and Theranostics
Source: Int J Mol Sci. 2022 Jan 25;23(3):1339. doi: 10.3390/ijms23031339 (PMC8835851; doi:10.3390/ijms23031339)
Supplement: Supplementary file 1 [file ijms-23-01339-s001.zip › Supplementary Data Set S1/MF and Cancer.Data/PDF/2806912330/kwf020.pdf]

American Journal of Epidemiology
Vol. 156, No. 3
Copyright © 2002 by the Johns Hopkins Bloomberg School of Public Health
All rights reserved
Printed in U.S.A.
DOI: 10.1093/aje/kwf020

Endometrial Cancer Incidence in Relation to Electric Blanket Use

Jane A. McElroy[1,2], Polly A. Newcomb[1,3], Amy Trentham-Dietz[1,4], John M. Hampton[1], Marty S. Kanarek[2,4], and Patrick L. Remington[1,4]

[1] University of Wisconsin Comprehensive Cancer Center, Madison, WI.
[2] Gaylord Nelson Institute for Environmental Studies, University of Wisconsin, Madison, WI.
[3] Fred Hutchinson Cancer Research Center, Seattle, WA.
[4] Department of Population Health Sciences, University of Wisconsin, Madison, WI.

Received for publication August 28, 2001; accepted for publication March 17, 2002.

Endometrial cancer is associated with endogenous and exogenous estrogen excess. Some investigators have posited that electromagnetic fields may influence cancer risk through estrogenic hormonal mechanisms; however, there have been no studies reporting on electric blanket exposure in relation to endometrial cancer. The authors examined this possible association between endometrial cancer risk and electric blanket or mattress cover use as part of a population-based, case-control study. This analysis included incident endometrial cancer cases 40–79 years of age, interviewed during 1994 (n = 148; response rate, 87%) and identified from the Wisconsin tumor registry. Female controls of similar age were randomly selected from population lists (n = 659; response rate, 85%). Information regarding electric blanket and mattress cover use and endometrial cancer risk factors was obtained through structured telephone interviews approximately 1 year after diagnosis. After adjustment for age, body mass index, and postmenopausal hormone use, the risk of endometrial cancer was similar among ever users (odds ratio = 1.04, 95% confidence interval: 0.70, 1.55) and among current users (odds ratio = 0.87, 95% confidence interval: 0.49, 1.54) as compared with never users. Despite its small size and potential misclassification of exposure, this study provides evidence against an association between electric blanket or mattress cover use and endometrial cancer. *Am J Epidemiol* 2002;156:262–7.

case-control studies; electromagnetic fields; endometrial neoplasms

Abbreviation: CI, confidence interval.

Exposure to electromagnetic fields has been suggested to increase the risk of hormone-dependent cancers such as breast, ovarian, testicular, prostatic, and endometrial (1–8). One suggested pathway includes deficient melatonin function (9). This pineal secretory product has antiestrogenic properties (2, 10–12), and melatonin secretion is significantly reduced in postmenopausal women (13), the group with the highest incidence of endometrial cancer (14, 15). Some studies have shown that melatonin secretion decreases in response to 50/60-Hz magnetic field exposure (16–20) such as produced by electric blankets—the greatest contributor to electromagnetic exposure of in-home appliances on a time- and body-average basis (21).

Although some investigators have suggested that electromagnetic field exposure may influence cancer risk through its effects on hormonal mechanisms, the failure to observe a clear association between electromagnetic field exposure from electric blanket or mattress cover use and risk of breast cancer (22–28) or prostatic cancer (8) argues against this. Because the relation between hormonal exposures is stronger for endometrial cancer than for breast cancer, an association between electric blanket use and endometrial cancer risk might be more readily detected (29–31). We present data on endometrial cancer risk in relation to the use of electric blankets or mattress covers from our population-based, case-control study.

Correspondence to Jane A. McElroy, University of Wisconsin Comprehensive Cancer Center, Room 752 WARF, 610 Walnut St., Madison, WI 53726 (e-mail: jamcelroy@wisc.edu).

Downloaded from https://academic.oup.com/aje/article/156/3/262/71397 by guest on 19 November 2021

MATERIALS AND METHODS

All participants were female Wisconsin residents 40–79 years of age. Invasive endometrial cancer cases (diagnosed between 1991 and 1994) were identified by a mandatory statewide cancer registry. According to an institutionally approved protocol, the physician of record for each eligible case was contacted by mail to obtain permission to approach the subject. Eligibility was limited to cases with listed telephone numbers, driver's licenses verified by self-report (if aged less than 65 years) and, if aged more than or equal to 65 years, a Medicare card (n = 856). A total of 745 cases (87.0 percent of eligible cases) participated in the study interview between June 1991 and June 1995. The reasons for nonparticipation included physician refusal (n = 6), subject refusal (n = 53), inability to locate the subject (n = 2), and death (n = 50). Of the interviewed cases, 98.0 percent had histologic confirmation of invasive endometrial carcinoma according to the tumor registry report.

Community controls were selected randomly from lists of licensed drivers (if aged less than 65 years) and Medicare beneficiary files compiled by the Health Care Financing Administration (if aged 65–79 years). The controls were selected at random to yield an age distribution similar to that of the cases in a parallel breast cancer study (22) and were those who met the eligibility criterion of having a listed telephone number. Controls were eligible for the study if they reported no previous diagnosis of uterine cancer. Of the 4,362 eligible controls, 521 refused, 35 could not be located, and 88 were deceased. A total of 3,718 (85.2 percent) completed the study interview. Exclusions after the interview included 1,304 controls who reported a history of hysterectomy and six women for whom interview data were determined to be inconsistent. In all, 2,408 controls were available for analysis.

The structured 45-minute telephone interview elicited information on known or suspected risk factors of breast cancer including exogenous hormone use, reproductive experiences, physical activity, medical and family history, and demographics prior to an assigned reference date. For cases, this was the date of diagnosis of the endometrial cancer. For comparability, controls were assigned a reference date based on similarly aged cases (within 5-year strata). Trained study staff conducted interviews by telephone without prior knowledge of the subjects' disease status. For 82 percent of the cases and 95 percent of the controls, the interviewer remained unaware of the case-control status of the subject until the end of the interview.

Cases and controls were asked about their lifetime exposures including electric blanket or mattress cover use. Women were queried, "Have you ever slept with an electric blanket or mattress cover when it was turned on?" Women who reported a positive history were further asked, "How long did you use it?" Respondents were also asked if they were using an electric blanket or mattress cover during the reference year. ("Were you using it in (the reference year)?") Electric blanket use history was near the beginning of the interview.

Subjects for analysis

The history of electric blanket or mattress cover use was included in the questionnaire from June 1994 to December 1994. Analysis was limited to women interviewed during this period (159 cases and 690 controls). We excluded 11 cases and 31 controls who could not provide complete information on electric blanket usage or other covariates. Therefore, 148 cases and 659 controls remained for this analysis.

Reliability substudy

To assess the reliability of the questionnaire, we interviewed a sequential sample of controls again. After an average of 3.4 months (range, 2–6 months), 186 controls (83 percent) were successfully recontacted and reinterviewed. Cohen's kappa with 95 percent lower confidence limits measured the reliability of the subject's responses to the question about never/ever and never/former/current electric blanket or mattress cover use (32). The intraclass correlation coefficient measured the reproducibility of the reported duration of electric blanket or mattress cover use (32). The kappa for never/ever use was 0.82 (lower confidence limit, 0.74), and the kappa for never/former/current use was 0.83 (lower confidence limit, 0.75). The intraclass correlation coefficient for duration of electric blanket or mattress cover use was 0.77 (lower confidence limit, 0.72).

Analysis

Odds ratios and 95 percent confidence intervals from logistic regression models were used to estimate risk (33). Conditional models were stratified according to age. Covariates for the models were chosen by forward stepwise regression (p entry = 0.20, p removal = 0.30). Possible covariates included known or suspected risk factors for endometrial cancer: menopausal status, age at menopause, body mass index, parity, history of smoking, hormone replacement therapy use, education, and family history of endometrial cancer. After forward stepwise regression, the covariates remaining in the model were menopausal status (premenopausal or postmenopausal, defined as no menstruation for ≥6 months), body mass index (quartiles, self-reported reference weight (kg) divided by height (m) squared), and hormone replacement therapy use (never, former, current). The participants' responses to questions about factors at the reference date are defined as current.

Ever use versus never use of an electric blanket or mattress cover was included in the logistic regression models as a dichotomous variable. Indicator variables were created to represent never/former/current and duration-of-use categories of electric blanket or mattress cover users. Tests for p trends in electric blanket use were evaluated by entering an ordinal term representing never use and tertiles of duration of use into the regression models.

RESULTS

Compared with controls, women with endometrial cancer were more likely to have a heavier body mass, to be

Downloaded from https://academic.oup.com/aje/article/156/3/262/71397 by guest on 19 November 2021

TABLE 1. Characteristics of women with endometrial cancer and controls aged 40–79 years, Wisconsin, 1991–1994

Characteristic	Cases (%) (n = 148)	Controls (%)* (n = 659)	Odds ratio†	95% confidence interval†
Body mass index (kg/m²)				
<22.7	13	26	1	
22.7–25.5	18	24	1.52	0.80, 2.88
25.6–29.0	20	22	1.6	0.84, 3.03
≥29.1	45	22	3.72	2.10, 6.57
Unknown	5	6	1.65	0.66, 4.10
Parity				
0	14	10	1	
1	7	8	0.81	0.34, 1.89
2	26	22	0.91	0.48, 1.73
≥3	53	60	0.68	0.38, 1.20
Smoking history				
Never	66	55	1	
Former	24	29	0.74	0.48, 1.15
Current	10	16	0.53	0.30, 0.96
Menopausal status				
Postmenopausal	83	84	1	
Premenopausal	17	16	2.19	0.96, 4.97
Age at menopause (years)‡				
<45	9	12	1	
45–49	21	21	1.43	0.64, 3.16
50–54	37	39	1.25	0.60, 2.60
≥55	20	17	1.61	0.71, 3.64
Unknown	13	11	1.72	0.71, 4.14
Hormone replacement therapy‡				
Never	65	82	1	
Former	10	6	1.96	0.95, 4.02
Current	25	12	3.36	1.93, 5.85

* Percentages are age adjusted to the distribution of cases.
† Logistic regression models conditional on age.
‡ Among postmenopausal women only.

Downloaded from https://academic.oup.com/aje/article/156/3/262/71397 by guest on 19 November 2021

nonsmokers, or (for postmenopausal women) to use hormone replacement therapy (table 1). The mean ages at the reference date were 63.1 years for cases and 65.0 years for controls.

After adjustment for age, women with endometrial cancer were slightly more likely than controls to have ever used an electric blanket or mattress cover (table 2). Of the endometrial cancer cases, 46 percent had ever used an electric blanket or mattress cover compared with 42 percent of the controls.

Among controls, ever users of an electric blanket or mattress cover were more likely than never users to have a history of hormone replacement use (21 percent vs. 15 percent, p value = 0.06). No other risk factor showed a statistically significant difference between never/ever electric blanket or mattress cover users in the control population.

Compared with women who had never used an electric blanket or mattress cover, the multivariable-adjusted odds ratio of endometrial cancer for women who had ever used an electric blanket or mattress cover was 1.04 (95 percent

TABLE 2. Multivariate odds ratios of endometrial cancer according to patterns of electric blanket use, Wisconsin, 1991–1994

	Cases		Controls*		Odds ratio†	95% confidence interval†	Odds ratio‡	95% confidence interval‡
	No.	%	No.	%				
Never users	80	54	387	58	1		1	
Ever users§	68	46	272	42	1.19	0.82, 1.73	1.04	0.70, 1.55
Former users	44	30	174	27	1.21	0.79, 1.86	1.12	0.71, 1.76
Current users	23	16	98	15	1.11	0.66, 1.88	0.87	0.49, 1.54
Duration of use (months)§								
1–7	23	16	85	13	1.27	0.74, 2.19	1.24	0.71, 2.17
8–30	23	16	86	13	1.19	0.70, 2.03	1.00	0.56, 1.78
>30	22	15	93	8	1.24	0.72, 2.13	1.00	0.56, 1.80
p trend						0.35		0.97

* Percentages are age adjusted to the distribution of cases.
† Logistic regression models conditional on age.
‡ Logistic regression models conditional on age and adjusted for body mass index, menopausal status, and hormone replacement therapy.
§ Former/current user status was not known for one case. The duration of use was not known for eight controls.

confidence interval (CI): 0.70, 1.55) (table 2). The odds ratio of endometrial cancer associated with current use was 0.87 (95 percent CI: 0.49, 1.54). The odds ratio for former users was 1.12 (95 percent CI: 0.71, 1.76). An association between endometrial cancer and duration of electric blanket or mattress cover use was not observed (p trend = 0.97).

A modest interaction between ever use of an electric blanket or mattress cover and body mass index was suggested in relation to the risk of endometrial cancer; the positive association between increasing body mass and endometrial cancer risk was attenuated among users of electric blankets (p value = 0.03). However, no significant interactions were discerned between hormone replacement therapy or any other risk factor and ever use of an electric blanket or mattress cover.

DISCUSSION

In this population-based study, electric blanket or mattress cover use was not significantly associated with endometrial cancer risk. To our knowledge, this is the first study to assess the association between electric blanket or mattress cover use and endometrial cancer risk. Our confidence in these findings is strengthened by high participation rates (87 percent of eligible cases and 85 percent of eligible controls) and by our ability to control for many potentially confounding factors.

Some limitations should be considered in interpreting our results. Because all information in this study was based on reports from women themselves, some bias in reporting may have occurred. However, in our study population, the reliability of reported electric blanket or mattress cover use was reassuring for both ever/never use and currency of use. The small sample size constrained our analysis and limited the precision of our estimates. Thus, we cannot rule out the existence of a small increase (or decrease) in risk. Finally, our interviewers were blinded as to the case or control status for more than 80 percent of the participants, thereby minimizing differential interaction by the interviewer to the respondent.

Complete and accurate reporting of electromagnetic field exposure information is critical to any study of this type. Our history was brief, and a more complete exposure history might be possible. Some details are likely to be readily obtained, such as use patterns (throughout the night, seasonal, or bed warming only). However, other characteristics of exposure such as purchase date (engineering changes in wire configurations occurred in the 1980s) and style (various wiring configurations alter potential exposure levels) (21) would be difficult to collect. In addition, other sources of electromagnetic field exposure such as occupational history or other in-home exposures were not obtained.

Recent findings have suggested two distinct types of endometrial carcinoma: type I and type II. These carcinomas have fundamental differences in the hormonal milieu in which the tumors develop. Type I carcinoma is associated with the usual risk factors of hyperestrogenism, whereas type II seems largely unrelated to hormonal imbalance (34, 35). This study did not analyze the different histopathologic types separately. If electromagnetic field exposure is associated with only one type of endometrial cancer, then the authors' findings might be attenuated.

Researchers have suggested several mechanisms for a possible oncostatic action of melatonin (36) such as scavenging radicals (37) enhancing immunology (38), stimulating gap-junction intercellular communication (39, 40), and/or downregulating circulating levels of neuroendocrine reproductive axis hormones (41). Experimental data with rodents of 50/60-Hz magnetic field exposure and melatonin secretion are inconclusive (42–45). One recent study

Downloaded from https://academic.oup.com/aje/article/156/3/262/71397 by guest on 19 November 2021

reported that melatonin inhibited proliferation of a human endometrial cancer cell line (Ishikawa cells) in vitro (46). In a clinical study, women with endometrial cancer had significantly lower serum concentrations of melatonin than those of healthy controls (47). Human experimental studies that examined melatonin changes from magnetic field exposure have failed to observe consistent alterations (19, 20, 48–50). In contrast, a few epidemiologic studies have suggested that magnetic field exposure alters melatonin levels (17, 51–54). Some researchers suggest a possible cumulative effect of magnetic field exposure on the stability of individual melatonin measurement over time (43, 49). Chronic exposure conditions in humans have yet to be adequately explored; therefore, it is not possible to make a definite conclusion about the effect of magnetic field exposure on melatonin levels.

In conclusion, to the authors' knowledge, this is the first report on the topic of endometrial cancer and exposure to electric blanket or mattress covers. Although the authors' findings do not demonstrate an association, the study's small size cannot rule out that an association exists. These findings, when considered with subsequent research, may provide further insight into the effects of electromagnetic field exposure on human health.

ACKNOWLEDGMENTS

This work was supported in part by grants from the American Cancer Society (ACS PDT–446) and the National Institutes of Health (CA 47147).

The authors are grateful to Drs. Barry Storer, Kathleen Egan, Meir Stampfer, Walter Willett, Linda Titus-Ernstoff, and John Baron for advice and criticism at various stages in this study; the staff of the Wisconsin Cancer Reporting System for assistance with data; Felicia Roberts, Lorene Seman, Lisa Sieczkowski, Emogene Dodsworth, Amy Benedict, Carol Magoon, Jerry Phipps, and Dennis Anderson for data collection and study management; and Mary Pankratz for technical support.

REFERENCES

1. Loomis D, Savitz D, Ananth CV. Electronic work may increase risk of breast cancer deaths. J Environ Health 1994;57:26.
2. Stevens RG. Biologically based epidemiological studies of electric power and cancer. Environ Health Perspect 1993; 101(suppl 4):93–100.
3. Stevens RG. Electric power use and breast cancer: a hypothesis. Am J Epidemiol 1987;125:556–61.
4. Kelsey JL, Whittemore AS. Epidemiology and primary prevention of cancers of the breast, endometrium, and ovary. A brief overview. Ann Epidemiol 1994;4:89–95.
5. Cos S, Sanchez-Barcelo EJ. Melatonin, experimental basis for a possible application in breast cancer prevention and treatment. Histol Histopathol 2000;15:637–47.
6. Floderus B, Stenlund C, Persson T. Occupational magnetic field exposure and site-specific cancer incidence: a Swedish cohort study. Cancer Causes Control 1999;10:323–32.
7. Verreault R, Weiss NS, Hollenbach KA, et al. Use of electric blankets and risk of testicular cancer. Am J Epidemiol 1990; 131:759–62.
8. Zhu K, Weiss NS, Stanford JL, et al. Prostate cancer in relation to the use of electric blanket or heated water bed. Epidemiology 1999;10:83–5.
9. Baldwin WS, Barrett JC. Melatonin: receptor-mediated events that may affect breast and other steroid hormone-dependent cancers. Mol Carcinog 1998;21:149–55.
10. Blask DE, Hill SM. Effects of melatonin on cancer: studies on MCF–7 human breast cancer cells in culture. J Neural Transm Suppl 1986;21:433–49.
11. Hill SM, Blask DE. Effects of the pineal hormone melatonin on the proliferation and morphological characteristics of human breast cancer cells (MCF–7) in culture. Cancer Res 1988;48: 6121–6.
12. Wurtman RJ. Melatonin in humans. J Neural Transm Suppl 1986;21:1–8.
13. Sack RL, Lewy AJ, Erb DL, et al. Human melatonin production decreases with age. J Pineal Res 1986;3:379–88.
14. Ries LAG, Eisner MP, Kosary CL, et al. SEER cancer statistics review, 1973–1997. Bethesda, MD: National Cancer Institute, 2000. (NIH publication no. 00–2789).
15. Grady D, Ernster VL. Endometrial cancer. In: Schottenfeld D, Fraumeni JF Jr, eds. Cancer epidemiology and prevention. 2nd ed. Oxford, United Kingdom: Oxford University Press, 1996: 1058–89.
16. Wilson BW, Wright CW, Morris JE, et al. Evidence for an effect of ELF electromagnetic fields on human pineal gland function. J Pineal Res 1990;9:259–69.
17. Burch JB, Reif JS, Yost MG, et al. Nocturnal excretion of a urinary melatonin metabolite among electric utility workers. Scand J Work Environ Health 1998;24:183–9.
18. Kato M, Honma K, Shigemitsu T, et al. Effects of exposure to a circularly polarized 50-Hz magnetic field on plasma and pineal melatonin levels in rats. Bioelectromagnetics 1993;14:97–106.
19. Karasek M, Woldanska-Okonska M, Czernicki J, et al. Chronic exposure to 2.9 mT, 40 Hz magnetic field reduces melatonin concentrations in humans. J Pineal Res 1998;25:240–4.
20. Wood AW, Armstrong SM, Sait ML, et al. Changes in human plasma melatonin profiles in response to 50 Hz magnetic field exposure. J Pineal Res 1998;25:116–27.
21. Florig HK, Hoburg JF. Power-frequency magnetic fields from electric blankets. Health Phys 1990;58:493–502.
22. McElroy JA, Newcomb PA, Remington PL, et al. Electric blanket or mattress cover use and breast cancer incidence in women 50–79 years of age. Epidemiology 2001;12:613–17.
23. Laden F, Neas LM, Tolbert PE, et al. Electric blanket use and breast cancer in the Nurses' Health Study. Am J Epidemiol 2000;152:41–9.
24. Zheng T, Holford TR, Mayne ST, et al. Exposure to electromagnetic fields from use of electric blankets and other in-home electrical appliances and breast cancer risk. Am J Epidemiol 2000;151:1103–11.
25. Coogan PF, Aschengrau A. Exposure to power frequency magnetic fields and risk of breast cancer in the Upper Cape Cod Cancer Incidence Study. Arch Environ Health 1998;53:359–67.
26. Gammon MD, Schoenberg JB, Britton JA, et al. Electric blanket use and breast cancer risk among younger women. Am J Epidemiol 1998;148:556–63.
27. Vena JE, Freudenheim JL, Marshall JR, et al. Risk of premenopausal breast cancer and use of electric blankets. Am J Epidemiol 1994;140:974–9.
28. Vena JE, Graham S, Hellmann R, et al. Use of electric blankets and risk of postmenopausal breast cancer. Am J Epidemiol 1991;134:180–5.

Downloaded from https://academic.oup.com/aje/article/156/3/262/71397 by guest on 19 November 2021

29. Newcomb PA, Trentham-Dietz A, Egan KM, et al. Fracture history and risk of breast and endometrial cancer. Am J Epidemiol 2001;153:1071–8.
30. Pike MC, Ross RK. Progestins and menopause: epidemiological studies of risks of endometrial and breast cancer. Steroids 2000;65:659–64.
31. Ballard-Barbash R, Swanson CA. Body weight: estimation of risk for breast and endometrial cancers. Am J Clin Nutr 1996; 63(suppl):437S–41S.
32. Armstrong BK, White E, Saracci R. Principles of exposure measurement in epidemiology. Oxford, United Kingdom: Oxford University Press, 1992.
33. Breslow NE, Day NE, eds. Statistical methods in cancer research. Vol I. The analysis of case-control studies. Lyon, France: International Agency for Research on Cancer, 1980. (IARC scientific publication no. 32).
34. Emons G, Heyl W. Hormonal treatment of endometrial cancer. J Cancer Res Clin Oncol 2000;126:619–23.
35. Sherman ME. Theories of endometrial carcinogenesis: a multidisciplinary approach. Mod Pathol 2000;13:295–308.
36. Brainard GC, Kavet R, Kheifets LI. The relationship between electromagnetic field and light exposures to melatonin and breast cancer risk: a review of the relevant literature. J Pineal Res 1999;26:65–100.
37. Reiter RJ, Tan DX, Osuna C, et al. Actions of melatonin in the reduction of oxidative stress. A review. J Biomed Sci 2000;7: 444–58.
38. Maestroni GJ. The immunoneuroendocrine role of melatonin. J Pineal Res 1993;14:1–10.
39. Trosko JE. Human health consequences of environmentally-modulated gene expression: potential roles of ELF-EMF induced epigenetic versus mutagenic mechanisms of disease. Bioelectromagnetics 2000;21:402–6.
40. Ubeda A, Trillo MA, House DE, et al. Melatonin enhances junctional transfer in normal C3H/10T1/2 cells. Cancer Lett 1995;91:241–5.
41. Molis T, Spriggs L, Hill S. Melatonin modulation of estrogen receptor expression in MCF–7 human breast cancer cells. Int J Oncol 1993;3:687–94.
42. Reiter RJ, Anderson LE, Buschbom RL, et al. Reduction of the nocturnal rise in pineal melatonin levels in rats exposed to 60-Hz electric fields in utero and for 23 days after birth. Life Sci 1988;42:2203–6.
43. Selmaoui B, Touitou Y. Sinusoidal 50-Hz magnetic fields depress rat pineal NAT activity and serum melatonin. Role of duration and intensity of exposure. Life Sci 1995;57:1351–8.
44. Boorman GA, McCormick DL, Ward JM, et al. Magnetic fields and mammary cancer in rodents: a critical review and evaluation of published literature. Radiat Res 2000;153:617–26.
45. Olcese J, Reuss S. Magnetic field effects on pineal gland melatonin synthesis: comparative studies on albino and pigmented rodents. Brain Res 1986;369:365–8.
46. Kanishi Y, Kobayashi Y, Noda S, et al. Differential growth inhibitory effect of melatonin on two endometrial cancer cell lines. J Pineal Res 2000;28:227–33.
47. Grin W, Grunberger W. A significant correlation between melatonin deficiency and endometrial cancer. Gynecol Obstet Invest 1998;45:62–5.
48. Selmaoui B, Lambrozo J, Touitou Y. Magnetic fields and pineal function in humans: evaluation of nocturnal acute exposure to extremely low frequency magnetic fields on serum melatonin and urinary 6-sulfatoxymelatonin circadian rhythms. Life Sci 1996;58:1539–49.
49. Graham C, Cook MR, Sastre A, et al. Multi-night exposure to 60 Hz magnetic fields: effects on melatonin and its enzymatic metabolite. J Pineal Res 2000;28:1–8.
50. Graham C, Cook MR, Riffle DW. Human melatonin during continuous magnetic field exposure. Bioelectromagnetics 1997;18:166–71.
51. Burch JB, Reif JS, Noonan CW, et al. Melatonin metabolite levels in workers exposed to 60-Hz magnetic fields: work in substations and with 3-phase conductors. J Occup Environ Med 2000;42:136–42.
52. Coogan PF, Clapp RW, Newcomb PA, et al. Occupational exposure to 60-Hertz magnetic fields and risk of breast cancer in women. Epidemiology 1996;7:459–64.
53. Forssen UM, Feychting M, Rutqvist LE, et al. Occupational and residential magnetic field exposure and breast cancer in females. Epidemiology 2000;11:24–9.
54. Loomis DP, Savitz DA, Ananth CV. Breast cancer mortality among female electrical workers in the United States. J Natl Cancer Inst 1994;86:921–5.

Downloaded from https://academic.oup.com/aje/article/156/3/262/71397 by guest on 19 November 2021
