# Supplementary material for: Magnetic Fields and Cancer: Epidemiology, Cellular Biology, and Theranostics
Source: Int J Mol Sci. 2022 Jan 25;23(3):1339. doi: 10.3390/ijms23031339 (PMC8835851; doi:10.3390/ijms23031339)
Supplement: Supplementary file 1 [file ijms-23-01339-s001.zip › Supplementary Data Set S1/MF and Cancer.Data/PDF/2820067436/i1943-4618-16-2-55.pdf]

A REVIEW OF PROTOCOLS AND GUIDELINES ADDRESSING THE EXPOSURE OF OCCUPANTS TO ELECTROMAGNETIC FIELD RADIATION (EMFR) IN BUILDINGS

Shabnam Monadizadeh,[1] Charles J. Kibert,[2] Jiaxuan Li,[3] Junghoon Woo,[4] Ashish Asutosh,[4] Samira Roostaie,[4] Maryam Kouhirostami[5]

Downloaded from http://meridian.allenpress.com/jgb/article-pdf/16/2/55/2845768/i1943-4618-16-2-55.pdf by Texas A & M University user on 28 October 2021

HIGHLIGHTS

- A comprehensive review of increasing electromagnetic field radiation (EMFR) impacts on building occupant health
- Scientific evidence identifying adverse effects of EMFR
- Governments and public health agencies are creating regulations to reduce EMFR exposure
- National and international regulations with respect to the thresholds they set to protect human health
- Provides a robust foundation for researchers to use in additional studies of EMFR impacts in various built environment scenarios

ABSTRACT

A significant share of the technology that has emerged over the past several decades produces electromagnetic field (EMFR) radiation. Communications devices, household appliances, industrial equipment, and medical equipment and devices all produce EMFR with a variety of frequencies, strengths, and ranges. Some EMFR, such as Extremely Low Frequency (ELF), Radio Frequency (RF), and Ionizing Range (IR) radiation have been shown to have harmful effects on human health. Depending on the frequency and strength of the radiation, EMFR can have health effects at the cellular level as well as at brain, nervous, and cardiovascular levels. Health authorities have enacted regulations locally and globally to set critical values to limit the adverse effects of EMFR. By introducing a more comprehensive field of EMFR study and

1.Ph.D., Project Manager, Theissen Training Systems, Powell Center for Construction and Environment, M. E. Rinker, Sr. School of Construction Management, University of Florida
2.Holland Professor, Powell Center for Construction and Environment, M. E. Rinker, Sr. School of Construction Management, University of Florida
3.Ph.D. Student, Powell Center for Construction and Environment, M. E. Rinker, Sr. School of Construction Management, University of Florida
4.Ph.D., Powell Center for Construction and Environment, M. E. Rinker, Sr. School of Construction Management, University of Florida
5.Ph.D. Candidate, UF School of Architecture, College of Design, Construction & Planning, University of Florida
6. Ph.D. Student, Powell Center for Construction and Environment, M. E. Rinker, Sr. School of Construction Management, University of Florida
*Corresponding author at: Powell Center for Construction and Environment, M. E. Rinker, Sr. School of Construction Management, University of Florida, 304 Rinker / P.O. Box 115703, Gainesville, FL 32611-5703; email: m.kouhirostami@ufl.edu

practice, architects and designers can design for a safer electromagnetic (EM) indoor environment, and, as building and construction specialists, will be able to monitor and reduce EM radiation. This paper identifies the nature of EMFR in the built environment, the various EMFR sources, and its human health effects. It addresses European and US regulations for EMFR in buildings and provides a preliminary action plan. The challenges of developing measurement protocols for the various EMFR frequency ranges and determining the effects of EMFR on building occupants are discussed. This paper argues that a mature method for measuring EMFR in building environments and linking these measurements to human health impacts will foster occupant health and lead to the adequate development of safeguards for occupants of buildings in future research.

KEYWORDS

electromagnetic field, human health, biological effects of exposure to EMF, electromagnetic field regulation in buildings

Downloaded from http://meridian.allenpress.com/jgb/article-pdf/16/2/55/2845768/i1943-4618-16-2-55.pdf by Texas A & M University user on 28 October 2021

1. INTRODUCTION

Today, more than ever, humans are being exposed to radiation produced by electrical and wireless devices in private and public spaces, either related to environmental or occupational exposures. These devices produce radiation and operate over a wide range of electromagnetic field (EMFR) frequencies that can have major and minor impacts on building occupant health. The widespread presence of EMFR radiation in buildings and its potential dangers should raise concern and spur awareness by building owners, designers, and occupants to, first, understand the quality and behavior of electromagnetic radiation, and, subsequently, to reduce and eliminate its influence. In addition to the known components of the indoor environmental quality (IEQ) such as air quality, lighting, views, odors, chemicals, toxic and combustible materials, and thermal and acoustic comfort, electromagnetic fields have also been proven to have an impact on physical and mental human wellbeing (Tomitsch and Dechant 2015). EMFR pollution is sometimes referred to as *electro-smog* or *electromagnetic smog*.

This research includes a literature review of the effects of EMFR found in buildings on human health, European and US regulations on electromagnetic safety, and comparisons of reference levels of EMFR to the limits set by regulations. The purpose of this research is to better define an optimal EMFR indoor environmental quality (IEQ), one of the key sustainable building principles, to support the health of the occupants. The motivation is to positively influence building design and occupant health, and to further the building science needed to detect and mitigate electromagnetic fields using an interdisciplinary approach.

In 1820, Hans Christian Oersted connected electricity to magnetic forces for the first-time. In the 19th century, Gauss, Ampere, Coulomb, Faraday, Henry, and Maxwell developed the knowledge and mathematical framework for elaborating the relationship between electricity and magnetism (Lambrozo and Souques 2012). Based on its effect on atoms or molecules, EMFR is categorized into two major groups: Nonionizing Radiation (NIR) and Ionizing Radiation (IR). NIR's energy is below the level that ionizes hydrogen (H) and oxygen (O) atoms which is 13.6 electron-volts (eV). NIR is divided into various sub-categories: the static field (0 Hz),

extremely low-frequency (ELF; 0–300 Hz), intermediate frequency (IF; 300 Hz–100 kHz), and radiofrequency EMFR (RF; 10 MHz–300 GHz). IR is characterized by high energy and very short wavelengths and includes gamma- and x-rays. This range of radiation also includes particulate sources such as neutrons, electrons (beta particles), and alpha particles (McColl et al. 2015).

Concerns were first raised about building EMFR in the 1960s and reports were written regarding the health hazards caused by EMFR. In 1979, Wertheimer and Leeper found that the risk of leukemia was double for children who lived in close proximity to power transmission lines. However, it was later determined that the actual cause of their illness was the electrical current in the water pipes (Merzenich et al. 2008; Frei and Röösli 2014). Based on their research into childhood leukemia, Merzenich and colleagues (Martiny 2010) found no elevated risk of childhood leukemia due to electrical transmission lines.

Previous biochemistry research showed that EMFR affects cellular and tissue responses and can alter the synthesis of DNA, RNA and proteins, which in turn causes changes in hormone production, immune responses, gene expressions, cell growth rate, and cell differentiation (Tenforde, 1991; Kocaman, et al. 2018).

There are many sources of EMFR radiation, including electrical and wireless devices such as electrical household devices, telecommunication devices, radiofrequency identification (RFID) tagging systems, industrial induction heating, microwave ovens, data transmission systems, radars, antennas, medical diagnostic and treatment devices, X-Rays, computerized tomography (CT) scanners, radon gas emitted from soil and rocks, food contaminants, nuclear leakage, and many others (Saliev et al. 2019; Chiaramello et al. 2019). Buildings are equipped with electrical conduits, switches, plugs, panel boards and other devices that control the facility's electric power and are therefore sources or pathways for EMFR. To ensure good indoor environmental quality for occupant health, both international and American health authorities and institutions have published guidelines and reference levels that suggest limits on building occupant exposure to EMFR (WHO 2007).

This paper is structured as follows: Section 2 provides a general literature review of EMFR knowledge, previous research, and current regulations. Section 2.1 reviews the various frequency ranges of EMFR and their known impacts on human health. Section 2.2 shows sources of EMFR in building and the urban surrounding and effect to occupancy health. Section 2.3 discusses the principles and limitations suggested by international organizations and introduces basic measurement approaches. Analysis and future directions of EMFR impact research are discussed in Section 3 and Section 4 is the summary and conclusion.

2. BACKGROUND

2.1 EMFR Frequency Range and Affects on Human Health

This section explains the subcategories of NIR and its known health effects.

2.1.1 Static fields

Static electric and magnetic fields do not propagate, nor do they correspond to any type of wave or flux (Aurengo and Perrin 2012). A static electric field is associated with the presence of a fixed electrical charge, whereas a magnetic field is caused by the movement of an electric charge. Static electric fields are created by the surface of the earth both in good weather (100–150 V/m) and in stormy weather (10–15 kV/m). They are also found around high voltage equipment and electrical transmission lines. An example of static EMFR is the separation of positive and

Downloaded from http://meridian.allenpress.com/jgb/article-pdf/16/2/55/2845768/i1943-4618-16-2-55.pdf by Texas A & M University user on 28 October 2021

negative changes caused by rubbing electrically insulated objects, causing a separation of positive and negative charges. The origins of the static magnetic fields vary. For instance, the geomagnetic field is linked to the magma at the center of the earth (30 to 70 μT depending on the geographic location). Static magnetic fields are also associated with electrical transmission lines, rail transport (traction and magnetic levitation), magnetic resonance imaging (MRI) devices which have fields 50,000 times greater than the earth's magnetic field, with the electrochemical industry, and with generators and accelerators (Veyret and Perrin 2012).

2.1.2 Weak Range Radiation

The extremely low frequency electric (ELF) electromagnetic range is below 300 Hz and has very long wavelengths (around 108 meters) (See Table 1). The uncoupled electric and magnetic components of the field interact differently with the human body. Biochemical research on the effects of ELF has shown that this frequency range can have effects on cellular and tissue responses; can cause alterations in the synthesis of DNA, RNA and proteins; produces changes in hormone production, immune responses, gene expressions, cell growth rate and cell differentiation (Tenforde 1991). For example many studies show the direct effect of nonthermal LF on the immune system (Doyon and Johansson 2017; Salehi et al. 2017; He et al. 2014; Abu Khadra et al. 2015; Bilgici et al. 2013)

2.1.3 Midrange radiation

Midrange radiation is nonionizing and includes low-frequency (LF), medium-frequency (MF), high-frequency (HF), very high-frequency (VHF), ultrahigh-frequency (UHF), super high-frequency (SHF), and extremely high-frequency (EHF) waves (See Table 2). The radiation in the intermediate zone between 300 Hz and 10 MHz is produced by a variety of manmade sources including longwave and mediumwave radio systems, TV broadcasting, and anti-theft devices. The sources of these electromagnetic fields include AM and FM radio waves, television signals, long-range navigation, commercial radar systems, cellular telephones, microwave ovens and alarm systems.

Frequencies in these ranges have two effects: induction (induced currents) seen in radio-frequency (RF) waves and thermal effects which are seen in microwave bands. Induction is

TABLE 1. Extremely low-frequency field (ELF) and very low-frequency (VLF) uses and potential health impacts.

EMFR Region	Frequency Range	Source	Potential health impacts
Extremely Low Frequency (ELF)	1–100 Hz	Schumann Resonance (8, 14.1, 20.3, 26.4, 32.5 Hz),	Stimulus to cells, and tissues
	50–60 Hz	Natural phenomena, Electricity usage and appliances, Transmission lines, Lighting	Alteration in DNA, RNA and proteins<br>Changes in hormones
Very Low Frequency (VLF)	100–10,000	Induction heating devices Electronic surveillance systems	None identified
	3kHz–30kHz	Visual display units	None idetntided

Downloaded from http://meridian.allenpress.com/jgb/article-pdf/16/2/55/2845768/i1943-4618-16-2-55.pdf by Texas A & M University user on 28 October 2021

used to generate heat in induction cooking, a relatively new cooking method that is more commonly used in household cooking devices due to its energy efficiency and safety features such as its non-burning capability. The RF range is used by wireless communications receiving and transmitting systems. A significant number of studies have been carried out that focused on the impacts of this range, about 2,421 papers on health, biology, epidemiology, reviews and dosimetry topics. The RF frequencies extend from 10 MHz to 300 GHz, with the wireless communication frequency range being around 1 GHz. Interactions with biological tissue consist mostly of absorption and subsequent heating in the case of high-power ranges, such as microwaves. In the case of wireless communication, the waves produce a lower power level (Bernard and Veyret 2013). In the FM and television range, powerful emitters are used (up to 25 kW per analog and 10 kW per digital TV channel). The antennas have a height of 40–350 meters and cover an area with a radius of up to 30 kilometers. Currently, there are many RF wireless communication devices, such as late generation mobile phones, DECT, Wi-Fi, Bluetooth, ULB, RFID, and remote controls, to name but a few. Most wireless technologies are based on two-way communication between the base and emitters-receivers; each mobile phone is a radio emitter-receiver operating within 800–2100 MHz. For instance, the maximum power emitted by a GSM 900 is 2 W; 50% of the power will be absorbed by the head of the user if the source is held close to the ear. This effect depends on the water content and the dielectric property of the body. The greater the water content, the more absorption takes place (blood fluid). If the body can dissipate the heat through thermoregulation, and blood flow, there is no temperature rise in the body (Veyret and Perrin 2012).

2.1.4 Nonionizing optical radiation

The optical radiation frequency range includes infrared (IR), visible light (VIS), and ultraviolet (UV) spectrums. It sources are lasers, incandescent lights, medical holography, medical material processing, optical radar, optical fiber communication, surgical applications, dermatological applications, and photocoagulation (Tenforde 1996)

All bodies emit infrared radiation (IR) as a function of their physical temperature. This phenomenon is described by Planck's law of black-body radiation. To evaluate IR, irradiance E (W/m^2), and energy exposure H (J/m^2) are measured. The biological effects caused by IR are thermal in nature, and the depth of their influence is within millimeters in the skin. Normally, the skin plays a major role in thermal regulation by means of its pores. IR can disturb this process and cause an unwanted heat absorption followed by the skin's reaction of changing its pigmentation and blood flow (Courant 2012).

Laser is an acronym for *Light Amplification by Stimulated Emission of Radiation*. Since 1960, the year the laser was invented by Professor Maiman in California, diverse types of lasers have been used in many applications such as military, space, telecommunication, medicine, show business, bar code readers and domestic products. Laser beams are created by repeatedly exciting atoms via an energy-pumping device to generate a beam of coherent light. The type of laser radiation depends on the generated wavelengths and the specific functional applications. In biomedical engineering, lasers are used to create thermal, photochemical, photo-ablative or disruptive effects on living tissues for therapeutic or cell eliminating applications. The nature of the effects is dependent on several factors: wavelength, exposure time, delivered energy, and the type of affected tissue. The eye is one of the main targets of the visible optical radiation exposure followed by a less sensitive organ, the skin, which is linked to cutaneous damage through exposure (Courant 2012).

Downloaded from http://meridian.allenpress.com/jgb/article-pdf/16/2/55/2845768/i1943-4618-16-2-55.pdf by Texas A & M University user on 28 October 2021

TABLE 2. Frequency fields in the midrange of electromagnetic radiation.

Frequency Range	EMFR Region (Non-ionizing)	Abbreviation	Source	Effect
10^5 Hz	Low Frequency	LF	Radio, Base stations, Navigation Systems, RFID	Decreased enzyme activity<br>Altered cerebral blood flow
10^6 Hz	Medium Frequency	MF	AM Radio, Long-range Communication, Welding and Sealing Devices	Stimulation of excitable tissues (varies with intensity) >100 kHz
10^7 Hz	High Frequency	HF	FM Radio, RFID, GPS	Stimulation of excitable tissues (varies with intensity)
10^8 Hz	Very High Frequency	VHF	TV	
10^9 (300 MHz–3 GHz)	Ultra-High Frequency<br>Microwave (L, C, X, and Ku-band)	UHF	TV<br>Medical Diathermy<br>Microwave ovens<br>RFID	Molecular agitation
10^{10} (3 GHz–30 GHz)	Super High Frequency	SHF	Cell Phones, Radar, Satellite, Long and shortwave radio, RFID	Molecular agitation
10^{11} (30 GHz–300 GHz)	Extremely High Frequency	EHF	Radar, Satellite Communication	Some cancers, cataracts, reproductive malfunction

2.1.5 Ionizing high frequency range radiation

Ionizing radiation with high energy and very short wavelengths includes gamma-rays, x-rays and nuclear particles such as neutrons, alpha particles (helium nuclei), beta particles (electrons). Unlike waves and neutrons that can penetrate the body, the alpha and beta particles are unable to enter organic tissues. However, the risk that the particles impose is a result of inhalation or ingestion. Radioactive substances are the main sources of ionizing radiation and are measured in terms of their radioactive decay rate by using the unit, Becquerel (Bq), which equals one nuclear decay event per second. Natural sources include radon-222, uranium-238, and cosmic rays. Manmade sources are the by-products and leakages in facilities using nuclear materials, including uranium-235, X-rays, computerized tomography (CT) scans, radiotherapy (medical applications) and they are also seen in food sources (McColl et al. 2015).

Ionizing radiation has adverse and possibly carcinogenic effects on the biological body and, in high dosages, is fatal. The absorbed quantity or dosage of ionizing radiation received by a body is measured by the unit Gray (Gy) which equals 1 J/kg. In buildings, the most prominent

Downloaded from http://meridian.allenpress.com/jgb/article-pdf/16/2/55/2845768/i1943-4618-16-2-55.pdf by Texas A & M University user on 28 October 2021

source of ionizing radiation is radon, an inert natural gas that decays into radioactive isotopes. Radon can penetrate into living spaces via infiltration from the ground. Radon is the single greatest source (41.6%) of all radioactive exposures. It is present in rocks and soils and can penetrate through cracks and openings in buildings (McColl et al. 2015).

2.2 EMFR in Building Construction: Manipulation and Mitigation

In order to identify, register, and mitigate the environmental electromagnetic field, all primary and secondary EMFR sources and the radius of their influence, along with the specific intensity and frequency range, have to be identified. These sources vary depending on the location and the building types. For example, the type of field found in homes is very different from those found in hospitals or manufacturing settings.

Not all frequency ranges and their effects are adverse. For example, electromagnetic phenomena are increasingly being used in the health and medical industry. Therapy that uses magnets of medium intensity is called bio-magnetic therapy. This type of restorative therapy is claimed to be natural and safe because it equalizes the cell's pH levels, leading to a healthy cell environment. At a microscopic level, Luo (Luo 2009) suggests that it is possible to reverse the entropy production rate of transferring disease or harmful information from cancerous cells to healthy cells. The entropy flow or information flow can be introduced by a low intensity electromagnetic frequency or ultrasound irradiation. The frequency and intensity of the EMFR radiation have different effects on different parts of the body. Figure 1 indicates the relationship between magnetic field frequency and its potential health effects.

FIGURE 1. Biomagnetic phenomena for magnetic fields having different intensities and frequencies affecting different organs and cells in the body (Ueno and Iwasaka, 1996; Ueno et al. 2007).

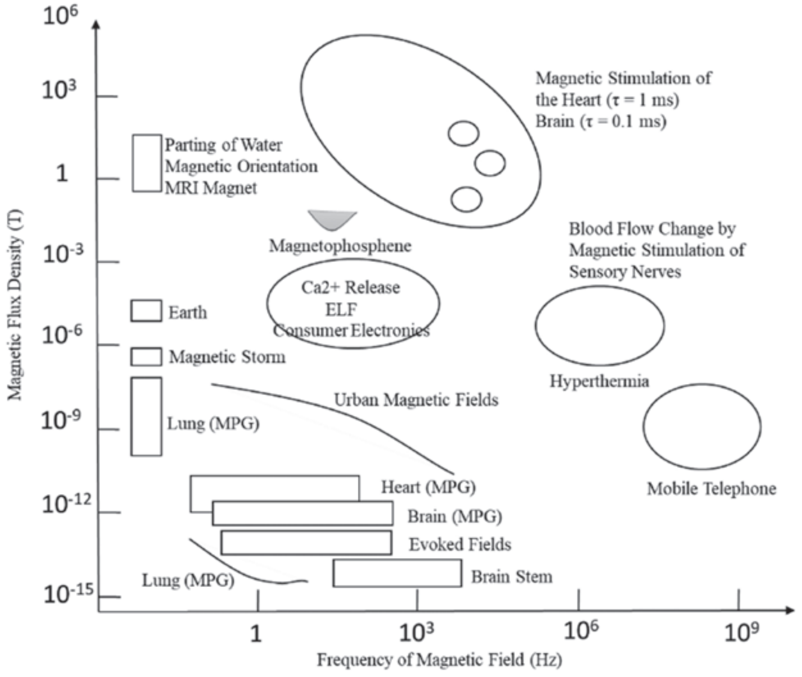

Downloaded from http://meridian.allenpress.com/jgb/article-pdf/16/2/55/2845768/i1943-4618-16-2-55.pdf by Texas A & M University user on 28 October 2021

When there is an EMFR change over a given time frame, there are two possible types of biophysical effect: a) An induced internal electric field (Ei) in the body, and b) energy absorption in the organic tissue which is quantified as the specific absorption rate (SAR). Based on research in the 1960s, many concerns have been raised about the health hazards caused by magnetic fields and the resulting electric charges in the body.

Different frequency bands of EMFR from different sources have different health impacts on human health. Table 3 shows the frequency bands of EMFR, their corresponding sources, and health risks they impose on human health.

2.2.1 Indoor sources

The sources of exposure may be primary or secondary. In the presence of a strong primary source, there may be secondary sources due to the induced electric energy found in the metal objects (Karpowicz et al. 2016). In a residential surrounding, the extremely low frequency-magnetic fields (ELF-MF) varies between less than 0.01 microtesla (μT) to above 0.3 μT. The hot spots may be due to interior ground currents or exterior power lines. Usually, the electric fields in residences are not influenced by the outdoor electrical lines. In offices, schools, and stores, ELF-MF emissions are greater because of shorter distances to the source and the increased time duration spent around devices such as printers. Other common devices include computer servers, metal detectors, theft detectors, and electronic article surveillance.

In the built environment there are many types of wiring. The EMFR produced by wires adds up vectorially and results in a total net exposure. When two insulated wires in a cable touch, the MF cancels. With separated electric lines, the cancellation is incomplete resulting in a MF > 0. The phase of the current or the timing of its peaks is variable and depends on the MF frequencies.

Building components, such as transformers in large apartment buildings and water pipe grounds, are the sources of MF in the building and have to be considered through EF, and MF assessment. Fluctuations in weather and occupancy power usage, such as daily and seasonal activity cycles, air conditioner use, and differences between weekdays, and weekends also need to be considered. This is also true for sources of "dirty electricity" such as high-frequency transients (HFT) caused by flipping a switch that creates an abrupt surge of electricity (Bowman 2014)

Appliances and household electrical equipment emit ELF-MF radiation which is either a by-product of the usage (e.g. light, heat, and electronics), or result of the electromagnetism in the operating mechanism (e.g. electric motors, cathode ray tubes (CRTs) used in previous generations of televisions and monitors). The relatively recent emergence of plasma and liquid crystal has produced displays that have lower emissions. The level of emissions in homes and workplaces depends on the amount of time spent using the equipment (Bowman 2014). It has been reported that one-third of a person's ELF-MF exposure is caused by appliances (Behrens et al. 2004).

2.2.2 Outdoor Sources

In an urban setting, electrical generators, nearby transmission lines (primary and secondary), substations, and distribution lines (overhead or underground) transport electrical currents into homes, schools, and offices. Determinants of ELF-MF exposures are the electric currents, degree of cancellation, distance from the line, and duration of the exposure. The intensity of ELF-EF exposure depends on the voltage, distortion, and/or shielding from the metal objects, the distance to the lines, and the duration of exposure (Bowman 2014; Sagar et al. 2018)

Downloaded from http://meridian.allenpress.com/jgb/article-pdf/16/2/55/2845768/i1943-4618-16-2-55.pdf by Texas A & M University user on 28 October 2021

TABLE 3. EMFR Frequency Bands, Applications, and Risks.

Frequency Band	Health Risk	Source/Application	Occupational Hazard
Nonionizing Static Magnetic Field (SMF)<br>0 Hz	No known health risk	Static field<br>Electric motors<br>Sewing machines<br>Repetitive tools like lathe, press and drill<br>Electric transportation	
Nonionizing Extremely Low Frequency (ELF)<br>3 to 3000 Hz	Childhood Leukemia (Ahlbom et al. 2000)<br>Cancer (WHO 2002)<br>Glioma (Turner et al. 2013)<br>Plasma membrane damage allowing Ca. influx (Stratton et al. 2013)<br>Breast cancer (Hardell and Sage 2008)<br>Alzheimer's Disease (Hardell and Sage 2008)<br>Motor Neuron Disease Parkinson Disease (Hardell and Sage 2008)<br>Tumors (Hardell and Sage 2008)<br>Biophysical effects: vertigo, nausea, impaired blood flow, nerve, muscle or cardiac excitation, localized or whole-body temperature rise (Zradziński 2015)	Direct currents (DC)<br>Schumann resonance<br>Electric utility:<br>(power stations, transmission lines, substations, distribution lines, destination)<br>Electric construction equipment<br>Electric transportation<br>Electronics (Semi-conductors)<br>(Petrochemical) Industry: Panels, Cables, Transformers, Monitors, Buses of Indoor Substation (Hosseini et al. 2015)	Electricians<br>Technicians<br>Sealers<br>Welders<br>Physiotherapists<br>Surgeons<br>Health care personnel
Nonionizing Intermediate Frequency (IF)<br>3 to 300 KHz<br>Very Low Frequency (VLF)	Reducing melatonin levels that protect against cancer cell growth<br>Reducing calcium levels in neurons causing stress<br>Enhancing free radical activity<br>DNA damage<br>Genotoxic effects (Hardell and Sage 2008)	Induction heating<br>Surveillance systems<br>Cooking, sterilization of medical and dental devices<br>Antennas in HF, VHF, UHF frequency range<br>Telecommunication<br>Visual display units (VDU)<br>Electronic anti-theft systems (EAS)	Dielectric welders<br>Physiotherapists<br>Radio communication and TV broadcasting technicians (Zradziński 2015) (Zradziński 2015)

Downloaded from http://meridian.allenpress.com/jgb/article-pdf/16/2/55/2845768/i1943-4618-16-2-55.pdf by Texas A & M University user on 28 October 2021

TABLE 3. (Continued)

Frequency Band	Health Risk	Source/Application	Occupational Hazard
Nonionizing Intermediate Frequency (IF)<br>300 KHz to10 MHz<br>Medium Frequency (MF)<br>High Frequency (HF)	Reproductive and neurodegenerative disorders (Frei and Röösli 2014)<br>Fatigue and concentration due to using screens<br>Chromosomal aberration in electricians and micronuclei (Celikler 2009)	Industrial heating<br>Radio frequency identification (RFID) device<br>Broadcasting AM (long-wave)<br>Short-wave diathermy<br>HF dryers<br>Electro heating<br>Timber industry<br>Automobile industry	Electricians<br>Panelists<br>Metal-processing<br>Electrical industry<br>Physiotherapists
Nonionizing Radio Frequency (RF)<br>10 MHz to 300GHz<br>Low Frequency (LF)<br>Medium Frequency (MF)<br>High Frequency (HF)<br>Very High Frequency (VHF)<br>Ultra-High Frequency (UHF)<br>Medium Wave (MW)<br>Super High Frequency (SHF)<br>Extremely High Frequency (EMFR)	Brain, central system, (Baan et al. 2011; Cardis et al. 2011)<br>Acoustic Neuroma, Glioma, Meningioma (Hardell and Sage 2008)<br>Effect on epileptic disorder (Hardell and Sage 2008)<br>Memory, learning, and brain activity (Hardell and Sage 2008; Ntzouni et al. 2013)<br>Genotoxic (Hardell and Sage 2008; Phillips, et al. 1998)<br>Dermatological symptoms stinging, burning and itching (Hagström et al. 2012)<br>Dermatological symptoms Erythema, Papules, Pustules (Gangi and Johansson 1997)<br>Sleeplessness, quality and duration (Liu et al. 2014)<br>Catecholamine levels affecting adrenaline, Noradrenaline and Dopamine (Singh and Kapoor 2015)	Cell & mobile phones<br>Antennas<br>Long-range communication<br>Welding and sealing devices<br>Diagnosis and treatment devices<br>FM, AM, Two-way & terrestrial system trunked radio, relays (Zradziński 2015)<br>Enhanced digital access communication system (EDAC)<br>Transmitters generating RF<br>Wireless network<br>Broadcasting & TV<br>Medical Diathermy<br>Microwave ovens<br>Radars<br>(GSM) communication<br>High speed internet long term evolution (LTE)<br>Universal mobile telecomm. system (UMCS)	Dispatch communication systems<br>Radio communication<br>Public administration<br>Military, police, fire services (Zradziński 2015)<br>Communication technicians<br>Data transmission technicians<br>Medical application specialists<br>Gastronomy workers<br>Communication specialists<br>Transmission specialists

Downloaded from http://meridian.allenpress.com/jgb/article-pdf/16/2/55/2845768/i1943-4618-16-2-55.pdf by Texas A & M University user on 28 October 2021

TABLE 3. (Continued)

Frequency Band	Health Risk	Source/Application	Occupational Hazard
Nonionizing Optical/ Laser Frequency<br>Infrared (IR)<br>Visible<br>Ultraviolet (UV)<br>430 to 770 THz		Heat Lamps<br>Planets<br>Light received by Human Eyes<br>UV Lamps<br>Sun rays	
Ionizing Range<br>>770 THz<br>X-Rays (X)<br>Gamma Rays (Y)	Cancer (McColl et al. 2015)<br>Organ cell killing (McColl et al. 2015)<br>DNA damage (McColl et al. 2015) Childhood Leukemia (McColl et al. 2015)	Particles: Neutrons and electrons<br>Charged particles<br>Radon-222, Uranium-238,<br>Gamma rays, Cosmic rays<br>Uranium-235, X-rays<br>Computerized Tomography (CT) scans, Radiotherapy<br>Food sources<br>Radionuclides in rocks, soil, and buildings<br>Leakage in nuclear power plants, nuclear weapons testing	Airline pilots<br>Miners

2.2.3 RF-MF Near-Field and Far-Field Radiation

Near-field source of RF-MF is the region where the distance from a radiating antenna is less than the wavelength of the radiated EMFR. Far-field radiation is created at a distance greater than the radiation wavelength. In the near-field, physical conditions are complex; the waves may be absorbed, or transmitted, but in the far-field zone, the energy is conserved and diminishes with the inverse or inverse-square of the distance to the source. The exposure from the near-field is typically localized in parts of the body area measured by the specific absorption rate (SAR). It is a calculation of how much RF energy is absorbed by the body and how much heat it produces. For example, when a cell phone or cordless phone is pressed to the head, the radiation causes heat. SAR is expressed in watts per kilogram of tissue (W/Kg). The near-field exposure is short-term and localized, whereas the far-field exposures affect the whole-body. Unlike ELF-EMFR, for this range of radiation (near-field and far-field), the primary effect on the body is heat generation (Frei and Röösli, 2014).

Increasingly, the incidence and duration of calls using smartphones and other communication devices is constantly rising. Everyday phone usage extends far beyond calling and texting. Data transmission, organizational communication, and the type of used network are main factors to be considered in identifying the spectral range, and the duration of the effects. In a European study, it was reported that the UMTS network has an improved power control

Downloaded from http://meridian.allenpress.com/jgb/article-pdf/16/2/55/2845768/i1943-4618-16-2-55.pdf by Texas A & M University user on 28 October 2021

technology, with 100–500 times lower power than GSM. DECT cordless phones on the other hand, have no power regulation and emit 10 mW on a continual basis (Frei and Röösli 2014).

Network operation in homes and offices transfers data through a low-voltage distribution power line referred to as power line communication (PLC). The frequency band these transmission systems operate in is from 1 to 30 MHz and the result is complex EMC patterns and problems such as electromagnetic noise interfering with the radio frequencies (Korovkin et al. 2003). Some studies show that high background electromagnetic noises can cause the loss of ability to navigation and orientation in a urban context (Šenfeldr et al. 2020; Keary et al. 2009; Engels et al. 2014; Pakhomov et al. 2017; Koprivica et al. 2016).

GSM and UMTS antenna base stations are often installed on the outside of the buildings or the close vicinity of the buildings. Some of the locations where antennas are installed include the edge of a roof, a mast on the roof, a side wall, and the ground floor.

The measurements that Koprivica and colleagues (Mladen Koprivica 2015) performed in Serbia showed that the maximum recorded EF value exceeded the European guideline values. The maximum values due to the antennas installed on the masts on the roof were generally lower than for antennas installed on the sides of buildings (Koprivica et al. 2016). Antennas located on the sides of buildings occur more frequently in areas with high population density. These antennas emit radiation within the frequency range of 0.9 GHz to 2.1 GHz (Koprivica et al. 2016).

A secondary source of EMFR is high voltage alternative current transmission lines that have electrostatic coupling, resistive coupling, and inducing effects on the underground metallic pipelines. Accidents sometimes occur due to the close proximity of power lines to water pipes (Munteanu et al. 2014).

2.2.4 UV Radiation (UVR)

Ultraviolet radiation (UVR) sources are either solar or artificial. Most types of glass used in building windows absorb solar UVR. Ultraviolet A (UVA) radiation has a longer wavelength and is associated with skin aging. Ultraviolet B (UVB) radiation has a shorter wavelength and is associated with skin burning. Ultraviolet C (UVC) radiation is the most damaging type of UV radiation. However, it does not reach the Earth's surface because it is completely filtered out by the atmosphere.

The frequency range and the irradiance of terrestrial UVR depends on the angle and the elevation of the sun in the sky. The ozone layer in the stratosphere (~10 to 50 km above sea level) and pollutants such as ozone, NO_2 and SO_2 in the troposphere affect the UVR irradiance (Madronich 1993). Clouds affect the intensity or irradiance of the UV radiation. The cloud cover factor (C) and the cloudiness factor (F) (the amount of water in the cloud) are used to calculate the effect of clouds on irradiance. Interestingly, solar UV radiation exposure can increase during periods of high sun and light overcast because the light clouds scatter the UV radiation even further, causing more exposure relative to a clear sunny sky (H.Sliney 1995). This is referred to as the "broken-cloud effect" and it can increase ground level UVB radiation by up to 40%. Ground and water surface reflection factors also influence the UV radiation exposure (Dobbinson and Knight 2001).

Artificial UV radiation can be produced by arc discharge, lamps, and lasers. A few examples include germicidal lamps, general lighting fluorescent lamps, metal halide lamps, mercury lamps, xenon lamps, quartz halogen lamps, welding arcs, ultraviolet lasers, and light emitting diodes (LEDs). To eliminate the UV radiation emitted by the lamps, a plastic cover or diffuser

Downloaded from http://meridian.allenpress.com/jgb/article-pdf/16/2/55/2845768/i1943-4618-16-2-55.pdf by Texas A & M University user on 28 October 2021

can be used. Also, optical projection systems that use filters, mirrors, lenses, and optical fibers have the ability to change the concentration and the spectral distribution of these radiations (Dobbinson and Knight 2001).

2.2.5 Space Typologies

The intended space typology to be monitored is the living and working space. Residential spaces, schools and offices share the same characteristics and fall into this category because the occupants spend most of their time in homes and offices. Other typologies such as transportation areas, for example, high-speed trains, navigation systems, etc., industrial zones; e.g. welding machines, metal detectors, etc., hospitals; e.g. MRI, CT scan, surgery equipment, X-rays, etc., commercial facilities; e.g. RFID devices, have each their own unique circumstances that cause different kind of fields and intensities.

EFs in residences are low and not affected by outside power lines (Bowman 2014). The main determinants of residential ELF-MF exposures are the electric power system, including water pipe grounds as well as power lines and appliance use. Appliance sources of ELF-MFs are lighting, heating, electronics, electric motors, and the direct use of MFs in applications such as induction cooking ranges.

In schools and offices, sources of ELF-MF are generally similar to residential sources, but exposures are usually higher because of the time using electric equipment. Students, on average, have less total weight average (TWA) exposures to ELF-MFs than teachers, who have less than office workers. However, exposure distributions overlap among these three groups. Although metal detectors and EAS (theft detectors) have introduced elevated MFs into schools, offices, and stores, their emissions have a short range, so exposures are limited to people who are near them regularly (Bowman 2014).

2.3 Standard and Regulations

International and American health authorities and institutions publish guidelines and reference levels designed to limit the amount of EMFR exposure. Some of the well-known organizations that are active in creating and updating guidelines include the International Commission on Nonionizing Radiation Protection (ICNIRP), the International Commission on Radiological Protection (ICRP), the Association Advancing Occupational and Environmental Health, the World Health Organization (WHO), the Institute of Electrical and Electronics Engineers Standards Association (IEEE-SA), the International Agency for Research on Cancer (IARC), National Legislation on Occupational Safety and Health (Directive 2013/35/EU), the United States Access Board under Disability Act, the United Nations (UN), the Federal and Communications Commission (FCC), and the Institute for Robotic Process Automation (IRPA).

The understanding of the adverse effects of EMFR and the effects and triggers it has on various types of human cells are increasingly being studied, analyzed, and articulated. Based on the most recent discoveries, scientific and health control agencies set limits on EMFR exposure based on targeted public or occupational measures. Until 2010 and based on the collected scientific evidence, it was concluded that certain acute and chronic health problems are related to the availability and exposure to certain frequencies and intensities of EMFR radiation. For example, neurons are directly affected when a surface electric charge results in neurobehavioral responses ranging from perception to annoyance. Myelinated nerve fibers of the central nervous system (CNS) can be stimulated by transcranial (localized brain-cell excitation) magnetic stimulation

Downloaded from http://meridian.allenpress.com/jgb/article-pdf/16/2/55/2845768/i1943-4618-16-2-55.pdf by Texas A & M University user on 28 October 2021

(Phillips and O'Reilly 1998), as well as can be muscle cells, and cardiac muscle tissues (Phillips and O'Reilly 1998). Flickering light in the periphery of the visual field (Saunders and Jefferys 2002), cognitive process effect on memory (Saunders and Jefferys 2002), excited brain cells causing slower hand movement (Saunders and Jefferys 2002), and transient arousal or mild stress in animals (WHO 2007) are a few such effects. At the ionizing level, inhalation of the naturally available gas Radon is found to have a high health risk and has proven to be one of the major causes of lung cancer. The WHO and ICRP advise that the limits of Radon exposure should be within the 100–300 Becquerel per cubic meter (Bq/m^3) range. In fact, Radon is the cause of 1/10 of all lung cancers in Europe (McColl et al. 2015).

2.3.1 Guidelines in US

EMFR measurement standards in US have been developed by the International Electrotechnical Commission (IEC), The Institute of Electrical and Electronics Engineers (IEEE), European standardization in the area of electrical engineering (CENELEC), the International Telecommunications Union (ITU) and other standardization bodies. In 1960, the American Standards Association approved the initiation of the Radiation Hazards Standards project under the co-sponsorship of the Department of the Navy and the Institute of Electrical and Electronics Engineers. Prior to 1988, C95 standards were developed by the C95 accredited standards committee and submitted to the American National Standards Institute (ANSI) for approval and issuance as ANSI C95 standards. Between 1988 and 1990, the committee was converted to Standards Coordinating Committee 28 under sponsorship of the IEEE Standards Board, and in 2001, became also known as the International Committee on Electromagnetic Safety (ICES). In accordance with policies of the IEEE, C95 standards are issued and developed as IEEE standards, as well as being submitted to ANSI for recognition (IEEE and I.S.C.C. 28 2002).

The present scope of ICES is: "Development of standards for the safe use of electromagnetic energy in the range of 0 Hz–300 GHz relative to the potential hazards due to exposure of such energy to man, volatile materials, and explosive devices. The committee will coordinate with other committees whose scopes are contiguous with ICES" (IEEE and I.S.C.C. 28 2002).

This standard was developed by an ICES Subcommittee 3 (SC 3) formed in 1991 to address the frequency range from 0–3 kHz (SC 3). In the early years, the subcommittee discussed the science relating to both long term and short-term exposures and concluded that the effects of long-term (chronic) exposure were not convincingly established as were effects of short-term exposures (IEEE and I.S.C.C. 28 2002).

There are five subcategories for the different safety zones introduced by the IEEE International Committee on Electromagnetic Safety for military workplaces and military forces (IEEE 2003):

Zone 0: Unrestricted access, all persons allowed (see Figure 2)

Zone 1: Restricted access, informed persons allowed, unaware public/worker excluded (see Figure 3)

Zone 2: Restricted expert only (REO), restricted access, only experts trained on specific equipment allowed, heightened safety precaution, unaware public/worker excluded

Zone 3: Restricted no access or off-limits; requires signage and possibly barriers, all persons excluded.

Downloaded from http://meridian.allenpress.com/jgb/article-pdf/16/2/55/2845768/i1943-4618-16-2-55.pdf by Texas A & M University user on 28 October 2021

FIGURE 2. Graphic representation of the zone 0 ERLs.

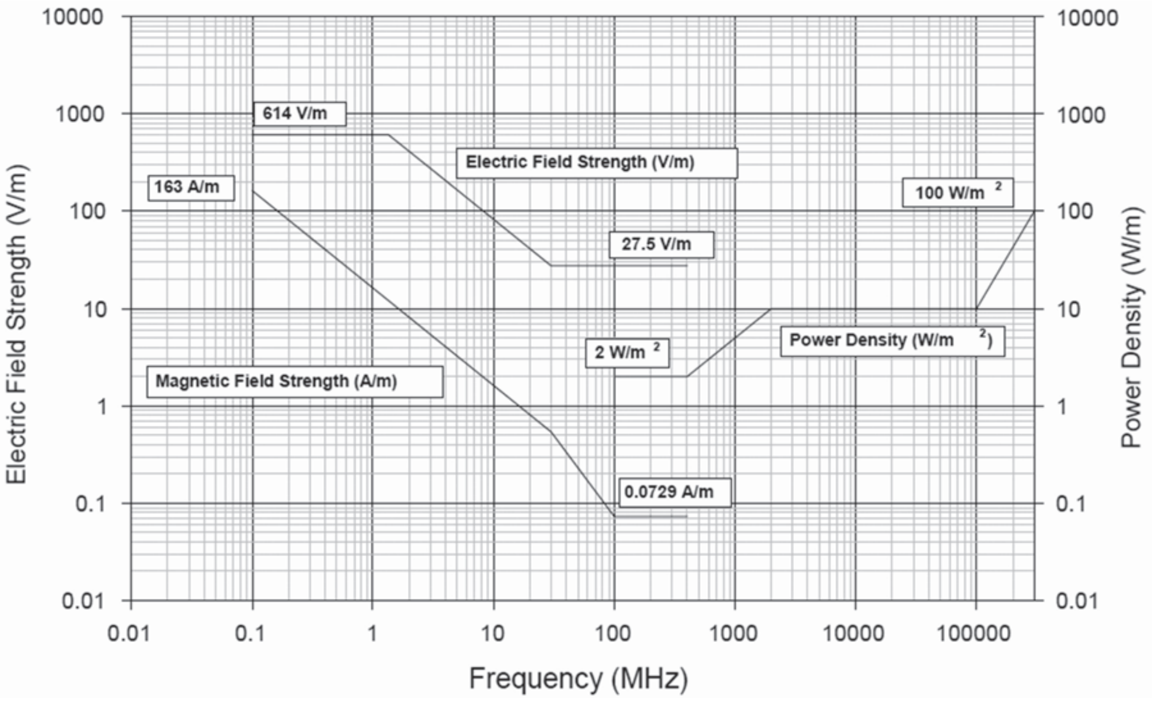

FIGURE 3. Graphic representation of the zone 1 ERLs.

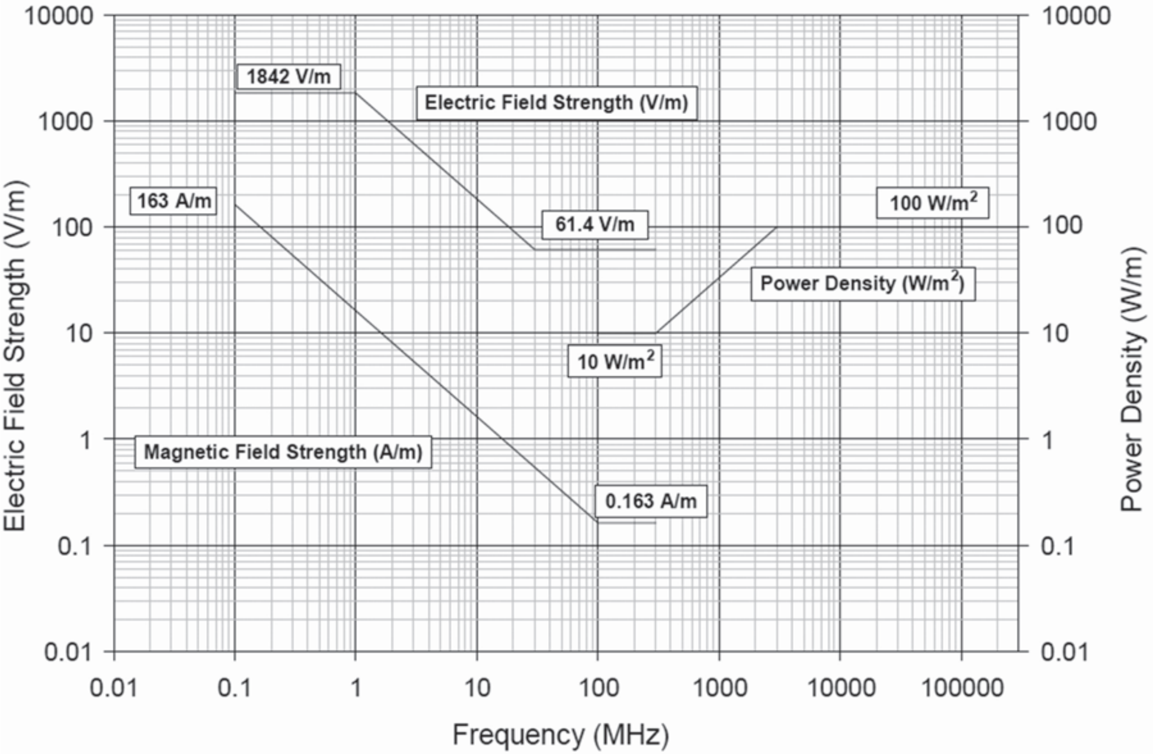

Downloaded from http://meridian.allenpress.com/jgb/article-pdf/16/2/55/2845768/i1943-4618-16-2-55.pdf by Texas A & M University user on 28 October 2021

2.3.2 Guidelines in Europe

The International Commission on Non-Ionizing Radiation Protection (ICNIRP) is a non-profit private organization that was chartered in Germany in 1992. It was the leader in creating a public platform that evaluates scientific knowledge to permit the development of a set of guidelines to protect the public and the environment from the hazardous effects of the non-ionizing range of EMFR. ICNIRP aims and objectives are designed to (1) protect people and the environment against the adverse effects of non-ionizing radiation (NIR), (2) develop and disseminate science-based advice from experts from different countries and disciplines such as biology, epidemiology, medicine, physics, and chemistry on limiting exposure to NIR, (3) provide exposure guidelines publicly and freely available online, and (4) organize workshops informing the public on scientific knowledge (ICNIRP 2010).

The restrictions in ICNIRP guidelines are based on established evidence regarding acute effects to protect workers and general public from the adverse effect of ELF-EMF. Careful review of epidemiological and biological data concerning chronic conditions showed no evidence related to low frequency EMFR exposure. Biological effects of exposure to low frequency electromagnetic fields have been reviewed by the International Agency for Research on Cancer (IARC), ICNIRP, and the World Health Organization (WHO 2002; WHO 2007; ICNIRP 2010) and national expert groups. Those publications provided the scientific basis for the 2010 (current) guidelines. The basis for the guidelines is two-fold: Exposure to low-frequency electric fields may cause well-defined biological responses, ranging from perception to annoyance, through surface electric-charge effects. In addition, the only well-established effects in volunteers exposed to low frequency magnetic fields are the stimulation of central and peripheral nervous tissues and the induction in the retina of phosphenes, a perception of faint flickering light in the periphery of the visual field that is reversible (Cervetto et al. 2007). The retina is part of the CNS and is regarded as an appropriate, albeit conservative, model for induced electric field effects on CNS neuronal circuitry in general (ICNIRP 2010). Dosimetry: Historically, magnetic field models assumed that the body has a homogeneous and isotropic conductivity and applied simple circular conductive loop models to estimate induced currents in different organs and body regions. Electric fields induced by time varying electric and magnetic fields were computed by using simple homogeneous ellipsoid models. In recent years, more realistic calculations based on anatomically and electrically refined heterogeneous models (Xi et al. 1994; Dimbylow 2006; Dimbylow 2005; Bahr 2007) resulted in a much better knowledge of internal electric fields in the body from exposure to electric and magnetic fields (ICNIRP 2010).

For the purpose of this research, the suggested guidelines by ICNIRP on the LF is discussed. LF fields are mostly related to the electric power supplies, AC current and power lines. The exposure limits are called reference levels and refer to the "in the air" values that would influence the "in the body" magnetic effect. Figure 4 and 5 show some of the published basic restrictions of ICNIRP. Each graph has two lines, one for the general public (ICNIRP-GP) guidelines and one for occupational exposure.

Based on the European regulations, a residential or public building shall be assessed, and the areas of the affected frequency range be identified. The European Parliament suggests that all building owners should conduct an assessment procedure. It is especially important that employers guarantee a healthy and safe condition for their employees. There are certain rules that are set by Directive 2013/35/EU where it is required that competent persons and services identify EMFR and suggest ways to reduce it to meet the permitted reference levels. The provided data shall have all the traceable information such as the frequency, duration, type of exposure,

Downloaded from http://meridian.allenpress.com/jgb/article-pdf/16/2/55/2845768/i1943-4618-16-2-55.pdf by Texas A & M University user on 28 October 2021

FIGURES 4 AND 5: Graph showing occupational (solid line) and general public (dotted line) reference allowance of magnetic field (left) and electric field (right) exposure. Source: ICNIRP, 2020.

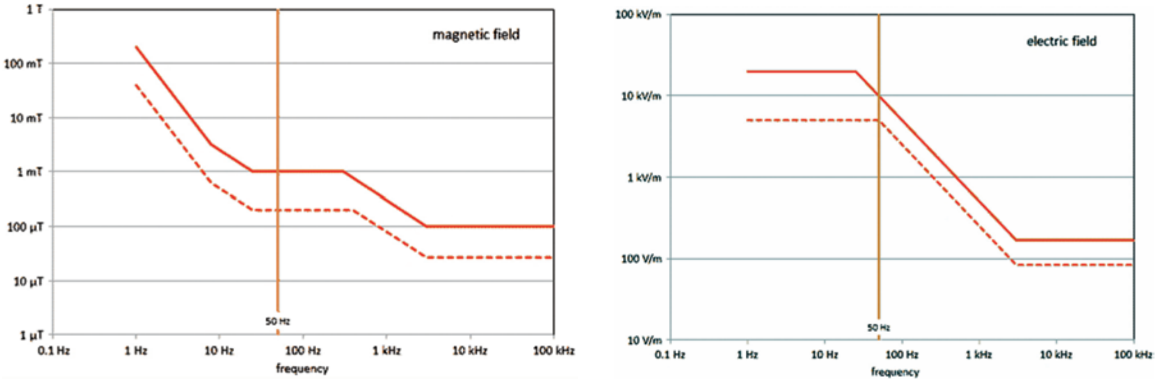

distribution over the worker's body, and over the volume of the work place, direct biophysical effects, particular risks, particular workers wearing active or passive implanted medical device (pacemakers, insulin pumps, pregnancy), indirect effects, equipment manufacturer's information, the effect of multiple sources of exposure or simultaneous exposure to multiple frequency fields, and any other specific information regarding that particular environment. Moreover, the risk assessment information must be updated on a regular basis. In Article 5 of this document, an action plan explains how to reduce the risk for an occupational space (see Table 4).

Electromagnetic (EM) radiation in Sweden is perceived as a hazardous exposure. Due to various health complaints and job losses, electromagnetic hypersensitivity (EHS) is recognized as a functional impairment, therefore the health system offers them assistance and service in accordance with the Swedish Act concerning Support and Service for Persons with Certain Functional Impairments, and the Swedish Social Services Act (prop. 1999/2000:79): It is considered that

TABLE 4. Directive 2013/35/EU, Article 5 suggests ways to assess an EM polluted space.

	**Provision aimed at avoiding or reducing risks:**
1	Identifying the source
2	Technical measuring of the EM radius
3	Identifying replacement strategies
4	Use of interlocks, shielding or similar protective solution
5	Warning markings or signals
6	Measuring spark discharges and contact currents
7	Limitation of duration and intensity of exposure
8	Availability of personal protection equipment
9	Appropriate equipment grounding, human to equipment bonding, clothing

Downloaded from http://meridian.allenpress.com/jgb/article-pdf/16/2/55/2845768/i1943-4618-16-2-55.pdf by Texas A & M University user on 28 October 2021

no human being is impaired, but instead there are shortcomings in the environment which cause the impairment (Hagström et al. 2012)

2.3.3 Difference between the ICNIRP and national (US) guidelines and standards

Some of the observed differences in the two ICNIRP and IEEE documentation and standards are explained in the table below (Table 5).

2.3.4 Measurement and assessment

Based on the European regulations, all residential and public buildings shall be assessed and the zones of the EMFR frequency range identified. The European Parliament suggests that all building owners conduct an assessment procedure. It is especially important that employers guarantee a healthy and safe condition for their employees. Directive 2013/35/EU established the requirements for identifying EMFR in buildings. This Directive requires that competent persons and services identify EMFR and suggest ways to reduce the EMG to meet the permitted reference levels. The resulting data must include key information such as the frequency, duration, type of exposure, distribution over the worker's body, and over the volume of the work place, direct biophysical effects, risks, workers who wear active or passive implanted medical device (pacemakers, insulin pumps, pregnancy), indirect effects, equipment manufacturer information, the effect of multiple sources of exposure or simultaneous exposure to multiple frequency fields, and any other specific information regarding that particular environment. Moreover, the risk assessment information has to be updated on a regular basis. Measurements are either done as spot or long-term (within a period of time) measurements. Safety is determined by the measurement of Ei and SAR in the affected body (e.g. arms, head) partial body, or whole body (WB) area. In order to assess the induced or SAR biophysical EM influences, virtual models or phantoms are used (see Figure 6). The model's design is based on realistic factors such as dimensions, posture, spatial resolution, electric contact with the ground, exposure intensity, dielectric properties (conductivity σ and permittivity ε) of the different masses such as muscle, brain, fat, bone, and heart.

TABLE 5. Difference between the European ICNIRP guides and the US IEEE Guides.

ICNIRP Guidelines on low-frequency field	IEEE (National Standards) Guidelines on low-frequency field
They do NOT address product performance standards, which are intended to limit EMF emissions from specific devices under specified test conditions.<br>They do NOT deal with the techniques used to measure any of the physical quantities that characterize electric, magnetic and electromagnetic fields.<br>Compliance with the present guidelines may not necessarily preclude interference with, or effects on, medical devices such as metallic prostheses, cardiac pacemakers and implanted defibrillators and cochlear implants.	Comprehensive descriptions of instrumentation and measurement techniques for accurately determining EMF physical quantities are found in IEC 2004, 2005a; IEEE 1994, 2008.<br>Standards produced by IEEE are more focused on the measurement methods.<br>Information and reference levels are not open to the general public or exceedingly difficult to navigate.

Downloaded from http://meridian.allenpress.com/jgb/article-pdf/16/2/55/2845768/i1943-4618-16-2-55.pdf by Texas A & M University user on 28 October 2021

FIGURE 6. Virtual phantoms: a) homogenous, cylindrical, b) heterogeneous, anatomical (Laura), c) homogenous in sitting position (CIOP-MAN), and d) semi-homogenous in standing position (CIOP-MAN) (Dobbinson and Knight 2001).

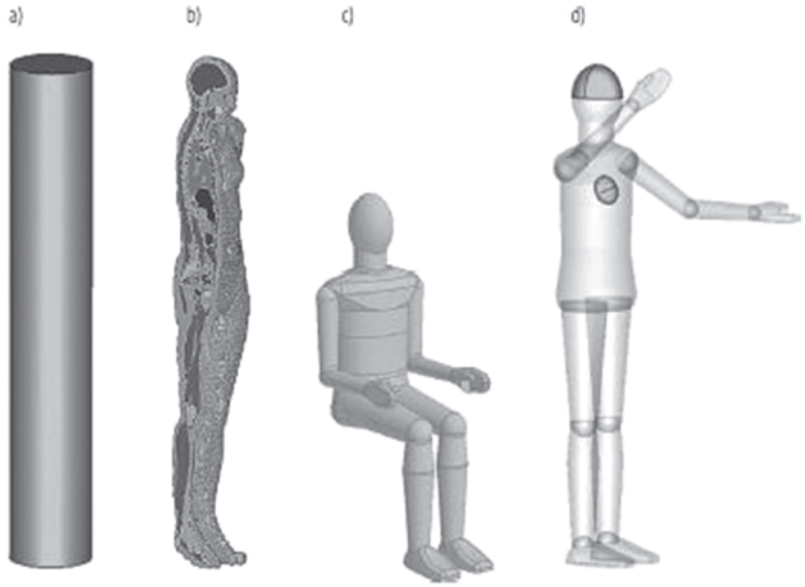

3. ANALYSIS

3.1 Regulation

Two major sources of published guidelines that are followed in Europe and the US are the IEEE and ICNIRP. Table 6 shows a comparison of the ELF-EMF values by both agencies each grouped in the two categories of stringent and less stringent values.

For the purpose of the experimentation, ICNIRP-GP values are used to compare the spot measurement values and their hazardous effect. As mentioned earlier, some of the individual countries have their own limiting levels that are even more stringent than the ICNRIP-GP values. For example, in Russia the field intensity of the power frequency range is (10 μT) and in Switzerland (1 μT) versus ICNIRP-GP (200 μT) (See Table 7).

3.2 Source of exposure

The most common sources of exposure within the ELF-MF zone are near appliances such as electric stove, TV set, Hi-fi systems, hair dryers, electric blankets, dimmers, wall outlets, fuse boxes, computer screens, and similar electrical devices. Table 8 shows the magnetic field values of some of the kitchen and household appliances. As shown, at a very close distance (0–15 cm) to certain equipment (vacuum cleaner, electric saw, drill, electric shaver, hairdryer, hand-held mixer) are above the ICNIRP-GP reference values.

3.3 Biological area of concern

By comparing the IEEE and ICNIRP reference limit lines, it is obvious that ICNIRP designed for the general public zone is the only regulatory limiting reference that addresses the avoidance of the magnetic field related to the power frequency and the intensities in question. The

Downloaded from http://meridian.allenpress.com/jgb/article-pdf/16/2/55/2845768/i1943-4618-16-2-55.pdf by Texas A & M University user on 28 October 2021

TABLE 6. A numerical comparison of the different reference limits values in Europe and US.

Frequency (Hz)	ICNIRP general public (μT)	ICNIRP occupational (μT)	IEEE-zone 0 unrestricted (μT)	IEEE-zone 1 restricted (μT)
0	400000	2000000		
<0.153			118000	353000
1	40000	200000	18100	54300
8	625	3125	2262.5	6787.5
20	250	1250	904	2715
25	200	1000	904	2710
50	200	1000	904	2710
167	200	1000	904	2710
300	200	1000	904	2710
400	200	750	904	2710
751	106.52	399.46	904	2710

TABLE 7. Reference limits of general public and occupational levels in different countries.

	General Public	Occupational
IEEE	904	2710
ICNIRP	200	1000
Poland	48	160
Russia	10	100
Switzerland	1	

ELF-EMF frequencies found in the normal living and working situations are 50 Hz in Europe and parts of the world, and 60 Hz in US. The intensity of the magnetic fields that are generated by domestic conduits and wiring vary between 0.01 and 1 millitesla (mT) (Reale and Amerio, 2013). Appliances generate fields of 0.1–100 mT (Renew and Swanson 1994; Reale and Amerio 2013). This data, although several decades old, is still valid since most buildings are still using older versions of wiring and appliances that were designed and used when the buildings were built. Table 9 shows some of the published research that has been done using the European frequency (50 Hz) and estimated field intensities that are present in a typical public environment.

The WHO fact sheet (2017) mentions that common daily exposure to EMF ranges from 0.07 μT, for an average European residential power frequency magnetic field, to about 20 μT

Downloaded from http://meridian.allenpress.com/jgb/article-pdf/16/2/55/2845768/i1943-4618-16-2-55.pdf by Texas A & M University user on 28 October 2021

TABLE 8. Magnetic field values of kitchen and household appliances and their relationship to ICNIRP-GP at 50 60 Hz (www.statesassembly.gov.je).

	Distance away from source in centimeters		
**Distance**	**3**	**30**	**100**
Electric cooker top	50	8	0.04
Microwave oven	200	8	0.6
Refrigerator	2	0.3	0.04
Coffee machine	10	0.2	0.02
Hand-held mixer	700	10	0.25
Toaster	20	1	0.02
ICNIRP GP	200	200	200
Hairdryer	2000	7	0.3
Electric shaver	1500	9	0.3
Drill	800	3.5	0.2
Electric saw	1000	25	1
Vacuum cleaner	800	20	2
Washing machine	50	3	0.15
Clothes dryer	8	2	0.1
Clothes iron	30	0.3	0.03

under power lines. All in all, the correct frequency and waveform are important, but they are not the only factors for the effects. The intensity of the exposure, the age of the recipient, and diet affect the immune system and, consequently, biological susceptibility (Reale and Amerio 2013). Some of the examples of possible positive biological effects, either harmful or therapeutic, are listed below:

- Effect (1): (1-a; 2 mT or 2,000 µT), (1-b; 10 mT or 10,000 µT)
  In vitro effects of low-level, ELF on DNA damage in human leucocytes by comet assay, exposure of 50/60 Hz, resulted in magnetic field millitesla (mT) range, blood samples were exposed to 5 doses of EMF (2,3,5,7 and 10mT at 50 Hz) (Ahuja et al. 1999).
- Effect (2): (2-a; 0.2 mT or 200 µT), (2-b; 6.4 mT or 640 µT)
  Effects of ELF-EMF on DNA of testicular cells and sperm chromatin structure in mice exposure of 50 Hz, 0.2 mT or 6.4 mT, duration: 4 weeks, the percentage of sperms with abnormal chromatin structure, increased in the two exposed groups. (Hong 2005)
- Effect (3): (3; 1 mT or 1,000 µT)
  Cell type-specific genotoxic effects of intermittent ELF-EMF, exposure of 50 Hz, sinusoidal, resulting magnetic field 1 mT, duration for 1–24h, identified three responder (Ivancsits 2005)

Downloaded from http://meridian.allenpress.com/jgb/article-pdf/16/2/55/2845768/i1943-4618-16-2-55.pdf by Texas A & M University user on 28 October 2021

TABLE 9. A comparison of the regulatory values and reported effects caused by ELF-MF in μT.

(Hz)	Regulatory values and biological effect caused by ELF-MF	MF B (μT)
50	ICNIRP General Public (GP)	5
50	ICNIRP Occupational (OCC)	22818
50	IEEE—zone 0 (Unrestricted)	17394
50	IEEE—zone 1 (Restricted)	52182
50	Effect 1	2000
50	Effect 1	10000
50	Effect 2	200
50	Effect 2	640
50	Effect 3	1000
50	Effect 4	1000

- Effect (4): (4; 1 mT or 1,000 μT)
  Induction of DNA strand breaks by intermittent exposure to ELF_EMF in human diploid fibroblasts, exposure of 50Hz, sinusoidal, resulting magnetic field 1000 micro-T, duration for 24h, a genotoxic potential of intermittent EMF (Ivancsits 2002).

By looking at the above table (Table 7), the reference ICNIRP-GP guideline zoned for the general public is the best limiting factor to keep the mentioned effects caused by power frequency (50 Hz) magnetic field away from reaching to the building occupants.

Figure 7 shows some other activities that are based on the ELF-MF and very low-level magnetic field intensity levels. Obviously, ICNIRP-GP does not address the natural cellular interruptions that are present in the natural body's range of magnetic field activities. Some of the areas that may not be covered by the reference limits are MF effects that are involved in for example bone mineralization, cellular activities and brain activities.

4. CONCLUSIONS

Humans spend most of their life in buildings, and, as a result, building occupant health is an important social issue we are facing now. Awareness of the various ranges of EMFR and their effects on human health is becoming more established worldwide. Standards for limiting EMFR exposure are reaching a consensus in Europe and America. The motivation for this research was a concern about the health impacts of EMFR and, therefore, the establishment of permissible EMFR levels. The answers are not simple and require a frequency spectrum map that correlates frequency and intensity with potential effects on human cells and organs. To answer the question about what frequency ranges are more problematical than others, it must be understood that each range affects the body in different ways. The published guidelines addressing the limitations of EMFR exposure are constantly evolving based on contemporary science, and they are always attempting to catch up to address the results produced by research. When it comes to buildings, for example, the effects of, and exposure limits to, indoor environmental pollutants are a

Downloaded from http://meridian.allenpress.com/jgb/article-pdf/16/2/55/2845768/i1943-4618-16-2-55.pdf by Texas A & M University user on 28 October 2021

FIGURE 7. Relation between biological activity and ICNIRP general public regulatory reference guide.

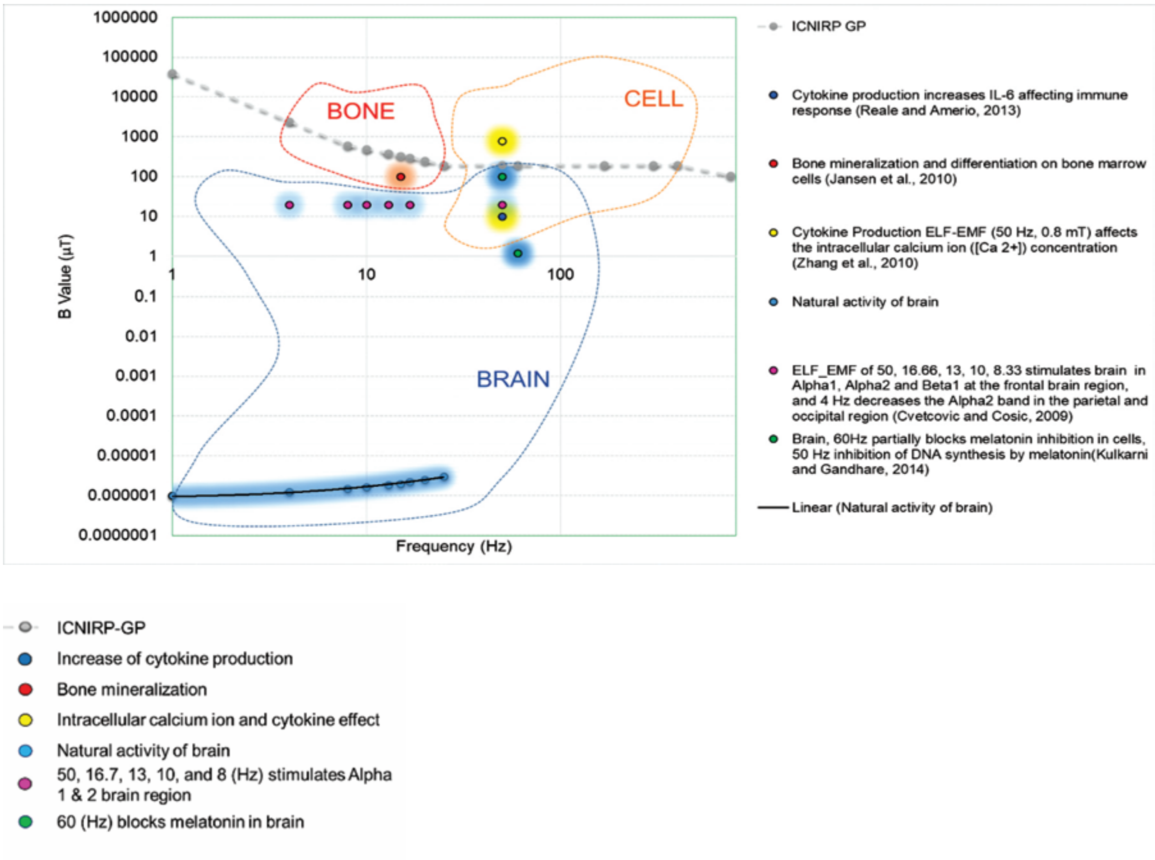

function of the information that is developed about airborne toxic chemicals, so that architects can avoid these pollutants in their designs. The first step in developing better knowledge about EMFR exposure is to detect and actively measure their levels in the built environment. This is possible if there is public awareness about the problem of EMFR exposure and the availability of specialists who can share and connect their data in such a way that the result is a valuable resource where patterns and observations lead to procedures and systems. The result would be a valuable data tool to draw conclusions and influence the makers of EMFR producing equipment and building power distribution systems. EMFR public awareness and education is the key to creating safe EMFR zones, to introducing the concept of EMFR safe zones into green building requirement and agendas. For example the Collaboration for High-Performance Schools (Šenfeldr et al. 2020) provided guidance for "Low-EMF Best Practice."

Another purpose of this research was to determine if there are measurement protocols that are workable, reliable, and accessible for use in building settings. An additional product was the comparison and consolidation of the most established published EMFR guidelines for the ELF range and the comparison of the results to the available health related scientific data. The ELF-EMFR range was selected because the biological and health effects are more developed for this range. The biological and health impact scope of this study is limited because it is based

Downloaded from http://meridian.allenpress.com/jgb/article-pdf/16/2/55/2845768/i1943-4618-16-2-55.pdf by Texas A & M University user on 28 October 2021

on existing data and risk factors that are limited in scope. As a follow-on to this paper, experiments conducted to test the published protocols by detecting and recording the frequency and intensity values of the ELF-EMFR radiation. The test room was an office that was equipped with typical lighting, electronic devices, switches, outlets, and wiring. The results of this research will be published in a forthcoming paper.

One of the most important challenges in buildings is that electric wiring and EMFR sources are often hidden inside walls, ceilings, and floors. Building electrical plans lack precise information about the direction, quantity and traffic of the cables and conduits. This can be easily resolved by using 3D building information modeling (BIM) software to plan, record changes, and simulate the fields. With this tool, it would be possible to properly plan and locate EMFR sources in lower human traffic areas. Another challenge is finding suitable instrumentation for measuring the EMFR. Finding a cost-effective EMFR measuring instrument that covers a wide variety of ranges and is both accurate and simple to use is required. Using the devices requires experienced specialists with the technical know-how on how to read and interpret the data.

Education is the key to awareness, detection, and mitigation. "Field management" is a new field of study that examines the quantitative aspects of the radiation and suggests solutions for mitigation. This field of study can be taught as an independent field of study in building technology schools to train on-site technicians, building designers and electrical engineers.

Building designers, specialists, and occupants need to be aware of the abundance and variety of electromagnetic fields in the indoor built environment. This research carefully studied and analyzed the frequency and intensity of EMFR and the radius of influence that exists around electronic devices, transmission lines, electrical infrastructure, and building electrical systems. The next step will be to use research based on actual conditions in various types of buildings to update and validate existing EMFR guidelines.

ACKNOWLEDGMENTS

This research was supported by the Powell Center for Construction & Environment at the University of Florida.

REFERENCES

Ahlbom, A. et al., "A pooled analysis of magnetic fields and childhood leukaemia," Br. J. Cancer, vol. 83, no. 5, pp. 692–698, 2000.

Ahuja, Y. R. Vijayashree, B. Saran, R. Jayashri, E. L. Manoranjani, J. K. and Bhargava, S. C. "In vitro effects of low-level, low-frequency electromagnetic fields on DNA damage in human leucocytes by comet assay," Indian J. Biochem. Biophys., vol. 36, no. 5, pp. 318–322, 1999.

Aurengo, A. and Perrin, A. "Some Physical Reminders Regarding Electromagnetic Radiation," in American Journal of Physics, 2012, pp. 1–9.

Baan, R. et al., "Carcinogenicity of radiofrequency electromagnetic fields.," Lancet Oncol., vol. 12, no. 7, pp. 624–626, 2011.

Bahr, A. Bolz, T. and Hennes, C. "Numerical dosimetry ELF: Accuracy of the method, variability of models and parameters, and the implication for quantifying guidelines," Health Phys., vol. 92, no. 6, pp. 521–530, 2007.

Behrens, T. Terschüren, C. Kaune, W. T. and Hoffmann, W. "Quantification of lifetime accumulated ELF-EMF exposure from household appliances in the context of a retrospective epidemiological case-control study," J. Expo. Anal. Environ. Epidemiol., vol. 14, no. 2, pp. 144–153, 2004.

Bilgici, B. Akar, A. Avci, B. and Tuncel, O. K. "Effect of 900 MHz radiofrequency radiation on oxidative stress in rat brain and serum," Electromagn. Biol. Med., vol. 32, no. 1, pp. 20–29, 2013.

Downloaded from http://meridian.allenpress.com/jgb/article-pdf/16/2/55/2845768/i1943-4618-16-2-55.pdf by Texas A & M University user on 28 October 2021

Bowman, J. D. “Exposures to elf-emf in the everyday environment,” in The Epidemiology of Electromagnetic Fields, 2014, pp. 93–124.

Buldak, R. J. et al., “Short-term exposure to 50Hz x`-EMF alters the cisplatin-induced oxidative response in AT478 murine squamous cell carcinoma cells,” Bioelectromagnetics, vol. 33, no. 8, pp. 641–651, 2012.

Cardis, E. et al., “Risk of brain tumours in relation to estimated RF dose from mobile phones: Results from five interphone countries,” Occup. Environ. Med., vol. 68, no. 9, pp. 631–640, 2011.

Celikler, S. Aydemir, N. Vatan, O. Kurtuldu, S. and Bilaloglu, R. “A biomonitoring study of genotoxic risk to workers of transformers and distribution line stations.,” Int. J. Environ. Health Res., vol. 19, no. 6, pp. 421–430, 2009.

L Cervetto G C Demontis, and C Gargini. “Cellular mechanisms underlying the pharmacological induction of phosphenes.” Journal of Pharmacoogy, vol. 150, no. 4, pp. 383–390, 2007

Chiaramello, E. et al., “Radio frequency electromagnetic fields exposure assessment in indoor environments: A review,” Int. J. Environ. Res. Public Health, vol. 16, no. 6, 2019.

Courant, D. “Lasers,” Electromagn. Fields, Environ. Heal., pp. 127–141, 2012.

Dimbylow, P. “Development of pregnant female, hybrid voxel-mathematical models and their application to the dosimetry of applied magnetic and electric fields at 50 Hz,” Phys. Med. Biol., vol. 51, no. 10, pp. 2383–2394, 2006.

Dimbylow, P. “Development of the female voxel phantom, NAOMI, and its application to calculations of induced current densities and electric fields from applied low frequency magnetic and electric fields,” Phys. Med. Biol., vol. 50, no. 6, pp. 1047–1070, 2005.

Dobbinson, S. and Knight, K. “Protecting workers from ultraviolet radiation in sunlight,” Journal of Occupational Health and Safety-Australia and New Zealand, vol. 17(6), pp. 587–589, 2001.

Doyon, P. R. and Johansson, O. “Electromagnetic fields may act via calcineurin inhibition to suppress immunity, thereby increasing risk for opportunistic infection: Conceivable mechanisms of action,” Med. Hypotheses, vol. 106, pp. 71–87, 2017.

Engels, S. et al., “Anthropogenic electromagnetic noise disrupts magnetic compass orientation in a migratory bird,” Nature, vol. 509, no. 7500, pp. 353–356, 2014.

Frei, P. and Röösli, M. “Exposure to radiofrequency electromagnetic fields in our everyday environment,” Epidemiol. Electromagn. Fields, pp. 125–140, 2014.

Gangi, S. and Johansson, O. “Skin changes in ‘screen dermatitis’ versus classical UV- and ionizing irradiation-related damage—Similarities and differences. Two neuroscientists’ speculative review,” Exp. Dermatol., vol. 6, no. 6, pp. 283–291, 1997.

Hagström, M. Auranen, J. Johansson, O. and Ekman, R. “Reducing electromagnetic irradiation and fields alleviates experienced health hazards of VDU work,” Pathophysiology, vol. 19, no. 2, pp. 81–87, 2012.

Hardell, L. and Sage, C. “Biological effects from electromagnetic field exposure and public exposure standards,” Biomed. Pharmacother., vol. 62, no. 2, pp. 104–109, 2008.

Hong, R. Zhang, Y. Liu, Y. and Weng, E.Q. “Effects of extremely low frequency electromagnetic fields on DNA of testicular cells and sperm chromatin structure in mice,” Zhonghua Lao Dong Wei Sheng Zhi Ye Bing Za Zhi, vol. 23, no. 6, pp. 414–417, 2005.

Hosseini, M. Monazzam, M. R. Matin, L. F. and Khosroabadi, H. “Hazard zoning around electric substations of petrochemical industries by stimulation of extremely low-frequency magnetic fields,” Environ. Monit. Assess., vol. 187, no. 5, 2015.

He, G. L. et al., “The amelioration of phagocytic ability in microglial cells by curcumin through the inhibition of EMF-induced pro-inflammatory responses,” J. Neuroinflammation, vol. 11, 2014.

ICNIRP, “Low Frequency Fields—Fact Sheet 2010,” ICNIRP, 2010. [Online]. Available: https://www.icnirp.org/en/applications/power-lines/index.html.

ICNIRP, “ICNIRP Guidlines For Limiting Exposure To Electromagnetic Fields (100 KHZ TO 300 GHZ)” ICNIRP, 2020. [Online]. Available: https://www.icnirp.org/cms/upload/publications/ICNIRPrfgdl2020.pdf

IEEE and I. S. C. C. 28, “IEEE Standard for Safety Levels With Respect to Human Exposure to Electromagnetic Fields, 0–3 kHz,” IEEE Std C95.6-2002, 2002.

I. International Committee on Electromagnetic Safety SCC and IEEE Technical Committee, IEEE Std C95.1-2345-2014 IEEE Standard for Military Workplaces-Force Health Protection Regarding Personnel Exposure to Electric, Magnetic, and Electromagnetic Fields, 0 Hz to 300 GHz. 2003.

Downloaded from http://meridian.allenpress.com/jgb/article-pdf/16/2/55/2845768/i1943-4618-16-2-55.pdf by Texas A & M University user on 28 October 2021

Ivancsits, S. Pilger, A. Diem, E. Jahn, O. and Rüdiger, H. W. "Cell type-specific genotoxic effects of intermittent extremely low-frequency electromagnetic fields," Mutat. Res.—Genet. Toxicol. Environ. Mutagen., vol. 583, no. 2, pp. 184–188, 2005.

Ivancsits, S. Diem, E. Pilger, A. Rüdiger, H. W. and Jahn, O. "Induction of DNA strand breaks by intermittent exposure to extremely-low-frequency electromagnetic fields in human diploid fibroblasts," Mutat. Res.—Genet. Toxicol. Environ. Mutagen., vol. 519, no. 1–2, pp. 1–13, 2002.

Karpowicz, J. Bienkowski, P. and Kieliszek, J. "Model of the minimum requirements regarding electric and magnetic field strength measurement devices for use in the near-field occupational exposure in compliance testing with respect to the requirements of European Directive 2013/35/EU," IEEE Int. Symp. Electromagn. Compat., vol. 2016-Novem, pp. 668–671, 2016.

Keary, N. et al., "Oscillating magnetic field disrupts magnetic orientation in Zebra finches, Taeniopygia guttata," Front. Zool., vol. 6, no. 1, 2009.

Khadra, M. Abu K. Khalil, A. M. Samak, M. Abu and Aljaberi, A. "Evaluation of selected biochemical parameters in the saliva of young males using mobile phones," Electromagn. Biol. Med., vol. 34, no. 1, pp. 72–76, 2015.

Kocaman, A. Altun, G. Kaplan, A. A. Deniz, Ö. G. Yurt, K. K. and Kaplan, S. "Genotoxic and carcinogenic effects of non-ionizing electromagnetic fields," Environ. Res., vol. 163, pp. 71–79, 2018.

Koprivica, M. Petrić, M. Nešković, N. and Nešković, A. "Statistical analysis of electromagnetic radiation measurements in the vicinity of indoor microcell GSM/UMTS base stations in Serbia," Bioelectromagnetics, vol. 37, no. 1, pp. 69–76, 2016.

Korovkin, N. Marthe, E. Rachidi, F. and Selina, E. "Mitigation of electromagnetic field radiated by PLC systems in indoor environment," Int. J. Commun. Syst., vol. 16, no. 5, pp. 417–426, 2003.

Lambrozo, J. and Souques, M. "Electricity and Extremely Low Frequency Electric and Magnetic Fields," in Electromagnetic Fields, Environment and Health, A. Perrin and M. Souques, Eds. Paris: Springer Paris, 2012, pp. 35–50.

Liu, H. et al., "Occupational electromagnetic field exposures associated with sleep quality: A cross-sectional study," PLoS One, vol. 9, no. 10, 2014.

Luo, L. F. "Entropy production in a cell and reversal of entropy flow as an anticancer therapy," Front. Phys. China, vol. 4, no. 1, pp. 122–136, 2009.

Martiny, K. Lunde, M. and Bech, P. "Transcranial low voltage pulsed electromagnetic fields in patients with treatment-resistant depression," Biol. Psychiatry, 2010.

McColl, N. et al., "European Code against Cancer 4th Edition: Ionising and non-ionising radiation and cancer," Cancer Epidemiol., 2015.

Merzenich, H. et al., "Childhood Leukemia in Relation to Radio Frequency Electromagnetic Fields in the Vicinity of TV and Radio Broadcast Transmitters," Am. J. Epidemiol., vol. 168, pp. 1169–1178, 2008.

Munteanu, C. Purcar, M. Bursasiu, D. Merdan, E. and Farcas, V. "CAD/CAE modeling of the human exposure to electric field inside a high voltage substation," EPE 2014—Proc. 2014 Int. Conf. Expo. Electr. Power Eng., pp. 476–479, 2014.

Ntzouni, M. P. Skouroliakou, A. Kostomitsopoulos, N. and Margaritis, L. H. "Transient and cumulative memory impairments induced by GSM 1.8 GHz cell phone signal in a mouse model," Electromagn. Biol. Med., vol. 32, no. 1, pp. 95–120, 2013.

Pakhomov, A. et al., "Very weak oscillating magnetic field disrupts the magnetic compass of songbird migrants," J. R. Soc. Interface, vol. 14, no. 133, 2017.

Phillips, J. L. Ivaschuk, O. Ishida-Jones, T. Jones, R. A. Campbell-Beachler, M. and Haggren, W. "DNA damage in molt-4 T-lymphoblastoid cells exposed to cellular telephone radiofrequency fields in vitro," Bioelectrochemistry Bioenerg., vol. 45, no. 1, pp. 103–110, 1998.

Phillips, K. and O'Reilly, C. "Demography and Diversity in Organizations: A Review of 40 Years of Research," Res. Organ. Behav., vol. 20, pp. 77–140, 1998.

Pogosyan,A. Gaynor, L.D. Eusebio, A. and Brown, P. "Boosting Cortical Activity at Beta-Band Frequencies Slows Movement in Humans," Curr. Biol., vol. 19, no. 19, pp. 1637–1641, 2009.

Reale, M. and Amerio, P. "Extremely low frequency electromagnetic field and cytokines production," in Electromagnetic Fields: Principles, Engineering Applications and Biophysical Effects, 2013, pp. 239–253.

Renew, D. C. and Swanson, J. "Power frequency fields and people," Eng. Sci. Educ. J., vol. 3, no. 2, pp. 71–79, 1994.

Downloaded from http://meridian.allenpress.com/jgb/article-pdf/16/2/55/2845768/i1943-4618-16-2-55.pdf by Texas A & M University user on 28 October 2021

Schmiedel, S. and Blettner, M. "The association between extremely low-frequency electromagnetic fields and childhood leukaemia in epidemiology: Enough is enough," Br. J. Cancer, vol. 103, no. 7, pp. 931–932, 2010.
Sagar, S. et al., "Comparison of radiofrequency electromagnetic field exposure levels in different everyday microenvironments in an international context," Environ. Int., vol. 114, pp. 297–306, 2018.
Salehi, I. Sani, K. G. and Zamani, A. "Exposure of rats to extremely low-frequency electromagnetic fields (ELF-EMF) alters cytokines production," Electromagn. Biol. Med., vol. 32, no. 1, pp. 1–8, 2013.
Saliev, T. Begimbetova, D. Masoud, A. R. and Matkarimov, B. "Biological effects of non-ionizing electromagnetic fields: Two sides of a coin," Prog. Biophys. Mol. Biol., vol. 141, pp. 25–36, 2019.
Saunders, R. D. and Jefferys, J. G. R. "Weak electric field interactions in the central nervous system," Health Phys., vol. 83, no. 3, pp. 366–375, 2002.
Šenfeldr, M. Maděra, P. Kotásková, P. Fialová, J. Kundrata, M. and Rieger, V. "The Green Roofs and Facades as a Tool of Climate Cooling in the Urban Environment," pp. 39–75, 2020.
Shoogo, U. and Shigemitsu, T. "Biological Effects of Static Magnetic Field," Biol. Eff. Magn. Electromagn. Fields, 2007.
Singh, S. and Kapoor, N. "Occupational EMF exposure from radar at X and Ku frequency band and plasma catecholamine levels," Bioelectromagnetics, vol. 36, no. 6, pp. 444–450, 2015.
Sliney, D. H. (1995). "UV radiation ocular exposure dosimetry." Journal of Photochemistry & Photobiology, B: Biology, 31, 69–77.
Stratton, D. Lange, S. and Inal, J. M. "Pulsed extremely low-frequency magnetic fields stimulate microvesicle release from human monocytic leukaemia cells," Biochem. Biophys. Res. Commun., vol. 430, no. 2, pp. 470–475, 2013.
Tenforde, T. S. "Biological interactions of extremely-low-frequency electric and magnetic fields," J. Electroanal. Chem., vol. 320, no. 1, pp. 1–17, 1991.
Tenforde, T. S. "Spectrum and intensity of environmental electromagnetic fields from natural and man-made sources," Adv. Chem. Ser., vol. 250, pp. 29–35, 1996.
Tomitsch, J. and Dechant, E. "Exposure to electromagnetic fields in households-Trends from 2006 to 2012," Bioelectromagnetics, vol. 36, no. 1, pp. 77–85, 2015.
Turner, M. C. et al., "Allergy and brain tumors in the INTERPHONE study: Pooled results from Australia, Canada, France, Israel, and New Zealand," Cancer Causes Control, vol. 24, no. 5, pp. 949–960, 2013.
Ueno, S. and Iwasaka, M. "Magnetic Nerve Stimulation and Effects of Magnetic Fields on Biological, Physical and Chemical Processes," Biol. Eff. Magn. Electromagn. Fields, pp. 1–21, 1996.
Veyret, B. and Perrin, A. "Wireless communications and radiofrequency fields," Electromagn. Fields, Environ. Heal., pp. 63–79, 2012.
WHO, "Exposure to extremely low frequency fields. International EMF Project Fact Sheet No. 322," 2007. [Online]. Available: https://www.who.int/peh-emf/publications/facts/fs322/en/.
WHO, "Some traditional herbal medicines, some mycotoxins, naphthalene and styrene.," IARC Monogr. Eval. Carcinog. Risks Hum., vol. 82, pp. 1–556, 2002.
Xi, W. Stuchly, M. A. and Gandhi, O. P. "Induced electric currents in models of man and rodents from 60 Hz magnetic fields," IEEE Trans. Biomed. Eng., vol. 41, no. 11, pp. 1018–1023, Nov. 1994.
Zradziński, P. "Examination of virtual phantoms with respect to their possible use in assessing compliance with the electromagnetic field exposure limits specified by Directive 2013/35/EU," Int. J. Occup. Med. Environ. Health, vol. 28, no. 5, pp. 781–792, 2015.

Downloaded from http://meridian.allenpress.com/jgb/article-pdf/16/2/55/2845768/i1943-4618-16-2-55.pdf by Texas A & M University user on 28 October 2021

Downloaded from http://meridian.allenpress.com/jgb/article-pdf/16/2/55/2845768/i1943-4618-16-2-55.pdf by Texas A & M University user on 28 October 2021
