# Supplementary material for: Magnetic Fields and Cancer: Epidemiology, Cellular Biology, and Theranostics
Source: Int J Mol Sci. 2022 Jan 25;23(3):1339. doi: 10.3390/ijms23031339 (PMC8835851; doi:10.3390/ijms23031339)
Supplement: Supplementary file 1 [file ijms-23-01339-s001.zip › Supplementary Data Set S1/MF and Cancer.Data/PDF/3001103096/50ACDF3EDD8F44D69DCA51CA77188086.pdf]

İSTANBUL ÜNİVERSİTESİ YAYINEVİ

RESEARCH / ARAŞTIRMA
DOI: 10.26650/IUITFD.2020.0041
İst Tıp Fak Derg 2020 / J Ist Faculty Med 2020

EFFECTS OF EXTREMELY LOW-FREQUENCY MAGNETIC FIELD ON HEALTHY FIBROBLASTS AND BREAST CANCER CELLS

ÇOK DÜŞÜK FREKANSLI MANYETİK ALANIN SAĞLIKLI FİBROBLAST VE MEME KANSERİ HÜCRELERİNE ETKİSİ

Handan KAYHAN[1], Babek ERDEBİLLİ[2], Sevim GÖNEN[3], Meriç Arda EŞMEKAYA[4], Ersen ERTEKİN[5], Ayşe Gülnihal CANSEVEN[4]

Gazi University, Faculty of Medicine, [1]Department of Adult Hematology, [3]Department of Nephrology, [4]Department of Biophysics, Ankara, Turkey
[2]Yıldırım Beyazıt University, Department of Industrial Engineering, Ankara, Turkey
[5]Adnan Menderes University, Faculty of Medicine, Department of Radiology, Aydın, Turkey

ORCID IDs of the authors: H.K. 0000-0003-4967-2740; B.E. 0000-0001-8860-3903; S.G. 0000-0002-2059-6193; M.A.E. 0000-0003-0469-4954; E.E. 0000-0001-7182-0725; A.G.C. 0000-0003-0716-7981

Cite this article as: Kayhan H, Erdebilli B, Gonen S, Esmekaya MA, Ertekin E, Canseven AG. Effects of extremely low-frequency magnetic field on healthy fibroblasts and breast cancer cells. J Ist Faculty Med 2020;83(4):384-9. doi: 10.26650/IUITFD.2020.0041

ABSTRACT

Objective: Modern people are exposed to many environmental factors, including Extremely Low-Frequency Magnetic Fields (ELF-MF). ELF-MFs are emitted from everything that produces electricity or that it passes through. Some studies have shown no significant detrimental effect of ELF-MFs on biological systems, while other studies have shown that ELF-MFs increase the risk of the development of childhood leukaemia (1). Moreover, the 40 Hz-7mT range ELF-MF has been used as a magnetotherapy instrument (2). In this study, we aimed to compare the effect of ELF-MF on healthy dermal fibroblasts and breast cancer cells (MCF7).

Material and Method: After the administration of 1 mT-50 Hz ELF-MF to healthy and cancer cells for different exposure times, apoptosis/necrosis levels were investigated and cell cycles and proliferation indexes were examined. The Analytic Hierarchy Process (AHP) was applied to find the most effective dose.

Results: The ELF-MF did not show any significant effect on MCF7 cells. However, it caused increased apoptosis and decreased proliferation of healthy fibroblast cells. Furthermore, no changes in the cell cycle were observed in either cell line. As a result of AHP, the most effective exposure was determined to be 1 hour.

Conclusion: Our results showed that while the ELF-MF reduces the viability of healthy fibroblasts, it has not observed therapeutic effect on breast cancer cells.

Keywords: Extremely low-frequency magnetic field, ELF-MF, apoptosis, proliferation, MCF, healthy fibroblasts

ÖZET

Amaç: Modern insan, çok düşük frekanslı manyetik alan (ELF-MF) da dâhil olmak üzere birçok çevresel etkene maruz kalmaktadır. ELF-MF, içinden elektrik geçen veya üreten her şeyden yayılır. Bazı çalışmalar ELF-MF'nin biyolojik sistemler üzerinde önemli bir zararlı etkisi olmadığını göstermişken, diğer çalışmalar ELF-MF'nin çocukluk çağı lösemisi geliştirme riskini arttırdığını göstermiştir (1). Dahası, 40 Hz-7mT gücündeki ELF-MF bir magnetoterapi aracı olarak kullanılmaktadır (2). Bu çalışmada, ELF-MF'nin sağlıklı dermal fibroblastlar ve meme kanseri hücreleri (MCF7) üzerindeki etkisinin karşılaştırılması amaçlanmıştır.

Gereç ve Yöntem: Farklı maruziyet süreleri için sağlıklı ve tümör hücrelerine 1 mT-50 Hz ELF-MF uygulandıktan sonra apoptoz/nekroz seviyeleri araştırılmıştır; hücre döngüleri ve proliferasyon indeksleri incelenmiştir. En etkili dozu bulmak için Analitik Hiyerarşi Süreci (AHP) uygulanmıştır.

Bulgular: ELF-MF, MCF7 hücreleri üzerinde herhangi bir anlamlı bir etki göstermemiştir. Bununla birlikte, sağlıklı fibroblast hücrelerinde apoptozun artmasına ve proliferasyonunu düşmesine sebep olmuştur. Ayrıca, her iki hücre hattında da hücre döngüsünde herhangi bir değişiklik gözlemlenmemiştir. AHP sonucunda en etkili maruziyetin 1 saat olduğu belirlenmiştir.

Sonuç: Elde ettiğimiz sonuçlar, ELF-MF'nin sağlıklı fibroblastların canlılığını azaltırken meme kanseri hücreleri üzerinde herhangi bir terapötik etki yaratmadığını göstermiştir.

Anahtar Kelimeler: Çok düşük frekanslı manyetik alan, ELF-MF, apoptoz, proliferasyon, MCF, sağlıklı fibroblast

Corresponding author/İletişim kurulacak yazar: handankayhan@gazi.edu.tr

Submitted/Başvuru: 23.04.2019 • **Revision Requested/Revizyon Talebi:** 22.05.2020 •
Last Revision Received/Son Revizyon: 04.06.2020 • **Accepted/Kabul:** 17.06.2020 • **Published Online/Online Yayın:** 19.10.2020

©Telif Hakkı 2020 J Ist Faculty Med - Makale metnine jmed.istanbul.edu.tr web sayfasından ulaşılabilir.
©Copyright 2020 by J Ist Faculty Med - Available online at jmed.istanbul.edu.tr

INTRODUCTION

Extremely low frequency (ELF) fields are electromagnetic fields (EMF) with frequencies below 300 Hz. They are included in the lower part of the electromagnetic spectrum. Many studies have reported that ELF exposure causes significant changes in cell survival, cell cycle progression, DNA integrity, and proliferation (3-8). However, there are many conflicting studies regarding the consequences of ELF-MF in cells (5). Some studies have shown that magnetic therapy can be utilized as an invasive method used in the treatment of several diseases (9-17). In contrast, other epidemiological studies have concluded that continuous environmental magnetic field exposure increases the risk of cancer (18, 19). The cellular response to ELF-MF may depend on many parameters including frequency, waveform, the strength and the exposure duration of the electromagnetic field, and genetic/biological characteristics of the cells (4, 20, 21).

The present study investigated and compared the effect of ELF-MF on healthy fibroblasts and breast cancer cells. Healthy and tumour cells were exposed to a 1 mT ELF-MF at 50 Hz frequency for 1, 3, and 5 hours, and then the cell cycle, proliferation, and apoptosis/necrosis were observed in comparison.

MATERIAL AND METHOD

In-vitro ELF-EMF exposure system

In vitro exposures were performed using the Helmholtz paired coil-based ELF exposure system (Figure 1) settled in a 5 % CO_2 incubator at 37°C. A 50 Hz sinusoidal magnetic field, which was homogeneous throughout the axis of the coil pair, was generated with this system. The identical coils configured in the Helmholtz design were made up of 100 turns of copper wire with a diameter of 13 cm. The power generator which produced the AC magnetic field was connected to the coils. The coils were settled in a horizontal plane to produce a vertically oriented magnetic field. The distance between the coils was 6.5 cm. The electrical parameters of the coils are given in Table 1. The magnetic field was measured using a Hall-Effect Gaussmeter (Yokogawa, Tokyo, Japan). The petri dishes containing cell suspensions were located at the centre of the coil-based exposure system which had a homogenous ELF-EMF distribution. The cells were exposed to a 50 Hz ELF-MF with a field strength of 1 mT for 1, 3, and 5 hours.

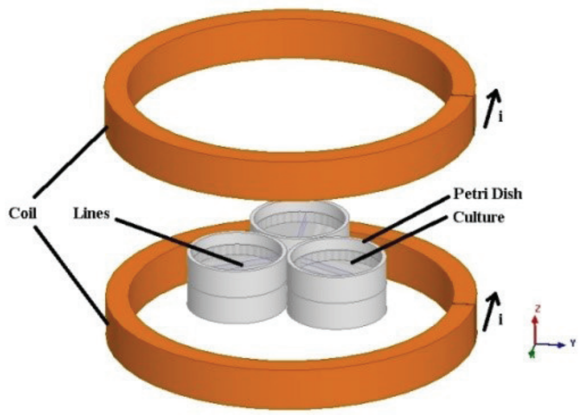

Figure 1: Schematic diagram of the Helmholtz coil-based ELF exposure system.

Table 1: The electrical parameters of the coils.

Coil Parameters	Value
Number of turns	100
Inner diameter (cm)	11.4
Outer diameter (cm)	13
Thickness (cm)	1.5
Resistance	5.15
Inductance	5.4

Cell culture

Human, neonatal, healthy primary dermal fibroblast (ATCC® PCS-201-010™) and MCF7 cell lines (ATCC® HTB-22™, human metastatic breast cancer cells) were maintained in Dulbecco's modified Eagle medium supplemented with 10% heat-inactivated fetal bovine serum (FBS), 50 U/ml penicillin and 50 µg/ml streptomycin. The cells were cultured in a 95% humidified atmosphere containing 5% CO_2 at 37 °C. These adhesive cells were passaged every three days by trypsinization and washed with Ca and Mg-free phosphate-buffered saline (PBS) and fed with the same media. They were seeded on a 24-well plate 24 h before starting the experiments. 2×10^5 cells were plated for each well. The control group, consisting of both healthy fibroblasts and MCF7 cells, was kept in a separate incubator with the same conditions and was not exposed to an ELF-MF.

Cell survival

WST-1 Assay (Roche, Cell Proliferation Reagent WST-1) was applied for cell viability. This is a colorimetric test for the relative quantitation of cell proliferation in a culture medium. Briefly; after the exposures, 10000 cells/well in 90 µl DMEM were plated in 96-well plates and 10 µl of cell proliferation reagent WST-1 was added to each well and incubated for 2 hours. After incubation, the plate was read with the TECAN-Sunrise ELISA Reader (Switzerland) at 450 nm with a reference of 630 nm. The reference readings were subtracted from the original readings at 450 nm. The results were presented as a percent of cell viability (% of control). All experiments were performed in triplicate under blind conditions.

Annexin-V-FITC/ Propidium iodide (PI) Staining

The percentages of apoptotic and necrotic cells were analysed using Annexin-V-FITC and Propidium Iodide (PI)

staining (eBioscience, Annexin V-FITC Apoptosis Detection Kit) using a flow cytometer (BD Bioscience, FACSCalibur) in this study. The change in the plasma cell membrane is one of the earliest features of apoptosis. The exposure of phosphatidylserine (PS) from the cytoplasmic surface of the plasma cell membrane to the external surface is one of the hallmarks of early-phase apoptosis. The membrane remains intact in this phase. The translocation PS to the external surface may be determined using several fluorescence probes. Annexin-V is a 35–36 k DA Ca^{2+} dependent anti-coagulant protein and has a high affinity for binding PS. Necrotic cells are permeable for Propidium Iodide (PI) which intercalates in DNA bases and emits red fluorescence.

Briefly, when ELF treatments were applied, the cells were washed and resuspended in 195 µl binding buffer at a density of $2x10^5$ cells/ml. Then, 5 µl Annexin-V-FITC (eBioscience) dye was added to each cell suspension (195 µl). The cells were gently vortexed and incubated for 10 min in the dark. Afterwards, the cells were washed at 1200 rpm for 5 minutes, the supernatant was discarded and the pellet was resuspended in 190 µl binding buffer. 10 µl PI (20 µg/ml) was added to each cell suspension and the samples were read and analysed using the FacsCalibur Flow Cytometer (Becton–Dickinson). All experiments were performed in triplicate under blind conditions.

Analytical Hierarchy Process (AHP)

AHP is a numerical method that allows you to sort decision options and select one of them according to the multiple criteria specified. To implement this method, the following steps are performed: first, each decision option is given numerical points showing how much it meets the set criteria. For example, we used the averages of our test results. Then, by making binary comparisons, a matrix is created for each alternative. These matrices are normalized and their consistency is checked. Then, with the help of matrix algebra, an average score is obtained for each alternative. The alternative with the highest score is the most appropriate alternative according to the decision maker's comparisons.

Statistical method

All data were analysed using the independent one-tailed Student's t-test. $p<0.05$ was considered as statistically significant.

RESULTS

Our data showed that the ELF-MF did not affect proliferation, apoptosis, and cell cycle of breast cancer cells (MCF7). However, the proliferation of healthy breast fibroblasts was decreased (Figure 2) and apoptosis (Figure 3) was increased significantly. For the first time, the effect of ELF-MF on healthy fibroblasts and breast cancer cells was investigated simultaneously.

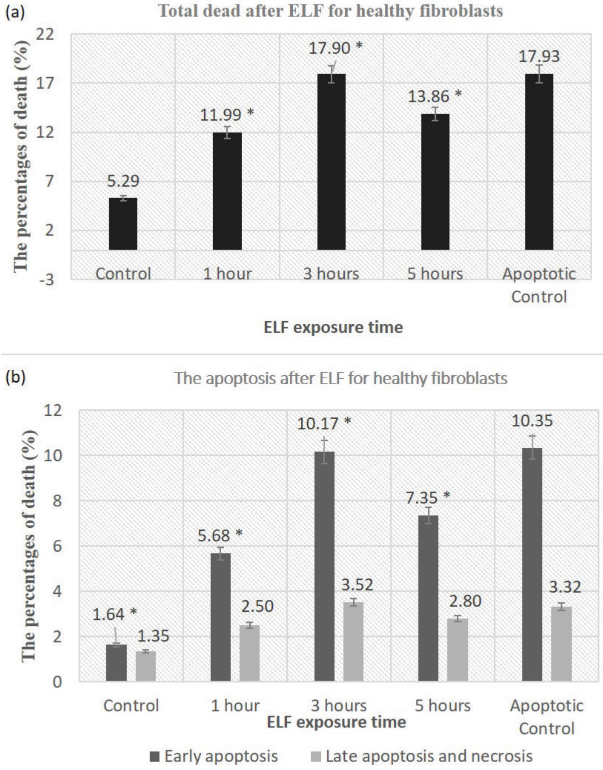

Figure 2: The total dead (a), early apoptosis, late apoptosis and necrosis (b) percentages after 1 mT-50 Hz ELF-MF exposures on healthy fibroblasts. It was observed that the ELF-MF triggers apoptosis but does not cause necrotic death. *The statistically significant percentages.

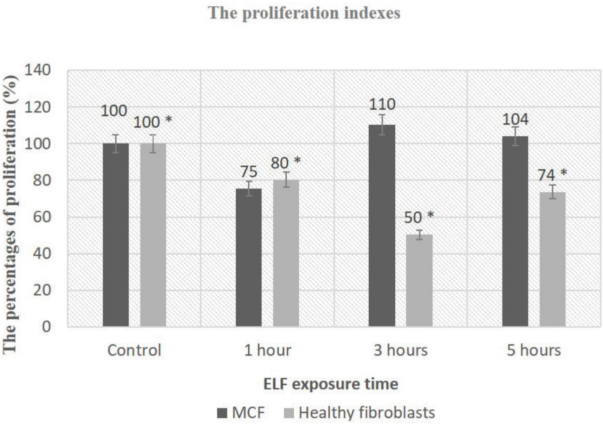

Figure 3: The figure shows viability rates in breast cancer cells and healthy fibroblasts after 1 mT-50 Hz ELF-MF exposure. *The statistically significant percentages.

The AHP method was applied and as a result of the AHP matrix, we concluded that 1-hour ELF-MF exposure was the most effective exposure time.

DISCUSSION

Studies have shown that ELF-MFs can have different effects in different tissues/cell lines. The biological mechanism of the cells' response to it has not been elucidated yet. After ELF-MF exposure, activation of inflammatory pathways or significant reactive oxygen species (ROS) accumulation occurs in some cell lines, while some other cell lines were found to have no significant effect. This may be due to genetic differences. Although ELF-MFs seem to have very low energy to cause mutations in DNA, they may make the cells more vulnerable to the harmful effects of the environment by epigenetic means and thus pave the way for tumour formation. Indeed, a study showed that ELF-MFs did not directly affect the methylation or histone modifications of leukaemia cells but made them susceptible to DNA and histone modifications by stabilizing active chromatins (1). Some groups showed that oxidative stress levels were increased and reactive oxygen species (ROS) accumulation occurred after ELF-MF exposure (22, 23). The increase in the amount of ROS formation may cause DNA damage implicitly and apoptotic cell death. ELF EMFs may affect the permeability of the plasma cell membrane which may cause dysfunction of ion channels existing on the membrane. Moreover, it has been observed that free radical scavengers have the ability to prevent genotoxic effects. These observations supported the idea the ELF-EMFs may induce ROS production which may lead to DNA damage (8, 24). The Fenton reaction in which hydroxyl radicals are generated may play an important role in free radical generation in ELF exposed systems (25). Moreover, it was indicated that the ELF-MFs altered some antioxidant genes such as *SOD2, GSTM3, MGST1,* and *MGST3* (26). Some groups showed that immune cell activation occurred due to the 50 Hz and 1 mT ELF-EMF exposure. ELF EMFs may also induce cytokine formation (IL-IB) in addition to free radicals in human monocytes (27-29).

In this study, we have seen the effects of ELF-MFs on two different cell lines, one healthy group and one tumour cells group, with a healthy primary dermal fibroblast and with human metastatic breast cancer cells (MCF7). The apoptotic activity of cancer cells exposed to ELF-MFs has been investigated in different tumour types in the literature. A 50 Hz, 45 mT ELF treatment for 1- 2.5 h induced apoptotic cell death in Human Myelogenous Leukemic cell lines (HL60 and ML-1) cells (30). No significant change was observed in the apoptotic rate of healthy peripheral leukocytes in the same exposure conditions (30). The number of apoptotic cells and micronucleus formation was increased significantly when SCL II (human squamous cell carcinoma) cells were treated with 50 Hz and 0.8–1 mT ELF-EMF for 48 and 72 h (31). Chen et al (32) reported a significant reduction in viability of HeLa cells that were exposed to a 60 Hz, 1.2 mT ELF for 72 h. Similarly, in another study, the survival rate of PC–12 cells was decreased when they were exposed to the ELF at 60 Hz (33). The short term effects of exposure to a 1.5 mT, 1 Hz ELF field has been shown in human colon adenocarcinoma cells. The proliferation rate of cells was significantly decreased due to 360 min ELF exposure (33). Dexamethasone induced cell proliferation inhibition was observed in HCA–2 human adenocarcinoma cells that were exposed to 25 Hz, 1 mT ELF for durations of between 45 and 120 min (34). ELF EMFs may also be used in cancer treatment. A 0.5 mT (1 h per day) ELF-EMF exposure reduced implanted tumour growth in mice (35). Similarly, the survival rate of human colon and breast adenocarcinoma cells was decreased due to 1 mT ELF exposure (36).

The results of our study showed that the ELF-MF did not affect breast cancer cells. However, it led to the death of healthy cells. Also, the ELF-MF did not alter the cell cycle of healthy fibroblasts showing that it did not cause any arrest i.e. the DNA repair mechanism might not work. Although ELF-MFs have been shown to cause changes in inflammatory pathways, immune system, ROS accumulation, histones, and some genes, studies to date have still needed research on how the mechanism works. The study we conducted showed the damaging effect of ELF-MFs on healthy cells.

Ethics Committee Approval: Ethics committee approval is not required.

Informed Consent: Informed consent is not required.

Peer Review: Externally peer-reviewed.

Author Contributions: Conception/Design of Study- H.K.; Data Acquisition- H.K.; Data Analysis/Interpretation- H.K., S.G.; Drafting Manuscript- H.K., M.A.E.; Critical Revision of Manuscript- E.E., S.G.; Final Approval and Accountability- H.K., B.E., S.G., M.A.E., E.E., A.G.C.; Technical or Material Support- H.K., A.G.C., S.G.; Supervision- M.A.E.

Conflict of Interest: Authors declared no conflict of interest.

Financial Disclosure: Authors declared no financial support.

Etik Komite Onayı: Etik kurul onayı gerekmemektedir.

Bilgilendirilmiş Onam: Bilgilendirilmiş onam gerekmemektedir.

Hakem Değerlendirmesi: Dış bağımsız.

Yazar Katkıları: Çalışma Konsepti/Tasarım-H.K.; Veri Toplama- H.K.; Veri Analizi/Yorumlama- H.K., S.G.; Yazı Taslağı- H.K., M.A.E.; İçeriğin Eleştirel İncelemesi- E.E., S.G.; Son Onay ve Sorumluluk- H.K., B.E., S.G., M.A.E., E.E., A.G.C.; Malzeme ve Teknik Destek- H.K., A.G.C., S.G.; Süpervizyon- M.A.E

Çıkar Çatışması: Yazarlar çıkar çatışması beyan etmemişlerdir.

Finansal Destek: Yazarlar finansal destek beyan etmemişlerdir.

REFERENCES

1. Manser M, Sater MR, Schmid CD, Noreen F, Murbach M, Kuster N, et al. ELF-MF exposure affects the robustness of epigenetic programming during granulopoiesis. Sci Rep 2017;7:43345. [CrossRef]
2. Goraca A, Ciejka E, Piechota A. Effects of extremely low frequency magnetic field on the parameters of oxidative stress in heart. J Physiol Pharmacol 2010;61(3):333-8.
3. Lange S, Viergutz T, Simko M. Modifications in cell cycle kinetics and in expression of G1 phase-regulating proteins in human amniotic cells after exposure to electromagnetic fields and ionizing radiation. Cell Prolif 2004;37(5):337-49. [CrossRef]
4. Trillo MA, Martinez MA, Cid MA, Leal J, Ubeda A. Influence of a 50 Hz magnetic field and of all-trans-retinol on the proliferation of human cancer cell lines. Int J Oncol 2012;40(5):1405-13. [CrossRef]
5. Cid MA, Ubeda A, Hernandez-Bule ML, Martinez MA, Trillo MA. Antagonistic effects of a 50 Hz magnetic field and melatonin in the proliferation and differentiation of hepatocarcinoma cells. Cell Physiol Biochem 2012;30(6):1502-16. [CrossRef]
6. Wei M, Guizzetti M, Yost M, Costa LG. Exposure to 60-Hz magnetic fields and proliferation of human astrocytoma cells in vitro. Toxicol Appl Pharm 2000;162(3):166-76. [CrossRef]
7. Eleuteri AM, Amici M, Bonfili L, Cecarini V, Cuccioloni M, Grimaldi S, et al. 50 Hz extremely low frequency electromagnetic fields enhance protein carbonyl groups content in cancer cells: effects on proteasomal systems. J Biomed Biotechnol 2009;2009:834239. [CrossRef]
8. Lai H, Singh NP. Acute exposure to a 60 Hz magnetic field increases DNA strand breaks in rat brain cells. Bioelectromagnetics 1997;18(2):156-65. [CrossRef]
9. Kaszuba-Zwoinska J, Ciecko-Michalska I, Madroszkiewicz D, Mach T, Slodowska-Hajduk Z, Rokita E, et al. Magnetic field anti-inflammatory effects in Crohn's disease depends upon viability and cytokine profile of the immune competent cells. J Physiol Pharmacol 2008;59(1):177-87.
10. Sutbeyaz ST, Sezer N, Koseoglu F, Kibar S. Low-frequency Pulsed Electromagnetic Field Therapy in Fibromyalgia A Randomized, Double-blind, Sham-controlled Clinical Study. Clin J Pain 2009;25(8):722-8. [CrossRef]
11. Markov MS. Magnetic field therapy: A review. Electromagn Biol Med 2007;26(1):1-23. [CrossRef]
12. Lappin MS, Lawrie FW, Richards TL, Kramer ED. Effects of a pulsed electromagnetic therapy on multiple sclerosis fatigue and quality of life: A double-blind, placebo controlled trial. Altern Ther Health M 2003;9(4):38-48.
13. Bertolino G, Braga AD, Rosa KDLD, Brito LCD, de Araujo JE. Macroscopic and histological effects of magnetic field exposition in the process of tissue reparation in Wistar rats. Arch Dermatol Res 2006;298(3):121-6. [CrossRef]
14. Cane V, Botti P, Soana S. Pulsed Magnetic-Fields Improve Osteoblast Activity during the Repair of an Experimental Osseous Defect. J Orthopaed Res 1993;11(5):664-70. [CrossRef]
15. Cameron IL, Sun LZ, Short N, Hardman WE, Williams CD. Therapeutic Electromagnetic Field (TEMF) and gamma irradiation on human breast cancer xenograft growth, angiogenesis and metastasis. Cancer Cell Int 2005;5:23. [CrossRef]
16. Yamaguchi S, Ogiue-Ikeda M, Sekino M, Ueno S. The effect of repetitive magnetic stimulation on the tumor development. Ieee T Magn 2004;40(4):3021-3. [CrossRef]
17. Kirson ED, Gurvich Z, Schneiderman R, Dekel E, Itzhaki A, Wasserman Y, et al. Disruption of cancer cell replication by alternating electric fields. Cancer Res 2004;64(9):3288-95. [CrossRef]
18. Barbault A, Costa FP, Bottger B, Munden RF, Bomholt F, Kuster N, et al. Amplitude-modulated electromagnetic fields for the treatment of cancer: Discovery of tumor-specific frequencies and assessment of a novel therapeutic approach. J Exp Clin Cancer Res 2009;28(1):51. [CrossRef]
19. Chen G, Lu D, Chiang H, Leszczynski D, Xu Z. Using model organism Saccharomyces cerevisiae to evaluate the effects of ELF-MF and RF-EMF exposure on global gene expression. Bioelectromagnetics 2012;33(7):550-60. [CrossRef]
20. Tenuzzo B, Chionna A, Panzarini E, Lanubile R, Tarantino P, Di Jeso B, et al. Biological effects of 6 mT static magnetic fields: A comparative study in different cell types. Bioelectromagnetics 2006;27(7):560-77. [CrossRef]
21. Focke F, Schuermann D, Kuster N, Schar P. DNA fragmentation in human fibroblasts under extremely low frequency electromagnetic field exposure. Mutat Res-Fund Mol M 2010;683(1-2):74-83. [CrossRef]
22. Luukkonen J, Liimatainen A, Juutilainen J, Naarala J. Induction of genomic instability, oxidative processes, and mitochondrial activity by 50Hz magnetic fields in human SH-SY5Y neuroblastoma cells. Mutat Res 2014;760:33-41. [CrossRef]
23. Tiwari R, Lakshmi NK, Bhargava SC, Ahuja YR. Epinephrine, DNA integrity and oxidative stress in workers exposed to extremely low-frequency electromagnetic fields (ELF-EMFs) at 132 kV substations. Electromagn Biol Med 2015;34(1):56-62. [CrossRef]
24. Lai H, Singh NP. Melatonin and a spin-trap compound block radiofrequency electromagnetic radiation-induced DNA strand breaks in rat brain cells. Bioelectromagnetics 1997;18(6):446-54. [CrossRef]
25. Lai H, Singh NP. Magnetic-field-induced DNA strand breaks in brain cells of the rat. Environ Health Persp 2004;112(6):687-94. [CrossRef]
26. Mahmoudinasab H, Sanie-Jahromi F, Saadat M. Effects of extremely low-frequency electromagnetic field on expression levels of some antioxidant genes in human MCF-7 cells. Mol Biol Res Commun 2016;5(2):77-85.
27. Rollwitz J, Lupke M, Simko M. Fifty-hertz magnetic fields induce free radical formation in mouse bone marrow-derived promonocytes and macrophages. Biochim Biophys Acta 2004;1674(3):231-8. [CrossRef]
28. Frahm J, Lantow M, Lupke M, Weiss DG, Simko M. Alteration in cellular functions in mouse macrophages after exposure to 50 Hz magnetic fields. J Cell Biochem 2006;99(1):168-77. [CrossRef]
29. Lupke M, Rollwitz J, Simko M. Cell activating capacity of 50 Hz magnetic fields to release reactive oxygen intermediates in human umbilical cord blood-derived monocytes and in Mono Mac 6 cells. Free Radic Res 2004;38(9):985-93. [CrossRef]
30. Hisamitsu T, Narita K, Kasahara T, Seto A, Yu Y, Asano K. Induction of apoptosis in human leukemic cells by magnetic fields. Jpn J Physiol 1997;47(3):307-10. [CrossRef]

31. Simko M, Kriehuber R, Weiss DG, Luben RA. Effects of 50 Hz EMF exposure on micronucleus formation and apoptosis in transformed and nontransformed human cell lines. Bioelectromagnetics 1998;19(2):85-91. [CrossRef]
32. Chen YC, Chen CC, Tu W, Cheng YT, Tseng FG. Design and fabrication of a microplatform for the proximity effect study of localized ELF-EMF on the growth of in vitro HeLa and PC-12 cells. J Micromech Microeng 2010;20(12):125023. [CrossRef]
33. Ruiz-Gomez MJ, de la Pena L, Prieto-Barcia MI, Pastor JM, Gil L, Martinez-Morillo M. Influence of 1 and 25 Hz, 1.5 mT magnetic fields on antitumor drug potency in a human adenocarcinoma cell line. Bioelectromagnetics 2002;23(8):578-85. [CrossRef]
34. Ruiz-Gomez A, Ruiz-Gomez MJ, Pastor JM, De la Pena L, Martinez M. Stochastic simulation of logistic growth of a colon adenocarcinoma cell line. Europ J Cancer 1999;35:S28.
35. Hannan CJ, Jr., Liang Y, Allison JD, Pantazis CG, Searle JR. Chemotherapy of human carcinoma xenografts during pulsed magnetic field exposure. Anticancer Res 1994;14(4A):1521-4.
36. Tofani S, Barone D, Berardelli M, Berno E, Cintorino M, Foglia L, et al. Static and ELF magnetic fields enhance the in vivo anti-tumor efficacy of cis-platin against lewis lung carcinoma, but not of cyclophosphamide against B16 melanotic melanoma. Pharmacol Res 2003;48(1):83-90. [CrossRef]
