# Supplementary material for: Magnetic Fields and Cancer: Epidemiology, Cellular Biology, and Theranostics
Source: Int J Mol Sci. 2022 Jan 25;23(3):1339. doi: 10.3390/ijms23031339 (PMC8835851; doi:10.3390/ijms23031339)
Supplement: Supplementary file 1 [file ijms-23-01339-s001.zip › Supplementary Data Set S1/MF and Cancer.Data/PDF/3120985801/Maes-Verschaeve2016_Article_GeneticDamageInHum.pdf]

Arch Toxicol (2016) 90:2337–2348
DOI 10.1007/s00204-016-1769-9

REVIEW ARTICLE

Genetic damage in humans exposed to extremely low-frequency electromagnetic fields

A. Maes[1] · **L. Verschaeve**[1,2]

Received: 26 May 2016 / Accepted: 15 June 2016 / Published online: 23 June 2016
© Springer-Verlag Berlin Heidelberg 2016

Abstract The classification of extremely low-frequency magnetic fields by the International Agency for Research on Cancer in the group of 'possible human carcinogens' (group 2B) is essentially based on epidemiologic evidence showing an association between MF exposures and childhood leukaemia. Despite many in vitro and in vivo investigations, there is no established causal relationship yet. However, human cytogenetic biomonitoring studies that were conducted in the past show predominantly positive results, i.e. increased cytogenetic damage in peripheral blood lymphocytes or buccal cells of ELF-MF-exposed subjects. This is important given the established link between observed cytogenetic damage in cells of people and an increased cancer risk. We here conducted an evaluation of the published investigations and found that many of the studies clearly have shortcomings, which often prevent any firm conclusion. As a matter of fact, there are reasons to believe that effects are not that impressive. However, the totality of the studies cannot simply be disregarded warranting further caution and the application, to a certain extent, of the precautionary principle.

Keywords Magnetic fields · ELF · Cytogenetic damage · Lymphocytes · Buccal cells · Workers

✉ L. Verschaeve
luc.verschaeve@wiv-isp.be

1 Scientific Institute of Public Health, O.D. Food, Medicines and Consumers Safety, J. Wytsmanstreet 14, 1050 Brussels, Belgium

2 Department of Biomedical Sciences, University of Antwerp, Antwerp, Belgium

Introduction

Extremely low-frequency magnetic fields (ELF-MF) have been classified by the International Agency for Research on Cancer (IARC) as 'possible carcinogenic to humans' (group 2B). This was essentially because of the observed association with childhood leukaemia (IARC 2002). Although some scientists are in favour of a re-evaluation based on new analyses and recent less convincing study results (e.g. Leitgeb 2015a, b), this association is at present still accepted (SCENIHR 2015). However, a causal relationship between magnetic field exposures and childhood leukaemia was never established and laboratory investigations also did not provide convincing supportive evidence (EHC 2007; Schmiedel and Blettner 2010; SCENIHR 2015). For example, results from studies on ELF-MF-induced genetic effects are controversial and most scientists do not consider that the available data clearly point towards such effects. Because of the low energy levels in molecular interactions, it is physically highly implausible that ELF fields cause direct genetic damage. However, it has been theorised that ELF may enhance such damage from other sources (e.g. endogenous radicals) or that epigenetic (non-genotoxic) interference in signal transduction may enhance cancer formation. Yet, studies on the effects of ELF magnetic field exposure of cells did generally not show genotoxic effects at magnetic flux densities well above those found in daily life situations. There is, however, some evidence that ELF magnetic fields may interact with DNA-damaging agents and be co-genotoxic (Vijayalaxmi and Obe 2005; Bergqvist et al. 2003; EHC 2007; Udroiu et al. 2010; Markkanen 2009). It also should be stressed that, as pointed out by Udroiu et al. (2010), possible aneugenic effects of electromagnetic fields did not get much attention so far despite the growing interest for the link between aneuploidy and

carcinogenesis. Some evidence of ELF-MF-induced aneuploidy was already published yet (Udroiu et al. 2006; Maes et al. 2016).

As genetic damage is very often a prerequisite for cancer, not only in vitro and in vivo animal studies were conducted but also several cytogenetic biomonitoring studies in people who were occupationally exposed to electric and magnetic fields. Most of these studies showed an increased frequency of genetic damage in the white blood or exfoliated buccal cells of the workers (Table 1). Despite above-mentioned uncertainties and lack of convincing evidence in in vitro and in vivo investigations, these human studies are often seen as alarming and supportive for an ELF-MF-induced cancer risk. However, a careful and critical investigation of these studies is needed to identify possible methodological shortcomings and hence better appreciate the validity of the studies. A previous critical evaluation was, for example, performed with respect to cytogenetic biomonitoring studies of subjects being exposed to radiofrequency fields, and this revealed the presence of many shortcomings preventing any clear conclusion, even when the majority of studies showed genetic damage in the blood and buccal cells of the exposed subjects (Verschaeve 2009). The same might be true when ELF (electro)magnetic fields are considered. We here present an evaluation of cytogenetic biomonitoring studies on ELF-(electro)magnetic field (ELF-EMF)-exposed subjects as published in the scientific literature.

Cytogenetic investigations of human subjects occupationally exposed to ELF-EM fields

Several but yet relatively few studies were published on the cytogenetic damage in cells from ELF-EMF-exposed persons. Most investigations were on peripheral blood lymphocytes. In some of the studies also buccal epithelial cells were investigated. A short overview of these studies and their conclusions is given here (Table 1).

Bauchinger et al. (1981) investigated structural chromosome aberrations and sister chromatid exchanges (SCE) in blood cells of subjects following long-term exposure to electric and magnetic fields. The chromosome analysis was carried out in the lymphocytes of 32 workers who were occupationally exposed for more than 20 years to 50 Hz alternating electric and magnetic fields from 380 kV switchyards. As a control group, 22 workers of a similar age were included. Their occupation was also similar but did not coincide with ELF-EM exposure. There was no difference in the frequencies of chromosome aberrations and SCE between both groups.

Skyberg et al. (1993) investigated 13 laboratory employees exposed to electromagnetic fields. From them, seven were high-voltage (up to 200 kV) laboratory cable splicers and six engineers exposed to static, alternating or pulsed electric and magnetic fields. Matched controls consisted of 20 subjects with a similar job description (but no exposure), age and smoking behaviour. The alternating 50 Hz magnetic fields were usually 5–10 µT but may occasionally have reached much higher values (±500 µT whole-body exposure; ±10,000 µT at the level of the hands). Chromosome aberrations, SCEs and aneuploidy (numerical chromosome aberrations) were studied in the subject's peripheral white blood cells. In this study, an increased frequency in structural chromosome aberrations was found but not in SCEs or aneuploidy.

Valjus et al. (1993) examined lymphocytes from power line inspectors and maintenance personnel who had a more than 10-year exposure to electromagnetic fields. They found a twofold increase in the incidence of chromatid breaks compared with unexposed controls, but no difference with respect to SCEs and micronuclei.

Nordenson et al. (1984, 1988, 2001) performed several cytogenetic biomonitoring studies on occupationally exposed subjects. A study of chromosome aberrations in peripheral blood lymphocytes of 20 switchyard workers at 400 kV substations revealed increased frequencies of such aberrations compared with the controls (Nordenson et al. 1984). In a follow-up study, 38 employees of electric power companies were studied; amongst them, 19 of the subjects were involved in the repair and maintenance of circuit breakers and disconnectors in 400 kV substations. The other 19 individuals served as controls and were only exposed to normal environmental electromagnetic fields. The frequency of cells with chromosomal aberrations and micronuclei was significantly increased compared with the frequencies in the control cells. SCEs were not increased (Nordenson et al. 1988). Another study of Nordenson et al. (2001) was conducted on train engine drivers, train dispatchers, office workers and policemen. The drivers were exposed to magnetic fields ranging from a few µT to more than 100 µT. Chromosome aberrations were again investigated in peripheral lymphocytes. A pilot study of 18 engine drivers revealed a significant four times higher frequency of cells with chromosome aberrations compared with a control group of 16 office workers. A follow-up study of another 30 engine drivers and 30 policemen (used a controls) again showed a significant increase in the frequency of cells with chromosome-type aberrations.

A study by Othman et al. (2001) was specifically devoted to aneuploidy and involved 18 male traffic controllers and engineers exposed to electromagnetic fields. They had a statistically increased frequency of monosomy of chromosome 7 and 17 and loss of the Y chromosome compared with a matched control population of five male individuals. The numerical chromosome aberrations were investigated with fluorescence in situ hybridisation (FISH) techniques.

Another investigation of Skyberg et al. (2001) was again on high-voltage laboratory workers exposed to electromagnetic fields and mineral oil. The study population consisted

Table 1 Overview of human biomonitoring studies of workers occupationally exposed to ELF-electromagnetic fields

Cytogenetic endpoint studied	Field exposure	Number of persons	Number of cells per person	Results	References
CA and SCE	50 Hz E and M fields from 380 kV switchyards systems; exposure for more than 20 years (during working hours)<br>Magnetic flux densities and electric field strengths were not specified	32 Workers<br>22 Controls (similar occupation but no exposure)	500 (CA) and 50 (SCE)	No effects	Bauchinger et al. (1981)
CA, SCE and aneuploidy	50 Hz and DC/static fields; high-voltage cables 1 m above the floor; several hours/day; 10 kV/m and 15 µT. When touching the cable workers were exposed to up to 500 µT (body) and 10,000 µT (hand)<br>1 Hz–3 MHz; as above; up to 100 (3 µs) pulses per workday; 1 day per week; 2 kV and 20 µT	13 Exposed subjects: 7 high-voltage laboratory workers and 6 engineers<br>20 Control subjects (similar occupation but no exposure, same age and smoking behaviour)	200 (CA and aneuploidy) and 50 (SCE)	Increased frequency of CA but not SCE or aneuploidy	Skyberg et al. (1993)
CA, SCE and MN	Power linesmen with exposure to 50 Hz, 110 kV or 400 kV power systems for ≥10 years	27 Non-smoking power linesmen compared to 27 non-smoking telephone linesmen as controls (matched for age and geographical location)	100 (CA), 30 (SCE) and 500 (MN)	Twofold increase in chromatid breaks but no effect on SCE and MN<br>Smoking behaviour may be a confounding factor	Valjus et al. (1993)
CA (including G banding)	High-voltage cable worker who had been working 8 h a day for the previous 8 years in a power substation with exposure to 154 kV at 50 Hz	One 32-year-old, high-voltage cable worker	85 in the test person; no control (?)	High level of CA (including endoreduplications)	Erdal et al. (1999)
CA	50 Hz; Switchyard workers exposed 1–8 weeks during working time at 400 kV substations	20 Workers compared to 17 control subject	200	The rates of chromatid and chromosome breaks were found to be significantly increased compared with the controls	Nordenson et al. (1984)

Table 1 continued

Cytogenetic endpoint studied	Field exposure	Number of persons	Number of cells per person	Results	References
CA, SCE and MN	50 Hz; workers involved in the repair and maintenance of circuit breakers and disconnectors in 400 kV substations. Workers were occupationally exposed for a period of 13 ± 9 years	19 Exposed and 19 control subjects	100–200 (CA), ≤20 (SCE) and 1000 (MN)	Increased frequencies op CA and MN but not SCE	Nordenson et al. (1988)
CA	Railway engine drivers; 16.66 Hz magnetic fields; up to 100 µT with widely variations; 0.13–0.18 µT for referent train dispatchers; exposure continuous for whole working day	18 (Drivers) and 7 (dispatchers) as concurrent referents + a control group of 16 office workers<br>30 Drivers and 30 referent policemen in follow-up study	100	Increased frequency of chromosome-type aberrations in engine drivers compared with other groups	Nordenson et al. (2001)
Aneuploidy	Air traffic controllers and engineers being exposed to EMF-fields from radar screens, antennae, satellite installations, etc. for 10–27 years. Exposure is thus not only to ELF-EMF!!	18 Exposed and 5 control individuals	100	Overall, increased frequencies of monosomy of the chromosomes 7, 17 and Y	Othman et al. (2001)
CA (normal lymphocyte cultures but also cultures treated with hydroxyurea and caffeine)	60 Hz; a generator soldering and transformer testers group working in high-voltage laboratories. Exposures were to different magnetic and electric field strengths (e.g. 6 µT–7 mT). Exposure duration was also variable	24 Exposed and 24 matched controls (smoking, alcohol and coffee consumption was taken into account)	200	No effect in conventional lymphocyte cultures but increased CA frequencies in DNA synthesis and DNA repair inhibited cultures.<br>Years of exposure and smoking increased the risk	Skyberg et al. (2001)
CA	Subjects exposed to EMF from video display monitors	10 Exposed and 10 control subjects	200	Significant increase in CA in exposed vs. control subjects	Higino Estécio and Silva (2002)
SCE	Subjects professionally exposed to ELF-MF (various occupations)	70 Workers divided in low (0.1 µT) and high (>0.4 µT) exposed subjects	30	No effects	Gobba et al. (2003)
CA and MN	Subjects working for 1–10 years with photocopying machines at a rate of 8–10 h per day for 6 days a week	98 Exposed and 90 unexposed controls (clerks, attenders and students)	≤100 for CA and 2000 for MN	Significant increase in MN in lymphocytes and buccal epithelial cells due to the exposure (ELF-EMF but also toxic chemicals)	Goud et al. (2004)

Table 1 continued

Cytogenetic endpoint studied	Field exposure	Number of persons	Number of cells per person	Results	References
MN	Subjects occupationally exposed to EM fields from video display monitors. Average working time was 14 ± 7.44 years	20 Exposed and 20 unexposed (?) control subjects (matched for age and sex)	2000	Increased frequency of MN and broken egg cells in exfoliated buccal cells from the exposed subjects	Carbonari et al. (2005)
CA and MN	50 Hz; Workers working for 19 ± 7 years in transformer and distribution line stations (154–380 kV). Electric and magnetic field strengths resp. between 130–1500 V/m and 0.25–17 A/m	32 Transformer workers and 23 office workers<br>17 Control subjects	50 (CA), 1000 (MN)	Significantly higher frequencies of both CA and MN in the 'electrical workers'. CA increased with the years of exposure	Celikler et al. (2009)
CA and SCE	Electric train engine drivers. Exposure 'assumed' to be high	15 Electric train engine drivers and 15 controls consisting of train guards (same age and socio-economic status)	100 (CA) and ±30 (SCE)	No effect (and no indications of synergistic effects with mitomycin C)	Gadhia et al. (2010)
MN and SCE	50 Hz; welders exposed to EMF via electric arc welding apparatus during the working shift (7 am to 5 pm). Magnetic flux density of 0.03–345.06 μT (mean value = 7.81 μT)	21 Welders and 21 non-exposed controls (matched for age, residence and smoking habit)	2000 for MN and 100 for SCE	Micronuclei frequency in the exposed workers was significantly higher but the sister chromatid exchange frequency was significantly lower in exposed subjects compared with the controls	Dominici et al. (2011)
'DNA comets'	Same as Dominici et al. (2011)	Id	50	DNA damage (tail intensity and tail moment) was significantly lower in the exposed group compared to the control group	Villarini et al. 2015)
CA and MN	50–60 Hz; electrical employees in transformers and power line (direct exposure) and office workers in places adjacent to electric supply substations (indirect exposure). Exposure duration from 20 ± 4.7 (direct) and 23 ± 6 years (indirect). Electric field strength 300–1500 V/m; magnetic field strength 0.25–17 A/m). As Celikler et al. (2009)?	50 Exposed and 20 control subjects	100 for CA and ≥1000 for MN	Significant increase in both CA and MN in exposed vs. control subjects	Balamuralikrishnan et al. (2012)

Table 1 continued

Cytogenetic endpoint studied	Field exposure	Number of persons	Number of cells per person	Results	References
'DNA comets'	Workers occupationally exposed for 2–30 years (mean = 9 years) to EMF from 132 kV substations	142 Exposed subjects and 151 controls (matched for age, socio-economic status and life-style factors)	200	Tendency towards increased DNA damage and increased oxidative stress parameters	Tiwari et al. (2015)
CA and SCE	50 Hz; workers occupationally exposed for 3–19 years) to electromagnetic field from a 132–230 kV electric supply substation	15 Workers and 8 controls	200 for CA & 25 for SCE	Increased frequency of CA but not of SCE. Cell proliferation indices and the mitotic index were lower in the exposed subjects	Khalil et al. (1993)
CA, SCE and MN	Subjects professionally exposed to ELF-MF (mean exposure = 0.35 μT)	109 Exposed subjects. 31 workers exposed to magnetic flux densities exceeding 1 μT were re-evaluated	Approximately 200 metaphases for CA; not mentioned for SCE and MN	No differences seen between low (<0. μT), moderate (>0.2 μT) and high (>1 μT) exposures	Scaringi et al. (2007)
'DNA comets' and MN	60 Hz; Human volunteers exposed for 4 h to magnetic flux density of 200 μT	20 Exposed and 10 control subjects	50 for DNA comets & 25 for SCE	No effects	Albert et al. (2009)
CA	50 Hz; subjects living close to power lines or professionally exposed via video display units<br>No exposure assessment done	24 VDU workers and 10 residential exposures<br>17 Control subjects	200	No effects	Maes (1998)

CA chromosome aberrations, *SCE* sister chromatid exchanges, *MN* micronuclei

of 24 individuals who were compared to 24 matched controls. The exposed group included employees from the high-voltage laboratory and generator soldering department. Due to their activities, they were exposed to both electric and magnetic fields as well as oil mist and vapour. The authors did not find excessive cytogenetic damage in the exposed subjects compared with the unexposed controls but found indications that the electromagnetic fields in combination with mineral oil exposure may produce chromosomal aberrations.

Higino Estécio and Silva (2002) found a significant higher frequency of aberrant metaphases and anomalies per cell in individuals exposed to radiation from video display monitors. Ten occupationally exposed individuals were studied, and the results were compared to these obtained in ten control subjects. The frequency of chromatid breaks was higher in the blood cells from EMF-exposed subjects compared with the controls.

Gobba et al. (2003) performed an investigation on peripheral blood lymphocytes from 70 workers exposed to various levels of ELF-EMF covering different occupations without the (known) involvement of exposure to mutagens and carcinogens. SCE frequencies, high-frequency cells (HFC) and the number of SCEs in HFC were investigated. No genotoxic effects were found at exposure levels of approximately 2 µT (the exposure levels currently found in most workplaces).

Goud et al. (2004) performed a micronucleus test in blood cells from subjects who regularly used photocopying machines and who were therefore exposed to toxic components of toners, toxic gazes as ozone, volatile organic components (VOCs) and extremely low-frequency electromagnetic fields. A total of 98 workers were included in this study as well as 90 age- and sex-matched controls. The workers had an increased frequency of both chromosome aberrations and micronuclei in their white blood cells. Increased micronucleus frequencies were also found in their buccal epithelial cells. Due to exposure to chemical agents as well and smoking as a confounding factor, it is very difficult to ascribe the results to the electromagnetic fields only.

Carbonari et al. (2005) found increased micronucleus frequencies as a result of exposure to electromagnetic fields from computer cathode ray tube video display monitors. Exposure was for at least 5 years and thus involved extremely low and very low electromagnetic fields. In this study, ten male and ten female occupational users of microcomputers were involved. The control population consisted of 20 unexposed subjects matched for age and gender. They were selected from the general population living in the same city. The frequency of micronuclei was studied in exfoliated buccal cells. Cells from EMF-exposed individuals had a higher frequency of micronuclei compared with the frequency in control cells. The effect was also significantly more pronounced in female individuals.

Another study was on occupational exposure to electric and magnetic fields involving 55 workers in transformer and distribution line stations in the Bursa province of Turkey (Celikler et al. 2009). The experimental group consisted of 32 technicians working inside the transformers and 23 office workers (outside the transformers). There were 17 control subjects who were working in different workplaces or were retired, housewives and students. Chromosome aberrations and micronucleus frequencies in peripheral lymphocytes were higher in the exposed 'electrical' workers. The frequency of chromosome aberrations furthermore increased with the years of exposure.

A cytogenetic investigation on railway engine drivers who were exposed to ELF-EMF was conducted by Gadhia et al. (2010). In this study, sister chromatid exchanges and structural chromosome aberrations were investigated. It was assumed that the engine drivers were exposed to relatively high magnetic field densities whereas their exposure to other (chemical) agents was assumed low and usually negligible. This study did not show any increased cytogenetic damage in the ELF-EMF-exposed subject and hence did not support the hypothesis that ELF-EMFs are genotoxic. This study involved a total of 15 railway engine drivers as the exposed population and 15 train guards as unexposed controls. Both groups matched with respect to age, habits and socio-economic conditions.

Welders are exposed to ELF magnetic field intensities that are 2–200 times higher than the exposure in most 'electrical occupations' and in households. The subjects who participated in the study of Dominici et al. (2011) were exposed to 0.03 µT up to a few hundred µT from electric arc welding apparatus. Exposure was, however, always lower than the 2004 European unit action value of 500 µT. In this study, cytogenetic effects were examined by means of the micronucleus and SCE test in the lymphocytes of 21 welders who were enrolled in two different welding companies in central Italy. The control population consisted of 21 non-exposed blood donors matched for age, residence and smoking habit. The exposed group showed 'dose-dependent' and significantly higher frequencies of micronuclei compared with the control group. On the other hand, there was a significant decrease in the frequency of SCEs.

Results of the alkaline comet assay in peripheral blood lymphocytes of the same welders and controls were published separately (Villarini et al. 2015). Data were presented for comet tail length, tail intensity and tail moment. According to the authors, there was significant less DNA damage (tail intensity and tail moment) in the blood cells of exposed welders compared with the unexposed probands.

Balamuralikrishnan et al. (2012) studied 70 Indian subjects from whom 50 were occupationally exposed to

low-frequency electromagnetic fields and 20 were unexposed controls. The 50 exposed subjects were subdivided into a group of 28 power line and transformer workers (direct exposure) and 22 electrical board office workers (indirect exposures). Lymphocytes from exposed subjects had higher frequencies of structural chromosome aberrations and micronuclei compared with the frequencies in cells from the control subjects. Chromosome aberrations and micronuclei frequencies increased with age in both exposed and non-exposed subjects, but this was statistically significant only in the EMF-exposed subjects. According to the authors, chronic occupational exposure to EMFs may lead to an increased risk of genetic damage among the electrical workers.

Tiwari et al. (2015) used the alkaline comet assay to investigate DNA damage in cells from workers at 132 kV substations who were exposed to ELF-EMFs for more than 2 years. Blood sample of 142 exposed subjects and 151 non-exposed individuals was analysed. A 'tendency' towards increased DNA damage was found in the exposed subjects compared with non-exposed controls, but statistical significance was not stated.

Khalil et al. (1993) investigated workers from a 132–230 kV supply station and found increased frequencies of chromosomal aberrations but not of sister chromatid exchanges.

Scaringi et al. (2007) briefly described the results of a cytogenetic investigation on ELF-MF exposed subjects (no precision). They found no difference between workers with low (<0.2 μT) and higher exposure levels (>0.2 μT and >1 μT). It was not clear how many cells were investigated per individual (especially for SCE and MN).

Other than professional exposures to ELF-MFs

All above studies were on occupationally exposed subjects. To our knowledge, there were only two investigations on other ELF-EMF-exposed persons. Albert et al. (2009) found no cytogenetic effects in human volunteers exposed for 4 h to magnetic flux densities of 200 μT, whereas Maes (1998) studied chromosomal aberrations in VDU workers and residentially (power line) exposed individuals. Here also, no cytogenetic effects could be attributed to the exposure. However, this was only a limited pilot experiment lacking any data on exposure levels or possible confounding factors.

Discussion

We now know that a high frequency of structural chromosomal aberrations in lymphocytes is predictive of an increased cancer risk, irrespective of the cause of the aberrations (Bonassi et al. 1995, 2000, 2007, 2008; Hagmar et al. 1998, 2004). The chromosome aberration test is therefore predictive for cancer at least at the level of a population. It is not predictive at the individual level as many factors may be responsible for an increased chromosome aberration frequency (recent illness or viral infection, etc.). Recent studies also provided evidence that an increased micronucleus frequency in peripheral lymphocytes is associated with an increased risk of cancer and other age-related degenerative diseases (Bonassi et al. 2007, 2011; Murgia et al. 2008; Migliore et al. 2011; Andreassi et al. 2011). Previous studies (e.g. Hagmar et al. 1998) did not find such an association, but the size of the cohort was too small and the material too heterogeneous to provide reliable findings. Moreover, most of the data were not obtained by using the more sensitive ex vivo/in vitro cytokinesis-block methodology (Mateuca et al. 2006). A high(er) micronucleus frequency in blood cells of a given population thus indicates that this population has a higher cancer risk. As for structural chromosome aberrations, this holds true at the level of the population but not at the individual level.

Sister chromatid exchanges and 'DNA comets' can be used as indicator tests for DNA damage and biomarkers of exposure rather than as biomarkers of effect as they do not necessarily correspond to an increased mutation risk. SCEs actually detect symmetrical or asymmetrical exchanges between sister chromatids of a single chromosome which are probably related to recombinational repair. The alkaline comet assay on the other hand detects single and double DNA breaks and alkali labile site that may or may not result in mutagenesis. Although both tests are well-known genotoxicity tests and hence related to carcinogenesis, the link with carcinogenesis in humans is no established yet. The tests, however, remain important.

Because of the association between genetic effects and cancer (at least in many instances), several studies were carried out on possible (cyto)genetic effects in subjects who were occupationally exposed to extremely low-frequency electromagnetic fields. Most of these studies showed increased genetic damage and hence overall the conclusion might be rather alarming. However, these studies need to be carefully examined. According to Gobba et al. (2003), no firm conclusions could be drawn yet with respect to possible ELF-induced genotoxicity in occupational exposed persons. This conclusion was amongst others based on the controversial data and lack of replication studies. We also noted the increased chromosomal aberrations in cable splicers (Skyberg et al. 1993), but when all the 13 employees of the study were compared with job-matched referents, no statistically significant differences were found. From the seven cable splicers, actually only three subjects were recently exposed and the other four had been on sick

leave and were transferred to other departments within the company. Statistically significant increases in chromosome breaks were found only in the three subjects which is a far too low study population to base any conclusion on, especially as smoking was also identified as a confounder. The more recent study of Skyberg et al. (2001) in welders was furthermore not able to find any increased cytogenetic damage. From the 24 exposed subjects, 12 were working in the high-voltage laboratory and 12 were employed in the generator soldering department where exposure was also to oil mist and vapours. Differences in response with regard to different genetic endpoints (e.g. increased levels of structural chromosome aberrations but not of micronuclei and sister chromatid exchanges; Valjus et al. 1993) may contribute to the confusion although this may, at least partly, be ascribed to other measured endpoints and hence other mechanisms of action. It is, for example, well known that ionising radiations produce structural chromosome aberrations but much less SCEs (Evans 1977). The same was seen with radiofrequency fields (Verschaeve et al. 2010), and this was apparently also confirmed in the investigations on ELF-MFs.

Other studies were performed since but final conclusions yet remain difficult to draw, for example as a result of other contradictory findings as shown by the study by Gadhia et al. (2010) in train engine drivers whose results were in contradiction with the findings of Nordenson et al. (2001). As a matter of fact, we identified a number of shortcoming or discussion points that may hinder a proper evaluation of ELF-EMF-induced genotoxicity in humans and explain the present lack of any clear answer with respect to genotoxic effects of ELF-EMF in humans:

- To start with, most studies were not accompanied by robust dosimetric evaluations (see Table 1). Often only a very superficial job description was given as the only estimate of a 'higher' exposure level compared with the control population (e.g. Bauchinger et al. 1981; Valjus et al. 1993; Nordenson et al. 1984, 1988; Higino Estécio and Silva 2002; Gadhia et al. 2010). When measurements of electric and/or magnetic fields were done, the overall exposure of involved subjects yet remain uncertain due to job variations (e.g. variable exposure durations, engine drivers switching from one engine to another, no information on 'other' potential exposures as for example from computer screens in subject supposed to be exposed to other ELF-EMF sources as the main exposure, etc.).
- Most of the studies mention the use of 'matched' control populations, but often it is not clear what this means. For example, they may be matched with respect to age, gender and life style, but other factors may be important as well but were largely ignored. Bauchinger et al. (1981) mentioned that control subjects had a similar occupation than the 380 kV switchyard workers, but it is not clear what this actually means. Carbonari et al. (2005) indicated that they used a protocol published by the International Commission for Protection against Environmental Mutagens and Carcinogens (Carrano 1988) to obtain necessary information on 'life styles and personal factors', but little is done with that information. In their study, exposure of video display workers was quantified as the number of working years (14.45 on the average) but apparently also the controls that they have designated as 'unexposed' had an average working time with video display monitors of 11.7 years. It is difficult then to understand in what both exposed and unexposed populations actually differed.
- Othman et al. (2001) supposedly investigated ELF-EMF-exposed subjects, but exposure was to EMF-fields from radar screens, antennae, satellite installations and closed circuit televisions. Exposure was therefore also, and essentially, to other forms of 'non-ionising radiations' (radiofrequencies). It is not clear from the paper what exposure was prevailing. As a matter of fact, all studies dating from later than the early 1990s should preferentially also consider exposure to radiofrequency electromagnetic fields as from mobile phones and other wireless communication devices, but no study actually did. This might be important as IARC also classified radiofrequency (RF) electromagnetic fields (as from mobile phones) into class 2B (possible carcinogenic to humans; IARC 2013), and the RF exposure might, at least in some of the studies, be more important than the ELF-MF exposures that were supposedly investigated. The study of Skyberg et al. (2001) also involved exposures to other agents than electromagnetic fields (mineral oil). The same holds true for the investigations on welders (Dominici et al. 2011; Villarini et al. 2015) and frequent users of photocopying machines (Goud et al. 2004). In welders, welding fumes were possible important confounders. Dominici et al. (2011) and Villarini et al. (2015) reported higher frequencies of micronucleated cells but lower frequencies of SCEs and DNA damage according to the comet assay. They highlighted the fact that reduced SCE frequencies were already reported as a result of exposure to chromium and/or nickel present in the welding fumes and may be explained by a reduced DNA repair capacity. The results in the comet assay were explained by different chromium and/or nickel (or other metals) exposure levels, which lead to DNA–protein cross-links at lower concentrations. Goud et al. (2004) showed increased micronucleus levels in white blood cells and buccal cells of frequent users of photocopying machines, but exposure was also to toxic

VOCs and other compounds. Smoking could also be an important confounder.

- The study of Celikler et al. (2009) and Balamuralikrishnan et al. (2012) involved power line and transformer workers. Although both studies reported higher frequencies of chromosomal aberrations and micronuclei, there are reasons for concern. For example, especially the Indian study reported very low micronucleus frequencies compared with historical values in most if not all of the laboratories worldwide. Frequencies of 1.32 ± 1.12 and 1.18 ± 0.73 per 1000 cells were found in exposed subjects compared with 0.45 ± 0.60 per 1000 cells in the controls. Even the frequencies in the exposed population were much lower than what is normally reported in unexposed control cells. Bonassi et al. (2001), for example, reported an overall median micronucleus frequency in non-exposed (i.e. normal) subjects of 6.5 per thousand and an interquartile range between 3 and 12 per thousand. These values were based on a database of nearly 7000 subjects. Another example is provided by Rastkhah et al. (2016) who reported from 6 to 21 micronuclei per 1000 binucleated cells as the average baseline frequency. There are numerous other examples in the scientific literature.
- The study of Tiwari et al. (2015) only reported a tendency to higher DNA damage levels in substation workers reflecting over interpretation of the data rather than a real effect.
- The value of cytogenetic biomonitoring studies is, amongst others, largely dependent on two important parameters, i.e. the number of investigated cells per person and the number of individuals that were investigated in as well the test population as their controls. The requested number of cells can be calculated with statistical tools. Statistical methods have demonstrated that, in order to detect a doubled chromosome aberration frequency in a human biomonitoring study, one should investigate at least 200 metaphase figures per person and at least 20 persons per group (Whorton et al. 1979; Whorton 1985). This holds true only if confounders can be maximally excluded (no smokers or drug users, no medication or chronic diseases, same age distribution between the groups, no expected exposure to other potential mutagens, etc.). If confounders cannot be sufficiently excluded, it is necessary to increase the sample size (cell number and/or number of individuals). Calculations of the number of cells and individuals that are needed in a cytogenetic study are, however, seldom done, and often a compromise is adopted between what is considered feasible in terms of time and work load and what is yet supposed to be enough. It is nevertheless assumed that one should at least investigate 200–500 metaphase figures per sample. That also the number of involved persons is important is obvious. Not all persons react in the same way, and a representative sample of the population is needed (Verschaeve 2015). Scientists do not completely agree on the number of cells and subjects that should be investigated in order to conduct a well-designed and statistically robust cytogenetic biomonitoring study, but generally speaking the numbers of 200 cells for chromosome aberrations, 50 for SCEs, 100 for analysis of 'DNA comets' and 2000 for analysis of micronuclei are considered to be minimal requirements, together with 20–50 subjects in both the test population and control group. It is clear that in the above-mentioned studies (Table 1) these numbers were not always achieved. Many studies therefore provided results that were statistically not sufficiently robust.
- Many of the above reported studies which showed cytogenetic damage in the peripheral blood lymphocytes or buccal cells of exposed subjects concern exposure levels which may be assumed higher than those of the 'non-professionally exposed controls', but exposure levels were yet usually not very high. Exposure levels were in many cases probably much lower than the exposure levels that were applied in in vitro and in vivo investigations. These experimental studies nevertheless largely produced negative findings. The same holds true for the study of Albert et al. (2009) where an exposure to 200 μT magnetic flux densities also did not induce genetic effects. Here one may argue yet that the exposure was limited in time (4 h only). According to a WHO report (EHC 2007), studies of the effects of ELF magnetic fields on cells have generally shown no induction of genotoxicity at fields below 50 mT, although some more recent data show effects at 35 μT. According to SCENIHR (2015), positive effects may be expected above approximately 100 μT. Whatever the real value is, these exposure levels are still considerably higher than the alleged exposure levels in most of the professionally exposed subject investigated in the above-mentioned cytogenetic biomonitoring studies. It is therefore difficult to believe that all reported cytogenetic effects are really due to the ELF-MF, rather than to other factors, as for example, exposure to other (genotoxic) agents, methodological shortcomings resulting in for example poor statistical power, over interpretation of data or, sometimes even bad science.

Above considerations show that there are many shortcomings and reasons to minimise the scope of the findings. However, there yet is the fact that only five out of 22 studies (23 %) did not show cytogenetic damage in the investigated ELF-EMF-exposed subjects, and hence, this still is reason for concern. The evaluation of the investigations does not mean that exposures to extremely low-frequency magnetic

fields are cleared from any suspicion and that no protective measures need to be taken by authorities in order to reasonably apply the precautionary principle. Indeed, no consistent evidence of harm does not equal evidence of no harm, and we may not expect totally consistent results from scientific research when such a complex matter is concerned.

Conclusion

According to above investigations presenting a number of shortcomings and contradictions between the study results, no firm conclusion can be drawn with respect to alleged ELF-EMF induced genetic effects in exposed subjects. We still should be alert as some indications of induced genetic effects and carcinogenesis cannot be simply disregarded. Cytogenetic biomonitoring studies that were conducted so far did have important shortcomings. For this reason, we believe that more thorough and better controlled investigations using the right genetic endpoints on adequate numbers of cells and individuals still should be envisaged.

Acknowledgments This literature review was conducted as part of our activities within the Belgian BioElectroMagnetics Group (BBEMG).

References

Albert GC, McNamee JP, Marro L, Bellier PV, Prato FS, Thomas AW (2009) Assessment of genetic damage in peripheral blood of human volunteers exposed (whole-body) to a 200 muT, 60 Hz magnetic field. Int J Radiat Biol 85:144–152

Andreassi MG, Barale R, Iozzo P, Picano E (2011) The association of micronucleus frequency with obesity, diabetes and cardiovascular disease. Mutagenesis 26:77–83

Balamuralikrishnan B, Balachandar V, Kumar SS, Stalin N, Varsha P, Devi SM, Arun M, Manikantan P, Venkatesan C, Sasikala K, Dharwadjar SN (2012) Evaluation of chromosomal alterations in electric workers occupationally exposed to low frequency of electromagnetic fields (EMFs) in Coimbatore population, India. Asian Pac J Cancer Prev 13:2961–2966

Bauchinger M, Hauf R, Schmid E, Dresp J (1981) Analysis of structural chromosome changes and SCE after occupational long-term exposure to electric and magnetic fields from 380 kV-systems. Radiat Environ Biophys 19:235–238

Bergqvist U, Brix J, de Gruijl F, de Seze R,Hietanen M, Jeffereys JGR, Lagroye I, Lotz GW, Owen RD, Repacholi MH, Saunders R, Tenforde TS, Verschaeve L, Veyret B (2003) Review of experimental investigations of EMF biological effects (0-100 kHz)—ICNIRP Standing committee II. In: Matthes R, McKinley A, Bernhardt J, Vecchia P, Veyret B (eds) Exposure to static and low frequency electromagnetic fields, biological effects and health consequences. ICNIRP13/2003, ISBN 3-934994-03-2

Bonassi S, Abbondandolo A, Camurri L, Dal PL, De FM, Degrassi F, Forni A, Lamberti L, Lando C, Padovani P (1995) Are chromosome aberrations in circulating lymphocytes predictive of future cancer onset in humans? Preliminary results of an Italian cohort study. Cancer Genet Cytogenet 79:133–135

Bonassi S, Hagmar L, Stromberg U, Montagud AH, Tinnerberg H, Forni A, Heikkila P, Wanders S, Wilhardt P, Hansteen IL, Knudsen LE, Norppa H (2000) Chromosomal aberrations in lymphocytes predict human cancer independently of exposure to carcinogens. European Study Group on Cytogenetic Biomarkers and Health. Cancer Res 60:1619–1625

Bonassi S, Fenech M, Lando C, Lin YP, Ceppi M, Chang WP, Holland N, Kirsch-Volders M, Zeiger E, Ban S, Barale R, Bigatti MP, Bolognesi C, Jia C, Di Giorgio M, Ferguson LR, Fucic A, Lima OG, Hrelia P, Krishnaja AP, Lee TK, Migliore L, Mikhalevich L, Mirkova E, Mosesso P, Müller WU, Odagiri Y, Scarffi MR, Szabova E, Vorobtsova I, Vral A, Zijno A (2001) HUman MicroNucleus project: international database comparison for results with the cytokinesis-block micronucleus assay in human lymphocytes: I. Effect of laboratory protocol, scoring criteria, and host factors on the frequency of micronuclei. Environ Mol Mutagen 37:31–45

Bonassi S, Znaor A, Ceppi M, Lando C, Chang WP, Holland N, Kirsch-Volders M, Zeiger E, Ban S, Barale R, Bigatti MP, Bolognesi C, Cebulska-Wasilewska A, Fabianova E, Fucic A, Hagmar L, Joksic G, Martelli A, Migliore L, Mirkova E, Scarfi MR, Zijno A, Norppa H, Fenech M (2007) An increased micronucleus frequency in peripheral blood lymphocytes predicts the risk of cancer in humans. Carcinogenesis 28:625–631

Bonassi S, Norppa H, Ceppi M, Stromberg U, Vermeulen R, Znaor A, Cebulska-Wasilewska A, Fabianova E, Fucic A, Gundy S, Hansteen IL, Knudsen LE, Lazutka J, Rossner P, Sram RJ, Boffetta P (2008) Chromosomal aberration frequency in lymphocytes predicts the risk of cancer: results from a pooled cohort study of 22 358 subjects in 11 countries. Carcinogenesis 29:1178–1183

Bonassi S, El-Zein R, Bolognesi C, Fenech M (2011) Micronuclei frequency in peripheral blood lymphocytes and cancer risk: evidence from human studies. Mutagenesis 26:93–100

Carbonari K, Gonçalves L, Roth D, Moreira P, Fernández R, Martino-Roth MdG (2005) Increased micronucleated cell frequency related to exposure to radiation emitted by computer cathode ray tube video display monitors. Genet Mol Biol 28:469–474

Carrano AV (1988) Considerations for population monitoring using cytogenetic techniques. ICPEMC—International Commission For Protection Against Environmental Mutagens and Carcinogens. Publication N. 14. Mutat Res 204:379–406

Celikler S, Aydemir N, Vatan O, Kurtuldu S, Bilaloglu R (2009) A biomonitoring study of genotoxic risk to workers of transformers and distribution line stations. Int J Environ Hyg Health Res 19:421–430

Dominici L, Villarini M, Fatigoni C, Monarca S, Moretti M (2011) Genotoxic hazard evaluation in welders occupationally exposed to extremely low-frequency magnetic fields (ELF-MF). Int J Hyg Environ Health 215:68–75

EHC (2007) Environmental health criteria 238: extremely low frequency fields. World Health Organization, Geneva. ISBN 978-92-4-157238-5

Erdal ME, Erdal N, Oğuzkan S (1999) Chromosomal aberrations in peripheral lymphocytes of a high-voltage power lineman exposed to electromagnetic fields. Turk J Med Sci 29:335–336

Evans HJ (1977) Molecular mechanisms in the induction of chromosome aberrations. In: Scott D, Bridges BA, Sobels FH (eds) Progress in genetic toxicology. Elsevier/North Holland Biomedical Press, Amsterdam, pp 57–74. ISBN 0-444-80014-X

Gadhia P, Chakraborty S, Pithawala M (2010) Cytogenetic studies on railway engine drivers exposed to extremely low frequency electromagnetic fields (ELF-EMF). Int J Hum Genet 10:263–269

Gobba F, Rocatto L, Sinigaglia B, Temperani P (2003) Sister chromatid exchanges (SCE) and high frequency cells in workers occupationally exposed to extremely low frequency magnetic fields. Med Lav 94:450–458 **(in Italian)**

Goud KI, Hasan Q, Balakrishna N, Rao KP, Ahuja YR (2004) Genotoxicity evaluation of individuals working with photocopying machines. Mutat Res 563:151–158

Hagmar L, Bonassi S, Stromberg U, Brøgge A, Knudsen LE, Norppa H, Reuterwall C (1998) Chromosomal aberrations in lymphocytes predict human cancer: a report from the European Study Group on Cytogenetic Biomarkers and Health (ESCH). Cancer Res 58:4117–4121

Hagmar L, Stromberg U, Bonassi S, Hansteen IL, Knudsen LE, Lindholm C, Norppa H (2004) Impact of types of lymphocyte chromosomal aberrations on human cancer risk: results from Nordic and Italian cohorts. Cancer Res 64:2258–2263

Higino Estécio MR, Silva AE (2002) Chromosome abnormalities caused by computer video display monitors' radiation. Rev Saúde Pública 36:330–336 **(in Portuguese)**

IARC (2002) IARC monographs on the evaluation of carcinogenic risks to humans. Vol. 80, non-ionizing radiation, part 1: static and extremely low-frequency (ELF) electric and magnetic fields. IARC Press, Lyon. ISBN 92-832-1280-0

IARC (2013) IARC monographs on the evaluation of carcinogenic risks to humans. Vol. 102, non- ionizing radiation, part 2: radiofrequency electromagnetic fields. IARC Press, Lyon. ISBN 978-92-832-1325-3

Khalil AM, Quassem W, Amoura F (1993) Cytogenetic changes in human lymphocytes from workers occupationally exposed to high-voltage electromagnetic fields. Electro Magnetobiol 12:17–26

Leitgeb N (2015a) Synoptic analysis of epidemiologic evidence of glioma risk from mobile phones. J Electromagn Anal 7:233–243

Leitgeb N (2015b) Synoptic analysis clarifies childhood leukemia risk from ELF magnetic field exposure. J Electromagn Anal 7:245–258

Maes A (1998) Genetic effects of non-ionizing radiations, especially microwaves and extremely low frequency fields. PhD thesis, Free University of Brussels (VUB), Brussels (**in Dutch**)

Maes A, Anthonissen R, Wambacq S, Simons K, Verschaeve L (2016) The cytome assay as a tool to investigate the possible association between exposure to extreme low frequency magnetic fields and an increased risk for Alzheimer's disease. J Alzheimer's Dis 50:741–749

Markkanen A (2009) Effects of electromagnetic fields on cellular responses to agents causing oxidative stress and DNA damage. Kuopio University Publications C. Natural and Environmental Sciences vol 253, 59 p. ISBN 978-951-27-1191-8

Mateuca R, Lombaert N, Aka PV, Decordier I, Kirsch-Volders M (2006) Chromosomal changes: induction, detection methods and applicability in human biomonitoring. Biochimie 88:1515–1531

Migliore L, Coppedè F, Fenech M, Thomas P (2011) Association of micronucleus frequency with neurodegenerative diseases. Mutagenesis 26:85–92

Murgia E, Ballardin M, Bonassi S, Rossi AM, Barale R (2008) Validation of micronuclei frequency in peripheral blood lymphocytes as early cancer risk biomarker in a nested case-control study. Mutat Res 639:27–34

Nordenson I, Hansson Mild K, Nordström S, Sweins A, Birke E (1984) Clastogenic effects in human lymphocytes of power frequency electric fields: in vivo and in vitro studies. Radiat Environ Biophys 23:191–201

Nordenson I, Hansson Mild K, Ostman U, Ljungberg H (1988) Chromosomal effects in lymphocytes of 400 kV-substation workers. Radiat Environ Biophys 27:39–47

Nordenson I, Hansson Mild K, Järventaus H, Hirvonen A, Sandström M, Wilén J, Blix N, Norppa H (2001) Chromosome aberrations in peripheral lymphocytes of train engine drivers. Bioelectromagnetics 22:306–315

Othman EO, Aly MS, El Nahas SM (2001) Aneuploidy in workers occupationally exposed to electromagnetic fields detected by FISH. Cytologia 66:117–125

Rastkhah E, Zakeri F, Ghoranneviss M, Rajabpour MR, Farshidpour MR, Mianji F, Bayat M (2016) The cytokinesis-blocked micronucleus assay: dose–response calibration curve, background frequency in the population and dose estimation. Radiat Environ Biophys 55:41–51

Scaringi M, Temperani P, Rossi P, Bravo G, Gobba F (2007) Evaluation of the genotoxicity of the extremely low frequency-magnetic fields (ELF-MF) in workers exposed for professional reasons. Giorn It Med Lavoro Ergon 29:420–421 **(in Italian)**

SCENIHR (2015) Opinion on Potential health effects of exposure to electromagnetic fields (EMF) European Commission, DG Health and Food Safety. http://ec.europa.eu/health/scientific_committees/emerging/docs/scenihr_o_041.pdf

Schmiedel S, Blettner M (2010) The association between extremely low-frequency electromagnetic fields and childhood leukaemia in epidemiology: enough is enough? Br J Cancer 103:931–932

Skyberg K, Hansteen I-L, Vistnes AM (1993) Chromosome aberrations in lymphocytes of high-voltage cable splicers exposed to electromagnetic fields. Scand J Work Environ Health 19:29–34

Skyberg K, Hansteen I-L, Vistnes AM (2001) Chromosomal aberrations in lymphocytes of employees in transformer and generator production exposed to electromagnetic fields and mineral oil. Bioelectromagnetics 22:150–160

Tiwari R, Lakshmi NK, Bhargava SC, Ahuja YR (2015) Epinephrine, DNA integrity and oxidative stress in workers exposed to extremely low-frequency electromagnetic fields (ELF-EMFs) at 132 kV substations. Electromagn Biol Med 34:56–62

Udroiu I, Cristaldi M, Icradi LA (2006) Clastogenicity and aneuploidy in newborn and adult mice exposed to 50 Hz magnetic fields. Int J Radiat Biol 82:561–567

Udroiu I, Giuliani L, Icradi LA (2010) Genotoxic properties of extremely low frequency electromagnetic fields. Eur J Oncol Libr 5:123–134

Valjus J, Norppa H, Järventaus H, Sorsa M, Nykyri E, Salomaa S, Järvinen P, Kjader J (1993) Analysis of chromosomal aberrations, sister chromatid exchanges and micronuclei among power linesmen with long-term exposure to 50-Hz electromagnetic fields. Radiat Environ Biophys 32:325–336

Verschaeve L (2009) Genetic damage in subjects exposed to radiofrequency radiation. Mut Res 681:259–270

Verschaeve L (2015) Genetic toxicology: tests and applications. University Press, Antwerp. ISBN 978-90-5718-283-9

Verschaeve L, Juutilainen J, Lagroye I, Miyakoshi J, van Rongen E, Saunders R, de Seze R, Tenforde T, Veyret B, Xu Z (2010) In vitro and in vivo genotoxicity of radiofrequency fields. Mut Res 705:252–268

Vijayalaxmi, Obe G (2005) Controversial cytogenetic observations in mammalian somatic cells exposed to extremely low frequency electromagnetic radiation: a review and future research recommendations. Bioelectromagnetics 26:412–430

Villarini M, Dominici L, Fatigoni C, Levorato S, Vannini S, Monarca S, Moretti M (2015) Primary DNA damage in welders occupationally exposed to extremely-low-frequency magnetic fields (ELF-MF). Ann Ig 27:511–519

Whorton EB Jr (1985) Some experimental design and analysis considerations for cytogenetics studies. Environ Mutagen 7(Suppl 4):9–15

Whorton EB Jr, Bee DE, Kilian DJ (1979) Variations in the proportion of abnormal cells and required sample sizes for human cytogenetic studies. Mutat Res 64:79–86
