# Supplementary material for: Magnetic Fields and Cancer: Epidemiology, Cellular Biology, and Theranostics
Source: Int J Mol Sci. 2022 Jan 25;23(3):1339. doi: 10.3390/ijms23031339 (PMC8835851; doi:10.3390/ijms23031339)
Supplement: Supplementary file 1 [file ijms-23-01339-s001.zip › Supplementary Data Set S1/MF and Cancer.Data/PDF/3163656974/1048291118758460.pdf]

Check for updates

NEW SOLUTIONS: A Journal of Environmental and Occupational Health Policy
2018, Vol. 28(1) 55–78
© The Author(s) 2018
Reprints and permissions: sagepub.com/journalsPermissions.nav
DOI: 10.1177/1048291118758460
journals.sagepub.com/home/new
SAGE

Work and Female Breast Cancer: The State of the Evidence, 2002–2017

Connie L. Engel[1], M. Sharima Rasanayagam[1], Janet M. Gray[1,2], and Jeanne Rizzo[1]

Abstract

The authors undertook a scoping review to assess the literature from 2002 to 2017 on the relationship between occupation and female breast cancer. Case–control, cohort, and meta-analytic studies suggest that women working as flight attendants, in medical professions, some production positions, sales and retail, and scientific technical staff are likely to have elevated risk of breast cancer. In addition, occupational exposures to night-shift work, ionizing radiation, some chemicals, job stress, and sedentary work may increase risk of breast cancer. Occupational physical activity appears to decrease risk. Workplace exposures to passive smoke and occupational exposure to nonionizing radiation do not appear to affect breast cancer risk. Some studies of occupational categories and workplace exposures indicate that risk may be modified by duration of exposure, timing of exposure, dose, hormone-receptor subtypes, and menopausal status at diagnosis. The compelling data from this review reveal a substantial need for further research on occupation and breast cancer.

Keywords

breast cancer, occupation, women's health, research gaps, exposures, environmental health

[1]Breast Cancer Prevention Partners, San Francisco, CA, USA
[2]Vassar College, Poughkeepsie, NY, USA

Corresponding Author:
Connie L. Engel, Breast Cancer Prevention Partners, 1388 Sutter Street, Suite 400, San Francisco, CA 94109, USA.
Email: connie@bcpp.org

Introduction

Breast cancer is the most common cancer among women, and the second highest cause of cancer deaths in the United States, after lung cancer.[1] In 2018, an estimated 266,120 new cases of breast cancer will be diagnosed in women in the United States.[1] Breast cancer in men is relatively uncommon, with the incidence rates about 1 percent of the incidence for females.[2] Incidence and mortality vary substantially by race and ethnicity. Furthermore, distinct subtypes of breast cancer, estrogen-receptor positive/progesterone-receptor positive (ER+/PR+, also referred to as Luminal A and B), human epidermal growth factor receptor 2 positive (HER2+), and basal subtypes respond to different treatments, have different prognoses, and affect different groups.[3] Basal breast cancer, also called triple negative, is the most difficult to treat, has a less positive prognosis, and occurs more often in younger women,[4] especially young African-American[5] and Latina women.[6]

This article explores the state of the evidence linking women's occupation and workplace exposures to breast cancer. We begin with a discussion of women's presence in the work force and 2015 research that found a paucity of data on women, work, and cancer. We then describe the method of our scoping review. This is followed by substantive discussions of our findings on occupations and breast cancer and workplace exposures and breast cancer. Detailed data from our review are provided in the supplemental materials. A second paper, also in this volume, reviews the gaps in the literature and provides recommendations for future research.

Because of the dramatically higher level of breast cancer among women, we focus our review on occupation and breast cancer in women. With that in mind, there is some evidence that men who serve as first responders—as policemen,[7] firefighters,[8] and members of the armed forces[9]—may have elevated rates of breast cancer. A case–control study in France also found increased risk of male breast cancer among auto mechanics and men who work with alkylphenols.[10] The rarity of male breast cancer means these findings deserve careful follow-up.

According to the U.S. Department of Labor, 53.7 percent of women over the age of 16 participate in the U.S. labor market, and the majority of women work full-time.[11,12] Over time, association between job status and gender has shifted, with a growing presence of women in management and professional fields. However, over the last four decades, women continue to be more highly represented in people-oriented jobs than jobs dealing primarily with inanimate objects and mechanical systems, regardless of job status levels.[13]

Occupational cohorts such as miners, production workers, and others have functioned for decades as "canaries in the coal mine" for identifying carcinogenic agents. These workers bear a disproportionate cancer burden while acting

as sentinels for the rest of society.[14] Occupational contributions to risk of female breast cancer are critically understudied, although the percentage of occupational cancer studies that include women has increased in recent years.[15] Based upon a review of the literature from 1991 to 2009, 50 percent of studies still include only men, while only 9.1 percent of studies focus exclusively on women. The remaining 41 percent of studies include both. For breast cancer specifically—a disease that is a hundred times more likely in women than in men—13.4 percent of studies included only men. The studies that included only women (27.4 percent) provided fewer risk estimates than those studies exploring men only.[15]

Association of a given occupation with breast cancer risk may be a result of other related factors, such as reproductive history, physical activity, alcohol use, smoking, or socioeconomic status. Over time, more studies have accounted for social and reproductive factors that are likely to correlate with occupation and confound results; nevertheless, few studies stratify results by race/ethnicity, tumor subtype, and menopausal status. The studies reporting breast cancer risk by occupational status vary in how they address these potential covariates. These gaps are explored more fully in our second paper.

Within any occupational category, work activities, work shift patterns, and exposures to chemical and physical agents may vary notably. For instance, nurses can be exposed to solvents, endocrine disruptors in cleaning products and medical devices, antimicrobials and sterilizing agents, ionizing radiation, chemotherapy drugs, and night-shift work, all of which have been linked to breast cancer. Flight attendants also experience night-shift work and circadian disruption,[16] alongside exposures to flame retardants in airplanes,[17] and combustion products from jet fuel.

Cancer may not be linked to a single agent that overwhelms the system.[18] It may, instead, emerge when the multiple checks and balances that keep the body in a state of health and equilibrium are disrupted by different events and exposures that collectively knock out the body's various protections. This means that the multiple exposures encountered in any occupation could act in concert to lead to an elevated risk for breast and other cancers.

Methods

We undertook a scoping review of occupation and breast cancer to examine the extent and rigor of the research on breast cancer and occupation. Our aim was to identify occupations and workplace exposures that may convey increased risk of breast cancer, occupations, and exposures with inconclusive findings that require additional research and methodological needs for future studies. Scoping studies were first described by Arksey and O'Malley in 2005 as a rigorous approach to map the literature in a field of study.[19] Scoping reviews are especially relevant for complex areas or research where different research methods

Table 1. Search Terms for Specific Occupations and Exposures.

Accountants	Hairdressers	Printing
Agriculture	Cosmetologists	Production workers
Benzene	Journalists	Professionals
Chemical production	Lab tech	Radiological technicians
Clothing	Lawyers	Retail
Dry cleaning	Librarians	Rubber
Factory workers	Military	Solvent exposure
Flight attendants	Nurses	Teachers
Firefighters	Plastic	Textile
	Police	

may apply. The approach offers a systematic, rigorous, transparent, and comprehensive approach to review the literature. Several authors have further refined the methodology and application of scoping reviews.[20–23]

We searched PubMed to identify potential studies for inclusion in a review of the literature linking work in specific fields and occupational exposures to breast cancer incidence. Search terms were identified though scans of the literature, discussions within a nine-month study group series on occupation and breast cancer, and keywords from studies from a prior white paper review on this topic.[24] Search terms included "breast cancer" or "breast neoplasm" and "job," "work," "occupation," and "workplace." We also searched for breast cancer incidence and twenty-seven occupations or job titles or exposures identified in our earlier review and from job categories (see Table 1).

Our initial search yielded 11,204 articles. We subsequently limited our review to articles published after 2002, which reduced the total number to 8742 articles. We reviewed the titles of these articles to assess potential relevance for our review, which left us with 475 articles for screening. Two reviewers read abstracts for each of the 475 articles for the review. To be included, articles needed to evaluate breast cancer incidence and occupational roles or exposures and report study results (generally as risk ratios, odds ratios (ORs), or standardized incidence ratios). We included studies with null findings. We excluded studies that reported risk of breast cancer mortality but did not provide data on breast cancer incidence. We also excluded papers that were not available in English. If the two initial reviewers disagreed, a third reviewer served as the tie breaker. After the review of the first 125 articles, reviewers looked at discordant recommendations for inclusion and discussed why they included or excluded articles. We did not change our initial recommendations; all discordant recommendations still went to Reviewer 3. After Reviewer 3 offered the tie-breaking vote, the entire team reviewed their recommendations. In this way, we clarified which studies met those criteria.

In most cases, abstracts provided data adequate to assess inclusion or exclusion. For sixteen articles, it was unclear whether studies met criteria for inclusion from the abstract alone. In these cases, the entire team reviewed the full article to ensure each study included data relevant to the review. Eight of these studies were included.

The screening process yielded a total of 182 articles, published between 2002 and 2017, reporting data on breast cancer and occupation and workplace exposures (see Figure 1). Papers were read and data were tabulated by occupation or workplace exposure (see Supplement 1). In this process, forty papers were excluded (see Supplement 2), because they did not meet the criteria, yielding a final tally of 142 articles included in the review (see Supplement 3).

In keeping with Arksey and O'Malley's[19] steps for the literature review, we also charted the data. We approached the 142 selected studies in two ways. First, we tabulated relevant aspects of study design for each report. This included publication details, study design, key occupations or exposures studied, the inclusion of breast cancer risk factors as covariates (reproductive history, genetic and other biological aspects, family history, physical activity, smoking, and alcohol use), interactions with other exposures or occupational histories, the richness of job history and job role data, community-level exposures, and the inclusion of workers in the research design and process. These data are thoroughly described in the second paper, and the table is included in the Supplemental materials for that paper.

Second, we tabulated the relevant risk and hazard estimates from each study, by occupation or workplace exposure. Many studies included multiple data points. We established the following guidelines for which data to enter: (1) When studies reported data adjusted for confounders or covariates as well as unadjusted data, we included adjusted data; (2) In studies focused on specific occupations or exposures, we included null findings as well as results that indicated elevated risk and in a few cases, reduced risk; (3) Where data were reported for subgroups, we reported on subgroups with elevated risk and their comparison; and (4) For studies that reported on exposures within a specific occupation, we entered the data as an exposures study.

For a subset of studies that reported on breast cancer risk for multiple occupational categories, we took a slightly different approach. Three of the coauthors individually read each of these articles. We held a conference to discuss and make recommendations on the data and/or criteria for data entry for each article. In these cases, we tabulated occupations with risk elevations and confidence intervals above one that met a minimum sample size based upon the studies' total sample. We also tabulated null findings from the studies of multiple occupations if data on that occupation were already included from studies of single occupations. We did not include data from records-linkage studies that reported on multiple occupations if the study did not

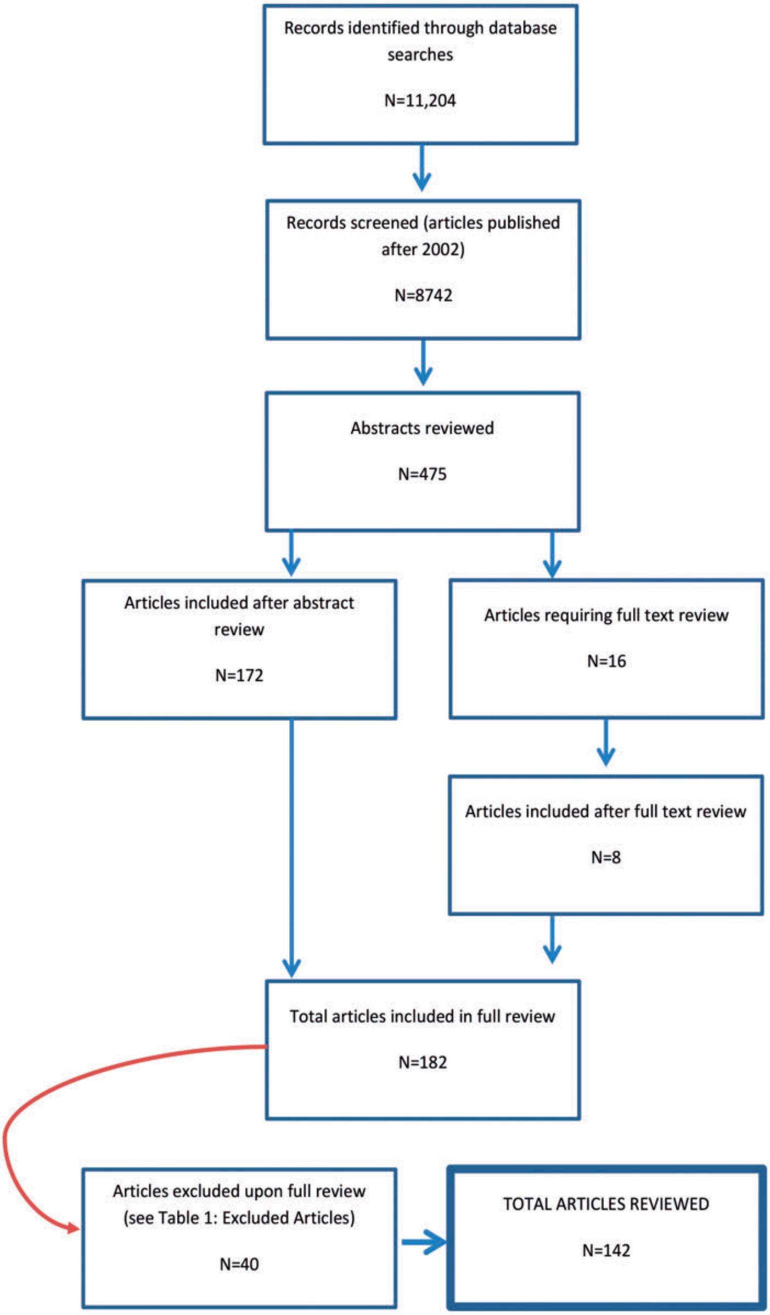

Figure 1. Illustration of the literature search and article review process for the scoping review. We screened the titles of 8742 articles found through the literature searches described in Table 1. This yielded 475 potentially relevant articles. Two individuals read 475 abstracts, with a third breaking the tie in cases of disagreement. After full screening, we included 142 articles in the review.

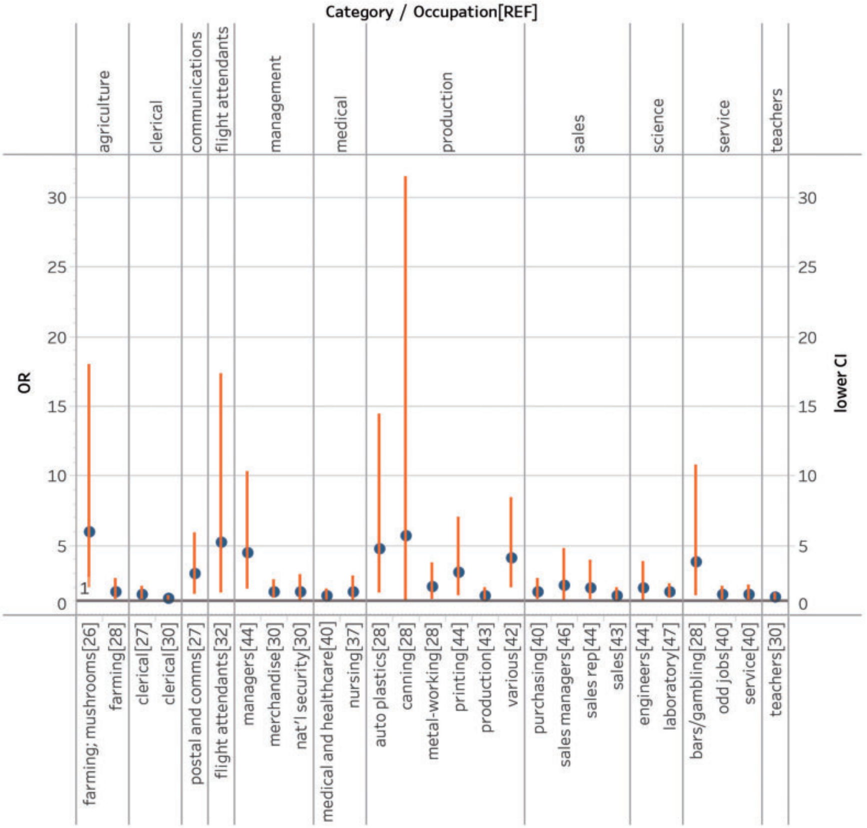

Figure 2. Occupation and breast cancer, odds ratios. Circles represent odds ratios reported for workplace exposure categories, with lines showing the 95% confidence intervals. All tabled data were significantly different from OR=1. Data for other statistics (SIR, RR, and HR) and null findings are reported in Supplement 1.

adjust for other breast cancer risk factors. Unless otherwise noted, results reported in the following sections are significantly elevated.

Results for Occupation

We report data on occupational categories using the U.S. Department of Labor Standard Occupational Categories.[25] Included studies relied on different occupational coding schemes. However, the broad categories overlapped considerably. Figure 2 illustrates occupational categories with statistically significant odds ratios. Complete data from our review, including other statistics and

null findings, are presented in Supplement 1. Our discussion will focus on key findings and questions for future research.

Agriculture

Overall, data do not support a clear link between work in farming and agriculture and breast cancer. One study found a sixfold increase in breast cancer among women who farmed mushrooms (OR 6.02; 95% CI: 2.01–18.00).[26] Another study found a twofold increased risk among women who worked as farmers for more than ten years (OR 2.08; 95% CI: 1.15–3.74)[27]; and yet another study found elevated risk for ER-breast cancer (OR 1.71; 95% CI: 1.12–2.62).[28] Brophy et al.[28] also found that work in agriculture may interact with occupations later in life to magnify risk. Studies of agricultural work, in particular, need to consider physical activity as a potential covariate.

Teachers

Despite long-standing reports that teachers have elevated rates of breast cancer, the literature from the past fifteen years does not consistently support this conclusion. A 2002 report from the California Teachers' Study found a 50 percent increased risk of breast cancer among a cohort of teachers, compared to age-adjusted cancer-registry data (RR 1.51; 95% CI: 1.48–1.54).[29] However, that study did not control for breast cancer risk factors like reproductive history. More recent case–control studies have not found increased risk. For, example, a 2003 study of women aged twenty to forty-four years found elevated risk among female teachers, but only among parous women (OR 1.3; 95% CI: 1.0–1.7, parous women; OR 1.00; 95% CI: .6–1.5, nulliparous women).[30]

Flight Attendants

Flight attendants are one of the most extensively researched occupational groups. Studies from around the world have consistently found elevated risk of breast cancer, with most studies reporting 37 to 50 percent increased risk. A 2016 meta-analysis found a 40 percent increased risk (OR = 1.40, 95% CI = 1.30–1.50).[31] Some studies have begun to explore duration of employment,[32] cumulative radiation dose,[33] and degree of sleep disruption[34] as modifiers of risk. Thus far, these results are inconsistent, although the duration of employment may be one important variable. Flight attendants experience multiple exposures that can increase breast cancer risk, including night-shift work and other circadian rhythm disruption,[16] exposure to ionizing radiation above background levels,[33] and chemicals such as flame retardants[17] and pesticides.

Medical Professions

Nurses are frequently cited as an occupational group at higher risk for breast cancer. The more recent data, however, are mixed. Most studies we reviewed found risk estimates slightly above one; but few of them were statistically significant. Two cohort studies found elevated risk in the 10 to 14 percent range: SIR 1.1; 95% CI: 1.1–1.2[35] and SIR 1.14; 95% CI: 1.09–1.19.[36] Longer duration of employment and work in a hospital setting may confer slightly higher elevated risk (OR 1.7; 95% CI: 1.04–2.79).[37] Since nursing is a broad profession, with work in many different settings and with likely exposure to different agents (e.g., chemotherapeutic agents, ionizing radiation), depending upon work setting, future studies need to look more closely at subspecialties among nurses.

Studies of other medical personnel, including physicians, suggest possible risk elevations for some medical workers. One study found almost a threefold risk for orthopedic surgeons (SPR 2.9; 95% CI: 1.66–4.71),[38] a group that may be exposed to high levels of radiation during procedures. Other studies found double the risk among physicians (SIR 2.03; 95% CI 1.62–2.51)[39] and 40 percent increased risk among medical and healthcare personnel (OR 1.4; 94% CI, 1.00–1.90).[40] Most of the research on radiological technicians is based upon a large U.S. cohort that spans the early years of radiation use in medical settings, and most of the reports focus on mortality. Only two studies reported on breast cancer incidence in this occupation. Overall, contemporary radiological technicians do not seem to face elevated breast cancer risk, but those who started work before 1940 had double breast cancer risk (RR 2.00; 95% CI 1.1–3.4).[41]

Production Work

Production work can be divided into multiple subcategories, based upon the material produced. Research does not support elevated breast cancer risk for workers who produce electronics, although the data may not reflect the computers currently in wide use. For other materials, results vary. In many cases, breast cancer risk is elevated for subgroups, but not necessarily for an entire study sample. For instance, metalworking doubles risk for ER+/PR+ breast cancer (OR 2.03; 95% CI: 1.11–3.71), but not for other subtypes (overall OR 1.71; 95% CI .99–2.95).[28] Women in both auto plastics and food canning have an increased risk of breast cancer, but this increase is more extreme for premenopausal women (about a fivefold risk; OR 5.7; 95% CI: 1.03–31.5), and double for postmenopausal women (OR 2.35; 95% CI: 1.00–5.53).[28] Study findings are inconsistent regarding breast cancer risk for women working in manufacturing and machinery,[40,42,43] printing and paper production,[28,44] and textiles.[42,44,45]

Sales and Related Occupations

Risk for breast cancer was elevated in workers in retail, sales, and merchandise in all the studies we reviewed. Risk elevations ranged from a 42 percent increase (OR 1.42; 95% CI 1.00–2.00)[43] to more than double the risk (OR 2.2; 95% CI 1.00–4.80).[46] All the studies were adjusted for reproductive risk factors and most for family history, which further underscores the need for more extensive research on workers in these fields.

Science Occupations

Work in science occupations, such as in laboratory settings[47] and engineering[44] may increase breast cancer risk. This area requires further study, since only three articles reported on breast cancer risk among women working in nonmedical scientific fields.

Hairdressers and Cosmetologists

A 2009 meta-analysis found a modest (about 10 percent) increase in breast cancer risk among hairdressers and cosmetologists, when they analyzed case–control studies only (OR 1.1; 95% CI: 1.05–1.16).[48] Other studies have not found evidence for elevated risk (in fact, one study found reduced risk; OR .85; 95% CI: .82–.89).[49]

Occupational Exposures and Breast Cancer

Some of the strongest evidence for concerns about occupational health risks emerges from studies that have examined the potential links between specific occupational exposures and breast cancer risk. Most exposure studies tend to focus on single (or a few) exposures, despite worker exposures to mixtures at the work site and multiple chemicals across the day. Studies that include exposure estimates used methods including worker self-report, routine badge monitoring (especially common for radiation workers), historical records, levels of chemicals in air or dust, levels of dermal contact, and biomonitoring for levels of workplace chemicals in the body. Breast cancer risk appears to be elevated among women with workplace exposures to night-shift work, ionizing radiation, solvents, pesticides, and other chemicals. Job strain and sedentary work are also linked to elevated breast cancer risk, while workplace physical activity reduces risk (see Figure 3).

Night-Shift Work

The International Agency for Research on Cancer designates shift work involving circadian rhythm disruption as probably carcinogenic.[50] A record linkage

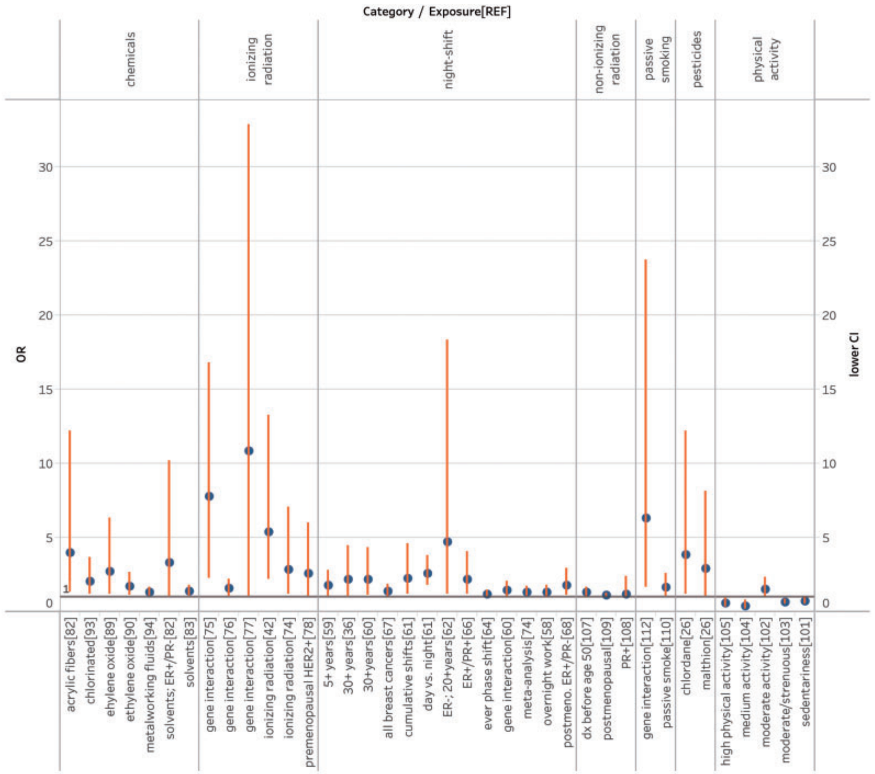

Figure 3. Work exposures and breast cancer, odds ratios. Circles represent Odds Ratios reported for workplace exposure categories, with lines showing the 95% confidence intervals. All tabled data were significantly different from OR=1. Workplace physical activity (in many cases) reduced risk; while other exposures increased risk. Data for other statistics (SIR, RR, and HR) and null findings are reported in Supplement 1.

study of occupation and cancer in Britain estimated that night-shift work may account for 4.5 percent of breast cancer cases and death.[51] A similar study in the United States estimated that night-shift work could account for 5.7 percent of the overall breast cancer incidence.[52]

Night-shift work is by far the most extensively reported occupational exposure, with eleven case–control studies, five nested case control studies, eight cohort studies, and seven meta-analyses in the published literature from 2002 to 2017. Night-shift work is often assessed via recall in interviews or questionnaires, from which researchers can assess both the timing of shifts, the frequency of shift rotations, and the duration of shift work.

Meta-analyses examining breast cancer risk among night-shift workers found 4 percent (RR 1.04; 95% CI: 1.00–1.10)[53] to 79 percent (RR 1.79;

95% CI: 1.25–2.57)[54] increased risk of breast cancer, with some indication that risk may vary by occupation[16,54] and duration of shift work.[55,56] One meta-analysis established the importance of considering other breast cancer risk factors such as reproductive history and family history. They found a 15 percent increased risk of breast cancer (RR 1.15; 95% CI: 1.05–1.25) in studies that adjusted for other risk factors. The effect was not present in unadjusted studies (RR 1.28; 95% CI: .85–1.94).[57] A 2015 meta-analysis found a steady dose–response to duration of shift work and higher risk for those with rotating shifts (RR 1.06; 95% CI: 1.01–1.10; $p = .01$ for linear trend).[56]

Individual studies suggest breast cancer risk is associated with longer duration of night-shift work,[58–63] with shift timing and patterns,[64] and with occupation.[65] Women who worked night shifts before their first pregnancy had a higher risk for breast cancer (OR 1.95; 95% CI 1.13–3.35).[58] Studies have also found differential risk for different tumor receptor subtypes of breast cancer: one study found twofold higher risk of ER+/PR+ breast cancer among women who worked frequent night shifts for five or more years (OR 2.2; 95% CI 1.2–4.1).[66] Another study found a 38 percent higher risk for all breast cancers (OR 1.38; 95% CI: 1.01–1.88), but further analysis by subtype and menopausal status found 85–180 percent elevated risk among premenopausal women, depending upon tumor subtype; (OR 2.80; 95% CI: 1.36–5.76 for HER2+ breast cancer among premenopausal women), but no elevation in risk among postmenopausal women (OR 1.03; 95% CI: 0.38–2.81 for HER2+ breast cancer among postmenopausal women).[67]

An innovative study found that chronotype may be differentially associated with breast cancer risk based upon the duration of shift work (OR 2.09; 95% CI: 1.03–4.22 for morning chronotype and one to four years of shift work).[68] Researchers are now investigating potential mechanisms for elevated risks of breast cancer among night-shift workers. Findings from studies examining melatonin levels in night-shift nurses[69,70] and reproductive and sex hormone levels in night-shift workers have been mixed.[70–73]

Ionizing Radiation

Ionizing radiation is one of the most well-studied and well-accepted environmental risk factors for breast cancer. The findings on breast cancer risk from occupational radiation exposures, however, are mixed.

A study of radiological technicians spanning six decades found greater estimated cumulative exposure to radiation increases risk of breast cancer compared to minimal exposure.[41] A nested case–control of medical diagnostic workers in China found elevated risk of breast cancer among those working from 1950 to 2011 (OR 2.9; 95% CI 1.19–7.04).[74]

One new approach is examining whether individuals' genetic variability may increase vulnerabilities to ionizing radiation.[75] Radiological technologists

monitored for radiation dose did not have elevated breast cancer risk as a group. However, among those with a specific genetic variant, exposed to higher levels of radiation, risk was 60 percent higher than those with the same dose and a different variant (OR 1.6: 95% CI: 1.1–2.2).[76] Similarly, a study of physicians monitored for radiation exposure found no evidence for increased risk among physicians with the highest dose of radiation, while those with a specific variant of a gene single-nucleotide polymorphism had more than a tenfold risk (OR 10.9; 95% CI: 1.1–32.9).[77]

Breast cancer subtype has also been found to be associated with age at exposure: premenopausal women likely exposed to ionized radiation at work were significantly more likely to be diagnosed with HER2+ breast cancer (OR 2.57; 95% CI: 1.09–6.03).[78]

Chemicals, Including Pesticides

Solvents. Benzene and other solvents are among the most thoroughly studied chemicals regarding breast cancer risk. A 2015 study found elevated breast cancer risk among women with occupational exposures to solvents, although the results were not statistically significant (OR 1.15; 95% CI: 0.98–1.35).[79]

Occupational studies of solvent exposure suggest breast cancer risk and outcome may vary by ethnicity and race. In a study of army enlisted women with high volatile organic compound exposures, black women had higher incidence compared to white women of the same age and solvent exposure levels (IRR 1.48; 95% CI: 1.03–2.12).[80] Parity may be another factor influencing breast cancer development; women exposed to solvents at work prior to the birth of their first child had an increased risk of breast cancer (OR 1.39; 95% CI: 1.03–1.86), while ever having a solvent job was not linked to increased risk of breast cancer (OR 1.04; 95% CI: 0.88–1.24).[81] Two studies of solvent exposure evaluated risk on the basis of breast cancer subtype. One study found threefold elevated risk of ER+/PR− breast cancers among women exposed before the age of 36 (OR 3.31; 95% CI 1.07–10.20),[82] while another study found ever being exposed to solvents was associated with 40 percent higher risk or ER−/PR− breast cancer (OR 1.4; 95% CI: 1.1–1.8).[83]

Pesticides. Approximately 16 percent of the entire global spending on pesticides comes from the United States.[84] More than 17,000 individual pesticides are registered for use in the United States,[85] although a much smaller number are widely used.[86] As a result, assessing the full scope of occupational pesticide exposure and health effects is daunting.

Women make up only about 3 percent of licensed pesticide applicators, although some women apply pesticides through their husbands' licenses.[86] As a result, it is difficult to parse direct pesticide exposure (e.g., from applying pesticides) and indirect exposures (e.g., from proximity to fields or from washing

clothes worn during pesticide application). Among women farming in Iowa and North Carolina, approximately half had applied pesticides at least once. Among those women, an increased risk of breast cancer was associated with terbufos use (RR 2.6; 95% CI: 1.1–5.9) and long-term methyl bromide use (RR 3.2; 95% CI: 1.2–8.7).[87]

A study of Latina agricultural workers in California found increased risk of breast cancer associated with use of malathion (OR 2.95; 95% CI: 1.07–8.11), chlordane (OR 3.85; 95% CI: 1.22–12.20), and 2,4-D (OR 2.14; 95% CI: 1.06–4.32).[26] Studies of atrazine[87] and organochlorine pesticides as a class[88] have not found evidence of elevated breast cancer risk.

Other chemicals. Work exposures to acrylic fibers have been linked to breast cancer, with up to a sevenfold increased risk with exposure before the age of 36 (OR 7.69; 95% CI: 1.47–40.24).[82]

Workers in sterilization facilities with the highest exposure levels and longest exposure time to ethylene oxide (a chemical used to sterilize instruments) had elevated breast cancer risk (OR 3.55; 95% CI: 1.58–7.93 for the highest exposures, measured in ppm-years[89] and OR 1.74; 95% CI: 1.16–2.65 for the highest cumulative exposures with a 15-year lag).[90]

The results are mixed in occupational studies of women exposed to polychlorinated biphenyls (PCBs). One study found elevated incidence among non-white women exposed to PCBs (OR 1.33; 95% CI: 1.14–1.56), with no effect among white women (OR 1.00: 95% CI 1.00–1.00).[91] However, an earlier examination of the same cohort revealed reduced mortality from breast cancer.[92] A study of male breast cancer found associations with exposures to PCBs, alkylphenols, and dioxins.[10]

Chlorinated compounds more than doubled breast cancer risk among women working in electrical manufacturing for more than ten years (OR 2.10; 95% CI: 1.21–3.66).[93] Exposure to metal-working fluids may increase risk of premenopausal breast cancer (OR 1.33; 95% CI: 1.05–1.67).[94]

Job Strain

Studies indicate job strain, job stress, and long work hours appear to increase breast cancer risk. These studies used a variety of measures to assess job strain. Among studies of self-perceived job stress or strain, only one study found elevated breast cancer risk (hazard ratio (HR) 1.40; 95% CI; 1.1–1.9)[95]; and a meta-analysis did not find increased risk (OR 0.97; 95% CI: 0.82–1.14).[96] However, studies that defined job stress based upon job authority and the capacity to hire and fire found 57 to 82 percent increased risk of breast cancer (HR 1.57; 95% CI: 1.12–2.18 with high job authority and HR 1.82; 95% CI: 1.26–2.63 among women with professional jobs adjusted for job authority).[97] A second study by the same author found similar elevations in breast cancer

risk among women with the ability to hire, fire, and influence pay in 1975 (HR 1.58; 95% CI: 1.32–1.84) and 1993 (HR 1.52; 95% CI: 1.10–1.95).[98] Another study found that working long hours (more than fifty-five hours/week compared to thirty-five to forty hours/week) increased breast cancer risk by 60 percent (HR 1.60; 95% CI: 1.12–2.29).[96]

Sedentary Work and Workplace Physical Activity

A study of African-American women found risk of ER−/PR− breast cancer was 70 percent higher among women with the highest total sitting time (HR 1.70; 95% CI: 1.13–2.55).[99] The results of this study were fairly consistent with another study. Both found risk was elevated by 20 to 30 percent among women with more sedentary jobs (HR 1.36: 95% CI: 1.06–1.76[99] and HR 1.20; 95% CI: 1.05–1.37)[100] and that sedentary jobs before menopause or the age of 55 increased risk by 54 percent (HR 1.54; 95% CI: 1.13–2.08[99] and HR 1.54; 95% CI: 1.20–1.96[100]). A third study did not find increased risk of breast cancer based upon self-reported occupational sitting and found that postmenopausal women in the highest category of occupational sitting had reduced breast cancer risk (OR 0.71: 95% CI: 0.52–0.97).[101]

In contrast, higher occupational physical activity appears to reduce breast cancer risk. Most studies report statistically significant reductions in risk of breast cancer among women with the most active jobs. This protective effect may be most pronounced among postmenopausal women.[102–106]

Nonionizing Radiation

Overall, the data do not suggest that occupational exposures to nonionizing radiation (also described as electro-magnetic fields) increase the risk of breast cancer. None of the studies found an effect looking at overall risk elevations in the women studied. Two studies found modestly elevated risk of about 20 to 30 percent among younger women: OR 1.32: 95% CI: 1.03–1.69 (before the age of 50)[107] and OR 1.21: 95% CI: 1.02–2.39 (PR+ tumors, before the age of 35).[108] However, another study found increased risk among postmenopausal women (OR 1.12; 95% CI: 1.02–1.24) but not premenopausal women (OR 1.15; 95% CI: .79–1.70).[109]

Passive Smoking

Workplace passive smoking also appears unlikely to confer elevated breast cancer risk, although workplace passive smoking effects may be overpowered by the effects of active smoking or other passive smoking exposures in multivariate models. One meta-analysis found 66 percent elevated risk due to passive smoke exposure at work (OR 1.66 95% CI: 1.07–2.59),[110] while a global meta-analysis found no evidence of increased risk (RR 1.03; 95% CI: 0.99–1.07).[111]

One study found an interaction between a specific gene variant and adult workplace exposure to tobacco smoke (OR 6.33; 95% CI: 1.69–23.71).[112]

Discussion

Based upon our scoping review of the most recent literature on occupation and breast cancer, risk for breast cancer may be elevated for women working as flight attendants, in medical professions, in some production jobs, in sales and retail work, and in scientific jobs. In addition, occupational exposures to night-shift work, ionizing radiation, some chemicals, job stress, and sedentary work may increase risk of breast cancer.

While nurses and teachers are often cited as two occupational groups with elevated breast cancer risk, the most recent data suggest that teachers may not have increased risk of breast cancer once other risk factors, such as reproductive history and family history, are included in the analysis. For nurses, the findings are mixed, but suggestive of a modest increased risk. However, nursing is a particularly large and diverse job category that includes diverse work settings (hospital and clinic), job roles (administrative and floor nurse), specialty (oncology, surgery, and pediatrics), and exposures (ionizing radiation, night-shift work, chemotherapeutic agents, and sterilants). These findings underscore the need to evaluate actual job roles and exposures when assessing occupational risk for breast cancer.

Indeed, job titles may serve more as a proxy for exposures than as specific risk factors in and of themselves, and refinement of job activities and exposures could help to clarify the relationship between occupation and breast cancer.

Some studies of both occupations and workplace exposures indicate risk may be modified by duration of exposure, timing of exposure, dose, hormone-receptor subtypes, menopausal status at diagnosis, and interactions with accepted risk factors such as parity and family history. These findings are consistent with the larger literature on breast cancer and the environment, which has asserted that breast cancer causation is a complex interaction of genes and environment that develops across the life course.[113] Our second paper explores these and other key methodological gaps in the literature on breast cancer and occupation as well as recommendations to fill those gaps.

Declaration of Conflicting Interests

The author(s) declared no potential conflicts of interest with respect to the research, authorship, and/or publication of this article.

Funding

The author(s) received no financial support for the research, authorship, and/or publication of this article.

Supplemental Material

Supplementary material is available for this article online.

References

1. American Cancer Society. Cancer Facts and Figures: 2018. Report for the American Cancer Society, Surveillance Research, 2018.
2. American Cancer Society. Cancer Facts and Figures – Table 1. Estimated Number* of New Cancer Cases and Deaths by Sex, US, 2018. Report for the American Cancer Society, Surveillance Research, 2018.
3. Alizart M, Saunus J, Cummings M, et al. Molecular classification of breast carcinoma. *Diagn Histopathol (Oxf)* 2012; 18: 97–103.
4. Turkoz FP, Solak M, Petekkaya I, et al. Association between common risk factors and molecular subtypes in breast cancer patients. *Breast* 2013; 22: 344–350.
5. Amend K, Hicks D and Ambrosone CB. Breast cancer in African-American women: differences in tumor biology from European-American women. *Cancer Res* 2006; 66: 8327–8330.
6. Howlader N, Altekruse SF, Li CI, et al. US incidence of breast cancer subtypes defined by joint hormone receptor and HER2 status. *J Natl Cancer Inst* 2014; 106: pii: dju055.
7. Wirth M, Vena JE, Smith EK, et al. The epidemiology of cancer among police officers. *Am J Ind Med* 2013; 56: 439–453.
8. Ma F, Fleming LE, Lee DJ, et al. Mortality in Florida professional firefighters, 1972 to 1999. *Am J Ind Med* 2005; 47: 509–517.
9. Ruckart PZ, Bove FJ, Shanley E, et al. Evaluation of contaminated drinking water and male breast cancer at Marine Corps Base Camp Lejeune, North Carolina: a case control study. *Environ Health* 2015; 14: 74.
10. Villeneuve S, Cyr D, Lynge E, et al. Occupation and occupational exposure to endocrine disrupting chemicals in male breast cancer: a case–control study in Europe. *Occup Environ Med* 2010; 67: 837–844.
11. BLS Reports. *Women in the labor force: a databook*. Washington, DC: US Bureau of Labor Statistics. Report no. 1059, December 2015. Available at: https://www.bls.gov/opub/reports/womens-databook/archive/women-in-the-labor-force-a-databook-2015.pdf
12. Byars-Winston A, Fouad N, Wen Y. Race/ethnicity and sex in U.S. occupations, 1970-2010: Implications for research, practice, and policy. *J Vocat Behav* 2015; 87: 54–70.
13. Lippa RA, Preston K, Penner J. Women's Representation in 60 occupations from 1972 to 2010: more women in high-status jobs, few women in things-oriented jobs. *PloS One* 2014; 9: e95960
14. Infante PF. Cancer and blue-collar workers: who cares? *New Solut* 1995; 5: 52–57.
15. Hohenadel K, Raj P, Demers PA, et al. The inclusion of women in studies of occupational cancer: a review of the epidemiologic literature from 1991–2009. *Am J Ind Med* 2015; 58: 276–281.
16. He C, Anand ST, Ebell MH, et al. Circadian disrupting exposures and breast cancer risk: a meta-analysis. *Int Arch Occup Environ Health* 2015; 88: 533–547.

17. Allen JG, Stapleton HM, Vallarino J, et al. Exposure to flame retardant chemicals on commercial airplanes. *Environ Health* 2013; 12: 17.
18. Goodson WH, Lowe L, Carpenter DO, et al. Assessing the carcinogenic potential of low-dose exposures to chemical mixtures in the environment: the challenge ahead. *Carcinogenesis* 2015; 36(Suppl 1): S254–S296.
19. Arksey H, O'Malley L. Scoping studies: towards a methodological framework. *Int J Soc Res Methodol* 2005; 8: 19–32.
20. Armstrong R, Hall BJ, Doyle J, et al. Scoping the scope' of a Cochrane review. *J Public Health (Oxf)* 2011; 33: 147–150.
21. Levac D, Colquhoun H, O'Brien KK. Scoping studies: advancing the methodology. *Implement Sci* 2010; 5: 69
22. Peters MD, Godfrey CM, Khalil H, et al. Guidance for conducting systematic scoping reviews. *Int J Evid Based Healthc* 2015; 13: 141–146.
23. Cacchione PZ. The evolving methodology of scoping reviews. *Clin Nurs Res* 2016; 25: 115–119.
24. Breast Cancer Fund. Working women and breast cancer: the state of the evidence, https://www.bcpp.org/science-policy/ (2017, accessed 5 June 2017).
25. US Department of Labor. Standard occupational categories, https://www.bls.gov/soc/ (2010, accessed 27 April 2017).
26. Mills PK and Yang R. Breast cancer risk in Hispanic agricultural workers in California. *Int J Occup Environ Health* 2005; 11: 123–131.
27. Gardner KM, Shu XO, Jin F, et al. Occupations and breast cancer risk among Chinese women in urban Shanghai. *Am J Ind Med* 2002; 42: 296–308.
28. Brophy JT, Keith MM, Watterson A, et al. Breast cancer risk in relation to occupations with exposure to carcinogens and endocrine disruptors: A Canadian case-control study. *Environ Health* 2012; 11: 87
29. Bernstein L, Allen M, Anton-Culver H, et al. High breast cancer incidence rates among California teachers: Results from the California Teachers Study (United States). *Cancer Causes Control* 2002; 13: 625–635.
30. Teitelbaum SL, Britton JA, Gammon MD, et al. Occupation and breast cancer in women 20-44 years of age (United States). *Cancer Causes Control* 2003; 14: 627–637.
31. Liu T, Zhang C and Liu C. The incidence of breast cancer among female flight attendants: an updated meta-analysis. *J Travel Med* 2016; 23: 1–7.
32. Rafnsson V, Sulem P, Tulinius H, et al. Breast cancer risk in airline cabin attendants: a nested case-control study in Iceland. *Occup Environ Med* 2003; 60: 807–809.
33. Schubauer-Berigan MK, Anderson JL, et al. Breast cancer incidence in a cohort of U.S. flight attendants. *Am J Ind Med* 2015; 58: 252–266.
34. Reynolds P, Cone J, Layefsky M, et al. Cancer incidence in California flight attendants (United States). *Cancer Causes Control* 2002; 13: 317–324.
35. Kjaer TK, Hansen J. Cancer incidence among large cohort of female Danish registered nurses. *Scand J Work Environ Health* 2009; 35: 446–453.
36. Lie J-AS, Andersen A, Kjaerheim K. Cancer risk among 43000 Norwegian nurses. *Scand J Work Environ Health* 2007; 33: 66–73.
37. Santi SA, Meigs ML, Zhao Y, et al. A case-control study of breast cancer risk in nurses from Northeastern Ontario, Canada. *Cancer Causes Control* 2015; 26: 1421–1428.

38. Chou LB, Chandran S, Harris AHS, et al. Increased breast cancer prevalence among female orthopedic surgeons. *J Womens Health* 2012; 21: 683–689.
39. Innos K, Rahu K, Baburin A, et al. Cancer incidence and cause-specific mortality in male and female physicians: a cohort study in Estonia. *Scand J Public Health* 2002; 30: 133–140.
40. Ji B-T, Blair A, Shu X-O, et al. Occupation and breast cancer risk among Shanghai women in a population-based cohort study. *Am J Ind Med* 2008; 51: 100–110.
41. Doody MM, Freedman DM, Alexander BH, et al. Breast cancer incidence in U.S. radiologic technologists. *Cancer* 2006; 106: 2707–2715.
42. Shaham J, Gurvich R, Goral A, et al. The risk of breast cancer in relation to health habits and occupational exposures. *Am J Ind Med* 2006; 49: 1021–1030.
43. Ekpanyaskul C, Khuhaprema T, Wiangnon S, et al. Case-control study of occupational categories and breast cancer risk in Thailand. *Asian Pac J Cancer Prev* 2010; 11: 793–797.
44. Peplonska B, Stewart P, Szeszenia-Dabrowska N, et al. Occupation and breast cancer risk in Polish women: A population-based case-control study. *Am J Ind Med* 2007; 50: 97–111.
45. Kuzmickiene I, Didziapetris R, Stukonis M. Cancer incidence in the workers cohort of textile manufacturing factory in Alytus, Lithuania. *J Occup Environ Med* 2004; 46: 147–153.
46. Villeneuve S, Févotte J, Anger A, et al. Breast cancer risk by occupation and industry: Analysis of the CECILE study, a population-based case-control study in France. *Am J Ind Med* 2011; 54: 499–509.
47. Shaham J, Gurvich R, Kneshet Y. Cancer incidence among laboratory workers in biomedical research and routine laboratories in Israel: Part I-the cohort study. *Am J Ind Med* 2003; 44: 600–610.
48. Takkouche B, Regueira MC, Montes MA. Risk of cancer among hairdressers and related workers: a meta-analysis. *Int J Epidemiol* 2009; 38: 1512–1531.
49. Quach T, Doan-Billing PA, Layefsky M, et al. Cancer incidence in female cosmetologists and manicurists in California, 1988–2005. *Am J Epidemiol* 2010; 172: 691–699.
50. IARC. *Agents classified by the IARC monographs*. Vols 1–120. Report, January 2018. Lyon, France: IARC.
51. Slack R, Young C and Rushton L. Occupational cancer in Britain. *Br J Cancer* 2012; 107: S49–S55.
52. Purdue MP, Hutchings SJ, Rushton L, et al. The proportion of cancer attributable to occupational exposures. *Ann Epidemiol* 2015; 25: 188–192.
53. Ijaz S, Verbeek J, Seidler A, et al. Night-shift work and breast cancer – A systematic review and meta-analysis. *Scand J Work Environ Health* 2013; 39: 431–447.
54. Kamdar BB, Tergas AI, Mateen FJ, et al. Night-shift work and risk of breast cancer: A systematic review and meta-analysis. *Breast Cancer Res Treat* 2013; 138: 291–301.
55. Wang F, Yeung KL, Chan WC, et al. A meta-analysis on dose-response relationship between night shift work and the risk of breast cancer. *Ann Oncol* 2013; 24: 2724–2732.

56. Lin X, Chen W, Wei F, et al. Night-shift work increases morbidity of breast cancer and all-cause mortality: a meta-analysis of 16 prospective cohort studies. *Sleep Med* 2015; 16: 1381–1387.
57. Jia Y, Lu Y, Wu K, et al. Does night work increase the risk of breast cancer? A systematic review and meta-analysis of epidemiological studies. *Cancer Epidemiol* 2013; 37: 197–206.
58. Menegaux F, Truong T, Anger A, et al. Night work and breast cancer: a population-based case–control study in France (the CECILE study). *Int J Cancer* 2013; 132: 924–931.
59. Lie JA, Kjuus H, Zienolddiny S, et al. Night work and breast cancer risk among Norwegian nurses: assessment by different exposure metrics. *Am J Epidemiol* 2011; 173: 1272–1279.
60. Grundy A, Richardson H, Burstyn I, et al. Increased risk of breast cancer associated with long-term shift work in Canada. *Occup Environ Med* 2013; 70: 831–838.
61. Hansen J, Lassen CF. Nested case-control study of night shift work and breast cancer risk among women in the Danish military. *Occup Environ Med* 2012; 69: 551–556.
62. Rabstein S, Harth V, Pesch B, et al. Night work and breast cancer estrogen receptor status–results from the German GENICA study. *Scand J Work Environ Health* 2013; 39: 448–455.
63. Schernhammer ES, Kroenke CH, Laden F, et al. Night work and risk of breast cancer. *Epidemiology* 2006; 17: 108–111.
64. Fritschi L, Erren TC, Glass DC, et al. The association between different night shiftwork factors and breast cancer: A case-control study. *Br J Cancer* 2013; 109: 2472–2480.
65. Megdal SP, Kroenke CH, Laden F, et al. Night work and breast cancer risk: a systematic review and meta-analysis. *Eur J Cancer* 2005; 41: 2023–2032.
66. Lie J-AS, Kjuus H, Zienolddiny S, et al. Breast cancer among nurses: is the intensity of night work related to hormone receptor status? *Am J Epidemiol* 2013; 178: 110–117.
67. Cordina-Duverger E, Koudou Y, Truong T, et al. Night work and breast cancer risk defined by human epidermal growth factor receptor-2 (HER2) and hormone receptor status: A population-based case-control study in France. *Chronobiol Int* 2016; 33: 783–787.
68. Papantoniou K, Castaño-Vinyals G, Espinosa A, et al. Breast cancer risk and night shift work in a case–control study in a Spanish population. *Eur J Epidemiol* 2016; 31: 867–878.
69. Grundy A, Tranmer J, Richardson H, et al. The influence of light at night exposure on melatonin levels among Canadian rotating shift nurses. *Cancer Epidemiol Biomarkers Prev* 2011; 20: 2404–2412.
70. Langley AR, Graham CH, Grundy AL, et al. A cross-sectional study of breast cancer biomarkers among shift working nurses. *BMJ Open* 2012; 2: e000532
71. Davis S, Mirick DK, Chen C, et al. Night shift work and hormone levels in women. *Cancer Epidemiol Biomarkers Prev* 2012; 21: 609–618.
72. Gómez-Acebo I, Dierssen-Sotos T, Papantoniou K, et al. Association between exposure to rotating night shift versus day shift using levels of

6-sulfatoxymelatonin and cortisol and other sex hormones in women. *Chronobiol Int* 2015; 32: 128–135.
73. Nagata C, Nagao Y, Yamamoto S, et al. Light exposure at night, urinary 6-sulfatoxymelatonin, and serum estrogens and androgens in postmenopausal Japanese women. *Cancer Epidemiol Biomarkers Prev* 2008; 17: 1418–1423.
74. Wang FR, Fang QQ, Tang WM, et al. Nested case-control study of occupational radiation exposure and breast and esophagus cancer risk among medical diagnostic X ray workers in Jiangsu of China. *Asian Pac J Cancer Prev* 2015; 16: 4699–4704.
75. Sigurdson AJ, Bhatti P, Doody MM, et al. Polymorphisms in apoptosis-and proliferation-related genes, ionizing radiation exposure, and risk of breast cancer among U.S. Radiologic Technologists. *Cancer. Epidemiol Biomarkers Prev* 2007; 16: 2000–2007.
76. Bhatti P, Doody MM, Alexander BH, et al. Breast cancer risk polymorphisms and interaction with ionizing radiation among U.S. radiologic technologists. *Cancer Epidemiol Biomarkers Prev* 2008; 17: 2007–2011.
77. Bhatti P, Struewing JP, Alexander BH, et al. Polymorphisms in DNA repair genes, ionizing radiation exposure and risk of breast cancer in U.S. radiologic technologists. *Int J Cancer* 2008; 122: 177–182.
78. Buitenhuis W, Fritschi L, Thomson A, et al. Occupational Exposure to Ionizing Radiation and Risk of Breast Cancer in Western Australia. *J Occup Environ Med* 2013; 55: 1431–1435.
79. Glass DC, Heyworth J, Thomson AK, et al. Occupational exposure to solvents and risk of breast cancer. *Am J Ind Med* 2015; 58: 915–922.
80. Rennix CP, Quinn MM, Amoroso PJ, et al. Risk of breast cancer among enlisted Army women occupationally exposed to volatile organic compounds. *Am J Ind Med* 2005; 48: 157–167.
81. Ekenga CC, Parks CG, D'Aloisio AA, et al. Breast cancer risk after occupational solvent exposure: the influence of timing and setting. *Cancer Res* 2014; 74: 3076–3083.
82. Labreche F, Goldberg MS, Valois MF, et al. Postmenopausal breast cancer and occupational exposures. *Occup Environ Med* 2010; 67: 263–269.
83. Peplonska B, Stewart P, Szeszenia-Dabrowska N, et al. Occupational exposure to organic solvents and breast cancer in women. *Occup Environ Med* 2009; 67: 722–729.
84. Atwood D and Paisley-Jones C. Pesticides industry sales and usage: 2008-2012 market estimates. Report, Environmental Protection Agency, Washington, DC, 2017.
85. Pesticide Action Network. PAN pesticides database, http://www.pesticideinfo.org/Search_Chemicals.jsp (2014, accessed 6 February 2015).
86. Engel LS, Hill DA, Hoppin JA, et al. Pesticide use and breast cancer risk among farmers' wives in the agricultural health study. *Am J Epidemiol* 2005; 161: 121–135.
87. Beane Freeman LE, Rusiecki JA, et al. Atrazine and cancer incidence among pesticide applicators in the Agricultural Health Study (1994–2007). *Environ Health Perspect* 2011; 119: 1253–1259.
88. Andreotti G, Hou L, Beane F, et al. Body mass index, agricultural pesticide use, and cancer incidence in the Agricultural Health Study cohort. *Cancer Causes Control* 2010; 21: 1759–1775.

89. Mikoczy Z, Tinnerberg H, Björk J, et al. Cancer incidence and mortality in Swedish sterilant workers exposed to ethylene oxide: updated cohort study findings 1972–2006. *Int J Environ Res Public Health* 2011; 8: 2009–2019.
90. Steenland K, Whelan E, Deddens J, et al. Ethylene oxide and breast cancer incidence in a cohort study of 7576 women (United States). *Cancer Causes Control* 2003; 14: 531–539.
91. Silver SR, Whelan EA, Deddens JA, et al. Occupational exposure to polychlorinated biphenyls and risk of breast cancer. *Environ Health Perspect* 2009; 117: 276.
92. Prince MM, Hein MJ, Ruder AM, et al. Update: cohort mortality study of workers highly exposed to polychlorinated biphenyls (PCBs) during the manufacture of electrical capacitors, 1940-1998. *Environ Health* 2006; 5: 1.
93. Oddone E, Edefonti V, Scaburri A, et al. Female breast cancer and electrical manufacturing: results of a nested case-control study. *J Occup Health* 2014; 56: 369–378.
94. Thompson D, Kriebel D, Quinn MM, et al. Occupational exposure to metalworking fluids and risk of breast cancer among female autoworkers. *Am J Ind Med* 2005; 47: 153–160.
95. Kuper H, Yang L, Theorell T, et al. Job strain and risk of breast cancer. *Epidemiology* 2007; 18: 764–768.
96. Heikkila K, Nyberg ST, Madsen IEH, et al. IPD-Work Consortium. Long working hours and cancer risk: a multi-cohort study. *Br J Cancer* 2016; 114: 813–818.
97. Pudrovska T, Carr D, McFarland M, et al. Higher-status occupations and breast cancer: A life-course stress approach. *Soc Sci Med* 2013; 89: 53–61.
98. Pudrovska T. Job authority and breast cancer. *Soc Forces* 2013; 92: 1–24.
99. Nomura SJO, Dash C, Rosenberg L, et al. Sedentary time and breast cancer incidence in African American women. *Cancer Causes Control* 2016; 27: 1239–1252.
100. Johnsson A, Broberg P, Johnsson A, et al. Occupational sedentariness and breast cancer risk. *Acta Oncol* 2017; 56: 75–80.
101. Lynch BM, Courneya KS, Friedenreich CM. A case-control study of lifetime occupational sitting and likelihood of breast cancer. *Cancer Causes Control* 2013; 24: 1257–1262.
102. Dorn J, Vena J, Brasure J, et al. Lifetime physical activity and breast cancer risk in pre- and postmenopausal women. *Med Sci Sports Exerc* 2003; 35: 278–285.
103. John EM, Horn-Ross PL, Koo J. Lifetime physical activity and breast cancer risk in a multiethnic population: the San Francisco Bay area breast cancer study. *Cancer Epidemiol Biomarkers Prev* 2003; 12: 1143–1152.
104. Kruk J. Lifetime occupational physical activity and the risk of breast cancer: a case-control study. *Asian Pac J Cancer Prev* 2009; 10: 443–448.
105. Kruk J, Aboul-Enein HY. Occupational physical activity and the risk of breast cancer. *Cancer Detect Prev* 2003; 27: 187–192.
106. Ekenga CC, Parks CG, Sandler DP. A prospective study of occupational physical activity and breast cancer risk. *Cancer Causes Control* 2015; 26: 1779–1789.
107. Li W, Ray RM, Thomas DB, et al. Occupational exposure to magnetic fields and breast cancer among women textile workers in Shanghai, China. *Am J Epidemiol* 2013; 178: 1038–1045.
108. Labrèche F, Goldberg MS, Valois M-F, et al. Occupational exposures to extremely low frequency magnetic fields and postmenopausal breast cancer. *Am J Ind Med* 2003; 44: 643–652.

109. McElroy JA, Egan KM, Titus-Ernstoff L, et al. Occupational exposure to electromagnetic field and breast cancer risk in a large, population-based, case-control study in the United States. *J Occup Environ Med* 2007; 49: 266–274.
110. Chen C, Huang Y-B, Liu X-O, et al. Active and passive smoking with breast cancer risk for Chinese females: a systematic review and meta-analysis. *Chin J Cancer* 2014; 33: 306–316.
111. Lee PN and Hamling JS. Environmental tobacco smoke exposure and risk of breast cancer in nonsmoking women. An updated review and meta-analysis. *Inhal Toxicol* 2016; 28: 431–454.
112. Anderson LN, Cotterchio M, Mirea L, et al. Passive cigarette smoke exposure during various periods of life, genetic variants, and breast cancer risk among never smokers. *Am. J Epidemiol* 2012; 175: 289–301.
113. IBCERCC. Breast cancer and the environment. Prioritizing prevention. Report for the Cancer and Environmental Research Coordinating Committee (IBCERCC), February 2013. Washington, DC: IBCERCC.

Author Biographies

Connie L. Engel leads Breast Cancer Prevention Partners' efforts to translate the science linking chemicals and radiation to breast cancer. She has led work on occupation and breast cancer, coordinated product testing efforts, coauthored multiple reports, and used new tools to present the science of breast cancer and the environment. Her dissertation research at Arizona State University focused on the conjunction of science and advocacy in the environmental breast cancer movements. This work drew from multiple disciplines including sociology, psychology, legal studies, and women and gender studies.

M. Sharima Rasanayagam oversees Breast Cancer Prevention Partners' science-related activities to ensure that the organization continues to be a national leader in science-based environmental health advocacy. Before coming to BCPP, Sharima was the founding academic coordinator at the U.C. Berkeley Institute for the Environment, where she built the institute from the ground up. Previously, she served as Consul for Science and Technology at the U.K. Consulate-General in San Francisco, where she built collaborative research and business ties between scientists in the United Kingdom and California. Sharima holds a PhD in microbiology from the University of Kent at Canterbury, U.K.

Janet M. Gray is a Vassar professor and director of the college's Science, Technology, and Society Program. She has a background in behavioral neuroscience, and her laboratory research has focused on the effects of estrogens and mixed antiestrogens, especially tamoxifen, on brain activity and behavior. In recent years, her research and writing focus has turned toward engaging the public in the complex issues surrounding breast cancer and environmental

risks. Gray is the editor of the fifth (2008) and sixth (2010) editions of the Breast Cancer Prevention Partners' groundbreaking report, State of the Evidence: The Connection Between Breast Cancer and the Environment. She led a multidisciplinary group of students and professionals in the development of a widely distributed interactive tool that explores the scientific literature on breast cancer and environmental risks.

Jeanne Rizzo's vision guided Breast Cancer Prevention Partners to adopt its bold mission to prevent breast cancer by eliminating our exposure to toxic chemicals and radiation linked to the disease. Under her leadership the organization continues its commitment to strong science, smart public policy, and consumer education. Ms. Rizzo is cofounder of Cancer Free Economy Network and a recipient of many awards, most recently the National Institute of Environmental Health Sciences' "Champion of Environmental Health Research Award". A nurse, then an award-winning music, theater and film producer, Ms. Rizzo produced the documentary *Climb Against the Odds: Mt. McKinley*, which chronicles Breast Cancer Prevention Partners' 1998 expedition.
