# Supplementary material for: Magnetic Fields and Cancer: Epidemiology, Cellular Biology, and Theranostics
Source: Int J Mol Sci. 2022 Jan 25;23(3):1339. doi: 10.3390/ijms23031339 (PMC8835851; doi:10.3390/ijms23031339)
Supplement: Supplementary file 1 [file ijms-23-01339-s001.zip › Supplementary Data Set S1/MF and Cancer.Data/PDF/3370616733/Brain_Cancer_Risk_and_Electromagnetic_Fields_E.pdf]

ResearchGate

See discussions, stats, and author profiles for this publication at: https://www.researchgate.net/publication/215658255

Brain Cancer Risk and Electromagnetic Fields (EMFs): Assessing the Geomagnetic Component

Article *in* Archives of Environmental Health An International Journal · July 2001

DOI: 10.1080/00039890109604462

CITATIONS
17

READS
222

3 authors, including:

Abraham R Liboff
Oakland University

111 PUBLICATIONS **3,298** CITATIONS

SEE PROFILE

Some of the authors of this publication are also working on these related projects:

Project helical proton-hopping aqueous paths View project

All content following this page was uploaded by Abraham R Liboff on 19 July 2014.

The user has requested enhancement of the downloaded file.

Brain Cancer Risk and Electromagnetic Fields (EMFs): Assessing the Geomagnetic Component

TIM E. ALDRICH*
KURTIS W. ANDREWS
Merck Medco Managed Care
Franklin, New Jersey
ABRAHAM R. LIBOFF
Physics Department
Oakland University
Rochester, Minnesota

ABSTRACT. Cancer cluster studies in North Carolina identified several communities in which there existed an elevated risk of brain cancer. These findings prompted a series of case-control studies. The current article, which originated from the results of the 3rd of such studies, is focused on inclusion of the earth's own geomagnetic fields that interact with electromagnetic fields generated from distribution power lines. This article also contains an assessment of the contribution of confounding by residential (e.g., urban, rural) and case characteristics (e.g., age, race, gender). Newly diagnosed brain cancer cases were identified for a 4-county region of central North Carolina, which the authors chose on the basis of the results of earlier observations. A 3:1 matched series of cancer cases from the same hospitals in which the cases were diagnosed served as the comparison group. Extensive geographic information was collected and was based on an exact place of residence at the time of cancer diagnosis, thus providing several strategic geophysical elements for assessment. The model for this assessment was based on the effects of these two sources of electromagnetic fields for an ion cyclotron resonance mechanism of disease risk. The authors used logistic regression models that contained the predicted value for the parallel component of the earth's magnetic field; these models were somewhat erratic, and the elements were not merged productively into a single statistical model. Interpretation of these values was difficult; therefore, the modeled values for the model elements, at progressive distances from the nearest power-line segments, are provided. The results of this study demonstrate the merits of using large, population-based databases, as well as using rigorous Geographic Information System techniques, for the assessment of ecologic environmental risks. The results also suggest promise for exposure classification that is compatible with the theoretical biological mechanisms posited for electromagnetic fields.

<Key words: brain cancer, cancer registries, electromagnetic fields, EMFs, Geographic Information Systems>

RISK FACTORS for brain cancer are poorly understood, and few strong associations exist. Although brain cancer demonstrates the log-linear relationship with age that is typical for other cancers, gender, smoking, and/or alcohol do not appear to be associated with brain cancer. Caucasians are about twice as likely to develop brain cancer as are African Americans. The racial differences may result from a diagnostic selection bias, from differing access to health care, or from genetic traits.[1]

The rising incidence of brain cancer during the past 2

*Dr. Aldrich is currently affiliated with the Department of Epidemiology and Biostatistics, University of South Carolina, School of Public Health, Columbia, South Carolina.

decades has led to increased concern about environmental and occupational exposures.[2–4] The rarity and rapid life-threatening course of brain cancer make performance of research studies difficult. Employment in chemical,[4,5] nuclear,[6] utility,[7,8] and petroleum industries,[9,10] as well as in poultry and farm processing,[11,12] are associated with higher risks of brain cancer. Electrical occupations have been implicated specifically for brain cancer risk, including specific cellular types of the disease.[13–15] Both residential and paternal occupational exposure to electromagnetic fields (EMFs) are potential causal exposures for childhood brain cancer.[16,17]

In North Carolina, reports of cancer clusters called attention to 5 distinct communities that experienced higher rates of brain cancer than expected. The initial investigation of 1 of these reported brain cancer clusters did not reveal any consistent association with a specific risk factor.[18] Nonetheless, subsequent reports and suggestive findings prompted the first case-control study of this series.[19] Given the burgeoning capabilities for Geographic Information System (GIS) studies and the use of rapid case finding within the large population-based cancer registry,[20,21] we undertook a second case-control study and conducted rapid case ascertainment for a 1-yr period (i.e., 1994) with a very frugal budget.[22] That evaluation was disheartening, although the GIS component was most impressive; in fact, the evaluation of the GIS value for this sort of exploratory, environmental work became our principal product.

We decided to conduct a 3rd case-control study of brain cancer in 4 of the counties. In this study, we expanded the data for incidence over a 5-yr interval (i.e., to improve sample size consideration) and used 3:1 matched cancer patients for comparison. We sought to control for confounding factors that may have been possibly related to brain cancer risk (e.g., age, race, gender).[23] For the residential exposure metric, we used a formula proposed for ion cyclotron resonance (ICR) that incorporated the orientations of power lines relative to geomagnetic North, as well as several other aspects of this source.[24] In several studies, researchers have reported the theory of a direct current (DC) magnetic field interacting with an alternating current (AC); effects ranged from calcium efflux in chicken brain tissue[25] to implications of human hematopoietic effects.[25,26] GIS methods would greatly facilitate this sort of complex exposure assessment in which a variety of databases available from federal, state, and private sources are used.

Method

We designed the study to test the null hypothesis of no difference between brain cancer cases and matched control subjects with respect to residential environmental exposure to extremely low-frequency (i.e., 60 Hz) magnetic fields from high-voltage electrical-distribution power lines (i.e., 44 kV or greater). The newly diagnosed cases included 251 adults who were diagnosed with malignant brain cancer between January 1, 1990, and December 31, 1994, and who lived in a 4-county area of central North Carolina. The study counties maximized the effect of referral patterns into the state's large medical centers. We selected the 753 comparison subjects randomly from cancer cases diagnosed at the same institution (Table 1); their cancers were likely not related to the same environmental exposures as was brain cancer (leukemia, lymphoma, and breast cancer cases were excluded). Matching criteria for the 3:1 selection of comparison subjects were county of residence, institution at which diagnosis was made, and age (± 5 yr). The comparability of the 2 groups was quite good with respect to general descriptive attributes (Table 1).

Power line information was provided from power companies that served the 4 counties. Information extended to the 44-kV level; there was a total of 1,763 km (1,095 statute mi) of electrical power lines within these counties, present in 2,169 linear segments. The largest constituents were 100-kV lines (1,180 segments, 832 km [517 mi]) and 230-kV lines (493 segments, 509 km [316 mi]); 49 segments were 500-kV lines that extended for 93 km (58 mi). Given the simple density, we did not make assessments under 44 kV.

Address matching was very good with the GIS capabilities. Urban and suburban addresses matched (i.e., in excess of 90%). Rural addresses were the most poorly matched; the matching rate for rural counties varied from 74% to 88%. For the unmatched subjects, we used topographic maps and zip codes, plus 4 postal addresses, to locate the address and to assign an approximate residence location. This was a technique used rigorously with earlier brain-cancer studies for 2 of the 4 counties.[18,27] With this procedure, the mail carrier's route to the residence is "followed" on detailed topographic maps (124,000), with the residence location assigned by simple linear interpolation between specified box locations, whenever the specific box location is not found explicitly on the topographic map. Given our prior experience, we undertook field work with only a sample of the addresses (i.e., 10 cases and 20 controls) to facilitate analysis of proximity to power lines. This zip-code-assisted mapping procedure proved very satisfactory for the scale of the spatial analyses performed.

We digitized the residential data for each case/control for purposes of mapping. Address matching was accomplished with Arc/Info GIS software[28] and with the street-network databases of the Transportation Information Management System.[29] We obtained successful address matches for 216 cases (86.1%) and 639 (84.9%) of the comparison subjects. We also made field visits to verify a sample of the addresses and to seek cases that did not match (field workers were blinded to case-control status; they searched 38 total addresses, of which half were cases). The unmatched addresses were mainly post office boxes and rural addresses. The Arc/Info addressing procedure assigns a geographic coordinate to each matched address and creates a point feature in a map database—each point representing a residential location along a street network. Once the

Table 1.—Descriptive Characteristics of Cases and Controls in a 4-County Study Area of Central North Carolina, 1990–1994

Characteristic	Cases	Controls	Crude OR	Adjusted* OR	Adjusted* OR
Number of subjects	251	753			
Mean age (yr)	60.4	65.2	0.80	0.79	
Gender			0.87	0.88	
Male (%)	51	55			
Female (%)	49	45			
Race			1.77	2.01	
Black (%)	11	18			
White (%)	89	82			
Location					
Urban (%)	70	71	1.01	0.94	
Suburban (%)	9	7	1.00	0.89	
Rural (%)	21	22			
Address matched	216 (86.1%)	639 (84.9%)			
Linear distance (m) to nearest power lines					
Mean	1,394.6	1,246.0			
Median	1,042.7	897.7			
Specific distance(s) (m)† to power line(s)					
< 61 (*n*)	7 (3.24%)	24 (3.76%)	0.84	29.5	14.43
≥ 61–152 (*n*)	12 (5.56%)	34 (5.32%)	1.02	22.4	56.12
>152–305 (*n*)	23 (10.65%)	64 (10.02%)	1.04	∞	∞
>305–610 (*n*)	33 (15.28%)	110 (17.21%)	0.86	1.85	1.35
> 610 (*n*)	141 (65.28%)	407 (63.69%)	1.00	1.00	1.00
Mean magnetic intensity (μT)	532.1	532.0			
Orientation (degrees) to north of nearest power-line segment	8.55	5.47			

Notes: OR = odds ratio, and μT = microTesla. The editors changed specific distances to power lines, originally expressed in feet, to meters.
*Adjustment varied for the ORs shown. For the demographic ORs, we adjusted for the other demographic variables (e.g., age-adjusted for race and gender). For the distance ORs, the first adjustment shown is for geomagnetic intensity (MG_Intent) and dip angle (DIP); for the second, the adjustment is for declination of magnetic North (DECL). None of the ORs was statistically significant at the $p < .05$ level.
†Distance = perpendicular distance from residence to power line.

point database of cases and controls was created, we calculated distances to power-line segments, with adjustments for the geomagnetic contribution to the total electromagnetic field at each location.

Liboff et al.[24] proposed that the parallel geomagnetic component of the total geomagnetic field intensity is a function of the uniform geomagnetic field with dip angle (α), the angle of elevation (β) between the residence and the nearest power line, the angle (θ) between the power-line axis and magnetic North, and B_τ the total ambient geomagnetic field intensity:

$$B = B_\tau \sin \alpha \cos \beta + B_\tau \cos \alpha \sin \beta \sin \theta.$$

Both the dip angle α and the total field intensity B_τ can be determined with federal GIS databases.[30] We estimated the values for the angles θ (magnetic North) and β (elevation) with GIS functions compiled from the U.S. Geological Survey topographical maps.[31]

The study was based on a sample size of 251 newly diagnosed malignant brain-cancer cases identified from the study area. This sample size permitted detection of a 2-fold risk difference between the study groups for factors with a 10% frequency in the comparison group ($\alpha = 0.05$ and $\beta = 0.10$). We included conventional risk analysis (e.g., multivariate logistic regression) in our analysis testing, and odds ratios (ORs) expressed the risk associated with electromagnetic fields.

Results

The majority of cases and controls were address-matched (86.1% and 84.9%, respectively). The urban and suburban address matching approached 100%. For rural subjects, the GIS (automated) matching was successful for 68% and 78%, respectively, of cases and controls. The mapping assignments and field work verification described earlier were performed for 10 of 17 rural cases that did not address-match initially with 20 of the 36 unmatched controls. Only these field-verified residence locations were included with the data analysis inasmuch as inclusion of the remainder (map assigned only) had no effect on the study results whatsoever.

Logistic regression for risk of brain cancer, posed as a function of the geomagnetic field intensity, was modeled with the single effects for the following variables: distance (perpendicular distance from the residence to the nearest power line); Orient_Decl (orientation declination [θ]); DIP (dip angle for the residence approximated from the World Magnetic Model[30] [WMM] [α]); DECL (declination of magnetic North [again, approximated

Table 2.—Formula Elements (24) Projected from Geographic Information Systems Model

Formula elements	Distance (m) to power lines*					
	< 30	30–61	> 61–152	> 152–305	> 305–610	> 610
Magnetic intensity (μT)						
n	5	3	13	24	36	155
$\bar{x}$	532.03	531.28	532.32	532.29	531.91	532.10
SD	0.445	1.146	0.4785	0.672	0.958	0.821
Dip angle (°)						
n	5	3	13	24	36	155
$\bar{x}$	66.35	65.19	66.07	66.06	66.02	66.050
SD	0.057	0.171	0.060	0.097	0.128	0.113
Orientation (°)†						
n	5	3	13	24	36	155
$\bar{x}$	12.19	45.52	11.92	2.14	–7.06	14.47
SD	70.560	31.546	36.814	54.153	56.398	51.310
Distance (m)						
n	5	3	13	24	36	155
$\bar{x}$	17.32	53.09	103.39	232.34	458.21	2,017.45
SD	8.677	4.490	24.747	46.514	81.790	1,240.867
Declination (°)						
n	5	3	13	24	36	155
$\bar{x}$	–6.57	–6 .44	–6.50	–6.49	–6.57	–6.62
SD	0.0836	0.0390	0.0854	0.0927	0.166	0.185
RELAngle (°)‡						
n	4	2	12	22	29	139
$\bar{x}$	3.72	0.76	0.78	0.77	0.78	0.78
SD	5.745	0.050	0.018	0.017	0.010	0.004

Notes: Cells include sample size for attribute and distance, the mean value, and the standard deviation. *SD* = standard deviation, $\bar{x}$ = mean, μT = microTesla, and *n* = number of cases.
*Distance = perpendicular distance from residence to power line.
†Orientation = degree to north of nearest power-line segment.
‡RELAngle = elevation of residence from a straight line immediately under power lines.

from EPOCH WMM]); MG_Intent (total magnetic intensity, based upon EPOCH WMM [B_τ]); and RELAngle (relative angle between the residences, determined with digital elevation models[31] [β]). Single-effects models indicated statistically significant ORs for both MG_Intent of 1.25 (i.e., 1.02, 1.52 [upper and lower confidence limits, respectively]) and DIP of 4.86 (i.e., 1.13, 20.96 [upper and lower confidence limits, respectively]). None of the other variables provided a statistically significant elevated OR, and only 1 of the other simple-effects ORs exceeded 1.10 (DECL [1.50 {odds ratio}]). A simple stepwise selection led us to suspect an interaction for DIP and DECL, given their effect on MG_Intent when both variables remained in the statistical model. A saturated logistic model, which contained all of the single-effects terms and an interaction term for DIP and DECL (DIP*DECL), did not reveal an optimally parsimonious model; collinearity effects disrupted most model configurations. Furthermore, truncations of distances revealed erratic effects on these variables.

We tested a series of model configurations with combinations of distances (Table 1) and used principal-components analysis to select best representatives for analysis. We evaluated models with MG_Intent and DIP angle present over graduated distances. Odds ratios from simple linear progressions are shown in Table 1, as are sample sizes. Likewise, a single-effects model with DECL was tested. Given the unproductive regression analyses, we decided that simply describing the ICR variables as a function of distance would be informative. We have provided a pair of simple descriptive tables (Tables 2 and 3) that contain values for the ICR formula to describe the distance patterns with the variables for cases and comparison subjects.

Discussion

The results of the current analysis were difficult for us to interpret; nonetheless, they suggest that a substantial improvement in ecologic exposure classification may be discerned for a function of the earth's magnetic field—interacting with that formed by an electrical distribution power line. The fact that this effect may be delimited spatially suggests the presence of spatial "window" effects. The data suggest that the simple distance measures (which have been used in the past for epidemiologic evaluations of the role of electromagnetic fields) were included without an adjustment for the geomagnetic component. This adjustment may demonstrate a role for EMFs in the carcinogenesis of brain tumors via an ICR mechanism.[24]

It is important that one recognize that this work began as a follow-up on reports of cancer clusters—a good public-health practice.[32] One of the initial bases of study was the provision of a prototype for the sort of expedited research that is possible within the frame-

Table 3.—Formula Elements (24) Projected from Geographic Information Systems Model—Comparison Subjects

	Distance (m) to power lines*					
Formula elements	< 30	> 30–61	> 61–152	> 152–305	> 305–610	> 610
Magnetic intensity (μT)						
n	15	12	36	67	116	418
$\bar{x}$	531.79	531.93	531.95	531.94	531.96	531.97
SD	0.761	0.533	0.7760	0.816	0.878	0.835
Dip angle (°)						
n	15	12	36	67	116	418
$\bar{x}$	65.99	66.03	66.02	66.02	66.02	66.03
SD	0.111	0.073	0.106	0.109	0.122	0.116
Orientation (°)†						
n	15	12	36	67	116	418
$\bar{x}$	–13.921	20.71	1.42	–5.83	3.45	7.76
SD	64.754	56.59	51.983	52.878	52.639	53.094
Distance (m)						
n	15	12	36	67	116	418
$\bar{x}$	15.01	41.31	112.14	227.10	457.32	1,797.46
SD	10.535	6.929	21.634	45.004	92.331	1,214.730
Declination (°)						
n	15	12	36	67	116	418
$\bar{x}$	–6.55	–6.62	–6.560	6.59	–6.57	–6.61
SD	0.149	0.134	0.130	0.153	0.149	0.185
RELAngle (°)‡						
n	15	10	30	61	105	376
$\bar{x}$	1.53	0.81	0.79	0.78	0.78	0.78
SD	0.463	0.107	0.037	0.018	0.009	0.004

Notes: Cells include sample size for attribute and distance, the mean value, and the standard deviation. *SD* = standard deviation, $\bar{x}$ = mean, μT = microTesla, and *n* = number of cases.
*Distance = perpendicular distance from residence to power line.
†Orientation = degrees to north of nearest power-line segment.
‡RELAngle = elevation of residence from a straight line immediately under power lines.

work of a population-based cancer-registry system. The method of rapid reporting is an innovation pioneered for environmental and clinical studies in North Carolina.[22,32] Our choice to apply this approach to brain cancer also illustrated the extraordinary value of GIS methods for the assessment of environmental exposures.[33,34] The results from this analysis offer evidence that a metric of EMFs, incorporating a geomagnetic component, may finally provide some clarification on the protracted quandary about EMFs and cancer risk.

* * * * * * * * * * *

Project funding was provided from private foundation funds of the Lineberger Comprehensive Cancer Center at the University of North Carolina–Chapel Hill. Many individuals helped with this study: Carol Hanchette and Robert Chastain of the Geographic Analysis Section of the North Carolina Center for Health Statistics, Department of Environment, Health and Natural Resources; and David Popson, Steven Choy, and Erin White of the North Carolina Central Cancer Registry verified addresses and collected data from field studies. In addition, the assistance of the staff of the participating hospitals (Alamance Regional Medical Center, Cabarrus Regional Medical Center, Central Carolina Hospital, Carolinas Medical Center, Davie Community Hospital, Duke University Medical Center, Highpoint Regional Medical Center, Rowan Memorial Hospital, Moses Cone Memorial Hospital, North Carolina Baptist Hospital, Presbyterian Hospital, University of North Carolina Hospitals and Clinics, and Wesley Long Hospital) is deeply appreciated; they conducted case finding and data collection beyond the levels requested for incidence reporting. The electrical utilities that serve the study area were most cooperative: Carolina Power and Light, Duke Power, Randolph Electricity Management Corporation, Davidson Electricity Management Corporation, and High Point Regional Electric Corporation.

Submitted for publication September 21, 1999; revised; accepted for publication August 1, 2000.

Requests for reprints should be addressed to Tim E. Aldrich, Department of Epidemiology and Biostatistics, University of South Carolina, Norman J. Arnold School of Public Health, Columbia, SC 29208.

* * * * * * * * * * *

References

1. Levin VA, Gustin PH, Leibel S. Neoplasms of the central nervous system. In DeVita VT Jr, Hellman S, Rosenberg SA (Eds). Cancer: Principles and Practice. Philadelphia, PA: J. B. Lippincott Co., 1993; pp 1679–1737 (chapter 48).
2. Thomas TL, Waxweiler RJ. Brain tumors and occupational risk factors: a review. Scand J Work Environ Health 1986; 12:1–15.
3. Davis DL, Hole D, Fox J, et al. International trends in cancer mortality in France, West Germany, Italy, Japan, England, Wales, and the USA. Lancet 1990; 336:474–81.
4. Blair A, Zhan SH. Agricultural health: pesticides and cancer. Health Environ Digest 1992; 5:1–4.
5. Cordier S, Poisson M, Gerin M, et al. Gliomas and exposure to wood preservatives. Br J Ind Med 1988; 45:705–09.
6. Carpenter AV, Flander WD, Fromme E, et al. Chemical exposures and central nervous cancer: a case-control study among workers at two nuclear facilities. Am J Ind Med 1988; 13:351–62.
7. Savitz DA, Loomis DP. Magnetic field exposure in relation to leukemia and brain cancer morality among electric utility workers. Am J Epidemiol 1995; 141:123–34.
8. Guenel P, Nicolau J, Imbernon E, et al. Exposure to 50-Hz electrical field and incidence of leukemia, brain tumors, and other

cancers among French utility workers. Am J Epidemiol 1996; 144:1107–21.
9. Waxweiler RJ, Alexander V, Leffingwell SS, et al. Mortality from brain tumors and other causes in a cohort of petrochemical workers. J Natl Cancer Inst 1983; 870:75–81.
10. Enterline PE. Brain tumors in the chemical industry. J Occup Med 1983; 25:321.
11. Morantz RA, Neuberger JS, Baker LH, et al. Epidemiological findings in a brain-tumor cluster in Western Missouri. J Neurosurg 1985; 62:856–60.
12. Blair A, Malker H, Cantor KP, et al. Cancer among farmers. Scand J Work Environ Health 1985; 11:397–407.
13. Thomas TL, Stolley PD, Stemhagen A, et al. Brain tumor mortality risk among men with electrical and electronic jobs: a case-control study. J Natl Cancer Inst 1987; 79:233–38.
14. Mack W, Preston-Martin S, Peters JM. Astrocytoma risk related to job exposure to electric and magnetic fields. Bioelectromagnetics 1991; 12:57–66.
15. Rodvall Y, Ahlbom A, Spannare B, et al. Glioma and occupational exposure in Sweden: a case-control study. Int J Cancer (in press).
16. Savitz DA, John EM, Kleckner RC. Magnetic field exposure from electronic appliances and childhood cancer. Am J Epidemiol 1990; 131:763–73.
17. Johnson CC, Spitz MR. Childhood nervous system tumors: an assessment of risk associated with paternal occupations involving use, repair or manufacture of electrical and electronic equipment. Int J Epidemiol 1989; 18:756–62.
18. Aldrich TE, Lindsey J, Morris P. Evaluation of Cancer Cluster Reports in North Carolina; Raleigh, NC: Department of Environment, Health and Natural Resources, 1991; CHES study no 56.
19. Aldrich TE, Savitz LA, Witte P. Evaluating clusters of adverse health outcomes. In: Ricketts TC, Savitz LS, Gesler WM, Osborne DN (Eds). Geographic Methods for Health Services Research. Lanham, MD: University Press of America, 1994; 277–302.
20. Laszlo J, Cox E, Angle C. Special article on tumor registries: The Hospital Tumor registry—present status and future prospects. Cancer 1976; 38:395–402.
21. Aldrich TE, Vann D, Moorman PG, et al. Rapid reporting of cancer incidence in population-based study of breast cancer: one constructive use of a central cancer registry. Breast Cancer Res Treat 1995; 35:61–64.
22. Aldrich TE. Research use of central registries. In: Menck H (Ed). Central Cancer Registries—Design Management and Uses. Chur, Switzerland: Harwood Academic Publishers, 1999; 2nd ed.
23. Inskip PD, Linet MS, Heineman EF. Etiology of brain tumors in adults. Epidemiol Rev 1995; 17(2):382–414.
24. Liboff AR, McLeod BR. Power lines and the geomagnetic field. Bioelectromagnetics 1995; 16:227–30.
25. Blackman CF, Benane SG, Elliott DJ, et al. Influences of electromagnetic fields on the efflux of calcium ions from brain tissues *in vitro*: a three-model analysis consistent with the frequency response up to 510 Hz. Bioelectromagnetics 1988; 9:215–28.
26. Bowman JD, Thomas DC, London SJ, et al. Hypothesis: the risk of childhood leukemia is related to combinations of power frequency and static magnetic fields. Bioelectromagnetics 1991; 12:48–59.
27. Liboff AR, Jenrow KA, McLeod BR. ELF-induced proliferation at 511 mG in HSB-2 cell culture S a function of 60-Hz field intensity. In: Blank M (Eds). Electricity and Magnetism in Biology and Medicine. San Francisco, CA: San Francisco Press, 1985.
28. Arc/Info, Environmental Systems Research Institute, Inc. (380 New York Street, Redlands, CA 92373).
29. Transportation Information Management System. Administered by the North Carolina Department of Public Instruction, Raleigh, NC 27601.
30. United States Geological Survey. The Magnetic Fields in the United States (Box 25046, MS-968, Denver Federal Center, CO 80225). Charts GP986F, GP-986I; 1985.
31. United States Geological Survey. 1:24,000 Maps. Washington, DC: U.S. Department of the Interior, 1983.
32. Aldrich TE, Leaverton P. Sentinel indicators for emerging environmental health problems. Ann Rev Public Health 1993; 14:205–17.
33. Ricketts TC, Savitz LA, Osborne DN. Using Geographic Methods to Understand Health Issues. Washington, DC: Agency for Health Care Policy, 1997; publication no 97-NO13.
34. Pinholster G. Effects of Nonionizing electromagnetic radiation. Environ Health Perspect 1993; 101(4):292–95.

View publication stats
