# Supplementary material for: Magnetic Fields and Cancer: Epidemiology, Cellular Biology, and Theranostics
Source: Int J Mol Sci. 2022 Jan 25;23(3):1339. doi: 10.3390/ijms23031339 (PMC8835851; doi:10.3390/ijms23031339)
Supplement: Supplementary file 1 [file ijms-23-01339-s001.zip › Supplementary Data Set S1/MF and Cancer.Data/PDF/3593771486/OCCUPA~2.PDF]

Environmental Research 164 (2018) 467–474

Contents lists available at ScienceDirect

Environmental Research

journal homepage: www.elsevier.com/locate/envres

Occupational extremely low frequency magnetic fields (ELF-MF) exposure and hematolymphopoietic cancers – Swiss National Cohort analysis and updated meta-analysis

Anke Huss[a,b,*], Adrian Spoerri[b], Matthias Egger[b,c], Hans Kromhout[a], Roel Vermeulen[a,d], for the Swiss National Cohort

[a] Institute for Risk Assessment Sciences, Utrecht University, PO Box 80178, 3508 TD Utrecht, The Netherlands
[b] Institute of Social and Preventive Medicine (ISPM), University of Bern, Switzerland
[c] School of Social and Community Medicine, University of Bristol, United Kingdom
[d] Julius Centre for Public Health Sciences and Primary Care, University Medical Centre, Utrecht, The Netherlands

ABSTRACT

Purpose: Previous studies have examined risks of leukaemia and selected lymphoid malignancies in workers exposed to extremely low frequency magnetic fields (ELF-MF). Most studies evaluated hematolymphopoietic malignancies as a combined category, but some analyses suggested that effects may be contained to some specific leukaemia or lymphoma subtypes, with inconsistent results.
Methods: We examined exposure to ELF-MF and mortality 1990–2008 from different types of hematolymphopoietic cancers in the Swiss National Cohort, using a job exposure matrix for occupations recorded at censuses 1990 and 2000. We analysed 3.1 million workers exposed at different levels to ELF-MF: ever-high, only-medium, only-low exposure using Cox proportional hazard models. We evaluated risk of death from acute myeloid leukaemia (AML), chronic myeloid leukaemia, lymphoid leukaemia, diffuse large B-cell lymphomas, follicular lymphoma, Waldenström's macroglobulinemia, multiple myeloma and Hodgkin lymphoma.
Results: Mortality from hematolymphopoietic cancers was not associated with exposure to ELF-MF with the exception of an increase in ever-high exposed men of myeloid leukaemias (HR 1.31, 95% CI 1.02–1.67), and AML (HR 1.26, 95%CI 0.93–1.70). If workers had been high exposed during their vocational training and at both censuses, these HR increased to 2.24 (95%CI 0.91–5.53) and 2.75 (95%CI 1.11–6.83), respectively.
Conclusions: Our analysis provided no convincing evidence for an increased risk of death from a range of hematolymphopoietic cancers in workers exposed to high or medium levels of ELF magnetic fields. However, we observed an increased risk of acute myeloid leukaemia in workers exposed to high levels for a longer duration. Observed risks are in line with meta-analysed previous reports on ELF-MF exposure and AML risk, with a summary relative risk of 1.21 (95%CI 1.08–1.37).

1. Introduction

In 2001, the International Agency for Research on Cancer classified extremely-low frequency magnetic fields (ELF-MF) as “possibly carcinogenic”, based on the observation of increased risks of leukaemia in children exposed to these fields. For adults, ELF-MF exposures at work can be represent the major determinant of personal exposure. van Tongeren et al. (2004) Epidemiological studies addressing occupational ELF-MF exposure and risk of leukaemia in adults have yielded slightly increased risks in exposed workers: a meta-analysis from 2008 (Kheifets et al., 2008) reported summary relative risks (sRR) of 1.16 (95%CI 1.11–1.22) for leukaemia, based on 56 study reports. Several studies have suggested that stronger effects may be observed for some of the disease subtypes. Feychting et al. (1997); Floderus et al. (1999); Sorahan (2014) For myeloid neoplasms, the mentioned meta-analysis (Kheifets et al., 2008) reported sRRs for acute myeloid leukaemia (AML) of 1.23 (95%CI 1.08–1.41) and for chronic myeloid leukaemia (CML) of 1.22 (95%CI 0.98–1.52). For acute lymphocytic leukaemia (ALL), the sRR was 1.37 (95%CI 1.11–1.69) and for chronic lymphocytic leukaemia (CLL) 1.35 (95%CI 1.10–1.65). In general, however, no clear exposure-response pattern emerged from the studies that evaluated exposure levels included in this meta-analysis (Kheifets et al.,

* Corresponding author at: Institute for Risk Assessment Sciences, Utrecht University, The Netherlands.
E-mail address: a.huss@uu.nl (A. Huss).

https://doi.org/10.1016/j.envres.2018.03.022
Received 15 November 2017; Received in revised form 26 February 2018; Accepted 12 March 2018
Available online 24 March 2018
0013-9351/ © 2018 Elsevier Inc. All rights reserved.

2008).

Since the meta-analysis from 2008 (Kheifets et al., 2008), several publications appeared, but they reported inconsistent results. A large study in electric utility workers from England found an increased risk for ALL but not for CLL, AML or CML (Sorahan, 2014). In contrast, a study of the Dutch general population observed an increased risk for AML, but not for ALL, CLL and CML (Koeman et al., 2014). No evidence of an increased risk emerged from a very large study performed in the Nordic Occupational Cancer Cohort evaluating risk of AML (Talibov et al., 2015). Regarding other lymphoid malignancies in exposed workers, the results were also inconsistent: the risk of follicular lymphoma in ever-high exposed workers was increased in the Dutch study (Koeman et al., 2014), and an Australian case-control study found a slightly increased risk of non-Hodgkin lymphoma cases (Karipidis et al., 2007). Electrical workers who had worked for 20 years or longer had a slightly increased risk of non-Hodgkin lymphoma compared to persons with shorter duration of exposure in a large US-American cohort of utility workers, but risks were not elevated for Hodgkin's disease or multiple myeloma (Schroeder and Savitz, 1997). Increased risks of non-Hodgkin lymphoma also emerged from a Canadian case-control study in workers of electrical industries (Band et al., 2004).

We used a job exposure matrix approach to evaluate the possible association between occupational exposure to ELF-MF and mortality from different types of hematolymphopoietic cancers in the Swiss National Cohort (SNC), a longitudinal study of mortality of the entire population of Switzerland. We also performed a meta-analysis to examine the association of occupational exposure to ELF-MF with AML and to evaluate in how far heterogeneity of results could be explained with exposure-assessment related characteristics of the individual studies.

2. Methods

Described in detail elsewhere (Bopp et al., 2009; Spoerri et al., 2010), the SNC is a longitudinal study based on national censuses. First, censuses 1990 and 2000 were linked using deterministic and probabilistic record linkage methods. Key variables were sex, date of birth, place of residence, marital status, religion, nationality and date of birth. In a second step the census data were linked to the mortality and emigration registries. Coverage of the census 2000 was virtually complete with 98.6% (Renaud, 2004), and 94% of all deaths between 1991 and 2008 could be linked to a census record.

Linkage of records is less complete in younger persons and we therefore excluded persons younger than 30 years at time of entry of the 1990 census. The cantonal ethics committees of Bern and Zurich approved of the study. More information can be found under www.swissnationalcohort.ch.

2.1. Exposure and outcome information

We analysed causes of death from different malignant neoplasms of the lymphoid and haematopoietic tissue, recorded anywhere on the death certificate. We used codes of the International Classification of Diseases and Related Health Problems, versions 8 and 10 (ICD8/10) for this purpose, a list of all ICD8 and 10 codes is given in Table 1. ICD8 was used in Switzerland until 1994 and ICD10 was introduced in 1995. As a negative control outcome, we also evaluated lung cancer (ICD10 code C34 and ICD8 code 162).

The Swiss censuses included information about occupation (i.e. job title at the time point of the census) and highest qualification obtained. About 18,000 individual occupations were listed which were translated into the International Standard Classification of Occupations from 1988 (ISCO88) by the Federal Statistical Office. In contrast to an earlier publication (Huss et al., 2015), we excluded occupational codes that provided only as major group codes (e.g. ISCO88 code 3000). We assigned intensity of the magnetic field exposure (low, medium, high) to

Table 1
ICD 8 and ICD 10 codes.

	ICD8	ICD10
Any hematolymphopoietic cancer	200–209	C81-C96
All types of leukaemias (except lymphoid, new definition)	205–207	C92-C95
All types of leukaemias (including lymphoid, old definition)	204–207	C91-C95
Myeloid leukaemias	205	C92
AML	205.0	C92.0
CML	205.1	C92.1
Lymphomas		
Non-Hodgkin Lymphomas	200, 202, 204	C82-C85 + C88 + C91
Lymphoid leukaemias	204	C91
ALL	204.0	C91.0
CLL	204.1	C91.1
B-cell lymphomas		
Follicular Lymphoma	202.0	C82
Diffuse large B-cell lymphoma	–	C83.3
Waldenström's macroglobulinemia	–	C83.0 + C88.0
Multiple myeloma	203	C90
Hodgkin lymphoma	201	C81
Lung cancer	162	C34

Analysed causes of death as registered anywhere on the death certificate. Codes are given as International Classification of Diseases and Related Health Problems, versions 8 or 10 (ICD8/10).

ISCO88 occupational codes and vocational training (or highest qualification obtained) using a modified version of a previously published job exposure matrix (Bowman et al., 2007; Huss et al., 2013; Koeman et al., 2013). In this JEM, the median intensity of the exposures corresponded to approximately 0.11, 0.19 and 0.52 μT for low, medium and high exposure, respectively (Koeman et al., 2013). High exposed occupations are usually those where workers are placed in close proximity to machines that use a lot of energy, examples include train drivers, pilots, welders or machine operators (see also list of ISCO88 unit groups of “high” exposed occupations in eTable 1). All homemakers as well as people without an occupation at the time of the census were assigned low exposure. The censuses also provide information on the highest vocational training a person received, which is also coded according to ISCO88.

2.2. Statistical analysis

We used Cox proportional hazard models to evaluate the association between exposure to ELF-MF and mortality from hematolymphopoietic cancers. We used age as the underlying time scale and adjusted analyses for sex, nationality, educational level, language region and civil status. For the main analysis, participants entered the risk set on 4 December 1990, the day of the 1990 census. The observation time ended on 31st December 2008, the date of emigration or death, whichever came first. We also evaluated the two censuses separately to assess if observed patterns were consistent over time: In the analysis of census 1990, participants entered the risk set on 4 Dec 1990, but the observation time ended at the latest on 3 December 2000. For the analysis of census 2000, persons entered the risk set on 4 December 2000 and observation time ended on 31 December 2008. Two outcomes, diffuse large B-cell lymphoma and Waldenström's macroglobulinemia were only introduced in ICD10. For these outcomes participants entered the risk set on 1 January 1995, the date of the introduction of ICD10 in Switzerland. Women work less often in occupations with high exposure levels than men and may perform different tasks within a certain occupation, which is why several previous publications reported risk estimates restricted to men (Sorahan, 2014; Koeman et al., 2014; Schroeder and Savitz, 1997; Band et al., 2004). We therefore stratified the main analyses by sex.

ELF-MF exposure was analysed in three categories, as ever-high,

Table 2
Exposure levels and characteristics of the study population.

	Exposure level	1990 Low	1990 Medium	1990 High	2000 Low	2000 Medium	2000 High
Average age at entry	Mean (SD)	45 (9)	45 (9)	45 (10)	45 (9)	45 (9)	44 (9)
% women		54	34	8	52	36	7
% educ. level	low	23	30	27	14	22	17
	medium	53	57	60	55	63	67
	high	24	13	13	31	15	16
% foreign nationals		16	29	30	19	29	27
Language region %	German	73	74	71	73	75	71
	French	23	22	24	23	21	24
	Italian	4	4	5	4	4	5
Civil status	Single	9	11	10	17	17	18
	Married	76	74	79	73	71	73
	Widowed	5	4	3	1	2	1
	Divorced	10	11	8	9	10	8

Exposure levels: the median intensity of the exposures corresponded to approximately 0.11, 0.19 and 0.52 µT for low, medium and high exposure. Educational level was classified as low (compulsory schooling or less), medium (vocational training) or high (tertiary education).

only-medium or only-low, stratifying analyses to account for differences in baseline risk over time ("strata" command in Stata). In the main analysis, we used the earliest recorded exposure. If the number of ever-high exposed cases was less than five, we collapsed the ever-high and only-medium exposure categories into a combined "exposed" category. If the number of exposed cases was below five, no risk estimates were calculated. For persons who could be assigned an exposure at both censuses, we further classified exposure across censuses. Due to low numbers, we combined workers exposed at only-medium or ever-high levels into two exposure categories of "ever medium or high" in either 1990 or in 2000, or "always medium or high" (exposed in both censuses) and compared these to workers exposed "always low" (not exposed in either census).

Educational level was classified as low (compulsory schooling or less), medium (vocational training) or high (tertiary education). The censuses combined retirees and disabled persons in a single group. Because the outcomes of interest may be a reason to receive a disability pension we excluded pensioners from analyses. We restricted our analysis to economically active people (aged up to 65 for men and up to 62 for women), and in a sensitivity analyses we set all homemakers and persons seeking a job to low exposure. We explored potential confounding by other chemical exposures at work, in particular solvents, pesticides and herbicides, metals (Koeman et al., 2014; Matheson et al., 2005), and risk of electrical shocks at work (Huss et al., 2013). These exposures were again assigned to occupational codes using dedicated JEMs, and adjusted for in the main model.

We tested models for the proportionality assumption using statistical tests based on Schoenfeld residuals, and the assumption was met for all exposure variables.

2.3. Meta-analysis

We performed a meta-analysis on the association between occupational exposure to ELF-MF and risk of AML. We searched EMBASE and MEDLINE and additionally screened a specialist data base (www.EMF-Portal.com). Search terms and a flow chart are provided in the Supplementary Materials eFig. 1. We included peer-reviewed papers published in English language until September 2017 if they reported risk estimates of AML in association with occupational exposure to ELF-MF or occupational titles grouped as "electrical workers". Data were extracted from the individual studies by A.H., and in case of doubt, discussed and questions resolved with R.V. If risk estimates were presented for more than two ELF-MF exposure levels (e.g. high vs low and medium vs low), we pooled risk estimates across all presented exposure categories (except the reference group), using a fixed effects within-study meta-analysis. In this way we obtained a risk estimate for "any" ELF-MF exposure. In addition, we extracted risk estimates if authors had reported higher ELF-MF exposure category ("highest vs lowest"). We preferred adjusted risk estimates over unadjusted ones.

We calculated summary risk estimates with a random effects meta-analysis, and an I^2 value, which gives an indication of heterogeneity between the studies. We used meta-regression to evaluate if study results differed depending on whether exposure had been assessed as ELF-MF exposure (e.g. assigned with a job exposure matrix) or was based on job titles grouped as "electrical occupations". We also evaluated if study results differed depending on whether the cumulative exposure of the full occupational history (i.e. all occupations a person had) was used to assign ELF-MF exposure, or just some points in time (e.g. at a census). Finally, we checked if results differed depending on if the original studies had evaluated incident AML cases, or mortality.

All analyses were performed using Stata version 12 (StataCorp, College Station, Texas, USA). Results are reported as hazard ratios (HRs) with 95% confidence intervals (95%CI).

3. Results

The census 1990 contained information on 3.055 million persons aged between 30 and 62 years (women) or 30 and 65 years (men). Of these, we excluded 263,619 due to missing occupational information and 89,935 who received a pension. This left us with 2.701 million persons for the analysis restricted to 1990–2000, of which 559,803 reported to be homemakers, or seeking a job. The 2000 census had information on 3.471 million persons aged between 30 and 62 (women) or 30–65 (men). We excluded 854,706 persons with missing occupational information and 100,963 pensioners, which left us with 2.515 million person for the analysis restricted to 2000–2008, of which 498,524 reported to be homemakers. A total of 3.147 million persons contributed to the main analysis from 1990 to 2008, and 1.010 million persons contributed to the duration analysis of both censuses combined. Characteristics of our study population are given in Table 2.

No clear exposure-response relationships emerged with increasing intensity or duration of exposure in the main analysis combining the two censuses (Table 3), the separate census analyses (Supplementary material eTable 2) or when we combined the exposures across the censuses for those people who could be assigned an exposure level for both censuses (Supplementary material eTable 3). HRs were slightly elevated for myeloid leukaemias with an HR for ever-high exposed men of 1.31 (95% CI 1.02–1.67) in the combined census analysis, primarily due to a slightly elevated risk of AML with an HR of 1.26 (95%CI 0.93–1.70) (Table 4). The increase in risks were restricted to men, HR for women could not be assessed for most outcomes, as there were too few "ever high" exposed women for most of the outcomes (Table 4).

Table 3
Risk estimates for ever-exposure to ELF-MF and mortality from hematolymphopoietic cancers, follow-up time 1990–2008.

	Exposure level	N	HR 1990–2008 Sex-adjusted	HR 1990–2008 Fully adjusted
**Any hematolymphopoietic cancer**	Low	5223	Referent	Referent
	Medium	1738	**1.07 (1.01–1.13)**	1.04 (0.99–1.10)
	High	287	**1.12 (1.00–1.27)**	1.09 (0.97–1.23)
**leukaemias (except lymphoid)**	Low	1301	Referent	Referent
	Medium	402	1.00 (0.89–1.12)	0.98 (0.87–1.10)
	High	78	**1.26 (1.00–1.58)**	1.22 (0.97–1.54)
**leukaemias (incl. lymphoid)**	Low	1910	Referent	Referent
	Medium	617	1.04 (0.95–1.14)	1.02 (0.93–1.12)
	High	113	1.20 (0.99–1.46)	1.17 (0.97–1.42)
**Myeloid leukaemias**	Low	1161	Referent	Referent
	Medium	360	1.00 (0.89–1.13)	0.98 (0.87–1.10)
	High	72	**1.31 (1.03–1.66)**	1.26 (0.99–1.60)
**AML**	Low	814	Referent	Referent
	Medium	248	0.99 (0.86–1.14)	0.97 (0.84–1.13)
	High	48	1.27 (0.95–1.70)	1.24 (0.92–1.66)
**CML**	Low	256	Referent	Referent
	Medium	83	1.04 (0.81–1.34)	1.01 (0.78–1.30)
	High	15	1.20 (0.71–2.02)	1.14 (0.67–1.93)
**Non-Hodgkin Lymphomas**	Low	2598	Referent	Referent
	Medium	888	**1.09 (1.01–1.18)**	1.07 (0.99–1.15)
	High	142	1.10 (0.93–1.30)	1.06 (0.89–1.26)
**Lymphoid leukaemias**	Low	613	Referent	Referent
	Medium	215	1.11 (0.95–1.30)	1.09 (0.93–1.27)
	High	35	1.09 (0.77–1.53)	1.06 (0.75–1.50)
**ALL**	Low	109	Referent	Referent
	Exposed[a]	39	1.04 (0.72–1.50)	1.07 (0.73–1.55)
**CLL**	Low	458	Referent	Referent
	Medium	157	1.07 (0.89–1.29)	1.04 (0.87–1.25)
	High	27	1.08 (0.73–1.60)	1.04 (0.71–1.55)
**Follicular Lymphoma**	Low	65	Referent	Referent
	Exposed[a]	14[a]	0.62 (0.35–1.11)	0.60 (0.33–1.08)
**Diffuse large B-cell lymphoma**	Low	95	Referent	Referent
	Exposed[a]	34	1.02 (0.69–1.51)	1.00 (0.67–1.50)
**Waldenström's macroglub.**	Low	93	Referent	Referent
	Medium	16	0.54 (0.32–0.91)	0.49 (0.29–0.85)
	High	8	1.58 (0.76–3.26)	1.42 (0.68–2.94)
**Multiple myeloma**	Low	1142	Referent	Referent
	Medium	375	1.05 (0.93–1.18)	1.04 (0.92–1.17)
	High	54	0.99 (0.75–1.30)	0.96 (0.73–1.27)
**Hodgkin lymphoma**	Low	200	Referent	Referent
	Medium	61	1.00 (0.75–1.34)	0.99 (0.74–1.33)
	High	12	1.27 (0.71–2.29)	1.25 (0.69–2.26)
**Lung cancer**	Low	14854	Referent	Referent
	Medium	5801	**1.21 (1.18–1.25)**	**1.11 (1.07–1.14)**
	High	1079	**1.35 (1.27–1.43)**	**1.21 (1.14–1.29)**

Sex-adjusted HR calculated taking age as underlying time scale and adjusted for sex. Fully adjusted models additionally adjusted for nationality, educational level, language region and civil status.

[a] exposed: If there were less than 5 cases in the high exposed group, medium and high exposed were collapsed to an "exposed" category.

There were only few outcomes with enough exposed cases to evaluate if workers had been high exposed at the census 1990 and 2000 and additionally at vocational training (Supplementary material eTable 4). The HR for myeloid leukaemia in men who had been both high exposed in 1990 and in 2000 was 1.51 (95%CI 0.66–3.46), compared to being only low exposed in both censuses. For AML, the corresponding HR was 1.85 (95%CI 0.80–4.24). If workers had additionally been high exposed during vocational training, the HRs increased to 2.24 (95%CI 0.91–5.53) and 2.75 (95%CI 1.11–6.83) for myeloid leukaemias and AML in men, respectively. These risk estimates were based on 6 high exposed cases of myeloid leukaemia during both censuses and the same amount of AML cases; and on 5 cases each if workers had additionally been high exposed during vocational training. Adjusting the HR for myeloid leukaemia and AML for potential other occupational exposures (pesticides, solvents or risk of shocks) did not materially affect HRs. HRs slightly increased when we adjusted for metal exposure HR for myeloid leukaemia in ever-high ELF-MF exposed men was 1.38 (95%CI 1.06–1.79). HRs were slightly attenuated when we set all homemakers to low exposure; HRs for ever-high exposed persons were 1.25 (95%CI 0.98–1.59) and 1.22 (95%CI 0.91–1.63) for myeloid leukaemia and AML, respectively. Finally, elevated risks were also observed for lung cancer mortality, ever medium/ high ELF-MF exposed workers had an HR of 1.25 (95%CI 1.14–1.38), and in always medium/ high exposed workers this HR was also 1.25 (95%CI 1.15–1.36), see also Table 3.

The forest plot of the meta-analysis on occupational ELF-MF exposure and AML risk is shown in Fig. 1. Including our study results, the overall summary relative risk (sRR) was 1.21 (95%CI 1.08–1.37). In some studies, cumulative ELF-MF exposure had been evaluated over the full occupational history. In this group, the sRR was 1.51 (95%CI 1.06–1.37); studies that had evaluated ELF-MF exposure at just one or two points in time did not identify increased risks of AML, the sRR was 0.97 (95%CI 0.83–1.13), this difference was statistically significant ($p = 0.03$). Studies on electrical occupations resulted in a sRR of 1.32 (95%CI 1.14–1.53). Summary RR were not statistically significantly different if authors had assessed incidence or mortality ($p = 0.7$).

Table 4
Risk estimates for ever-exposure to ELF-MF and mortality from hematolymphopoietic cancers, follow-up time 1990–2008, stratified by sex.

	Exposure level	N	HR fully adj., men	N	HR fully adj., women
**Any hematolymphopoietic cancer**	Low	3817	Referent	1406	Referent
	Medium	1322	1.04 (0.97–1.11)	416	1.05 (0.94–1.18)
	High	274	1.10 (0.97–1.24)	13	0.95 (0.55–1.65)
**leukaemias (except lymphoid)**	Low	919	Referent	382	Referent
	Medium	305	0.99 (0.87–1.13)	99[a]	0.92 (0.73–1.17)
	High	76	**1.26 (1.00–1.60)**	–	–
**leukaemias (incl. lymphoid)**	Low	1395	Referent	515	Referent
	Medium	487	1.05 (0.94–1.16)	134[a]	0.92 (0.76–1.13)
	High	109	1.19 (0.98–1.45)	–	–
**Myeloid leukaemias**	Low	815	Referent	352	Referent
	Medium	271	0.99 (0.86–1.14)	92[a]	0.93 (0.73–1.18)
	High	70	**1.31 (1.02–1.67)**	–	–
**AML**	Low	562	Referent	252	Referent
	Medium	180	0.96 (0.81–1.14)	70[a]	1.03 (0.78–1.35)
	High	46	1.26 (0.93–1.70)	–	–
**CML**	Low	184	Referent	72	Referent
	Medium	65	1.06 (0.79–1.41)	18[a]	0.78 (0.45–1.34)
	High	15	1.23 (0.73–2.10)	–	–
**Non-Hodgkin Lymphomas**	Low	1951	Referent	647	Referent
	Medium	682	1.05 (0.96–1.14)	209[a]	1.11 (0.95–1.31)
	High	139	1.08 (0.91–1.29)	–	–
**Lymphoid leukaemias**	Low	480	Referent	133	Referent
	Medium	182	1.14 (0.96–1.35)	35[a]	0.93 (0.63–1.37)
	High	33	1.05 (0.73–1.49)	–	–
**ALL**	Low	67	Referent	42	Referent
	Exposed	30[a]	1.21 (0.78–1.89)	9[a]	0.83 (0.39–1.75)
**CLL**	Low	377	Referent	81	Referent
	Medium	135	1.06 (0.87–1.29)	23[a]	1.00 (0.61–1.62)
	High	26	1.03 (0.69–1.54)	–	–
**Follicular Lymphoma**	Low	41	Referent	24	Referent
	Exposed[a]	10[a]	0.62 (0.31–1.25)	< 5[a]	–
**Diffuse large B-cell lymphoma**	Low	59	Referent	36	Referent
	Exposed[a]	26[a]	1.10 (0.69–1.76)	8[a]	0.76 (0.35–1.69)
**Waldenström's macroglub.**	Low	75	Referent	18	Referent
	Medium	15	0.56 (0.32–0.98)	< 5[a]	–
	High	7	1.30 (0.60–2.84)	–	–
**Multiple myeloma**	Low	808	Referent	334	Referent
	Medium	279	1.04 (0.91–1.20)	96	0.98 (0.77–1.24)
	High	48	0.91 (0.68–1.22)	6	1.70 (0.75–3.84)
**Hodgkin lymphoma**	Low	141	Referent	58	Referent
	Medium	49	1.02 (0.73–1.42)	16[a]	0.97 (0.54–1.74)
	High	12	1.25 (0.69–2.27)	–	–

Exposure as ever only low, ever medium or ever high as reported in census 1990 or census 2000. Model 1: Adjusted for nationality, educational level, language region and civil status, taking age as underlying time scale, in men. Model 2: Same as Model 1, but in women.

[a] For less than 5 high exposed cases, risk estimates of medium and high exposed were combined. In the case of fewer than 5 medium or high exposed cases, corresponding risk estimates were omitted.

4. Discussion

We assigned levels of exposure to ELF-MF to occupations reported in the census 1990 and 2000 in the Swiss population and evaluated risks of a range of different types of hematolymphopoietic malignancies by ELF-MF exposure. We observed increased risks of few malignancies in ever-high exposed workers, but associations generally failed to reach conventional levels of statistical significance. Also no statistically significant increased risks emerged when we analysed information from two censuses, indicating longer duration of exposure. For myeloid leukaemia and AML, we observed an increased risk in men ever-high exposed at both censuses. This HR further increased in men who had additionally been high exposed during vocational training, but HRs were based on few exposed cases. Our findings are in line with meta-analysed results on previous studies evaluating occupational exposure to ELF-MF and risk of AML. Overall, results indicate that studies evaluating cumulative exposure were more likely to observe increased risks. Risks could not be assessed in women due to low numbers of exposed persons.

Strengths of our analysis include that we could study the entire Swiss population aged 30 years or older, with almost 20 years of follow-up during which a large number of deaths from hematolymphopoietic malignancies occurred. Occupational information (i.e. job title) was collected at two censuses ten years apart. Note that the ELF-MF JEM does not have a time axis, and that it is unclear if exposure levels may have changed during the time frame of the study (1990–2008). We were unable to account for effects of actual cumulative exposure to ELF-MF during one's lifetime, or to assess the effect of timing of exposure on the outcomes. However, when we assigned exposure to vocational training as well as to both censuses, increased risk for AML emerged in high-exposed workers, and our results are in line those from studies that identified high exposed workers and evaluated the full occupational history of study participants (eFig. 2).

Many previous publications have reported associations of ELF-MF on a combined but heterogeneous group of hematolymphopoietic malignancies. In this case, it is not possible to assess if associations with the exposure are restricted to subgroups of leukaemia or lymphoma. In a previous meta-analysis, associations of magnetic field exposure were stronger with subtypes like CLL or ALL than with hematolymphopoietic cancers overall. Since this meta-analysis, several new studies have been published that addressed occupational ELF-MF exposure and risk of AML (Sorahan, 2014; Koeman et al., 2014; Talibov et al., 2015). Because our results indicated that possibly high ELF-MF exposure in combination with duration of exposure may represent a risk factor for

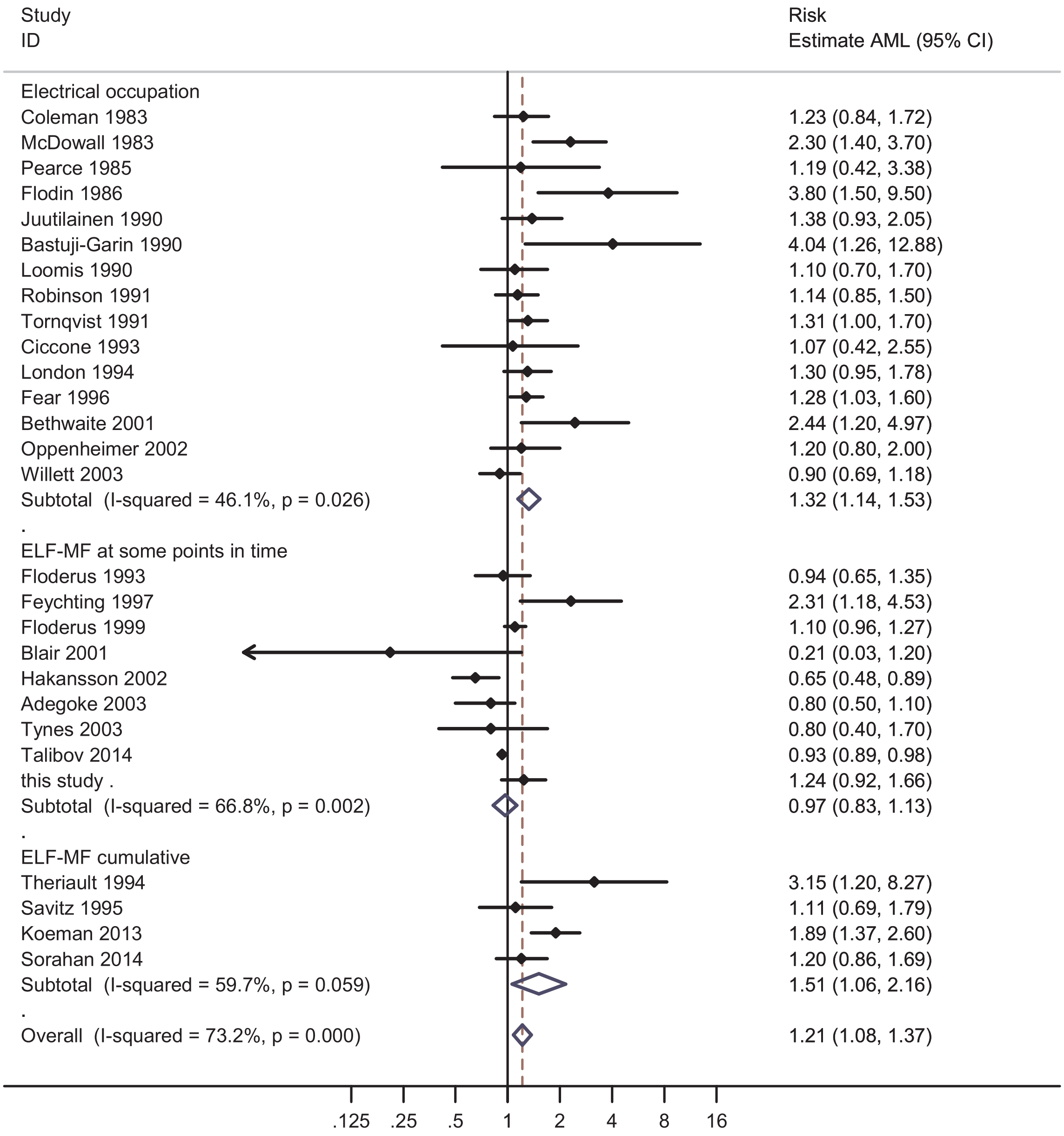

Fig. 1. Meta-analysis of occupational exposure to ELF-MF and risk of acute myeloid leukaemia. Overview over studies analysing electrical occupations, or (any) occupational ELF-MF exposure compared to background, and risk of acute myelogenous leukaemia. Most studies evaluated job codes as "electrical occupations" or assigned magnetic field exposures to job codes using a job exposure matrix. Often this was based on few points in time, e.g. at baseline, census, or a primary held job. Four studies evaluated the full occupational history and assigned cumulative magnetic field exposure to all occupations study participants had held.

AML, we performed a systematic search of studies and meta-analysed studies that had reported results for AML. As shown in Fig. 1, the combined sRR was 1.21 (95%CI 1.08–1.37), and our results of ever-high exposed workers was well in line with this estimate.

One previous study reported that residential ELF-MF exposure was associated with a subtype of AML (AML with multi-lineage dysplasia) but not with total AML (Wong et al., 2009). Incidence of AML subtypes has been shown to vary substantially by sex, age and race or ethnicity group (Dores et al., 2012). Given that these subtypes of AML were generally not reported, we could not evaluate whether a differential distribution of subtypes across the meta-analysed studies could be underlying the observed heterogeneity. Nevertheless, given that the thus far evaluated hematolymphopoietic cancers represent a diverse group of diseases, our findings underline that future investigation into the separate entities are more likely to be more informative compared to grouping them together.

Some publications also summarised all non-Hodgkin lymphomas as a group (Karipidis et al., 2007; Schroeder and Savitz, 1997; Linet et al., 1993; Mester et al., 2006; Miller et al., 1996; t Mannetje et al., 2016; Villeneuve et al., 2000; Zheng et al., 2002). Subtypes of non-Hodgkin lymphoma such as follicular lymphoma or diffuse large B-cell lymphoma or other lymphomas, such as multiple myeloma, or Hodgkin lymphoma, were less frequently analysed (Floderus et al., 1999; Schroeder and Savitz, 1997; t Mannetje et al., 2016; Zheng et al., 2002; Coggon et al., 1986; Fear et al., 1996; Milham, 1985). Two previous studies reported statistically significant increased risk of follicular lymphoma in workers occupationally exposed to magnetic fields (Koeman et al., 2014; Mester et al., 2006). Another study observed increased risks of follicular lymphoma in workers of electric power systems industries (Band et al., 2004) and hypothesised a possible association with magnetic field exposure. Follicular lymphoma was also increased in male and in female workers in the telephone communication industry (Zheng et al., 2002), but another study provided no evidence of an increased risk of follicular lymphoma in electrical or

electronics workers or in welders (t Mannetje et al., 2016), two groups expected to have relatively high exposure to magnetic fields. In our analysis we only observed four ever-high exposed men with follicular lymphoma, resulting in an HR of 1.51 (95% CI 0.53–4.26, compared to only low exposure), which does not provide clear evidence for or against such an association.

Another limitation is the use of mortality and not incidence data. Survival from hematolymphopoietic cancers is not equal across subtypes (De Angelis et al., 2014), and in addition, survival has changed over the last decades more for some than for other leukaemia and lymphoma subtypes (Sant et al., 2014). We accounted for changes in baseline risk over time but cannot exclude that differences in survival among different groups of hematolymphopoietic cancers may have affected our ability to detect an association with occupational exposure to ELF magnetic fields. Reassuringly, in line with our results on AML, a previous meta-analysis on occupational exposure to ELF-magnetic fields and leukaemia did not provide evidence that studies evaluating incidence or mortality provided deviating results (Kheifets et al., 2008).

We censored people who were already retired at the time point of the census 1990 because we could not assign any meaningful exposure to this part of the population. With a retirement age at the time of the census 1990 of 65 for men and 62 for women, follow-up for men was up to a maximum age of 83 and for women up to age 80. Median age at death for all of the evaluated outcomes was well below that age, with the exception of CLL and Waldenström's macroglobulinemia, where the median age at death in our population was 81 years. Censoring all people at retirement age would be primarily expected to result in a loss of power to detect a potential association. It would only raise a problem if occupational magnetic field exposure at work had a lagged effect on hematolymphopoietic cancers. Earlier studies that have evaluated effect of timing of exposure, however, rather indicated that risk estimates increased slightly if exposure was restricted to the 10–15 years before diagnosis (Schroeder and Savitz, 1997; Floderus et al., 1993). Due to low numbers of exposed women, only few studies had enough power for an informative analysis (i.e. more than just very few exposed female cases) and reported differences in risk estimates across men and women (Floderus et al., 1999; Hakansson et al., 2002; Willett et al., 2003), but there was heterogeneity also between the studies. One study indicated that exposed men had an increased risk of ALL, but women had an increased risk of CLL (Floderus et al., 1999). Another study, however, indicated an increased risk of ALL only in women (Willett et al., 2003). Heterogeneity of results between men and women may be a consequence from differences in exposure levels within the same occupational groups (Forssen et al., 2004), from differences in susceptibility of the diseases in question, or just chance findings. To ensure comparability with previous studies we therefore stratified our results by sex, but encountered the same problem as many of the other studies.

There is concern of potential confounding by lifestyle factors such as smoking. Information on such factors was not available in our data base. However, previous studies that had information on lifestyle and that could thus account for potential confounding generally did not report material effects on risk estimates (Koeman et al., 2014; Floderus et al., 1993), suggesting that the correlation of the confounders with ELF-MF exposure is not likely to be very strong. In our analysis, lung cancer mortality among ELF-MF exposed workers was elevated in medium and high exposed workers, but mortality from AML was only elevated in the high exposed workers. This nevertheless means that residual confounding by smoking may indeed have affected our risk estimates, although we are unfortunately not able to evaluate its extent. We tried to account for other exposures that workers may have been exposed to, but found these also to only have very small effects on our risk estimates. This was in line with other studies that accounted for other chemical exposures at work, such as pesticides, benzene or solvents (Koeman et al., 2014; Floderus et al., 1993; Bastuji-Garin et al., 1990) who found risk estimates not materially affected. No mechanism has been shown how exposure to ELF-MF could cause hematolymphopoietic malignancies, although it has been hypothesised that exposure to ELF-MF generates reactive oxygen species (ROS), which in turn may eventually trigger genotoxic events (Consales et al., 2012).

Overall, our analysis is in line with previous reports stating that if there is any risk of occupational magnetic field exposure on hematolymphopoietic cancers, it appears to be restricted to some subtypes of these cancers, and it appears to be small.

Acknowledgements

We would like to thank Judith Lupatsch for help with inspection of the ISCO88 codes, and Joseph Bowman for making the original ELF-MF JEM available, and the Swiss Federal Statistical Office, whose support made the Swiss National Cohort possible.

All authors declare that they have no conflicts of interest.

This study was supported by ZonMW within the programme Electromagnetic Fields and Health Research, The Netherlands (grants No. 85800001 and 85200001). The Swiss National Cohort is supported by the Swiss National Science Foundation (grant No. 33CS30_134273/1).

Appendix A. Supporting information

Supplementary data associated with this article can be found in the online version at http://dx.doi.org/10.1016/j.envres.2018.03.022.

References

Band, P.R., Le, N.D., Fang, R., Gallagher, R., 2004. Identification of occupational cancer risks in British Columbia: a population-based case-control study of 769 cases of non-Hodgkin's lymphoma analyzed by histopathology subtypes. J. Occup. Environ. Med. 46, 479–489.

Bastuji-Garin, S., Richardson, S., Zittoun, R., 1990. Acute leukaemia in workers exposed to electromagnetic fields. Eur. J. Cancer 26, 1119–1120.

Bopp, M., Spoerri, A., Zwahlen, M., Gutzwiller, F., Paccaud, F., Braun-Fahrlander, C., et al., 2009. Cohort profile: the Swiss National Cohort–a longitudinal study of 6.8 million people. Int. J. Epidemiol. 38, 379–384.

Bowman, J.D., Touchstone, J.A., Yost, M.G., 2007. A population-based job exposure matrix for power-frequency magnetic fields. J. Occup. Environ. Hyg. 4, 715–728.

Coggon, D., Pannett, B., Osmond, C., Acheson, E.D., 1986. A survey of cancer and occupation in young and middle aged men. II. non-respiratory cancers. Br. J. Ind. Med. 43, 381–386.

Consales, C., Merla, C., Marino, C., Benassi, B., 2012. Electromagnetic fields, oxidative stress, and neurodegeneration. Int. J. Cell Biol. 2012, 683897.

De Angelis, R., Sant, M., Coleman, M.P., Francisci, S., Baili, P., Pierannunzio, D., et al., 2014. Cancer survival in Europe 1999–2007 by country and age: results of EUROCARE-5-a population-based study. Lancet Oncol. 15, 23–34.

Dores, G.M., Devesa, S.S., Curtis, R.E., Linet, M.S., Morton, L.M., 2012. Acute leukemia incidence and patient survival among children and adults in the United States, 2001–2007. Blood 119, 34–43.

Fear, N.T., Roman, E., Carpenter, L.M., Newton, R., Bull, D., 1996. Cancer in electrical workers: an analysis of cancer registrations in England, 1981-87. Br. J. Cancer 73, 935–939.

Feychting, M., Forssen, U., Floderus, B., 1997. Occupational and residential magnetic field exposure and leukemia and central nervous system tumors. Epidemiology 8, 384–389.

Floderus, B., Persson, T., Stenlund, C., Wennberg, A., Ost, A., Knave, B., 1993. Occupational exposure to electromagnetic fields in relation to leukemia and brain tumors: a case-control study in Sweden. Cancer Causes Control 4, 465–476.

Floderus, B., Stenlund, C., Persson, T., 1999. Occupational magnetic field exposure and site-specific cancer incidence: a Swedish cohort study. Cancer Causes Control 10, 323–332.

Forssen, U.M., Mezei, G., Nise, G., Feychting, M., 2004. Occupational magnetic field exposure among women in Stockholm County, Sweden. Occup. Environ. Med. 61, 594–602.

Hakansson, N., Floderus, B., Gustavsson, P., Johansen, C., Olsen, J.H., 2002. Cancer incidence and magnetic field exposure in industries using resistance welding in Sweden. Occup. Environ. Med. 59, 481–486.

Huss, A., Vermeulen, R., Bowman, J.D., Kheifets, L., Kromhout, H., 2013. Electric shocks at work in Europe: development of a job exposure matrix. Occup. Environ. Med. 70, 261–267.

Huss, A., Spoerri, A., Egger, M., Kromhout, H., Vermeulen, R., 2015. Occupational exposure to magnetic fields and electric shocks and risk of ALS: the Swiss National Cohort. Amyotroph. Lateral Scler. Frontotemporal Degener. 16, 80–85.

Karipidis, K., Benke, G., Sim, M., Fritschi, L., Yost, M., Armstrong, B., et al., 2007. Occupational exposure to power frequency magnetic fields and risk of non-Hodgkin

lymphoma. Occup. Environ. Med. 64, 25–29.
Kheifets, L., Monroe, J., Vergara, X., Mezei, G., Afifi, A.A., 2008. Occupational electromagnetic fields and leukemia and brain cancer: an update to two meta-analyses. J. Occup. Environ. Med. 50, 677–688.
Koeman, T., Slottje, P., Kromhout, H., Schouten, L.J., Goldbohm, R.A., van den Brandt, P.A., et al., 2013. Occupational exposure to extremely low-frequency magnetic fields and cardiovascular disease mortality in a prospective cohort study. Occup. Environ. Med. 70, 402–407.
Koeman, T., van den Brandt, P.A., Slottje, P., Schouten, L.J., Goldbohm, R.A., Kromhout, H., et al., 2014. Occupational extremely low-frequency magnetic field exposure and selected cancer outcomes in a prospective Dutch cohort. Cancer Causes Control 25, 203–214.
Linet, M.S., Malker, H.S., McLaughlin, J.K., Weiner, J.A., Blot, W.J., Ericsson, J.L., et al., 1993. non-Hodgkin's lymphoma and occupation in Sweden: a registry based analysis. Br. J. Ind. Med. 50, 79–84.
Matheson, M.C., Benke, G., Raven, J., Sim, M.R., Kromhout, H., Vermeulen, R., et al., 2005. Biological dust exposure in the workplace is a risk factor for chronic obstructive pulmonary disease. Thorax 60, 645–651.
Mester, B., Nieters, A., Deeg, E., Elsner, G., Becker, N., Seidler, A., 2006. Occupation and malignant lymphoma: a population based case control study in Germany. Occup. Environ. Med. 63, 17–26.
Milham Jr., S., 1985. Mortality in workers exposed to electromagnetic fields. Environ. Health Perspect. 62, 297–300.
Miller, A.B., ToT, Agnew, D.A., Wall, C., Green, L.M., 1996. Leukemia following occupational exposure to 60-Hz electric and magnetic fields among Ontario electric utility workers. Am. J. Epidemiol. 144, 150–160.
Renaud A.: Coverage Estimation for the Swiss Population Census 2000. Swiss Federal Statistical Office, 2005. Accessible at: www.bfs.admin.ch/bfs/en/home/statistics/catalogues-databases/publications.assetdetail.341896.html.
Sant, M., Minicozzi, P., Mounier, M., Anderson, L.A., Brenner, H., Holleczek, B., et al., 2014. Survival for haematological malignancies in Europe between 1997 and 2008 by region and age: results of EUROCARE-5, a population-based study. Lancet Oncol. 15, 931–942.
Schroeder, J.C., Savitz, D.A., 1997. Lymphoma and multiple myeloma mortality in relation to magnetic field exposure among electric utility workers. Am. J. Ind. Med. 32, 392–402.
Sorahan, T., 2014. Magnetic fields and leukaemia risks in UK electricity supply workers. Occup. Med. 64, 150–156.
Spoerri, A., Zwahlen, M., Egger, M., Bopp, M., 2010. The Swiss National Cohort: a unique database for national and international researchers. Int. J. Public Health 55, 239–242.
t Mannetje, A., De Roos, A.J., Boffetta, P., Vermeulen, R., Benke, G., Fritschi, L., et al., 2016. Occupation and risk of non-hodgkin lymphoma and its subtypes: a pooled analysis from the interlymph consortium. Environ. Health Perspect. 124, 396–405.
Talibov, M., Guxens, M., Pukkala, E., Huss, A., Kromhout, H., Slottje, P., et al., 2015. Occupational exposure to extremely low-frequency magnetic fields and electrical shocks and acute myeloid leukemia in four Nordic countries. Cancer Causes Control 26, 1079–1085.
van Tongeren, M., Mee, T., Whatmough, P., Broad, L., Maslanyj, M., Allen, S., et al., 2004. Assessing occupational and domestic ELF magnetic field exposure in the uk adult brain tumour study: results of a feasibility study. Radiat. Prot. Dosim. 108, 227–236.
Villeneuve, P.J., Agnew, D.A., Miller, A.B., Corey, P.N., Purdham, J.T., 2000. Leukemia in electric utility workers: the evaluation of alternative indices of exposure to 60 Hz electric and magnetic fields. Am. J. Ind. Med. 37, 607–617.
Willett, E.V., McKinney, P.A., Fear, N.T., Cartwright, R.A., Roman, E., 2003. Occupational exposure to electromagnetic fields and acute leukaemia: analysis of a case-control study. Occup. Environ. Med. 60, 577–583.
Wong, O., Harris, F., Yiying, W., Hua, F., 2009. A hospital-based case-control study of acute myeloid leukemia in Shanghai: analysis of personal characteristics, lifestyle and environmental risk factors by subtypes of the WHO classification. Regul. Toxicol. Pharmacol. 55, 340–352.
Zheng, T., Blair, A., Zhang, Y., Weisenburger, D.D., Zahm, S.H., 2002. Occupation and risk of non-Hodgkin's lymphoma and chronic lymphocytic leukemia. J. Occup. Environ. Med. 44, 469–474.
