# Supplementary material for: Magnetic Fields and Cancer: Epidemiology, Cellular Biology, and Theranostics
Source: Int J Mol Sci. 2022 Jan 25;23(3):1339. doi: 10.3390/ijms23031339 (PMC8835851; doi:10.3390/ijms23031339)
Supplement: Supplementary file 1 [file ijms-23-01339-s001.zip › Supplementary Data Set S1/MF and Cancer.Data/PDF/3637400466/Rogers2021_Article_IntraoperativeMRIForBrainTu.pdf]

Journal of Neuro-Oncology (2021) 151:479–490
https://doi.org/10.1007/s11060-020-03667-6

TOPIC REVIEW

Intraoperative MRI for Brain Tumors

Cara Marie Rogers[1] · Pamela S. Jones[2] · Jeffrey S. Weinberg[3]

Received: 27 May 2020 / Accepted: 23 November 2020
© Springer Science+Business Media, LLC, part of Springer Nature 2021

Abstract

Introduction The use of intraoperative imaging has been a critical tool in the neurosurgeon's armamentarium and is of particular benefit during tumor surgery. This article summarizes the history of its development, implementation, clinical experience and future directions.

Methods We reviewed the literature focusing on the development and clinical experience with intraoperative MRI. Utilizing the authors' personal experience as well as evidence from the literature, we present an overview of the utility of MRI during neurosurgery.

Results In the 1990s, the first description of using a low field MRI in the operating room was published describing the additional benefit provided by improved resolution of MRI as compared to ultrasound. Since then, implementation has varied in magnetic field strength and in configuration from floor mounted to ceiling mounted units as well as those that are accessible to the operating room for use during surgery and via an outpatient entrance to use for diagnostic imaging. The experience shows utility of this technique for increasing extent of resection for low and high grade tumors as well as preventing injury to important structures while incorporating techniques such as intraoperative monitoring.

Conclusion This article reviews the history of intraoperative MRI and presents a review of the literature revealing the successful implementation of this technology and benefits noted for the patient and the surgeon.

Keywords Surgery · Brain tumor · MRI · Glioma · Glioblastoma · Stereotaxy · Stereotactic biopsy · Pituitary tumor · Pediatric

Introduction

The emergence and innovation of intraoperative magnetic resonance imaging (iMRI) over the last several decades has been of particular benefit to the practice of neurosurgical oncology. While a variety of surgical guidance and imaging modalities exist, iMRI provides the highest quality evaluation of surgical execution and assessment of the dynamic changes that occur during surgery in near real time. Understanding the clinical benefits of iMRI as well as its potential limitations is important in the consideration of its utilization.

✉ Jeffrey S. Weinberg
jweinberg@mdanderson.org

1 Department of Neurosurgery, Virginia Tech Carilion, Roanoke, VA, USA

2 Department of Neurosurgery, Massachusetts General Hospital, Harvard Medical School, Boston, MA, USA

3 Department of Neurosurgery, University of Texas M.D. Anderson Cancer Center, Houston, TX, USA

The evolution of iMRI systems

Intraoperative MRI was first introduced to the neurosurgical community in the mid 1990s when General Electric and Brigham and Women's Hospital (BWH) in Boston, Massachusetts developed the Signa 0.5 Tesla Magnetic Resonance Therapy (MRT) Unit [1–3]. The vision for the MRT was fueled by a desire for an open-configuration MRI that would allow surgery to be performed in the scanner with continual image guidance. All MRI systems at the time were closed-configuration units that did not allow access to the patient while in the scanner. Fundamental alterations in the engineering and physics of the magnet and coils had to be made in order for this open-configuration model to come to fruition. The "double donut" magnet system was ultimately chosen in which two superconducting magnets and coils were placed in separate but communicating vertically oriented units. A vertical cleft between the coils allowed the surgeon access to the patient while acquiring concurrent images [1, 2, 4–6].

The open-configuration concept was designed to provide surgical access to the patient within the scanner and allow for real-time imaging during the operation; that is to be able to perform surgery while the scan was being completed and with the patient in the same position, thereby reducing the inefficiency of moving the patient multiple times especially when many scans are needed. Maintaining the patient in a static position is thought to improve the accuracy of registration by reducing the risk of patient movement from the surgical position to the scanning position. However the spatial access to the patient in the open-configuration system is significantly restricted. The MRT unit provided a relatively small, 56 cm wide area of access, which created unique challenges with the fixed location of the space and this narrow corridor for the surgeon to stand. Another downside to this concept is that all surgical instruments are required to be MR compatible (non-ferromagnetic) to prevent the attraction of the instruments to the magnet and to also limit radiofrequency noise, which would result in imaging artifact. MR compatible instrumentation adds an additional cost to the implementation of this technology [1, 5, 7–9].

The next iteration of iMRI technology came in the form of magnets with ultra low-field strengths of 0.12 T and 0.15 T. This technological advancement was desired because the smaller magnet allowed for reduced size and increased portability. Medtronic introduced PoleStar in 2000 with the N-10 iMRI containing a 0.12 T magnet and later the N-15 iMRI with a 0.15 T magnet [10]. Benefits of open-configuration ultra low-field systems are their compact structure, mobility, and ease of use [4]. Medical centers can build a magnet shielding cabinet (MSC) in a conventional operating room to house the mobile unit, which is then taken out, draped, and positioned over or around the patient when an image is needed [10]. The investment required for the early iMRI systems was prohibitive for many hospital systems, but the ultra low-field system required much less extensive operative suite remodeling and minimal alterations to the operative equipment and instruments, allowing for a lower implementation cost. The most significant downside of the lower field open-configuration systems, is that they produce a lower quality image when compared to the high field diagnostic MRI scanners, thereby potentially limiting their operative benefit [2–4, 8, 9, 11, 12].

The limitations posed by low-field, open-configuration units and the desire to have higher quality imaging led to the conversion to a closed-configuration system, high-field strength MRI starting in the early 2000's. High-field iMRI models are available in 1.5 T and 3 T strengths and provide image resolution and definition that are equivalent to diagnostic MRI scanners and have the ability to provide advanced imaging protocols including diffusion weighted, perfusion, spectroscopy, and diffusion tensor imaging. Since these units are closed-configuration, there is no access to the patient during scanning, and they require a unique operating room setup in which the operative field is separate from the scanner. These can be stationary systems in the same or nearby room to the operative suite into which the patient is transported, or ceiling mounted systems that are stored in a shielded alcove and then brought into the operating room on a rail system and over to the operative table [13]. Siemens Sonata is a 1.5 T MRI that was first introduced into the operating room 2002 and utilized a rotating table with a base that swiveled the table from an operative position into the bore of the magnet [14]. The IMRIS system which launched in 2005, is a 1.5 T magnet designed on a ceiling-mounted rail system that can move the scanner to the patient. The rail system can be tailored to allow the scanner to be stationed in a separate room for use as a diagnostic scanner, and also move between operating rooms for use in multiple concurrent surgeries [10, 15]. Since the operative field is outside of the magnetic field, this allows the team to use regular instruments and equipment as well as full access to the patient while in the operative position. These high-field systems can be arranged solely as intraoperative scanners that are designed with operative workflow and efficiency in mind, or for dual functionality for surgery and diagnostics to optimize utilization and lessen the economic burden of the investment [1, 2, 4, 9, 13, 16, 17].

Throughout this evolution of iMRI technology, the consensus within the neurosurgical community became to prioritize high quality imaging with optimal spatial and temporal resolution. Therefore, many of the technological advancements in iMRI in recent years have focused on higher field strength with advancement in image acquisition technology [18]. A focus has also been on customization, so that an iMRI suite can be configured to fit the unique needs of each hospital. A system should be chosen and a suite designed to maximize utility, efficiency, and incorporate all the additional modalities desired by the surgeons. Incorporating the armamentarium of tools available in a conventional operating room was important as well. Suites can be equipped with microscopes, endoscopes, MRI-compatible stereotactic systems, robotic systems, laser technologies, and even hybrid suites with MR and X-ray capabilities have allowed integration of angiography. While the technology was created originally for cranial oncologic purposes, the technology is now being utilized in vascular, functional, and spinal procedures which makes it significantly more versatile and potentially cost effective for a hospital system [5, 10, 18].

Benefits

Accounting for the brain shift phenomenon

Intraoperative MRI provides near real-time information about the dynamic changes that occur in surgery known as

the brain shift phenomenon [1, 19]. The anatomic position of structures shift within the cranial vault due to a myriad of forces. Administration of anesthetic medications, osmotic agents, changes in overall fluid volume status, and hyperventilation can alter the physiological properties of the tissue. By opening the skull and dura, the brain tissue is exposed to atmospheric pressure resulting in a loss of the forces that act on a closed system leading to gravitational shift. Size of the craniotomy, physical manipulation of the tissue, egress of cerebrospinal fluid from opening of the dura or entry into a ventricle, and removal of a space-occupying lesion can also impact the anatomic configuration of structures [20, 21]. This cumulative distortion is nonlinear and can be more profound in patients with hydrocephalus, parenchymal atrophy, edema, or lesions producing significant mass effect. Gering et al. investigated the extent and direction of brain shift and found that it is a continuous dynamic process that impacts specific regions of brain tissue differently [22]. Nimsky and colleagues showed that shift varies greatly throughout the cranial vault with up to 24 mm of shift at the cortical surface and beyond 3 mm at the deep tumor border [21]. The accuracy of stereotactic navigation that utilizes preoperative imaging can therefore decrease precipitously due to these anatomic shifts during surgery. Stereotactic navigation simply becomes an ineffectual tool when the surgeon recognizes the loss in accuracy due to the aforementioned factors, yet can become a dangerous instrument that misleads a surgeon if the inaccuracy is overlooked. Shahar et al. found that in tumors adjacent to the corticospinal tract, there was a mean intraoperative shift in the tumor to corticospinal tract distance of 3.18 mm [23]. If the surgeon does not appreciate this shift, eloquent tissue can easily be violated leading to devastating consequences. Once the accuracy of navigation is effectually lost, updating the image via an intraoperative scan, allows the surgeon to restore their orientation and understanding of the spatial anatomy. Multiple studies have concluded that the only way to provide accurate intraoperative guidance is to obtain serial intraoperative imaging, which is provided in greatest detail by MRI [21–25].

Patient position has also been shown to impact brain position [26]. In a typical operating room, stereotactic navigation is registered to the patient using a preoperative diagnostic image, that was obtained with the patient in a supine position. Surgical position, dictated by the tumor location, may not be supine, thereby negatively impacting the accuracy of navigation. Therefore, utilizing iMRI may enhance the accuracy of navigation as the patient is registered to a scan performed in the operative position.

Extent of resection and survival

The success of oncologic surgeries is increasingly being defined by the extent of resection (EOR) with a goal of gross total resection (GTR) or maximal safe cytoreduction. Lacroix et al. found that the only modifiable factor that impacted survival in glioblastoma patients, was the EOR. This benefit was found when 89% or more of the enhancing disease was resected, with the most significant survival advantage of over 4 months when 93% or more of the enhancing tumor was removed [27]. Sanai et al. showed a significant survival advantage even at subtotal EORs as low as of 78% in newly diagnosed glioblastomas [28]. Oppenlander et al. found that in recurrent glioblastoma, an 80% EOR was found to provide an improvement in overall survival (OS) [29]. Scherer et al. reported that in low-grade gliomas, GTR was associated with significantly longer progression free survivals (PFS) and OS [30]. Roelz et al. also showed a significant survival advantage in diffuse low grade gliomas, with 5 year OS of 82% with initial maximal safe resection versus only 54% when biopsied [31]. Ultimately, maximal safe resection has been shown by numerous studies to have a significant prognostic benefit by improving OS and has become the foundation for a multidisciplinary approach to low- and high-grade brain tumors [32–35].

The unparalleled ability of MRI to define pathological tissue has proved superior to surgeon assessment and other imaging modalities. Hatiboglu et al. found that with gliomas, even when the surgeon feels that the goals of surgery have been achieved, iMRI demonstrated unexpected residual tumor resulting in additional resection in 47% of cases [36]. Golub et al. showed in a meta-analysis published in 2020, that iMRI was superior to conventional navigation in achieving GTR. Roder et al. found in 117 glioblastoma patients reviewed retrospectively, that iMRI was superior to both conventional and 5-ALA guided surgery in achieving smaller residual volumes and more frequent GTRs. Coburger et al. found that the highest sensitivity for detection of residual tumor in low grade gliomas was with iMRI when compared to linear array ultrasound and conventional ultrasound [37]. MRI is therefore considered the gold standard when determining the EOR [8, 33]. Utilization of iMRI has the benefit of optimizing the EOR in an oncologic surgery by providing the surgeon with a more accurate assessment of the tissue at the surgical margins than direct visualization, tactile feedback, or even ultrasound. This allows for intraoperative evaluation as to the EOR and aids the surgeon in removal of any safely accessible residual pathology. Surgeons are able to navigate with the newly obtained intraoperative images, guiding them to the residual area of interest [5, 36, 38, 39].

Utilization of iMRI has been shown in numerous studies to correlate with greater EOR and improved patient PFS and even OS. Bohinski et al. and Knauth et al. both showed that iMRI identified residual resectable tumor in 53% of cases thereby leading to greater EOR in glioma patients [17, 40]. In a randomized, prospective study of iMRI as compared to conventional surgery, Senft et al. found that, among the

58 patients enrolled, more patients in the iMRI group had complete tumor resection (23/24 [96%]) than in the control group (17/25 [68%], $p = 0.023$) [41]. Senft et al. also demonstrated in 103 glioma patients that iMRI led to further resection in 30.1% of cases and that OS was superior in those with complete resections [42]. Zhang et al. reported that patients with nonfunctioning pituitary adenomas undergoing endoscopic transphenoidal resection, use of iMRI to confirm GTR resulted in extended PFS [43]. In Golub's meta analysis, the improved rates of GTR seen with iMRI correlated to prolonged PFS and OS in high-grade gliomas [44]. This benefit can be applied to a wide range of pathologies including surgically curable tumors as well as infiltrative tumors where optimal cytoreduction may provide a meaningful survival advantage or reduction in the need for toxic adjuvant therapies [8, 16, 38, 45].

Identifying surgical complications

The use of iMRI can potentially mitigate surgical complications because it allows for visualization beyond the surgically exposed surface. Hemorrhage deep to the resection cavity can be identified and evacuated prior to closure [46]. Areas of tissue ischemia can be visualized on an intraoperative scan and medical interventions potentially initiated to prevent further infarct [47]. Development of hydrocephalus could be recognized and treated with external ventricular drainage if appropriate. Theoretically, complications can be identified intraoperatively allowing for earlier interventions and thereby reducing neurological compromise and the need for potential reoperations [16, 48, 49].

Clinical applications (Literature summary presented in Table 1)

Low grade gliomas

The utility of iMRI in the resection of low grade gliomas is arguably the most evident. As mentioned previously, gross total or maximal safe resection of low grade gliomas is associated with significantly longer PFS and OS. This benefit is so profound in low grade gliomas, that reoperation to remove unintended residual is oftentimes warranted [30, 31, 50]. Utilization of iMRI allows the surgeon to ensure that the goals of surgery have been accomplished prior to closing, thereby eliminating the potential need for a reoperation for residual disease. Yahanda and colleagues reported that they performed further resection in almost half (49.7%) of their low grade gliomas cases due to findings discovered on iMRI, which led to a GTR in 64% of these cases [50].

Resection of low grade gliomas with the use of iMRI is also extremely valuable because these tumors oftentimes have an appearance and texture similar to normal brain tissue. This characteristic can make defining the border between normal and abnormal tissue more challenging. In low grade gliomas located in ineloquent tissue, a generous resection beyond the borders typically defined by the FLAIR sequences may be a viable option to ensure maximal cytoreduction [51]. However, achieving GTR in tumors near eloquent regions can become difficult unless there are obvious anatomic borders such as sulci or fissures to guide the surgeon. If these borders do not exist, then a surgeon in a conventional operating room may have to choose between a more conservative approach with the potential of leaving residual tumor behind versus an aggressive approach with the potential of causing neurological injury. The use of iMRI guidance allows the surgeon to err on the side of caution, reimage when the stereotactic navigation becomes unreliable secondary to shift, utilize neurophysiologic monitoring, and identify remaining pathological tissue that may not be obvious on gross examination [9, 33, 52].

High grade gliomas

Intraoperative MRI can be an invaluable tool in high grade gliomas located within or adjacent to eloquent regions. The gross appearance of high grade gliomas is typically distinguishable from the surrounding parenchyma and defining the plane between normal and abnormal simpler than with low grade gliomas. However, manipulation of this plane could be fraught with danger when dealing with eloquent structures. Traction on eloquent tissue or deviating out of the plane into eloquent brain could lead to devastating neurological consequences. Any prognostic benefit that an aggressive resection offers is negated by a postoperative neurological deficit. In addition to reduced quality of life and functional status, Rahman et al. found that survival was reduced by over 5 months whether the deficit was transient or persistent [53]. Therefore, a fine balance must be targeted between maximal resection of disease and preservation of neurological function [35, 40, 54–56].

Pediatric tumors

The benefits of iMRI in the resection of pediatric tumors are similar to those in low grade gliomas since complete resection when feasible is imperative in this population. Maximal safe resection with ideally GTR is the standard of care in children because it leads to significant improvements in PFS and OS. In pediatric low-grade gliomas, 10 year PFS is only 50% with subtotal resection versus 85% with GTR [57]. Multiple studies have demonstrated the utility of intraoperative MRI in aiding to achieve an increase in the EOR of pediatric brain tumors [58, 59]. Giordano and colleagues found that iMRI led to the identification and removal of

Table 1 Primary literature on clinical applications of intraoperative MRI

Author	Year	Application	Analysis	Magnet strength	Findings
High-grade glioma					
Hatiboglu et al. [36]	2009	Enhancing and non-enhancing gliomas	Prospective, single center	1.5 T	EOR increased from 76% to 96% ($p < 0.001$). In cases with GTR, 52% of them (15/29) were helped by additional resection following iMRI
Senft et al. [41]	2011	Contrast-enhancing gliomas	Randomized prospective, single center	1.5 T, 3.0 T	Enrolled 58 pts., more patients in the iMRI group had complete tumor resection (23/24 [96%]) than in the control group (17/25 [68%], $p = 0.023$)
Napolitano et al. [39]	2014	Glioblastoma	Prospective, single center	3.0 T	In 58 cases with iMRI and 38 without (control), there was a significant difference in reaching larger EOR from use of an iMRI as compared to control ($P = 0.049$)
Coburger et al. [83]	2015	Glioblastoma	Prospective, single center	1.5 T	Significant increase of EOR when combining 5-ALA and iMRI compared to use of iMRI alone ($p < 0.004$)
Low-grade glioma					
Maldaun et al. [81]	2014	Eloquent glioma	Prospective, single center	1.5 T	In 42 cases, further resection after iMRI was performed in 17 cases (40.5%); the mean EOR in these cases increased from 56% to 67% after further resection.
Coburger et al. [37]	2015	Low-grade glioma	Prospective, single center	1.5 T	In 13 cases, the highest sensitivity for residual tumor detection was found in iMRI (83%), followed by linear array ultrasound (79%), followed by conventional ultrasound (21%)
Coburger et al. [52]	2016	Low-grade glioma	Retrospective, multi-center	0.2 T to 1.5 T	In 288 cases, use of high-field iMRI was significantly associated with GTR, and GTR was significantly associated with improved PFS
Pediatrics					
Shah et al. [61]	2012	Pediatric brain lesions	Retrospective, single center	1.5 T	In 42 patients in iMRI group and 103 in conventional group, there were 8 reoperations (7.77%) in the conventional group compared with none in the iMRI group in 2 weeks post-operatively, which trended toward significance in a 1-tailed test ($p = 0.06$)
Giordano et al. [38]	2017	Various pediatric pathologies	Retrospective, single center	1.5 T	iMRI allowed localization of residual lesion and attainment of GTR through further resection in 18% of craniopharyngioma cases (4/22) and 27% of low grade gliomas (4/15).
Day, EL and RM Scott [58]	2019	Pediatric brain tumors	Retrospective, single center	1.5 T	In 53 cases, iMRI technology was useful in 38 (64.4%) of the 59 cases, most frequently for its help in assessing extent of resection.

Table 1 (continued)

Author	Year	Application	Analysis	Magnet strength	Findings
Karsy et al. [59]	2019	Pediatric brain tumors	Retrospective, multi-center		In 280 patients who underwent resection using iMRI, 131 (46.8%) had some residual tumor and underwent additional resection; determining the impact of iMRI on EOR and outcomes remains challenging because iMRI use varies among providers nationally
Pituitary					
Coburger et al. [70]	2014	Pituitary adenoma	Retrospective, single center	1.5 T	In 143 cases, 63 with iMRI and 76 without (control), GTR was achieved significantly more often in the iMRI group (91%) than in control group (73%) ($p < 0.034$). Planned STR showed higher mean volumes of residual tumor in control group; there was non-significant trend towards improved PFS in iMRI group
Sylvester et al.[72]	2015	Pituitary adenoma	Retrospective, single center	1.5 T	In 446 cases, iMRI and endoscopy modalities combined increased extent of resection status (OR 2.05, 95% CI 1.21–3.46) compared to conventional transsphenoidal microsurgery
Zaidi et al. [69]	2016	Pituitary adenoma	Retrospective, single center	3.0 T	In 23 cases, iMRI helped convert 1 STR and 4 NTRs to GTRs, increasing the number of GTRs from 12 (60%) to 16 (80%)
Other					
Bernays et al. [66]	2002	Stereotactic biopsy	Prospective, single center	0.5 T	All biopsy samples had pathological tissue and 111/114 (97.4%) yielded a specific neuropathological diagnosis
Thomas et al. [73]	2016	LITT	Retrospective, single center	1.5 T	In 8 patients with newly diagnosed GBM group, the median PFS and the median OS after the procedure were 2 months and 8 months, respectively. No patient demonstrated radiographic shrinkage of the tumor on follow-up imaging. In the 13 patients with recurrent GBM, 5 demonstrated a response to LITT, with radiographic shrinkage of the tumor following ablation. The median PFS was 5 months, and the median OS was greater than 7 months.

residual tumor in 27% of glioma and 18% of craniopharyngioma cases resulting in GTR that would otherwise have been subtotal. They also reported that iMRI prompted further resection of unintended residual in 21–52% of cases improving their reoperation rate of 7–14% without iMRI to 0% [16, 38, 60]. Shah et al. showed that utilization of iMRI potentially reduces the need for early reoperation in pediatric lesions with 7.7% of their conventional resections requiring reoperation in the first 2 weeks versus none when iMRI was used [61].

A practical benefit of iMRI in the pediatric population is that the volumetric scan needed for registration of navigation, can be performed at the beginning of the case. This eliminates the need for an additional scan prior to surgery that may be distressing for children and may require general anesthesia, particularly in the infant population.

Stereotactic biopsy

Stereotactic biopsies are employed to obtain tissue pathology and the diagnostic yield is reported in multiple large series to be between 90 and 95%. This leaves 5–10% of patients without the necessary diagnosis to guide their treatment [62–65]. Lesions that are smaller, deeper, lack enhancement, or have extensive necrosis or large cystic components have the lowest diagnostic yields. Failures to obtain pathological tissue can be secondary to faulty targeting or the result of brain shift [62]. The use of iMRI allows the surgeon to verify placement of the cannula and adjust intraoperatively if necessary to ensure the lesion will be sampled appropriately [25]. Studies utilizing iMRI to obtain stereotactic brain biopsies have reported diagnostic yields over 97% [66, 67]. While iMRI may not be essential to all biopsies, it can be particularly advantageous in the biopsy of small deep lesions that pose the greatest risk of nondiagnostic yield with traditional stereotactic procedures.

Pituitary and skull base tumors

A growing body of evidence suggests that certain patients with skull base tumors may benefit from the guidance of an intraoperative MRI scan. It can be useful in identifying remnants of tumor that may be difficult to visualize in narrow operative corridors unique to these surgeries. Tumors frequently distort the normal anatomy and then the shift of structures due to debulking may further disorient the surgeon [8]. An intraoperative scan may aid the surgeon in determining whether they have accomplished their goals of surgery in terms of debulking of the mass or decompression of critical structures. Although it may not be advantageous for all skull base surgeries, one group found that up to 15.7% of their operations benefited from the guidance of an iMRI by improving EOR or avoiding gratuitous exploration [68]. Multiple studies have established the efficacy of iMRI in transphenoidal resection of pituitary adenomas through identification and removal of residual tumor thereby increasing the rate of GTR, and, in some cases, showing a trend towards improved PFS with these increased resection rates [69–72].

Laser interstitial thermal therapy

Laser interstitial thermal therapy (LITT) is an emerging technique that can be utilized in the treatment of a multitude of brain lesions. LITT is a form of thermal ablation that involves the stereotactic-guided placement of an MRI-compatible optical fiber that delivers focused light energy into a lesion to cause thermal injury and immediate necrosis with near real time MR thermometry guidance. The procedure requires a laser, an MRI, and a linked workstation that produces thermal maps that allow the surgeon to monitor the pattern of thermal injury. There are currently two commercial platforms available in the United States including Visualase from Medtronic and NeuroBlate from Monteris Medical. The main differences between these systems are the wavelength of their laser, cooling mechanism, and patterns of heat production and distribution. The NeuroBlate system utilizes a 1064 nm diode laser that emits light in the form of optical pulses, is cooled with CO_2, and has both a diffusing and side-firing tip. The Visualase system utilizes a 980 nm diode laser that emits light in the form of a continuous wave, is cooled with saline, and offers a diffusing tip.

LITT can be used in a spectrum of cranial oncologic conditions including both benign and malignant tumors, primary and metastatic tumors, recurrent tumors, and treatment effect secondary to radiation. It is a minimally invasive procedure requiring shorter hospitalizations and recovery times than open surgery. It is an excellent alternative for surgically inaccessible lesions and patients who are not ideal medical candidates for more invasive procedures [73–75]. LITT is also increasingly being used in the treatment of spinal oncologic tumors and in epilepsy but discussion of these uses is not in the scope of this paper.

The convenience and efficiency of iMRI technology is truly apparent when performing LITT procedures. Utilization of an iMRI streamlines the LITT process substantially by allowing the catheter to be placed with conventional stereotactic guidance in the same room as the iMRI. However, hospital systems that do not have an iMRI suite can use a diagnostic scanner for LITT. They can create an MRI-compatible, sterile operating field within their diagnostic MRI room and place the catheter using the ClearPoint system which utilizes a percutaneous SmartFrame mount [76, 77]. Alternatively, the catheter can be placed in a traditional operating room followed by transporting the patient to a diagnostic MRI for ablation. However, there are several

potential drawbacks of this setup. There is the theoretical risk to sterility and catheter stability with transporting a patient from an operating room to a diagnostic scanner. This setup is also less ideal if the catheter is found to be malpositioned or if multiple trajectories are desired as this may require multiple trips back to the operating room [78, 79].

Special considerations

Surgical adjuncts

Within the intraoperative MRI suite, surgeons are able to combine all surgical adjuncts utilized in a conventional operating room. Hatiboglu et al. showed that the use electrocorticography in the iMRI suite with cortical and subcortical mapping is easily performed without adverse effects or difficulty [80]. Multiple studies have demonstrated that combining awake craniotomies for mapping of eloquent regions with iMRI guidance is a viable and safe option and aids in greater EOR and prognosis. High field iMRI systems also allow for the integration of functional imaging such as diffusion tensor images (DTI) to ensure that eloquent cortical and subcortical structures are respected throughout the procedure, protecting them from unintended damage [56, 81, 82].

The use of 5-aminolevulinic acid (5-ALA) in the resection of high-grade gliomas for visualization of enhancing malignant tissue is gaining popularity and can be used as an adjunct to iMRI as long as the microscope has the appropriate filter [23, 32]. Given that 5-ALA is a more recently developed surgical adjunct, the available literature has not been able to elucidate a clear advantage of using iMRI and 5-ALA together. In a prospective study published by Coburger et al. in 2015, they found that the EOR in 33 glioblastoma patients was significantly maximized when 5-ALA was used in combination with iMRI compared to 144 patients who underwent resection with iMRI alone [83]. However, a systematic review conducted by Coburger et al. in 2019, concluded that neither iMRI or 5-ALA is superior but a combination could potentially assist in a supra-maximal resection [84]. A meta-analysis recently conducted by Golub et al. concluded that iMRI and 5-ALA are both individually advantageous in achieving maximal resection and survival benefit, however, they report that the current evidence is uncertain whether they provide an additive benefit [44].

Operative time

The use of iMRI guidance has been shown to significantly increase operative time in comparison to traditional operating rooms. There is additional time needed for setup, registration, draping and redraping of the patient, transport of the patient into and out of the scanner, and intraoperative scanning times. This additional operative time must be taken into account in patient selection and in surgeon and operating room staff scheduling. While multiple studies have concluded that iMRI guided surgery invariably takes more time, no studies have shown an increase in adverse events (e.g. wound infections) [33, 81, 85–87].

Image quality

Image quality can be negatively affected by artifact from a variety of factors including motion, metallic susceptibility, radiofrequency noise from inadequately shielded electronics in the operating room, the brain-air interface, and blood products [49, 88, 89]. These factors need to be reduced or eradicated when possible to ensure optimal image definition. There are also limitations to the utility of repeated contrast enhanced T1 weighted images due to alterations in the blood brain barrier caused by surgical manipulation. Tissue not originally enhancing may demonstrate contrast enhancement once the blood brain barrier is violated or manipulated during surgery [90]. These factors need to be accounted for when interpreting the images obtained during surgery. In our opinion, intraoperative consultation with a neuroradiologist is invaluable.

Safety considerations

The magnetic field of a MRI is always on and must be taken into consideration in every aspect of the setup and delivery of care. The patient, as well as all personnel, must be screened and cleared for entrance into the iMRI suite. Any individual with metallic implants (e.g. pacemakers, defibrillators, cochlear implants, shunts, pumps, stimulators, shrapnel, ect), may be unsafe to enter. The magnetic field can alter the functionality of a medical device, pull the device within the field, or cause the implant to become hot. Safety of the patient and staff is of paramount importance so extensive training with regularly scheduled recertification courses should be completed by staff members to ensure an understanding and adherence to safety precautions [91–93].

All equipment that will enter into the magnetic field must be MR compatible including carts, monitors, poles, probes, lines, catheters, head fixation devices, and body warmers. Meticulous surgical counts must be preformed every time the patient is transported between the operative position and the scanner to ensure that MRI incompatible instruments are not inadvertently introduced into the magnetic field [86, 93]. Alternatively, with an added investment, MRI compatible instruments can be used to avoid this concern.

Given that the patient will be within the MRI scanner during a portion of their procedure, the anesthesia team must take this distance into consideration and use extended ventilator circuits and access tubing. The magnet can generate heat within

electrical wires or cables and burn the patient and therefore cannot be in direct contact with skin [86].

Economic considerations

The economic efficiency of iMRI has to be taken into consideration for the healthcare system as a whole and for a hospital system contemplating its purchase. A major hindrance to universal implementation is the high cost of purchasing a system, ranging from $3 to $7 million, not including the cost of renovating the operative suite [94]. Further expenses to purchase shields and MRI compatible equipment and instruments are required. There are also continual and considerable costs required for maintenance of the system and to employ highly trained specialized personnel to keep the room running [95]. Multiple studies have also shown that operative time is significantly increased in iMRI guided cases and this results in additional costs [33]. The use of iMRI for tumor resection also increases operative costs due to the additional Current Procedural Terminology (CPT) code added to the standard craniotomy for tumor resection code for iMRI interpretation, a potential financial incentive for the hospital system but additional burden to the healthcare system.

On the other hand, there are also a multitude of potential economic advantages associated with iMRI for a hospital and for the healthcare system. A study by the University of Minnesota found that in comparison to first time adult brain tumor resections performed in conventional operating rooms, iMRI guided surgeries resulted in 54.9% shorter lengths of stay, 12.2% lower hospital charges, and 14.4% lower total hospital costs. They also reported that while conventional surgery resulted in immediate repeat resections for residual tumor in 20% of adults and 30% of pediatric cases, there were no repeat resections required after iMRI guided surgery. The mean time to repeat resection was 11.3 months in adults and 18 months in children for iMRI guided surgery in comparison to 9.3 months in adults and 13.3 months in children with conventional surgery [55]. The ability to offer iMRI technology to patients has enormous marketing potential for a hospital system, especially those that want to be able to tout comprehensive and innovative oncologic treatments. Hospitals can also offset the cost by constructing a suite that can be used for diagnostic purposes when not being used for an operative case. Therefore, while there are many upfront and continuing costs to the implementation of iMRI, there may also be substantial clinical and financial benefits [55].

Future directions

For a technology that was first conceptualized and created over 30 years ago, it has advanced remarkably since then and gained global traction. The Health Policy Institute reported in 2016 that 12% of hospitals have access to iMRI technology in the United States [96]. There is also a growing body of literature investigating the utility of iMRI in the treatment of brain tumors, with over 2600 results in a PubMed search [97]. However, the current available data is not strong enough to recommend that iMRI be included into the standard of care for brain tumors. As Jenkinson and colleagues reported in their review of the literature published in 2018, the evidence to support the use of iMRI in the removal of brain tumors is considered low and very-low quality. This is predominantly because the majority of studies utilize highly selective inclusion criteria favoring younger age and higher functional status, are single-institution studies, and oftentimes conducted at specialized centers [98]. At this time, due to the exorbitant cost associated with investment and maintenance of iMRI technology, and the lack of strong evidence to suggest its superiority over less costly intraoperative imaging and guidance modalities, universal implementation is not feasible. In order to determine whether iMRI will be considered a luxury modality versus a necessity for brain tumor removal in the future, will need to be determined by randomized controlled multi-institutional studies. Ideally, through rigorous research that focuses on neurological and functional outcomes as well as survival, we will be able to elucidate which, if any, pathologies are best approached with iMRI guided resections. As we have watched the technology advance and expand over time, the utility of iMRI is being appreciated for pathologies other than brain tumors and by clinicians other than neurosurgeons. As iMRI technology evolves, the focus should be on optimizing image quality, integration of potentially valuable surgical or intraoperative imaging adjuncts, efficiency of operative setup and workflow, flexibility to accommodate the scope of potential operative positions, effective utilization, and cost-effectiveness.

Conclusion

Intraoperative MRI is a valuable tool in a neurosurgeon's armamentarium in the treatment of brain tumors. The technology is advancing at a rapid pace and providing surgeons with a plethora of innovative techniques targeted toward greater accuracy and improved efficiency. Surgeons must consider the potential benefits of iMRI guidance in each surgical case and weigh them against the additional time and costs associated with its use. Ultimately, iMRI has been shown to be of great benefit in a variety of pathologies and patient populations in helping surgeons to achieve their surgical goals of maximal resection while minimizing morbidity.

References

1. Black PM et al (1997) Development and implementation of intraoperative magnetic resonance imaging and its neurosurgical applications. Neurosurgery 41(4):831–842 discussion 842-5
2. Mislow JM, Golby AJ, Black PM (2010) Origins of intraoperative MRI. Magn Reson Imaging Clin N Am 18(1):1–10
3. Schulder M, Catrambone J, Carmel PW (2005) Intraoperative magnetic resonance imaging at 0.12 T: is it enough? Neurosurg Clin N Am 16(1):143–154
4. Fahlbusch R, Samii A (2016) Intraoperative MRI. Neurosurg Focus 40(3):E3
5. Alexander E 3rd et al (1997) The present and future role of intraoperative MRI in neurosurgical procedures. Stereotact Funct Neurosurg 68(1–4 Pt 1):10–17
6. Bisdas S et al (2015) Intraoperative MR imaging in neurosurgery. Clin Neuroradiol 25(Suppl 2):237–244
7. Dietrich J et al (1999) Brain tumor resections in an open 0.5-T MRT. 2 years' experiences from the neuroradiological viewpoint. Radiologe 39(11):988–994
8. Hlavac M, Wirtz CR, Halatsch ME (2017) Intraoperative magnetic resonance imaging. HNO 65(1):25–29
9. Nimsky C, Carl B (2017) Historical, current, and future intraoperative imaging modalities. Neurosurg Clin N Am 28(4):453–464
10. Hushek SG et al (2008) MR systems for MRI-guided interventions. J Magn Reson Imaging 27(2):253–266
11. Gerlach R et al (2008) Feasibility of Polestar N20, an ultra-low-field intraoperative magnetic resonance imaging system in resection control of pituitary macroadenomas: lessons learned from the first 40 cases. Neurosurgery 63(2):272–284 discussion 284-5
12. Kubben PL et al (2014) Intraoperative magnetic resonance imaging versus standard neuronavigation for the neurosurgical treatment of glioblastoma: a randomized controlled trial. Surg Neurol Int 5:70
13. Mutchnick I, Moriarty TM (2014) Intraoperative MRI in pediatric neurosurgery-an update. Transl Pediatr 3(3):236–246
14. Fahlbusch R (2011) Development of intraoperative MRI: a personal journey. Acta Neurochir Suppl 109:9–16
15. Chicoine MR et al (2011) Implementation and preliminary clinical experience with the use of ceiling mounted mobile high field intraoperative magnetic resonance imaging between two operating rooms. Acta Neurochir Suppl 109:97–102
16. Choudhri AF et al (2015) Intraoperative MRI in pediatric brain tumors. Pediatr Radiol 45(Suppl 3):S397–S405
17. Bohinski RJ et al (2001) Glioma resection in a shared-resource magnetic resonance operating room after optimal image-guided frameless stereotactic resection. Neurosurgery 48(4):731–742 discussion 742-4
18. Jolesz FA (2011) Intraoperative imaging in neurosurgery: where will the future take us? Acta Neurochir Suppl 109:21–25
19. Lippmann H, Kruggel F (2005) Quasi-real-time neurosurgery support by MRI processing via grid computing. Neurosurg Clin N Am 16(1):65–75
20. Elias WJ, Fu KM, Frysinger RC (2007) Cortical and subcortical brain shift during stereotactic procedures. J Neurosurg 107(5):983–988
21. Nimsky C et al (2000) Quantification of, visualization of, and compensation for brain shift using intraoperative magnetic resonance imaging. Neurosurgery 47(5):1070–1079 discussion 1079-80
22. Nabavi A et al (2001) Serial intraoperative magnetic resonance imaging of brain shift. Neurosurgery 48(4):787–797 discussion 797-8
23. Shahar T et al (2014) Preoperative imaging to predict intraoperative changes in tumor-to-corticospinal tract distance: an analysis of 45 cases using high-field intraoperative magnetic resonance imaging. Neurosurgery 75(1):23–30
24. Gering DT et al (2001) An integrated visualization system for surgical planning and guidance using image fusion and an open MR. J Magn Reson Imaging 13(6):967–975
25. Nauta HJ (1994) Error assessment during "image guided" and "imaging interactive" stereotactic surgery. Comput Med Imaging Graph 18(4):279–287
26. Dho YS et al (2019) Positional effect of preoperative neuronavigational magnetic resonance image on accuracy of posterior fossa lesion localization. J Neurosurg:1–10
27. Lacroix M et al (2001) A multivariate analysis of 416 patients with glioblastoma multiforme: prognosis, extent of resection, and survival. J Neurosurg 95(2):190–198
28. Sanai N et al (2011) An extent of resection threshold for newly diagnosed glioblastomas. J Neurosurg 115(1):3–8
29. Oppenlander ME et al (2014) An extent of resection threshold for recurrent glioblastoma and its risk for neurological morbidity. J Neurosurg 120(4):846–853
30. Scherer M et al (2019) Surgery for diffuse WHO grade II Gliomas: volumetric analysis of a Multicenter retrospective cohort from the German study Group for Intraoperative Magnetic Resonance Imaging. Neurosurgery
31. Roelz R et al (2016) Residual tumor volume as best outcome predictor in low grade Glioma - a nine-years near-randomized survey of surgery vs. Biopsy Sci Rep 6:32286
32. Krivosheya D, Prabhu SS (2017) Combining functional studies with intraoperative MRI in Glioma surgery. Neurosurg Clin N Am 28(4):487–497
33. Rao G (2017) Intraoperative MRI and maximizing extent of resection. Neurosurg Clin N Am 28(4):477–485
34. Aghi MK et al (2015) The role of surgery in the management of patients with diffuse low grade glioma: a systematic review and evidence-based clinical practice guideline. J Neuro-Oncol 125(3):503–530
35. Hervey-Jumper SL, Berger MS (2016) Maximizing safe resection of low- and high-grade glioma. J Neuro-Oncol 130(2):269–282
36. Hatiboglu MA et al (2009) Impact of intraoperative high-field magnetic resonance imaging guidance on glioma surgery: a prospective volumetric analysis. Neurosurgery 64(6):1073–1081 discussion 1081
37. Coburger J et al (2015) Linear array ultrasound in low-grade glioma surgery: histology-based assessment of accuracy in comparison to conventional intraoperative ultrasound and intraoperative MRI. Acta Neurochir 157(2):195–206
38. Giordano M et al (2017) Intraoperative magnetic resonance imaging in pediatric neurosurgery: safety and utility. J Neurosurg Pediatr 19(1):77–84
39. Napolitano M et al (2014) Glioblastoma surgery with and without intraoperative MRI at 3.0T. Neurochirurgie 60(4):143–150
40. Knauth M et al (1999) Intraoperative MR imaging increases the extent of tumor resection in patients with high-grade gliomas. AJNR Am J Neuroradiol 20(9):1642–1646
41. Senft C et al (2011) Intraoperative MRI guidance and extent of resection in glioma surgery: a randomised, controlled trial. Lancet Oncol 12(11):997–1003
42. Senft C et al (2010) Low field intraoperative MRI-guided surgery of gliomas: a single center experience. Clin Neurol Neurosurg 112(3):237–243
43. Zhang Z et al (2019) High-field intraoperative magnetic resonance imaging increases extent of resection and progression-free survival for nonfunctioning pituitary adenomas. World Neurosurg 127:e925–e931
44. Golub D et al (2020) Intraoperative MRI versus 5-ALA in high-grade glioma resection: a network meta-analysis. J Neurosurg:1–15

45. Masuda Y et al (2018) Evaluation of the extent of resection and detection of ischemic lesions with intraoperative MRI in glioma surgery: is intraoperative MRI superior to early postoperative MRI? J Neurosurg:1–8
46. Sakurada K et al (2010) Detection of acute subdural hemorrhage using intraoperative MR imaging during glioma surgery: a case report. No Shinkei Geka 38(12):1115–1120
47. Cui Z et al (2019) Early detection of cerebral ischemic events on intraoperative magnetic resonance imaging during surgical procedures for deep brain stimulation. Acta Neurochir 161(8):1545–1558
48. Keles GE (2004) Intracranial neuronavigation with intraoperative magnetic resonance imaging. Curr Opin Neurol 17(4):497–500
49. Lewin JS, Metzger A, Selman WR (2000) Intraoperative magnetic resonance image guidance in neurosurgery. J Magn Reson Imaging 12(4):512–524
50. Yahanda AT et al (2020) A multi-institutional analysis of factors influencing surgical outcomes for patients with newly diagnosed grade I Gliomas. World Neurosurg 135:e754–e764
51. Li YM et al (2016) The influence of maximum safe resection of glioblastoma on survival in 1229 patients: can we do better than gross-total resection? J Neurosurg 124(4):977–988
52. Coburger J et al (2016) Low-grade Glioma surgery in intraoperative magnetic resonance imaging: results of a Multicenter retrospective assessment of the German study Group for Intraoperative Magnetic Resonance Imaging. Neurosurgery 78(6):775–786
53. Rahman M et al (2017) The effects of new or worsened postoperative neurological deficits on survival of patients with glioblastoma. J Neurosurg 127(1):123–131
54. McGirt MJ et al (2009) Association of surgically acquired motor and language deficits on overall survival after resection of glioblastoma multiforme. Neurosurgery 65(3):463–469 discussion 469-70
55. Hall WA et al (2003) Costs and benefits of intraoperative MR-guided brain tumor resection. Acta Neurochir Suppl 85:137–142
56. Pichierri A, Bradley M, Iyer V (2019) Intraoperative magnetic resonance imaging-guided Glioma resections in awake or asleep settings and feasibility in the context of a public health system. World Neurosurg X 3:100022
57. Pollack IF, Agnihotri S, Broniscer A (2019) Childhood brain tumors: current management, biological insights, and future directions. J Neurosurg Pediatr 23(3):261–273
58. Day EL, Scott RM (2019) The utility of intraoperative MRI during pediatric brain tumor surgery: a single-surgeon case series. J Neurosurg Pediatr:1–7
59. Karsy M et al (2019) Evaluation of pediatric glioma outcomes using intraoperative MRI: a multicenter cohort study. J Neuro-Oncol 143(2):271–280
60. Giordano M et al (2016) Neurosurgical tools to extend tumor resection in pediatric hemispheric low-grade gliomas: iMRI. Childs Nerv Syst 32(10):1915–1922
61. Shah MN et al (2012) Intraoperative magnetic resonance imaging to reduce the rate of early reoperation for lesion resection in pediatric neurosurgery. J Neurosurg Pediatr 9(3):259–264
62. Lara-Almunia M, Hernandez-Vicente J (2019) Frame-based stereotactic biopsy: description and Association of Anatomical, radiologic, and surgical variables with diagnostic yield in a series of 407 cases. J Neurol Surg A Cent Eur Neurosurg 80(3):149–161
63. Chen CC et al (2009) Stereotactic brain biopsy: single center retrospective analysis of complications. Clin Neurol Neurosurg 111(10):835–839
64. Silva EU et al (2009) Stereotactic biopsy for intracranial lesions: clinical-pathological compatibility in 60 patients. Arq Neuropsiquiatr 67(4):1062–1065
65. Ersahin M et al (2011) The safety and diagnostic value of frame-based and CT-guided stereotactic brain biopsy technique. Turk Neurosurg 21(4):582–590
66. Bernays RL et al (2002) Histological yield, complications, and technological considerations in 114 consecutive frameless stereotactic biopsy procedures aided by open intraoperative magnetic resonance imaging. J Neurosurg 97(2):354–362
67. Kucharczyk J et al (2001) Cost-efficacy of MR-guided neurointerventions. Neuroimaging Clin N Am 11(4):767–772 xii
68. Chakraborty S et al (2017) Intraoperative MRI for resection of intracranial meningiomas. J Exp Ther Oncol 12(2):157–162
69. Zaidi HA et al (2016) The utility of high-resolution intraoperative MRI in endoscopic transsphenoidal surgery for pituitary macroadenomas: early experience in the advanced multimodality image guided operating suite. Neurosurg Focus 40(3):E18
70. Coburger J et al (2014) Determining the utility of intraoperative magnetic resonance imaging for transsphenoidal surgery: a retrospective study. J Neurosurg 120(2):346–356
71. Hlavac M et al (2019) Intraoperative MRI in transsphenoidal resection of invasive pituitary macroadenomas. Neurosurg Rev 42(3):737–743
72. Sylvester PT et al (2015) Combined high-field intraoperative magnetic resonance imaging and endoscopy increase extent of resection and progression-free survival for pituitary adenomas. Pituitary 18(1):72–85
73. Thomas JG et al (2016) Laser interstitial thermal therapy for newly diagnosed and recurrent glioblastoma. Neurosurg Focus 41(4):E12
74. Swartz LK et al (2019) Outcomes in patients treated with laser interstitial thermal therapy for primary brain Cancer and brain metastases. Oncologist
75. Ali FS et al (2019) Cerebral radiation necrosis: incidence, pathogenesis, diagnostic challenges, and future opportunities. Curr Oncol Rep 21(8):66
76. Mohyeldin A, Elder JB (2017) Stereotactic biopsy platforms with intraoperative imaging guidance. Neurosurg Clin N Am 28(4):465–475
77. Bartek J Jr et al (2019) Biopsy and ablation of H3K27 Glioma using skull-mounted Smartframe device: technical case report. World Neurosurg 127:436–441
78. Pruitt R et al (2017) Complication avoidance in laser interstitial thermal therapy: lessons learned. J Neurosurg 126(4):1238–1245
79. Salem U et al (2019) Neurosurgical applications of MRI guided laser interstitial thermal therapy (LITT). Cancer Imaging 19(1):65
80. Hatiboglu MA et al (2010) Utilization of intraoperative motor mapping in glioma surgery with high-field intraoperative magnetic resonance imaging. Stereotact Funct Neurosurg 88(6):345–352
81. Maldaun MV et al (2014) Awake craniotomy for gliomas in a high-field intraoperative magnetic resonance imaging suite: analysis of 42 cases. J Neurosurg 121(4):810–817
82. Leuthardt EC et al (2011) Use of movable high-field-strength intraoperative magnetic resonance imaging with awake craniotomies for resection of gliomas: preliminary experience. Neurosurgery 69(1):194–205 discussion 205-6
83. Coburger J et al (2015) Surgery for Glioblastoma: impact of the combined use of 5-Aminolevulinic acid and intraoperative MRI on extent of resection and survival. PLoS One 10(6):e0131872
84. Coburger J, Wirtz CR (2019) Fluorescence guided surgery by 5-ALA and intraoperative MRI in high grade glioma: a systematic review. J Neuro-Oncol 141(3):533–546
85. Barone DG, Lawrie TA, Hart MG (2014) Image guided surgery for the resection of brain tumours. Cochrane Database Syst Rev 1:CD009685
86. Berkow LC (2016) Anesthetic management and human factors in the intraoperative MRI environment. Curr Opin Anaesthesiol 29(5):563–567

87. Lu CY et al (2018) Clinical application of 3.0 T intraoperative magnetic resonance combined with multimodal neuronavigation in resection of cerebral eloquent area glioma. Medicine (Baltimore) 97(34):e11702
88. Masuda Y et al (2018) Evaluation of the extent of resection and detection of ischemic lesions with intraoperative MRI in glioma surgery: is intraoperative MRI superior to early postoperative MRI? J Neurosurg 131(1):209–216
89. Makary M et al (2011) Clinical and economic outcomes of low-field intraoperative MRI-guided tumor resection neurosurgery. J Magn Reson Imaging 34(5):1022–1030
90. Kubben PL et al (2012) Correlation between contrast enhancement on intraoperative magnetic resonance imaging and histopathology in glioblastoma. Surg Neurol Int 3:158
91. Cherkashin MA et al (2016) Surgical safety cheklist at the management of the hybrid operating room. Angiol Sosud Khir 22(2):54–59
92. Iturri-Clavero F et al (2016) "Low-field" intraoperative MRI: a new scenario, a new adaptation. Clin Radiol 71(11):1193–1198
93. Henrichs B, Walsh RP (2014) Intraoperative MRI for neurosurgical and general surgical interventions. Curr Opin Anaesthesiol 27(4):448–452
94. Board, A. WSJ: Intraoperative imaging gaining traction. February 19, 2015 18, 2020]; Available from: https://www.advisory.com/daily-briefing/2015/02/19/intraoperative%20imaging
95. RL, B (2010) Intraoperative Imaging: Current Trends, Technology, and Future Directions, in Transsphenoidal Surgery, G.L. ER Laws, (Ed). p. 56–69
96. Database, A.A.S. (2016) Percentage of Hospitals with Access to Intraoperative Magnetic Resonance Imaging. 2016 September 18. 2020]; Available from: https://www.neimanhpi.org/data_series/percentage-of-hospitals-with-access-to-intraoperative-magnetic-resonance-imaging/#/map/2016
97. PubMed. 2020 9/22/2020]; Available from: https://pubmed.ncbi.nlm.nih.gov/?term=intraoperative+mri+and+brain+tumors
98. Jenkinson MD et al (2018) Intraoperative imaging technology to maximise extent of resection for glioma. Cochrane Database Syst Rev 1:CD012788

Publisher's Note Springer Nature remains neutral with regard to jurisdictional claims in published maps and institutional affiliations.
