# Supplementary material for: Magnetic Fields and Cancer: Epidemiology, Cellular Biology, and Theranostics
Source: Int J Mol Sci. 2022 Jan 25;23(3):1339. doi: 10.3390/ijms23031339 (PMC8835851; doi:10.3390/ijms23031339)
Supplement: Supplementary file 1 [file ijms-23-01339-s001.zip › Supplementary Data Set S1/MF and Cancer.Data/PDF/3806969750/ExposuretoElectromagneticFieldsInducesOxidativ.pdf]

ResearchGate

See discussions, stats, and author profiles for this publication at: https://www.researchgate.net/publication/319087781

Exposure to Electromagnetic Fields Induces Oxidative Stress and Pathophysiological Changes in the Cardiovascular System

Article *in* Journal of Biotechnology · October 2017

DOI: 10.15406/jabb.2017.04.00096

CITATIONS
7

READS
881

2 authors, including:

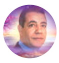

Azab Elsayed Azab
Sabratha Universty

130 PUBLICATIONS **382** CITATIONS

SEE PROFILE

Some of the authors of this publication are also working on these related projects:

Project Effects of electromagnetic fields on human and animals Health View project

Project Anti-dyslipidemic and Antiatherogenic Effects of Some Natural Products View project

All content following this page was uploaded by Azab Elsayed Azab on 30 October 2017.

The user has requested enhancement of the downloaded file.

MedCrave
Step into the World of Research
Journal of Applied Biotechnology & Bioengineering

Exposure to Electromagnetic Fields Induces Oxidative Stress and Pathophysiological Changes in the Cardiovascular System

Abstract

Objectives: This study aimed to highlight on the influence of exposure to electromagnetic fields (EMFs) on the cardiovascular system in humans and experimental animals, from the recent articles regarding the cardiovascular effects of exposure to EMFs. EMFs might produce a variety of adverse in vivo effects such as heart problems, chest pain, and cardiovascular system disorders. Previous studies showed that an association between elevated magnetic field exposure and mortality of employer in electric utility industry jobs from arrhythmia-related causes and acute myocardial infarction influence heart rate variability by changing autonomic balance. EMF exposure can affect structure and function of cardiovascular system and may facilitate myocardial infarction by nuclear changing of cardiomyocytes. Exposure to EMFs induced pain or pressure in the chest area, heart palpitations and/or an irregular heartbeat. The symptoms resemble a heart attack and thus contribute to even more anxiety. Also, exposure to EMFs caused highly significant increases in the activeties of serum creatinine phosphokinase, lactate dehydrogenase and aspartate amino-transferase enzymes, and decreases in plasma calcium level and total anti-oxidant capacity. Rats exposed to EMF showed increases in blood pressure, the absolute and relative whole heart and left ventricular weights. On the other hand, the heart rate was significantly reduced in rats exposed to EMF. The ECG recording of experimental animals exposed to EMF showed a significantly higher R and T voltages, increase in QRS duration, and prolonged P-R and QT-c intervals. A serious histopathological changes in the heart were seen in experimental animals exposed to EMFs, these changes includes increases the number of apoptotic cells, dark brown stain muscle fiber nuclei, marked cell vacuolation, hyperemia muscle fiber degeneration, distortion of some cardiac myocytes, mononuclear cellular infiltration and histological structure of the myocytes spaces were seen. Ultra structural of the myocardial tissue and sarcomere in experimental animals exposed to EMFs showed that lose of area in sarcomeres, irregular structural of myocardial cells, and ruptures of sarcomeres, lose of mitochondria cristae, blebs of mitochondria.

Conclusion: It can be concluded that exposure of human and experimental animals to EMFs have been a negative effect on the heart and blood vessels by causing a histopathological changes and disturbances in the functions of the organs of the cardiovascular system.

Keywords: Cardiovascular system; Heart function; Histopathology; Ultrastrucure; ECG; Oxidative stress

Research Article

Volume 4 Issue 2 - 2017

Azab Elsayed Azab[1]* and Shaban Ali Ebrahim[2]

[1]*Department of Zoology, Faculty of Science, Zawia University, Libya*
[2]*Department of Physics, Faculty of Science, Zawia University, Libya*

***Corresponding author:** Azab Elsayed Azab, Department of Zoology, Faculty of Science, Alejelat, Zawia University, Libya, Email: azabelsaied@yahoo.com

Received: August 12, 2017 | **Published:** October 30, 2017

Abbreviations: EMFs: Electromagnetic Fields; MWR: Microwave Radiation; NIR: Non-Ionizing Radiation; RF-EMW: Radiofrequency Electromagnetic Waves; RF: Radiofrequency; EMW: Electromagnetic Wave; SNS: Sympathetic Nervous System; PNS: Parasympathetic Nervous System; PRA: Plasma Renin Activity; AMI: Acute Myocardial Infarction; SNAP: N-Acetyl-Snitroso- Dl-Penicillinaminamide; ROS: Reactive Oxygen Species; SOD: Superoxide Dismutase; GPX: Glutathione Peroxidase; MRI: Magnetic Resonance Imaging; LVH: Left Ventricular Hypertrophy

Introduction

The use of electrical devices has gradually increased throughout the last century, and scientists have suggested that electromagnetic fields (EMF) generated by such devices may have harmful effects on living creatures [1]. The natural electromagnetic sources are includes sun, some distant stars, atmospheric discharges like thunder, or human body, and unnatural or human made sources are includes printers, vacuum cleaners, cellular phones, hair

Copyright:
©2017 Azab et al.

dryers, refrigerators, washing machines, kettles microwaves, cables that carry electrical currents, television and computers, electrical home gadgets, radio and television base stations, mobile phone base stations and phone equipment, home wiring airport, and transformers [2-6]. The most frequent sources of non-ionizing radiation (NIR) are mobile phones and cell towers which emit microwave radiation (MWR). They emit radiofrequency electromagnetic waves (RF-EMW) [7-9]. WiFi routers emit a beacon signal that is continuous as long as the device is activated. When information is either uploaded or download, the radiation levels increase both at the router and at the computer. The same is true for cordless phones and wireless baby monitors [10]. Any electrical device can be a source of electromagnetic field (EMF). Radiofrequency (RF) energy is a type of nonionizing radiation that is not strong enough to cause ionization of atoms and molecules [11,12]. Low level of EMF can emit by cellular phones, so because of global using of this device; estimation of the unforeseen risks from mobile communication has become a social and ethical problem. As electromagnetic wave (EMW) enters the tissue, its speed and wavelength changes depending upon the electrical properties of the tissue environment. EMW has two types of effects; thermal and non-thermal [1,13,14].

Cardiac muscle cells are especially differentiated to transmit electrical stimuli, with myocardial cells enabling the heart to contract, sinoatrial and atrioventricular nodule cells creating the electrical stimulus, and endocardial cells spread the ventricles inward. These heart cells have four important physiological features: inducibility, contractibility, autonomy, and transmissibility [1,15]. Because of its inducibility, changes in the contraction and rhythm of the heart can result from external stimuli. It is possible for EMF to affect heart function by influencing these physiological features [1]. Development of various heart diseases and daily exposure with EMF hypothesized an association between exposure to magnetic fields and acute cardiovascular disease [12]. At greater EMF strengths or shorter exposures, the ability of the body to develop compensation mechanisms is reduced and the potential for heart-related effects increases [1]. The main effects of electromagnetic fields have increased body temperature. High temperatures and cause damage to internal body organs, vessels, internal bleeding causes. In fact, one of the mechanisms that cause cell damage, which is why hypothermia [9,16,17]. With the impact of EM fields, body atoms and molecules may lose the electrical balance between them, biochemical activities may be affected and electrical structure in the communication of cells and tissues may be disrupted [18,19]. The EMF penetrate the human body and act ions on all organs, altering the cell membrane potential and the distribution of ions and dipoles [20-23]. Chemical and physical processes at the atomic level are the bases of reactions between biomolecules in an EMF, since the field can magnetically affect the chemical bonds between adjacent atoms with consequent production of free radicals [23-26].

Students in schools with Wi-Fi are complaining of headaches, dizziness, nausea, vomiting, insomnia, blurred vision, difficulty concentrating, weakness, and heart palpitations. In the same area, 4 students had sudden cardiac arrests during exercise class within a 2-year period. When other students moved to a different school his symptoms disappeared [10]. Acute cardiovascular changes in humans deliberately exposed to RFEMF are documented in studies relating to magnetic resonance imaging (MRI) and, at higher levels, of RFEMF used to deliberately destroy myocardial tissue in cardiac ablation therapy [27]. Some people who are electrically hypersensitive complain of pain or pressure in the chest area, heart palpitations, and/or an irregular heartbeat, accompanied by feelings of anxiety that develop rapidly. The symptoms resemble a heart attack and thus contribute to even more anxiety [10]. People who lived closest to the antennas of mobile phone base stations experienced the following symptoms more often than those who lived further away: cardiovascular problems, loss of appetite, headaches, dizziness and nausea, fatigue, feeling of discomfort, movement difficulties, depression, sleep disturbance, hearing disruptions, visual disruptions, difficulty concentrating and memory loss, irritability, and skin problems [10,28]. Studies have shown that mammalian and human cells that electromagnetic radiation generated free radicals cause oxidative stress and impaired antioxidant system and genotoxic effect [9,29].

Effects of EMF on heart function

Olson et al. [30] reported an elevation in CK, LDH and GOT after exposure to radiofrequency. Zhang et al. [31] reported that, electromagnetic shield applied to volunteers showed a highly significant effect on serum level of creatinine phosphokinase. Yuan et al. [32] have seen increased levels of LDH in the serum of volunteers occupationally exposed to very high frequency radiations. These observations indicate that exposure to the radiofrequency radiation increases cell death and change the homeostasis of the tissue [33]. Peppes et al. [34] showed the significant positive correlations between angiographic findings, and peak serum myocardial enzyme levels (CPK, AST and LDH), and inflammatory biomarkers were identified. Therefore, due to result in this study the effect of radiation increased cardiac enzymes in the exposed group and can be suggested that cardiovascular disease increased in this group. Also, Aberumand et al. [9] reported that male white mice exposed to the phone calls 10 times, each time for 10 minutes per day for one month showed that creatine phosphokinase, LDH, AST and high-density lipoprotein which reflect cardiac function were increased and a different pathological damage in the heart in the group exposed mobile ray compared to control group. The mobile radiation is harmful effects on enzyme activity and tissue. Exposure to electromagnetic fields is responsible for changes in enzyme and can effected on healthy. In addition, Jeong et al. [35] reveals an association between elevated magnetic field exposure and mortality of employer in electric utility industry jobs from arrhythmia-related causes and acute myocardial infarction (AMI). Also, Savitz et al. [36] recorded that a higher mortality due to arrhythmia or acute myocardial infarction in workers who are exposed to high magnetic field for a longer time. Roshangar et al. [37] concluded that EMF exposure can affect structure and function of cardiovascular system and may facilitate myocardial infarction by nuclear changing of cardiomyocytes.

This may be due to that magnetic fields interact with moving charges in cells and change their velocities [38]. However, Cell membranes have been identified as a primary site of interaction

Citation: Azab AE, Ebrahim SA (2017) Exposure to Electromagnetic Fields Induces Oxidative Stress and Pathophysiological Changes in the Cardiovascular System. J Appl Biotechnol Bioeng 4(2): 00096. DOI: 10.15406/jabb.2017.04.00096

with the low frequency fields [39]. Therefore, the alterations in these charges and molecules consider the first step in the production of biological effects as magnetic field interact with moving charges and change enzymatic activity [40]. In addition, EMF may regulate the rate and the amount of product of biochemical reaction possibly through free radical mechanism including direct influence on enzyme action [24].

Effect of EMF on heart rate and blood pressure

Bellieni et al. [41] found that EMF generated by incubators can alter heart rate variability in newborns especially in prelatures. Study of Andrzejak et al. [42] demonstrated that the mobile phone may influence heart rate variability by changing autonomic balance. Jeong et al. [35] showed 1-day exposure to ELF-EMF suppressed the values of QT intervals in ECG by affecting ventricular repolarization and increased basal HR. During the exposure to radiation from the cordless phone base station, subject experienced a slight increase in heart rate (from 65 to 86 bpm), an irregular heartbeat, and changes in the response of the sympathetic and parasympathetic nervous system (SNS and PNS), respectively. This up regulation of the SNS and down regulation of the PNS is an example of the "fightor- flight" response, indicating physiologic stress. During periods of this type of stress, the body redirects most of the blood and energy from the internal organs to the arms and legs to prepare the organism for fighting or fleeing a stressful situation. Intermittent exposure may not cause a problem but if the exposure is continuous and long-term, the immune system of the body will be compromised and the body will not be able to repair itself, resulting in symptoms that are commonly experienced by those who are electrically hypersensitive. This inability to heal is what then accelerates the symptoms of aging [10]. When students with Wolff-Parkinson- White syndrome are exercising and are exposed to microwave radiation, the combined stress on the heart can lead to supraventricular tachycardia [10,43]. Mohamed et al. [44] reported that rats exposed to cell phone EMF for 2hrs or 3hrs/day for 4 and 8 weeks showed that the heart rate was significantly reduced. Reduction in the heart rate could possibly be explained by an increase in plasma renin activity and parasympathetic tone in these animals [44]. Andrzejak et al. [42] reported that the sympathetic tone was lowered, while parasympathetic tone was suggested to be increased in humans during cell phone call, thus modifying the functioning of circulatory system. High concentration of renin-angiotensin II was reported to lead to significant baroreceptor-mediated bradycardia [45].

Braune et al. [46] reported that mobile phones caused a rise of blood pressure of 5-10 mmHg each time of exposure. Li et al. [47] found that blood pressure in rats was increased through hours after exposure to mobile phone EMF. Mohamed et al. [44] reported that rats exposed to cell phone EMF for 4 and 8 weeks showed that systolic blood pressure was significantly increased in all EMF-exposed rats compared to their controls. The observed increase in blood pressure could be the result of increased plasma renin activity (PRA), which was increased in all the exposed groups. Earlier, it was stated that one of the early and most common consequences of chronic hypertension is left ventricular hypertrophy (LVH), and that the renin-angiotensin system contributes to the development of LVH in hypertension [44,48,49]. Mohamed et al. [44] recorded that rats exposed to cell phone EMF for 4 and 8 weeks for either 1h/day, 2hrs/day or 3hrs/day, exposure being carried out six days/ week, at fixed time of the day showed that Plasma renin activity was increased in all exposed rats, the increase being statistically significant in rats exposed to EMF 3hrs/day for 4 weeks, and in all the groups exposed to EMF for 8 weeks. The hypertrophic myocardial changes could be explained by the associated increased blood pressure in these groups [44]. On the other hand, Soviet researchers reported indications that long term RFEMF may cause hypotension and bradycardia or tachycardia, but these findings have not been replicated elsewhere in humans [27].

Effect of EMF on heart weight

Mohamed et al. [44] reported that rats exposed to cell phone EMF for 4 and 8 weeks showed that the absolute and relative whole heart and left ventricular weights were increased, particularly in the groups exposed to EMF for longer periods indicating hypertrophic changes. The increased blood pressure encountered in this study could explain the changes in absolute and relative cardiac weights.

Effect of EMF on the microscopical structure of the heart

Previous studies showed that some pathological damages in heart include inflammation, hemorrhage, and congestion [9,12,50,51]. Khaki et al. [12] reported that heart ventricular section from a Wistar male rat exposed to EMF (50 Hz) for 40 consequence day shows increasing in dark brown stain muscle fiber nuclei, number of apoptotic cells, hyperemia muscle fiber degeneration, and histological structure of the myocytes spaces were seen, Programmed cell death (Apoptosis) is characterized by shrinkage of the cell and the nucleus. The nuclear chromatin is condensed into sharply delineated masses, and eventually breaks up. Mohamed et al. [44] revealed that the cardiac muscle specimens of rats exposed to cell phone EMF for 4 weeks for either 1h/day, 2hrs/day showed that exhibited congestion of blood vessels without apparent affection of cardiac myocytes. In rats exposed for 3hrs/day for 4 weeks as well as in all the 8 weeks exposure groups, apparent hypertrophy of many of the cardiac myocytes, with deeply acidophilic sarcoplasm and vesicular nuclei, were detected, accompanied by mononuclear cellular infiltration of the cardiac muscle. However, distortion of some cardiac myocytes, together with some areas of complete degeneration and fragmentation of the cells were observed. Loss of the regular arrangement of the cardiac myocytes was observed in the 8 weeks-2 hrs and 3 hrs /day exposure groups, together with marked cell vacuolation, particularly in the 8 weeks- 3hrs/day EMF-exposed group. Aberumand et al. [9] reported that the cardiac muscle of male white mice exposed to the phone calls 10 times, each time for 10 minutes per day for one month showed that congestion of blood vessels and extravasations of RBCs. In addition, disruption of few fibers and hypertrophy of many of the cardiac myositis with condense nuclei was detected, accompanied by infiltration of inflammatory cells. Oxidative stress through H_2O_2 and the NO donor, N-acetyl-Snitroso-DL-penicillinaminamide

(SNAP), induced apoptosis in ventricular cardiomyocytes isolated from a rat heart [12]. Also, Hanafy et al. [50] reported that the effect of RF radiation in generation of free radicals, increased lipid peroxidation and tissue damage with vascular congestion that aggregate of blood vessels and extravasation of RBCs in the myocardium, together with the disruption of few cardiac fibers. It is believed that EMF with high energy waves cause the rise of local temperature where these waves contact together and like ionizing rays lead to the formation of free radicals and create their destructive effects. Free radicals attack lipids, changing in their nature and breaking protein bounds cause cell damage [50,52,53]. Also, Khaki et al. [54] reported that cellular damage caused by oxidative stress of exposure to electromagnetic radiation can induce apoptosis in various tissues of the body.

Effect of EMF on the ultra structure of the heart

Ultra structural study of the myocardial tissue and sarcomere of Wistar male rat exposed to EMF (50 Hz) for 40 consequence day, were showed that lose of mitochondria, blebs of mitochondria, lose of area in sarcomeres and irregular structural of myocardial cells, sarcomeres were ruptures. Authors suggested that necrosis is present by the quick loss of cellular homeostasis, fast swelling as a result of the accumulation of water, electrolytes and starts with plasma membrane rupture and cause to the disruption of cellular organelles [12].

EMF induce oxidative stress

Reactive oxygen species (ROS) are natural consequences of oxidative cell metabolism. Over production of ROS and imbalance of oxidant/antioxidant system are effective factors in the oxidative stress of cellular structures such as lipids, proteins and nucleic acids [55]. Various environmental factors may intervene in this Phenomenon including long-term exposure to ELF-MF [56]. Free radical scavenging enzymes such as catalase, superoxide dismutase (SOD), glutathione peroxidase (GPX) are the first line cellular defense against oxidative injury [57]. Animal studies conducted significant decrease in total antioxidant activity such as SOD , GPX , vitamins E and A concentrations and increase of MDA (a product of polyunsaturated fatty acid peroxidation and used as an indicator of oxidative stress in cells and tissues) and plasma selenium concentration in erythrocytes and plasma after EMF exposure [58].

Acute exposure to RF fields of cell phones could modulate the oxidative stress and free radical generation by production of extracellular superoxide [59], enhancing lipid peroxidation and reducing the activation of SOD and GSH-Px, free radical scavengers [60]. Mohamed et al. [44] concluded that rats exposed to cell phone EMF for 4 and 8 weeks for either 1h/day, 2hrs/day or 3hrs/day, exposure being carried out six days/ week, at fixed time of the day showed that the plasma total anti-oxidant capacity was significantly decreased in all exposed groups, for either 4 or 8 weeks, while the MDA level in the cardiac tissue was only significantly elevated in the 8 weeks- 3hrs/day exposed group compared to the matched control group. So the decrease in plasma total antioxidant capacity might be the result of its exhaustion in defending the free radical believed to be generated with RF-EMF. The significantly increased cardiac MDA content encountered in the present study in the 8 weeks-3hrs/day exposed group, with the longest duration of exposure, points to the limits of the cardiac antioxidants to cope with the excessive MDA generation due to RFEMF exposure [61,62]. It has been suggested that increased total oxidant status levels due to RF radiation emitted from GSM cell phones might play a role in inducing oxidative damage by increasing lipid peroxidation and oxidative stress [63,64].

Effect of EMF on ECG

Mohamed et al. [44] reported that the ECG recording of rats exposed to EMF for 4 weeks revealed a significantly higher R voltage in the group exposed for 3hrs/day, a significant increase in QRS duration in the groups exposed for 2hrs and 3hrs/day and significant prolongation of QT-c interval in the group exposed for 3hrs/day. The ECG recording of rats exposed to EMF for 8 weeks revealed significantly higher R and T voltages, and significantly prolonged P-R and QT-c intervals in the groups exposed for 2hrs or 3hrs/day, the QRS duration being significantly increased in all the 8 weeks- exposed groups. The increased R wave voltage observed could be due to the increased thickness of the left ventricular wall. This left ventricular hypertrophy, evident also from the histopathological examination, could explain the higher T wave voltage observed [44].

Mohamed et al. [44] found that rats exposed to cell phone EMF for 4 and 8 weeks for either 1h/day, 2hrs/day or 3hrs/day, showed that plasma calcium level was significantly decreased in all the exposed groups. Hypocalcemia, might be one of the mechanisms by which EMF interacts with biological tissues that RF radiation from cell phone could alter [60]. The observed significant hypocalcaemia associated with exposure to EMF occurred as a result of alteration of intracellular signaling pathways resulted from RF radiation exposure through changes in Ca2+ permeability across cell membranes [65]. It has been reported that calcium positive ions strengthen cell membranes because they bind together the negatively charged phospholipid molecules, and that electromagnetic radiation could lead to the replacement of calcium with monovalent ions that weakens the membrane and makes it more likely to tear and form pores [66,67]. The prolonged P-R interval could be explained by the increased tone of the parasympathetic system, or possibly as a result of the encountered hypocalcaemia which cause diminished conduction velocity in the heart [44,68]. The significantly prolonged QT-**C** interval in the groups with prolonged exposure to EMF could be attributed to the associated increased renin activity that was reported to remodel the cardiac ion channels, resulting in prolongation of ventricular repolarization and thus prolongation of QT-C interval [69].

Conclusion

It can be concluded that exposure of human and experimental animals to EMFs have been a negative effect on the heart and blood vessels by causing a histopathological changes and disturbances in the functions of the organs of the cardiovascular system. This may be due to that magnetic fields interact with moving charges in cells, change their velocities and altering the cell membrane potential and the distribution of ions and dipoles. The alterations in these charges and molecules consider the first step in the production of

Citation: Azab AE, Ebrahim SA (2017) Exposure to Electromagnetic Fields Induces Oxidative Stress and Pathophysiological Changes in the Cardiovascular System. J Appl Biotechnol Bioeng 4(2): 00096. DOI: 10.15406/jabb.2017.04.00096

biological effects as magnetic field interact with moving charges and change enzymatic activity. EMFs may regulate the rate and the amount of product of biochemical reaction possibly through free radical mechanism including direct influence on enzyme action.

Conflict of Interest

None.

References

1. Elmas O (2016) Effects of electromagnetic field exposure on the heart: a systematic review. Toxicol Ind Health 32(1): 76-82.
2. Van Deventer TE, Saunders R, Repacholi MH (2005) WHO health risk assessment process for static fields. Prog Biophys Mol Biol 87(2-3): 355-363.
3. Ongel K, Gumral N, Ozguner F (2009) The potential effects of electromagnetic field: A Review. Cell Memb Free Rad Res 1(3): 85-89.
4. Gholampour F, Owji SM, Javadifar TS (2011) Chronic exposure to extremely low frequency electromagnetic field Induces mild renal damages in rats. Inter J Zool Res 7(6): 393-400.
5. Khaki AA, Hemmati AA, Nobahari R (2015) A study of the effects of electromagnetic field on islets of Langerhans and insulin release in rats. Cres J Med Biol Sci 2(1): 1-5.
6. Ebrahim S, Azab AE, Albasha MO, Albishti N (2016) The biological effects of electromagnetic fields on human and experimental animals. Inter Res J Natur Appl Sci 3(10): 106-121.
7. Boris Đ, Sokolovi D, Krsti D, Petkovi D, Jovanovi J, et al. (2010) Biochemical and Histopathological Effects of Mobile Phone Exposure on Rat Hepatocytes and Brain. ActaMedica Median 49(1): 37-42.
8. Hamada AJ, Singh A, Agarwal A (2011) Cell Phones and their Impact on Male Fertility: Fact or Fiction. Open ReprodSci J 5: 125-137.
9. Aberumand M, Mansouri E, Pourmotahari F, Mirlohi M, Abdoli Z (2016) Biochemical and histological effects of mobile phone radiation on enzymes and tissues of mice. Res J Pharm Biol Chem Sci 7(5): 1962-1971.
10. Havas M (2013) Radiation from wireless technology affects the blood, the heart, and the autonomic nervous system. Rev Environ Health 28(2-3): 75-84.
11. Erogul O, Oztas E, Yildirim I, Kir T, Aydur E, et al. (2006) Effects of electromagnetic radiation from cellular phone on human sperm motility: an in vitro study. Arc Med Res 37(7): 840-843.
12. Khaki AA, Khaki A (2012) Amelioration of myocardial apoptosis by using Ocimum basilicum in rats after exposure to electromagnetic field: light and transmission microscopic study. Inter J Biosci 2(10): 1-10
13. Seker SS, Cerezci O (1991) Biological effects of electromagnetic fields, safety standards and prevention intentions. Bogazici University Publications, Istanbul, Turkey, p. 8-12.
14. Santini R, Santini P, Danze JM (2002) Study of the health of people living in the vicinity of mobile phone base stations: 1. Influence of distance and sex. Pathol Biol 50: 369-373.
15. Sanalan Y (1999) There is no nuclear radiation. Symposium on the Effects of Electromagnetic Pollution on the Way to Bilisim Community. Ankara, Turkey, p. 1-4.
16. Koylu H (2001) Physiology is circulating respiratory evacuation. Tugra Printing House, Isparta, Turkey, p. 17-47.
17. Jacobson JI (1997) Influence of electromagnetism on genomic and other biological structures. J Indian Med Assoc 95(7): 429-433.
18. Spencer PJ, Gollapudi BB, Waechter JM (2007) Induction of micronuclei by phenol in the mouse bone marrow: I. Association with chemically induced hypothermia. Toxicol Sci 97(1): 120-127.
19. Zusman I, Yaffe P, Pinus H, Ornay A (1990) Effects of pulsing electromagnetic filede on the prenatal and postnatal development in mice and rats in vivo and in vitro studies. Teratology 42(2): 157-170.
20. Sert C (2016) Interaction of electromagnetic field-biological system and three important diseases: cancer, diabet and cardiac diseases. JSM Anat Physiol 1(1): 1001.
21. Duda D, Grzesik J, Pawlicki K (1991) Changes in liver and kidney concentration of copper, manganese, cobalt and iron in rats exposed to static and low-frequency (50 Hz) magnetic fields. J Trace Elem Electrolytes Health Dis 5(3): 181-186.
22. Berg H (1993) Electrostimulation of cell metabolism by low frequency electric and electromagnetic fields. Bioelectrochem Bioenerg 31(1): 1-25.
23. Kula B, Drozdz M (1996) A study of magnetic field effects on fibroblasts cultures, Part 2: The evaluation of effects of static and extremely low frequency magnetic fields on free radical processes in fibroblasts cultures. Bioelectrochem Bioenerg 39(1): 27-30.
24. Sallam SM, Awad AM (2008) Effect of static magnetic field on the electrical properties and enzymes function of rat liver. Romanian J Biophys 18(4): 337-347.
25. Till U, Timmel CR, Brocklerhurst B, Hore PJ (1998) The influence of very small magnetic fields on radical recombination reactions in the limit of slow recombination. Chem Phys Lett 298(1-3): 7-14.
26. Rollwitz J, Lupke M, Simko M (2004) Fifty-hertz magnetic fields induce free radical formation in mouse bone marrow-derived promonocytes and macrophages. Biochim Biophys Acta 1674(3): 231-238.
27. Simko M, and Mattsson MO (2004) Extremely low frequency electromagnetic fields as effectors of cellular responses in vitro, possible immune cell activation. J Cell Biochem 93(1): 83-92.
28. Black DR, Heynick LN (2003) Radiofrequency effects on blood cells, cardiac, endocrine, and immunological functions. Bioelectromag 6: S187-S195.
29. Mashevich M, Folkman D, Kesar A, Barbul A, Korenstein R, et al. (2003) Exposure of Human Peripheral Blood Lymphocytes to Electromagnetic Fields Associated with Cellular Phones Leads to Chromosomal Instability. Bioelectromag 24(2): 82-90.
30. Olsen RG, David TD (1984) Hypothermia and electromagnetic rewarming in the Rehesus monkey. Aviat Space Environ Med 55(12): 1-7.
31. Zhang J, Clement D, Taunton J (2000) The efficiency of farabloc, an electromagnetic shield, in attenuating delayed- onset muscle sorness. Clin J Sport. Med 10(1): 15-21.
32. Yuan ZQ, Li F, Wang DG, Wang Y, Zhang P (2004) Effect of low intensity and very high frequency electromagnetic radiation on occupationally exposed personnel. Zhonghua Lao Dong Wei Sheng Zhi Ye Bing Za Zhi 22(4): 267-269.

33. Shivashankara AR (2015) Effect of Cell Phone Use on Salivary Total Protein, Enzymes and Oxidative Stress Markers in Young Adults: A Pilot Study. J Clin Diagnostic Res 9(2): 19-22.

34. Peppes V, Rammos G, Manios E, Koroboki E, Rokas S, et al. (2008) Correlation between myocardial enzyme serum levels and markers of inflammation with severity of coronary artery disease and Gensini score: A hospital-based, prospective study in Greek patients. ClinInterv Aging 3(4): 699-710.

35. Jeong JH, Kim JS, Lee BC, Min YS, Kim DS, et al. (2005) Influence of exposure to electromagnetic field on the cardiovascular system". Autonom Autacoid Pharm 25(1): 23-17.

36. Savitz DA, Liao D, Sastre A, Kleckner RC, Kavet R (1999) Magnetic field exposure and cardiovascular disease mortality among electric utility workers. Am J Epidemiol 149(2): 135-142.

37. Roshangar B, Soleimani Rad J, Ansaree R, Roshangar L (2012) Effect of low frequency Electromagnetic Field on cardiovascular system: An ultrastructural and immunohistochemical study. Annals Biol Res 3(1): 81-87.

38. Goodman R, Blank M (2002) Insights into electromagnetic interaction mechanisms. J Cellular Physiol 192(1): 16-22.

39. Adey WR (1988) Cell membranes: the electromagnetic environment and cancer promotion. Neuro chem Res 13(7): 671-677.

40. Vizcaino V (2003) Biological effects of low frequency electromagnetic fields. Radiobiologia 3: 44-46.

41. Bellieni CV, Acampa M, Maffei M, Maffei S, Perrone S, et al. (2008) Electromagnetic fields produced by incubators influence heart rate variability in newborns. Arch Dis Child Fetal Neonatal Ed 93(4): 298-301.

42. Andrzejak R, Poreba R, Poreba M, Derkacz A, Skalik R, et al. (2008) The Influence of the Call with a Mobile Phone on Heart Rate Variability Parameters in Healthy Volunteers. Ind Health 46(4): 409-417.

43. Sinatra S (2011) The negative health impact of wireless technologies and Wi-Fi. Talk at Total Health Show, MetroToronto Convention Centre, Canada, p. 8-11.

44. Mohamed FA, Ahmed AA, El Kafoury BMA, Lasheen NN (2011) Study of the cardiovascular effects of exposure to electromagnetic field. Life Sci J 8(1): 260-274.

45. Yeung MY, Smyth JP (2000) Hypertension and bradycardia associated with high dose of dexamethasone in premature infant with border line hyponatremia. Australian. Journal of hospital pharmacy 30(5): 205-207.

46. Braune S, Wrocklage C, Raczek J, Gailus T, Lücking CH (1998) Resting blood pressure increase during exposure to a radio-frequency electromagnetic field. Lancet 351(9119): 1857-1858.

47. Li BF, Guo GZ, Ren DQ, Jing Li, Zhang RB (2008) Electromagnetic pulses induce fluctuations in blood pressure in rats. Int J Radiat Biol 83(6): 421-429.

48. Devereux RB, Wachtell K, Gerdts E, Boman K, Nieminen MS, et al. (2004) Prognostic significance of left ventricular mass change during treatment of hypertension. JAMA 292(19): 2350-2356.

49. Crowley SD, Gurley SB, Herrera MJ, Ruiz P, Griffiths R, et al. (2006) Angiotensin II causes hypertension and cardiac hypertrophy through its receptors in the kidney. Proc Natl Acad Sci USA 103(47): 17985-17989.

50. Hanafy LK, Karam SH, Saleh A (2010) The adverse effects of mobile phone radiation on some visceral organs. Res J Med Med Sci 5(1): 95-99.

51. Sepehrimanesh M, Azarpira N, Saeb M, Nazifi S, Kazemipour N, et al. (2014) Pathological changes associated with experimental 900-MHz electromagnetic wave exposure in rats. Comp Clin Path 23(5): 1629-1631.

52. Zare S, Alivandi S, Ebadi A (2007) Histological studies of the low frequency electromagnetic fields effect on liver, testes and kidney in guinea pig. World Applied Sciences Journal 2(5): 509-511.

53. Jadidi M, Safari M, Baghian A (2013) Effects of extremely low frequency electromagnetic fields on cell proliferation. Koomesh 15(1): 1-10.

54. Khaki A, Fathiazad F, Nouri M, Khaki AA (2011) Effect of Ocimum basilicum on apoptosis in testis of rats after exposure to electromagnetic field. African J Pharm Pharmacol 5(12): 1534-1537.

55. Meral I, Mert H, Nihat M, Deger Y, Yorukc I, et al. (2007) Effects of 900-MHz electromagnetic field emitted from cellular phone on brain oxidative stress and some vitamin levels of guinea pigs. Brain Res 1169: 120-124.

56. Frahm J, Lantow M, Lupke M, Weiss DG, Simko M (2006) Alteration in cellular functions in mouse macrophages after exposure to 50 Hz magnetic fields. J Cell Biochem 99(1): 168-177.

57. Sharma M, Kishore K, Gupta SK, Joshi SS, Arya DH (2001) Cardio protective potential of Ocimum sanctum in isoproterenol induced myocardial infarction in rats. Molecular and Cellular Biochemistry 225(1-): 75-83.

58. Sharifian A, Gharavi M, Pasalar P, Aminian O (2009) Effect of extremely low frequency magnetic field on antioxidant activity in plasma and red blood cells in spot welders Int Arch Occup Environ Health 82(2): 259-266.

59. Friedman J, Kraus S, Hauptman Y, Schiff Y, Seger R (2007) Mechanism of short-term ERK activation by electromagnetic fields at mobile phone frequencies. Biochem J 405(3): 559-568.

60. Moustafa YM, Moustafa RM, Belacy A, Abou El Ela SH, et al. (2001) Effects of acute exposure to the radiofrequency fields of cellular phones on plasma lipid peroxide and antioxidase activities in human erythrocytes. J Pharm Biomed Anal 26(4): 605-608.

61. Ozguner F, Altinbas A, Ozaydin M, Dogan A, Vural H, et al. (2005) Mobile phone induced myocardial oxidative stress: protection by a novel antioxidant agent caffeic acid phenethyl ester. Toxicol Ind Health 21(9): 223-230.

62. Devrim E, Erguder IB, Kiliçoglu B, Yaykasl E, Cetin R, et al. (2008) Effects of electromagnetic radiation use on oxidant/antioxidant status and dna turn-over enzyme activities in erythrocytes and heart, kidney, liver, and ovary tissues from rats: possible protective role of vitamin C. Toxicol Mech Methods 18(9): 679-683.

63. Dasdag S, Bilgin HM, Akdag MZ, Celik H, Aksen F (2008) Effect of long term mobile phone exposure on oxidative-antioxidative processes and nitric oxide in rats. Biotechnol Equi Diagn Press 22(4): 992-997.

64. Ongel K, Gumral N, Ozguner F (2009) The potential effects of electromagnetic field: A review. Cell Memb Free Rad Res 1(3): 85-89.

65. Maskey D, Kim M, Aryal B, Pradhan J, Choi IY, et al. (2010) Effect of 835 MHz radiofrequency radiation exposure on calcium binding proteins in the hippocampus of the mouse brain. Brain Res 1313: 232-241.

66. Ha BY (2001) Stabilization and destabilization of cell membranes by multivalent ions. Phys Rev E 64: 51902- 51907.

67. Melikov KC, Frolov VA, Shcherbakov A, Samsonov AV, Chizmadzhev YA, et al. (2001) Voltage-induced nonconductive pre-pores and metastable single pores in unmodified planar lipid bilayer. Biophys J 80(4): 1829-1836.

68. Cascio WE, Yan GX, Kléber AG (1990) Passive electrical properties, mechanical activity, and extracellular potassium in arterially perfused and ischemic rabbit ventricular muscle. Effects of calcium entry blockade or hypocalcemia. Circ Res 66(6): 1461-1473.

69. Raizada V, Skipper B, Luo W, Garza L, Hines CW, et al. (2005) Renin-angiotensin polymorphisms and QTc interval prolongation in end-stage renal disease. Kidney Int 68(3): 1186-1189.

Citation: Azab AE, Ebrahim SA (2017) Exposure to Electromagnetic Fields Induces Oxidative Stress and Pathophysiological Changes in the Cardiovascular System. J Appl Biotechnol Bioeng 4(2): 00096. DOI: 10.15406/jabb.2017.04.00096

View publication stats
