# Supplementary material for: Magnetic Fields and Cancer: Epidemiology, Cellular Biology, and Theranostics
Source: Int J Mol Sci. 2022 Jan 25;23(3):1339. doi: 10.3390/ijms23031339 (PMC8835851; doi:10.3390/ijms23031339)
Supplement: Supplementary file 1 [file ijms-23-01339-s001.zip › Supplementary Data Set S1/MF and Cancer.Data/PDF/3833687916/rspb20180590.pdf]

PROCEEDINGS B

rspb.royalsocietypublishing.org

Review

Cite this article: Juutilainen J, Herrala M, Luukkonen J, Naarala J, Hore PJ. 2018 Magnetocarcinogenesis: is there a mechanism for carcinogenic effects of weak magnetic fields? *Proc. R. Soc. B* **285**: 20180590. http://dx.doi.org/10.1098/rspb.2018.0590

Received: 25 March 2018
Accepted: 27 April 2018

Subject Category:
Development and physiology

Subject Areas:
biophysics, cellular biology, health and disease and epidemiology

Keywords:
ELF magnetic fields, cancer, radical pair mechanism, cryptochrome, genomic instability

Author for correspondence:
Jukka Juutilainen
e-mail: jukka.juutilainen@uef.fi

Electronic supplementary material is available online at https://dx.doi.org/10.6084/m9.figshare.c.4092539.

THE ROYAL SOCIETY PUBLISHING

Magnetocarcinogenesis: is there a mechanism for carcinogenic effects of weak magnetic fields?

Jukka Juutilainen[1], Mikko Herrala[1], Jukka Luukkonen[1], Jonne Naarala[1] and P. J. Hore[2]

[1]Department of Environmental and Biological Sciences, University of Eastern Finland, Kuopio, Finland
[2]Department of Chemistry, University of Oxford, Oxford, UK

JJ, 0000-0002-9578-7381; JN, 0000-0002-5240-968X; PJH, 0000-0002-8863-570X

Extremely low-frequency (ELF) magnetic fields have been classified as possibly carcinogenic, mainly based on rather consistent epidemiological findings suggesting a link between childhood leukaemia and 50–60 Hz magnetic fields from power lines. However, causality is not the only possible explanation for the epidemiological associations, as animal and *in vitro* experiments have provided only limited support for carcinogenic effects of ELF magnetic fields. Importantly, there is no generally accepted biophysical mechanism that could explain such effects. In this review, we discuss the possibility that carcinogenic effects are based on the radical pair mechanism (RPM), which seems to be involved in magnetoreception in birds and certain other animals, allowing navigation in the geomagnetic field. We review the current understanding of the RPM in magnetoreception, and discuss cryptochromes as the putative magnetosensitive molecules and their possible links to cancer-relevant biological processes. We then propose a hypothesis for explaining the link between ELF fields and childhood leukaemia, discuss the strengths and weaknesses of the current evidence, and make proposals for further research.

1. Introduction

Epidemiological studies have rather consistently reported an association between childhood leukaemia and 50–60 Hz magnetic fields from power lines, prompting the International Agency for Research on Cancer to classify extremely low-frequency (ELF; $\leq$300 Hz) magnetic fields as possibly carcinogenic to humans [1]. The epidemiological findings indicate that the risk of leukaemia increases for time-average magnetic flux densities above about 0.3–0.4 μT [2]. However, drawing conclusions concerning the causality of the epidemiological associations is difficult, as animal and *in vitro* experiments have provided only limited support for the epidemiological findings, and there is no generally accepted biophysical mechanism that could explain carcinogenic effects of low-level magnetic fields [1,3]. The radical pair mechanism (RPM) is considered one of the more plausible mechanisms for explaining biological effects of weak magnetic fields [3]. The RPM affects chemical reactions involving radical pairs and, for a radical pair formed in a singlet state, increases the concentration of free radicals in low fields (low-field effect, LFE) and decreases it in high fields (high-field effect, HFE) [4,5]. The LFE occurs below about 1 mT, and could therefore potentially explain adverse health effects of weak environmental ELF magnetic fields. However, although the RPM is theoretically well developed, and has been experimentally demonstrated in cell-free chemical systems [6], its practical biological relevance has not been established. Much of the discussion on the role of radicals in ELF magnetic field effects focuses on radical-induced DNA damage. However, the expected magnitude of the LFE is small [5], cells have defence mechanisms to regulate the levels

© 2018 The Author(s) Published by the Royal Society. All rights reserved.

of free radicals and magnetic field effects on whole-cell radical concentrations may not be observable at the magnetic flux densities relevant to the LFE [7].

The biological relevance of radicals is not limited to macromolecular damage associated with increased levels of reactive oxygen species (ROS); they are also a part of normal cell physiology, including intracellular signal transduction [8]. Magnetic field effects on radical levels, in spite of their small magnitude, could therefore potentially have multiple biological consequences if they occur in cellular organelles or molecules that are key components in biological regulatory networks. From this point of view, it is of interest that several animal species are able to detect weak magnetic fields at microtesla levels for the purposes of orientation and navigation in the geomagnetic field. Although the detection mechanisms are still to be fully determined, magnetically sensitive reactions of radical pairs in cryptochromes (CRYs) seem to be involved, at least in birds [9]. The geomagnetic field is essentially static (i.e. it has almost no time-dependence). An ELF magnetic field, in contrast, is an oscillating field generated by alternating current, such as the 50 Hz current used in transmission of electric power. One of the major challenges is to explain how a 0.3–0.4 μT ELF magnetic field could lead to significant biological effects in the presence of the much stronger (25–65 μT) geomagnetic field.

In this paper, we discuss the possibility that carcinogenic effects result from biological detection of weak ELF magnetic fields by magnetically sensitive radical reactions in important regulatory molecules such as CRY. We review the current understanding of the RPM in magnetoreception and its links to cancer-relevant biological processes, as well as experimental evidence for effects of ELF magnetic fields that may have a bearing on carcinogenesis. We then propose a hypothesis for explaining the link between ELF fields and childhood leukaemia, discuss the strengths and weaknesses of the current evidence, and make proposals for further research.

2. The radical pair mechanism, cryptochromes and carcinogenesis

(a) Radical pair magnetoreception

Although it is clear that birds use the geomagnetic field as a source of navigational information, the underlying sensory mechanisms are obscure [9]. It appears that birds have two separate sensors, one for geographical location (a magnetic map), the other for direction-finding (a magnetic compass) [10]. The map sensor probably involves ferrimagnetic iron oxide particles [10,11] while the (light-dependent) compass sense seems to rely on photo-induced radical pairs (figure 1), probably in CRYs [9,12]. Much of the evidence for a CRY-based compass is circumstantial (see [9] for a recent review and a tutorial on the RPM). We summarize it briefly here.

Spectroscopic measurements on purified CRYs suggest that they could be suitable as magnetoreceptors. Absorption of blue light by the non-covalently bound flavin adenine dinucleotide (FAD) cofactor triggers a series of electron transfers within the protein from a triad or tetrad of tryptophan (TrpH) residues to the FAD [13]. The [$FAD^{\bullet -}$ $TrpH^{\bullet +}$] radical pair so formed is sensitive to weak magnetic fields and leads to long-lived forms of the protein that could act as signalling states [14,15]. Although effects of magnetic fields weaker than approximately 1 mT have yet to be reported for purified CRYs, the principle of a geomagnetic (50 μT) chemical sensor has been demonstrated (for a carotenoid–porphyrin–fullerene model system) [16].

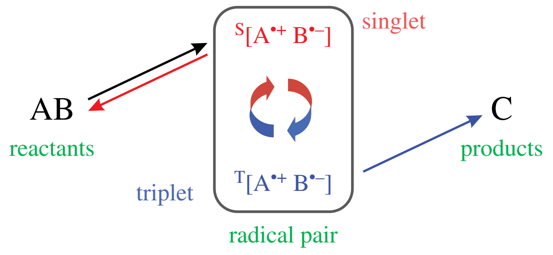

Figure 1. A simple radical pair reaction scheme. Reactant molecules, AB, are converted into products, C, via a short lived radical pair, [$A^{\bullet +}$ $B^{\bullet -}$], formed by the transfer of an electron from A to B (black arrow). The electron spins, one in each radical, can have either a singlet (red) or a triplet (blue) configuration. Singlet and triplet states differ in the relative orientation of the two spins: anti-parallel (singlet) or parallel (triplet). The red and blue arrows represent spin-selective chemical reactions: reversion of the singlet to AB by back electron transfer and forward reaction of the triplet to form C. The curved arrows represent the oscillatory, quantum mechanical interconversion of the singlet and triplet states, driven by internal magnetic interactions within the radicals. Whether a given radical pair reacts to form AB or C depends on the probability that it is singlet or triplet at the moment of reaction. If application of a magnetic field increases the triplet probability, the result will be an increased yield of the product C. Note that the radical pair need not be formed by electron transfer and that other reaction schemes are possible. (Online version in colour.)

Migratory songbirds process magnetic compass information in a small area of the forebrain that receives its input from ganglion cells in the retinas of both eyes via the thalamofugal visual pathway [10]. Although CRYs have been found in a number of retinal cell types [17], the exact location of the magnetoreceptors is not known. Experiments on genetically modified insects suggest that CRY mediates magnetic behavioural responses but cannot distinguish between its potential roles as a magnetoreceptor and as a transducer of magnetic information furnished by a different sensor [18].

Theoretical considerations support the principle of a radical pair compass and indicate that the $FAD^{\bullet -}$ radical in CRY has magnetic properties that make it particularly well suited as a component of a magnetic sensor [19–21]. These calculations also suggest that $TrpH^{\bullet +}$ may not be the optimum partner and that a much simpler radical could offer substantially higher detection sensitivity [19,20].

From both experimental and theoretical studies, it is clear that radiofrequency magnetic fields (1–100 MHz) are capable of modifying the responses of radical pairs to static magnetic fields and can be used as a general test for the operation of the RPM [22]. On this basis, arguably the most convincing evidence for radical pair magnetoreception is that migratory birds can be prevented from using their magnetic compass by exposure to weak magnetic fields with frequencies in the range 0.1 − 10 MHz [19,23,24]. However, the reports of radiofrequency field effects on captive birds have not been consistent and the current theoretical model is unable to account for the magnitude of the effects [9,25]. It should also be noted that the effects of radiofrequency fields are distinct from those of ELF magnetic fields, whose frequencies are far too low to be in resonance with the coherent spin dynamics of a radical pair.

Little is known about the transduction of CRY-mediated magnetic field effects [9]. In *Drosophila* neurons, light-activated CRY induces membrane depolarization and increased action potential firing through closure of voltage-gated K^+ channels [26]. This process is enhanced in the presence of a 100 mT magnetic field [27]. By analogy with the blue-light signalling properties of plant cryptochromes [28], it seems likely that once the FAD cofactor has been photo-reduced, the first step is a change in the conformation of the C-terminal region of the protein that alters the ability of the CRY to bind to other proteins.

(b) Possible connections to cancer-relevant biological processes

Apart from their possible role in magnetoreception in animals, magnetically sensitive radical pair reactions may be linked to the regulation of other biological functions. In this section, we discuss the possible connections between RPM-based magnetosensitivity and carcinogenesis.

As CRYs are key molecules in the circadian clock system [29], it is possible that circadian rhythms could be affected by magnetic fields. Indeed, light-dependent magnetosensitivity of the circadian clock has been reported in *Drosophila* [30,31] and in mammalian experimental systems (electronic supplementary material, table S1). Suggestive evidence of magnetic field effects on circadian rhythms has been found in mice and cows [32–34], and effects on the expression of circadian clock genes were reported in a human fibroblast cell line exposed to a 50 Hz magnetic field [35].

Magnetosensitivity of the circadian clock fits with the hypothesis [36] that disruption of circadian timing is the mechanism that links ELF magnetic fields to cancer. This discussion focuses on the possible impact of magnetic fields on the systemic, central regulation of the circadian clock and refers to evidence of the involvement of the circadian system in carcinogenesis. However, magnetosensitivity of the central clock alone would not explain the *in vitro* findings that suggest ELF magnetic field effects on the expression of circadian genes [35] and various cancer-relevant cellular processes [37,38]. The master circadian oscillator in mammals is located in the suprachiasmatic nuclei in the hypothalamus, but a complete functioning circadian regulation system (the peripheral clock) is also found in peripheral tissues and in cultured cells [39]. Magnetosensitivity of the cellular circadian system could therefore lead to cell-level responses to magnetic fields [40]. Importantly, the molecular clock system seems to be linked to other cellular systems with relevance to cancer.

It has become clear that the circadian clock is closely coupled with regulation of the cell cycle and cellular responses to DNA damage, such as repair, cell-cycle checkpoints and apoptosis [29,39,41]. The practical significance of the link between the circadian system and DNA damage responses is illustrated by findings showing that genetic and environmental disturbances of the circadian regulation system are associated with increased risk of cancer [42,43]. Currently there is also interest in chronotherapy (i.e. the administration of anticancer drugs or radiotherapy at specific times of the day to optimize the therapeutic outcomes and/or to minimize adverse side effects) [29,44].

Recent studies have shown a link between the clock system and regulation of ROS levels and oxidative stress responses [45]. The link between the clock system and ROS appears to be a two-way interaction: not only is antioxidant defence controlled by the circadian clock, but the circadian rhythms seem to be influenced by (and probably based on) redox oscillations which occur both on the circadian time scale and as ultradian rhythms with periods much shorter than 24 h [46,47]. Indeed, the living cell can be seen as a complex oscillator that coordinates (among many other things) responses to DNA damage and oxidative stress (electronic supplementary material, figure S1).

3. Experimental evidence for cancer-relevant biological effects of weak magnetic fields

(a) Effects on DNA damage responses

Most genotoxicity studies have not shown any genetic damage from exposure to magnetic fields alone, except for extremely strong fields [1]. However, several groups have reported findings suggesting that ELF magnetic fields enhance the effects of known DNA damaging agents [1]. Such co-exposures were evaluated in a meta-analysis of 65 studies that had combined ELF magnetic fields with other toxic chemical or physical agents [37]. When the findings were examined as a function of magnetic flux density, a non-linear 'dose–response' was found, showing a minimum in the percentage of positive findings at fields between 1 and 3 mT (electronic supplementary material, figure S2). This pattern arose in an exploratory analysis with no *a priori* hypothesis, but it fits nicely with the RPM, as the crossover between the LFE and the HFE should occur approximately in this flux density range [14].

The suggested ability of magnetic fields to alter biological responses to genotoxic agents implies that they might modify DNA damage responses. Cellular response to DNA damage is a complex process involving detection of the damage, activation of DNA repair pathways, activation of cell-cycle checkpoints to arrest the cell cycle and allow time for repair, and initiation of apoptosis in case of severe damage [29]. There have been a few studies of ELF magnetic field effects on these processes, including suppression of apoptosis [48–50], changes in cell-cycle distribution [50–55], altered expression of genes and proteins involved in cell-cycle regulation and DNA damage responses [52–57], increased rate but decreased fidelity of DNA repair [58], and no effect on DNA repair rate [59] (for details, see electronic supplementary material, table S2).

(b) Effects on reactive oxygen species

Much of the discussion of possible biological effects of ELF fields centres on ROS and the damage that they can cause in cells. It should be noted that ELF magnetic fields, unlike blue light and ultraviolet radiation, have nowhere near enough energy to break chemical bonds, induce electron transfer reactions or otherwise create radical pairs. Any magnetic field effect therefore requires an existing radical pair reaction. Furthermore, most ROS radicals are very unlikely to generate magnetic field effects. Superoxide ($O_2^{\bullet -}$), hydroxyl radical ($OH^\bullet$) and nitric oxide ($NO^\bullet$, a reactive nitrogen species) undergo exceedingly rapid spin relaxation (probably in nanoseconds) precluding significant effects of weak fields [60].

Any magnetic field effects on the concentrations of these radicals could only arise from upstream pairs of organic radicals.

Reactive oxygen species are generated in cells both by external agents such as ionizing or UV radiation and by normal physiological processes. High levels of ROS can cause damage to DNA and other biological molecules, but they also have important roles in cell signalling and homeostasis [8]. It follows that any magnetic field effects on cellular ROS levels would be relevant to an assessment of the possible carcinogenic effects of magnetic fields. Mattsson & Simko [38] have reviewed studies investigating oxidative responses in cell cultures exposed to ELF magnetic fields. The authors concluded that ELF magnetic fields consistently alter ROS levels in many cell types and experimental designs. The evidence was strongest for fields in excess of 1 mT, but effects were also reported at or below 100 μT. The effect size was moderate: the majority of studies reported changes in ROS levels of less than 50%.

The small size of the magnetic field-induced change in ROS levels, given the effective cellular antioxidant defence mechanisms, is not likely to result in a major increase in DNA damage. However, even small changes in ROS levels might be important because of the role of ROS in cell signalling. One of the key ROS in cell signalling is superoxide, and several studies [61–65] have reported effects of ELF magnetic fields on cytosolic and mitochondrial superoxide levels (electronic supplementary material, table S3). Magnetic field effects on superoxide levels seem to require some time to develop [61,62] and exhibit other time-dependent changes [65]. These findings, together with the small size of the effects, indicate magnetic fields effects on ROS signalling, rather than induction of oxidative stress.

(c) Magnetic field-induced genomic instability

There is increasing evidence that induced genomic instability (IGI) plays a role in environmentally induced cancer. IGI is the delayed *de novo* appearance of genetic damage in the progeny of exposed cells (or organisms) many cell generations after exposure [66]. IGI was originally found in cells exposed to ionizing radiation, but several other agents have been reported to induce it [67]. As the development of cancer requires accumulation of multiple genetic changes, IGI is potentially highly relevant [68].

Four studies have addressed induction of genomic instability *in vitro* in the progeny of cells exposed to ELF magnetic fields, and all reported positive findings (electronic supplementary material, table S4). In one of the studies, bleomycin-induced chromosomal instability was enhanced by a 60 Hz magnetic field applied continuously for up to 240 h after the bleomycin treatment [69]. The other studies reported increased frequencies of micronuclei [61,70] and microsatellite mutations (particularly allelic imbalance) [71] multiple cell generations after a 12–24 h magnetic field exposure with or without combined treatment with menadione or ionizing radiation.

In an experiment relevant to current understanding of childhood leukaemia (electronic supplementary material, figure S3), enhanced development of leukaemias/lymphomas and some other malignancies was reported in rats that were exposed pre- and postnatally to 50 Hz magnetic fields at 20 or 1000 μT, and postnatally to a single 0.1 Gy dose of ionizing radiation [72]. Such a finding can be interpreted as induction of latent genomic instability manifested as an increased incidence of malignancy only following exposure to a second postnatal leukaemogen [73].

4. Synthesis and hypothesis

Weak magnetic fields apparently affect circadian rhythms in animals, and there is also evidence of such effects in cultured human cells. Because the circadian clock is linked to regulation of DNA damage responses and ROS-related processes, it is reasonable to think that magnetic fields could affect these cellular functions and consequently the stability of biological systems. This postulate is supported by many independent studies (reviewed above) reporting that ELF magnetic fields may modify DNA damage responses and affect ROS signalling. Furthermore, four studies consistently suggest that magnetic field exposure may lead to or enhance genomic instability. The proposed link between perturbation of the circadian clock system and genomic instability is supported by a recent study on the mechanisms of transgenerational genomic instability induced by ionizing radiation: altered expression of genes involved in rhythmic processes and the circadian clock were found in the offspring of irradiated male mice [74].

As a synthesis of the studies reviewed above, we propose a hypothesis for explaining the link between environmental magnetic fields and childhood leukaemia (figure 2). It is based on the role of CRYs in magnetoreception and findings indicating that the circadian regulation system (including CRYs) is coupled to DNA damage responses and defence against ROS. As CRYs and the whole circadian regulation system are found in peripheral tissues and in cultured cells, magnetic field effects could occur in all cells and not only in cells specialized in magnetoreception. Sensitivity to weak magnetic fields might be an intrinsic property of living cells, which has served as the basis for the evolution of a magnetic sense in certain species (such as the magnetic compass in birds).

5. Discussion

(a) Strength of evidence

The proposed hypothesis is based on several independent lines of research. Some pieces of the puzzle stem from well-established science, but evidence is weaker for other elements.

The evidence for the involvement of CRY in RPM-based magnetoreception (reviewed in §2a) is relatively strong, at least in birds. It should be noted, however, that the studies reviewed cannot distinguish between CRY as the magnetic detector and CRY as a (e.g. downstream) component in magnetic signal transduction. Furthermore, it is not clear how these findings translate into magnetosensitivity in humans. If human cells are sensitive to weak magnetic fields, this could be incidental due to the presence of CRY and/or other magnetically sensitive molecules or structures. In this case, however, CRY-mediated magnetic field effects on humans are probably much smaller than those thought to be involved in avian magnetoreception. The avian magnetoreceptor has presumably evolved to be exquisitely sensitive to the geomagnetic field (approx. 50 μT), but no optimization of magnetosensitivity could have occurred in human cells if it had not been driven by evolutionary pressure. Another possibility is that

rspb.royalsocietypublishing.org Proc. R. Soc. B 285: 20180590

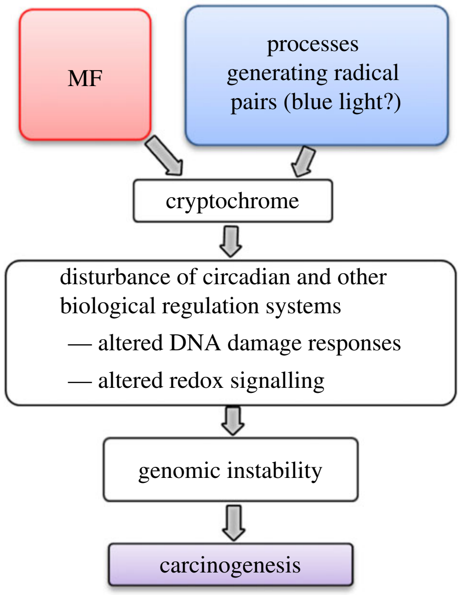

Figure 2. Hypothesis. The primary interaction mechanism is magnetic field (MF) effects on radical reactions in cryptochromes. Because the circadian clock is closely coupled with the regulation of responses to DNA damage and ROS, the primary interaction could lead to dysregulation of these systems, impaired DNA damage responses, genomic instability and finally to cancer. (Online version in colour.)

magnetosensitivity has been a useful property during evolution, and perhaps continues to be so in human cells. (a) It has been proposed that natural fields below 60 Hz resulting from the Schumann resonance could act as secondary zeitgebers [30,75]. A major problem with this proposal is that the Schumann resonance magnetic fields are extremely weak, of the order of 1 pT. (b) It has also been proposed that electromagnetic fields generated by cells may have roles in intercellular or intracellular communication (which implies that there must be mechanisms for reception of electromagnetic fields). The literature on such effects is rather extensive but controversial [76]. (c) Apart from direction-finding, it has been proposed that magnetoreception could also be involved more generally in spatial perception [77,78].

There is also rather strong evidence that circadian rhythms can be influenced by magnetic fields (§2b). This is well documented in *Drosophila*, and to a lesser extent in mammals and cultured human cells.

Links between the circadian clock system and the regulation of DNA damage responses and ROS-related cellular processes have been convincingly shown in multiple studies (§2b). Such a role for the circadian clock is an essential component of the hypothesis proposed in this paper, as it forms a plausible causal link between magnetosensitive biological molecules (CRYs) and the suggested carcinogenic effect of ELF magnetic fields.

With regard to the next step of the proposed link between ELF magnetic fields and cancer, there is evidence from many independent studies that exposure to magnetic fields alters DNA damage responses (§3a) and affects ROS-related cellular processes (§3b). However, there are inconsistencies in the data, and the size of the magnetic field effect is generally small. On the other hand, small effects are not surprising within the framework of the proposed hypothesis, as we do not consider magnetic fields to be a strong toxic insult, but rather a regulating agent that could affect DNA damage responses and ROS signalling because they are linked to a magnetosensitive molecule (e.g. CRY).

Evidence of induction of genomic instability by ELF magnetic fields is limited, being addressed in only four *in vitro* studies (§3c). However, all these studies consistently indicate induction of genomic instability in cells exposed to 50–60 Hz magnetic fields at 100–1000 μT.

A major problem in using the RPM to explain carcinogenic effects of <1 μT ELF magnetic fields is the short lifetime (at most a few microseconds) of spin coherence in biologically plausible radical pairs [21]. As a consequence, a 50–60 Hz field would be 'perceived' by the radical pair as effectively static. It is then difficult to see how an ELF field of intensity <1 μT could significantly affect a radical pair reaction in the presence of the much stronger (25–65 μT) geomagnetic field. To explain any ELF magnetic field effects at magnetic flux densities relevant to the suspected carcinogenic risk of environmental fields, it will be necessary to identify credible mechanisms that would allow a 50–60 Hz field to cause a disproportionately large effect.

Very low experimental thresholds for ELF magnetosensitivity have been reported by Prato *et al*. [79,80], who observed that reducing the ambient magnetic field induced analgesia in mice, and that 10–240 Hz fields as weak as 25 nT (at 120 Hz) or 33 nT (at 30 Hz) reduced the effect. No mechanism for this extraordinary sensitivity to ELF fields was proposed. It should be noted, however, that these experiments were conducted in the absence of the geomagnetic field, which might have increased the animals' sensitivity to an alternating magnetic field. Studies involving both static and ELF magnetic fields have provided some evidence that the latter might disrupt the biological effects of a static field on body orientation of ruminants [81] and on proliferation of cultured human cells [82]. While these studies do not provide a mechanistic explanation for increased sensitivity to weak ELF magnetic fields, they may provide clues for developing theoretical models that predict effects of ELF fields in different static magnetic field conditions.

Experimental studies on cancer-related biological endpoints (reviewed above) have generally tested relatively high flux densities of 100 μT and above, and experimental data on effects below 1 μT are essentially lacking. It is therefore unclear whether these findings could serve as an explanation for the epidemiological findings below 1 μT. On the other hand, human exposure to ELF magnetic fields ≥100 μT does occur particularly in occupational environments, so the experimental results and mechanisms discussed in the present paper may be relevant to human health effects even if they do not explain the childhood leukaemia findings. Micronuclei and superoxide levels in mammalian cells have been found to be affected by fields down to 10 μT [62], but further studies at lower magnetic flux densities will be critical for evaluation of the credibility of human health effects at 0.4 μT. However, this will be challenging because of the background ELF magnetic fields of cell culture incubators, and because it will be increasingly difficult to show statistically significant effects if the effect size decreases with field strength.

(b) Further research

The proposed hypothesis has implications concerning the design and interpretation of further studies on the link between ELF magnetic fields and cancer. A study design

aspect that may be important is the duration and timing of magnetic field exposure. Magnetic field effects that are mediated by disturbances of the circadian clock system might require that magnetic field exposure is long enough (e.g. 24 h) so that it can interfere with the natural rhythm (e.g. by functioning as a false signal resembling continuous light), or so that magnetic field exposure is received at the right time with respect to the natural oscillations. For example, it was found that exposure to a 60 Hz field protected cultured human cells from heat-induced apoptosis only if the duration of the exposure was at least 12 h [49]. In a study on diurnal rhythms of pain threshold in mice [34], different effects were observed depending on whether the animals were exposed to a 60 Hz magnetic field at night or during the day. On the other hand, even a short magnetic field exposure may be sufficient in some exposure designs: application of a 50 Hz field for 1 h induced circadian oscillation of clock genes in human fibroblast cells that were not rhythmic before the exposure [35].

It should also be noted that conventional 'toxicological' dose–responses cannot always be expected if magnetic field effects are mediated by biological regulation pathways. Increasing field strength does not necessarily lead to more pronounced disturbance of biological regulation (once the threshold for disturbance has been exceeded). Therefore, lack of a conventional increasing exposure–response relationship may not always speak against causality.

Importantly, further studies can be designed to test predictions of the hypothesis, including the following:

(1) As predicted by the RPM, the exposure–response relationship should be biphasic, consisting of the LFE below about 1 mT and the HFE at higher magnetic flux densities. There is one reservation, however: if there is a mechanism for larger effects from ELF fields than from static magnetic fields of comparable strength, it is not possible to estimate how such an amplification or resonance mechanism would affect the exposure–response relationship.
(2) CRY should respond to ELF magnetic fields, and magnetic field effects should depend on the presence of functional CRY in the exposed cells or organisms.
(3) Responses to ELF magnetic fields should also be observed in other molecules (downstream of CRY) of the circadian clock system, as well as in the related DNA damage response and antioxidant defence systems.
(4) Exposure to ELF magnetic fields should lead to induction of genomic instability.

Some of these predictions are already supported by limited experimental evidence [35,52,61]. However, these findings need to be confirmed. It would also be useful to evaluate several steps of the proposed causal chain (e.g. CRY–DNA damage responses–genomic instability) in a single study, which would have the power to show causal relationships.

One more prediction follows if human magnetosensitivity is assumed to be based on magnetic field effects on light-induced radical pairs, according to the current hypothesis of avian magnetoreception. In this case, magnetic field effects should depend on the presence of blue light. In a recent study designed to test this hypothesis [63], blue light was not necessary for magnetic field effects on $O_2^{\bullet -}$ levels in human neuroblastoma cells exposed to 50 Hz, 100 µT fields. This does not necessarily mean that the magnetoreceptor is not CRY if non-photochemical processes are involved in inducing the signalling (radical) state of CRY. Mammalian CRYs may have no photoreceptor function [83,84], but their biological activity seems nevertheless to be associated with the radical state of CRY [85].

6. Concluding remarks

Evidence from numerous studies suggests that cancer-relevant biological processes can be influenced by ≥100 µT, 50–60 Hz magnetic fields, and a plausible mechanism for such effects is offered by radical pair reactions in specific target molecules (such as CRY) linked to biological regulatory networks. Independent replication of the key findings suggesting ELF field effects is of crucial importance for progress in this area of research. The experimental findings at fields ≥100 µT do not directly explain the epidemiological association between childhood leukaemia and ≥0.4 µT ELF magnetic fields. Furthermore, while the radical pair mechanism appears to be involved in sensing the static geomagnetic field (approx. 50 µT) by animals, it remains unclear how it could explain human health effects of ELF magnetic fields weaker than 1 µT.

Further theoretical and experimental work will be necessary to identify credible mechanisms that might allow a 50–60 Hz field to cause a disproportionately large effect. However, health implications of the current empirical and theoretical findings should not be ignored; human exposure to ELF fields ≥100 µT does occur, particularly in occupational environments.

Although mechanisms for biological effects of weak ELF fields remain obscure, radical pair chemistry of CRYs appears to be the most plausible working hypothesis that can be used to guide further research. However, explaining the suspected health effects of very weak (approx. 1 µT) ELF magnetic fields is undeniably challenging within the framework of this working hypothesis, and work on alternative mechanisms [86–88] should continue.

Data accessibility. This article has no additional data.

Authors' contributions. All authors assessed and discussed the relevant literature. J.J. and P.J.H. wrote the manuscript; J.L., J.N. and M.H. commented on it. All authors gave final approval for publication.

Competing interests. We declare we have no competing interests.

Funding. This study was supported by the Academy of Finland (grant no. 298378)

References

1. IARC. 2002 *Non-ionizing radiation, part 1: static and extremely low frequency (ELF) electric and magnetic fields*, vol. 80. Lyon, France: International Agency for Research on Cancer.
2. Ahlbom A *et al.* 2000 A pooled analysis of magnetic fields and childhood leukaemia. *Br. J. Cancer* **83**, 692–698. (doi:10.1054/bjoc.2000.1376)
3. WHO. 2007 *Extremely low frequency fields*. Geneva, Switzerland: World Health Organization.
4. Brocklehurst B, McLauchlan KA. 1996 Free radical mechanism for the effects of environmental

electromagnetic fields on biological systems. *Int. J. Radiat. Biol.* **69**, 3–24. (doi:10.1080/095530096146147)

5. Timmel CR, Till U, Brocklehurst B, McLauchlan KA, Hore PJ. 1998 Effects of weak magnetic fields on free radical recombination reactions. *Mol. Phys.* **95**, 71–89. (doi:10.1080/00268979809483134)
6. Eveson RW, Timmel CR, Brocklehurst B, Hore PJ, McLauchlan KA. 2000 The effects of weak magnetic fields on radical recombination reactions in micelles. *Int. J. Radiat. Biol.* **76**, 1509–1522. (doi:10.1080/09553000050176270)
7. Markkanen A, Naarala J, Juutilainen J. 2010 A study on the effects of 50 Hz magnetic fields on UV-induced radical reactions in murine fibroblasts. *J. Radiat. Res.* **51**, 609–613. (doi:JST.JSTAGE/jrr/10038)
8. Finkel T. 2003 Oxidant signals and oxidative stress. *Curr. Opin. Cell. Biol.* **15**, 247–254. (doi:S0955067403000024)
9. Hore PJ, Mouritsen H. 2016 The radical-pair mechanism of magnetoreception. *Annu. Rev. Biophys.* **45**, 299–344. (doi:10.1146/annurev-biophys-032116-094545)
10. Mouritsen H, Heyers D, Güntürkün O. 2016 The neural basis of long-distance navigation in birds. *Annu. Rev. Physiol.* **78**, 133–154. (doi:10.1146/annurev-physiol-021115-105054)
11. Mora CV, Davison M, Wild JM, Walker MM. 2004 Magnetoreception and its trigeminal mediation in the homing pigeon. *Nature* **432**, 508–511. (doi:10.1038/nature03077)
12. Ritz T, Adem S, Schulten K. 2000 A model for photoreceptor-based magnetoreception in birds. *Biophys. J.* **78**, 707–718. (doi:S0006-3495(00)76629-X)
13. Liedvogel M, Maeda K, Henbest K, Schleicher E, Simon T, Timmel CR, Hore PJ, Mouritsen H. 2007 Chemical magnetoreception: bird cryptochrome 1a is excited by blue light and forms long-lived radical-pairs. *PLoS ONE* **2**, e1106. (doi:10.1371/journal.pone.0001106)
14. Maeda K *et al.* 2012 Magnetically sensitive light-induced reactions in cryptochrome are consistent with its proposed role as a magnetoreceptor. *Proc. Natl Acad. Sci. USA* **109**, 4774–4779. (doi:10.1073/pnas.1118959109)
15. Sheppard DM *et al.* 2017 Millitesla magnetic field effects on the photocycle of an animal cryptochrome. *Sci. Rep.* **7**, 42228. (doi:10.1038/srep42228)
16. Maeda K, Henbest KB, Cintolesi F, Kuprov I, Rodgers CT, Liddell PA, Gust D, Timmel CR, Hore PJ. 2008 Chemical compass model of avian magnetoreception. *Nature* **453**, 387–390. (doi:10.1038/nature06834)
17. Bolte P *et al.* 2016 Localisation of the putative magnetoreceptive protein cryptochrome 1b in the retinae of migratory birds and homing pigeons. *PLoS ONE* **11**, e0147819. (doi:10.1371/journal.pone.0147819)
18. Gegear RJ, Casselman A, Waddell S, Reppert SM. 2008 Cryptochrome mediates light-dependent magnetosensitivity in *Drosophila*. *Nature* **454**, 1014–1018. (doi:10.1038/nature07183)
19. Ritz T, Wiltschko R, Hore PJ, Rodgers CT, Stapput K, Thalau P, Timmel CR, Wiltschko W. 2009 Magnetic compass of birds is based on a molecule with optimal directional sensitivity. *Biophys. J.* **96**, 3451–3457. (doi:10.1016/j.bpj.2008.11.072)
20. Lee AA, Lau JC, Hogben HJ, Biskup T, Kattnig DR, Hore PJ. 2014 Alternative radical pairs for cryptochrome-based magnetoreception. *J. R. Soc. Interface* **11**, 20131063. (doi:10.1098/rsif.2013.1063)
21. Kattnig DR, Solov'yov IA, Hore PJ. 2016 Electron spin relaxation in cryptochrome-based magnetoreception. *Phys. Chem. Chem. Phys.* **18**, 12 443–12 456. (doi:10.1039/c5cp06731f)
22. Henbest KB, Kukura P, Rodgers CT, Hore PJ, Timmel CR. 2004 Radio frequency magnetic field effects on a radical recombination reaction: a diagnostic test for the radical pair mechanism. *J. Am. Chem. Soc.* **126**, 8102–8103. (doi:10.1021/ja048220q)
23. Engels S *et al.* 2014 Anthropogenic electromagnetic noise disrupts magnetic compass orientation in a migratory bird. *Nature* **509**, 353–356. (doi:10.1038/nature13290)
24. Schwarze S, Schneider NL, Reichl T, Dreyer D, Lefeldt N, Engels S, Baker N, Hore PJ, Mouritsen H. 2016 Weak broadband electromagnetic fields are more disruptive to magnetic compass orientation in a night-migratory songbird (*Erithacus rubecula*) than strong narrow-band fields. *Front. Behav. Neurosci.* **10**, 55. (doi:10.3389/fnbeh.2016.00055)
25. Hiscock HG, Mouritsen H, Manolopoulos DE, Hore PJ. 2017 Disruption of magnetic compass orientation in migratory birds by radiofrequency electromagnetic fields. *Biophys. J.* **113**, 1475–1484. (doi:10.1016/j.bpj.2017.07.031)
26. Fogle KJ, Baik LS, Houl JH, Tran TT, Roberts L, Dahm NA, Cao Y, Zhou M, Holmes TC. 2015 Cryptochrome-mediated phototransduction by modulation of the potassium ion channel beta-subunit redox sensor. *Proc. Natl Acad. Sci. USA* **112**, 2245–2250. (doi:10.1073/pnas.1416586112)
27. Giachello CNG, Scrutton NS, Jones AR, Baines RA. 2016 Magnetic fields modulate blue-light-dependent regulation of neuronal firing by cryptochrome. *J. Neurosci.* **36**, 10 742–10 749. (doi:10.1523/jneurosci.2140-16.2016)
28. Chaves I *et al.* 2011 The cryptochromes: blue light photoreceptors in plants and animals. *Annu. Rev. Plant Biol.* **62**, 335–364. (doi:10.1146/annurev-arplant-042110-103759)
29. Sancar A, Lindsey-Boltz LA, Kang TH, Reardon JT, Lee JH, Ozturk N. 2010 Circadian clock control of the cellular response to DNA damage. *FEBS Lett.* **584**, 2618–2625. (doi:10.1016/j.febslet.2010.03.017)
30. Yoshii T, Ahmad M, Helfrich-Forster C. 2009 Cryptochrome mediates light-dependent magnetosensitivity of *Drosophila's* circadian clock. *PLoS Biol.* **7**, e1000086. (doi:10.1371/journal.pbio.1000086)
31. Fedele G *et al.* 2014 Genetic analysis of circadian responses to low frequency electromagnetic fields in *Drosophila melanogaster*. *PLoS Genet.* **10**, e1004804. (doi:10.1371/journal.pgen.1004804)
32. Kumlin T, Heikkinen P, Laitinen JT, Juutilainen J. 2005 Exposure to a 50-Hz magnetic field induces a circadian rhythm in 6-hydroxymelatonin sulfate excretion in mice. *J. Radiat. Res.* **46**, 313–318. (doi:10.1269/jrr.46.313)
33. Rodriguez M, Petitclerc D, Burchard JF, Nguyen DH, Block E. 2004 Blood melatonin and prolactin concentrations in dairy cows exposed to 60 Hz electric and magnetic fields during 8 h photoperiods. *Bioelectromagnetics* **25**, 508–515. (doi:10.1002/bem.20024)
34. Choi YM, Jeong JH, Kim JS, Lee BC, Je HD, Sohn UD. 2003 Extremely low frequency magnetic field exposure modulates the diurnal rhythm of the pain threshold in mice. *Bioelectromagnetics* **24**, 206–210. (doi:10.1002/bem.10094)
35. Manzella N *et al.* 2015 Circadian gene expression and extremely low-frequency magnetic fields: an *in vitro* study. *Bioelectromagnetics* **36**, 294–301. (doi:10.1002/bem.21915)
36. Vanderstraeten J, Verschaeve L, Burda H, Bouland C, de Brouwer C. 2012 Health effects of extremely low-frequency magnetic fields: reconsidering the melatonin hypothesis in the light of current data on magnetoreception. *J. Appl. Toxicol.* **32**, 952–958. (doi:10.1002/jat.2761)
37. Juutilainen J, Kumlin T, Naarala J. 2006 Do extremely low frequency magnetic fields enhance the effects of environmental carcinogens? A meta-analysis of experimental studies. *Int. J. Radiat. Biol.* **82**, 1–12. (doi:10.1080/09553000600577839)
38. Mattsson MO, Simko M. 2014 Grouping of experimental conditions as an approach to evaluate effects of extremely low-frequency magnetic fields on oxidative response in *in vitro* studies. *Front. Public Health* **2**, 132. (doi:10.3389/fpubh.2014.00132)
39. Reddy AB, Wong GK, O'Neill J, Maywood ES, Hastings MH. 2005 Circadian clocks: neural and peripheral pacemakers that impact upon the cell division cycle. *Mutat. Res.* **574**, 76–91. (doi:10.1016/j.mrfmmm.2005.01.024)
40. Lagroye I, Percherancier Y, Juutilainen J, De Gannes FP, Veyret B. 2011 ELF magnetic fields: animal studies, mechanisms of action. *Prog. Biophys. Mol. Biol.* **107**, 369–373. (doi:10.1016/j.pbiomolbio.2011.09.003)
41. Kondratov RV, Antoch MP. 2007 Circadian proteins in the regulation of cell cycle and genotoxic stress responses. *Trends Cell Biol.* **17**, 311–317. (doi:10.1016/j.tcb.2007.07.001)
42. Kelleher FC, Rao A, Maguire A. 2014 Circadian molecular clocks and cancer. *Cancer Lett.* **342**, 9–18. (doi:10.1016/j.canlet.2013.09.040)
43. Hoffman AE *et al.* 2010 The core circadian gene Cryptochrome 2 influences breast cancer risk, possibly by mediating hormone signaling. *Cancer Prev. Res.* **3**, 539–548. (doi:10.1158/1940-6207.CAPR-09-0127)
44. Chan S *et al.* 2017 Does the time of radiotherapy affect treatment outcomes? A review of the literature. *Clin. Oncol.* **29**, 231–238. (doi:10.1016/j.clon.2016.12.005)

45. Wilking M, Ndiaye M, Mukhtar H, Ahmad N. 2013 Circadian rhythm connections to oxidative stress: implications for human health. *Antioxid. Redox Signal.* **19**, 192–208. (doi:10.1089/ars.2012.4889)
46. Lloyd D, Murray DB. 2007 Redox rhythmicity: clocks at the core of temporal coherence. *Bioessays* **29**, 465–473. (doi:10.1002/bies.20575)
47. Mellor J. 2016 The molecular basis of metabolic cycles and their relationship to circadian rhythms. *Nat. Struct. Mol. Biol.* **23**, 1035–1044. (doi:10.1038/nsmb.3311)
48. Kumlin T, Heikkinen P, Kosma VM, Alhonen L, Jänne J, Juutilainen J. 2002 p53-independent apoptosis in UV-irradiated mouse skin: possible inhibition by 50 Hz magnetic fields. *Radiat. Environ. Biophys.* **41**, 155–158. (doi:10.1007/s00411-002-0153-8)
49. Robison JG, Pendleton AR, Monson KO, Murray BK, O'Neill KL. 2002 Decreased DNA repair rates and protection from heat induced apoptosis mediated by electromagnetic field exposure. *Bioelectromagnetics* **23**, 106–112. (doi:10.1002/bem.103)
50. Markkanen A, Juutilainen J, Naarala J. 2008 Pre-exposure to 50 Hz magnetic fields modifies menadione-induced DNA damage response in murine L929 cells. *Int. J. Radiat. Biol.* **84**, 742–751. (doi:10.1080/09553000802360836)
51. Markkanen A, Juutilainen J, Lang S, Pelkonen J, Rytömaa T, Naarala J. 2001 Effects of 50 Hz magnetic field on cell cycle kinetics and the colony forming ability of budding yeast exposed to ultraviolet radiation. *Bioelectromagnetics* **22**, 345–350. (doi:10.1002/bem.60)
52. Luukkonen J, Höytö A, Sokka M, Liimatainen A, Syväoja J, Juutilainen J, Naarala J. 2017 Modification of p21 level and cell cycle distribution by 50 Hz magnetic fields in human SH-SY5Y neuroblastoma cells. *Int. J. Radiat. Biol.* **93**, 240–248. (doi:10.1080/09553002.2017.1235298)
53. Marcantonio P, Del Re B, Franceschini A, Capri M, Lukas S, Bersani F, Giorgi G. 2010 Synergic effect of retinoic acid and extremely low frequency magnetic field exposure on human neuroblastoma cell line BE(2)C. *Bioelectromagnetics* **31**, 425–433. (doi:10.1002/bem.20581)
54. Huang CY, Chang CW, Chen CR, Chuang CY, Chiang CS, Shu WY, Fan TC, Hsu IC. 2014 Extremely low-frequency electromagnetic fields cause G1 phase arrest through the activation of the ATM-Chk2-p21 pathway. *PLoS ONE* **9**, e104732. (doi:10.1371/journal.pone.0104732)
55. Liu Y, Liu WB, Liu KJ, Ao L, Cao J, Zhong JL, Liu JY. 2016 Overexpression of miR-26b-5p regulates the cell cycle by targeting CCND2 in GC-2 cells under exposure to extremely low frequency electromagnetic fields. *Cell Cycle* **15**, 357–367. (doi:10.1080/15384101.2015.1120924)
56. Lange S, Richard D, Viergutz T, Kriehuber R, Weiss DG, Simko M. 2002 Alterations in the cell cycle and in the protein level of cyclin D1, p21CIP1, and p16INK4a after exposure to 50 Hz MF in human cells. *Radiat. Environ. Biophys.* **41**, 131–137. (doi:10.1007/s00411-002-0145-8)
57. Lange S, Viergutz T, Simko M. 2004 Modifications in cell cycle kinetics and in expression of G1 phase-regulating proteins in human amniotic cells after exposure to electromagnetic fields and ionizing radiation. *Cell Prolif.* **37**, 337–349. (doi:10.1111/j.1365-2184.2004.00317.x)
58. Luukkonen J, Liimatainen A, Höytö A, Juutilainen J, Naarala J. 2011 Pre-exposure to 50 Hz magnetic fields modifies menadione-induced genotoxic effects in human SH-SY5Y neuroblastoma cells. *PLoS ONE* **6**, e18021. (doi:10.1371/journal.pone.0018021)
59. Woodbine L, Haines J, Coster M, Barazzuol L, Ainsbury E, Sienkiewicz Z, Jeggo P. 2015 The rate of X-ray-induced DNA double-strand break repair in the embryonic mouse brain is unaffected by exposure to 50 Hz magnetic fields. *Int. J. Radiat. Biol.* **91**, 495–499. (doi:10.3109/09553002.2015.1021963)
60. Hogben HJ, Efimova O, Wagner-Rundell N, Timmel CR, Hore PJ. 2009 Possible involvement of superoxide and dioxygen with cryptochrome in avian magnetoreception: origin of Zeeman resonances observed by *in vivo* EPR spectroscopy. *Chem. Phys. Lett.* **480**, 118–122. (doi:10.1016/j.cplett.2009.08.051)
61. Luukkonen J, Liimatainen A, Juutilainen J, Naarala J. 2014 Induction of genomic instability, oxidative processes, and mitochondrial activity by 50 Hz magnetic fields in human SH-SY5Y neuroblastoma cells. *Mutat. Res.* **760**, 33–41. (doi:10.1016/j.mrfmmm.2013.12.002)
62. Kesari KK, Juutilainen J, Luukkonen J, Naarala J. 2016 Induction of micronuclei and superoxide production in neuroblastoma and glioma cell lines exposed to weak 50 Hz magnetic fields. *J. R. Soc. Interface* **13**, 20150995. (doi:10.1098/rsif.2015.0995)
63. Höytö A, Herrala M, Luukkonen J, Juutilainen J, Naarala J. 2017 Cellular detection of 50 Hz magnetic fields and weak blue light: effects on superoxide levels and genotoxicity. *Int. J. Radiat. Biol.* **93**, 646–652. (doi:10.1080/09553002.2017.1294275)
64. Mannerling AC, Simko M, Mild KH, Mattsson MO. 2010 Effects of 50-Hz magnetic field exposure on superoxide radical anion formation and HSP70 induction in human K562 cells. *Radiat. Environ. Biophys.* **49**, 731–741. (doi:10.1007/s00411-010-0306-0)
65. Feng B, Ye C, Qiu L, Chen L, Fu Y, Sun W. 2016 Mitochondrial ROS release and subsequent Akt activation potentially mediated the anti-apoptotic effect of a 50-Hz magnetic field on FL cells. *Cell. Physiol. Biochem.* **38**, 2489–2499. (doi:10.1159/000445599)
66. Baverstock K. 2000 Radiation-induced genomic instability: a paradigm-breaking phenomenon and its relevance to environmentally induced cancer. *Mutat. Res.* **454**, 89–109. (doi:10.1016/S0027-5107(00)00100-7)
67. Korkalainen M, Huumonen K, Naarala J, Viluksela M, Juutilainen J. 2012 Dioxin induces genomic instability in mouse embryonic fibroblasts. *PLoS ONE* **7**, e37895. (doi:10.1371/journal.pone.0037895)
68. Streffer C. 2010 Strong association between cancer and genomic instability. *Radiat. Environ. Biophys.* **49**, 125–131. (doi:10.1007/s00411-009-0258-4)
69. Cho YH, Jeon HK, Chung HW. 2007 Effects of extremely low-frequency electromagnetic fields on delayed chromosomal instability induced by bleomycin in normal human fibroblast cells. *J. Toxicol. Environ. Health A* **70**, 1252–1258. (doi:10.1080/15287390701429281)
70. Kesari KK, Luukkonen J, Juutilainen J, Naarala J. 2015 Genomic instability induced by 50 Hz magnetic fields is a dynamically evolving process not blocked by antioxidant treatment. *Mutat. Res. Genet. Toxicol. Environ. Mutagen.* **794**, 46–51. (doi:10.1016/j.mrgentox.2015.10.004)
71. Mairs RJ *et al.* 2007 Microsatellite analysis for determination of the mutagenicity of extremely low-frequency electromagnetic fields and ionising radiation *in vitro*. *Mutat. Res.* **626**, 34–41. (doi:10.1016/j.mrgentox.2006.08.005)
72. Soffritti M *et al.* 2016 Life-span exposure to sinusoidal-50 Hz magnetic field and acute low-dose gamma radiation induce carcinogenic effects in Sprague-Dawley rats. *Int. J. Radiat. Biol.* **92**, 202–214. (doi:10.3109/09553002.2016.1144942)
73. Lord BI, Woolford LB. 2002 Induction of stem cell cycling in mice increases their sensitivity to a chemical leukaemogen: implications for inherited genomic instability and the bystander effect. *Mutat. Res.* **501**, 13–17. (doi:10.1016/S0027-5107(02)00011-8)
74. Gomes AM, Barber RC, Dubrova YE. 2015 Paternal irradiation perturbs the expression of circadian genes in offspring. *Mutat. Res.* **775**, 33–37. (doi:10.1016/j.mrfmmm.2015.03.007)
75. Close J. 2012 Are stress responses to geomagnetic storms mediated by the cryptochrome compass system? *Proc. R. Soc. B* **279**, 2081–2090. (doi:10.1098/rspb.2012.0324)
76. Cifra M, Fields JZ, Farhadi A. 2011 Electromagnetic cellular interactions. *Prog. Biophys. Mol. Biol.* **105**, 223–246. (doi:10.1016/j.pbiomolbio.2010.07.003)
77. Phillips JB, Muheim R, Jorge PE. 2010 A behavioral perspective on the biophysics of the light-dependent magnetic compass: a link between directional and spatial perception? *J. Exp. Biol.* **213**, 3247–3255. (doi:10.1242/jeb.020792)
78. Burger T, Lucova M, Moritz RE, Oelschlager HH, Druga R, Burda H, Wiltschko W, Wiltschko R, Nemec P. 2010 Changing and shielded magnetic fields suppress c-Fos expression in the navigation circuit: input from the magnetosensory system contributes to the internal representation of space in a subterranean rodent. *J. R. Soc. Interface* **7**, 1275–1292. (doi:10.1098/rsif.2009.0551)
79. Prato FS, Desjardins-Holmes D, Keenliside LD, DeMoor JM, Robertson JA, Stodilka RZ, Thomas AW. 2011 The detection threshold for extremely low frequency magnetic fields may be below 1000 nT-Hz in mice. *Bioelectromagnetics* **32**, 561–569. (doi:10.1002/bem.20661)

80. Prato FS, Desjardins-Holmes D, Keenliside LD, DeMoor JM, Robertson JA, Thomas AW. 2013 Magnetoreception in laboratory mice: sensitivity to extremely low-frequency fields exceeds 33 nT at 30 Hz. *J. R. Soc. Interface* **10**, 20121046. (doi:10.1098/rsif.2012.1046)
81. Burda H, Begall S, Cerveny J, Neef J, Nemec P. 2009 Extremely low-frequency electromagnetic fields disrupt magnetic alignment of ruminants. *Proc. Natl Acad. Sci. USA* **106**, 5708–5713. (doi:10.1073/pnas.0811194106)
82. Naarala J, Kesari KK, McClure I, Chavarriaga C, Juutilainen J, Martino CF. 2017 Direction-dependent effects of combined static and ELF magnetic fields on cell proliferation and superoxide radical production. *Biomed. Res. Int.* **2017**, 5675086. (doi:10.1155/2017/5675086)
83. Peirson SN, Thompson S, Hankins MW, Foster RG. 2005 Mammalian photoentrainment: results, methods, and approaches. *Methods Enzymol.* **393**, 697–726. (doi:10.1016/S0076-6879(05)93037-1)
84. Kutta RJ, Archipowa N, Johannissen LO, Jones AR, Scrutton NS. 2017 Vertebrate cryptochromes are vestigial flavoproteins. *Sci. Rep.* **7**, 44906. (doi:10.1038/srep44906)
85. Vieira J *et al.* 2012 Human cryptochrome-1 confers light independent biological activity in transgenic *Drosophila* correlated with flavin radical stability. *PLoS ONE* **7**, e31867. (doi:10.1371/journal.pone.0031867)
86. Shaw J, Boyd A, House M, Woodward R, Mathes F, Cowin G, Saunders M, Baer B. 2015 Magnetic particle-mediated magnetoreception. *J. R. Soc. Interface* **12**, 20150499. (doi:10.1098/rsif.2015.0499)
87. Zhadin M, Barnes F. 2005 Frequency and amplitude windows in the combined action of DC and low frequency AC magnetic fields on ion thermal motion in a macromolecule: theoretical analysis. *Bioelectromagnetics* **26**, 323–330. (doi:10.1002/bem.20095)
88. Binhi V, Prato FS. 2017 A physical mechanism of magnetoreception: extension and analysis. *Bioelectromagnetics* **38**, 41–52. (doi:10.1002/bem.22011)
