# Supplementary material for: Magnetic Fields and Cancer: Epidemiology, Cellular Biology, and Theranostics
Source: Int J Mol Sci. 2022 Jan 25;23(3):1339. doi: 10.3390/ijms23031339 (PMC8835851; doi:10.3390/ijms23031339)
Supplement: Supplementary file 1 [file ijms-23-01339-s001.zip › Supplementary Data Set S1/MF and Cancer.Data/PDF/4023452685/1-s2.0-S0048969712003919-main.pdf]

Science of the Total Environment 426 (2012) 454–458

Contents lists available at SciVerse ScienceDirect

Science of the Total Environment

journal homepage: www.elsevier.com/locate/scitotenv

Concern that "EMF" magnetic fields from power lines cause cancer

Michael Repacholi *

Department of Information Engineering, Electronics and Telecommunications (DIET), University of Rome "La Sapienza", Via Eudossiana 18, Rome, 00184 Italy

ARTICLE INFO

Article history:
Received 29 January 2012
Received in revised form 8 March 2012
Accepted 8 March 2012
Available online 24 April 2012

Keywords:
Power lines
Magnetic fields
Childhood cancer
Health research assessment
Precautionary measures

ABSTRACT

In 2002, the International Agency for Research on Cancer (IARC, 2002) categorized extremely low frequency (ELF) (including the power frequencies of 50 and 60 Hz) magnetic fields as "possibly carcinogenic to humans." That was based on pooled analyses of epidemiological research that reported an association between exposure to low-level magnetic fields and childhood leukemia. In 2007 a task group of scientific experts convened by the World Health Organization (WHO) acknowledged the IARC categorization but found that the laboratory studies and other research results did not support the association. Taking all evidence into account WHO reported that it could not confirm the existence of any health consequences from exposure to low-level magnetic fields.

There remains continuing concern by some people that exposure to power frequency magnetic fields may cause adverse health effects, particularly childhood leukemia. Public health authorities need to fully understand the reasons for that ongoing concern and effective ways to address it. This paper describes what drives the concern, including how people perceive risks, how WHO and other public health authorities assess scientific research to determine whether health risks exist and the conclusions they have reached about power frequency magnetic fields. This paper also addresses the scientific basis of international exposure guidelines for power frequency magnetic fields and what precautionary measures are warranted to address the concern.

© 2012 Published by Elsevier B.V.

1. Introduction

Concern that magnetic fields (MF) from power lines, commonly referred to as either "electromagnetic fields (EMF)" or "extremely low frequency (ELF) fields" may cause childhood leukemia has continued to be expressed by some for many years. The common abbreviation "ELF MF" is used in this paper to refer to power frequency magnetic fields. The International EMF Project of the World Health Organization (WHO), whose mission is the attainment by all peoples of the highest possible level of health, has addressed that concern for over 15 years.

A fundamental lesson to be learned from WHO's work is that it is very important for all stakeholders to understand (a) what drives concern about EMF, (b) how scientific studies are examined by public health authorities like WHO to determine whether an exposure has been shown to cause a health effect, and (c) what the scientific studies of EMF and health effects tell us. From that understanding, authorities can identify a science-based public health approach to addressing any concerns and setting appropriate policies.

Power lines and electric appliances have separate electric and magnetic fields around them when current flows in them, not the coupled electric and magnetic fields that are called "electromagnetic" fields from higher frequency sources such as radio and TV broadcasting.

It is necessary to address the misunderstanding that arises when the media and individuals refer to power frequency fields as "radiation." Radiation is simply the manner in which energy comes from its source. A common example of radiation is heat coming from the sun or from a home heater. The term radiation, however, is commonly understood to refer to the type of energy emitted from a device like a damaged nuclear facility or an X-ray machine. Those devices produce extremely high frequency radiation, beginning at a frequency of around a billion, billion hertz. Such extremely high frequency radiation has enough energy to break or damage the bonds holding the molecules in cells together, such as DNA, and is known scientifically as "ionizing" radiation. It is well established from research that ionizing radiation can break or damage the bonds of DNA and that can result in cancer.

Power frequency (50 and 60 Hz) fields are at the opposite end of the electromagnetic spectrum and produce extremely low frequency fields. Those fields are not capable of delivering enough energy to break any bonds between molecules such as DNA. Power frequency ELF MF, therefore, is "non-ionizing" radiation and referring to it as "radiation" can mislead people into believing it has the same capability to damage cells as does ionizing radiation from devices such as X-ray machines.

2. Bases for ELF MF concern

The principal impetus for concern about ELF MF continues to be proposals by utilities to build new high voltage power transmission facilities in the vicinity of residential areas. Those lines carry large

* Tel.: +39 3397698787.
E-mail address: mrepacholi@yahoo.com.

0048-9697/$ – see front matter © 2012 Published by Elsevier B.V.
doi:10.1016/j.scitotenv.2012.03.030

amounts of electricity from generating stations to areas where there is a public demand for it, often past residences that do not benefit directly from the power lines. In addition, some people living near power lines worry that their children may be at some health risk.

Some scientific studies have suggested there is a relationship between the ELF MF from power lines and childhood cancer while other studies suggest the opposite. This apparent disagreement is addressed in more detail later. There are many personal factors, however, that can influence a person's concern about a potential health risk (WHO, 2002). Those factors generally fall into 3 categories: personal factors, the nature of the risk, and external factors. Personal factors include the person's age, sex, education, social and cultural background, and personal experiences. Surveys have identified several characteristics about the nature of a perceived risk that can drive concern, including unfamiliarity with the underlying source of exposure (WHO, 2002). While electric power facilities, such as transmission and distribution lines, and electrical appliances have been familiar to people for nearly a century, the ELF MFs resulting from their operation are unfamiliar because they cannot be seen, can be difficult to understand, and there is often inadequate, conflicting, or incomplete information about what scientific studies tell us about health effects and exposure levels. In addition, people are less familiar with large transmission lines than they are with the much smaller distribution lines that are in their neighborhoods and with the electrical devices they use daily.

People typically are more concerned about a perceived risk if they have no choice about being exposed to it or they have a lack of control. For example, most people feel reasonably safe when driving a vehicle because it is their choice to do so and they can control their speed and other actions, even though there are very well established risks to health from driving.

Some people have more concern about exposure to ELF MF from power transmission lines, even though they may have greater exposure from electric wiring and appliances in their home, over which they do have control. The concern appears to arise because people feel they do not have a choice about a transmission line being located near their home and thus no control over the level of their exposure to the magnetic fields from it. This occurs even though they can participate in regulatory proceedings about whether and where transmission facilities are to be located because they may feel they have no personal control over the outcome of those proceedings.

People also typically have concern about exposure to perceived risks if they see no personal benefit that compensates for their exposure. For example, they may see no benefit from a transmission line that is near to but not supplying electricity to their home, even though all of us use electricity that has been transmitted on lines running past other people's homes.

Some health effects are more dreaded than others and that can increase concern about a risk. Cancer is not uncommon (about 1 in 5 people will get some form of it in their lifetime), but it remains a dreaded disease. Health effects in children, particularly cancer, naturally increases concern.

Non-personal (external) factors can also strongly drive concern. If easily understood scientific information is not readily available or is not provided in easily understood language by non-experts, concern increases because of our natural fear of the unknown or that people feel they may be misled by use of scientific jargon. Providing accurate scientific information to the public is a responsibility of both government authorities and utilities. However, we must recognize that such information will not reduce the concern of those who have already made up their mind. Information that presents an unbalanced view of the science – in either direction – can also increase concern because people sense that the source of the information has a hidden agenda and that feeds their fear of the unknown. Reliable, balanced information can be obtained from the WHO International EMF Project web site.

Public concern can be increased if the media only run stories on studies that report a health effect and ignore the studies that report no health effect. Concern increases more when government officials do not listen to peoples' concerns, do not understand its bases, do not set up a process for a fair hearing of all viewpoints, and when they make decisions to merely please either a utility or an opposition group rather than deciding the health effects issue on scientific merit.

3. Public ELF MF exposure

Everyone is exposed to ELF MF whenever electric current flows in household appliances, office equipment, power lines, medical devices, personal electric appliances such as hair dryers, shavers etc. WHO has very helpful material about people's ELF exposure on its website (http://www.who.int/peh-emf/en/). A key point WHO makes, based on their detailed study, is that "even the exposure of people living in the vicinity of high voltage transmission lines differs very little from the average exposure in the population" (WHO, 2012).

4. Assessment of scientific studies

When making a health risk assessment of scientific research, all relevant studies should be considered with an open mind. Limiting consideration to only those studies that support a preconceived conclusion is advocacy, not science. Each of the studies should be evaluated individually to determine how much confidence should be placed in the results. The evaluation should take into account the quality of the design, conduct of the study and analysis of the data. If a study is poorly designed, conducted, or analyzed and that produces a serious flaw, one should have less confidence in the study result whether it does or does not it report a health effect.

After studies have been evaluated individually, the data from all of the studies should be considered and synthesized to determine whether or not the studies, as a whole, show that the exposure causes a health effect. The synthesis must be objective, giving appropriate weight to studies based on their quality, not on their results. Finally, the synthesized data should be objectively assessed. The "Hill Criteria" (Hill, 1965) are commonly recognized by scientists as identifying factors that should be considered to determine whether an exposure is the cause of the health effect. While there are a number of factors to consider, there are 4 key questions:

a) Do the studies overall show a statistically significant and strong relationship between the exposure and the health effect? Statistical significance is important because it provides a scientifically accepted criterion for identifying study results that occur by chance from results that warrant some confidence. The stronger the statistically significant relationship (i.e., the greater the risk estimate or correlation) between the exposure and the health effect, the more confidence one can have that the exposure causes the health effect.
b) Are the results of different types of studies consistent? When considering effects such as cancer, the two most important types of studies for assessing whether there is a causal relationship are epidemiology studies and in vivo animal studies. Epidemiology studies are observational (i.e., non-experimental) studies of whether an exposure is associated with the incidence of a health effect in a particular population group. In vivo studies are controlled laboratory experiments in which animals are exposed to accurately known doses to determine whether they cause a health effect. One can have more confidence in the overall results of the epidemiology studies when they are generally consistent with each other. The same is true for in vivo studies. Both types of studies have different limitations so one can have much more confidence about whether an exposure causes a health effect if the results of both types of studies are consistent with each other. If the results

of the epidemiology and in vivo studies are inconsistent, there is doubt about whether the results of either type of study are correct and good reason to doubt that the studies considered as a whole show that the exposure causes the health effect.

c) Are the results of the studies generally consistent in finding a statistically significant dose–response relationship between the exposure and the incidence of the health effect? This question reflects the general rule that the greater the exposure (dose) the greater the health effect. Typically this means that as the exposure is increased (either in intensity or time or both), the greater the incidence or severity of the health effect in the exposed group. If the results of the epidemiology and in vivo studies are generally consistent in reporting a statistically significant dose–response relationship, that substantially increases confidence that the exposure causes the health effect. If not, there is good reason to doubt whether it does.
d) Is it biologically plausible that the exposure is capable of causing the health effect? If it is biologically plausible, one can have more confidence that it does. If it is not, there is reason to doubt whether the exposure causes the health effect. A key element in answering this question is whether research has consistently demonstrated a biological mechanism by which an exposure can cause the health effect. An example of such a mechanism is the consistently demonstrated ability of exposure to ultraviolet radiation from the sun to cause damage to the DNA in skin cells, which then can result in skin cancer.

A clear explanation of how responsible public health authorities either conduct assessments or rely on assessments of the scientific studies, for example WHO, is an important first step in addressing the concern about ELF MF.

5. Conclusions from scientific research on health

Research on EMF and possible health effects has been conducted for over 44 years, and as of February 2012 more than US $490 million has been spent on it and approximately 2900 ELF studies have been conducted that only relate to cancer (PubMed, 2012). While uncertainties remain as they do with almost all exposure and are being researched, a wide variety of possible health effects have been studied and so there is now a huge amount of scientific knowledge about EMF and possible health effects, particularly cancers.

WHO convened a Task Group of internationally recognized experts to examine the scientific research. It also addressed questions related to the four Hill Criteria described earlier. The conclusion of the Task Group is described on WHO's website: "Based on a recent in-depth review of the scientific literature, the WHO concluded that current evidence does not confirm the existence of any health consequences from exposure to low level electromagnetic fields" (WHO, 2012). Government appointed scientific panels and agencies have also found that ELF MF has not been shown to cause any health effects (e.g., U.S. National Institute of Environmental Health Sciences (NIEHS, 2002), Health Canada (2010), The Australian Radiation Protection and Nuclear Safety Agency (ARPANSA, 2012), Swedish Radiation Protection Authority (SSM, 2010), European Commission (SCENIHR, 2009)).

WHO provides a clear description of the childhood leukemia issue in the Fact Sheet on its website: "In 2002, IARC published a monograph classifying extremely low frequency magnetic fields (that included the power frequencies of 50 and 60 Hz) as "possibly carcinogenic to humans" (IARC, 2002). This classification is used to denote an agent for which there is limited evidence of carcinogenicity in humans and less than sufficient evidence for carcinogenicity in experimental animals {other examples include coffee and welding fumes}" (WHO, 2007a). The classification was based on a statistical association calculated from a pooled analysis of epidemiology studies of childhood leukemia, but as WHO points out the analysis was "weakened by methodological problems." It is important to recognize, as the WHO Task Force pointed out in its report, that "virtually all of the laboratory evidence and the mechanistic evidence fail to support" the reported statistical association between ELF MF and childhood leukemia (WHO, 2007a,b).

The question of whether it is biologically plausible that ELF MF exposure is capable of causing a health effect is important and can also help put study results in perspective. ELF MF is a physical force and Maxwell's laws of electromagnetism are accepted as accurately describing the interactions between ELF MF fields and all matter, including biological matter (Valberg et al., 2007). For ELF MF to cause a health effect there must be a mechanism by which the physical force can cause or contribute to an alteration of the structure or function of a body's cells or the molecules they contain, such as DNA (Parkinson, 1985). More than a 1,000 mechanism studies have been conducted over decades but science simply has not been able to confirm there is a mechanism by which the ELF MF people are normally exposed to can cause any health effect (WHO, 2007a,b).

Overall, the WHO Task Group found no substantive health issues related to power frequency electric fields at the levels generally encountered by the public (WHO, 2007a,b) and in this decade little concern has been expressed about them. In the last 2 decades research has focused on magnetic fields.

Explaining the key conclusions of responsible public health authorities that conduct their own reviews, such as the Health Protection Agency in the UK, or the conclusions reached by competent peer review committees is a critical step in addressing public concerns about ELF MF.

6. The BioInitiative Report

The BioInitiative Report (2007), which has received the attention by a number of health protection agencies, puts forward a view that is entirely different from the conclusions of the experts appointed by WHO and the various governmental panels and agencies described earlier.

It contends that ELF is linked to a very wide variety of diseases and conditions, including Alzheimer's disease, heart disease, Parkinson's disease, many types of cancer, headaches, skin disorders, sleeplessness, loss of appetite, genotoxic effects, stress reactions, and fatigue. None of the many expert panels appointed by government health agencies or other public health authorities has found that ELF MF causes or contributes to any of those diseases or conditions. The Report, in section IV, recommends limiting exposure from power lines to 0.1 μT (1 mG), which would make delivery of electric power very uneconomical and is a level of exposure exceeded by common household electrical appliances. In light of its rather extreme positions, it is useful for all stakeholders to understand how the BioInitiative positions were arrived at in order to see the report for what it is and is not.

The BioInitiative Report, written with the purpose of demonstrating that the current exposure limits are inadequate and need revision, was prepared by a self-appointed group of activists and scientists. It does not use the widely accepted Hill (1965) criteria discussed earlier or any other objective criteria for examining a body of scientific studies.

The BioInitiative Report has been evaluated by experts for the European Commission and by a number of national health agencies (European Commission EMF-NET Coordinated Action, 2007; German Federal Office for Radiation Protection, 2008; Health Council of the Netherlands, 2008). The Netherlands Health Council best summarizes the evaluations: "In view of the way the BioInitiative Report was compiled, the selective use of scientific data and the other shortcomings mentioned earlier, the Committee concludes that the BioInitiative Report is not an objective and balanced reflection of the current state of scientific knowledge". In short, the BioInitiative Report should be viewed as an advocacy piece for a particular point of view rather than an objective analysis of the scientific studies.

The BioInitiative Report's conclusions are inconsistent with the detailed and balanced reviews of the WHO (2007a,b) and recent reviews conducted by committees of experts appointed by the Swedish Government (SSM, 2010) and by Health Canada (2010). Health Canada concluded that ELF MF exposure limits were not needed in Canada “because the scientific evidence is not strong enough to conclude that exposures cause health problems for the public.”

Putting the BioInitiative Report in proper perspective is necessary because the report has had wide circulation.

7. Precautionary measures

The “precautionary principle” can be used for determining if an activity or product should be prohibited or restricted even though there is not a scientific consensus that it has or will cause serious harm to the environment or public health. There are many formulations of the precautionary principle and there is seemingly endless debate about how it should be applied (Foster et al., 2000). Some formulations can be interpreted as prohibiting an activity or product unless there is absolute proof of its safety. In the European Union, where the policy receives perhaps more attention than elsewhere, a cost–benefit analysis is called for when appropriate and feasible (CEC, 2000).

Some argue that precaution justifies restricting ELF MF from power lines, while restricting ELF MF from other sources such as appliances to the same levels are not mentioned (BioInitiative Report, 2007). Based on the finding of the WHO Task Force that the science “does not confirm the existence of any health consequences from exposure to low level electromagnetic fields” and similar findings of many government appointed scientific panels and agencies cited earlier, others reasonably argue that no restrictions on ELF MF are warranted. The WHO Task Force, however, took the middle ground.

The WHO Task Force's starting point was the recommendation that policy-makers establish exposure guidelines based on those issued by the International Commission on Non-Ionizing Radiation Protection (ICNIRP, 2010) or the Institute of Electrical and Electronics Engineers (IEEE, 2002). Both organizations conducted evaluations of the scientific studies to provide the basis for their guidelines. The ELF exposure guidelines are based on all scientifically established biological effects. Those effects are acute (not chronic) nervous tissue stimulation and induction of retinal phosphenes (perception of faint flickering light) (ICNIRP, 2010). They only occur at very high field levels to which people are rarely, if ever, exposed.

The ICNIRP general public exposure guideline for ELF MF was reexamined and updated in 2010 and set to 200 micro tesla (μT) or 2000 milligauss (mG). This level was revised to take account of the results of the latest scientific studies, a change in the type of basic restriction from induced current density to induced electric field strength, and more accurate dosimetry. Many people do not realize that a high level of precaution is already built into these guidelines, which assume worst-case exposure conditions and then applies a safety factor for the general public of 50 times less than the lowest exposure established to cause any health effect. No research has established any health effects of ELF MF below the ICNIRP exposure guideline. Further, if the scientific basis for and the built-in safety factors of the ICNIRP and IEEE exposure guidelines are effectively explained, they can be an effective means for addressing concern about health effects from exposure to ELF MF.

Typical levels for a large high voltage transmission line (500 kV in the USA) under the line are on average less than 8.7 μT (87 mG) and less than 3 μT (30 mG) on average at the edge of the right of way (about 20 m from the centre of the line) (NIEHS, 2002). The right of way is the corridor under transmission lines, with a width depending on the voltage of the line, which allows space for maintenance to be carried out. Typical levels for the distribution lines, which have no right of way, and run to homes and facilities that use electric power range from 1 to 2 μT (10–20 mG) for both overhead and underground lines, with maximum levels reaching 7 μT (70 mG) under an overhead distribution line and 4 μT (40 mG) above an underground distribution line. As NIEHS (2002) notes, the ELF MF level under a 500 kV transmission line can double during peaks loads, which occurs about 1% of the time. In the UK, as in most of Europe, uses higher voltages (220–240 V versus 110 V in the USA) and so lower currents are needed to provide the same power. A typical average magnetic field under 400 kV lines is 5.7 μT (57 mG) and from 400 kV underground cables can be up to 24 μT (240 mG) directly over the cable, depending on how deep the cables are buried. If a person is 20 m from the cable the magnetic field falls to less than 1 μT (National Grid, 2012a,b). Total personal exposure from ELF MF depends on the amount of use of electrical appliances, the level of fields already existing in the home from electric wiring and on the distance of the home from power lines. It has been calculated that appliances and exposures in the home can contribute up to 50% of a person's total exposure to ELF MF (National Grid, 2012c). The ICNIRP guideline and typical average exposures to various sources of exposure to magnetic fields are show in the Table 1.

As can be seen from Table 1, even under a typical 500 kV high voltage transmission line the average ELF MF is less than 5% of the ICNIRP guideline limits. ELF MF exposures from appliances are typically more localized and fall off faster with distance from the appliance, but can be at much higher levels than from transmission or distribution lines.

The WHO Task Force recommended that the ICNIRP exposure guidelines not be reduced by national authorities “to some arbitrary level in the name of precaution” because that “undermines the scientific foundation of the guidelines” (WHO, 2007a,b).

The WHO also concluded that implementing “other suitable precautionary measures to reduce exposure is reasonable and warranted” provided that they are “very low cost” and do not compromise the “obvious health, social, and economic benefits of electric power” (WHO, 2007a). Requiring higher towers, wider rights-of way, or undergrounding of lines in an effort to reduce exposure to ELF MF can add substantial costs and thus would not be considered “very low cost” measures. A good example of a “very low cost” (perhaps no cost) measure is designing new transmission lines so that on parallel wires (conductors) current is always flowing in the opposite direction, resulting in the

Table 1
Typical average exposures to sources of power frequency magnetic fields in the UK (National Grid, 2012a,b) and the USA (NIEHS, 2002) compared to the international public exposure limits (ICNIRP, 2010).

Source of magnetic fields	Field (μT)	Field (mG)
International standards: ICNIRP (2010)	200	2000
500 kV high voltage transmission line (USA)		
Typical average under line	8.7	87
Typical average 20 m from centre of line	3	30
400 kV high voltage transmission line (UK)		
Typical average under line	5.7	57
Typical average 20 m from centre of line	2	20
24 kV distribution lines, overhead and underground (USA)		
Typical maximum over underground and beneath overhead lines	4–7	40–70
Typical average beneath overhead and over underground lines	1–2	10–20
33 kV distribution lines (UK)		
Typical average under line	1.5	15
Typical average 20 m from center of line	0.1	1
400 kV underground cables (UK)		
Typical average directly over line	20 to 24	200–240
Typical average 20 m from centre of line	<1	<10
Typical person exposures to all sources of magnetic fields		
USA mean 24 h exposure	<0.05	<0.5
Appliance: Maximum exposure to a hair dryer at about 10 cm)	<70	<700
UK average exposure on a typical day	<0.11	1.1
Appliance: using an electric razor	2000	20,000

magnetic fields from one wire partially canceling the fields from the other and thus reducing the total ELF MF (this design is often referred to as “cancellation,” “opposite” or “reverse” phasing).

The WHO Task Force recommended other policy measures as well, perhaps most importantly, open and effective communications by all stakeholders. That is the best approach for addressing the various factors that can influence a person's concern about a potential health risk, but it may not influence those who have already made up their minds.

Using precaution as the basis for requiring unnecessary and costly measures to reduce ELF MF in an effort to assuage the concern of outspoken opponents of new power lines will likely be counterproductive because it will reinforce their view that there is a scientific basis for their concern.

8. Conclusions

After more than 40 years of research and thousands of studies costing millions of dollars, science has not been able to show that ELF MF exposure below the level of the ICNIRP (2010) guidelines causes any health effects. WHO's recommendation that while research continues the ICNIRP exposure guidelines be followed and that only “very low cost” precautionary measures be taken to reduce magnetic field levels is a science-based and sensible approach. Indeed, a science-based and sensible approach is the only credible approach public health authorities can take to address concern about ELF MF.

Conflict of interest statement

Aside from the support provided by the author's institution, no funding was provided to conduct this review. The author has no conflict of interest.

References

ARPANSA. The Australian Radiation Protection and Nuclear Safety Agency. Extremely low frequency electric and magnetic fields. Cited 8 March 2012 See http://www.arpansa.gov.au/radiationprotection/basics/elf.cfm.

BioInitiative Report. A rationale for a biologically-based public exposure standard for electromagnetic fields (ELF and RF). See http://www.bioinitiative.org/report/index.htm.

CEC Commission of the European Communities. Communication from the Commission on the precautionary principle. Brussels http://ec.europa.eu/dgs/health_consumer/library/pub/pub07_en.pdf 2000. Cited 8 March 2012 See.

European Commission EMF-NET Coordination, Action. Comments on the BioInitiative Report. Cited 8 March 2012 See http://web.jrc.ec.europa.eu/emf-net/doc/efrtdocuments/EMF-NET%20Comments%20on%20the%20BioInitiative%20Report%2030OCT2007.pdf.

Foster K, Vecchia P, Repacholi M. Science and the precautionary principle. Science 2000;288:979–81.

German Bundesamt für Strahlenschutz (Federal Office for Radiation Protection, BfS). Comments on the Bioinitiative report. Cited 8 March 2012 See http://www.emf-forschungsprogramm.de/int_forschung/wirk_mensch_tier/Synopse_EMF-Studien_2008.pdf. (in German).

Health Canada. It's your health: electric and magnetic fields at extremely low frequencies. Cited 8 March 2012 See http://www.hc-sc.gc.ca/hl-vs/alt_formats/pdf/iyh-vsv/environ/magnet-eng.pdf.

Health Council of the Netherlands. BioInitiative report. Cited 8 March 2012 See http://www.gezondheidsraad.nl/en/publications/bioinitiative-report-0.

Hill AB. The environment and disease: association or causation? Proc R Soc Med 1965;58(5):295–300.

IARC Working Group on the Evaluation of Carcinogenic Risks to Humans. Non-ionizing radiation, Part 1: Static and extremely low-frequency (ELF) electric and magnetic fields. Lyon, IARC, 2002 (Monographs on the Evaluation of Carcinogenic Risks to Humans, 80).

ICNIRP International Commission on Non-Ionizing Radiation Pretection. Guidelines for limiting exposure to time-varying electric and magnetic fields (1 Hz–100 kHz). Health Phys 2010;99(6):818–36. Cited 8 March 2012 See: http://www.icnirp.de/documents/LFgdl.pdf.

IEEE Institute of Electrical and Electronics Engineers. IEEE standard for safety levels with respect to human exposure to electromagnetic fields, 0–3 kHz. IEEE standard C95.6-2002. IEEE New York, USA; 2002.

National Grid. Transmission — table of calculated magnetic fields produced by underground cables in operation in the UK. http://www.emfs.info/Sources+of+EMFs/Underground/ 2012. accessed 18-2-2012.

National Grid. What is the average field from a transmission line? http://www.emfs.info/Sources+of+EMFs/Overhead+power+lines/ 2012. accessed 18-2-2012.

National Grid. Personal exposure. EMFs info. Electric and magnetic fields. Cited 8 March 2012 See http://www.emfs.info/Sources+of+EMFs/Overhead+power+lines/ 2012.

NIEHS National Institute of Environmental Health Sciences. EMF electric and magnetic fields associated with the use of electric power, questions & answers; 2002. p. 36–7. Cited 8 March 2012 See: http://www.niehs.nih.gov/health/assets/docs_p_z/results_of_emf_research_emf_questions_answers_booklet.pdf.

Parkinson WC. Electromagnetic fields in biological studies. Ann Biomed Eng 1985;13:491–514.

PubMed Central. Database of research publications. (Cited 18 February 2012) See http://www.ncbi.nlm.nih.gov/pmc/?term=Extremely+low+frequency+fields,+cancer.

SCENIHR Scientific Committee on Emerging and Newly Identified Health Risks. Health effects of exposure to EMF. Brussels, Belgium: Directorate-General for Health and Consumers, European Commission; 2009. Cited 8 March 2012 See: http://ec.europa.eu/health/ph_risk/committees/04_scenihr/docs/scenihr_o_022.pdf.

SSM. Swedish Radiation Protection Authority. Recent research on EMF and health risk. Report 2010:44; 2010. Cited 8 March 2012 See: http://www.stralsakerhetsmyndigheten.se/Global/Publikationer/Rapport/Stralskydd/2010/SSM-Rapport-2010-44.pdf.

Valberg PA, van Deventer TE, Repacholi MH. Base stations and wireless networks — radiofrequency (RF) exposures and health consequences. Environ Health Perspect 2007;115:416–24.

WHO World Health Organization. Establishing a dialogue on risks from electromagnetic fields; 2002. Cited 8 March 2012 See: http://www.who.int/peh-emf/publications/risk_hand/en/.

WHO World Health Organization. Environmental health criteria 238, extremely low frequency fields; 2007a. Cited 8 March 2012 See http://www.who.int/peh-emf/publications/elf_ehc/en/index.html.

WHO World Health Organization. Electromagnetic fields and public health: exposure to extremely low frequency fields. Fact Sheet No. 322; 2007b. Cited 8 March 2012 See http://www.who.int/mediacentre/factsheets/fs322/en/index.html.

WHO World Health Organization. What are electromagnetic fields? Cited 8 March 2012 See http://www.who.int/peh-emf/about/WhatisEMF/en/index.html.
