# Supplementary material for: Magnetic Fields and Cancer: Epidemiology, Cellular Biology, and Theranostics
Source: Int J Mol Sci. 2022 Jan 25;23(3):1339. doi: 10.3390/ijms23031339 (PMC8835851; doi:10.3390/ijms23031339)
Supplement: Supplementary file 1 [file ijms-23-01339-s001.zip › Supplementary Data Set S1/MF and Cancer.Data/PDF/4080726443/rsif.2015.0995.pdf]

INTERFACE

rsif.royalsocietypublishing.org

Research

Cite this article: Kesari KK, Juutilainen J, Luukkonen J, Naarala J. 2016 Induction of micronuclei and superoxide production in neuroblastoma and glioma cell lines exposed to weak 50 Hz magnetic fields. *J. R. Soc. Interface* **13**: 20150995.
http://dx.doi.org/10.1098/rsif.2015.0995

Received: 16 November 2015
Accepted: 21 December 2015

Subject Areas:
biochemistry, biophysics, environmental science

Keywords:
genotoxicity, reactive oxygen species, extremely low-frequency magnetic field, exposure–response relationship

Author for correspondence:
Kavindra Kumar Kesari
e-mail: kavindra.kesari@uef.fi

THE ROYAL SOCIETY
PUBLISHING

Downloaded from https://royalsocietypublishing.org/ on 09 December 2021

Induction of micronuclei and superoxide production in neuroblastoma and glioma cell lines exposed to weak 50 Hz magnetic fields

Kavindra Kumar Kesari, Jukka Juutilainen, Jukka Luukkonen and Jonne Naarala

Department of Environmental Science, University of Eastern Finland, PO Box 1627, 70211 Kuopio, Finland

Extremely low-frequency (ELF) magnetic fields (MF) have been associated with adverse health effects in epidemiological studies. However, there is no known mechanism for biological effects of weak environmental MFs. Previous studies indicate MF effects on DNA integrity and reactive oxygen species, but such evidence is limited to MFs higher (greater than or equal to 100 μT) than those generally found in the environment. Effects of 10 and 30 μT fields were studied in SH-SY5Y and C6 cells exposed to 50-Hz MFs for 24 h. Based on earlier findings, menadione (MQ) was used as a cofactor. Responses to MF were observed in both cell lines, but the effects differed between the cell lines. Micronuclei were significantly increased in SH-SY5Y cells at 30 μT. This effect was largest at the highest MQ dose used. Increased cytosolic and mitochondrial superoxide levels were observed in C6 cells. The effects on superoxide levels were independent of MQ, enabling further mechanistic studies without co-exposure to MQ. The micronucleus and mitochondrial superoxide data were consistent with a conventional rising exposure–response relationship. For cytosolic superoxide, the effect size was unexpectedly large at 10 μT. The results indicate that the threshold for biological effects of ELF MFs is 10 μT or less.

1. Introduction

Human exposure to extremely low-frequency (ELF) magnetic fields (MF) occurs wherever electricity is generated, transmitted or used, and possible health impacts are being discussed [1]. ELF MFs were classified as a ‘possibly carcinogenic to humans’ (group 2B) by the International Agency for Research on Cancer [2]. This classification was mainly based on epidemiological studies indicating increased risk of childhood leukaemia associated with living close to power lines. Several epidemiological studies have also reported that the risk of Alzheimer’s disease may be associated with exposure to ELF MFs [3–5]. The epidemiological findings suggest increased risks above about 0.3–0.4 μT, i.e. at MF levels that are considerably lower than the reference levels of the International Commission on Non-Ionizing Radiation Protection [6], for exposure of the general public (200 μT) and occupational exposure (1000 μT). There are currently no generally accepted mechanisms that could explain biological effects of weak ELF MFs at the levels suggested by epidemiological studies, although hypothetical mechanisms have been suggested, and there is wide interest in magneto-sensitive radical pair reactions as the basis for biological effects of weak MFs [7–11]. Because of the very low interaction energy, direct DNA damage by ELF MFs is not plausible, but there is evidence that ELF MFs might enhance the effects of DNA damaging agents [12].

We have previously shown that exposure to ELF MFs (50 Hz, 100–300 μT) alter cellular responses to DNA damage induced by menadione (MQ), resulting in decreased integrity of the genome [13,14]. A more recent study also indicated that similar exposure may induce genomic instability (persistently elevated

© 2016 The Author(s) Published by the Royal Society. All rights reserved.

Downloaded from https://royalsocietypublishing.org/ on 09 December 2021

frequency of genetic changes in the progeny of exposed cells), which is highly relevant for possible carcinogenic effects [15]. These findings may serve as a basis for understanding how weak environmental MFs could enhance cancer-relevant biological processes. However, all these experiments were conducted using MFs of 100 μT or higher, so they do not allow the estimation of the likelihood of biologically meaningful effects at very low MF levels (below 1 μT) that have been associated with human health in epidemiological studies.

In this study, we assessed cellular changes induced by ELF MFs at 10 and 30 μT to explore the exposure–response relationship below 100 μT. In addition to the human SH-SY5Y neuroblastoma cells used in previous studies, rat C6 glioma cells were used to shed light on the generalizability of the results obtained with SH-SY5Y cells. Micronuclei were assessed as a measure of genotoxicity, and cytosolic and mitochondrial superoxide concentrations as indicators of changes in reactive oxygen species (ROS). The rationales for measuring these endpoints were our own previous findings [15], as well as other studies suggesting ELF MF effects on endpoints related to genotoxicity and ROS [16–18]. The link between micronuclei and adverse health effects is obvious, as genotoxicity is a well-known mechanism of carcinogenesis, and it has also been implicated in Alzheimer's disease [19]. The link between ROS and human health are partly related to their physiological role [20], and they also contribute to a wide range of pathologies and many of the implicated diseases (cancers, cardiovascular and neurological diseases) are leading causes of death [21–23].

MQ was included as a cofactor in our previous studies, as ELF MFs seemed to alter cellular responses to MQ-induced DNA damage [13,14]. Although MF exposure was found to induce cellular changes also without MQ in the latest study [15], it was included as a cofactor in the present study to enhance comparability with previous studies. MQ also served as a known inducer of DNA damage and ROS, to confirm that the assays responded as expected.

2. Material and methods

2.1. Cell cultures

The human neuroblastoma cell line, SH-SY5Y (acquired from Dr Sven Påhlman, University of Uppsala, Sweden) was grown in Dulbecco's modified Eagle's medium (DMEM) containing 4.5 g l^{-1} glucose, 10% fetal bovine serum (FBS) and a mixture of 50 U ml^{-1} penicillin/50 μg ml^{-1} streptomycin (Carlsbad, USA). The rat C6 glioma cell line (acquired from Prof. Nikolaus Plesnila, Institute for Stroke and Dementia Research (ISD), LMU Munich Medical School, Munich, Germany) was grown in DMEM containing 1 g l^{-1} glucose, 10% FBS and a mixture of 50 U ml^{-1} penicillin/50 μg ml^{-1} streptomycin. Both cell lines were maintained at 37°C and 5% CO_2 in a humidified atmosphere. The cells were detached by 0.02% EDTA (prepared in Ca^{2+}- and Mg^{2+}-free phosphate-buffered saline), with 0.1% trypsin added for the C6 cells.

For the superoxide assays, 2×10^5 neuroblastoma SH-SY5Y cells or 3×10^4 glioma C6 cells were seeded on 48-well plates (Costar, Corning, NY, USA) 20 h prior to the onset of MF exposure. In the micronucleus assays, 1×10^5 SH-SY5Y cells and 3.6×10^3 C6 cells were seeded on 48-well plates.

2.2. Experimental design

Twenty hours after seeding, cells were exposed to a 50 Hz MF for 24 h, using magnetic flux densities of 0 (sham exposure, power supply of the MF exposure system turned off), 10 or 30 μT. After 24 h of MF exposure or incubation (control cells), the cells were exposed to MQ (or incubated) for 3 h. In the cytosolic and mitochondrial superoxide assays, cells were taken into assays immediately after the MF exposure. A series of MQ concentrations was selected so that only the highest concentration slightly decreased viability of the cells. The MQ concentrations were 1, 5, 10 and 20 for SH-SY5Y cells and 1, 5, 10, 15, 20 and 50 μM for C6 cells.

2.3. Magnetic field exposure

The exposure system with 50 Hz, 100 μT has been described previously by Markkanen *et al.* [13] and Luukkonen *et al.* [15]. In short, the 50 Hz signal was generated by a Wavetek Waveform Generator model 75 (Wavetek, San Diego, CA, USA) and amplified by a Peavey M-3000 Power Amplifier (Peavey Electronics corp., Meridian, MS, USA). Horizontal MF was generated by a pair of coils (340 × 460 mm) in a Helmholtz-type configuration (220 mm distance between the coils) inside a temperature-controlled cell culture incubator (Heraeus HERACell) with 5% CO_2. The cell culture dishes were placed at the centre of the MF coil system, where the MF is homogeneous. The control cells were incubated in an identical incubator. The background AC MF level inside the incubators varied with time due to the on–off cycle of the heating system, and there were also minor differences between the four locations used for cell cultures. The maximum (short peak value when heating is switched on by the thermostat, in the location with the highest field) was 2.35 μT in the control incubator and 1.83 μT in the incubator used for MF exposure, and the minimum values (heater off) were about 0.01 μT. The predominant direction of the background AC MF was horizontal, from front to back of the incubator (perpendicular to the experimental 50 Hz field). The static MF in the incubators was almost vertical, and it was 29.5 μT in the control incubator and 27.5 μT in the incubator used for MF exposure. Magnetic flux density was monitored with a Hirst GM08 Gaussmeter and Hirst Axial Fluxgate Probe AFG100 (Hirst Magnetic Instruments Ltd, Cornwall, UK).

2.4. Micronucleus assay

The flow cytometric micronucleus analysis described by Bryce *et al.* [24] was used. The cells were first washed twice after the MQ treatment (or incubation). As micronucleus formation requires at least one cell division after exposure, SH-SY5Ycells were then incubated for 72 h and C6 cells for 24 h. The DMEM medium was then removed from the 48-well plates and the plates were incubated for 20 min on ice. Thereafter, 150 μl cold EMA solution (8.5 μg ml^{-1} in 2% FBS in PBS) was added into each well, followed by 30 min of light activation on ice under a light bulb. After 30 min, EMA solution was removed and washed once with 500 μl of 2% FBS in PBS (w/o Ca^{2+} and Mg^{2+}, +4°C). Thereafter, 250 μl cold Lysis 1 solution (0.3 μl IGEPAL ml^{-1}, 0.584 mg NaCl ml^{-1}, 0.5 mg RNase A ml^{-1}, 1 mg sodium citrateml^{-1} and 0.4 μM SYTOX Green in MilliQ-water) was added to each well followed by 1 h of incubation at 37°C in a CO_2 incubator. Subsequently, 250 μl of Lysis 2 solution (15 mg citric acid ml^{-1}, 85.6 mg sucrose ml^{-1}, 0.4 μM SYTOX Green and one drop of 6 μm fluorescent beads (Molecular Probes, Eugene, USA), +20°C) was added to each well followed by 30 min of incubation at room temperature in the dark. All samples from each well were then transferred to flow cytometer tubes and analysed by a flow cytometer (Becton Dickinson FACScalibur, Becton Dickinson, San Jose, CA, USA). Data were acquired and analysed by CellQuest Pro software v. 5.2.1 (Becton Dickinson, San Jose, CA, USA). A total of 2×10^5 gated events were scored per sample. A 3 h treatment with methyl methanesulfonate (Sigma-Aldrich, Germany) was used

Downloaded from https://royalsocietypublishing.org/ on 09 December 2021

Table 1. Micronucleus frequency and cytosolic and mitochondrial superoxide concentrations in human neuroblastoma SH-SY5Y cells after combined exposure to sham MF treatment and menadione (MQ). *p*-Values are given for the effects of the two factors (sham, MQ) and their interaction. The values presented here are mean ± s.e.m.

	MQ concentration (μM)					*p*-values from factorial ANOVA		
	0	1	5	10	20	sham	MQ	sham × MQ
micronuclei (%)								
control	0.29 ± 0.07	0.28 ± 0.08	0.32 ± 0.06	0.42 ± 0.05	0.52 ± 0.03	0.200	0.004	0.965
sham	0.35 ± 0.04	0.35 ± 0.07	0.32 ± 0.07	0.51 ± 0.09	0.57 ± 0.05			
cytosolic superoxide (RFU)								
control	183 ± 11	191 ± 21	168 ± 11	183 ± 21	187 ± 11	0.070	0.915	0.951
sham	203 ± 12	196 ± 17	198 ± 14	199 ± 18	206 ± 6			
mitochondrial superoxide (RFU)								
control	1759 ± 43	1565 ± 135	1691 ± 88	1549 ± 105	2083 ± 304	0.585	0.024	0.977
sham	1888 ± 43	1693 ± 57	1725 ± 109	1481 ± 176	2165 ± 352			

as a positive control in the micronucleus assay, 25 μg ml^{-1} for the SH-SY5Y cells and 40 μg ml^{-1} for the C6 cells.

2.5. Mitochondrial and cytosolic superoxide production

Mitochondrial and cytosolic superoxide productions were analysed as described previously [14]. Briefly, mitochondrial superoxide production was measured by MitoSOX Red (3,8-phenanthridinediamine, 5-(6′-triphenylphosphoniumhexyl)-5,6-dihydro-6-phenyl) fluorescent probe using a final concentration of 1 μM and 492 nm excitation/595 nm emission wavelengths. Production of cytosolic superoxide was measured by the DHE (dihydroethidium), probe using a final concentration of 10 μM and 485 nm excitation/595 nm emission wavelengths.

Immediately after the exposures, the medium was removed from the 48-well plates and the cell cultures were loaded (30 min, +20°C, in dark) with the assay-specific probe in 0.5 ml of buffer. Thereafter, fluorescence was measured by a multiwell fluorometer (Perkin Elmer HTS 7000 Plus, Norwalk CT, USA). A blank (no cells) was included in all measurements. Blank values were subtracted from the absolute values.

Fluorometric detection using DHE and MitoSOX Red may not be fully specific to superoxide [25]. Unspecificity of the probes was not a problem in this study, as the aim was to explore biological responses to MFs below 100 μT rather than to measure superoxide levels with absolute specificity. The reader should be aware of the limitations of the probes but, for simplicity, we use the terms 'cytosolic superoxide' and 'mitochondrial superoxide' throughout the paper for the ROS levels measured by DHE and MitoSOX Red, respectively.

2.6. Viability of cells

Viability of cells was assayed with propium iodide (PI) by adding 50 μM PI (Molecular Probes) into the samples before incubation for 20 min in the dark (+20°C). This was followed by measurement of fluorescence (Perkin Elmer HTS 7000 Plus, Norwalk CT, USA; excitation 540 nm/emission 610 nm). The samples were then supplemented with 160 μM digitonin, incubated on a plate shaker for 20 min (in the dark, +20°C), and fluorescence was measured as describe above. Digitonin was used for demolishing cell membrane integrity. This enables PI to enter all cells, allowing calculation of viability from the relative PI value before and after the addition of digitonin [26]. The maximum PI fluorescence value (after digitonin treatment) reflects the number of cells, and also enabled possible intergroup differences in cell count to be checked (due to, e.g. differences in proliferation).

2.7. Statistical analysis

As a balanced factorial design was used in this study, factorial ANOVA was performed with MF (or sham MF) and MQ as fixed factors and replicate as a random factor. The interactions MF × MQ (or sham MF × MQ) were included in the model. The general linear model procedure of SPSS (SPSS Inc., Chicago, IL, USA) was used for the analysis. Three independent experiments with three (superoxide levels) or two (micronuclei) samples per group were performed. The factorial ANOVA combines evidence for MF effects over all MQ levels (five to seven MQ concentrations), resulting in much higher statistical power than would be reached in a simple experiment with two to three replicates and three samples in each replicate. A *p*-value of less than 0.05 was considered statistically significant.

3. Results

3.1. Sham exposure experiments

Sham exposure experiments were performed to check that the MF exposure system itself (without current connected and therefore without an MF) would not cause biological responses by changing the culture conditions. None of the biological endpoints measured was significantly affected by sham exposure (tables 1 and 2). Cytosolic superoxide level in SH-SY5Y cells was slightly higher in the sham-exposed than in control cells, but the difference was not statistically significant ($p = 0.07$), and no indication of an effect on cytosolic superoxide was seen in the C6 cells. This was the only nearly significant *p*-value for the sham treatment. In contrast to the sham MF exposure, MQ affected micronucleus frequency and mitochondrial superoxide level statistically significantly in both cell lines. Cytosolic superoxide level was not affected by MQ in the sham exposure experiments.

3.2. Viability of cells and effects on proliferation

Viability of both cell lines was generally decreased at the highest MQ concentration used, and this effect was statistically

Downloaded from https://royalsocietypublishing.org/ on 09 December 2021

Table 2. Micronucleus frequency and cytosolic and mitochondrial superoxide concentrations in rat C6 glioma cells after combined exposure to sham MF treatment and menadione (MQ). *p*-Values are given for the effects of the two factors (sham, MQ) and their interaction. The values presented here are mean ± s.e.m.

	MQ concentration (μM)							*p*-values from factorial ANOVA		
	0	1	5	10	15	20	50	sham	MQ	sham × MQ
micronuclei (%)										
control	3.88 ± 0.62	3.68 ± 1.09	4.50 ± 1.00	5.32 ± 1.57	5.79 ± 1.72	7.55 ± 1.60		0.744	0.010	0.992
sham	3.97 ± 0.41	3.63 ± 0.82	4.32 ± 1.02	5.49 ± 1.49	5.67 ± 0.77	6.51 ± 2.03				
cytosolic superoxide (RFU)										
control	92 ± 9	83 ± 8	79 ± 4	83 ± 4	95 ± 16	119 ± 20	86 ± 17	0.454	0.211	0.998
sham	86 ± 11	91 ± 10	93 ± 11	90 ± 14	101 ± 22	130 ± 27	91 ± 28			
mitochondrial superoxide (RFU)										
control	644 ± 86.1	474 ± 132	579 ± 89.3	466 ± 157	575 ± 131	395 ± 87	746 ± 122	0.170	0.026	0.994
sham	735 ± 97	610 ± 95	692 ± 98	501 ± 97	571 ± 16	479 ± 56	816 ± 36			

significant for SH-SY5Y cells in both the 10 and 30 μT experiments, and in the 10 μT experiment for C6 cells (figure 1). No MF effects or MQ × MF interactions on viability were observed. Similar effects were observed in the sham exposure experiments: cell viability was statistically significantly decreased by MQ in both cell lines (by about 5–10% in the highest MQ dose groups), but was not affected by sham MF exposure (data not shown). The maximum PI fluorescence values after digitonin treatment (used as a measure of the final number of cells) did not show any differences between the MF-treated groups and corresponding control groups, indicating that MF exposure did not affect proliferation. Also, no MQ-related differences in this measure were observed at most of the MQ doses used. Apparently, MQ effects on proliferation were not observable immediately after the 3-h MQ treatment. A slight decrease in cell number was seen in cells exposed to the highest doses of MQ, which is explainable by the reduced viability observed at these doses.

3.3. Formation of micronuclei

In SH-SY5Y cells, the frequency of micronuclei was higher in almost all MF-exposed groups both at 10 and 30 μT than in the corresponding control groups (figure 2*a*,*b*). However, the MF effect was significant only at 30 μT. The frequency of micronuclei increased consistently with increasing MQ dose, and the effect of MQ was statistically significant in both 10 and 30 μT experiments. The MQ × MF interaction was significant in the 30 μT experiment, which reflects the fact that the apparent MF effect was clearly larger than in other groups in the group exposed to 20 μM MQ. The positive control increased the level of micronuclei (0.417 ± 0.032) statistically significantly ($p < 0.001$) in SH-SY5Y cells treated with methyl methane sulfonate in comparison to control cells (0.109 ± 0.017).

In C6 cells, no statistically significant MF effects or MQ × MF interactions were observed, although the micronucleus frequency was slightly higher in most MF-exposed groups compared to the corresponding control or MQ-only exposed groups (figure 2*c*,*d*). A rising trend with increasing MQ was seen in both cases, but the MQ effect was significant only in the 10 μT experiment. Also in the glioma C6 cells, the positive control (methyl methane sulfonate for 3 h) increased the level of micronuclei (3.59 ± 0.45) statistically significantly ($p = 0.014$) compared with the control cells (2.25 ± 0.21). The values presented here are mean ± s.e.m.

3.4. Cytosolic superoxide production

In SH-SY5Y cells, no MF effects on cytosolic superoxide level were found (figure 3*a*,*b*), consistent with previous findings [15]. An MQ-related increase of cytosolic superoxide was seen at the dose of 20 μM, and the MQ effect was statistically significant in both the 10 and 30 μT experiments. This result is in contrast with the sham exposure experiment, in which MQ did not affect the cytosolic superoxide level even at 20 μM. This difference, together with the apparent lack of effect at lower MQ doses (including 10 μM), may indicate that the MQ effect on cytosolic superoxide levels is very sensitive to changes in MQ concentration, and possibly to other differences in experimental conditions.

In C6 cells, the cytosolic superoxide level was systematically higher in the MF-exposed groups than in the

Downloaded from https://royalsocietypublishing.org/ on 09 December 2021

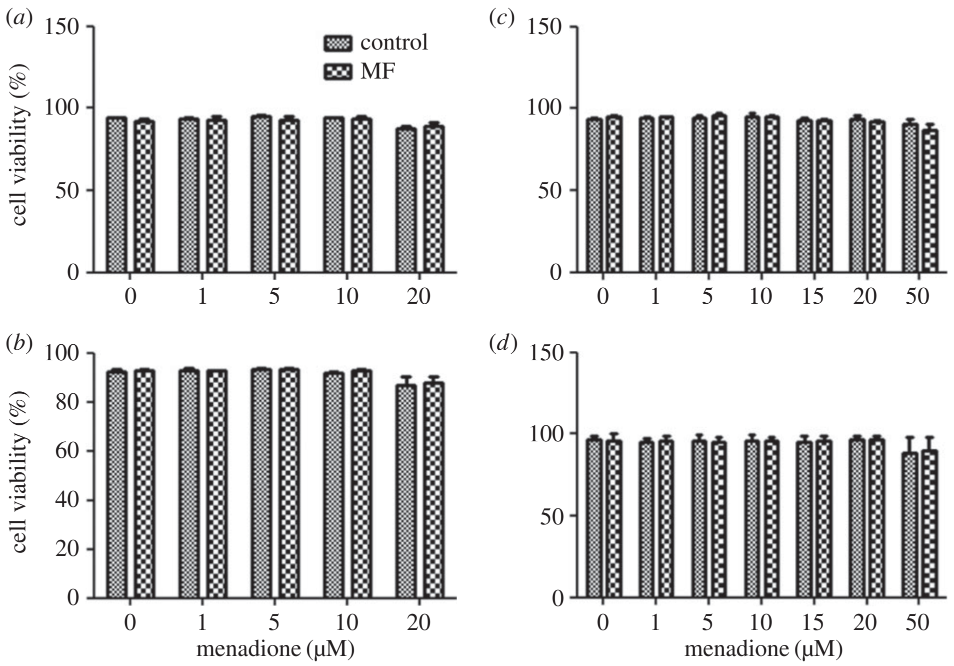

Figure 1. Viability of cell in human SH-SY5Y neuroblastoma (*a*,*b*) and rat C6 glioma (*c*,*d*) cells exposed to 50 Hz MFs at 10 μT (*a*,*c*) or 30 μT (*b*,*d*). The MF treatments were combined with different menadione (MQ) concentrations. The data shown are mean ± s.e.m. values. Three experiments were performed with three samples per group in each experiment. Factorial ANOVA was used for statistical testing; statistically significant effects for the two factors (MF and MQ) and their interactions are reported below. SH-SY5Y, 10 μT experiment: MQ $p < 0.001$. SH-SY5Y, 30 μT experiment: MQ $p = 0.006$. C6 cells, 10 μT experiment: MQ $p < 0.001$. C6 cells, 30 μT experiment: none.

corresponding control groups, and the MF effect was statistically significant at both 10 and 30 μT (figure 3*c*,*d*). Surprisingly, the MF effect was more pronounced at 10 μT than at 30 μT, suggesting a non-conventional exposure–response relationship. No MQ effect was found in this cell line, consistent with what was observed in the sham exposure experiment. The unexpected 'decrease' (not statistically significant) at high MQ levels is at least partly explained by an outlier, a single exceptionally low value in the 50 μM group (the impact of the outlier is also visible in the width of the error bar).

3.5. Mitochondrial superoxide production

In SH-SY5Y cells, the mitochondrial superoxide level was systematically higher in the MF-exposed than in the corresponding control groups, particularly in the 30 μT experiment (figure 4*a*,*b*). However, there was no statistically significant MF effect. This result differs from the results of our earlier study, in which a 100 μT MF (using an identical exposure protocol) increased the mitochondrial superoxide level significantly [15]. A statistically significant MQ effect was observed in both the 10 and 30 μT experiments.

In C6 cells, the production of mitochondrial superoxide was systematically higher in the MF-exposed groups than in the corresponding control groups, and a statistically significant MF effect was observed at both 10 and 30 μT (figure 4*c*,*d*). Statistically significant MQ effects on mitochondrial superoxide were also found in both the 10 and 30 μT experiments.

3.6. Superoxide measurements immediately after magnetic field exposure

Measurements reported in figures 3 and 4 were done after the MQ treatment, i.e. 3 h after the end of the 24-h MF exposure, but cytosolic and mitochondrial superoxide levels were also measured immediately after the MF exposure, without MQ treatment. No significant MF effects on cytosolic or mitochondrial superoxide levels were observed in these measurements (table 3).

3.7. Exposure–response relationships

For easier judgement of the exposure–response relationships, the data on micronucleus frequencies and cytosolic and mitochondrial superoxide levels were plotted as a function of magnetic flux density (figure 5). As the MF effect (when such an effect was observed) was similarly observed at all MQ levels (figures 2–4), the data of all MQ levels (including zero MQ) were pooled for figure 5: for each MQ–MF combination, a relative value in the MF-treated group was calculated by dividing the value observed in the MF group by the value observed in the control group with same MQ concentration. For comparison, also 100-μT data are shown for the SH-SY5Y cells, as such data obtained with an identical study protocol were available from our earlier studies [14,15]. Micronucleus frequency showed a rising trend with increasing magnetic flux density in SH-SY5Y cells (figure 5*a*). As described above, this effect was significant at 30 μT (but not at 10 μT) in this study, and it was also significant ($p < 0.01$) at 100 μT in the previous study [14]. In C6 cells, relative micronucleus frequency was only slightly above 1.0 at both field strengths (figure 5*d*), and (as described above) no statistically significant MF effect on micronuclei was observed in this cell line. The cytosolic superoxide level showed no consistent exposure response pattern (figure 5*b*) and no statistically significant effects on SH-SY5Y cells in this study at 10 and 30 μT or at 100 μT in the previous study [15]. In C6 cells, in contrast, the MF effect on the cytosolic superoxide level was observed (and

Downloaded from https://royalsocietypublishing.org/ on 09 December 2021

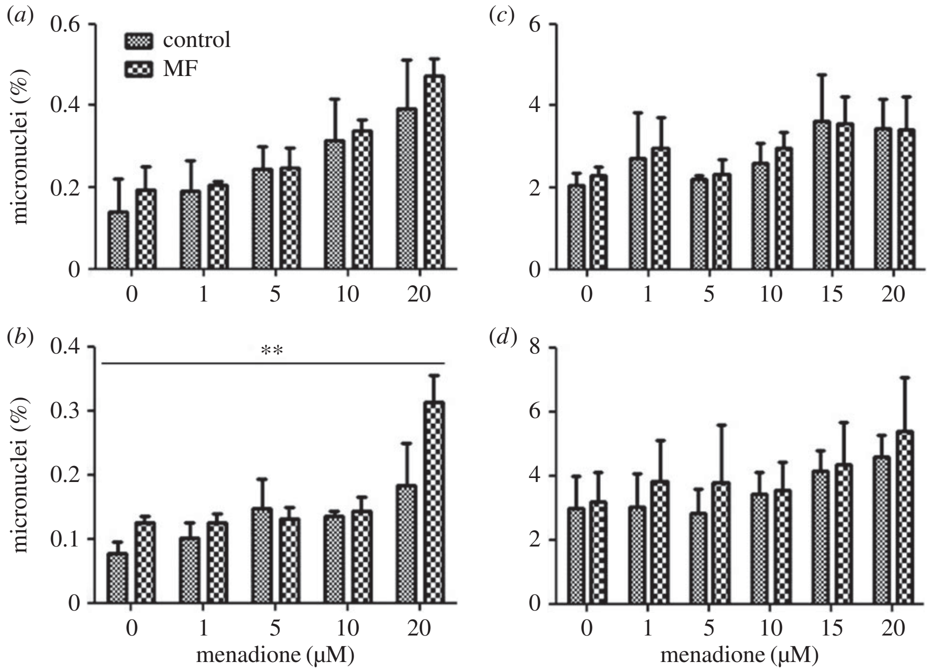

Figure 2. Frequency of micronuclei in human SH-SY5Y neuroblastoma (*a*,*b*) and rat C6 glioma (*c*,*d*) cells exposed to 50 Hz MFs at 10 μT (*a*,*c*) or 30 μT (*b*,*d*). The MF treatments were combined with different menadione (MQ) concentrations. The data shown are mean ± s.e.m. values. Three experiments were performed with two samples per group in each experiment. Factorial ANOVA was used for statistical testing; statistically significant effects for the two factors (MF and MQ) and their interactions are reported below. SH-SY5Y, 10 μT experiment: MQ $p < 0.001$. SH-SY5Y, 30 μT experiment: MF $p = 0.002$; MQ $p < 0.001$; MF × MQ $p = 0.024$. C6 cells, 10 μT experiment: MQ $p = 0.019$. C6 cells, 30 μT experiment: none. The significances for the MF effects are given in the figure: **$p < 0.01$.

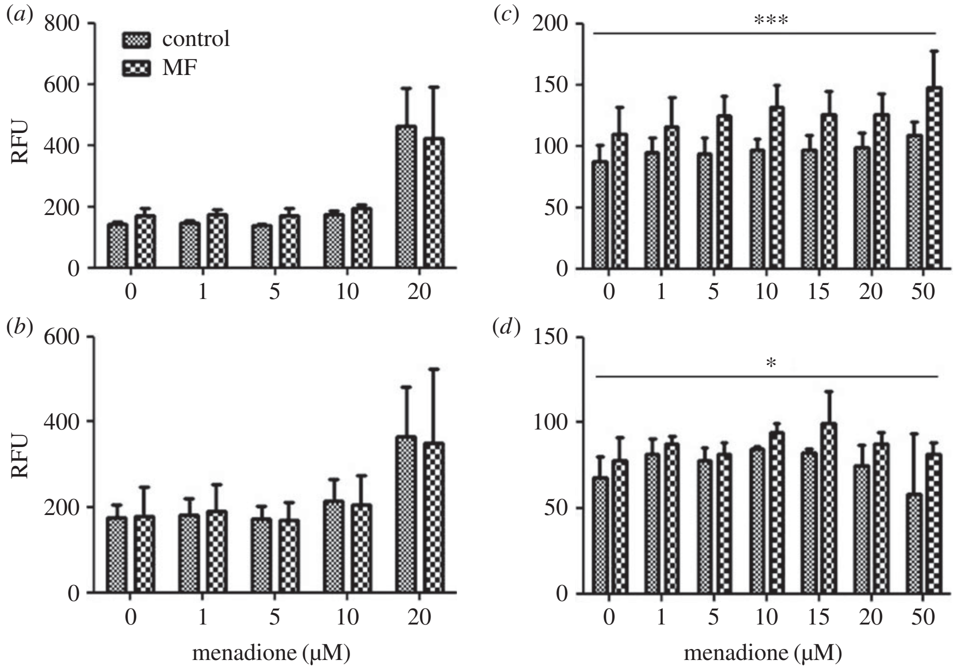

Figure 3. Cytosolic superoxide level in human SH-SY5Y neuroblastoma (*a*,*b*) and rat C6 glioma (*c*,*d*) cells exposed to 50 Hz MFs at 10 μT (*a*,*c*) or 30 μT (*b*,*d*). The MF treatments were combined with different menadione (MQ) concentrations. The data shown are mean ± s.e.m. values. Three experiments were performed with three samples per group in each experiment. Factorial ANOVA was used for statistical testing; statistically significant effects for the two factors (MF and MQ) and their interactions are reported below. SH-SY5Y, 10 μT experiment: MQ $p < 0.001$. SH-SY5Y, 30 μT experiment: MQ $p = 0.028$. C6 cells, 10 μT experiment: MF $p < 0.001$. C6 cells, 30 μT experiment: MF $p = 0.028$. The significances for the MF effects are given in the figure: *$p < 0.05$; ***$p < 0.001$.

statistically significant) at both magnetic flux densities, but did not show an increasing trend from 10 to 30 μT (figure 5*d*). The mitochondrial superoxide level was not statistically significantly affected in SH-SY5Y cells in this study (as described earlier), but the exposure–response pattern suggests a rising trend from 10 to 30 μT (figure 5*c*). However, the relative mitochondrial superoxide level observed in SH-SY5Y cells exposed to the 30 μT MF is not smaller than that observed in cells exposed at 100 μT in the previous study. In C6 cells, a significant MF effect on the mitochondrial superoxide level was observed at both MF strengths, and a rising trend from 10 to 30 μT can be seen (figure 5*e*).

rsif.royalsocietypublishing.org J. R. Soc. Interface 13: 20150995

Downloaded from https://royalsocietypublishing.org/ on 09 December 2021

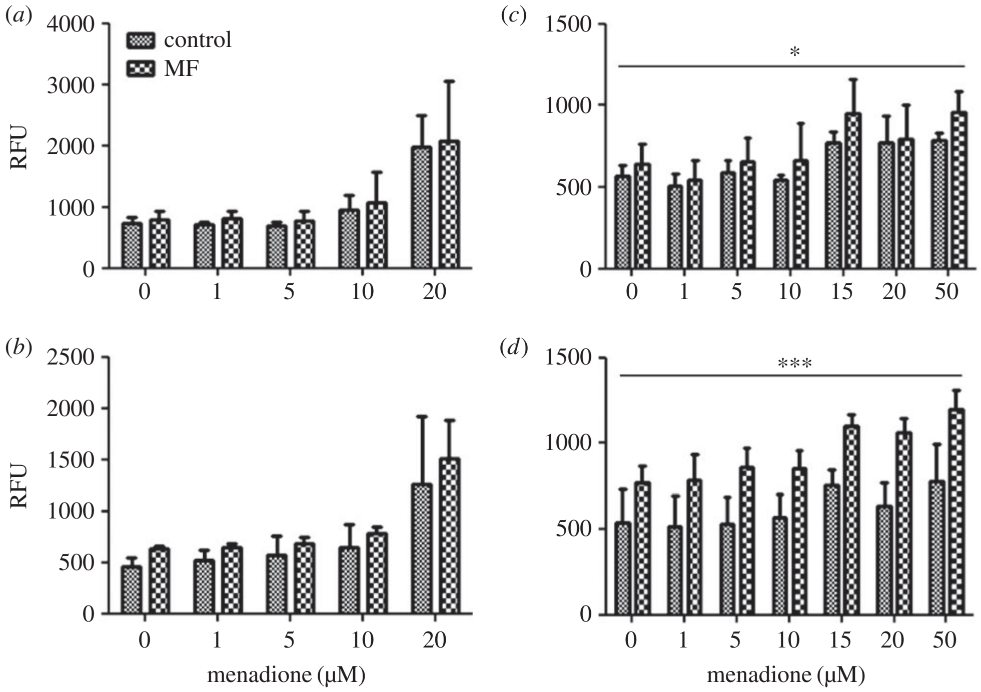

Figure 4. Mitochondrial superoxide level in human SH-SY5Y neuroblastoma (*a*,*b*) and rat C6 glioma (*c*,*d*) cells exposed to 50 Hz MFs at 10 μT (*a*,*c*) or 30 μT (*b*,*d*). The MF treatments were combined with different menadione (MQ) concentrations. The data shown are mean ± s.e.m. values. Three experiments were performed with three samples per group in each experiment. Factorial ANOVA was used for statistical testing; statistically significant effects for the two factors (MF and MQ) and their interactions are reported below. SH-SY5Y, 10 μT experiment: MQ $p = 0.001$. SH-SY5Y, 30 μT experiment: MQ $p = 0.001$. C6 cells, 10 μT experiment: MF $p = 0.05$; MQ $p = 0.002$. C6 cells, 30 μT experiment: MF $p < 0.001$; MQ $p = 0.007$. The significances for the MF effects are given in the figure: *$p < 0.05$; ***$p < 0.001$.

Table 3. Cytosolic and mitochondrial superoxide levels (in relative fluorescence units; mean ± s.e.m.) in human SH-SY5Y and rat C6 glioma cells immediately after a 24-h exposure to 50 Hz MFs at flux densities of 10 or 30 μT.

		10 μT	control	30 μT	control
cytosolic superoxide	SH-SY5Y	236 ± 10	215 ± 18	244 ± 35	272 ± 47
	C6	81 ± 8	74 ± 6	74 ± 7	63 ± 5
mitochondrial superoxide	SH-SY5Y	1099 ± 50	1007 ± 46	995 ± 34	949 ± 21
	C6	241 ± 13	250 ± 26	319 ± 33	268 ± 26

4. Discussion

In this study, two cell lines (human SH-SY5Y neuroblastoma and rat C6 glioma) were used to study their responses to weak 50 Hz MFs. Micronuclei as well as cytosolic and mitochondrial superoxide levels were measured, as these endpoints are relevant to the suspected adverse health effects of ELF MFs (cancer, Alzheimer's disease), and effects on these endpoints were found in our previous studies. The results confirmed our earlier finding of increased micronucleus frequency in SH-SY5Y cells exposed to a 50 Hz MF at 100 μT [14], and showed that this effect can also be observed at a lower magnetic flux density (30 μT). Although no statistically significant increase was observed at 10 μT, the data do not exclude a rising exposure–response relationship between 10 and 100 μT (figure 5). In contrast to the SH-SY5Y cells, no significant effect on micronuclei was detected in C6 cells.

The cytosolic superoxide level was not affected in SH-SY5Y cells, which is consistent with earlier findings showing no significant effect on cytosolic superoxide in the same cell line exposed at 100 μT [15]. However, cytosolic superoxide was affected in C6 cells by MF exposure at both 10 and 30 μT. The size of the effect was similar at both magnetic flux densities and no clear rising exposure–response relationship was observable (figure 5), but the data are inconclusive because of the wide confidence interval (large experimental variability) at 30 μT. Also the mitochondrial superoxide level was statistically significantly affected only in C6 cells. In this case, however, the SH-SY5Y data are consistent with a rising exposure–response relationship from 10 to 100 μT, when the statistically significant effect at 100 μT in our previous study is taken into account (figure 5). A rising trend with increasing magnetic flux density is also obvious in the C6 data.

Previous data on the exposure–response relationship below 100 μT is scarce. Mannerling *et al.* [17] reported a flat exposure–response relationship between 25 and 100 μT: the cellular superoxide anion radical level (determined with the nitro blue tetrazolium assay) and HSP70 protein level were increased in human chronic myelogenic leukemia cells exposed to 50 Hz MFs for 1 h, and the effect size was similar at 25, 50 and 100 μT. Flat exposure–response relationships have been previously reported for other biological responses to ELF MFs [27,28]. Mannerling *et al.* [17] measured immediate responses after an 1-h MF

Downloaded from https://royalsocietypublishing.org/ on 09 December 2021

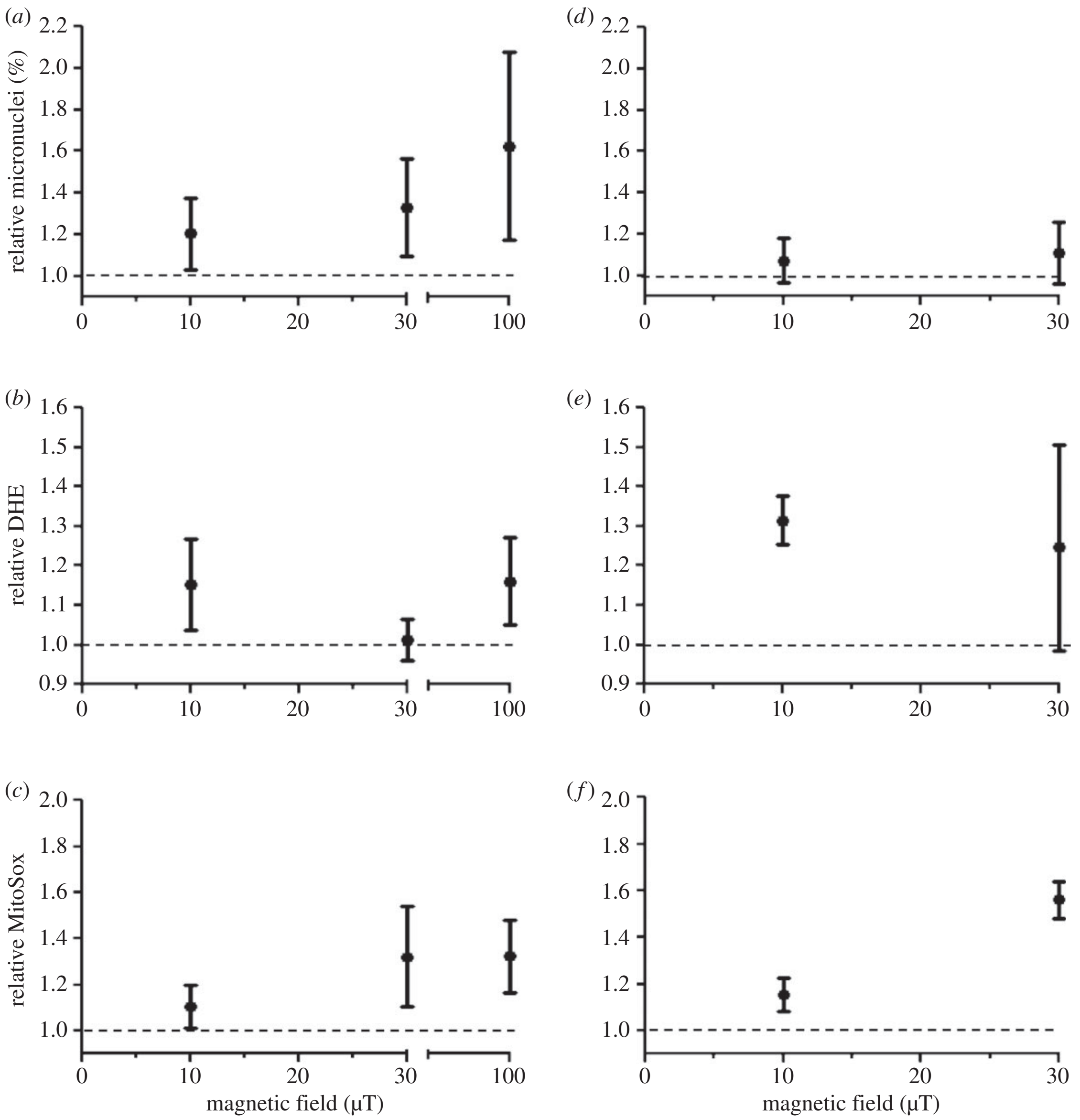

Figure 5. Exposure–response relationship for micronucleus formation (*a*,*d*), cytosolic superoxide level (*b*,*e*) and mitochondrial superoxide level (*c*,*f*) in human SH-SY5Y neuroblastoma (*a*–*c*) and rat C6 glioma (*d*–*f*) cells exposed to 50 Hz MFs. The data for 10 and 30 μT were pooled over all menadione (MQ) levels from the results of the present study and are given as relative values (value observed in the MF exposed sample divided by the value measured in the corresponding non-exposed sample), with 95% CIs. The data for 100 μT (SH-SY5Y cells) were similarly calculated from the data of the previous studies by Luukkonen *et al.* [14,15]. $N = 15$–21 (five to seven MQ levels, three replicate experiments).

exposure, whereas we measured responses after a 24 h exposure. The results are therefore not directly comparable, but it is nevertheless of interest that our results on the cytosolic superoxide level in C6 are consistent with those of Mannerling *et al.*, showing no dependency on MF intensity between 30 and 10 μT. In any case, we did not identify a threshold for the biological effects of 50 Hz MFs. If a threshold exists, it must be 10 μT or less. The type of sloping exposure–response relationship observed for micronuclei and mitochondrial superoxide indicates that effects with decreasing effect size might occur down to the level (0.4 μT) associated with human health effects in epidemiological studies. However, experimental demonstration of effects at such low field strengths will be challenging, as very large groups are required to show statistically significant effects, when the effect size is small.

The differences in the responses of the two cell lines are of interest. Micronucleus frequency was statistically significantly affected only in SH-SY5Y, while cytosolic and mitochondrial superoxide levels were significantly affected only in C6 cells. This shows that, although MF effects on both cell lines were observed, the responses may vary between cell lines. It should be noted that the differences between the cell lines may be only quantitative, reflecting different effect sizes in different cell lines: a slight increase in micronuclei (possibly just not statistically significant with this sample size) was seen in the MF groups also in C6 cells (figure 2*d*). Similarly, mitochondrial superoxide was slightly increased in the MF-treated groups in SH-SY5Y cells (figure 4*a*,*b*) and was statistically significantly increased at 100 μT in our previous study with SH-SY5Y cells [15].

In this study, superoxide levels were increased 3 h after the end of the 24 h MF exposure, while no significant effects were observed immediately after the MF treatment. This is consistent with our earlier findings [15]. It should be noted, however, that the statistical power to detect MF effects was lower immediately after exposure: only two exposure groups (MF and control) were compared in this case, whereas

Downloaded from https://royalsocietypublishing.org/ on 09 December 2021

all the different MQ exposure levels contributed to the MF-control comparison in measurements done 3 h after MF exposure (figures 3 and 4). Mannerling *et al*. [17] reported effects on the cellular superoxide level straight after a 1-h MF exposure, indicating that immediate responses to short-term MF treatment can also be observed. We have focused on 24 h exposures, because of our previous studies showing effects with this protocol and earlier results indicating that a relatively long-term exposure (12 or 24 h) is needed for MF effects on apoptosis [29]. This type of effect may require some time to develop; they probably represent slower (possibly adaptive) responses to long-term MF 'stress', and there is evidence that such effects may persist for a long time after the MF exposure [15,29]. The relationship between the immediate responses and the more slowly developing cellular changes are not known at present.

The administration of MQ as a cofactor was based on previous findings indicating that MF alters cellular responses to MQ [13,14]. Menadione is a semi-quinone that undergoes one-electron reduction in the mitochondrial respiratory chain, followed by one electron transfer to molecular oxygen, producing superoxide anions, essential oxygen radicals [30], which at physiologically relevant levels are rather non-reactive with DNA. Menadione probably causes non-oxidative DNA damage [31] via activation of Ca^{2+}-dependent nucleases [32]. Importantly, the results of the current study did not show any interactions between MF and MQ in their effects on cytosolic and mitochondrial superoxide levels, indicating that the effect of the MF on these variables is independent of the presence and dose of MQ (figures 3 and 4). This is consistent with the findings of Luukkonen *et al*. [15] and indicates that these endpoints reflect the biological responses to MF alone, which must occur during the 24 h MF treatment that alters responses to subsequent MQ exposure. As endpoints responding to MF alone have now been identified, further studies on the mechanisms of MF effects can be conducted without co-exposure with MQ.

The radical pair mechanism (RPM) is considered as a plausible mechanism for explaining the biological effects of weak MFs [7]. Based on quantum mechanics, the RPM predicts MF effects on the lifetime of radical pairs. This mechanism is theoretically well known [7,33–34] and has been experimentally demonstrated in chemical systems [35]. In biology, the RPM seems to be involved in animal navigation, through detection of the geomagnetic field in specific magneto-sensitive molecules [35]. It should be noted that the present experiments were done in the static geomagnetic field of about 30 µT. In its present form, the RPM predicts similar effects for a static field and a low-frequency (such as 50 Hz) time-varying MF, so effects of the 10-µT field in the presence of the stronger geomagnetic field are not easily explained by the RPM. The findings of this study therefore emphasize the need to develop theoretical understanding of possible specific effects of weak low-frequency alternating MFs, as discussed by Vanderstraeten & Gillis [9] and Barnes & Greenebaum [11].

In conclusion, induction of micronuclei and cytosolic and mitochondrial superoxide levels were investigated in two cell lines exposed to 50 Hz MFs for 24 h. Responses to MF exposure were observed in both cell lines, but the statistically significant effects observed (increased micronuclei versus increased superoxide levels) differed between the two cell lines. The results suggest that the threshold, if it exists, for biological responses to 50 Hz MFs is 10 µT or less. MF effects on cytosolic and mitochondrial superoxide levels were independent of co-exposure to MQ.

Authors' contributions. K.K., J.J., J.L. and J.N. conceived and designed the study. K.K. performed the experiments, analysed the data and drafted the manuscript. All authors contributed to manuscript revisions and approved the final version.

Competing interests. The authors declare that there are no competing interests.

Funding. This research was supported by strategic funding of the University of Eastern Finland (to J.N.) and the University's post doc funding (to K.K.).

Acknowledgement. The authors would like to express sincere thanks to Ms Hanne Säppi for her skilful technical assistance.

References

1. Grellier J, Ravazzani P, Cardis E. 2014 Potential health impacts of residential exposures to extremely low frequency magnetic fields in Europe. *Environ. Int*. **62**, 55–63. (doi:10.1016/j.envint.2013.09.017)
2. IARC. 2002 *Non-ionizing radiation, part 1: static and extremely low frequency (ELF) electric and magnetic fields. IARC Monographs on the Evaluation of Carcinogenic Risks to Humans*, vol. 80. Lyon, France: International Agency for Research on Cancer.
3. Hug K, Roosli M, Rapp R. 2006 Magnetic field exposure and neurodegenerative diseases-recent epidemiological studies. *Soz. Praventivmed*. **51**, 210–220. (doi:10.1097/00001648-200611001-01174)
4. García AM, Sisternas A, Hoyos SP. 2008 Occupational exposure to extremely low frequency electric and magnetic fields and Alzheimer disease: a meta-analysis. *Int. J. Epidemiol*. **37**, 329–340. (doi:10.1093/ije/dym295)
5. Vergara X, Kheifets L, Greenland S, Oksuzyan S, Cho YS, Mezei G. 2013 Occupational exposure to extremely low-frequency magnetic fields and neurodegenerative disease: a meta-analysis. *J. Occup. Environ. Med*. **55**, 135–146. (doi:10.1097/JOM.0b013e31827f37f8)
6. Ahlbom IC, Cardis E, Green A, Linet M, Savitz D, Swerdlow A, ICNIRP (International Commission for Non Ionizing Radiation Protection). 2001 Standing committee on epidemiology: review of the epidemiologic literature on EMF and health. *Environ. Health. Perspect*. **109**, 911–933. (doi:10.2307/3454653)
7. World Health Organization (WHO). 2007 Extremely low frequency fields. Geneva: World Health Organization. *Environ. Health Criteria* **238**, 347–355.
8. Lagroye I, Percherancier Y, Juutilainen J, De Gannes FP, Veyret B. 2011 ELF magnetic fields: animal studies, mechanisms of action. *Prog. Biophys. Mol. Biol*. **107**, 369–373. (doi:10.1016/j.pbiomolbio.2011.09.003)
9. Vanderstraeten J, Gillis P. 2010 Theoretical evaluation of magnetoreception of power-frequency fields. *Bioelectromagnetics* **31**, 371–379. (doi:10.1002/bem.20568)
10. Maeda KA *et al*. 2012 Magnetically sensitive light-induced reactions in cryptochrome are consistent with its proposed role as a magnetoreceptor. *Proc. Natl Acad. Sci. USA* **109**, 4774–4779. (doi:10.1073/pnas.1118959109)
11. Barnes FS, Greenebaum B. 2015 The effects of weak magnetic fields on radical pairs. *Bioelectromagnetics* **36**, 45–54. (doi:10.1002/bem.21883)
12. Juutilainen J, Kumlin T, Naarala J. 2006 Do extremely low frequency magnetic fields enhance the effects of environmental carcinogens? A meta-analysis of experimental studies.

Int. J. Radiat. Biol. **82**, 1–12. (doi:10.1080/09553000600577839).
13. Markkanen A, Juutilainen J, Naarala J. 2008 Pre-exposure to 50 Hz magnetic fields modifies menadione-induced DNA damage response in murine L929 cells. *Int. J. Radiat. Biol*. **84**, 742–751. (doi:10.1080/09553000802360836).
14. Luukkonen J, Liimatainen A, Höytö A, Juutilainen J, Naarala J. 2011 Pre-exposure to 50 Hz magnetic fields modifies menadione-induced genotoxic effects in Human SH-SY5Y neuroblastoma cells. *PLoS ONE* **6**, e18021. (doi:0.1371/journal.pone.0018021)
15. Luukkonen J, Liimatainen A, Juutilainen J, Naarala J. 2014 Induction of genomic instability, oxidative processes, and mitochondrial activity by 50 Hz magnetic fields in human SH-SY5Y neuroblastoma cells. *Mutat. Res./Fundam. Mol. Mech. Mutagen*. **760**, 33–41. (doi:10.1016/j.mrfmmm.2013.12.002)
16. Falone S, Mirabilio A, Carbone MC, Zimmitti V, Di Loreto S, Mariggio MA, Mancinelli R, Di Ilio C, Amicarelli F. 2008 Chronic exposure to 50 Hz magnetic fields causes a significant weakening of antioxidant defence systems in aged rat brain. *Int. J. Biochem. Cell Biol*. **40**, 2762–2770. (doi:10.1016/j.biocel.2008.05.022)
17. Mannerling AC, Simko M, Mild KH, Mattsson MO. 2010 Effects of 50-Hz magnetic field exposure on superoxide radical anion formation and HSP70 induction in human K562 cells. *Radiat. Environ. Biophys*. **49**, 731–741. (doi:10.1007/s00411-010-0306-0)
18. Wolf FI, Torsello A, Tedesco B, Fasanella S, Boninsegna A, D'Ascenzo M, Grassi C, Azzena GB, Cittadini A. 2005 50-Hz extremely low frequency electromagnetic fields enhance cell proliferation and DNA damage: possible involvement of a redox mechanism. *Biochim. Biophys. Acta Mol. Cell Res*. **1-2**, 120–129. (doi:10.1016/j.bbamcr.2004.09.005)
19. Coppedè F, Migliore L. 2010 Evidence linking genetics, environment, and epigenetics to impaired DNA repair in Alzheimer's disease. *J. Alzheimers Dis*. **20**, 953–966. (doi:10.3233/JAD-2010-1415)
20. Finkel T. 2011 Signal transduction by reactive oxygen species. *J. Cell Biol*. **194**, 7–15. (doi:10.1083/jcb.201102095)
21. Waris G, Ahsan H. 2006 Reactive oxygen species: role in the development of cancer and various chronic conditions. *J. Carcinog*. **5**, 14. (doi:10.1186/1477-3163-5-14)
22. Sorce S, Krause KH. 2009 NOX enzymes in the central nervous system: from signaling to disease. *Antioxid. Redox Signal*. **11**, 2481–2504. (doi:10.1089/ARS.2009.2578)
23. Loukogeorgakis SP *et al*. 2010 Role of NADPH oxidase in endothelial ischemia/reperfusion injury in humans. *Circulation* **121**, 2310–2316. (doi:10.1161/CIRCULATIONAHA.108.814731)
24. Bryce SM, Bemis JC, Avlasevich SL, Dertinger SD. 2007 *In vitro* micronucleus assay scored by flow cytometry provides a comprehensive evaluation of cytogenetic damage and cytotoxicity. *Mutat. Res*. **630**, 78–91. (doi:10.1016/j.mrgentox.2007.03.002)
25. Debowska K *et al*. 2015 Toward selective detection of reactive oxygen and nitrogen species with the use of fluorogenic probes—limitations, progress, and perspectives. *Pharmacol. Rep*. **67**, 756–764. (doi:10.1016/j.pharep.2015.03.016)
26. Naarala JT, Loikkanen JJ, Ruotsalainen MH, Savolainen KM. 1995 Lead amplifies glutamate-induced oxidative stress. *Free Radic. Biol. Med*. **19**, 689–693. (doi:10.1016/0891-5849(95)00067-8)
27. Juutilainen J, Läära E, Saali K. 1987 Relationship between field strength and abnormal development in chick embryos exposed to 50 Hz magnetic fields. *Int. J. Radiat. Biol. Relat. Stud. Phys. Chem Med*. **52**, 787–793. (doi:10.1080/09553008714552301)
28. Mullins JM, Penafiel LM, Juutilainen J, Litovitz TA. 1999 Dose–response of electromagnetic field-induced ornithine decarboxylase activity. *Bioelectrochem. Bioenerg*. **48**, 193–199. (doi:10.1016/S0302-4598(98)00229-3)
29. Robinson KM, Janes MS, Pehar M, Monette JS, Ross MF, Hagen TM, Murphy MP, Beckman JS. 2006 Selective fluorescent imaging of superoxide *in vivo* using ethidium-based probes. *Proc. Natl Acad. Sci. USA* **103**, 15 038–15 043. (doi:10.1073/pnas.0601945103)
30. Halliwell B, Gutteridge J. 2007 *Free radicals in biology and medicine*, 4th edn, pp. 46–50, 188–190, 453–454. New York, NY: Oxford University Press.
31. Fischer-Nielsen A, Corcoran GB, Poulsen HE, Kamendulis LM, Loft S. 1995 Menadione-induced DNA fragmentation without 8-oxo-29-deoxyguanosine formation in isolated rat hepatocytes. *Biochem. Pharmacol*. **49**, 1469–1474. (doi:10.1016/0006-2952(94)00525-Q)
32. McConkey DJ, Hartzell P, Nicotera P, Wyllie AH, Orrenius S. 1988 Stimulation of endogenous endonuclease activity in hepatocytes exposed to oxidative stress. *Toxicol. Lett*. **42**, 123–130. (doi:10.1016/0378-4274(88)90069-0)
33. Brocklehurst B, McLauchlan KA. 1996 Free radical mechanism for the effects of environmental electromagnetic fields on biological systems. *Int. J. Radiat. Biol*. **69**, 3–24. (doi:10.1080/095530096146147)
34. Timmel CR, Till U, Brocklehurst B, McLauchlan KA, Hore PJ. 1998 Effects of weak magnetic fields on free radical recombination reactions. *Mol. Phys*. **95**, 71–89. (doi:10.1080/00268979809483134)
35. Eveson RW, Timmel CR, Brocklehurst B, Hore PJ, McLauchlan KA. 2000 The effects of weak magnetic fields on radical recombination reactions in micelles. *Int. J. Radiat. Biol*. **76**, 1509–1522. (doi:10.1080/09553000050176270)

Downloaded from https://royalsocietypublishing.org/ on 09 December 2021
