# Supplementary material for: Magnetic Fields and Cancer: Epidemiology, Cellular Biology, and Theranostics
Source: Int J Mol Sci. 2022 Jan 25;23(3):1339. doi: 10.3390/ijms23031339 (PMC8835851; doi:10.3390/ijms23031339)
Supplement: Supplementary file 1 [file ijms-23-01339-s001.zip › Supplementary Data Set S1/MF and Cancer.Data/PDF/4127301238/nch030.pdf]

Radiation Protection Dosimetry (2004), Vol. 108, No. 3, pp. 227–236
DOI: 10.1093/rpd/nch030

ASSESSING OCCUPATIONAL AND DOMESTIC ELF MAGNETIC FIELD EXPOSURE IN THE UK ADULT BRAIN TUMOUR STUDY: RESULTS OF A FEASIBILITY STUDY

Martie van Tongeren[1,2,*], Terry Mee[3], Pamela Whatmough[4], Lisa Broad[2], Myron Maslanyj[3], Stuart Allen[3], Ken Muir[4] and Patricia McKinney[5,6]
[1]Centre for Occupational and Environmental Health, University of Manchester, Oxford Road, Humanities Building, Manchester M13 9PL, UK
[2]Institute of Occupational Health, University of Birmingham, Edgbaston, Birmingham B15 2TT, UK
[3]National Radiological Protection Board (NRPB), Chilton, Didcot, Oxon OX11 0RQ, UK
[4]Department of Epidemiology and Public Health, University of Nottingham, Nottingham NG7 2UH, UK
[5]Information and Statistics Division of the Common Services Agency, National Health Service Scotland, Trinity Park House, Edinburgh EH5 3SQ, Scotland
[6]Unit of Epidemiology and Health Services Research, Leeds University, Leeds LS2 9JT, UK

Downloaded from https://academic.oup.com/rpd/article/108/3/227/1593973 by University of Torino user on 19 November 2021

Received October 14 2003, amended December 22 2003, accepted December 31 2003

The feasibility of measuring exposure to extremely low frequency magnetic fields (ELF MF) in the UK Adult Brain Tumour Study (UKABTS) was examined. During the study, 81 individuals and 30 companies were approached with 79 individuals and 25 companies agreeing to participate. Exposure data were collected using EMDEX II dosemeters worn by the participants for 3–4 consecutive days. Data were collected over a total of 321 d, including non-occupational periods. The results showed occupational exposure to be the main determinant of overall exposure. Moderate to strong correlations were found between arithmetic mean exposure and all other metrics with the possible exception of maximum exposure. Significant differences in exposure were found between job categories with large variability in certain categories. Highest average exposures were found for security officers (arithmetic mean, AM: 0.78 μT), secretaries (AM: 0.48 μT) and dentists (AM: 0.42 μT). Welding and working near high-voltage power lines were associated with elevated exposure. In summary, acceptably precise measures of ELF MF exposure are feasible at relatively moderate cost. The results were used to develop a protocol for data collection from subjects in the UKABTS.

INTRODUCTION

Adult brain tumours present a challenging detriment to modern society. Among the most lethal of cancers, their reported incidence has been rising steadily around the world[(1–5)]. Although the increase is more prevalent in the elderly, brain tumours do impinge perceptibly on younger populations, accounting for 10.7% of all registered cancer deaths in Scotland in those less than 45 y of age[(6)]. Much of the increasing incidence results from improving detection and diagnosis[(7,8)]. Yet the degree to which increased exposure to carcinogens in the environment or other factors are implicated remains unclear[(7,9–12)].

In recent decades, epidemiological studies have explored the involvement of a wide range of environmental agents in the aetiology of adult brain cancer[(13)]. Among the factors postulated to influence the onset of brain cancer is exposure to extremely low frequency (ELF) electromagnetic fields (EMF), in particular magnetic fields (MF). The early epidemiological studies that examined this association tended to rely on job title, often derived from an uncorroborated death certificate, with certain 'electrical' occupations taken broadly to indicate high exposure. With the advent of commercially available dosemeters, more recent studies have tended to assign exposures using job exposure matrices, classifying jobs and relating these to summary measures from a representative group of workers. These exposure matrices have a number of inherent limitations, in that only a proportion of jobs can be classified by measurements. In addition, Rodvall *et al.*[(14)] showed that occupation itself is not necessarily a good predictor of EMF exposure with high exposure found in various jobs in non-electrical industries, whilst Harrington *et al.*[(15)] found that EMF exposure can vary markedly even in narrowly defined occupational groups.

In 2001, the UK Adult Brain Tumour Study (UKABTS) was set up to investigate a number of possible causes of brain tumours. The UKABTS is part of an international case–control study of adult brain tumours (the INTERPHONE study), co-ordinated at the International Agency for Research on Cancer (IARC), i.e. investigating the association

*Corresponding author: martie.van-tongeren@man.ac.uk

Radiation Protection Dosimetry Vol. 108 No. 3 © Oxford University Press 2004; all rights reserved

between mobile phone use and incidence of brain cancers[16]. In addition to mobile phone use, the UKABTS will also investigate the role of occupational exposure to solvents and pesticides, and occupational EMF exposure, including ELF MFs in the aetiology of brain tumours. Information on occupation, tasks and use of equipment, will be gathered during interviews using Computer Assisted Personal Interview (CAPI) software. The INTERPHONE study is developing an expert exposure assessment method for determining occupational EMF exposure through the interpretation of information from the CAPI.

The World Health Organisation (WHO) international EMF project recommended that in order to aid future research EMF studies should gather as much information relevant to alternative metrics as possible[17]. Direct measures of exposure can supplement existing exposure matrices and increase the precision of exposure estimates. In addition alternative exposure metrics can be investigated. Given sufficient statistical power such measures can provide alternative analyses of possible disease association. Therefore, this study was carried out to determine whether collecting direct measures of ELF MF exposure is feasible in the context of the UKABTS[18].

METHODS

Study participants

The UKABTS is operating from four study centres: Scotland, Trent, West Midlands and West Yorkshire. Subjects that were enrolled in the Trent region during the period June 2001 and February 2002 were assessed as to their competence (mainly based on their health status) to participate in the feasibility study. To maximise the use of time and instruments preference was given to subjects in the Nottingham area, although subjects in the Leicester and Sheffield areas were also included in order to broaden the diversity of jobs. Initially, competent subjects were asked during the main CAPI interview whether they would be prepared to participate. Individuals who agreed were then contacted by phone in order to seek permission to obtain measurements. Individuals were asked, where appropriate, for permission to contact employers for the purpose of placing meters with company staff. With permission employers were approached by a member of the research team. The employers were provided with details about the study and a letter of endorsement from the Health and Safety Executive. When a subject was no longer in post, the employer was asked to select an appropriate proxy employee, based on subject name and a job description. In addition to the UKABTS subjects, a number of employees from the University of Nottingham with potentially higher exposures were selected for investigation.

Data collection

Emdex II magnetic field meters (Enertech Consultants Ltd, California, USA) were used to record data. The meters were programmed to record the resultant magnetic flux density in both broadband (40–800 Hz) and harmonic (100–800 Hz) frequency ranges at a sampling interval of 5 s. The meter has a dynamic range of 0.01–300 μT with a resolution of 0.01 μT in the frequency range 40–800 Hz, and an overall accuracy of 10% in the dynamic range 0.01–10 μT (±0.005 μT, accounting for the resolution in the low range). The meter used in the study had an enhanced memory capability enabling the collection of data with the above settings for about 6 d.

The meters were worn at the waist, and left at the bedside, at bed height, overnight. During activities where it was impracticable to wear a meter, such as sport, bathing etc., the subjects were asked to place the meter as close as was practicable at waist height. Participants completed a short diary identifying periods at work, at home or travelling to and from work and recording when the instrument was not worn. A short questionnaire was also completed on a daily basis to identify potentially important MF sources and high exposure tasks.

All meters were calibrated using a Helmholtz coil facility that has an uncertainty of 2.5%, traceable to national standards. Before and after each measurement, meters were checked to be functioning correctly by means of a check source generating a nominal field of 5 μT. Exposure and check source data were downloaded after each assessment using EMCALC software. The Emdex II meter attaches a 'bad data' flag to each measurement where either the reading is beyond the dynamic range of the instrument or the measured field is changing very rapidly. Individual records were examined as part of a quality control process and instances where the data looked suspect were followed up with the aim of identifying sources and, where possible, repeating the measurements to reflect more typical circumstances.

Statistical analysis

For each day of the measurement period, data were divided into specific periods to cover work, travel to and from work, and domestic and other activities. The following metrics were computed in the broadband range: time weighted average (TWA), standard deviation (SD), geometric mean (GM), per cent of time above 0.2 μT (% >0.2 μT), intermittency (ITM) and maximum value (MAX). The TWA in

Downloaded from https://academic.oup.com/rpd/article/108/3/227/1593973 by University of Torino user on 19 November 2021

harmonic range (H-TWA) was also computed. Intermittency is the mean absolute difference between adjacent readings, and is a measure of field variation. The metrics were calculated separately for each day and each activity period using the 5 s interval data.

The distributions of all daily exposure metrics were skewed toward low values and, apart from the % >0.2 μT metric, data were log transformed prior to statistical analyses. For % >0.2 μT, a transformation according to $[(x + 0.001)^{0.25}]$ was applied to give an approximate normal distribution.

Pearson correlation coefficients were calculated to determine if occupational exposure and total exposure were associated, and to examine the relationship between the various exposure metrics calculated for the occupational exposure period.

Random effects models were used to estimate the variance components for the whole occupational dataset, and the components were subsequently used to estimate the extent of the theoretical attenuation of a presumed exposure–response relationship, using[19]:

$$\mathrm{OR}_{\mathrm{obs}} = \mathrm{OR}_{\mathrm{true}}^{\rho_{\mathrm{TA}}^2} \quad (1)$$

where $\mathrm{OR}_{\mathrm{obs}}$ is the observed odds ratio for exposed versus not exposed, and $\mathrm{OR}_{\mathrm{true}}$ is the real underlying OR. ρ_{TA} is the validity coefficient:

$$\rho_{\mathrm{TA}} = \sqrt{\frac{\sigma_{\mathrm{bw}}^2}{\sigma_{\mathrm{bw}}^2 + (\sigma_{\mathrm{ww}}^2/k)}} \quad (2)$$

where σ_{bw}^2 is the estimated between-worker variance and σ_{ww}^2 is the estimated within-worker variance component of exposure and k is the number of measurements on each individual. This analysis assumes that exposure data is available for each individual in the study. The within-worker variance is a measure of the precision of the exposure estimate, whilst the between-worker variance is a measure of the range of individual mean exposure levels in the study. In this case, a larger between-worker variance is beneficial as this indicates a greater contrast in exposure between individuals in the study and hence will improve the ability to accurately determine the true exposure–response association.

Next, all individuals were grouped into job categories, based on similarity of job titles, although these were not necessarily from the same work place. Random effects models were used to estimate the variance components for each job category. In this case, the estimates of the between-worker variance provides information on homogeneity of exposure within the job categories. Large between-worker variation indicates that workers are grouped inappropriately. This can result in significant misclassification of exposure when exposure estimates based on data from the whole group are applied to each individual within the group. Also, the exercise provides some information on the likelihood of misclassification of exposure when using proxies in place of true study subjects. Finally, mixed effects models were used to estimate the effects of job and task on occupational exposure, at the same time estimating the pooled variance components (within or day-to-day variance and between individual variance).

All analyses were carried out using SAS for Windows, Release 8.02 (SAS Institute Inc., Cary, NC, USA).

RESULTS

Of a total of 81 individuals approached, 79 (98%) agreed to participate, and 25 of the 30 (83%) companies approached consented to on-site measurements. Of the five company refusals, four related to requests for proxy employees, whilst the other refusal was because of safety considerations. Exposure data were available from 321 days, 229 of which included occupational measurements ($n = 62$ individuals), 299 included measurements during domestic or other activity measurements ($n = 75$ individuals), and 200 included periods of travel to and from work ($n = 57$ individuals).

Table 1 shows the summary statistics for the daily TWA exposure metric for occupational, travel and domestic exposure levels for all the pilot study participants. In addition to the arithmetic mean (AM) and minimum and maximum values, the GM and the geometric SD (GSD) are given. The highest levels of exposure were observed during occupational periods. The mean occupational TWA exposure was 0.2 μT, compared with 0.13 μT for travel periods and 0.08 μT for domestic periods (including other non-occupational and non-travel activities). Exposure levels for the selected occupations were significantly higher than for occupational TWA exposure for the UKABTS participants ($p < 0.05$). There were no statistically significant differences between the populations in exposure during travel and domestic periods. The average daily exposure (taking all measurements during all periods together) was 0.13 μT (GM = 0.09 μT).

The correlation between exposure during occupational periods and the average exposure over a whole day was greater than 0.8 for the TWA, SD, % >0.2 μT, ITM and MAX metrics (Table 2). The correlation coefficients for GM and H-TWA were 0.73 and 0.55, respectively. Correlations between the exposure at home and total daily average exposure were generally much lower (data not shown), with the exception of the metric GM and % >0.2 μT, whilst the correlation between exposure during travel periods and daily average exposure was poor (<0.35). The results indicate that exposure at work is the main determinant of total exposure.

Downloaded from https://academic.oup.com/rpd/article/108/3/227/1593973 by University of Torino user on 19 November 2021

Table 1. Summary statistics for daily TWA exposure estimates.

	Population*	N	AM (μT)	GM (μT)	GSD	Min. (μT)	Max. (μT)
Domestic and other	Full study	299	0.08	0.05	2.39	0.00	1.61
	UKABTS	201	0.09	0.06	2.54	0.01	1.61
	Interest	98	0.06	0.05	2.08	0.00	0.27
Travel	Full study	200	0.13	0.10	1.93	0.01	1.58
	UKABTS	107	0.12	0.10	1.97	0.01	0.38
	Interest	93	0.14	0.10	1.89	0.03	1.58
Occupational	Full study	229	0.22	0.11	3.04	0.01	3.05
	UKABTS	131	0.20	0.09	3.15	0.01	3.05
	Interest	98	0.26	0.16	2.64	0.02	1.30
Total	Full study	321	0.13	0.09	2.38	0.01	1.84
	UKABTS	219	0.13	0.08	2.44	0.01	1.84
	Interest	102	0.14	0.10	2.18	0.02	1.09

*The analyses were carried out for the whole group (full study), and separate for study subjects of the UKABTS and for individuals selected for interest readings, who do not participate in the case–control study (Interest).
N = number of daily measurements; AM = arithmetic mean of daily means; GM = geometric mean of daily means; Min. = minimum result of daily means; Max. = maximum result of daily means.

Table 2. Pearson's correlation coefficients for total and occupational exposure for the various exposure metrics using transformed daily exposure metrics.

TWA	SD	GM	% >0.2 μT	ITM	MAX	H-TWA
0.83	0.83	0.73	0.81	0.90	0.84	0.55

TWA: time-weighted average of broadband measures across the work shift; GM: geometric mean of broadband measures across the work shift; SD: standard deviation of broadband measures across the work shift; ITM: intermittency of broadband measures across the work shift; MAX: maximum broadband measure across the work shift; H-TWA: Time weighted average of measurements in harmonic frequency range across the work shift; % >0.2 μT: Percent of time above 0.2 μT across the work shift.

Table 3 gives the correlation matrix of daily occupational exposure metrics collected from 62 individuals on 229 d. TWA exposure was strongly correlated with exposure expressed as GM, H-TWA and % >0.2 μT. Correlation was also strong between SD and ITM and MAX. Weak correlations were found between MAX and TWA, GM, H-TWA and % >0.2 μT.

Figure 1 shows a scatter plot of the broadband (TWA) and harmonic (H-TWA) exposure metrics. There appear to be two H-TWA populations, one with a fairly constant relationship between the two metrics and one with a relatively stable harmonic content, irrespective of the TWA level. The first group, with harmonic exposure less than half the broadband level, includes a secretary, two dentists, a dental nurse, a forklift driver, a mineral resources engineer and a forecourt attendant (working adjacent to a bank of fridges and freezers). In the second group is a security officer working close to an overhead HV line, a plastics-extrusion operator, two engineer/machinists and an electrician. A continuing programme of harmonic measurements will enable further exploration of such groups and may provide more discrimination.

Table 3. Correlation matrix for daily occupation exposure using log-transformed daily exposure metrics.

Exposure metric	TWA	GM	SD	ITM	MAX	H-TWA
GM	0.92					
SD	0.79	0.57				
ITM	0.70	0.55	0.81			
MAX	0.51	0.33	0.85	0.74		
H-TWA	0.86	0.79	0.66	0.62	0.40	
% >0.2 μT	0.83	0.71	0.78	0.66	0.54	0.71

TWA: time-weighted average of broadband measures across the work shift; GM: geometric mean of broadband measures across the work shift; SD: standard deviation of broadband measures across the work shift; ITM: intermittency of broadband measures across the work shift; MAX: maximum broadband measure across the work shift; H-TWA: Time weighted average of measurements in harmonic frequency range across the work shift; % >0.2 μT: Percent of time above 0.2 μT across the work shift.

The potential attenuation of a hypothetical exposure response association due to random measurement error in occupational exposure (day-to-day variability) was estimated for a number of metrics based on the estimated variance components and assuming that exposure data were available for all the individuals in the study. Table 4 gives the within- and between-worker variance component for occupational

Downloaded from https://academic.oup.com/rpd/article/108/3/227/1593973 by University of Torino user on 19 November 2021

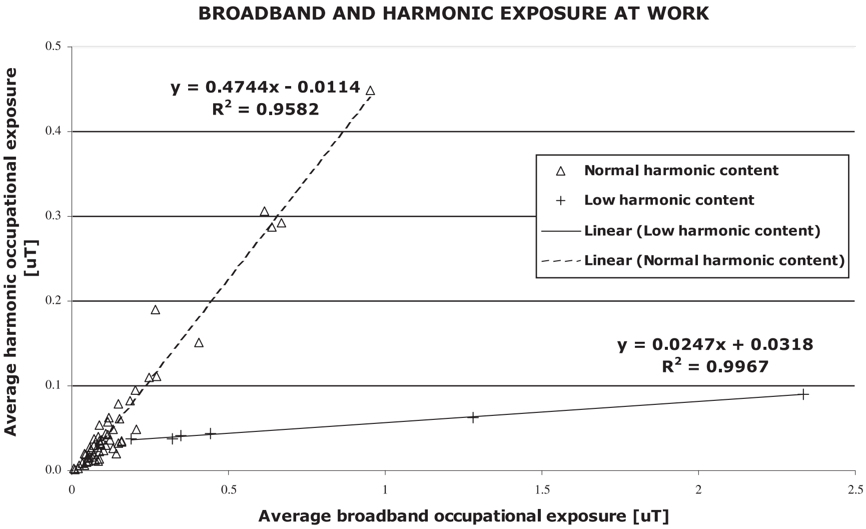

Figure 1. Relationship between occupational broadband and harmonic exposure.

Table 4. Within- and between-subject variance components of occupational ELF-MF exposure with validation coefficient and expected observed odds ratios (assuming a real OR of 1.5).

	$_bS^2$	$_wS^2$	1 d		3 d		5 d		10 d	
			ρ_{TA}	OR_{obs}	ρ_{TA}	OR_{obs}	ρ_{TA}	OR_{obs}	ρ_{TA}	OR_{obs}
TWA	0.92	0.26	0.88	1.37	0.96	1.45	0.97	1.47	0.99	1.48
H-TWA	1.02	0.40	0.85	1.34	0.94	1.43	0.96	1.46	0.98	1.48
ITM	0.87	0.24	0.89	1.38	0.96	1.45	0.97	1.47	0.99	1.48
MAX	0.66	0.87	0.66	1.19	0.83	1.33	0.89	1.38	0.94	1.43

$_bS^2$: between-individual variance component of exposure; $_wS^2$: within-individual variance component of exposure; ρ_{TA} = validity coefficient; OR_{obs} = expected observed odds ratio.

exposures and the predicted observed odds ratios (for each increase of 1 μT) with a presumed real odds ratio of 1.5. The between-worker variance ranged from 0.66 for MAX to 1.02 for H-TWA, with the within-worker variance ranging from 0.24 for ITM to 0.87 for MAX. When only measurements are available for 1 d, the ORs are reduced from 1.5 to 1.37 for TWA, 1.34 for H-TWA, 1.38 for ITM and 1.19 for MAX, respectively. When measurements are available for 3 d, the expected observed ORs are 1.45, 1.43, 1.45 and 1.33, respectively. Increasing the number of days only leads to marginally improvement in observed ORs for TWA, H-TWA and ITM.

These estimates were based on measurements that were taken on consecutive days. However, it is likely that the real day-to-day variance is underestimated in such data. For nine participants, repeat exposure data were available after 2–7 months. For these participants, the day-to-day variance was increased when the repeat data was included, compared to that when only data from the first visit were used (data not shown). However, the differences were relatively small, and had little impact on the predicted observed OR.

Next, the exposure data was summarised by job category (Table 5). There were statistically significant differences in exposure between the job categories, with relatively high exposures found for the security officers (AM TWA = 0.78 μT), secretaries (AM TWA = 0.48 μT), dentists (AM TWA = 0.42 μT) and dental nurses (AM TWA = 0.30 μT). High levels in the harmonic range were found for secretaries (AM = 0.21 μT), dentists (AM = 0.18 μT) and dental nurses (AM = 0.14 μT). For the ITM exposure metric, highest levels were found for dentists (AM = 0.06 μT) and security officers (AM = 0.05 μT), whilst for the MAX metric the highest levels were found for fork lift truck drivers (AM = 17.8 μT), printers (AM = 8.4 μT) and dental nurses (AM = 6.9 μT).

Downloaded from https://academic.oup.com/rpd/article/108/3/227/1593973 by University of Torino user on 19 November 2021

Table 5. Summary statistics for the various occupational exposure metrics by job title.

Job title	Id	Days	TWA				H-TWA				ITM				MAX			
			AM	GM	$_{tot}$GSD	$_{bw}$GSD	AM	GM	$_{tot}$GSD	$_{bw}$GSD	AM	GM	$_{tot}$GSD	$_{bw}$GSD	AM	GM	$_{tot}$GSD	$_{bw}$GSD
Manager	12	41	0.07	0.05	2.34	1.99	0.03	0.01	3.58	2.66	0.01	0.01	2.02	1.6	1.39	0.88	2.77	1.00
Engineer	9	34	0.20	0.15	2.09	1.59	0.05	0.04	2.04	1.65	0.03	0.02	2.24	1.91	4.45	2.71	2.83	1.92
Radiographer	4	14	0.10	0.09	1.69	1.14	0.03	0.02	1.82	1.58	0.02	0.01	2.03	1.92	2.60	1.78	2.59	2.25
Security officer	5	18	0.78	0.27	6.08	5.98	0.05	0.04	1.83	1.72	0.05	0.03	3.45	3.33	5.84	3.13	3.02	2.39
Shop assistant	4	14	0.16	0.13	2.01	1.97	0.07	0.05	2.93	2.88	0.04	0.03	2.84	2.76	2.16	1.14	2.82	1.77
Dentist	3	16	0.42	0.29	2.89	2.59	0.18	0.10	3.82	3.07	0.06	0.03	4.60	4.32	4.60	2.88	3.30	1.95
Warp knitter	3	9	0.10	0.09	1.23	1.15	0.04	0.04	1.41	1.28	0.04	0.04	1.20	1.00	2.98	2.73	1.62	1.51
Dental nurse	2	11	0.30	0.24	2.07	1.72	0.14	0.10	2.37	1.90	0.04	0.03	4.15	3.99	6.85	3.83	4.63	3.39
Fork lift driver	2	9	0.15	0.12	2.52	2.31	0.09	0.05	5.87	5.33	0.11	0.08	3.84	3.39	17.80	8.81	2.79	1.60
Farmer	2	8	0.05	0.03	5.64	5.45	0.01	0.00	4.13	3.72	0.01	0.01	4.41	3.92	3.22	1.24	6.07	2.80
Lecturer/scientist	3	9	0.08	0.07	1.61	1.33	0.03	0.03	2.10	1.79	0.01	0.01	1.52	1.36	1.10	0.94	1.90	1.00
Secretary	3	12	0.48	0.23	3.97	1.85	0.21	0.08	5.16	2.50	0.02	0.02	2.30	1.00	2.61	1.74	3.05	1.00
Printer	2	10	0.15	0.14	1.56	1.40	0.03	0.02	2.48	1.81	0.02	0.01	1.84	1.00	8.44	3.02	8.83	6.18
Other	8	24	0.12	0.08	2.89	2.73	0.02	0.02	2.84	2.66	0.04	0.02	3.04	2.60	4.22	1.86	4.45	3.11

Id: number of workers with measurements; Days: number of days with measurements; AM: arithmetic mean of daily exposure metrics; GM: geometric mean of daily exposure metrics; $_{tot}$GSD: total geometric standard deviation of daily exposure metrics; $_{bw}$GSD: between individual geometric standard deviation of daily exposure metrics.

Downloaded from https://academic.oup.com/rpd/article/108/3/227/1593973 by University of Torino user on 19 November 2021

Table 5 also provides the estimates of total GSD and the between-subject GSD. The variance in the exposure data was extremely high for some job titles, e.g. in the case of TWA exposure, the GSD for security officers, was 6.1 and for farmers the GSD was 5.6. Generally, the variance was due to the large differences in individual mean exposure within a job category. For security officers, the between-subject GSD was 6.0 and for farmers this was 5.5. Only a few job titles could be considered homogeneously exposed, i.e. all subjects within the group had similar mean exposures (between-subject GSD < 1.2). For TWA exposure, low between-subject GSDs were found for radiographers and warp knitters, whilst for H-TWA none of the job categories were homogeneously exposed. For ITM, homogeneous exposure was found for warp knitters, secretaries and printers, whilst for MAX, the groups of managers, lecturers/scientists and secretaries were homogenously exposed.

Participants reported occupational activities involving electrical equipment or tools on 199 out of the 229 data-logging days. The majority involved working with computers and working with tools or equipment operated by electrical motors. Table 6 provides occupational ELF MF exposure levels for those days when the specified tasks were carried out. When no specified tasks or activities were identified, the AM of TWA exposure was 0.05 μT with a GM of 0.03 μT (GSD 2.41). The TWA, ITM and MAX exposures were significantly increased on those days when at least one specified activity was carried out (TWA: GM = 0.13 μT; GSD 2.80; ITM: GM = 0.02 μT; GSD 2.92 MAX: GM = 2.23 μT; GSD 3.27). Welding was associated with statistically significantly elevated exposure for all exposure metrics, whilst working near high voltage lines was associated with statistically significantly increased levels for TWA, ITM and MAX. Finally, working with electrical motors and with electrical transport equipment was associated with statistically significantly increased levels for ITM and MAX, with working with high-current wires being associated with elevated levels of MAX. Working in a powerstation was also associated with elevated exposure levels, but this difference was not statistically significant. Most of the findings in Table 5 were confirmed using multivariate analysis (data not shown). These data should be interpreted with care because for some activities few measurements were available.

DISCUSSION

The study set out to assess the feasibility of using direct measurements of exposure in a case–control study of adult brain tumours in the UK. The practicality of making measurements and validity and precision of exposure estimates was assessed.

Individuals were willing to undertake a 3–4 d period of personal measurements; only two people refused to participate and only three indicated a reluctance to be involved in further measurements. Of the UKABTS subjects approached 17 out of 18 (94%) cases consented as did 21 out of 22 (95%) controls.

Another area of concern was the co-operation of employers especially when employee anxieties regarding potential hazards could be aroused. A

Table 6. Summary statistics for the various occupational exposure metrics by activities.

Tasks	*n*	TWA			H-TWA			ITM			MAX		
		AM	GM	GSD	AM	GM	GSD	AM	GM	GSD	AM	GM	GSD
No. EMF tasks	30	0.05	0.03	2.41	0.01	0.01	3.49	0.01	0.01	2.30	1.61	0.81	3.37
Any EMF task	199	0.25	0.13*	2.80	0.07	0.04	2.99	0.04	0.02*	2.92	4.63	2.23**	3.27
Computer	135	0.21	0.12	2.65	0.06	0.03	2.83	0.02	0.01	2.61	3.15	1.68	3.05
Electrical transport	14	0.35	0.15	2.85	0.08	0.05	2.97	0.10	0.06*	2.71	15.19	6.79**	3.06
Motors	99	0.24	0.17	2.19	0.08	0.05	2.42	0.04	0.03*	2.56	5.09	3.07**	2.83
Welding	3	0.30	0.24*	2.16	0.16	0.11**	2.84	0.11	0.09***	1.95	17.13	15.32*	1.86
Soldering	2	0.05	0.05	1.49	0.02	0.02	1.21	0.01	0.01	1.36	1.88	1.76	1.66
Assembling	2	0.05	0.04	1.99	0.02	0.01	2.47	0.01	0.01	1.04	1.54	1.18	2.93
Other electrical tools	22	0.20	0.13	2.96	0.06	0.03	3.12	0.03	0.02	2.76	4.52	2.50	2.94
Induction heaters	1	0.07	0.07	—	0.02	0.02	—	0.01	0.01	—	1.18	1.18	—
Power stations	6	0.65	0.26	3.56	0.05	0.04	1.66	0.08	0.04	3.50	8.85	3.22	3.81
Working near high-voltage lines	28	0.59	0.27*	3.63	0.04	0.03	2.06	0.06	0.04*	2.61	6.83	4.46**	2.51
High current wires	16	0.82	0.37	4.34	0.05	0.04	2.34	0.07	0.05	2.89	8.56	5.08*	2.96

n: number of days with measurements; AM: arithmetic mean; GM: geometric mean; GSD: geometric standard deviation.
$^{*}p < 0.05$; $^{**}p < 0.01$; $^{***}p < 0.001$.

Downloaded from https://academic.oup.com/rpd/article/108/3/227/1593973 by University of Torino user on 19 November 2021

company compliance rate of 83% points to a general willingness to support such studies at least on a one-off basis. Four of the five company refusals concerned requests for proxy employees.

The diagnosis of brain tumours carries a poor prognosis and is more prevalent in the older population. This means that the use of proxy exposure data is a crucial element of any proposed extension of the study. In two instances company refusal appeared to be based on the view that the meter could represent a physical hazard when carrying out work in confined spaces.

Of the 17 proxy selections 14 (82%) were considered to be good primary subject matches in terms of job description and working environment. However, on a small number of occasions, the tasks were not comparable with those of the primary subject for a proportion of the dosimetic period, and it was unclear whether the proxies were appropriate. Consequently, proxy selection needs to be kept under surveillance, with proxies asked to describe their general duties for comparison with information provided by the study subject. Where the information is incompatible, exposure estimates will need to be excluded from the analyses. The present findings are in line with other studies[20,21] suggesting that proxy selection should ideally be based on the detailed matching of company, job title, task and specific location.

Few technical problems have been encountered in the collection of exposure data in a wide spectrum of environments. 'Bad data' flags, due to a rapidly changing environment, were found in only three of the 79 exposure traces and then attached to less than 0.03% of the readings. The routine inspection of exposure traces proved to be a valuable procedure, revealing periods of questionable data, which could be investigated and excluded if necessary. For two individuals, overnight periods of constantly elevated levels matched precisely when the monitor was not worn. The high values were interpreted to be the result of placing the monitor close to local sources such as electrical appliances, and the data were excluded from the exposure calculations. Consistently high readings were also seen in the occupational exposure of a radiographer involved with magnetic resonance imaging (MRI). A survey of the work environment showed no general areas with high levels and it transpired that the meter had been left close to a piece of equipment with an operating battery charger that gave readings of 3 μT at 3 cm and 0.35 μT at 30 cm.

The WHO international EMF project[17] has recommended that the design of EMF studies should be such as to include as much information relevant to alternative metrics as possible in order to aid future research. The correlation matrix, in Table 3, illustrating varying correlation between the considered exposure metrics, is in broad accord with other studies assessing exposure with the same instrumentation in children[22] and in adults[23]. TWA exposure was highly correlated with exposure summarised by GM, % >0.2 μT and by H-TWA, but not with the MAX exposure. In a study carried out in the UK electricity distribution and supply industry, for un-transformed exposures, correlation of TWA with median exposure, 90th percentile and SD metrics was reported as 0.84, 0.90 and 0.75, respectively[24]. In a French–Canadian study of electricity utilities, the correlation of TWA with 90th percentile and with maximum field was reported as 0.94 and 0.66, respectively[25]. Inclusion of highly correlated exposure metrics in exposure response analyses will provide little additional information, and on the basis of the results of this feasibility study the use of TWA, H-TWA, ITM and MAX will probably be sufficient in any future extension of this work.

Adult exposure readings appear to offer a reliable estimate of exposure over the longer term. Though day-to-day variation may be underestimated in data from consecutive days, the results showed that a 3-d measurement period provided exposure estimates with sufficient precision to examine the presumed exposure–response relationship for the TWA, ITM and H-TWA exposures. However, more measurements would be required to limit potential attenuation when using the MAX exposure metric. It should be noted that these analyses assumed that data were available for all individuals in the study, which is unlikely to be the case in the full study.

This study shows that for a range of metrics, occupational exposure is the major determinant of total exposure in both the full pilot study and the smaller population of UKABTS participants. In this light, the omission of residential exposure, in all but the current home, from the estimate of overall exposure would seem to be justifiable.

There were statistically significant differences in the exposures of certain occupational categories. However, the between-worker variance in exposure was high for many occupational categories, indicating that the individuals within the categories had greatly different exposures. In any case–control study of a disease which has a poor prognosis and is more prevalent in the elderly, there will be a need for proxies in order to estimate primary exposure. In this study it was possible only to consider the primary–proxy exposure relationship using the groups of individuals with the same job classification (Table 5). However, most group members would not be considered to be ideal proxies for others in the group as the workplace and job descriptions did not match precisely. On occasion, high variance between workers resulted from diversity in individual working environments that is unrelated to job category or tasks performed. The group of security

Downloaded from https://academic.oup.com/rpd/article/108/3/227/1593973 by University of Torino user on 19 November 2021

officers contained a small sub-group, working in the proximity of a high voltage overhead line and substation, that have consistently higher exposures than the remainder of the group. For other groups the variance in exposure could be related whether particular tasks are carried out as well as to how these tasks were carried out. A group of dentists, working in adjacent rooms of the same practice and generally performing the same range of tasks with the same equipment, would be considered as close to ideal proxies. Yet considerable variance in individual exposures were traced to changes in the relative position of the equipment used. These observations are consistent with a study reporting large differences in exposure in an apparently homogeneously exposed occupational group[(26)]. Within other groups where proxies were ideal in terms of precise workplace, job description, equipment used and working environment (e.g. the warp knitters) exposure levels were more consistent.

Due to the likely differential use of proxies between cases and controls and to the high variability in exposure the selection of ideal proxies, matched as far as possible on occupation, task and workplace is essential. During the main study, the following guidelines were used for selection of the proxies:

— Has another employee filled the position previously kept by the study subject?
— Are there other employees in the company who carry out the same or similar job in terms of use of equipment, at same the workplace/department?
— If not, can you identify employees with very similar tasks and using the same/similar equipment in other departments, building, sites, etc?
— If not, can you identify employees working in or very near the same area as the real study subject?

It was also decided to collect, whenever possible, data from multiple proxies. Information on general duties will be collected that will allow inappropriate proxies to be excluded and thereby improve precision and reduce the risk of misclassification. It is interesting to note that although electricians have been classified by a Swedish job matrix as being highly exposed[(27)], the electrician in the present study had geometric mean exposures of 0.04, 0.05 and 0.09 μT for work shifts on three successive days. An electrician in a Finnish study[(23)] had the lowest TWA and GM exposure measured, at 0.09 and 0.05 μT, respectively.

The ability of task-based questionnaires to identify high exposure has been considered in respect of the daily occupational questionnaire completed alongside the diary. Welding was associated with statistically significantly increased levels for all exposure metrics, consistent with what is reported in the literature (see e.g. Floderus *et al.*[(27)] and Bowman *et al.*[(28)]). Working near high-voltage lines was associated with increased levels for TWA, ITM and MAX, but not for H-TWA, whilst working with electrical motors and electrical transport equipment was associated with elevated ITM and MAX levels. However, the number of days on which tasks were performed was often low, the variance of exposure within task-specific days high and no account was taken of the duration and personal proximity to equipment involved in the task. Also, sometimes very disparate activities were grouped under one heading. An extension of exposure measurements in the UKABTS will increase the frequency of task-specific days and potentially increase the precision of exposure estimates associated with such days.

In summary, the study has shown that valid direct measures of occupational exposure are possible in the UKABTS and high levels of co-operation from employers and employees would be expected. The results also show that proxies need to be selected carefully and appropriateness reviewed based on the detailed job descriptions from primary and proxy subjects. In addition, it is suggested to collect, whenever possible, exposure data from multiple proxies. Although significant differences in exposure were observed for certain job categories and certain activities were associated with elevated exposure levels, there was little evidence that a task-related questionnaire response would be a reliable predictor of high exposure. Direct measurements appear to be the preferred mode of assigning accurate and precise exposure estimates and results from measurements on 3 d appeared to be of sufficient precision to be used in exposure response analyses, if data can be obtained relating to all subjects in the case–control study.

The results of this study form the basis for the further collection of data in the UKABTS and for developing exposure estimates for ELF MFs. Validated direct exposure measures from this previously unsampled population will add to the body of exposure data available to future studies.

ACKNOWLEDGEMENTS

The feasibility study was funded by the Health and Safety Executive in the United Kingdom. The authors wish to thank all participants, including all the companies that allowed measurements to be taken on their premises. Some tables and figures in this paper are reproduced by kind permission of National Radiological Protection Board.

REFERENCES

1. Muir, C. S., Storm, H. H. and Polednak, A. *Brain and other nervous system tumours*. In: Cancer Surveys Volume 19/20: Trends in Cancer Incidence and Mortality (Imperial Cancer Research Fund) (1994).

Downloaded from https://academic.oup.com/rpd/article/108/3/227/1593973 by University of Torino user on 19 November 2021

2. Polednak, A. P. *Time trends in incidence of brain and central nervous system cancers in Connecticut*. J. Natl. Cancer Inst. **83**, 1679–1681 (1991).
3. Davis, D. L., Hoel, D., Fox, J. and Lopez, A. D. *International trends in cancer mortality in France, West Germany, Italy, Japan, England and Wales and the USA*. Lancet **336**, 474–481 (1990).
4. Davis, D. L., Ahlbom, A., Hoel, D. and Percy, C. *Is brain cancer mortality increasing in industrial countries?* Am. J. Ind. Med. **19**, 421–431 (1991).
5. Desmeules, M., Mikklesen, T. and Mao, Y. *Increasing incidence of primary malignant brain tumors: influence of diagnostic methods*. J. Natl Cancer Inst. **84**, 442–445 (1992).
6. Grant, R., Collie, D. and Counsell, C. *The incidence of cerebral glioma in the working population; a forgotten cancer?* Br. J. Cancer **73**, 252–254 (1996).
7. Greig, N. H., Ries, L. G., Yancik, R. and Rapoport, S. I. *Increasing annual incidence of primary malignant brain tumors in the elderly*. J. Natl Cancer Inst. **82**, 1621–1624 (1990).
8. Black, P. M. *Brain tumors. Part 1*. N. Engl. J. Med. **324**, 1471–1476 (1991).
9. Davis, D. L. and Schwartz, J. *Trends in cancer mortality: US white males and females, 1968–83*. Lancet **1**, 633–636 (1988).
10. Marshall, E. *Experts clash over cancer data*. Science **250**, 900–902 (1990).
11. Davis, D. L., Ahlbom, A., Hoel, D. and Percy, C. *Is brain cancer mortality increasing in industrial countries?* Am. J. Indust. Med. **19**, 421–431 (1991).
12. Muir, C., Waterhouse, T., Mack, T., Powell, J and Whelan, S. (Eds) *Cancer incidence in five continents*. Vol. V. IARC Scientific Publication No. 88. (Lyon, France: International Agency for Research on Cancer) (1987).
13. Inskip, P. D., Linet, M. S. and Heineman, E. F. *Etiology of brain tumors in adults*. Epidemiol. Rev. **17**(2), 382–414 (1995).
14. Rodvall, Y., Ahlbom, A., Stenlund, C., Preston-Martin, S., Lindh, T. and Spannare, B. *Occupational exposure to magnetic fields and brain tumours in central Sweden*. Eur. J. Epidem. **14** 563–569 (1998).
15. Harrington, J. M., McBride, D. I., Sorahan, T., Paddle, G. M. and van Tongeren, M. *Occupational exposures to magnetic fields in relation to mortality from brain cancer among electricity—generation and transmission workers*. Occup. Environ. Med. **54**, 7–13 (1997).
16. Cardis, E. and Kilkenny, M. *International case–control study of adult brain, head and neck tumours: results of the feasibility study*. Radiat. Prot. Dosim. **83**, 179–183 (1999).
17. World Health Organisation. *The International EMF Project*. Available on http://www/who.int/peh-emf/project.
18. van Tongeren, M., Mee, T. J., Whatmough, P., Maslanyj, M. P., Allen, S. E., Muir, K. R. and McKinney, P. A. UK case control study of the aetiology of adult brain tumours and neuromas. Exposure to ELF magnetic fields – Report of a feasibility study. National Radiological Protection Board. NRPB-W50, Didcot, UK (2003).
19. Armstrong, B. K., White, E. and Saracci, R. *Principles of exposure measurement in epidemiology*. Monographs in Epidemiology and Statistics, Vol. 21 (Oxford, UK: Oxford Medical Publications, Oxford University Press) (1992).
20. Kheifets, L. *Occupational exposure assessment in epidemiological studies of EMF*. Radiat. Prot. Dosim. **83**, 61–69 (1999)
21. Kelsh, M. A., Kheifets, L. and Smith, R. *The impact of work environment, utility, and sampling design on occupational magnetic field exposure summaries*. Am. Ind. Hyg. Assoc. J. **61**, 174–182 (2000).
22. Folliart, D. E., Iriye, R. N., Tarr, K. J., Silva, M., Kavet, R. and Ebi, K. L. *Alternative magnetic field exposure metrics: relationship to TWA, appliance use, and demographic characteristics of children in a leukemia survival study*. Bioelectromagnetics **22**, 574–580 (2001).
23. Hansen, N. H., Sobel, E., Davanipour, Z., Gillette, L. M., Niiranen, J. and Wilson, B. W. *EMF exposure assessment in the Finnish garment industry: evaluation of proposed EMF exposure metrics*. Bioelectromagnetics **21**, 57–67 (2000).
24. Merchant, C. J., Renew, D. C. and Swanson, J. *Occupational exposures to power-frequency magnetic fields in the electricity supply industry*. J. Radiol. Prot. **14**, 155–164 (1994).
25. Armstrong, B. G., Deadman, J. E. and Theriault, G. *Comparison of indices of ambient exposure to 60 Hz electric and magnetic fields*. Bioelectromagnetics **11**, 337–347 (1990).
26. Rapapport, S. M., Kromhout, H. and Symanski, E. *Variation of exposure between workers in homogeneous groups*. Am. Ind. Hyg. Assoc. J. **54**, 654–662 (1993).
27. Floderus, B., Stenlund, C. and Persson, T. *Occupational magnetic field exposure and site-specific cancer incidence: a Swedish cohort study*. Cancer Causes Contr. **10**, 323–323 (1999).
28. Bowman, J. D., Garabrandt, D. H., Sobel, E. *et al. Exposures to extremely low frequency (ELF) electromagnetic fields in occupations with elevated leukaemia risks*. Appl. Ind. Hyg. **3**, 189–194 (1988).

Downloaded from https://academic.oup.com/rpd/article/108/3/227/1593973 by University of Torino user on 19 November 2021
