# Supplementary material for: Magnetic Fields and Cancer: Epidemiology, Cellular Biology, and Theranostics
Source: Int J Mol Sci. 2022 Jan 25;23(3):1339. doi: 10.3390/ijms23031339 (PMC8835851; doi:10.3390/ijms23031339)
Supplement: Supplementary file 1 [file ijms-23-01339-s001.zip › Supplementary Tables S1-S5/Supplementary Table S1.pdf]

International Journal of *Molecular Sciences*

Supplementary

Magnetic Fields and Cancer

Massimo E. Maffei [1,*]

[1] Dept. Life Sciences and Systems Biology, University of Turin, Via Quarello 15/a, 10135 Turin, Italy; massimo.maffei@unito.it
* Correspondence: massimo.maffei@unito.it; Tel.: +39011 6705967

Supplementary Table S1.

Relationship between magnetic fields and cancer in residential/domestic MF exposure epidemiologic studies

Type of cancer	Study	Source of MFs	Range of MFs	Location	Conclusions	Ref.
brain tumors glioma	population-based case-control study	power transmission lines	50/60 Hz 0.3 µT	France	Significant associations between cumulated duration living at< 50 m to high voltage lines and: i) all brain tumors (odd ratios 2.94; 95%CI 1.28-6.75); ii) glioma (odd ratios 4.96; 95%CI 1.56-15.77).	[1]
brain tumors	population-based case-control study of children under 15 years of age	residential power-frequency MFs	0.1>µT>0.4	Japan	A positive association was found between high-level exposure-above 0.4 µT-and the risk of brain tumors.	[2]
brain tumors, childhood	pooled analysis based on primary data	residential ELF-MF exposure	0.1-≥0.4 µT	U.K.	Results provide little evidence for an association between ELF-MF exposure and childhood brain tumors	[3]
brain cancer, childhood	meta-analysis	power lines	0.2-0.4 µT	U.S.A.	With the exception of high cut-point analyses (0.3/0.4 µT), where the possibility of a moderate risk increase cannot be excluded, No increase in childhood brain cancer risk was evident for any of the exposure metrics	[4]
brain cancer	meta-analysis of occupational studies	different sources	> 0.2 µT	worldwide	Little or no association between electric and MF exposure and brain cancer	[5]
breast cancer	melatonin hypothesis	exposure sessions of 30 min each of sham, continuous, and intermittent (15 s on/off cycles)	50 Hz 100 µT	Belgium	No indications that daytime acute MF exposure influences either melatonin secretion or aMT6s excretion	[6]

Type of cancer	Study	Source of MFs	Range of MFs	Location	Conclusions	Ref.
breast cancer	melatonin hypothesis	environments exposed to MFs	50-Hz weekly 0.1 to 2.6 μT	France	MFs do not have cumulative effects on melatonin secretion in humans	[7]
breast cancer	24-hour EMF measurements in the most lived-in room	residential EMF exposures	0.07-0.19 μT	U.S.A.	Results agree with other recent reports of no association between breast cancer and residential EMF exposures	[8]
breast cancer	electric bedding device and increased breast cancer risk	electric bedding device	n.a.	U.S.A.	The use of electric bedding devices may increase breast cancer risk in African-American women aged 20-64 years. Such an association might not vary substantially by menopausal status or estrogen receptor status	[9]
breast cancer	residential exposure to power-frequency MFs	wiring configuration associated with the highest MFs	40 Hz - 800 Hz; 0.01-0.4 μT	U.S.A.	Residential MF exposures commonly experienced by US women do not influence risk of breast cancer	[10]
breast cancer	melatonin hypothesis	at-home electric appliance use	60-Hz	U.S.A.	No support for the hypothesis that exposure to residential MFs is associated with an increased risk of developing breast cancer	[11]
breast cancer	melatonin hypothesis	residential MFs	0.3-0.4 μT	U.S.A.	MF exposure does not increase breast cancer risk , even within specific subgroups of women who might be more susceptible to the effects of such exposure	[12]
breast cancer	melatonin hypothesis	higher bedroom MF level	60-Hz	U.S.A.	Exposure to nighttime residential 60-Hz MFs can depress the normal nocturnal rise in melatonin	[13]
breast cancer	melatonin hypothesis	residential MFs	60-Hz 5-10 mG > ambient MF	U.S.A.	Exposure to MFs is associated with decreased nocturnal melatonin levels, but does not support the hypothesis that such exposure results in increased urinary levels of estrogens, LH, or FSH	[14]
breast cancer	melatonin hypothesis	continuous and intermittent MF exposure	60 Hz 127.3 μT	U.S.A.	Nocturnal secretion and metabolism of melatonin are not altered in humans by field exposure at an intensity over 600 times higher than that typically encountered in the home	[15]

Type of cancer	Study	Source of MFs	Range of MFs	Location	Conclusions	Ref.
breast cancer	melatonin hypothesis	home environment	< 0.5 μT	U.S.A.	No influence of nocturnal environmental MF exposure on aMT6s excretion in older adults	[16]
cancer	Electromagnetic-fields in the home-environment	color tv, computer monitor, micro-wave-oven, cellular phone,	2.45 GHz	U.S.A.	The consumption of microwave-cooked food and exposure of the human body to micro-waves over a long period of time may influence the nutritional state of diet individual and may contribute to induction of cancer	[17]
cancer, childhood	retrospective cohort study	high voltage power transformer station and transmission line	≥0.2 μT	Canada	Residential proximity to transformer stations is associated with a borderline risk of childhood cancer, but the absence of an association with transmission lines suggests no causal link	[18]
cancer, childhood	national case-control study	high-voltage power lines	0.1-≥0.4 μT	England and Wales	MF exposure during the year of birth is unlikely to be the whole cause of the association with distance from overhead power lines	[19]
cancer, childhood	meta-analysis	e high voltage power lines	≥ 0.2 μT	Worldwide	It cannot be concluded that children exposed to non-ionizing radiation have higher risks of childhood cancer	[20]
endometrial cancer	electric blanket exposure	electric blanket mattress cover	n.a.	U.S.A.	Provides evidence against an association between electric blanket or mattress cover use and endometrial cancer	[21]
hematological cancers	residence situated in a broad corridor around a high-voltage power line	high-voltage power line	0.05 - 0.20 μT	Norway	Occupational exposure showed no significant association to exposure for any site. Conclusions: Some elevated odds ratios were observed in the present study, but the results are based on small numbers and no firm conclusions can be drawn	[22]
leukemia	female urinary excretion of 6-sulfatoxymelatonin (6-OHMS	power lines	0.08-0.29 μT	Canada	Chronic residential exposure to MFs from high-power lines may accentuate the decrease in melatonin secretion observed in some vulnerable subgroups of the population	[23]
leukemia, acute lymphoblastic (ALL)	meta-analysis	stationary bedroom meas-urements	≥0.3 μT	Canada, Denmark, Germany,	ELF-MF exposure has no impact on the survival probability or risk of relapse in children with ALL	[24]

Type of cancer	Study	Source of MFs	Range of MFs	Location	Conclusions	Ref.
				Japan, U.K., U.S.A.		
leukemia	dose-response and time-related factors	high-voltage power lines	≥ 0.01 µT	Finland	Typical MFs of high-voltage power lines are not an important cause of leukemia in adults	[25]
leukemia	possible association between MFs exposure in the pediatric population	igh voltage power lines (≥132 kilo-volts	0.1-0.4 µT	Italy	More suggestive of an excess risk of leukemia among exposed children than of a null relation	[26]
leukemia, acute	residential MF exposure and proximity	Overhead transmission lines	≥ 0.3 µT	Malaysia	Residential proximity does not constitute any risk to children's health	[27]
leukemia, B-lineage acute lymphoblastic (B-ALL)	Residential 24-h ELF-MF measurements	children's rooms near their beds	≥0.4 µT	Mexico	ELF-MF exposure may be associated with the risk of B-ALL	[28]
leukemia<br>brain tumor<br>breast cancer, female	residential distance less than or equal to 100 m	power lines	≥0.2 µT	Taiwan	Association between MF exposure and a greater mean age at diagnosis for brain tumors	[29]
leukemia<br>brain/central nervous system cancers<br>malignant melanoma<br>breast cancer	case-control study in relation to distance and ELF-MF	overhead power lines	≥1 µT	U.K.	Results do not support an epidemiologic association of adult cancers with residential MFs in proximity to high-voltage overhead power lines	[30]

Type of cancer	Study	Source of MFs	Range of MFs	Location	Conclusions	Ref.
leukemia lung cancer, childhood non-melanoma skin cancer	cases that might occur near high-voltage powerlines	high-voltage powerlines	>0.1 µT	U.K.	Within 400 m of powerlines, this may result annually in 200-400 excess cases of lung cancer, 2000-3000 cases of other illnesses associated with air population and 2-6 cases of childhood leukemia. Seventeen cases of non-melanoma skin cancer might occur by exposure directly under powerlines	[31]
leukemia breast cancer brain cancer	pooled or meta-analyses	power lines or other sources of electricity	≥0.4 µT	worldwide	Strong evidence that excessive exposure to MFs increases risk of adult leukemia, male and female breast cancer and brain cancer	[32]
leukemia, childhood	upwind carcinogens are ionized when passing power lines	high-voltage power lines	≥0.4 µT	Australia	Increased risk of childhood leukemia near high voltage lines	[33]
leukemia, childhood	comparison of Australian residential MF exposure with those in other countries	power-frequency MFs	0.05-0.4 µT	Australia	A causal association between residential MFs and childhood leukemia is expected to be small	[34]
leukemia, childhood	measurements inside home and distance	power lines	≥0.3 µT	Brazil	Results do not provide support for an association between MFs and childhood leukemia, but small numbers and likely biases weaken the strength of this conclusion	[35]
leukemia, childhood	meta-analysis on mobility	200+ kilovolt power line	≥0.4 µT	Brazil, Denmark, France, Italy, Norway, Sweden, Switzerland, Tasmania,	Mobility appears to be an unlikely explanation for the associations observed between power lines exposure and childhood leukemia	[36]

Type of cancer	Study	Source of MFs	Range of MFs	Location	Conclusions	Ref.
				U.K., U.S.A.		
leukemia, child-hood	paired case-control study	house ELF-MF	≥0.4 μT	Czech Re-public	No indication of an association between ELF-ME exposure and child-hood leukemia was determined. This in contrast to the findings of previ-ous studies conducted in different countries	[37,38]
leukemia, child-hood CNS tumour malignant lym-phoma	pooled ana-lyses	50 to 400 kV facili-ties	≥ 0.4 μT	Danemark	The results for childhood leukemia were in line with large pooled anal-yses showing relative risks between 1.5 and 2.	[39]
leukemia, child-hood	interaction be-tween distance to nearest power line and domestic ra-don regarding risk of child-hood leukemia	132-400 kV over-head power line	n.a.	Denmark	No change in the estimated association between distance to power line and risk of child-hood leukemia	[40]
leukemia, child-hood	epidemiologi-cal study	high voltage under-ground cables	>0.4 μT	England and Wales	The absence of risk detected in relation to Underground cables tends to add to the argument that any risks from overhead lines may not be caused by MFs	[41]
leukemia, child-hood	carcinogenic effects are based on the radical pair mechanism (RPM)		≥ 100 μT	Finland	While the radical pair mechanism appears to be involved in sensing the static GMF by animals, it remains unclear how it could explain human health effects of ELF MFs weaker than 1 μT	[42]
leukemia, child-hood	meta-analysis	power-frequency MFs	> 0.2 μT	Germany	A dose-response-relationship was observed after combining the data of all German studies on MFs and childhood leukemia. The evidence for an	[43]

Type of cancer	Study	Source of MFs	Range of MFs	Location	Conclusions	Ref.
					association between childhood leukemia and MF exposure in our study comes from a measure of exposure during the night	
leukemia, childhood	exposure duration, frequency range, distance from the radiation sources, and location during measurement of MF density	power transmission lines	50-60 Hz ≥ 0.3/0.4 μT	India	No clear relation observed between specific source of MF and childhood leukemia.	[44]
leukemia, childhood	meta-analyses: modifiable and non-modifiable factors affecting the risk of childhood leukemia	multiple	≥ 0.4 μT	Iran	Exposure to EMFs is found to be a significant risk factor	[45]
leukemia, childhood	continuous exposure	child's bedroom	≥0.3 μT	Italy	Results may be affected by several sources of bias and they are noninformative	[46]
leukemia, childhood	Personal and bedroom measurements performed in children	power lines and built-in transformers	< 0.4 μT	Italy	Exposure levels in the city of Milan are always significantly far lower than 0.3–0.4 μT	[47]
leukemia, childhood	treatment in an infant incubator	incubators for each incubator model kept by the hospitals	0.23-4.36 μT	Sweden	Gives little evidence that exposure to MFs inside infant incubators is associated with an increased risk of childhood leukemia	[48]

Type of cancer	Study	Source of MFs	Range of MFs	Location	Conclusions	Ref.
leukemia, childhood	pooled analysis	power lines	0.6>µT<0.1	The Netherlands	The policy is effective in preventing new situations with long-term exposure of children to MFs from overhead power lines, but does not include underground cables and other sources of MFs	[49]
leukemia, childhood	population-based case-control study. within 50 m of >200 kV lines	high-voltage electric power transmission lines	200 kV n.a.	U.S.A.	Not clearly support an increased childhood leukemia risk associated with close proximity (<50 m) to higher voltage lines, but could be consistent with a small increased risk	[50]
leukemia, childhood	contact current on the domestic water pipe	water pipe used for grounding	0.1-0.6 µT	U.S.A.	No value of information for finding the odds ratio given the contact current hypothesis	[51]
leukemia, childhood	odds ratios (OR) adjusted for age, sex, Hispanic ethnicity, mother's race and household income	contact current exposure	0.01-0.25 µT	U.S.A.	No evidence of an association between childhood leukemia and exposure to contact currents or MFs	[52]
leukemia, childhood	interaction between distance and MFs exposure	power transmission lines	≥ 0.4 µT	U.S.A.	Findings argue against MFs as a sole explanation for the association between distance and childhood leukemia and in favor of some other explanation linked to characteristics of power lines	[53]
leukemia, childhood	large records-based case-control study	power lines	0.1>µT≥0.4	U.S.A.	Clear evidence of risk associated with greater exposure to MFs from power lines	[54]
leukemia, childhood	association between residential MFs and	high wire-codes	≥ 0.3 µT	U.S.A.	No statistically significant association between wire codes and childhood leukemia is observed	[55]

Type of cancer	Study	Source of MFs	Range of MFs	Location	Conclusions	Ref.
	childhood leukemia					
leukemia, childhood	pooled analyses and meta-regression analysis	power-frequency MFs	geoMF 25-65 µT	U.S.A.	Investigating whether GMF appears to be an effect modifier in studies of alternating MFs resulted in rather limited and not statistically significant, evidence for this.	[56]
leukemia, childhood	exposure assessment and data analysis plan	high-voltage overhead transmission line	> 0.4 µT	U.S.A.	The study provides for a powerful test on a hypothesized association between distance to power lines and childhood leukemia	[57]
leukemia, childhood	meta-analysis	different sources	0.4>µT<0.2	worldwide	Result indicate that MF exposure level may be associated with childhood leukemia	[58]
leukemia, childhood	meta-analysis	different sources	0.1-2.36 µT	worldwide	Statistical association between MF intensity and childhood leukemia (ranged 0.1-2.36 µT) and no relationship between exposure to >= 0.1 µT and childhood leukemia	[59]
leukemia, childhood acute (AL)	dose-based geographic zoning	nuclear power plants	63–150 kV	France	Potential risk factors related to the vicinity of nuclear power plants	[60]
leukemia, childhood acute (AL)	nationwide study	High-voltage overhead power lines	225-400 kV ≥0.4 µT	France	Supports the previous international findings of an increase in AL incidence close to power lines	[61]
leukemia, childhood acute lymphoblastic (ALL)	international follow-up study on survival probability or risk of relapse	ELF–MF exposures measured in homes	≥0.3 µT	Canada, Denmark, Germany, Japan, U.K. and U.S.A.	ELF-MF exposure has no impact on the survival probability or risk of relapse in children with ALL	[62]
malignant tumors	magnetic induction of the	power transmission lines	20-2511 nT	Russia	An increase in the incidence of malignant tumors has been noted as the induction of the MF produced by overhead power transmission lines increases.	[63]

Type of cancer	Study	Source of MFs	Range of MFs	Location	Conclusions	Ref.
	industrial frequency				The received epidemiological data demonstrate the role of MFs of industrial frequency as a risk factor for occurrence of oncological diseases in the population	
melanoma	distance to FM towers	FM broadcasting towers	~100MHz	Sweden	Melanoma is associated with exposure to FM broadcasting	[64]
neurogenic tumors	overnight exposure on the nocturnal urinary levels of biogenic amines	Helmholtz coils	50-Hz 10 μT	France	Nocturnal exposure to either continuous or intermittent MFs does not affect the nocturnal excretion of biogenic amines in healthy young men	[65]

References

1. Carles, C.; Esquirol, Y.; Turuban, M.; Piel, C.; Migault, L.; Pouchieu, C.; Bouvier, G.; Fabbro-Peray, P.; Lebailly, P.; Baldi, I. Residential proximity to power lines and risk of brain tumor in the general population. *Environmental Research* **2020**, *185*.
2. Saito, T.; Nitta, H.; Kubo, O.; Yamamoto, S.; Yamaguchi, N.; Akiba, S.; Honda, Y.; Hagihara, J.; Isaka, K.; Ojima, T., *et al.* Power-frequency magnetic fields and childhood brain tumors: A case-control study in japan. *Journal of Epidemiology* **2010**, *20*, 54-61.
3. Kheifets, L.; Ahlbom, A.; Crespi, C.M.; Feychting, M.; Johansen, C.; Monroe, J.; Murphy, M.F.G.; Oksuzyan, S.; Preston-Martin, S.; Roman, E., *et al.* A pooled analysis of extremely low-frequency magnetic fields and childhood brain tumors. *American Journal of Epidemiology* **2010**, *172*, 752-761.
4. Mezei, G.; Gadallah, M.; Kheifets, L. Residential magnetic field exposure and childhood brain cancer - a meta-analysis. *Epidemiology* **2008**, *19*, 424-430.
5. Kheifets, L.I. Electric and magnetic field exposure and brain cancer: A review. *Bioelectromagnetics* **2001**, S120-S131.
6. Crasson, M.; Beckers, V.; Pequeux, C.; Claustrat, B.; Legros, J.J. Daytime 50 hz magnetic field exposure and plasma melatonin and urinary 6-sulfatoxymelatonin concentration profiles in humans. *Journal of Pineal Research* **2001**, *31*, 234-241.
7. Touitou, Y.; Lambrozo, J.; Camus, F.O.; Charbuy, H. Magnetic fields and the melatonin hypothesis: A study of workers chronically exposed to 50-hz magnetic fields. *American Journal of Physiology-Regulatory Integrative and Comparative Physiology* **2003**, *284*, R1529-R1535.
8. Schoenfeld, E.R.; O'Leary, E.S.; Henderson, K.; Grimson, R.; Kabat, G.C.; Ahnn, S.; Kaune, W.T.; Gammon, M.D.; Leske, M.C.; Grp, E. Electromagnetic fields and breast cancer on long island: A case-control study. *American Journal of Epidemiology* **2003**, *158*, 47-58.
9. Zhu, K.M.; Hunter, S.; Payne-Wilks, K.; Roland, C.L.; Forbes, D.S. Use of electric bedding devices and risk of breast cancer in african-american women. *American Journal of Epidemiology* **2003**, *158*, 798-806.
10. London, S.J.; Pogoda, J.M.; Hwang, K.L.; Langholz, B.; Monroe, K.R.; Kolonel, L.N.; Kaune, W.T.; Peters, J.M.; Henderson, B.E. Residential magnetic field exposure and breast cancer risk: A nested case-control study from a multiethnic cohort in los angeles county, california. *American Journal of Epidemiology* **2003**, *158*, 969-980.
11. Davis, S.; Mirick, D.K.; Stevens, R.G. Residential magnetic fields and the risk of breast cancer. *American Journal of Epidemiology* **2002**, *155*, 446-454.
12. Davis, S.; Mirick, D.K. Residential magnetic fields, medication use, and the risk of breast cancer. *Epidemiology* **2007**, *18*, 266-269.
13. Davis, S.; Kaune, W.T.; Mirick, D.K.; Chen, C.; Stevens, R.G. Residential magnetic fields, light-at-night, and nocturnal urinary 6-sulfatoxymelatonin concentration in women. *American Journal of Epidemiology* **2001**, *154*, 591-600.
14. Davis, S.; Mirick, D.K.; Chen, C.; Stanczyk, F.Z. Effects of 60-hz magnetic field exposure on nocturnal 6-sulfatoxymelatonin, estrogens, luteinizing hormone, and follicle, stimulating hormone in healthy reproductive-age women: Results of a crossover trial. *Annals of Epidemiology* **2006**, *16*, 622-631.
15. Graham, C.; Cook, M.R.; Gerkovich, M.M.; Sastre, A. Melatonin and 6-ohms in high-intensity magnetic fields. *Journal of Pineal Research* **2001**, *31*, 85-88.

16. Youngstedt, S.D.; Kripke, D.F.; Elliott, J.A.; Assmus, J.D. No association of 6-sulfatoxymelatonin with in-bed 60-hz magnetic field exposure or illumination level among older adults. *Environmental Research* **2002**, *89*, 201-209.
17. Omura, Y.; Losco, M. Electromagnetic-fields in the home-environment (color tv, computer monitor, microwave-oven, cellular phone, etc) as potential contributing factors for the induction of oncogen c-fos ab1, oncogen c-fos ab2, integrin alpha-5-beta-1 and development of cancer, as well as effects of microwave on amino-acid-composition of food and living human brain. *Acupuncture & Electro-Therapeutics Research* **1993**, *18*, 33-73.
18. Auger, N.; Bilodeau-Bertrand, M.; Marcoux, S.; Kosatsky, T. Residential exposure to electromagnetic fields during pregnancy and risk of child cancer: A longitudinal cohort study. *Environmental Research* **2019**, *176*.
19. Kroll, M.E.; Swanson, J.; Vincent, T.J.; Draper, G.J. Childhood cancer and magnetic fields from high-voltage power lines in england and wales: A case-control study. *British Journal of Cancer* **2010**, *103*, 1122-1127.
20. Zaki, A.M.; Abd Rahim, M.A.; Zaidun, Z.; Ramdzan, A.R.; Isa, Z.M. Exposure to non-ionizing radiation and childhood cancer: A meta-analysis. *Middle East Journal of Cancer* **2020**, *11*, 1-11.
21. McElroy, J.A.; Newcomb, P.A.; Trentham-Dietz, A.; Hampton, J.M.; Kanarek, M.S.; Remington, P.L. Endometrial cancer incidence in relation to electric blanket use. *American Journal of Epidemiology* **2002**, *156*, 262-267.
22. Tynes, T.; Haldorsen, T. Residential and occupational exposure to 50 hz magnetic fields and hematological cancers in norway. *Cancer Causes & Control* **2003**, *14*, 715-720.
23. Levallois, P.; Dumont, M.; Touitou, Y.; Gingras, S.; Masse, B.; Gauvin, D.; Kroger, E.; Bourdages, M.; Douville, P. Effects of electric and magnetic fields from high-power lines on female urinary excretion of 6-sulfatoxymelatonin. *American Journal of Epidemiology* **2001**, *154*, 601-609.
24. Schuz, J.; Dasenbrock, C.; Ravazzani, P.; Roosli, M.; Schar, P.; Bounds, P.L.; Erdmann, F.; Borkhardt, A.; Cobaleda, C.; Fedrowitz, M., *et al.* Extremely low-frequency magnetic fields and risk of childhood leukemia: A risk assessment by the arimmora consortium. *Bioelectromagnetics* **2016**, *37*, 183-189.
25. Verkasalo, P.K. Magnetic fields and leukemia - risk for adults living close to power lines - introduction. *Scandinavian Journal of Work Environment & Health* **1996**, *22*, 7-&.
26. Malagoli, C.; Fabbi, S.; Teggi, S.; Calzari, M.; Poli, M.; Ballotti, E.; Notari, B.; Bruni, M.; Palazzi, G.; Paolucci, P., *et al.* Risk of hematological malignancies associated with magnetic fields exposure from power lines: A case-control study in two municipalities of northern italy. *Environmental Health* **2010**, *9*.
27. Hakim, A.S.B.; Abd Rahman, N.B.; Mokhtar, M.Z.; Bin Said, I.; Hussain, H.; Ieee. *Elf - emf correlation study on distance from overhead transmission lines and acute leukemia among children in klang valley, malaysia*. 2014; p 710-714.
28. Nunez-Enriquez, J.C.; Correa-Correa, V.; Flores-Lujano, J.; Perez-Saldivar, M.L.; Jimenez-Hernandez, E.; Martin-Trejo, J.A.; Espinoza-Hernandez, L.E.; Medina-Sanson, A.; Cardenas-Cardos, R.; Flores-Villegas, L.V., *et al.* Extremely low-frequency magnetic fields and the risk of childhood b-lineage acute lymphoblastic leukemia in a city with high incidence of leukemia and elevated exposure to elf magnetic fields. *Bioelectromagnetics* **2020**, *41*, 581-597.
29. Li, C.Y.; Lin, R.S.; Sung, F.C. Elevated residential exposure to power frequency magnetic field associated with greater average age at diagnosis for patients with brain tumors. *Bioelectromagnetics* **2003**, *24*, 218-221.
30. Elliott, P.; Shaddick, G.; Douglass, M.; de Hoogh, K.; Briggs, D.J.; Toledano, M.B. Adult cancers near high-voltage overhead power lines. *Epidemiology* **2013**, *24*, 184-190.

31. Henshaw, D.L. Does our electricity distribution system pose a serious risk to public health? *Medical Hypotheses* **2002**, *59*, 39-51.
32. Carpenter, D.O. Extremely low frequency electromagnetic fields and cancer: How source of funding affects results. *Environ Res* **2019**, *178*, 108688.
33. Redmayne, M. A proposed explanation for thunderstorm asthma and leukemia risk near high-voltage power lines: A supported hypothesis. *Electromagnetic Biology and Medicine* **2018**, *37*, 57-65.
34. Karipidis, K.K. Survey of residential power-frequency magnetic fields in melbourne, australia. *Radiation Protection Dosimetry* **2015**, *163*, 81-91.
35. Wünsch, V.; Pelissari, D.M.; Barbieri, F.E.; Sant'Anna, L.; de Oliveira, C.T.; de Mata, J.F.; Tone, L.G.; Lee, M.L.D.; de Andrea, M.L.M.; Bruniera, P., *et al.* Exposure to magnetic fields and childhood acute lymphocytic leukemia in sao paulo, brazil. *Cancer Epidemiology* **2011**, *35*, 534-539.
36. Amoon, A.T.; Oksuzyan, S.; Crespi, C.M.; Arah, O.A.; Cockburn, M.; Vergara, X.; Kheifets, L. Residential mobility and childhood leukemia. *Environmental Research* **2018**, *164*, 459-466.
37. Jirik, V.; Pekarek, L.; Janout, V.; Tomaskova, H. Association between childhood leukaemia and exposure to power-frequency magnetic fields in middle europe. *Biomedical and Environmental Sciences* **2012**, *25*, 597-601.
38. Jirik, V.; Pekarek, L.; Janout, V. Assessment of population exposure to extremely low frequency magnetic fields and its possible childhood health risk in the czech republic. *Indoor and Built Environment* **2011**, *20*, 362-368.
39. Pedersen, C.; Johansen, C.; Schuz, J.; Olsen, J.H.; Raaschou-Nielsen, O. Residential exposure to extremely low-frequency magnetic fields and risk of childhood leukaemia, cns tumour and lymphoma in denmark. *British Journal of Cancer* **2015**, *113*, 1370-1374.
40. Pedersen, C.; Brauner, E.V.; Rod, N.H.; Albieri, V.; Andersen, C.E.; Ulbak, K.; Hertel, O.; Johansen, C.; Schuz, J.; Raaschou-Nielsen, O. Distance to high-voltage power lines and risk of childhood leukemia - an analysis of confounding by and interaction with other potential risk factors. *Plos One* **2014**, *9*.
41. Bunch, K.J.; Swanson, J.; Vincent, T.J.; Murphy, M.F.G. Magnetic fields and childhood cancer: An epidemiological investigation of the effects of high-voltage underground cables. *J. Radiol. Prot.* **2015**, *35*, 695-705.
42. Juutilainen, J.; Herrala, M.; Luukkonen, J.; Naarala, J.; Hore, P.J. Magnetocarcinogenesis: Is there a mechanism for carcinogenic effects of weak magnetic fields? *Proc Biol Sci* **2018**, *285*.
43. Schuz, J.; Grigat, J.P.; Brinkmann, K.; Michaelis, J. Residential magnetic fields as a risk factor for childhood acute leukaemia: Results from a german population-based case-control study. *International Journal of Cancer* **2001**, *91*, 728-735.
44. Kokate, P.A.; Mishra, A.K.; Lokhande, S.K.; Bodhe, G.L. Extremely low frequency electromagnetic field (elf-emf) and childhood leukemia (cl) near transmission lines: A review. *Advanced Electromagnetics* **2016**, *5*, 30-40.
45. Tafrishi, R.; Seyfari, B.; Rahimi, R.; Chaichi, Z.; Tarazjani, A.D.; Marvi, N.; Maazallahi, M.; Dolatian, Z.; Ashrafinia, F. Modifiable and non-modifiable factors affecting the risk of childhood leukemia: An overview of meta-analysis. *International Journal of Pediatrics-Mashhad* **2021**, *9*, 13243-13248.
46. Salvan, A.; Ranucci, A.; Lagorio, S.; Magnani, C.; Grp, S.R. Childhood leukemia and 50 hz magnetic fields: Findings from the italian setil case-control study. *International Journal of Environmental Research and Public Health* **2015**, *12*, 2184-2204.

47. Liorni, I.; Parazzini, M.; Struchen, B.; Fiocchi, S.; Roosli, M.; Ravazzani, P. Children's personal exposure measurements to extremely low frequency magnetic fields in italy. *International Journal of Environmental Research and Public Health* **2016**, *13*.
48. Soderberg, K.C.; Naumburg, E.; Anger, G.; Cnattingius, S.; Ekbom, A.; Feychting, M. Childhood leukemia and magnetic fields in infant incubators. *Epidemiology* **2002**, *13*, 45-49.
49. Kelfkens, G.; Pruppers, M. Magnetic fields and childhood leukemia; science and policy in the netherlands. In *Embec & nbc 2017*, Eskola, H.; Vaisanen, O.; Viik, J.; Hyttinen, J., Eds. 2018; Vol. 65, pp 498-501.
50. Crespi, C.M.; Vergara, X.P.; Hooper, C.; Oksuzyan, S.; Wu, S.; Cockburn, M.; Kheifets, L. Childhood leukaemia and distance from power lines in california: A population-based case-control study. *British Journal of Cancer* **2016**, *115*, 122-128.
51. Peck, S.C.; Kavet, R. Research strategies for magnetic fields and cancer. *Risk Analysis* **2005**, *25*, 179-188.
52. Does, M.; Scelo, G.; Metayer, C.; Selvin, S.; Kavet, R.; Buffler, P. Exposure to electrical contact currents and the risk of childhood leukemia. *Radiation Research* **2011**, *175*, 390-396.
53. Crespi, C.M.; Swanson, J.; Vergara, X.P.; Kheifets, L. Childhood leukemia risk in the california power line study: Magnetic fields versus distance from power lines. *Environmental Research* **2019**, *171*, 530-535.
54. Kheifets, L.; Crespi, C.M.; Hooper, C.; Cockburn, M.; Amoon, A.T.; Vergara, X.P. Residential magnetic fields exposure and childhood leukemia: A population-based case-control study in california. *Cancer Causes & Control* **2017**, *28*, 1117-1123.
55. Slusky, D.A.; Does, M.; Metayer, C.; Mezei, G.; Selvin, S.; Buffler, P.A. Potential role of selection bias in the association between childhood leukemia and residential magnetic fields exposure: A population-based assessment. *Cancer Epidemiology* **2014**, *38*, 307-313.
56. Swanson, J.; Kheifets, L. Could the geomagnetic field be an effect modifier for studies of power-frequency magnetic fields and childhood leukaemia? *J. Radiol. Prot.* **2012**, *32*, 413-418.
57. Kheifets, L.; Crespi, C.M.; Hooper, C.; Oksuzyan, S.; Cockburn, M.; Ly, T.; Mezei, G. Epidemiologic study of residential proximity to transmission lines and childhood cancer in california: Description of design, epidemiologic methods and study population. *Journal of Exposure Science and Environmental Epidemiology* **2015**, *25*, 45-52.
58. Zhao, L.Y.; Liu, X.D.; Wang, C.P.; Yan, K.K.; Lin, X.J.; Li, S.; Bao, H.H.; Liu, X. Magnetic fields exposure and childhood leukemia risk: A meta-analysis based on 11,699 cases and 13,194 controls. *Leukemia Research* **2014**, *38*, 269-274.
59. Ghahremani, S.; Shiroudbakhshi, K.; Kordasiabi, A.H.S.; FiroozBakht, M.; Hosseinzadegan, M.; Ashrafinia, F.; Rahafard, S. Exposure to magnetic fields and childhood leukemia: An overview of meta-analysis. *International Journal of Pediatrics-Mashhad* **2020**, *8*, 11361-11365.
60. Sermage-Faure, C.; Laurier, D.; Goujon-Bellec, S.; Chartier, M.; Guyot-Goubin, A.; Rudant, J.; Hemon, D.; Clavel, J. Childhood leukemia around french nuclear power plantsuthe geocap study, 2002-2007. *International Journal of Cancer* **2012**, *131*, E769-E780.
61. Sermage-Faure, C.; Demoury, C.; Rudant, J.; Goujon-Bellec, S.; Guyot-Goubin, A.; Deschamps, F.; Hemon, D.; Clavel, J. Childhood leukaemia close to high-voltage power lines - the geocap study, 2002-2007. *British Journal of Cancer* **2013**, *108*, 1899-1906.
62. Schuz, J.; Grell, K.; Kinsey, S.; Linet, M.S.; Link, M.P.; Mezei, G.; Pollock, B.H.; Roman, E.; Zhang, Y.; McBride, M.L., *et al.* Extremely low-frequency magnetic fields and survival from childhood acute lymphoblastic leukemia: An international follow-up study. *Blood Cancer Journal* **2012**, *2*.

63. Gudina, M.V.; Borodin, A.S.; Tuzhilkin, D.A.; Pikalova, L.V. Malignant neoplasms on territories with different levels of magnetic fields of industrial frequency. In *24th international symposium on atmospheric and ocean optics: Atmospheric physics*, Matvienko, G.G.; Romanovskii, O.A., Eds. 2018; Vol. 10833.
64. Hallberg, O.; Johansson, O. Melanoma incidence and frequency modulation (fm) broadcasting. *Archives of Environmental Health* **2002**, *57*, 32-40.
65. Selmaoui, B.; Aymard, N.; Lambrozo, J.; Touitou, Y. Evaluation of the nocturnal levels of urinary biogenic amines in men exposed overnight to 50-hz magnetic field. *Life Sciences* **2003**, *73*, 3073-3082.
