# Supplementary material for: Magnetic Fields and Cancer: Epidemiology, Cellular Biology, and Theranostics
Source: Int J Mol Sci. 2022 Jan 25;23(3):1339. doi: 10.3390/ijms23031339 (PMC8835851; doi:10.3390/ijms23031339)
Supplement: Supplementary file 1 [file ijms-23-01339-s001.zip › Supplementary Tables S1-S5/Supplementary Table S2.pdf]

International Journal of
Molecular Sciences

Supplementary

Magnetic Fields and Cancer

Massimo E. Maffei [1,*]

[1] Dept. Life Sciences and Systems Biology, University of Turin, Via Quarello 15/a, 10135 Turin, Italy; massimo.maffei@unito.it

* Correspondence: massimo.maffei@unito.it; Tel.: +39011 6705967

Supplementary Table S2.

Relationship between magnetic fields and cancer in occupational MF exposure epidemiologic studies

Type of cancer	Study design and occupation	Range of MFs	Location	Conclusions	Ref.
brain cancer	meta-analysis of occupational studies	> 0.2 μT	worldwide	Little or no association between electric and magnetic field exposure and brain cancer	[1]
brain cancer	risks experienced by employees of the former Central Electricity Generating Board of England and Wales	0>μT>20 per year	U.K.	No evidence to support the hypothesis that exposure to magnetic fields is a risk factor for gliomas	[2]
brain tumour leukemia and	Swiss railway workers	1-21 μT annual expo-sure	Switzerland	Some evidence of an exposure-response association was found for myeloid leukemia and Hodgkin's disease, but not for other hematopoietic and lym-phatic malignancies and brain tumors	[3]
brain cancer	exposure to ELMFs	0.11-0.62 μT	Australia, Canada, France, Ger-many, Israel, New Zea-land, and the United Kingdom	Occupational ELF exposure may play a role in the later stages (promotion and progression) of brain tumorigenesis	[4]
brain cancer leukemia or	Case-cohort analysis of brain cancer and leukemia in electric utility workers	0.11-1.21 μT	U.S.A.	Magnetic field exposure remained unrelated to leukemia mortality and posi-tively associated with brain cancer mortality based on both cumulative and average magnetic field indices	[5]
breast cancer	exposure to extremely low fre-quency MFs among women	60 Hz; 0.022-3.636 μT	U.S.A., North Caro-lina	It is questionable whether exposure to power frequency magnetic fields is the cause of the increased breast cancer risk	[6]
breast cancer	Occupational exposure to MFs among men	0.3 > μT > 0.6	Canada	Limited support for the hypothesis that exposure to MF increases the risk breast cancer in men	[7]
breast cancer	exposure to MF	n.a.	n.a.	A spurious negative association between magnetic fields and breast cancer could occur if the exposed group included a large number of immigrants from Asia and Africa	[8]

Type of cancer	Study design and occupation	Range of MFs	Location	Conclusions	Ref.
breast cancer	exposure to commercial sewing machines at garment facilities	15-3000 Hz 0.04-3.7 μT	U.S.A.	The MF environment of sewing machine represents a work environment similar to other work or home environments	[9]
breast cancer	melatonin hypothesis for workers in light metal reduction plants and operators of medical MRT in hospitals	static MF 2-7 mT	Norway	No association between a single nocturnal exposure to a static MF and excretion of aMT6s in urine	[10]
breast cancer	melatonin hypothesis women occupationally exposed to extremely low-frequency MFs	0.1-1.0 μT	Finland	Supports the hypothesis that daytime occupational exposure to MF enhances the effects of nighttime light exposure on melatonin production	[11]
breast cancer leukemia malignant melanoma of the skin nervous system tumors non-Hodgkin lymphoma	possible carcinogenicity of electromagnetic fields and the scientific basis for environmen-occupational standard setting	n.a.	n.a.	An increased risk of leukemia in children and the existence of, or distance to, power lines in the vicinity of their residence, an increased risk of chronic lymphatic leukemia and occupational exposure to low frequency electromagnetic fields and an increased risk of breast cancer, malignant melanoma of the skin, nervous system tumors, non-Hodgkin lymphoma, acute lymphatic leukemia or acute myeloid leukemia and certain occupations	[12]
cancer in children	parent preconceptionally exposure at work to ELF-MFs	> 0.2 μT	Germany	Risk is not linked to preconceptional parental ELF-MF exposure	[13]
glioblastoma multiforme	exposure to MFs in men	0.3-0.6 μT	Canada	Occupational MF exposure increases the risk of glioblastoma multiforme	[14]
hematological cancers	residence situated in a broad corridor around a high-voltage power line	0.05 - 0.20 μT	Norway	Occupational exposure showed no significant association to exposure for any site. Conclusions: Some elevated odds ratios were observed in the present study, but the results are based on small numbers and no firm conclusions can be drawn	[15]
kidney pituitary gland biliary passages liver	Four exposure groups were used by stratifying on mean workday ELF-MF exposure,	0.163-0.503 μT	Sweden	Men in the very high exposure group showed an increased incidence of tumors of the kidney, pituitary gland, and biliary passages and liver; for these cancer sites an exposure-response relation was indicated. Women in the very high exposure group showed an increased incidence of astrocytoma I-IV, with	[16]

Type of cancer	Study design and occupation	Range of MFs	Location	Conclusions	Ref.
astrocytoma I-IV myeloma	using the lowest exposure group as reference			a clear exposure-response pattern. An association was suggested in the high exposure group only, for cancer of the corpus uteri and multiple myeloma	
leukemia				This large population based case-control study found little evidence to support an association between occupational exposure to electromagnetic fields and acute leukaemia	[17]
leukemia	MF exposure of staff working in the offices located above or close to transformer stations and electric enclosures	0.3-6.8 μT	Turkey	Most of the staff (83%) is under risk based on epidemiological studies that reported a statistically significant association between risk of leukemia and MF > 0.2 μT	[18]
leukemia	occupational exposure to ELFMFs	n.a.	n.a.	The lack of consistency regarding the type of leukemia associated with magnetic field exposure might be explained by differences between the study designs or the populations studied, but based on the existing evidence, no firm conclusions can be drawn	[19]
leukemia	employees of the former Central Electricity Generating Board	0.01-3.34 μT	England and Wales	There are no discernible excess risks of leukemia as a consequence of occupational exposure to magnetic fields in United Kingdom electricity generation and transmission workers	[20]
leukemia acute	subjects classified as having worked in one or more of the "electrical occupations"	0.1-3.7 μT	New Zealand	A dose-response effect was also found, indicating that acute leukemia risk was related to historical and current magnetic field exposures in an occupational context	[21]
leukemia acute myeloid	association between occupational exposure to ELF-MF or electrical shocks	n.a.	Finland, Iceland, Norway, Sweden	Results do not support an association between occupational ELF-MF or electric shock exposure and AML	[22]
leukemia acute myeloid (AML) chronic myeloid leukaemia lymphoid leukemia diffuse large B-cell lymphomas	job exposure matrix for occupations	0.11-0.52 μT	Switzerland	No convincing evidence for an increased risk of death from a range of hematolymphopoi-etic cancers in workers exposed to high or medium levels of ELF MFs.<br>An increased risk of acute myeloid leukemia is observed in workers exposed to high levels for a longer duration	[23]

Type of cancer	Study design and occupation	Range of MFs	Location	Conclusions	Ref.
follicular lymphoma Waldenstrom's macroglobulinemia multiple myeloma lymphoma Hodgkin					
leukemia childhood	parental occupational exposure to extremely low-frequency magnetic fields (ELF-MF)	n.a.	China	No association between parental occupational ELF-MF exposure and childhood leukemia risk	[24]
leukemia childhood	parental occupational exposure to extremely low-frequency magnetic fields (ELF-MF)	≥2 μT	multinational	No associations between parental occupational ELF-MF exposure and childhood leukemia	[25]
leukemia lymphatic	cytogenetic analysis on cultured peripheral lymphocytes of railway employees	16 2/3 Hz; 10-100 μT	Sweden	Exposure to MF at mean intensities of 2-15 μT can induce chromosomal damage	[26]
leukemia lymphoblastic	ELF magnetic field exposures for a wide range of jobs commonly held by women	0.66-0.70 μT	Canada	New information on expected levels of exposure in a wide range of jobs commonly held by women	[27]
lymphoma non-Hodgkin (NHL)	exposure to occupational MFs	50/60 Hz; 0.26-2.35 μT	Australia	Weak support for the hypothesis that MFs increase the risk of NHL	[28]
neuroblastoma	incidence in offspring of parental occupational exposures to EMF and radiation	> 0.4 μT	U.S.A and Canada	Scant supportive evidence of strong associations between parental exposures in electro-magnetic spectrum and neuroblastoma in offspring	[29]
neuroblastoma Nervous system tumor	meta-analysis of case-control and cohort studies	n.a.	worldwide	Limited evidence for the association between maternal occupational exposure to ELF-MF and increased risk of childhood CNS tumors	[30]
neuroma acoustic	association of occupations with ELF-EMF exposure	8.52 μT/year	Sweden	Occupational ELF-EMF was not associated with an increased risk for acoustic neuroma	[31]

Type of cancer	Study design and occupation	Range of MFs	Location	Conclusions	Ref.
thyroid cancer	ionizing radiation and extremely low-frequency MF in relation to occupational exposure	0.15-0.35 μT	Sweden	No support for the possibility of occupational exposure to ELFMF being a risk factor for the development of thyroid cancer	[32]

References

1. Kheifets, L.I. Electric and magnetic field exposure and brain cancer: A review. *Bioelectromagnetics* **2001**, S120-S131.
2. Sorahan, T. Magnetic fields and brain tumour risks in uk electricity supply workers. *Occupational Medicine-Oxford* **2014**, *64*, 157-165.
3. Roosli, M.; Lortscher, M.; Egger, M.; Pfluger, D.; Schreier, N.; Lortscher, E.; Locher, P.; Spoerri, A.; Minder, C. Leukaemia, brain tumours and exposure to extremely low frequency magnetic fields: Cohort study of swiss railway employees. *Occupational and Environmental Medicine* **2007**, *64*, 553-559.
4. Turner, M.C.; Benke, G.; Bowman, J.D.; Figuerola, J.; Fleming, S.; Hours, M.; Kincl, L.; Krewski, D.; McLean, D.; Parent, M.E., *et al.* Occupational exposure to extremely low-frequency magnetic fields and brain tumor risks in the interocc study. *Cancer Epidemiology Biomarkers & Prevention* **2014**, *23*, 1863-1872.
5. Savitz, D.A.; Cai, J.W.; van Wijngaarden, E.; Loomis, D.; Mihlan, G.; Dufort, V.; Kleckner, R.C.; Nylander-French, L.; Kromhout, H.; Zhou, H.B. Case-cohort analysis of brain cancer and leukemia in electric utility workers using a refined magnetic field job-exposure matrix. *American Journal of Industrial Medicine* **2000**, *38*, 417-425.
6. McCurdy, A.L.; Wijnberg, L.; Loomis, D.; Savitz, D.; Nylander-French, L.A. Exposure to extremely low frequency magnetic fields among working women and homemakers. *Annals of Occupational Hygiene* **2001**, *45*, 643-650.
7. Grundy, A.; Harris, S.A.; Demers, P.A.; Johnson, K.C.; Agnew, D.A.; Villeneuve, P.J.; Canadian Canc Registries, E. Occupational exposure to magnetic fields and breast cancer among canadian men. *Cancer Medicine* **2016**, *5*, 586-596.
8. Goodman, M.; Kelsh, M.; Ebi, K.; Iannuzzi, J.; Langholz, B. Evaluation of potential confounders in planning a study of occupational magnetic field exposure and female breast cancer. *Epidemiology* **2002**, *13*, 50-58.
9. Kelsh, M.A.; Bracken, T.D.; Sahl, J.D.; Shum, M.; Ebi, K.L. Occupational magnetic field exposures of garment workers: Results of personal and survey measurements. *Bioelectromagnetics* **2003**, *24*, 316-326.
10. Haugsdal, B.; Tynes, T.; Rotnes, J.S.; Griffiths, D. A single nocturnal exposure to 2-7 millitesla static magnetic fields does not inhibit the excretion of 6-sulfatoxymelatonin in healthy young men. *Bioelectromagnetics* **2001**, *22*, 1-6.
11. Juutilainen, J.; Kumlin, T. Occupational magnetic field exposure and melatonin: Interaction with light-at-night. *Bioelectromagnetics* **2006**, *27*, 423-426.
12. Hardell, L.; Holmberg, B.; Malker, H.; Paulsson, L.E. Exposure to extremely-low-frequency electromagnetic-fields and the risk of malignant diseases - an evaluation of epidemiologic and experimental findings. *European Journal of Cancer Prevention* **1995**, *4*, 3-107.
13. Hug, K.; Grize, L.; Seidler, A.; Kaatsch, P.; Schuz, J. Parental occupational exposure to extremely low frequency magnetic fields and childhood cancer: A german case-control study. *American Journal of Epidemiology* **2010**, *171*, 27-35.
14. Villeneuve, P.J.; Agnew, D.A.; Johnson, K.C.; Mao, Y.; Canadian Cancer Registries, E. Brain cancer and occupational exposure to magnetic fields among men: Results from a canadian population-based case-control study. *International Journal of Epidemiology* **2002**, *31*, 210-217.
15. Tynes, T.; Haldorsen, T. Residential and occupational exposure to 50 hz magnetic fields and hematological cancers in norway. *Cancer Causes & Control* **2003**, *14*, 715-720.

16. Hakansson, N.; Floderus, B.; Gustavsson, P.; Johansen, C.; Olsen, J.H. Cancer incidence and magnetic field exposure in industries using resistance welding in sweden. *Occupational and Environmental Medicine* **2002**, *59*, 481-486.
17. Willett, E.V.; McKinney, P.A.; Fear, N.T.; Cartwright, R.A.; Roman, E. Occupational exposure to electromagnetic fields and acute leukaemia: Analysis of a case-control study. *Occupational and Environmental Medicine* **2003**, *60*, 577-583.
18. Cam, S.T.; Firlarer, A.; Ozden, S.; Canseven, A.G.; Seyhan, N. Occupational exposure to magnetic fields from transformer stations and electric enclosures in turkey. *Electromagnetic Biology and Medicine* **2011**, *30*, 74-79.
19. Feychting, M. Occupational exposure to electromagnetic fields and adult leukaemia: A review of the epidemiological evidence. *Radiation and Environmental Biophysics* **1996**, *35*, 237-242.
20. Harrington, J.M.; Nichols, L.; Sorahan, T.; van Tongeren, M. Leukaemia mortality in relation to magnetic field exposure: Findings from a study of united kingdom electricity generation and transmission workers, 1973-97. *Occupational and Environmental Medicine* **2001**, *58*, 307-314.
21. Bethwaite, P.; Cook, A.; Kennedy, J.; Pearce, N. Acute leukemia in electrical workers: A new zealand case-control study. *Cancer Causes & Control* **2001**, *12*, 683-689.
22. Talibov, M.; Guxens, M.; Pukkala, E.; Huss, A.; Kromhout, H.; Slottje, P.; Martinsen, J.I.; Kjaerheim, K.; Sparen, P.; Weiderpass, E., *et al.* Occupational exposure to extremely low-frequency magnetic fields and electrical shocks and acute myeloid leukemia in four nordic countries. *Cancer Causes & Control* **2015**, *26*, 1079-1085.
23. Huss, A.; Spoerri, A.; Egger, M.; Kromhout, H.; Vermeulen, R.; Swiss Natl, C. Occupational extremely low frequency magnetic fields (elf-mf) exposure and hematolymphopoietic cancers - swiss national cohort analysis and updated meta-analysis. *Environmental Research* **2018**, *164*, 467-474.
24. Su, L.L.; Fei, Y.; Wei, X.X.; Guo, J.; Jiang, X.G.; Lu, L.Q.; Chen, G.D. Associations of parental occupational exposure to extremely low-frequency magnetic fields with childhood leukemia risk. *Leukemia & Lymphoma* **2016**, *57*, 2855-2862.
25. Talibov, M.; Olsson, A.; Bailey, H.; Erdmann, F.; Metayer, C.; Magnani, C.; Petridou, E.; Auvinen, A.; Spector, L.; Clavel, J., *et al.* Parental occupational exposure to low-frequency magnetic fields and risk of leukaemia in the offspring: Findings from the childhood leukaemia international consortium (clic). *Occupational and Environmental Medicine* **2019**, *76*, 746-753.
26. Nordenson, I.; Mild, K.H.; Jarventaus, H.; Hirvonen, A.; Sandstrom, M.; Wilen, J.; Blix, N.; Norppa, H. Chromosomal aberrations in peripheral lymphocytes of train engine drivers. *Bioelectromagnetics* **2001**, *22*, 306-315.
27. Deadman, J.E.; Infante-Rivard, C. Individual estimation of exposures to extremely low frequency magnetic fields in jobs commonly held by women. *American Journal of Epidemiology* **2002**, *155*, 368-378.
28. Karipidis, K.; Benke, G.; Sim, M.; Fritschi, L.; Yost, M.; Armstrong, B.; Hughes, A.M.; Grulich, A.; Vajdic, C.M.; Kaldor, J.M., *et al.* Occupational exposure to power frequency magnetic fields and risk of non-hodgkin lymphoma. *Occupational and Environmental Medicine* **2007**, *64*, 25-29.
29. De Roos, A.J.; Teschke, K.; Savitz, D.A.; Poole, C.; Grufferman, S.; Pollock, B.H.; Olshan, A.F. Parental occupational exposures to electromagnetic fields and radiation and the incidence of neuroblastoma in offspring. *Epidemiology* **2001**, *12*, 508-517.
30. Su, L.L.; Zhao, C.N.; Jin, Y.M.; Lei, Y.; Lu, L.Q.; Chen, G.D. Association between parental occupational exposure to extremely low frequency magnetic fields and childhood nervous system tumors risk: A meta-analysis. *Science of the Total Environment* **2018**, *642*, 1406-1414.

31. Carlberg, M.; Koppel, T.; Ahonen, M.; Hardell, L. Case-control study on occupational exposure to extremely low-frequency electromagnetic fields and the association with acoustic neuroma. *Environmental Research* **2020**, *187*.
32. Lope, V.; Perez-Gomez, B.; Aragones, N.; Lopez-Abentes, G.; Gustavsson, P.; Floderus, B.; Dosemeci, M.; Silva, A.; Pollan, M. Occupational exposure to ionizing radiation and electromagnetic fields in relation to the risk of thyroid cancer in sweden. *Scandinavian Journal of Work Environment & Health* **2006**, *32*, 276-284.
