# Supplementary material for: Magnetic Fields and Cancer: Epidemiology, Cellular Biology, and Theranostics
Source: Int J Mol Sci. 2022 Jan 25;23(3):1339. doi: 10.3390/ijms23031339 (PMC8835851; doi:10.3390/ijms23031339)
Supplement: Supplementary file 1 [file ijms-23-01339-s001.zip › Supplementary Tables S1-S5/Supplementary Table S3.pdf]

International Journal of
Molecular Sciences

MDPI

Supplementary

Magnetic Fields and Cancer

Massimo E. Maffei [1,*]

[1] Dept. Life Sciences and Systems Biology, University of Turin, Via Quarello 15/a, 10135 Turin, Italy; massimo.maffei@unito.it
* Correspondence: massimo.maffei@unito.it; Tel.: +39011 6705967

Supplementary Table S3.

Studies on human cells (In vitro cellular studies and Cell-free systems)

Type of cell	response to MF	Range of MFs	Duration	Methods/Cell types	Results	Ref.
amniotic (FL) human cells	MF interference with receptor clustering and phosphorylation	18.5 µT	2 h	Confocal microscopy analysis, Phosphorylation measurement of EGF receptor,	Membrane receptors could be one of the main targets where extremely-low frequency (ELF) MF interacts with cells, and the intensity threshold, in the case of EGF receptors, is between 0.05 and 0.1 mT	[1]
blood cells human	genotoxic effects of ELFMFs,	1 mT	48 h	Comet assay, sister chromatid exchange, chromosome aberrations, nuclear division and proliferation indexes.	Data obtained from the combined exposure to ELFMFs and ionizing radiation do not suggest any synergistic or antagonistic effect	[2]
blood cells human	Genotoxicity in Human Blood Cells Exposed to MFs	980-1020 µT	2 h	cytogenetic assay, micro-nucleus, sister chromatid exchanges and comet assays	A slight but significant decrease of cell proliferation was evident in treated samples	[3]
breast cancer cell	comparison of the effect of ELFMF on healthy dermal fibroblasts and breast cancer cells	50 Hz 1 mT	1, 3, 5 h	Human, neonatal, healthy primary dermal fibroblast and MCF7 cell lines, Cell survival and death.	ELF-MF reduces the viability of healthy fibroblasts and has no therapeutic effects on breast cancer cells	[4]
breast cancer cells	Combination of MF and UVC radiation	25 Hz, 0.75 mT	30 min	MCF-7 breast cancer cells exposed to MF and UVC (from 6.6 J/m^2 to 59.4 J/m^2), viability measured by the neutral red stain cytotoxicity test	PMF in combination with UVC have the ability to augment the cell killing effects of UVC radiation. In addition, the effects appear to be greater when PMF and UVC are applied at the same time	[5]
Caco 2 cells	Effects on Proteasomal Systems	50 Hz 1 mT	24-72 h	Cell Viability, 20S Proteasome Activities, Immunoblotting using anti-$p27^{kip-1}$ human and mouse monoclonal antibody	EMFs affect the proteasome functionality inducing an increase in its proteolytic activity.	[6]

Type of cell	response to MF	Range of MFs	Duration	Methods/Cell types	Results	Ref.
cancer cells human	effect on cell growth and cell proliferation in human cancer cell lines	50 -60 Hz 2, 20, 100, 500 μT	5 days	HL-60 (promyelocytic; CCL-240), K-562 (chronic myelogenous leukemia), MCF-7 (breast carcinoma), A-375 (malignant melanoma), H4 (glioma), cell proliferation, DNA synthesis and quantification	No support for cancer promoting or progressing effects of commercial frequency magnetic field exposure and did not indicate any mechanisms that would explain the reported association between magnetic field and carcinogenesis	[7]
fibroblast lung HeLa (human cervical carcinoma)	magnetic field induces DNA double-strand breaks and apoptosis in human cells	0- 60 Hz 6 mT	30 min every 24 h for 3 days	IMR90 (human lung fibroblast) primary cells and HeLa cells, Western blot analysis phospho-Chk2, phospho-p38, phospho-JNK, caspase-3, cleaved caspase-3, PARP and actin; Immunofluorescence microscopy	Repetitive exposure to MF with extremely low frequency can induce DNA DSBs and apoptosis through p38 activation	[8]
fibroblasts	Effects on structural morphology and proliferation of human cells in vitro	20, 500 μT	48, 72 h	Human MCF-7 cells; fibroblasts; lmmunofluorescence detection of BrdU labeling, cytokeratin; dual-parameter flow cytometry analysis; Detection of actin with fluorescently-labeled phalloidin and immunodetection of the nuclear antigen Ki-67 in human fibroblasts	Continuous exposure to magnetic fields does not result in any appreciable effect in both normal and tumor cells in vitro	[9]
glioma human cells	Changes in gene and protein expression	60-Hz 1.2 μT	3 h	human glioma SF767 cells gene expression (oncogene microarray) protein expression (two-dimensional gel electrophoresis), protein identification (peptide fingerprint, mass spectrometry)	limited but complicated response in the glioma cells to the magnetic field treatment	[10]

Type of cell	response to MF	Range of MFs	Duration	Methods/Cell types	Results	Ref.
Glioma Human Cells	Protein expression in MF-treated cells	1.2 μT	3 h	SF767 cells, proteins were resolved by 2D-PAGE, Peptide Mass Fingerprinting, MALDI-MS	A cytoskeletal intermediate filament protein increased following a low-level magnetic field.	[11]
HeLa cells	Effects on DNA and RNA Synthesis	60 Hz 0.25-0.5 T	0-60 min	DNA and RNA Synthesis, DNA Mismatch Repair	The rate and fidelity of DNA polymerase catalyzed DNA synthesis, as well as of RNA polymerase catalyzed RNA synthesis, were not statistically significantly affected	[12]
HL60 cells	Effects on Gene Expression of Cytokine Receptors TNFR, IL-6Rα, TGFβR1	0.1, 0.8 mT	72 h	Total RNA Extraction, Ribonuclease Protection Assay,	MF exposure at 0.1 and 0.8 mT for 72 h increased TNFR p75 and IL-6Rα mRNA ex-pression in HL60 cells	[13]
keratinocyte cell line (MNT-1-HaCaT) melanoma cells Murine and Human (B16F10 and MNT-1),	Skin pigmentation due to smartphone use	(LTE, 1.762 GHz) and 5G (28 GHz)	4 h per day	Cell Viability, qRT-PCR (tyrosinase and tyrosinase-related protein 1 (TRP-1)), Human Epidermal Skin Model,	Exposure may not affect melanin synthesis or skin pigmentation under normal smartphone use condition	[14]
keratinocyte human	ELF-EMF use in regenerative medicine for the treatment of skin injuries	1 H- 50 Hz, 25-200 μT	4 h and 24 h	Reactive oxygen species (ROS) production (glutathione content, antioxidant defense activity, and lipid peroxidation markers)	ELF-EMF induces a slight oxidative stress that does not overwhelm the metabolic capacity of the cells or have a cytotoxic effect	[15]
leucocytes	Effects of sinusoidal magnetic field on adherence inhibition	50 Hz. 0, 1, 10 mT	60 min	separation of CD4Ly cells (helper T cells), Leucocyte Adherence Inhibition test,	Adherence of leucocytes taken from cancer patients is strongly increased. The 1 mT magnetic field has stronger effect than the 10 mT field	[16]
leukemia human promylocytic human Burkitt lymphoma	decrease in apoptosis susceptibility in DNA repair rates	60 Hz 0.15 mT	4, 12, 24 h	human cancer cell lines HL-60, Raji and HL-60R, Cell proliferation assays, exposure to heat shock, neutral comet and repair assays,	The effects of EMF exposure are dependent on the duration of exposure. EMF exposure may contribute to cancer formation.	[17]

Type of cell	response to MF	Range of MFs	Duration	Methods/Cell types	Results	Ref.
leukocyte	Effects on adherence inhibition	0.5 mT	60 min	leukocyte adherence inhibition assay; cell adhesion/number of non-adherent cells	Adherence of T lymphocytes from healthy humans and from cancer patients before and after medical treatment is enhanced after exposure to MFs indicating an effect on immunity function.	[18]
leukocytes	Effects on Death of Proliferating Peripheral Blood Mononuclear Cells	50 Hz 45 mT	3 h	Isolation of Peripheral Blood Mononuclear Cells (PBMCs), Cell Death Evaluation	A low-frequency pulsing electromagnetic field induces cell death in native proliferating cells isolated from Acute myeloid leukemia (AML) patients. The increased vulnerability of proliferating PBMCs may be potentially applied in the therapy of AML	[19]
lymphoblastoid cell	MF sensitivity	50 Hz 60 μT	0, 24, 48, 72 h	Electron microscopy, flow-cytometry cell sorting, determination of hypodiploid cells and dislocated phosphatidylserine residues	ELF-MF increases the rate of cell death in normal cell lines but not in in cells from genetic instability syndromes. Genes implicated in genetic instability syndromes are relevant in modulating the response of cells to ELF-MF	[20]
lymphocytes, thymocytes, FTRL-5, Hep G2, U937, HeLa and 3DO cells human	comparative study of the effect of static MF	6 mT	24, 48 h	Apoptosis(spontaneous and drug-induced), cell viability, proliferation, Ca^{2+} concentration and morphology	Static MF exposure interfered with apoptosis in a cell type- and exposure time-dependent manner. The modulation of the apoptotic process by MFs could be used to develop new therapeutic strategies for cancer cells that have become chemoresistant.	[21]
lymphoid human cells	EMF effect on cell viability and death	50 Hz, 45 mT	3 h	human U937 lymphoid cells, cell death induction by puromycin and evaluation	PEMF protects U937 cells against puromycin -induced cell death	[22]
Mesenchymal Stem Cells	Effects on growth and differentiation	50 Hz 20 mT	23 days	cell metabolism; extracellular sodium and potassium concentrations; calcium level; cell viability and proliferation	ELF magnetic field may influence the early development of Mesenchymal Stem Cells related adult cells	[23]

Type of cell	response to MF	Range of MFs	Duration	Methods/Cell types	Results	Ref.
Natural Killer (NK) cells	effectiveness of SMFs in enhancing the killing ability of NK cells	0.4-T	4 h and 72 h	Effect of SMF on NK cell viability and cytotoxicity, Membrane fluidity measurement, P3/DAG and STAT3 pathways	NK cells pre-exposed to 0.4-T SMF show potential as a tool for immune-therapy treatment of cancer	[24]
Natural Killer (NK) cells	NK cell cytotoxic activity in exposed compared to controls.	50 Hertz (Hz), 2 mT		NK cytotoxic activity of splenocytes measured in vitro by natural anticandidial colorimetric index.	NK cell cytotoxic activity decreased in exposed compared to controls. Our data suggests that part of the immune system, the NK cell, can be suppressed by a 50 Hz magnetic field	[25]
osteosarcoma human cell	possibility that power frequency ELF fields can induce variations directly in the expression of cell adhesion molecules	50 Hz, 0.5 mT	7 and 14 days	Cell lines MG-63 and Saos-2, Scanning Electron Microscopy, growth, cell cycle and celle death analysis, CAM expression	No variations in surface morphology and cell death occurred be-tween control and exposed cells in both MG-63 and Saos-2 cells, while significant changes were noted in cell growth and fibronectin and CD44 expression in MG-63 cells	[26]
promyelocytic Human cell	Effects on calcium signaling in human neutrophil cell lines	50 Hz 5, 300, 500 μT	30 min	cell lines HL-60 and PLB-98, calcium indicator Fluo-4 NW, qPCR, Scanning Electron Microscopy,	Exposure to LF EMF does not affect calcium signalling in neutrophils in vitro	[27]
promyelocytic human cells-	effects on the expression of some protooncogenes	10 T	1, 2, 4, 8, 24, 36, 48, 72 h	HL-60 cell culture, Western blotting of c-Jun, c-Fos and c-Myc protooncogenes, c-Jun phosphorylation	A strong MF gradient may have significant biological effects. particularly regarding processes related to an elevation of e-jun gene expression	[28]

References

1. Sun, W.J.; Gan, Y.P.; Fu, Y.T.; Lu, D.Q.; Chiang, H. An incoherent magnetic field inhibited egf receptor clustering and phosphorylation induced by a 50-hz magnetic field in cultured fl cells. *Cellular Physiology and Biochemistry* **2008**, *22*, 507-514.
2. Testa, A.; Cordelli, E.; Stronati, L.; Marino, C.; Lovisolo, G.A.; Fresegna, A.M.; Conti, D.; Villani, P. Evaluation of genotoxic effect of low level 50 hz magnetic fields on human blood cells using different cytogenetic assays. *Bioelectromagnetics* **2004**, *25*, 613-619.
3. Stronati, L.; Testa, A.; Villani, R.; Marino, C.; Lovisolo, G.A.; Conti, D.; Russo, F.; Fresegna, A.M.; Cordelli, E. Absence of genotoxicity in human blood cells exposed to 50 hz magnetic fields as assessed by comet assay, chromosome aberration, micronucleus, and sister chromatid exchange analyses. *Bioelectromagnetics* **2004**, *25*, 41-48.
4. Kayhan, H.; Erdebilli, B.; Gonen, S.; Esmekaya, M.A.; Ertekin, E.; Canseven, A.G. Effects of extremely low-frequency magnetic field on healthy fibroblasts and breast cancer cells. *Journal of Istanbul Faculty of Medicine-Istanbul Tip Fakultesi Dergisi* **2020**, *83*, 384-389.
5. Ruiz-Gomez, M.J.; Martinez-Morillo, M. Enhancement of the cell-killing effect of ultraviolet-c radiation by short-term exposure to a pulsed magnetic field. *International Journal of Radiation Biology* **2005**, *81*, 483-490.
6. Eleuteri, A.M.; Amici, M.; Bonfili, L.; Cecarini, V.; Cuccioloni, M.; Grimaldi, S.; Giuliani, L.; Angeletti, M.; Fioretti, E. 50hz extremely low frequency electromagnetic fields enhance protein carbonyl groups content in cancer cells: Effects on proteasomal systems. *Journal of Biomedicine and Biotechnology* **2009**.
7. Yoshizawa, H.; Tsuchiya, T.; Mizoe, H.; Ozeki, H.; Kanao, S.; Yomori, H.; Sakane, C.; Hasebe, S.; Motomura, T.; Yamakawa, T., *et al.* No effect of extremely low-frequency magnetic field observed on cell growth or initial response of cell proliferation in human cancer cell lines. *Bioelectromagnetics* **2002**, *23*, 355-368.
8. Kim, J.; Ha, C.S.; Lee, H.J.; Song, K. Repetitive exposure to a 60-hz time-varying magnetic field induces DNA double-strand breaks and apoptosis in human cells. *Biochemical and Biophysical Research Communications* **2010**, *400*, 739-744.
9. Supino, R.; Bottone, M.G.; Pellicciari, C.; Caserini, C.; Bottiroli, G.; Belleri, M.; Veicsteinas, A. Sinusoidal 50 hz magnetic fields do not affect structural morphology and proliferation of human cells in vitro. *Histology and Histopathology* **2001**, *16*, 719-726.
10. Savage, R.E.; Kanitz, M.H.; Lotz, W.G.; Conover, D.; Hennessey, E.M.; Hanneman, W.H.; Witzmann, F.A. Changes in gene and protein expression in magnetic field-treated human glioma cells. *Toxicology Mechanisms and Methods* **2005**, *15*, 115-120.
11. Kanitz, M.H.; Witzmann, F.A.; Lotz, W.G.; Conover, D.; Savage, R.E. Investigation of protein expression in magnetic field-treated human glioma cells. *Bioelectromagnetics* **2007**, *28*, 546-552.
12. Harada, S.; Yamada, S.; Kuramata, O.; Gunji, Y.; Kawasaki, M.; Miyakawa, T.; Yonekura, H.; Sakurai, S.; Bessho, K.; Hosono, R., *et al.* Effects of high elf magnetic fields on enzyme-catalyzed DNA and rna synthesis in vitro and on a cell-free DNA mismatch repair. *Bioelectromagnetics* **2001**, *22*, 260-266.
13. Zhou, J.L.; Li, C.L.; Yao, G.D.; Chiang, H.A.; Chang, Z.L. Gene expression of cytokine receptors in hl60 cells exposed to a 50 hz magnetic field. *Bioelectromagnetics* **2002**, *23*, 339-346.
14. Kim, K.; Lee, Y.S.; Kim, N.; Choi, H.D.; Kang, D.J.; Kim, H.R.; Lim, K.M. Effects of electromagnetic waves with lte and 5g bandwidth on the skin pigmentation in vitro. *International Journal of Molecular Sciences* **2021**, *22*.

15. Calcabrini, C.; Mancini, U.; De Bellis, R.; Diaz, A.R.; Martinelli, M.; Cucchiarini, L.; Sestili, P.; Stocchi, V.; Potenza, L. Effect of extremely low-frequency electromagnetic fields on antioxidant activity in the human keratinocyte cell line nctc 2544. *Biotechnology and Applied Biochemistry* **2017**, *64*, 415-422.
16. Jandova, A.; Hurych, J.; Pokorny, J.; Cocek, A.; Trojan, S.; Nedbalova, M.; Dohnalova, A. Effects of sinusoidal magnetic field on adherence inhibition of leukocytes. *Electro- and Magnetobiology* **2001**, *20*, 397-413.
17. Robison, J.G.; Pendleton, A.R.; Monson, K.O.; Murray, B.K.; O'Neill, K.L. Decreased DNA repair rates and protection from heat induced apoptosis mediated by electromagnetic field exposure. *Bioelectromagnetics* **2002**, *23*, 106-112.
18. Jandova, A.; Pokorny, J.; Cocek, A.; Trojan, S.; Nedbalova, M.; Dohnalova, A. Effects of sinusoidal 0.5 mt magnetic field on leukocyte adherence inhibition. *Electromagnetic Biology and Medicine* **2004**, *23*, 81-96.
19. Kaszuba-Zwoinska, J.; Zdzilowska, E.; Chorobik, P.; Slodowska-Hajduk, Z.; Juszczak, K.; Zaraska, W.; Thor, P.J. Pulsing electromagnetic field and death of proliferating peripheral blood mononuclear cells from patients with acute myelogenic leukemia. *Advances in Clinical and Experimental Medicine* **2011**, *20*, 721-727.
20. Mangiacasale, R.; Tritarelli, A.; Sciamanna, I.; Cannone, M.; Lavia, P.; Barberis, M.C.; Lorenzini, R.; Cundari, E. Normal and cancer-prone human cells respond differently to extremely low frequency magnetic fields. *Febs Letters* **2001**, *487*, 397-403.
21. Tenuzzo, B.; Chionna, A.; Panzarini, E.; Lanubile, R.; Tarantino, P.; Di Jeso, B.; Dwikat, M.; Dini, L. Biological effects of 6 mt static magnetic fields: A comparative study in different cell types. *Bioelectromagnetics* **2006**, *27*, 560-577.
22. Kaszuba-Zwoinska, J.; Wojcik, K.; Bereta, M.; Ziomber, A.; Pierzchalski, P.; Rokita, E.; Marcinkiewicz, J.; Zaraska, W.; Thor, P. Pulsating electromagnetic field stimulation prevents cell death of puromycin treated u937 cell line. *Journal of Physiology and Pharmacology* **2010**, *61*, 201-205.
23. Yan, J.H.; Dong, L.A.; Zhang, B.H.; Qi, N.M. Effects of extremely low-frequency magnetic field on growth and differentiation of human mesenchymal stem cells. *Electromagnetic Biology and Medicine* **2010**, *29*, 165-176.
24. Lin, S.L.; Su, Y.T.; Feng, S.W.; Chang, W.J.; Fan, K.H.; Huang, H.M. Enhancement of natural killer cell cytotoxicity by using static magnetic field to increase their viability. *Electromagnetic Biology and Medicine* **2019**, *38*, 131-142.
25. Canseven, A.G.; Seyhan, N.; Mirshahidi, S.; Imir, T. Suppression of natural killer cell activity on candida stellatoidea by a 50 hz magnetic field. *Electromagnetic Biology and Medicine* **2006**, *25*, 79-85.
26. Santini, M.T.; Rainaldi, G.; Ferrante, A.; Indovina, P.L.; Vecchia, P.; Donelli, G. Effects of a 50 hz sinusoidal magnetic field on cell adhesion molecule expression in two human osteosarcoma cell lines (mg-63 and saos-2). *Bioelectromagnetics* **2003**, *24*, 327-338.
27. Golbach, L.A.; Philippi, J.G.M.; Cuppen, J.J.M.; Savelkoul, H.F.J.; Verburg-van Kemenade, B.M.L. Calcium signalling in human neutrophil cell lines is not affected by low-frequency electromagnetic fields. *Bioelectromagnetics* **2015**, *36*, 430-443.
28. Hirose, H.; Nakahara, T.; Zhang, Q.M.; Yonei, S.; Miyakoshi, J. Static magnetic field with a strong magnetic field gradient (41.7 t/m) induces c-jun expression in hl-60 cells. *In Vitro Cellular & Developmental Biology-Animal* **2003**, *39*, 348-352.
