# Supplementary material for: Magnetic Fields and Cancer: Epidemiology, Cellular Biology, and Theranostics
Source: Int J Mol Sci. 2022 Jan 25;23(3):1339. doi: 10.3390/ijms23031339 (PMC8835851; doi:10.3390/ijms23031339)
Supplement: Supplementary file 1 [file ijms-23-01339-s001.zip › Supplementary Tables S1-S5/Supplementary Table S4.pdf]

International Journal of *Molecular Sciences*

Supplementary

Magnetic Fields and Cancer

Massimo E. Maffei [1,*]

[1] Dept. Life Sciences and Systems Biology, University of Turin, Via Quarello 15/a, 10135 Turin, Italy; massimo.maffei@unito.it

* Correspondence: massimo.maffei@unito.it; Tel.: +39011 6705967

Supplementary Table S4.

Studies on animals

Type of cell	Animal	response to MF	Range of MFs	Duration	Methods/Cell types	Results	Ref.
adenocarcinoma, mammary murine	mice	Effects of MF on Tumor Experimental Models	50 Hz 2 mT	2, 4 weeks	C3H/DBA 2J female hybrid mice; X Rays Irradiation; tumor growth.	When X-ray radiation was applied, the cytotoxic effect of ionizing radiation was clear, but was not increased or modified by MF exposure	[1]
blood coagulation	mice	Effects on selected blood coagulation variables	50 Hz 15 μT	14 weeks.	OF1 mice; blood samples; thromboplastin time (APTT), prothrombin time (PT), fibrinogen, reptilase time (RT) and factor VIII activity determination;	Females showed a very significant shortening of the PT time associated with ELF-MF exposure. Exposure also caused significant increases in the female APTT and RT values,	[2]
breast cancer cells	mice	Effect of Magnetic Fields on Tumor Growth and Viability	94 mT	360 min daily for 4 weeks	metastatic mouse breast tumor cell line EpH4-MEKBcl2; Tumor growth progression; live luciferase-labeled tumor cells; Cell death assay	Exposure of the mice to MF suppresses tumor growth.	[3]
Carcinoma mice, Squamous Cell	mice	effect of Magnetic Field on Incidence of Squamous Cell Carcinomas	2 mT	6 h per day, 5 days per week for 52 consecutive weeks,	SENCAR mice; histological examination, Histopathology	No association between exposure to 60 Hz MF and the incidence of benign or malignant tumors	[4]
cerebellum	mice	DNA damage and apoptosis in the immature cerebellum	60 Hz 1 mT	2 h	Exposure to X-irradiation; Alkaline comet assay; Detection of apoptosis	Results do not support the hypothesis that acute MF exposure causes DNA damage in the cerebellums of immature mice	[5,6]
hematologic parameters	mice	effects on spleen hyperfunction	60 Hz 110 μT	1, 6, 8 months	hematologic parameters; biochemical parameters; weight	A chronic exposure of mice to a 60 Hz magnetic field could influence some	[7]

Type of cell	Animal	response to MF	Range of MFs	Duration	Methods/Cell types	Results	Ref.
					of heart, lung, liver, spleen, kidney and brain.	hematologic parameters and the weight of liver and spleen.	
lymphocytes splenic	mice	Effects on inflammatory genes and activation of splenic lymphocytes	50 Hz 500 μT	8h / day (5 days a week) for 60 days	20 Kunming mice; RT-qPCR IFN-γ, T-bet; IL-2, IL-4, GATA-3; Flow cytometry;	Exposure to 50 Hz magnetic field did not alter responses of inflammatory genes and activation of splenic lymphocytes in mice, except for body weight	[8]
lymphoma, development	mice	effects on promotion of lymphoma development	50 Hz 1 mT	7 days/week, 24 h/day, during nighttime (12 h).	AKR/J mice; survival time, body weight, lymphoma development, hematological parameters	Resulting data do not support the hypothesis that exposure to sinusoidal 50 Hz magnetic fields is a significant risk factor for hematopoietic diseases, even at this relatively high exposure level.	[9]
melatonin	mice	The influence of long-term exposure on nocturnal melatonin secretion	0.5- 77 μT	24 h	*Mus musculus* AKR and BALB/c strains; plasma melatonin; blood analysis	Long-term and continuous exposure to simulated powerline magnetic fields did not result in a decreased nocturnal melatonin secretion	[10]
neoplastic development	mice	Effects on the tumor promotion process and fertility	50 Hz 50 μT	12 h /day for 1 week, and up to 15.5 months.	C57BL/6NJ female mice and C3H/HeNJ male mice; Histological analysis; Morphometrical analysis of testis	Long-term exposure to magnetic fields is a significant risk factor for neoplastic development and fertility in mice	[11]
colon carcinogenesis	rats	Effects on Colon Carcinogenicity in a Medium-Term Bioassay	1 mT	12h/day for 7 weeks	male Sprague-Dawley rats; histopathological examination; Electron microscopy; Blue Feulgen DNA ploidy analysis; DNA cytometry; Proliferating cell nuclear antigen immunohistochemistry; Blood biochemistry	The action of an artificial MF on rats is not carcinogenic/or cancer-promoting in colon carcinogenesis	[12]

Type of cell	Animal	response to MF	Range of MFs	Duration	Methods/Cell types	Results	Ref.
colon tumor	rat	Variable E-cadherin expression in a MNU-induced colon tumor model	5 mT	6 h/day for 6 months	Wistar albino rats; histological analyses; immunohistochemistry, histopathology	Electromagnetic fields result in significant alterations in cell adhesion mechanisms	[13]
leukemia, lymphocytic	rats	effects on Large granular lymphocytic (LGL) leukemia	60 Hz 1 mT	20 h/day, 7 days/week, 16 weeks	Fischer/344 rats; blood analysis; Percent palpable spleens	Although the positive control group showed different body weight curves and developed signs of leukemia earlier than other groups, differences were not detected between expo-sure groups and ambient controls. Furthermore, there were no overall effects of magnetic fields on splenomegaly or survival in exposed animals. In addition, no significant and/or consistent differences were detected in hematological parameters between the magnetic field exposed and the ambient control groups	[14]
mammary epithelium	rats	Magnetic Field Exposure effect on Cell Proliferation and Melatonin Levels in the Mammary Gland of Rats	100 µT	2 weeks	Female Sprague Dawley rats; experiments with the tumor promoter TPA; bromodeoxyuridine and Ki-67 proliferation antigen Immunohistochemistry and labelling; determination of Melatonin	MF exposure results in an increased proliferative activity of the mammary epithelium, which is a likely explanation for the cocarcinogenic or tumor promoting effects of MF ex-posure observed in the 7,12-dimethylbenz(a)anthracene model of breast cancer	[15]
mammary	rats	Effectson mammary tumorigenesis in the DMBA model of breast cancer	50 Hz 100 µT	24 h/day for 26 weeks	Fischer 344 rats; Quantification of mammary tumors; whole-mount analysis	MF exposure significantly facilitated mammary tumorigenesis	[16]

Type of cell	Animal	response to MF	Range of MFs	Duration	Methods/Cell types	Results	Ref.
mammary, lung and skin	rats	Effects on mammary, lung and skin tumorigenesis	6.25 μT	8 h per day for 14 weeks	Sprague–Dawley female rats; Tumor Scoring and Histopathological Analysis; lung and skin tumor models	MF exposure does not appear to be a strong co-tumorigenic agent in the chosen murine mammary, lung and skin models	[17]

References

1. Galloni, P.; Marino, C. Effects of 50 hz magnetic field exposure on tumor experimental models. *Bioelectromagnetics* **2000**, *21*, 608-614.
2. Vallejo, D.; Hidalgo, M.A.; Hernandez, J.M. Effects of long-term exposure to an extremely low frequency magnetic field (15 microt) on selected blood coagulation variables in of1 mice. *Electromagn Biol Med* **2019**, *38*, 279-286.
3. Tatarov, I.; Panda, A.; Petkov, D.; Kolappaswamy, K.; Thompson, K.; Kavirayani, A.; Lipsky, M.M.; Elson, E.; Davis, C.C.; Martin, S.S., *et al.* Effect of magnetic fields on tumor growth and viability. *Comparative Medicine* **2011**, *61*, 339-345.
4. McLean, J.R.; Thansandote, A.; McNamee, J.P.; Tryphonas, L.; Lecuyer, D.; Gajda, G. A 60 hz magnetic field does not affect the incidence of squamous cell carcinomas in sencar mice. *Bioelectromagnetics* **2003**, *24*, 75-81.
5. McNamee, J.P.; Bellier, P.V.; McLean, J.R.N.; Marro, L.; Gajda, G.B.; Thansandote, A. DNA damage and apoptosis in the immature mouse cerebellum after acute exposure to a 1 mt, 60 hz magnetic field. *Mutation Research-Genetic Toxicology and Environmental Mutagenesis* **2002**, *513*, 121-133.
6. McNamee, J.P.; Bellier, P.V.; Chauhan, V.; Gajda, G.B.; Lemay, E.; Thansandote, A. Evaluating DNA damage in rodent brain after acute 60 hz magnetic-field exposure. *Radiation Research* **2005**, *164*, 791-797.
7. Cabrales, L.B.; Ciria, H.C.; Bruzon, R.P.; Quevedo, M.S.; Cespedes, M.C.; Salas, M.F. Elf magnetic field effects on some hematological and biochemical parameters of peripheral blood in mice. *Electro- and Magnetobiology* **2001**, *20*, 185-191.
8. Luo, X.; Jia, S.J.; Li, R.Y.; Gao, P.; Zhang, Y.W. Occupational exposure to 50 hz magnetic fields does not alter responses of inflammatory genes and activation of splenic lymphocytes in mice. *International Journal of Occupational Medicine and Environmental Health* **2016**, *29*, 277-291.
9. Sommer, A.M.; Lerchl, A. 50 hz magnetic fields of 1 mt do not promote lymphoma development in akr/j mice. *Radiation Research* **2006**, *165*, 343-349.
10. de Bruyn, L.; de Jager, L.; Kuyl, J.M. The influence of long-term exposure of mice to randomly varied power frequency magnetic fields on their nocturnal melatonin secretion patterns. *Environmental Research* **2001**, *85*, 115-121.
11. Qi, G.Y.; Zuo, X.X.; Zhou, L.H.; Aoki, E.; Okamula, A.; Watanebe, M.; Wang, H.P.; Wu, Q.H.; Lu, H.L.; Tuncel, H., *et al.* Effects of extremely low-frequency electromagnetic fields (elf-emf) exposure on b6c3f1 mice. *Environmental Health and Preventive Medicine* **2015**, *20*, 287-293.
12. Salim, E.I.; Omar, K.M.; Abou-Hattab, H.A.; Abou-Zaid, F.A. Pituitary toxicity but lack of rat colon carcinogenicity of a dc-magnetic field in a medium-term bioassay. *Asian Pacific Journal of Cancer Prevention* **2008**, *9*, 131-140.
13. Tuncel, H.; Shimamoto, F.; Cagatay, P.; Kalkan, M.T. Variable e-cadherin expression in a mnu-induced colon tumor model in rats which exposed with 50 hz frequency sinusoidal magnetic field. *Tohoku Journal of Experimental Medicine* **2002**, *198*, 245-249.
14. Anderson, L.E.; Morris, J.E.; Miller, D.L.; Rafferty, C.N.; Ebi, K.L.; Sasser, L.B. Large granular lymphocytic (lgl) leukemia in rats exposed to intermittent 60 hz magnetic fields. *Bioelectromagnetics* **2001**, *22*, 185-193.
15. Fedrowitz, M.; Westermann, J.; Loscher, W. Magnetic field exposure increases cell proliferation but does not affect melatonin levels in the mammary gland of female sprague dawley rats. *Cancer Research* **2002**, *62*, 1356-1363.

16. Fedrowitz, M.; Loscher, W. Exposure of fischer 344 rats to a weak power frequency magnetic field facilitates mammary tumorigenesis in the dmba model of breast cancer. *Carcinogenesis* **2008**, *29*, 186-193.
17. Lee, H.J.; Choi, S.Y.; Jang, J.J.; Gimm, Y.M.; Pack, J.K.; Choi, H.D.; Kim, N.; Lee, Y.S. Lack of promotion of mammary, lung and skin tumorigenesis by 20 khz triangular magnetic fields. *Bioelectromagnetics* **2007**, *28*, 446-453.
