# Supplementary material for: Magnetic Fields and Cancer: Epidemiology, Cellular Biology, and Theranostics
Source: Int J Mol Sci. 2022 Jan 25;23(3):1339. doi: 10.3390/ijms23031339 (PMC8835851; doi:10.3390/ijms23031339)
Supplement: Supplementary file 1 [file ijms-23-01339-s001.zip › Supplementary Tables S1-S5/Supplementary Table S5.pdf]

International Journal of
Molecular Sciences

Supplementary

Magnetic Fields and Cancer

Massimo E. Maffei [1,*]

[1] Dept. Life Sciences and Systems Biology, University of Turin, Via Quarello 15/a, 10135 Turin, Italy; massimo.maffei@unito.it

* Correspondence: massimo.maffei@unito.it; Tel.: +39011 6705967

Supplementary Table S5.

Involvement of Reactive Oxygen (ROS) and Reactive Nitrogen (RNS) Species

Type of cell/tissue/organ	response to MF	Range of MFs	Duration	Methods/Cell types	Results	Ref.
Blood platelets	Effects f MF from display screens on cell oxygen metabolism	1 kHz, 150 V/m, 220 V/m)	30, 60 min.	human blood platelets; activity of superoxide dismutase, catalase and malondialdehyde concentration;	exposure to electromagnetic radiation of 1 kHz frequency and 150 V/m and 220 V/m intensity may cause adverse effects within blood platelets' oxygen metabolism and thus may lead to physiological dysfunction of the organism	[1]
Brain	Effects on the antioxidant defense system in the brain	60 Hz 1.2 mT	3 h	Balb/c mouse; SOD activity; chemiluminescence from brain homogenates	MF significantly increased brain SOD activity. MF could deteriorate antioxidant defensive system by ROS, other than superoxide radicals	[2]
Breast cancer cells	LF-MFs inhibit proliferation by triggering apoptosis and altering cell cycle distribution	50- 275 Hz 1 mT	6, 12, 24, or 36 h	Cell Viability Assay, Apoptosis Assays, Western Blotting, Intracellular ROS Level	LF-MF effectively increases the level of ROS, suppresses the PI3K/AKT signaling pathway, and activates glycogen synthase kinase-3β (GSK-3β).	[3]
Breast cancer cells	Selenium reduces mobile phone (900 MHz)-induced oxidative stress, mitochondrial function, and apoptosis	900 MHz with 217 Hz pulses 12 μW/cm^2	1, 2, 6, and 24 h	MDA-MB-231 cells, cell viability, intracellular ROS production, mitochondrial membrane depolarization, cell apoptosis, and caspase-3 and caspase-9 values.	900 MHz EMR appears to induce apoptosis effects through oxidative stress and mitochondrial depolarization although incubation of selenium seems to counteract the effects on apoptosis and oxidative stress.	[4]
Carcinoma murine squamous cell	Influence on antioxidative melatonin properties	3 Hz-3 kHz 0.11 mT	16 min	AT478 murine squamous cell carcinoma; enzyme activity of MnSOD, Cu/ZnSOD, GSH-Px; MDA levels	Application of ELF-MF on treated cells caused an increase of both superoxide dismutase activity and MDA level, but influence of ELF-MF on GSH-Px activity was negligible	[5]
Carcinoma renal cell	Influence on Clear Cell Renal Carcinoma	50 Hz 4.5 mT	30 min/day for 5 days	EK293, 786-O, 769-P, and Caki1 cells; Cells Viability; Hanging Drop Assay; Migration and Invasion Assay; Apoptosis and Cell Cycle Analysis; ROS Generation	EMF could serve as a potential tool to manipulate cell viability through the involvement of ROS. ELF-EMF can be considered a factor that inhibits the progression of clear cell	[6]

Type of cell/tissue/organ	response to MF	Range of MFs	Duration	Methods/Cell types	Results	Ref.
Carcinosarcoma	Effects on growth and hepatic redox state	50 Hz	80 min. every other day 5 times in total	SOD, CAT, GSH content, glutathione peroxidase (GP) and thiobarbituric acid reactive substances (TBARS)	Treatment can inhibit Walker-256 carcinosarcoma growth and result in less pronounced oxidative stress damage to the liver of tumor-bearing rats. Non-ionizing EMF can be used to influence the hepatic redox state and combat cancer with reduced side effects.	[7]
Embryonic stem cells	Differential effects on embryonic development and vasculogenesis	0.04 µT	20 days	female BALB/c mice; Embryonic stem cell culture and inhibitor treatment; Measurement of ROS generation; Immunofluorescence	Vascular endothelial growth factor is an important mediator during embryonic development that can be influenced by high strength MFs, which in consequence leads to severe abnormalities in fetus organs and blood vessel formation	[8]
Erythro-leukemic cells	Effects on catalase, cytochrome P450 and nitric oxide synthase	50 Hz<br>1 mT	1, 3, 6, 9, 12, 18, 24 h	Human erythro-leukemic cell line K562; CAT activity; determination of superoxide anion; iNOS activity; Western blot analysis;	LF-EMF triggers protein activation mediated by ligands activating NADH oxidase that alters ROS-regulated pathways, particularly pro-proliferative and/or survival pathways. ELF-EMF affects not only the ROS product but also the enzymatic activity. Effects on oxidative metabolism and ROS signaling may be particularly important in hematologic malignancies.	[9]
Frontal cortex and hippocampus	Effects on antioxidative enzymes activity and DNA in brain	128 mT	1 h/day for 30 days	GPx, CuZn-SOD, CAT activities; malondialdehyde (MDA) concentration; DNA oxidation	Exposure decreases antioxidant enzyme activity in frontal cortex and hippocampus, but glutathione levels remained unchanged in the both brain structures. Exposure significant increase of metallothioneins level in frontal cortex indicating the absence of DNA oxidation	[10]
Keratinocyte	Effect on antioxidant activity	50 Hz<br>25- 200 µT	1, 2, or 4 h	human keratinocyte cell line NCTC 2544; ROS production with dihydrorhodamine (DHR); fluorescence microscopy;	ELF-EMF induces a slight oxidative stress that does not overwhelm the metabolic capacity of the cells or have a cytotoxic effect	[11]

Type of cell/tissue/organ	response to MF	Range of MFs	Duration	Methods/Cell types	Results	Ref.
				hemocytometry; GSH content; SOD activity; Lipid peroxidation;		
Liver	Effects of Long-term exposure of extremely low frequency magnetic field on oxidative/nitrosative stress	50Hz, 1mT	4h/day for 45 days	Wistar-Albino female and male rats, MDA and 3-nitrotyrosine (3-NT) detection	Long-term ELF-MF exposure may enhance the oxidative/nitrosative stress in liver tissue of the female rats and could have a deteriorative effect on cellular proteins rather than lipids by enhancing 3-NT formation.	[12]
Liver and kidney	Influence MF on cadmium toxicity: study of oxidative stress and DNA damage	128 mT	1 h/day for 30 days	Wistar male rats; Cadmium determination; Blood analysis; MDA assay; GPx, CAT and SOD activities; DNA and 8-oxodGuo analysis	The association between SMF and Cd failed to alter transaminases, MDA and 8-oxodGuo concentration. Cd treatment altered antioxidant enzymes and DNA in liver and kidney of rats. Moreover, SMF associated to Cd disrupt this antioxidant response in liver compared to Cd-treated rats	[13]
Myelogenic leukemia cells	MF exposure on superoxide radical anion formation and HSP70 induction	50 Hz 0.025, 0.050, 0.10 mT	1 h	Human chronic myelogenic leukemia cells (K562), Cell proliferation and cell cycle analysis, Protein isolation and HSP70 analysis, Western blot analysis and Flow cytometric measurements of HSP70, Superoxide radical anion analysis	An early response to ELF MF in K562 cells seems to be an increased amount of oxygen radicals, leading to HSP70 induction. Furthermore, the results suggest that there is a flux density threshold where 50-Hz MF exerts its effects on K562 cells, at or below 0.025 mT, and also that it is the MF, and not the induced electric field, which is the active parameter	[14]
Neuroblastoma and glioma cell lines	Induction of micronuclei and superoxide production in cells exposed to weak MFs	50 Hz 10, 30 mT	24 h	Micronucleus assay, Mitochondrial and cytosolic superoxide production	For cytosolic superoxide, the effect size was unexpectedly large at 10 μT. The results indicate that the threshold for biological effects of ELF MFs is 10 μT or less.	[15]
Neuroblastoma cell	Oxidative Stress and Neurodegeneration	50-Hz 1 mT	up to 24 h	neuroblastoma cell line SH-SY5Y; NOS activity; superoxide anion measurement; CAT activity; Cell viability	ELF-EMF exposure elevated the expression of NOS and O_2^-, which were countered by compensatory changes in antioxidant CAT activity	[16]

Type of cell/tissue/organ	response to MF	Range of MFs	Duration	Methods/Cell types	Results	Ref.
					and enzymatic kinetic parameters related to CYP-450 and CAT activity	
Neuroblastoma cells	Induction of genomic instability, oxidative processes, and mitochondrial activity by 50 Hz magnetic fields	50-Hz, 100-μT	24 h	Micronucleus frequency, Mitochondrial and cytosolic superoxide production, ROS production, and reduced glutathione level, Lipid peroxidation. Mitochondrial activity by 3-[4,5-dimethythiazol-2-yl]-2,5-diphenyl tetrazolium bromide (MTT)	MF exposure disturbs oxidative balance immediately after the exposure, which might explain our previous findings on MF altered cellular responses to menadione-induced DNA damage	[17]
Neuroblastoma cells	Cellular detection of MFs and effects on superoxide levels and genotoxicity	50 Hz 100 μT	24 h	Responses to menadione, Micronuclei, proliferation, viability, cytosolic and mitochondrial $O_2^{\bullet -}$ levels.	The results are consistent with MF effects on light-independent radical reactions.	[18]
Osteosarcoma cells	Effect on self-renewal ability through autophagic degradation of ferritin	0.2-0.4 T	1, 3, and 5 days	Murine osteosarcoma cell line K7M2 and humanosteosarcoma cell line MG63; Cell Counting; detection of ROS with dichlorodihydrofluorescein diacetate (DCFH-DA); Western Blot; siRNA Transfection; Immunohistochemistry; Immunofluorescence; Transmission Electron Microscopy; Fe^{2+} Detection Assay	Prolonged exposure to SMF induced the proliferation and tumor sphere formation. Moreover, SMF promoted the release of ferrous iron (Fe^{2+}) and provoked ROS	[19]
Prostate cancer cells	Effect on apoptosis through reactive oxygen species	60-Hz sinusoidal MF	various timing	DU145, PC3, and LNCaP cells; cell counting; trypan blue exclusion assay; Western blot analysis; flow cytometry; ELISA; RT-PCR; fluorescence microscopy; spectrofluorimetry	60-Hz sinusoidal MF-activated cell growth inhibition of prostate cancer in vitro. Apoptosis together with cell cycle arrest were the dominant causes of the MF-elicited cell growth inhibition, mediated by MF-induced ROS	[20]

Type of cell/tissue/organ	response to MF	Range of MFs	Duration	Methods/Cell types	Results	Ref.
Squamous cell carcinoma	Influence of ELF-EMF on antioxidative vitamin E Properties in AT478 murine cells culture in vitro	0.11 mT	16 min	Antioxidant enzyme activity (manganese- and copper-zinc-containing SOD and GPx) and lipid peroxidation (level of MDA)	ELF-EMF alters antioxidative activities of vitamin E in AT478 tumor cells. Confirms the role of vitamin E in decreasing susceptibility to lipid peroxidation in AT478 tumor cells	[21]
Squamous cell carcinoma	Short-term exposure to ELF-EMF alters the cisplatin-induced oxidative response	50 Hz, 1 mT	16 min	SOD and GSH-Px activity assay. MDA Assay. Alkaline Comet Assay	ELF-EMF lessened the effects of oxidative stress and DNA damage that were induced by cisplatin; however, ELF-EMF alone was a mild oxidative stressor and DNA damage inducer. We speculate that ELF-EMF exerts differential effects depending on the exogenous conditions. This information may be of value for appraising the pathophysiologic consequences of exposure to ELF-EMF.	[22]
THP1 cells	Influence on the antioxidant response and DNA integrity	250 mT	1, 2 and 3 h	THP1 cells (monocyte line); MDA concentration; GPx, CAT, SOD activities; DNA analysis, labile zinc analysis	SMF exposure did not cause oxidative stress and DNA damage in THP1 cells. However, SMF could alter the intracellular labile zinc fraction	[23]

References

1. Lewicka, M.; Henrykowska, G.A.; Pacholski, K.; Smigielski, J.; Rutkowski, M.; Dziedziczak-Buczynska, M.; Buczynski, A. The effect of electromagnetic radiation emitted by display screens on cell oxygen metabolism - in vitro studies. *Archives of Medical Science* **2015**, *11*, 1330-1339.
2. Lee, B.C.; Johng, H.M.; Lim, J.K.; Jeong, J.H.; Baik, K.Y.; Nam, T.J.; Lee, J.H.; Kim, J.; Sohn, U.D.; Yoon, G., *et al.* Effects of extremely low frequency magnetic field on the antioxidant defense system in mouse brain: A chemiluminescence study. *Journal of Photochemistry and Photobiology B-Biology* **2004**, *73*, 43-48.
3. Xu, A.; Wang, Q.; Lin, T. Low-frequency magnetic fields (lf-mfs) inhibit proliferation by triggering apoptosis and altering cell cycle distribution in breast cancer cells. *Int J Mol Sci* **2020**, *21*, 2952.
4. Kahya, M.C.; Nazıroğlu, M.; Çiğ, B. Selenium reduces mobile phone (900 mhz)-induced oxidative stress, mitochondrial function, and apoptosis in breast cancer cells. *Biological Trace Element Research* **2014**, *160*, 285-293.
5. Zwirska-Korczala, K.; Adamczyk-Sowa, M.; Polaniak, R.; Sowa, P.; Birkner, E.; Drzazga, Z.; Brzozowski, T.; Konturek, S.J. Influence of extremely-low-frequency magnetic field on antioxidative melatonin properties in at478 murine squamous cell carcinoma culture. *Biological Trace Element Research* **2004**, *102*, 227-243.
6. Cios, A.; Ciepielak, M.; Stankiewicz, W.; Szymanski, L. The influence of the extremely low frequency electromagnetic field on clear cell renal carcinoma. *Int J Mol Sci* **2021**, *22*.
7. Orel, V.E.; Krotevych, M.; Dasyukevich, O.; Rykhalskyi, O.; Syvak, L.; Tsvir, H.; Tsvir, D.; Garmanchuk, L.; Orel Vcapital Ve, C.; Sheina, I., *et al.* Effects induced by a 50 hz electromagnetic field and doxorubicin on walker-256 carcinosarcoma growth and hepatic redox state in rats. *Electromagn Biol Med* **2021**, 1-13.
8. Bekhite, M.M.; Finkensieper, A.; Abou-Zaid, F.A.; El-Shourbagy, I.K.; El-Fiky, N.K.; Omar, K.M.; Sauer, H.; Wartenberg, M. Differential effects of high and low strength magnetic fields on mouse embryonic development and vasculogenesis of embryonic stem cells. *Reproductive Toxicology* **2016**, *65*, 46-58.
9. Patruno, A.; Tabrez, S.; Pesce, M.; Shakil, S.; Kamal, M.A.; Reale, M. Effects of extremely low frequency electromagnetic field (elf-emf) on catalase, cytochrome p450 and nitric oxide synthase in erythro-leukemic cells. *Life Sciences* **2015**, *121*, 117-123.
10. Amara, S.; Douki, T.; Garel, C.; Favier, A.; Sakly, M.; Rhouma, K.B.; Abdelmelek, H. Effects of static magnetic field exposure on antioxidative enzymes activity and DNA in rat brain. *General Physiology and Biophysics* **2009**, *28*, 260-265.
11. Calcabrini, C.; Mancini, U.; De Bellis, R.; Diaz, A.R.; Martinelli, M.; Cucchiarini, L.; Sestili, P.; Stocchi, V.; Potenza, L. Effect of extremely low-frequency electromagnetic fields on antioxidant activity in the human keratinocyte cell line nctc 2544. *Biotechnology and Applied Biochemistry* **2017**, *64*, 415-422.
12. Erdal, N.; Gürgül, S.; Tamer, L.; Ayaz, L. Effects of long-term exposure of extremely low frequency magnetic field on oxidative/nitrosative stress in rat liver. *J Radiat Res* **2008**, *49*, 181-187.
13. Amara, S.; Abdelmelek, H.; Garrel, C.; Guiraud, P.; Douki, T.; Ravanat, J.L.; Favier, A.; Sakly, M.; Ben Rhouma, K. Influence of static magnetic field on cadmium toxicity: Study of oxidative stress and DNA damage in rat tissues. *Journal of Trace Elements in Medicine and Biology* **2006**, *20*, 263-269.
14. Mannerling, A.C.; Simko, M.; Mild, K.H.; Mattsson, M.O. Effects of 50-hz magnetic field exposure on superoxide radical anion formation and hsp70 induction in human k562 cells. *Radiation and Environmental Biophysics* **2010**, *49*, 731-741.

15. Kesari, K.K.; Juutilainen, J.; Luukkonen, J.; Naarala, J. Induction of micronuclei and superoxide production in neuroblastoma and glioma cell lines exposed to weak 50 hz magnetic fields. *J R Soc Interface* **2016**, *13*, 20150995.
16. Reale, M.; Kamal, M.A.; Patruno, A.; Costantini, E.; D'Angelo, C.; Pesce, M.; Greig, N.H. Neuronal cellular responses to extremely low frequency electromagnetic field exposure: Implications regarding oxidative stress and neurodegeneration. *Plos One* **2014**, *9*.
17. Luukkonen, J.; Liimatainen, A.; Juutilainen, J.; Naarala, J. Induction of genomic instability, oxidative processes, and mitochondrial activity by 50hz magnetic fields in human sh-sy5y neuroblastoma cells. *Mutation Research/Fundamental and Molecular Mechanisms of Mutagenesis* **2014**, *760*, 33-41.
18. Höytö, A.; Herrala, M.; Luukkonen, J.; Juutilainen, J.; Naarala, J. Cellular detection of 50 hz magnetic fields and weak blue light: Effects on superoxide levels and genotoxicity. *Int J Radiat Biol* **2017**, *93*, 646-652.
19. Zhao, B.; Yu, T.; Wang, S.; Che, J.; Zhou, L.; Shang, P. Static magnetic field (0.2-0.4 t) stimulates the self-renewal ability of osteosarcoma stem cells through autophagic degradation of ferritin. *Bioelectromagnetics* **2021**, *42*, 371-383.
20. Koh, E.K.; Ryu, B.K.; Jeong, D.Y.; Bang, I.S.; Nam, M.H.; Chae, K.S. A 60-hz sinusoidal magnetic field induces apoptosis of prostate cancer cells through reactive oxygen species. *International Journal of Radiation Biology* **2008**, *84*, 945-955.
21. Polaniak, R.; Buldak, R.J.; Karon, M.; Birkner, K.; Kukla, M.; Zwirska-Korczala, K.; Birkner, E. Influence of an extremely low frequency magnetic field (elf-emf) on antioxidative vitamin e properties in at478 murine squamous cell carcinoma culture in vitro. *International Journal of Toxicology* **2010**, *29*, 221-230.
22. Bułdak, R.J.; Polaniak, R.; Bułdak, Ł.; Żwirska-Korczala, K.; Skonieczna, M.; Monsiol, A.; Kukla, M.; Duława-Bułdak, A.; Birkner, E. Short-term exposure to 50 hz elf-emf alters the cisplatin-induced oxidative response in at478 murine squamous cell carcinoma cells. *Bioelectromagnetics* **2012**, *33*, 641-651.
23. Amara, S.; Abdelmelek, H.; Garrel, C.; Guiraud, P.; Douki, T.; Ravanat, J.L.; Favier, A.; Sakly, M.; Ben Rhouma, K. Zinc supplementation ameliorates static magnetic field-induced oxidative stress in rat tissues. *Environmental Toxicology and Pharmacology* **2007**, *23*, 193-197.
